# Computing microRNA-gene interaction networks in pan-cancer using miRDriver

**DOI:** 10.1038/s41598-022-07628-z

**Published:** 2022-03-08

**Authors:** Banabithi Bose, Matthew Moravec, Serdar Bozdag

**Affiliations:** 1 Center for Genetic Medicine, Feinberg School of Medicine, Northwestern University, Chicago, IL 60611 USA; 2 Department of Mathematical and Statistical Sciences, Marquette University, Milwaukee, WI 53201 USA; 3 Department of Computer Science and Engineering, University of North Texas, Denton, TX 76203 USA

**Keywords:** Cancer, Computational biology and bioinformatics

## Abstract

DNA copy number aberrated regions in cancer are known to harbor cancer driver genes and the short non-coding RNA molecules, i.e., microRNAs. In this study, we integrated the multi-omics datasets such as copy number aberration, DNA methylation, gene and microRNA expression to identify the signature microRNA-gene associations from frequently aberrated DNA regions across pan-cancer utilizing a LASSO-based regression approach. We studied 7294 patient samples associated with eighteen different cancer types from The Cancer Genome Atlas (TCGA) database and identified several cancer-specific and common microRNA-gene interactions enriched in experimentally validated microRNA-target interactions. We highlighted several oncogenic and tumor suppressor microRNAs that were cancer-specific and common in several cancer types. Our method substantially outperformed the five state-of-art methods in selecting significantly known microRNA-gene interactions in multiple cancer types. Several microRNAs and genes were found to be associated with tumor survival and progression. Selected target genes were found to be significantly enriched in cancer-related pathways, cancer hallmark and Gene Ontology (GO) terms. Furthermore, subtype-specific potential gene signatures were discovered in multiple cancer types.

## Introduction

MICRORNAs (miRNAs) are small non-coding RNAs that act as modulators of the target genes' expression either by inhibiting translation or promoting RNA degradation^1^. Several studies found miRNAs to be the regulators of cancer driver genes that promote tumor initiation, progression and proliferation^2–4^.

Several state-of-the-art methods utilize miRNA and gene expression data to infer miRNA-gene regulatory networks. Among these, ARACNe^5^ and ProMISe^6^ use mutual information-based algorithms and HiddenICP^7^, idaFast^8^ and jointIDA^9^ use invariant causal relationships, i.e., direct or indirect effects of miRNAs on targets to infer miRNA-gene regulatory networks.

Several studies found that DNA copy number aberrated areas, i.e., amplification and deletion regions harbor cancer-driving genes^10,11^ and miRNAs^12–14^.

Several studies integrated copy number data, DNA methylation and gene expression to compute miRNA-gene regulatory networks in cancer^15,16^ using regression-based approaches. These studies, however, mined miRNAs and target genes from the entire genomic locations.

**Figure 1:**
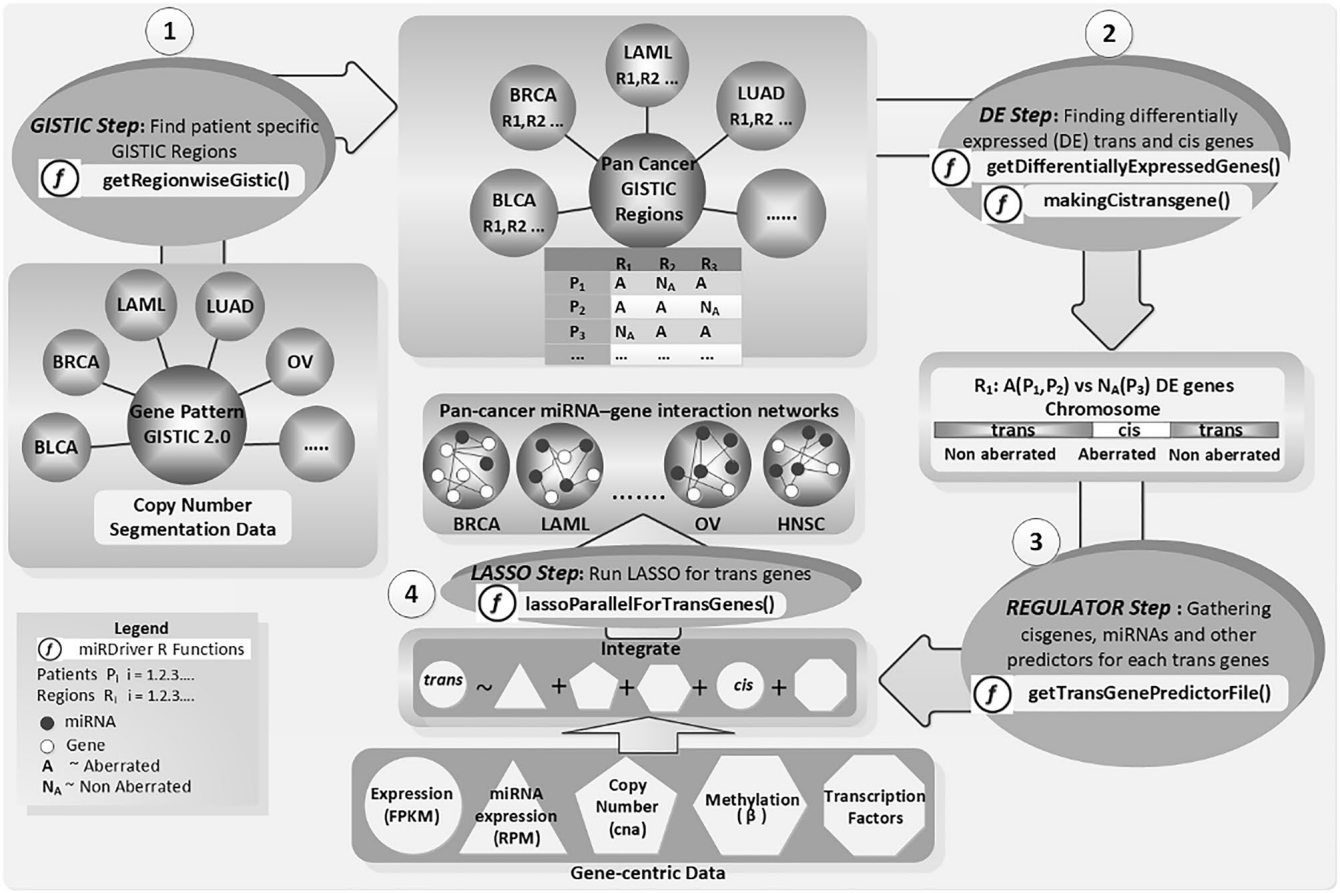
The overview of algorithmic steps used within the *miRDriver* computational pipeline: GISTIC step, Differential Expression step, REGULATOR step and LASSO step with R functions running on pan-cancer.

In our previous study, we developed a computational pipeline called *miRDriver* based on the hypothesis that copy number data from cancer patient samples can be utilized to discover driver miRNAs of cancer^17^. *miRDriver* assumes that miRNAs located within an aberrated region regulate the expression of the genes outside the aberration, extending the aberration effects across the genome and beyond the aberrated region. Since other factors can influence the expression of the genes outside the aberration, *miRDriver* integrates DNA methylation and copy number aberration (CNA) of these genes, transcription factors (TFs) and the expression of the genes located inside an aberration along with the miRNAs to select the regulatory miRNAs for these genes^17^. We computed frequently aberrated chromosomal copy number regions, namely, GISTIC regions, among tumor patient samples (see Materials and Methods). Then, for each GISTIC region, we computed differentially expressed (DE) genes between the tumor samples with the aberration and the samples that did not have the aberration. Afterward, we computed DE *trans* genes (genes outside of aberrated areas) and *cis* genes (genes inside of aberrated areas) for each GISTIC region. Finally, we applied a LASSO-based^18^ regression model to select miRNAs regulating DE genes' expression (Fig. 1).

*miRDriver* outperformed ARACNe, ProMISe, Hidden-ICP, ICP-PAM50, idaFast and jointIDA in retrieving significantly enriched miRNA-gene interactions with the known miRNA-gene interactions. *miRDriver* discovered several potentially novel interactions in multiple cancer types. Several oncogenic and tumor suppressor miRNAs and genes were found to be enriched in the computed miRNA-gene networks. Several miRNAs were found to be associated with patients' survival and disease progression. Selected target genes were found to be significantly enriched in cancer-related biological pathways and GO terms^19^. Furthermore, subtype-specific gene signatures were discovered in multiple cancer types.

In our previous publication, we have demonstrated *miRDriver*’s statistical robustness by applying it to two different cancer types. This study has unique contributions. In the current study, we present *miRDriver* as an R software package with various options for users to run our workflow. We have also demonstrated its application and biological importance by running *miRDriver* on eighteen different cancer types. We have presented extensive results on these cancer types that were not present in our prior publication. We have also presented pan-cancer-wide findings and their relevance to cancer. We have put together a resource of pan-cancer miRNA-gene interactions that will be useful to biologists, clinicians and scientists working on cancer research.

## Results

In this study, we integrated CNA, DNA methylation, TF-gene interactions, gene, and miRNA expression datasets in the *miRDriver* tool to compute miRNA-gene interactions based on DNA copy number aberrated regions in eighteen different cancer types from TCGA. Table 1 shows the cohort sizes for each data modality, the number of all GISTIC regions, the count of *trans* genes in the LASSO step, and the computed miRNA-gene interactions in eighteen different cancer types.

**Table 1:** TCGA cancer types in the study with cohort sizes in different data modalities and results of *miRDriver*.

TCGA cancer type name	Abbreviation	RSeq^a^	CNA^b^	450K^c^	27K^d^	miRNA^e^	GISTIC regions	*trans* genes^f^	Interactions (genes-miRNAs)^g^	Fisher *p*-value^j^
Adrenocortical carcinoma	ACC	79	180	80	*NA*	79	59	4683	308 (253–33)	3.38e−13
Bladder Urothelial Carcinoma	BLCA	411	810	437	*NA*	429	126	5466	578 (416–125)	4.28e−09
Breast invasive carcinoma	BRCA	1102	1103	895	343	1165	66	10,494	1852 (776–187)	17.9e−11
Cervical squamous cell carcinoma and endocervical adenocarcinoma	CESC	304	586	312	*NA*	311	91	4515	558 (349–86)	1.15e−05
Lymphoid Neoplasm Diffuse Large B-cell Lymphoma	DLBC	48	98	48	*NA*	47	42	3697	384 (288–31)	6.06e−16
Esophageal carcinoma	ESCA	161	373	202	*NA*	195	119	4961	738 (521–92)	4.35e−10
Head and Neck squamous cell carcinoma	HNSC	500	1090	580	*NA*	565	105	2591	326 (205–75)	2.47e−08
Kidney renal clear cell carcinoma	KIRC	534	1067	483	*NA*	570	100	3995	586 (501–29)	1.45e−06
Acute Myeloid Leukemia	LAML	151	397	194	418	188	46	3593	590 (431–21)	1.15e−04
Brain Lower Grade Glioma	LGG	511	1021	534	*NA*	528	87	1653	226 (151–45)	1.26e−08
Liver hepatocellular carcinoma	LIHC	371	767	430	*NA*	421	107	3593	316 (224–71)	1.37e−10
Lung adenocarcinoma	LUAD	524	1110	507	150	555	131	4602	1172 (747–142)	1.8e−4
Lung squamous cell carcinoma	LUSC	501	1038	412	161	511	131	2735	449 (266–105)	1.91e−05
Ovarian serous cystadenocarcinoma	OV	374	573	10	613	486	64	3117	1347 (548–147)	0.03
Pancreatic adenocarcinoma	PAAD	177	368	195	*NA*	182	75	2918	530 (371–55)	2.81e−12
Prostate adenocarcinoma	PRAD	498	1038	553	*NA*	544	95	4016	266 (239–43)	9.51e−03
Thyroid carcinoma	THCA	502	1025	571	*NA*	569	75	1138	204 (204–2)	1.58e−04
Uterine Corpus Endometrial Carcinoma	UCEC	547	1098	485	118	556	174	6106	1118 (688–152)	5.73e−09

*NA* not available.

Cohort sizes in ^a^Gene expression; ^b^Copy number aberration; ^c^450K DNA methylation; ^d^27K DNA methylation; ^e^miRNA expression datasets. ^f^No. of DE trans genes used in *miRDriver*'s LASSO step; ^g^No. of selected interactions with no. of selected DE trans genes and no. of selected miRNAs in the parenthesis; ^j^*p*-value of two-sided Fisher's exact test for enrichment of oncogenic miRNAs in each cancer type.

### Computed miRNAs were significantly enriched in the experimentally-validated oncogenic miRNAs

We performed a *two-sided Fisher's exact* test to check the association between the cancer-related miRNAs in OncomiRDB (see Materials and Methods) and the computed miRNAs by *miRDriver*. For each cancer type, the background set in the *Fisher's exact* test consisted of all TCGA miRNAs used in the LASSO step (see Materials and Methods) for that cancer type. For all cancer types, computed miRNAs were significantly enriched (*Fisher's exact* test *p-*value < 0.05) in the oncogenic miRNAs in OncomiRDB (Table 1).

### Computed miRNA-gene interactions were enriched in the known miRNA-gene interactions

To check if the miRNA-gene interactions computed by *miRDriver* were significantly enriched in the known miRNA-gene interactions, we performed a hypergeometric test for each miRNA's computed target genes in each cancer type. We considered only those miRNAs that had at least one known target in the ground truth data (i.e., known miRNA-gene interactions) (see Materials and Methods) from the computed target list. We labeled them as "*Eligible miRNAs*" for the hypergeometric test. The background set, i.e., the hypergeometric test universe, was the set of all the *trans* genes in the HGNC symbol^20^ that were common to the ground truth data. For fourteen cancer types, at least 50% of the "*Eligible miRNAs*" had significant enrichment (*p-*value < 0.05) (Table 2). The entire list of the computed miRNAs with individual hypergeometric *p*-values for all eighteen cancer types can be accessed in Supplemental Table S1.

**Table 2:** Target enrichment.

Cancer type	Eligible miRNAs^a^	Significant miRNAs^b^ (%)
ACC	4	0
BLCA	6	67
BRCA	59	63
CESC	8	88
DLBC	6	83
ESCA	5	60
HNSC	3	67
KIRC	3	67
LAML	7	43
LGG	2	100
LIHC	7	43
LUAD	4	50
LUSC	3	100
OV	27	89
PAAD	7	57
PRAD	1	100
THCA	1	0
UCEC	11	55

For fourteen different cancer types, at least 50% of the "*Eligible miRNAs*" had significantly enriched computed targets in the ground truth data (*p*-value < 0.05).

^a^No. of "Eligible miRNAs" for hypergeometric test for the enrichment of known targets; ^b^percentage of miRNAs with hypergeometric *p*-values < 0.05.

### miRDriver outperformed five state-of-the-art methods in inferring significant miRNA-gene interactions

We compared *miRDriver* with five state-of-the-art methods, namely, ARACNe, ProMISe, HiddenICP, idaFast and jointIDA, by running them on eighteen different cancer types from TCGA. For all these methods, we used gene expression data to compute miRNA-gene interaction networks for our comparison (see Materials and Methods). We performed the hypergeometric test to measure each miRNA's computed targets' enrichment significance in the known miRNA-gene interaction data. We selected only "*Eligible miRNAs*" (i.e., miRNAs with at least one known target in the ground truth data) for this test. We computed the overlapping "*Eligible miRNAs*" for *miRDriver* and each comparable method. We checked if the count of the "*Significant miRNAs*" (i.e., miRNAs with target enrichment test *p*-value < 0.05) in *miRDriver* was more (i.e., *miRDriver* won), less (i.e., *miRDriver* lost), or equal (i.e., there was a draw) than the other method in the overlap. *miRDriver* had more "*Significant miRNAs*" than all other methods for most of the cancer types. For ACC, LUSC and THCA, *miRDriver* and the different methods had no common "*Eligible miRNAs*"; hence, we eliminated these three cancer types from this test. Table 3 summarizes the comparison results in all the cancer types. Table 4 presents the comparison results for ovarian cancer (OV) in detail with the number of "*Eligible miRNAs*" and "*Significant miRNAs*" in all the methods. For a detailed comparison with all the cancer types, see Supplemental Table S2. We also compared *miRDriver* with sequence-based competing endogenous RNA (ceRNA) prediction tool, Cupid^21^ for BRCA. *miRDriver* outperformed Cupid as well. Cupid predicts miRNAs that are also predicted to "mediate" ceRNA interactions. For TCGA BRCA, the authors of Cupid predicted 299K candidate miRNA–target interactions. We filtered this list with 6504 input genes and 255 miRNAs, the same inputs we used in *miRDriver* for BRCA. We considered the top 2437 (top 1 percentile) of miRNA-gene interactions based on Cupid reported scores to get highly confident interactions for our comparison. The count of the "*Significant miRNAs*" in *miRDriver* was higher than Cupid in the overlap (see Supplemental Table S2).

**Table 3:**
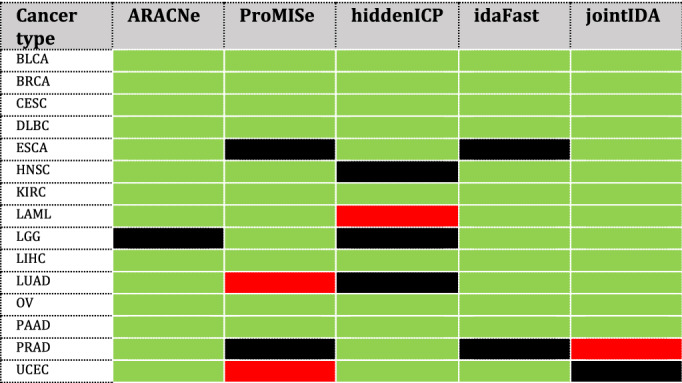
Comparison of *miRDriver* with other methods. We computed the overlapping miRNAs computed by *miRDriver* and each comparable method.

We checked if the count of the "*Significant miRNAs*" (i.e., miRNAs with target enrichment test *p*-value < 0.05) in *miRDriver* was more (i.e., *miRDriver* won), less (i.e., *miRDriver* lost), or equal (i.e., there was a draw) than the other method in the overlap. *miRDriver* had more "*Significant miRNAs*" than all other methods for most of the cancer types.

Green—*miRDriver* won; Red— *miRDriver* lost; Black—draw.

**Table 4:** Comparison results of *miRDriver* with five other methods in ovarian cancer.

Method	Input miRNAs	Input genes	Computed miRNAs	Selected genes	Eligible miRNAs^a^	Overlapping eligible miRNAs^b^	Method's computed miRNAs in overlap	*miRDriver*'s computed miRNAs in the overlap
miRDriver	198	2114	147	354	27	*NA*	*NA*	*NA*
ARACNe	198	2114	196	791	59	27	1	24
ProMise	198	2114	57	1938	34	22	0	17
hiddenICP	198	2114	198	2100	47	21	0	16
idaFast	50	1500	50	1194	32	22	0	17
jointIDA	50	1500	50	1294	32	22	0	17

^a^Eligible miRNAs had at least one known target in the ground truth data; ^b^Overlapping eligible miRNAs were with respect to *miRDriver*. For *miRDriver*, the number of significant miRNAs in every overlap with other methods was much higher. *NA* means not applicable.

### Computed genes were enriched in biological pathways, cancer hallmark and GO terms

To evaluate the functional roles of the computed target genes by *miRDriver* for each cancer type, we checked whether these genes were enriched in the biological pathways and GO terms^19^. For this purpose, we performed pathway enrichment analysis with the pathways in REACTOME^22^ and KEGG^23^ databases. For REACTOME pathway enrichment, we used R package *Pathfinder*^24^ and for KEGG pathways, hallmark gene set from the MSigDB^25,26^ database and GO enrichment, we used R package *clusterProfiler*^27^*.* We selected the pathways and GO terms with significant enrichment (multiple testing corrected, i.e., adjusted *p*-value < 0.05). We found 213 unique REACTOME pathways spanning over seventeen cancer types, twelve unique KEGG pathways in twelve cancer types and 224 unique enriched GO terms spanning over fifteen cancer types. Table 5 shows the enriched pathways and GO terms that were common in multiple cancer types. We provided the entire list of enriched pathways and GO terms for all the cancer types in Supplemental Table S3. Among these pathways, "*Immune System*" related pathways were found to play essential roles in cancer^28,29^. The G protein-coupled receptors (GPCRs)-related REACTOME pathways such as "*Signaling by GPCR"*, "*GPCR ligand binding"* and "*GPCR downstream signalling",* which were implicated in several cancer-related studies, were found to be enriched in the computed target genes in more than ten cancer types in our study. These pathways were found to play crucial roles in tumor development, invasion, migration, survival, and metastasis^30,31^. The GO terms, such as "*receptor ligand activity*" and "*receptor regulator activity*", enriched in at least five cancer types, were highlighted in several cancer studies for playing roles in drug toxicity, cell function, tumor growth^32–34^. The computed target genes in each cancer type were also enriched in the cancer hallmark gene set (Table 6).

**Table 5:** Enriched pathways and GO terms in pan-cancer.

REACTOME^a^	KEGG^b^	GO terms^c^
Immune System Metabolism ***Signal Transduction*** Innate Immune System Hemostasis Transport of small molecules Developmental Biology **Signaling by GPCR** Class A/1 (Rhodopsin-like receptors) ***GPCR ligand binding*** ***GPCR downstream signalling*** G alpha (i) signalling events Neuronal System	***Neuroactive Ligand Receptor Interaction*** Metabolism of Xenobiotics by Cytochrome P450 Steroid Hormone Biosynthesis Retinol Metabolism Drug Metabolism Cytochrome P450 Cytokine-Cytokine Receptor Interaction Systemic Lupus Erythematosus	***Receptor ligand activity*** ***Receptor regulator activity*** Ion gated channel activity Gated channel activity Cation channel activity Substrate-specific channel activity Passive transmembrane transporter activity Extracellular matrix Ion channel activity Nucleosome DNA packaging complex Nuclear nucleosome Protein-DNA complex Hormone activity

The pathways that appeared in more than four cancer types are in bold.

^a^REACTOME pathways, ^b^KEGG pathways and ^c^GO terms that were found to be enriched in at least two cancer types.

**Table 6:** Enriched cancer hallmark terms in pan-cancer for computed target genes.

Cancer type	Cancer Hallmark Terms	*p*-value
ACC	Epithelial Mesenchymal Transition	0.013
BRCA	Estrogen Response Late	0.003
BRCA	Estrogen Response Early	0.017
CESC	KRAS Signaling DN	0.022
CESC	HEDGEHOG Signaling	0.031
DLBC	KRAS Signaling DN	0.013
ESCA	Myogenesis	0.005
ESCA	Coagulation	0.007
HNSC	Myogenesis	0.009
KIRC	E2F Targets	0.000
KIRC	G2M Checkpoint	0.000
LAML	KRAS Signaling UP	0.002
LUAD	KRAS Signaling DN	0.007
PRAD	Myogenesis	0.017

Furthermore, *miRDriver* computed 22 common miRNAs that were shared in at least eight different cancer types among eighteen total cancer types used in the study (Table 7). The targets of these miRNAs could regulate the common biological processes in cancer. Hence, we performed a GO enrichment test with 1161 computed genes targeted by at least one of these 22 miRNAs among eighteen cancer types and found 49 GO terms with significant enrichment. Table 8 shows a few of these GO terms with their cancer-related citations; the entire list can be found in Supplemental Table S4.

**Table 7:**
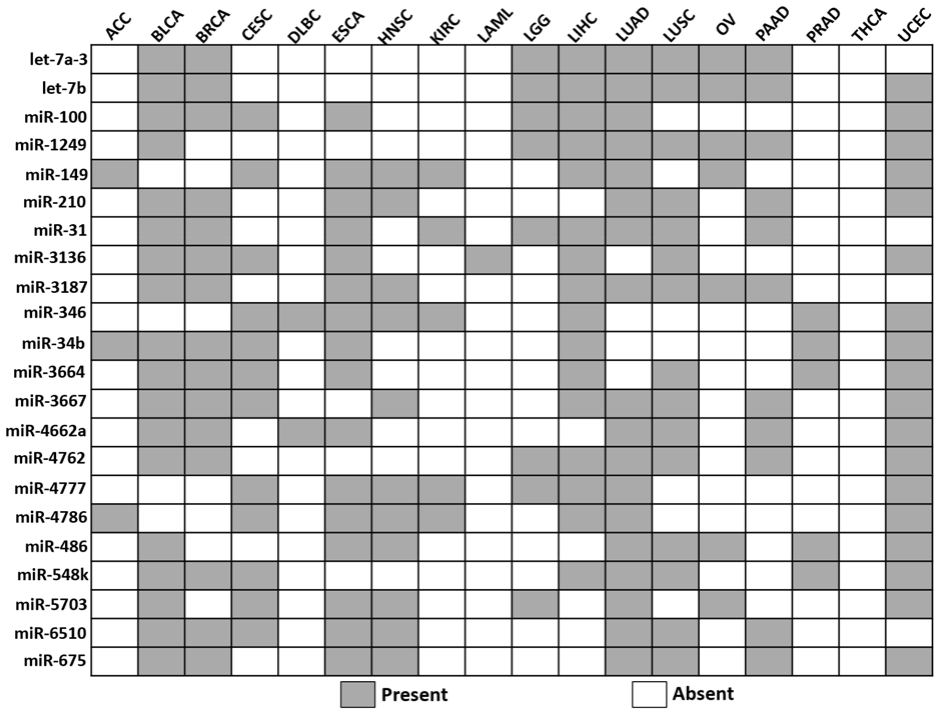
Twenty two common miRNAs computed by *miRDriver* in multiple cancer types.

**Table 8:** Enriched GO terms with the cancer-related citations in the targets of the common miRNAs in Table 7.

GO-ID	Description	Adjusted *p*-value
GO:000633	DNA replication-dependent nucleosome assembly^4,45^	5.25e−07
GO:0006342	Chromatin silencing^47,48^	1.17e−03
GO:0006323	DNA packaging^49,50^	5.29e−05
GO:0045814	Negative regulation of gene expression, epigenetic^51,52^	2.0894e−03
GO:0060964	Regulation of gene silencing by miRNA^53,54^	2.362e−02
GO:0060147	Regulation of post-transcriptional gene silencing^55,56^	2.767e−02
GO:0048018	Receptor ligand activity^32–34^	3.377e−03

Although there were common miRNAs across multiple cancer types, there were not many common miRNA-gene interactions due to a much higher number of *trans* genes than the miRNAs in this pan-cancer analysis. Table 9 presents fourteen common gene-miRNA interactions shared in two cancer types among 11,548 selected interactions from pan-cancer. Among these, RSPO3 and miR-22 interaction have been selected in LAML (leukemia) and LUAD (lung cancer). Interestingly, RSPO3 was found to play a role in leukemia^35^ and promote tumors in lung cancer^36^. miR-22 was found to play the anti-tumor role with therapeutic potential in acute myeloid leukemia^37^ and found to have roles in lung cancer via CNAs^38^. Another interaction PAX5 with miR-5699 was found in BLCA (bladder cancer) and OV (ovarian). Interestingly, PAX5 was found to have a role in bladder cancer^39^ and ovarian cancer^40^ as a co-regulator of PAX8. miR-5699 has a proven role in ovarian cancer treatment's oxidative response^41^. There are some miRNA-long noncoding RNA (lncRNA) interactions in Table 9. lncRNAs are known to have binding sites for miRNAs, also lncRNAs can be direct–indirect targets of miRNAs^42,43^. Several lncRNAs were found to be prevalent in cancer^44^. In our case, LINC01833- miR-1226, was found in BRCA (breast cancer) and LGG (brain cancer). LINC01833 was listed in the top five lncRNAs according to the prioritization of variation in ER-negative-associated lncRNAs in breast cancer^45^. miR-1266 was found to regulate the expression of the mucin 1 oncoprotein and induce cell death in a breast cancer study^46^.

**Table 9:** miRNA-gene interactions computed by *miRDriver* in multiple cancer types. Cancer type column shows in which cancer types the interactions are present.

Gene	miRNA	Cancer type
RSPO3	miR-22	LAML,LUAD
PAX5	miR-5699	BLCA,OV
LINC01833	miR-1226	BRCA,LGG
LINC01697	miR-5703	HNSC,UCEC
HIST1H4L	miR-3613	BLCA,LUAD
LINC02489	miR-375	CESC,OV
NR0B1	miR-346	HNSC,KIRC
GABRG2	miR-744	PAAD,UCEC
PLAC8	miR-6510	CESC,HNSC
BPIFC	miR-4469	LUSC, UCEC
RTL3	miR-26b	CESC,UCEC
SLC17A2	miR-5699	LUSC,PAAD

### Several cancer-related terms and pathways were enriched in the targets of the computed miRNAs

We checked the involvement of the computed miRNAs in cancer-related pathways. For this analysis, we collected all 556 miRNAs that were computed by *miRDriver* in at least one of the cancer type. We collected the computed target genes for each of these miRNAs from all the cancer types where that miRNA was present. We performed cancer hallmark gene set enrichment with these collected target genes of each miRNA. We found 38 unique enriched cancer hallmark terms (adjusted *p*-value < 0.05) for 134 miRNAs (Supplemental Table S5).

We also performed REACTOME pathway enrichment analysis with these collected target genes of each miRNA. We found 240 unique enriched REACTOME pathways (adjusted *p*-value < 0.05) for 69 miRNAs with these target genes (Supplemental Table S5). Eleven of these enriched pathways, such as, "*Epithelial-Mesenchymal Transition*", "*Hypoxia*", "*Inflammatory Response*", "*KRAS Signaling Up*", "*p53 Pathway*", "*P13 AKT MTOR Signaling*", "*Xenobiotic Metabolism*", "*Apoptosis*", "*DNA Repair*" and "*Immune*" were present in nineteen experimentally-validated cancer-related pathways for miRNAs^57^.

Furthermore, we performed an analysis to find *cancer-driving* miRNAs (i.e., tumor-suppressor, oncogenes or both) using the enriched cancer hallmark terms (Supplemental Table S5). We hypothesized that a miRNA could be a candidate *cancer-driving* miRNA if its target genes that were found to be enriched in the cancer hallmark terms could also be enriched in the known *cancer-driving* genes. Hence, for each of the enriched cancer hallmark terms, we gathered all the miRNAs with their target genes for which that term was enriched (Table 10). We downloaded a list of 83 *cancer-driving* genes found to be frequently mutated in different cancer types from the Catalogue Of Somatic Mutation In Cancer (COSMIC) database from the cancer gene census project^58^. We performed a hypergeometric test for the overlapping target genes with the 83 *cancer-driving* genes for each cancer hallmark term. The background gene set for this test was all 5604 target genes computed by *miRDriver* in pan-cancer. We considered the miRNAs related to the hypergeometric *p*-value < 0.05 as the candidate miRNAs to be evaluated as *cancer-driving* miRNAs since their targets were enriched in known *cancer-driving* genes. Furthermore, considering the fact that the up- or down-regulation of a miRNA causes the inverse regulation of its target genes^59–61^, we specifically checked the target genes of these candidate miRNAs for different cancer types that were found to have negative LASSO regression coefficient computed by *miRDriver* (Table 11). Interestingly, all of the target genes in this group (Table 11), except OLIG2, were found to be oncogene in the previous studies^62–68^. OLIG2 was found to be working as a tumor-suppressor gene (TSG) in human glioblastoma^69^. All the miRNAs except miR-5001 and miR-2276 were found to act as TSGs in cancer in several studies^70–74^. miR-5001 and miR-2276 were found to have evidence of working as oncogenes in endometrial cancer and colorectal cancer, respectively^75,76^. These studies support the findings of *miRDriver* in terms of connecting miRNAs and genes that were related inversely, having a possibility to be working as *drivers* in pairs of TSG-oncogene in different cancer types.

**Table 10:** Hallmark term-related target enrichment in cancer driver genes.

Hallmark	miRNAs^a^	Targets^b^	Overlap^c^	*p*-value^d^
Complement	5	42	3	0.018
E2F Targets	2	85	4	0.026
MTORC1 Signaling	1	12	2	0.011
Myogenesis	12	44	3	0.020
P53 Pathway	1	12	2	0.011
TNFA Signaling via NFKB	2	17	2	0.021
Pancreas Beta Cells	2	48	3	0.026

^a^No. of miRNAs in cancer hallmark term; ^b^No. of targets in the term; ^c^No. of overlapping targets in the cancer driver genes; ^d^Hypergeometric *p*-value of the overlap.

**Table 11:** miRNA targets with negative LASSO coefficient in different cancer types.

Cancer type	Target	miRNA
KIRC	COL1A1	miR-4728
KIRC	CYSLTR2	miR-346
KIRC	CYSLTR2	miR-4728
KIRC	ETV4	miR-4728
CESC	ISX	miR-5001
UCEC	ISX	miR-2276
UCEC	ISX	miR-4733
UCEC	ISX	miR-6842
PAAD	KCNJ5	miR-5699
KIRC	NTRK1	miR-4728
HNSC	OLIG2	miR-5699

### Computed target genes revealed the subtype-specific expression signature in multiple cancer types

We checked the subtype-specific association of gene expression of computed target genes in BRCA, LGG, LUSC and PAAD. We used the R package *TCGAbiolinks*^77^ to download the different subtype labels for the different cancer types. Since TPM (transcript per million reads) values are normalized and comparable across samples, for this analysis, we utilized RNA-Seq data in TPM of TCGA samples whose subtype labels were available. We applied log2(TPM + 1) transformation from Cancer Dependency Map [https://depmap.org]. For all these cancer types, we performed unsupervised clustering using gene expression of these target genes and compared these clusters with baseline (i.e., known) subtype clusters using Rand Index (RI) and *Uniform Manifold Approximation and Projection* (UMAP)^78^ plots.

**Figure 2:**
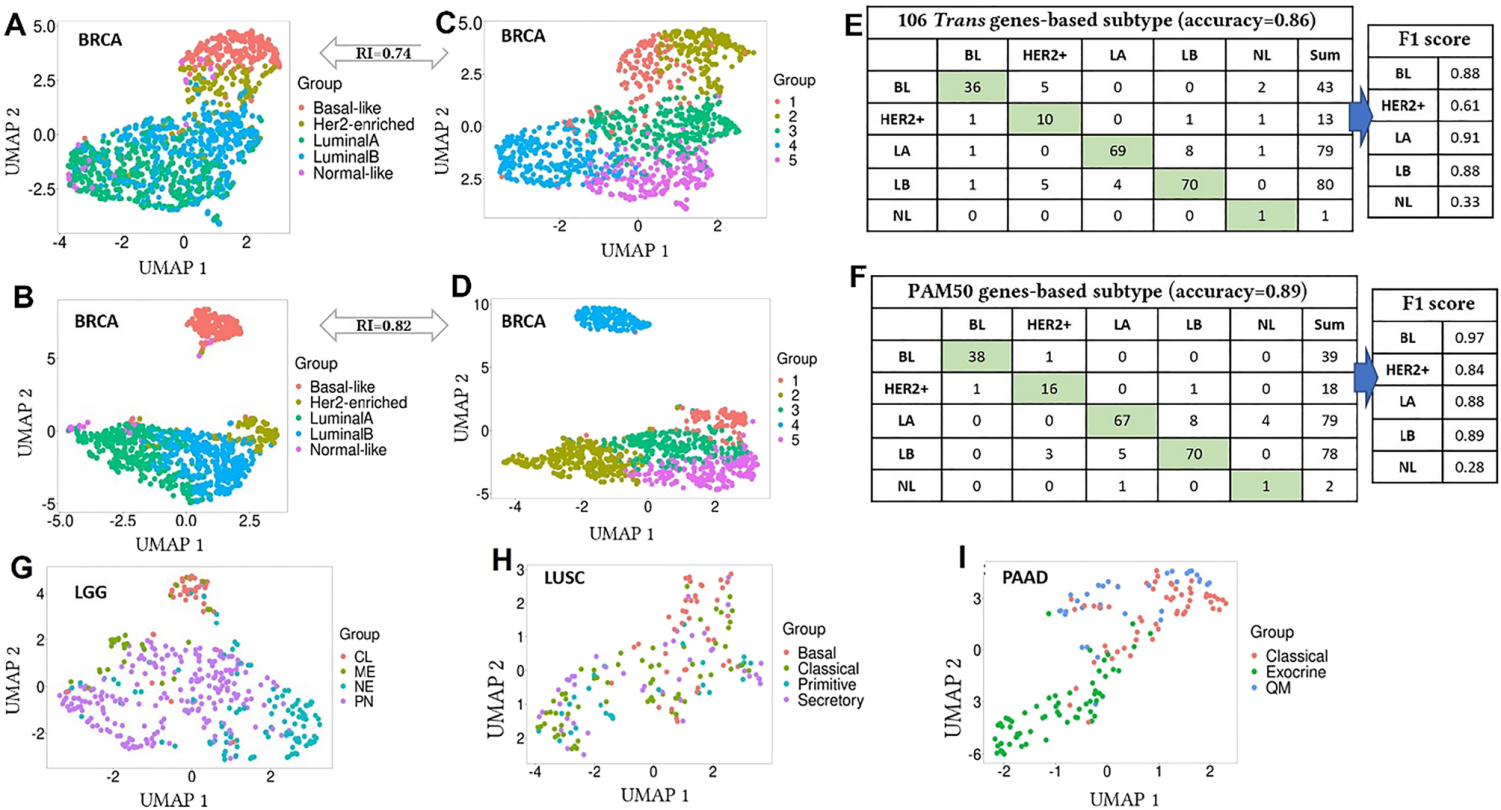
UMAP plots and confusion matrices are summarizing the classification and clustering of the cancer samples. (**A**, **B**) UMAP plots with high-degree target genes in BRCA with baseline and k-means clustering labels, respectively; (**C**, **D**) UMAP plots with PAM50 genes in BRCA with baseline and k-means clustering labels, respectively; (**E**, **F**) Confusion matrices of subtype-classification in BRCA with F1 scores with respect to the baseline labels, using high-degree target genes and PAM50 genes, respectively. Accuracy and F1 score were closer in both cases; (**G**) UMAP plot with all target genes using transcriptome-based baseline labels in LGG; (**H**) UMAP plots with high-degree target genes using expression-based baseline labels in LUSC; (**I**) UMAP plots with high-degree target genes using mRNA-based clusters^81^ as a baseline in PAAD.

For BRCA, we computed a UMAP plot using around 1000 BRCA samples and 106 high-degree genes (i.e., computed genes targeted by more than three miRNAs) to check the PAM50 gene-based subtypes^79^. These subtypes were, Basal-like (BL), HER2-enriched (HER2+), LuminalA (LA), LuminalB (LB) and Normal-like (NL) (Fig. 2A). We also computed the UMAP plot using the PAM50 genes with PAM50 gene-based subtypes (Fig. 2B). These UMAP plots show a clear separation between different subtype-specific clusters. We also performed an unsupervised clustering (*k-means*) (with R base package *Stats* with k = 5 and all other parameters as default) on the BRCA cohort with high-degree target genes (Fig. 2C) and with PAM50 genes (Fig. 2D). The computed RIs between five known subtype labels with the five predicted clusters by computed high-degree target genes and PAM50 genes were 0.74 and 0.82, respectively. This result shows that both the computed high-degree target genes and PAM50 gene set were able to detect subtype structure in BRCA samples with high accuracy.

Furthermore, we used the high-degree genes to classify the BRCA cohort into five different classes. For this purpose, we used R package *keras*^80^ (https://github.com/rstudio/keras) implementation of the *Random Forest* classifier with 80% samples for training with 10-fold cross-validation where 20% of data was held out to test the performance of the model. We achieved a high classification accuracy of 0.86. The same sample cohort was classified with PAM50 genes and achieved a classification accuracy of 0.89. Figure 2E,F present the confusion matrices for both cases with F1 scores. The F1 scores for the classification with high-degree target genes were comparable to F1 scores of the PAM50-based classification, which suggests that these high-degree target genes can serve as potential markers for PAM50-based subtype signatures in BRCA.

For the other cancer types except for LGG, we computed UMAP plots to check the baseline subtype clusters with the selected high-degree target genes. For these cancer types, since there was a fewer number of genes targeted by more than three miRNAs, we defined high-degree genes as the genes targeted by more than two miRNAs. For LGG, we used 402 samples with all 151 computed target genes since no gene was targeted by multiple miRNAs (Fig. 2G). For LUSC, we used 178 patient samples with 75 high-degree target genes (Fig. 2H), and in PAAD, we used 150 patient samples with 101 selected high-degree target genes (Fig. 2I). We also performed k-means clustering for all these cancer types. For LGG, LUSC and PAAD, the computed RIs between known subtype clusters with the predicted clusters were 0.71, 0.62 and 0.70, respectively. For LGG and PAAD in which we achieved high RI values, we visualized clear separation among the known subtype-specific clusters based on UMAP plots. For LUSC, although we achieved a lower RI value, the "*Basal*" cluster was separated from other clusters (Fig. 2H). These results showed that the computed high-degree target genes could reveal subtype-specific expression signatures in multiple cancer types.

### Computed miRNAs were found to be potential biomarkers for patients' survival and progression of the disease in each cancer type

**Figure 3:**
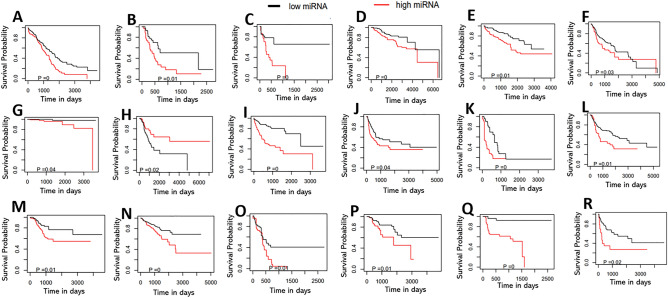
Adjusted Kaplan–Meier plots with adjusted log-rank test *p*-value for 18 common miRNAs in high and low expression groups, (**A**) let-7a-3 in OV with OS; (**B**) let-7b in PAAD with OS; (**C**) miR-149 in ACC with PFI; (**D**) miR-210 in BRCA with OS; (**E**) miR-31 in KIRC with OS; (**F**) miR-3187 in HNSC with OS; (**G**) miR-3664 in PRAD with OS; (**H**) miR-4777 in LUAD with DFI; (**I**) miR-4786 in LIHC with OS; (**J**) miR-3136 in BLCA with PFI; (**K**) miR-34b in ESCA with PFI; (**L**) miR-3667 in LUSC with PFI; M) miR-4662a in UCEC with PFI; (**N**) miR-548k in PRAD with PFI; (**O**) miR-6510 in PAAD with PFI; (**P**) miR-4762 in LUSC with DFI; (**Q**) miR-486 in HNSC with DFI; (**R**) miR-675 in ACC with PFI.

We performed survival analysis with the computed miRNAs to assess the miRNAs' prognostic relevance as clinical biomarkers for patients' survival (Fig. 3). For each miRNA, we divided the patient cohort of each cancer type into two groups, such as *high expression* and *low expression* for that miRNA. We considered the available clinical variables among age, race, gender, stage, and grade as independent variables (see Materials and Methods). To remove the confounding effect of multiple factors, we used the Adjusted Kaplan–Meier Estimator and computed adjusted survival curves by weighting the individual contributions by the inverse probability weighting (IPW) using the R package *IPWsurvival*^82^. We considered four different survival endpoints, namely, Overall Survival (OS), Progression Free Interval (PFI), Disease Specific Survival (DSS) and Disease Free Interval (DFI) (see Materials and Methods). We found several prognostic miRNAs (adjusted log-rank test *p*-value < 0.05) based on *Adjusted Kaplan–Meier* survival plots in multiple cancer types. Figure 3 shows the survival plots for the common miRNAs in different cancer types. Among 22 common miRNAs (Table 7), eighteen had significant survival differences in high and low miRNA expression patient groups in at least one cancer type (Fig. 3). We provided the survival plots for all miRNAs for eighteen cancer types in Supplemental Figure S1–S18.

### miRDriver discovered several cancer-specific miRNAs

In this study, *miRDriver* discovered 240 cancer-specific miRNAs, i.e., these miRNAs were selected in only one cancer type. We used the R package *OncoScore*^83^ to measure these miRNAs' association with cancer based on citation frequencies in cancer-related biomedical literature. Fifty percent of these miRNAs (i.e., 121) were found to be cited in cancer-related studies (Supplemental Table S6). Moreover, several of these miRNAs were found to be prognostic, i.e., associated with patients' survival based on *Adjusted Kaplan–Meier* survival analysis (adjusted log-rank test *p*-value < 0.05) (Table 12).

**Table 12:** Cancer-specific *miRDriver* miRNAs with citation frequency.

Cancer type	miRNA^a^	Citation^b^	Cancer type	miRNA^a^	Citation^b^
LIHC	miR-1288	2	BLCA	miR-3677	4
HNSC	miR-134	56	LUSC	miR-3934	1
KIRC	miR-194-1	1	BLCA	miR-4791	1
UCEC	miR-195	197	BLCA	miR-5003	1
KIRC	miR-215	69	UCEC	miR-552	12
LIHC	miR-3170	1	HNSC	miR-561	6
LUAD	miR-3651	1	PAAD	miR-6875	3

^a^These miRNAs were prognostically significant in survival analysis; ^b^OncoScore citation frequency.

### The copy number changes of the computed miRNAs were predictive of their expressions

We computed the Spearman correlation values between copy number and expression across all the samples of the computed miRNAs of *miRDriver* in eighteen different cancer types (Supplemental Figure S19). As expected, we observed that most miRNAs had a positive correlation between their copy number and expression. There were also some negative correlations, but this is not surprising as miRNA expression is dependent on regulatory factors beyond copy number events, too. Despite this, the positive median distribution of correlations across all cancer types supports our hypothesis that miRNA expression in copy number areas may be predictive of DE *trans* gene expression variation.

### Selected high-degree genes were highly significant as potential biomarkers to predict prognosis in cancer patients than low-degree genes in several cancer types

**Figure 4:**
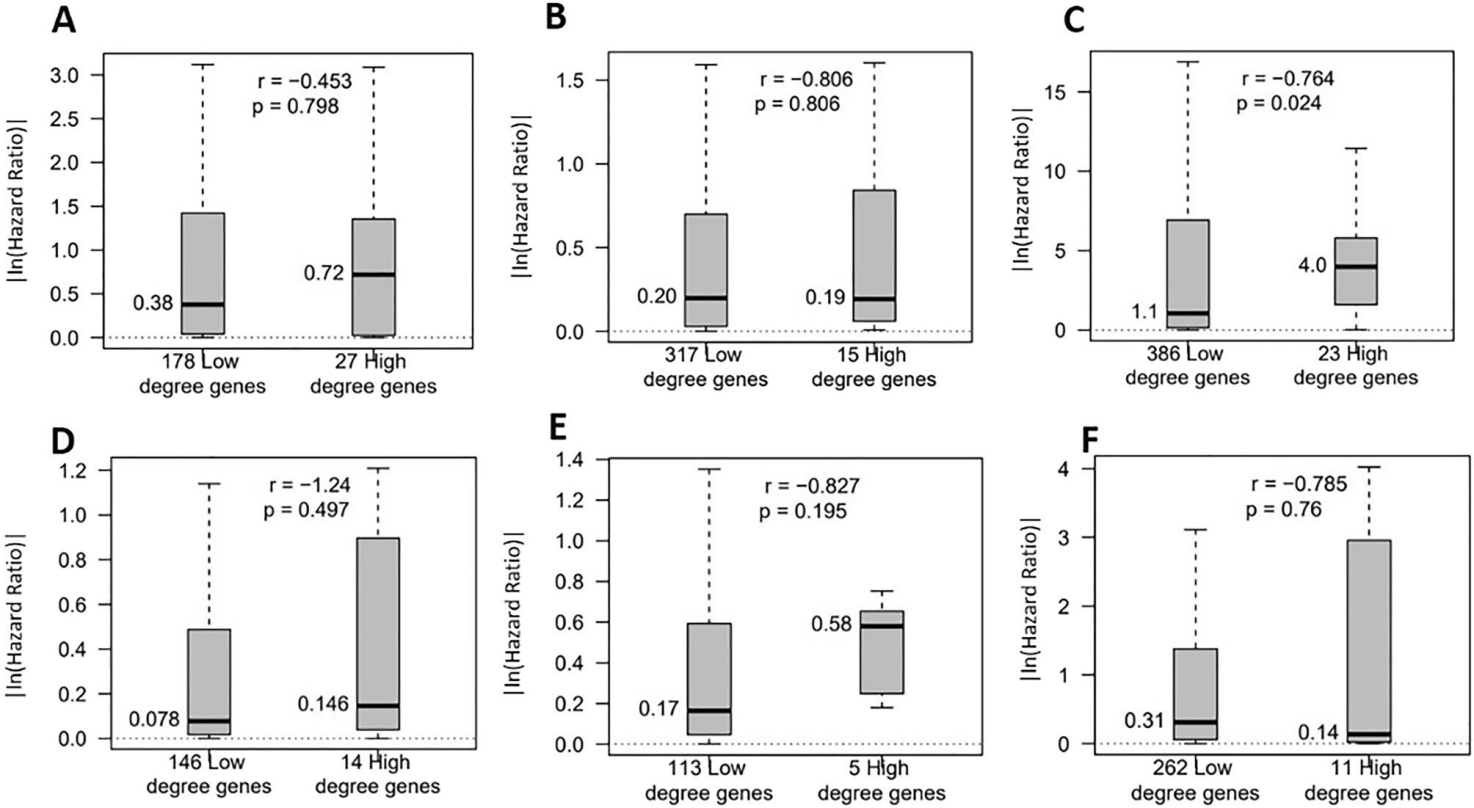
Boxplots of absolute values of the natural logarithm of hazard ratios in high-degree and low-degree genes with an r-value of Mann–Whitney test, (**A**) LUSC with OS, (**B**) BLCA with DSS, (**C**) ESCA with DFI, (**D**) HNSC with OS, (**E**) LGG with OS, (**F**) PAAD with OS. These plots show that computed high-degree genes were having higher |ln (Hazard Ratio) (*r*-value < 0) to predict disease survival and prognosis in cancer patients than low-degree genes.

We computed the hazard ratio (HR) of the selected high-degree target genes as the genes targeted by four or more miRNAs and low-degree target genes as the genes targeted by only one miRNA to get the optimized list of high-degree and low-degree genes. We performed the multivariate Cox regression analysis^84^ using these genes. Due to the low sample size of the high-degree target genes, we computed effect size using the *r-value* of the *Mann–Whitney* test with |ln (HR)|. Higher |ln (HR)| implies a higher association with an event's risk with an increase or decrease of gene expression. The *r-value* was negative if the |ln (HR)| values in the high-degree group were higher than the low-degree group and positive otherwise. We used OS, PFI, DSS and DFI as clinical endpoints in this analysis. We ran this analysis on fifteen different cancer types omitting the cancer types with no high-degree target gene (THCA and PRAD) and no clinical endpoint (LAML). In our previous work^17^ with BRCA and OV, we discussed the significance of high-degree target genes; hence, we omitted these two cancer types as well, leaving us thirteen cancer types for this analysis. Although the Wilcoxon rank-sum test *p-values* for the comparison between the boxplots of the two groups were insignificant (p-value > 0.05), we found negative *r-values* in most of the cancer types (see Fig. 4). The hazard ratio boxplots of all thirteen cancer types with *r-values* in different clinical endpoints can be found in Supplemental Figure S20–S23. Table 13 shows the high-degree target genes with OS in seven cancer types that had negative r-values. These genes were found to be cited in cancer-related work in a high percentage (≥ 50%) among total citations in biomedical literature by OncoScore. The entire list of high-degree genes with OncoScore frequencies has been provided in Supplemental Table S7.

**Table 13:** Cancer types with negative *r-values* from the ^a^Mann-Whitney test between low-degree and high-degree gene groups; ^b^Highly cited high-degree genes in these cancer types in cancer-related literature.

Cancer type	^a^r-value	^b^High degree genes
BLCA	− 0.76	BTNL3, HNF1A-AS1, MIR1205, NAA11, NOL4, OR10H5, PDZD3
ESCA	− 0.26	ANKRD26P3, C17orf64, CCDC60, FAM81B, LIN28A, MYLKP1
HNSC	− 1.24	BTBD17, DNM3OS, KLHL33, SMCO1
KIRC	− 0.03	HOTTIP
LGG	− 0.83	C20orf85, C7orf65
PAAD	− 0.79	ARHGAP36, C1QTNF1-AS1, TMPRSS15

## Materials and methods

All the experiments were conducted in accordance with relevant guidelines and regulations.

### Running miRDriver on pan-cancer

In this study, we conducted a pan-cancer analysis where we applied the *miRDriver* R package to identify copy-number derived miRNA-gene interactions. We integrated gene expression, CNA, DNA methylation, TF-gene interactions and miRNA expression data from eighteen different cancer types (Table 1). *miRDriver* has four computational steps: GISTIC Step, DE Step, REGULATOR Step, and *LASSO* Step. In the following paragraphs, we described the *miRDriver* R functions to run these steps. The entire pipeline of *miRDriver* running on pan-cancer is illustrated in Fig. 1.

To mine miRNAs that reside in the aberrated chromosomal regions of cancer patients, in the first step (i.e., *GISTIC* Step), we computed frequently aberrated chromosomal regions, namely, *GISTIC regions*, for eighteen different cancer cohorts. We utilized segmented chromosomal copy number profiles of each cancer cohort as inputs in GISTIC 2.0^85^ tool in *GenePattern*^86^ webserver and computed chromosomal regions that were frequently aberrated within each patient cohort using a confidence interval of 0.90. The GISTIC regions with a ${\mathrm{log}}_{2}$ ratio above 0.1 were considered amplified and the GISTIC regions with a ${\mathrm{log}}_{2}$ ratio below − $0.1$ were considered deleted. We further processed the GISTIC regions of each cancer type using the *getRegionWiseGistic* function in the *miRDriver* R package to gather patients from each region with their aberration status (i.e., aberrated and non-aberrated).

In the second step (i.e., the DE Step), we computed DE genes for each GISTIC region. We computed these DE genes between frequently aberrated and non-aberrated patient sample groups in each cancer type cohort using *getDifferentiallyExpressedGenes* function in *miRDrive*r with default parameters. This function employed *edgeR*^87^ package in R utilizing mRNA raw counts to compute DE genes among these two groups using absolute log fold change (logFC) ≥ 1 and adjusted *p*-value < 0.05. Using the *makingCisAndTransGenes* function, we annotated DE genes located inside the GISTIC region as *cis* genes and DE genes outside of the GISTIC region as *trans* genes. This step also retrieves the miRNAs (i.e., *cis* miRNAs) in each GISTIC region. Since the number of *cis* miRNAs per GISTIC region was extremely low, to avoid reducing the sensitivity and precision of our findings, we did not further filter *cis* miRNAs based on differential expression. The counts of *trans* genes, *cis* genes and *cis* miRNAs for each GISTIC region in eighteen different cancer types can be accessed from Supplemental Table S8.

In the *REGULATOR* Step (i.e., the third step) of *miRDriver*, we collected all the potential predictors, namely, *cis* genes, *cis* miRNAs, gene-centric copy number data, gene-centric DNA methylation beta values and TFs in each cancer type that could influence each DE *trans* gene's expression. We used the *getTransGenePredictorFile* function to gather all the predictors. This function only considered those *trans* genes that had at least one *cis* miRNA as a possible predictor.

In the *LASSO* Step, we computed the potential *cis* miRNAs that regulate the DE *trans* genes' expression variation. We used the *lassoParallelForTransGene* function in the *miRDriver* R package that utilized R package *glmnet*^88^ to perform LASSO to compute miRNA regulators of the DE *trans* genes. This function considered the gene-centric copy number, gene-centric DNA methylation, TFs, miRNA expression as independent variables and the *trans* gene's expression as the response variable. For each *trans* gene, out of all its candidate predictors (i.e., independent variables), LASSO selected a set of non-zero coefficient predictors. Since the independent variables selected by LASSO have been shown to be inconsistent, especially when the sample size gets large^89^, we ran LASSO 100 times for each *trans* gene and kept the *cis* miRNAs selected by LASSO at least 70 times. We found that miRNAs with threshold 70 to be the most consistent set of potential regulator miRNAs to be considered in the computed miRNA-gene interaction networks in each cancer type cohort (Supplemental Fig. S24). To optimize the regularization parameter λ of LASSO, for each of 100 runs, we applied 10-fold cross-validation and picked λ that provided the simplest model with the minimum cross-validation error.

Although miRNAs typically cause the inverse regulation of their target genes^59–61^, *miRDriver* considers both positively and negatively correlated miRNA-target pairs for each cancer type. Since *miRDriver* computes miRNA-gene interactions that could be direct or indirect interactions, a positive correlation between them is also possible. Furthermore, a positive correlation between miRNAs and their direct targets is also possible^90–93^. The computed miRNA-gene interactions in eighteen different cancer types can be accessed from Supplemental Table S9.

### Running state-of-the-art-methods

We compared *miRDriver* with five state-of-the-art methods, namely, ARACNe^5^, ProMISe^6^, HiddenICP^7^, idaFast^8^ and jointIDA^9^ by running them on datasets from eighteen cancer types in TCGA. Since these methods can only utilize gene expression data, we used gene expression data to compute miRNA-gene interaction networks for our comparison For ARACNe, ProMIse and hiddenICP, we used the same number of input genes and miRNAs that we used in *miRDriver* for each cancer type. Since idaFast and jointIDA methods have high computational complexity and therefore are not scalable to large datasets, we run these two methods with ≤ 50 top miRNAs and ≤ 1500 top genes selected by Feature Selection Based on The Most Variant Median Absolute Deviation (FSbyMAD)^94^ for each cancer type. After running ARACNe, we selected all of the miRNA-gene interactions that had non-zero scores to be compared with our method. For ProMIse, hiddenICP, idaFast and jointIDA, we considered the top 3, 3, 3.5 and 3.5 percentile of miRNA-gene interactions based on reported scores, respectively. Based on our previous work with the breast cancer cohort, these thresholds were chosen to get highly confident gene-miRNA interactions for comparison and were used for all eighteen different cancer types. The details of running these methods can be found in our previous publication^17^.

### Datasets to run miRDriver on pan-cancer

In this study, we utilized gene expression, CNA, DNA methylation, TF-gene interaction and miRNA expression data from eighteen different cancer types. We used the R Bioconductor package *TCGAbiolinks*^77^ to download the genomic data of cancer patient samples from TCGA. We retrieved gene expression quantification data for raw count (Illumina HiSeq) and RNA sequencing data with FPKM (Fragments Per Kilobase of the transcript, per Million, mapped reads) for all the cancer types. TCGA gene expression data consist of mRNAs (i.e., messenger RNAs), lncRNAs, and pseudogenes. Thus, our analysis considered all these RNAs.

We downloaded miRNAs' gene quantification expression with file type hg19.mirbase20.mirna and isoform gene quantification data with file type hg19.mirbase20.isoform from the legacy data of TCGA. For each cancer type, we used the miRNAs that have ≥ 0.01 RPM (reads per million mapped reads) value across ≥ 30% of the cohort.

We retrieved masked copy number variation (Affymetrix SNP Array 6.0) and computed the gene-centric copy number value compatible with hg38 using the R Bioconductor package *CNTools*^95^.

We downloaded DNA methylation data of Infinium HumanMethylation27 Bead-Chip (27K) and Infinium HumanMethylation450 Bead-Chip (450K) platforms from TCGA. Gene-specific beta values were calculated separately for both platforms. For the 450K platform, the average beta value for promoter-specific probes was considered due to their role in transcriptional silencing^96^. Given lower coverage in the 27K platform, we utilized all the probes. In this case, we set the DNA methylation of a gene as the average beta values of all its probes.

We downloaded experimentally-validated TF-gene interactions from TRED and TRRUST databases to incorporate TF-gene interactions in the LASSO step. Table 1 shows the sample sizes of different data modalities used in this study for eighteen different cancer types from TCGA.

### Datasets to evaluate miRDriver

To check the correlation between copy number and expression across all the samples of the computed miRNAs of *miRDriver*, we used TCGA*'*s masked copy number variation (Affymetrix SNP Array 6.0) data. We utilized the R Bioconductor package *CNTools*^95^ to compute the miRNA-centric copy number value by giving miRNA coordinates extracted from the TCGA's legacy data file type hg19.mirbase20.isoform.

To evaluate if the miRNAs computed by *miRDriver* were enriched in cancer-related miRNAs, we downloaded a list of 351 known oncogenic miRNAs from the oncomiRDB database^97^. Each miRNA listed in oncomiRDB is involved in at least one cancer-related phenotype or cellular process. We harmonized the names of oncomiRDB miRNAs regarding the miRBase^98^ database.

To check if the miRNA-gene interactions computed by *miRDriver* were significantly enriched in the known miRNA-gene interactions, we performed a hypergeometric test for the computed target genes of each miRNA. For this purpose, we compiled a list of experimentally-validated miRNA-gene interactions from *miRTarBasev6.1*, *TarBasev7.0* and *miRWalk* databases^99^ as our ground truth data. Considering that *miRDriver* could compute direct targets and the indirect downstream targets (i.e., targets of the direct targets), we included potential indirect targets to the ground truth dataset. Hence, for each miRNA-gene interaction where the gene was a known TF, we included the experimentally-validated targets of this TF obtained from *TRED* and *TRRUST* databases.

To assess the prognostic relevance of the *miRDriver*-selected miRNAs as clinical biomarkers, we performed multivariate survival analysis^82^ and multivariate Cox regression^84^. We downloaded the clinical data for eighteen different cancer types using *TCGAbiolinks*^77^. We considered the available clinical variables from age, race, gender, stage, and grade as independent variables whenever available (see Table 14).

**Table 14:**
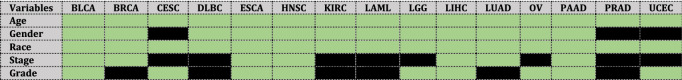
Availability of clinical variables in TCGA.

Green—Available; Black—Unavailable.

We considered four different endpoints, namely, OS, PFI, DSS and DFI. In OS, patients who were dead from any cause were considered as dead, otherwise censored. In PFI, patients having new tumor event whether it was a progression of the disease, local recurrence, distant metastasis, new primary tumor event, or died with cancer without new tumor event, including cases with a new tumor event whose type is N/A were considered as "event occurred" and all other patients were censored. DFI was similar to PFI with the inclusion of censored patients with new primary tumors in other organs; patients who were dead with tumors without new tumor event and patients with stage IV were excluded. In DSS, disease-specific survival time in days, last contact days, or death days, whichever was larger, was used to identify "event occurred" versus censored patients^100^.

We checked the subtype-specific association of gene expression of computed target genes in BRCA, LGG, KIRC, LUSC and PAAD. We used the R package *TCGAbiolinks*^77^ to download the different subtype labels for the different cancer types.

## Discussion

We developed a computational pipeline called *miRDriver*, which integrates multi-omics datasets such as CNA, DNA methylation, TFs, gene, and miRNA expression to infer copy number-derived miRNA-gene interactions in cancer. In the current study, we extended the use of *miRDriver* with an R package and carried out a comprehensive and rigorous analysis of the pan-cancer characterization of TCGA samples to infer miRNA-gene interaction networks integrating multi-omics datasets. We focused on DNA aberration regions of 7294 cancer samples associated with eighteen different cancer types uncovering the tissue-specific omics interplay in establishing the miRNA–gene associations. *miRDriver* outperformed several existing methods in all different cancer types used in the study. In each case, *miRDriver* was able to select many miRNA-gene interactions enriched in known miRNA-target databases. We observed that selected miRNAs by *miRDriver* were significantly enriched in the known cancer-related miRNAs.

Several cancer-related biological pathways and GO terms were found to be enriched in the computed genes. Among these, GPCR-related pathways, which play crucial roles in tumor development, invasion, migration, survival, and metastasis, were enriched in ten or more cancer types. More than 40% of the total computed genes were cited in cancer-related studies based on OncoScore frequency. Among these, at least 50% of genes had more than ten cancer-related citations.

We highlighted 22 common miRNAs that were frequently selected in multiple cancer types and explored their prognostic roles. Several of these miRNAs had significant survival differences in high and low-expression patient sample groups. Among these, miRNAs belonging to the let-7 family were found to act as both tumor suppressors and oncogene in several studies^101^. miR-100, miR-149, miR-210, miR-31, miR-346, miR-34b, miR-486 and miR-675 were cited in cancer-related studies with high OncoScore frequency. We found several enriched GO terms with the computed targets of these 22 common miRNAs. Among these, GO terms such as "*Regulation of gene silencing by miRNA*" and "*Regulation of post-transcriptional gene silencing*" were implicated in several cancer-related studies explaining the miRNAs' roles in cancer initiation and progression ^53,102^. The GO term "*Chromatin silencing*" was involved in cancer ^49,103^. The GO term "*DNA replication-dependent nucleosome assembly*" has been studied concerning cell fate and differentiation regulation and suggested to be explored in cancer in a recent study^104^.

We also assessed these common miRNAs as non-invasive biomarkers, such as the presence of these miRNAs as the circulating miRNAs that can be detected in organic liquids effectively after getting discharged by the tumor cells. For this purpose, we submitted these 22 miRNAs to the MiRandola^105^ database as a knowledge base for extracellular circulating miRNAs for inferring their relevance as non-invasive biomarkers. We found ten out of 22 common miRNAs, namely let-7b, miR-100, miR-1249, miR-149, miR-210, miR-31, miR-346, miR-34b, miR-486 and miR-675, to be as potent non-invasive biomarkers.

Although there were common miRNAs across multiple cancer types, there were not many common miRNA-gene interactions. Only fourteen common interactions were shared in at least two cancer types among ~ 10,000 computed interactions. Considering the much higher number of target genes than the miRNAs used in this analysis, these findings were not surprising. We discussed several of these interactions that were found to be in experimental studies.

We identified several cancer driver genes targeted by multiple miRNAs (i.e., high-degree genes) across different cancer types. Also, high-degree target genes have been shown to have a strong association with the molecular subtypes in multiple cancer types, such as BRCA, LGG, LUSC and PAAD. Specifically, in BRCA, 106 high-degree genes (three genes were common with PAM50 genes) were found to serve as subtype-specific gene signatures with high classification accuracy with respect to the baseline PAM50 gene-based subtypes. We compared the prognostic significance of low-degree target genes with high-degree target genes in the disease progression and survival hazards. We discovered high-degree genes to be more significant prognostic factors than low-degree genes. These findings point out that multiple miRNAs in coordination can impact the gene expression stronger than a single miRNA.

The presented pan-cancer-wide analysis discovering copy number-aberration-influenced miRNA-target associations may be used in future experimental work to validate the roles of the miRNAs in context-specific gene regulation to derive even greater confidence in their tissue-specific associations. We integrated several potential co-regulators such as CNA, DNA methylation, miRNA expression and TFs, that can influence *trans* gene's expression in the LASSO step. Other potential regulators such as histone modification and chromatin accessibility (such as ATAC-seq) could also be integrated. *miRDriver* outperformed the existing sequence-based ceRNA inference tool, Cupid. This analysis reveals that this work can be further examined by taking into account the presence of recognized target sites that contribute to gene regulation, as well as utilizing ceRNA interactions to improve the inferred miRNA-gene networks. *miRDriver* does compute both direct and indirect targets of miRNAs, which helps decipher the downstream biological processes and pathways regulated by these miRNAs. To identify the direct targets of these selected miRNAs, one could utilize sequence-based filtering.

Finally, in this study, we established *miRDriver* as an R software package and provided users with a variety of options for running our workflow with their preferred settings. Users can, for example, utilize the tool exclusively with up or down-regulated genes from amplified or deleted regions, or both. However, in these cases, the context in which miRNA-gene interactions are discovered will limit their detection. To receive the most comprehensive list of miRNA-gene interactions, we propose that users evaluate all of the directions. In the software, we have also included the flexibility to utilize user-defined TF-targets with evidence-based confidence levels filtering options for cancer-related TF-target interactions from the DoRothEA gene set resource^106^. In this study, however, we used only the highly confident TF-target interactions from TRED and TRRUST in the LASSO step as using many predictors in LASSO could affect its performance, and cause false positive and false negative interactions. Furthermore, considering gene expression is controlled at multiple levels, including transcriptional regulation and post-transcriptional regulation, our software provides the flexibility to run the LASSO step in two phases. In the first run, only the transcriptional predictors could be utilized to explain the expression variation. In the second run, post-transcriptional predictors and the residual of the first LASSO run can be utilized as the independent and dependent variables, respectively. Alternatively, if the user has the transcriptional and post-transcriptional expression change data, both LASSO runs can be performed in any order. The details of all these options can be accessed in the vignette of the *miRDriver* R package.

## Supplementary Information

Supplementary Information 1.

Supplementary Information 2.

Supplementary Information 3.

Supplementary Information 4.

Supplementary Information 5.

Supplementary Information 6.

Supplementary Information 7.

Supplementary Information 8.

Supplementary Information 9.

Supplementary Information 10.

Supplementary Information 11.

Supplementary Information 12.

Supplementary Information 13.

Supplementary Information 14.

Supplementary Information 15.

Supplementary Information 16.

Supplementary Information 17.

Supplementary Information 18.

Supplementary Information 19.

Supplementary Information 20.

Supplementary Information 21.

Supplementary Information 22.

Supplementary Information 23.

Supplementary Information 24.

Supplementary Information 25.

Supplementary Information 26.

Supplementary Information 27.

Supplementary Information 28.

Supplementary Information 29.

Supplementary Information 30.

Supplementary Information 31.

Supplementary Information 32.

Supplementary Information 33.

Supplementary Information 34.

## Data Availability

The *miRDriver* pipeline was developed as an R package. The source codes of the package are available at https://github.com/bozdaglab/miRDriver under Creative Commons Attribution Non Commercial 4.0 International Public License. The scripts for running the pipeline and the evaluation results can be accessed from the supplementary documents. The datasets can be accessed from Figshare via https://figshare.com/s/7400ad8445b2e78e4636 .
