# Supplementary material for: Computing microRNA-gene interaction networks in pan-cancer using miRDriver
Source: Sci Rep. 2022 Mar 8;12:3717. doi: 10.1038/s41598-022-07628-z (PMC8904490; doi:10.1038/s41598-022-07628-z)
Supplement: Supplementary file 10 — Supplementary Information 10. [file 41598_2022_7628_MOESM10_ESM.pdf]

Computing microRNA-gene interaction networks in pan-cancer using miRDriver

Banabithi Bose, Matthew Moravec, and Serdar Bozdag

Supplemental Figure S1

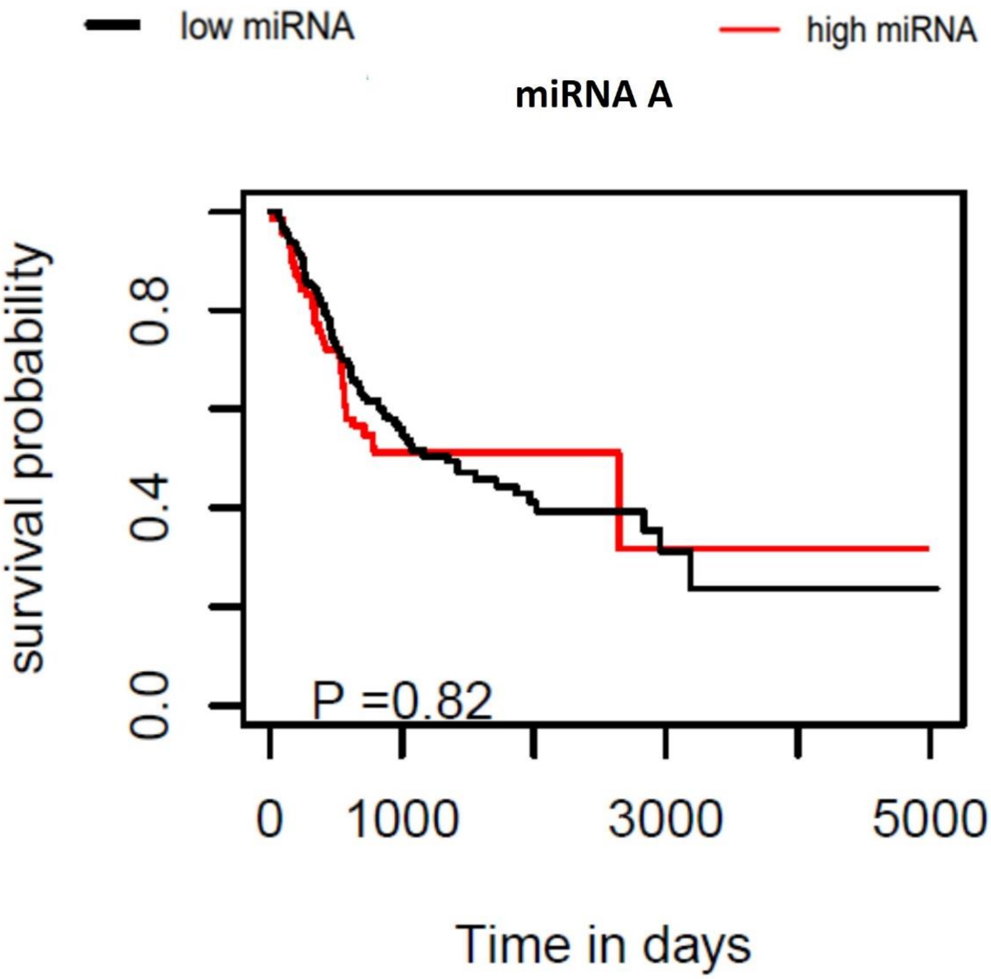

The *Adjusted Kaplan-Meier* survival plots for the computed miRNAs in high and low miRNA expression patient groups.

Cancer Type: ACC

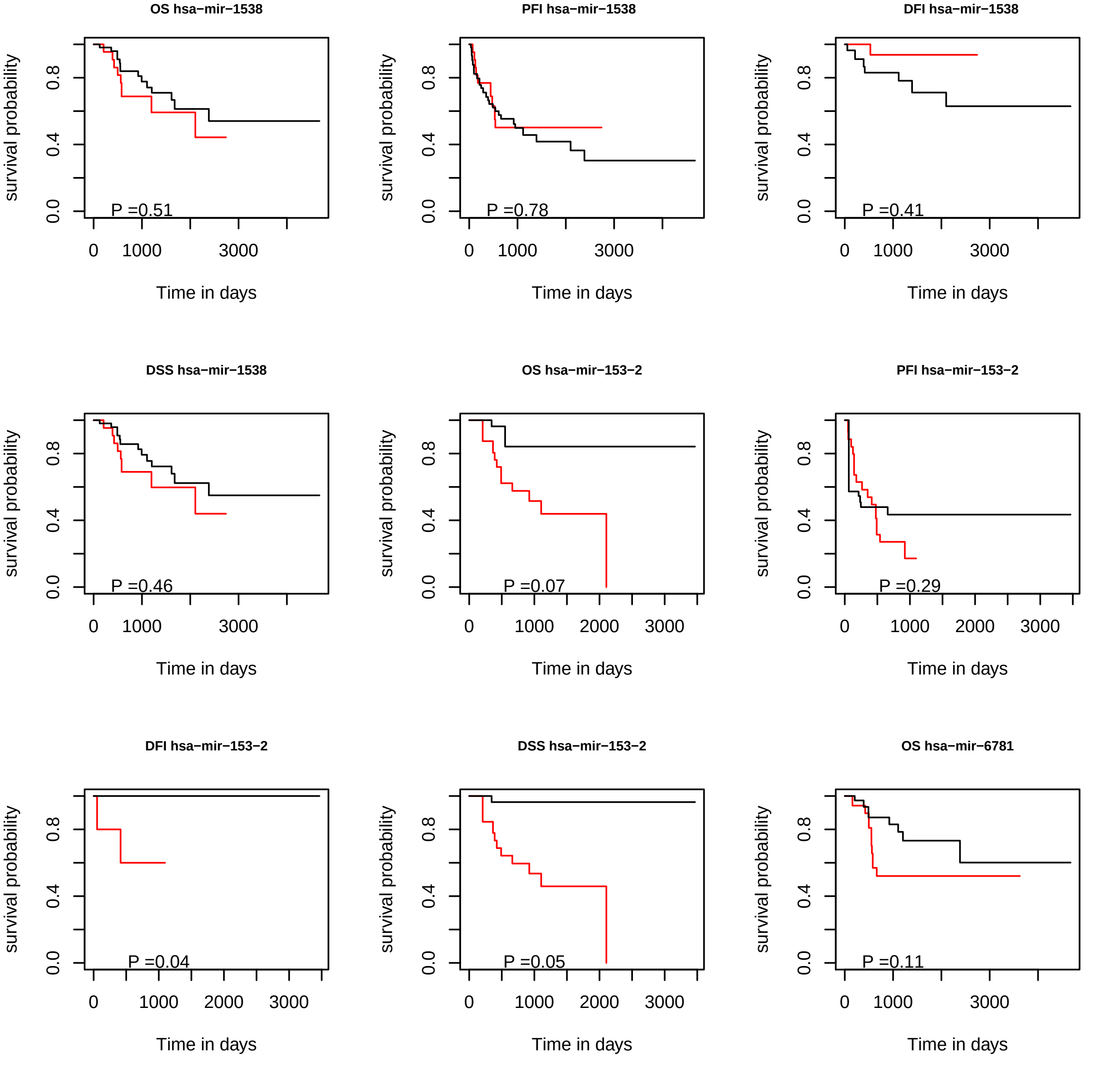

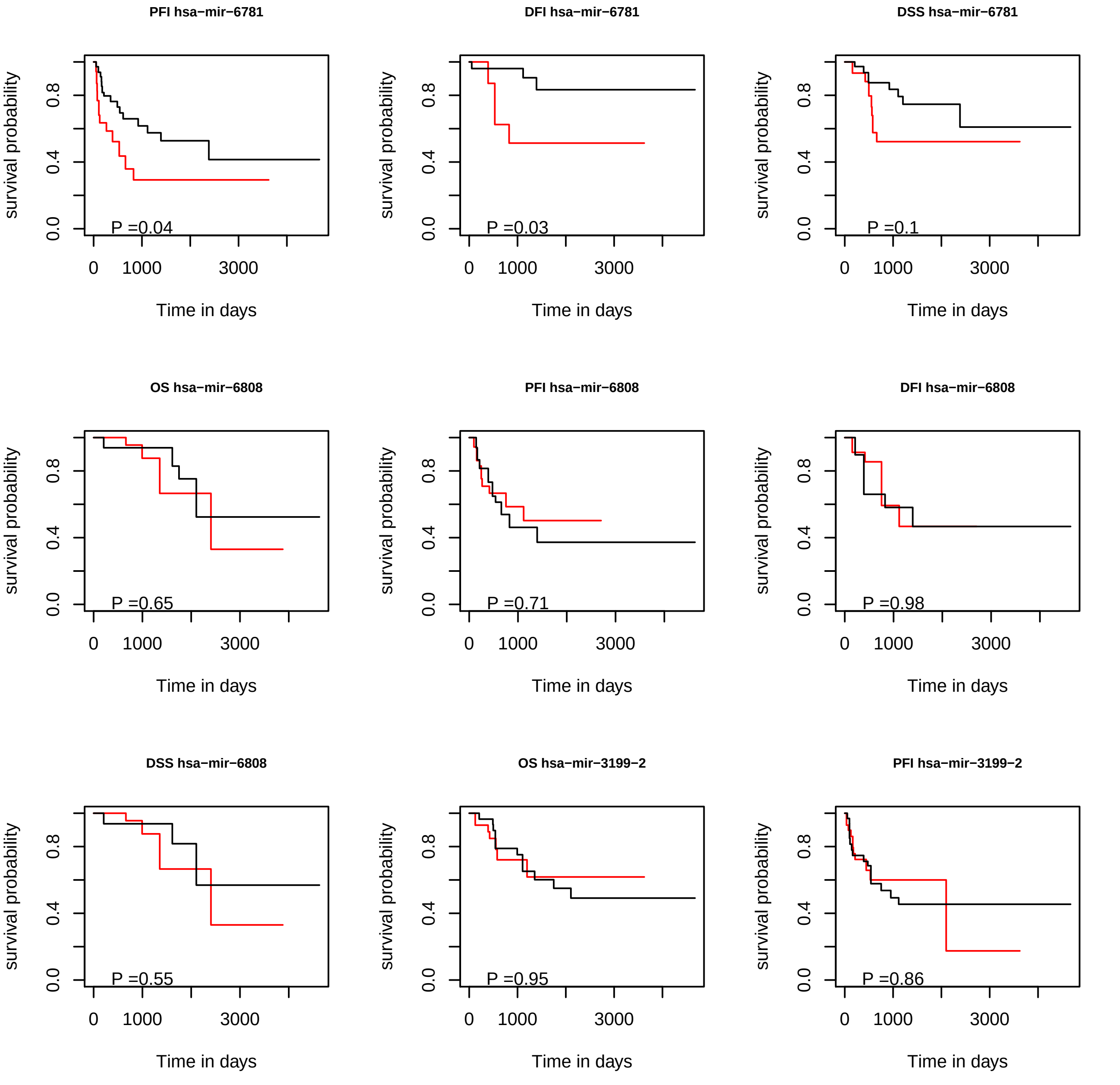
PFI hsa−mir−6781
P =0.04
DFI hsa−mir−6781
P =0.03
DSS hsa−mir−6781
P =0.1
OS hsa−mir−6808
P =0.65
PFI hsa−mir−6808
P =0.71
DFI hsa−mir−6808
P =0.98
DSS hsa−mir−6808
P =0.55
OS hsa−mir−3199−2
P =0.95
PFI hsa−mir−3199−2
P =0.86
survival probability
0.0
0.4
0.8
Time in days
0
1000
3000

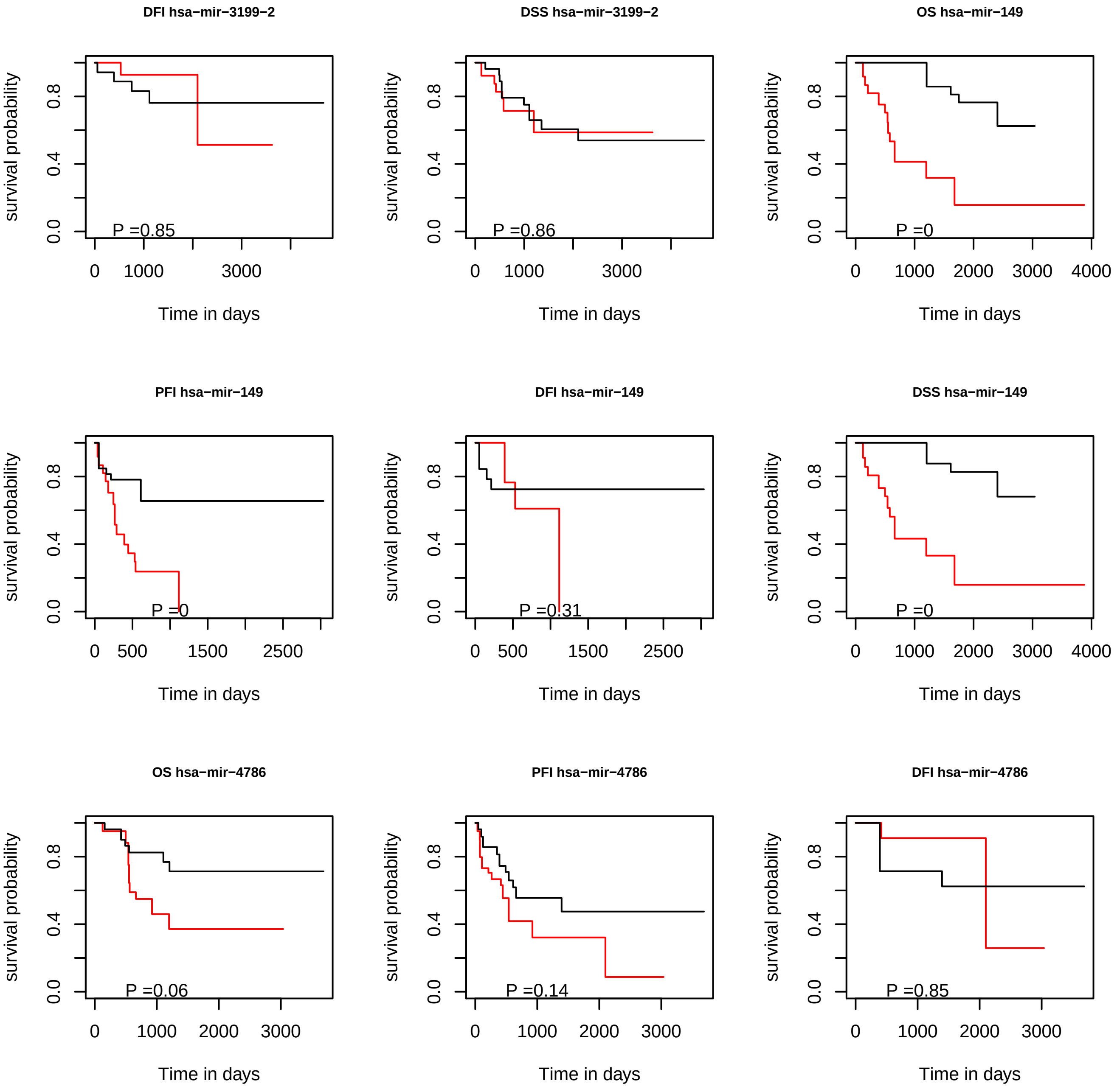

DFI hsa−mir−3199−2
survival probability
0.0
0.4
0.8
P =0.85
0
1000
3000
Time in days
DSS hsa−mir−3199−2
survival probability
0.0
0.4
0.8
P =0.86
0
1000
3000
Time in days
OS hsa−mir−149
survival probability
0.0
0.4
0.8
P =0
0
1000
2000
3000
4000
Time in days
PFI hsa−mir−149
survival probability
0.0
0.4
0.8
P =0
0
500
1500
2500
Time in days
DFI hsa−mir−149
survival probability
0.0
0.4
0.8
P =0.31
0
500
1500
2500
Time in days
DSS hsa−mir−149
survival probability
0.0
0.4
0.8
P =0
0
1000
2000
3000
4000
Time in days
OS hsa−mir−4786
survival probability
0.0
0.4
0.8
P =0.06
0
1000
2000
3000
Time in days
PFI hsa−mir−4786
survival probability
0.0
0.4
0.8
P =0.14
0
1000
2000
3000
Time in days
DFI hsa−mir−4786
survival probability
0.0
0.4
0.8
P =0.85
0
1000
2000
3000
Time in days

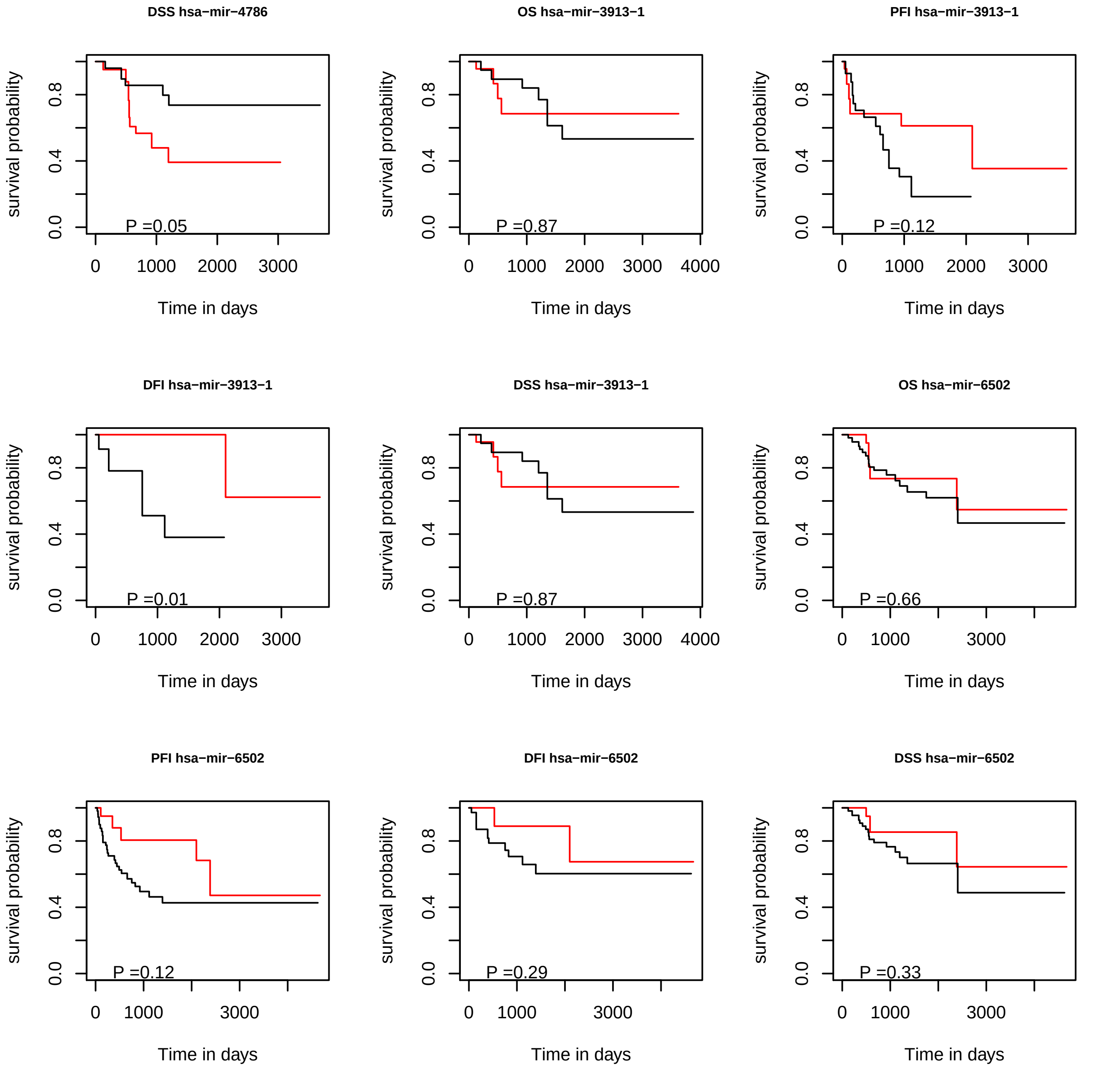
DSS hsa−mir−4786
survival probability
0.0
0.4
0.8
P =0.05
0
1000
2000
3000
Time in days
OS hsa−mir−3913−1
survival probability
0.0
0.4
0.8
P =0.87
0
1000
2000
3000
4000
Time in days
PFI hsa−mir−3913−1
survival probability
0.0
0.4
0.8
P =0.12
0
1000
2000
3000
Time in days
DFI hsa−mir−3913−1
survival probability
0.0
0.4
0.8
P =0.01
0
1000
2000
3000
Time in days
DSS hsa−mir−3913−1
survival probability
0.0
0.4
0.8
P =0.87
0
1000
2000
3000
4000
Time in days
OS hsa−mir−6502
survival probability
0.0
0.4
0.8
P =0.66
0
1000
3000
Time in days
PFI hsa−mir−6502
survival probability
0.0
0.4
0.8
P =0.12
0
1000
3000
Time in days
DFI hsa−mir−6502
survival probability
0.0
0.4
0.8
P =0.29
0
1000
3000
Time in days
DSS hsa−mir−6502
survival probability
0.0
0.4
0.8
P =0.33
0
1000
3000
Time in days

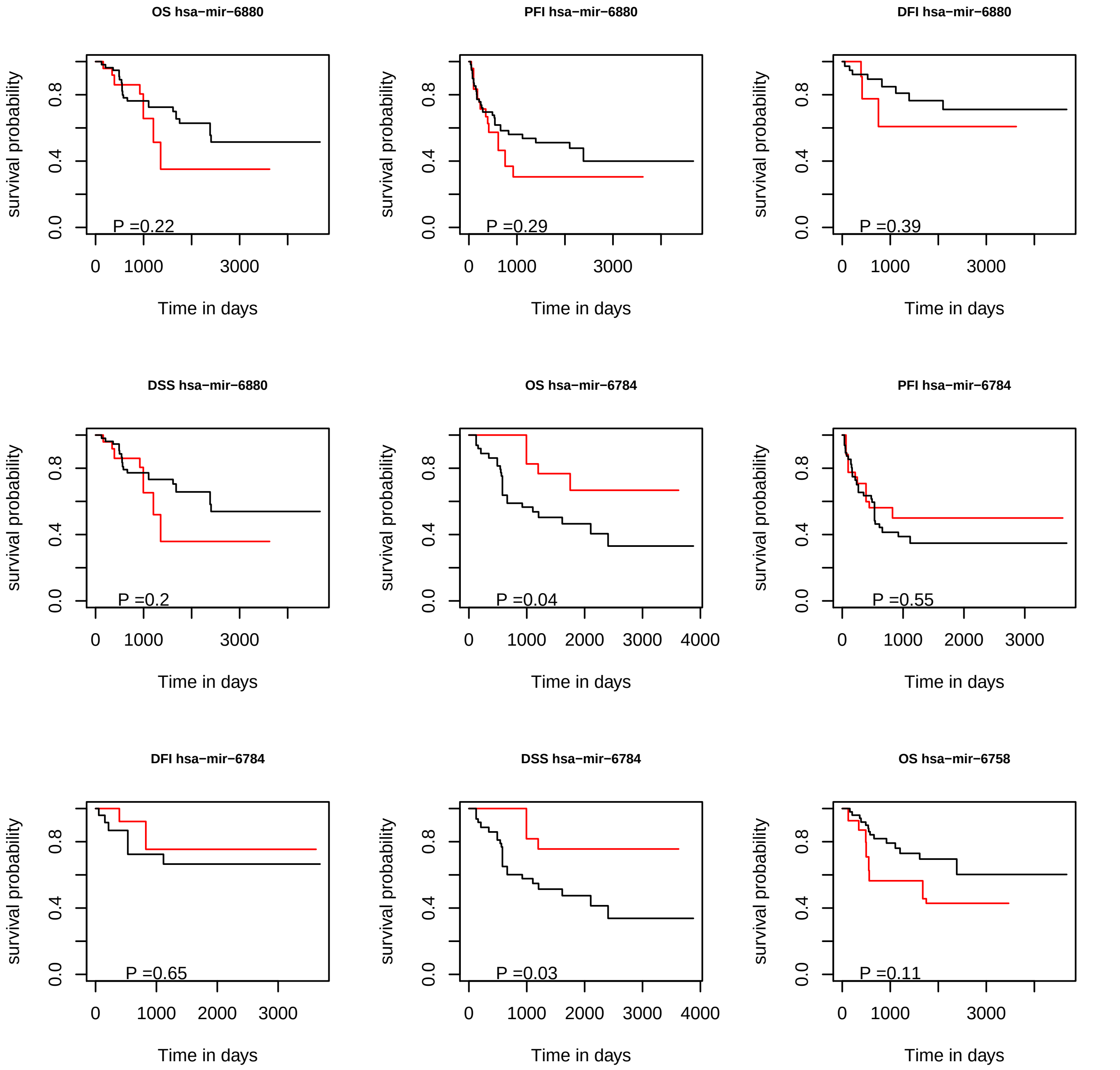
OS hsa−mir−6880
survival probability
0.0
0.4
0.8
P =0.22
0
1000
3000
Time in days
PFI hsa−mir−6880
survival probability
0.0
0.4
0.8
P =0.29
0
1000
3000
Time in days
DFI hsa−mir−6880
survival probability
0.0
0.4
0.8
P =0.39
0
1000
3000
Time in days
DSS hsa−mir−6880
survival probability
0.0
0.4
0.8
P =0.2
0
1000
3000
Time in days
OS hsa−mir−6784
survival probability
0.0
0.4
0.8
P =0.04
0
1000
2000
3000
4000
Time in days
PFI hsa−mir−6784
survival probability
0.0
0.4
0.8
P =0.55
0
1000
2000
3000
Time in days
DFI hsa−mir−6784
survival probability
0.0
0.4
0.8
P =0.65
0
1000
2000
3000
Time in days
DSS hsa−mir−6784
survival probability
0.0
0.4
0.8
P =0.03
0
1000
2000
3000
4000
Time in days
OS hsa−mir−6758
survival probability
0.0
0.4
0.8
P =0.11
0
1000
3000
Time in days

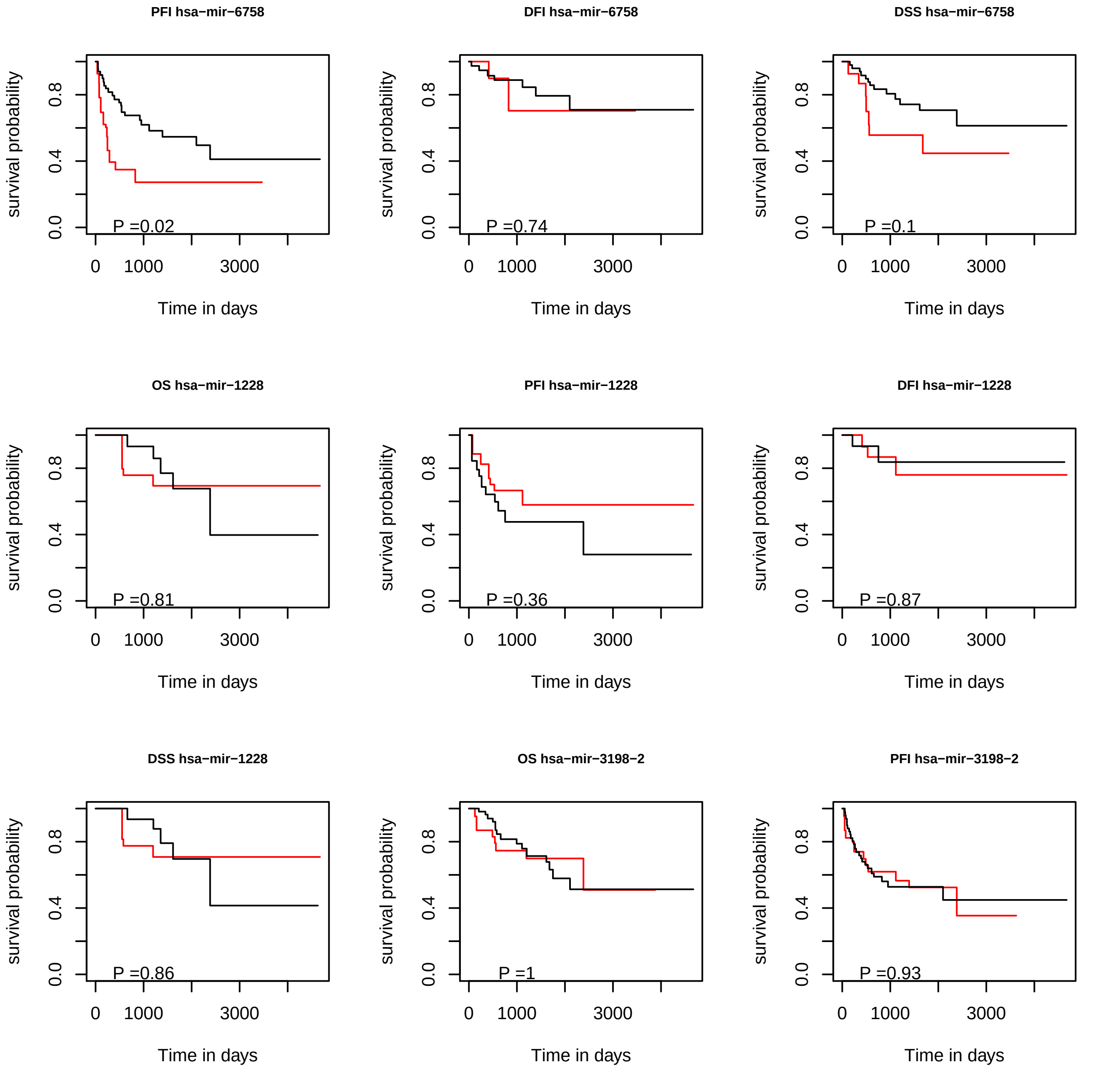
PFI hsa−mir−6758
P =0.02
DFI hsa−mir−6758
P =0.74
DSS hsa−mir−6758
P =0.1
OS hsa−mir−1228
P =0.81
PFI hsa−mir−1228
P =0.36
DFI hsa−mir−1228
P =0.87
DSS hsa−mir−1228
P =0.86
OS hsa−mir−3198−2
P =1
PFI hsa−mir−3198−2
P =0.93
survival probability
0.0
0.4
0.8
0
1000
3000
Time in days

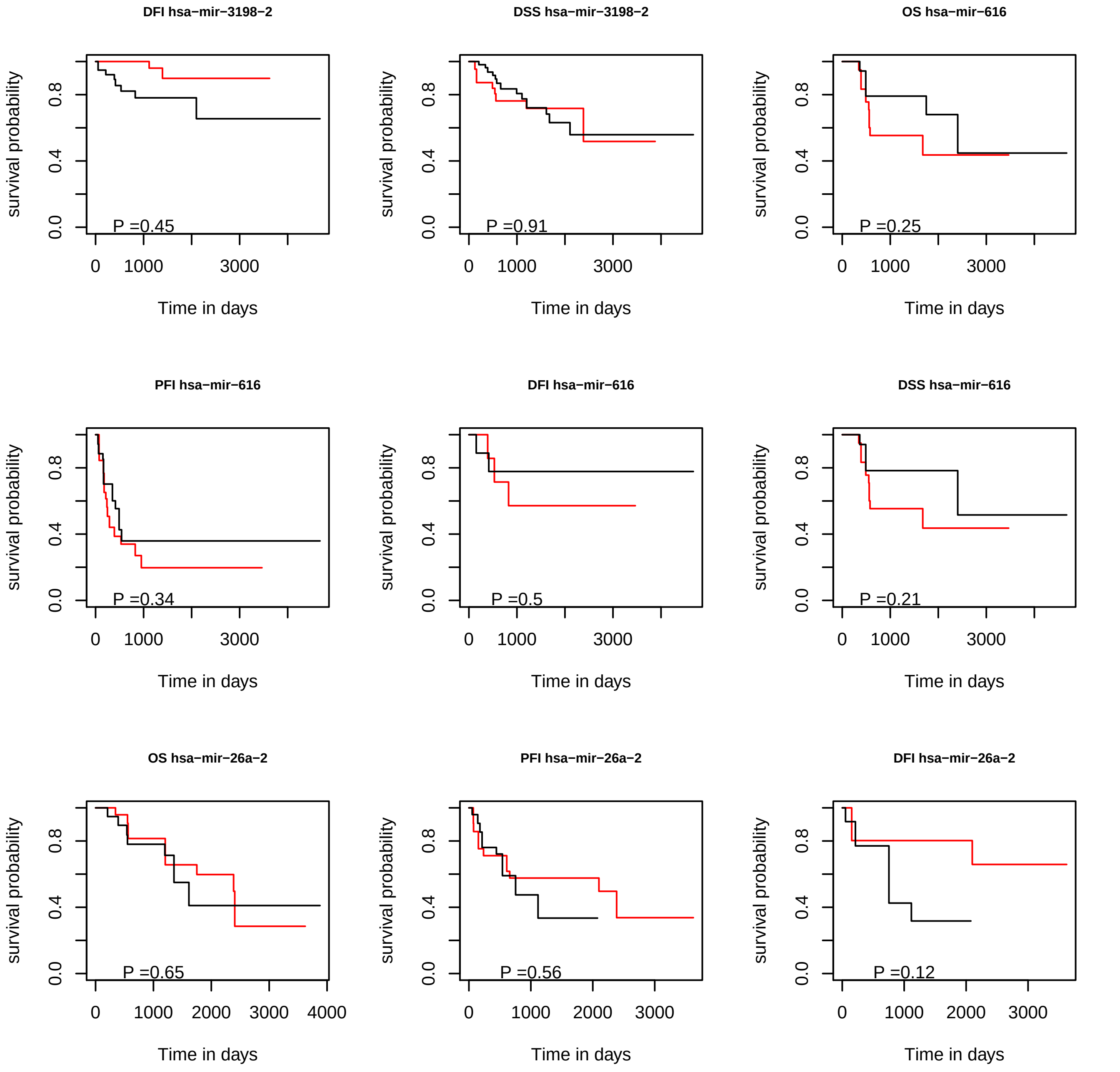
DFI hsa−mir−3198−2
P =0.45
DSS hsa−mir−3198−2
P =0.91
OS hsa−mir−616
P =0.25
PFI hsa−mir−616
P =0.34
DFI hsa−mir−616
P =0.5
DSS hsa−mir−616
P =0.21
OS hsa−mir−26a−2
P =0.65
PFI hsa−mir−26a−2
P =0.56
DFI hsa−mir−26a−2
P =0.12
survival probability
Time in days
0.0
0.4
0.8
0
1000
2000
3000
4000

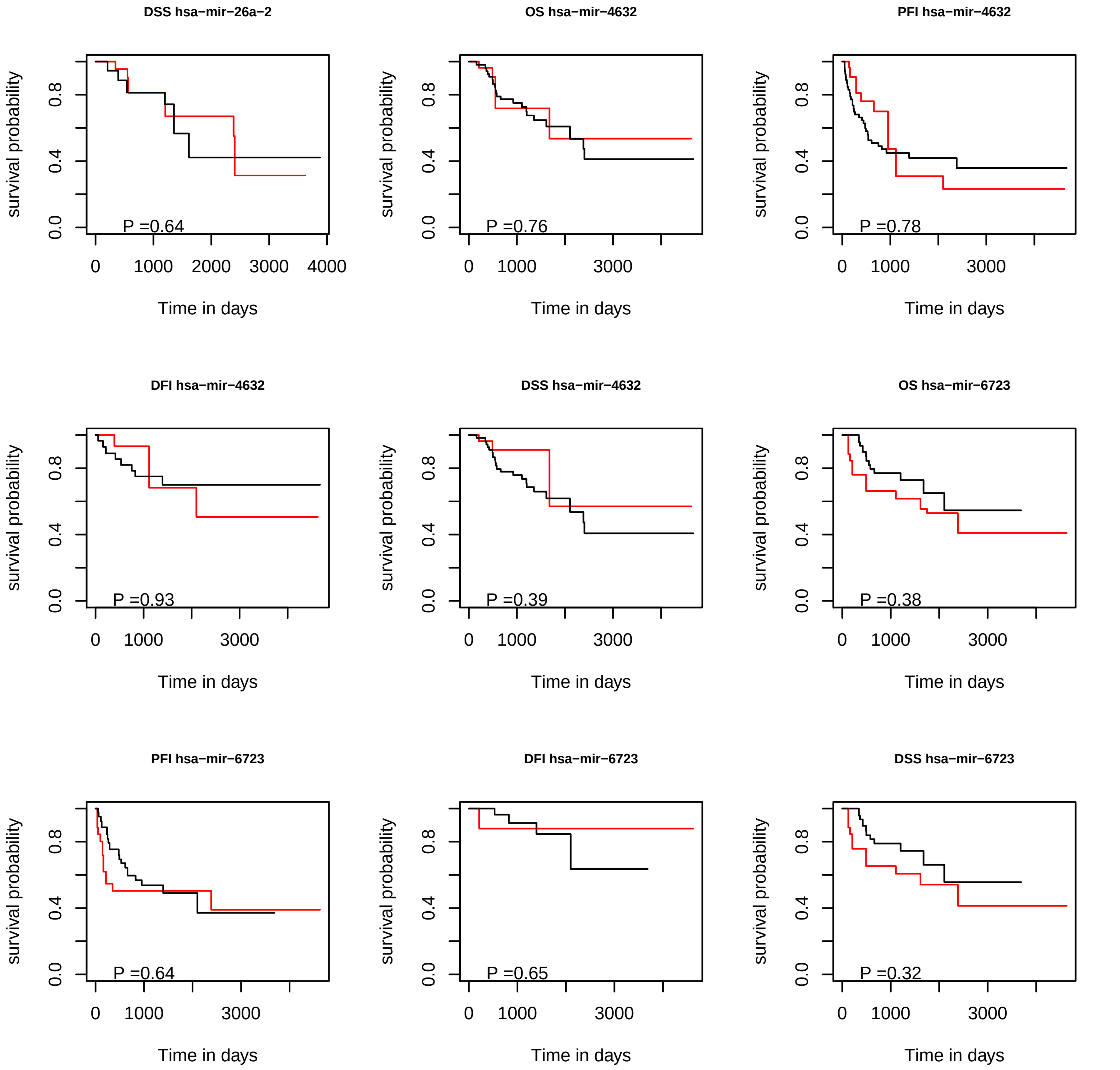
DSS hsa−mir−26a−2
P =0.64
OS hsa−mir−4632
P =0.76
PFI hsa−mir−4632
P =0.78
DFI hsa−mir−4632
P =0.93
DSS hsa−mir−4632
P =0.39
OS hsa−mir−6723
P =0.38
PFI hsa−mir−6723
P =0.64
DFI hsa−mir−6723
P =0.65
DSS hsa−mir−6723
P =0.32
survival probability
0.0
0.4
0.8
Time in days
0
1000
2000
3000
4000

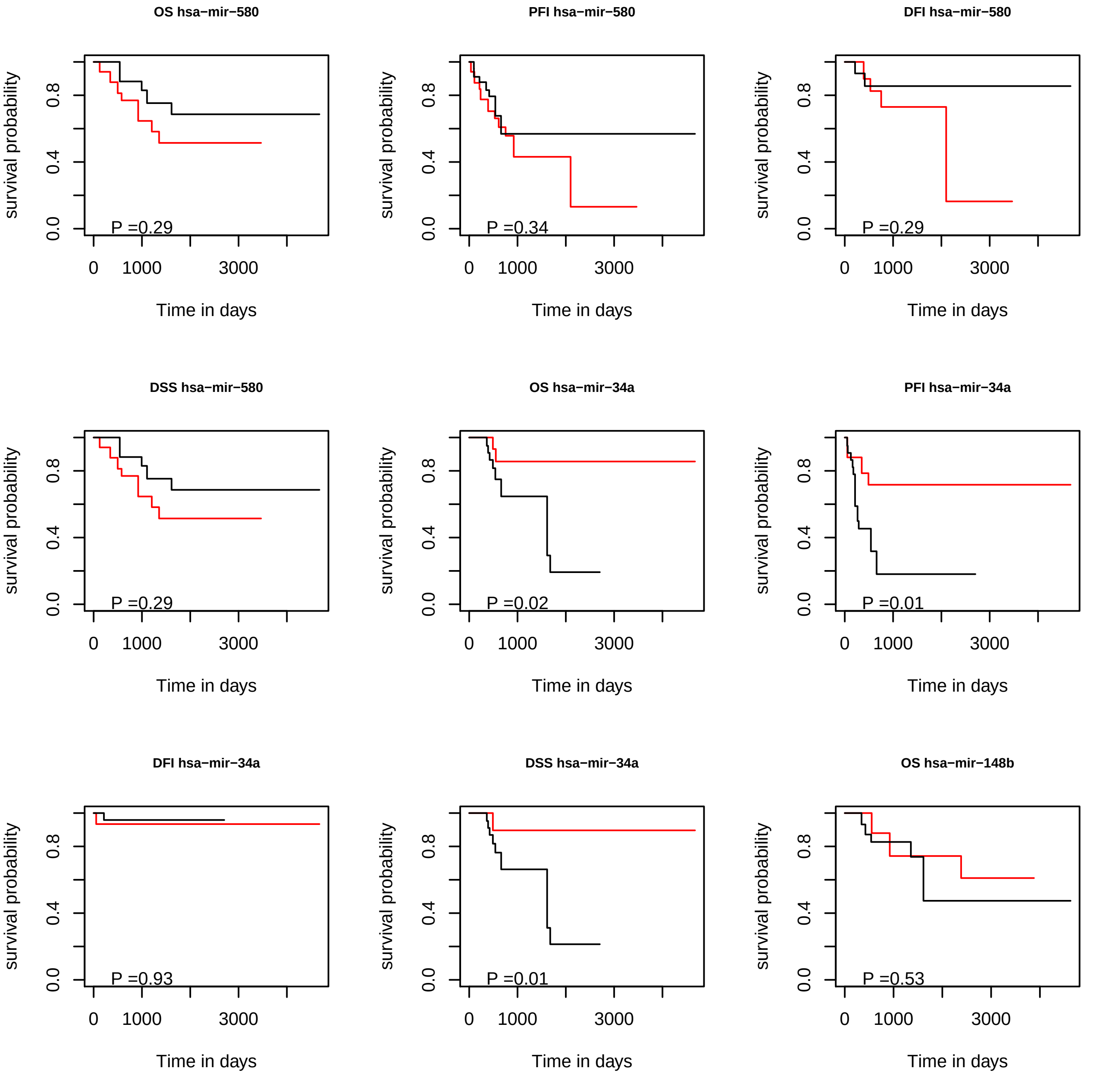
OS hsa−mir−580
P =0.29
PFI hsa−mir−580
P =0.34
DFI hsa−mir−580
P =0.29
DSS hsa−mir−580
P =0.29
OS hsa−mir−34a
P =0.02
PFI hsa−mir−34a
P =0.01
DFI hsa−mir−34a
P =0.93
DSS hsa−mir−34a
P =0.01
OS hsa−mir−148b
P =0.53
survival probability
Time in days
0.0
0.4
0.8
0
1000
3000

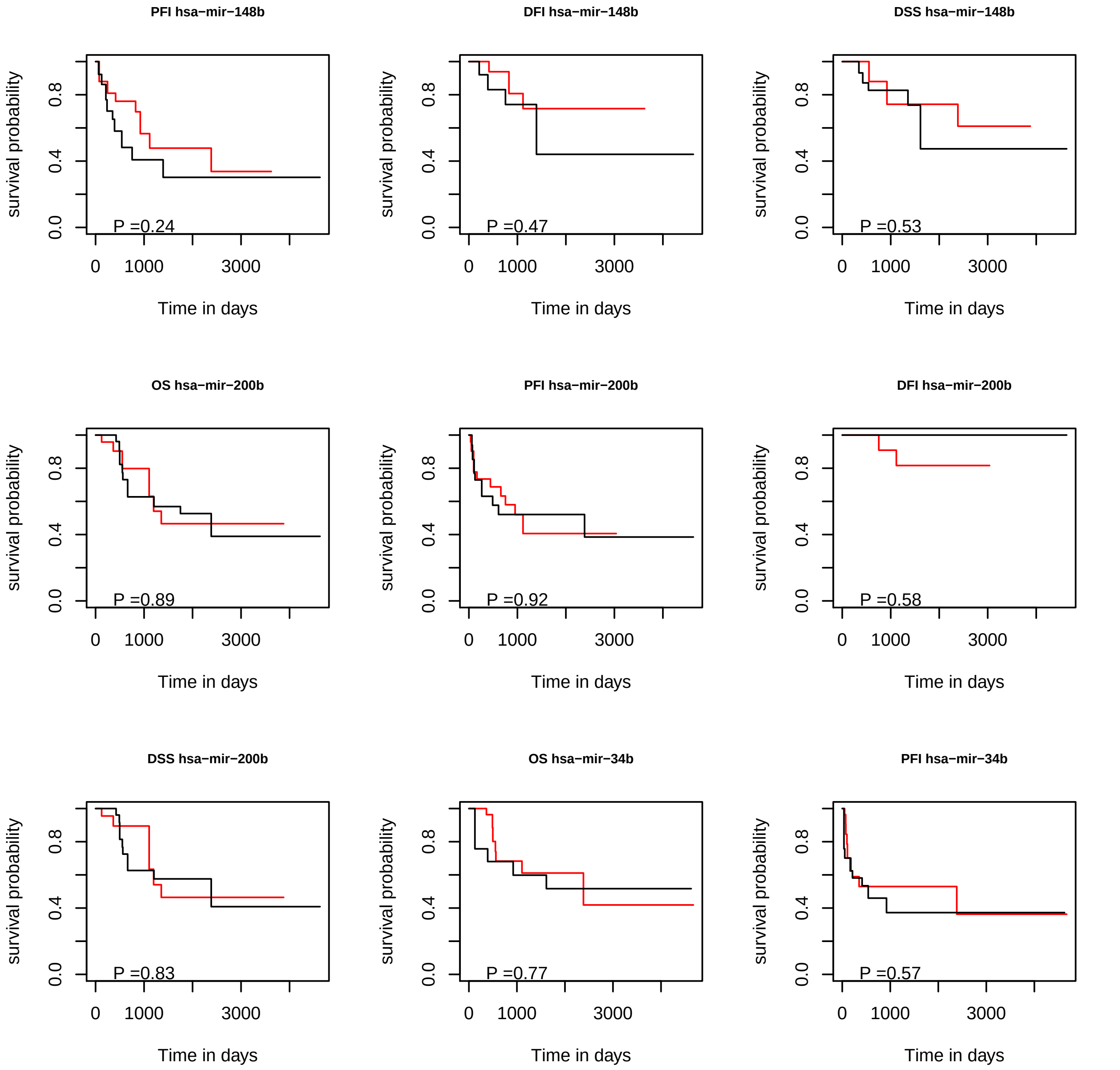
PFI hsa−mir−148b
P =0.24
DFI hsa−mir−148b
P =0.47
DSS hsa−mir−148b
P =0.53
OS hsa−mir−200b
P =0.89
PFI hsa−mir−200b
P =0.92
DFI hsa−mir−200b
P =0.58
DSS hsa−mir−200b
P =0.83
OS hsa−mir−34b
P =0.77
PFI hsa−mir−34b
P =0.57
survival probability
0.0
0.4
0.8
Time in days
0
1000
3000

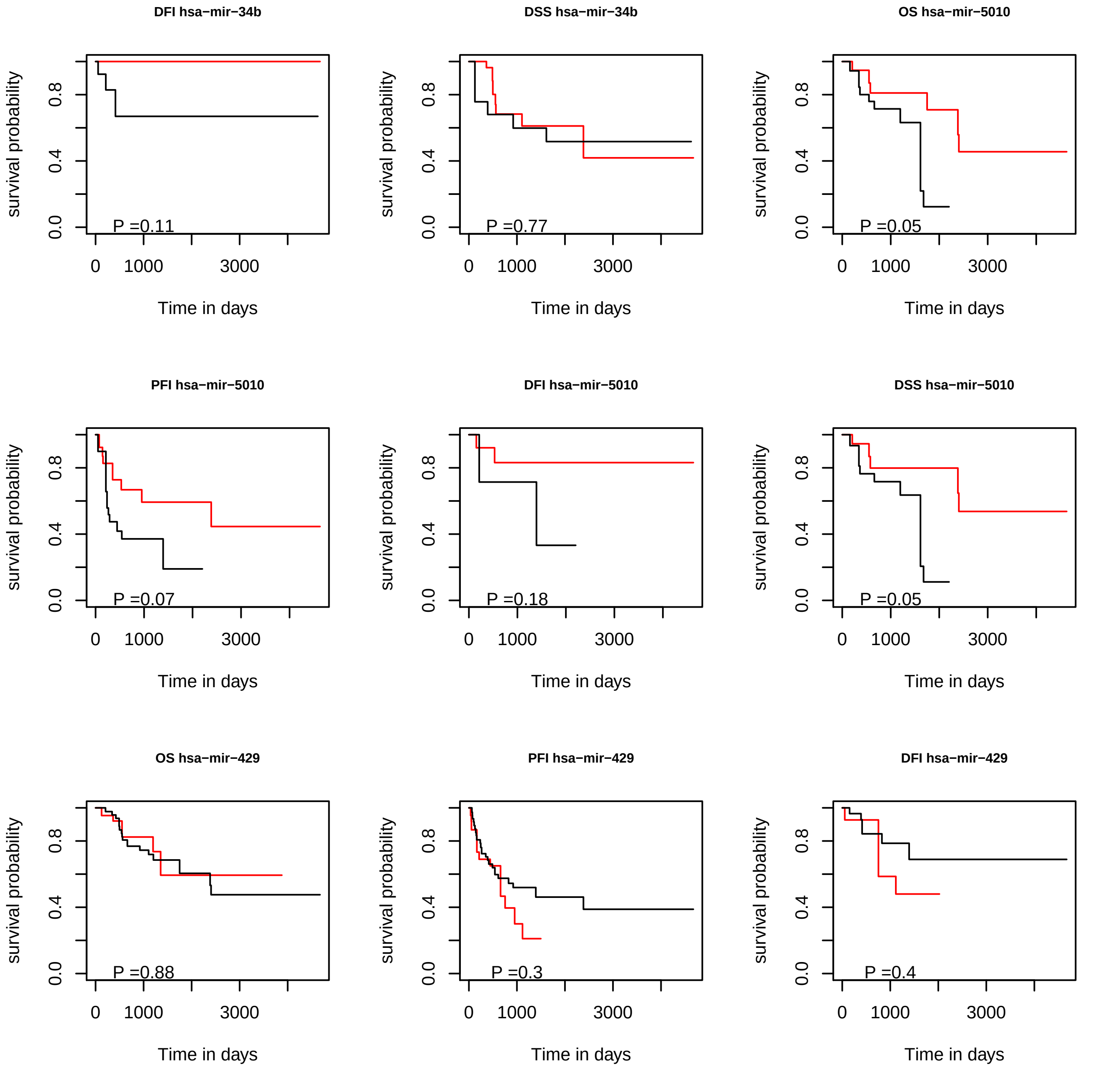
DFI hsa−mir−34b
survival probability
0.0
0.4
0.8
0
1000
3000
Time in days
P =0.11
DSS hsa−mir−34b
survival probability
0.0
0.4
0.8
0
1000
3000
Time in days
P =0.77
OS hsa−mir−5010
survival probability
0.0
0.4
0.8
0
1000
3000
Time in days
P =0.05
PFI hsa−mir−5010
survival probability
0.0
0.4
0.8
0
1000
3000
Time in days
P =0.07
DFI hsa−mir−5010
survival probability
0.0
0.4
0.8
0
1000
3000
Time in days
P =0.18
DSS hsa−mir−5010
survival probability
0.0
0.4
0.8
0
1000
3000
Time in days
P =0.05
OS hsa−mir−429
survival probability
0.0
0.4
0.8
0
1000
3000
Time in days
P =0.88
PFI hsa−mir−429
survival probability
0.0
0.4
0.8
0
1000
3000
Time in days
P =0.3
DFI hsa−mir−429
survival probability
0.0
0.4
0.8
0
1000
3000
Time in days
P =0.4

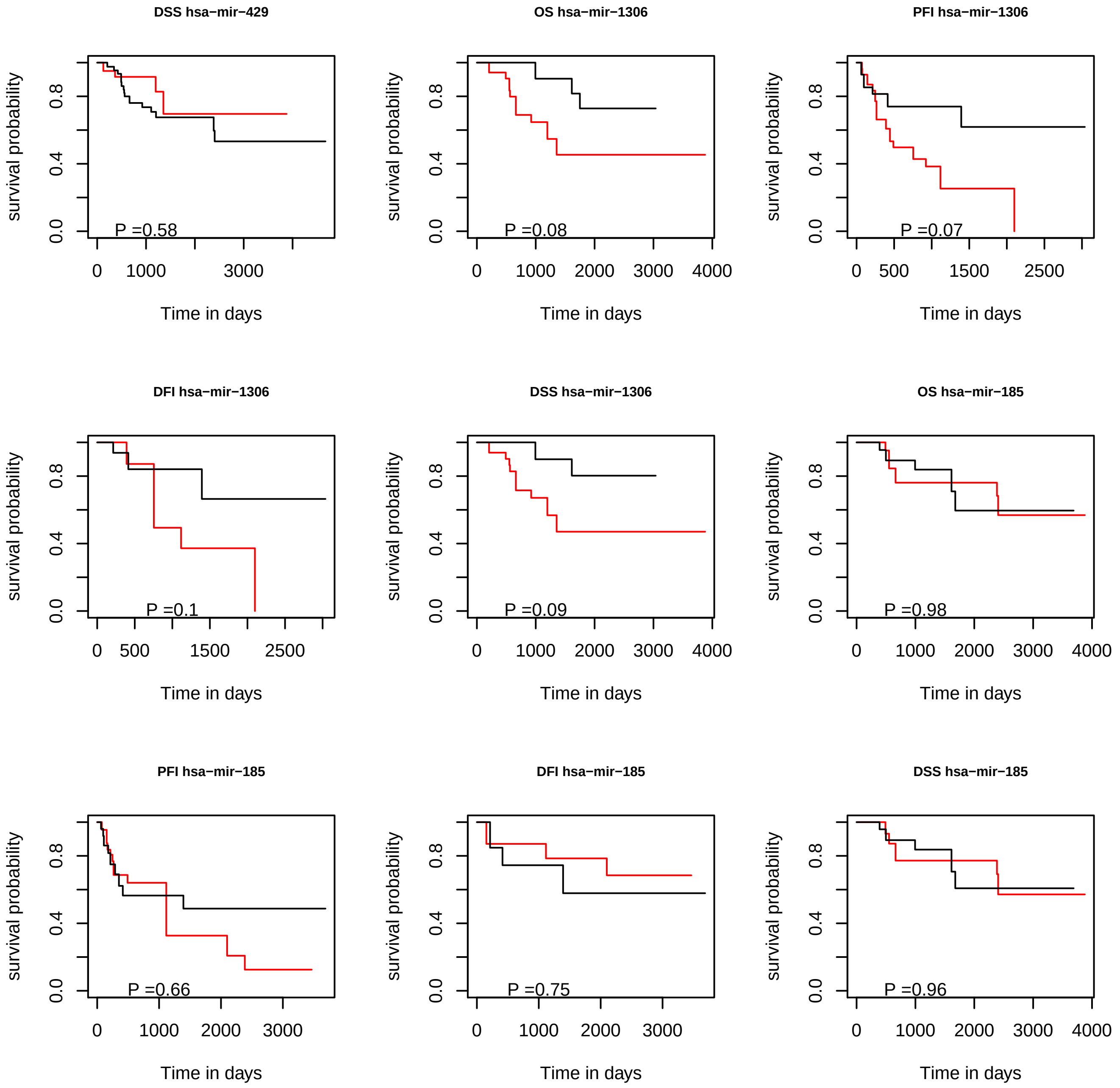
DSS hsa−mir−429
P =0.58
OS hsa−mir−1306
P =0.08
PFI hsa−mir−1306
P =0.07
DFI hsa−mir−1306
P =0.1
DSS hsa−mir−1306
P =0.09
OS hsa−mir−185
P =0.98
PFI hsa−mir−185
P =0.66
DFI hsa−mir−185
P =0.75
DSS hsa−mir−185
P =0.96
survival probability
Time in days

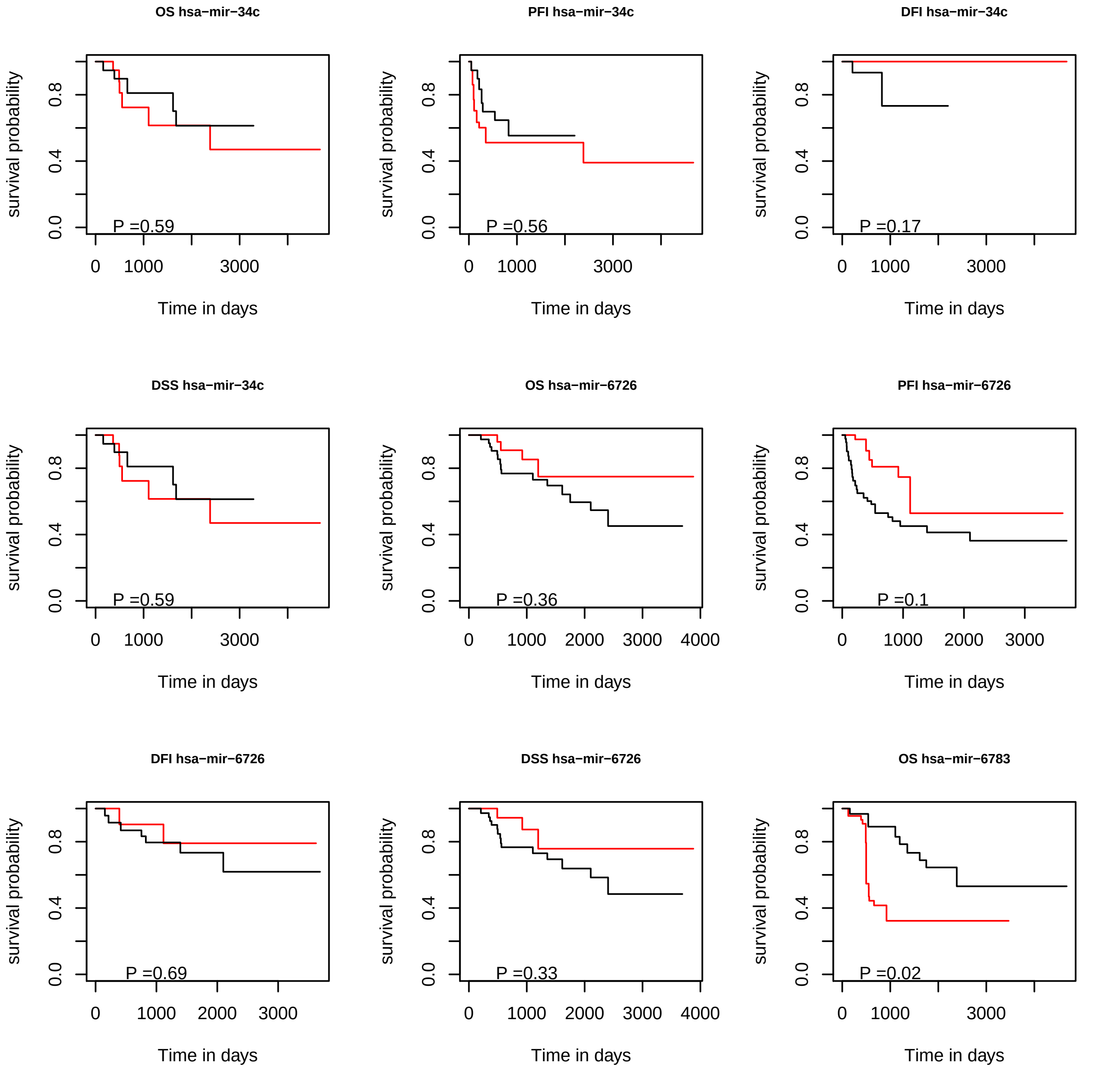
OS hsa−mir−34c
P =0.59
PFI hsa−mir−34c
P =0.56
DFI hsa−mir−34c
P =0.17
DSS hsa−mir−34c
P =0.59
OS hsa−mir−6726
P =0.36
PFI hsa−mir−6726
P =0.1
DFI hsa−mir−6726
P =0.69
DSS hsa−mir−6726
P =0.33
OS hsa−mir−6783
P =0.02
survival probability
Time in days
0.0
0.4
0.8
0
1000
2000
3000
4000

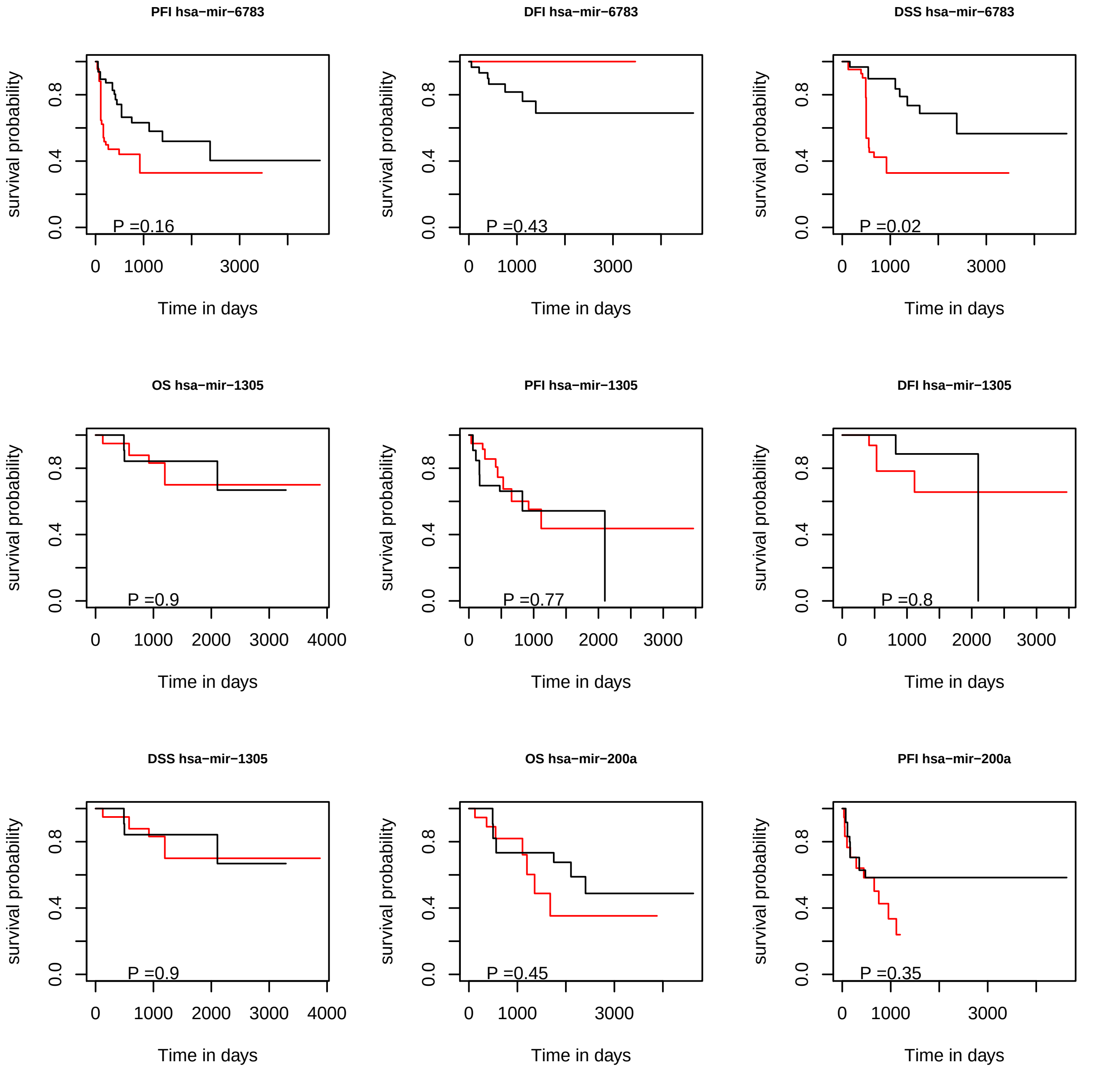

PFI hsa−mir−6783
P =0.16
DFI hsa−mir−6783
P =0.43
DSS hsa−mir−6783
P =0.02
OS hsa−mir−1305
P =0.9
PFI hsa−mir−1305
P =0.77
DFI hsa−mir−1305
P =0.8
DSS hsa−mir−1305
P =0.9
OS hsa−mir−200a
P =0.45
PFI hsa−mir−200a
P =0.35
survival probability
Time in days

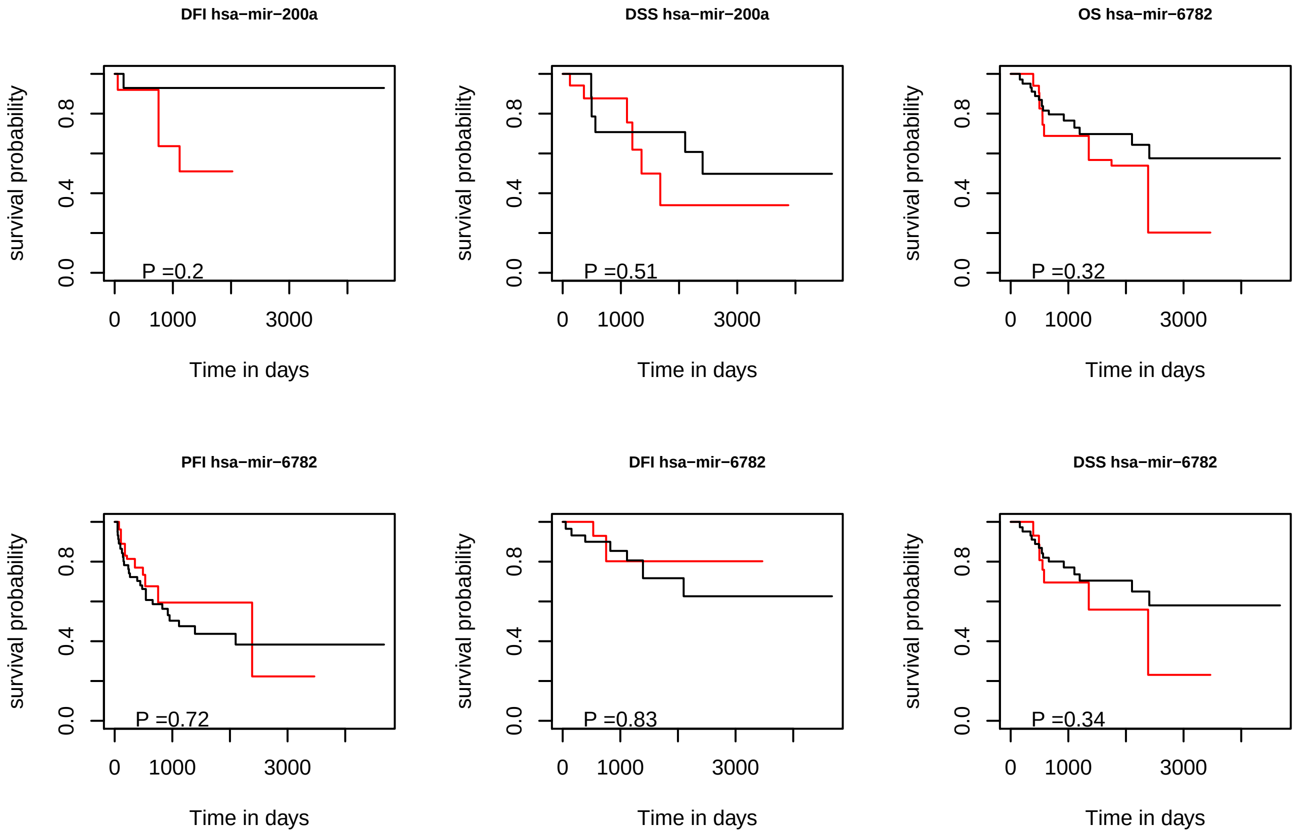
DFI hsa−mir−200a
DSS hsa−mir−200a
OS hsa−mir−6782
PFI hsa−mir−6782
DFI hsa−mir−6782
DSS hsa−mir−6782
survival probability
0.0
0.4
0.8
0
1000
3000
Time in days
P =0.2
P =0.51
P =0.32
P =0.72
P =0.83
P =0.34
