# Supplementary material for: Computing microRNA-gene interaction networks in pan-cancer using miRDriver
Source: Sci Rep. 2022 Mar 8;12:3717. doi: 10.1038/s41598-022-07628-z (PMC8904490; doi:10.1038/s41598-022-07628-z)
Supplement: Supplementary file 11 — Supplementary Information 11. [file 41598_2022_7628_MOESM11_ESM.pdf]

Computing microRNA-gene interaction networks in pan-cancer using miRDriver

Banabithi Bose, Matthew Moravec, and Serdar Bozdag

Supplemental Figure S2

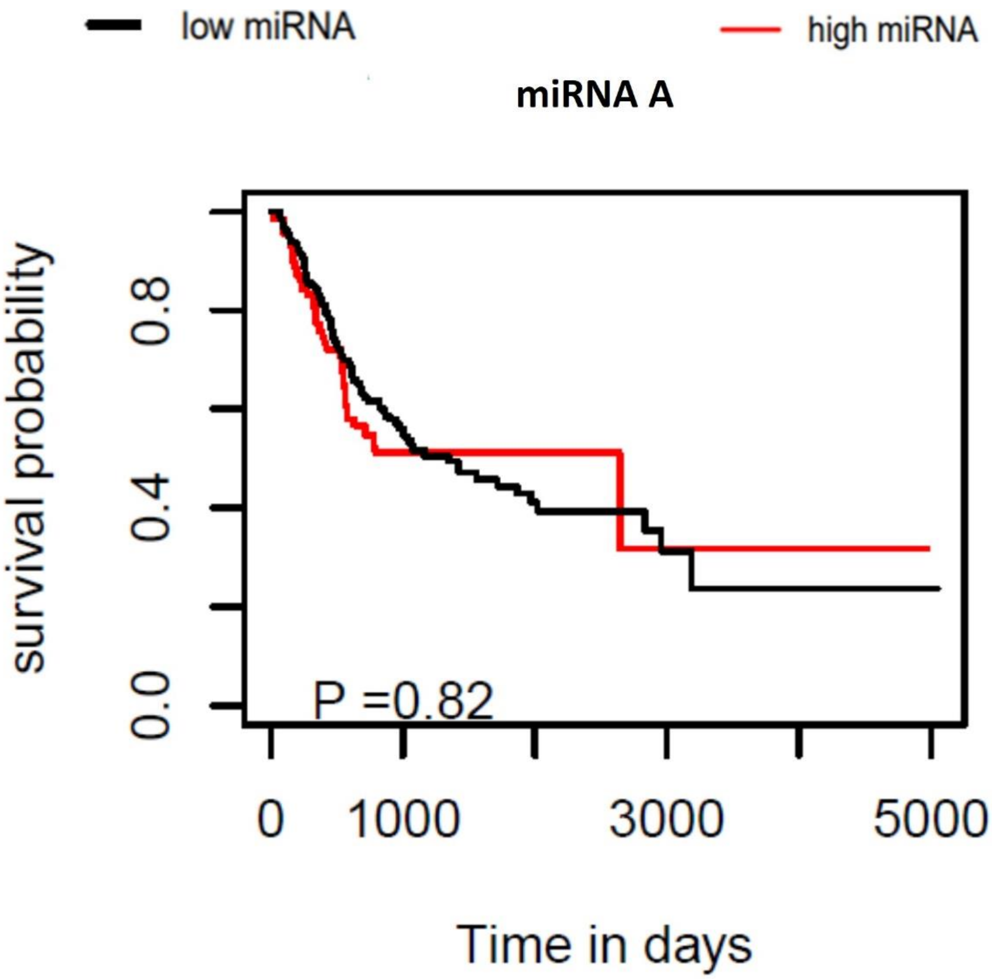

The *Adjusted Kaplan-Meier* survival plots for the computed miRNAs in high and low miRNA expression patient groups.

Supplemental Figure S2

Cancer Type: BLCA

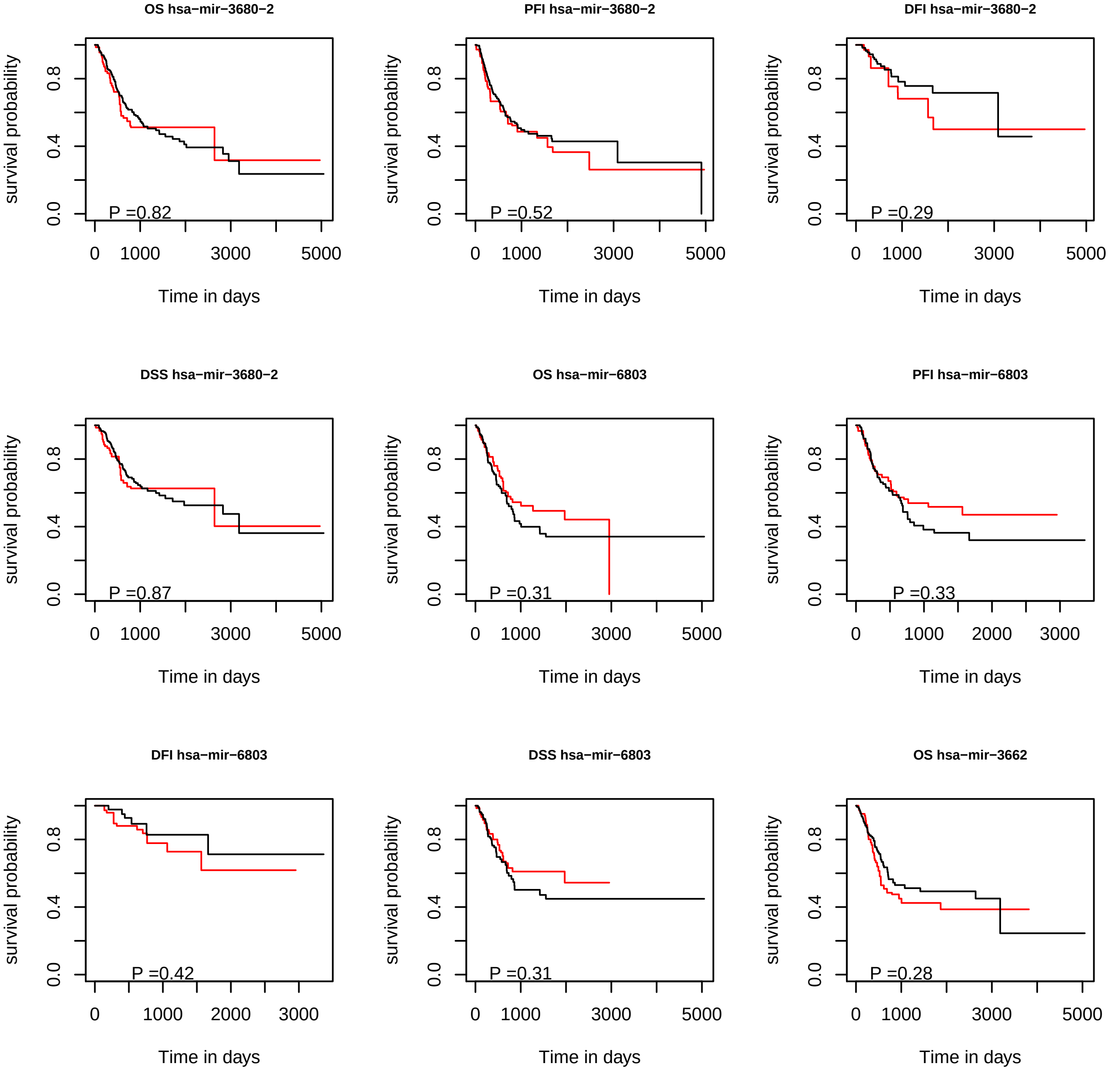

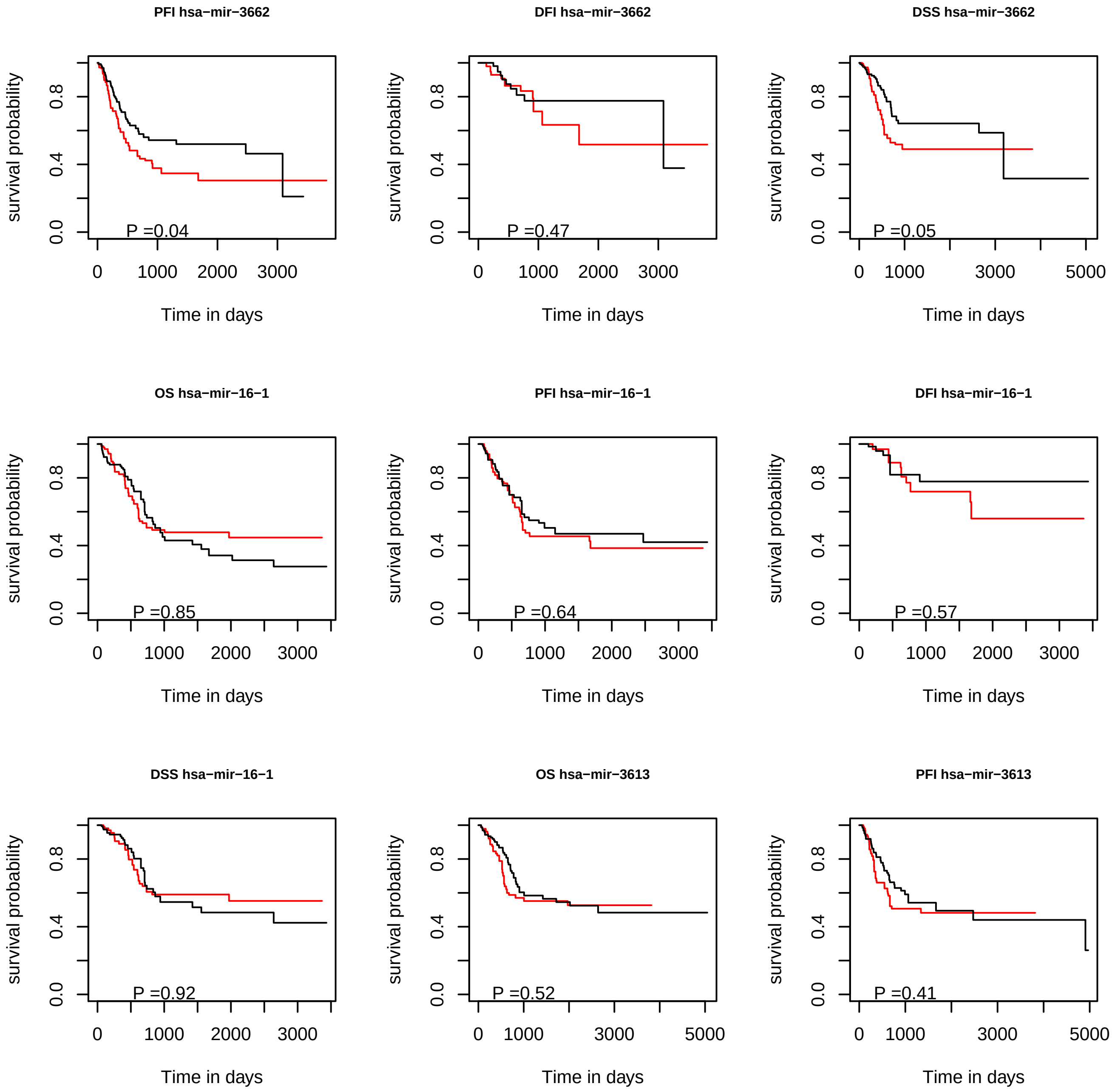
PFI hsa−mir−3662
P =0.04
DFI hsa−mir−3662
P =0.47
DSS hsa−mir−3662
P =0.05
OS hsa−mir−16−1
P =0.85
PFI hsa−mir−16−1
P =0.64
DFI hsa−mir−16−1
P =0.57
DSS hsa−mir−16−1
P =0.92
OS hsa−mir−3613
P =0.52
PFI hsa−mir−3613
P =0.41
survival probability
Time in days
0.0
0.4
0.8
0
1000
2000
3000
5000

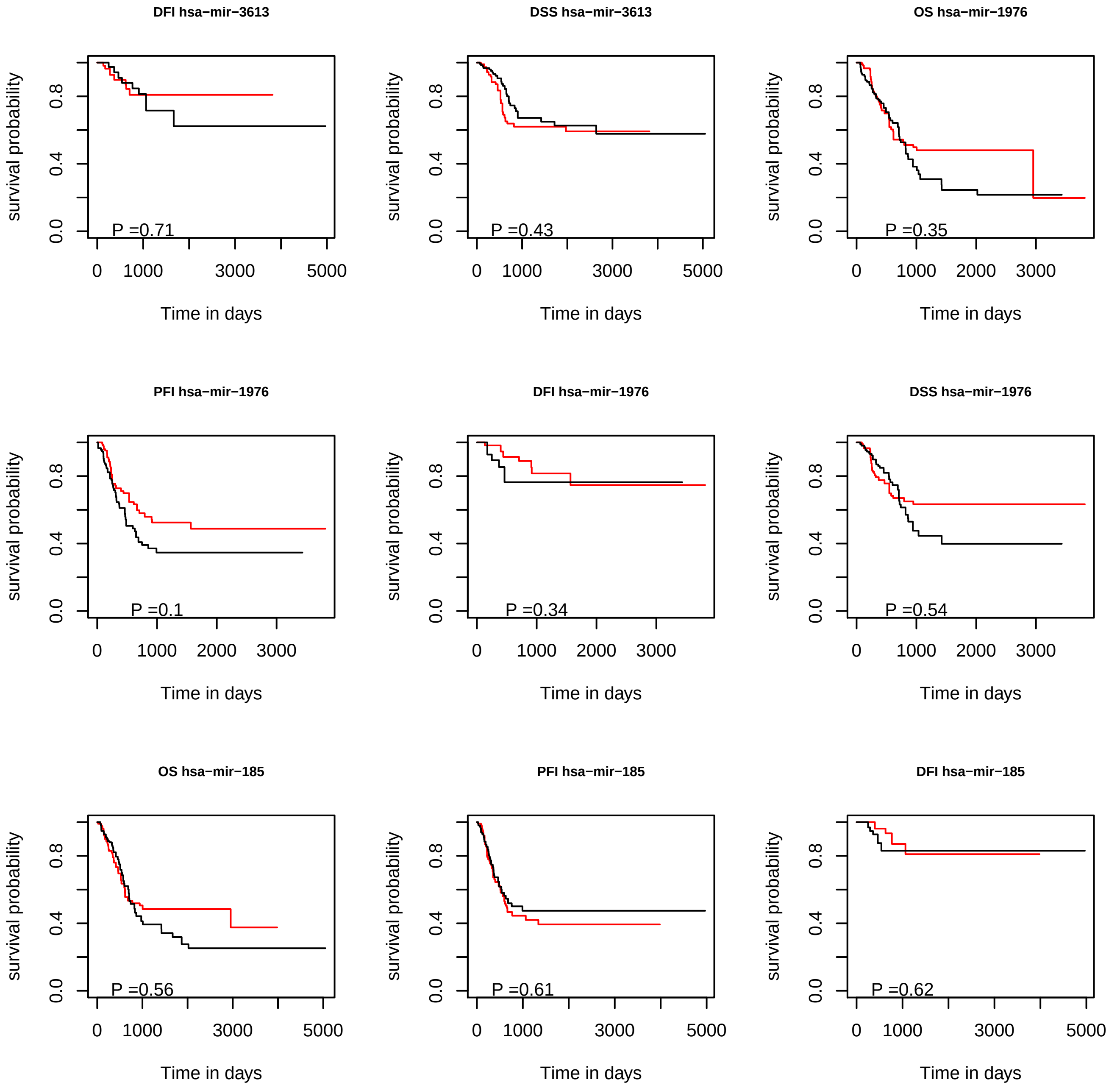
DFI hsa−mir−3613
survival probability
0.0
0.4
0.8
P =0.71
0
1000
3000
5000
Time in days
DSS hsa−mir−3613
survival probability
0.0
0.4
0.8
P =0.43
0
1000
3000
5000
Time in days
OS hsa−mir−1976
survival probability
0.0
0.4
0.8
P =0.35
0
1000
2000
3000
Time in days
PFI hsa−mir−1976
survival probability
0.0
0.4
0.8
P =0.1
0
1000
2000
3000
Time in days
DFI hsa−mir−1976
survival probability
0.0
0.4
0.8
P =0.34
0
1000
2000
3000
Time in days
DSS hsa−mir−1976
survival probability
0.0
0.4
0.8
P =0.54
0
1000
2000
3000
Time in days
OS hsa−mir−185
survival probability
0.0
0.4
0.8
P =0.56
0
1000
3000
5000
Time in days
PFI hsa−mir−185
survival probability
0.0
0.4
0.8
P =0.61
0
1000
3000
5000
Time in days
DFI hsa−mir−185
survival probability
0.0
0.4
0.8
P =0.62
0
1000
3000
5000
Time in days

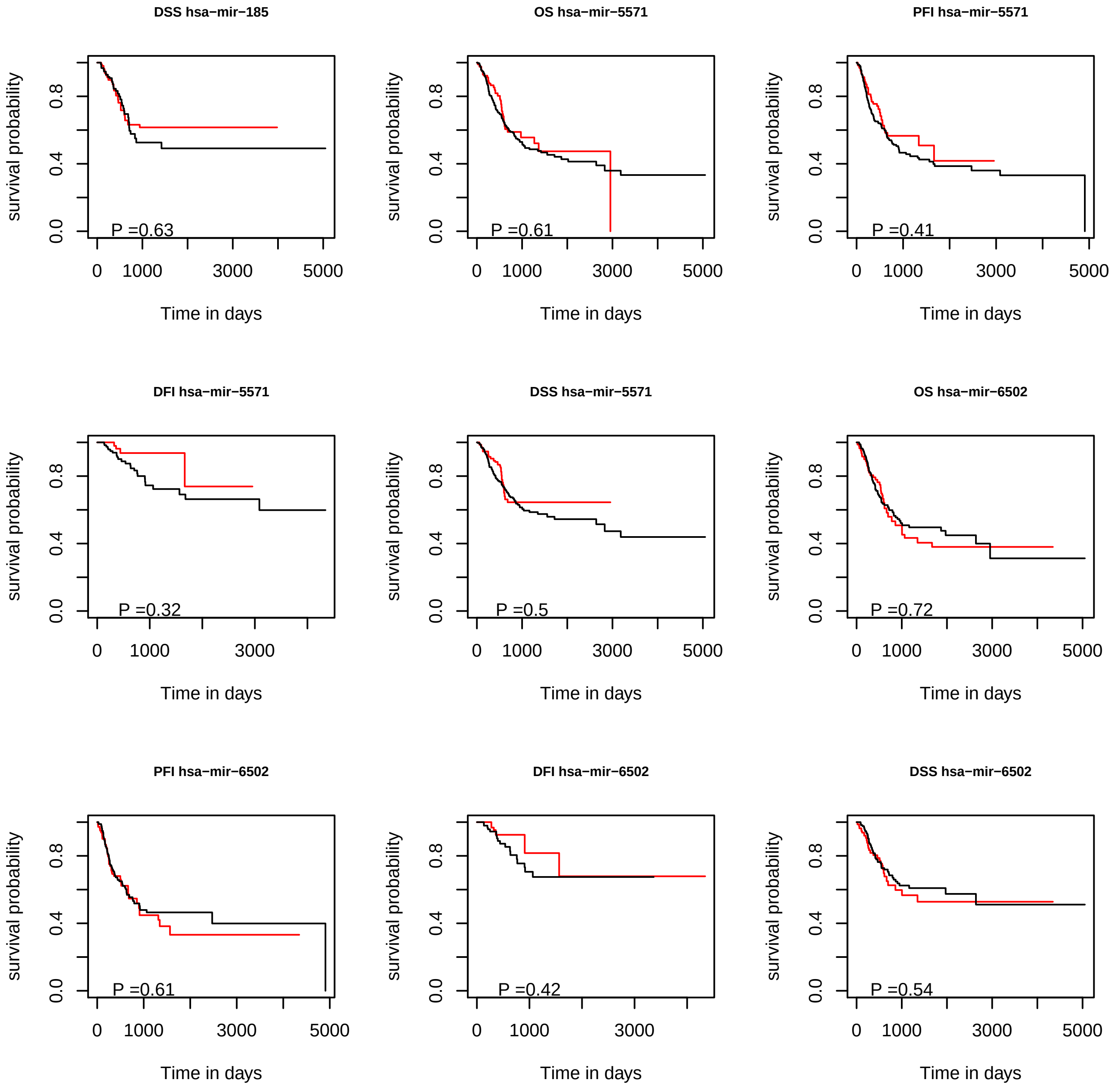
DSS hsa−mir−185
P =0.63
OS hsa−mir−5571
P =0.61
PFI hsa−mir−5571
P =0.41
DFI hsa−mir−5571
P =0.32
DSS hsa−mir−5571
P =0.5
OS hsa−mir−6502
P =0.72
PFI hsa−mir−6502
P =0.61
DFI hsa−mir−6502
P =0.42
DSS hsa−mir−6502
P =0.54
survival probability
0.0
0.4
0.8
Time in days
0
1000
3000
5000

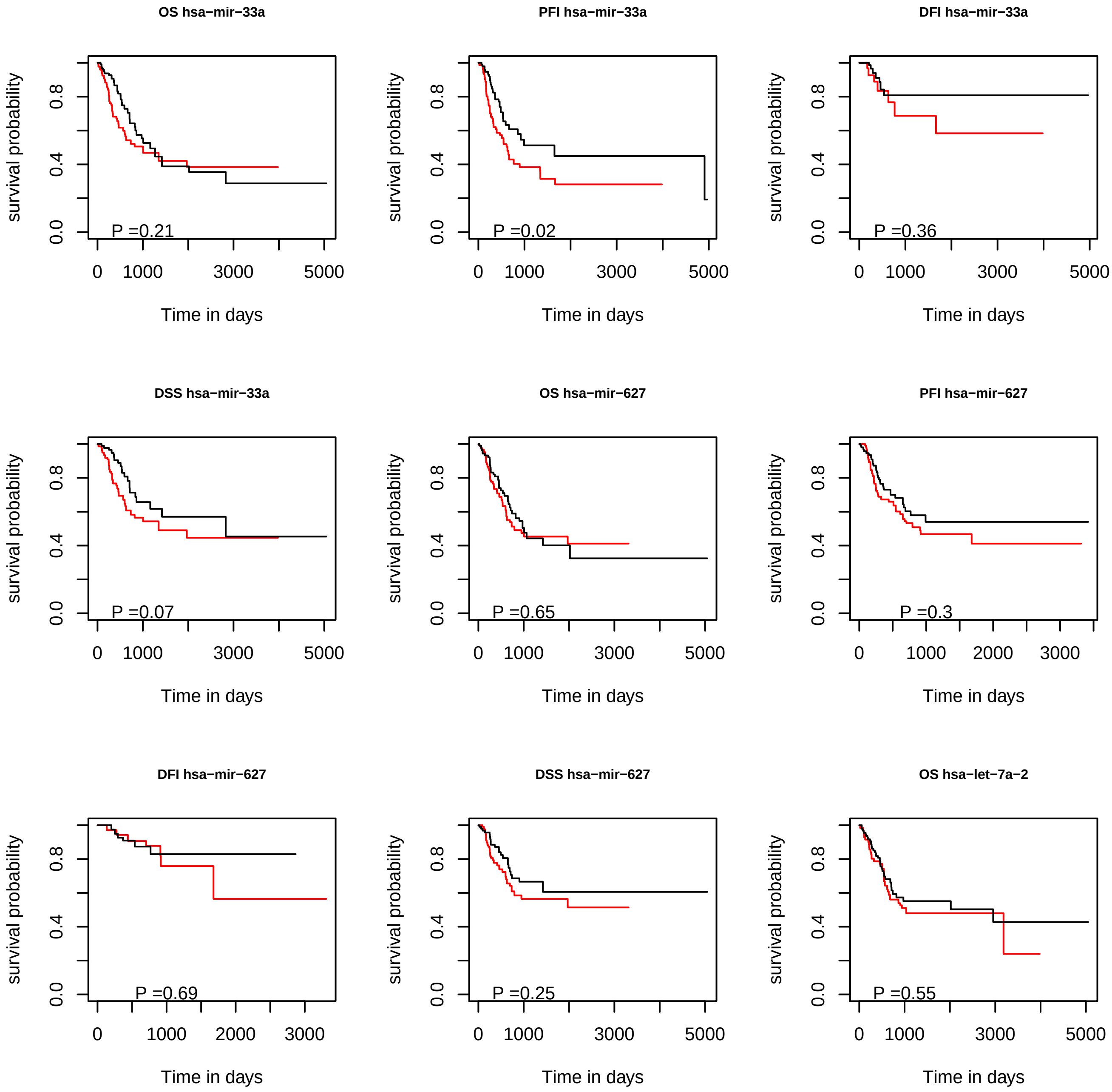
OS hsa−mir−33a
PFI hsa−mir−33a
DFI hsa−mir−33a
DSS hsa−mir−33a
OS hsa−mir−627
PFI hsa−mir−627
DFI hsa−mir−627
DSS hsa−mir−627
OS hsa−let−7a−2
survival probability
Time in days
0.0
0.4
0.8
0
1000
2000
3000
5000
P =0.21
P =0.02
P =0.36
P =0.07
P =0.65
P =0.3
P =0.69
P =0.25
P =0.55

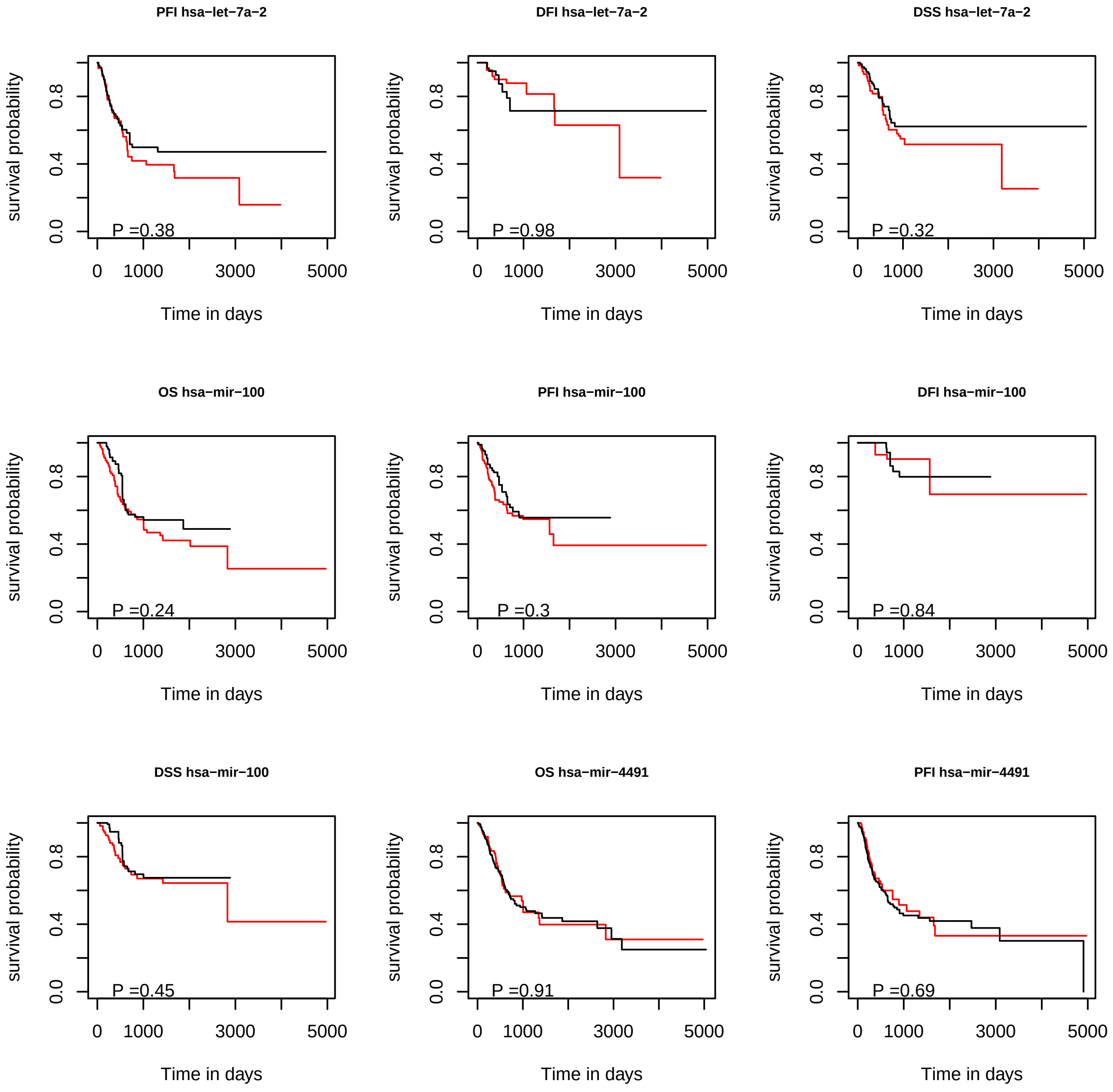
PFI hsa−let−7a−2
P =0.38
DFI hsa−let−7a−2
P =0.98
DSS hsa−let−7a−2
P =0.32
OS hsa−mir−100
P =0.24
PFI hsa−mir−100
P =0.3
DFI hsa−mir−100
P =0.84
DSS hsa−mir−100
P =0.45
OS hsa−mir−4491
P =0.91
PFI hsa−mir−4491
P =0.69
survival probability
Time in days
0.0
0.4
0.8
0
1000
3000
5000

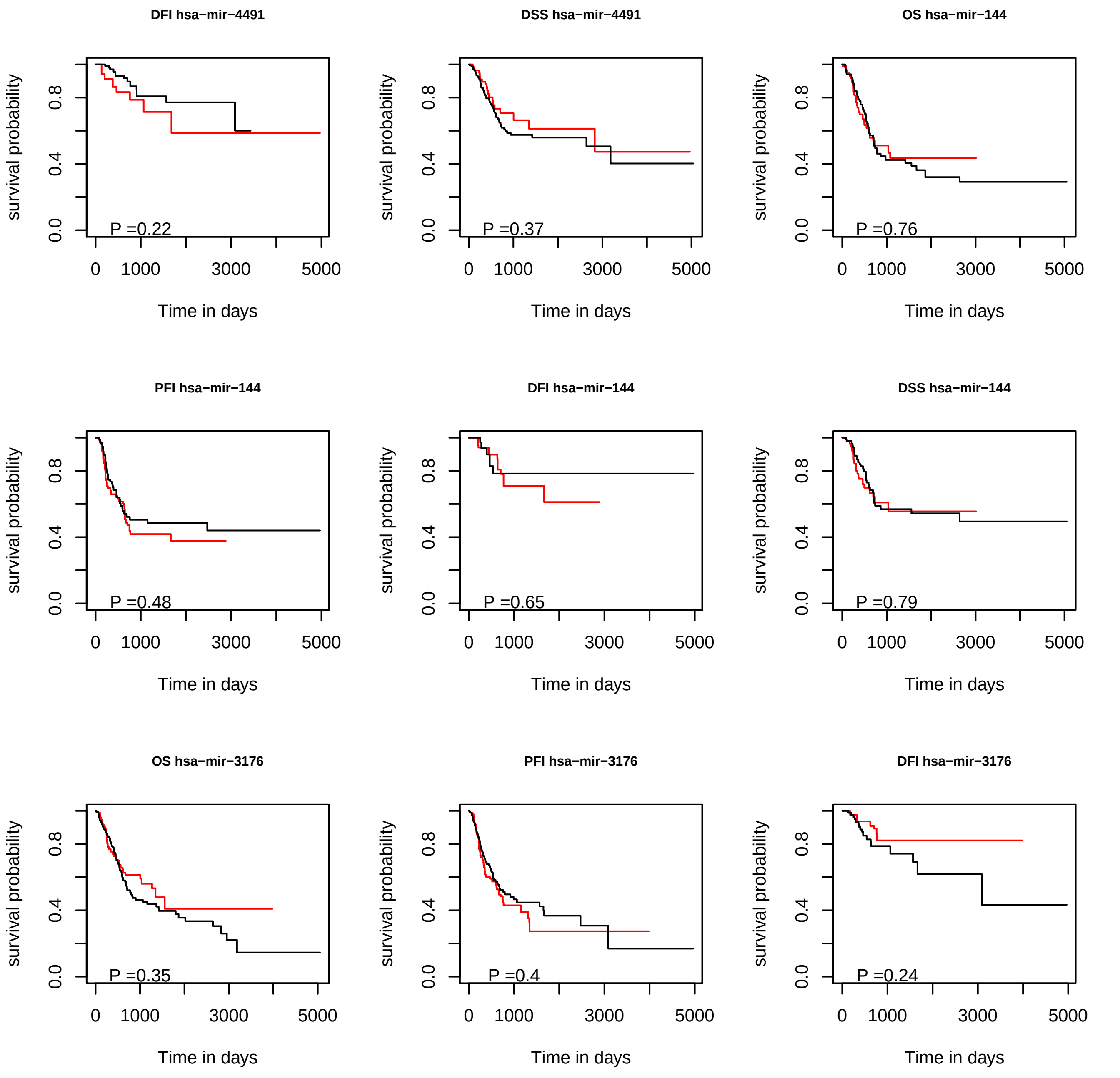
DFI hsa−mir−4491
P =0.22
DSS hsa−mir−4491
P =0.37
OS hsa−mir−144
P =0.76
PFI hsa−mir−144
P =0.48
DFI hsa−mir−144
P =0.65
DSS hsa−mir−144
P =0.79
OS hsa−mir−3176
P =0.35
PFI hsa−mir−3176
P =0.4
DFI hsa−mir−3176
P =0.24
survival probability
0.0
0.4
0.8
Time in days
0
1000
3000
5000

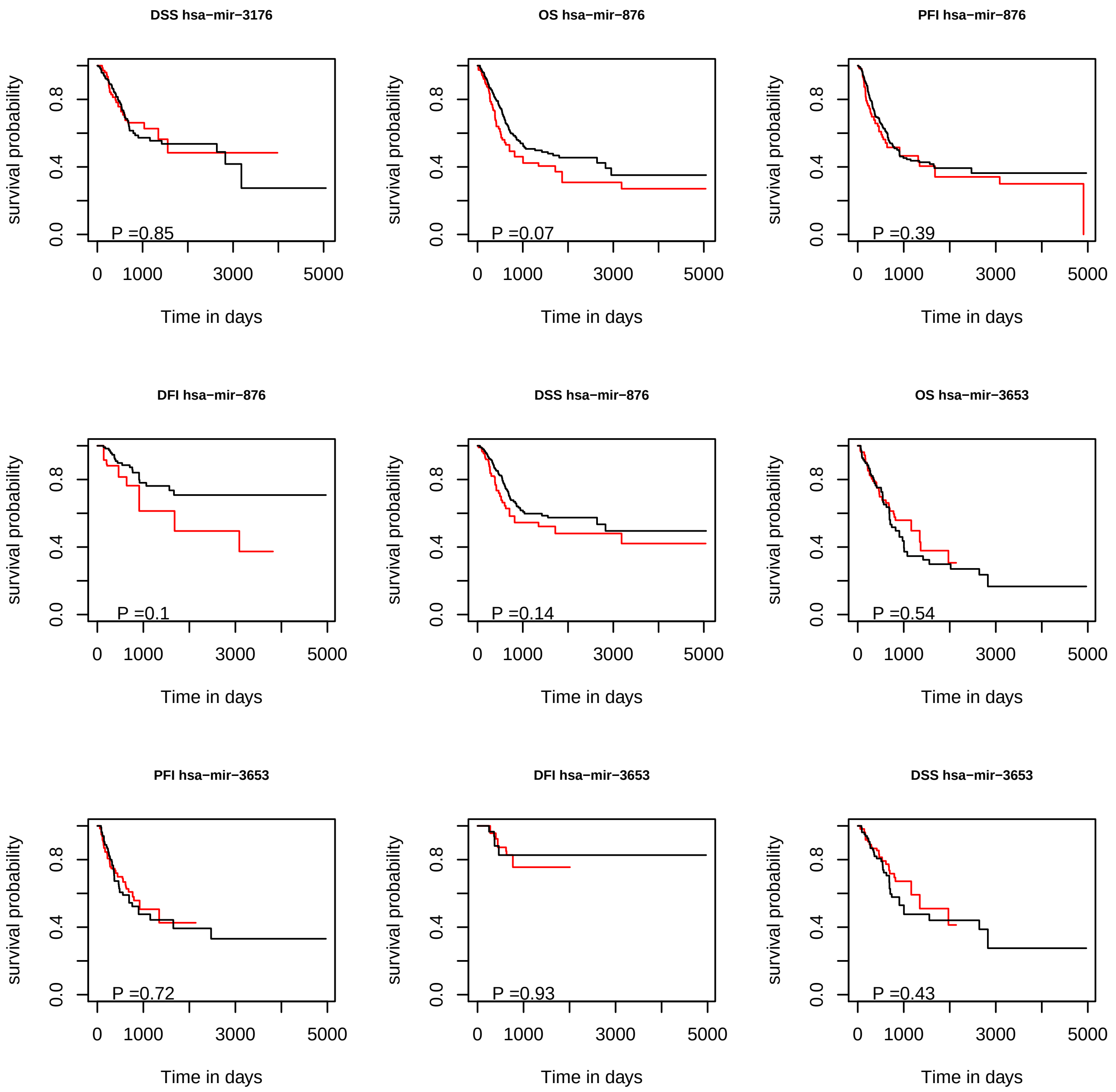
DSS hsa−mir−3176
P =0.85
OS hsa−mir−876
P =0.07
PFI hsa−mir−876
P =0.39
DFI hsa−mir−876
P =0.1
DSS hsa−mir−876
P =0.14
OS hsa−mir−3653
P =0.54
PFI hsa−mir−3653
P =0.72
DFI hsa−mir−3653
P =0.93
DSS hsa−mir−3653
P =0.43
survival probability
0.0
0.4
0.8
Time in days
0
1000
3000
5000

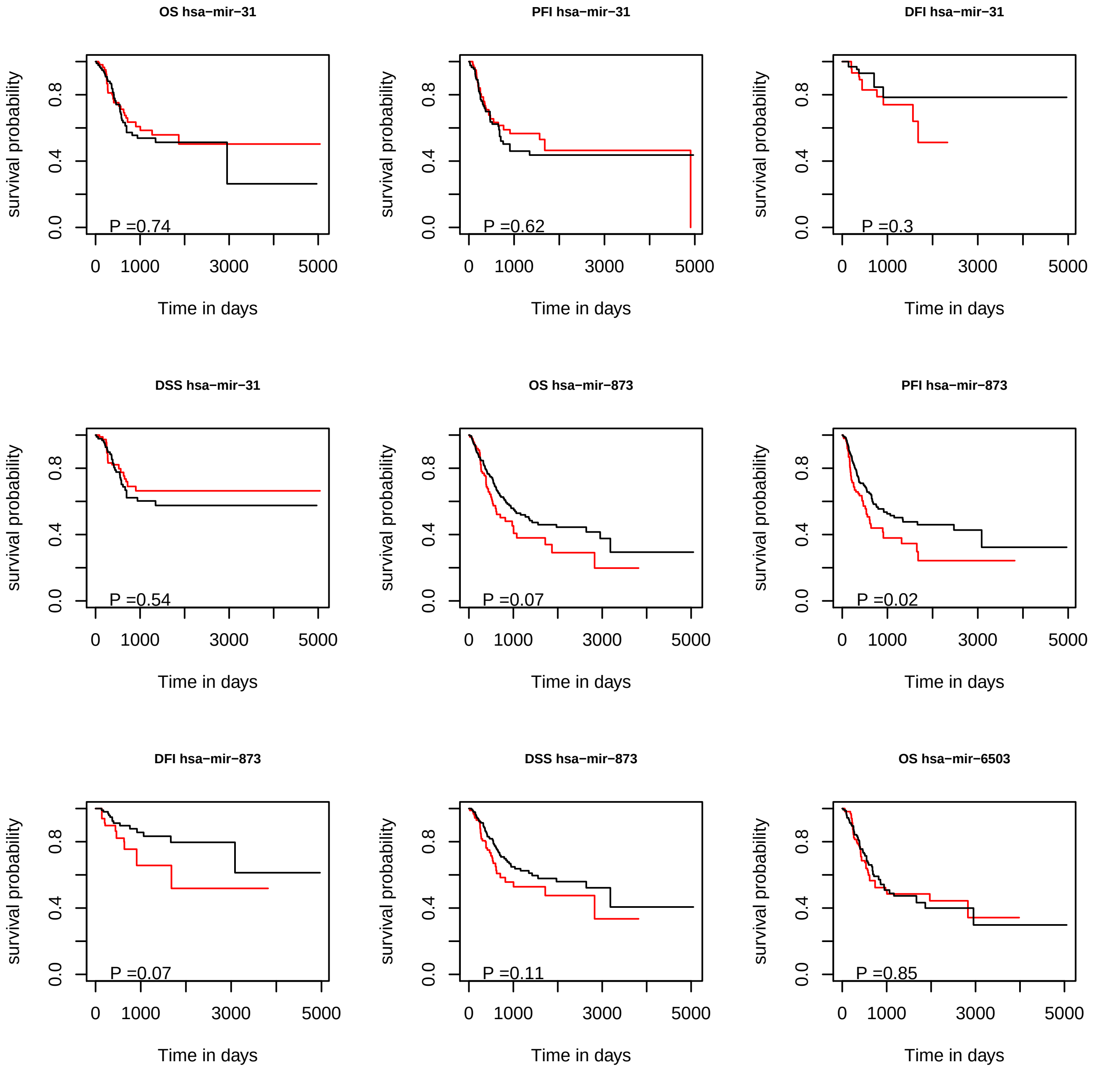
OS hsa−mir−31
survival probability
0.0
0.4
0.8
P =0.74
0
1000
3000
5000
Time in days
PFI hsa−mir−31
survival probability
0.0
0.4
0.8
P =0.62
0
1000
3000
5000
Time in days
DFI hsa−mir−31
survival probability
0.0
0.4
0.8
P =0.3
0
1000
3000
5000
Time in days
DSS hsa−mir−31
survival probability
0.0
0.4
0.8
P =0.54
0
1000
3000
5000
Time in days
OS hsa−mir−873
survival probability
0.0
0.4
0.8
P =0.07
0
1000
3000
5000
Time in days
PFI hsa−mir−873
survival probability
0.0
0.4
0.8
P =0.02
0
1000
3000
5000
Time in days
DFI hsa−mir−873
survival probability
0.0
0.4
0.8
P =0.07
0
1000
3000
5000
Time in days
DSS hsa−mir−873
survival probability
0.0
0.4
0.8
P =0.11
0
1000
3000
5000
Time in days
OS hsa−mir−6503
survival probability
0.0
0.4
0.8
P =0.85
0
1000
3000
5000
Time in days

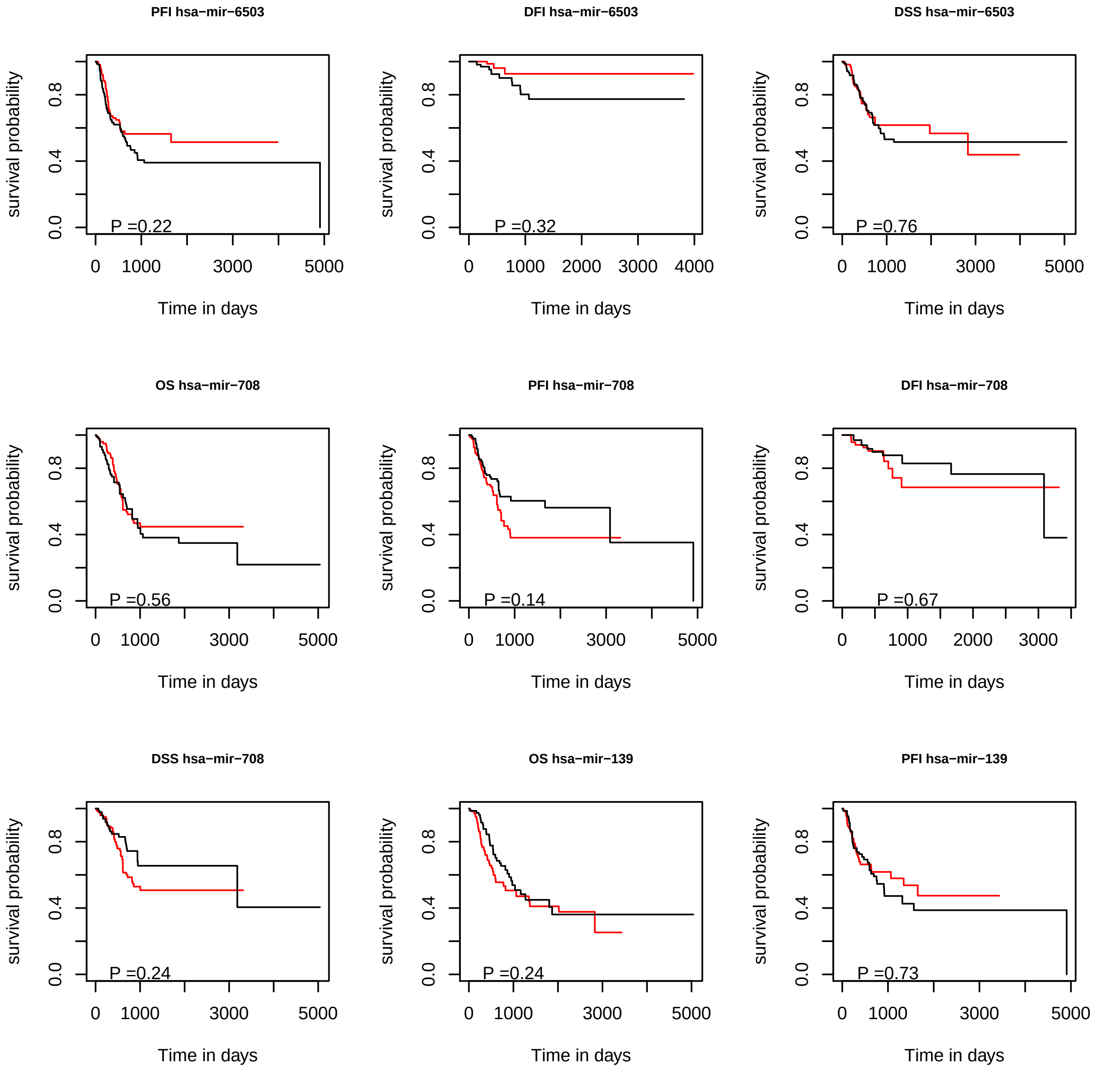
PFI hsa−mir−6503
survival probability
P =0.22
Time in days
DFI hsa−mir−6503
survival probability
P =0.32
Time in days
DSS hsa−mir−6503
survival probability
P =0.76
Time in days
OS hsa−mir−708
survival probability
P =0.56
Time in days
PFI hsa−mir−708
survival probability
P =0.14
Time in days
DFI hsa−mir−708
survival probability
P =0.67
Time in days
DSS hsa−mir−708
survival probability
P =0.24
Time in days
OS hsa−mir−139
survival probability
P =0.24
Time in days
PFI hsa−mir−139
survival probability
P =0.73
Time in days

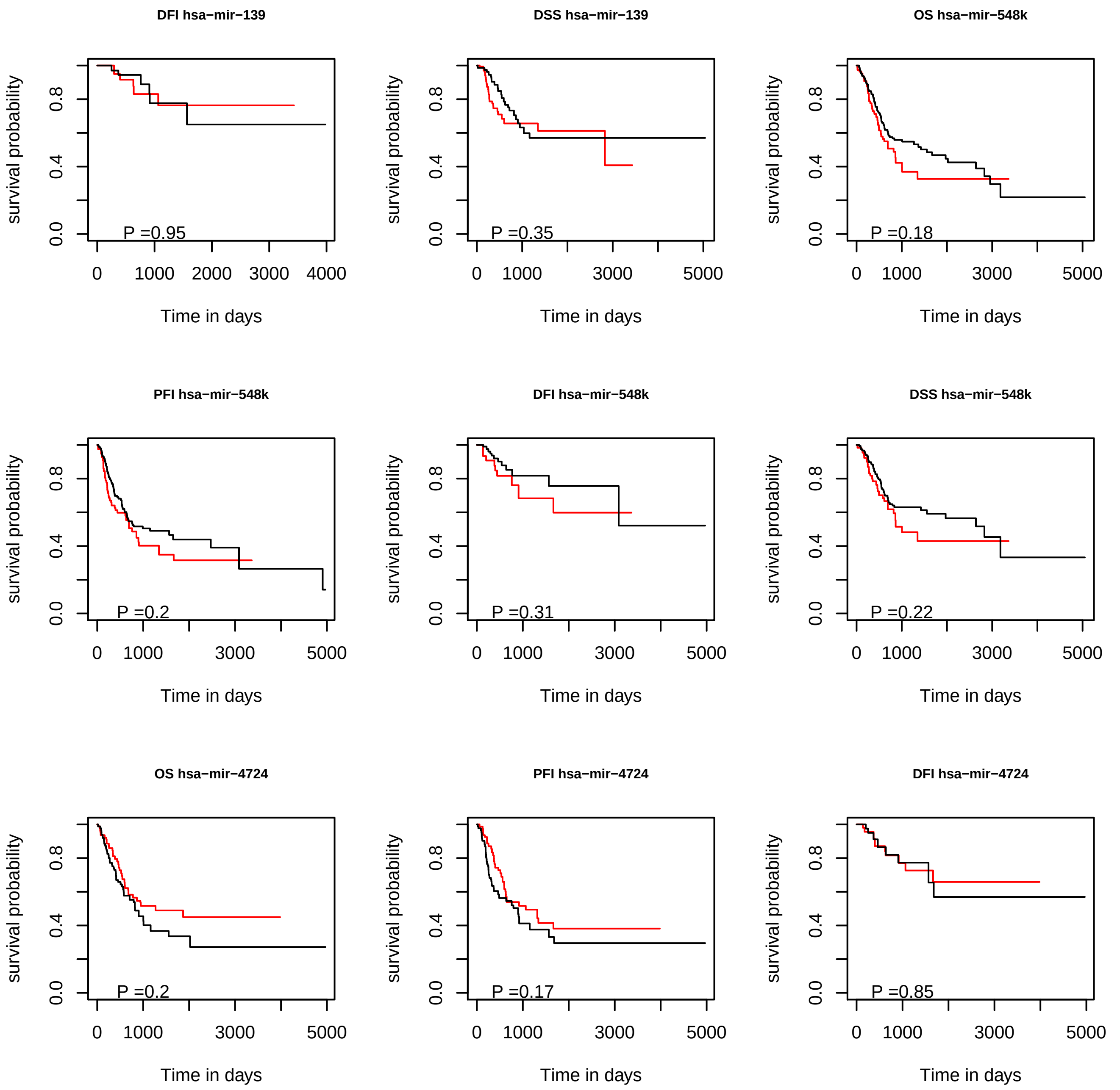
DFI hsa−mir−139
P =0.95
DSS hsa−mir−139
P =0.35
OS hsa−mir−548k
P =0.18
PFI hsa−mir−548k
P =0.2
DFI hsa−mir−548k
P =0.31
DSS hsa−mir−548k
P =0.22
OS hsa−mir−4724
P =0.2
PFI hsa−mir−4724
P =0.17
DFI hsa−mir−4724
P =0.85
survival probability
Time in days

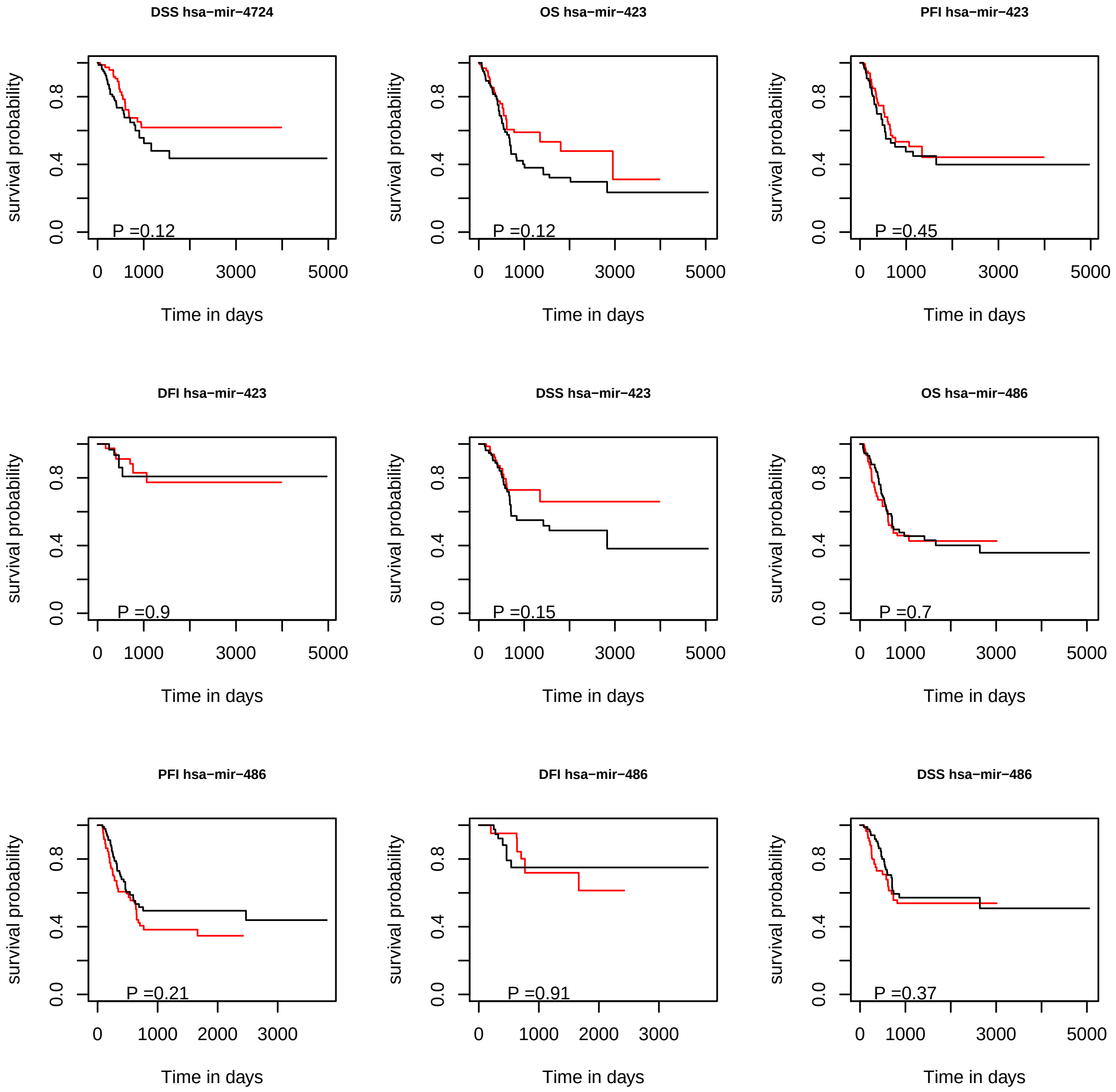
DSS hsa−mir−4724
P =0.12
OS hsa−mir−423
P =0.12
PFI hsa−mir−423
P =0.45
DFI hsa−mir−423
P =0.9
DSS hsa−mir−423
P =0.15
OS hsa−mir−486
P =0.7
PFI hsa−mir−486
P =0.21
DFI hsa−mir−486
P =0.91
DSS hsa−mir−486
P =0.37
survival probability
Time in days
0.0
0.4
0.8
0
1000
2000
3000
5000

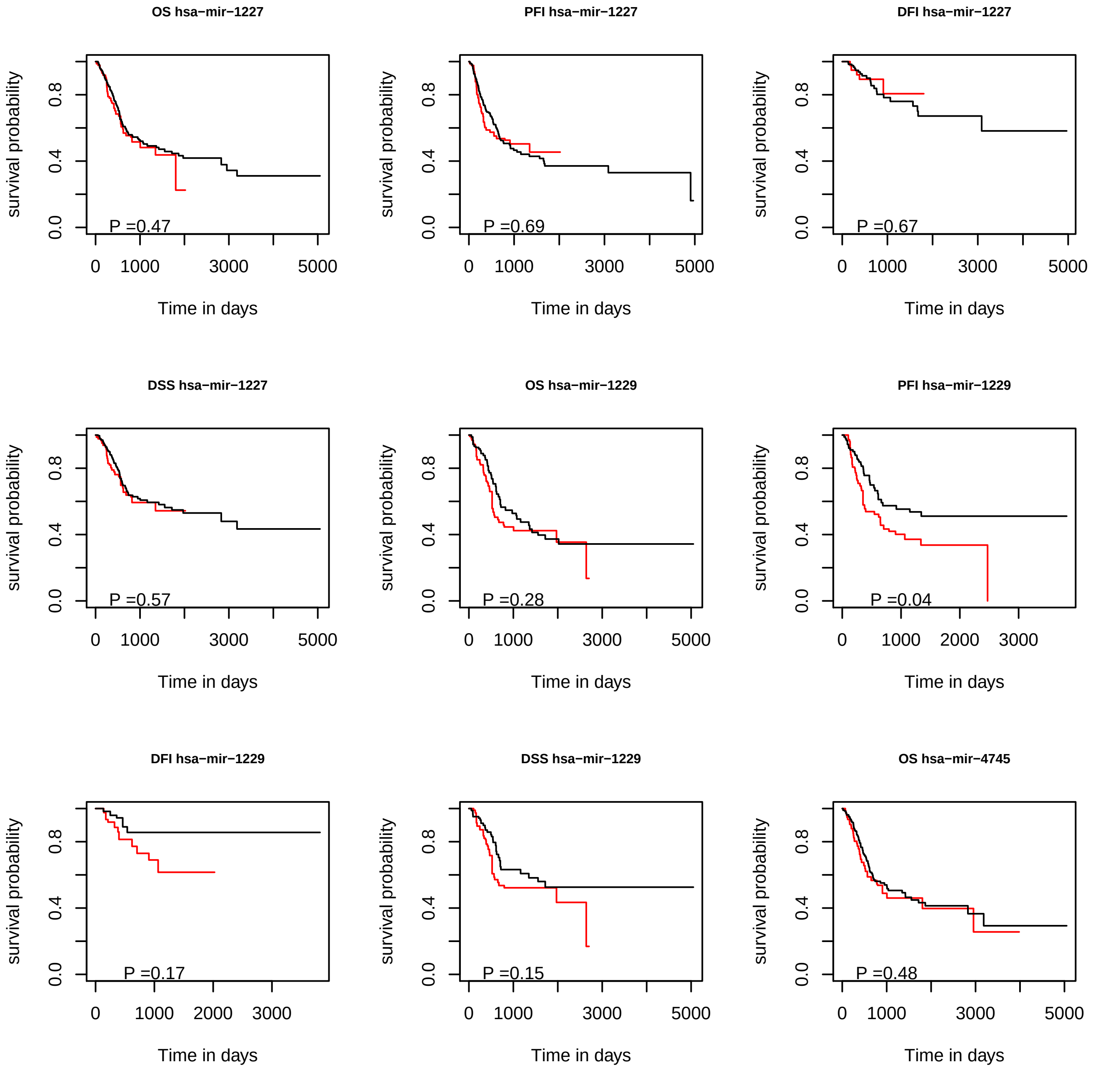
OS hsa−mir−1227
P =0.47
PFI hsa−mir−1227
P =0.69
DFI hsa−mir−1227
P =0.67
DSS hsa−mir−1227
P =0.57
OS hsa−mir−1229
P =0.28
PFI hsa−mir−1229
P =0.04
DFI hsa−mir−1229
P =0.17
DSS hsa−mir−1229
P =0.15
OS hsa−mir−4745
P =0.48
survival probability
Time in days

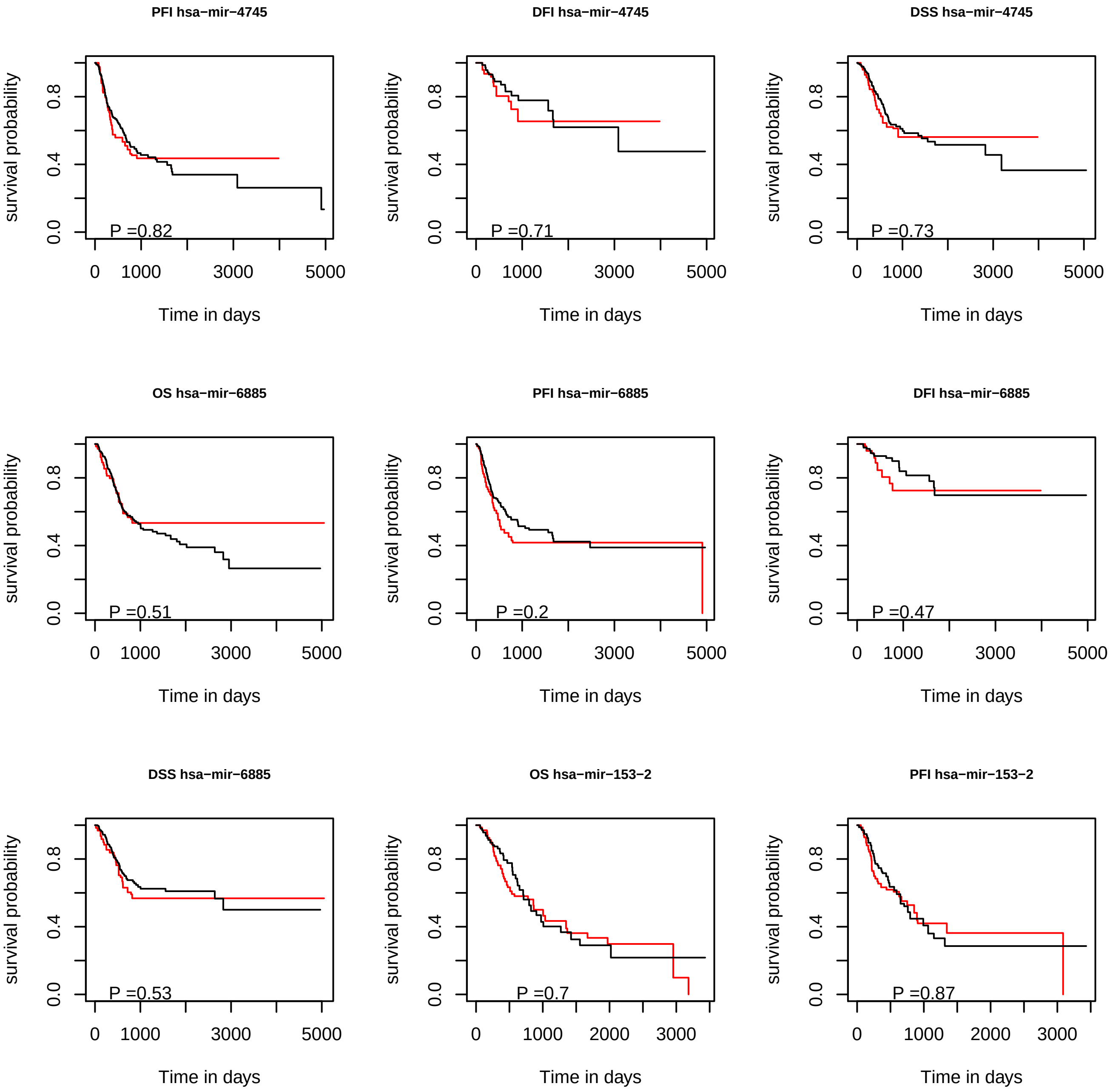
PFI hsa−mir−4745
P =0.82
DFI hsa−mir−4745
P =0.71
DSS hsa−mir−4745
P =0.73
OS hsa−mir−6885
P =0.51
PFI hsa−mir−6885
P =0.2
DFI hsa−mir−6885
P =0.47
DSS hsa−mir−6885
P =0.53
OS hsa−mir−153−2
P =0.7
PFI hsa−mir−153−2
P =0.87
survival probability
Time in days
0.0
0.4
0.8
0
1000
2000
3000
5000

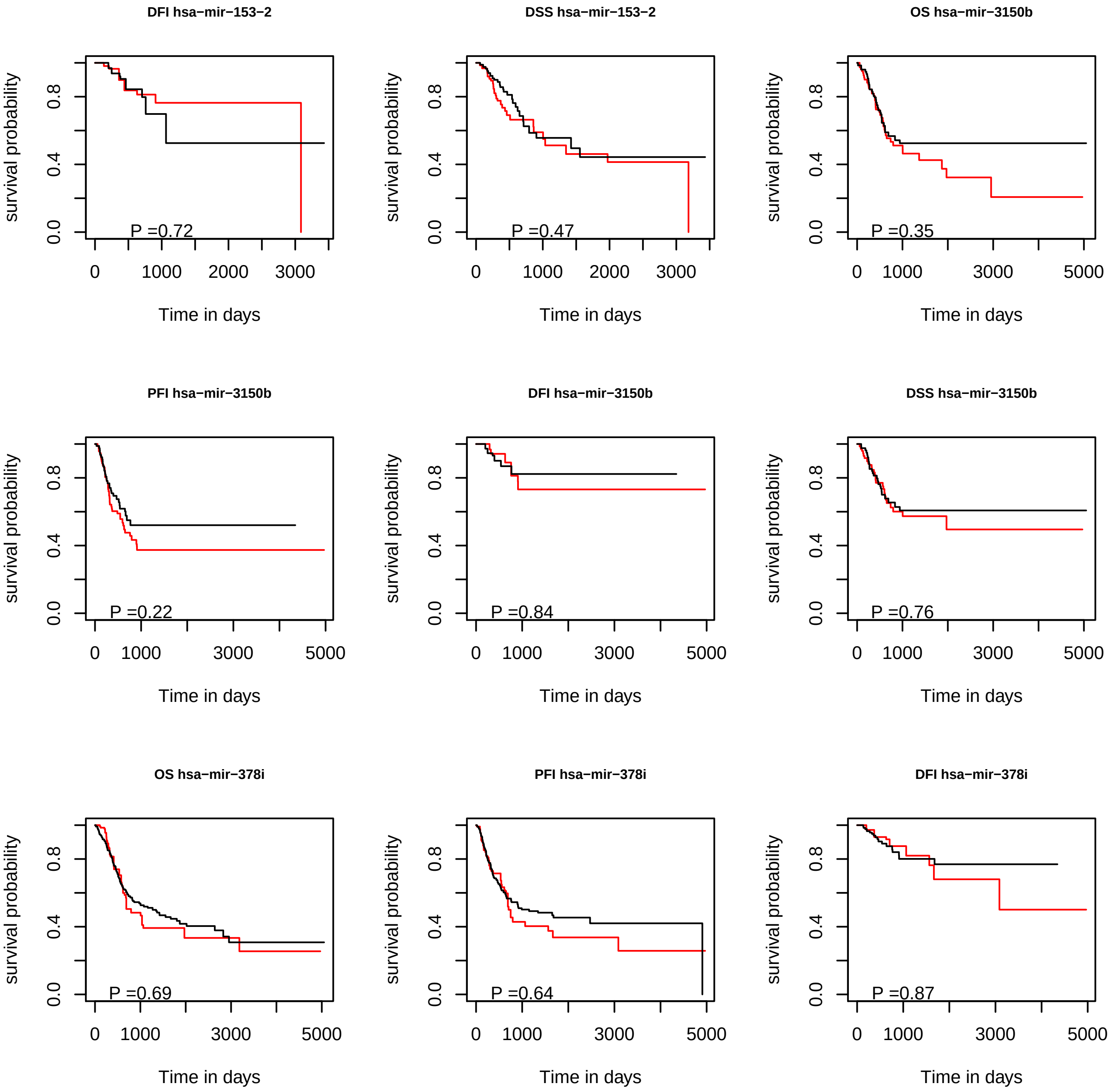
DFI hsa−mir−153−2
P =0.72
DSS hsa−mir−153−2
P =0.47
OS hsa−mir−3150b
P =0.35
PFI hsa−mir−3150b
P =0.22
DFI hsa−mir−3150b
P =0.84
DSS hsa−mir−3150b
P =0.76
OS hsa−mir−378i
P =0.69
PFI hsa−mir−378i
P =0.64
DFI hsa−mir−378i
P =0.87
survival probability
Time in days
0.0
0.4
0.8
0
1000
2000
3000
5000

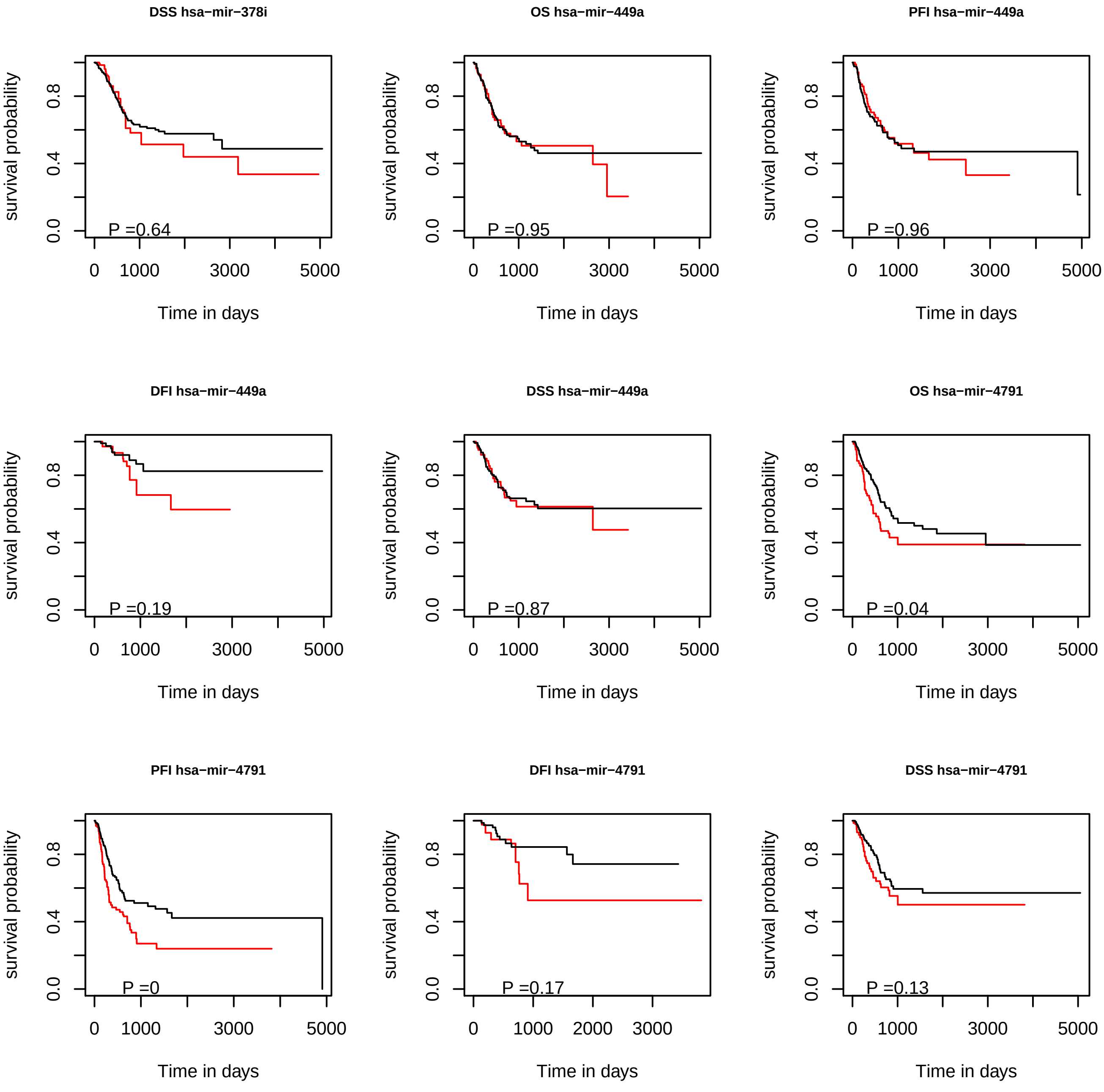
DSS hsa−mir−378i
P =0.64
OS hsa−mir−449a
P =0.95
PFI hsa−mir−449a
P =0.96
DFI hsa−mir−449a
P =0.19
DSS hsa−mir−449a
P =0.87
OS hsa−mir−4791
P =0.04
PFI hsa−mir−4791
P =0
DFI hsa−mir−4791
P =0.17
DSS hsa−mir−4791
P =0.13
survival probability
Time in days

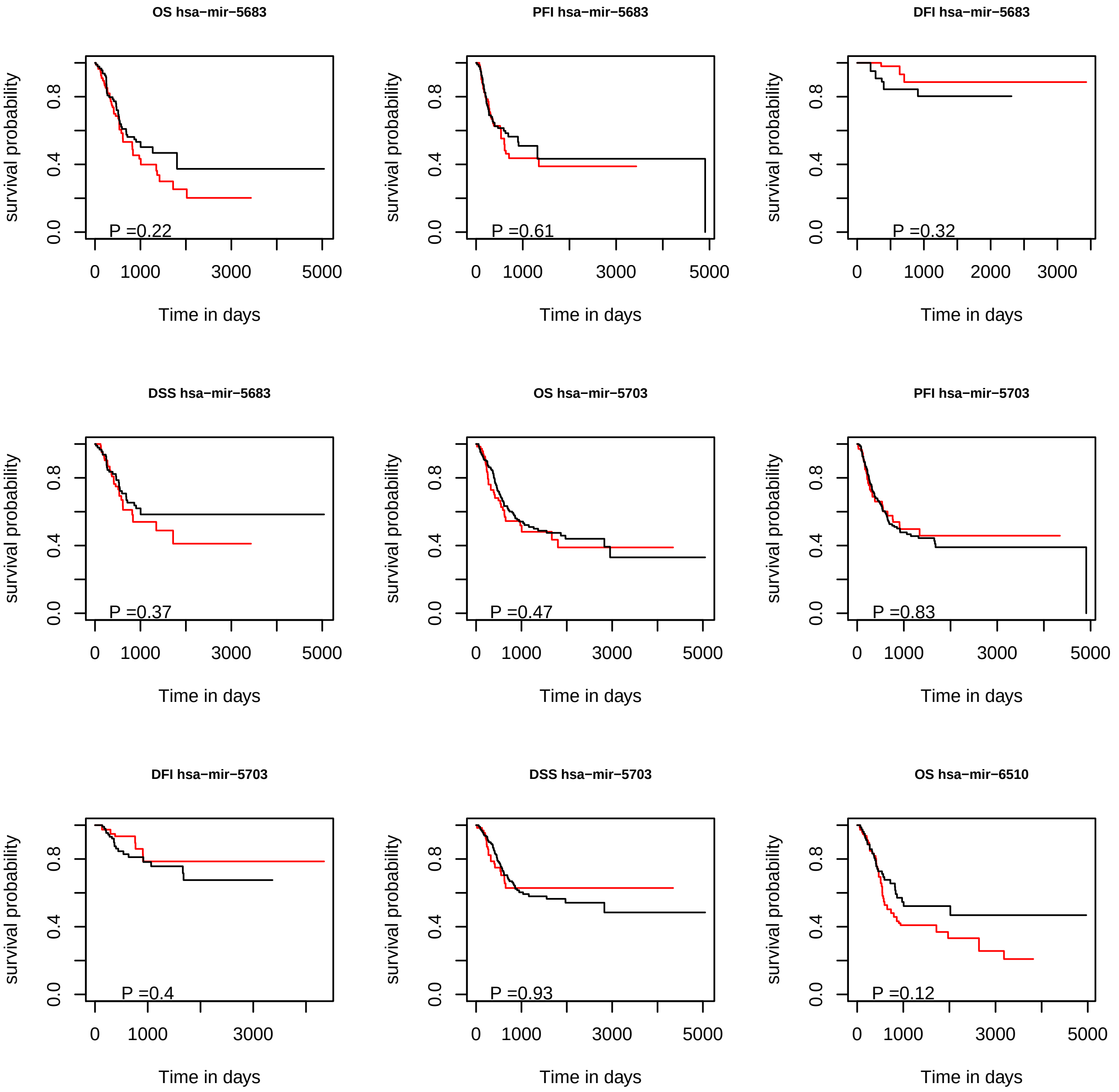
OS hsa−mir−5683
P =0.22
PFI hsa−mir−5683
P =0.61
DFI hsa−mir−5683
P =0.32
DSS hsa−mir−5683
P =0.37
OS hsa−mir−5703
P =0.47
PFI hsa−mir−5703
P =0.83
DFI hsa−mir−5703
P =0.4
DSS hsa−mir−5703
P =0.93
OS hsa−mir−6510
P =0.12
survival probability
Time in days

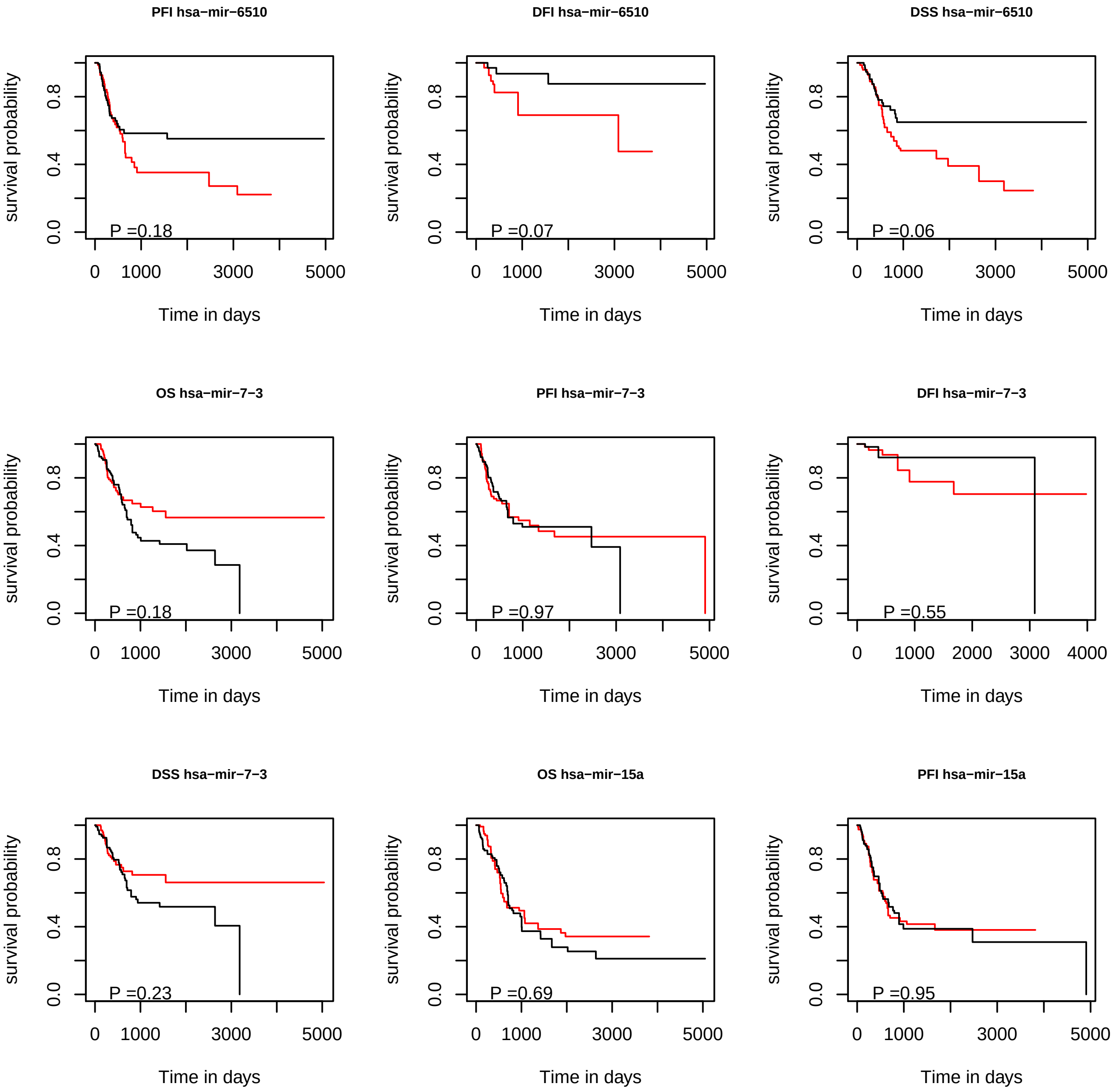
PFI hsa−mir−6510
P =0.18
DFI hsa−mir−6510
P =0.07
DSS hsa−mir−6510
P =0.06
OS hsa−mir−7−3
P =0.18
PFI hsa−mir−7−3
P =0.97
DFI hsa−mir−7−3
P =0.55
DSS hsa−mir−7−3
P =0.23
OS hsa−mir−15a
P =0.69
PFI hsa−mir−15a
P =0.95
survival probability
Time in days
0.0
0.4
0.8
0
1000
2000
3000
4000
5000

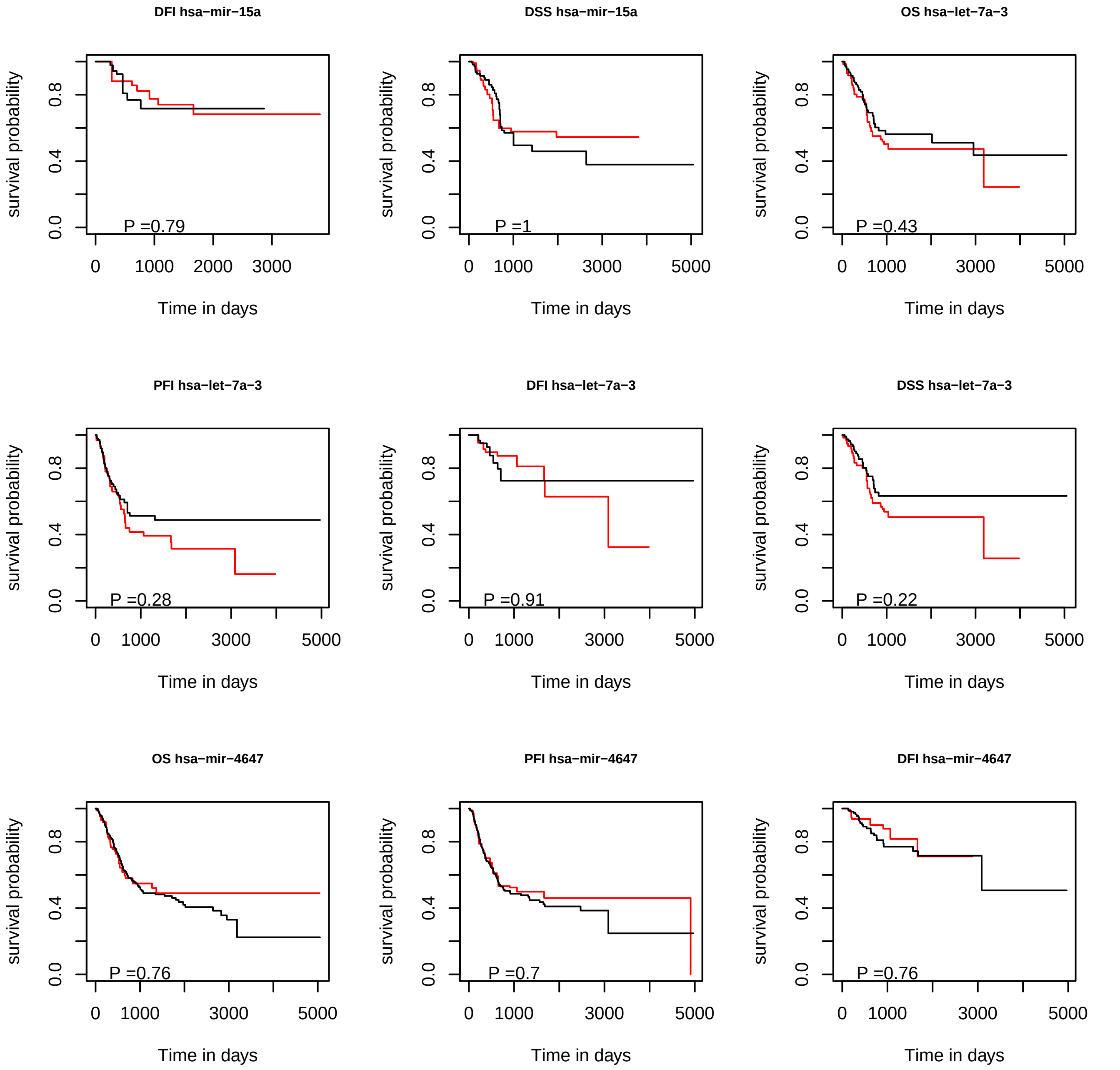
DFI hsa−mir−15a
P =0.79
DSS hsa−mir−15a
P =1
OS hsa−let−7a−3
P =0.43
PFI hsa−let−7a−3
P =0.28
DFI hsa−let−7a−3
P =0.91
DSS hsa−let−7a−3
P =0.22
OS hsa−mir−4647
P =0.76
PFI hsa−mir−4647
P =0.7
DFI hsa−mir−4647
P =0.76
survival probability
Time in days

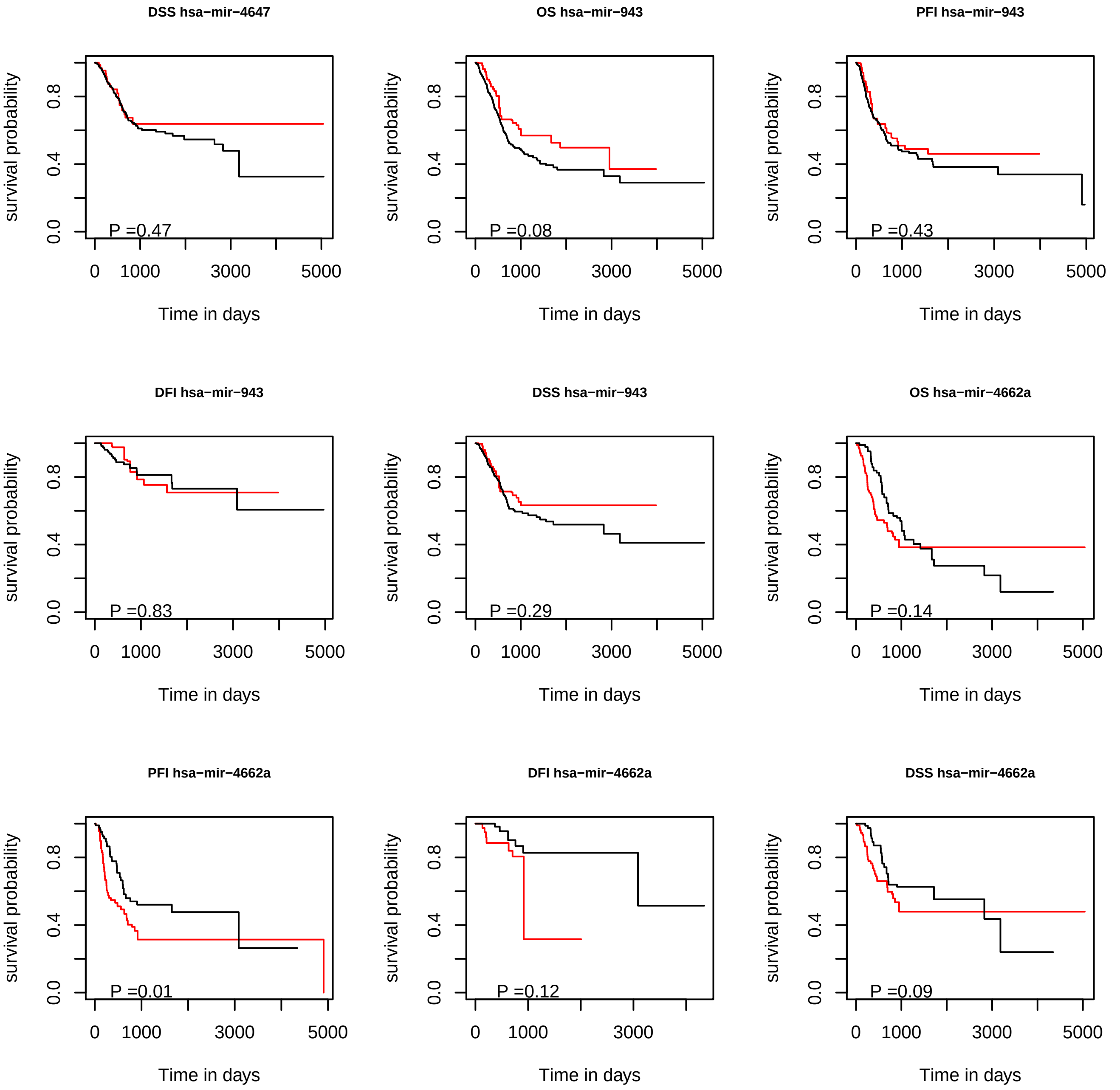
DSS hsa−mir−4647
P =0.47
OS hsa−mir−943
P =0.08
PFI hsa−mir−943
P =0.43
DFI hsa−mir−943
P =0.83
DSS hsa−mir−943
P =0.29
OS hsa−mir−4662a
P =0.14
PFI hsa−mir−4662a
P =0.01
DFI hsa−mir−4662a
P =0.12
DSS hsa−mir−4662a
P =0.09
survival probability
Time in days
0.0
0.4
0.8
0
1000
3000
5000

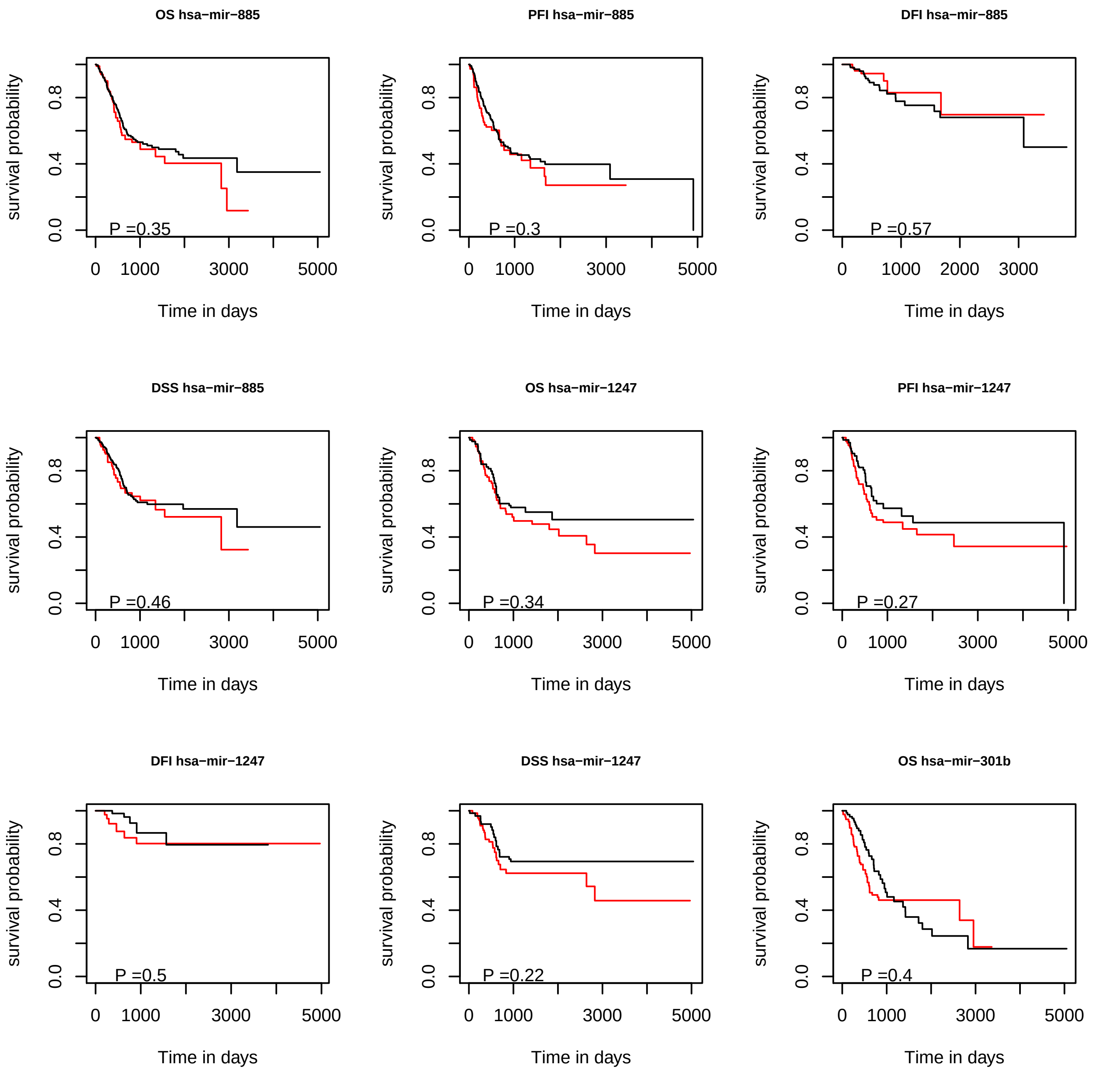
OS hsa−mir−885
P =0.35
PFI hsa−mir−885
P =0.3
DFI hsa−mir−885
P =0.57
DSS hsa−mir−885
P =0.46
OS hsa−mir−1247
P =0.34
PFI hsa−mir−1247
P =0.27
DFI hsa−mir−1247
P =0.5
DSS hsa−mir−1247
P =0.22
OS hsa−mir−301b
P =0.4
survival probability
0.0
0.4
0.8
Time in days
0
1000
2000
3000
5000

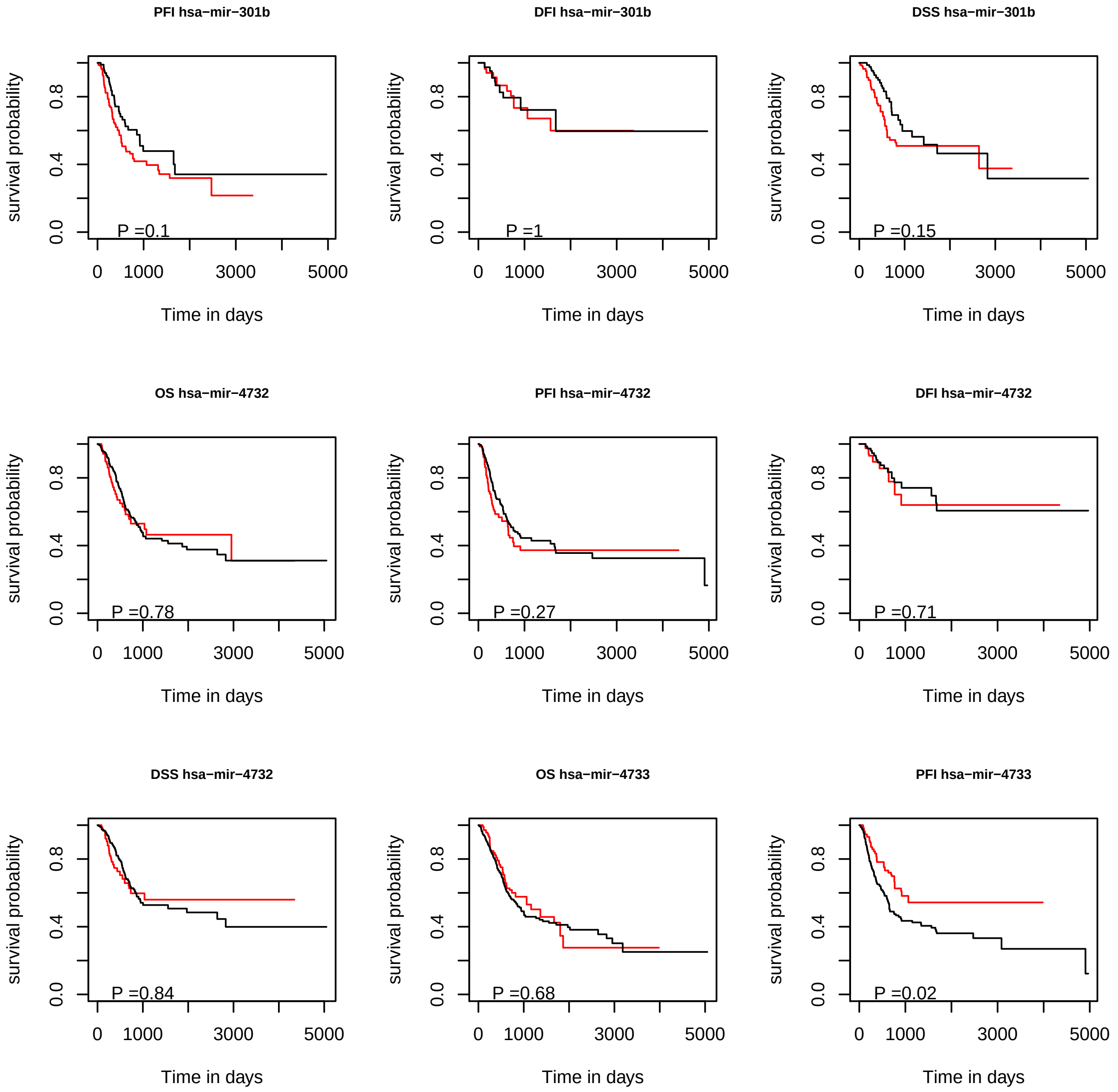
PFI hsa−mir−301b
P =0.1
DFI hsa−mir−301b
P =1
DSS hsa−mir−301b
P =0.15
OS hsa−mir−4732
P =0.78
PFI hsa−mir−4732
P =0.27
DFI hsa−mir−4732
P =0.71
DSS hsa−mir−4732
P =0.84
OS hsa−mir−4733
P =0.68
PFI hsa−mir−4733
P =0.02
survival probability
0.0
0.4
0.8
Time in days
0
1000
3000
5000

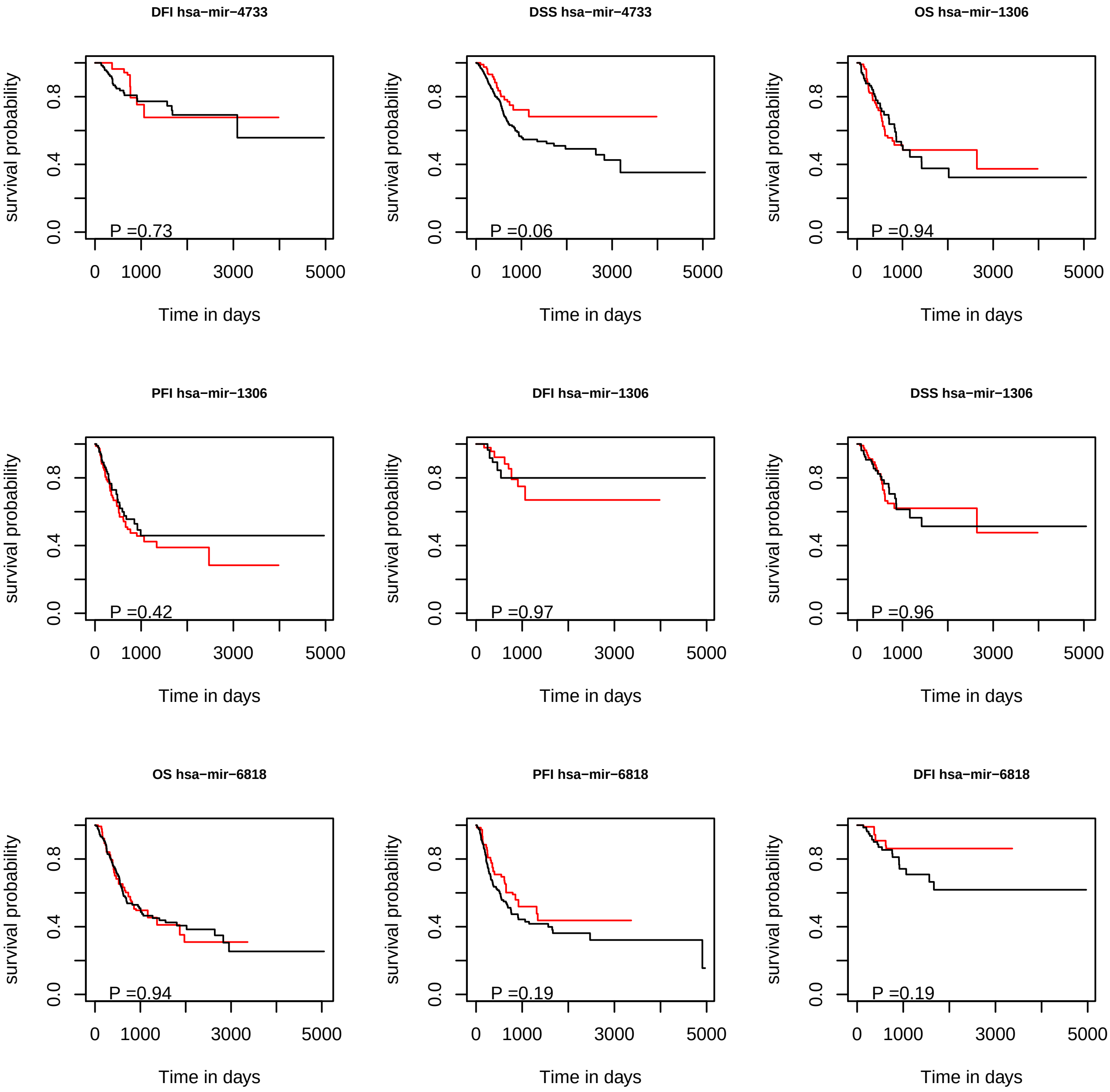
DFI hsa−mir−4733
P =0.73
DSS hsa−mir−4733
P =0.06
OS hsa−mir−1306
P =0.94
PFI hsa−mir−1306
P =0.42
DFI hsa−mir−1306
P =0.97
DSS hsa−mir−1306
P =0.96
OS hsa−mir−6818
P =0.94
PFI hsa−mir−6818
P =0.19
DFI hsa−mir−6818
P =0.19
survival probability
0.0
0.4
0.8
Time in days
0
1000
3000
5000

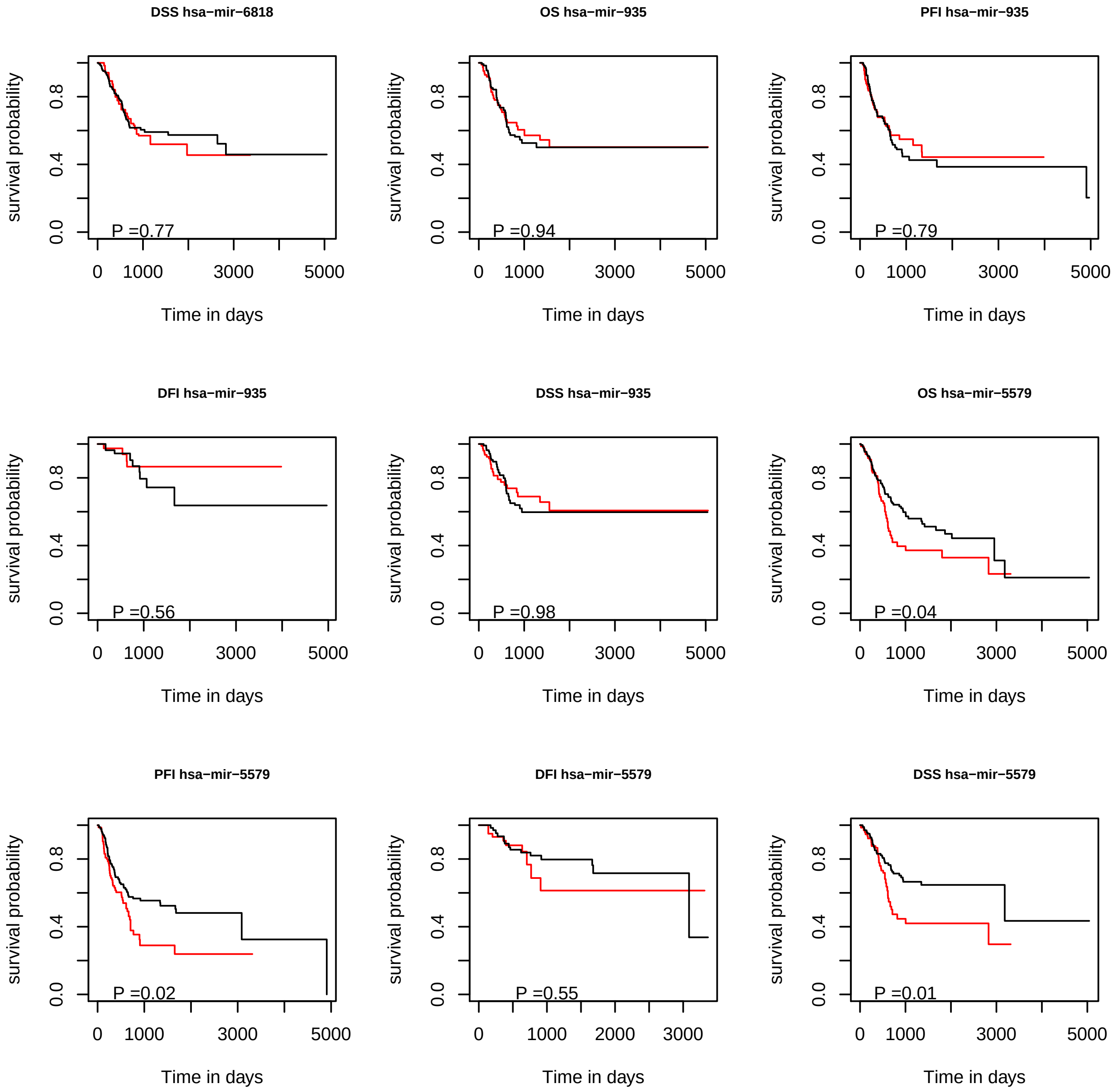
DSS hsa−mir−6818
P =0.77
OS hsa−mir−935
P =0.94
PFI hsa−mir−935
P =0.79
DFI hsa−mir−935
P =0.56
DSS hsa−mir−935
P =0.98
OS hsa−mir−5579
P =0.04
PFI hsa−mir−5579
P =0.02
DFI hsa−mir−5579
P =0.55
DSS hsa−mir−5579
P =0.01
survival probability
0.0
0.4
0.8
Time in days
0
1000
2000
3000
5000

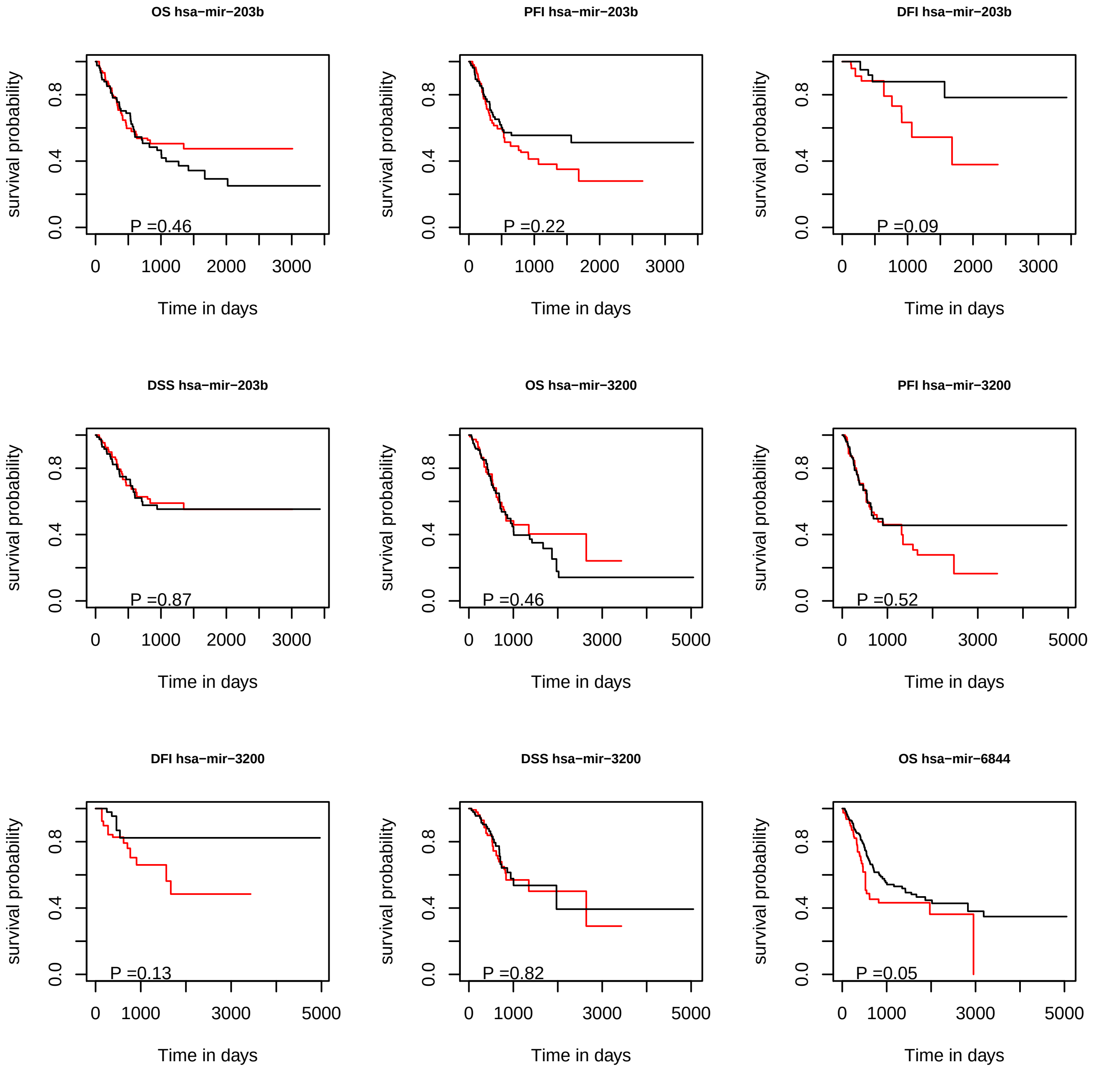
OS hsa−mir−203b
P =0.46
PFI hsa−mir−203b
P =0.22
DFI hsa−mir−203b
P =0.09
DSS hsa−mir−203b
P =0.87
OS hsa−mir−3200
P =0.46
PFI hsa−mir−3200
P =0.52
DFI hsa−mir−3200
P =0.13
DSS hsa−mir−3200
P =0.82
OS hsa−mir−6844
P =0.05
survival probability
Time in days
0.0
0.4
0.8
0
1000
2000
3000
5000

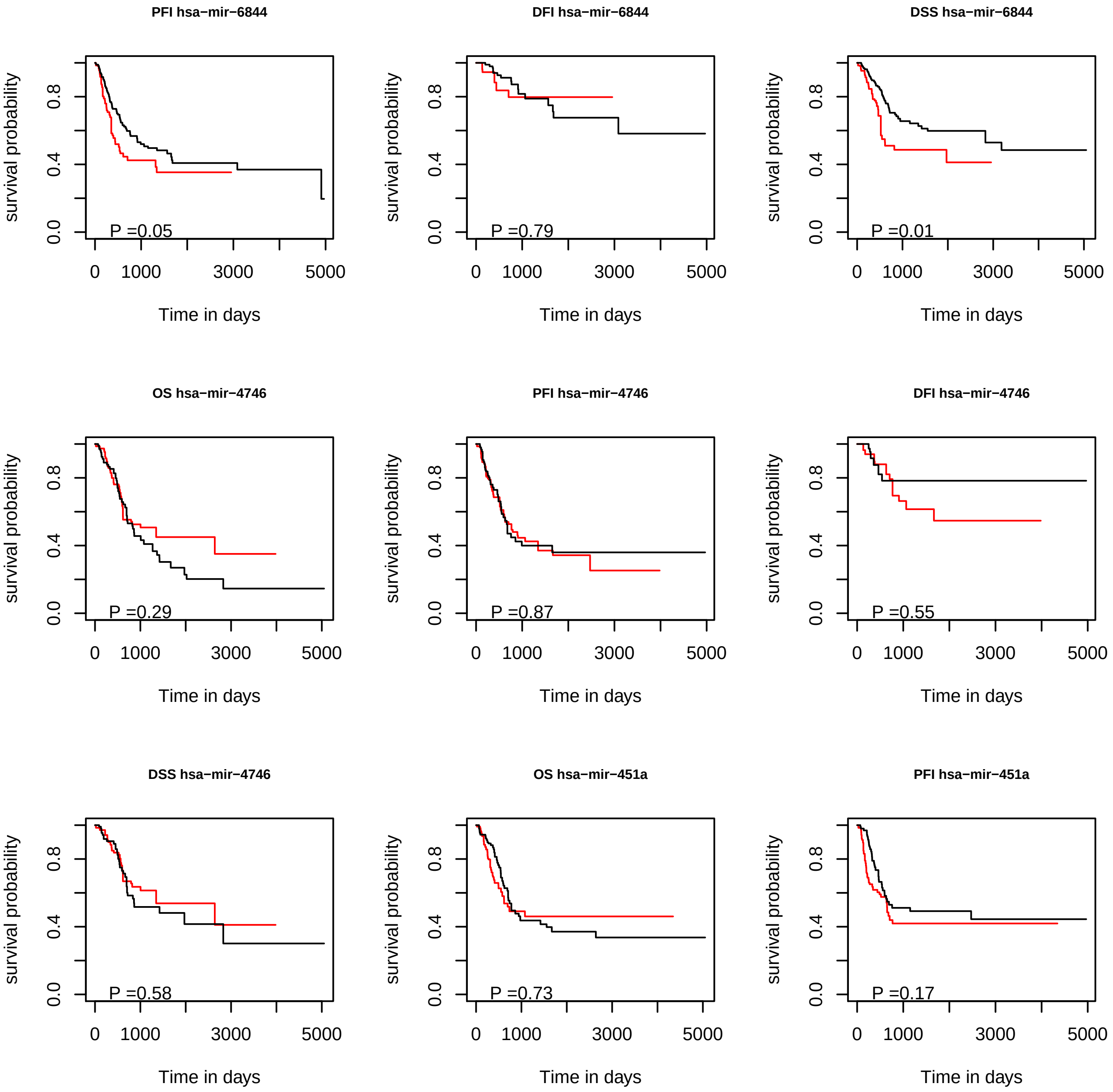

PFI hsa−mir−6844
P =0.05
DFI hsa−mir−6844
P =0.79
DSS hsa−mir−6844
P =0.01
OS hsa−mir−4746
P =0.29
PFI hsa−mir−4746
P =0.87
DFI hsa−mir−4746
P =0.55
DSS hsa−mir−4746
P =0.58
OS hsa−mir−451a
P =0.73
PFI hsa−mir−451a
P =0.17
survival probability
0.0
0.4
0.8
Time in days
0
1000
3000
5000

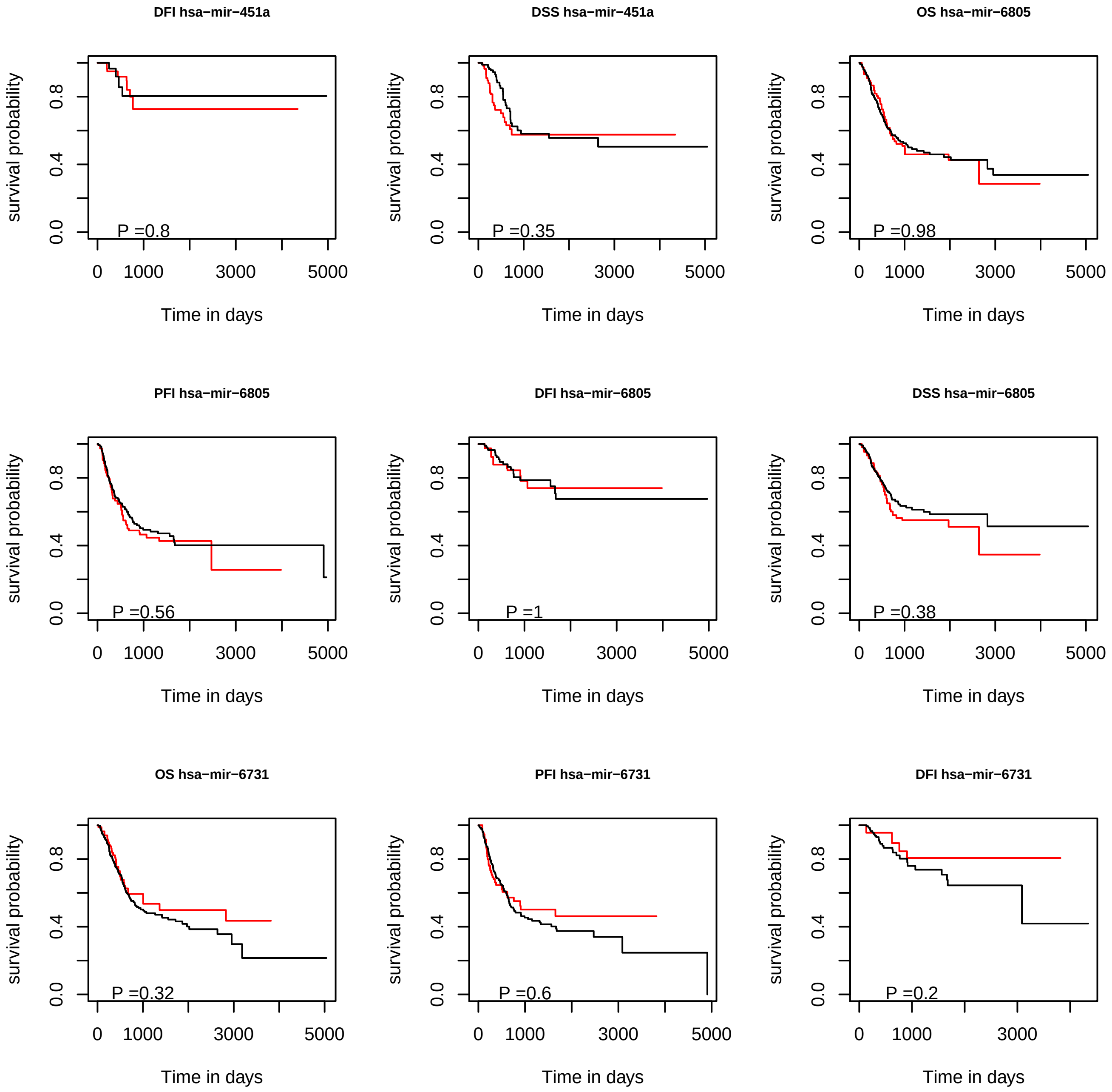
DFI hsa−mir−451a
P =0.8
DSS hsa−mir−451a
P =0.35
OS hsa−mir−6805
P =0.98
PFI hsa−mir−6805
P =0.56
DFI hsa−mir−6805
P =1
DSS hsa−mir−6805
P =0.38
OS hsa−mir−6731
P =0.32
PFI hsa−mir−6731
P =0.6
DFI hsa−mir−6731
P =0.2
survival probability
Time in days
0.0
0.4
0.8
0
1000
3000
5000

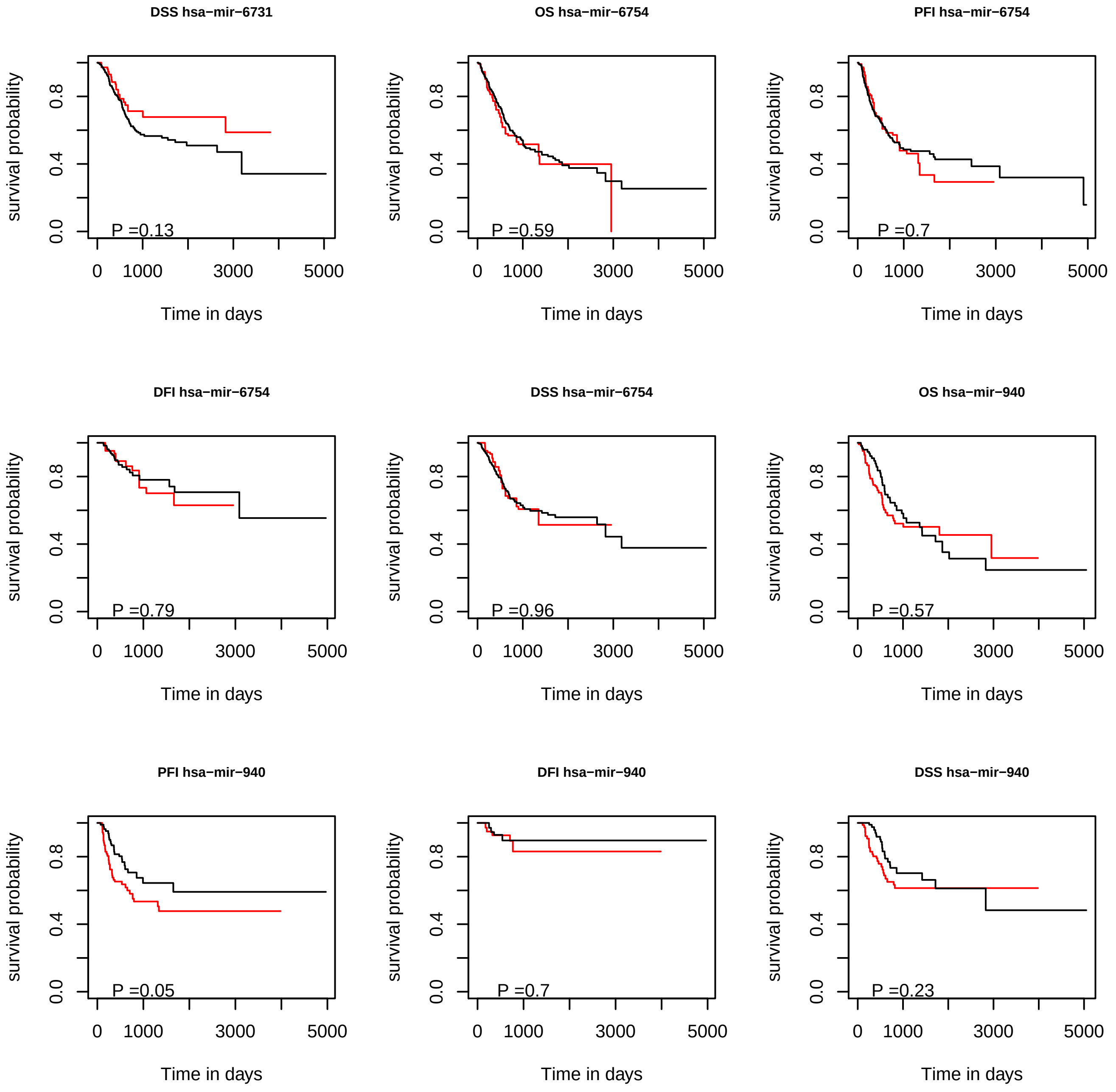
DSS hsa−mir−6731
P =0.13
OS hsa−mir−6754
P =0.59
PFI hsa−mir−6754
P =0.7
DFI hsa−mir−6754
P =0.79
DSS hsa−mir−6754
P =0.96
OS hsa−mir−940
P =0.57
PFI hsa−mir−940
P =0.05
DFI hsa−mir−940
P =0.7
DSS hsa−mir−940
P =0.23
survival probability
0.0
0.4
0.8
Time in days
0
1000
3000
5000

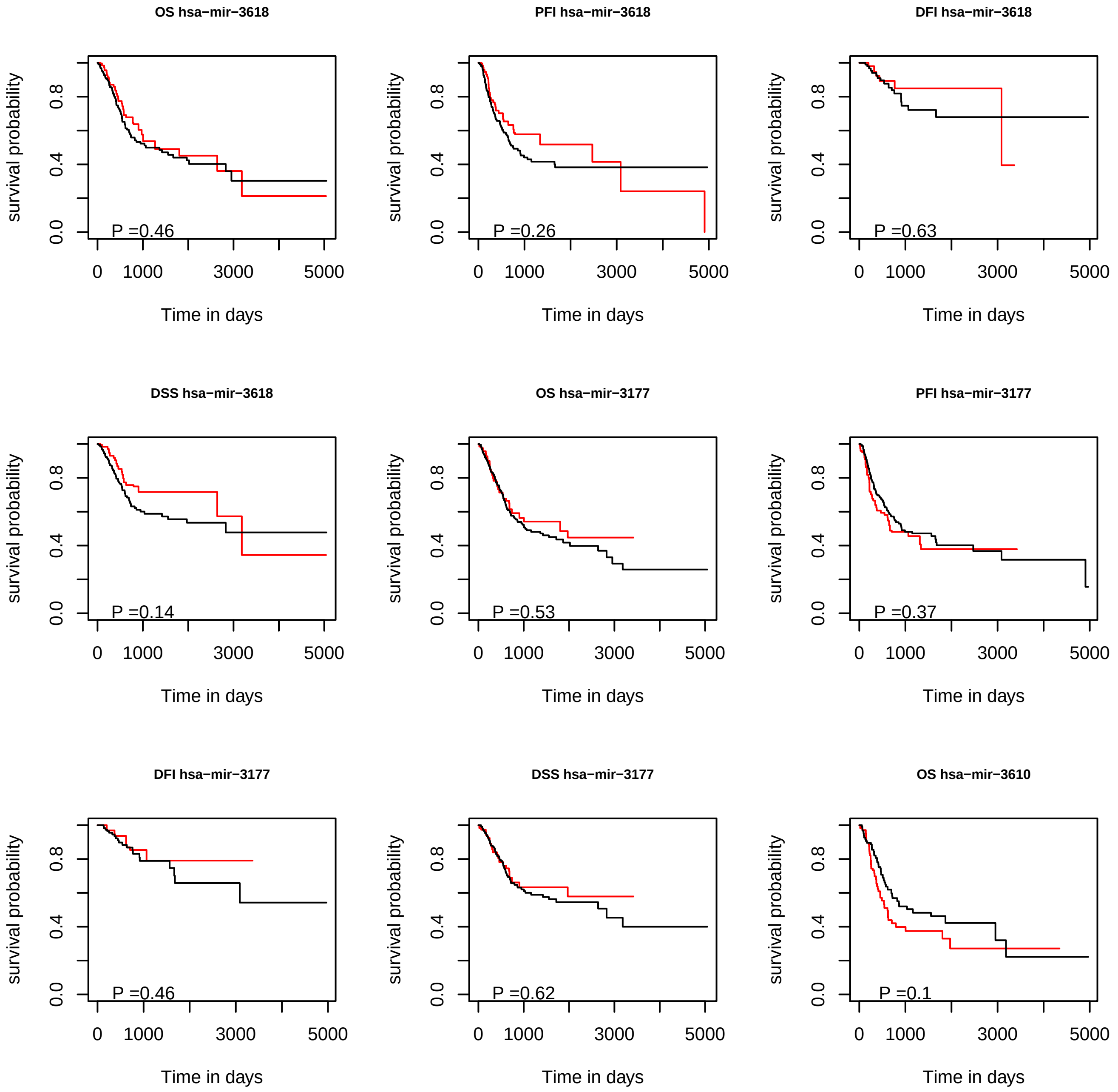
OS hsa−mir−3618
P =0.46
PFI hsa−mir−3618
P =0.26
DFI hsa−mir−3618
P =0.63
DSS hsa−mir−3618
P =0.14
OS hsa−mir−3177
P =0.53
PFI hsa−mir−3177
P =0.37
DFI hsa−mir−3177
P =0.46
DSS hsa−mir−3177
P =0.62
OS hsa−mir−3610
P =0.1
survival probability
0.0
0.4
0.8
Time in days
0
1000
3000
5000

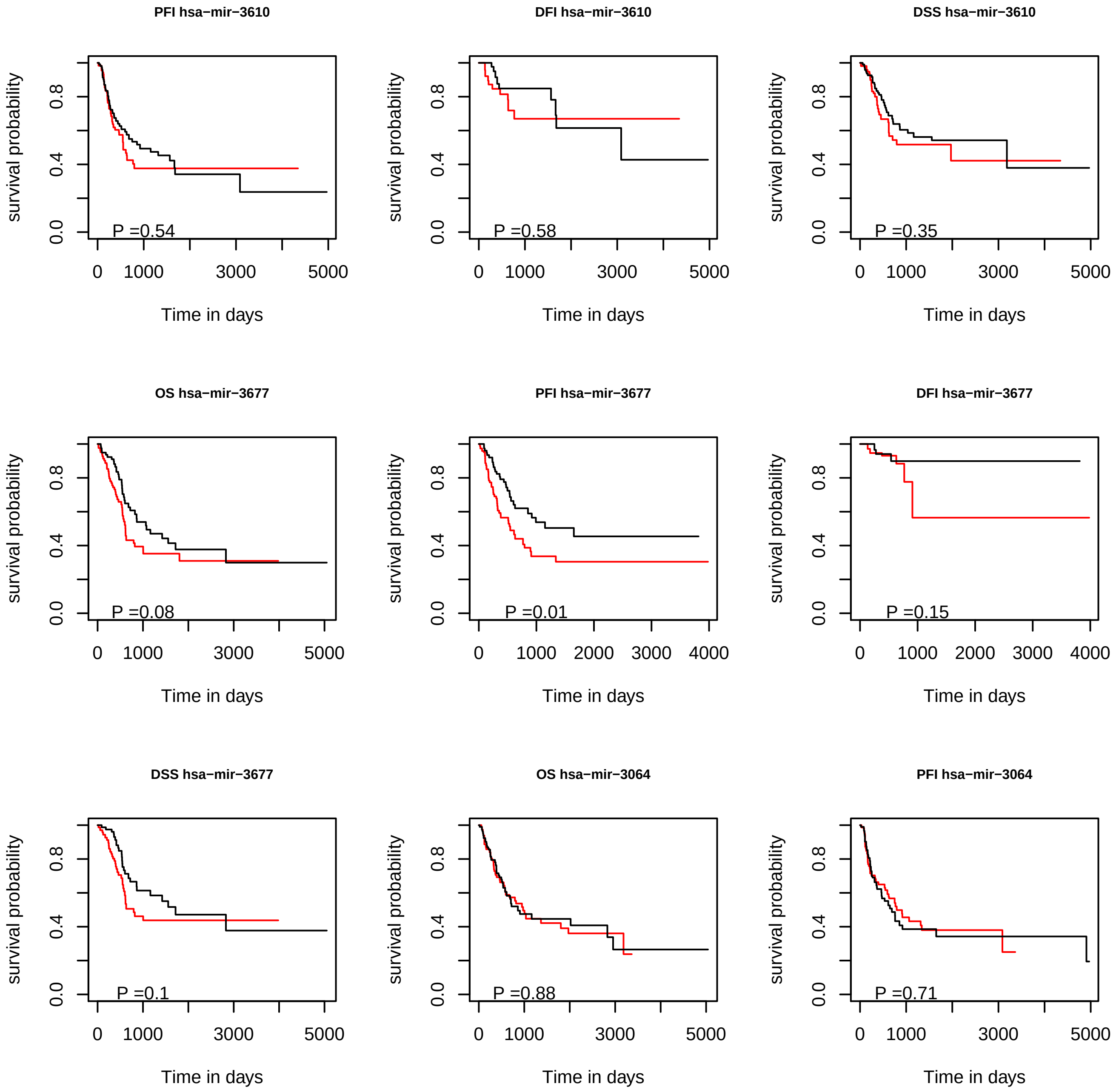
PFI hsa−mir−3610
P =0.54
DFI hsa−mir−3610
P =0.58
DSS hsa−mir−3610
P =0.35
OS hsa−mir−3677
P =0.08
PFI hsa−mir−3677
P =0.01
DFI hsa−mir−3677
P =0.15
DSS hsa−mir−3677
P =0.1
OS hsa−mir−3064
P =0.88
PFI hsa−mir−3064
P =0.71
survival probability
Time in days

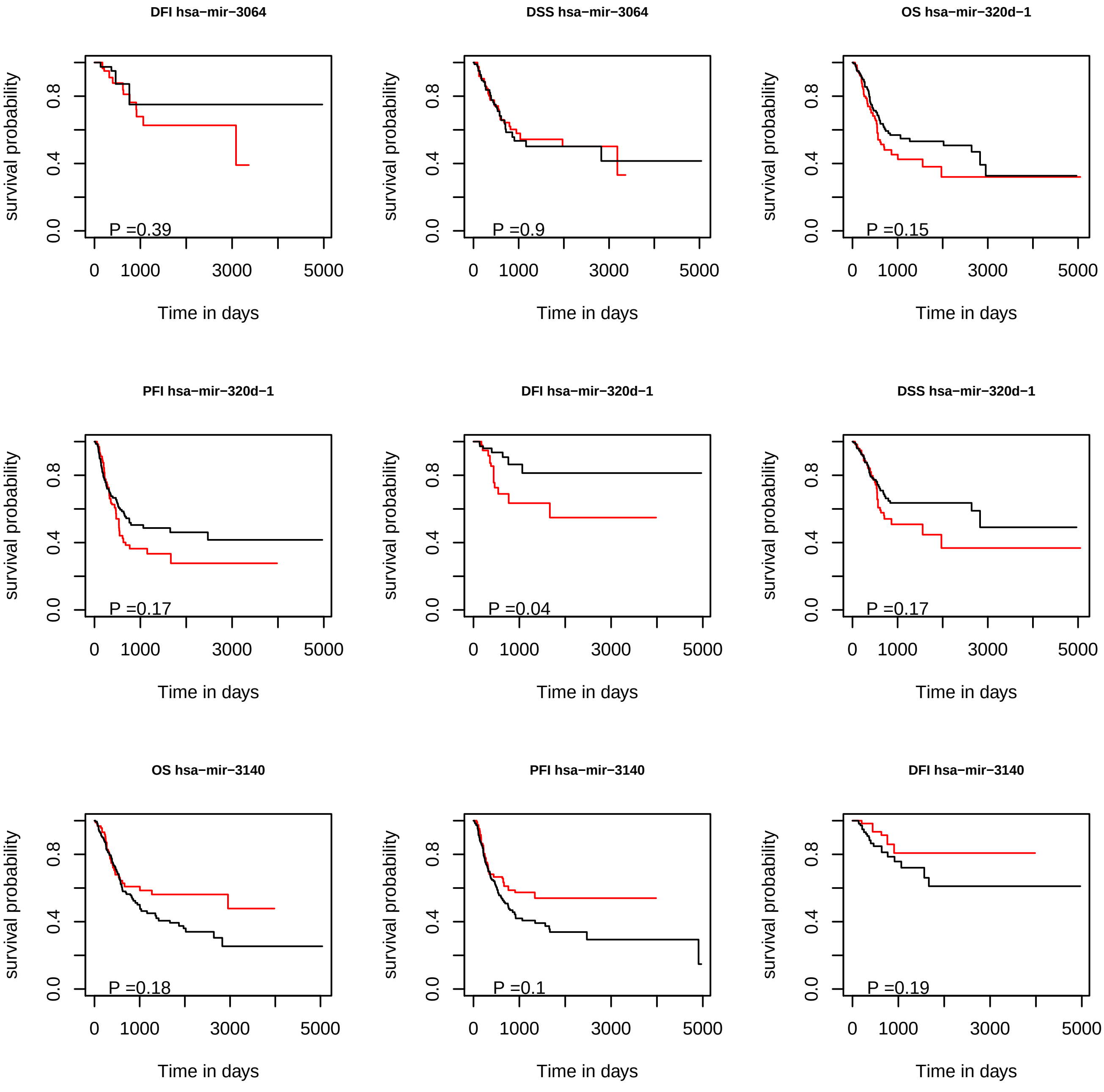
DFI hsa−mir−3064
P =0.39
DSS hsa−mir−3064
P =0.9
OS hsa−mir−320d−1
P =0.15
PFI hsa−mir−320d−1
P =0.17
DFI hsa−mir−320d−1
P =0.04
DSS hsa−mir−320d−1
P =0.17
OS hsa−mir−3140
P =0.18
PFI hsa−mir−3140
P =0.1
DFI hsa−mir−3140
P =0.19
survival probability
0.0
0.4
0.8
Time in days
0
1000
3000
5000

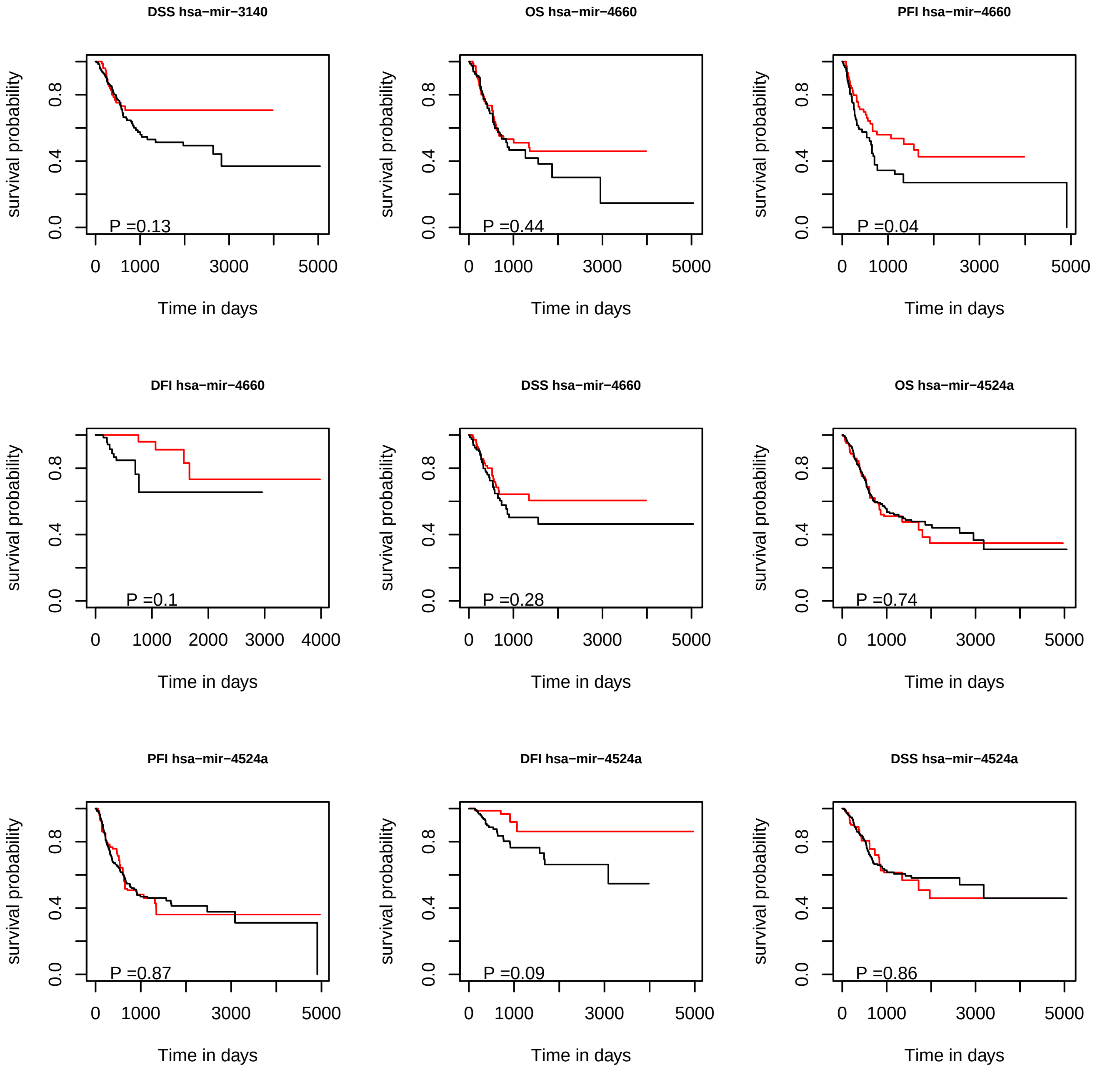
DSS hsa−mir−3140
survival probability
0.0 0.4 0.8
P =0.13
0 1000 3000 5000
Time in days
OS hsa−mir−4660
survival probability
0.0 0.4 0.8
P =0.44
0 1000 3000 5000
Time in days
PFI hsa−mir−4660
survival probability
0.0 0.4 0.8
P =0.04
0 1000 3000 5000
Time in days
DFI hsa−mir−4660
survival probability
0.0 0.4 0.8
P =0.1
0 1000 2000 3000 4000
Time in days
DSS hsa−mir−4660
survival probability
0.0 0.4 0.8
P =0.28
0 1000 3000 5000
Time in days
OS hsa−mir−4524a
survival probability
0.0 0.4 0.8
P =0.74
0 1000 3000 5000
Time in days
PFI hsa−mir−4524a
survival probability
0.0 0.4 0.8
P =0.87
0 1000 3000 5000
Time in days
DFI hsa−mir−4524a
survival probability
0.0 0.4 0.8
P =0.09
0 1000 3000 5000
Time in days
DSS hsa−mir−4524a
survival probability
0.0 0.4 0.8
P =0.86
0 1000 3000 5000
Time in days

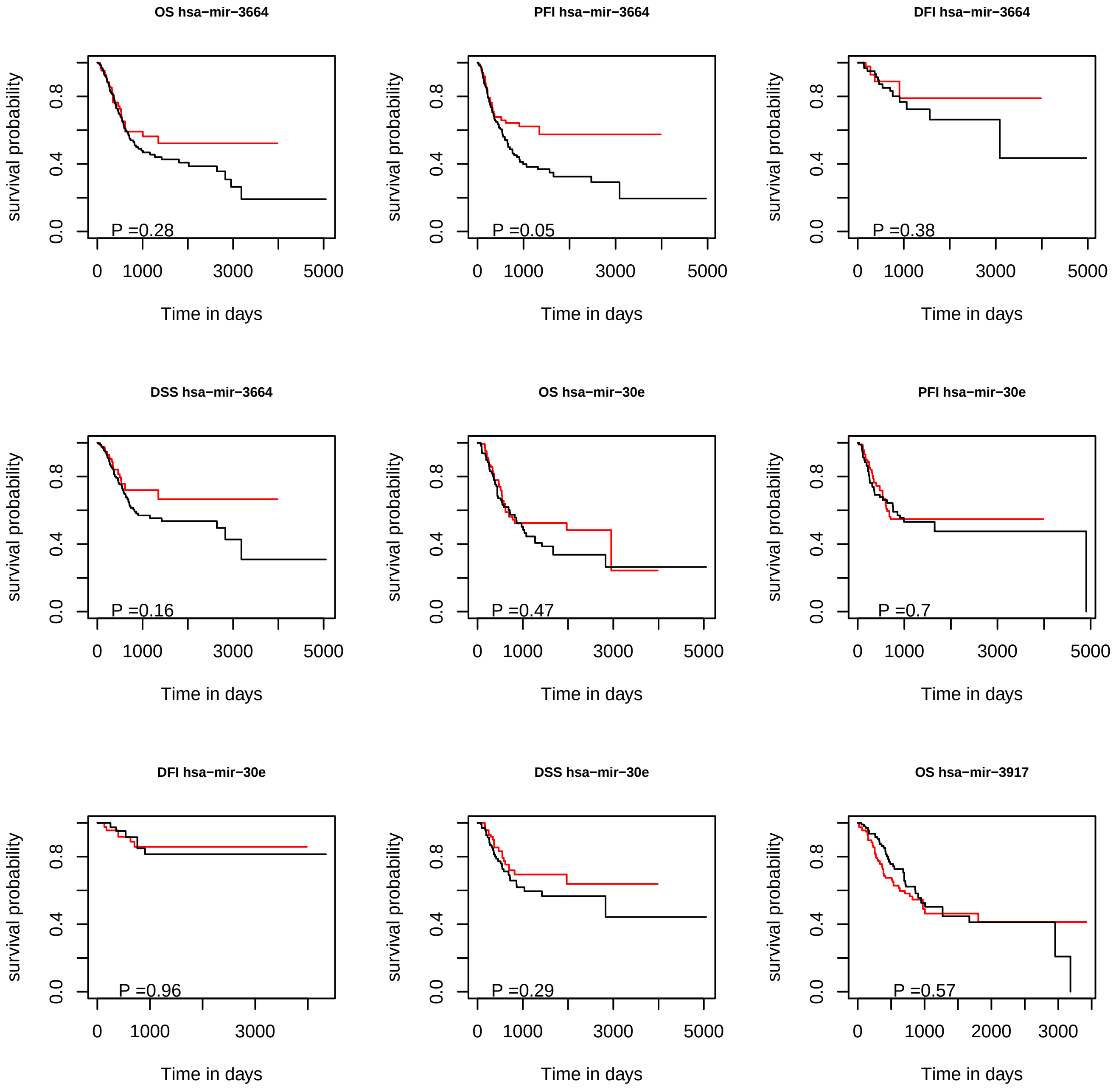

OS hsa−mir−3664
P =0.28
PFI hsa−mir−3664
P =0.05
DFI hsa−mir−3664
P =0.38
DSS hsa−mir−3664
P =0.16
OS hsa−mir−30e
P =0.47
PFI hsa−mir−30e
P =0.7
DFI hsa−mir−30e
P =0.96
DSS hsa−mir−30e
P =0.29
OS hsa−mir−3917
P =0.57
survival probability
Time in days
0.0
0.4
0.8
0
1000
2000
3000
5000

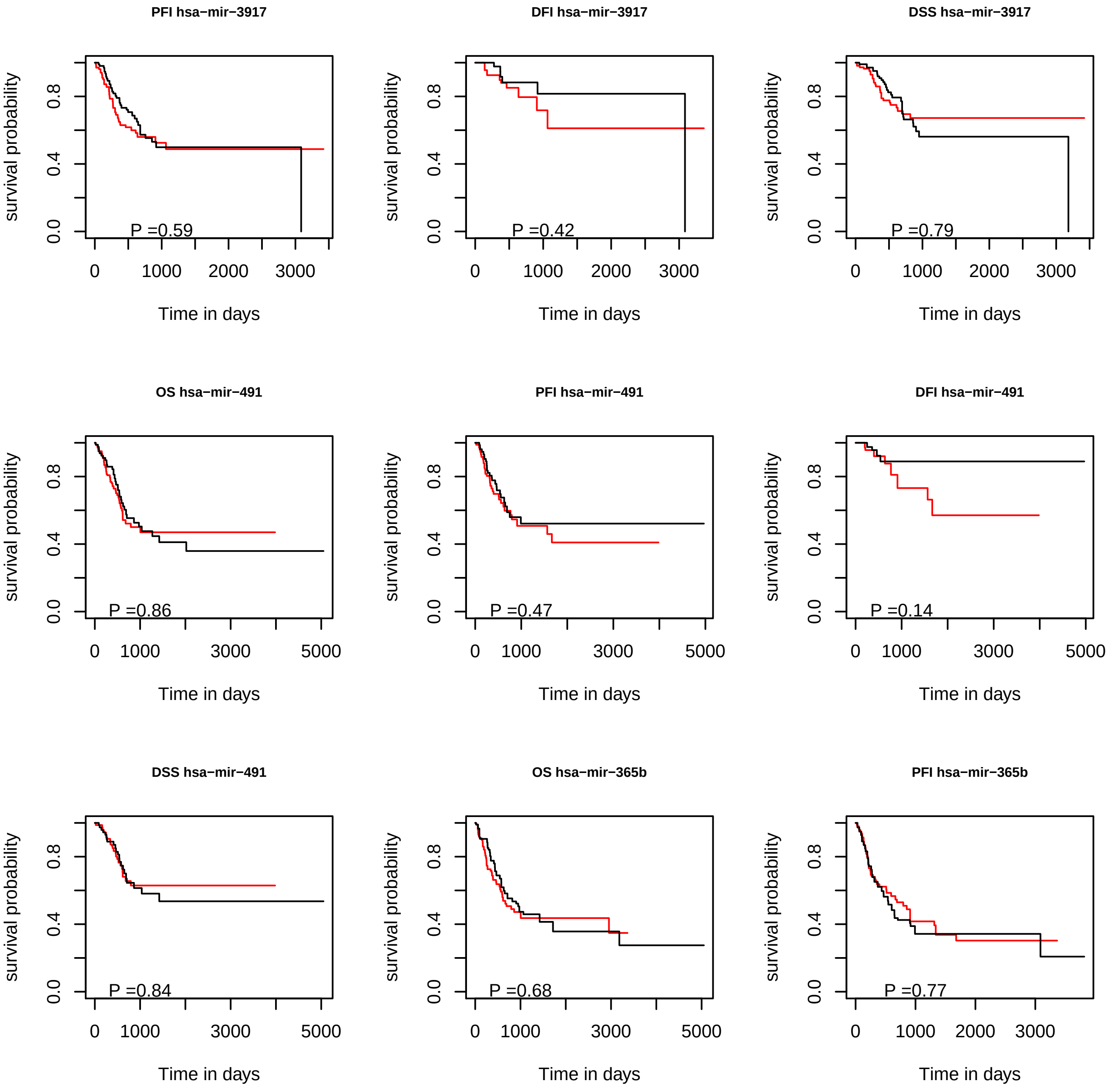
PFI hsa−mir−3917
P =0.59
DFI hsa−mir−3917
P =0.42
DSS hsa−mir−3917
P =0.79
OS hsa−mir−491
P =0.86
PFI hsa−mir−491
P =0.47
DFI hsa−mir−491
P =0.14
DSS hsa−mir−491
P =0.84
OS hsa−mir−365b
P =0.68
PFI hsa−mir−365b
P =0.77
survival probability
0.0
0.4
0.8
Time in days
0
1000
2000
3000
5000

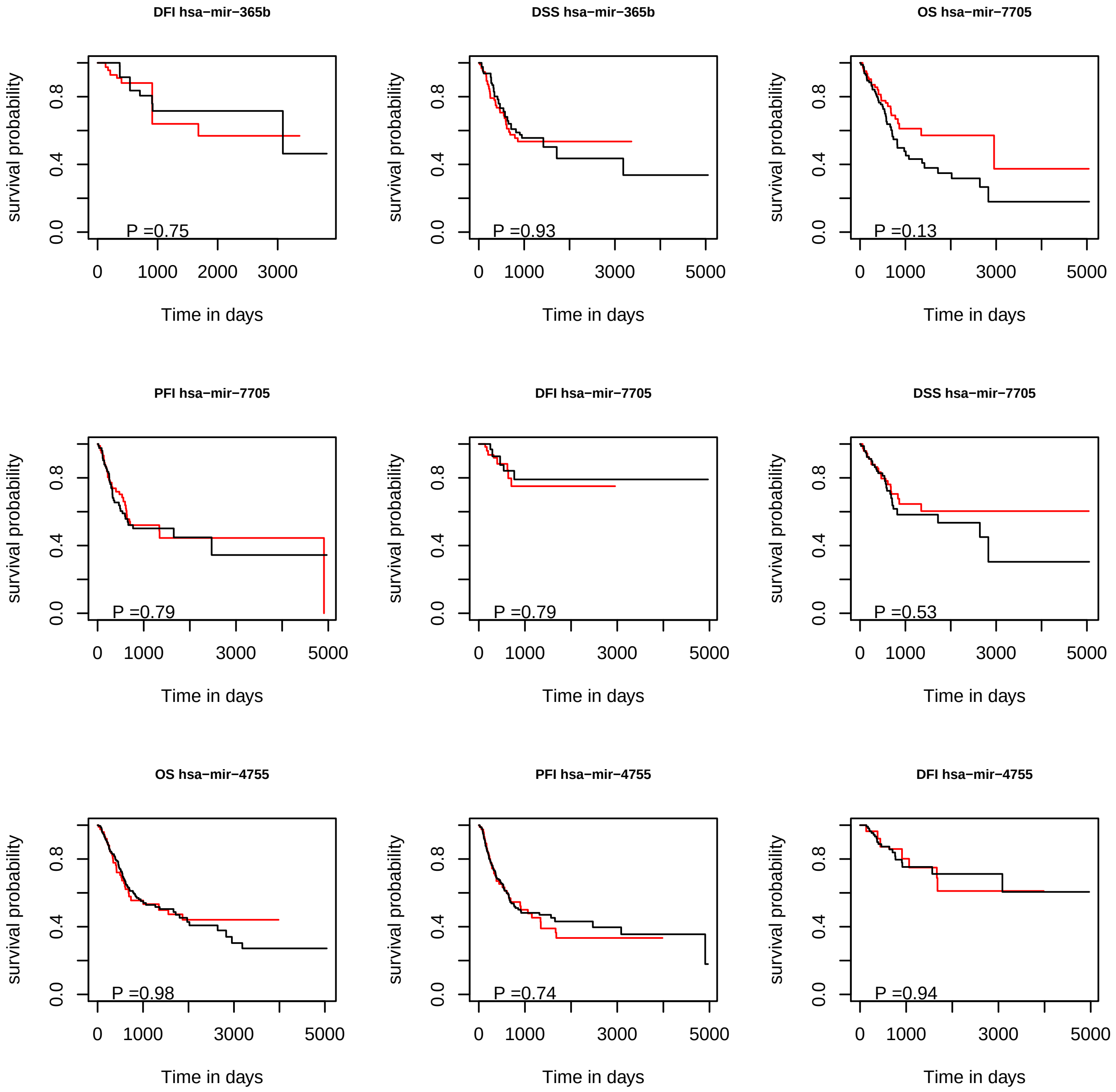
DFI hsa−mir−365b
P =0.75
DSS hsa−mir−365b
P =0.93
OS hsa−mir−7705
P =0.13
PFI hsa−mir−7705
P =0.79
DFI hsa−mir−7705
P =0.79
DSS hsa−mir−7705
P =0.53
OS hsa−mir−4755
P =0.98
PFI hsa−mir−4755
P =0.74
DFI hsa−mir−4755
P =0.94
survival probability
Time in days
0.0
0.4
0.8
0
1000
2000
3000
5000

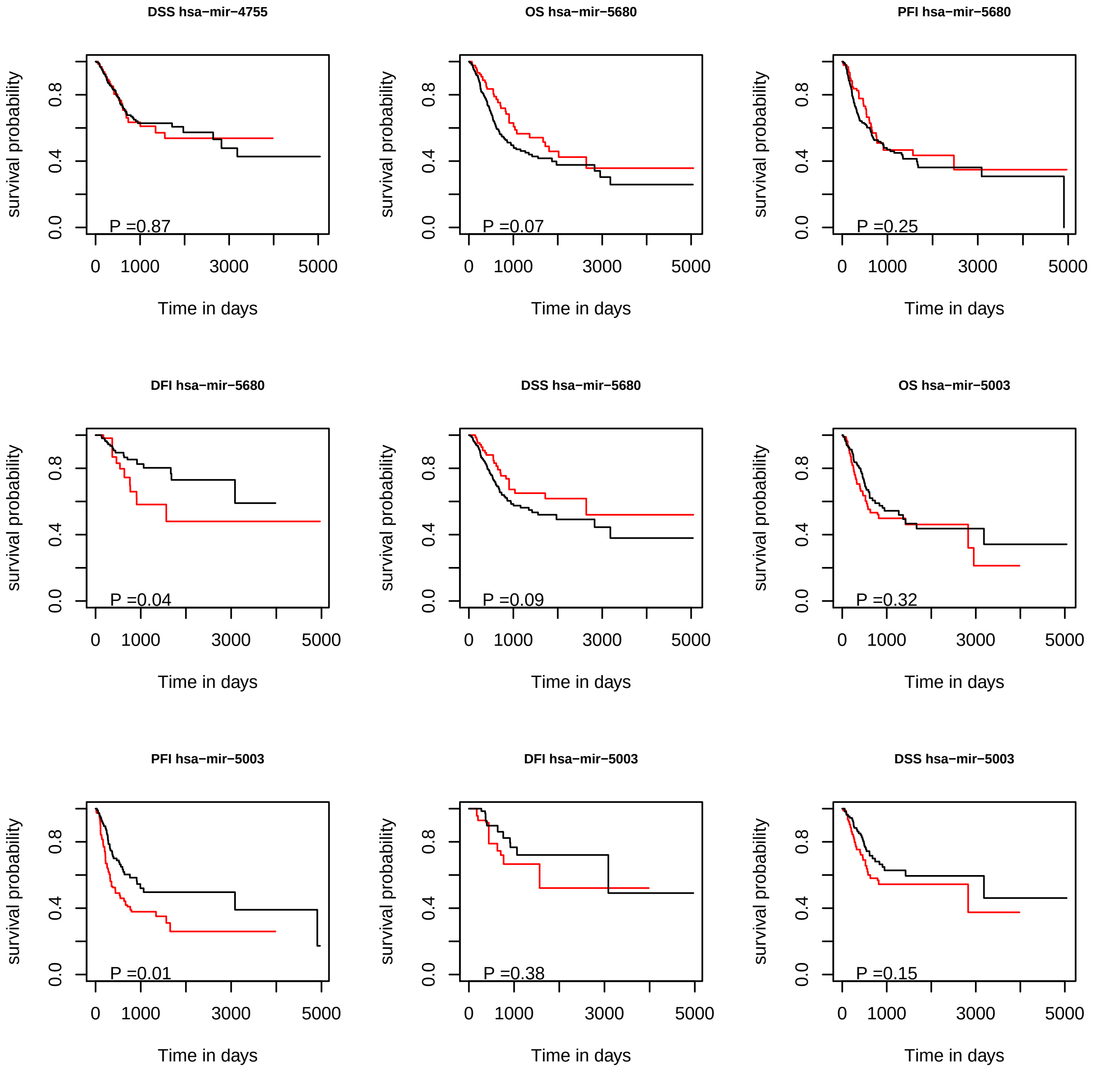
DSS hsa−mir−4755
P =0.87
OS hsa−mir−5680
P =0.07
PFI hsa−mir−5680
P =0.25
DFI hsa−mir−5680
P =0.04
DSS hsa−mir−5680
P =0.09
OS hsa−mir−5003
P =0.32
PFI hsa−mir−5003
P =0.01
DFI hsa−mir−5003
P =0.38
DSS hsa−mir−5003
P =0.15
survival probability
0.0
0.4
0.8
Time in days
0
1000
3000
5000

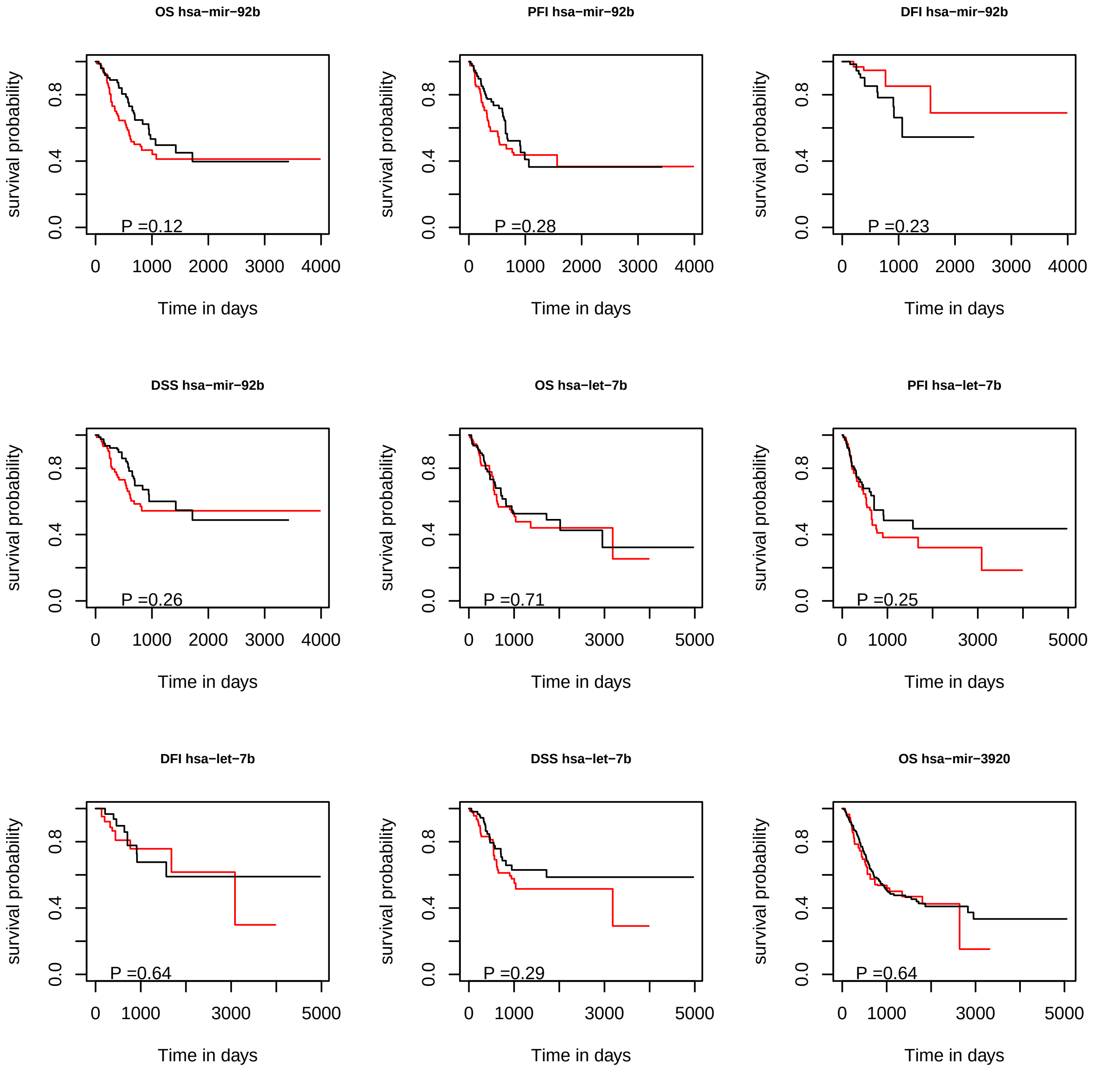

OS hsa−mir−92b
P =0.12
PFI hsa−mir−92b
P =0.28
DFI hsa−mir−92b
P =0.23
DSS hsa−mir−92b
P =0.26
OS hsa−let−7b
P =0.71
PFI hsa−let−7b
P =0.25
DFI hsa−let−7b
P =0.64
DSS hsa−let−7b
P =0.29
OS hsa−mir−3920
P =0.64
survival probability
Time in days

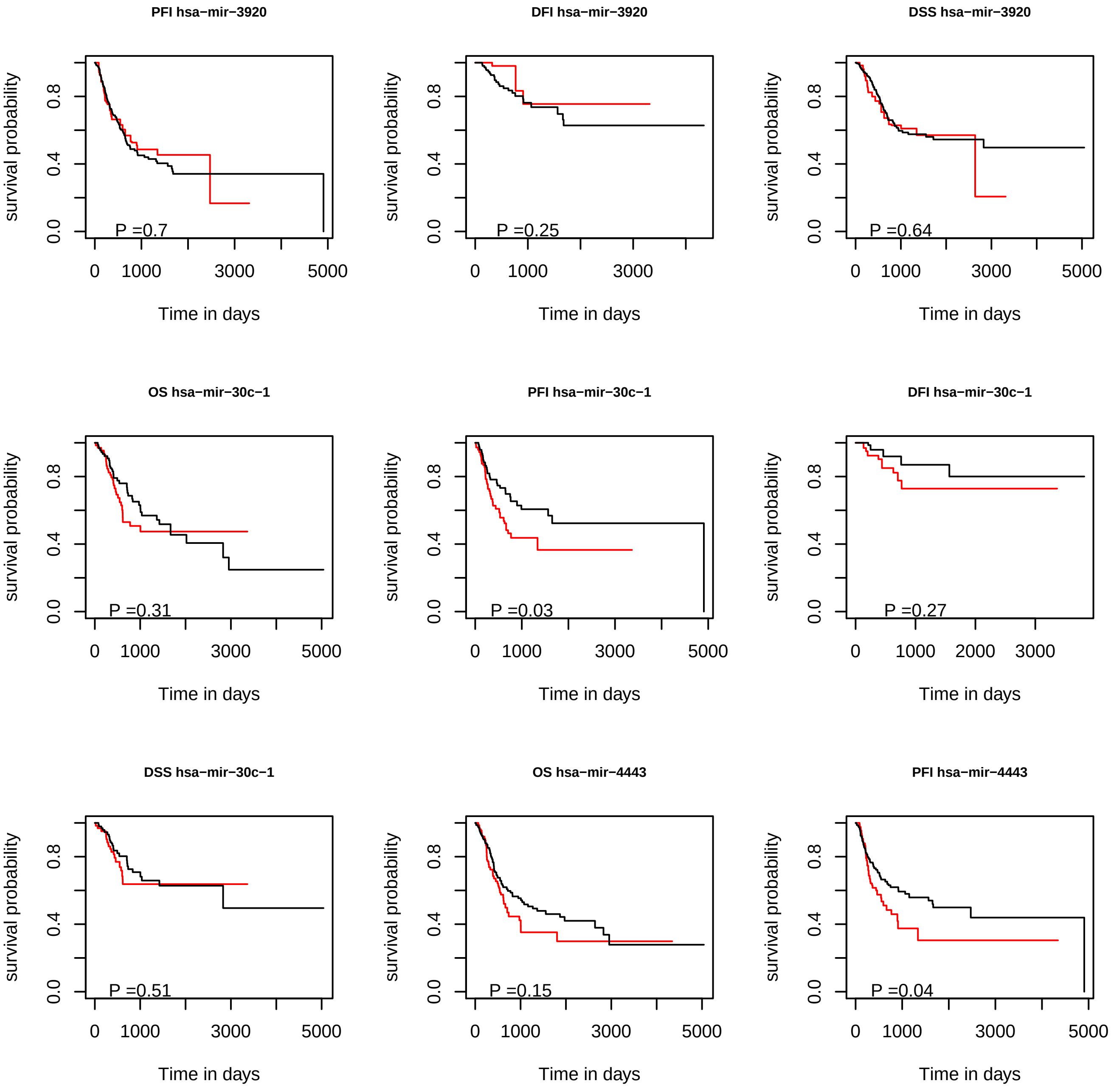

PFI hsa−mir−3920
P =0.7
DFI hsa−mir−3920
P =0.25
DSS hsa−mir−3920
P =0.64
OS hsa−mir−30c−1
P =0.31
PFI hsa−mir−30c−1
P =0.03
DFI hsa−mir−30c−1
P =0.27
DSS hsa−mir−30c−1
P =0.51
OS hsa−mir−4443
P =0.15
PFI hsa−mir−4443
P =0.04
survival probability
Time in days

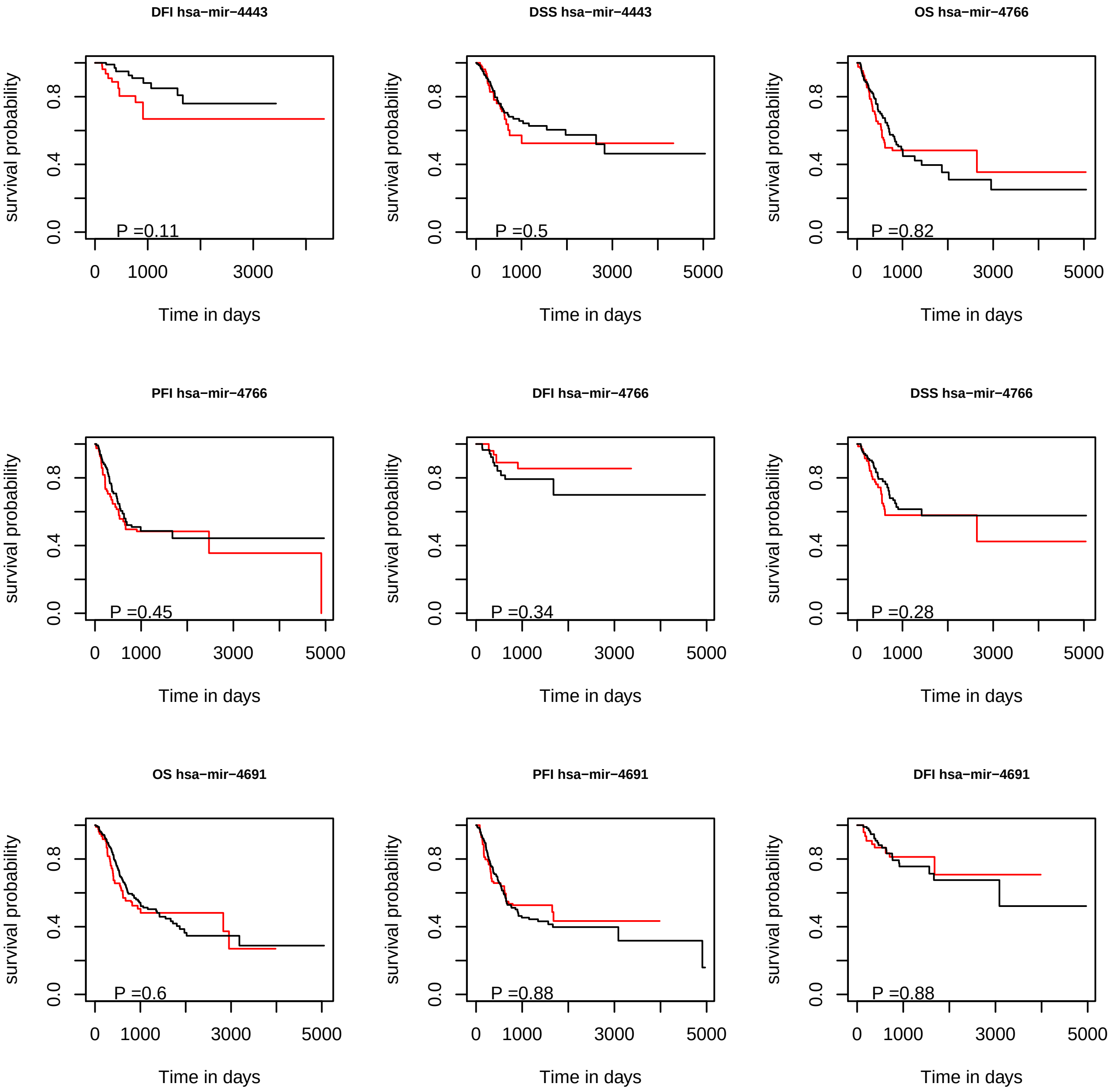
DFI hsa−mir−4443
survival probability
0.0
0.4
0.8
P =0.11
0
1000
3000
Time in days
DSS hsa−mir−4443
survival probability
0.0
0.4
0.8
P =0.5
0
1000
3000
5000
Time in days
OS hsa−mir−4766
survival probability
0.0
0.4
0.8
P =0.82
0
1000
3000
5000
Time in days
PFI hsa−mir−4766
survival probability
0.0
0.4
0.8
P =0.45
0
1000
3000
5000
Time in days
DFI hsa−mir−4766
survival probability
0.0
0.4
0.8
P =0.34
0
1000
3000
5000
Time in days
DSS hsa−mir−4766
survival probability
0.0
0.4
0.8
P =0.28
0
1000
3000
5000
Time in days
OS hsa−mir−4691
survival probability
0.0
0.4
0.8
P =0.6
0
1000
3000
5000
Time in days
PFI hsa−mir−4691
survival probability
0.0
0.4
0.8
P =0.88
0
1000
3000
5000
Time in days
DFI hsa−mir−4691
survival probability
0.0
0.4
0.8
P =0.88
0
1000
3000
5000
Time in days

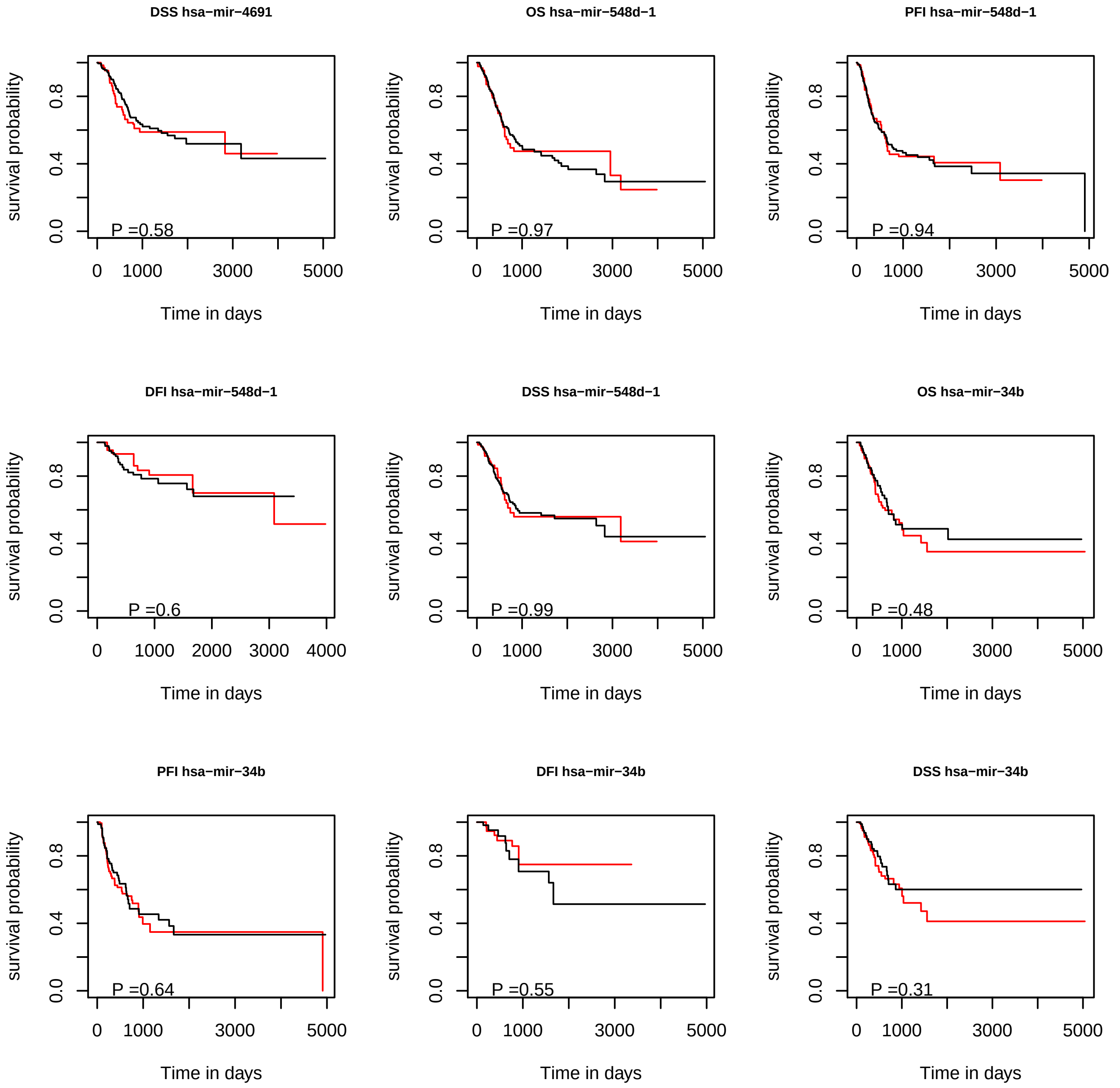
DSS hsa−mir−4691
P =0.58
OS hsa−mir−548d−1
P =0.97
PFI hsa−mir−548d−1
P =0.94
DFI hsa−mir−548d−1
P =0.6
DSS hsa−mir−548d−1
P =0.99
OS hsa−mir−34b
P =0.48
PFI hsa−mir−34b
P =0.64
DFI hsa−mir−34b
P =0.55
DSS hsa−mir−34b
P =0.31
survival probability
Time in days
0.0
0.4
0.8
0
1000
2000
3000
4000
5000

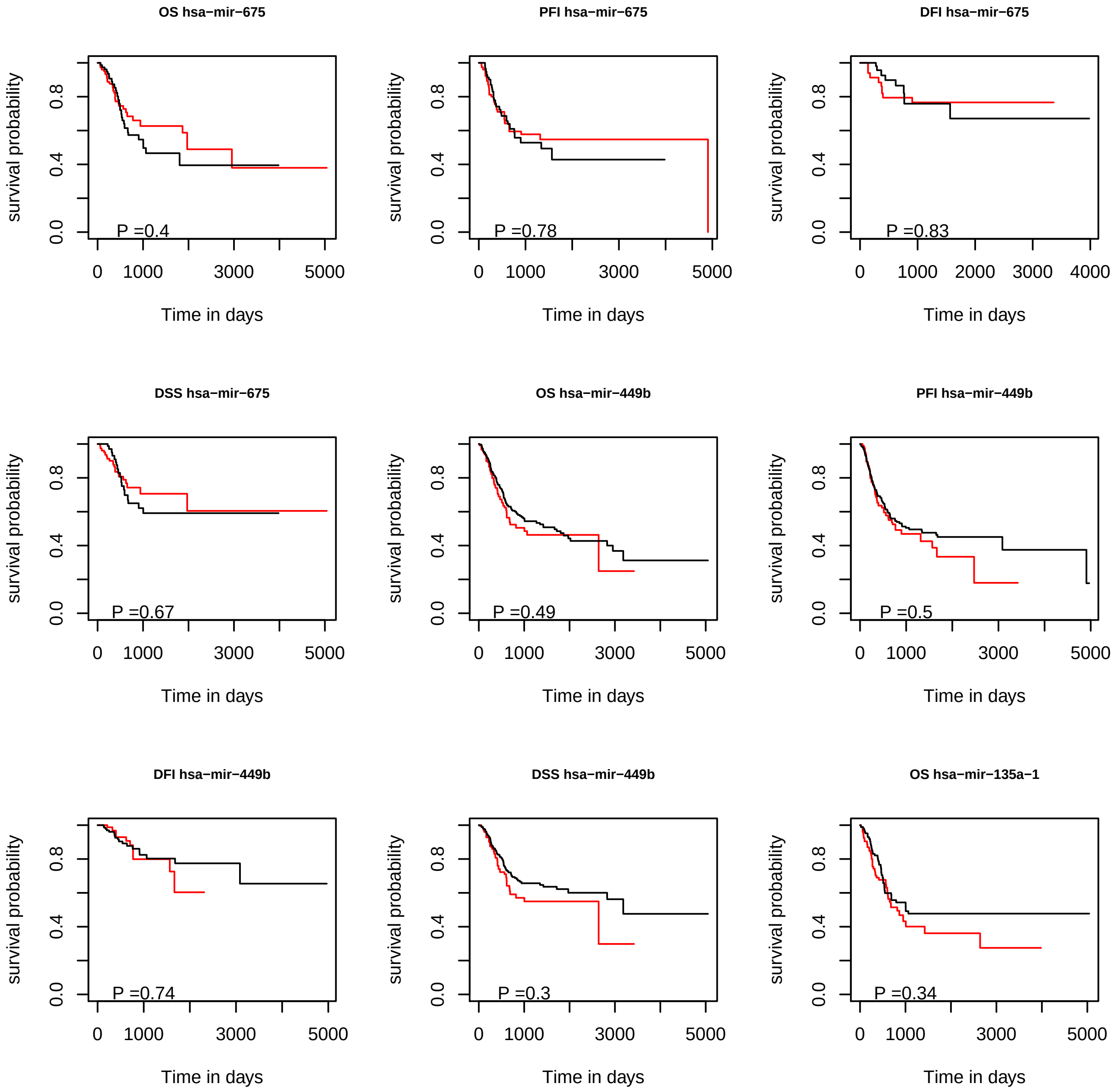
OS hsa−mir−675
P =0.4
PFI hsa−mir−675
P =0.78
DFI hsa−mir−675
P =0.83
DSS hsa−mir−675
P =0.67
OS hsa−mir−449b
P =0.49
PFI hsa−mir−449b
P =0.5
DFI hsa−mir−449b
P =0.74
DSS hsa−mir−449b
P =0.3
OS hsa−mir−135a−1
P =0.34
survival probability
Time in days

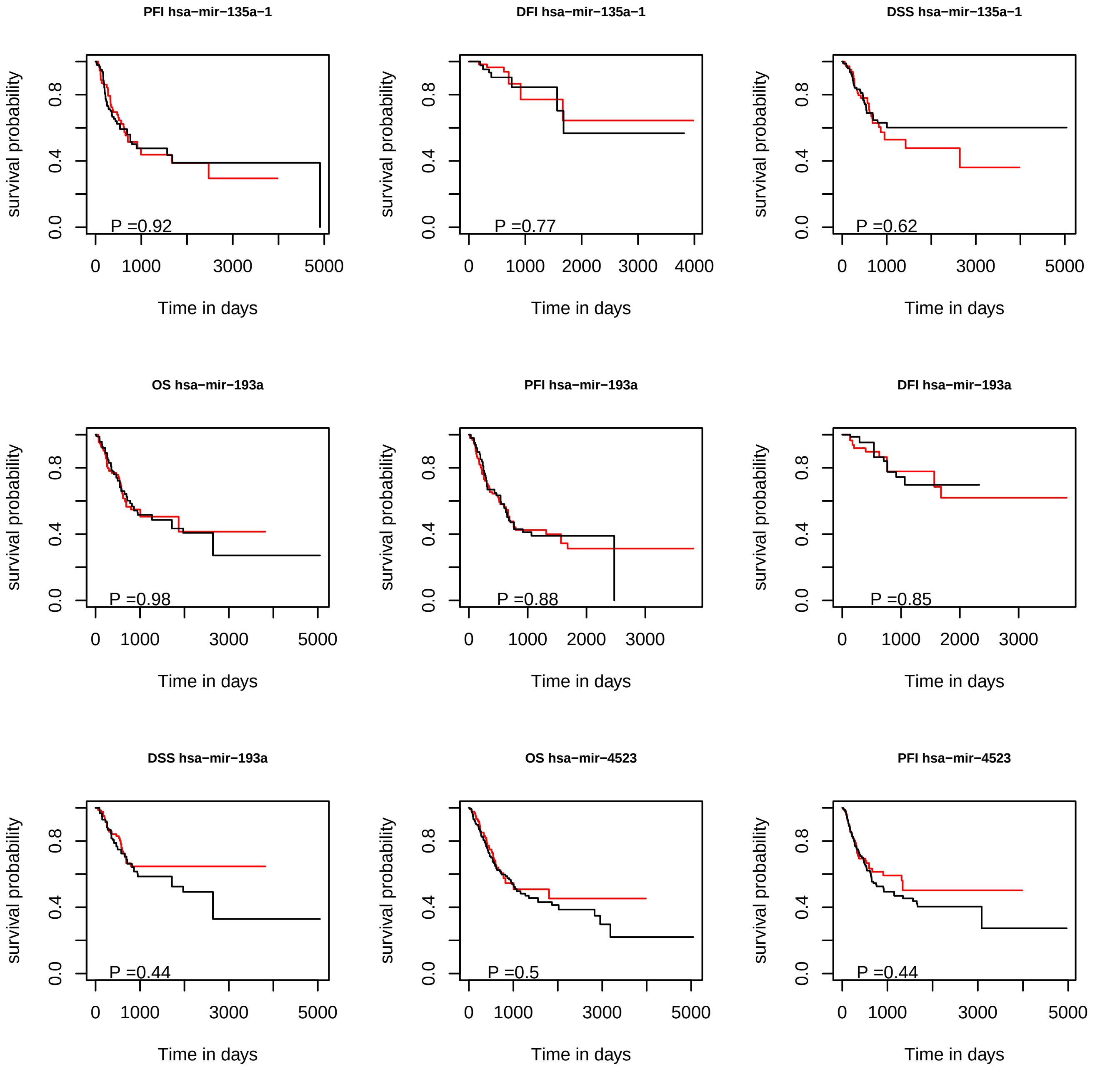
PFI hsa−mir−135a−1
P =0.92
DFI hsa−mir−135a−1
P =0.77
DSS hsa−mir−135a−1
P =0.62
OS hsa−mir−193a
P =0.98
PFI hsa−mir−193a
P =0.88
DFI hsa−mir−193a
P =0.85
DSS hsa−mir−193a
P =0.44
OS hsa−mir−4523
P =0.5
PFI hsa−mir−4523
P =0.44
survival probability
0.0
0.4
0.8
Time in days
0
1000
2000
3000
4000
5000

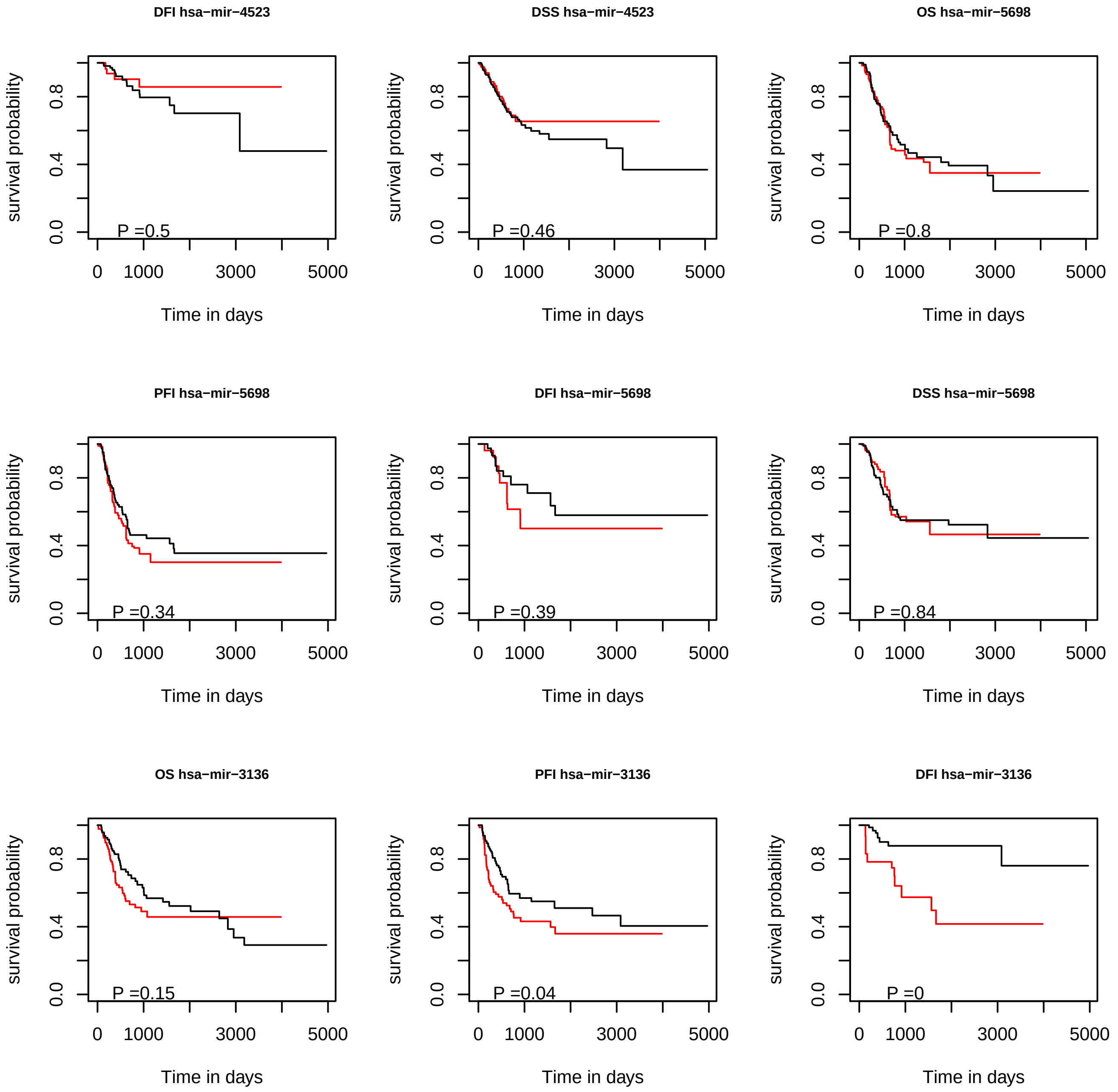
DFI hsa−mir−4523
P =0.5
DSS hsa−mir−4523
P =0.46
OS hsa−mir−5698
P =0.8
PFI hsa−mir−5698
P =0.34
DFI hsa−mir−5698
P =0.39
DSS hsa−mir−5698
P =0.84
OS hsa−mir−3136
P =0.15
PFI hsa−mir−3136
P =0.04
DFI hsa−mir−3136
P =0
survival probability
0.0
0.4
0.8
Time in days
0
1000
3000
5000

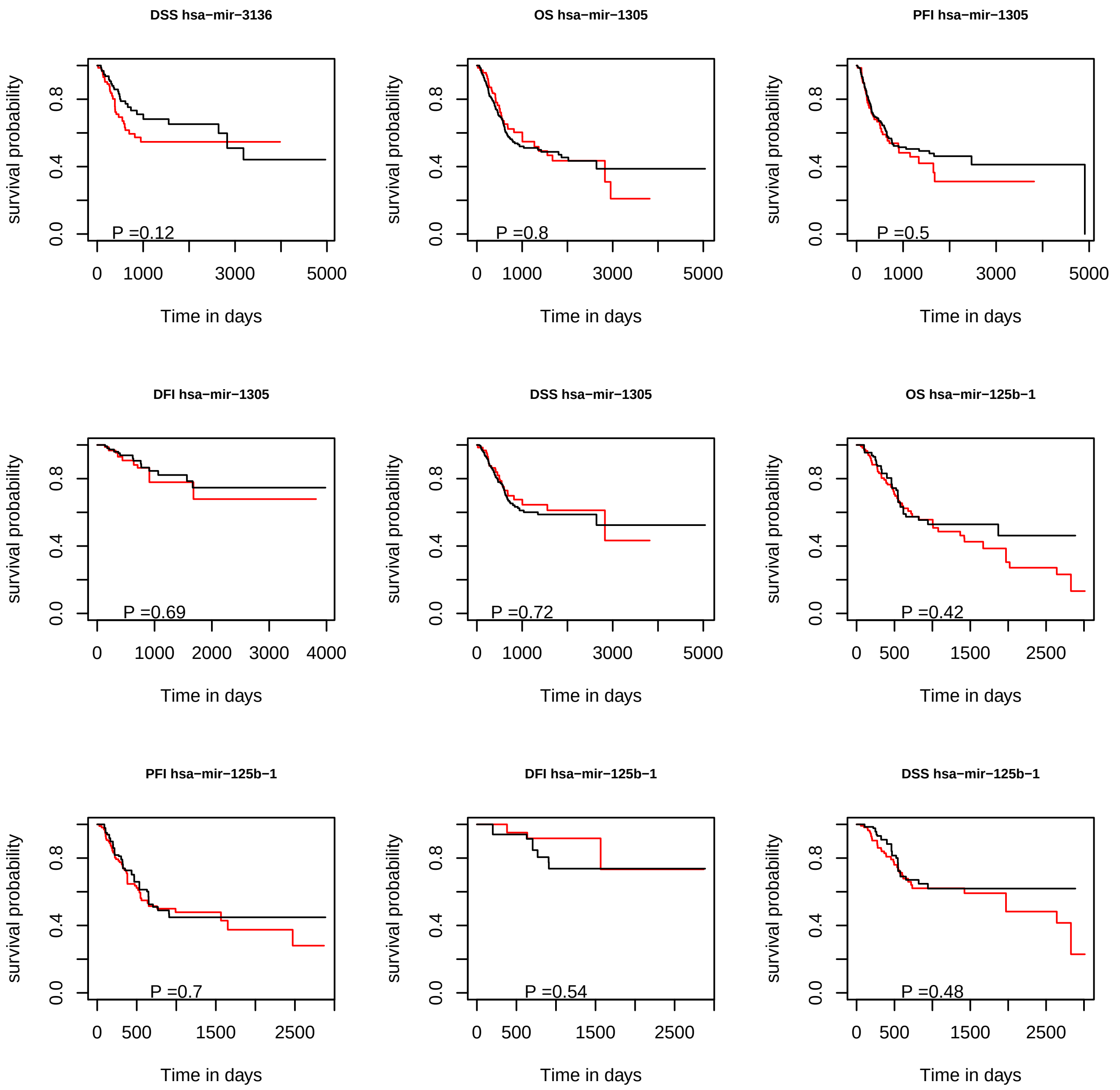

DSS hsa−mir−3136
P =0.12
OS hsa−mir−1305
P =0.8
PFI hsa−mir−1305
P =0.5
DFI hsa−mir−1305
P =0.69
DSS hsa−mir−1305
P =0.72
OS hsa−mir−125b−1
P =0.42
PFI hsa−mir−125b−1
P =0.7
DFI hsa−mir−125b−1
P =0.54
DSS hsa−mir−125b−1
P =0.48
survival probability
Time in days

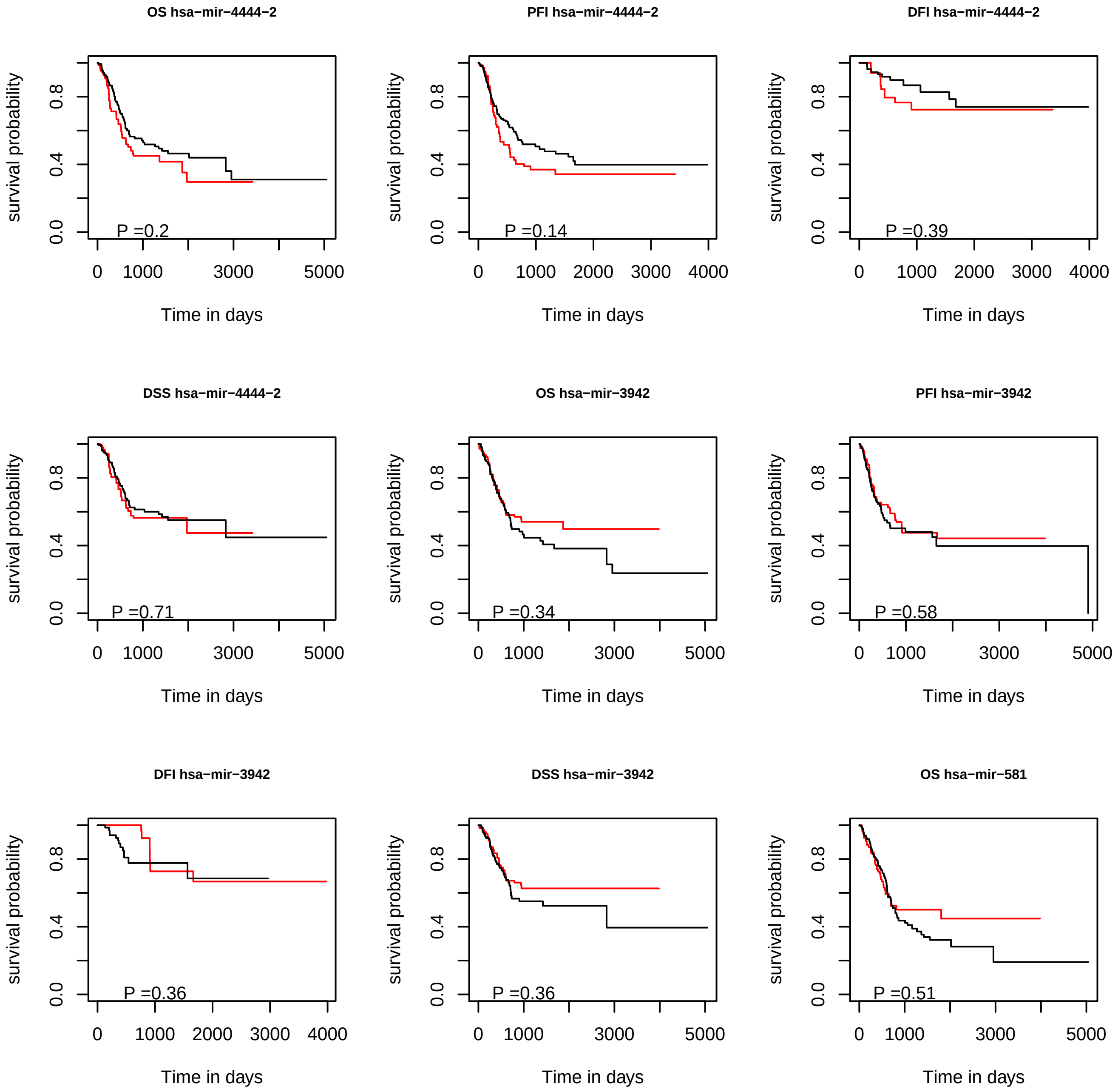
OS hsa−mir−4444−2
P =0.2
PFI hsa−mir−4444−2
P =0.14
DFI hsa−mir−4444−2
P =0.39
DSS hsa−mir−4444−2
P =0.71
OS hsa−mir−3942
P =0.34
PFI hsa−mir−3942
P =0.58
DFI hsa−mir−3942
P =0.36
DSS hsa−mir−3942
P =0.36
OS hsa−mir−581
P =0.51
survival probability
Time in days
0.0
0.4
0.8
0
1000
2000
3000
4000
5000

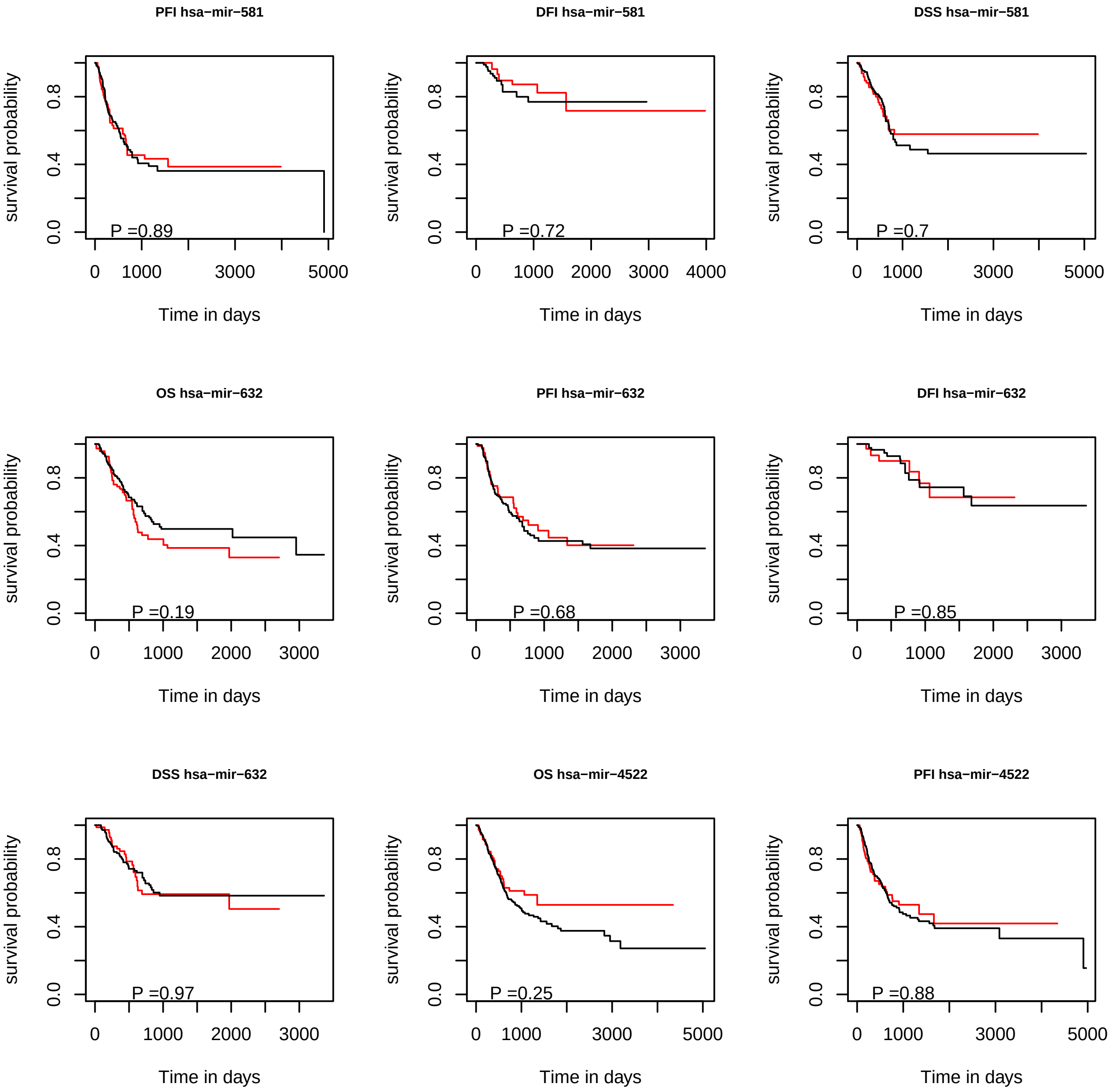
PFI hsa−mir−581
P =0.89
DFI hsa−mir−581
P =0.72
DSS hsa−mir−581
P =0.7
OS hsa−mir−632
P =0.19
PFI hsa−mir−632
P =0.68
DFI hsa−mir−632
P =0.85
DSS hsa−mir−632
P =0.97
OS hsa−mir−4522
P =0.25
PFI hsa−mir−4522
P =0.88
survival probability
Time in days

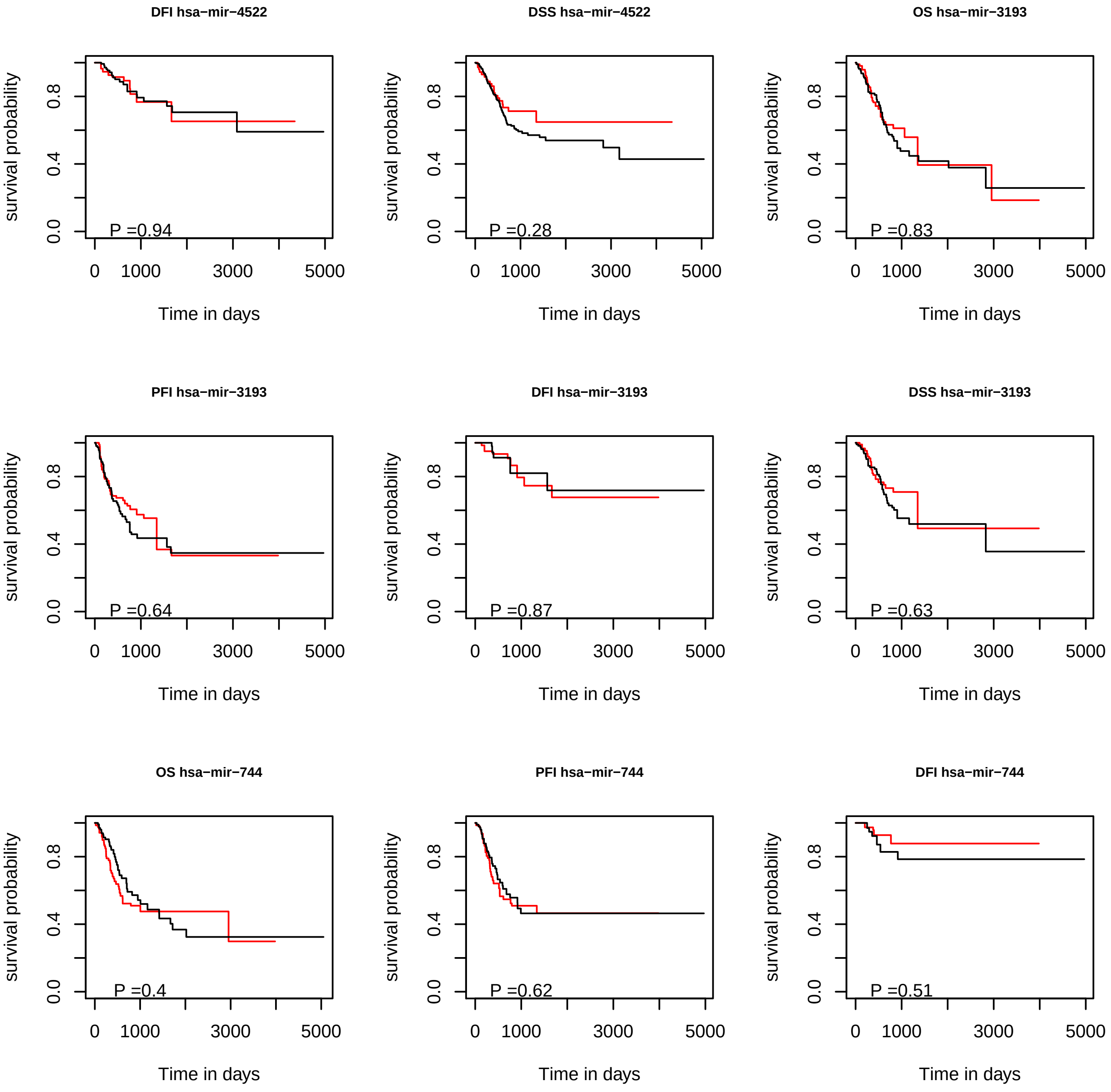
DFI hsa−mir−4522
P =0.94
DSS hsa−mir−4522
P =0.28
OS hsa−mir−3193
P =0.83
PFI hsa−mir−3193
P =0.64
DFI hsa−mir−3193
P =0.87
DSS hsa−mir−3193
P =0.63
OS hsa−mir−744
P =0.4
PFI hsa−mir−744
P =0.62
DFI hsa−mir−744
P =0.51
survival probability
0.0
0.4
0.8
Time in days
0
1000
3000
5000

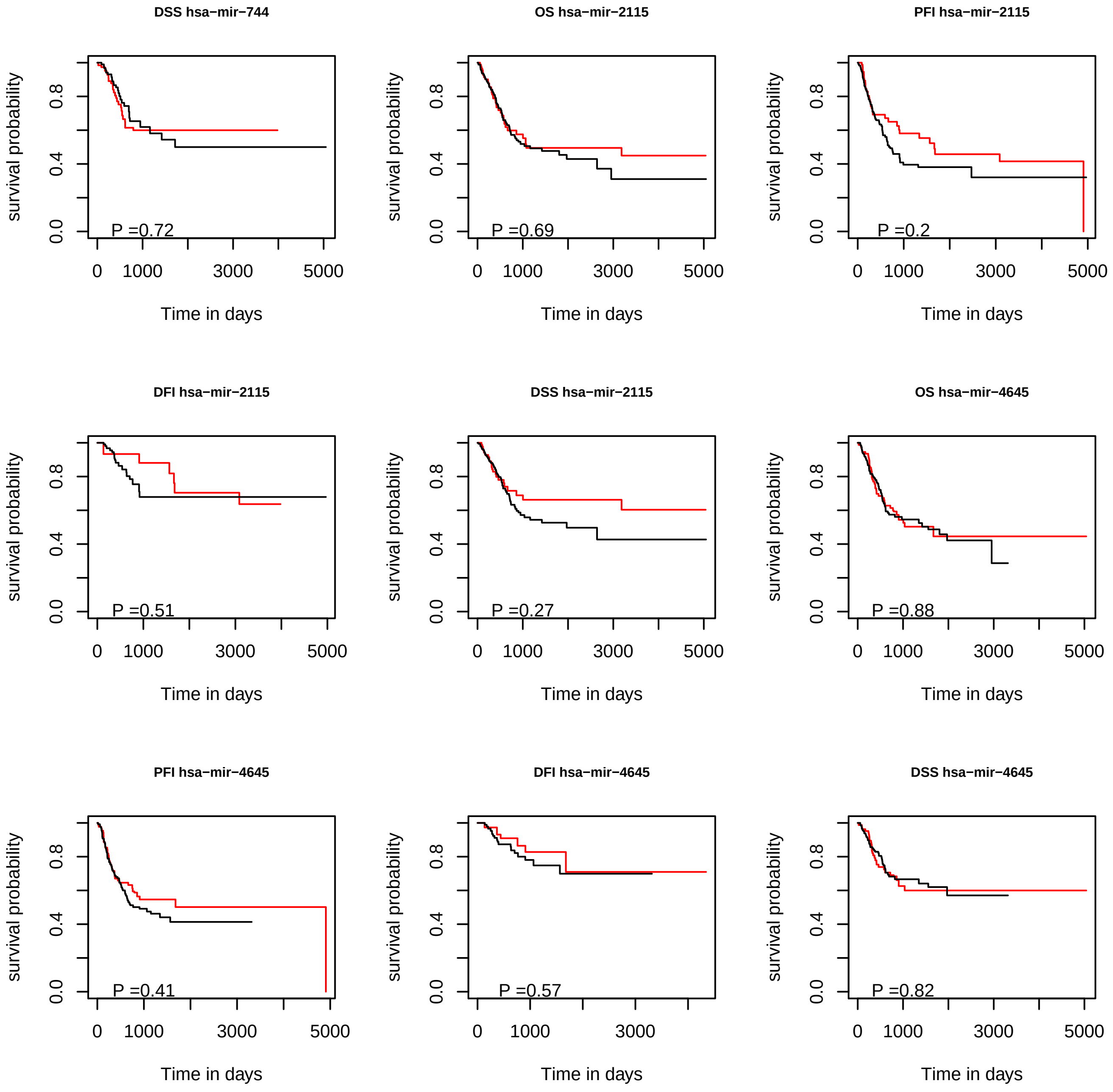

DSS hsa−mir−744
P =0.72
OS hsa−mir−2115
P =0.69
PFI hsa−mir−2115
P =0.2
DFI hsa−mir−2115
P =0.51
DSS hsa−mir−2115
P =0.27
OS hsa−mir−4645
P =0.88
PFI hsa−mir−4645
P =0.41
DFI hsa−mir−4645
P =0.57
DSS hsa−mir−4645
P =0.82
survival probability
Time in days
0.0
0.4
0.8
0
1000
3000
5000

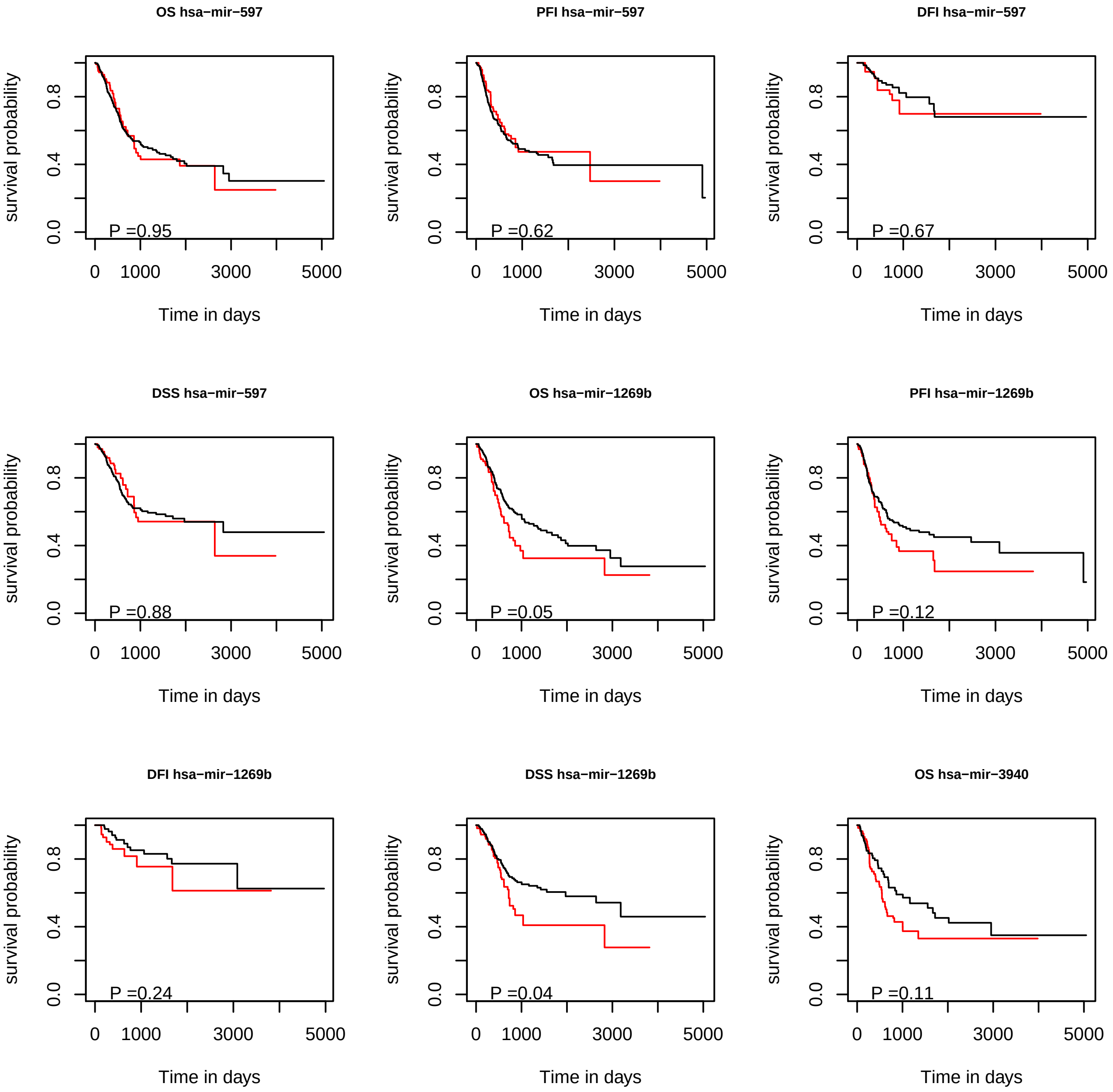
OS hsa−mir−597
P =0.95
PFI hsa−mir−597
P =0.62
DFI hsa−mir−597
P =0.67
DSS hsa−mir−597
P =0.88
OS hsa−mir−1269b
P =0.05
PFI hsa−mir−1269b
P =0.12
DFI hsa−mir−1269b
P =0.24
DSS hsa−mir−1269b
P =0.04
OS hsa−mir−3940
P =0.11
survival probability
0.0
0.4
0.8
Time in days
0
1000
3000
5000

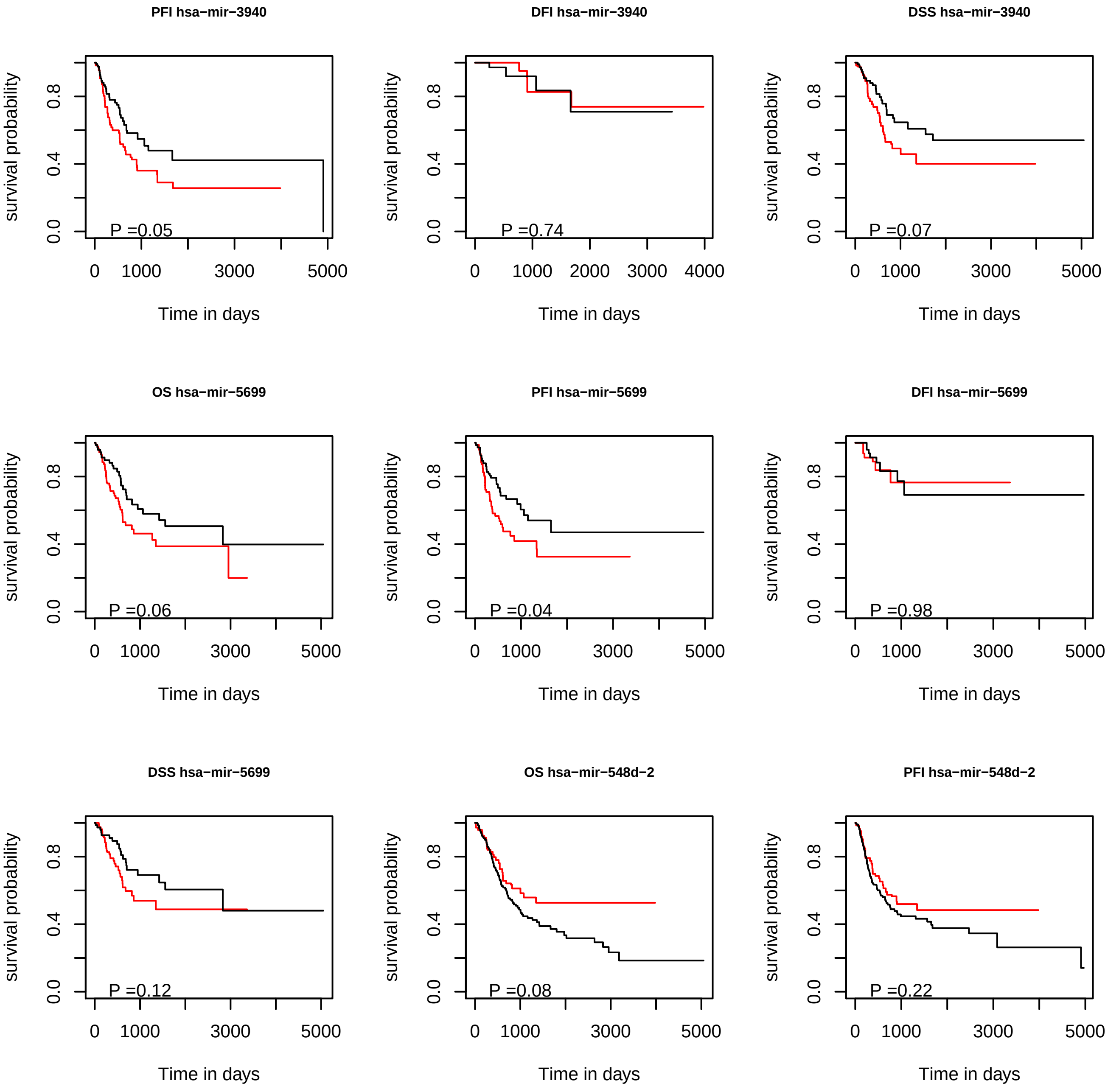
PFI hsa−mir−3940
survival probability
Time in days
P =0.05
DFI hsa−mir−3940
P =0.74
DSS hsa−mir−3940
P =0.07
OS hsa−mir−5699
P =0.06
PFI hsa−mir−5699
P =0.04
DFI hsa−mir−5699
P =0.98
DSS hsa−mir−5699
P =0.12
OS hsa−mir−548d−2
P =0.08
PFI hsa−mir−548d−2
P =0.22
0.0
0.4
0.8
0
1000
2000
3000
4000
5000

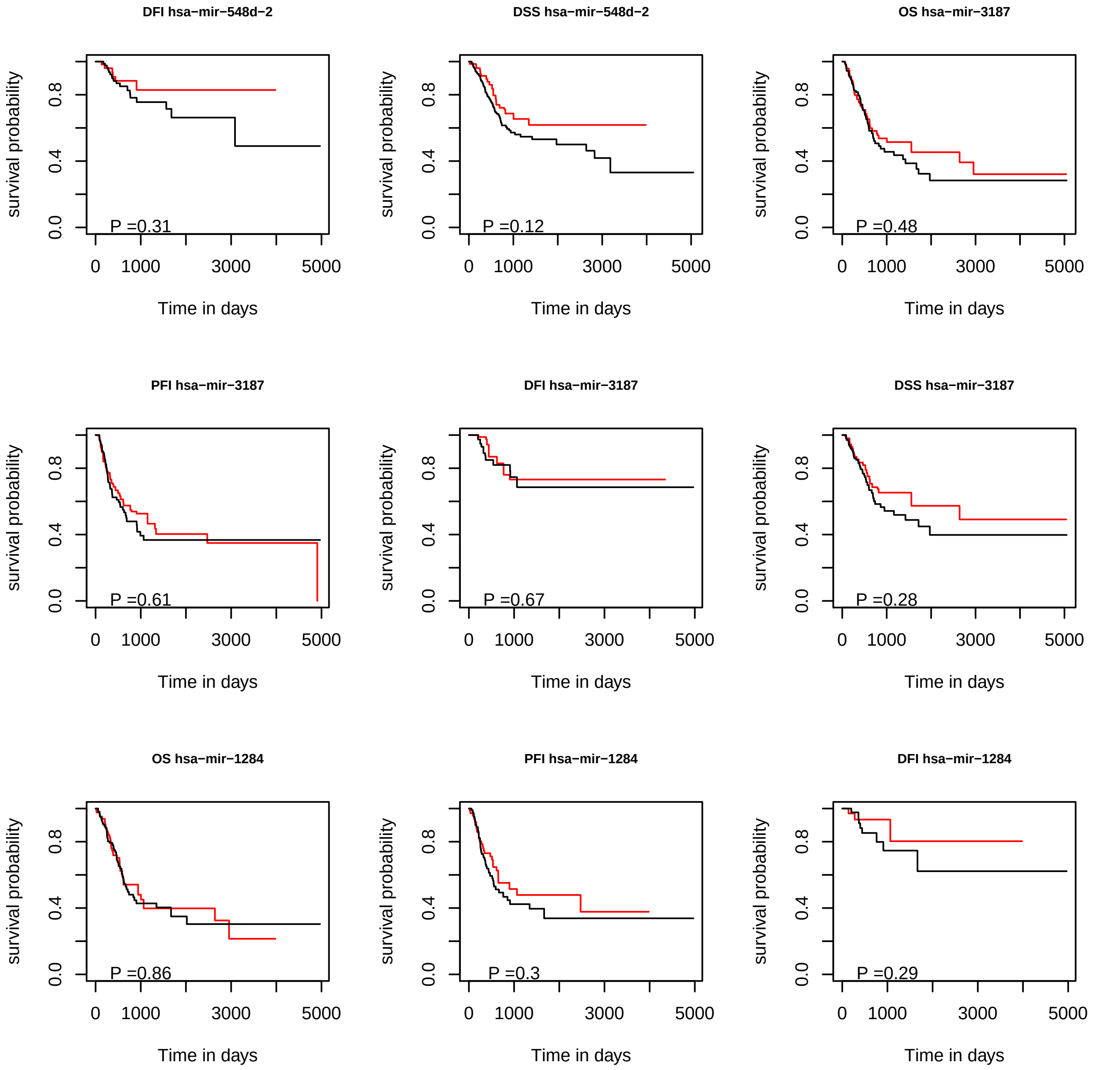
DFI hsa−mir−548d−2
P =0.31
DSS hsa−mir−548d−2
P =0.12
OS hsa−mir−3187
P =0.48
PFI hsa−mir−3187
P =0.61
DFI hsa−mir−3187
P =0.67
DSS hsa−mir−3187
P =0.28
OS hsa−mir−1284
P =0.86
PFI hsa−mir−1284
P =0.3
DFI hsa−mir−1284
P =0.29
survival probability
Time in days
0.0
0.4
0.8
0
1000
3000
5000

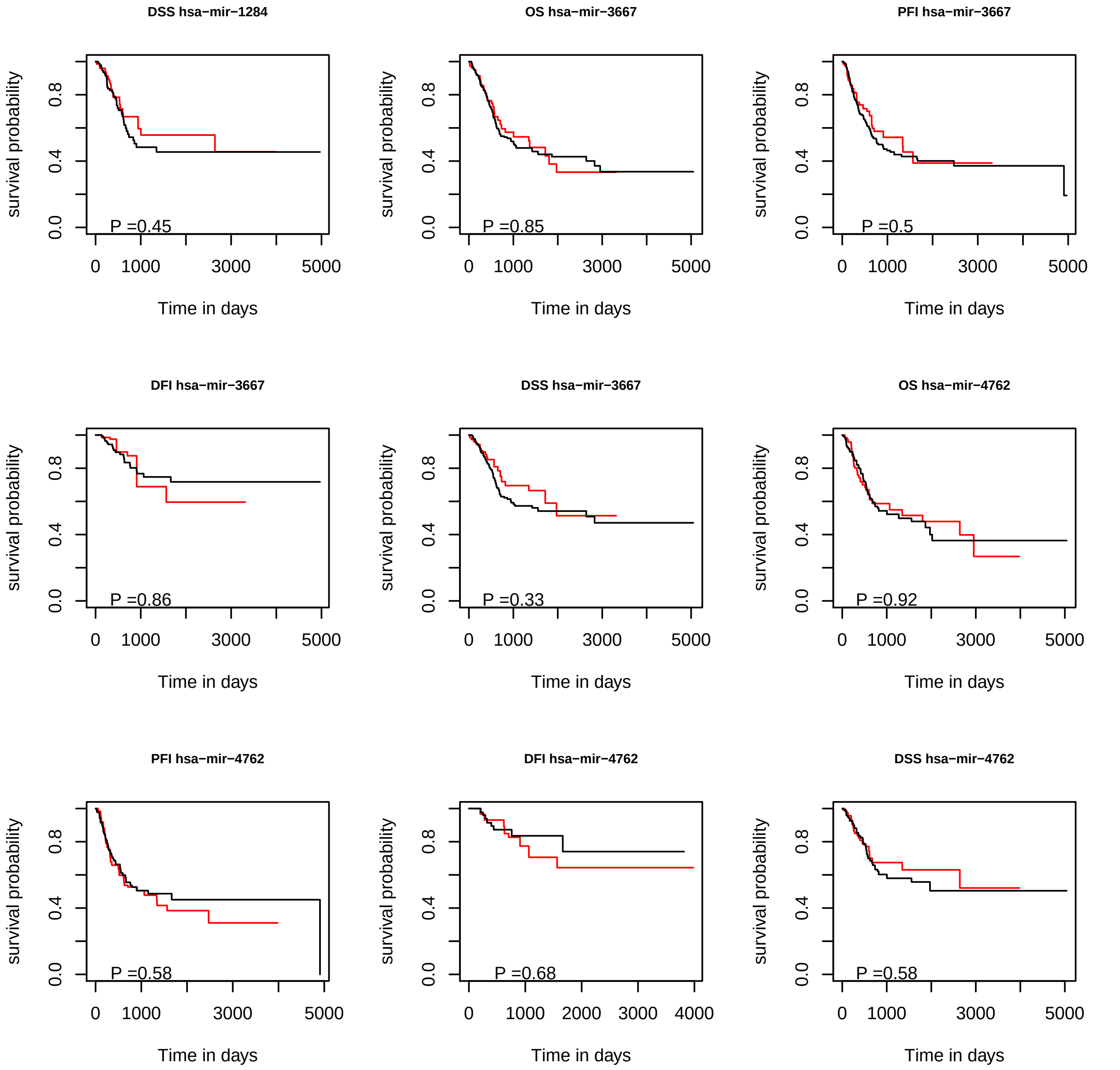
DSS hsa−mir−1284
P =0.45
OS hsa−mir−3667
P =0.85
PFI hsa−mir−3667
P =0.5
DFI hsa−mir−3667
P =0.86
DSS hsa−mir−3667
P =0.33
OS hsa−mir−4762
P =0.92
PFI hsa−mir−4762
P =0.58
DFI hsa−mir−4762
P =0.68
DSS hsa−mir−4762
P =0.58
survival probability
Time in days
0.0
0.4
0.8
0
1000
2000
3000
4000
5000

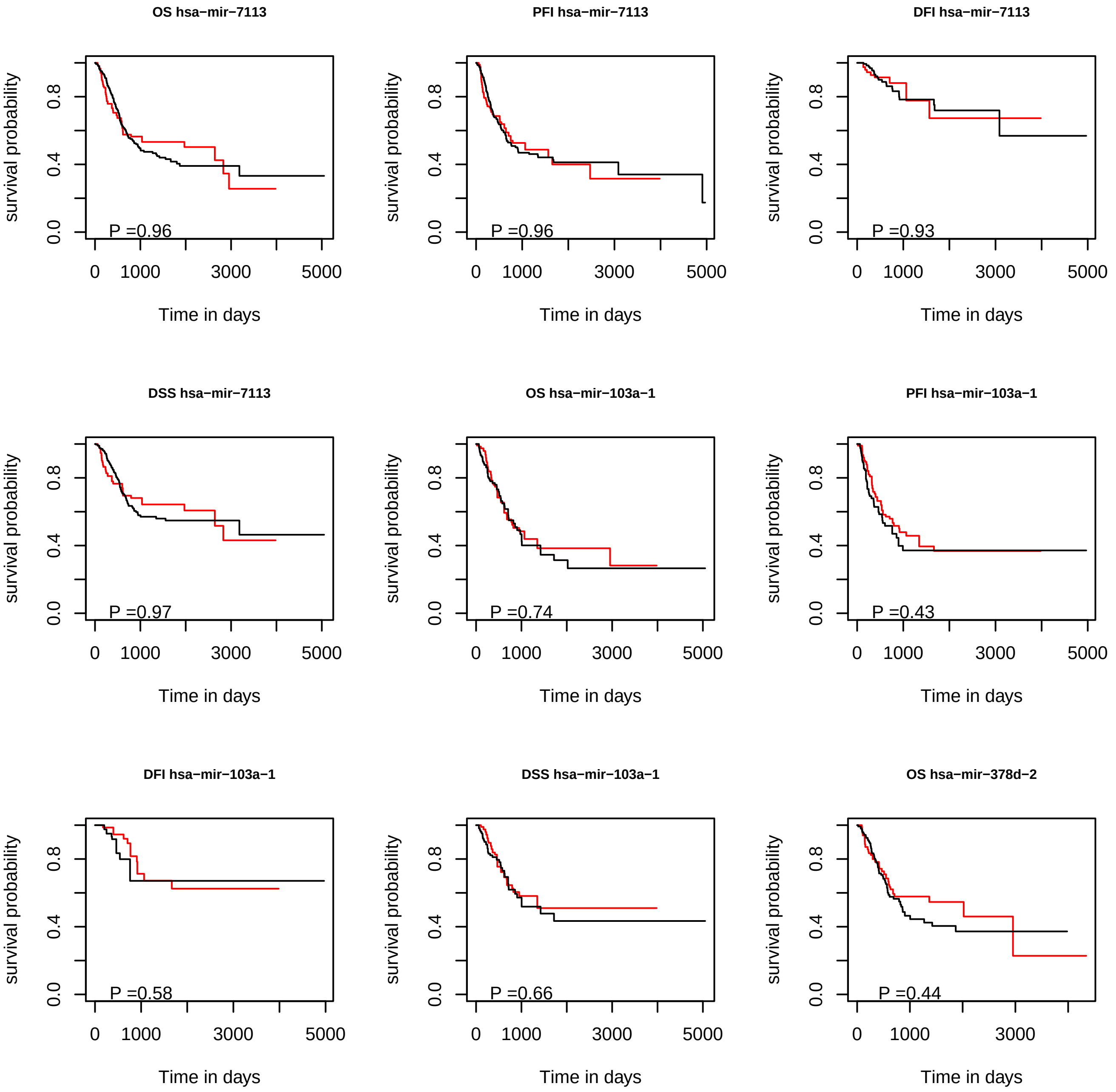
OS hsa−mir−7113
P =0.96
PFI hsa−mir−7113
P =0.96
DFI hsa−mir−7113
P =0.93
DSS hsa−mir−7113
P =0.97
OS hsa−mir−103a−1
P =0.74
PFI hsa−mir−103a−1
P =0.43
DFI hsa−mir−103a−1
P =0.58
DSS hsa−mir−103a−1
P =0.66
OS hsa−mir−378d−2
P =0.44
survival probability
Time in days
0.0
0.4
0.8
0
1000
3000
5000

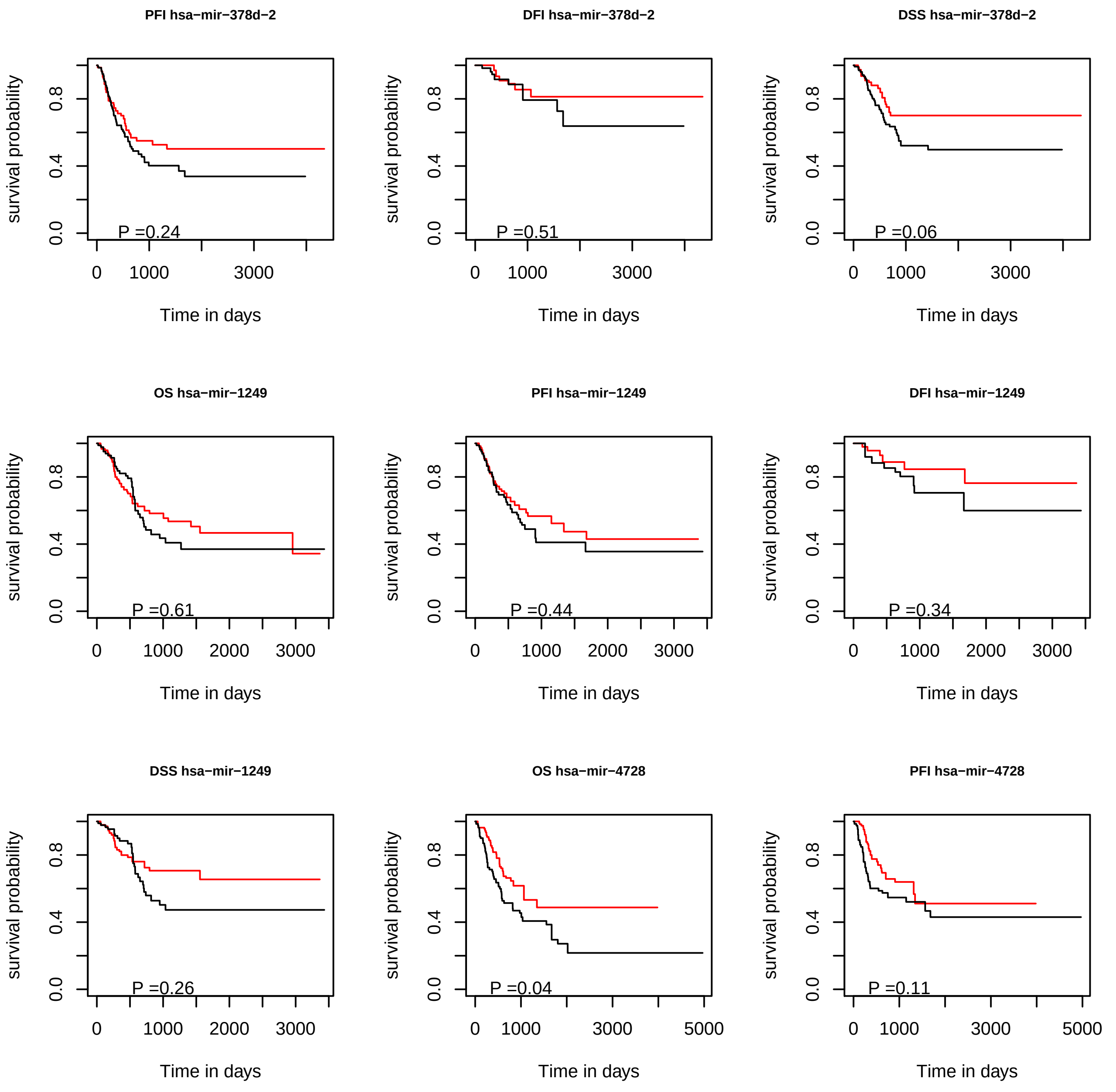

PFI hsa−mir−378d−2
survival probability
0.0
0.4
0.8
P =0.24
0
1000
3000
Time in days
DFI hsa−mir−378d−2
survival probability
0.0
0.4
0.8
P =0.51
0
1000
3000
Time in days
DSS hsa−mir−378d−2
survival probability
0.0
0.4
0.8
P =0.06
0
1000
3000
Time in days
OS hsa−mir−1249
survival probability
0.0
0.4
0.8
P =0.61
0
1000
2000
3000
Time in days
PFI hsa−mir−1249
survival probability
0.0
0.4
0.8
P =0.44
0
1000
2000
3000
Time in days
DFI hsa−mir−1249
survival probability
0.0
0.4
0.8
P =0.34
0
1000
2000
3000
Time in days
DSS hsa−mir−1249
survival probability
0.0
0.4
0.8
P =0.26
0
1000
2000
3000
Time in days
OS hsa−mir−4728
survival probability
0.0
0.4
0.8
P =0.04
0
1000
3000
5000
Time in days
PFI hsa−mir−4728
survival probability
0.0
0.4
0.8
P =0.11
0
1000
3000
5000
Time in days

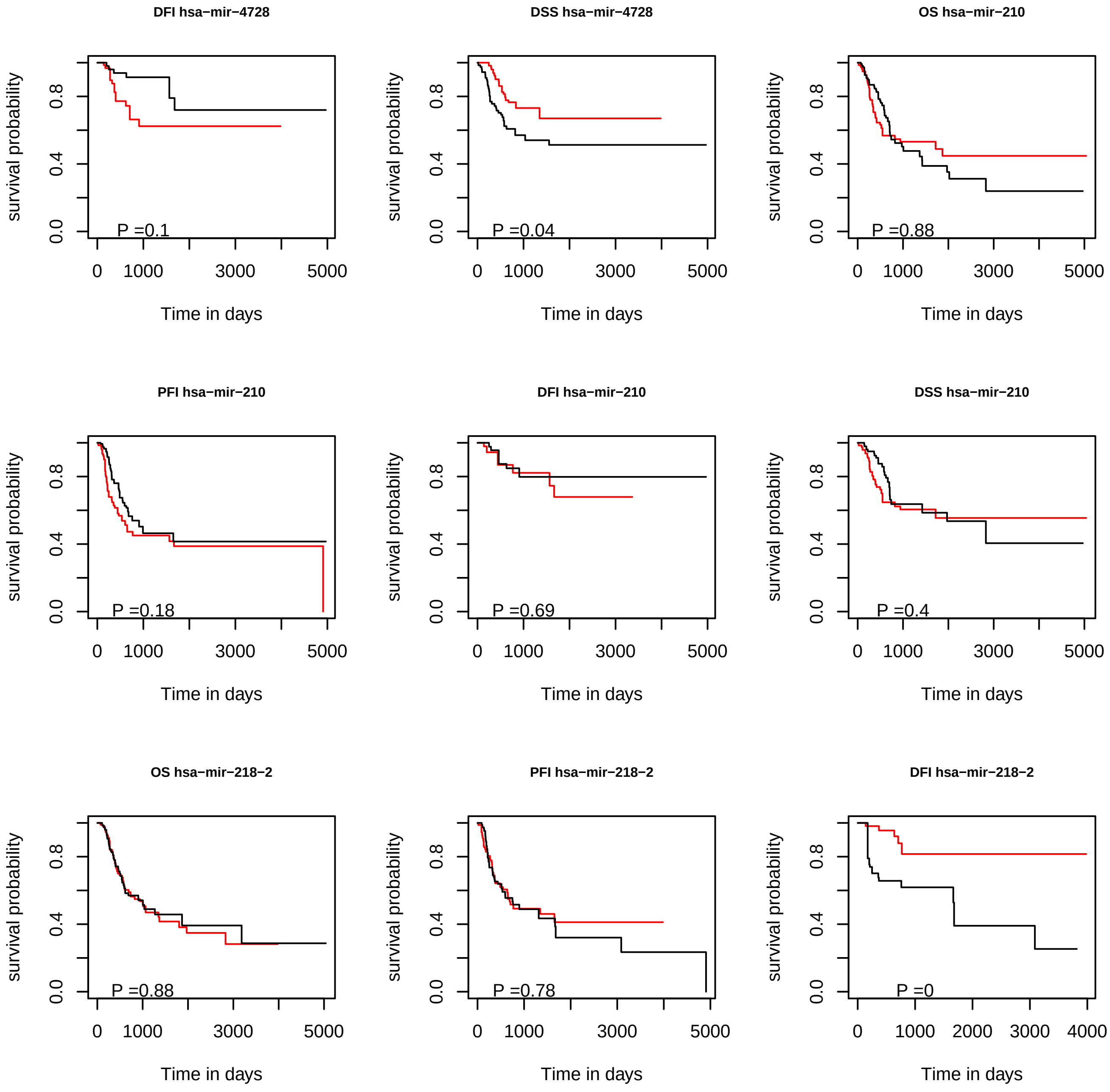
DFI hsa−mir−4728
survival probability
0.0 0.4 0.8
P =0.1
0 1000 3000 5000
Time in days
DSS hsa−mir−4728
survival probability
0.0 0.4 0.8
P =0.04
0 1000 3000 5000
Time in days
OS hsa−mir−210
survival probability
0.0 0.4 0.8
P =0.88
0 1000 3000 5000
Time in days
PFI hsa−mir−210
survival probability
0.0 0.4 0.8
P =0.18
0 1000 3000 5000
Time in days
DFI hsa−mir−210
survival probability
0.0 0.4 0.8
P =0.69
0 1000 3000 5000
Time in days
DSS hsa−mir−210
survival probability
0.0 0.4 0.8
P =0.4
0 1000 3000 5000
Time in days
OS hsa−mir−218−2
survival probability
0.0 0.4 0.8
P =0.88
0 1000 3000 5000
Time in days
PFI hsa−mir−218−2
survival probability
0.0 0.4 0.8
P =0.78
0 1000 3000 5000
Time in days
DFI hsa−mir−218−2
survival probability
0.0 0.4 0.8
P =0
0 1000 2000 3000 4000
Time in days

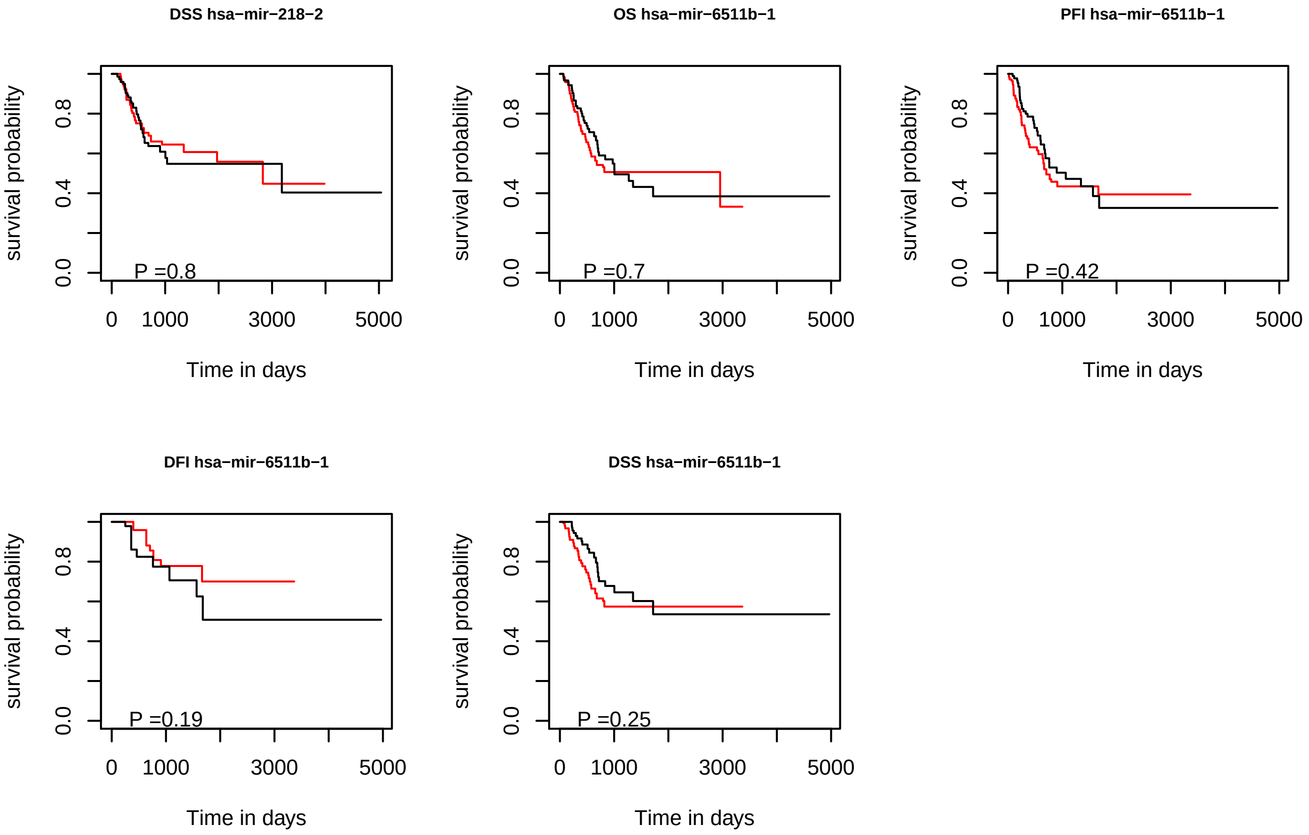
DSS hsa−mir−218−2
survival probability
0.0
0.4
0.8
0
1000
3000
5000
Time in days
P =0.8
OS hsa−mir−6511b−1
survival probability
Time in days
P =0.7
PFI hsa−mir−6511b−1
survival probability
Time in days
P =0.42
DFI hsa−mir−6511b−1
survival probability
Time in days
P =0.19
DSS hsa−mir−6511b−1
survival probability
Time in days
P =0.25
