# Supplementary material for: Computing microRNA-gene interaction networks in pan-cancer using miRDriver
Source: Sci Rep. 2022 Mar 8;12:3717. doi: 10.1038/s41598-022-07628-z (PMC8904490; doi:10.1038/s41598-022-07628-z)
Supplement: Supplementary file 12 — Supplementary Information 12. [file 41598_2022_7628_MOESM12_ESM.pdf]

Computing microRNA-gene interaction networks in pan-cancer using miRDriver

Banabithi Bose, Matthew Moravec, and Serdar Bozdag

Supplemental Figure S3

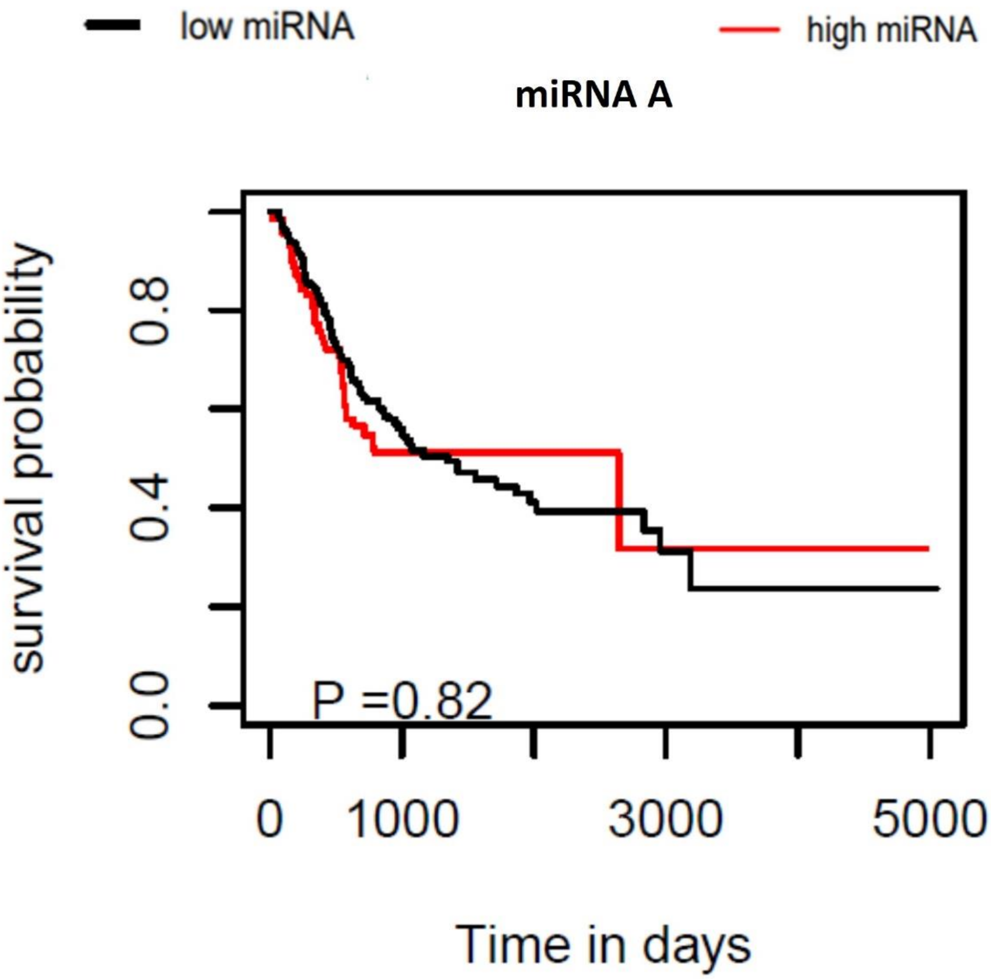

The *Adjusted Kaplan-Meier* survival plots for the computed miRNAs in high and low miRNA expression patient groups.

Supplemental Figure S3

Cancer Type: BRCA

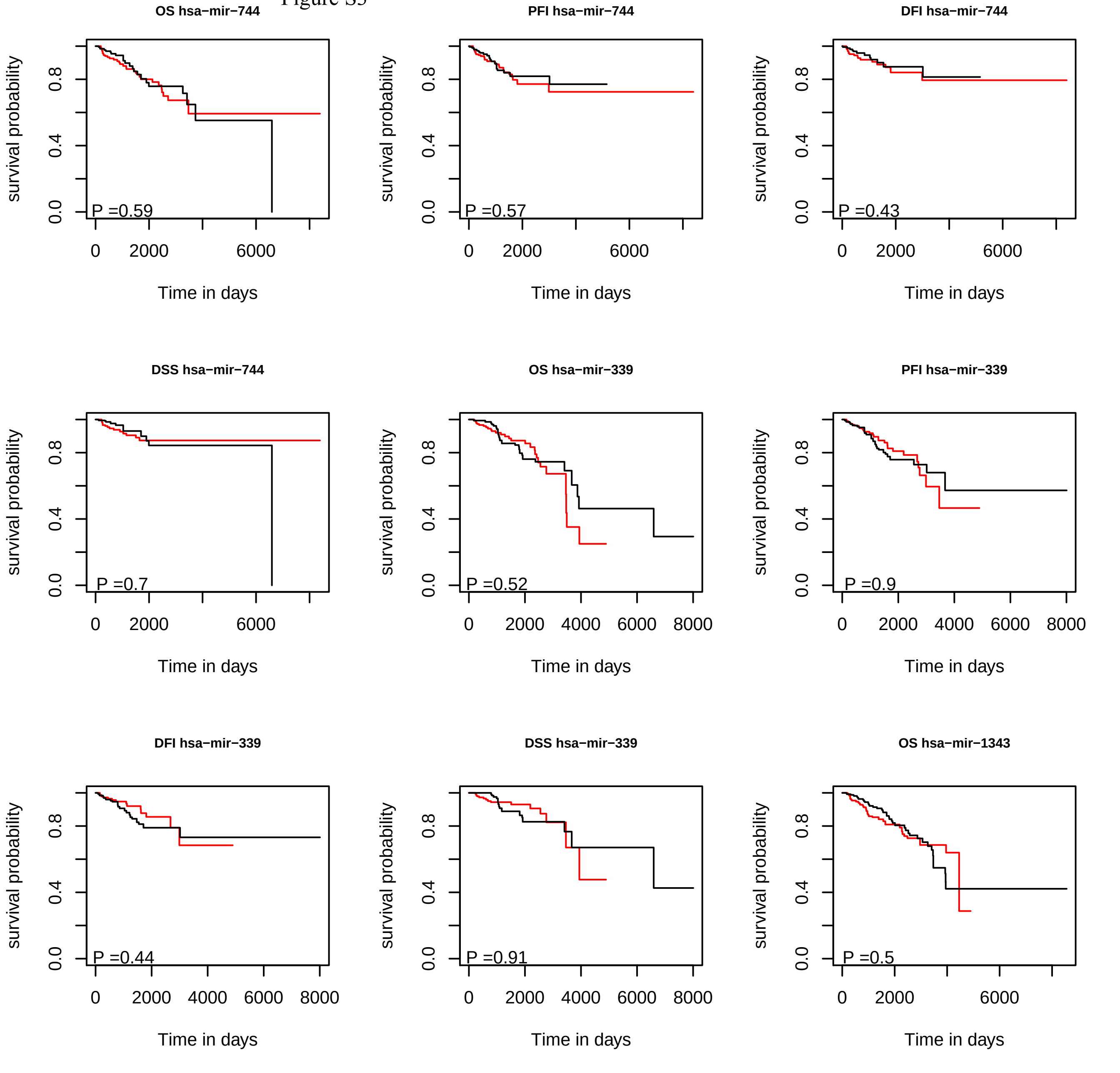

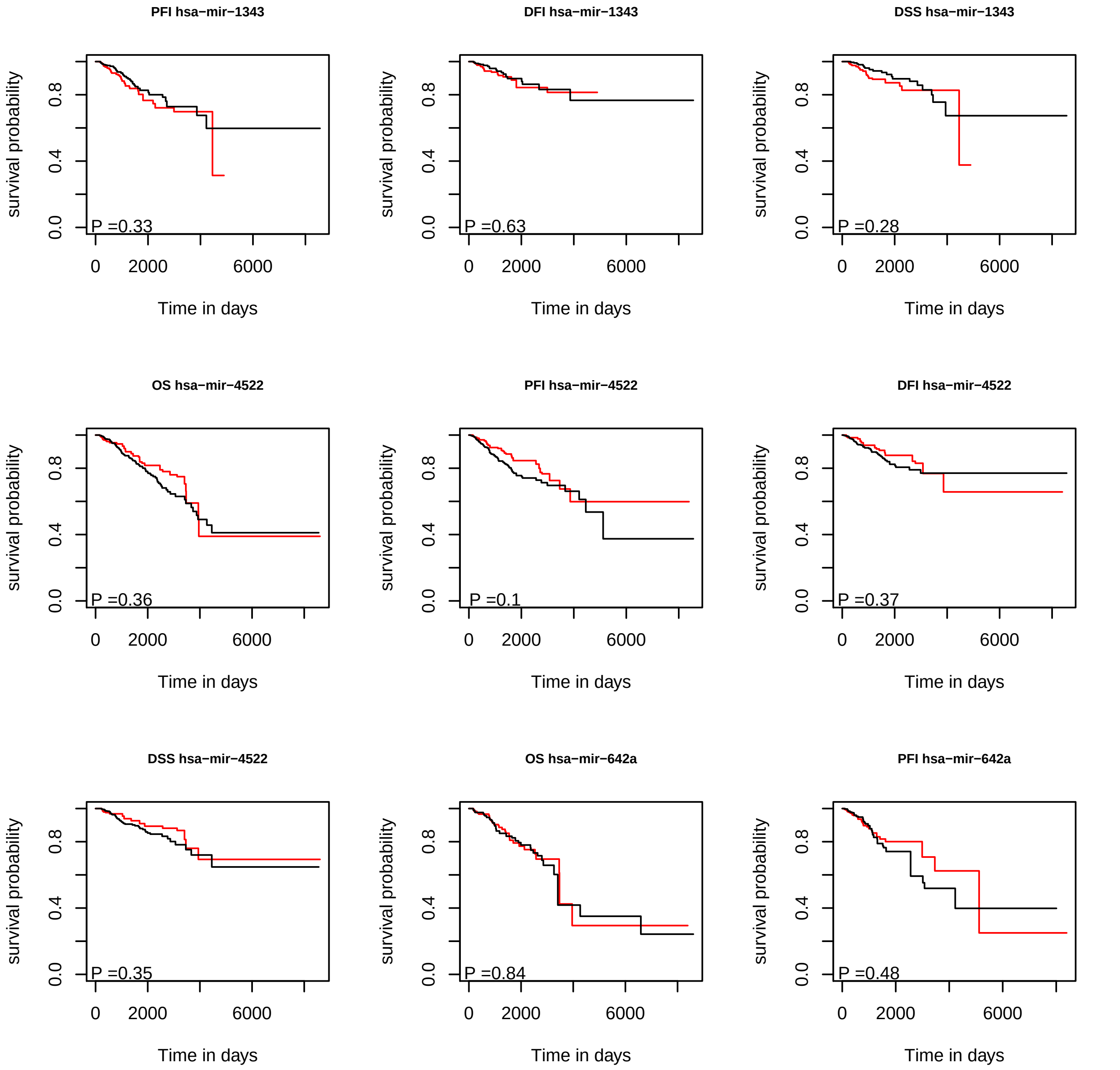
PFI hsa−mir−1343
P =0.33
DFI hsa−mir−1343
P =0.63
DSS hsa−mir−1343
P =0.28
OS hsa−mir−4522
P =0.36
PFI hsa−mir−4522
P =0.1
DFI hsa−mir−4522
P =0.37
DSS hsa−mir−4522
P =0.35
OS hsa−mir−642a
P =0.84
PFI hsa−mir−642a
P =0.48
survival probability
0.0
0.4
0.8
0
2000
6000
Time in days

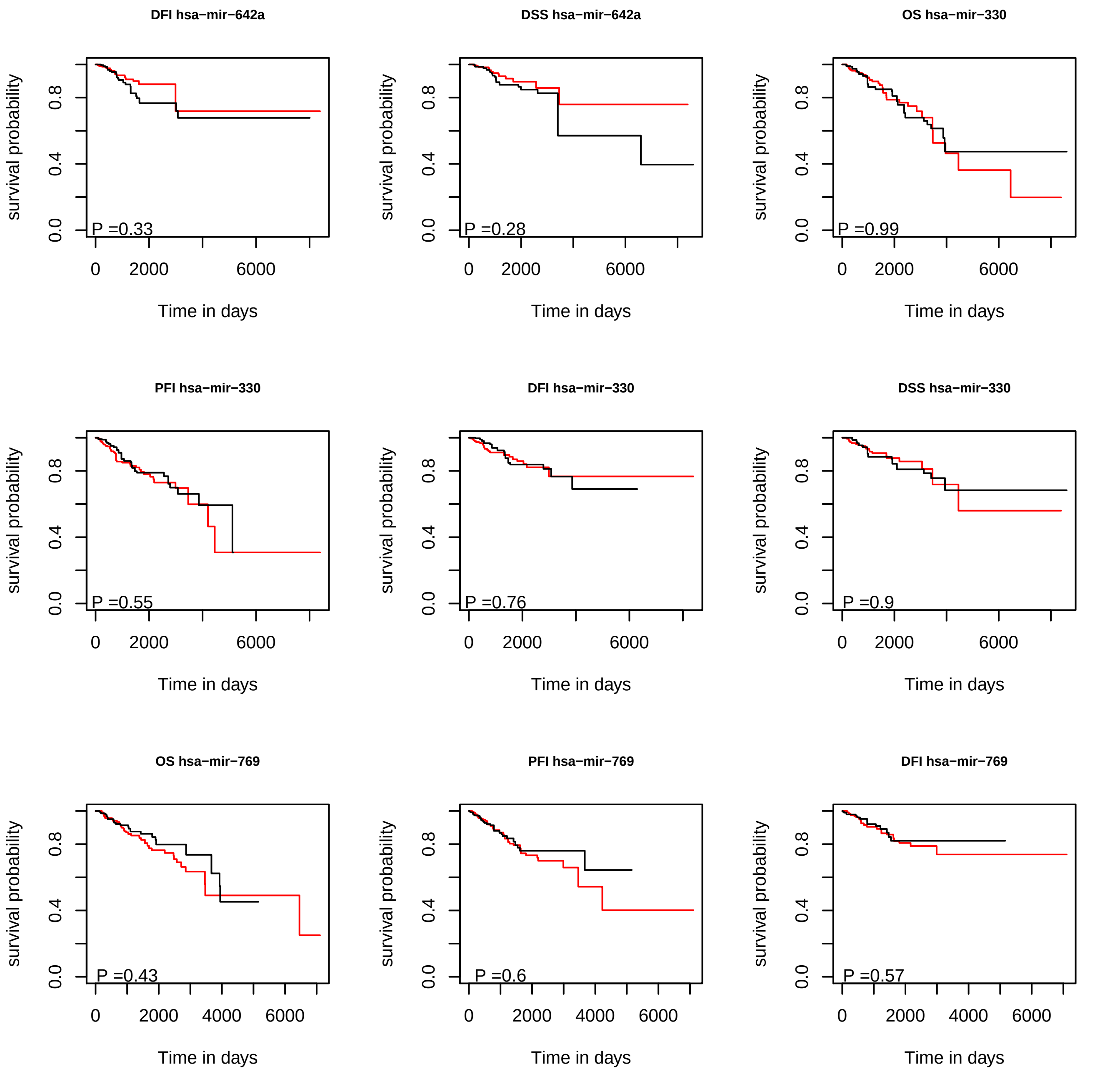
DFI hsa−mir−642a
P =0.33
DSS hsa−mir−642a
P =0.28
OS hsa−mir−330
P =0.99
PFI hsa−mir−330
P =0.55
DFI hsa−mir−330
P =0.76
DSS hsa−mir−330
P =0.9
OS hsa−mir−769
P =0.43
PFI hsa−mir−769
P =0.6
DFI hsa−mir−769
P =0.57
survival probability
Time in days

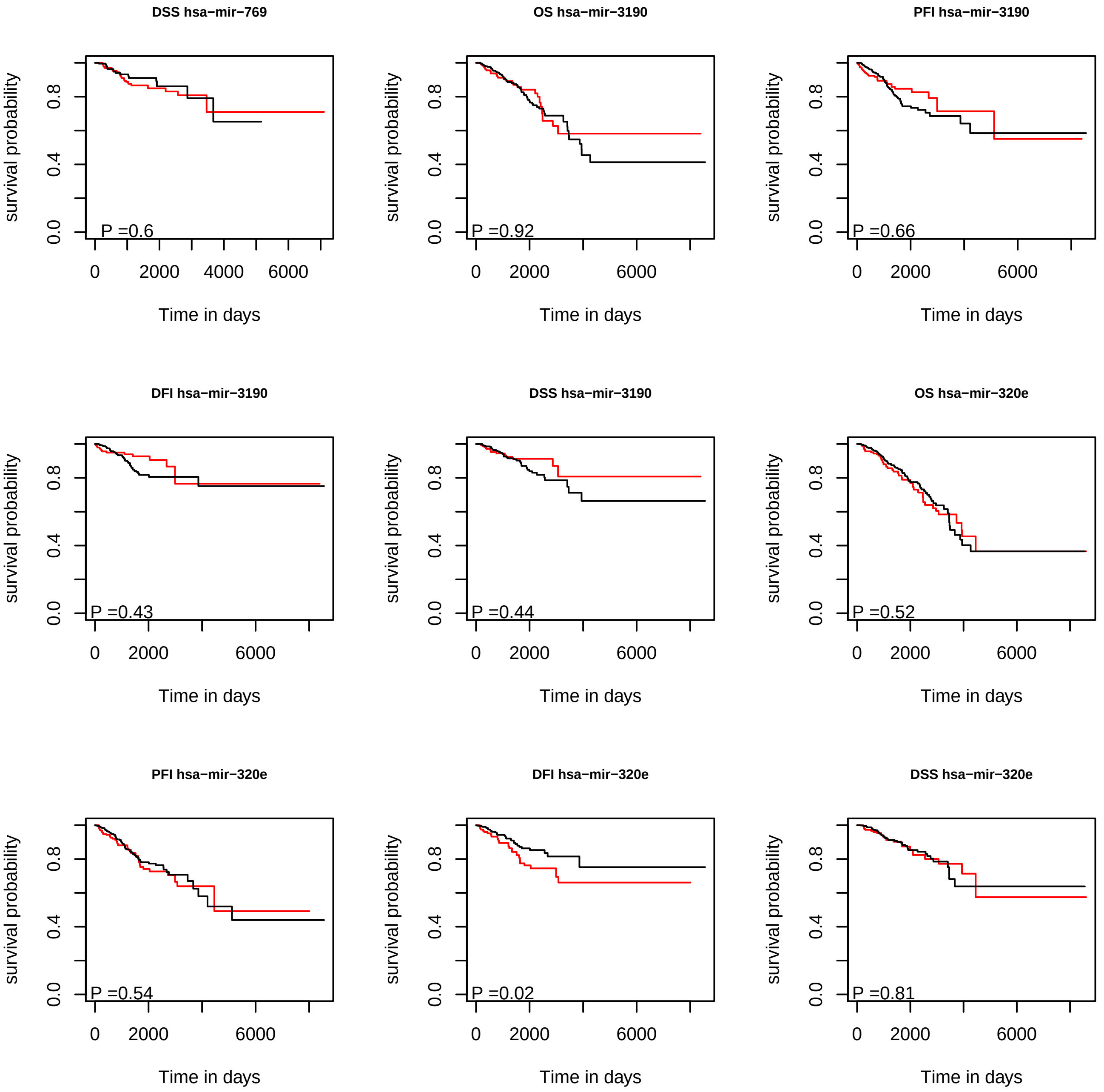
DSS hsa−mir−769
survival probability
0.0
0.4
0.8
P =0.6
0
2000
4000
6000
Time in days
OS hsa−mir−3190
survival probability
0.0
0.4
0.8
P =0.92
0
2000
6000
Time in days
PFI hsa−mir−3190
survival probability
0.0
0.4
0.8
P =0.66
0
2000
6000
Time in days
DFI hsa−mir−3190
survival probability
0.0
0.4
0.8
P =0.43
0
2000
6000
Time in days
DSS hsa−mir−3190
survival probability
0.0
0.4
0.8
P =0.44
0
2000
6000
Time in days
OS hsa−mir−320e
survival probability
0.0
0.4
0.8
P =0.52
0
2000
6000
Time in days
PFI hsa−mir−320e
survival probability
0.0
0.4
0.8
P =0.54
0
2000
6000
Time in days
DFI hsa−mir−320e
survival probability
0.0
0.4
0.8
P =0.02
0
2000
6000
Time in days
DSS hsa−mir−320e
survival probability
0.0
0.4
0.8
P =0.81
0
2000
6000
Time in days

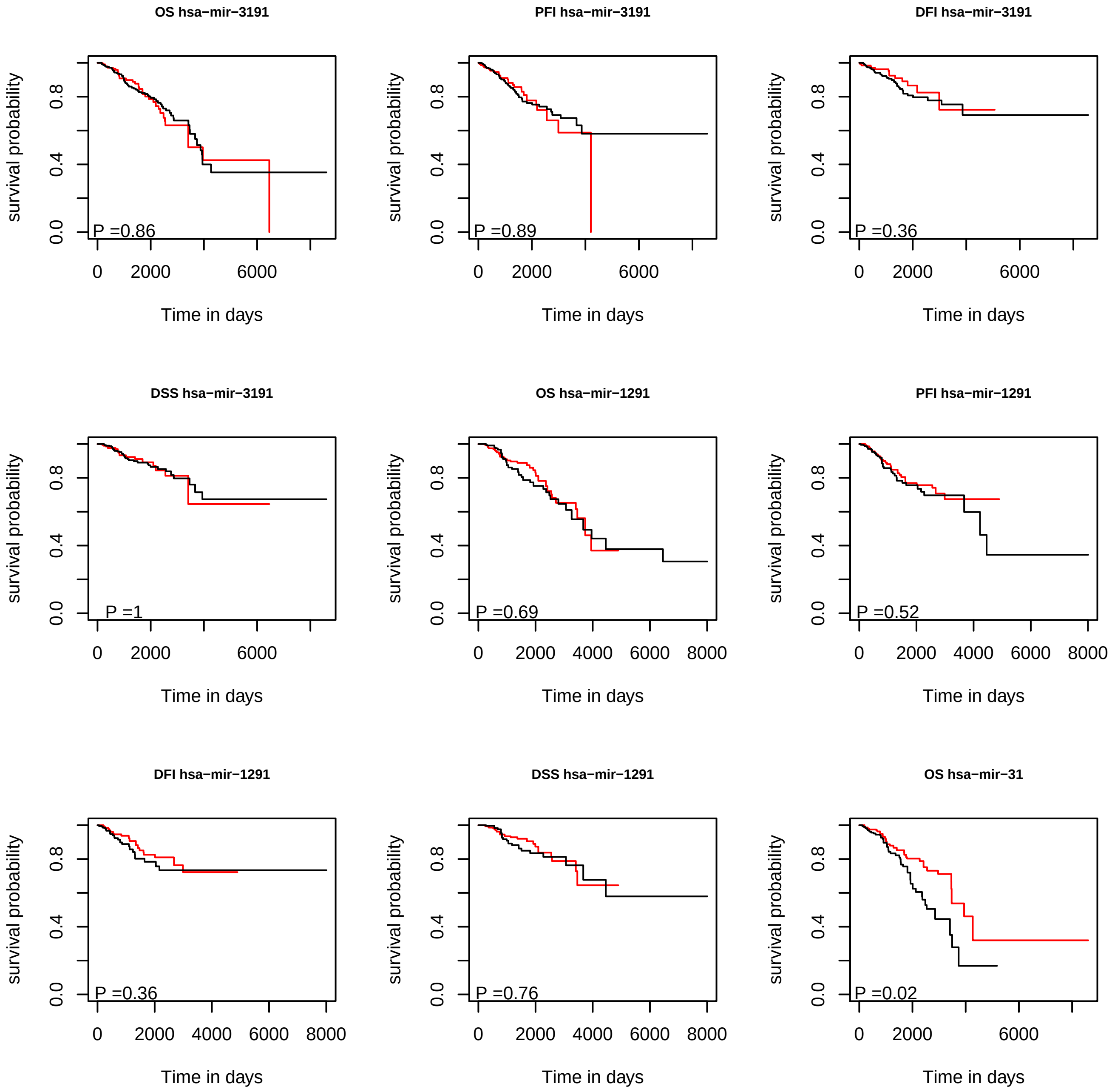
OS hsa−mir−3191
P =0.86
PFI hsa−mir−3191
P =0.89
DFI hsa−mir−3191
P =0.36
DSS hsa−mir−3191
P =1
OS hsa−mir−1291
P =0.69
PFI hsa−mir−1291
P =0.52
DFI hsa−mir−1291
P =0.36
DSS hsa−mir−1291
P =0.76
OS hsa−mir−31
P =0.02
survival probability
Time in days
0.0
0.4
0.8
0
2000
4000
6000
8000

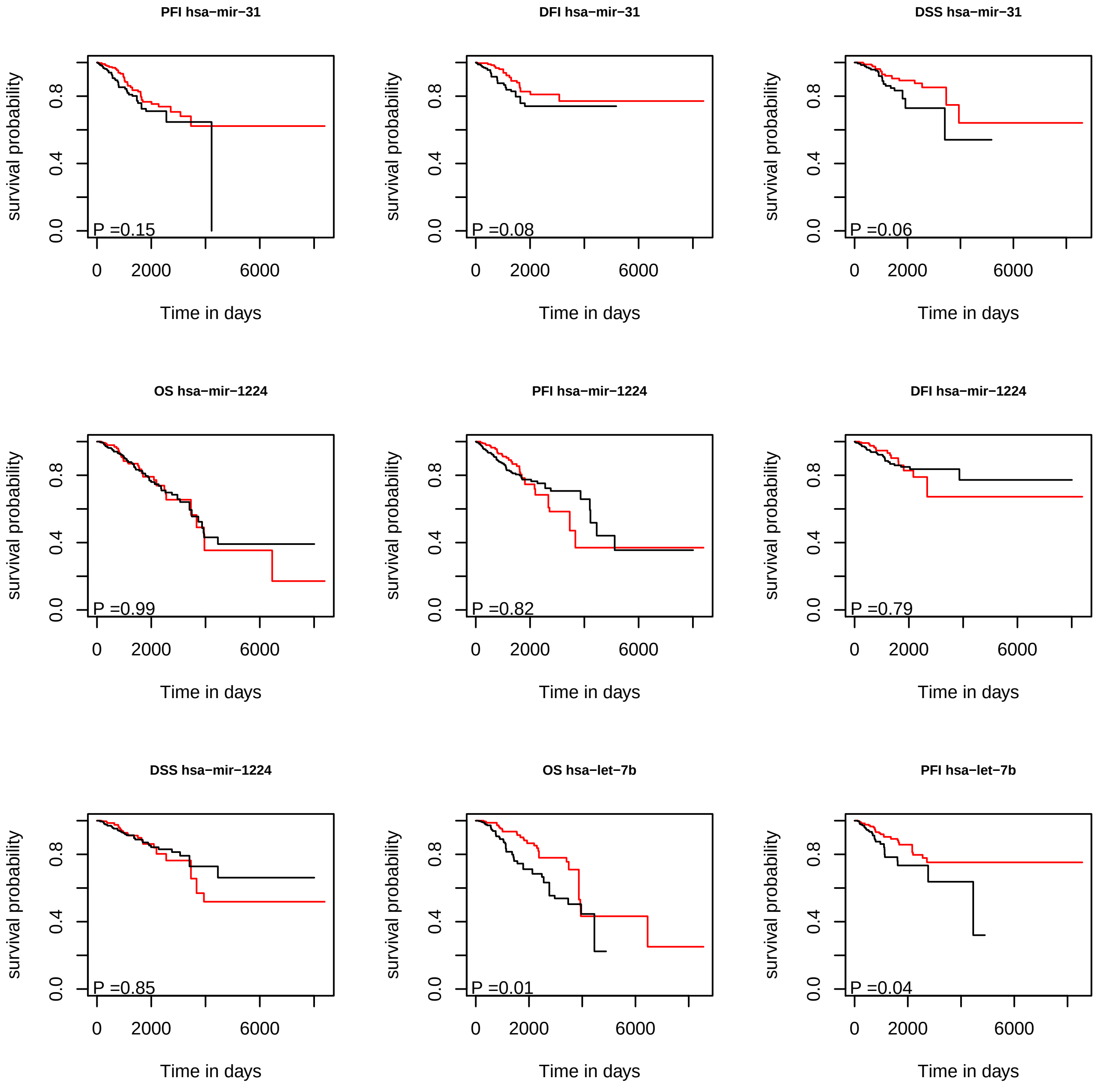
PFI hsa−mir−31
DFI hsa−mir−31
DSS hsa−mir−31
OS hsa−mir−1224
PFI hsa−mir−1224
DFI hsa−mir−1224
DSS hsa−mir−1224
OS hsa−let−7b
PFI hsa−let−7b
survival probability
Time in days
0.0
0.4
0.8
0
2000
6000
P =0.15
P =0.08
P =0.06
P =0.99
P =0.82
P =0.79
P =0.85
P =0.01
P =0.04

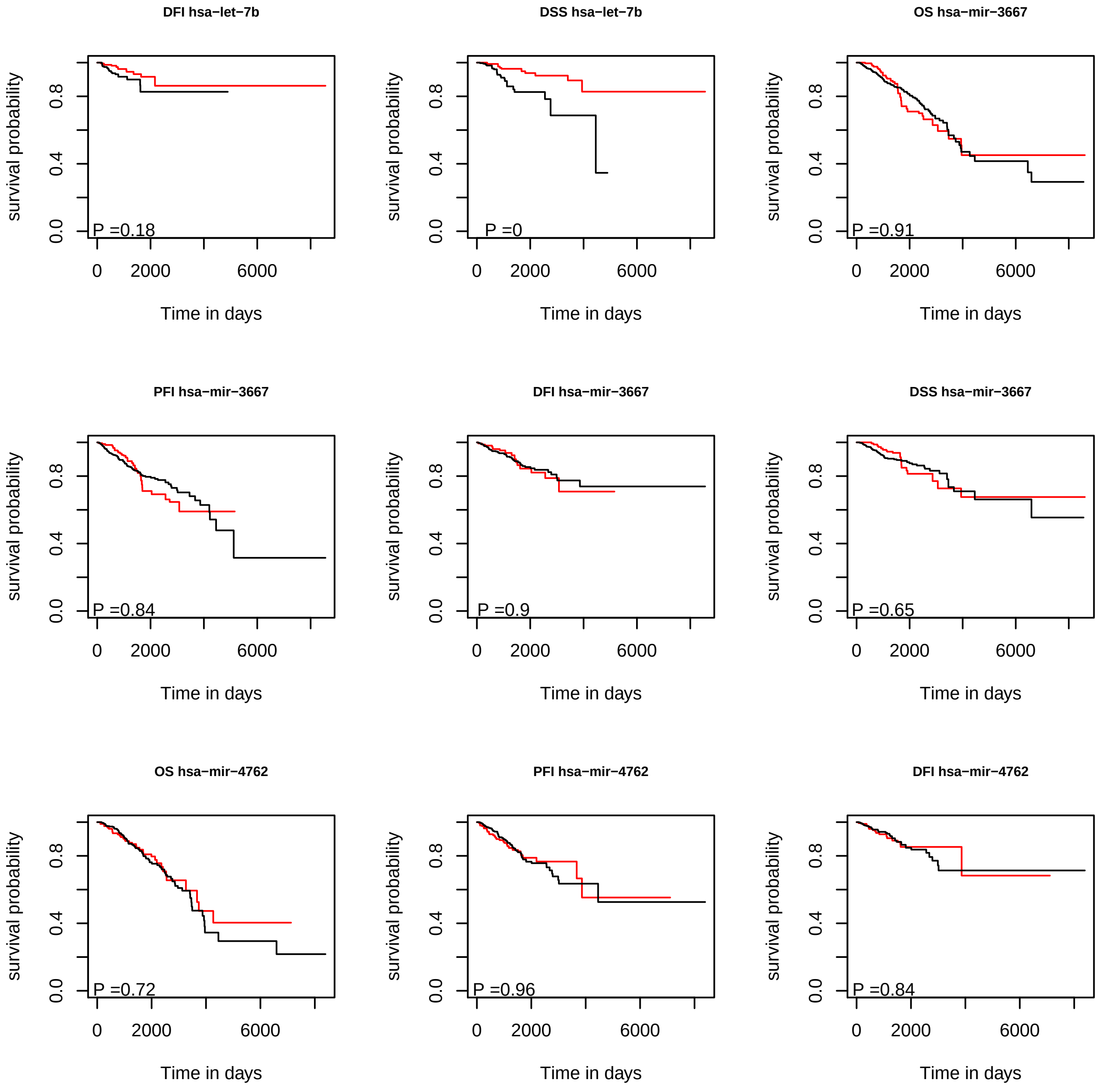
DFI hsa−let−7b
P =0.18
DSS hsa−let−7b
P =0
OS hsa−mir−3667
P =0.91
PFI hsa−mir−3667
P =0.84
DFI hsa−mir−3667
P =0.9
DSS hsa−mir−3667
P =0.65
OS hsa−mir−4762
P =0.72
PFI hsa−mir−4762
P =0.96
DFI hsa−mir−4762
P =0.84
survival probability
0.0
0.4
0.8
0
2000
6000
Time in days

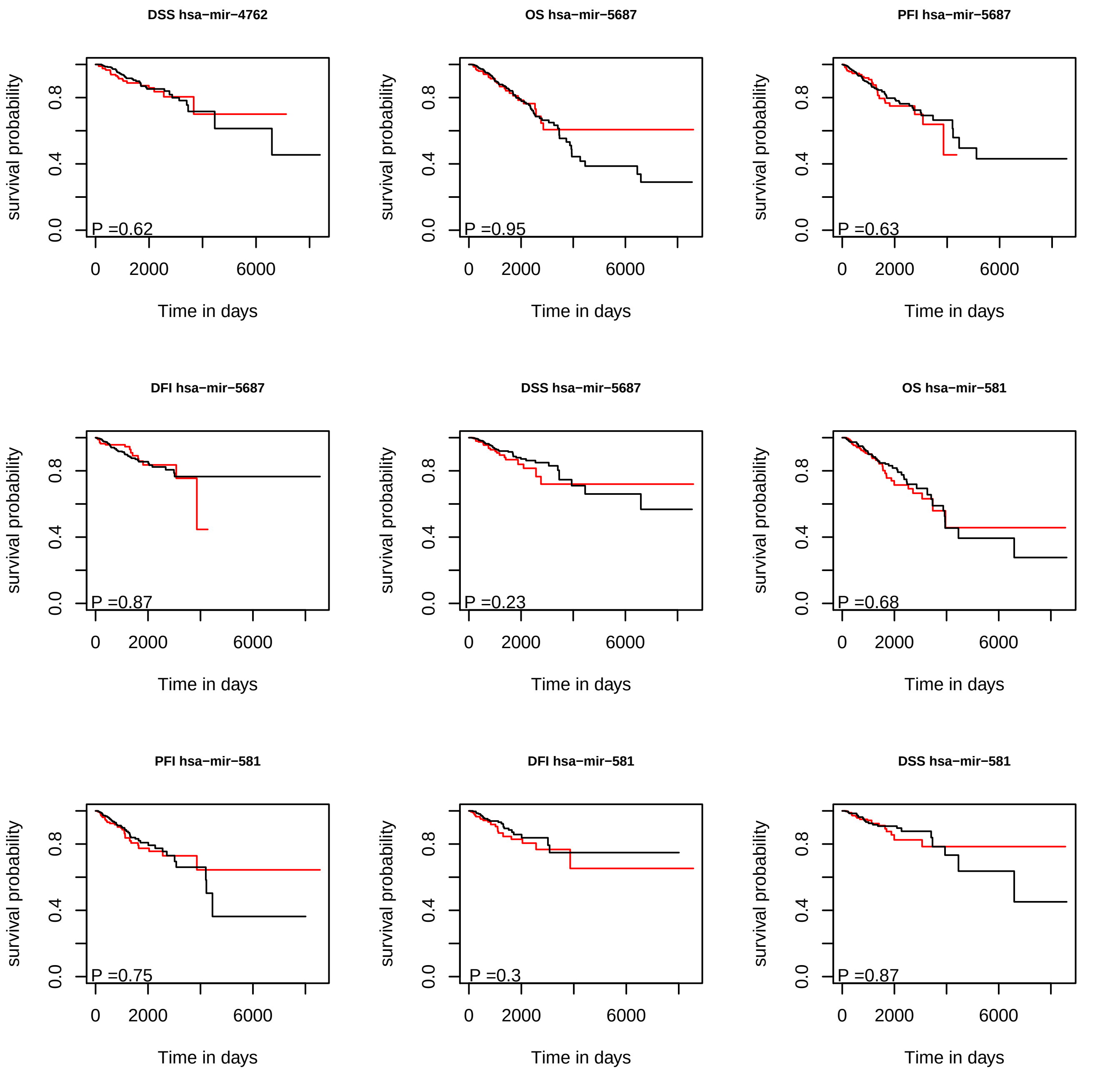
DSS hsa−mir−4762
P =0.62
OS hsa−mir−5687
P =0.95
PFI hsa−mir−5687
P =0.63
DFI hsa−mir−5687
P =0.87
DSS hsa−mir−5687
P =0.23
OS hsa−mir−581
P =0.68
PFI hsa−mir−581
P =0.75
DFI hsa−mir−581
P =0.3
DSS hsa−mir−581
P =0.87
survival probability
Time in days
0.0
0.4
0.8
0
2000
6000

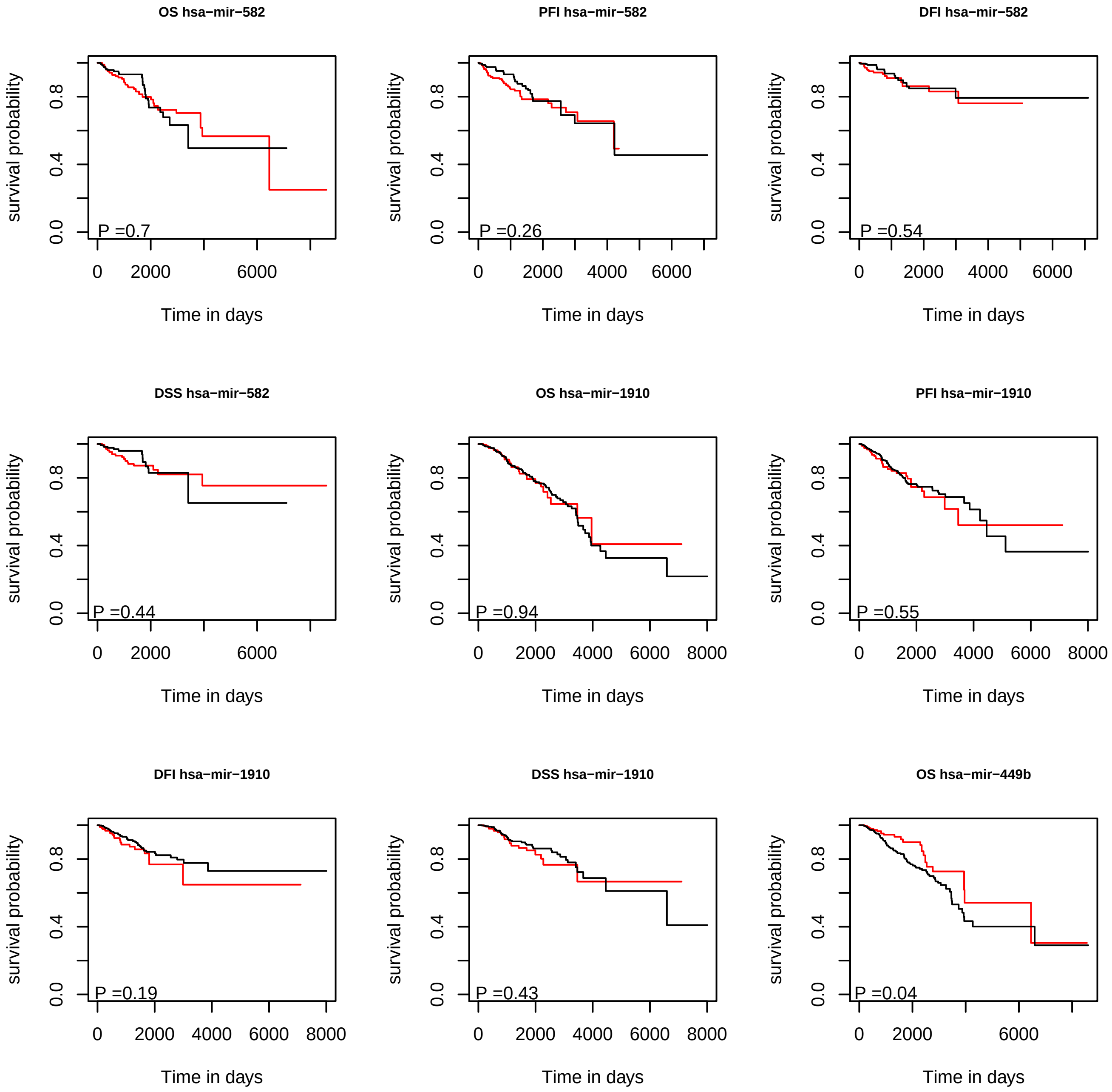
OS hsa−mir−582
P =0.7
PFI hsa−mir−582
P =0.26
DFI hsa−mir−582
P =0.54
DSS hsa−mir−582
P =0.44
OS hsa−mir−1910
P =0.94
PFI hsa−mir−1910
P =0.55
DFI hsa−mir−1910
P =0.19
DSS hsa−mir−1910
P =0.43
OS hsa−mir−449b
P =0.04
survival probability
Time in days
0.0
0.4
0.8
0
2000
4000
6000
8000

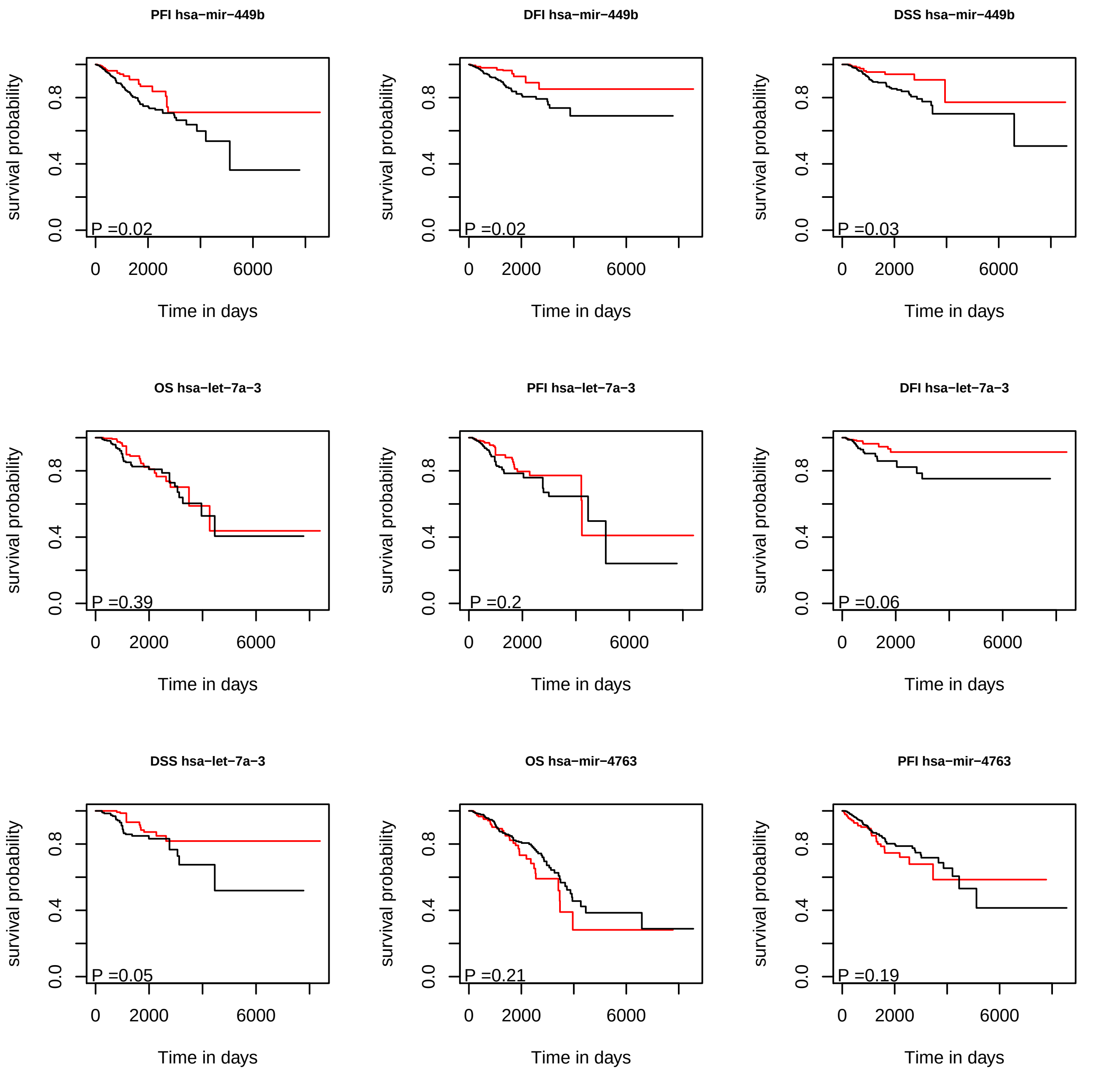

PFI hsa−mir−449b
DFI hsa−mir−449b
DSS hsa−mir−449b
P =0.02
P =0.02
P =0.03
OS hsa−let−7a−3
PFI hsa−let−7a−3
DFI hsa−let−7a−3
P =0.39
P =0.2
P =0.06
DSS hsa−let−7a−3
OS hsa−mir−4763
PFI hsa−mir−4763
P =0.05
P =0.21
P =0.19
survival probability
0.0
0.4
0.8
Time in days
0
2000
6000

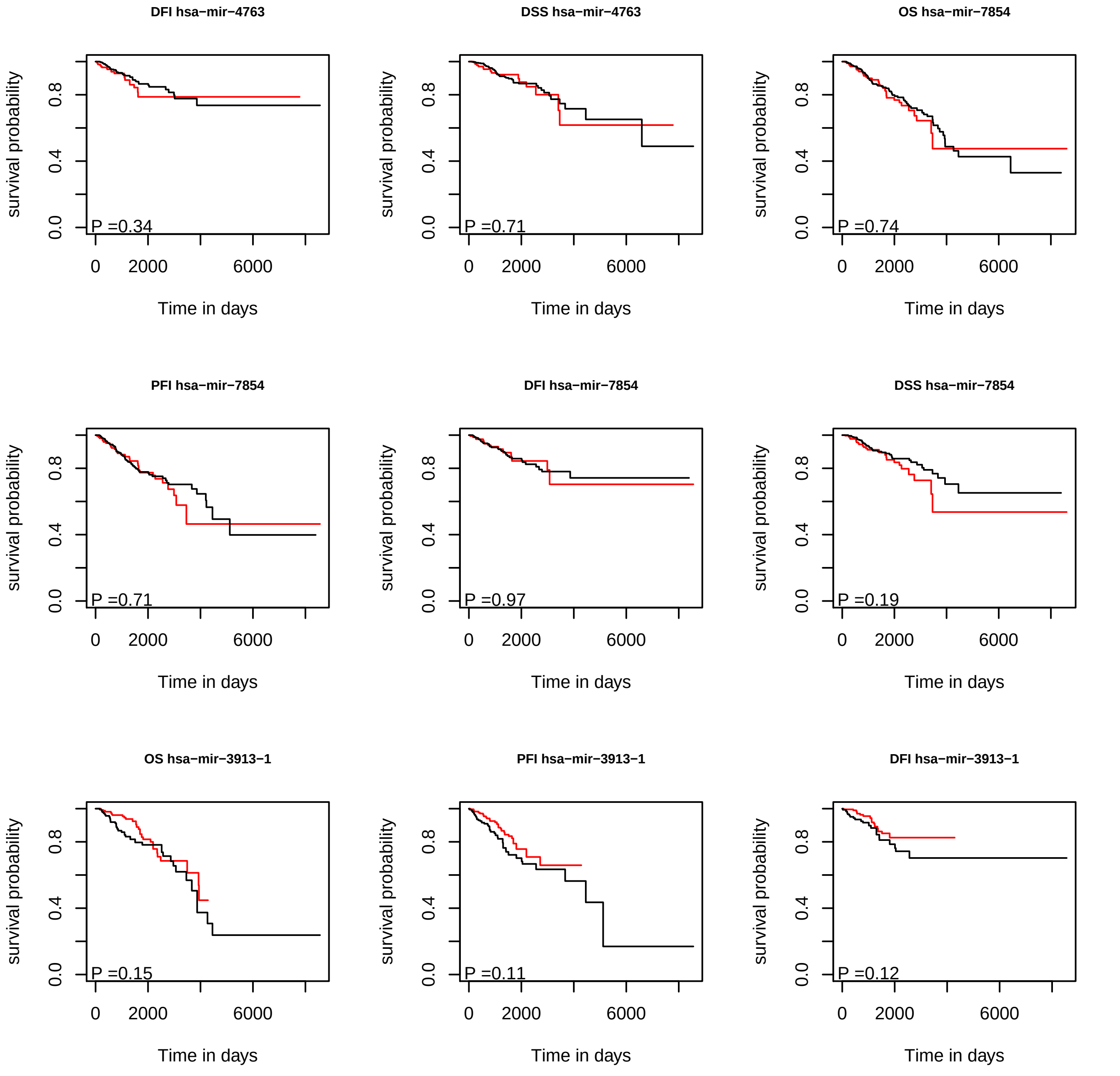

DFI hsa−mir−4763
P =0.34
DSS hsa−mir−4763
P =0.71
OS hsa−mir−7854
P =0.74
PFI hsa−mir−7854
P =0.71
DFI hsa−mir−7854
P =0.97
DSS hsa−mir−7854
P =0.19
OS hsa−mir−3913−1
P =0.15
PFI hsa−mir−3913−1
P =0.11
DFI hsa−mir−3913−1
P =0.12
survival probability
0.0
0.4
0.8
0
2000
6000
Time in days

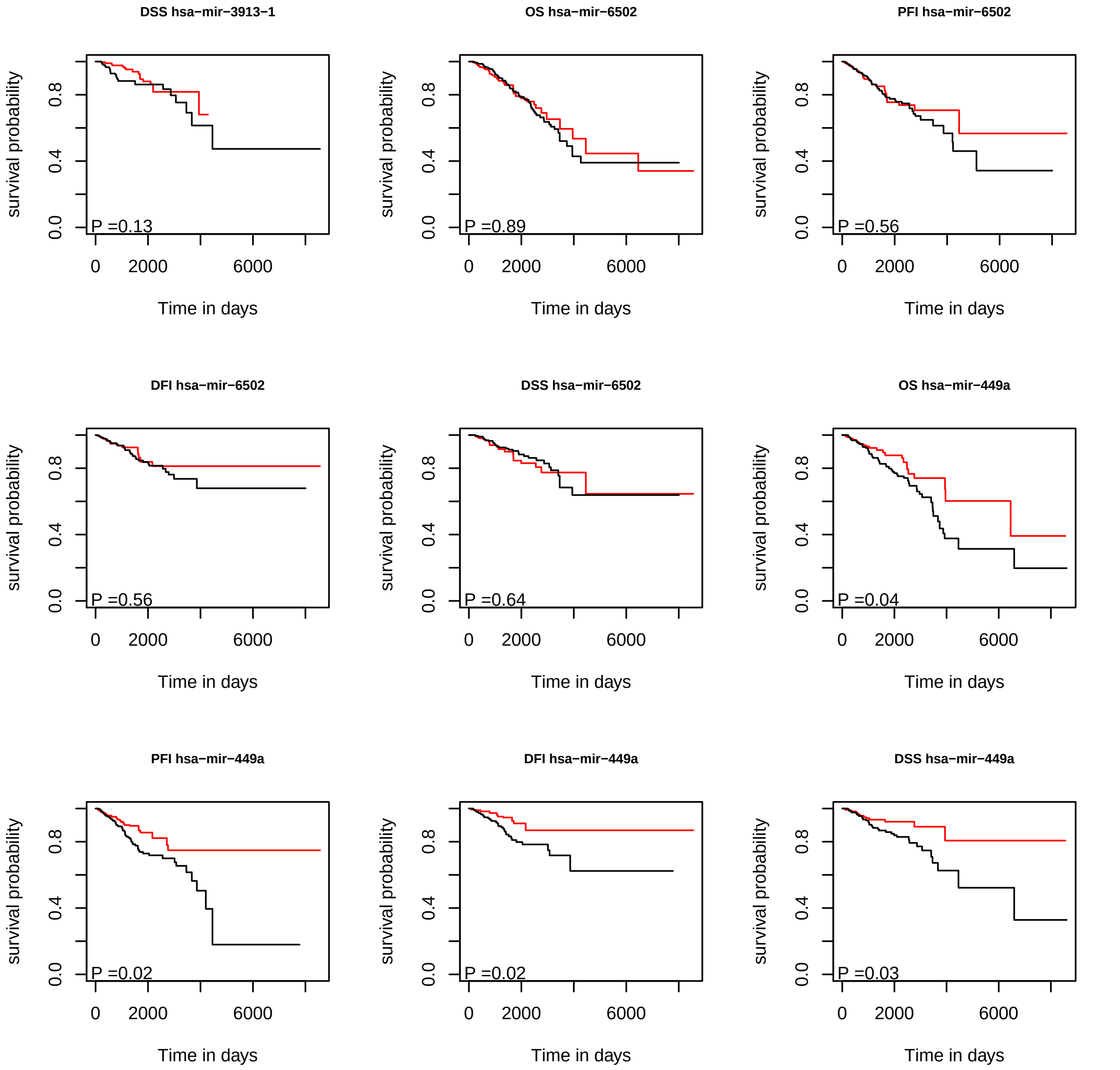
DSS hsa−mir−3913−1
OS hsa−mir−6502
PFI hsa−mir−6502
DFI hsa−mir−6502
DSS hsa−mir−6502
OS hsa−mir−449a
PFI hsa−mir−449a
DFI hsa−mir−449a
DSS hsa−mir−449a
survival probability
0.0
0.4
0.8
Time in days
0
2000
6000
P =0.13
P =0.89
P =0.56
P =0.56
P =0.64
P =0.04
P =0.02
P =0.02
P =0.03

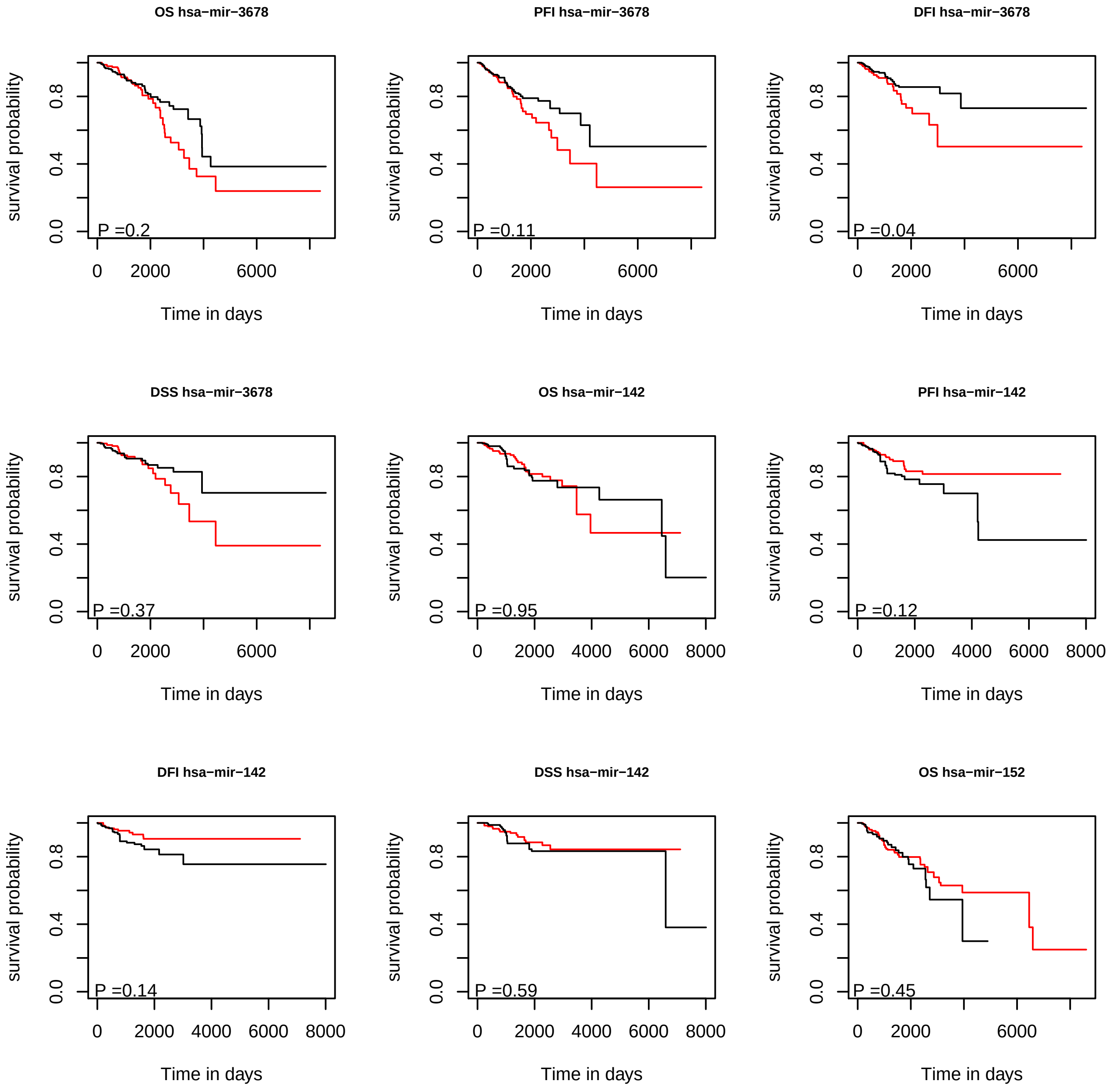
OS hsa−mir−3678
P =0.2
PFI hsa−mir−3678
P =0.11
DFI hsa−mir−3678
P =0.04
DSS hsa−mir−3678
P =0.37
OS hsa−mir−142
P =0.95
PFI hsa−mir−142
P =0.12
DFI hsa−mir−142
P =0.14
DSS hsa−mir−142
P =0.59
OS hsa−mir−152
P =0.45
survival probability
Time in days
0.0
0.4
0.8
0
2000
4000
6000
8000

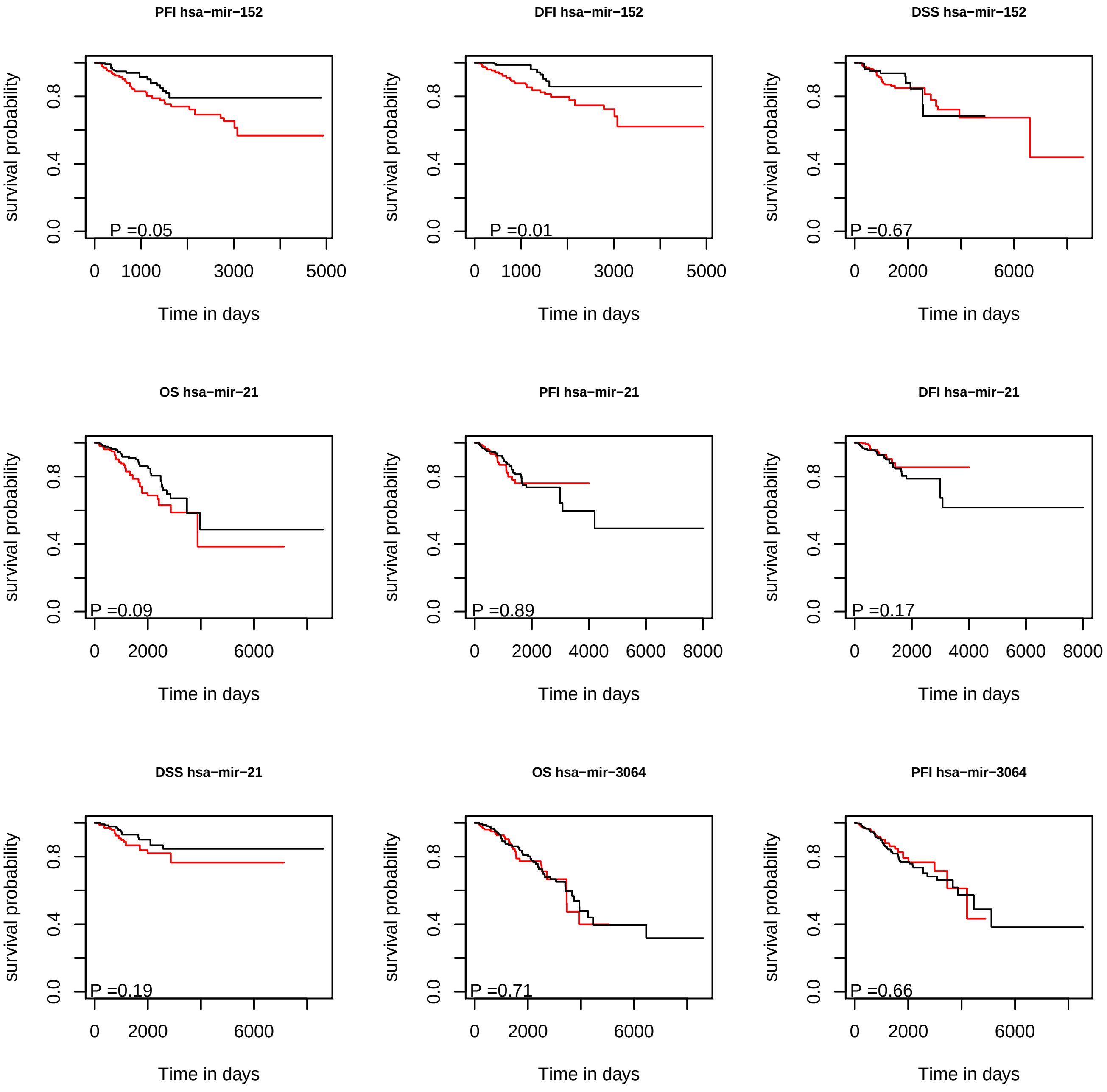
PFI hsa−mir−152
P =0.05
DFI hsa−mir−152
P =0.01
DSS hsa−mir−152
P =0.67
OS hsa−mir−21
P =0.09
PFI hsa−mir−21
P =0.89
DFI hsa−mir−21
P =0.17
DSS hsa−mir−21
P =0.19
OS hsa−mir−3064
P =0.71
PFI hsa−mir−3064
P =0.66
survival probability
Time in days

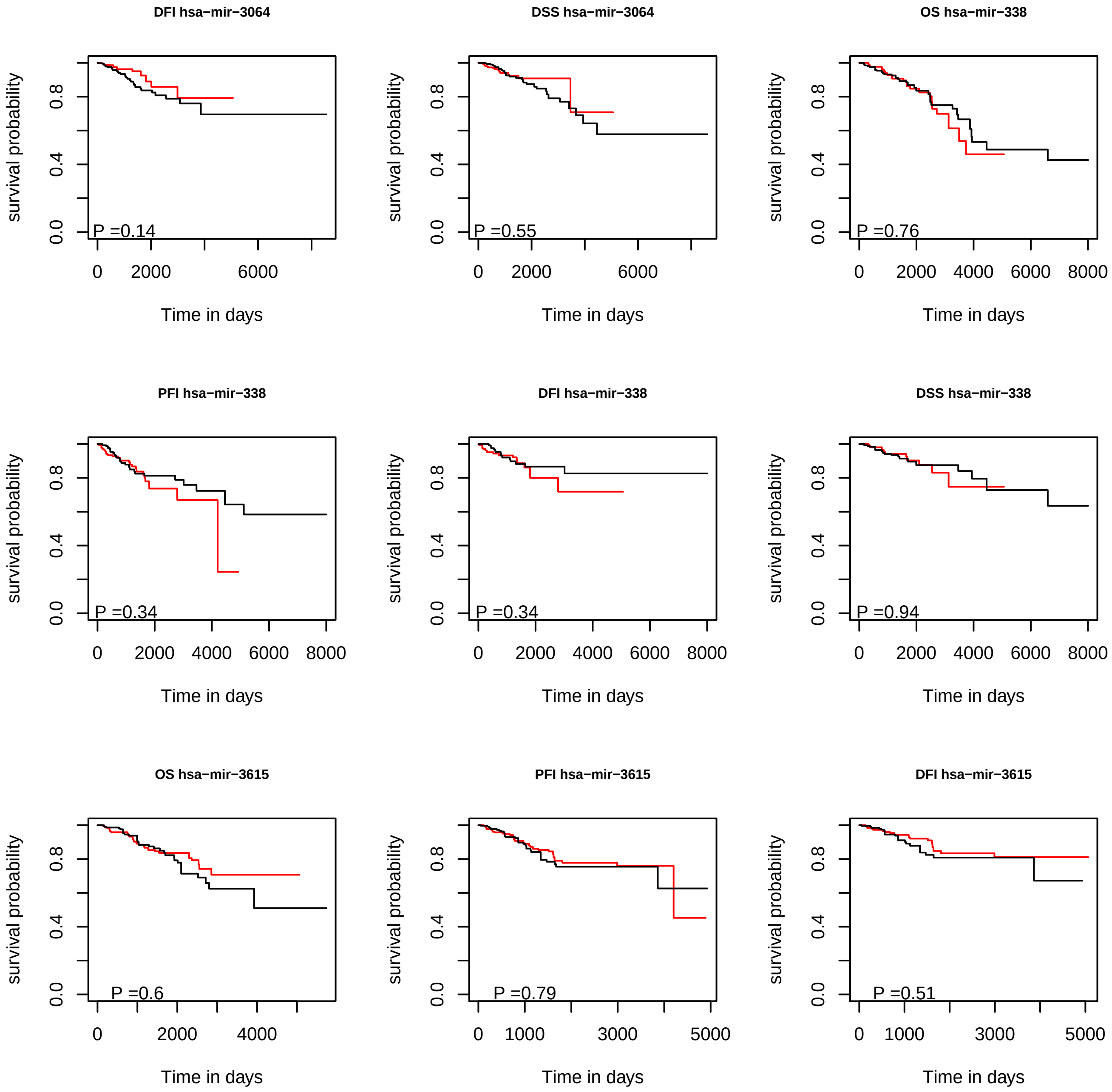

DFI hsa−mir−3064
P =0.14
DSS hsa−mir−3064
P =0.55
OS hsa−mir−338
P =0.76
PFI hsa−mir−338
P =0.34
DFI hsa−mir−338
P =0.34
DSS hsa−mir−338
P =0.94
OS hsa−mir−3615
P =0.6
PFI hsa−mir−3615
P =0.79
DFI hsa−mir−3615
P =0.51
survival probability
Time in days

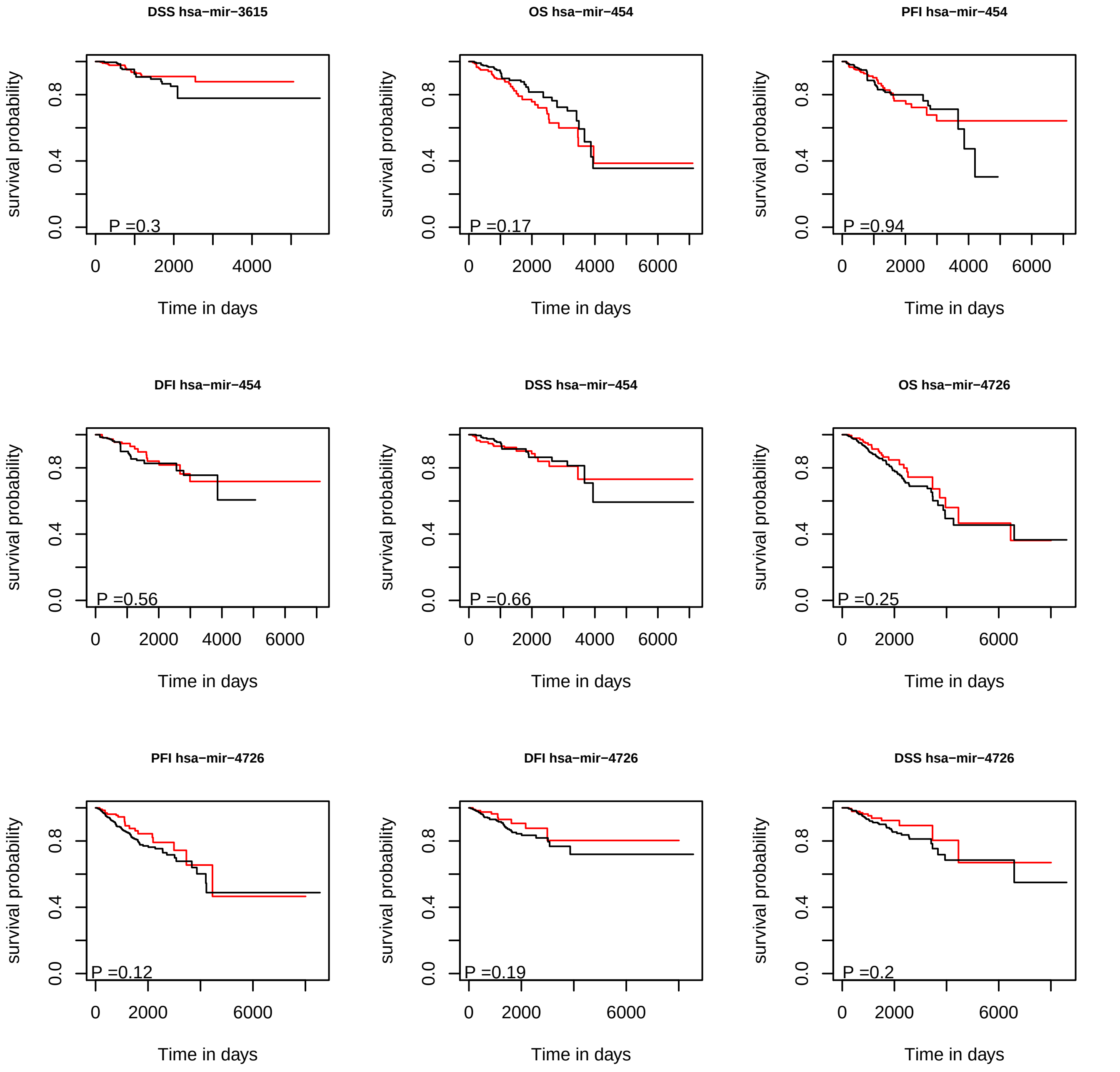
DSS hsa−mir−3615
P =0.3
OS hsa−mir−454
P =0.17
PFI hsa−mir−454
P =0.94
DFI hsa−mir−454
P =0.56
DSS hsa−mir−454
P =0.66
OS hsa−mir−4726
P =0.25
PFI hsa−mir−4726
P =0.12
DFI hsa−mir−4726
P =0.19
DSS hsa−mir−4726
P =0.2
survival probability
Time in days

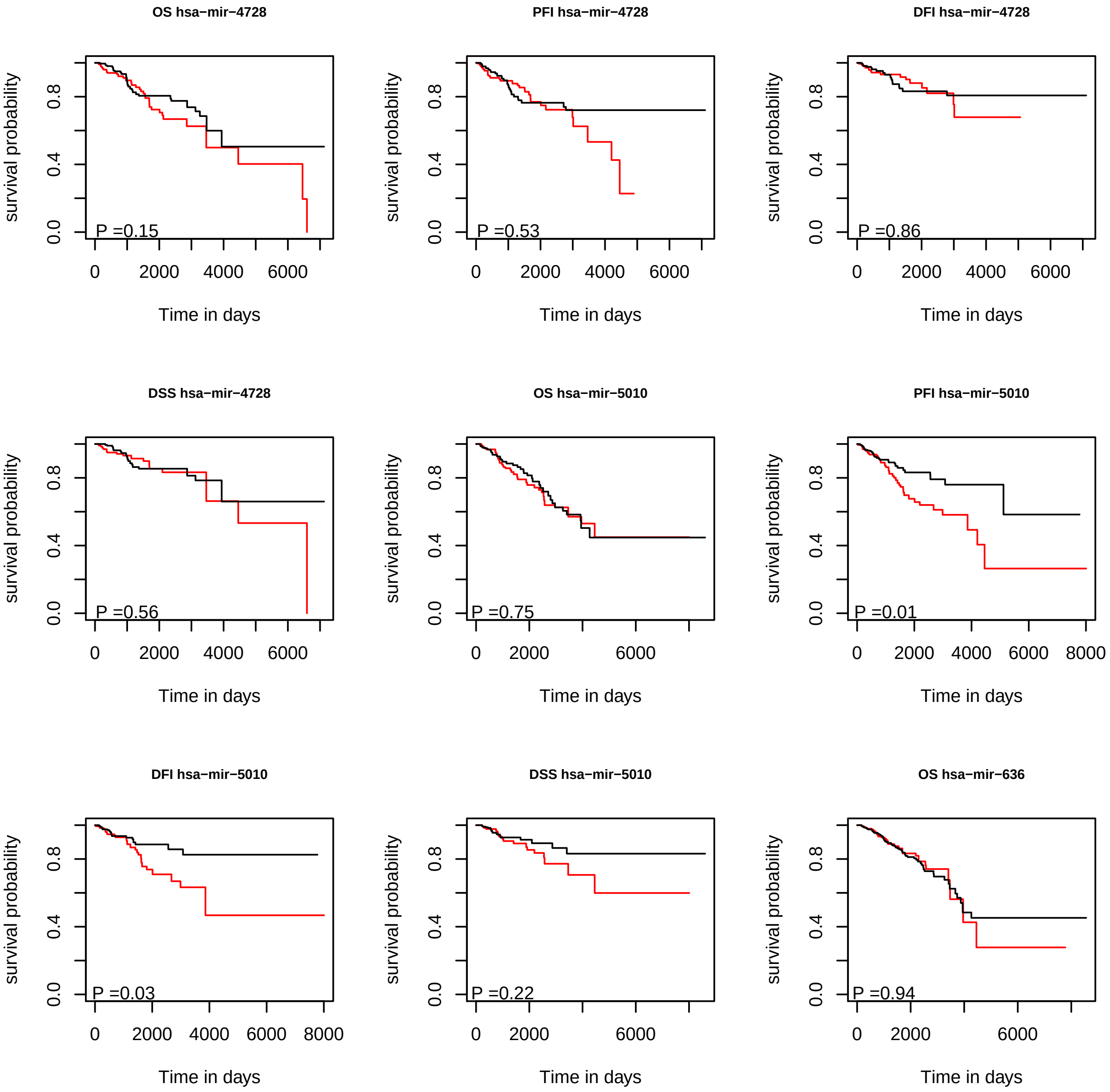
OS hsa−mir−4728
P =0.15
PFI hsa−mir−4728
P =0.53
DFI hsa−mir−4728
P =0.86
DSS hsa−mir−4728
P =0.56
OS hsa−mir−5010
P =0.75
PFI hsa−mir−5010
P =0.01
DFI hsa−mir−5010
P =0.03
DSS hsa−mir−5010
P =0.22
OS hsa−mir−636
P =0.94
survival probability
Time in days
0.0
0.4
0.8
0
2000
4000
6000
8000

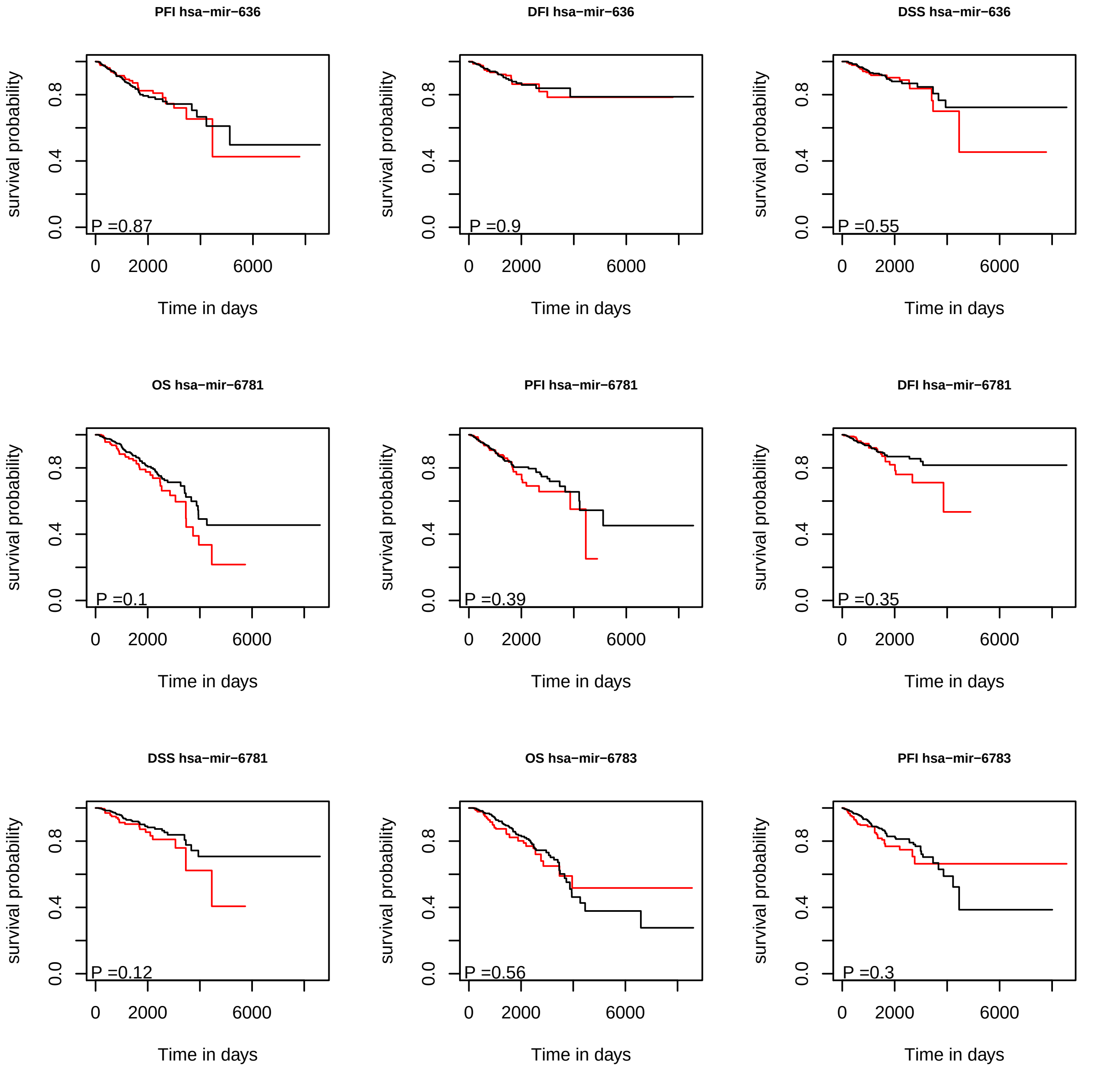
PFI hsa−mir−636
P =0.87
DFI hsa−mir−636
P =0.9
DSS hsa−mir−636
P =0.55
OS hsa−mir−6781
P =0.1
PFI hsa−mir−6781
P =0.39
DFI hsa−mir−6781
P =0.35
DSS hsa−mir−6781
P =0.12
OS hsa−mir−6783
P =0.56
PFI hsa−mir−6783
P =0.3
survival probability
0.0
0.4
0.8
Time in days
0
2000
6000

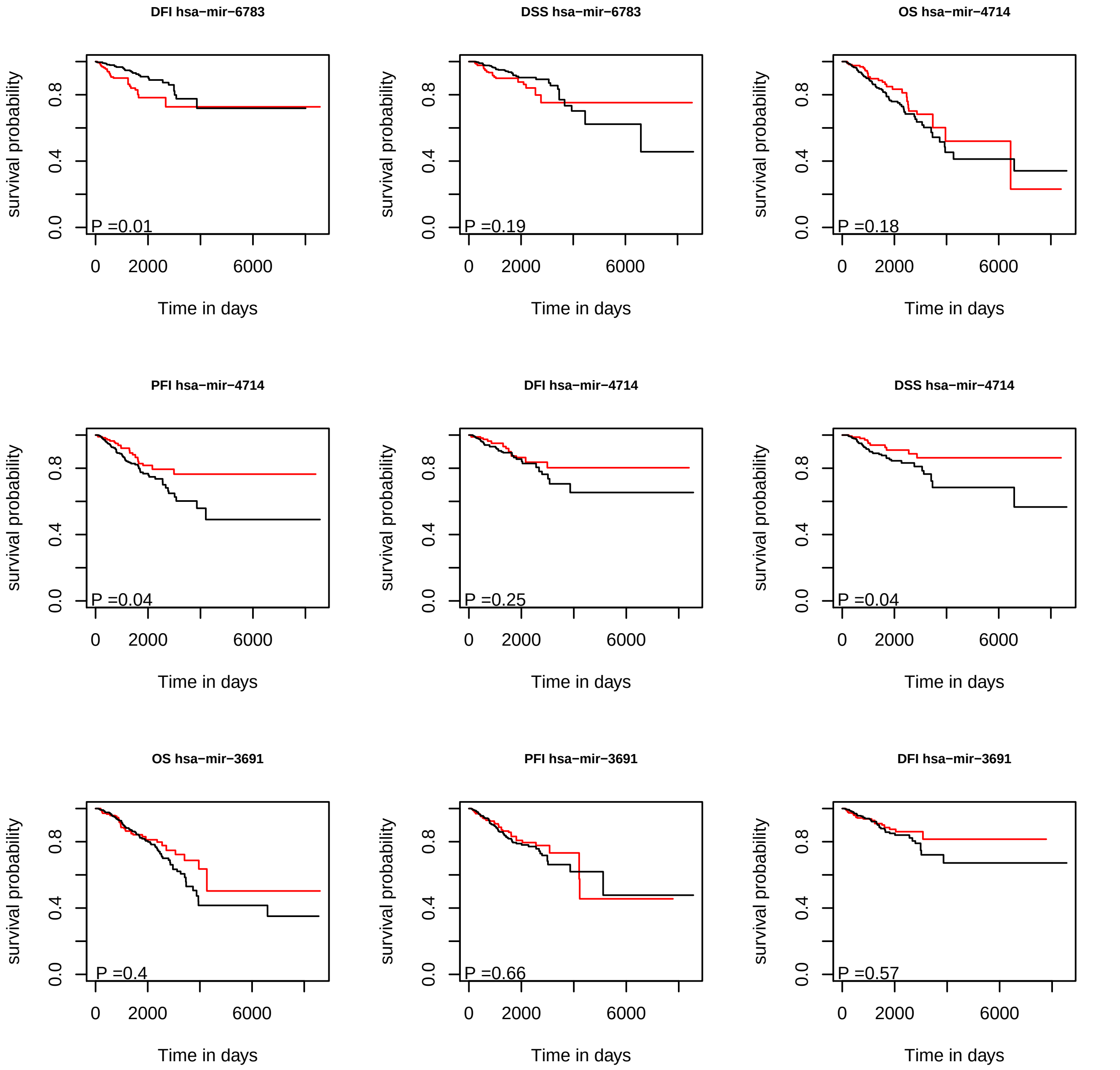
DFI hsa−mir−6783
P =0.01
DSS hsa−mir−6783
P =0.19
OS hsa−mir−4714
P =0.18
PFI hsa−mir−4714
P =0.04
DFI hsa−mir−4714
P =0.25
DSS hsa−mir−4714
P =0.04
OS hsa−mir−3691
P =0.4
PFI hsa−mir−3691
P =0.66
DFI hsa−mir−3691
P =0.57
survival probability
0.0
0.4
0.8
Time in days
0
2000
6000

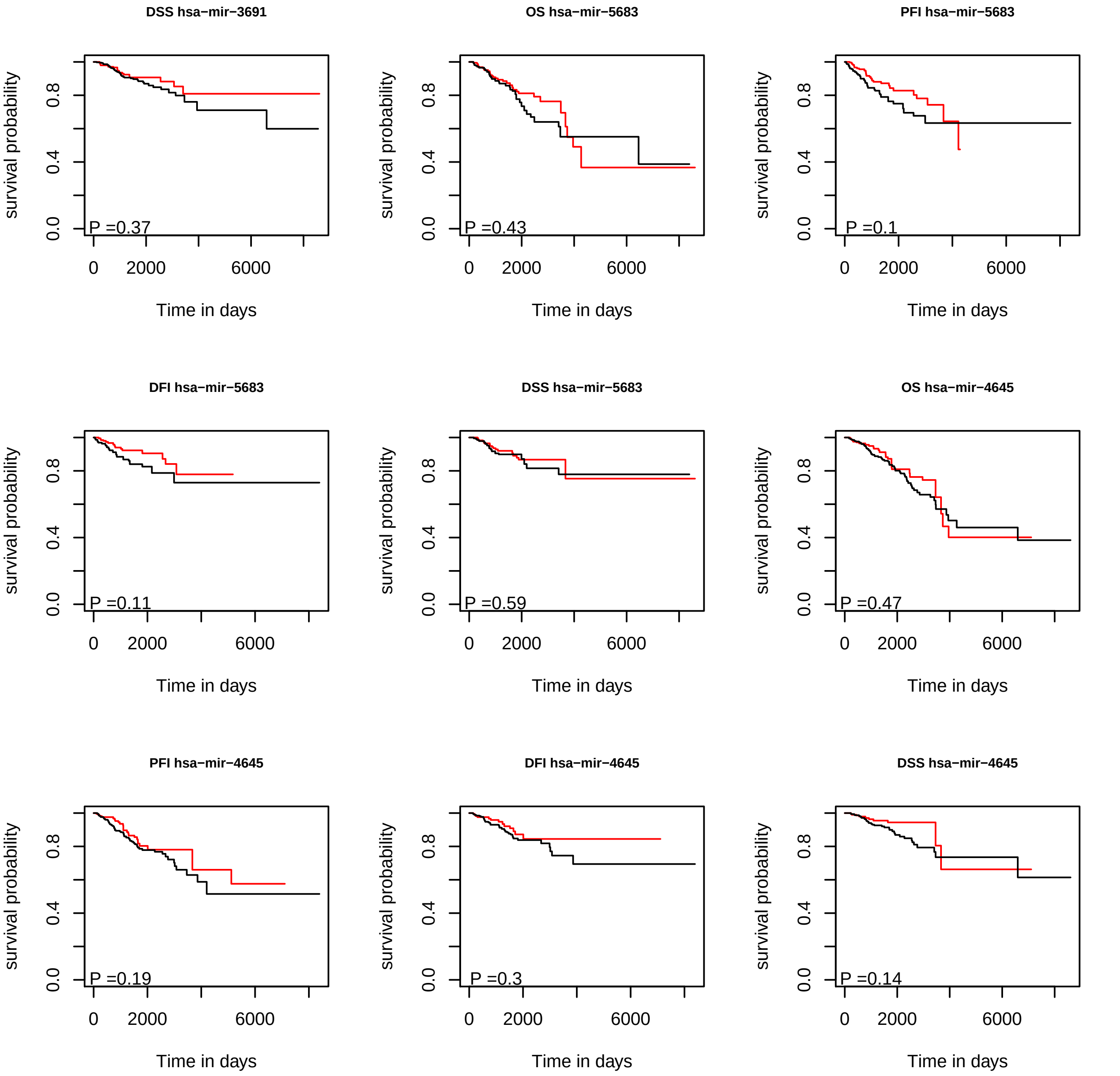
DSS hsa−mir−3691
P =0.37
OS hsa−mir−5683
P =0.43
PFI hsa−mir−5683
P =0.1
DFI hsa−mir−5683
P =0.11
DSS hsa−mir−5683
P =0.59
OS hsa−mir−4645
P =0.47
PFI hsa−mir−4645
P =0.19
DFI hsa−mir−4645
P =0.3
DSS hsa−mir−4645
P =0.14
survival probability
0.0
0.4
0.8
Time in days
0
2000
6000

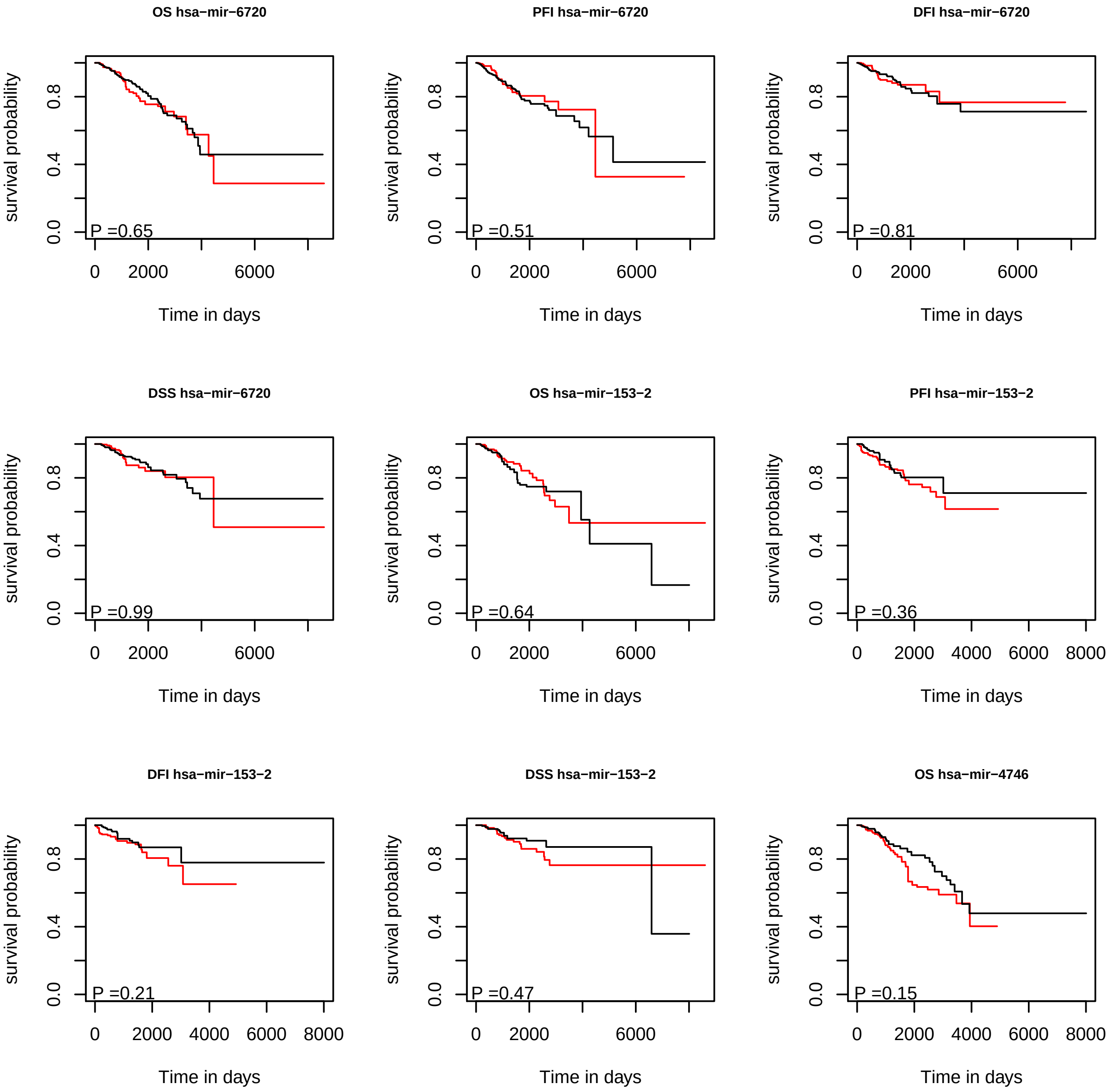
OS hsa−mir−6720
P =0.65
PFI hsa−mir−6720
P =0.51
DFI hsa−mir−6720
P =0.81
DSS hsa−mir−6720
P =0.99
OS hsa−mir−153−2
P =0.64
PFI hsa−mir−153−2
P =0.36
DFI hsa−mir−153−2
P =0.21
DSS hsa−mir−153−2
P =0.47
OS hsa−mir−4746
P =0.15
survival probability
Time in days
0.0
0.4
0.8
0
2000
4000
6000
8000

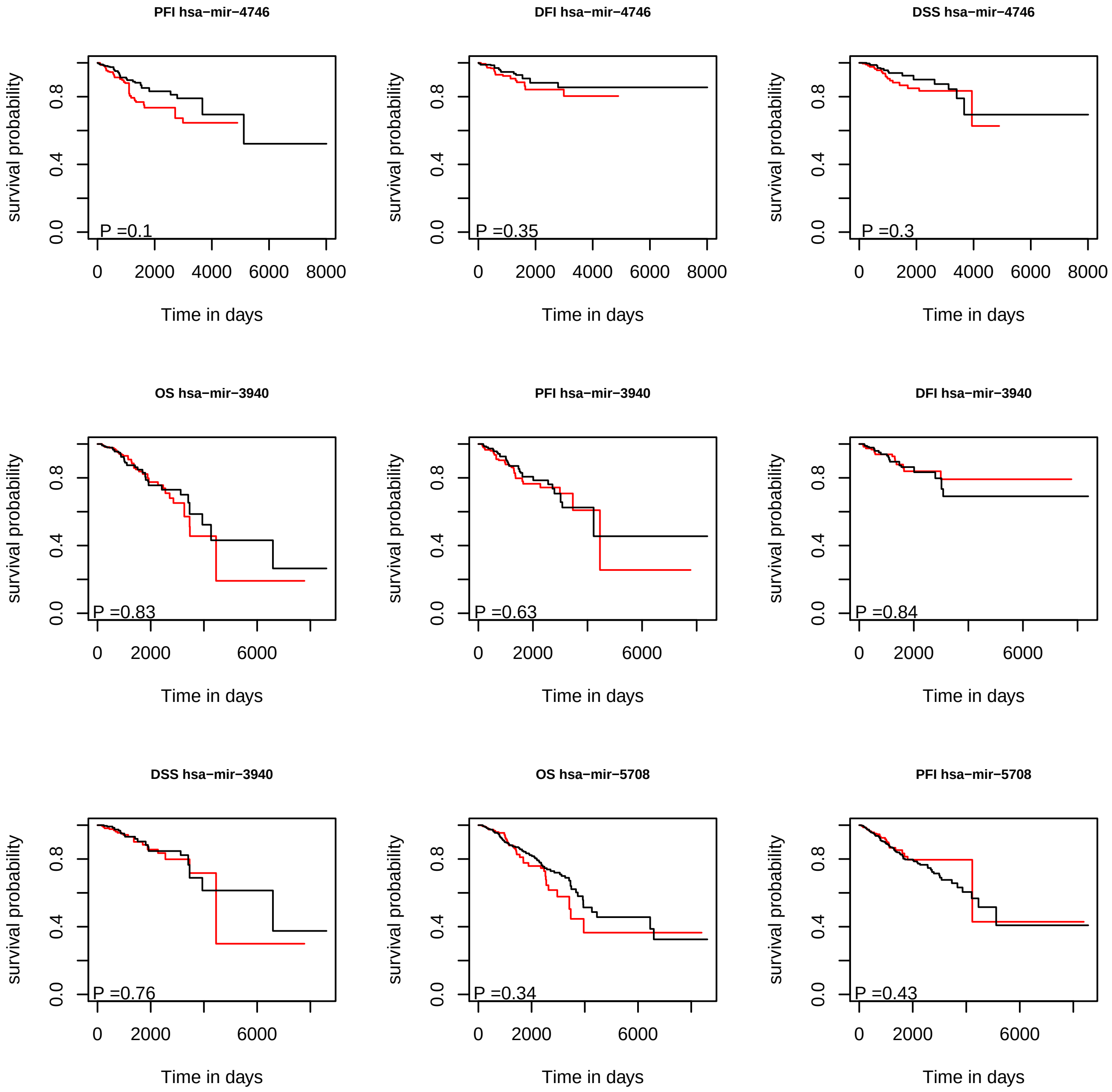

PFI hsa−mir−4746
P =0.1
DFI hsa−mir−4746
P =0.35
DSS hsa−mir−4746
P =0.3
OS hsa−mir−3940
P =0.83
PFI hsa−mir−3940
P =0.63
DFI hsa−mir−3940
P =0.84
DSS hsa−mir−3940
P =0.76
OS hsa−mir−5708
P =0.34
PFI hsa−mir−5708
P =0.43
survival probability
Time in days
0.0
0.4
0.8
0
2000
4000
6000
8000

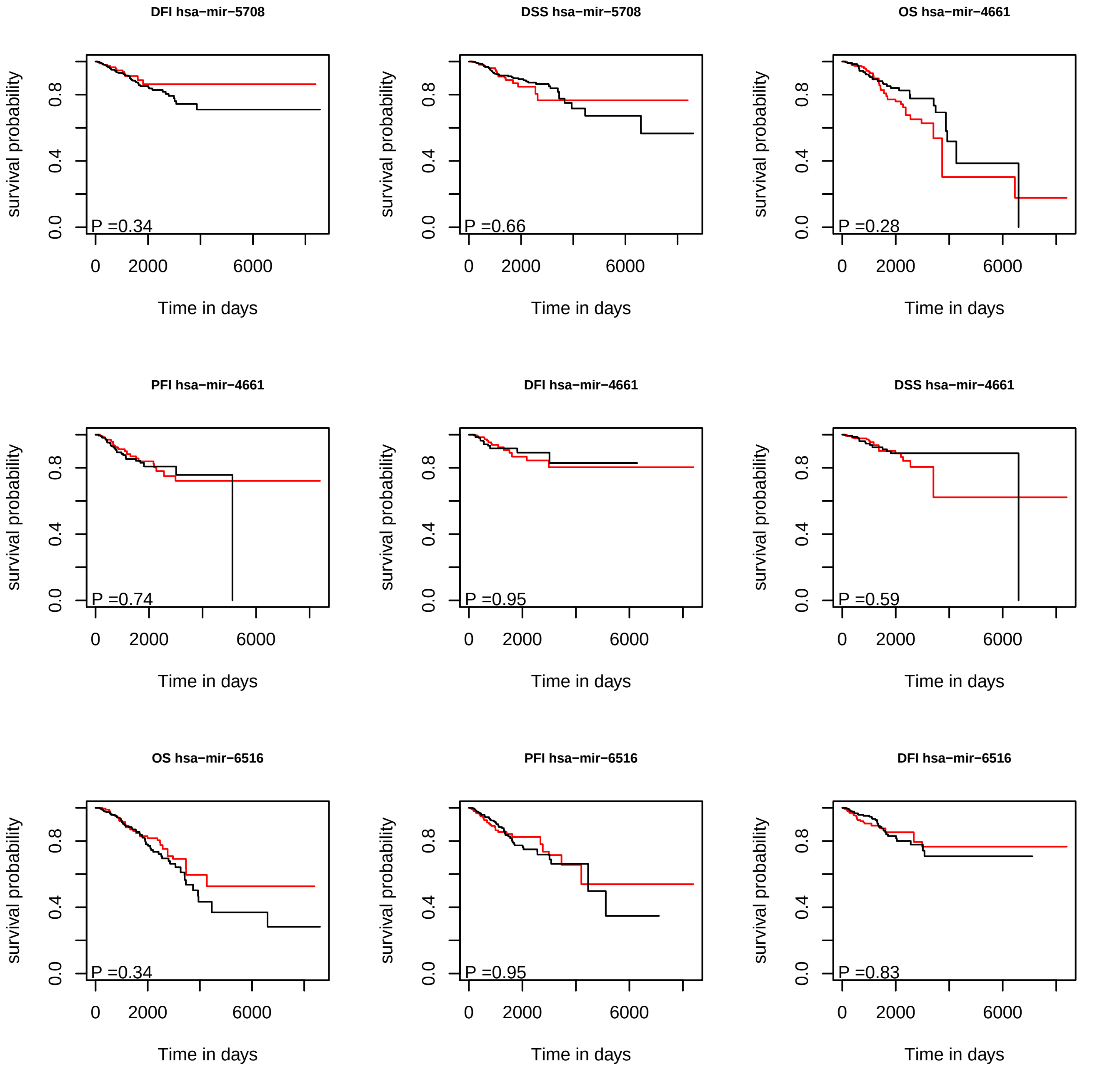

DFI hsa−mir−5708
P =0.34
DSS hsa−mir−5708
P =0.66
OS hsa−mir−4661
P =0.28
PFI hsa−mir−4661
P =0.74
DFI hsa−mir−4661
P =0.95
DSS hsa−mir−4661
P =0.59
OS hsa−mir−6516
P =0.34
PFI hsa−mir−6516
P =0.95
DFI hsa−mir−6516
P =0.83
survival probability
Time in days
0.0
0.4
0.8
0
2000
6000

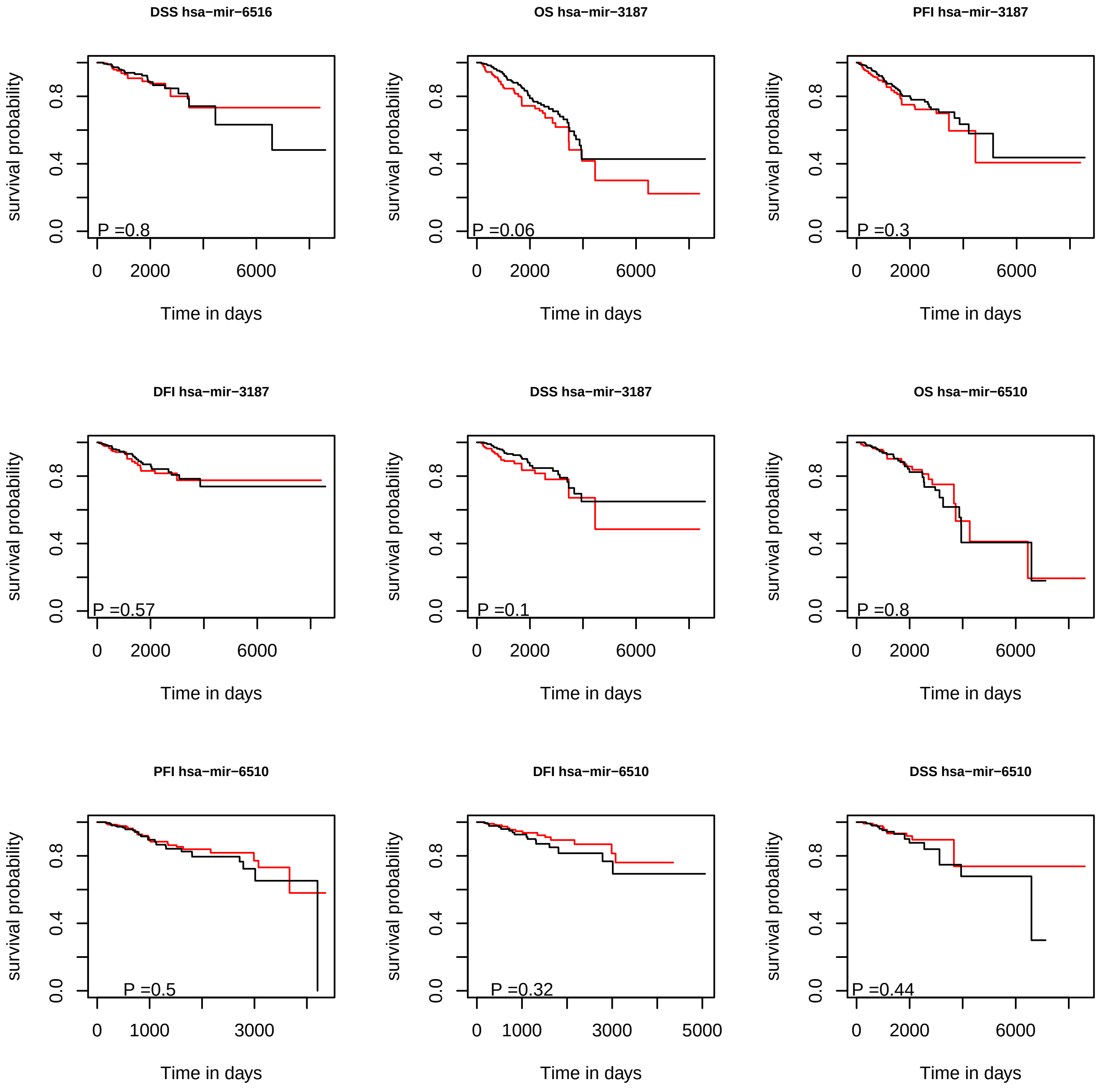
DSS hsa−mir−6516
survival probability
0.0
0.4
0.8
0
2000
6000
Time in days
P =0.8
OS hsa−mir−3187
survival probability
0.0
0.4
0.8
0
2000
6000
Time in days
P =0.06
PFI hsa−mir−3187
survival probability
0.0
0.4
0.8
0
2000
6000
Time in days
P =0.3
DFI hsa−mir−3187
survival probability
0.0
0.4
0.8
0
2000
6000
Time in days
P =0.57
DSS hsa−mir−3187
survival probability
0.0
0.4
0.8
0
2000
6000
Time in days
P =0.1
OS hsa−mir−6510
survival probability
0.0
0.4
0.8
0
2000
6000
Time in days
P =0.8
PFI hsa−mir−6510
survival probability
0.0
0.4
0.8
0
1000
3000
Time in days
P =0.5
DFI hsa−mir−6510
survival probability
0.0
0.4
0.8
0
1000
3000
5000
Time in days
P =0.32
DSS hsa−mir−6510
survival probability
0.0
0.4
0.8
0
2000
6000
Time in days
P =0.44

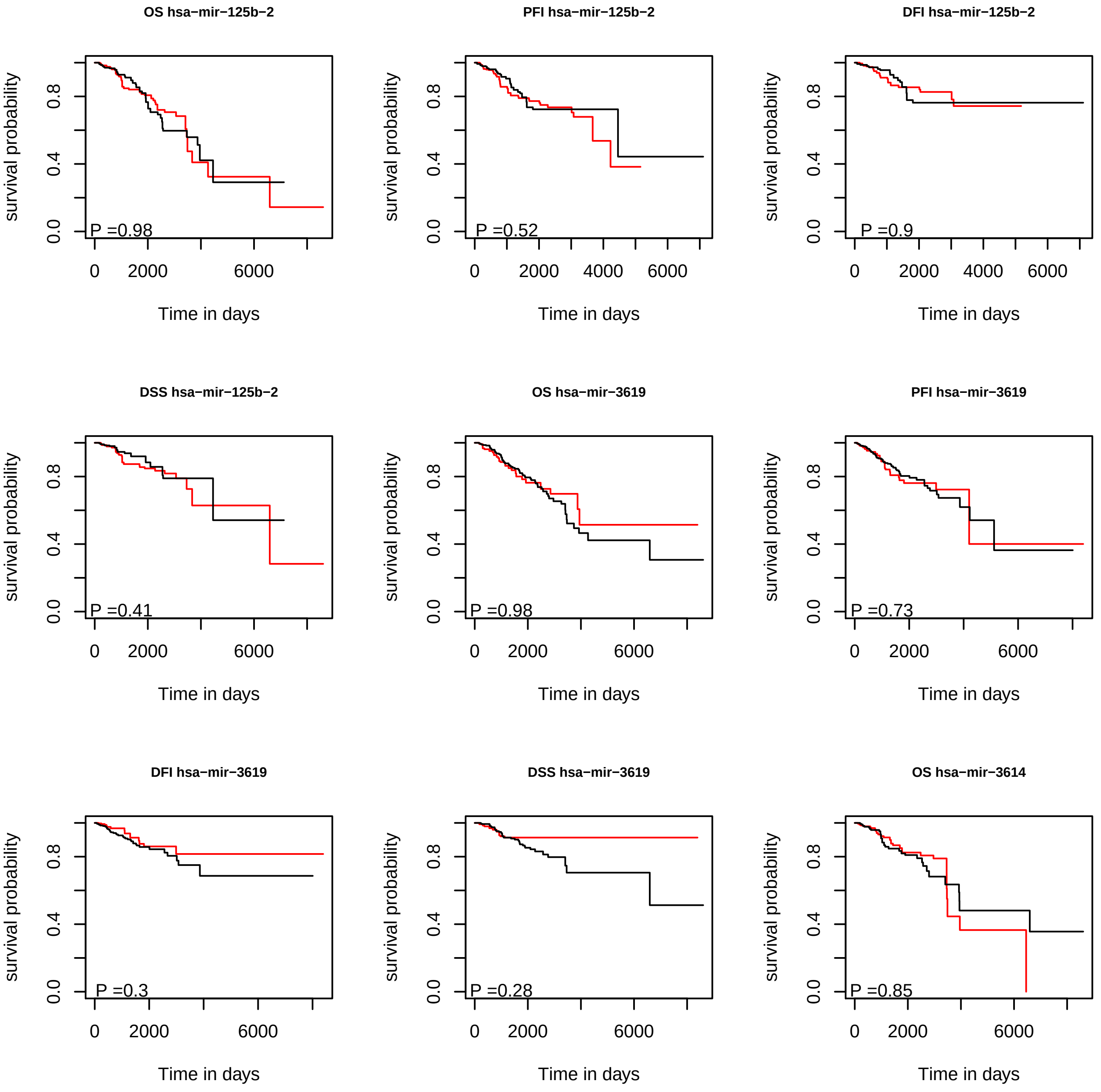
OS hsa−mir−125b−2
P =0.98
PFI hsa−mir−125b−2
P =0.52
DFI hsa−mir−125b−2
P =0.9
DSS hsa−mir−125b−2
P =0.41
OS hsa−mir−3619
P =0.98
PFI hsa−mir−3619
P =0.73
DFI hsa−mir−3619
P =0.3
DSS hsa−mir−3619
P =0.28
OS hsa−mir−3614
P =0.85
survival probability
Time in days
0.0
0.4
0.8
0
2000
4000
6000

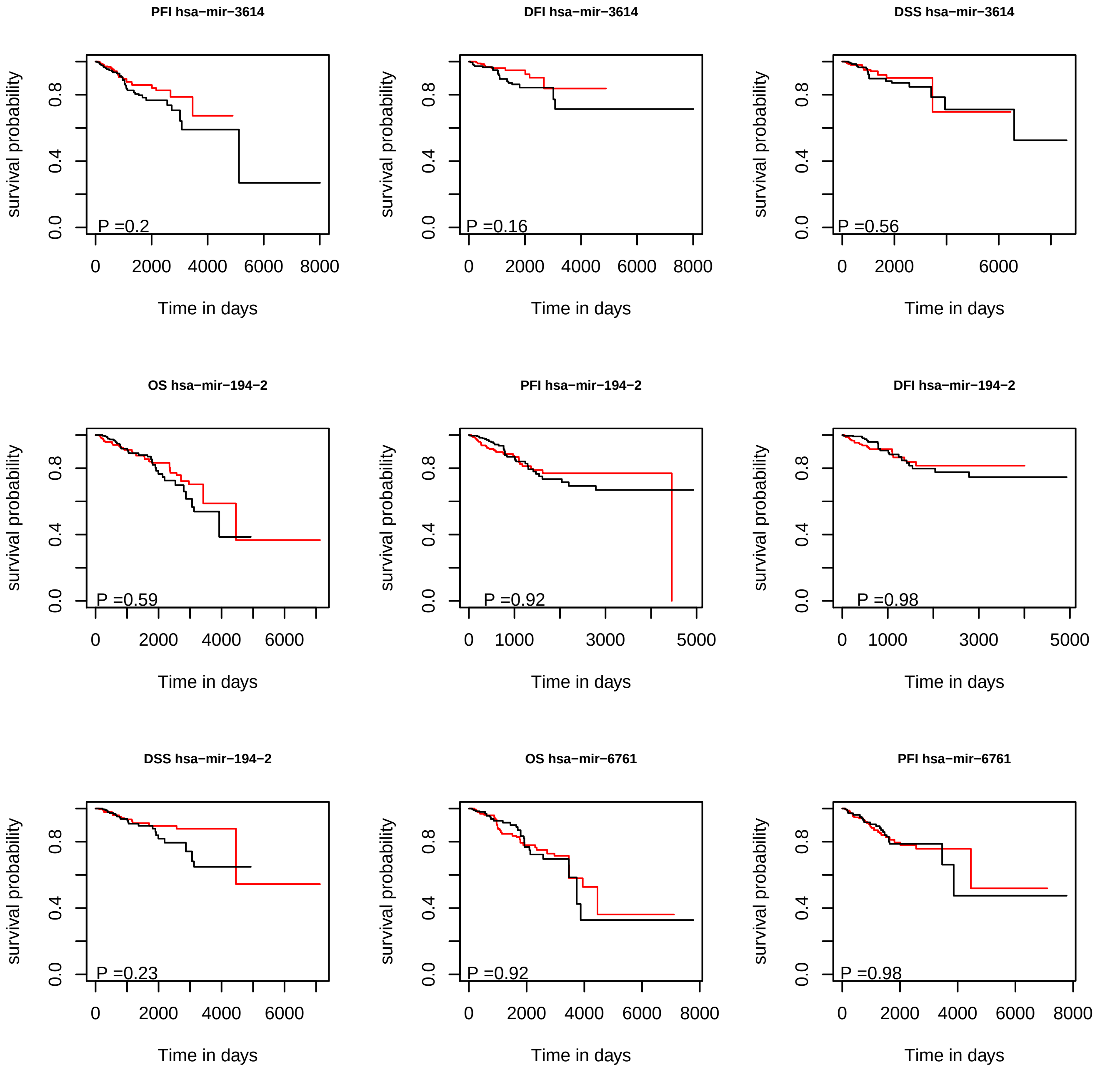
PFI hsa−mir−3614
P =0.2
DFI hsa−mir−3614
P =0.16
DSS hsa−mir−3614
P =0.56
OS hsa−mir−194−2
P =0.59
PFI hsa−mir−194−2
P =0.92
DFI hsa−mir−194−2
P =0.98
DSS hsa−mir−194−2
P =0.23
OS hsa−mir−6761
P =0.92
PFI hsa−mir−6761
P =0.98
survival probability
Time in days

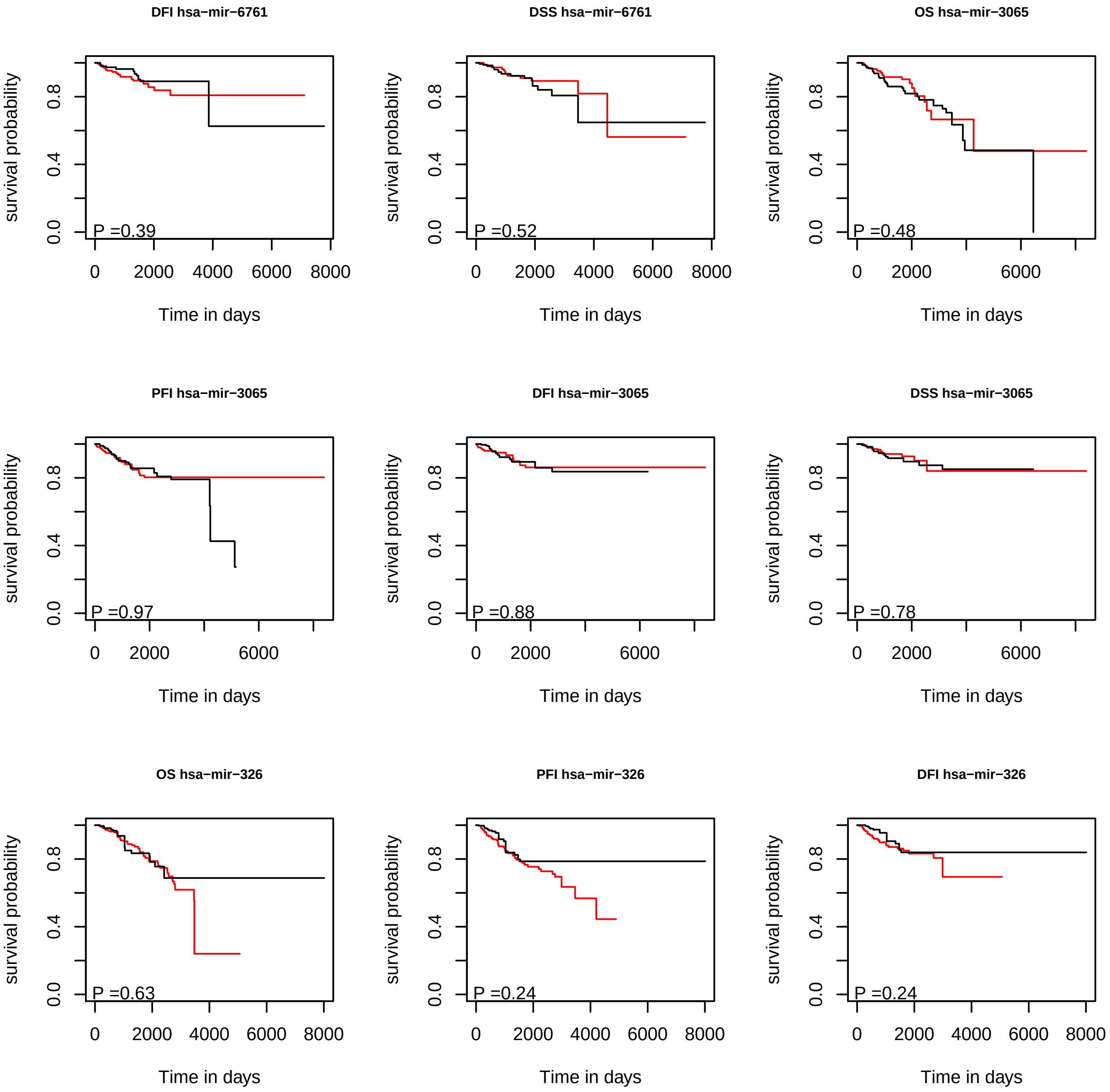

DFI hsa−mir−6761
P =0.39
DSS hsa−mir−6761
P =0.52
OS hsa−mir−3065
P =0.48
PFI hsa−mir−3065
P =0.97
DFI hsa−mir−3065
P =0.88
DSS hsa−mir−3065
P =0.78
OS hsa−mir−326
P =0.63
PFI hsa−mir−326
P =0.24
DFI hsa−mir−326
P =0.24
survival probability
0.0
0.4
0.8
Time in days
0
2000
4000
6000
8000

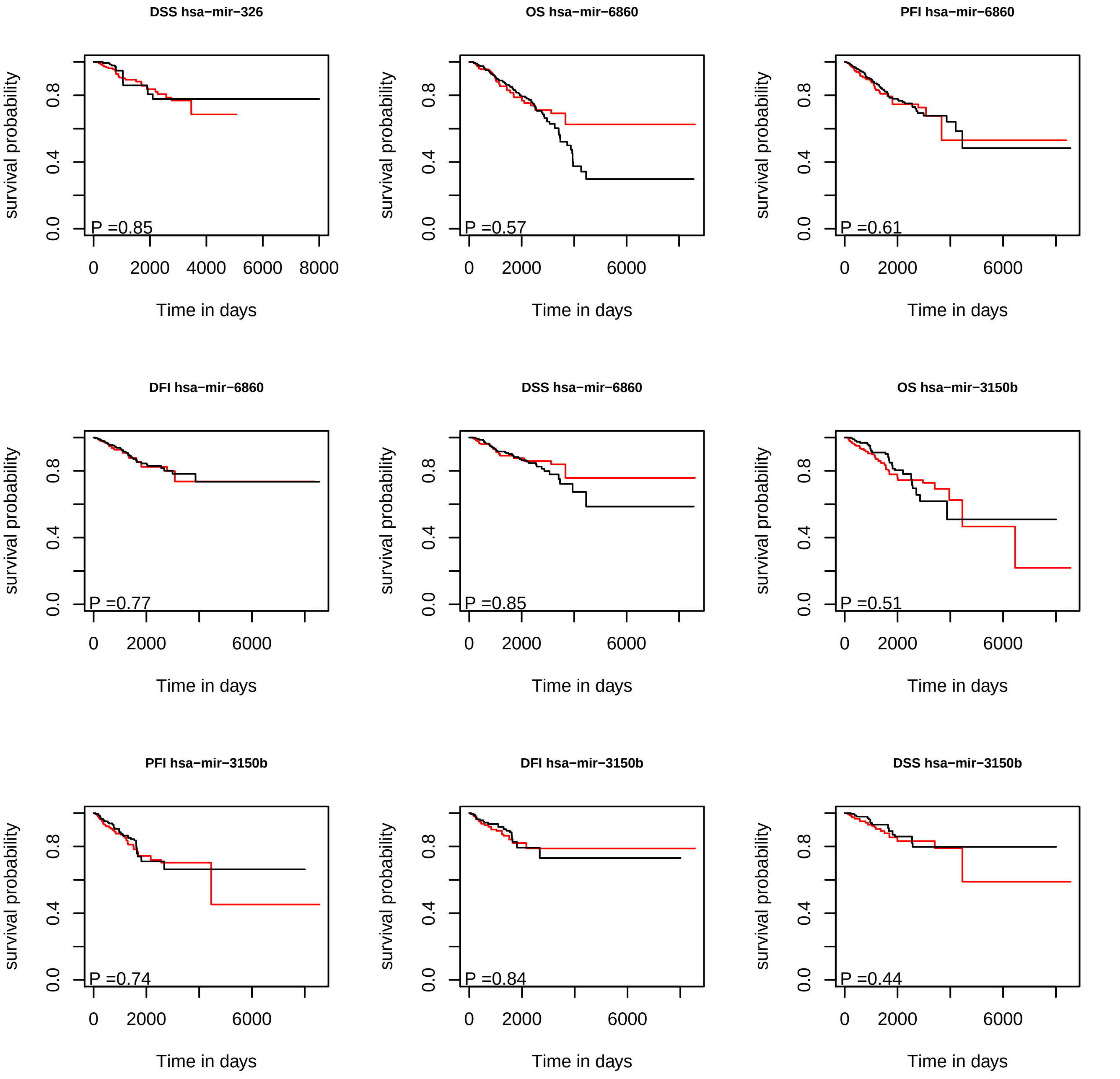

DSS hsa−mir−326
P =0.85
OS hsa−mir−6860
P =0.57
PFI hsa−mir−6860
P =0.61
DFI hsa−mir−6860
P =0.77
DSS hsa−mir−6860
P =0.85
OS hsa−mir−3150b
P =0.51
PFI hsa−mir−3150b
P =0.74
DFI hsa−mir−3150b
P =0.84
DSS hsa−mir−3150b
P =0.44
survival probability
0.0
0.4
0.8
Time in days
0
2000
4000
6000
8000

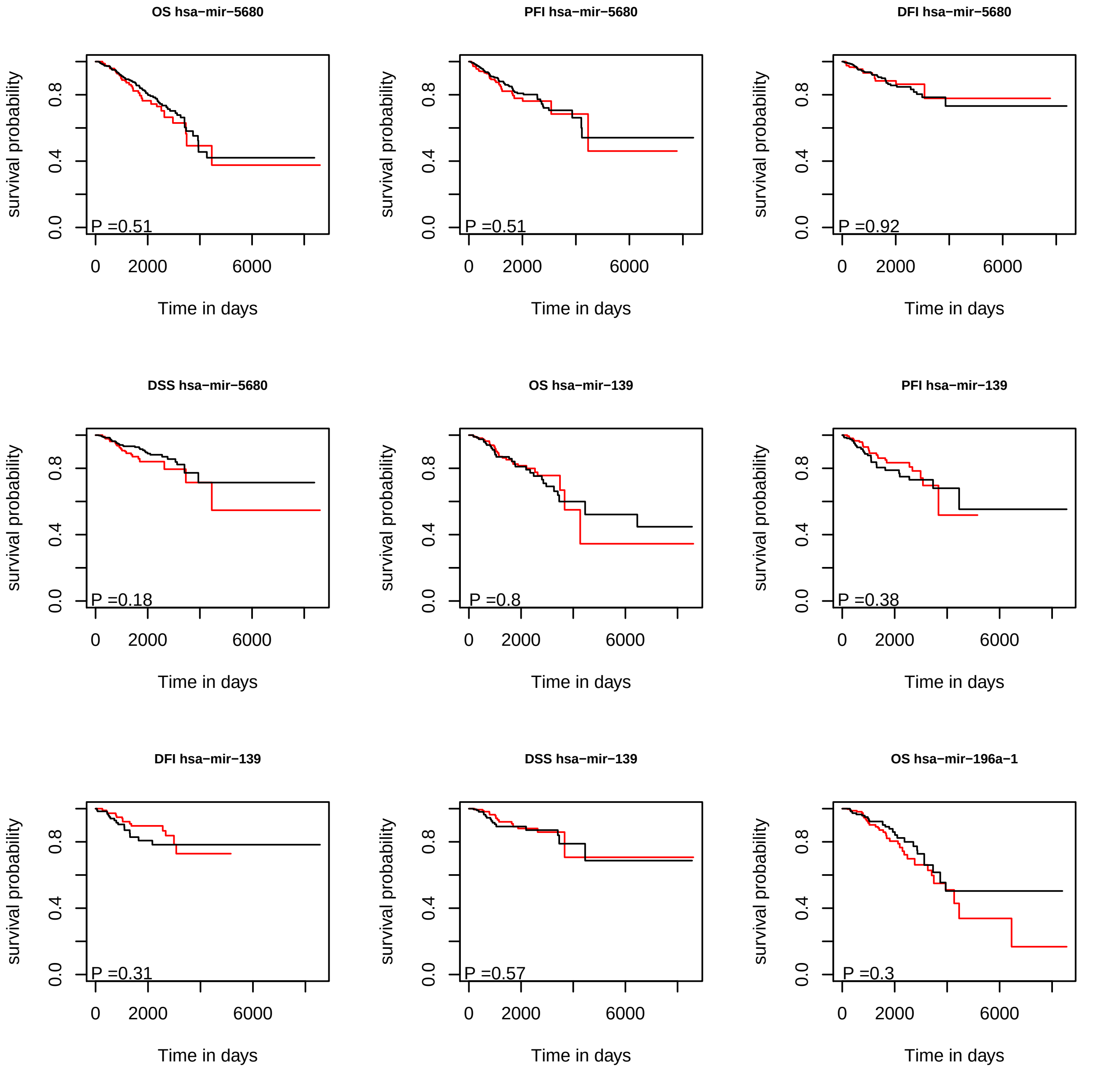
OS hsa−mir−5680
survival probability
0.0
0.4
0.8
P =0.51
0
2000
6000
Time in days
PFI hsa−mir−5680
survival probability
0.0
0.4
0.8
P =0.51
0
2000
6000
Time in days
DFI hsa−mir−5680
survival probability
0.0
0.4
0.8
P =0.92
0
2000
6000
Time in days
DSS hsa−mir−5680
survival probability
0.0
0.4
0.8
P =0.18
0
2000
6000
Time in days
OS hsa−mir−139
survival probability
0.0
0.4
0.8
P =0.8
0
2000
6000
Time in days
PFI hsa−mir−139
survival probability
0.0
0.4
0.8
P =0.38
0
2000
6000
Time in days
DFI hsa−mir−139
survival probability
0.0
0.4
0.8
P =0.31
0
2000
6000
Time in days
DSS hsa−mir−139
survival probability
0.0
0.4
0.8
P =0.57
0
2000
6000
Time in days
OS hsa−mir−196a−1
survival probability
0.0
0.4
0.8
P =0.3
0
2000
6000
Time in days

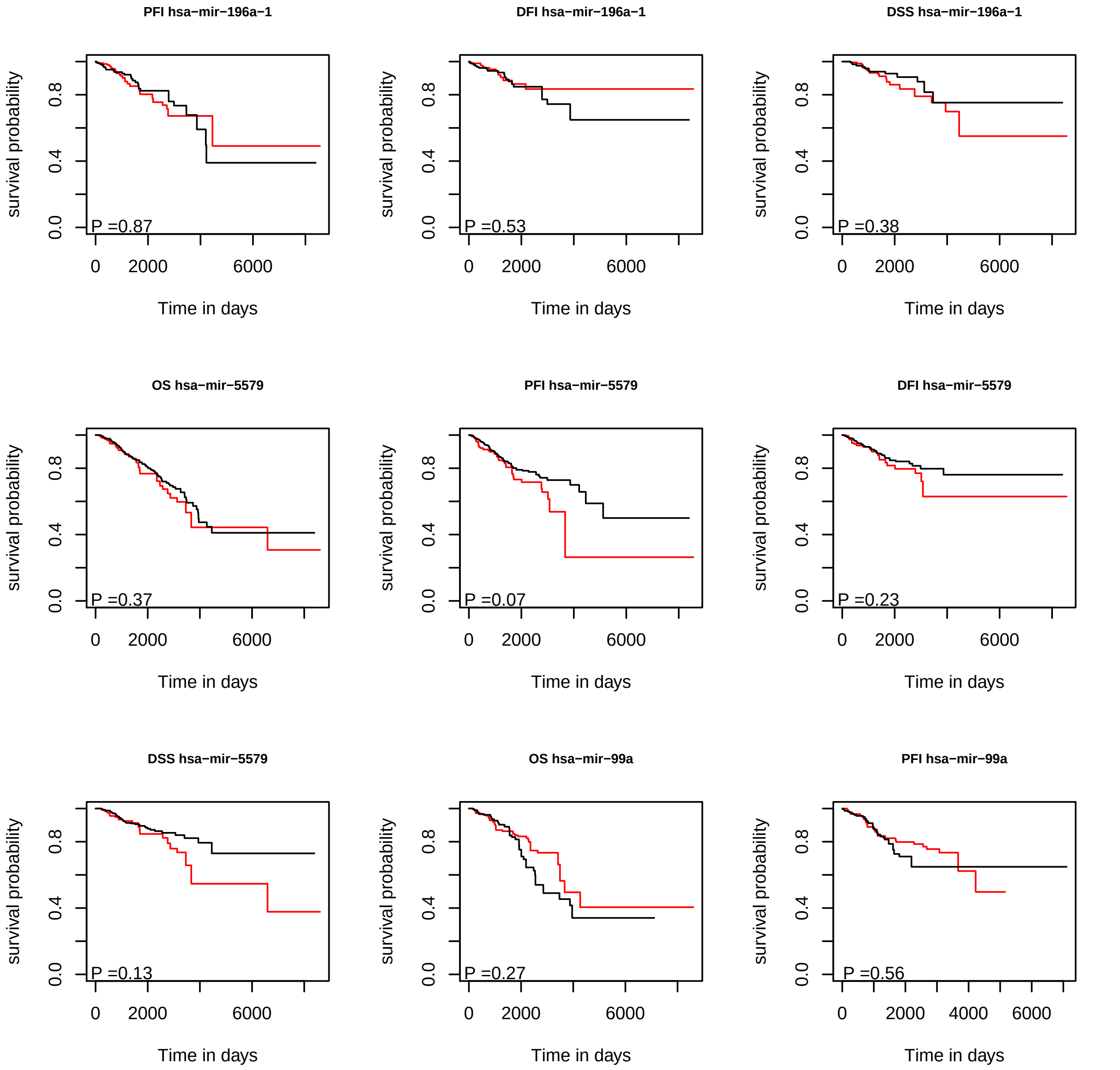

PFI hsa−mir−196a−1
DFI hsa−mir−196a−1
DSS hsa−mir−196a−1
OS hsa−mir−5579
PFI hsa−mir−5579
DFI hsa−mir−5579
DSS hsa−mir−5579
OS hsa−mir−99a
PFI hsa−mir−99a
P =0.87
P =0.53
P =0.38
P =0.37
P =0.07
P =0.23
P =0.13
P =0.27
P =0.56
survival probability
0.0
0.4
0.8
Time in days
0
2000
4000
6000

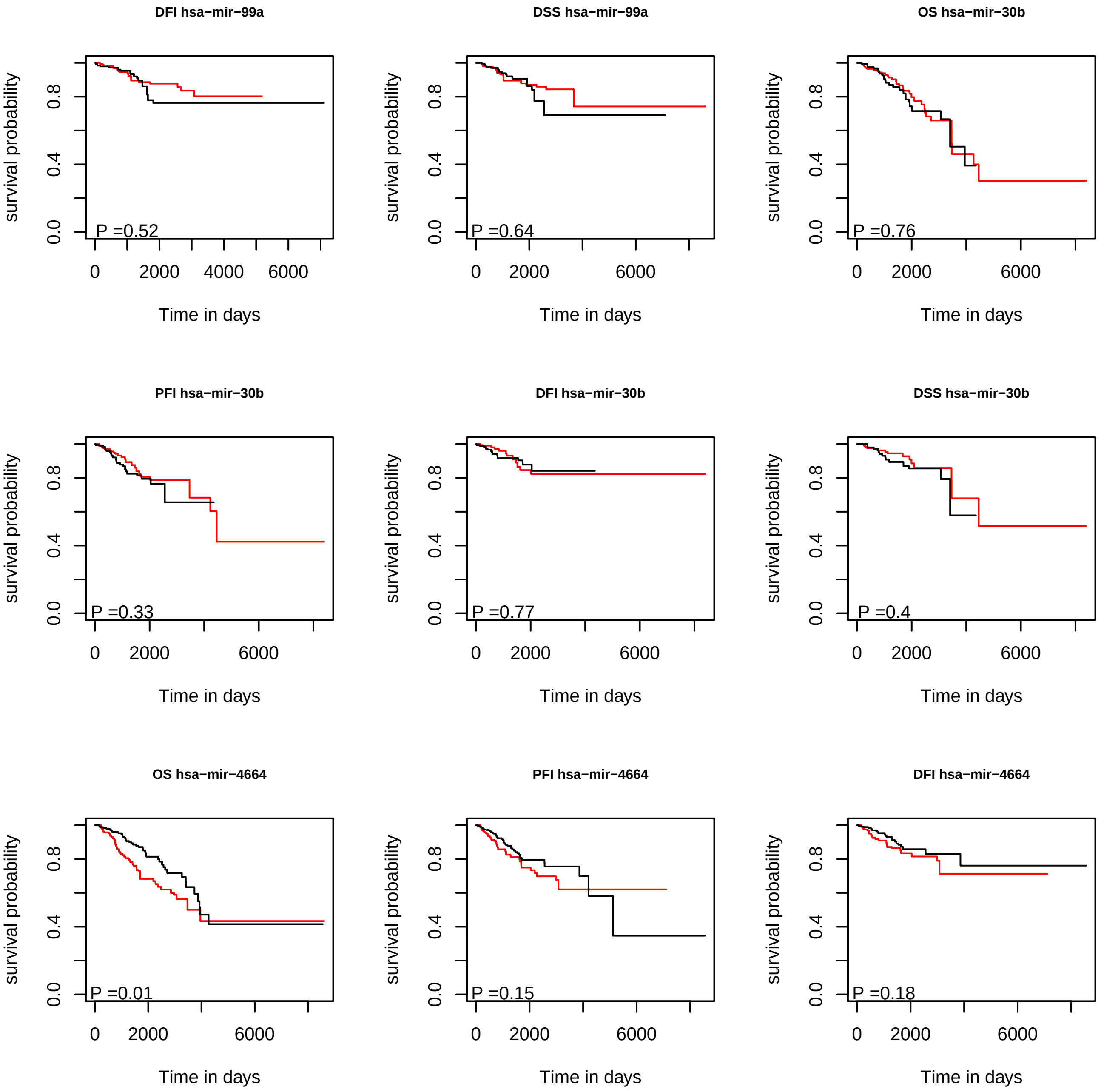
DFI hsa−mir−99a
P =0.52
DSS hsa−mir−99a
P =0.64
OS hsa−mir−30b
P =0.76
PFI hsa−mir−30b
P =0.33
DFI hsa−mir−30b
P =0.77
DSS hsa−mir−30b
P =0.4
OS hsa−mir−4664
P =0.01
PFI hsa−mir−4664
P =0.15
DFI hsa−mir−4664
P =0.18
survival probability
0.0
0.4
0.8
Time in days
0
2000
4000
6000

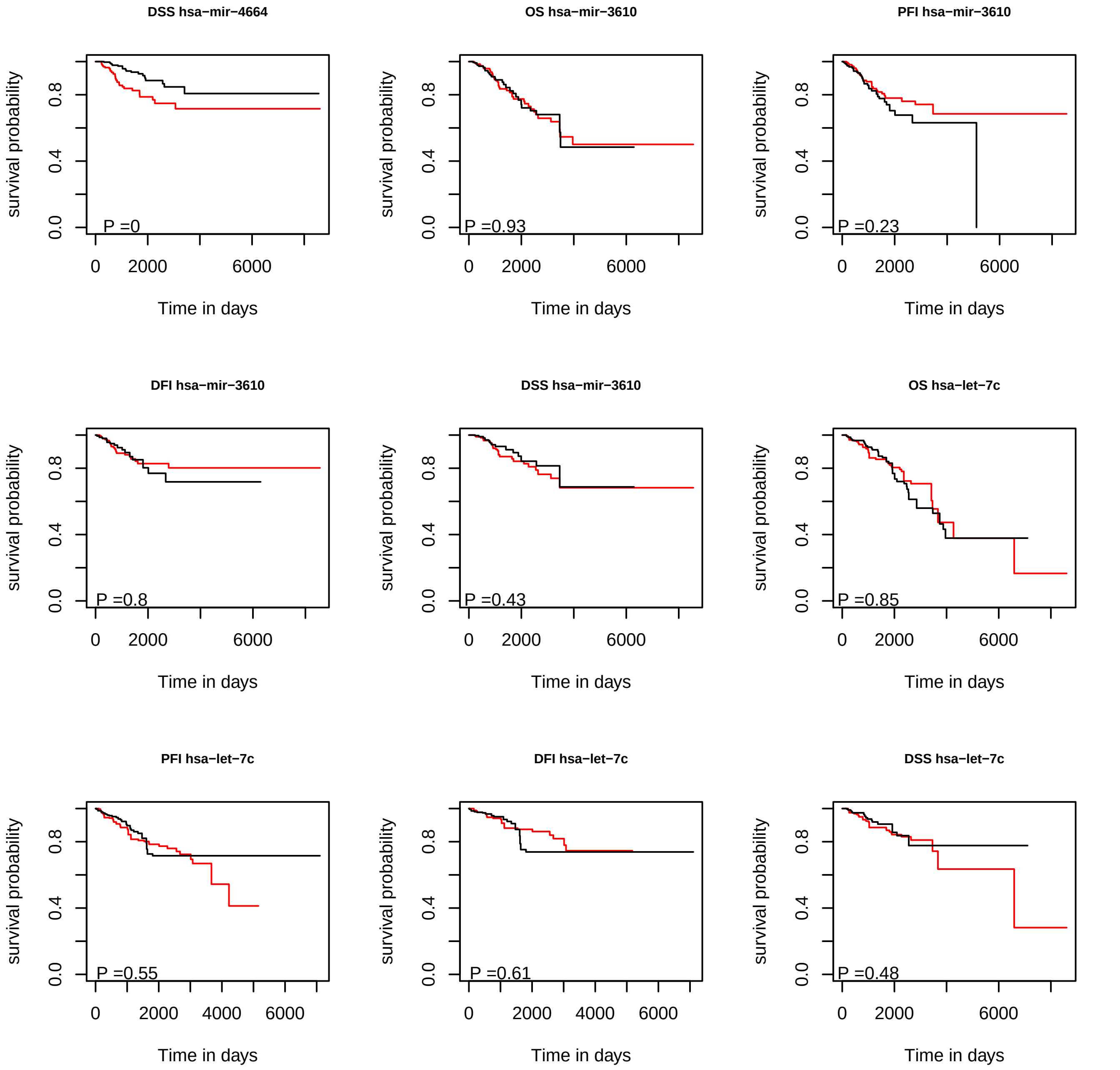
DSS hsa−mir−4664
P =0
OS hsa−mir−3610
P =0.93
PFI hsa−mir−3610
P =0.23
DFI hsa−mir−3610
P =0.8
DSS hsa−mir−3610
P =0.43
OS hsa−let−7c
P =0.85
PFI hsa−let−7c
P =0.55
DFI hsa−let−7c
P =0.61
DSS hsa−let−7c
P =0.48
survival probability
0.0
0.4
0.8
Time in days
0
2000
4000
6000

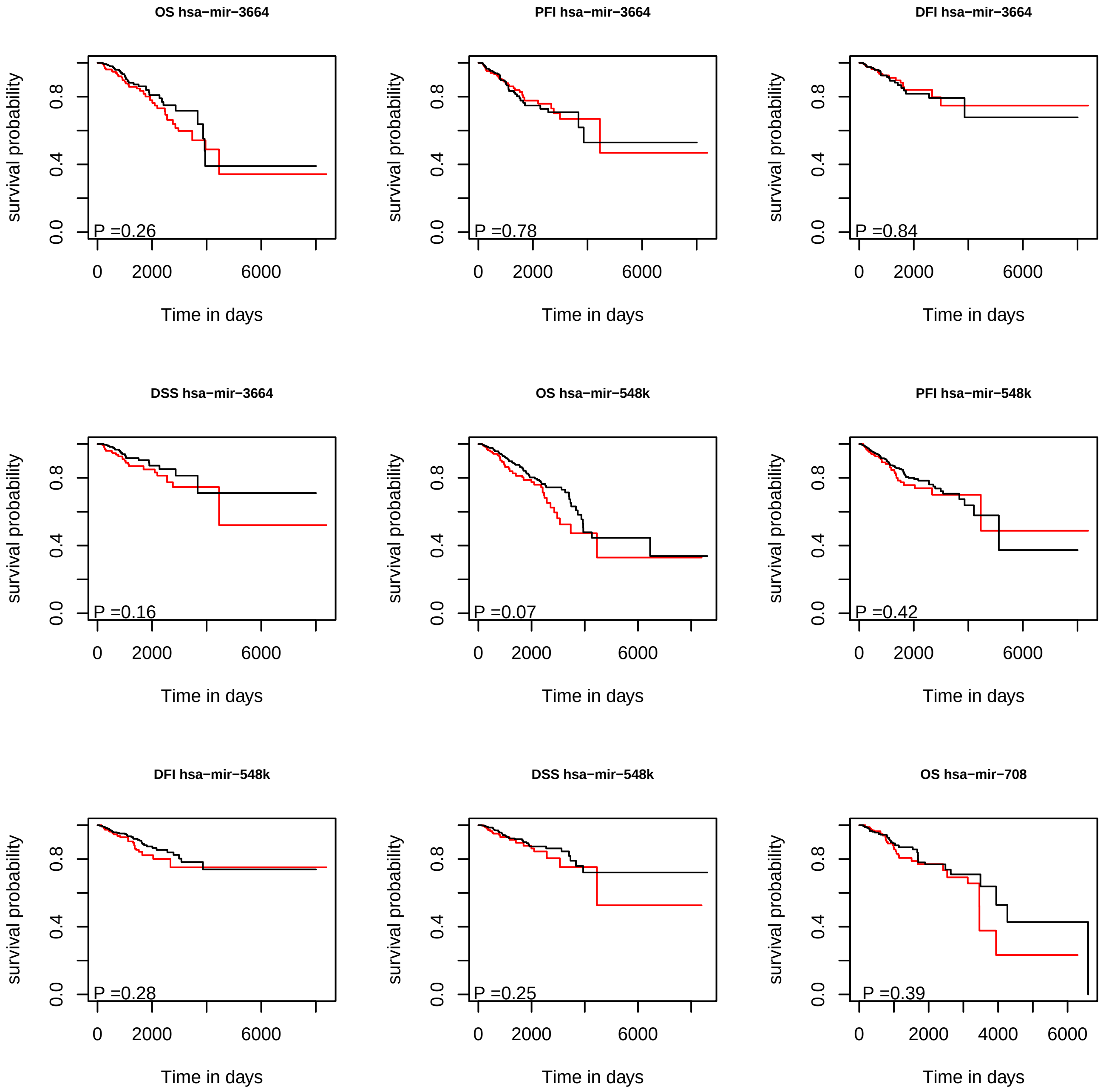

OS hsa−mir−3664
P =0.26
PFI hsa−mir−3664
P =0.78
DFI hsa−mir−3664
P =0.84
DSS hsa−mir−3664
P =0.16
OS hsa−mir−548k
P =0.07
PFI hsa−mir−548k
P =0.42
DFI hsa−mir−548k
P =0.28
DSS hsa−mir−548k
P =0.25
OS hsa−mir−708
P =0.39
survival probability
Time in days
0.0
0.4
0.8
0
2000
4000
6000

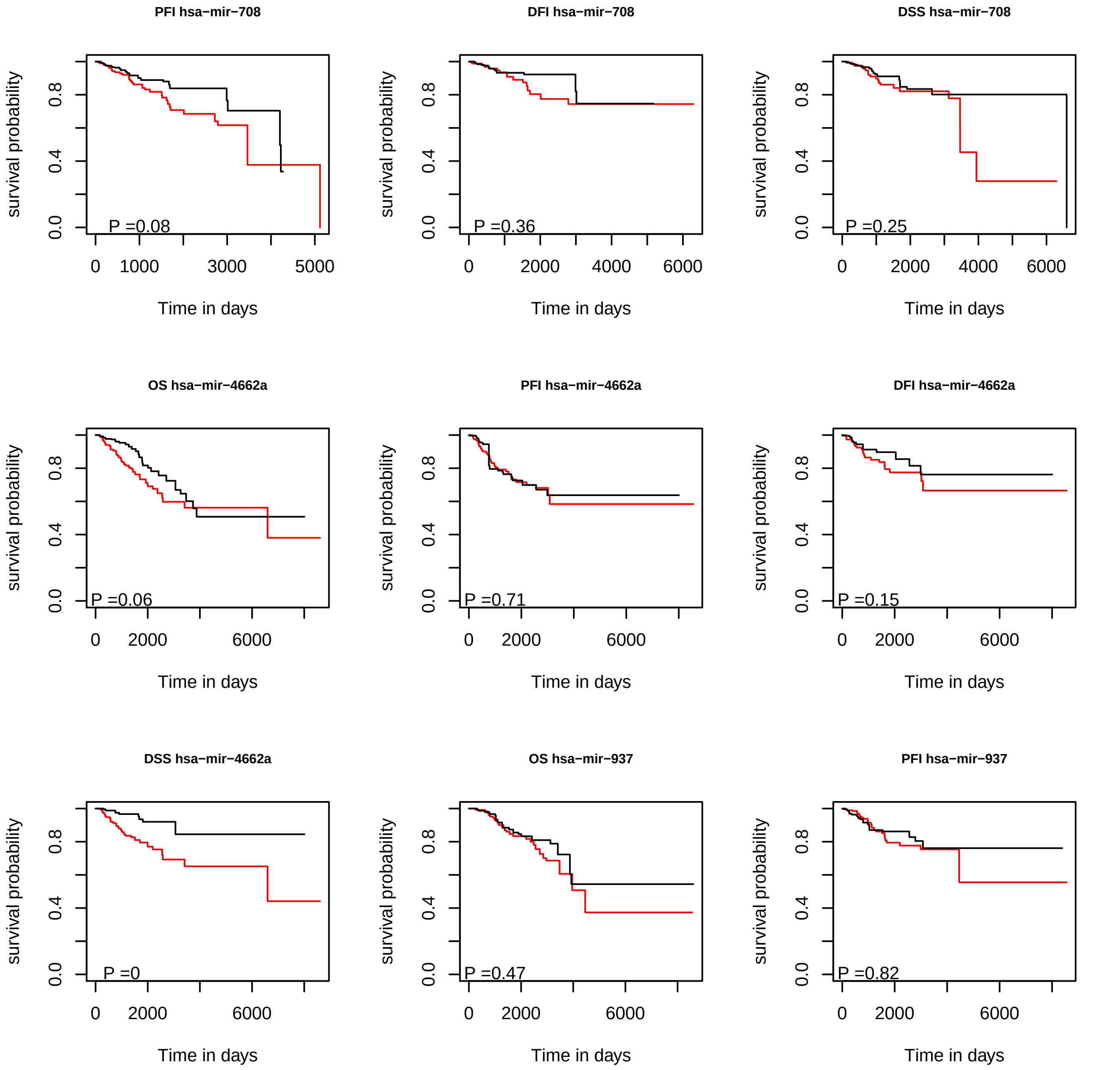
PFI hsa−mir−708
P =0.08
DFI hsa−mir−708
P =0.36
DSS hsa−mir−708
P =0.25
OS hsa−mir−4662a
P =0.06
PFI hsa−mir−4662a
P =0.71
DFI hsa−mir−4662a
P =0.15
DSS hsa−mir−4662a
P =0
OS hsa−mir−937
P =0.47
PFI hsa−mir−937
P =0.82
survival probability
Time in days

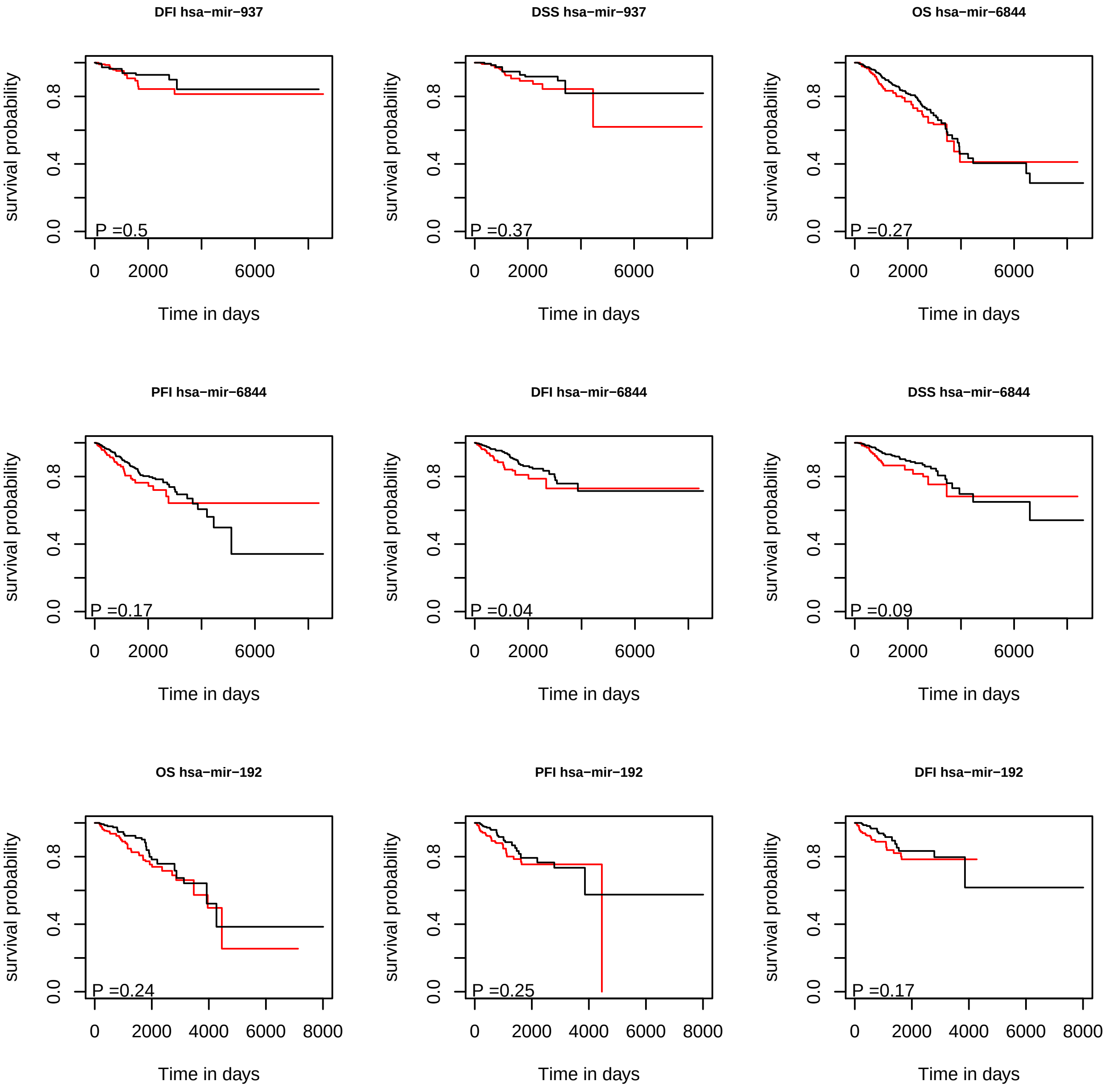
DFI hsa−mir−937
P =0.5
DSS hsa−mir−937
P =0.37
OS hsa−mir−6844
P =0.27
PFI hsa−mir−6844
P =0.17
DFI hsa−mir−6844
P =0.04
DSS hsa−mir−6844
P =0.09
OS hsa−mir−192
P =0.24
PFI hsa−mir−192
P =0.25
DFI hsa−mir−192
P =0.17
survival probability
0.0
0.4
0.8
Time in days
0
2000
4000
6000
8000

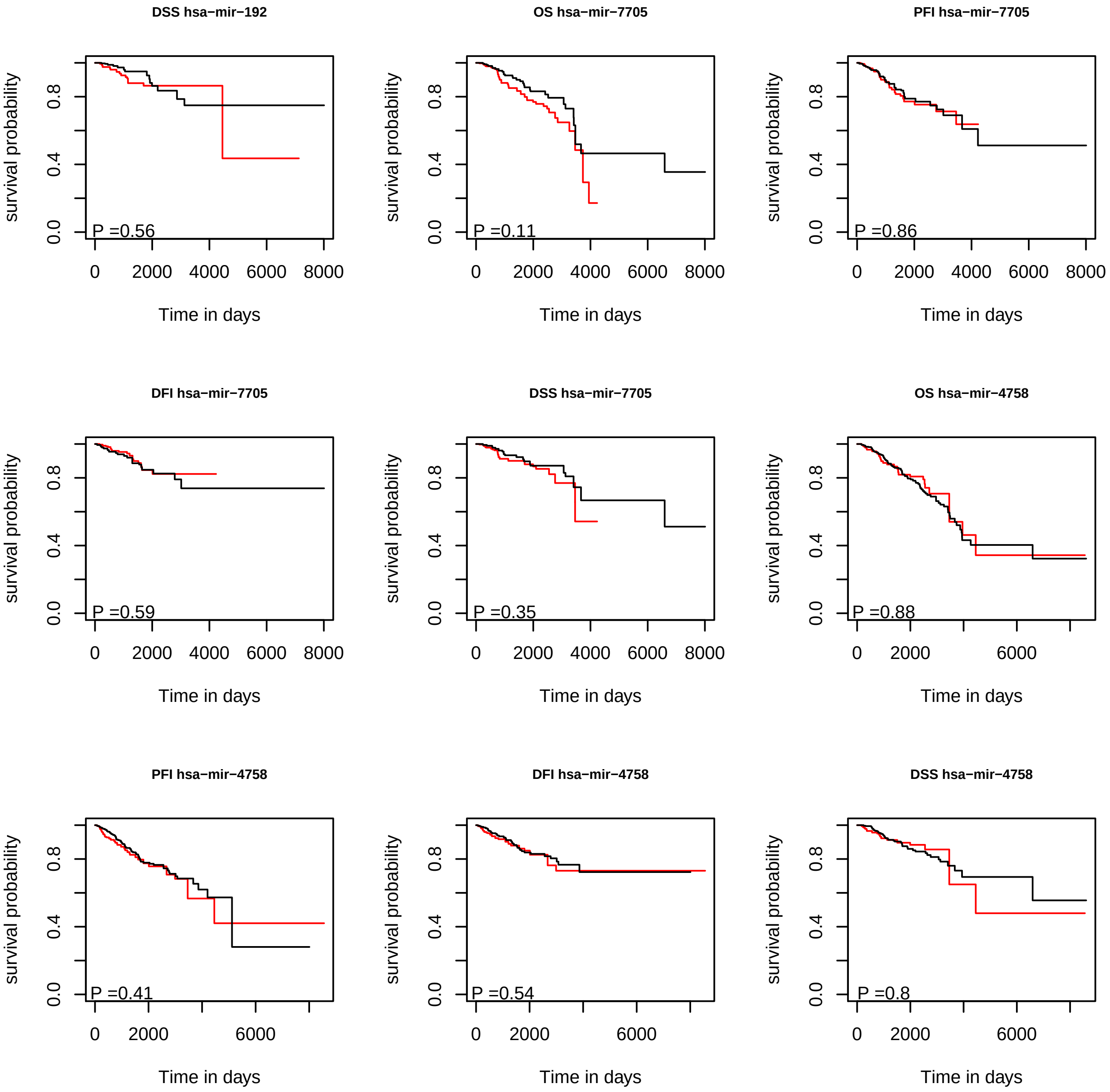
DSS hsa−mir−192
P =0.56
OS hsa−mir−7705
P =0.11
PFI hsa−mir−7705
P =0.86
DFI hsa−mir−7705
P =0.59
DSS hsa−mir−7705
P =0.35
OS hsa−mir−4758
P =0.88
PFI hsa−mir−4758
P =0.41
DFI hsa−mir−4758
P =0.54
DSS hsa−mir−4758
P =0.8
survival probability
Time in days
0.0
0.4
0.8
0
2000
4000
6000
8000

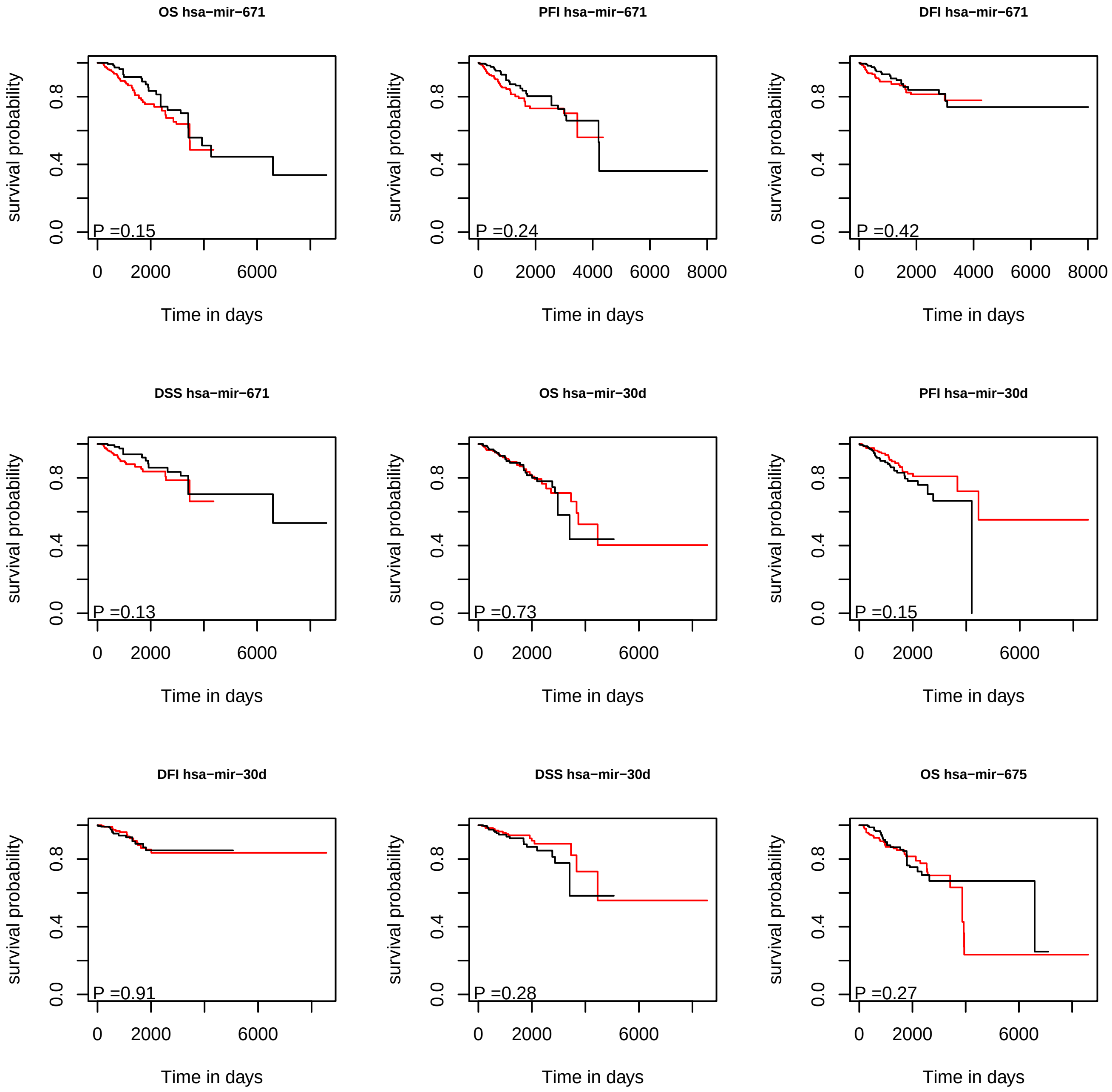

OS hsa−mir−671
P =0.15
PFI hsa−mir−671
P =0.24
DFI hsa−mir−671
P =0.42
DSS hsa−mir−671
P =0.13
OS hsa−mir−30d
P =0.73
PFI hsa−mir−30d
P =0.15
DFI hsa−mir−30d
P =0.91
DSS hsa−mir−30d
P =0.28
OS hsa−mir−675
P =0.27
survival probability
Time in days
0.0
0.4
0.8
0
2000
4000
6000
8000

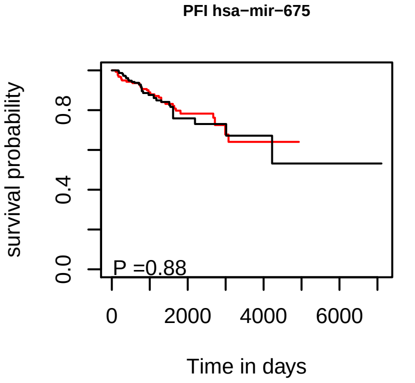
PFI hsa−mir−675
survival probability
0.0
0.4
0.8
P =0.88
0
2000
4000
6000
Time in days

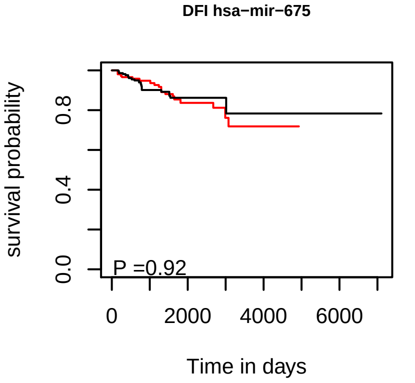
DFI hsa−mir−675
survival probability
0.0
0.4
0.8
P =0.92
0
2000
4000
6000
Time in days

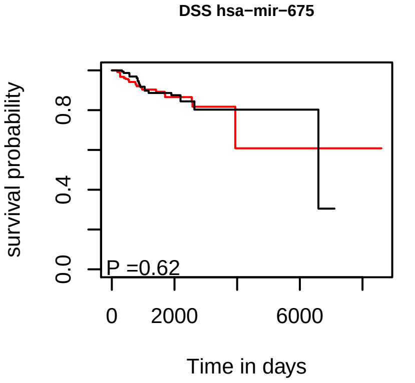
DSS hsa−mir−675
survival probability
0.0
0.4
0.8
P =0.62
0
2000
6000
Time in days

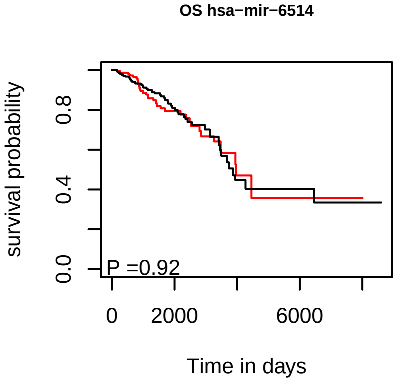
OS hsa−mir−6514
survival probability
0.0
0.4
0.8
P =0.92
0
2000
6000
Time in days

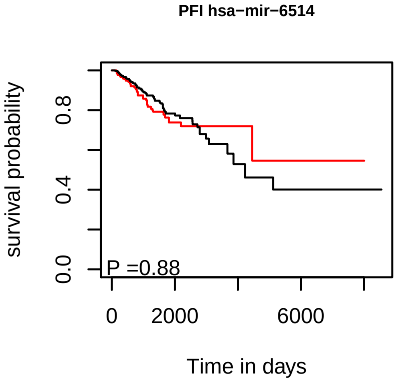
PFI hsa−mir−6514
survival probability
0.0
0.4
0.8
P =0.88
0
2000
6000
Time in days

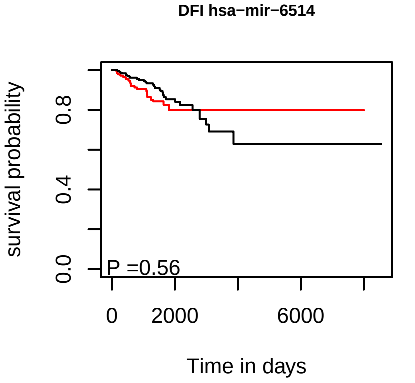
DFI hsa−mir−6514
survival probability
0.0
0.4
0.8
P =0.56
0
2000
6000
Time in days

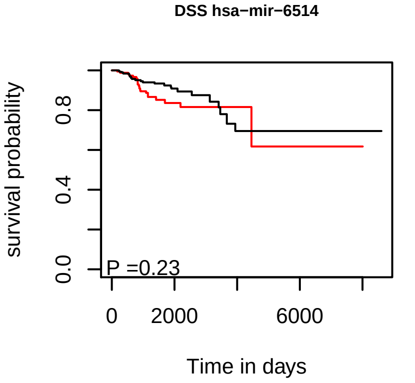
DSS hsa−mir−6514
survival probability
0.0
0.4
0.8
P =0.23
0
2000
6000
Time in days

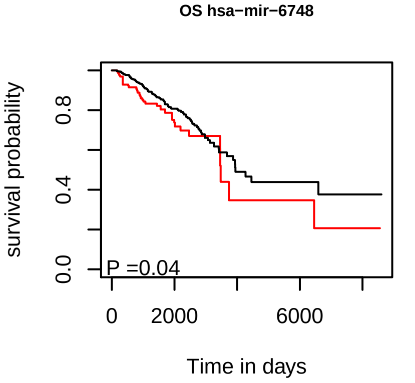
OS hsa−mir−6748
survival probability
0.0
0.4
0.8
P =0.04
0
2000
6000
Time in days

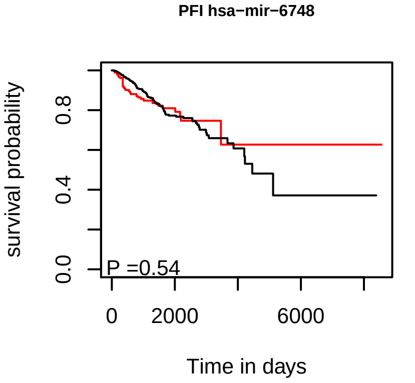
PFI hsa−mir−6748
survival probability
0.0
0.4
0.8
P =0.54
0
2000
6000
Time in days

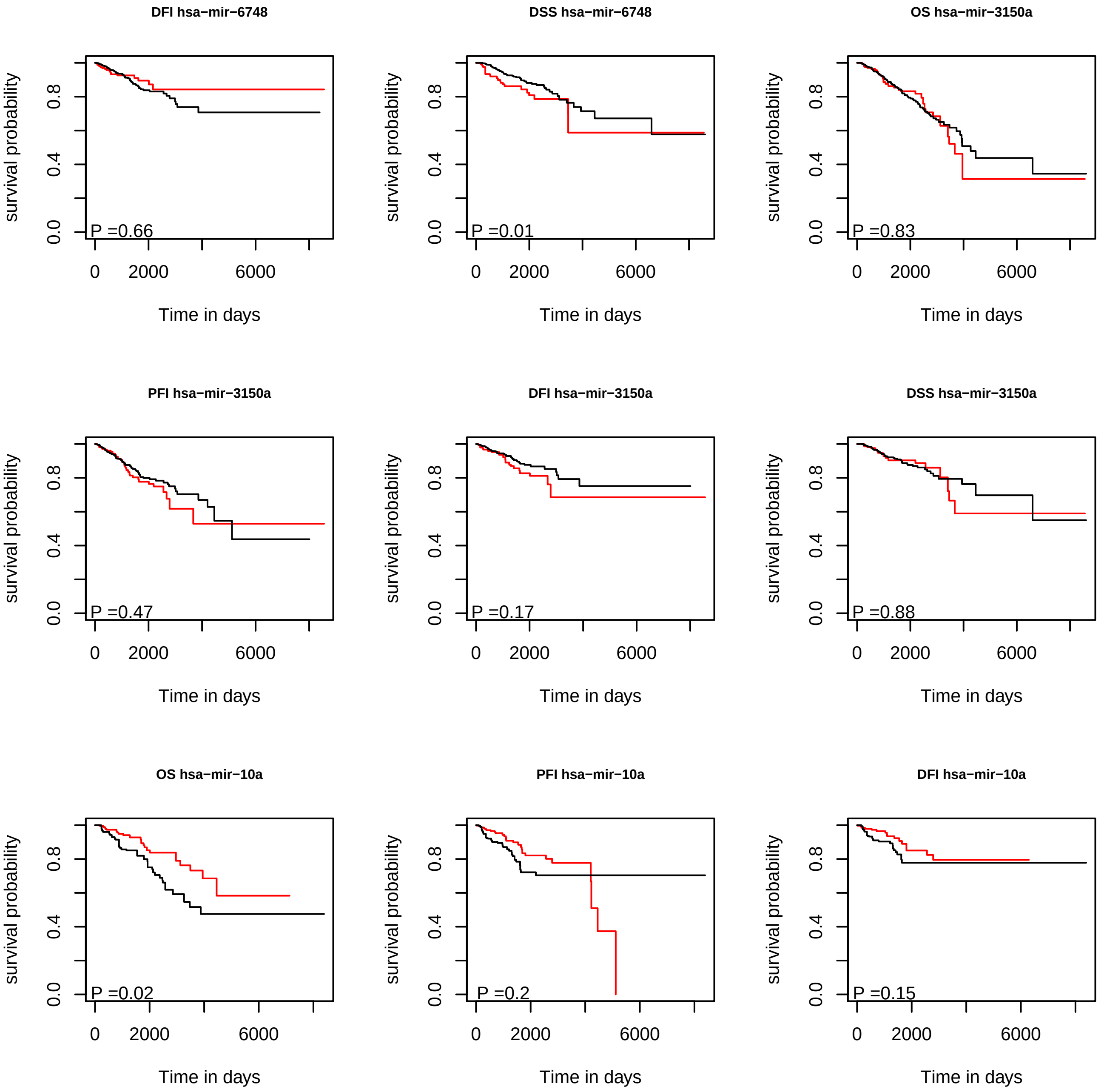
DFI hsa−mir−6748
P =0.66
DSS hsa−mir−6748
P =0.01
OS hsa−mir−3150a
P =0.83
PFI hsa−mir−3150a
P =0.47
DFI hsa−mir−3150a
P =0.17
DSS hsa−mir−3150a
P =0.88
OS hsa−mir−10a
P =0.02
PFI hsa−mir−10a
P =0.2
DFI hsa−mir−10a
P =0.15
survival probability
0.0
0.4
0.8
Time in days
0
2000
6000

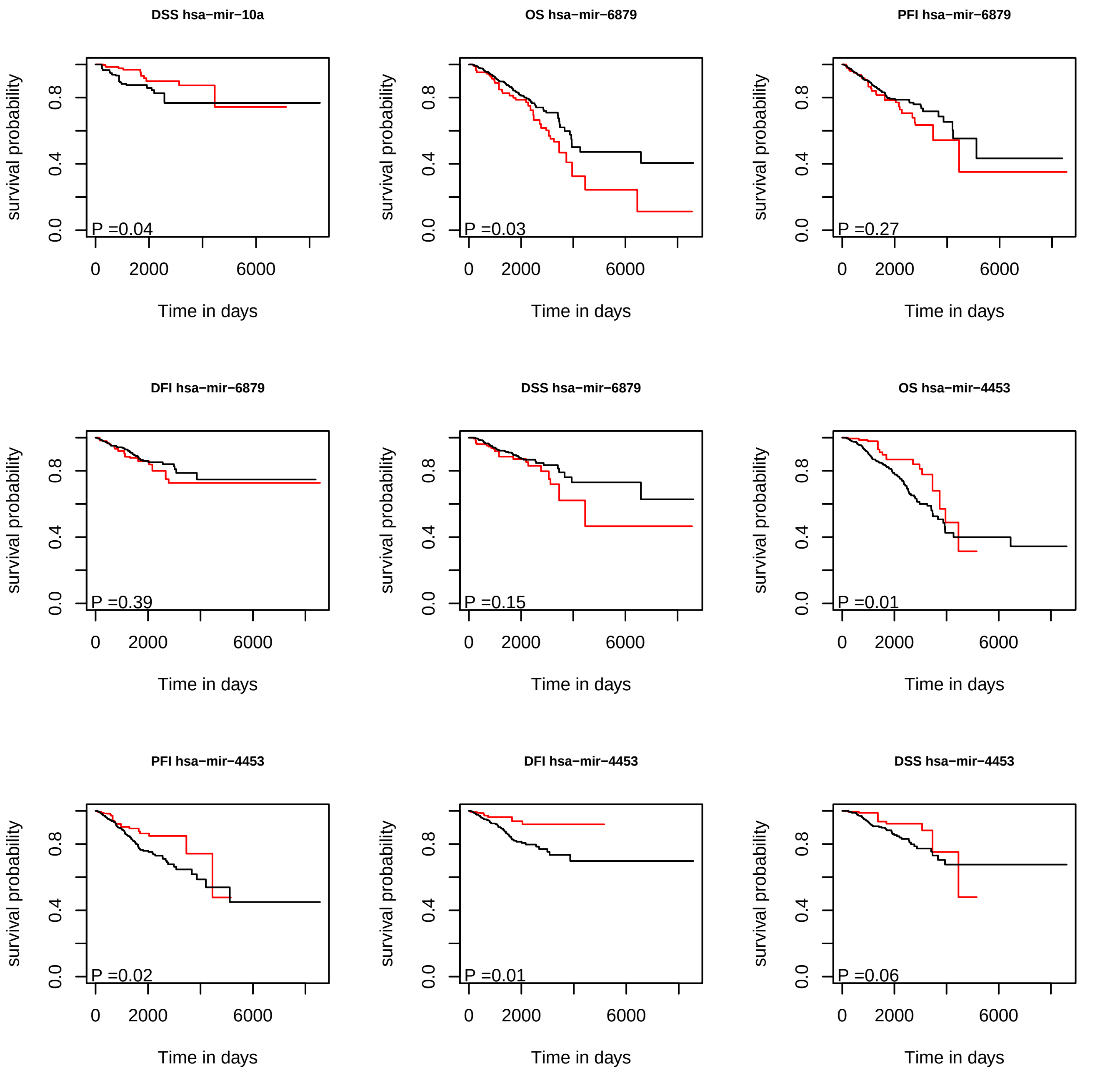
DSS hsa−mir−10a
P =0.04
OS hsa−mir−6879
P =0.03
PFI hsa−mir−6879
P =0.27
DFI hsa−mir−6879
P =0.39
DSS hsa−mir−6879
P =0.15
OS hsa−mir−4453
P =0.01
PFI hsa−mir−4453
P =0.02
DFI hsa−mir−4453
P =0.01
DSS hsa−mir−4453
P =0.06
survival probability
0.0
0.4
0.8
Time in days
0
2000
6000

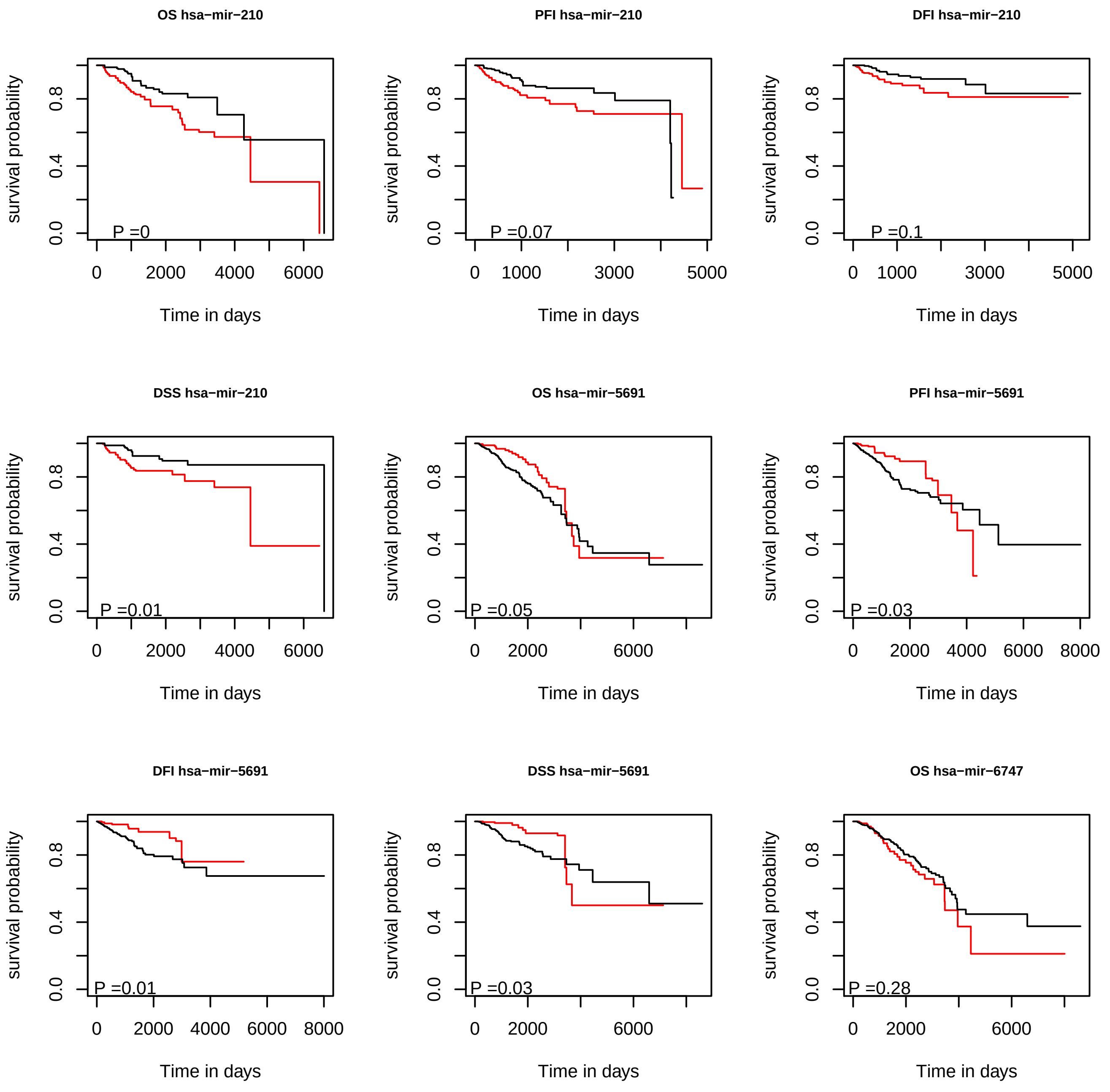
OS hsa−mir−210
P =0
PFI hsa−mir−210
P =0.07
DFI hsa−mir−210
P =0.1
DSS hsa−mir−210
P =0.01
OS hsa−mir−5691
P =0.05
PFI hsa−mir−5691
P =0.03
DFI hsa−mir−5691
P =0.01
DSS hsa−mir−5691
P =0.03
OS hsa−mir−6747
P =0.28
survival probability
Time in days
0.0
0.4
0.8
0
1000
2000
3000
4000
5000
6000
8000

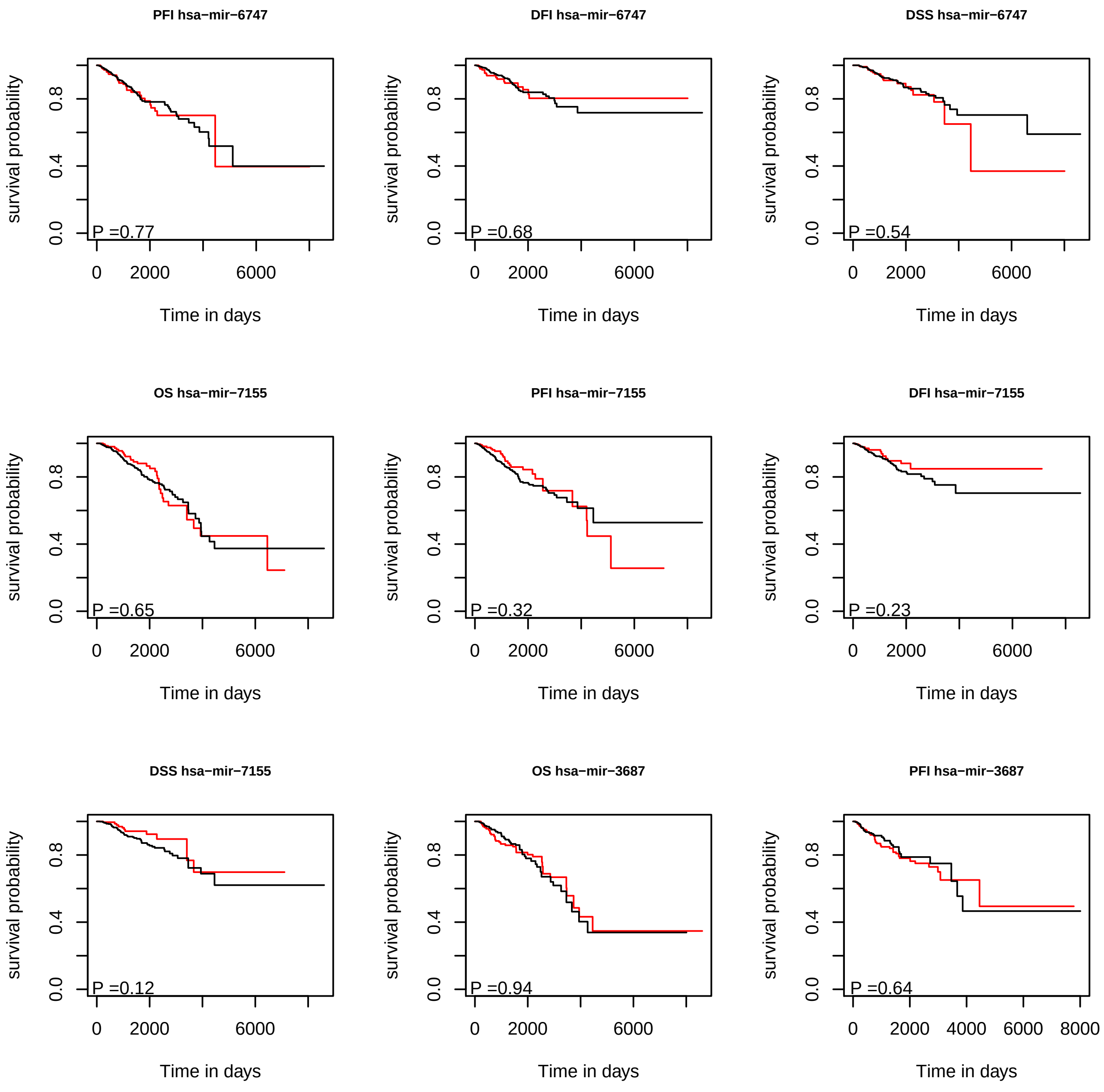
PFI hsa−mir−6747
P =0.77
DFI hsa−mir−6747
P =0.68
DSS hsa−mir−6747
P =0.54
OS hsa−mir−7155
P =0.65
PFI hsa−mir−7155
P =0.32
DFI hsa−mir−7155
P =0.23
DSS hsa−mir−7155
P =0.12
OS hsa−mir−3687
P =0.94
PFI hsa−mir−3687
P =0.64
survival probability
0.0
0.4
0.8
Time in days
0
2000
4000
6000
8000

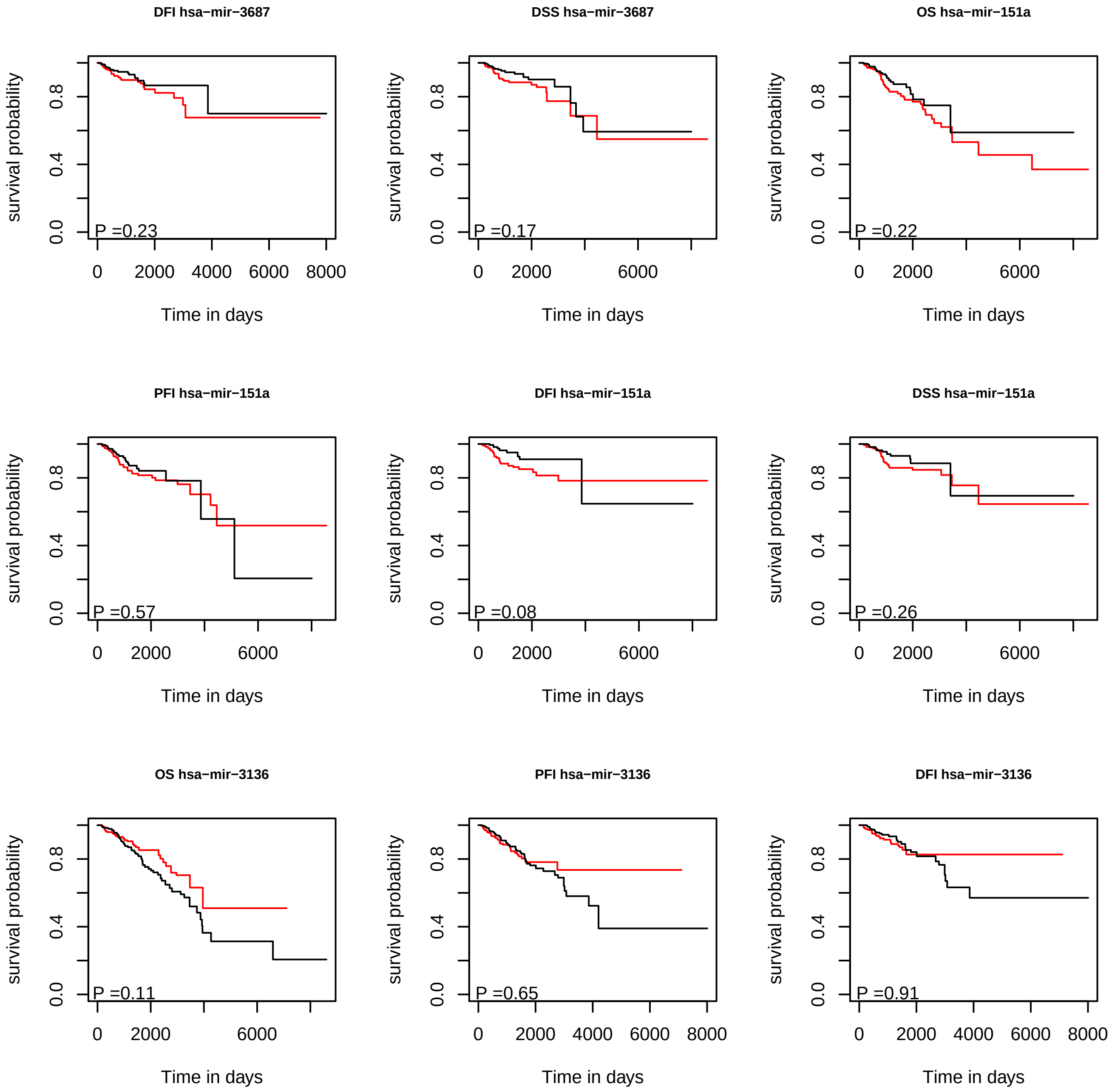
DFI hsa−mir−3687
DSS hsa−mir−3687
OS hsa−mir−151a
PFI hsa−mir−151a
DFI hsa−mir−151a
DSS hsa−mir−151a
OS hsa−mir−3136
PFI hsa−mir−3136
DFI hsa−mir−3136
survival probability
Time in days
P =0.23
P =0.17
P =0.22
P =0.57
P =0.08
P =0.26
P =0.11
P =0.65
P =0.91
0.0
0.4
0.8
0
2000
4000
6000
8000

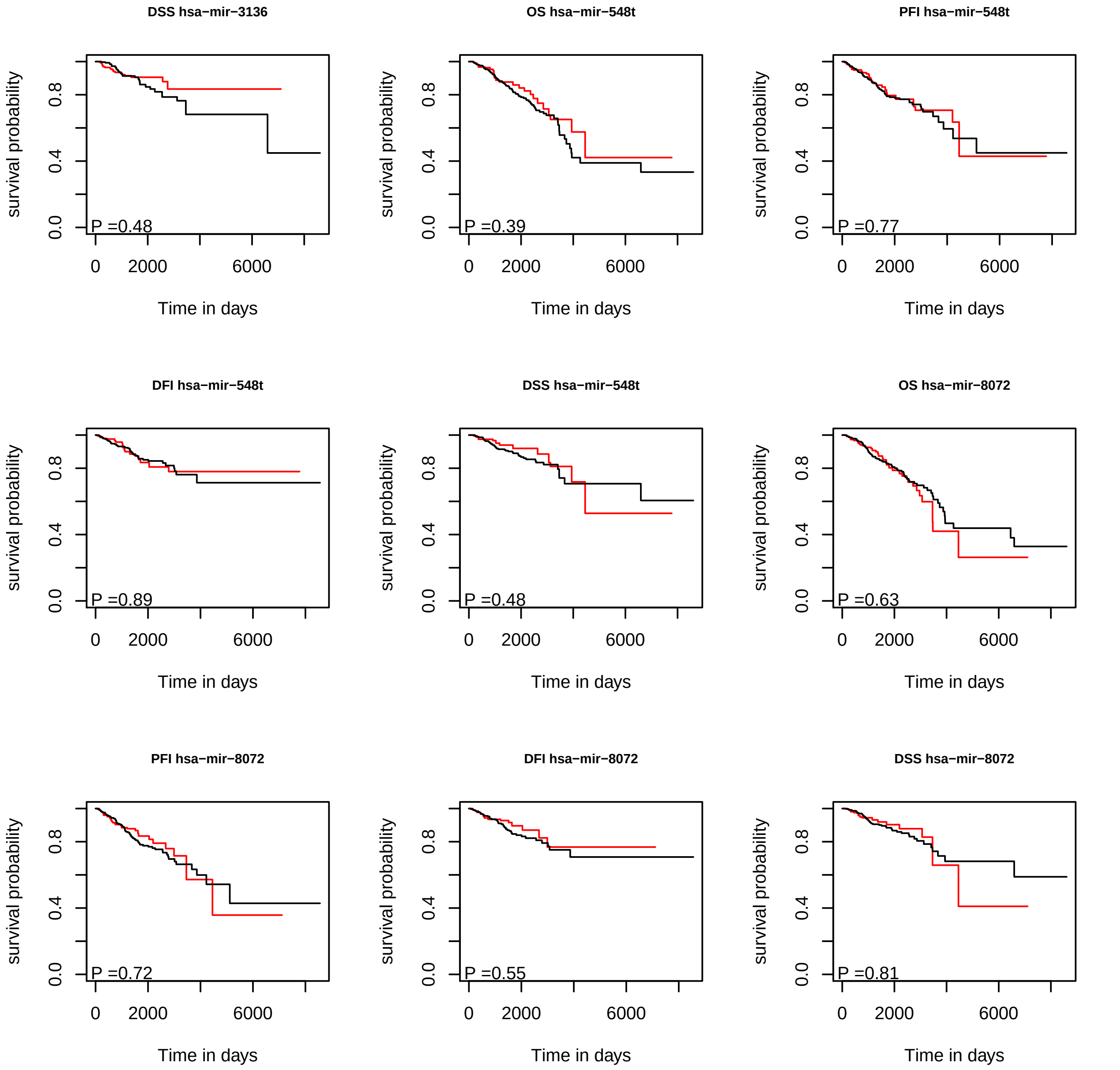
DSS hsa−mir−3136
P =0.48
OS hsa−mir−548t
P =0.39
PFI hsa−mir−548t
P =0.77
DFI hsa−mir−548t
P =0.89
DSS hsa−mir−548t
P =0.48
OS hsa−mir−8072
P =0.63
PFI hsa−mir−8072
P =0.72
DFI hsa−mir−8072
P =0.55
DSS hsa−mir−8072
P =0.81
survival probability
0.0
0.4
0.8
Time in days
0
2000
6000

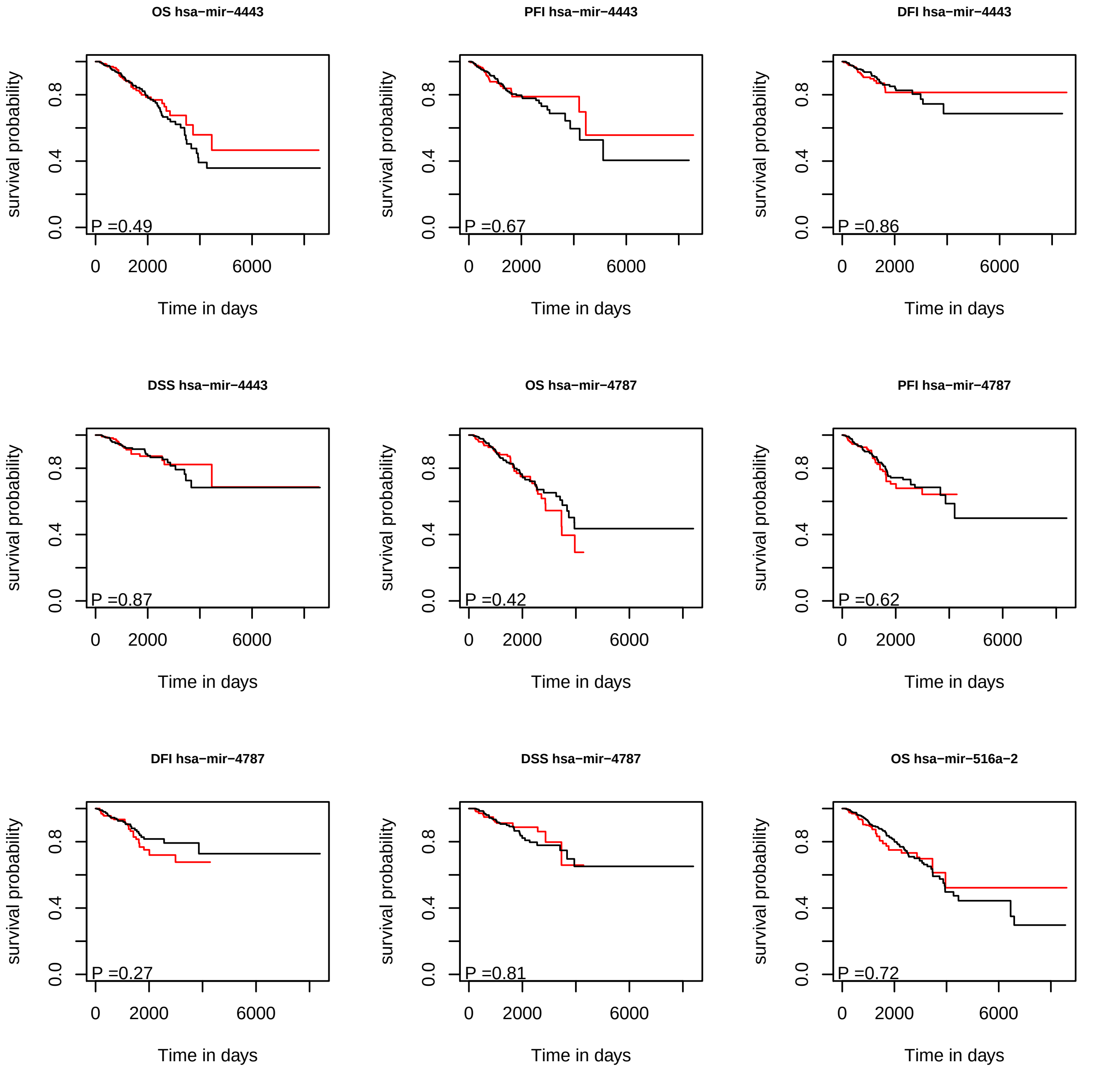

OS hsa−mir−4443
P =0.49
PFI hsa−mir−4443
P =0.67
DFI hsa−mir−4443
P =0.86
DSS hsa−mir−4443
P =0.87
OS hsa−mir−4787
P =0.42
PFI hsa−mir−4787
P =0.62
DFI hsa−mir−4787
P =0.27
DSS hsa−mir−4787
P =0.81
OS hsa−mir−516a−2
P =0.72
survival probability
0.0
0.4
0.8
0
2000
6000
Time in days

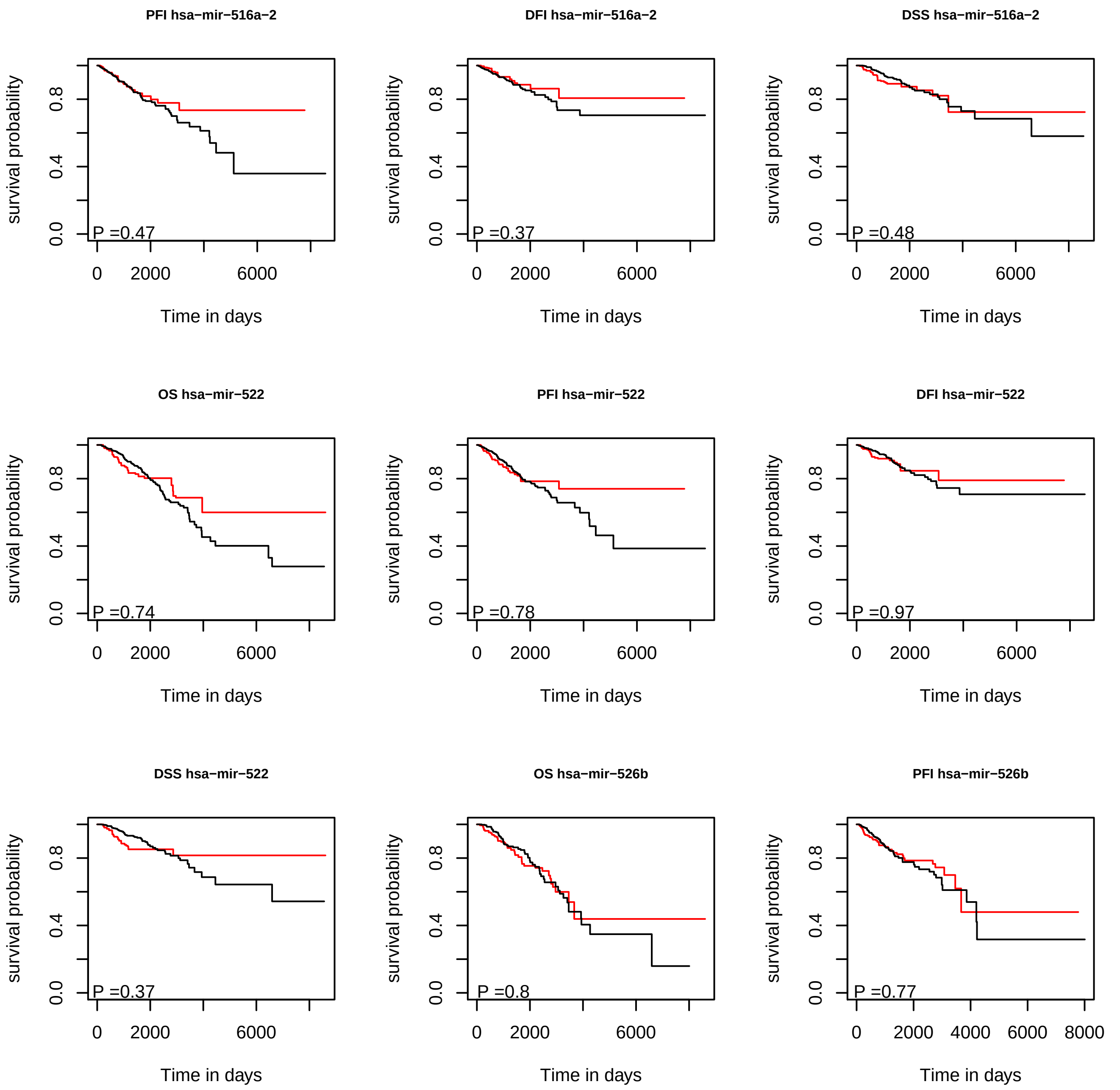
PFI hsa−mir−516a−2
survival probability
0.0
0.4
0.8
P =0.47
0
2000
6000
Time in days
DFI hsa−mir−516a−2
survival probability
0.0
0.4
0.8
P =0.37
0
2000
6000
Time in days
DSS hsa−mir−516a−2
survival probability
0.0
0.4
0.8
P =0.48
0
2000
6000
Time in days
OS hsa−mir−522
survival probability
0.0
0.4
0.8
P =0.74
0
2000
6000
Time in days
PFI hsa−mir−522
survival probability
0.0
0.4
0.8
P =0.78
0
2000
6000
Time in days
DFI hsa−mir−522
survival probability
0.0
0.4
0.8
P =0.97
0
2000
6000
Time in days
DSS hsa−mir−522
survival probability
0.0
0.4
0.8
P =0.37
0
2000
6000
Time in days
OS hsa−mir−526b
survival probability
0.0
0.4
0.8
P =0.8
0
2000
6000
Time in days
PFI hsa−mir−526b
survival probability
0.0
0.4
0.8
P =0.77
0
2000
4000
6000
8000
Time in days

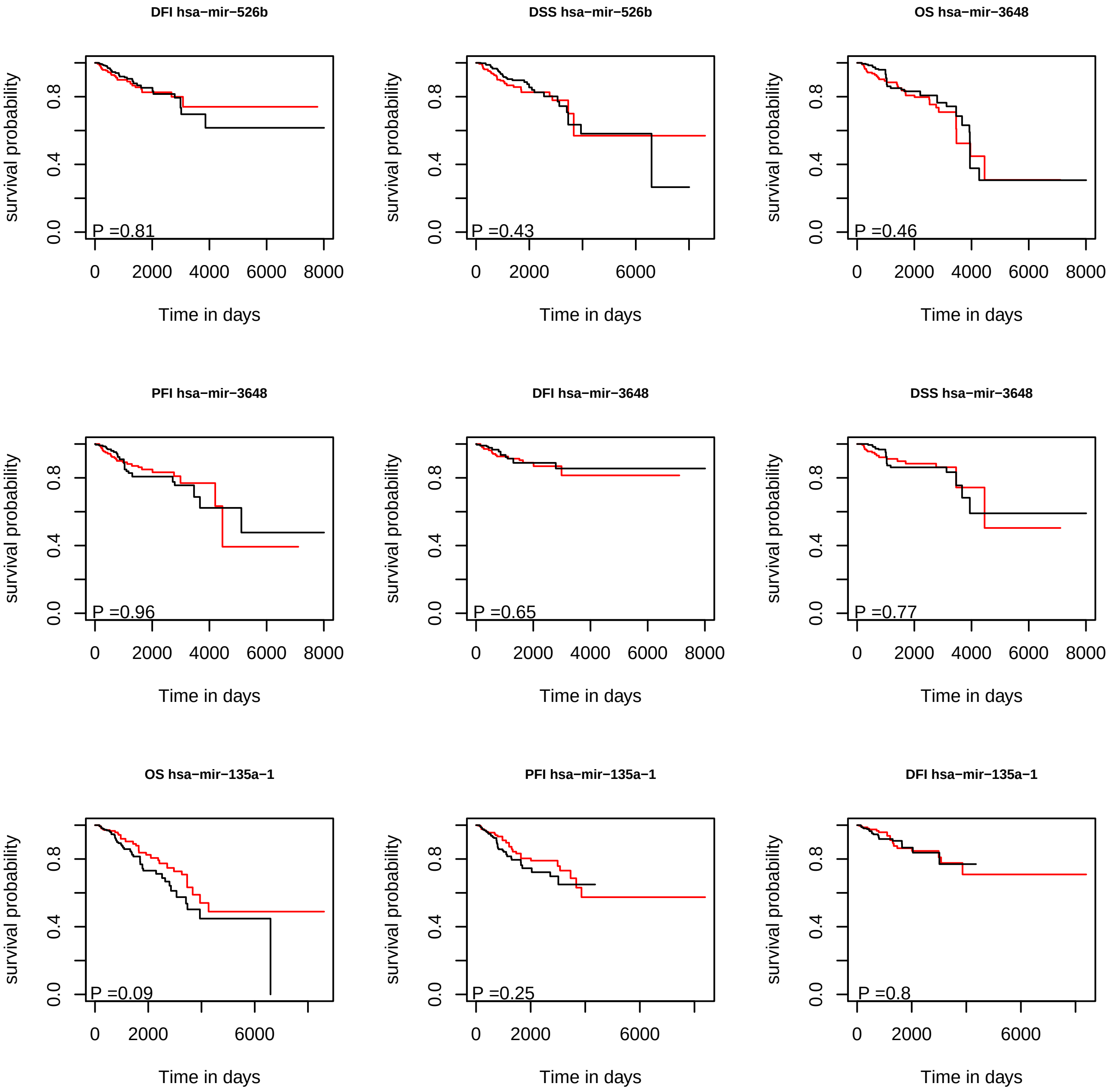

DFI hsa−mir−526b
P =0.81
DSS hsa−mir−526b
P =0.43
OS hsa−mir−3648
P =0.46
PFI hsa−mir−3648
P =0.96
DFI hsa−mir−3648
P =0.65
DSS hsa−mir−3648
P =0.77
OS hsa−mir−135a−1
P =0.09
PFI hsa−mir−135a−1
P =0.25
DFI hsa−mir−135a−1
P =0.8
survival probability
0.0
0.4
0.8
Time in days
0
2000
4000
6000
8000

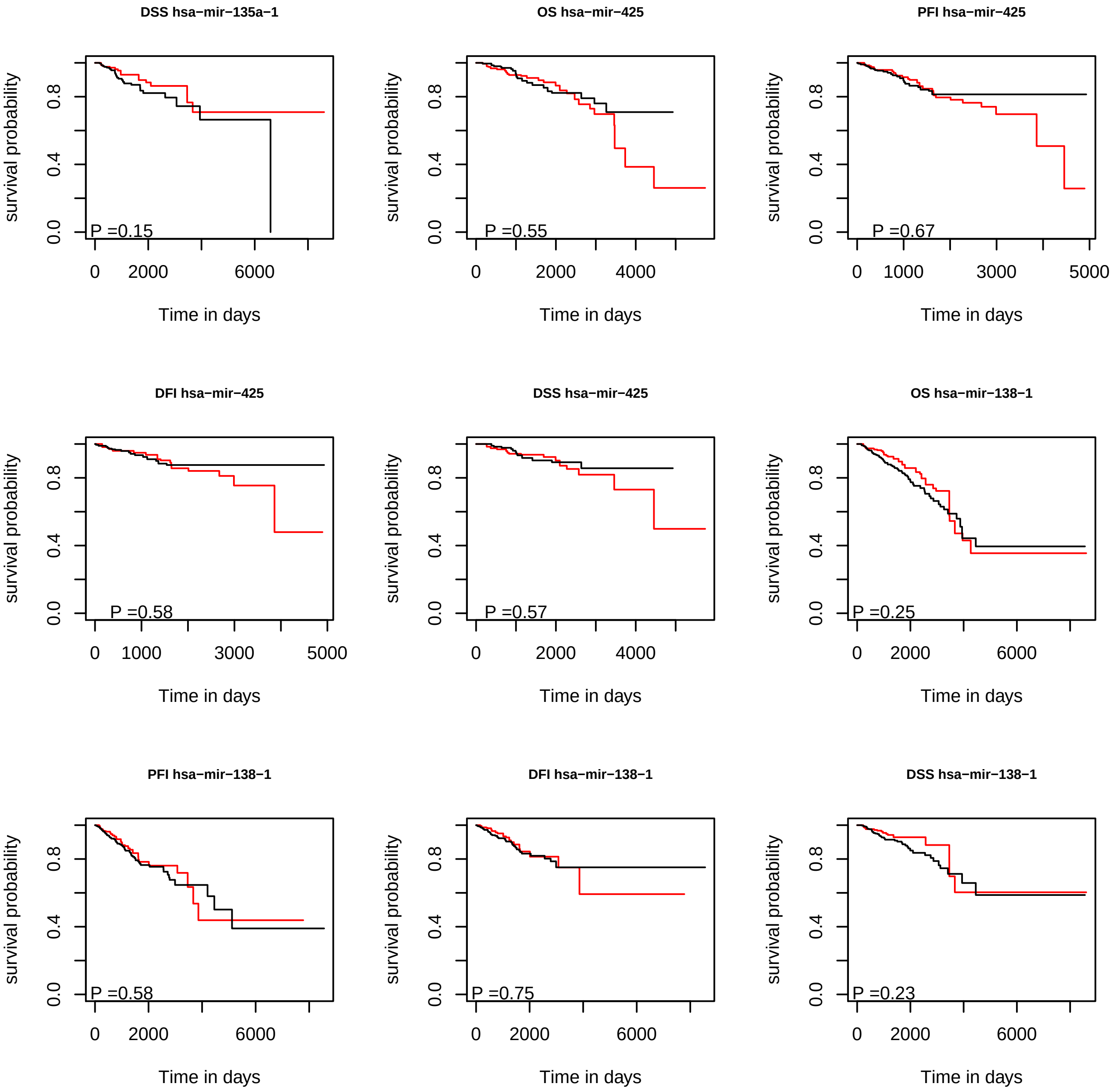
DSS hsa−mir−135a−1
P =0.15
OS hsa−mir−425
P =0.55
PFI hsa−mir−425
P =0.67
DFI hsa−mir−425
P =0.58
DSS hsa−mir−425
P =0.57
OS hsa−mir−138−1
P =0.25
PFI hsa−mir−138−1
P =0.58
DFI hsa−mir−138−1
P =0.75
DSS hsa−mir−138−1
P =0.23
survival probability
Time in days

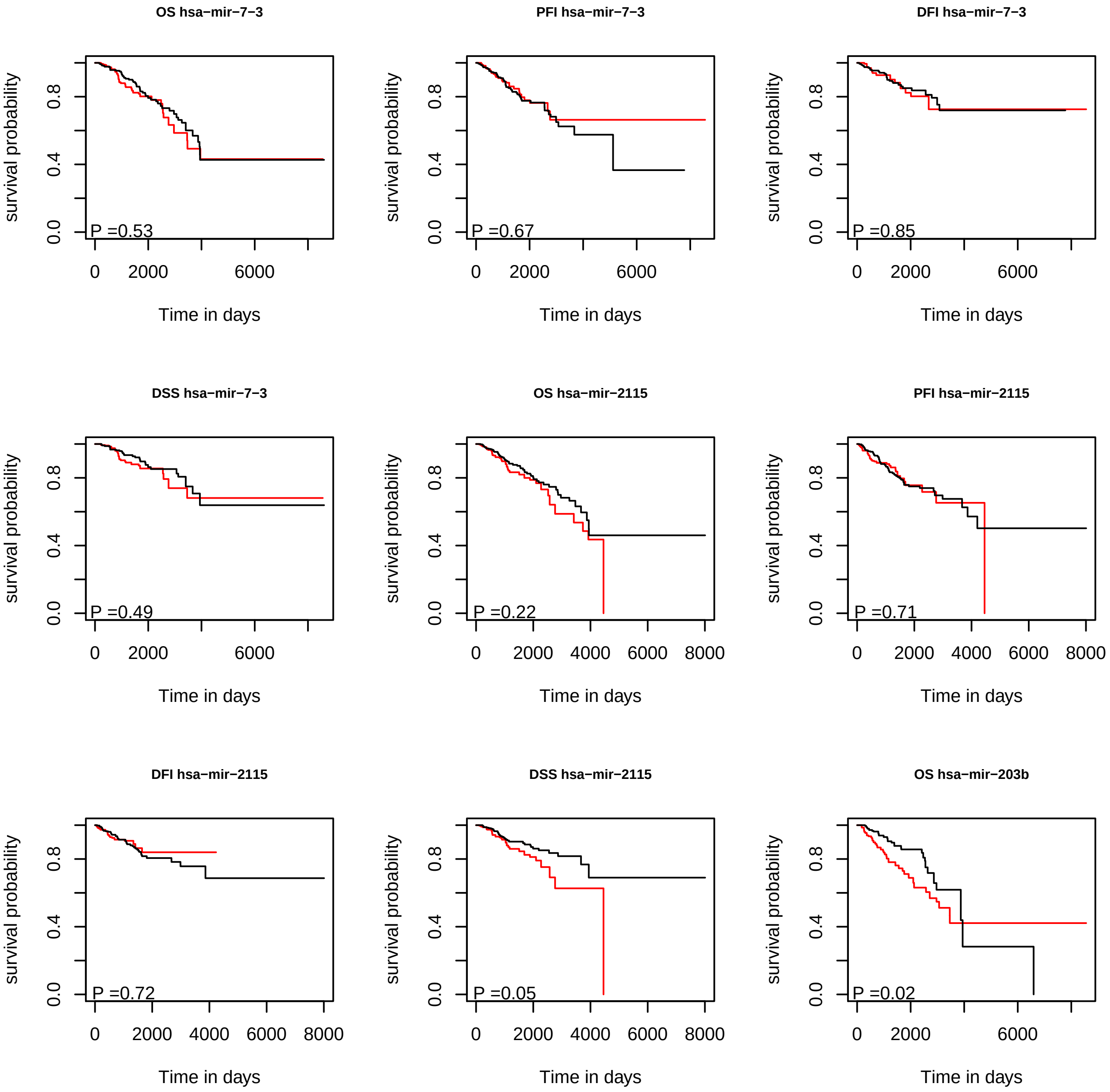
OS hsa−mir−7−3
P =0.53
PFI hsa−mir−7−3
P =0.67
DFI hsa−mir−7−3
P =0.85
DSS hsa−mir−7−3
P =0.49
OS hsa−mir−2115
P =0.22
PFI hsa−mir−2115
P =0.71
DFI hsa−mir−2115
P =0.72
DSS hsa−mir−2115
P =0.05
OS hsa−mir−203b
P =0.02
survival probability
Time in days
0.0
0.4
0.8
0
2000
4000
6000
8000

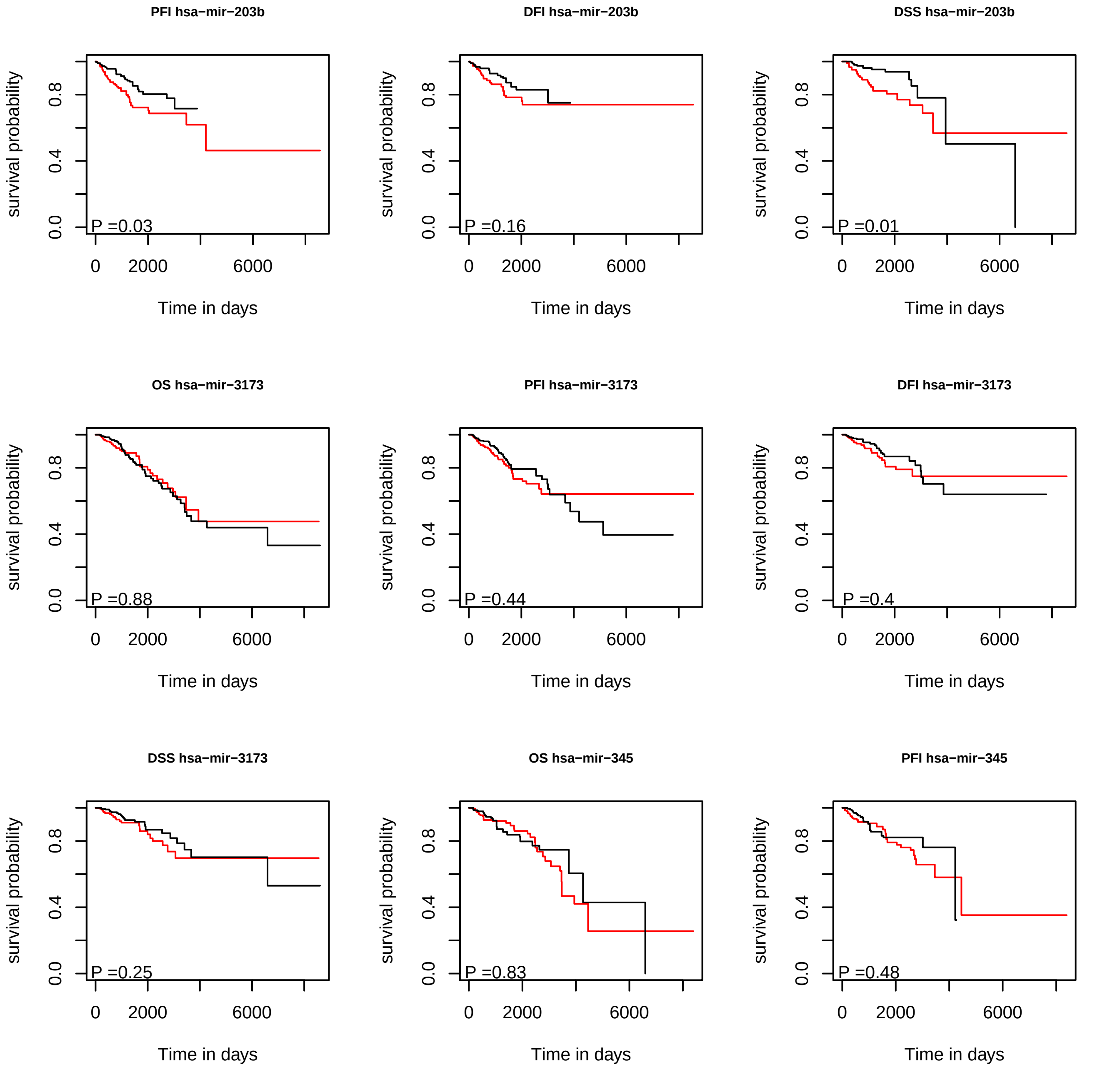
PFI hsa−mir−203b
P =0.03
DFI hsa−mir−203b
P =0.16
DSS hsa−mir−203b
P =0.01
OS hsa−mir−3173
P =0.88
PFI hsa−mir−3173
P =0.44
DFI hsa−mir−3173
P =0.4
DSS hsa−mir−3173
P =0.25
OS hsa−mir−345
P =0.83
PFI hsa−mir−345
P =0.48
survival probability
0.0
0.4
0.8
Time in days
0
2000
6000

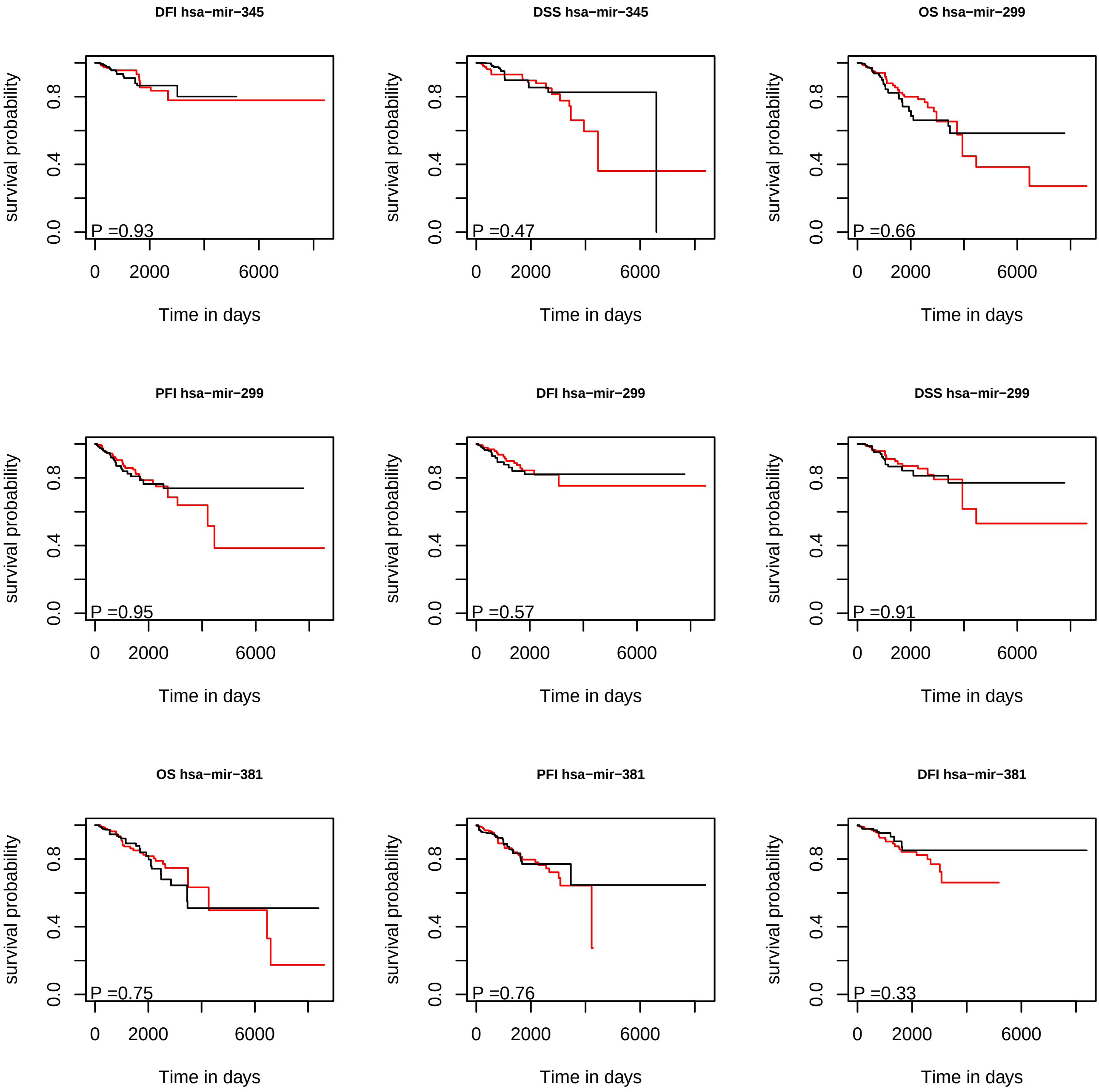
DFI hsa−mir−345
P =0.93
DSS hsa−mir−345
P =0.47
OS hsa−mir−299
P =0.66
PFI hsa−mir−299
P =0.95
DFI hsa−mir−299
P =0.57
DSS hsa−mir−299
P =0.91
OS hsa−mir−381
P =0.75
PFI hsa−mir−381
P =0.76
DFI hsa−mir−381
P =0.33
survival probability
0.0
0.4
0.8
Time in days
0
2000
6000

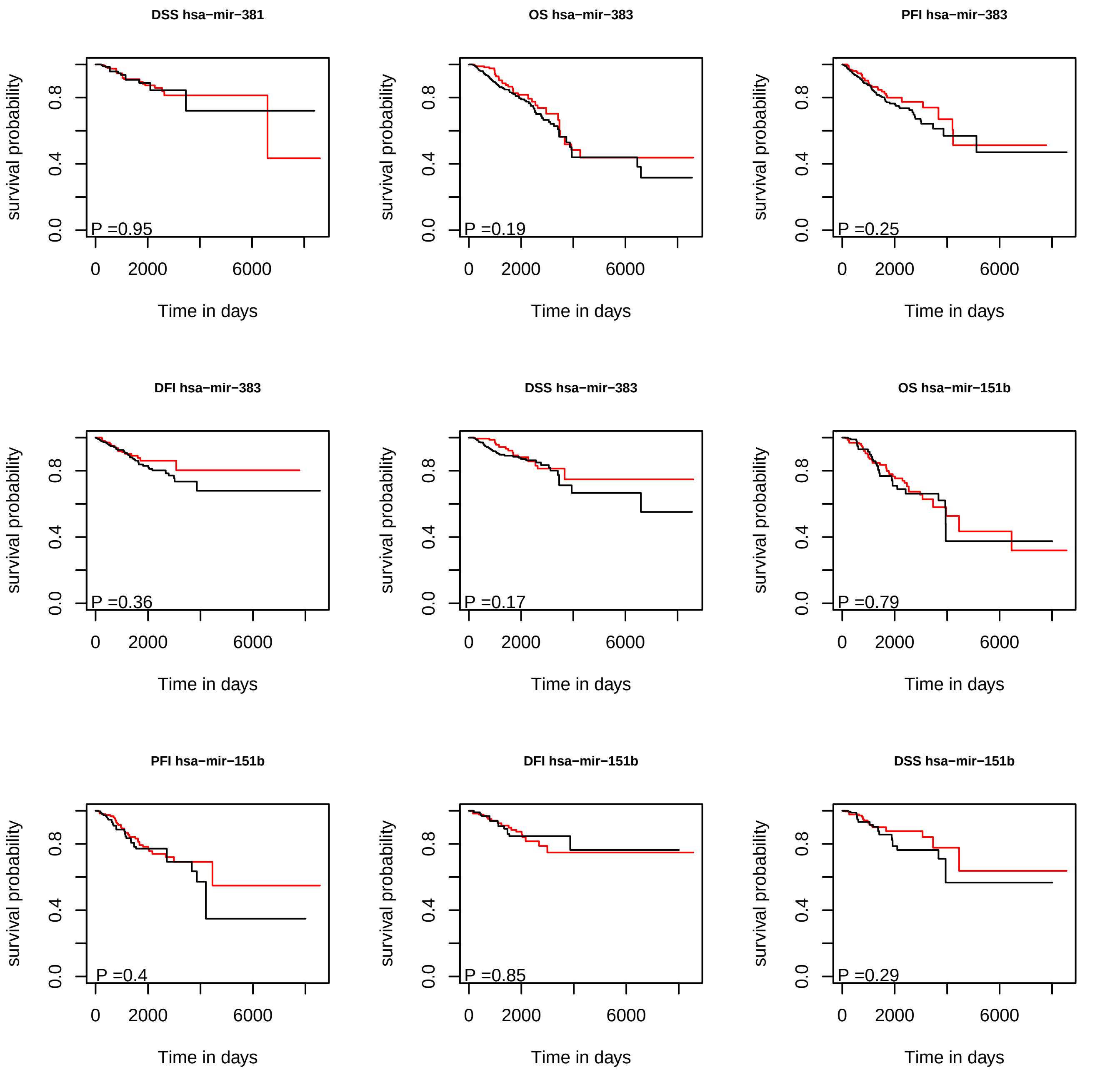
DSS hsa−mir−381
P =0.95
OS hsa−mir−383
P =0.19
PFI hsa−mir−383
P =0.25
DFI hsa−mir−383
P =0.36
DSS hsa−mir−383
P =0.17
OS hsa−mir−151b
P =0.79
PFI hsa−mir−151b
P =0.4
DFI hsa−mir−151b
P =0.85
DSS hsa−mir−151b
P =0.29
survival probability
Time in days
0.0
0.4
0.8
0
2000
6000

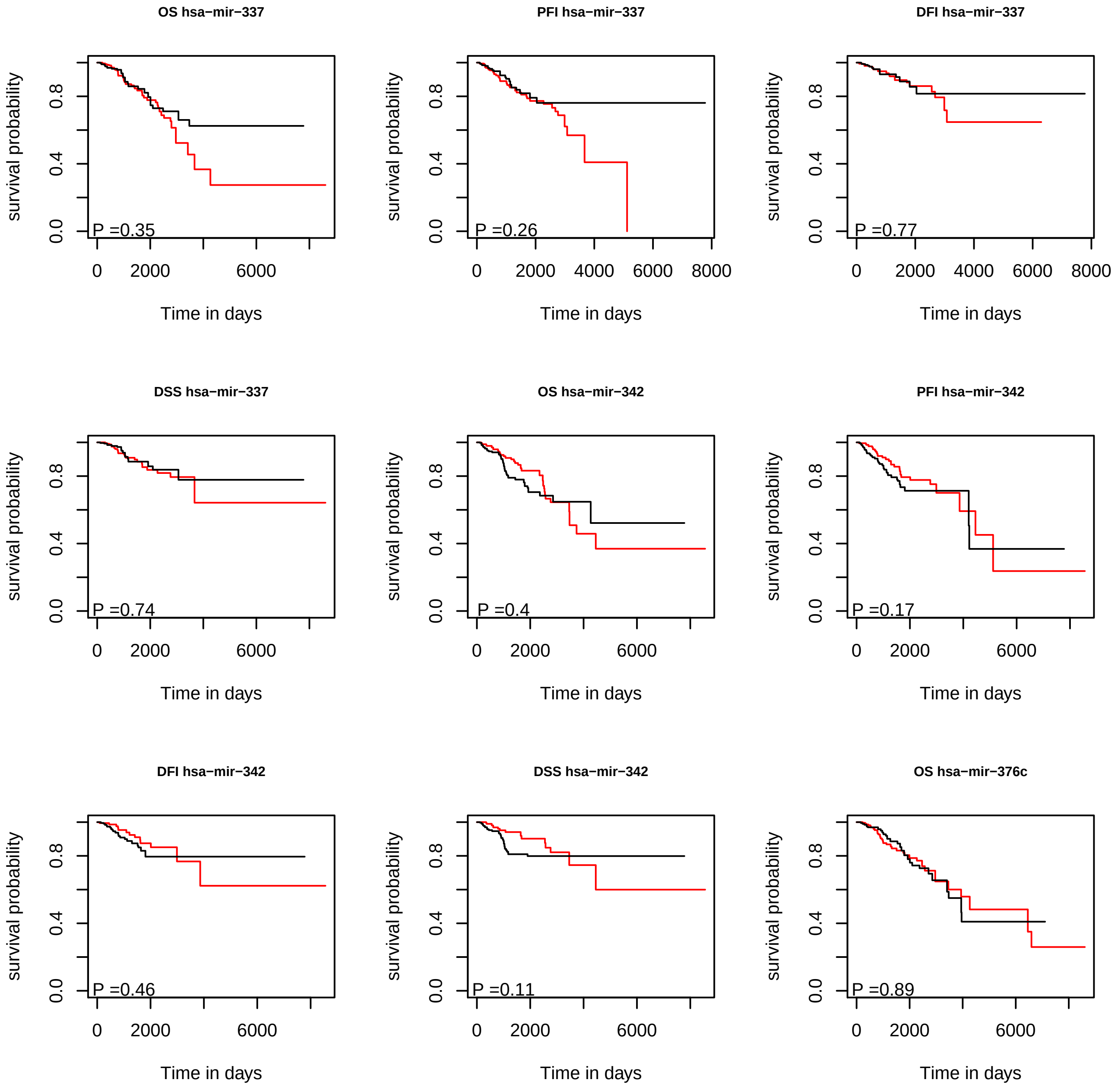

OS hsa−mir−337
P =0.35
PFI hsa−mir−337
P =0.26
DFI hsa−mir−337
P =0.77
DSS hsa−mir−337
P =0.74
OS hsa−mir−342
P =0.4
PFI hsa−mir−342
P =0.17
DFI hsa−mir−342
P =0.46
DSS hsa−mir−342
P =0.11
OS hsa−mir−376c
P =0.89
survival probability
0.0
0.4
0.8
Time in days
0
2000
4000
6000
8000

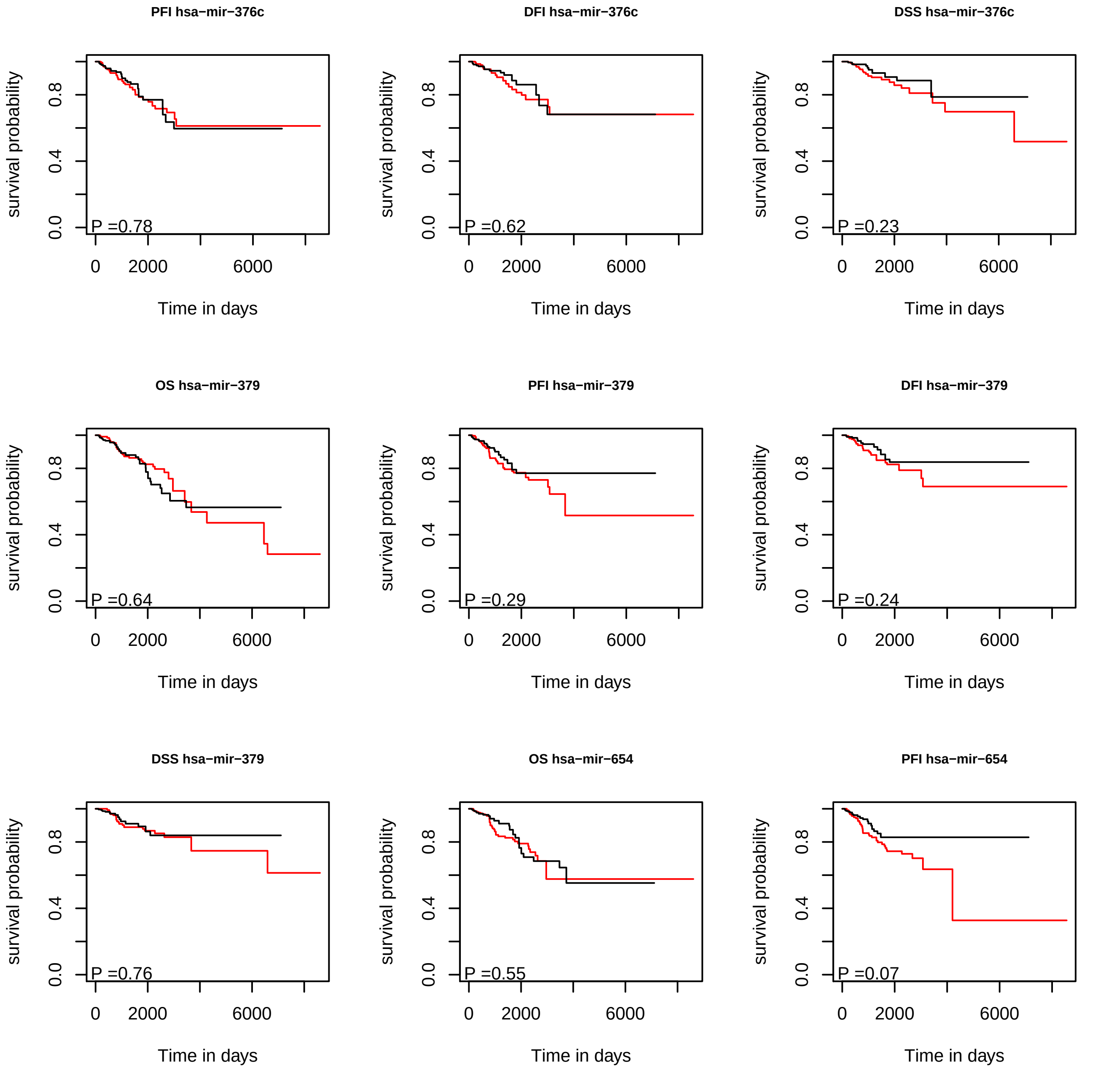
PFI hsa−mir−376c
P =0.78
DFI hsa−mir−376c
P =0.62
DSS hsa−mir−376c
P =0.23
OS hsa−mir−379
P =0.64
PFI hsa−mir−379
P =0.29
DFI hsa−mir−379
P =0.24
DSS hsa−mir−379
P =0.76
OS hsa−mir−654
P =0.55
PFI hsa−mir−654
P =0.07
survival probability
0.0
0.4
0.8
Time in days
0
2000
6000

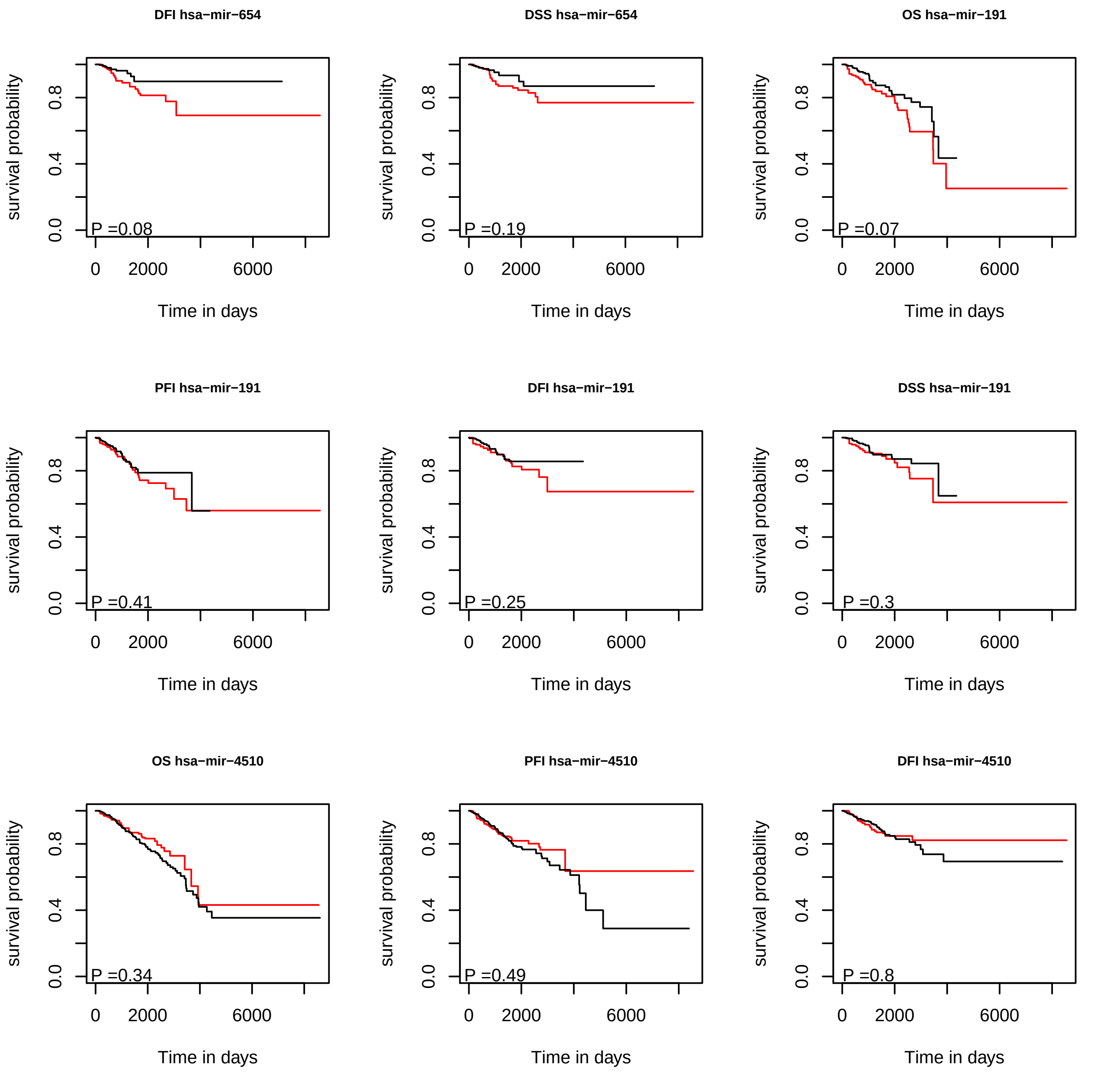
DFI hsa−mir−654
survival probability
0.0
0.4
0.8
P =0.08
0
2000
6000
Time in days
DSS hsa−mir−654
survival probability
0.0
0.4
0.8
P =0.19
0
2000
6000
Time in days
OS hsa−mir−191
survival probability
0.0
0.4
0.8
P =0.07
0
2000
6000
Time in days
PFI hsa−mir−191
survival probability
0.0
0.4
0.8
P =0.41
0
2000
6000
Time in days
DFI hsa−mir−191
survival probability
0.0
0.4
0.8
P =0.25
0
2000
6000
Time in days
DSS hsa−mir−191
survival probability
0.0
0.4
0.8
P =0.3
0
2000
6000
Time in days
OS hsa−mir−4510
survival probability
0.0
0.4
0.8
P =0.34
0
2000
6000
Time in days
PFI hsa−mir−4510
survival probability
0.0
0.4
0.8
P =0.49
0
2000
6000
Time in days
DFI hsa−mir−4510
survival probability
0.0
0.4
0.8
P =0.8
0
2000
6000
Time in days

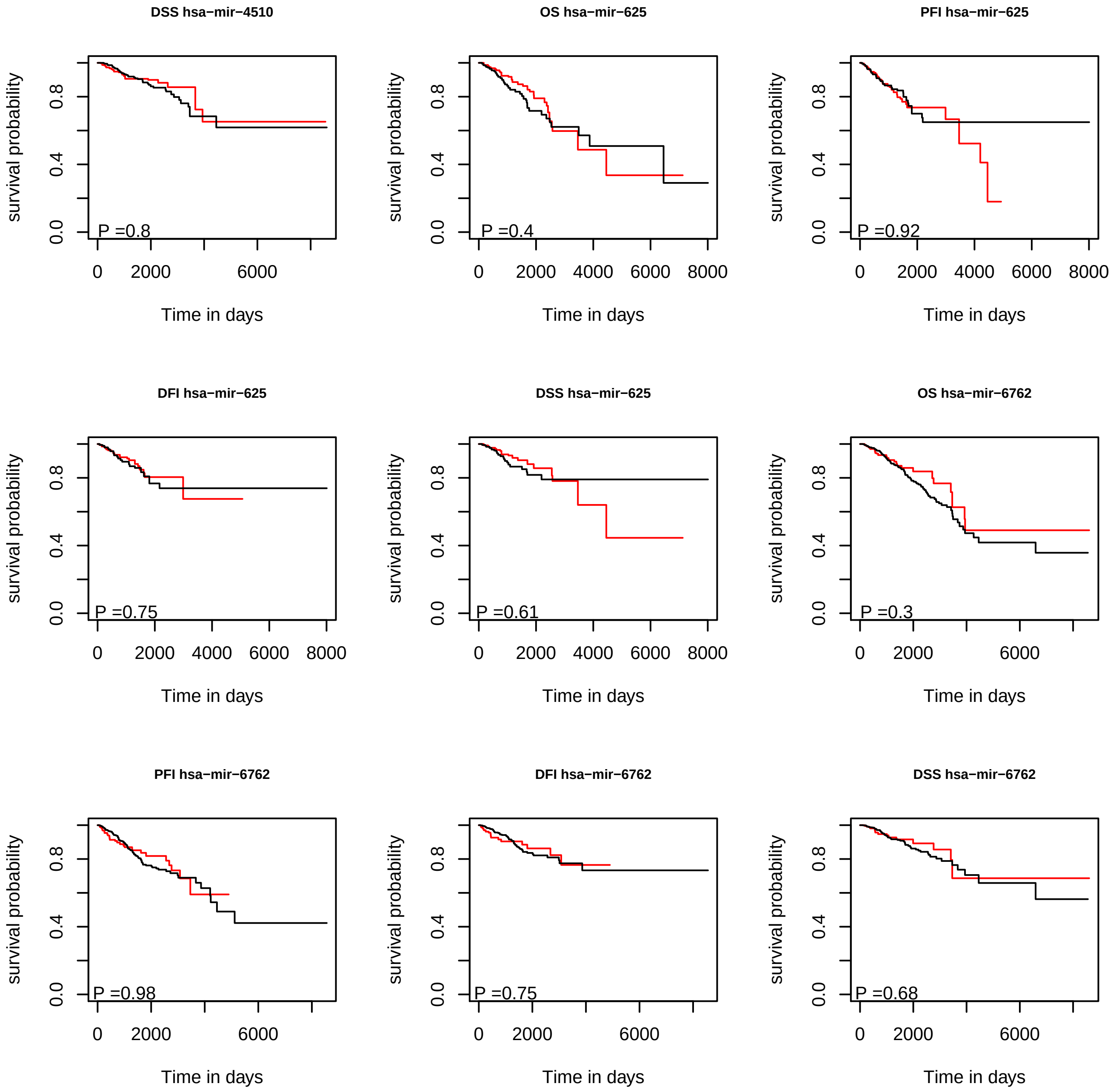
DSS hsa−mir−4510
P =0.8
OS hsa−mir−625
P =0.4
PFI hsa−mir−625
P =0.92
DFI hsa−mir−625
P =0.75
DSS hsa−mir−625
P =0.61
OS hsa−mir−6762
P =0.3
PFI hsa−mir−6762
P =0.98
DFI hsa−mir−6762
P =0.75
DSS hsa−mir−6762
P =0.68
survival probability
0.0
0.4
0.8
Time in days
0
2000
4000
6000
8000

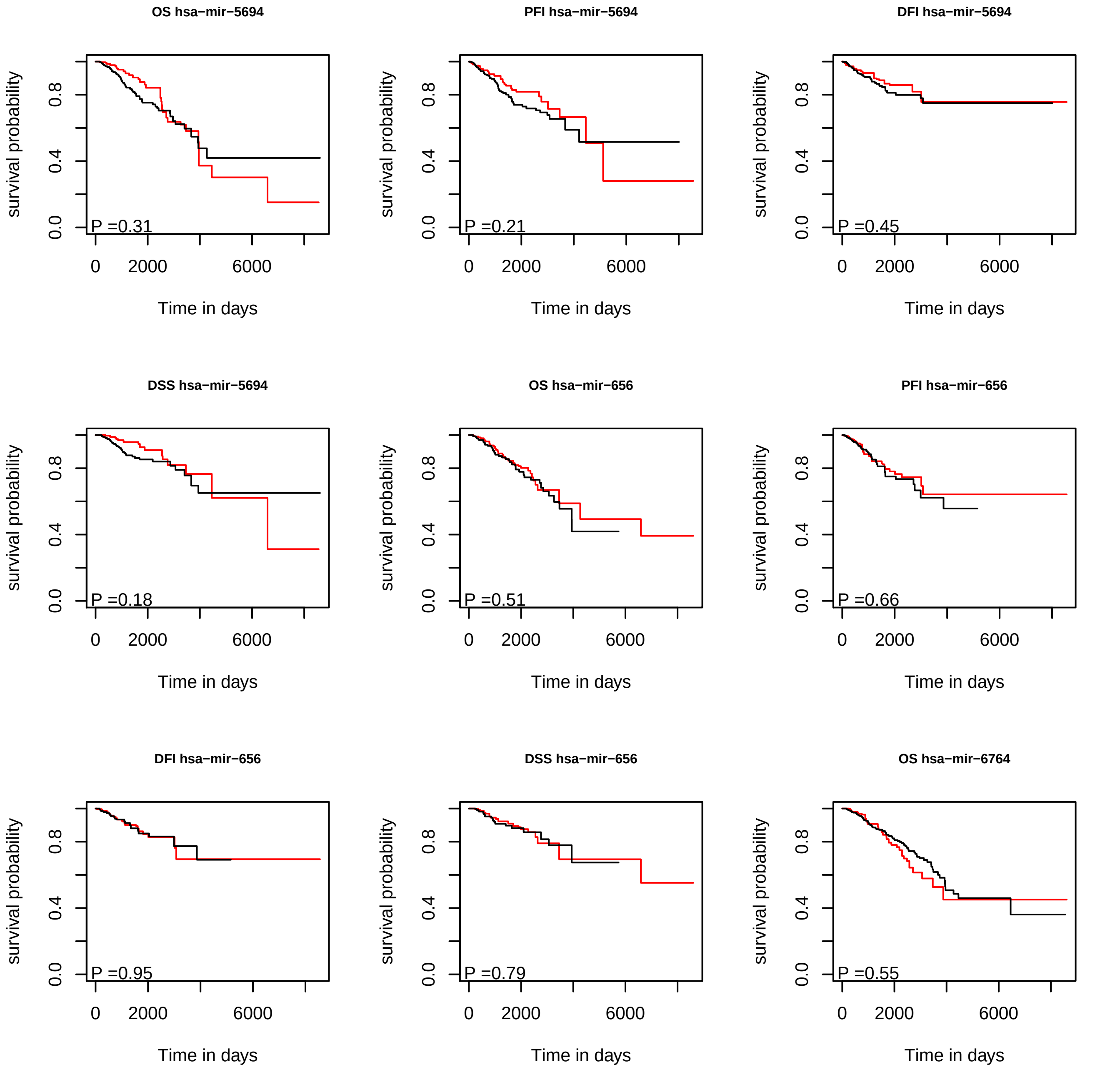
OS hsa−mir−5694
P =0.31
PFI hsa−mir−5694
P =0.21
DFI hsa−mir−5694
P =0.45
DSS hsa−mir−5694
P =0.18
OS hsa−mir−656
P =0.51
PFI hsa−mir−656
P =0.66
DFI hsa−mir−656
P =0.95
DSS hsa−mir−656
P =0.79
OS hsa−mir−6764
P =0.55
survival probability
0.0
0.4
0.8
0
2000
6000
Time in days

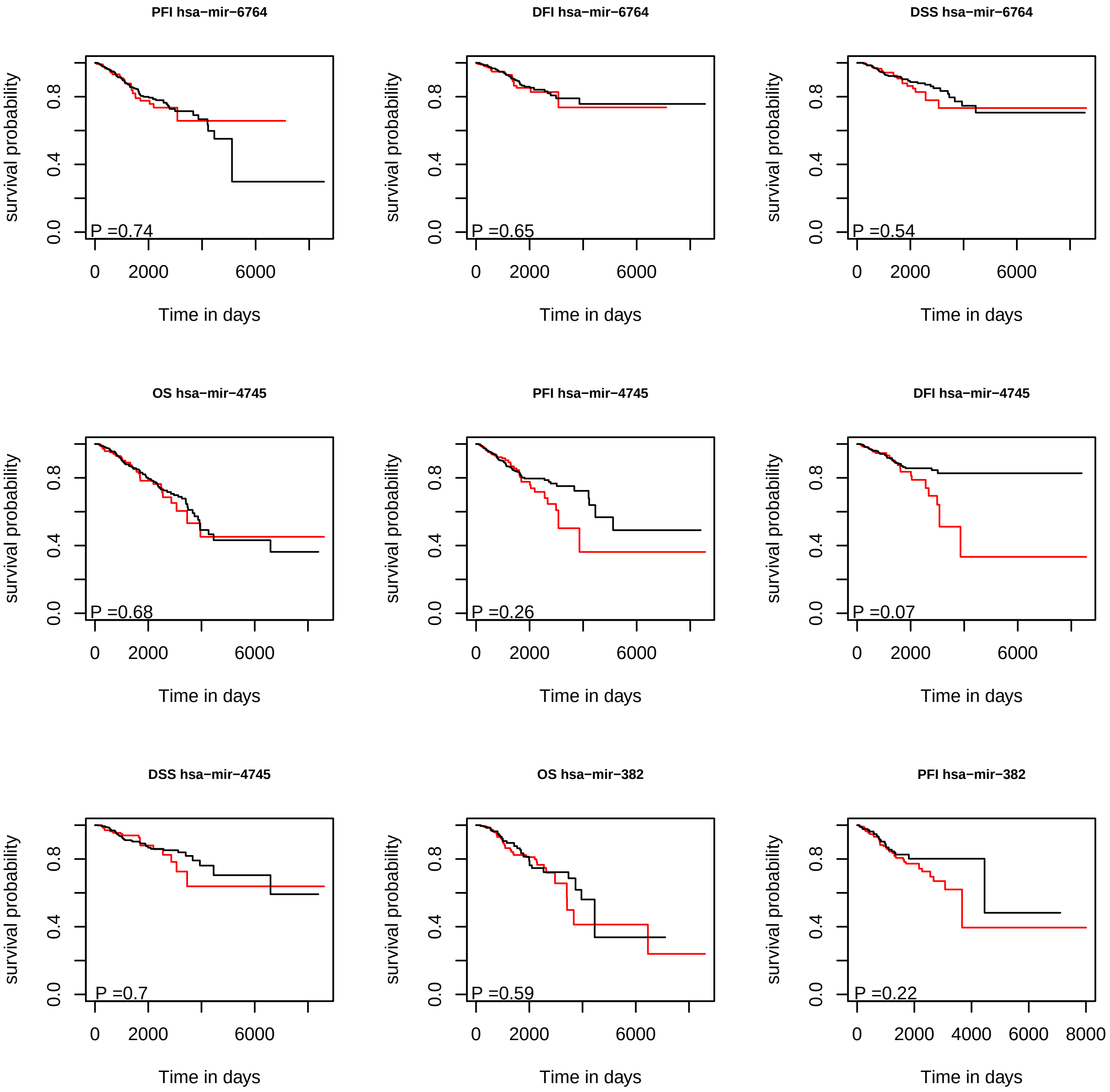

PFI hsa−mir−6764
survival probability
0.0
0.4
0.8
P =0.74
0
2000
6000
Time in days
DFI hsa−mir−6764
survival probability
0.0
0.4
0.8
P =0.65
0
2000
6000
Time in days
DSS hsa−mir−6764
survival probability
0.0
0.4
0.8
P =0.54
0
2000
6000
Time in days
OS hsa−mir−4745
survival probability
0.0
0.4
0.8
P =0.68
0
2000
6000
Time in days
PFI hsa−mir−4745
survival probability
0.0
0.4
0.8
P =0.26
0
2000
6000
Time in days
DFI hsa−mir−4745
survival probability
0.0
0.4
0.8
P =0.07
0
2000
6000
Time in days
DSS hsa−mir−4745
survival probability
0.0
0.4
0.8
P =0.7
0
2000
6000
Time in days
OS hsa−mir−382
survival probability
0.0
0.4
0.8
P =0.59
0
2000
6000
Time in days
PFI hsa−mir−382
survival probability
0.0
0.4
0.8
P =0.22
0
2000
4000
6000
8000
Time in days

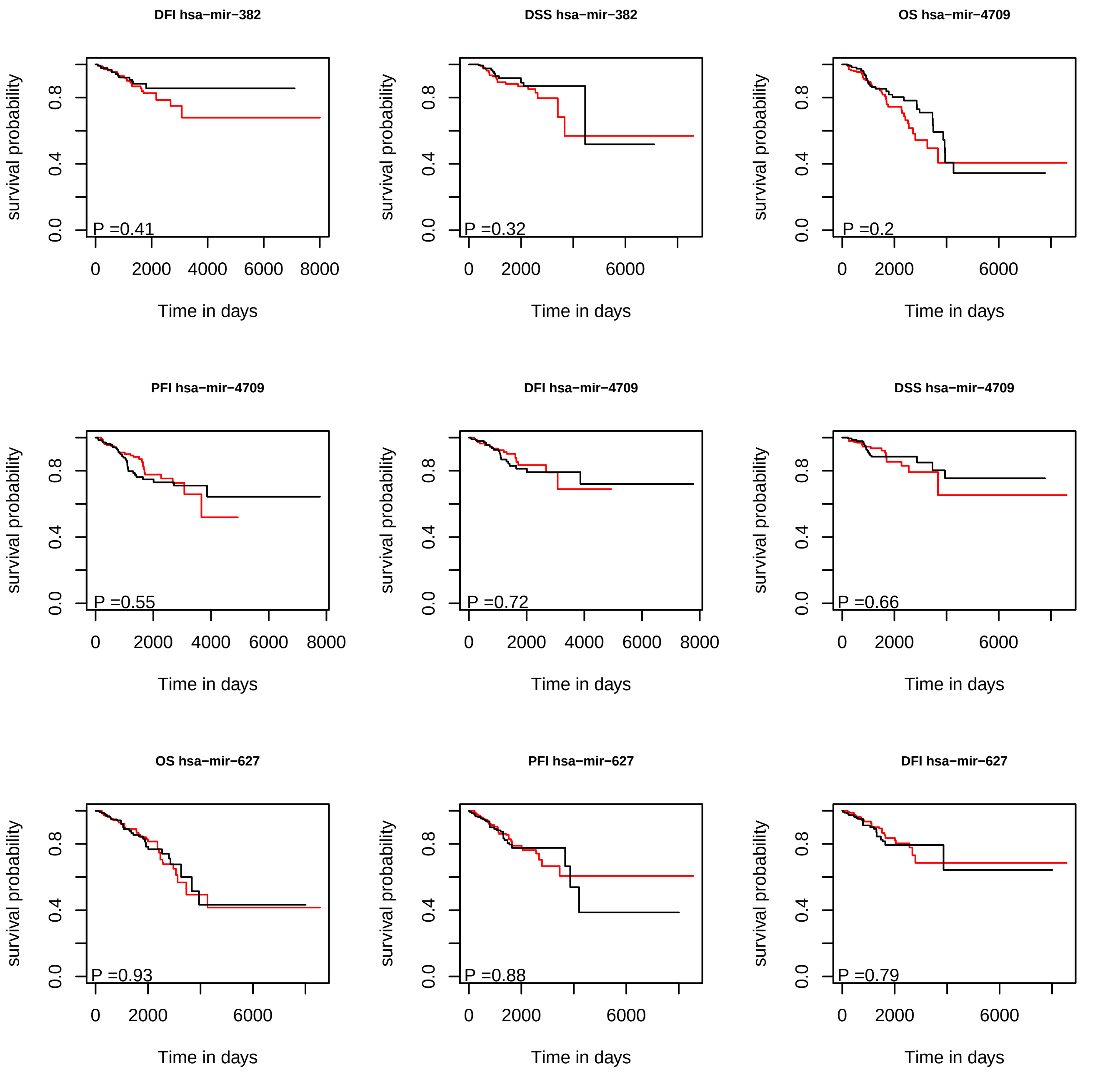
DFI hsa−mir−382
P =0.41
DSS hsa−mir−382
P =0.32
OS hsa−mir−4709
P =0.2
PFI hsa−mir−4709
P =0.55
DFI hsa−mir−4709
P =0.72
DSS hsa−mir−4709
P =0.66
OS hsa−mir−627
P =0.93
PFI hsa−mir−627
P =0.88
DFI hsa−mir−627
P =0.79
survival probability
Time in days
0.0
0.4
0.8
0
2000
4000
6000
8000

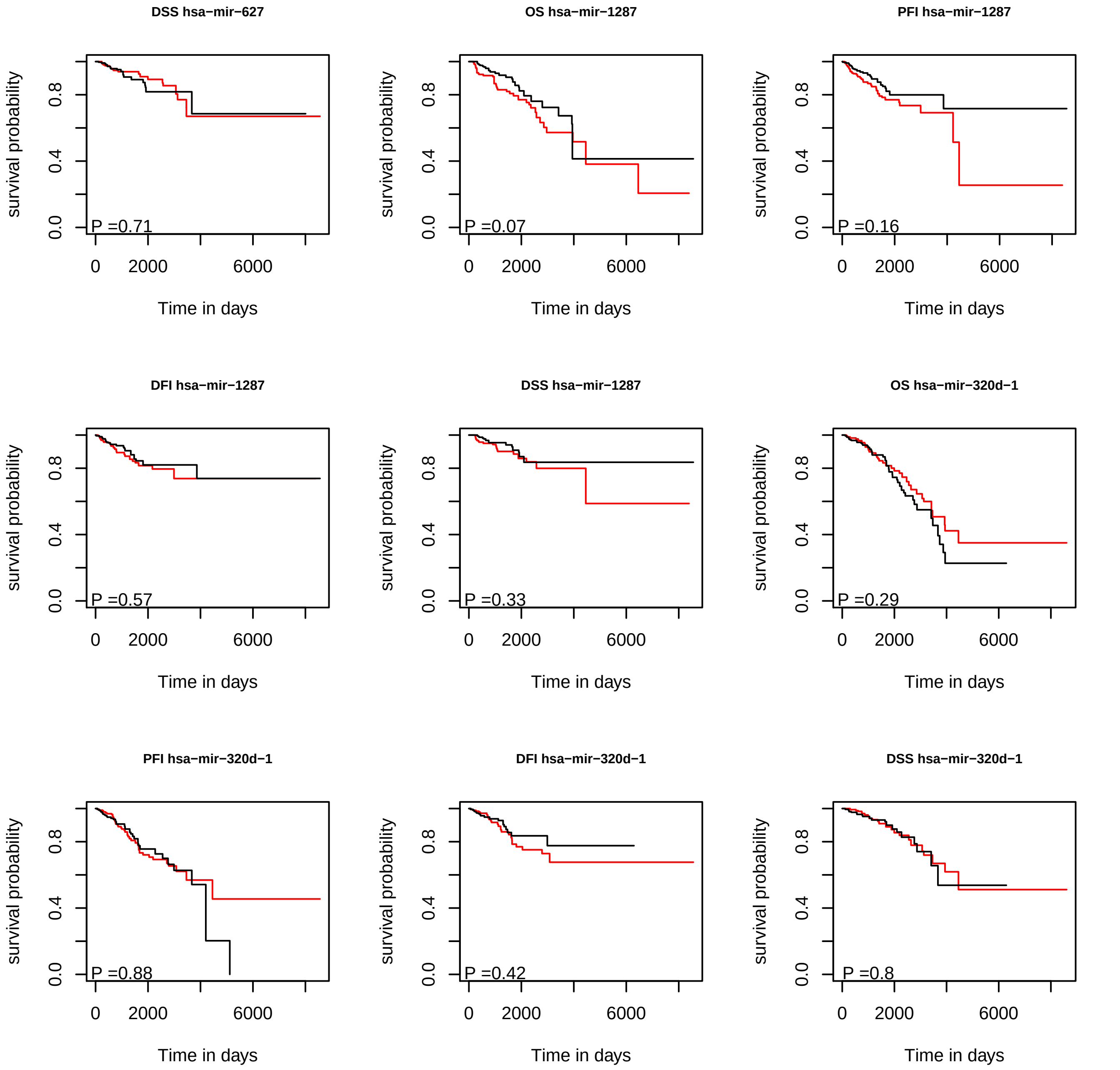
DSS hsa−mir−627
P =0.71
OS hsa−mir−1287
P =0.07
PFI hsa−mir−1287
P =0.16
DFI hsa−mir−1287
P =0.57
DSS hsa−mir−1287
P =0.33
OS hsa−mir−320d−1
P =0.29
PFI hsa−mir−320d−1
P =0.88
DFI hsa−mir−320d−1
P =0.42
DSS hsa−mir−320d−1
P =0.8
survival probability
Time in days
0.0
0.4
0.8
0
2000
6000

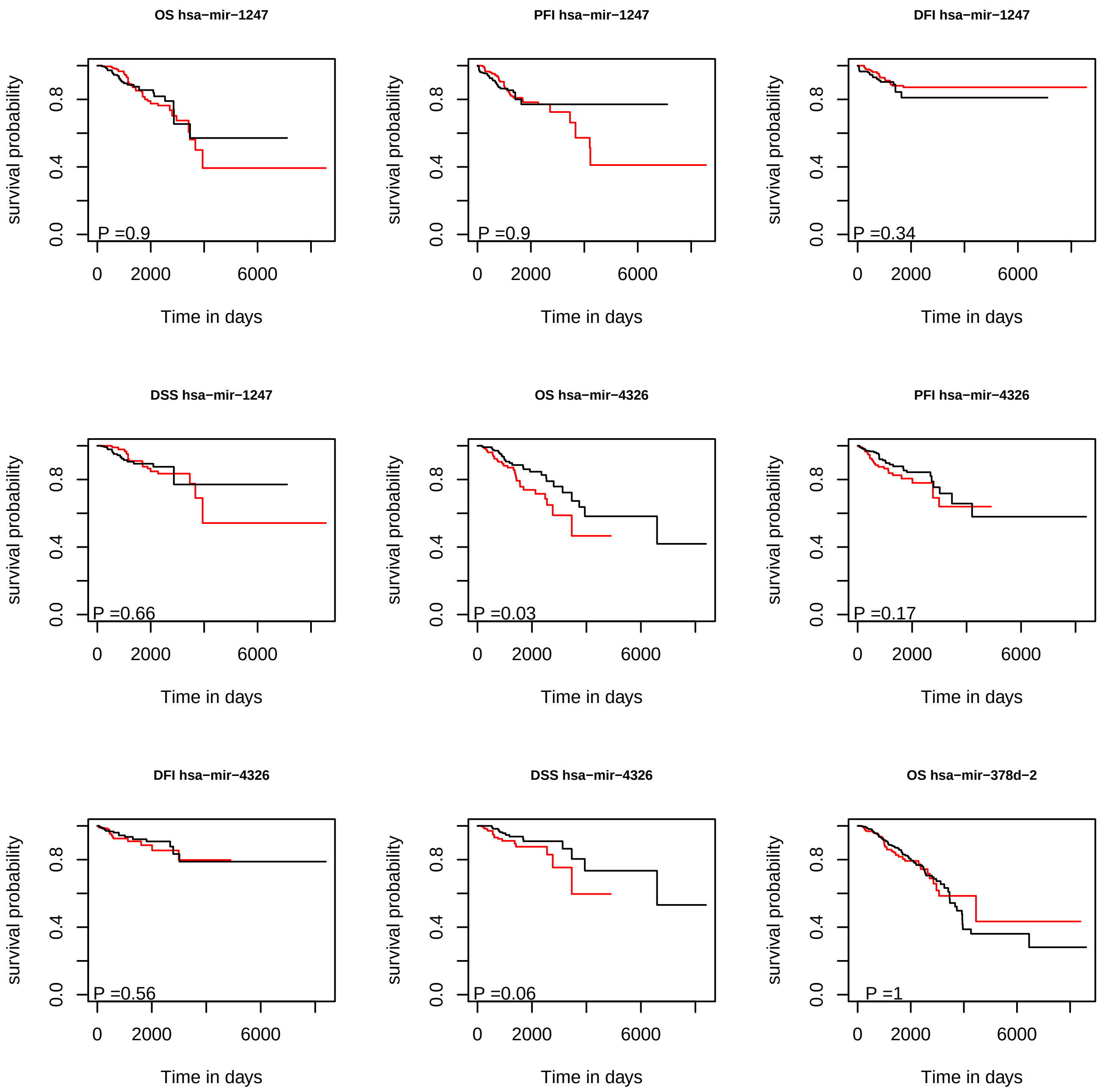
OS hsa−mir−1247
P =0.9
PFI hsa−mir−1247
P =0.9
DFI hsa−mir−1247
P =0.34
DSS hsa−mir−1247
P =0.66
OS hsa−mir−4326
P =0.03
PFI hsa−mir−4326
P =0.17
DFI hsa−mir−4326
P =0.56
DSS hsa−mir−4326
P =0.06
OS hsa−mir−378d−2
P =1
survival probability
Time in days
0.0
0.4
0.8
0
2000
6000

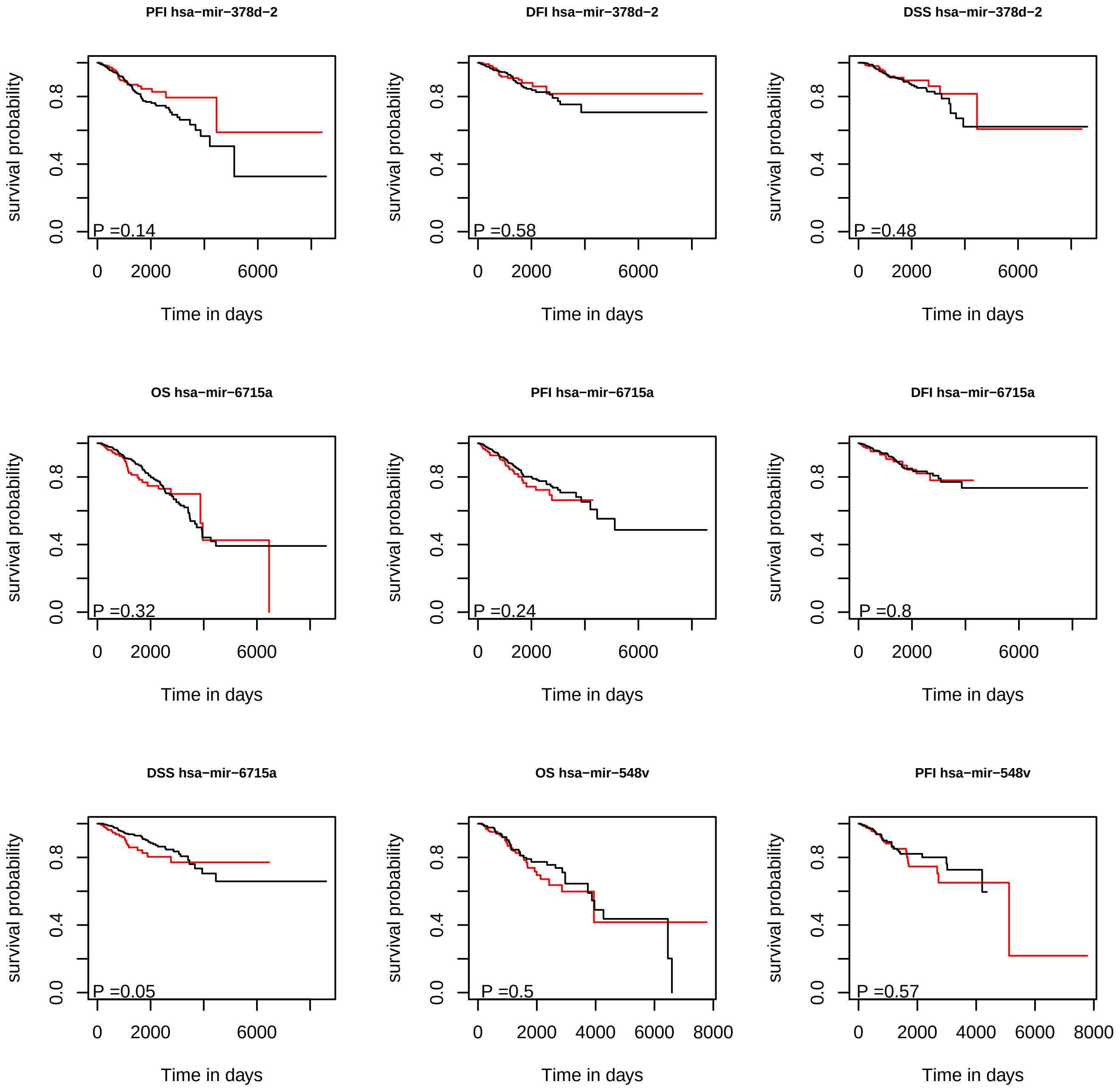
PFI hsa−mir−378d−2
P =0.14
DFI hsa−mir−378d−2
P =0.58
DSS hsa−mir−378d−2
P =0.48
OS hsa−mir−6715a
P =0.32
PFI hsa−mir−6715a
P =0.24
DFI hsa−mir−6715a
P =0.8
DSS hsa−mir−6715a
P =0.05
OS hsa−mir−548v
P =0.5
PFI hsa−mir−548v
P =0.57
survival probability
Time in days
0.0
0.4
0.8
0
2000
4000
6000
8000

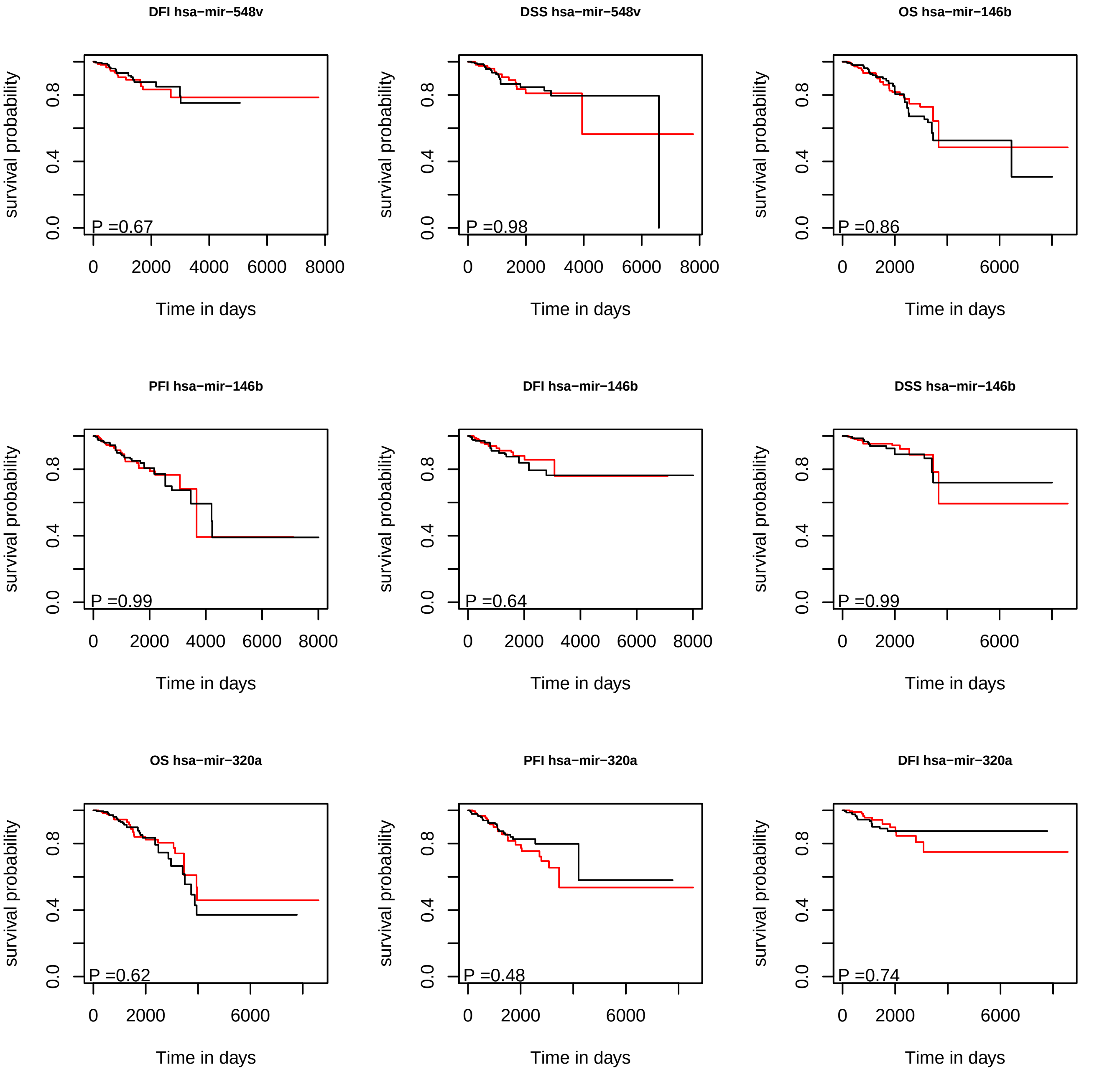
DFI hsa−mir−548v
P =0.67
DSS hsa−mir−548v
P =0.98
OS hsa−mir−146b
P =0.86
PFI hsa−mir−146b
P =0.99
DFI hsa−mir−146b
P =0.64
DSS hsa−mir−146b
P =0.99
OS hsa−mir−320a
P =0.62
PFI hsa−mir−320a
P =0.48
DFI hsa−mir−320a
P =0.74
survival probability
Time in days
0.0
0.4
0.8
0
2000
4000
6000
8000

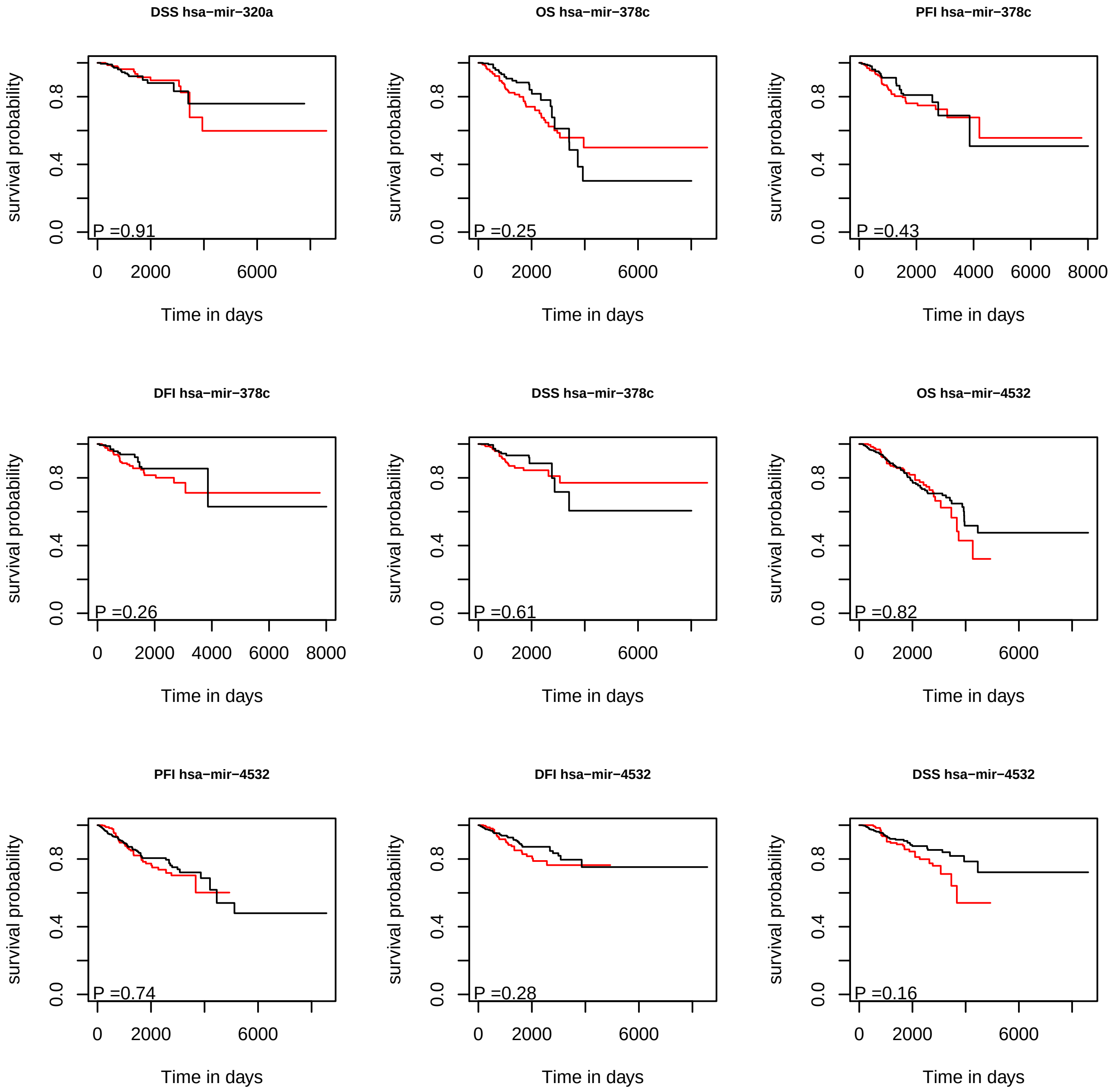
DSS hsa−mir−320a
P =0.91
OS hsa−mir−378c
P =0.25
PFI hsa−mir−378c
P =0.43
DFI hsa−mir−378c
P =0.26
DSS hsa−mir−378c
P =0.61
OS hsa−mir−4532
P =0.82
PFI hsa−mir−4532
P =0.74
DFI hsa−mir−4532
P =0.28
DSS hsa−mir−4532
P =0.16
survival probability
Time in days
0.0
0.4
0.8
0
2000
4000
6000
8000

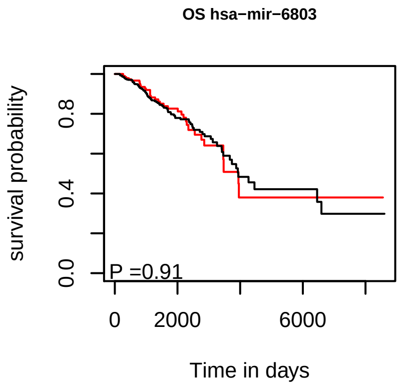
OS hsa−mir−6803
survival probability
0.0 0.4 0.8
P =0.91
0 2000 6000
Time in days

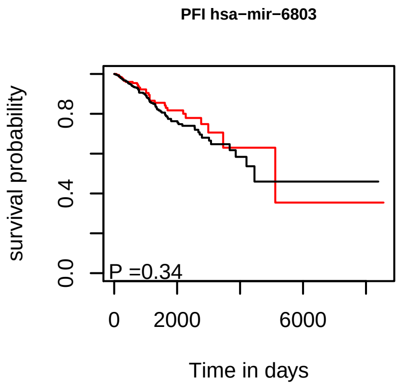
PFI hsa−mir−6803
survival probability
0.0 0.4 0.8
P =0.34
0 2000 6000
Time in days

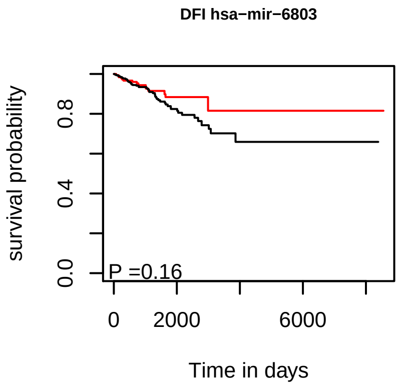
DFI hsa−mir−6803
survival probability
0.0 0.4 0.8
P =0.16
0 2000 6000
Time in days

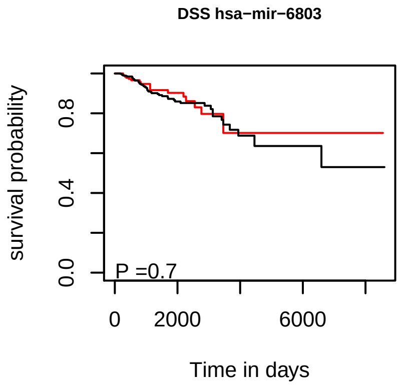
DSS hsa−mir−6803
survival probability
0.0 0.4 0.8
P =0.7
0 2000 6000
Time in days

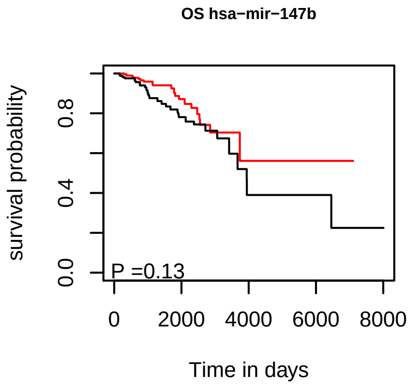
OS hsa−mir−147b
survival probability
0.0 0.4 0.8
P =0.13
0 2000 4000 6000 8000
Time in days

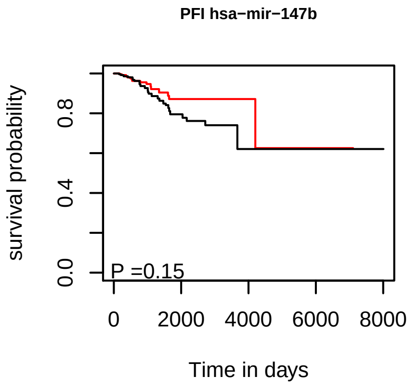
PFI hsa−mir−147b
survival probability
0.0 0.4 0.8
P =0.15
0 2000 4000 6000 8000
Time in days

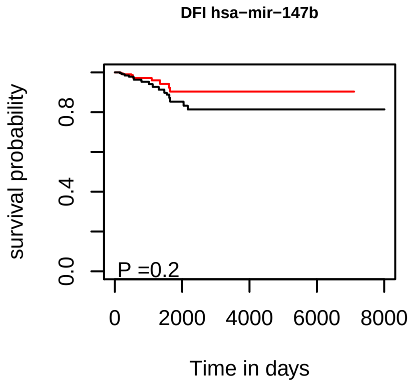
DFI hsa−mir−147b
survival probability
0.0 0.4 0.8
P =0.2
0 2000 4000 6000 8000
Time in days

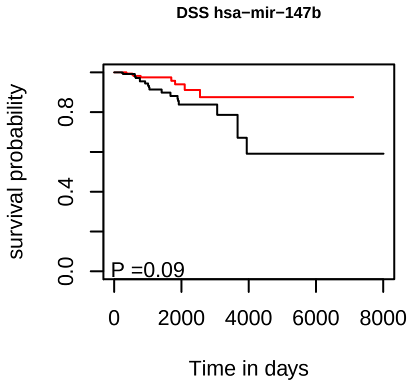
DSS hsa−mir−147b
survival probability
0.0 0.4 0.8
P =0.09
0 2000 4000 6000 8000
Time in days

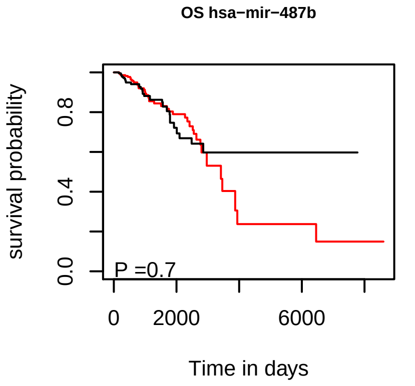
OS hsa−mir−487b
survival probability
0.0 0.4 0.8
P =0.7
0 2000 6000
Time in days

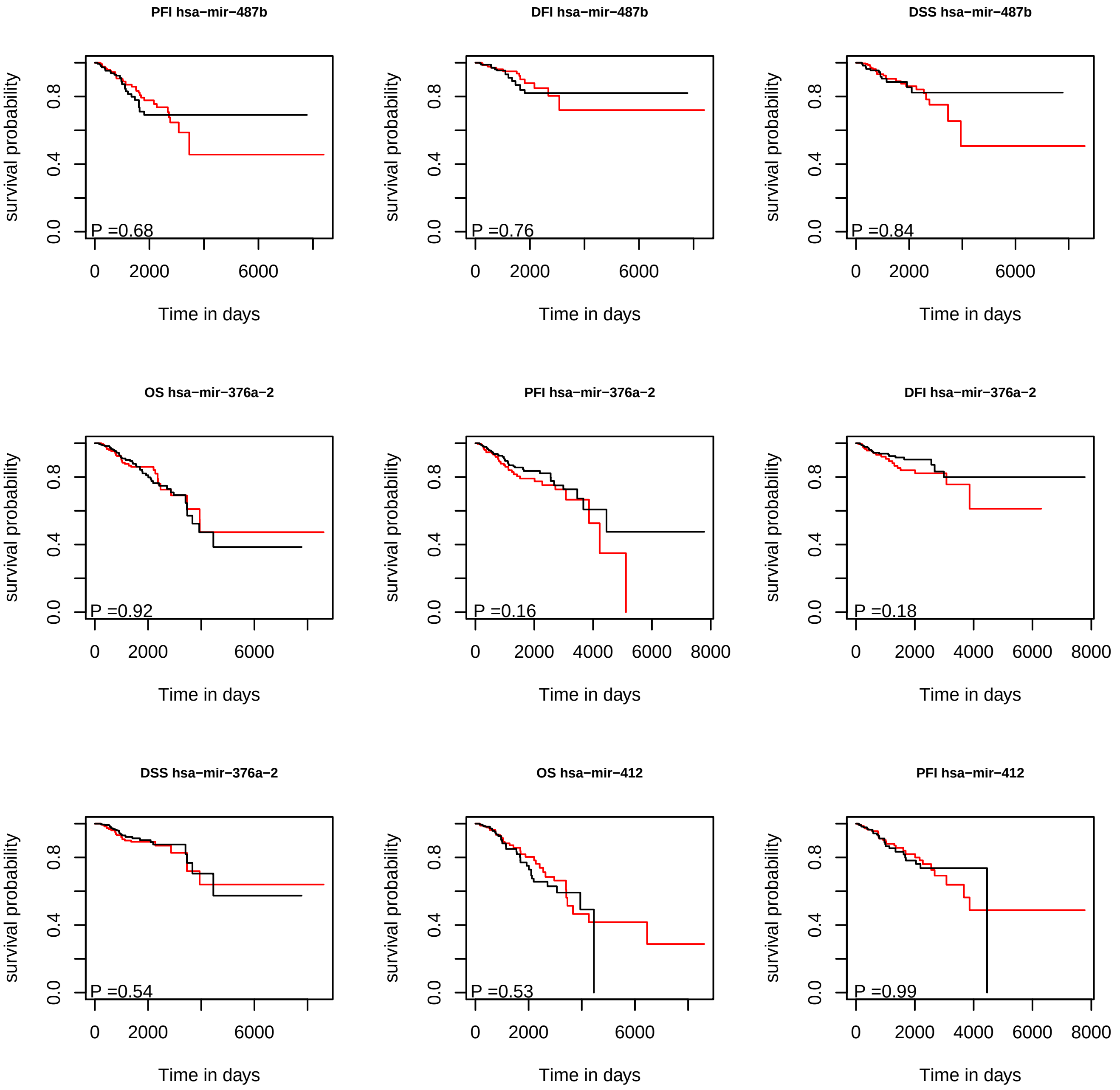
PFI hsa−mir−487b
P =0.68
DFI hsa−mir−487b
P =0.76
DSS hsa−mir−487b
P =0.84
OS hsa−mir−376a−2
P =0.92
PFI hsa−mir−376a−2
P =0.16
DFI hsa−mir−376a−2
P =0.18
DSS hsa−mir−376a−2
P =0.54
OS hsa−mir−412
P =0.53
PFI hsa−mir−412
P =0.99
survival probability
Time in days
0.0
0.4
0.8
0
2000
4000
6000
8000

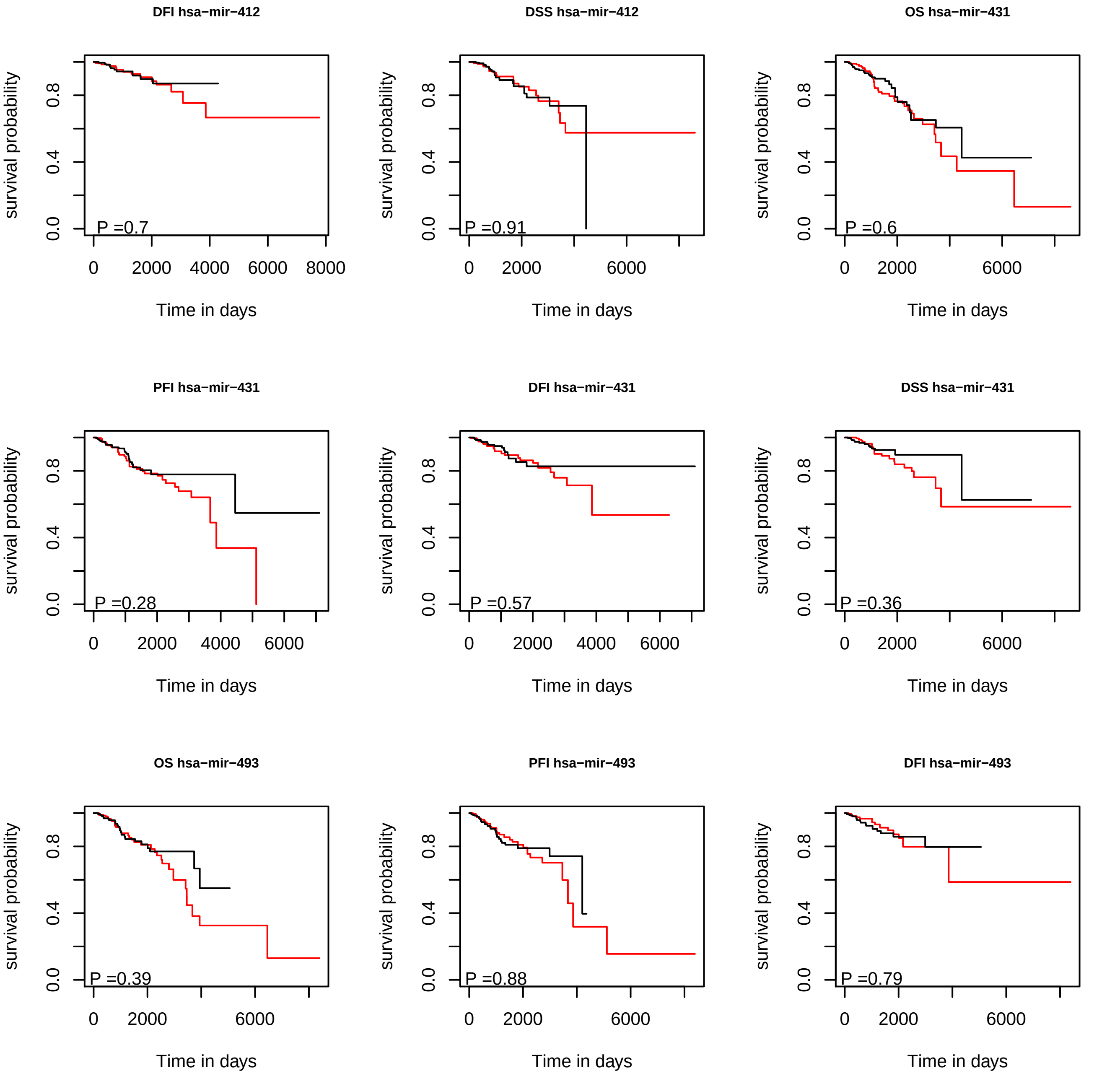
DFI hsa−mir−412
survival probability
0.0
0.4
0.8
P =0.7
0
2000
4000
6000
8000
Time in days
DSS hsa−mir−412
survival probability
0.0
0.4
0.8
P =0.91
0
2000
6000
Time in days
OS hsa−mir−431
survival probability
0.0
0.4
0.8
P =0.6
0
2000
6000
Time in days
PFI hsa−mir−431
survival probability
0.0
0.4
0.8
P =0.28
0
2000
4000
6000
Time in days
DFI hsa−mir−431
survival probability
0.0
0.4
0.8
P =0.57
0
2000
4000
6000
Time in days
DSS hsa−mir−431
survival probability
0.0
0.4
0.8
P =0.36
0
2000
6000
Time in days
OS hsa−mir−493
survival probability
0.0
0.4
0.8
P =0.39
0
2000
6000
Time in days
PFI hsa−mir−493
survival probability
0.0
0.4
0.8
P =0.88
0
2000
6000
Time in days
DFI hsa−mir−493
survival probability
0.0
0.4
0.8
P =0.79
0
2000
6000
Time in days

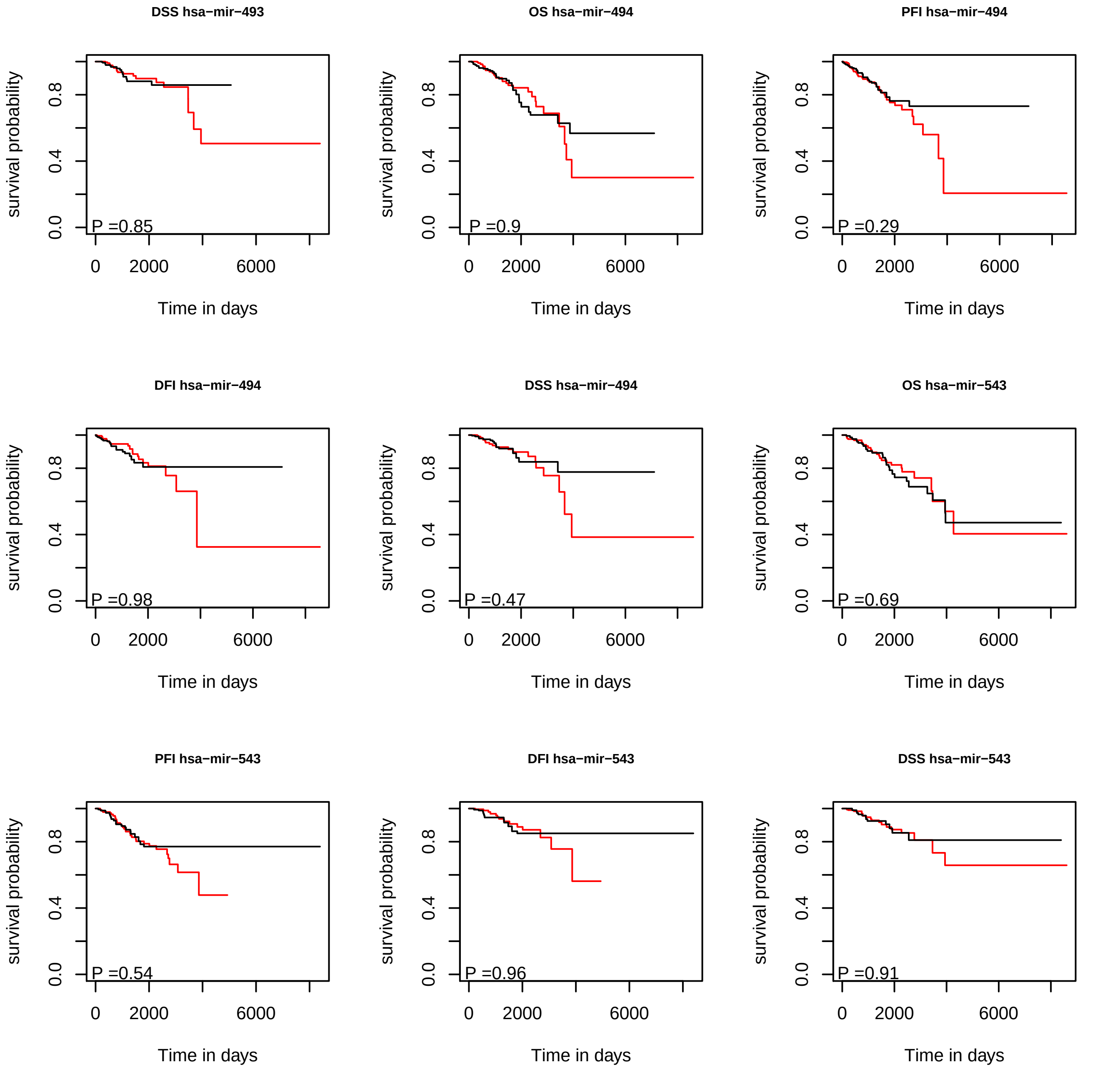
DSS hsa−mir−493
P =0.85
OS hsa−mir−494
P =0.9
PFI hsa−mir−494
P =0.29
DFI hsa−mir−494
P =0.98
DSS hsa−mir−494
P =0.47
OS hsa−mir−543
P =0.69
PFI hsa−mir−543
P =0.54
DFI hsa−mir−543
P =0.96
DSS hsa−mir−543
P =0.91
survival probability
Time in days
0.0
0.4
0.8
0
2000
6000

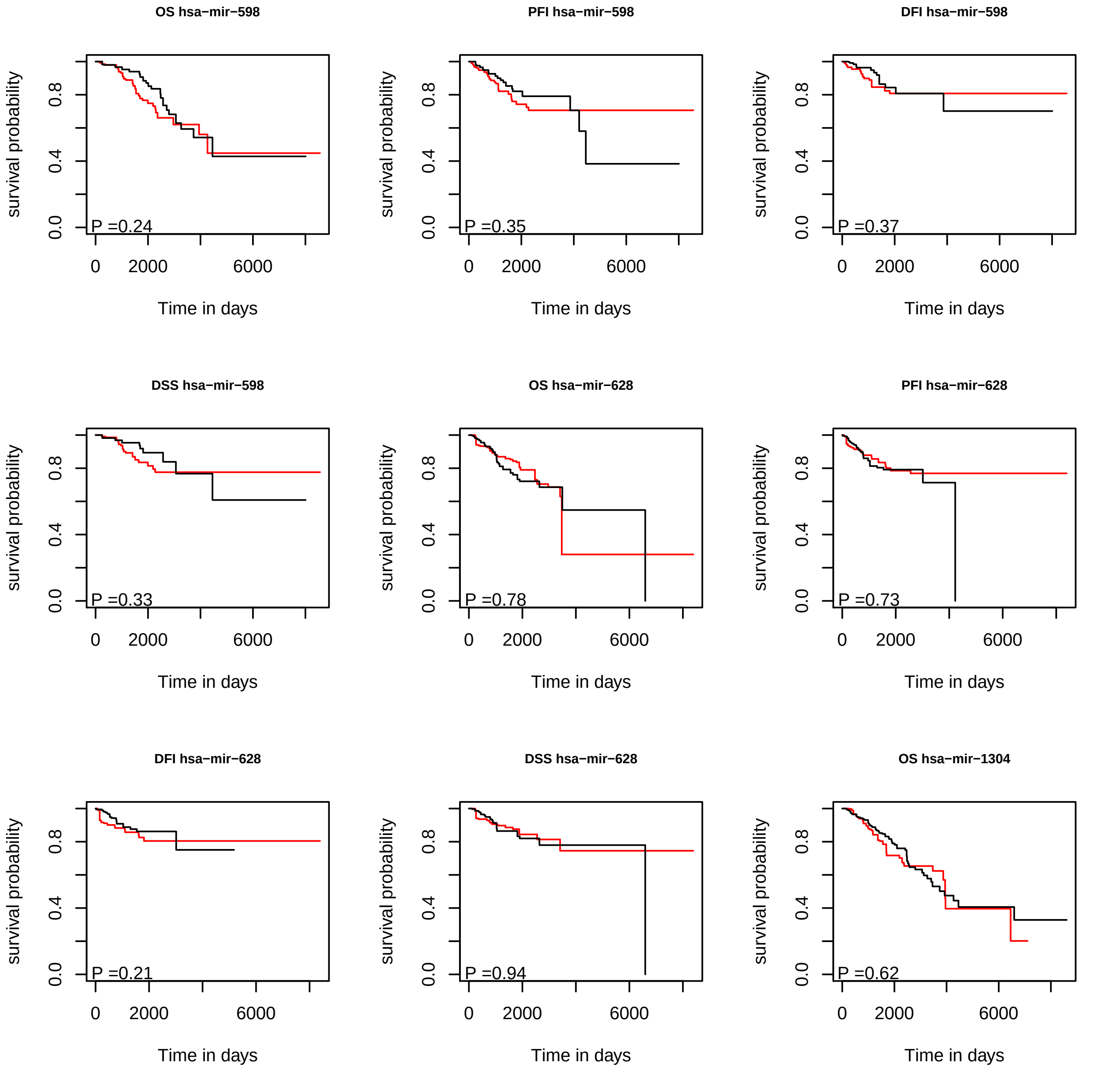
OS hsa−mir−598
P =0.24
PFI hsa−mir−598
P =0.35
DFI hsa−mir−598
P =0.37
DSS hsa−mir−598
P =0.33
OS hsa−mir−628
P =0.78
PFI hsa−mir−628
P =0.73
DFI hsa−mir−628
P =0.21
DSS hsa−mir−628
P =0.94
OS hsa−mir−1304
P =0.62
survival probability
0.0
0.4
0.8
0
2000
6000
Time in days

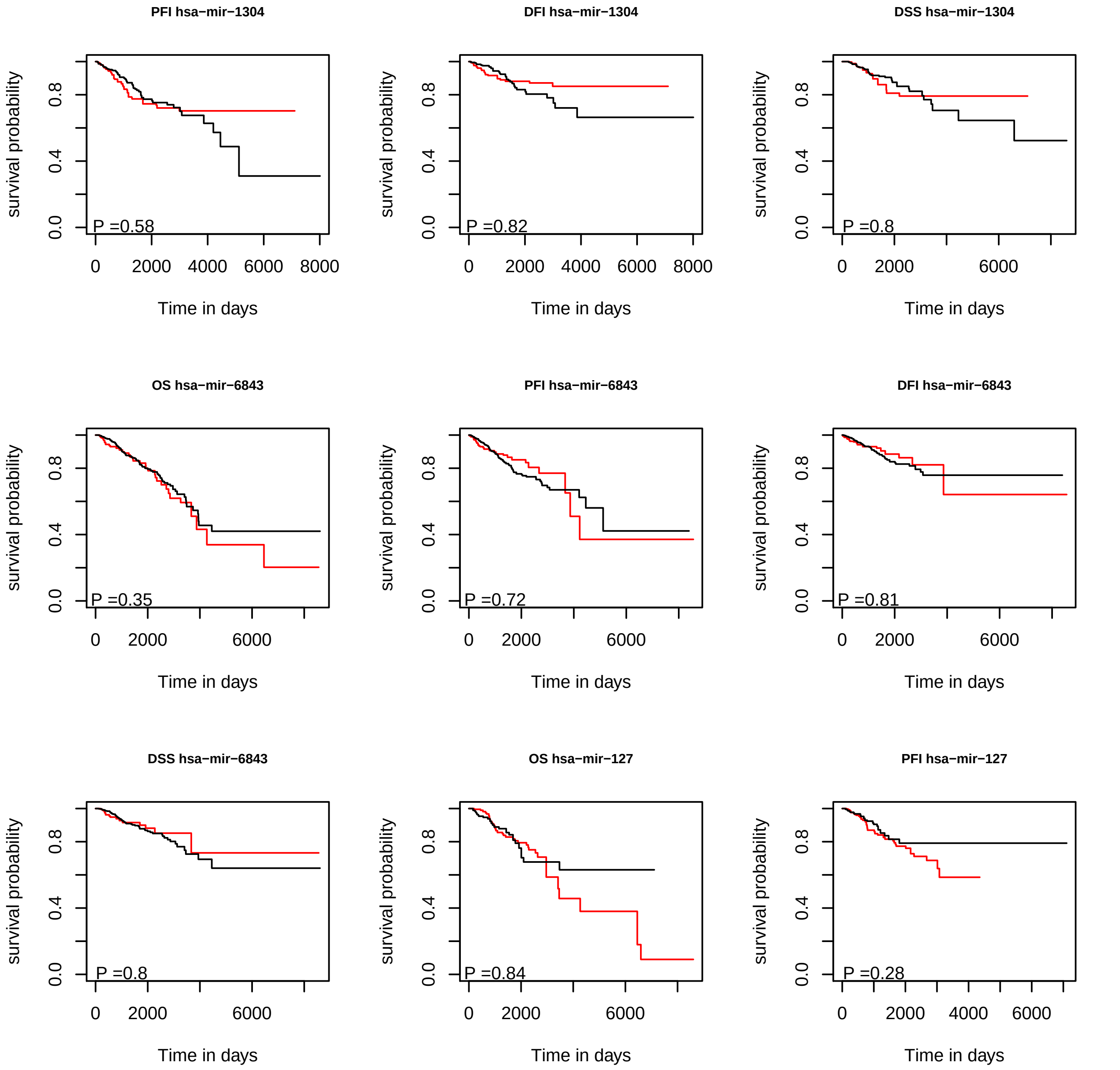
PFI hsa−mir−1304
P =0.58
DFI hsa−mir−1304
P =0.82
DSS hsa−mir−1304
P =0.8
OS hsa−mir−6843
P =0.35
PFI hsa−mir−6843
P =0.72
DFI hsa−mir−6843
P =0.81
DSS hsa−mir−6843
P =0.8
OS hsa−mir−127
P =0.84
PFI hsa−mir−127
P =0.28
survival probability
Time in days

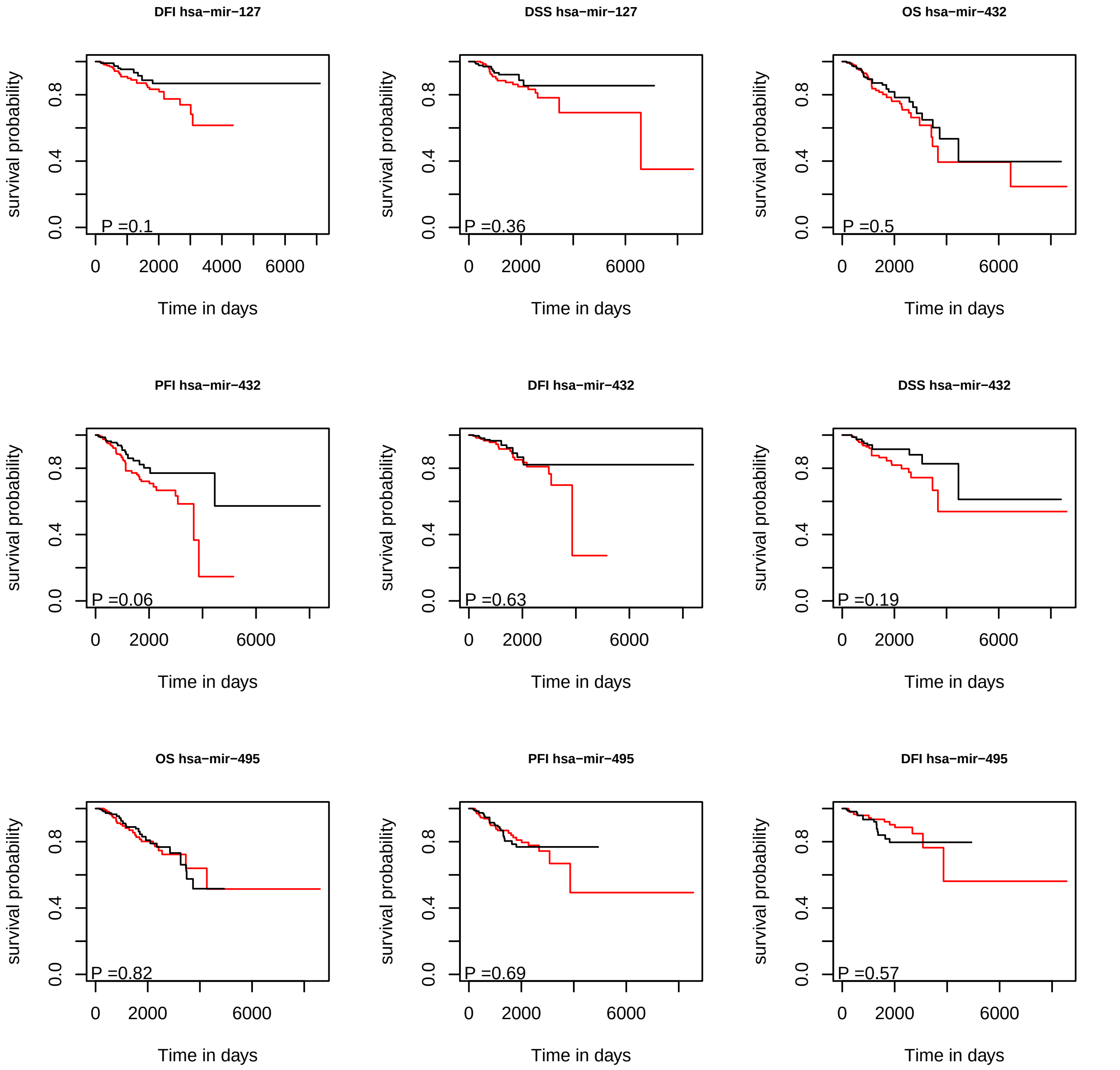
DFI hsa−mir−127
P =0.1
DSS hsa−mir−127
P =0.36
OS hsa−mir−432
P =0.5
PFI hsa−mir−432
P =0.06
DFI hsa−mir−432
P =0.63
DSS hsa−mir−432
P =0.19
OS hsa−mir−495
P =0.82
PFI hsa−mir−495
P =0.69
DFI hsa−mir−495
P =0.57
survival probability
Time in days

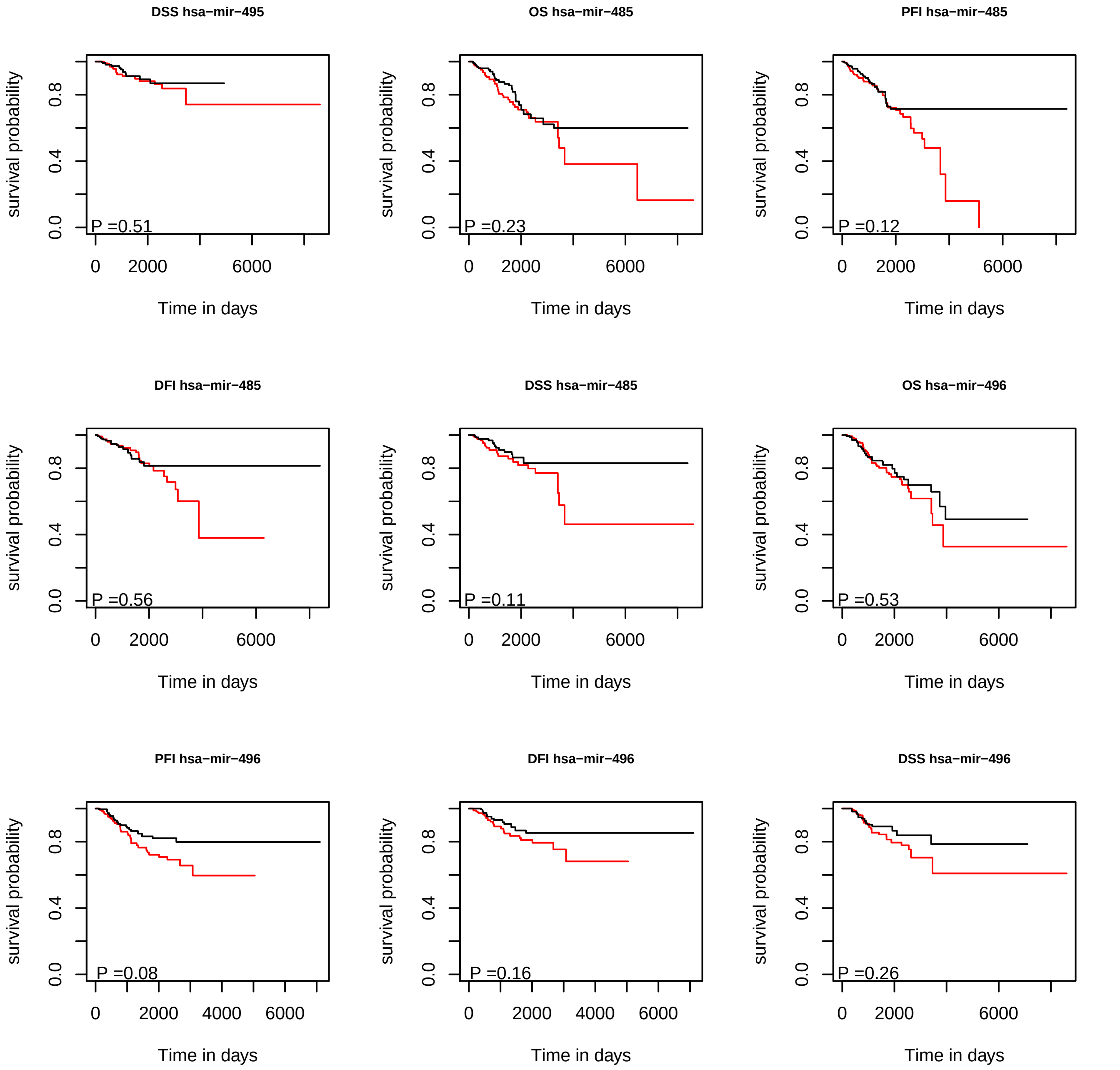
DSS hsa−mir−495
P =0.51
OS hsa−mir−485
P =0.23
PFI hsa−mir−485
P =0.12
DFI hsa−mir−485
P =0.56
DSS hsa−mir−485
P =0.11
OS hsa−mir−496
P =0.53
PFI hsa−mir−496
P =0.08
DFI hsa−mir−496
P =0.16
DSS hsa−mir−496
P =0.26
survival probability
Time in days
0.0
0.4
0.8
0
2000
4000
6000

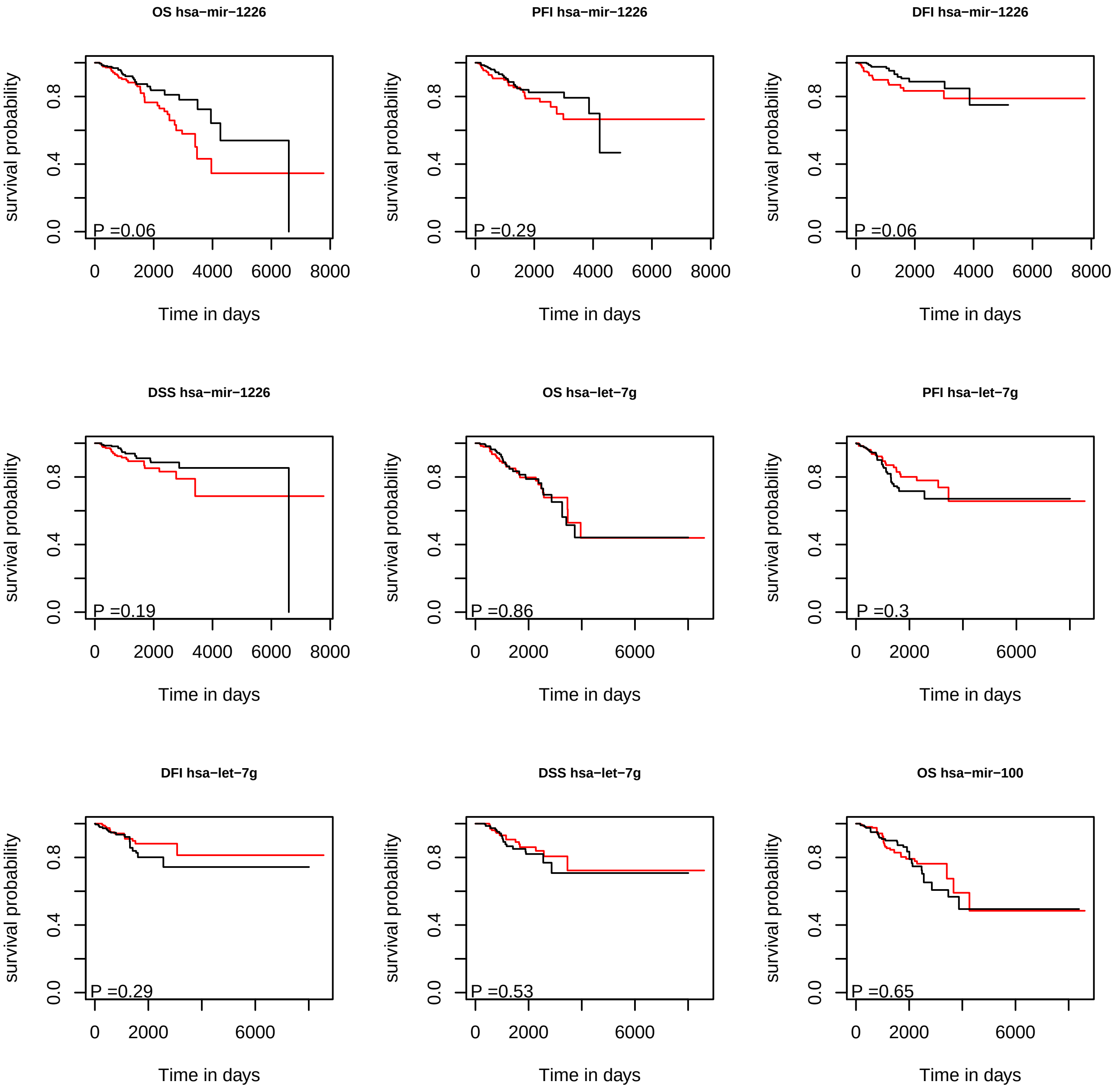

OS hsa−mir−1226
P =0.06
PFI hsa−mir−1226
P =0.29
DFI hsa−mir−1226
P =0.06
DSS hsa−mir−1226
P =0.19
OS hsa−let−7g
P =0.86
PFI hsa−let−7g
P =0.3
DFI hsa−let−7g
P =0.29
DSS hsa−let−7g
P =0.53
OS hsa−mir−100
P =0.65
survival probability
Time in days

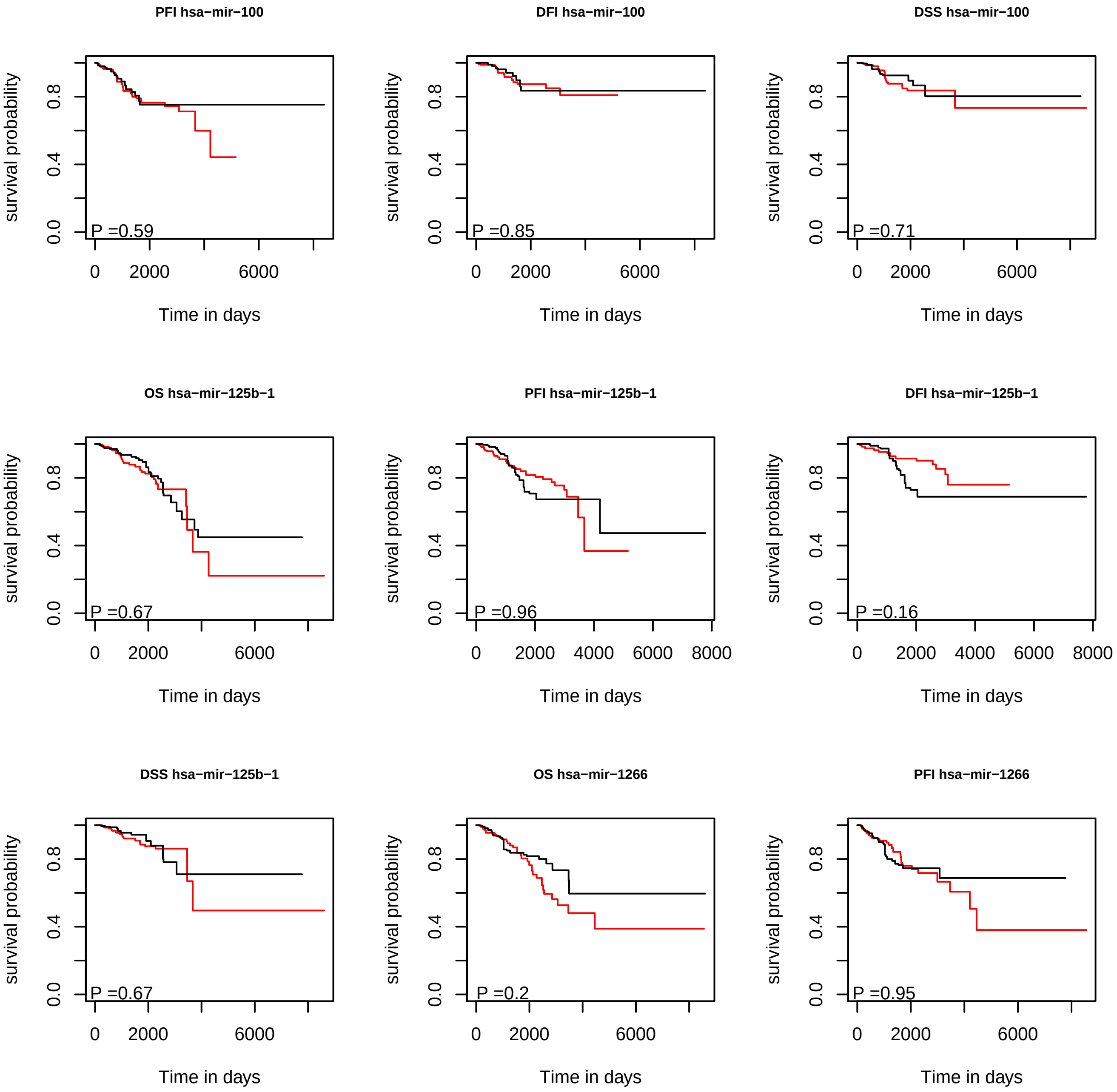
PFI hsa−mir−100
P =0.59
DFI hsa−mir−100
P =0.85
DSS hsa−mir−100
P =0.71
OS hsa−mir−125b−1
P =0.67
PFI hsa−mir−125b−1
P =0.96
DFI hsa−mir−125b−1
P =0.16
DSS hsa−mir−125b−1
P =0.67
OS hsa−mir−1266
P =0.2
PFI hsa−mir−1266
P =0.95
survival probability
Time in days

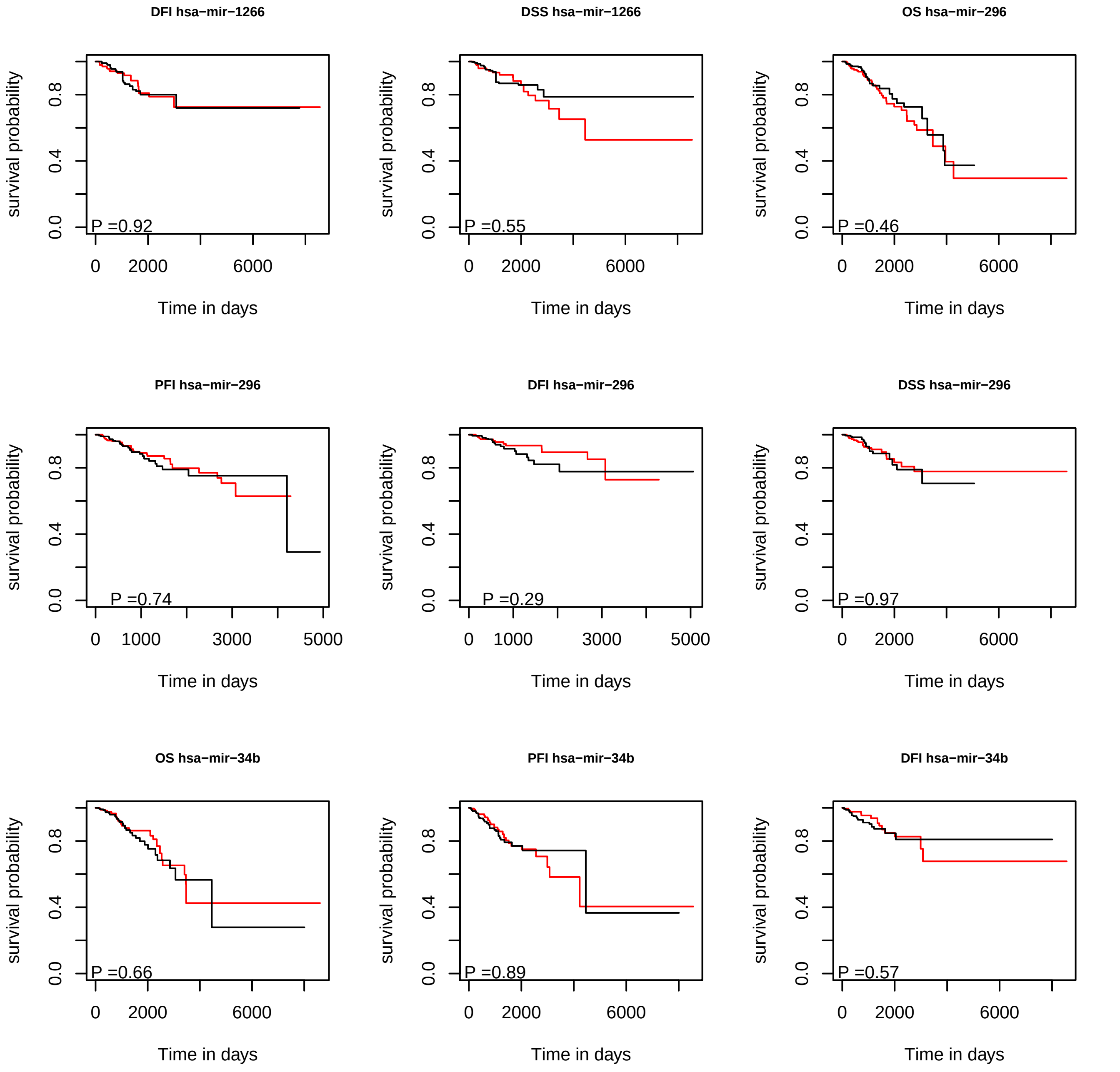

DFI hsa−mir−1266
P =0.92
DSS hsa−mir−1266
P =0.55
OS hsa−mir−296
P =0.46
PFI hsa−mir−296
P =0.74
DFI hsa−mir−296
P =0.29
DSS hsa−mir−296
P =0.97
OS hsa−mir−34b
P =0.66
PFI hsa−mir−34b
P =0.89
DFI hsa−mir−34b
P =0.57
survival probability
0.0
0.4
0.8
Time in days
0
1000
2000
3000
5000
6000

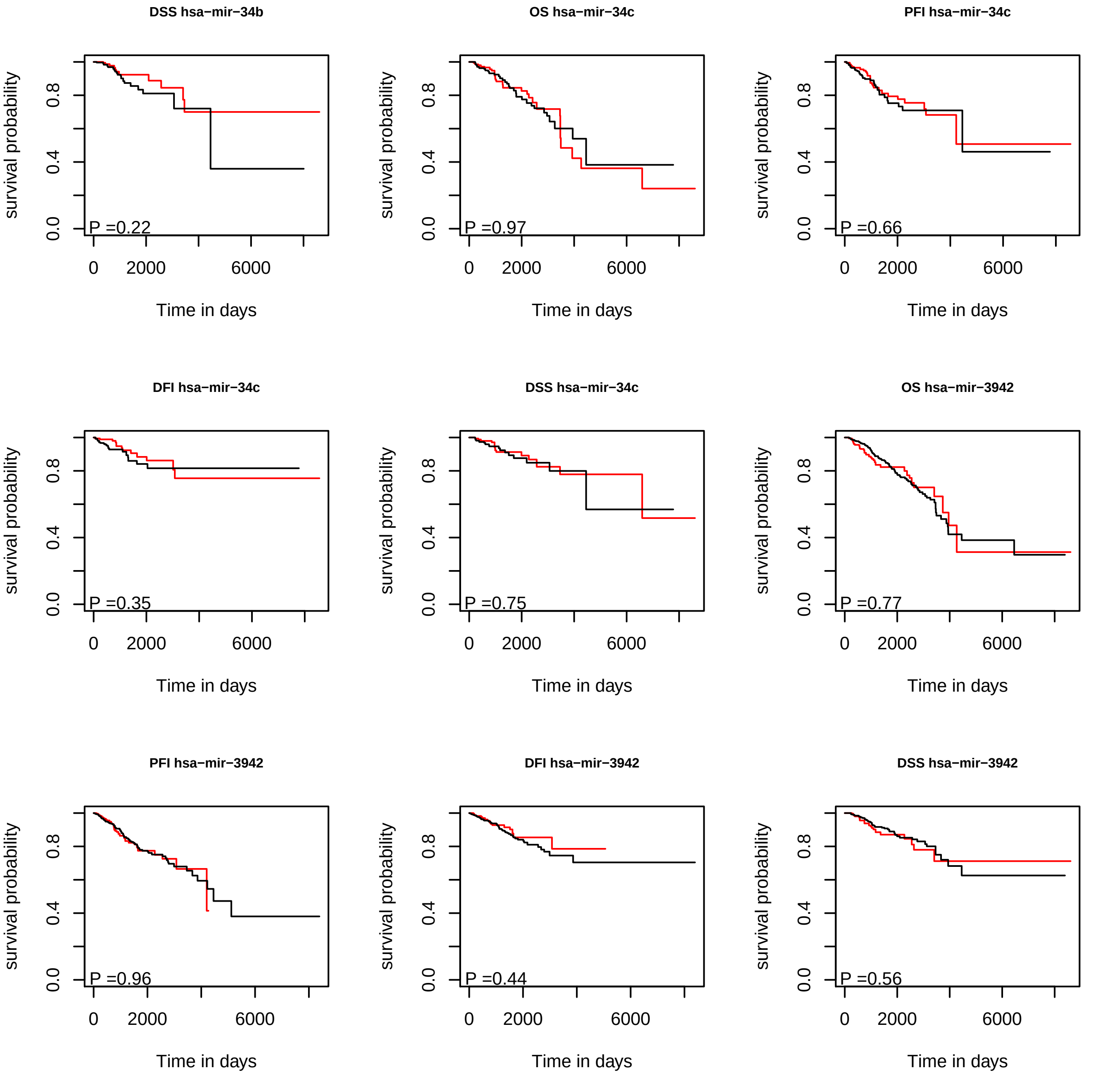
DSS hsa−mir−34b
P =0.22
OS hsa−mir−34c
P =0.97
PFI hsa−mir−34c
P =0.66
DFI hsa−mir−34c
P =0.35
DSS hsa−mir−34c
P =0.75
OS hsa−mir−3942
P =0.77
PFI hsa−mir−3942
P =0.96
DFI hsa−mir−3942
P =0.44
DSS hsa−mir−3942
P =0.56
survival probability
0.0
0.4
0.8
Time in days
0
2000
6000

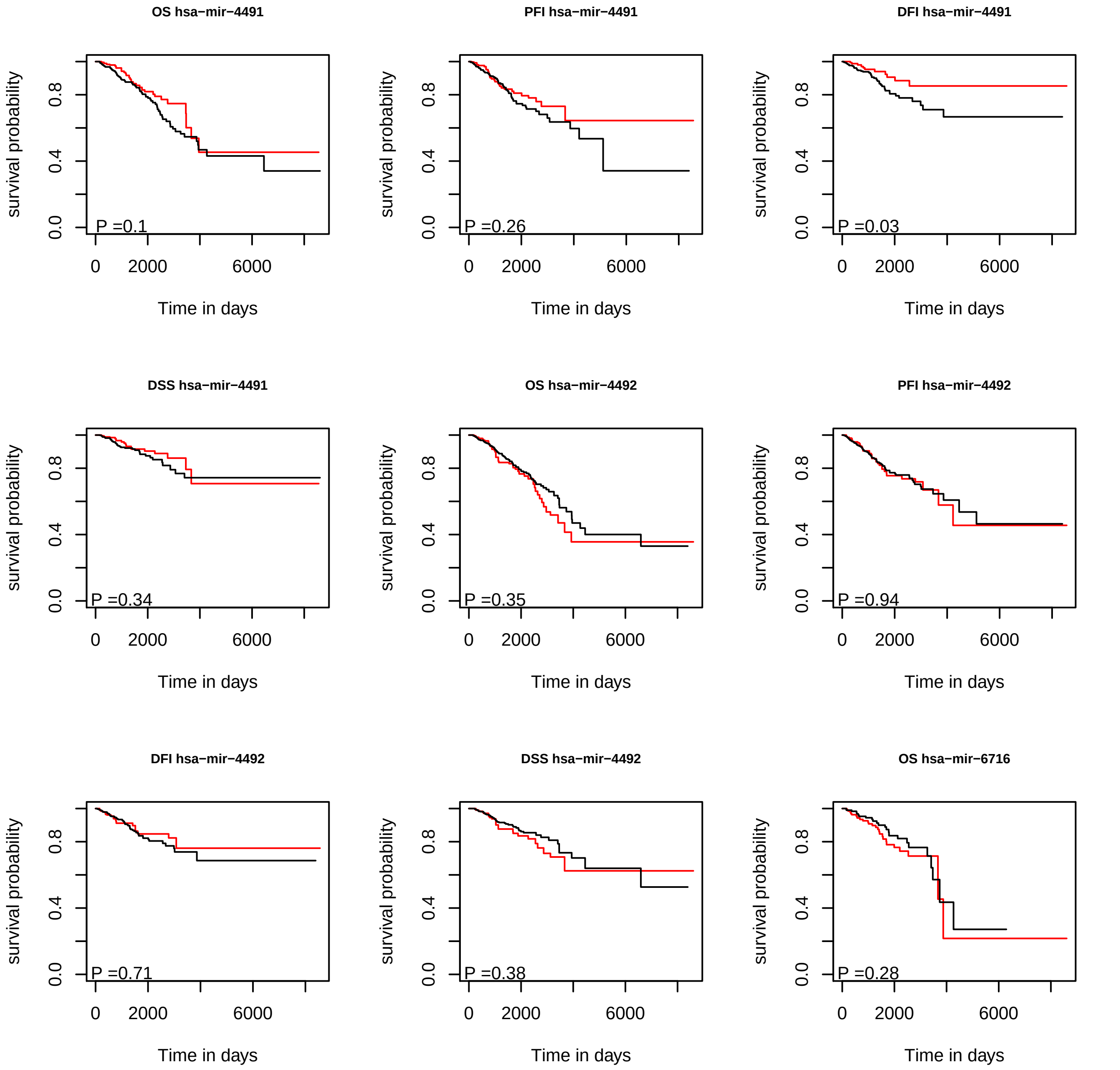

OS hsa−mir−4491
P =0.1
PFI hsa−mir−4491
P =0.26
DFI hsa−mir−4491
P =0.03
DSS hsa−mir−4491
P =0.34
OS hsa−mir−4492
P =0.35
PFI hsa−mir−4492
P =0.94
DFI hsa−mir−4492
P =0.71
DSS hsa−mir−4492
P =0.38
OS hsa−mir−6716
P =0.28
survival probability
0.0
0.4
0.8
0
2000
6000
Time in days

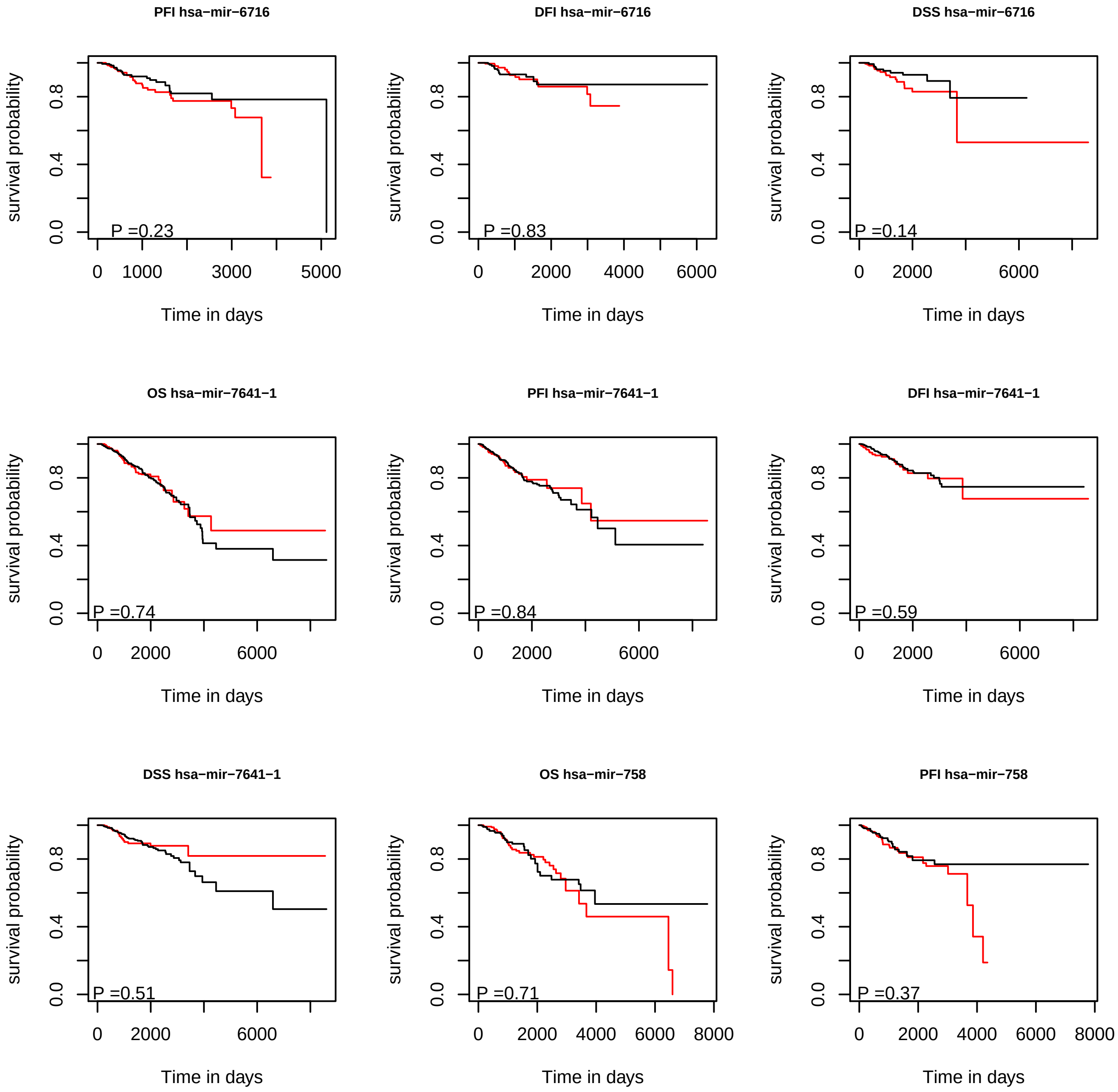
PFI hsa−mir−6716
P =0.23
DFI hsa−mir−6716
P =0.83
DSS hsa−mir−6716
P =0.14
OS hsa−mir−7641−1
P =0.74
PFI hsa−mir−7641−1
P =0.84
DFI hsa−mir−7641−1
P =0.59
DSS hsa−mir−7641−1
P =0.51
OS hsa−mir−758
P =0.71
PFI hsa−mir−758
P =0.37
survival probability
Time in days
0.0
0.4
0.8
0
1000
2000
3000
4000
5000
6000
8000

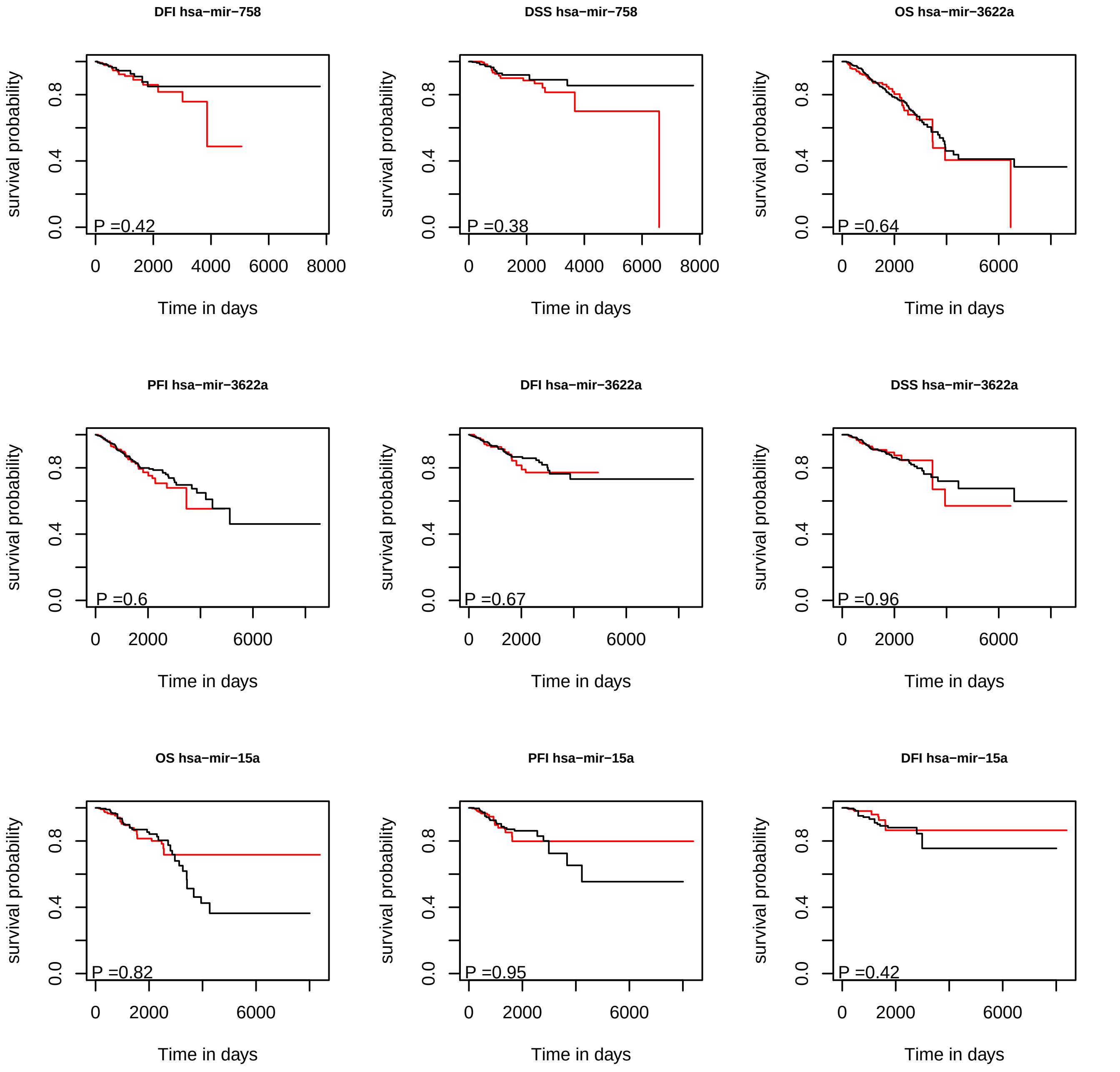
DFI hsa−mir−758
P =0.42
DSS hsa−mir−758
P =0.38
OS hsa−mir−3622a
P =0.64
PFI hsa−mir−3622a
P =0.6
DFI hsa−mir−3622a
P =0.67
DSS hsa−mir−3622a
P =0.96
OS hsa−mir−15a
P =0.82
PFI hsa−mir−15a
P =0.95
DFI hsa−mir−15a
P =0.42
survival probability
Time in days

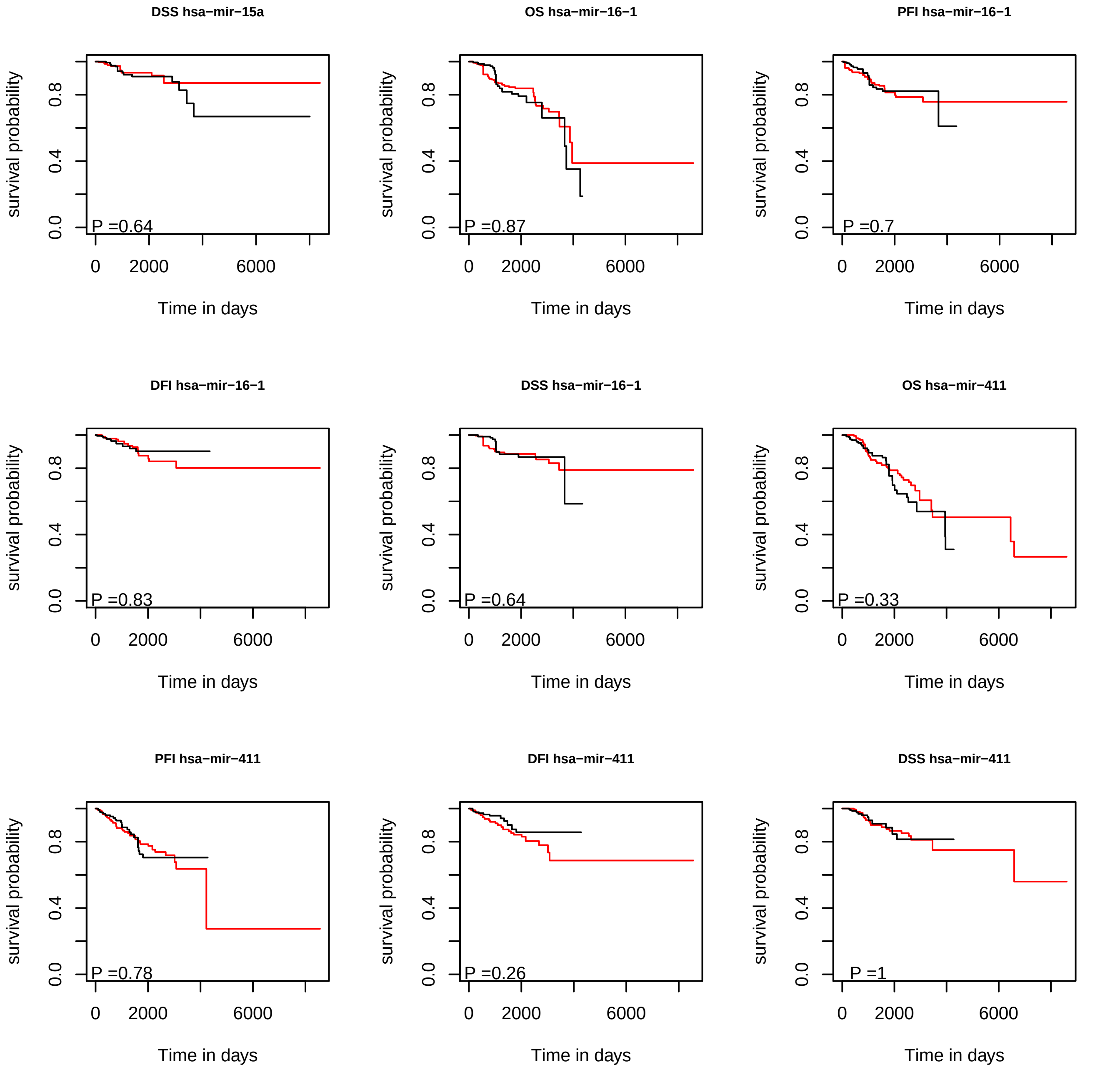
DSS hsa−mir−15a
P =0.64
OS hsa−mir−16−1
P =0.87
PFI hsa−mir−16−1
P =0.7
DFI hsa−mir−16−1
P =0.83
DSS hsa−mir−16−1
P =0.64
OS hsa−mir−411
P =0.33
PFI hsa−mir−411
P =0.78
DFI hsa−mir−411
P =0.26
DSS hsa−mir−411
P =1
survival probability
Time in days
0.0
0.4
0.8
0
2000
6000

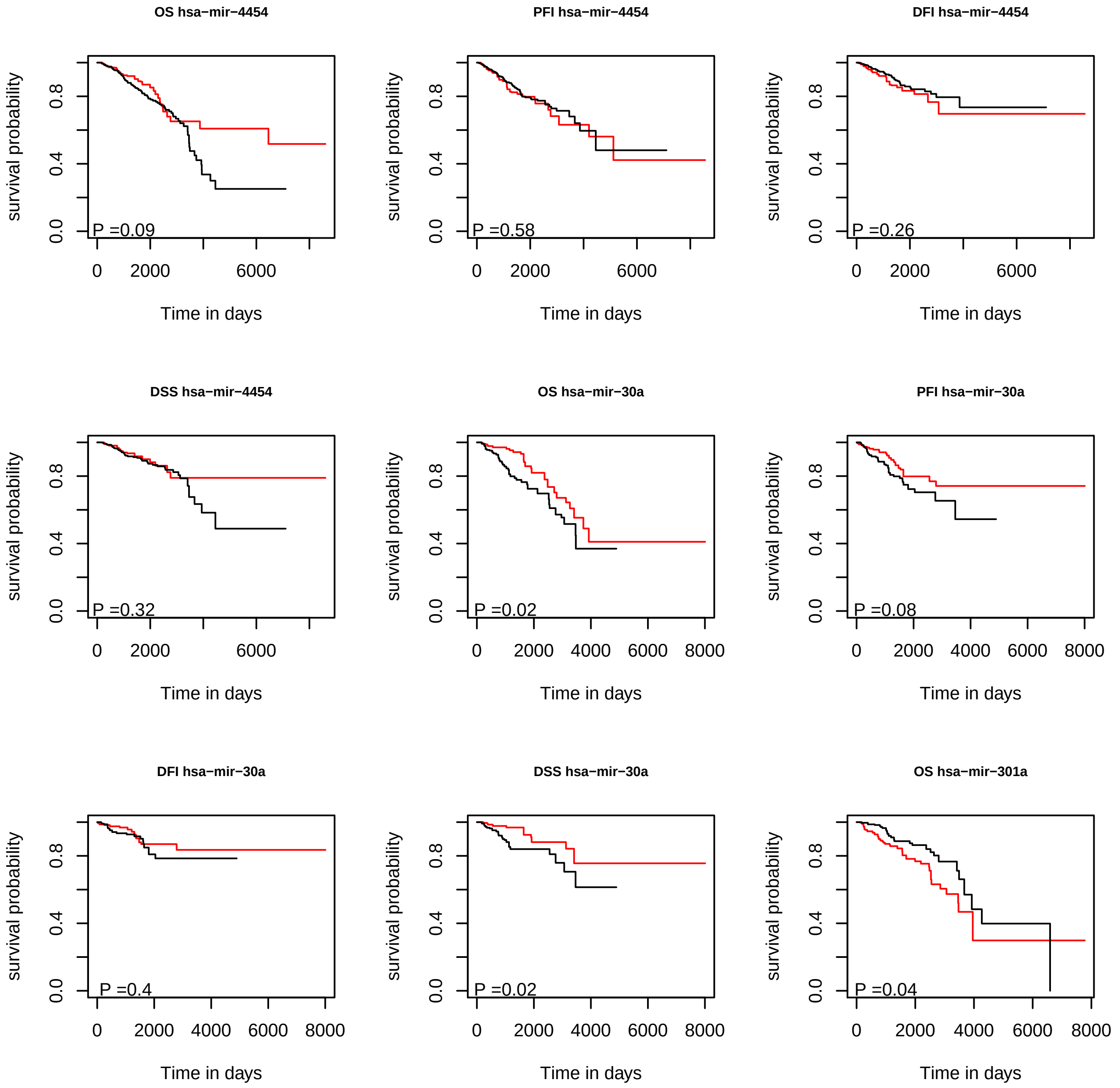
OS hsa−mir−4454
P =0.09
PFI hsa−mir−4454
P =0.58
DFI hsa−mir−4454
P =0.26
DSS hsa−mir−4454
P =0.32
OS hsa−mir−30a
P =0.02
PFI hsa−mir−30a
P =0.08
DFI hsa−mir−30a
P =0.4
DSS hsa−mir−30a
P =0.02
OS hsa−mir−301a
P =0.04
survival probability
Time in days

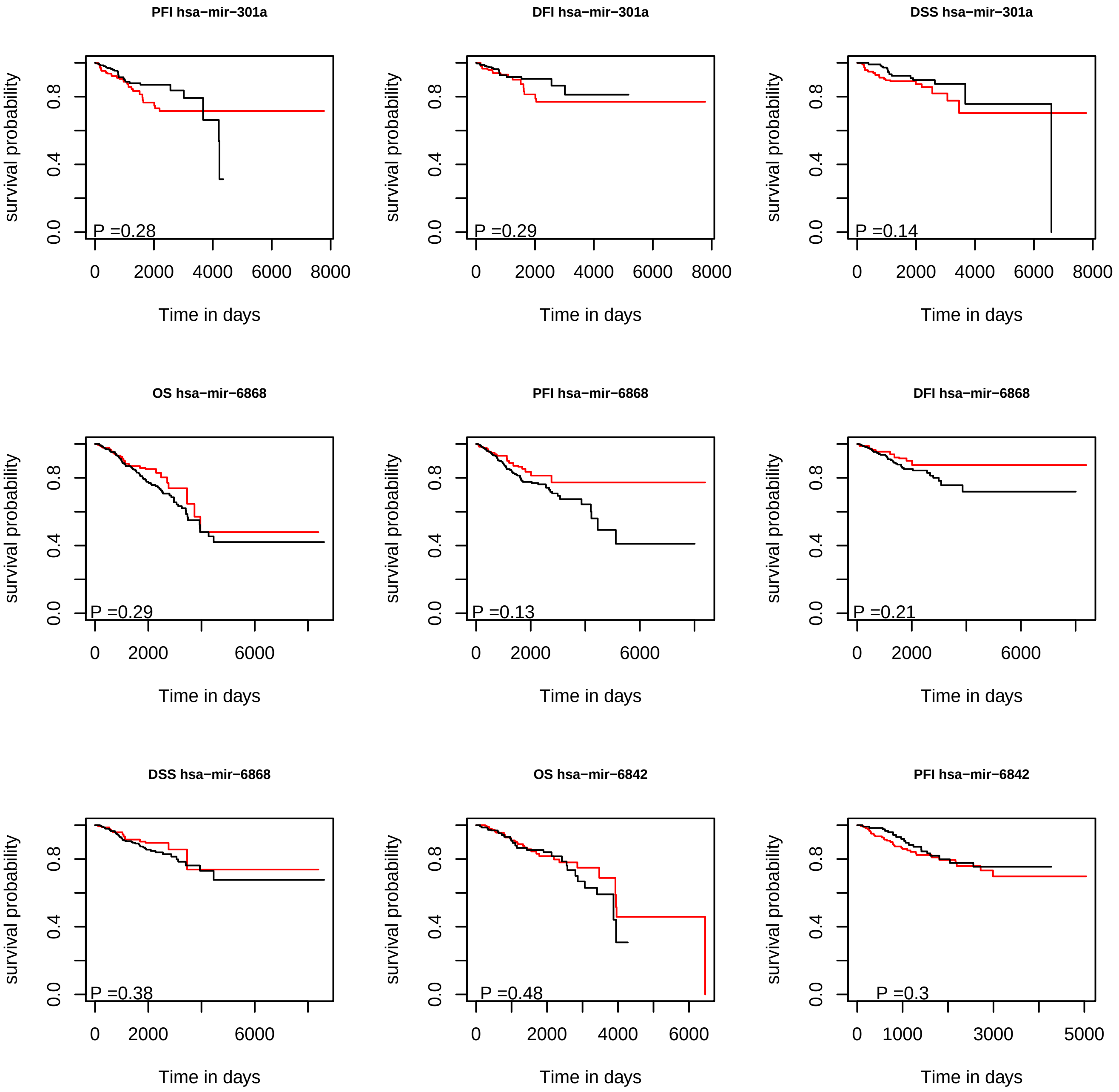
PFI hsa−mir−301a
P =0.28
DFI hsa−mir−301a
P =0.29
DSS hsa−mir−301a
P =0.14
OS hsa−mir−6868
P =0.29
PFI hsa−mir−6868
P =0.13
DFI hsa−mir−6868
P =0.21
DSS hsa−mir−6868
P =0.38
OS hsa−mir−6842
P =0.48
PFI hsa−mir−6842
P =0.3
survival probability
Time in days

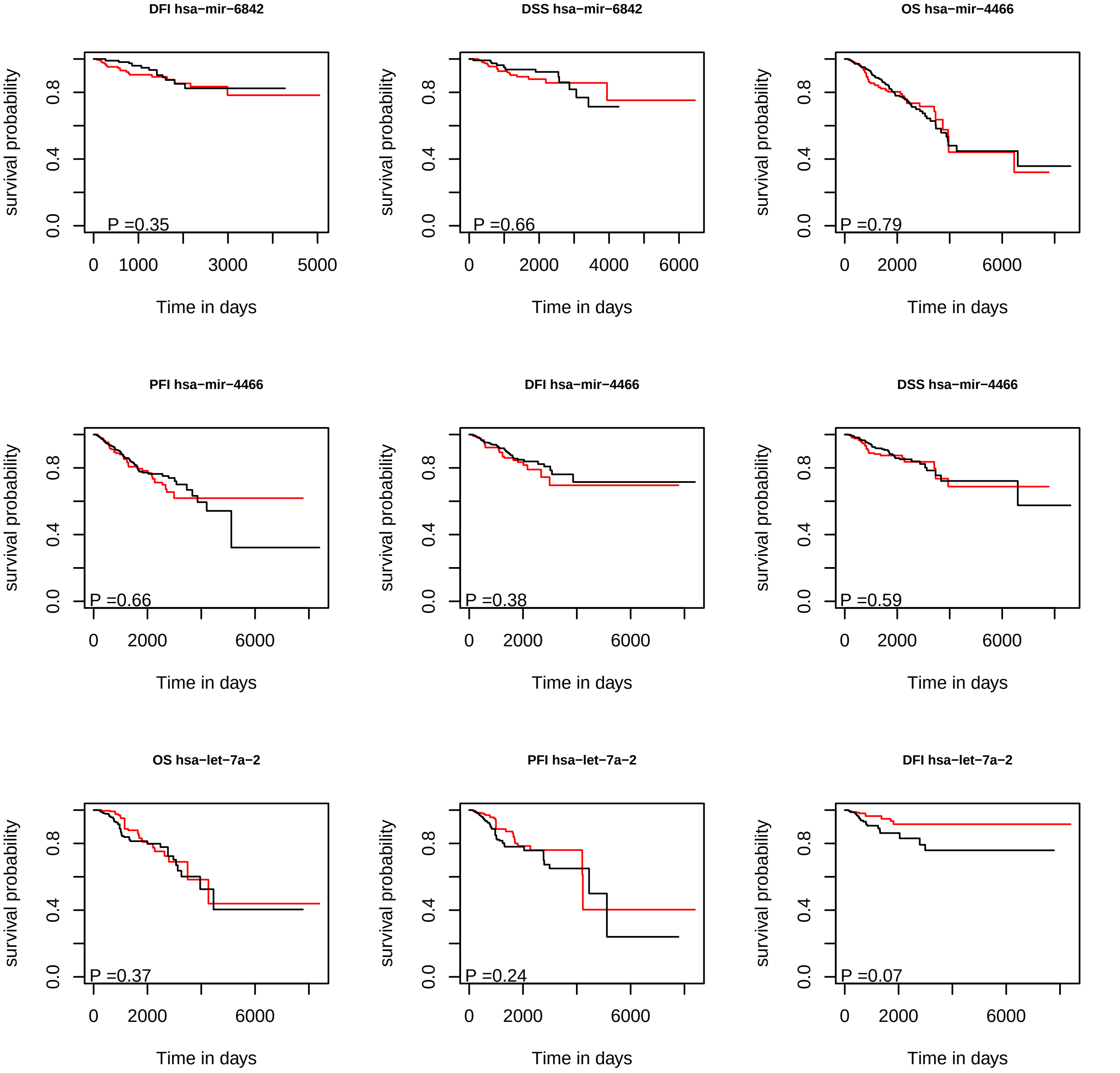
DFI hsa−mir−6842
survival probability
0.0 0.4 0.8
P =0.35
0 1000 3000 5000
Time in days
DSS hsa−mir−6842
survival probability
0.0 0.4 0.8
P =0.66
0 2000 4000 6000
Time in days
OS hsa−mir−4466
survival probability
0.0 0.4 0.8
P =0.79
0 2000 6000
Time in days
PFI hsa−mir−4466
survival probability
0.0 0.4 0.8
P =0.66
0 2000 6000
Time in days
DFI hsa−mir−4466
survival probability
0.0 0.4 0.8
P =0.38
0 2000 6000
Time in days
DSS hsa−mir−4466
survival probability
0.0 0.4 0.8
P =0.59
0 2000 6000
Time in days
OS hsa−let−7a−2
survival probability
0.0 0.4 0.8
P =0.37
0 2000 6000
Time in days
PFI hsa−let−7a−2
survival probability
0.0 0.4 0.8
P =0.24
0 2000 6000
Time in days
DFI hsa−let−7a−2
survival probability
0.0 0.4 0.8
P =0.07
0 2000 6000
Time in days

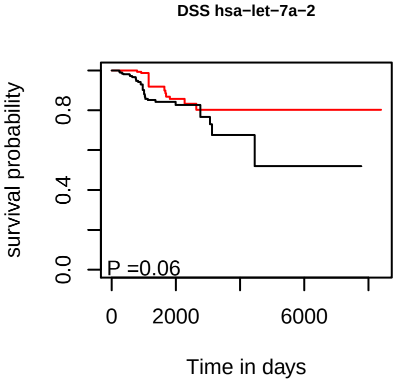
DSS hsa−let−7a−2
survival probability
0.0
0.4
0.8
P =0.06
0
2000
6000
Time in days
