# Supplementary material for: Computing microRNA-gene interaction networks in pan-cancer using miRDriver
Source: Sci Rep. 2022 Mar 8;12:3717. doi: 10.1038/s41598-022-07628-z (PMC8904490; doi:10.1038/s41598-022-07628-z)
Supplement: Supplementary file 13 — Supplementary Information 13. [file 41598_2022_7628_MOESM13_ESM.pdf]

Computing microRNA-gene interaction networks in pan-cancer using miRDriver

Banabithi Bose, Matthew Moravec, and Serdar Bozdag

Supplemental Figure S4

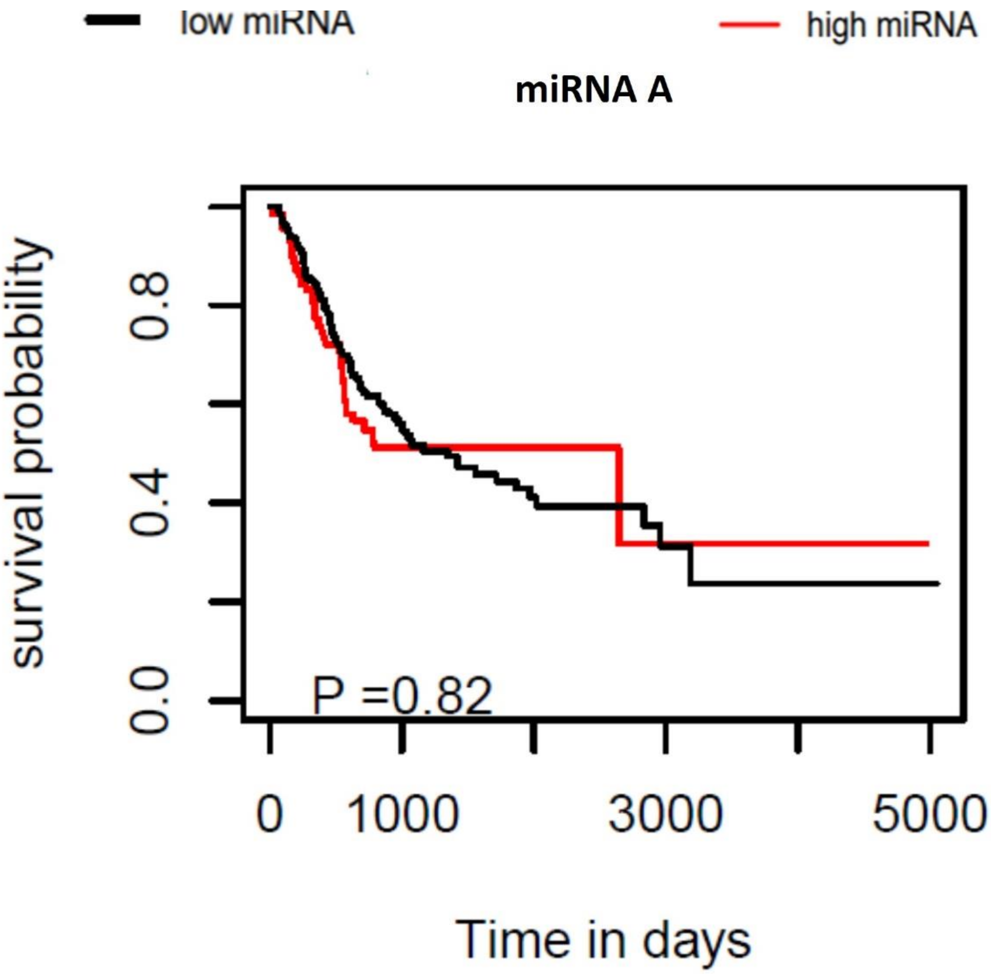

The *Adjusted Kaplan-Meier* survival plots for the computed miRNAs in high and low miRNA expression patient groups.

Supplemental Figure S4 Cancer Type: CESC

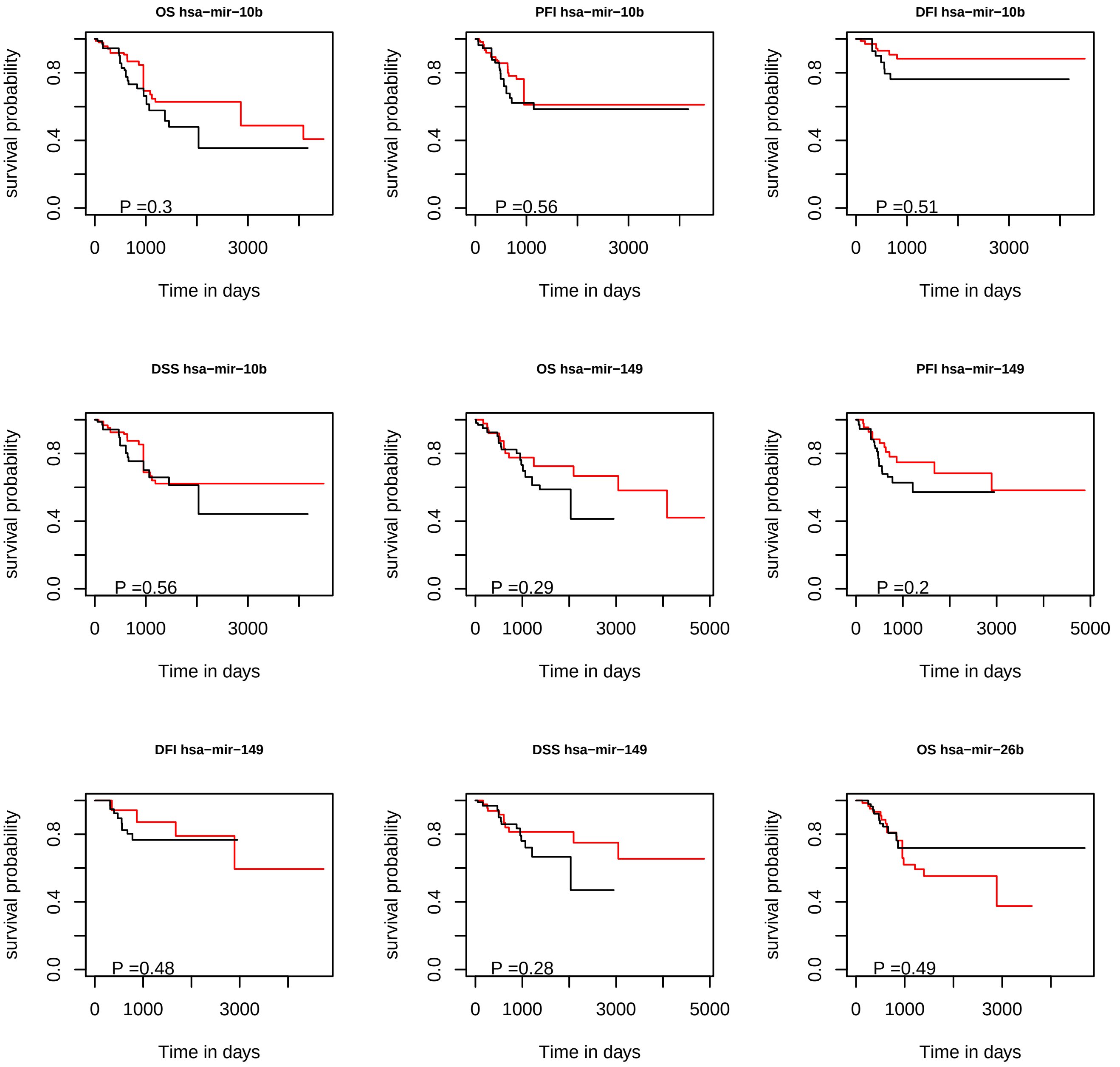

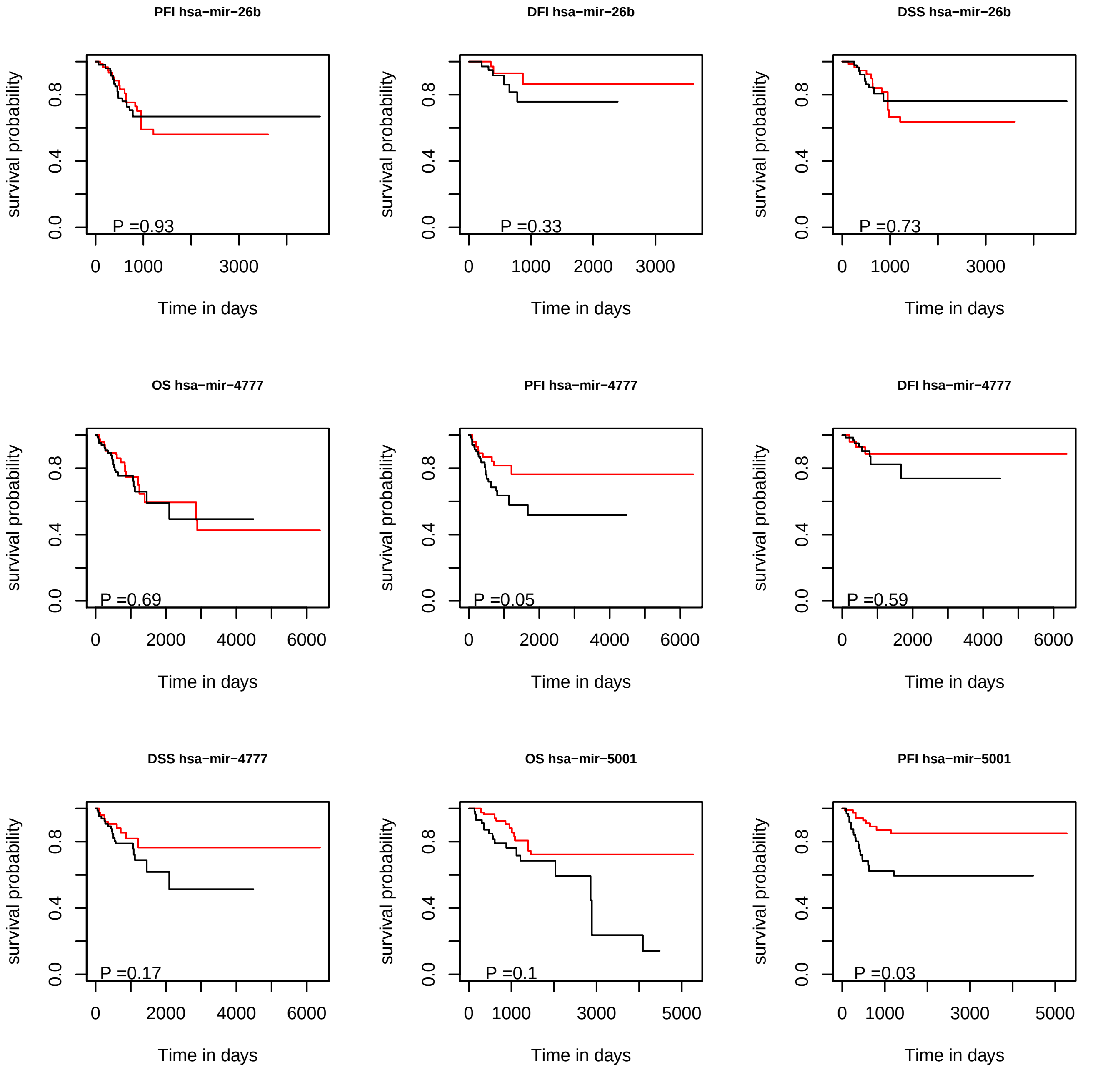
PFI hsa−mir−26b
P =0.93
DFI hsa−mir−26b
P =0.33
DSS hsa−mir−26b
P =0.73
OS hsa−mir−4777
P =0.69
PFI hsa−mir−4777
P =0.05
DFI hsa−mir−4777
P =0.59
DSS hsa−mir−4777
P =0.17
OS hsa−mir−5001
P =0.1
PFI hsa−mir−5001
P =0.03
survival probability
Time in days

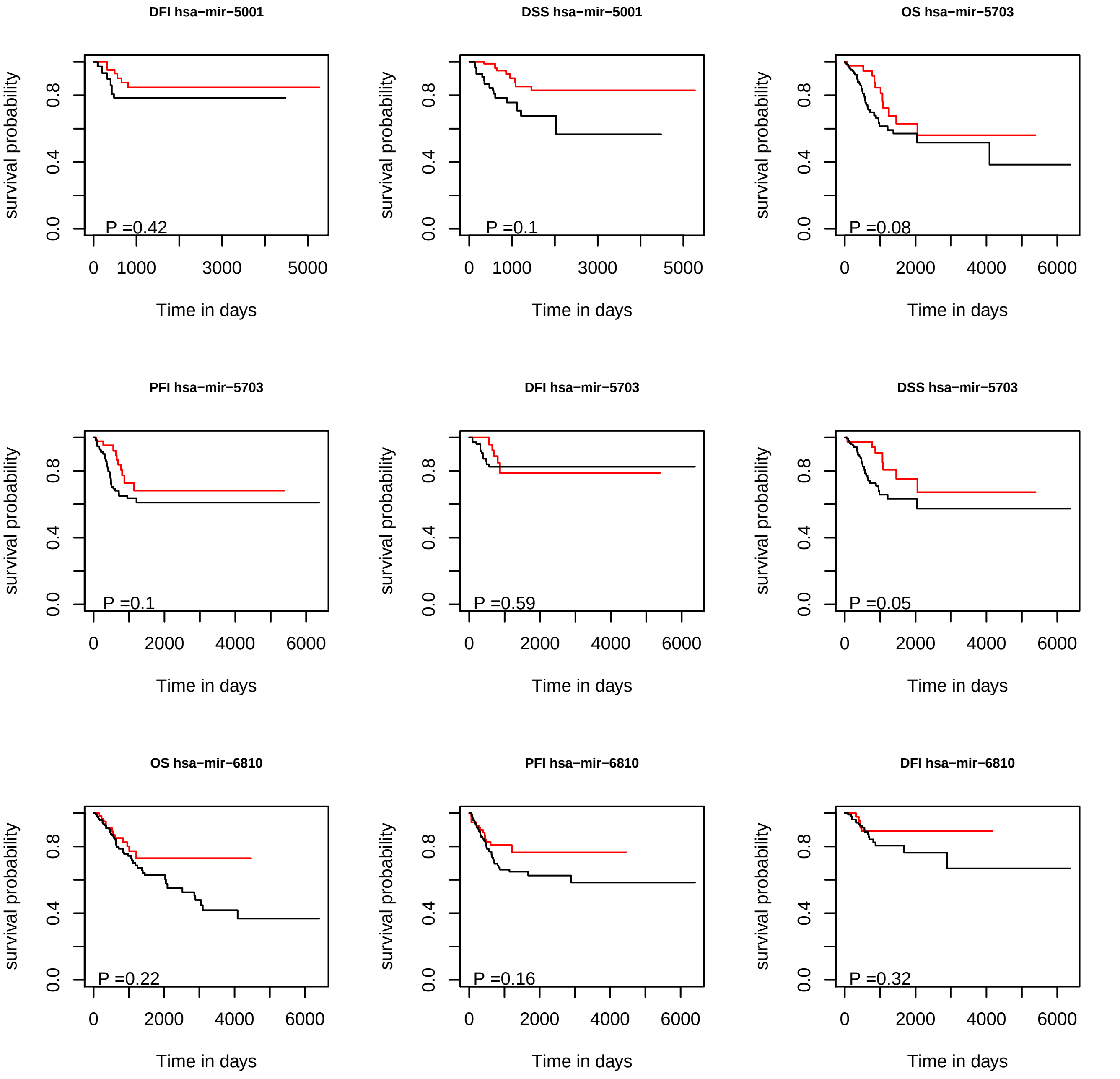

DFI hsa−mir−5001
survival probability
0.0
0.4
0.8
P =0.42
0
1000
3000
5000
Time in days
DSS hsa−mir−5001
survival probability
0.0
0.4
0.8
P =0.1
0
1000
3000
5000
Time in days
OS hsa−mir−5703
survival probability
0.0
0.4
0.8
P =0.08
0
2000
4000
6000
Time in days
PFI hsa−mir−5703
survival probability
0.0
0.4
0.8
P =0.1
0
2000
4000
6000
Time in days
DFI hsa−mir−5703
survival probability
0.0
0.4
0.8
P =0.59
0
2000
4000
6000
Time in days
DSS hsa−mir−5703
survival probability
0.0
0.4
0.8
P =0.05
0
2000
4000
6000
Time in days
OS hsa−mir−6810
survival probability
0.0
0.4
0.8
P =0.22
0
2000
4000
6000
Time in days
PFI hsa−mir−6810
survival probability
0.0
0.4
0.8
P =0.16
0
2000
4000
6000
Time in days
DFI hsa−mir−6810
survival probability
0.0
0.4
0.8
P =0.32
0
2000
4000
6000
Time in days

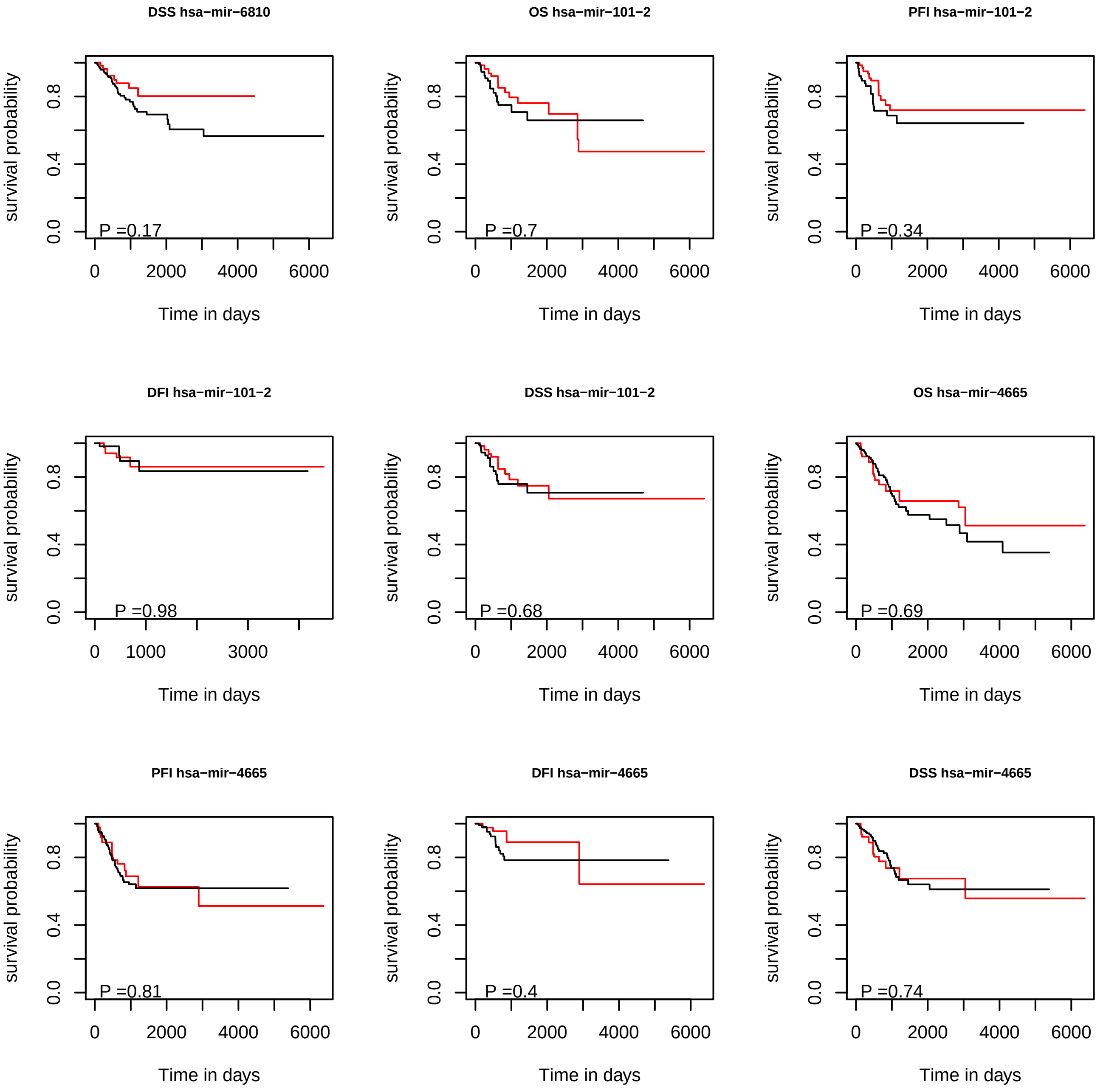
DSS hsa−mir−6810
P =0.17
OS hsa−mir−101−2
P =0.7
PFI hsa−mir−101−2
P =0.34
DFI hsa−mir−101−2
P =0.98
DSS hsa−mir−101−2
P =0.68
OS hsa−mir−4665
P =0.69
PFI hsa−mir−4665
P =0.81
DFI hsa−mir−4665
P =0.4
DSS hsa−mir−4665
P =0.74
survival probability
Time in days
0.0
0.4
0.8
0
1000
3000
2000
4000
6000

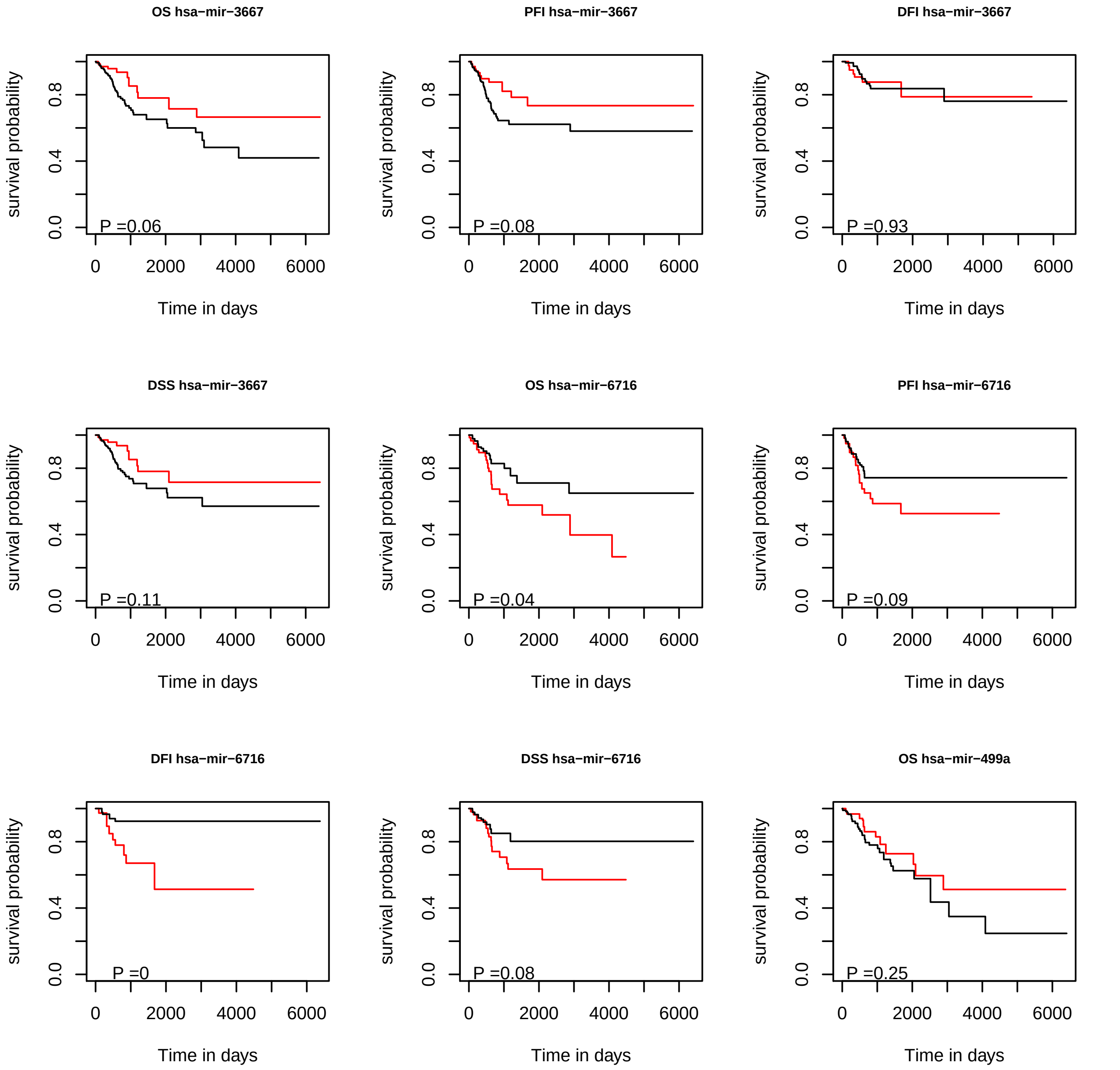
OS hsa−mir−3667
survival probability
0.0
0.4
0.8
P =0.06
0
2000
4000
6000
Time in days
PFI hsa−mir−3667
survival probability
0.0
0.4
0.8
P =0.08
0
2000
4000
6000
Time in days
DFI hsa−mir−3667
survival probability
0.0
0.4
0.8
P =0.93
0
2000
4000
6000
Time in days
DSS hsa−mir−3667
survival probability
0.0
0.4
0.8
P =0.11
0
2000
4000
6000
Time in days
OS hsa−mir−6716
survival probability
0.0
0.4
0.8
P =0.04
0
2000
4000
6000
Time in days
PFI hsa−mir−6716
survival probability
0.0
0.4
0.8
P =0.09
0
2000
4000
6000
Time in days
DFI hsa−mir−6716
survival probability
0.0
0.4
0.8
P =0
0
2000
4000
6000
Time in days
DSS hsa−mir−6716
survival probability
0.0
0.4
0.8
P =0.08
0
2000
4000
6000
Time in days
OS hsa−mir−499a
survival probability
0.0
0.4
0.8
P =0.25
0
2000
4000
6000
Time in days

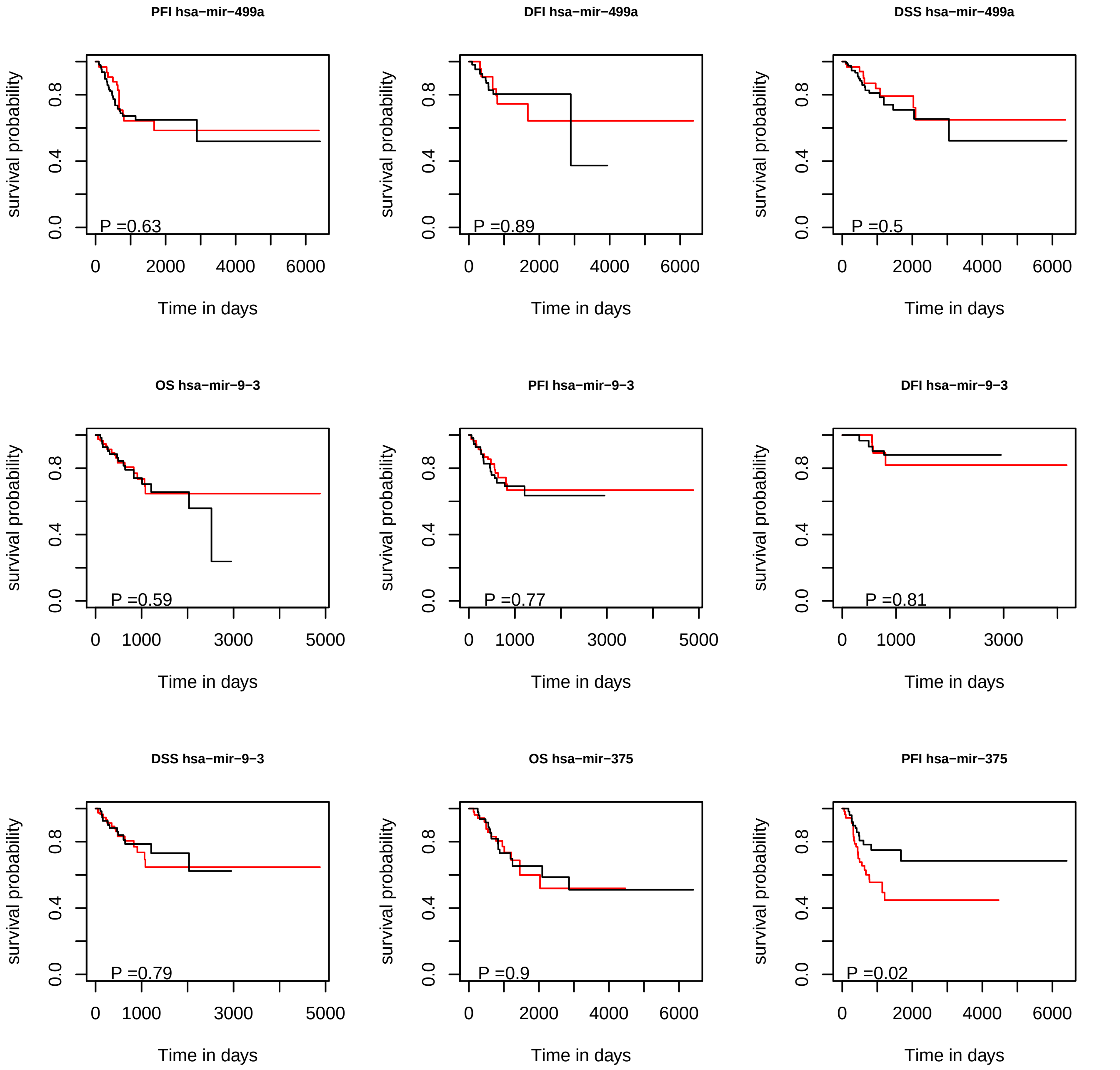
PFI hsa−mir−499a
P =0.63
DFI hsa−mir−499a
P =0.89
DSS hsa−mir−499a
P =0.5
OS hsa−mir−9−3
P =0.59
PFI hsa−mir−9−3
P =0.77
DFI hsa−mir−9−3
P =0.81
DSS hsa−mir−9−3
P =0.79
OS hsa−mir−375
P =0.9
PFI hsa−mir−375
P =0.02
survival probability
Time in days
0.0
0.4
0.8
0
1000
2000
3000
4000
5000
6000

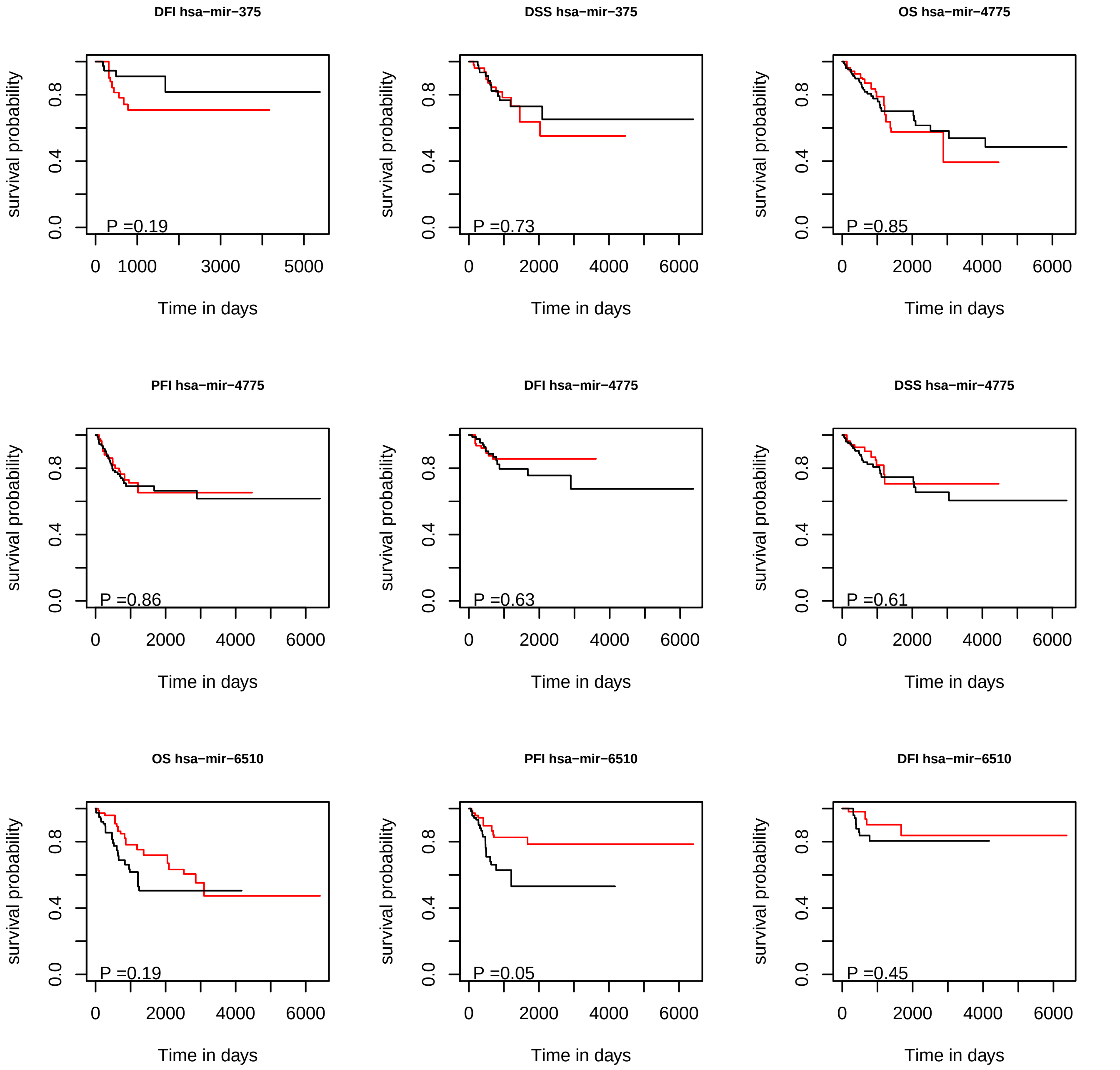
DFI hsa−mir−375
P =0.19
DSS hsa−mir−375
P =0.73
OS hsa−mir−4775
P =0.85
PFI hsa−mir−4775
P =0.86
DFI hsa−mir−4775
P =0.63
DSS hsa−mir−4775
P =0.61
OS hsa−mir−6510
P =0.19
PFI hsa−mir−6510
P =0.05
DFI hsa−mir−6510
P =0.45
survival probability
Time in days
0.0
0.4
0.8
0
1000
2000
3000
4000
5000
6000

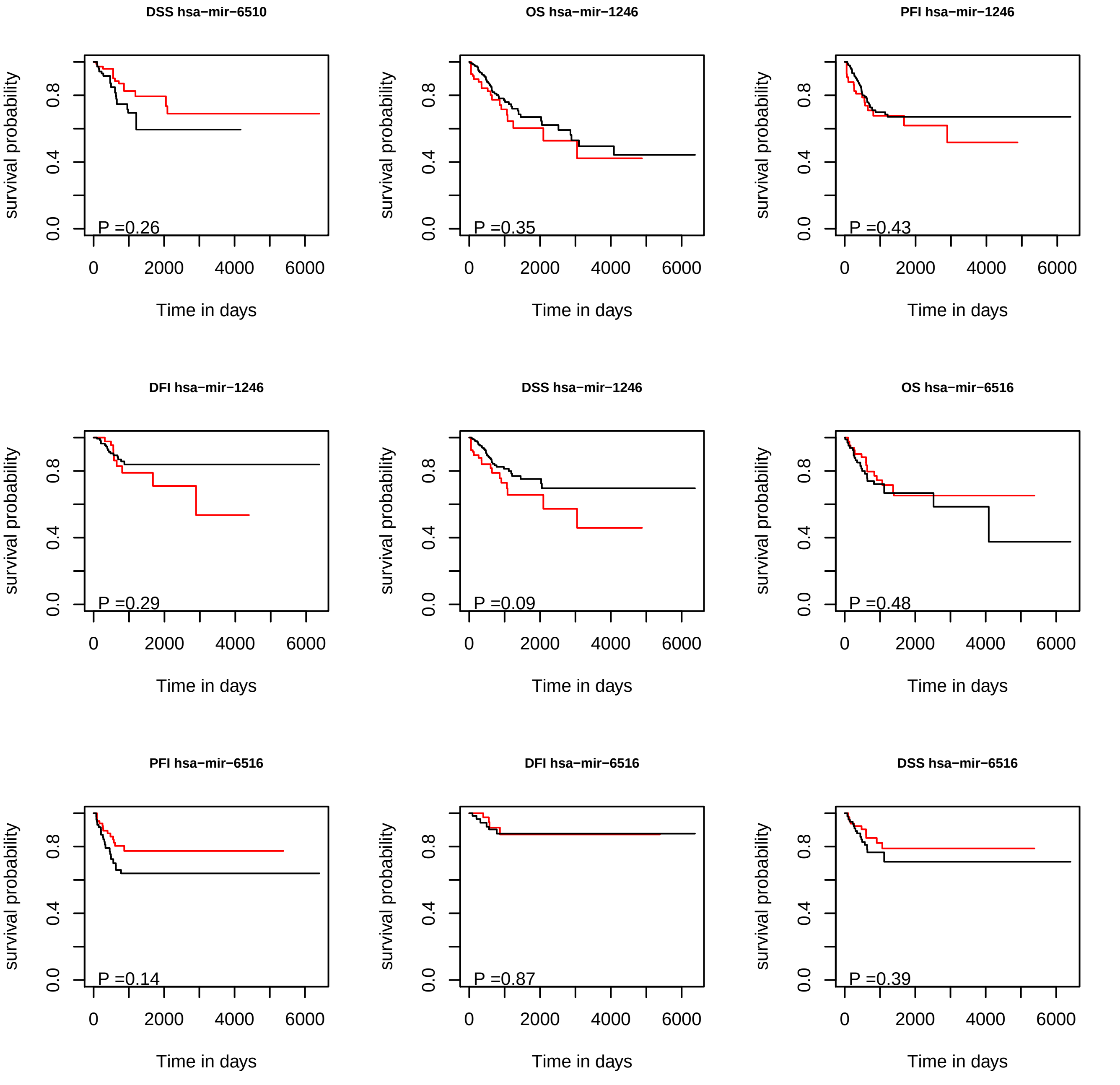
DSS hsa−mir−6510
P =0.26
OS hsa−mir−1246
P =0.35
PFI hsa−mir−1246
P =0.43
DFI hsa−mir−1246
P =0.29
DSS hsa−mir−1246
P =0.09
OS hsa−mir−6516
P =0.48
PFI hsa−mir−6516
P =0.14
DFI hsa−mir−6516
P =0.87
DSS hsa−mir−6516
P =0.39
survival probability
0.0
0.4
0.8
0
2000
4000
6000
Time in days

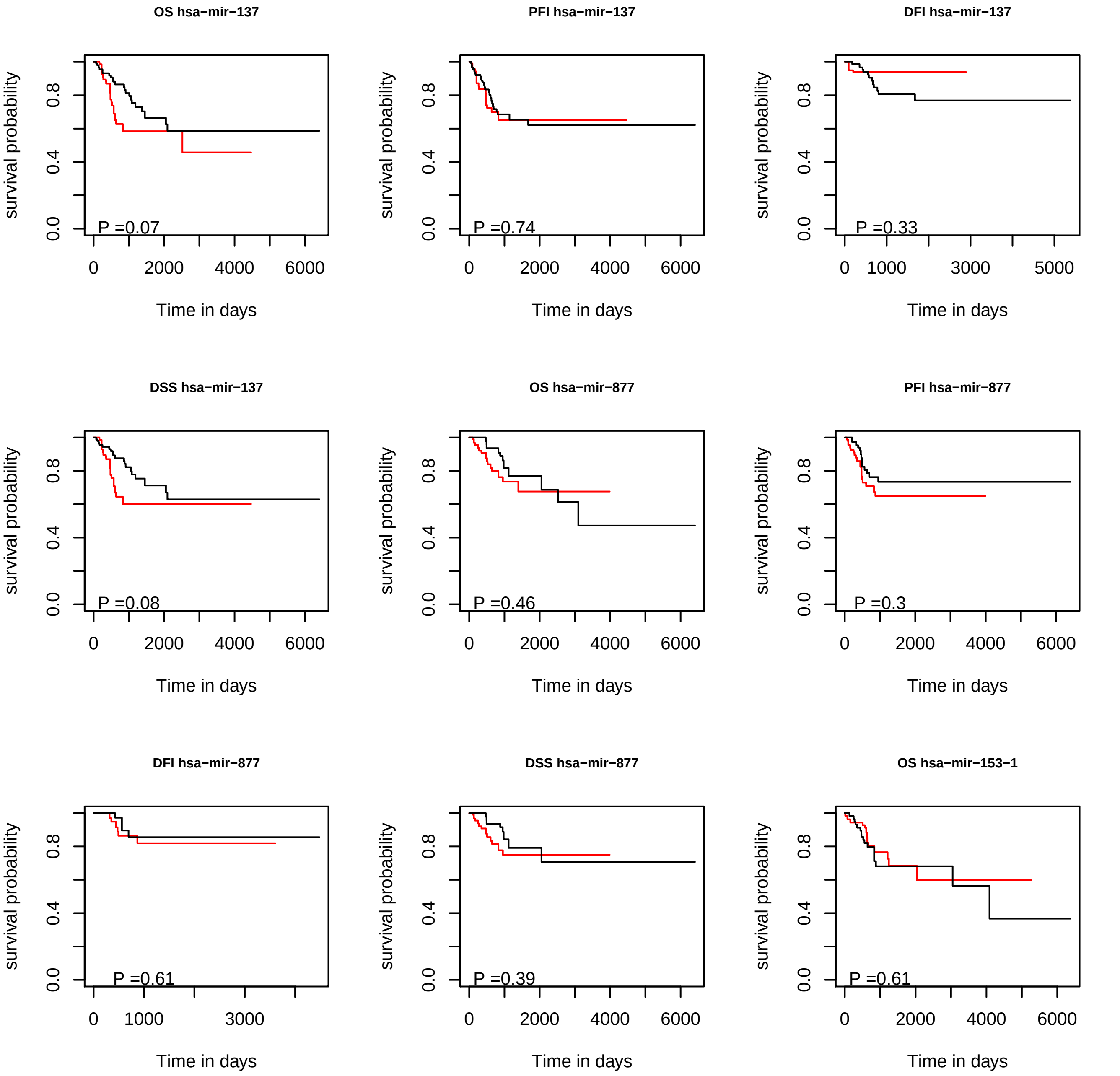
OS hsa−mir−137
survival probability
0.0
0.4
0.8
P =0.07
0
2000
4000
6000
Time in days
PFI hsa−mir−137
survival probability
0.0
0.4
0.8
P =0.74
0
2000
4000
6000
Time in days
DFI hsa−mir−137
survival probability
0.0
0.4
0.8
P =0.33
0
1000
3000
5000
Time in days
DSS hsa−mir−137
survival probability
0.0
0.4
0.8
P =0.08
0
2000
4000
6000
Time in days
OS hsa−mir−877
survival probability
0.0
0.4
0.8
P =0.46
0
2000
4000
6000
Time in days
PFI hsa−mir−877
survival probability
0.0
0.4
0.8
P =0.3
0
2000
4000
6000
Time in days
DFI hsa−mir−877
survival probability
0.0
0.4
0.8
P =0.61
0
1000
3000
Time in days
DSS hsa−mir−877
survival probability
0.0
0.4
0.8
P =0.39
0
2000
4000
6000
Time in days
OS hsa−mir−153−1
survival probability
0.0
0.4
0.8
P =0.61
0
2000
4000
6000
Time in days

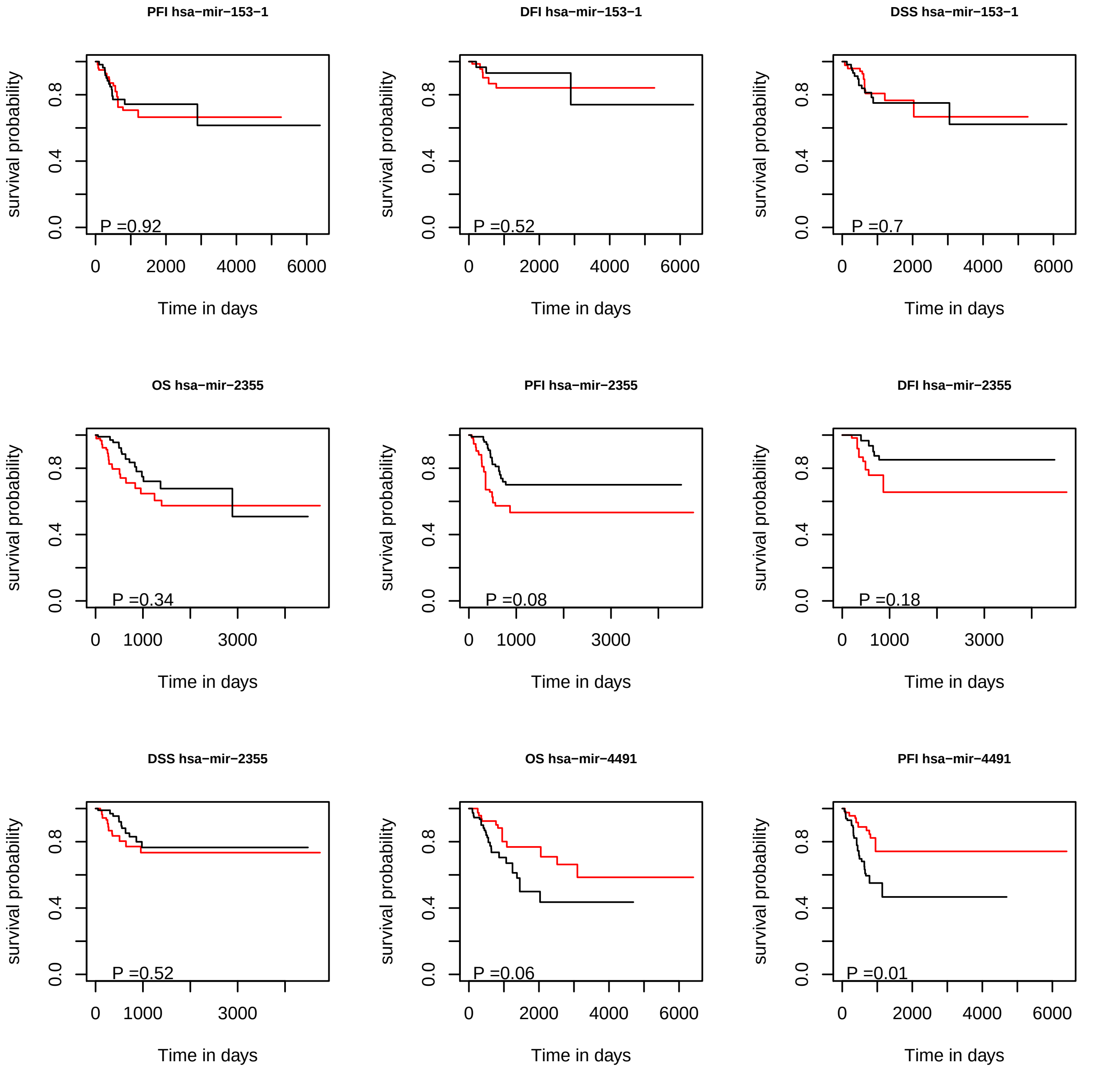

PFI hsa−mir−153−1
survival probability
0.0
0.4
0.8
P =0.92
0
2000
4000
6000
Time in days
DFI hsa−mir−153−1
survival probability
0.0
0.4
0.8
P =0.52
0
2000
4000
6000
Time in days
DSS hsa−mir−153−1
survival probability
0.0
0.4
0.8
P =0.7
0
2000
4000
6000
Time in days
OS hsa−mir−2355
survival probability
0.0
0.4
0.8
P =0.34
0
1000
3000
Time in days
PFI hsa−mir−2355
survival probability
0.0
0.4
0.8
P =0.08
0
1000
3000
Time in days
DFI hsa−mir−2355
survival probability
0.0
0.4
0.8
P =0.18
0
1000
3000
Time in days
DSS hsa−mir−2355
survival probability
0.0
0.4
0.8
P =0.52
0
1000
3000
Time in days
OS hsa−mir−4491
survival probability
0.0
0.4
0.8
P =0.06
0
2000
4000
6000
Time in days
PFI hsa−mir−4491
survival probability
0.0
0.4
0.8
P =0.01
0
2000
4000
6000
Time in days

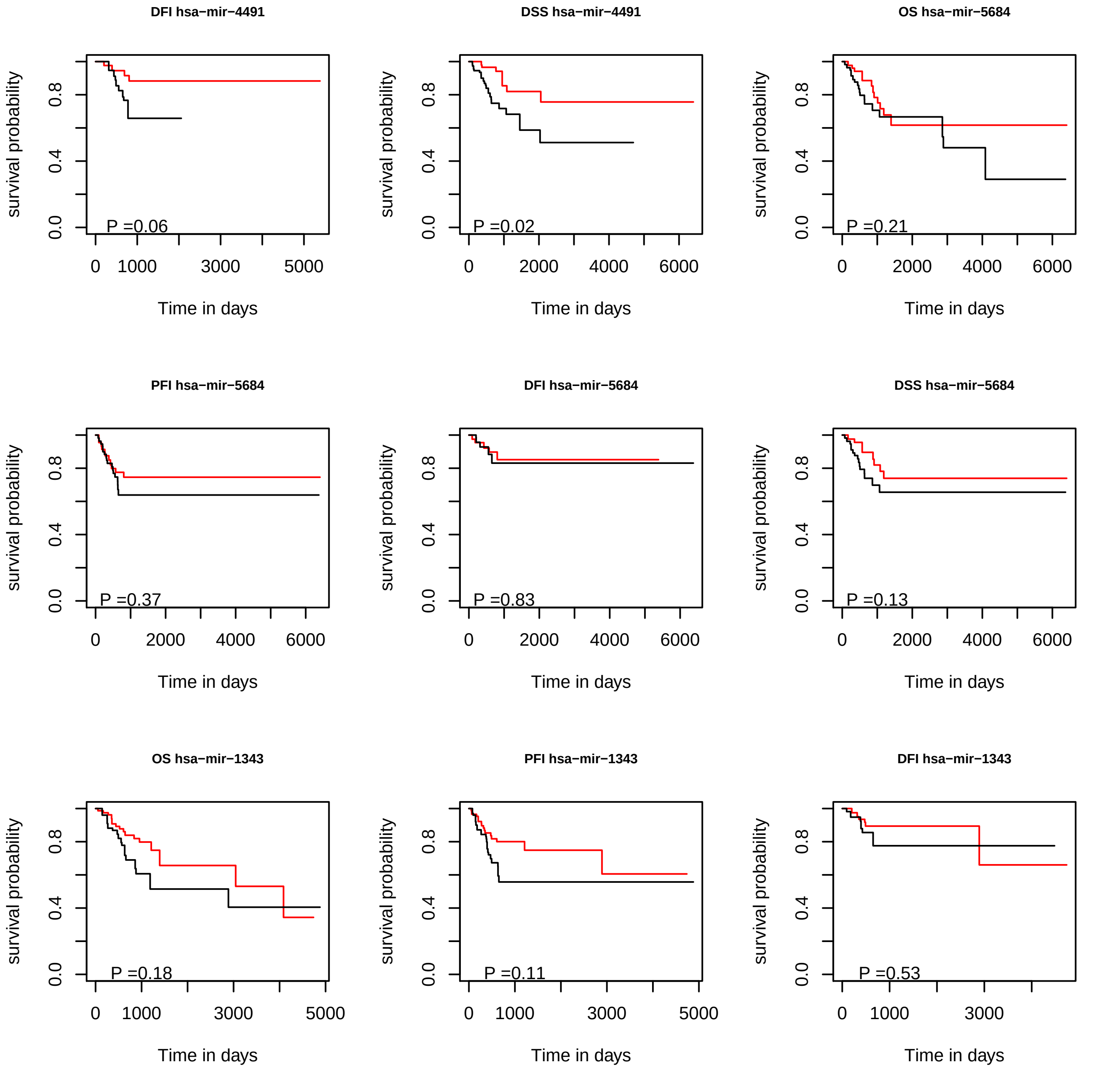
DFI hsa−mir−4491
P =0.06
DSS hsa−mir−4491
P =0.02
OS hsa−mir−5684
P =0.21
PFI hsa−mir−5684
P =0.37
DFI hsa−mir−5684
P =0.83
DSS hsa−mir−5684
P =0.13
OS hsa−mir−1343
P =0.18
PFI hsa−mir−1343
P =0.11
DFI hsa−mir−1343
P =0.53
survival probability
Time in days

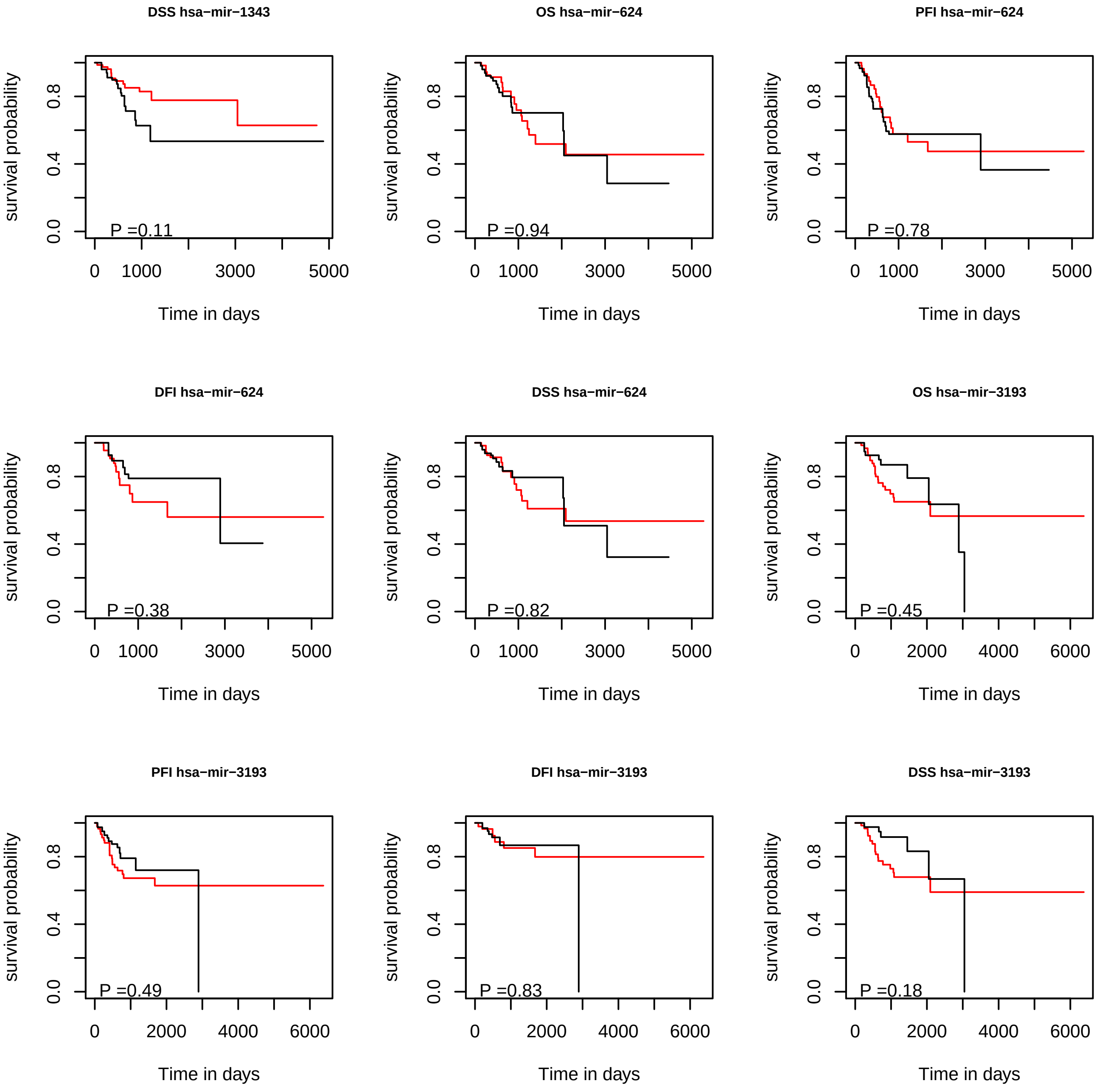

DSS hsa−mir−1343
survival probability
Time in days
P =0.11
OS hsa−mir−624
survival probability
Time in days
P =0.94
PFI hsa−mir−624
survival probability
Time in days
P =0.78
DFI hsa−mir−624
survival probability
Time in days
P =0.38
DSS hsa−mir−624
survival probability
Time in days
P =0.82
OS hsa−mir−3193
survival probability
Time in days
P =0.45
PFI hsa−mir−3193
survival probability
Time in days
P =0.49
DFI hsa−mir−3193
survival probability
Time in days
P =0.83
DSS hsa−mir−3193
survival probability
Time in days
P =0.18

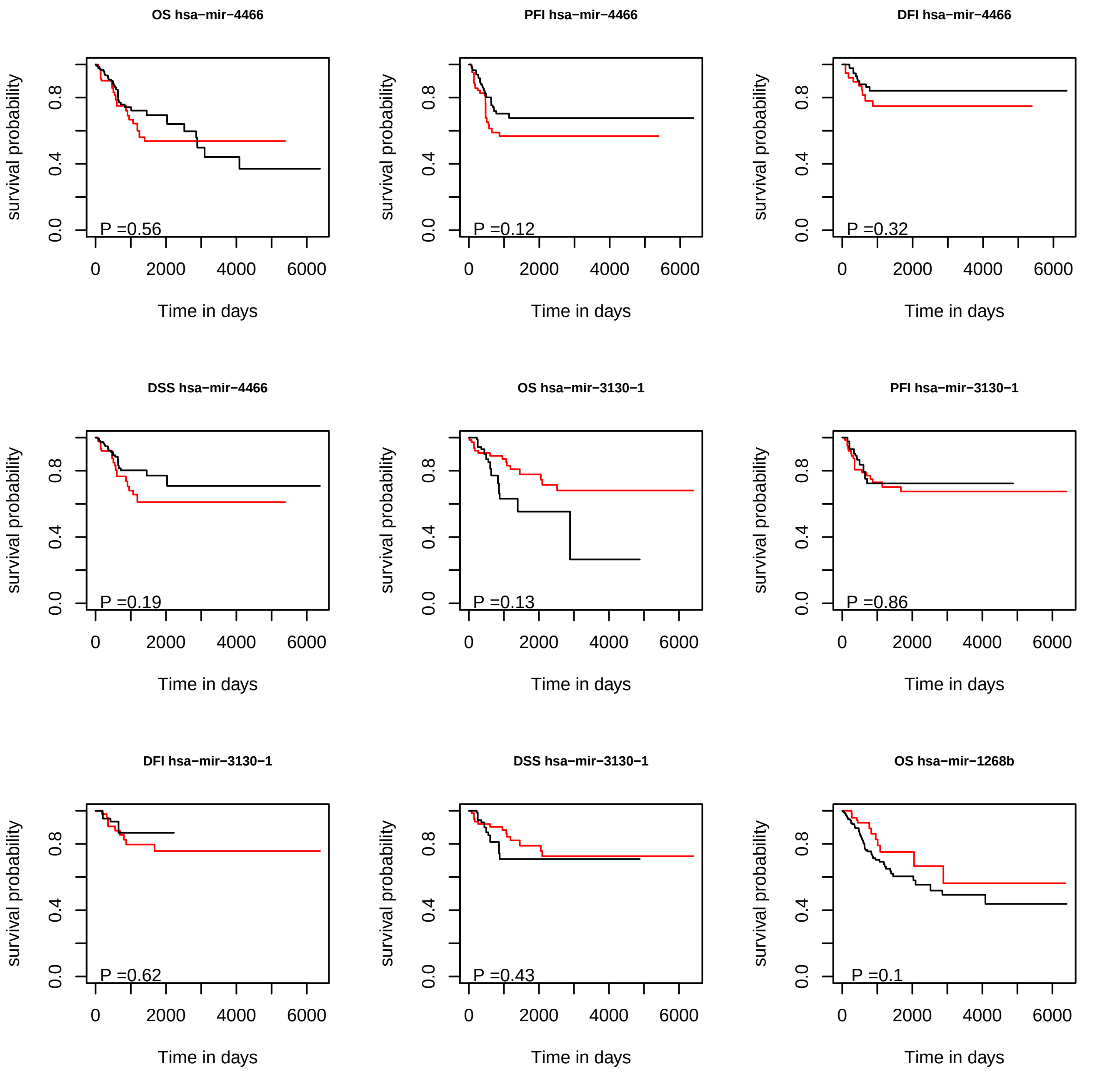
OS hsa−mir−4466
P =0.56
PFI hsa−mir−4466
P =0.12
DFI hsa−mir−4466
P =0.32
DSS hsa−mir−4466
P =0.19
OS hsa−mir−3130−1
P =0.13
PFI hsa−mir−3130−1
P =0.86
DFI hsa−mir−3130−1
P =0.62
DSS hsa−mir−3130−1
P =0.43
OS hsa−mir−1268b
P =0.1
survival probability
0.0
0.4
0.8
Time in days
0
2000
4000
6000

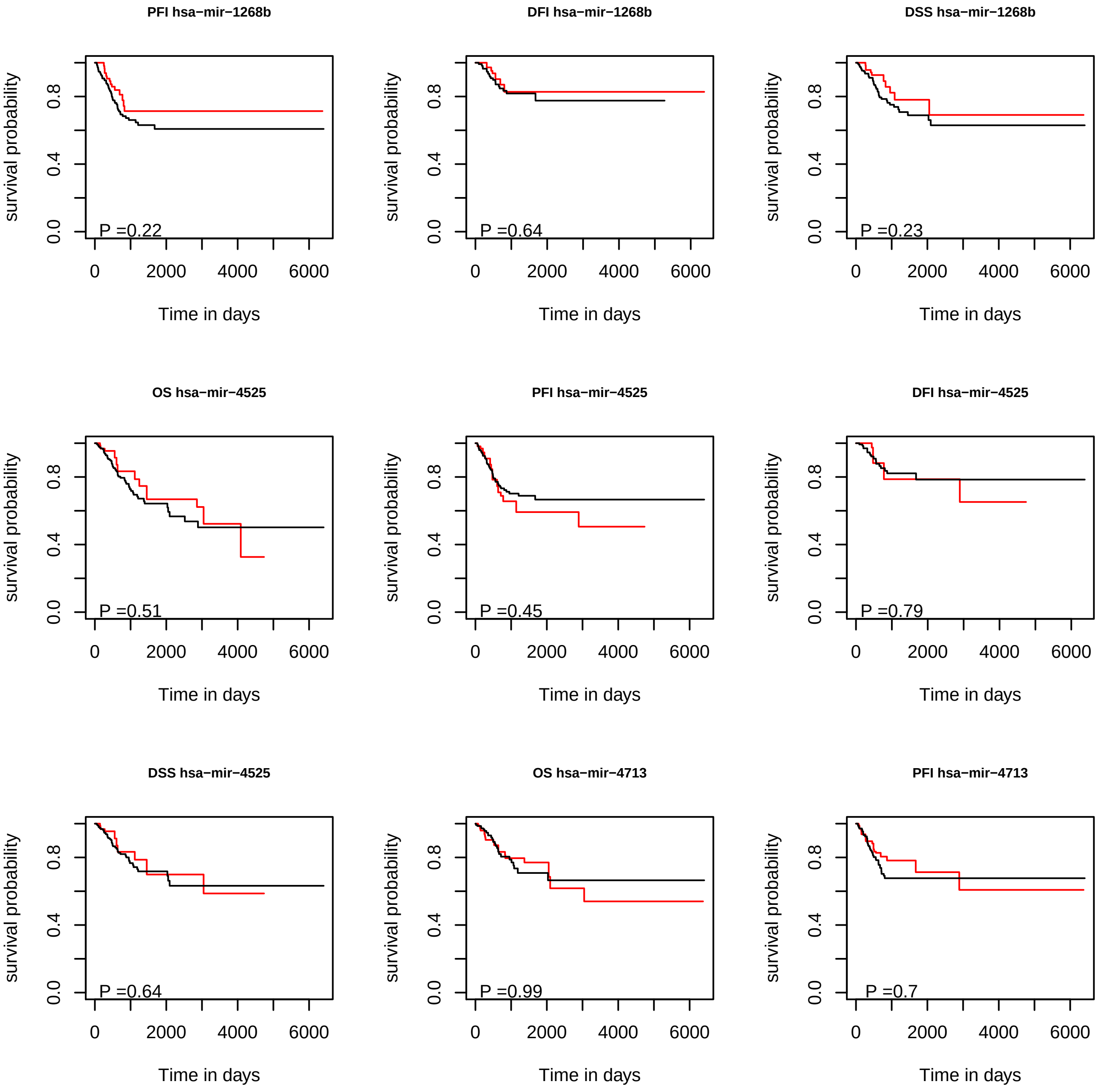
PFI hsa−mir−1268b
survival probability
0.0
0.4
0.8
P =0.22
0
2000
4000
6000
Time in days
DFI hsa−mir−1268b
survival probability
0.0
0.4
0.8
P =0.64
0
2000
4000
6000
Time in days
DSS hsa−mir−1268b
survival probability
0.0
0.4
0.8
P =0.23
0
2000
4000
6000
Time in days
OS hsa−mir−4525
survival probability
0.0
0.4
0.8
P =0.51
0
2000
4000
6000
Time in days
PFI hsa−mir−4525
survival probability
0.0
0.4
0.8
P =0.45
0
2000
4000
6000
Time in days
DFI hsa−mir−4525
survival probability
0.0
0.4
0.8
P =0.79
0
2000
4000
6000
Time in days
DSS hsa−mir−4525
survival probability
0.0
0.4
0.8
P =0.64
0
2000
4000
6000
Time in days
OS hsa−mir−4713
survival probability
0.0
0.4
0.8
P =0.99
0
2000
4000
6000
Time in days
PFI hsa−mir−4713
survival probability
0.0
0.4
0.8
P =0.7
0
2000
4000
6000
Time in days

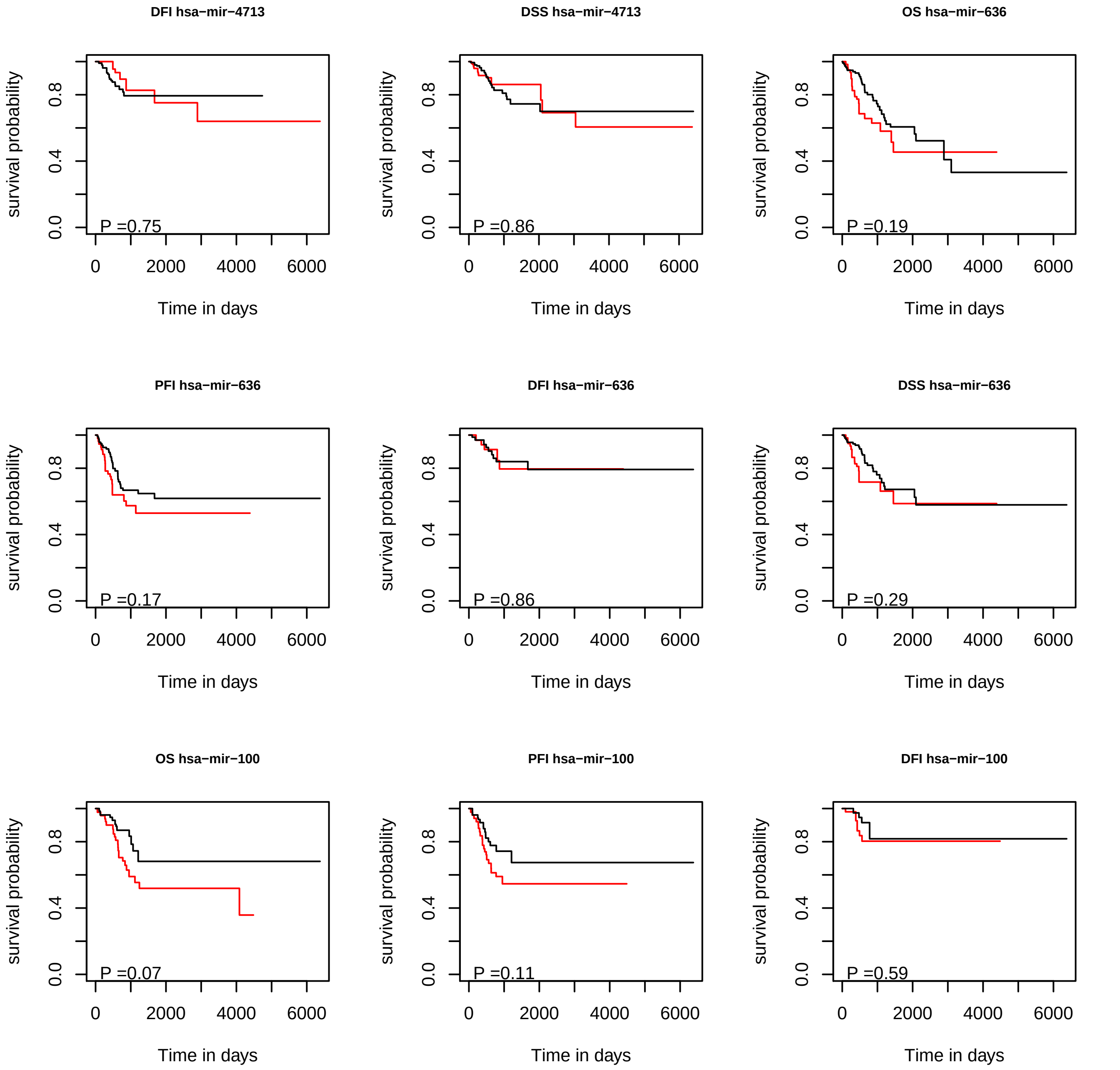
DFI hsa−mir−4713
P =0.75
DSS hsa−mir−4713
P =0.86
OS hsa−mir−636
P =0.19
PFI hsa−mir−636
P =0.17
DFI hsa−mir−636
P =0.86
DSS hsa−mir−636
P =0.29
OS hsa−mir−100
P =0.07
PFI hsa−mir−100
P =0.11
DFI hsa−mir−100
P =0.59
survival probability
0.0
0.4
0.8
Time in days
0
2000
4000
6000

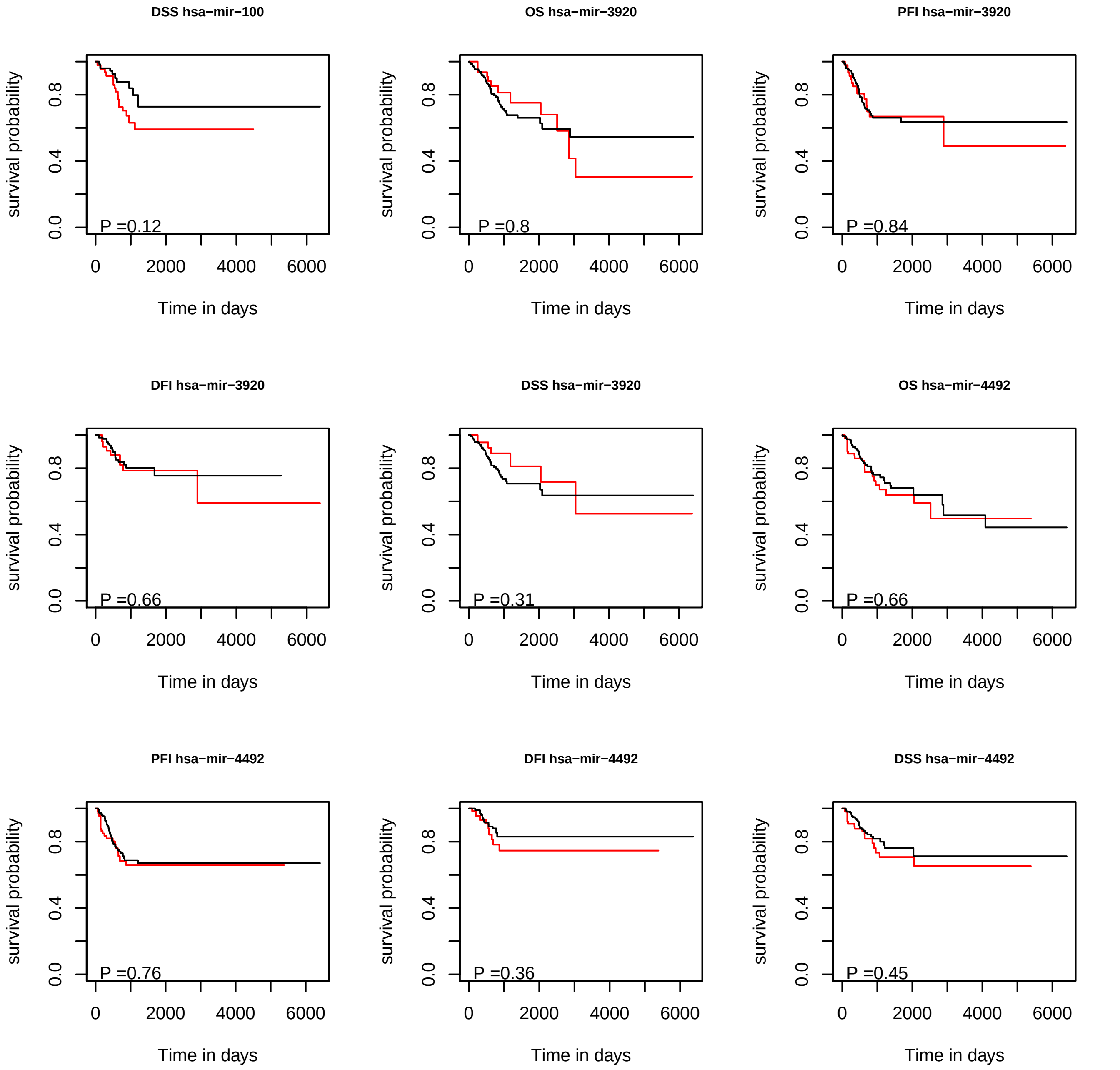
DSS hsa−mir−100
P =0.12
OS hsa−mir−3920
P =0.8
PFI hsa−mir−3920
P =0.84
DFI hsa−mir−3920
P =0.66
DSS hsa−mir−3920
P =0.31
OS hsa−mir−4492
P =0.66
PFI hsa−mir−4492
P =0.76
DFI hsa−mir−4492
P =0.36
DSS hsa−mir−4492
P =0.45
survival probability
0.0
0.4
0.8
Time in days
0
2000
4000
6000

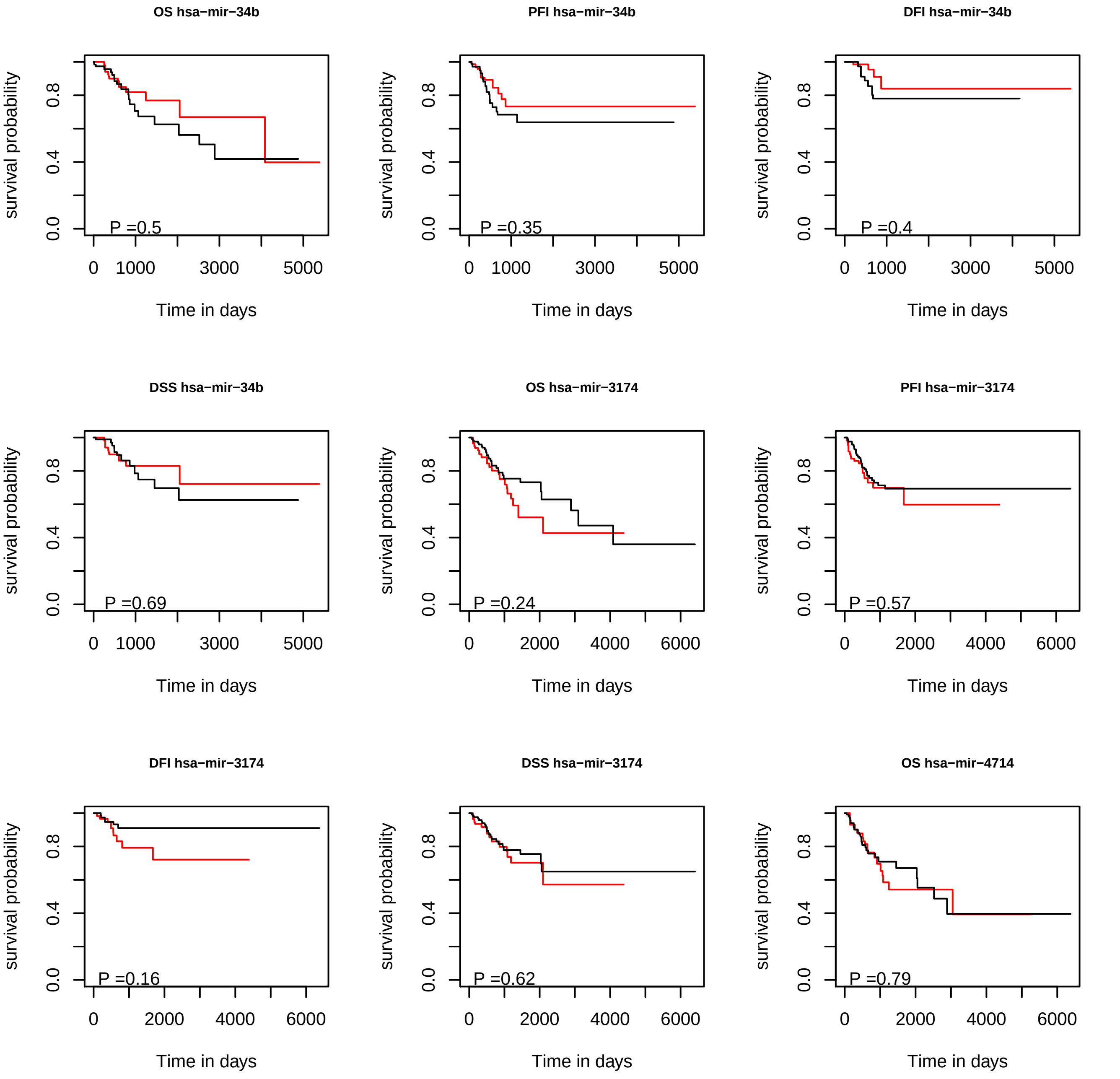
OS hsa−mir−34b
P =0.5
PFI hsa−mir−34b
P =0.35
DFI hsa−mir−34b
P =0.4
DSS hsa−mir−34b
P =0.69
OS hsa−mir−3174
P =0.24
PFI hsa−mir−3174
P =0.57
DFI hsa−mir−3174
P =0.16
DSS hsa−mir−3174
P =0.62
OS hsa−mir−4714
P =0.79
survival probability
Time in days
0.0
0.4
0.8
0
1000
2000
3000
4000
5000
6000

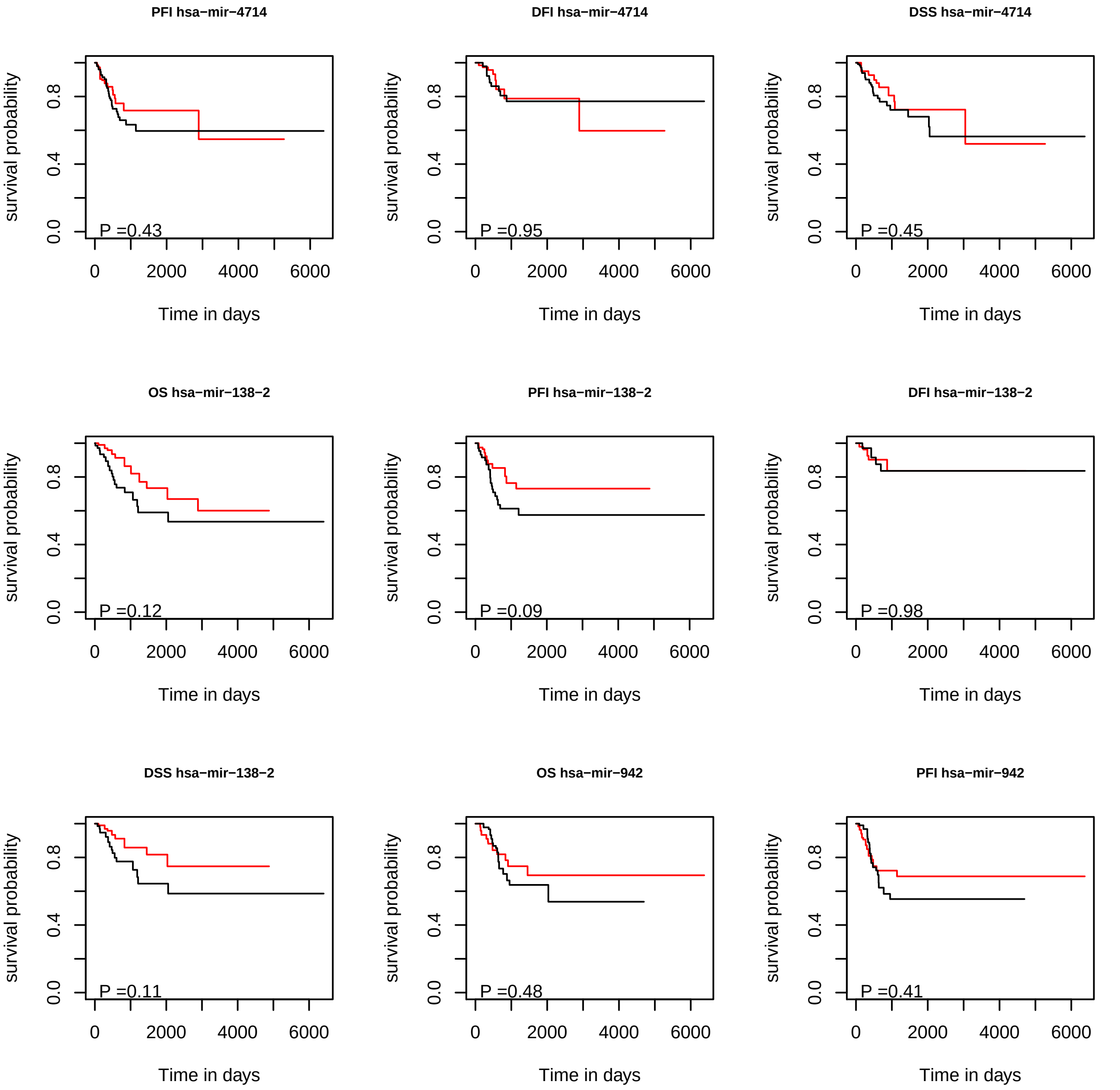
PFI hsa−mir−4714
DFI hsa−mir−4714
DSS hsa−mir−4714
OS hsa−mir−138−2
PFI hsa−mir−138−2
DFI hsa−mir−138−2
DSS hsa−mir−138−2
OS hsa−mir−942
PFI hsa−mir−942
survival probability
Time in days
0.0
0.4
0.8
0
2000
4000
6000
P =0.43
P =0.95
P =0.45
P =0.12
P =0.09
P =0.98
P =0.11
P =0.48
P =0.41

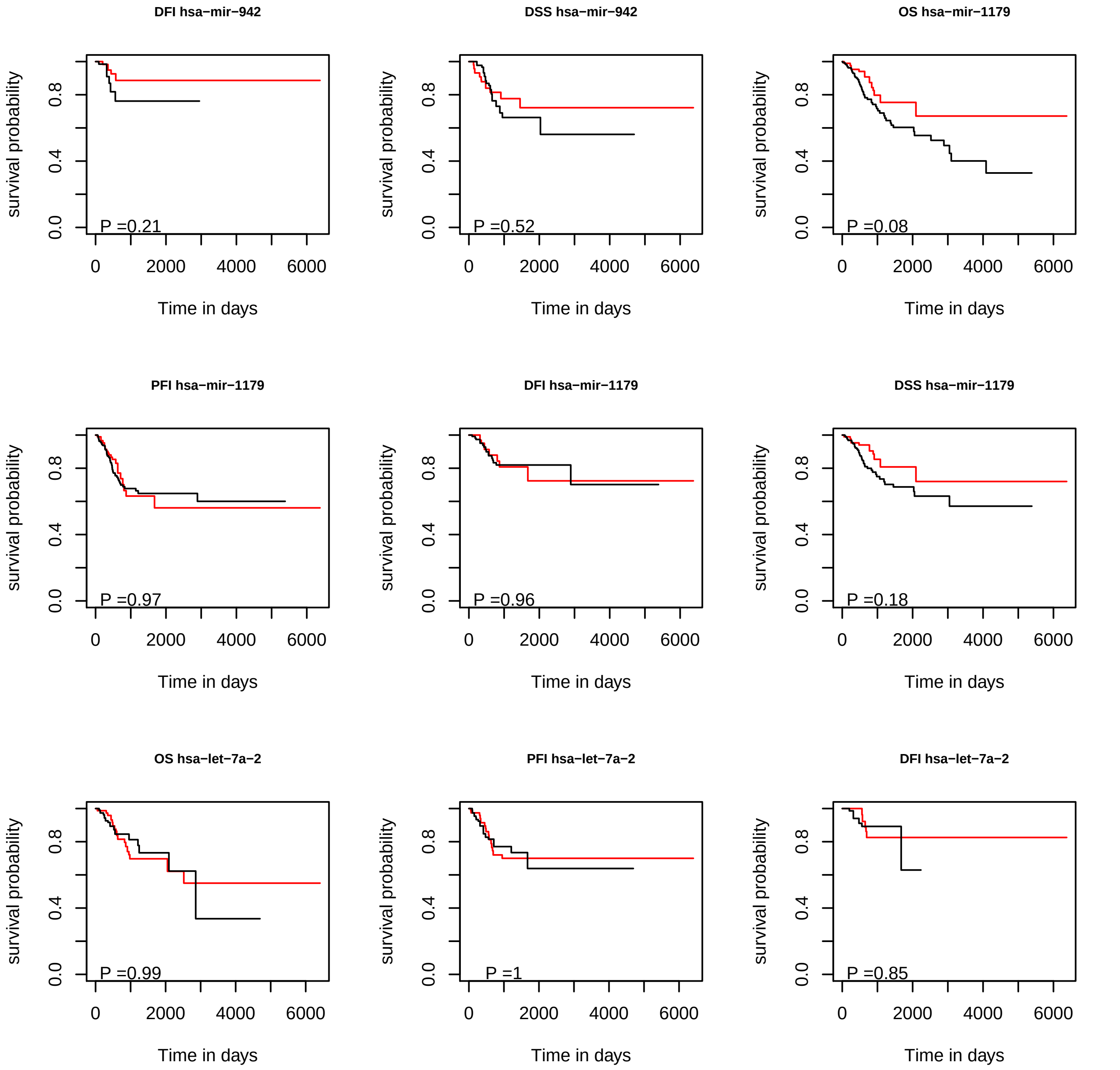
DFI hsa−mir−942
P =0.21
DSS hsa−mir−942
P =0.52
OS hsa−mir−1179
P =0.08
PFI hsa−mir−1179
P =0.97
DFI hsa−mir−1179
P =0.96
DSS hsa−mir−1179
P =0.18
OS hsa−let−7a−2
P =0.99
PFI hsa−let−7a−2
P =1
DFI hsa−let−7a−2
P =0.85
survival probability
Time in days
0.0
0.4
0.8
0
2000
4000
6000

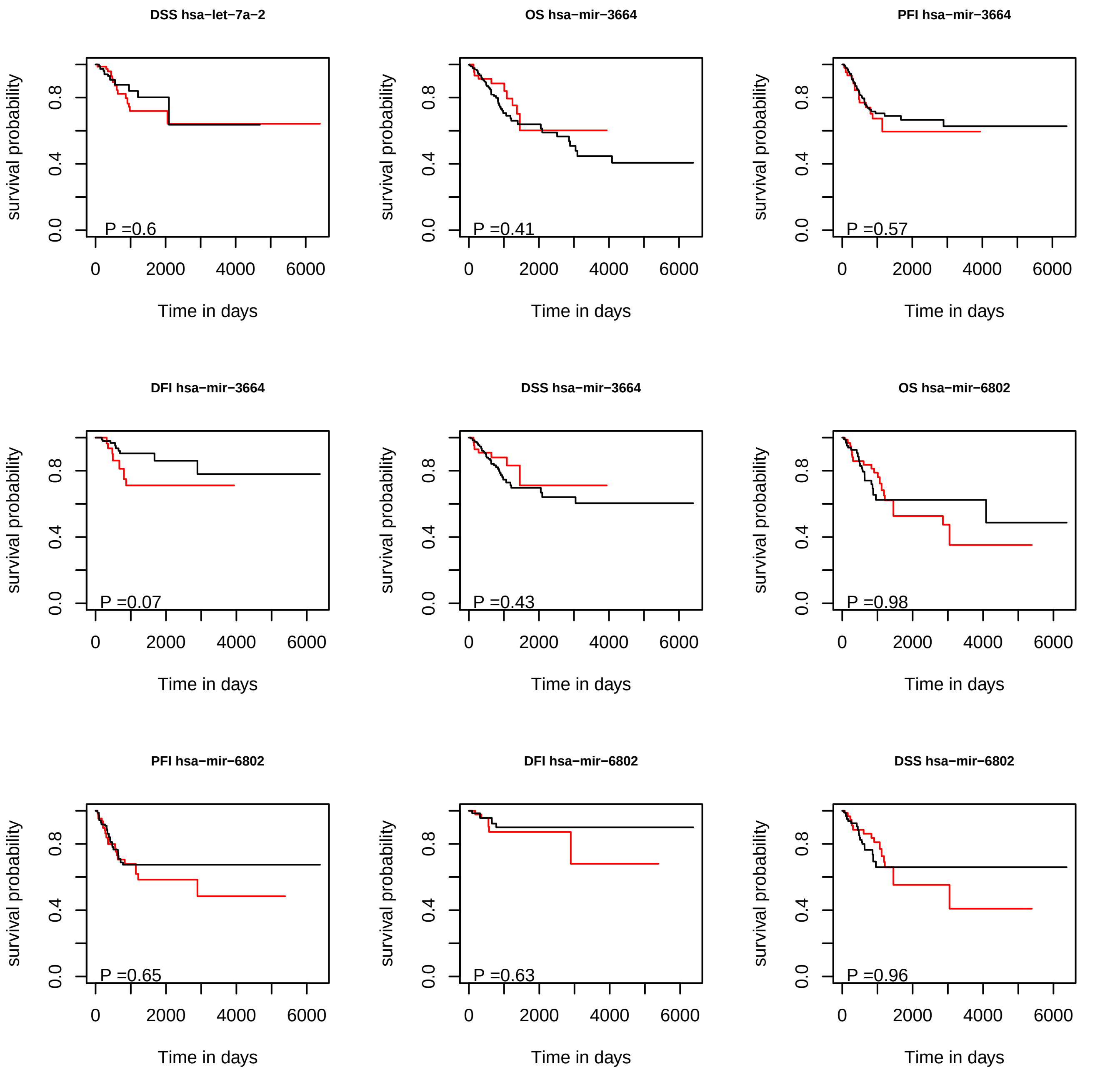
DSS hsa−let−7a−2
P =0.6
OS hsa−mir−3664
P =0.41
PFI hsa−mir−3664
P =0.57
DFI hsa−mir−3664
P =0.07
DSS hsa−mir−3664
P =0.43
OS hsa−mir−6802
P =0.98
PFI hsa−mir−6802
P =0.65
DFI hsa−mir−6802
P =0.63
DSS hsa−mir−6802
P =0.96
survival probability
0.0
0.4
0.8
Time in days
0
2000
4000
6000

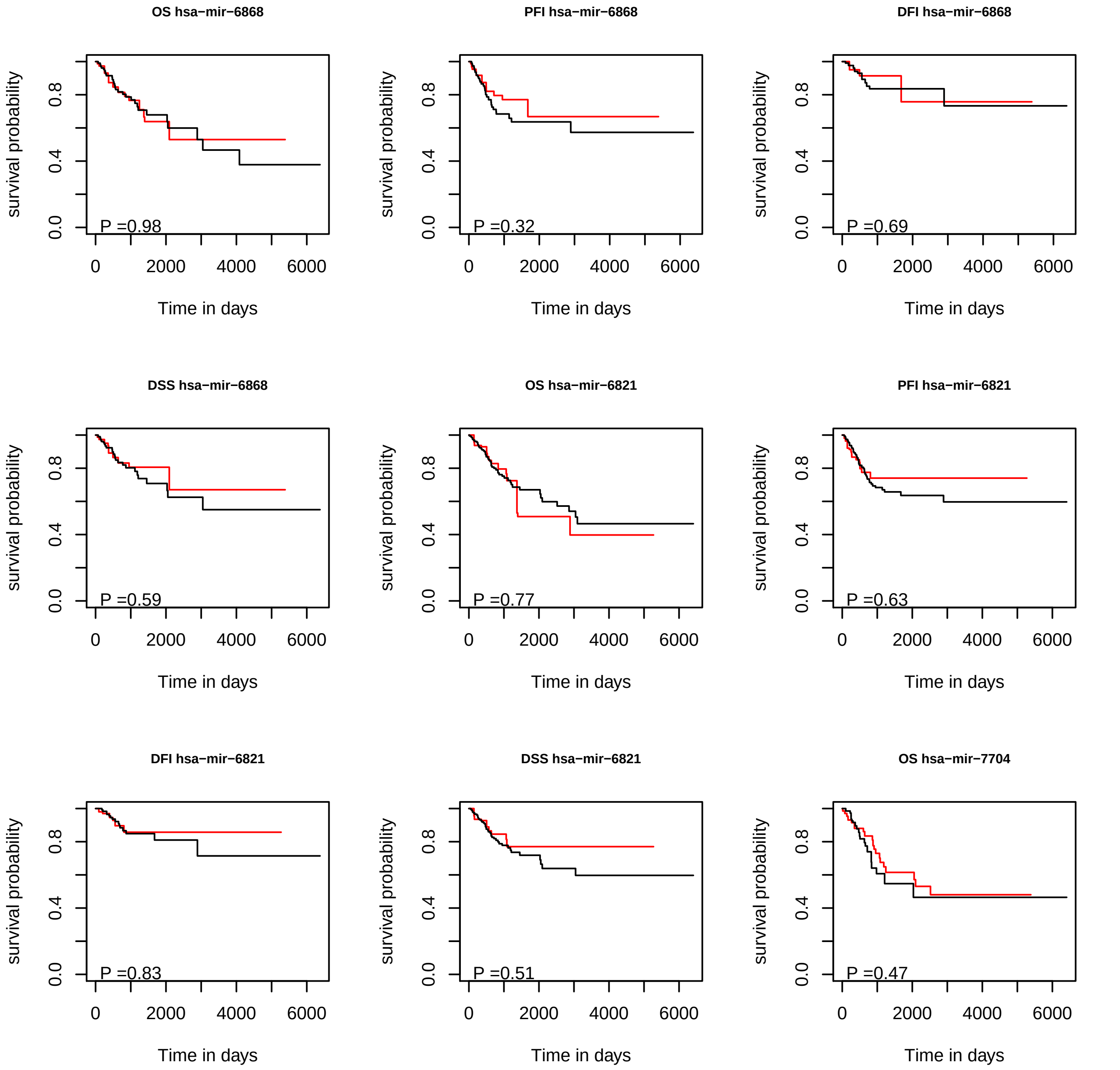
OS hsa−mir−6868
P =0.98
PFI hsa−mir−6868
P =0.32
DFI hsa−mir−6868
P =0.69
DSS hsa−mir−6868
P =0.59
OS hsa−mir−6821
P =0.77
PFI hsa−mir−6821
P =0.63
DFI hsa−mir−6821
P =0.83
DSS hsa−mir−6821
P =0.51
OS hsa−mir−7704
P =0.47
survival probability
Time in days
0.0
0.4
0.8
0
2000
4000
6000

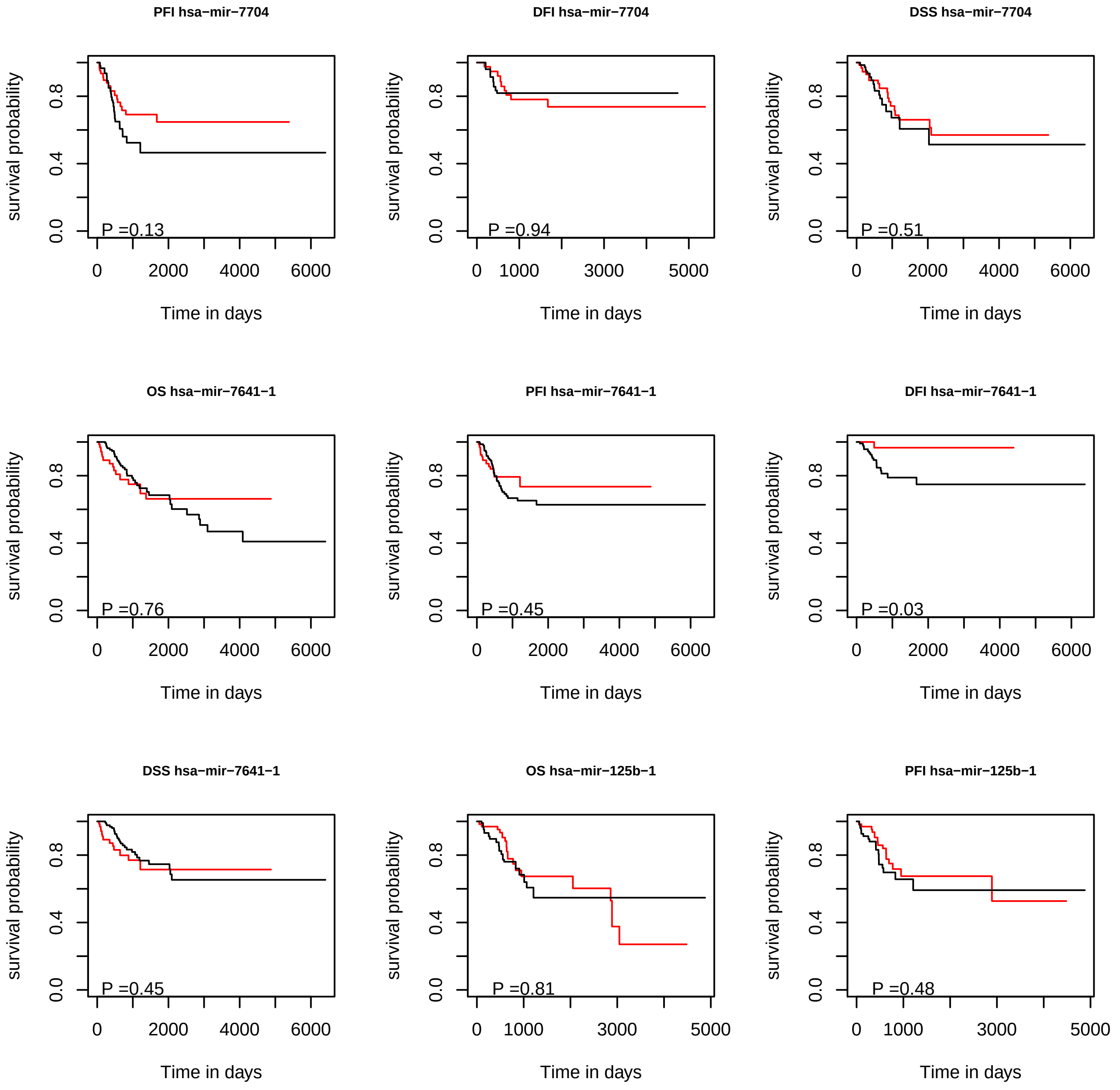

PFI hsa−mir−7704
P =0.13
DFI hsa−mir−7704
P =0.94
DSS hsa−mir−7704
P =0.51
OS hsa−mir−7641−1
P =0.76
PFI hsa−mir−7641−1
P =0.45
DFI hsa−mir−7641−1
P =0.03
DSS hsa−mir−7641−1
P =0.45
OS hsa−mir−125b−1
P =0.81
PFI hsa−mir−125b−1
P =0.48
survival probability
Time in days

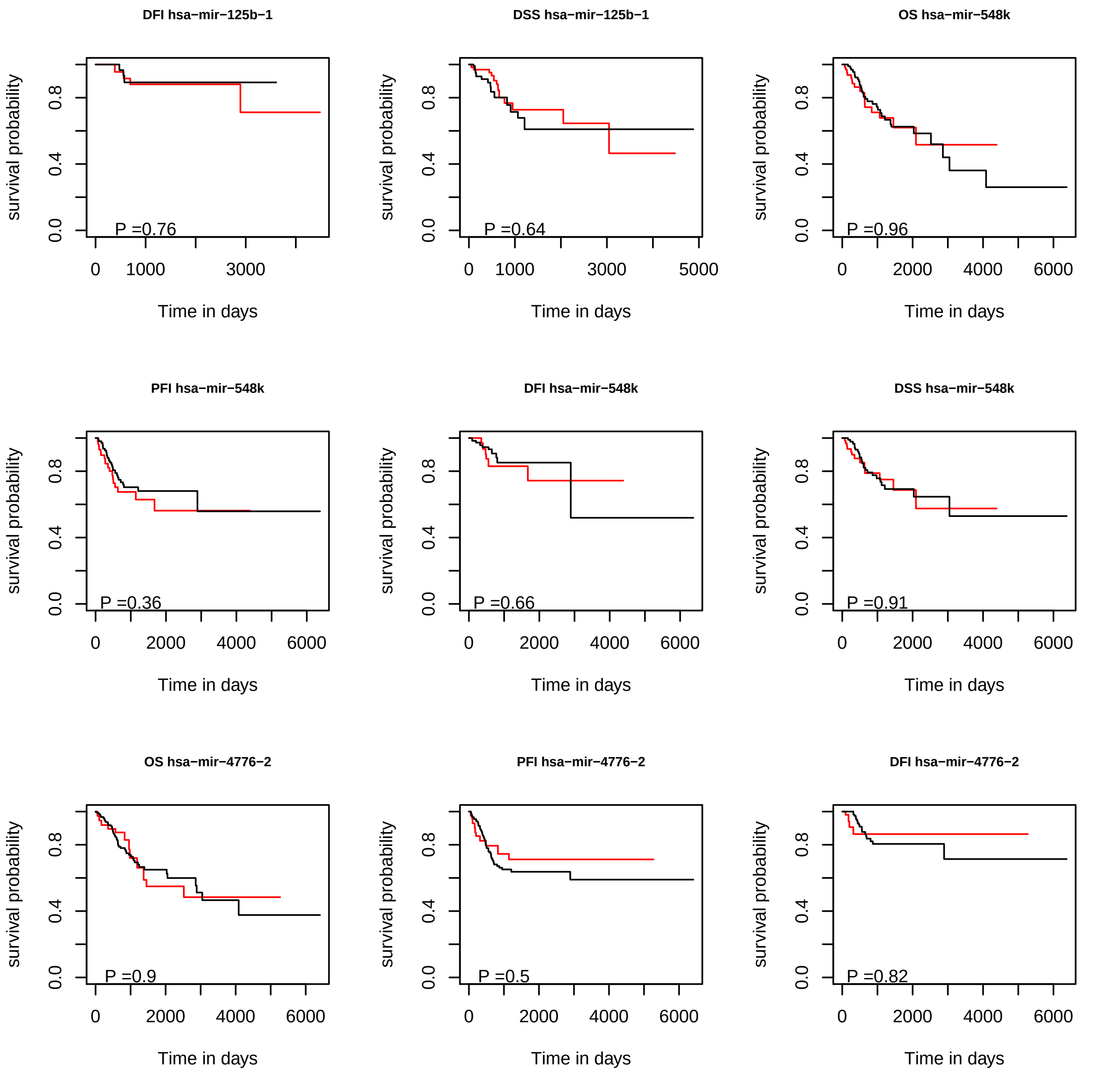
DFI hsa−mir−125b−1
P =0.76
DSS hsa−mir−125b−1
P =0.64
OS hsa−mir−548k
P =0.96
PFI hsa−mir−548k
P =0.36
DFI hsa−mir−548k
P =0.66
DSS hsa−mir−548k
P =0.91
OS hsa−mir−4776−2
P =0.9
PFI hsa−mir−4776−2
P =0.5
DFI hsa−mir−4776−2
P =0.82
survival probability
0.0
0.4
0.8
Time in days
0
1000
2000
3000
4000
5000
6000

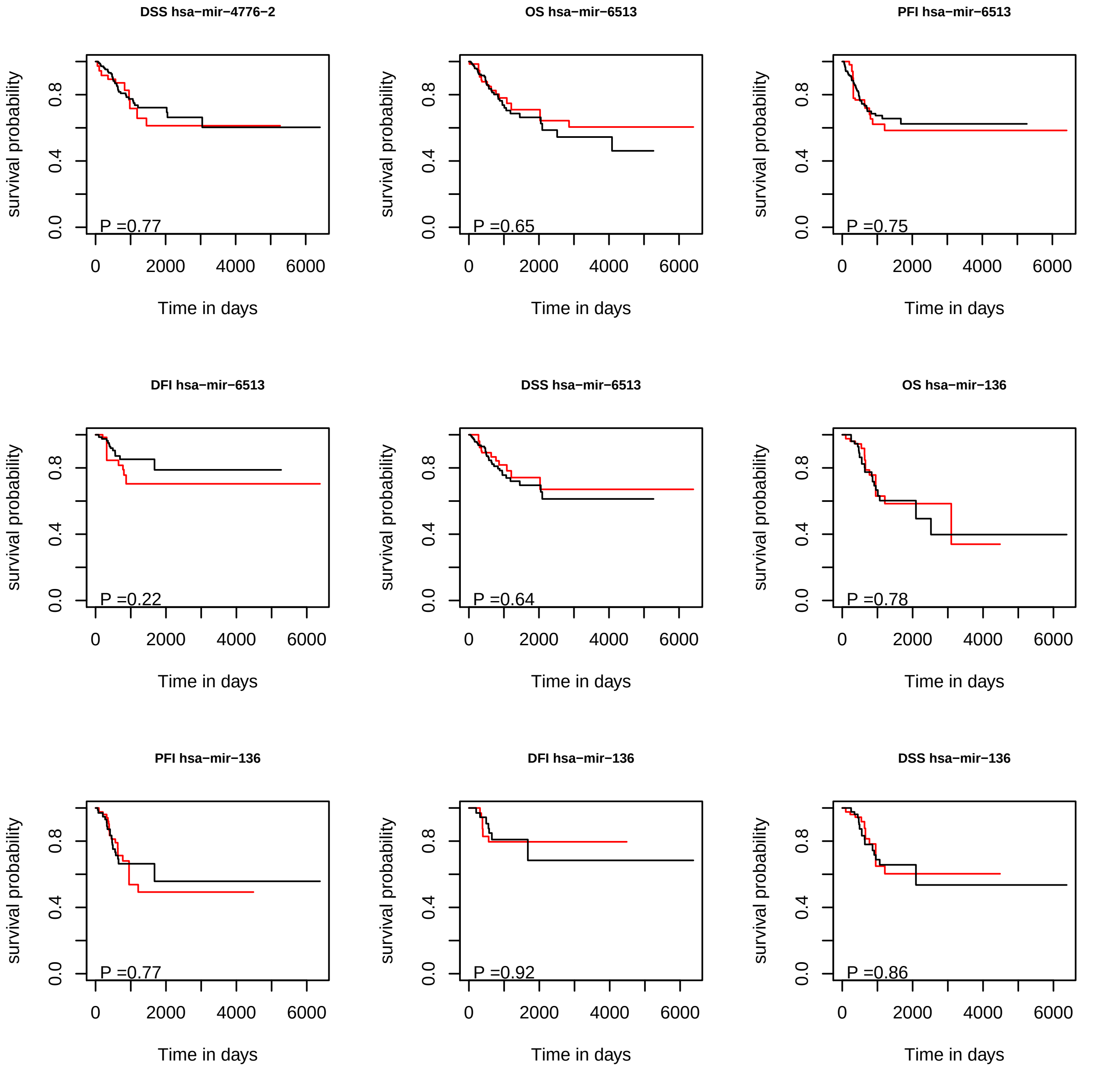
DSS hsa−mir−4776−2
P =0.77
OS hsa−mir−6513
P =0.65
PFI hsa−mir−6513
P =0.75
DFI hsa−mir−6513
P =0.22
DSS hsa−mir−6513
P =0.64
OS hsa−mir−136
P =0.78
PFI hsa−mir−136
P =0.77
DFI hsa−mir−136
P =0.92
DSS hsa−mir−136
P =0.86
survival probability
Time in days
0.0
0.4
0.8
0
2000
4000
6000

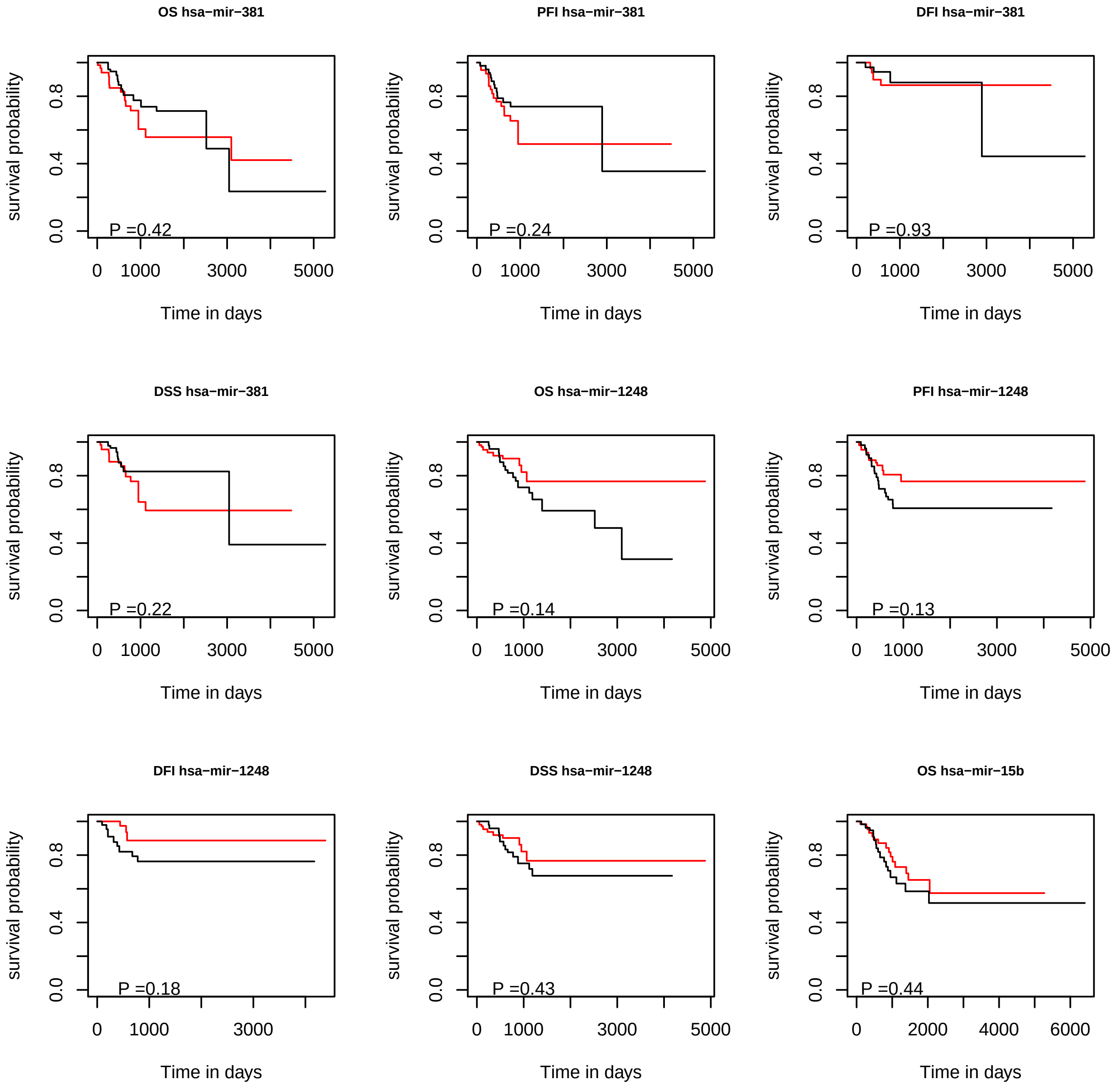
OS hsa−mir−381
P =0.42
PFI hsa−mir−381
P =0.24
DFI hsa−mir−381
P =0.93
DSS hsa−mir−381
P =0.22
OS hsa−mir−1248
P =0.14
PFI hsa−mir−1248
P =0.13
DFI hsa−mir−1248
P =0.18
DSS hsa−mir−1248
P =0.43
OS hsa−mir−15b
P =0.44
survival probability
Time in days

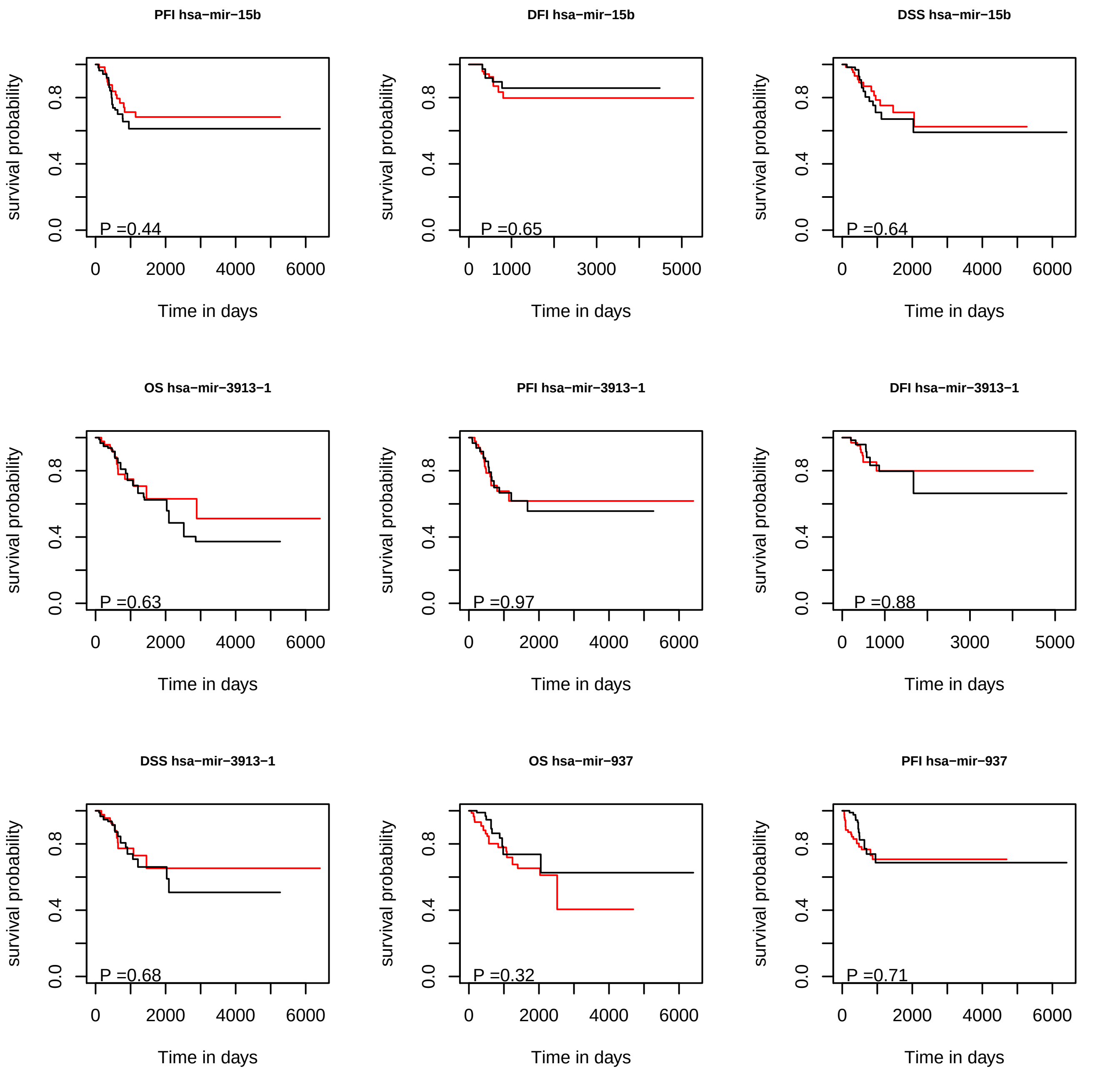

PFI hsa−mir−15b
P =0.44
DFI hsa−mir−15b
P =0.65
DSS hsa−mir−15b
P =0.64
OS hsa−mir−3913−1
P =0.63
PFI hsa−mir−3913−1
P =0.97
DFI hsa−mir−3913−1
P =0.88
DSS hsa−mir−3913−1
P =0.68
OS hsa−mir−937
P =0.32
PFI hsa−mir−937
P =0.71
survival probability
Time in days
0.0
0.4
0.8
0
1000
2000
3000
4000
5000
6000

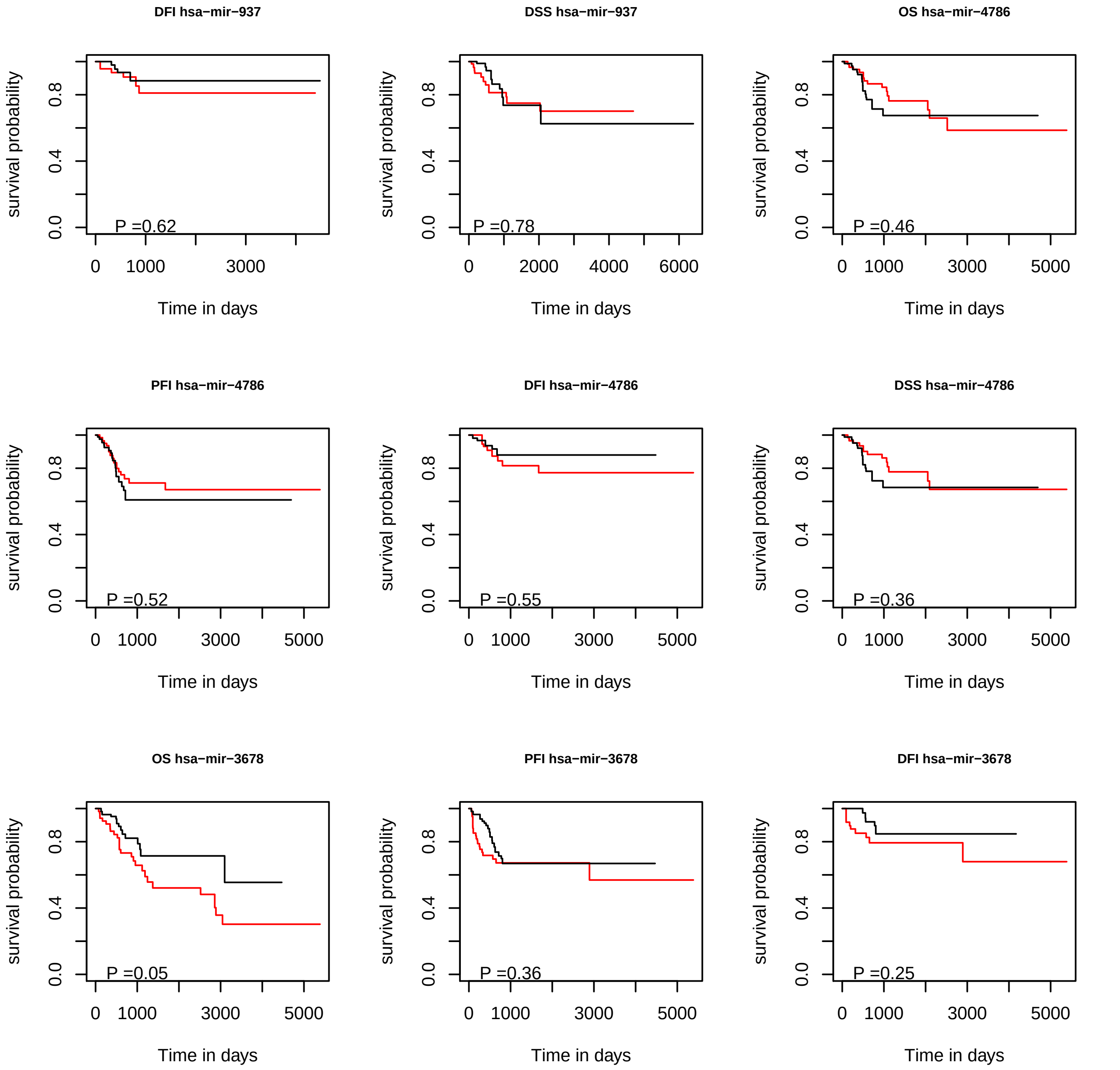
DFI hsa−mir−937
P =0.62
DSS hsa−mir−937
P =0.78
OS hsa−mir−4786
P =0.46
PFI hsa−mir−4786
P =0.52
DFI hsa−mir−4786
P =0.55
DSS hsa−mir−4786
P =0.36
OS hsa−mir−3678
P =0.05
PFI hsa−mir−3678
P =0.36
DFI hsa−mir−3678
P =0.25
survival probability
Time in days
0.0
0.4
0.8
0
1000
2000
3000
4000
5000
6000

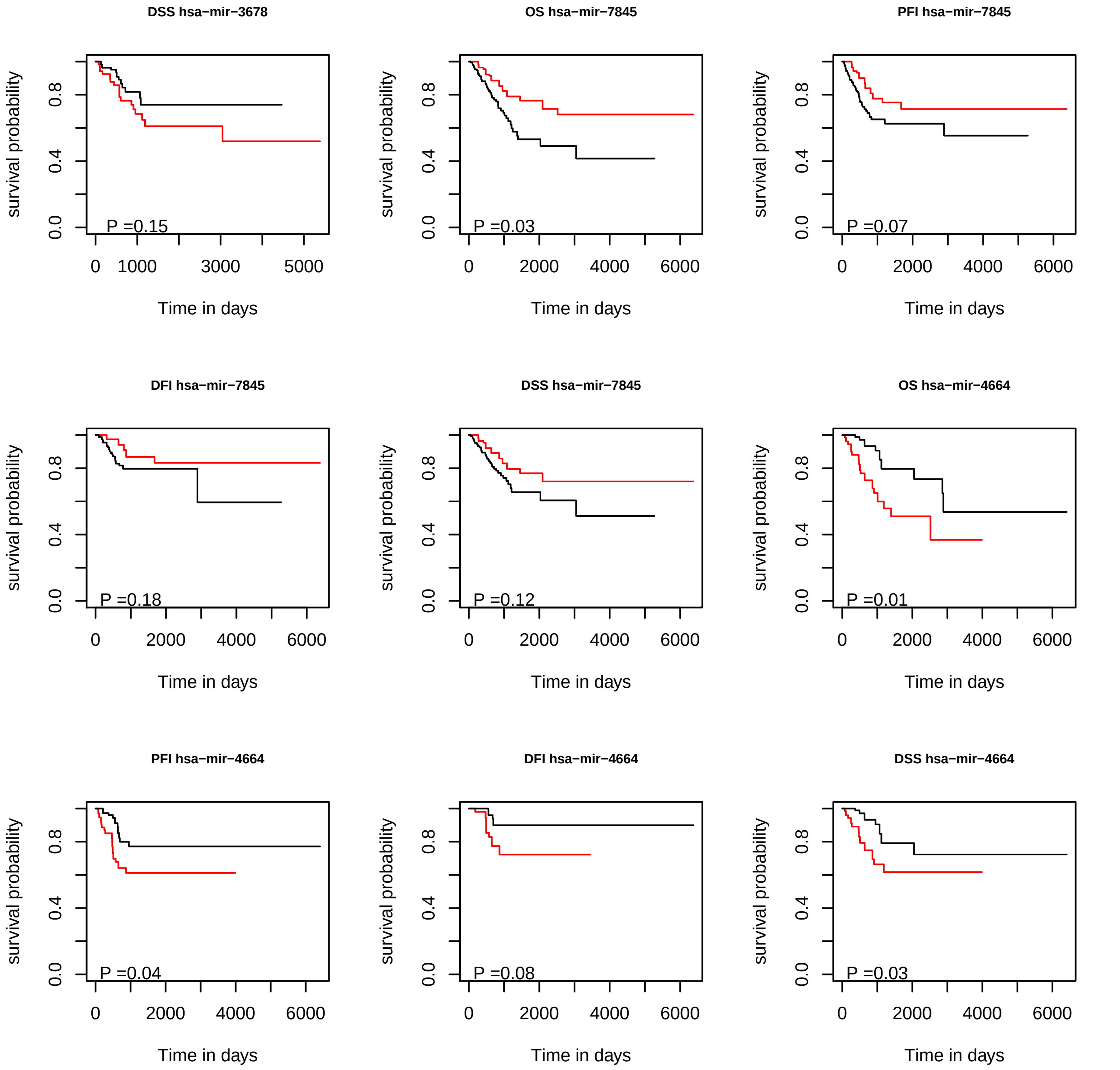

DSS hsa−mir−3678
P =0.15
OS hsa−mir−7845
P =0.03
PFI hsa−mir−7845
P =0.07
DFI hsa−mir−7845
P =0.18
DSS hsa−mir−7845
P =0.12
OS hsa−mir−4664
P =0.01
PFI hsa−mir−4664
P =0.04
DFI hsa−mir−4664
P =0.08
DSS hsa−mir−4664
P =0.03
survival probability
Time in days
0.0
0.4
0.8
0
1000
2000
3000
4000
5000
6000

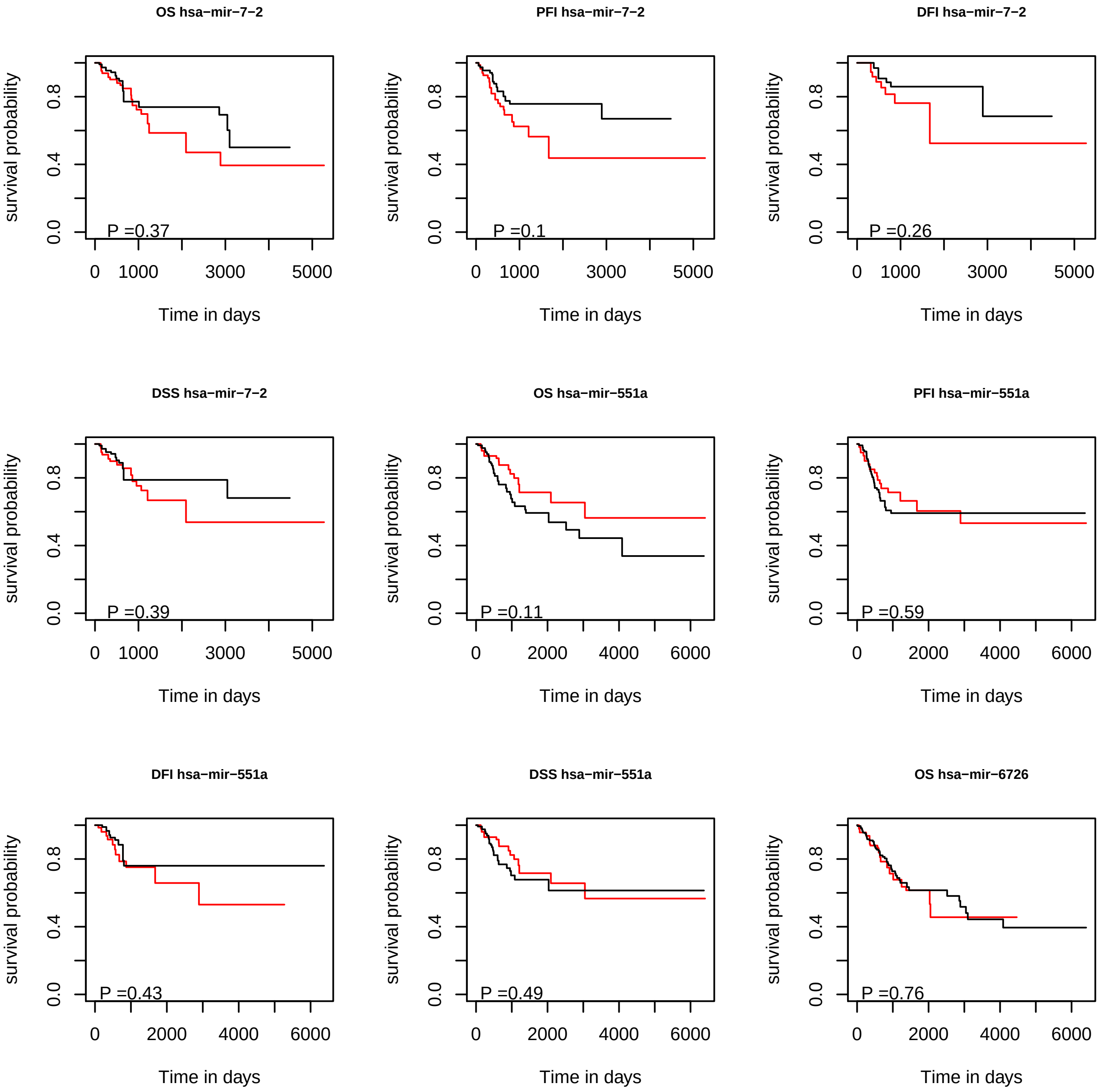
OS hsa−mir−7−2
P =0.37
PFI hsa−mir−7−2
P =0.1
DFI hsa−mir−7−2
P =0.26
DSS hsa−mir−7−2
P =0.39
OS hsa−mir−551a
P =0.11
PFI hsa−mir−551a
P =0.59
DFI hsa−mir−551a
P =0.43
DSS hsa−mir−551a
P =0.49
OS hsa−mir−6726
P =0.76
survival probability
0.0
0.4
0.8
Time in days
0
1000
3000
5000
2000
4000
6000

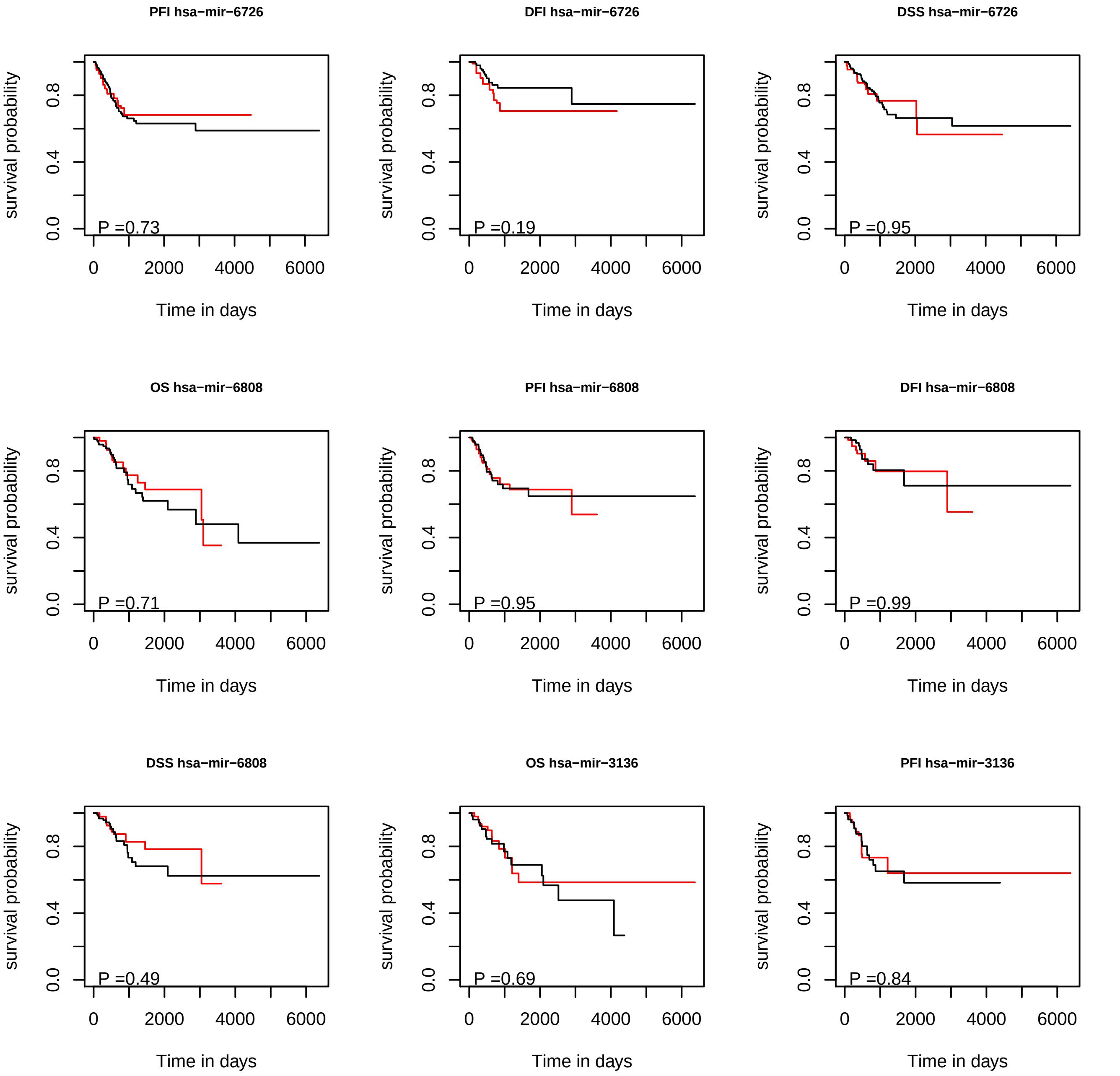

PFI hsa−mir−6726
P =0.73
DFI hsa−mir−6726
P =0.19
DSS hsa−mir−6726
P =0.95
OS hsa−mir−6808
P =0.71
PFI hsa−mir−6808
P =0.95
DFI hsa−mir−6808
P =0.99
DSS hsa−mir−6808
P =0.49
OS hsa−mir−3136
P =0.69
PFI hsa−mir−3136
P =0.84
survival probability
0.0
0.4
0.8
Time in days
0
2000
4000
6000

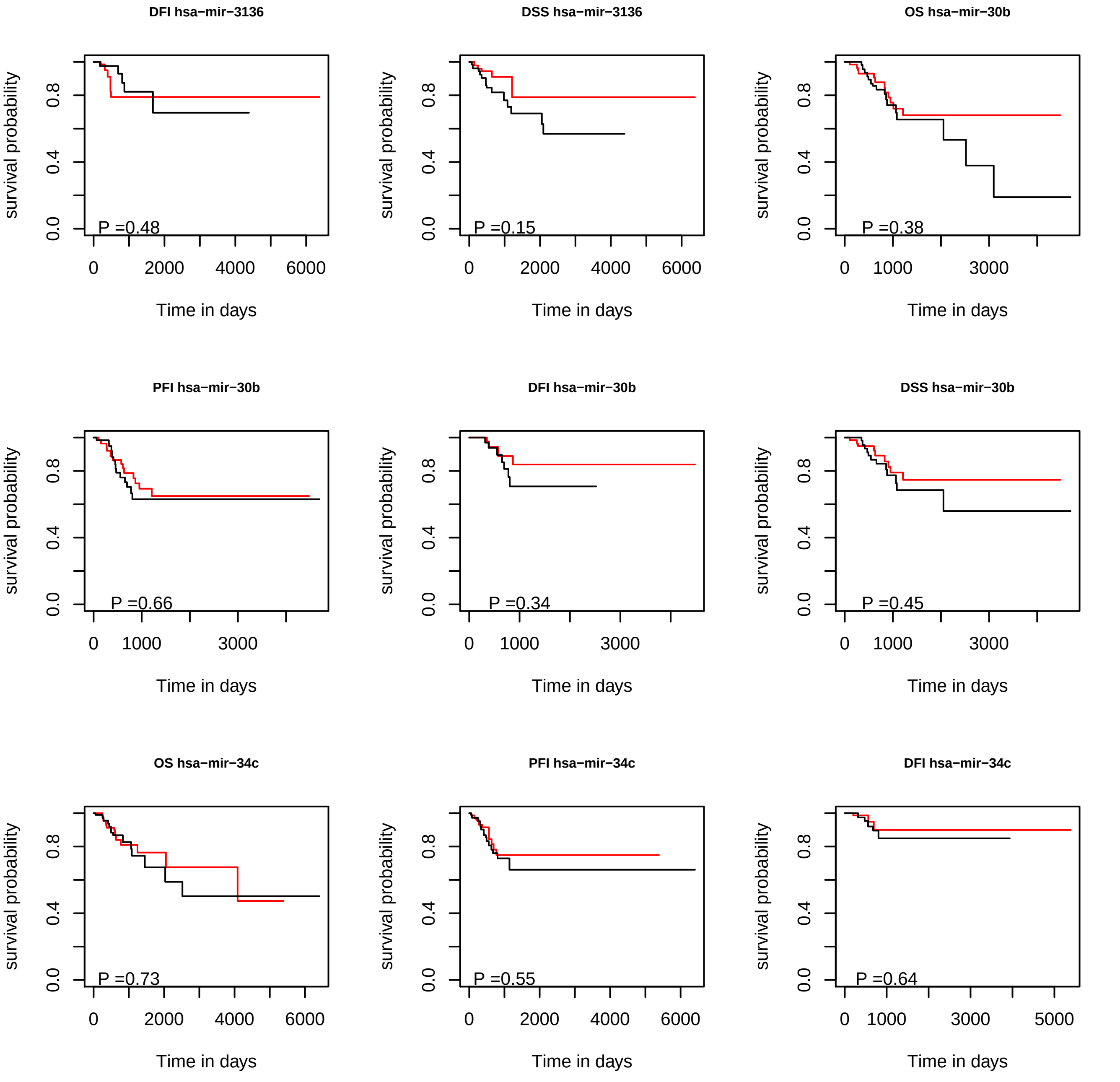
DFI hsa−mir−3136
P =0.48
DSS hsa−mir−3136
P =0.15
OS hsa−mir−30b
P =0.38
PFI hsa−mir−30b
P =0.66
DFI hsa−mir−30b
P =0.34
DSS hsa−mir−30b
P =0.45
OS hsa−mir−34c
P =0.73
PFI hsa−mir−34c
P =0.55
DFI hsa−mir−34c
P =0.64
survival probability
Time in days
0.0
0.4
0.8
0
1000
2000
3000
4000
5000
6000

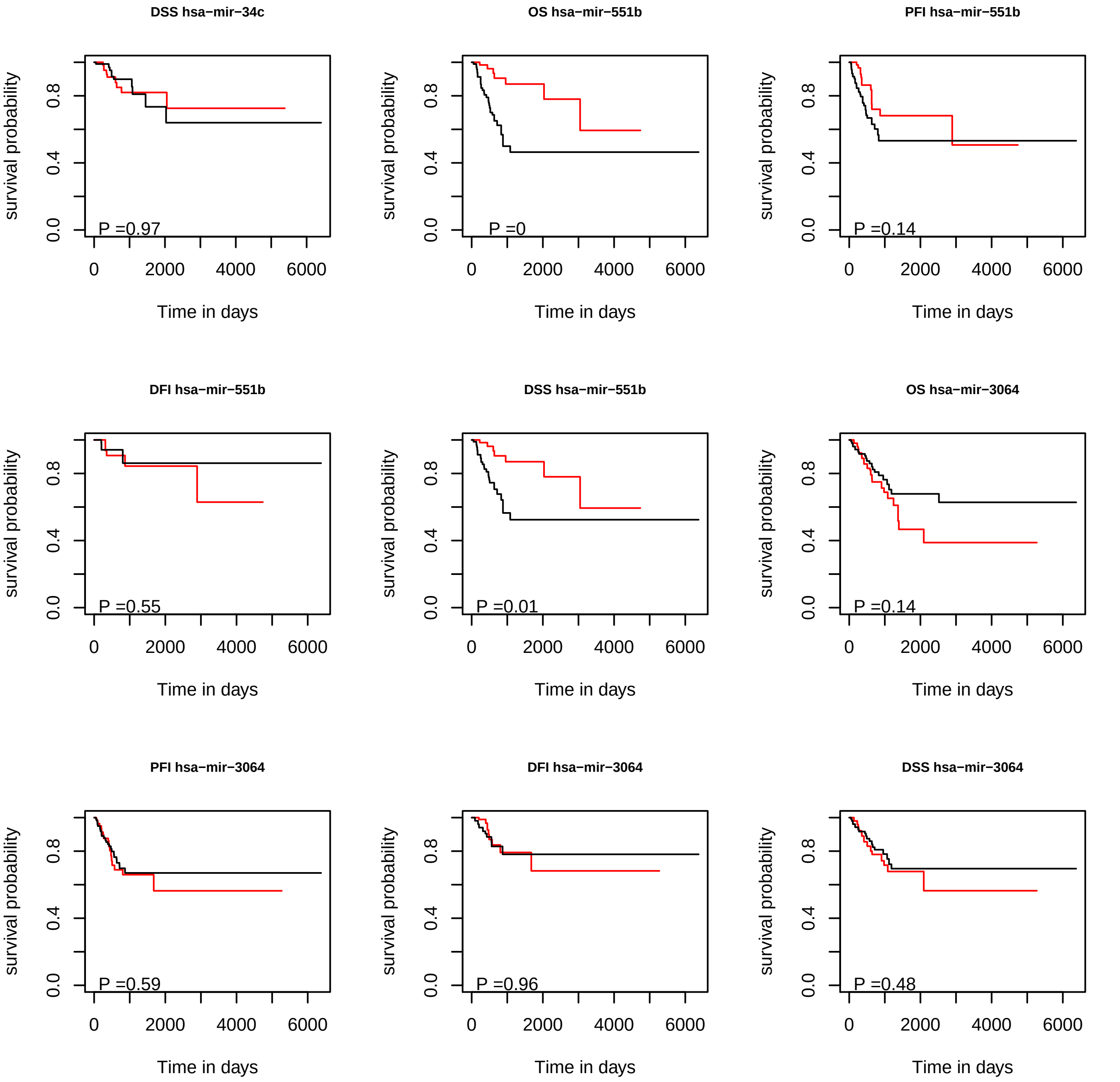
DSS hsa−mir−34c
survival probability
0.0
0.4
0.8
P =0.97
0
2000
4000
6000
Time in days
OS hsa−mir−551b
survival probability
0.0
0.4
0.8
P =0
0
2000
4000
6000
Time in days
PFI hsa−mir−551b
survival probability
0.0
0.4
0.8
P =0.14
0
2000
4000
6000
Time in days
DFI hsa−mir−551b
survival probability
0.0
0.4
0.8
P =0.55
0
2000
4000
6000
Time in days
DSS hsa−mir−551b
survival probability
0.0
0.4
0.8
P =0.01
0
2000
4000
6000
Time in days
OS hsa−mir−3064
survival probability
0.0
0.4
0.8
P =0.14
0
2000
4000
6000
Time in days
PFI hsa−mir−3064
survival probability
0.0
0.4
0.8
P =0.59
0
2000
4000
6000
Time in days
DFI hsa−mir−3064
survival probability
0.0
0.4
0.8
P =0.96
0
2000
4000
6000
Time in days
DSS hsa−mir−3064
survival probability
0.0
0.4
0.8
P =0.48
0
2000
4000
6000
Time in days

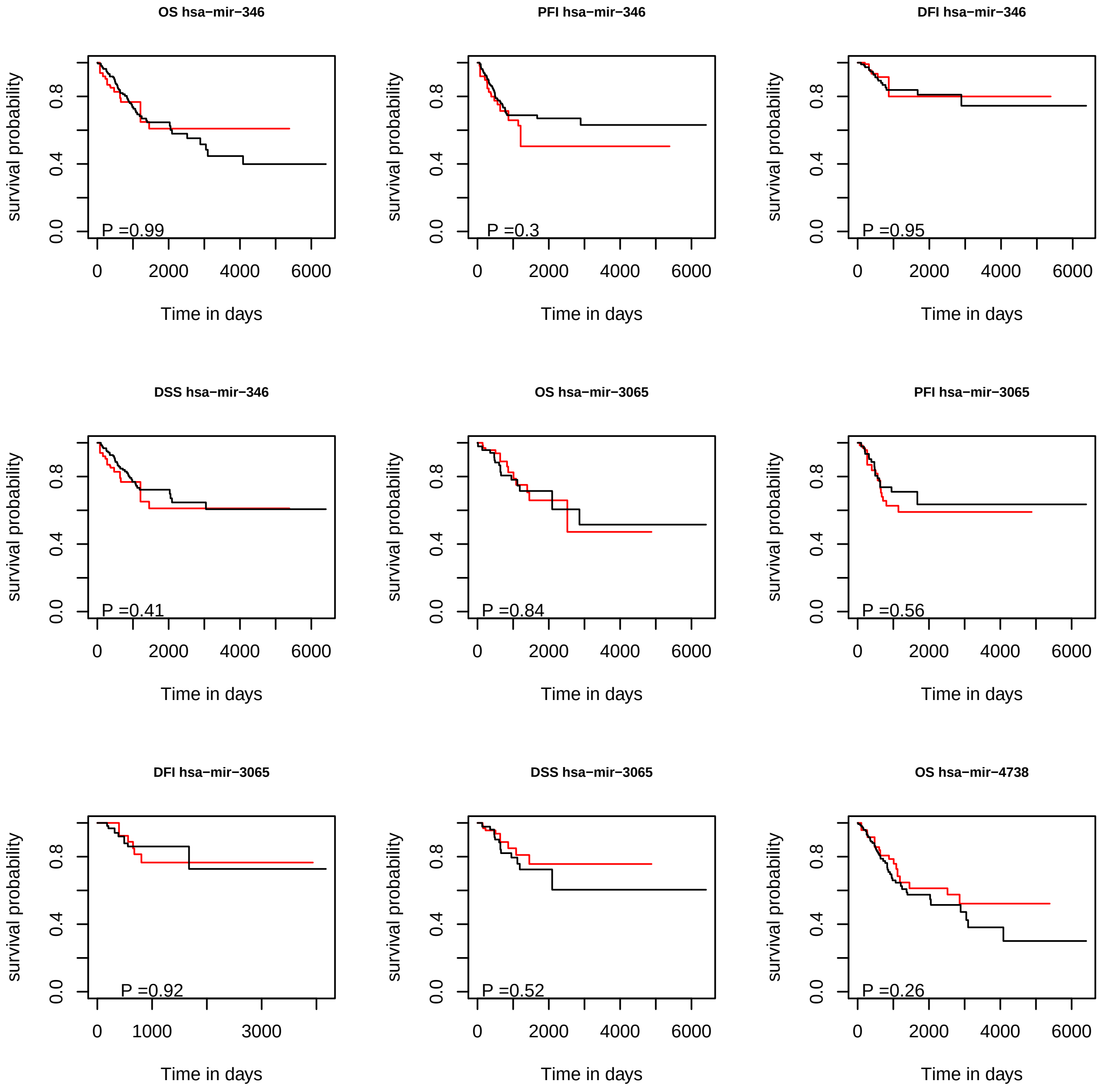
OS hsa−mir−346
P =0.99
PFI hsa−mir−346
P =0.3
DFI hsa−mir−346
P =0.95
DSS hsa−mir−346
P =0.41
OS hsa−mir−3065
P =0.84
PFI hsa−mir−3065
P =0.56
DFI hsa−mir−3065
P =0.92
DSS hsa−mir−3065
P =0.52
OS hsa−mir−4738
P =0.26
survival probability
Time in days
0.0
0.4
0.8
0
1000
2000
3000
4000
6000

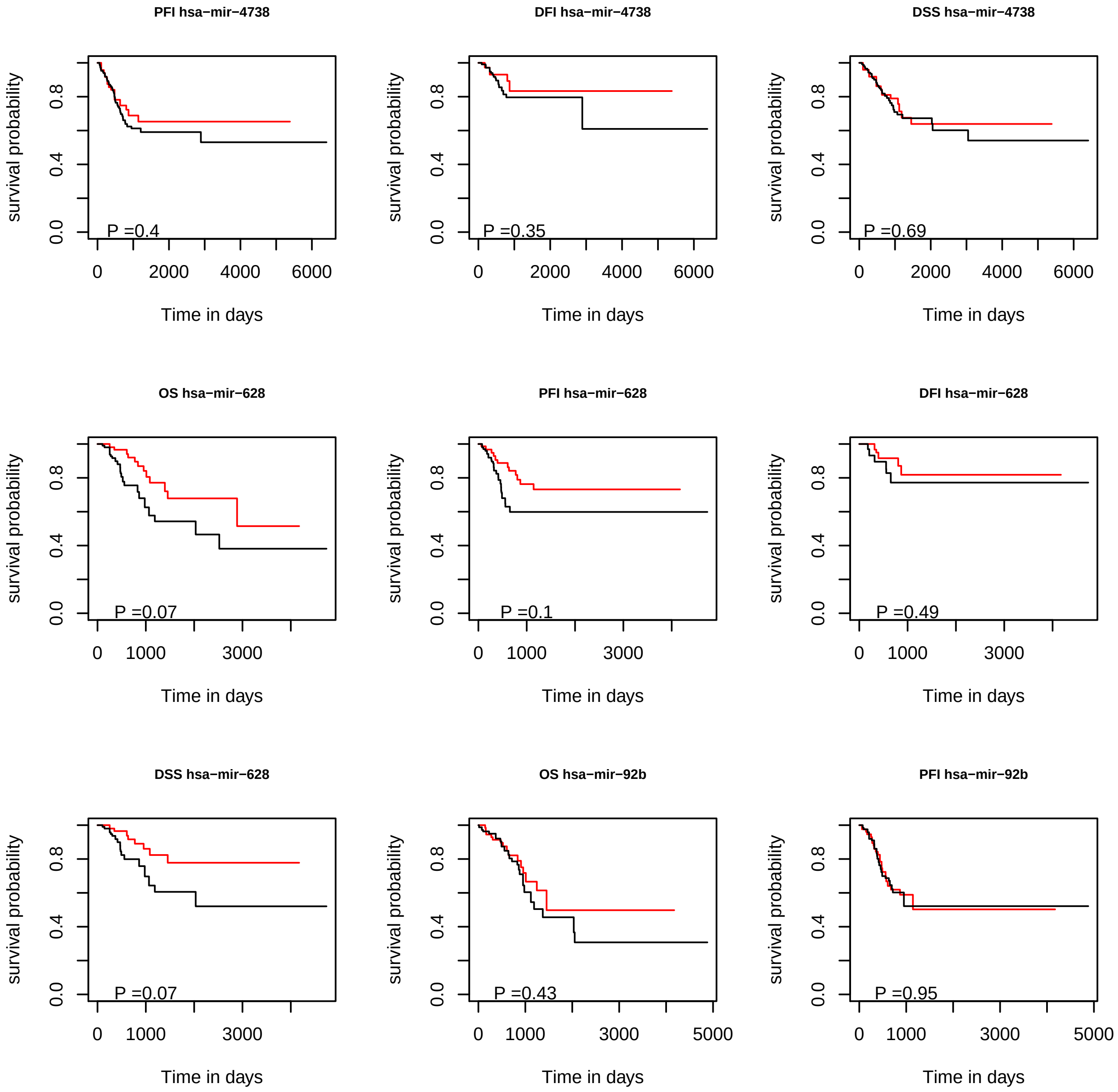
PFI hsa−mir−4738
P =0.4
DFI hsa−mir−4738
P =0.35
DSS hsa−mir−4738
P =0.69
OS hsa−mir−628
P =0.07
PFI hsa−mir−628
P =0.1
DFI hsa−mir−628
P =0.49
DSS hsa−mir−628
P =0.07
OS hsa−mir−92b
P =0.43
PFI hsa−mir−92b
P =0.95
survival probability
Time in days

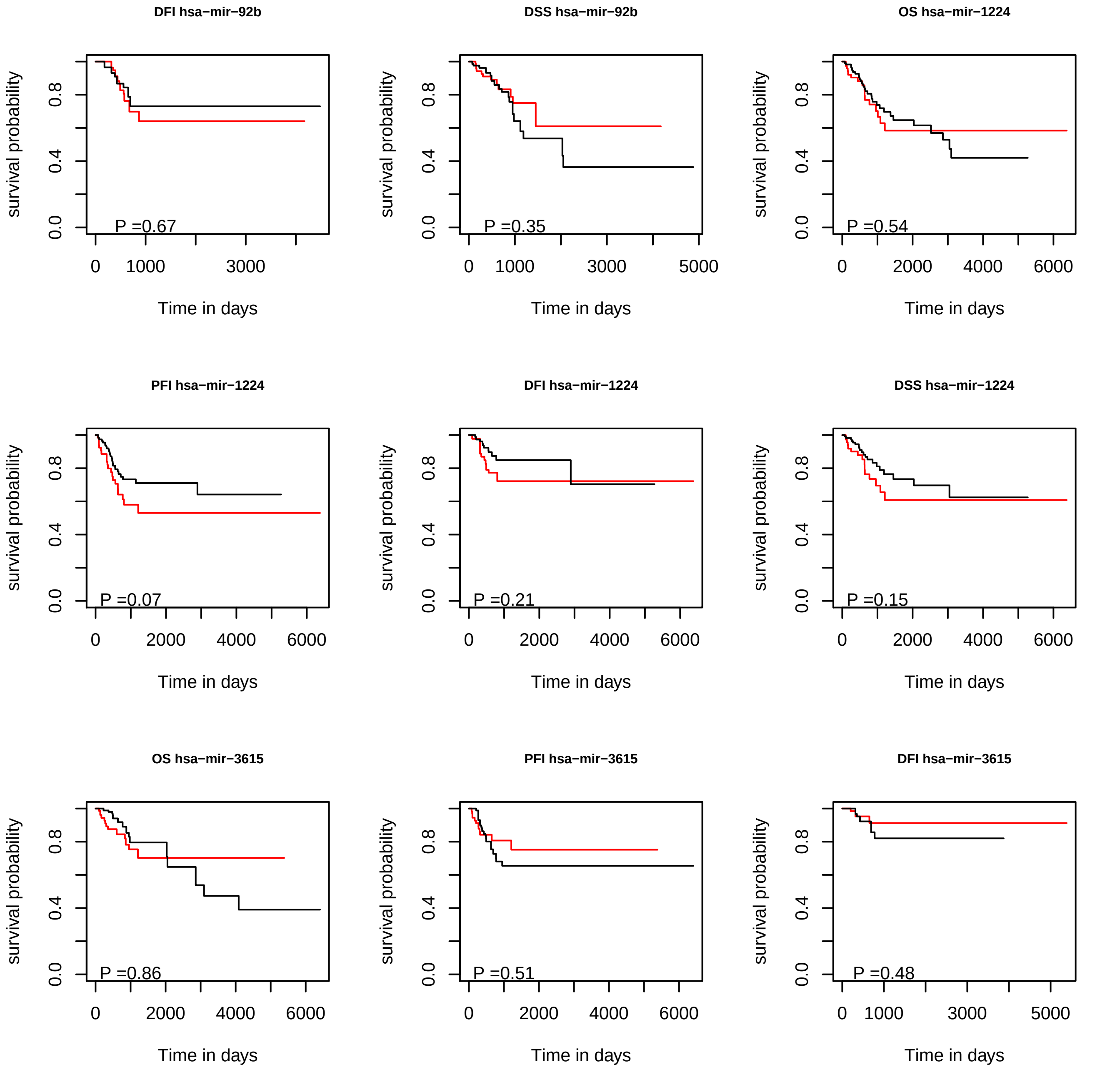
DFI hsa−mir−92b
survival probability
0.0
0.4
0.8
0
1000
3000
Time in days
P =0.67
DSS hsa−mir−92b
survival probability
0.0
0.4
0.8
0
1000
3000
5000
Time in days
P =0.35
OS hsa−mir−1224
survival probability
0.0
0.4
0.8
0
2000
4000
6000
Time in days
P =0.54
PFI hsa−mir−1224
survival probability
0.0
0.4
0.8
0
2000
4000
6000
Time in days
P =0.07
DFI hsa−mir−1224
survival probability
0.0
0.4
0.8
0
2000
4000
6000
Time in days
P =0.21
DSS hsa−mir−1224
survival probability
0.0
0.4
0.8
0
2000
4000
6000
Time in days
P =0.15
OS hsa−mir−3615
survival probability
0.0
0.4
0.8
0
2000
4000
6000
Time in days
P =0.86
PFI hsa−mir−3615
survival probability
0.0
0.4
0.8
0
2000
4000
6000
Time in days
P =0.51
DFI hsa−mir−3615
survival probability
0.0
0.4
0.8
0
1000
3000
5000
Time in days
P =0.48

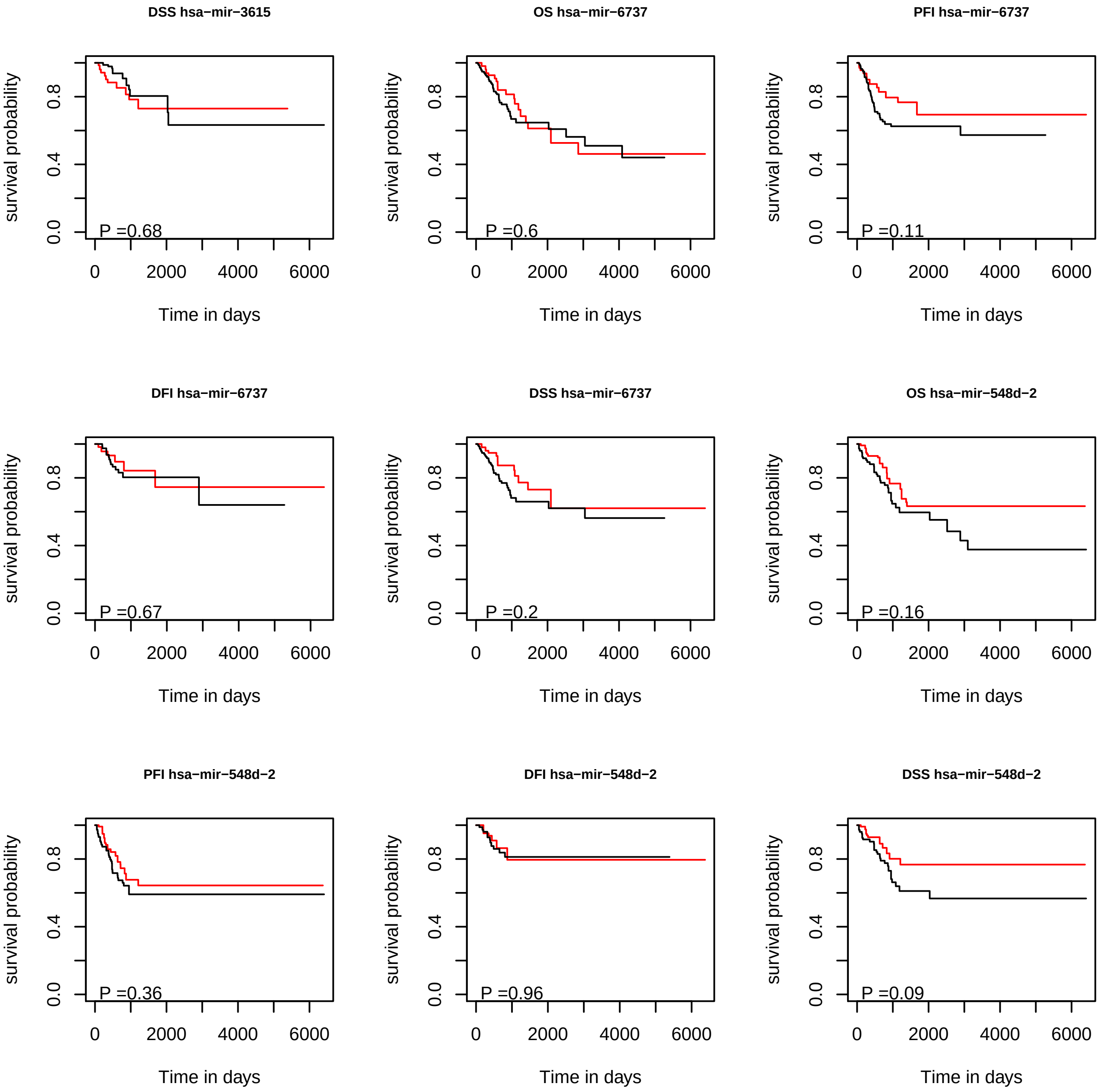
DSS hsa−mir−3615
P =0.68
OS hsa−mir−6737
P =0.6
PFI hsa−mir−6737
P =0.11
DFI hsa−mir−6737
P =0.67
DSS hsa−mir−6737
P =0.2
OS hsa−mir−548d−2
P =0.16
PFI hsa−mir−548d−2
P =0.36
DFI hsa−mir−548d−2
P =0.96
DSS hsa−mir−548d−2
P =0.09
survival probability
0.0
0.4
0.8
0
2000
4000
6000
Time in days

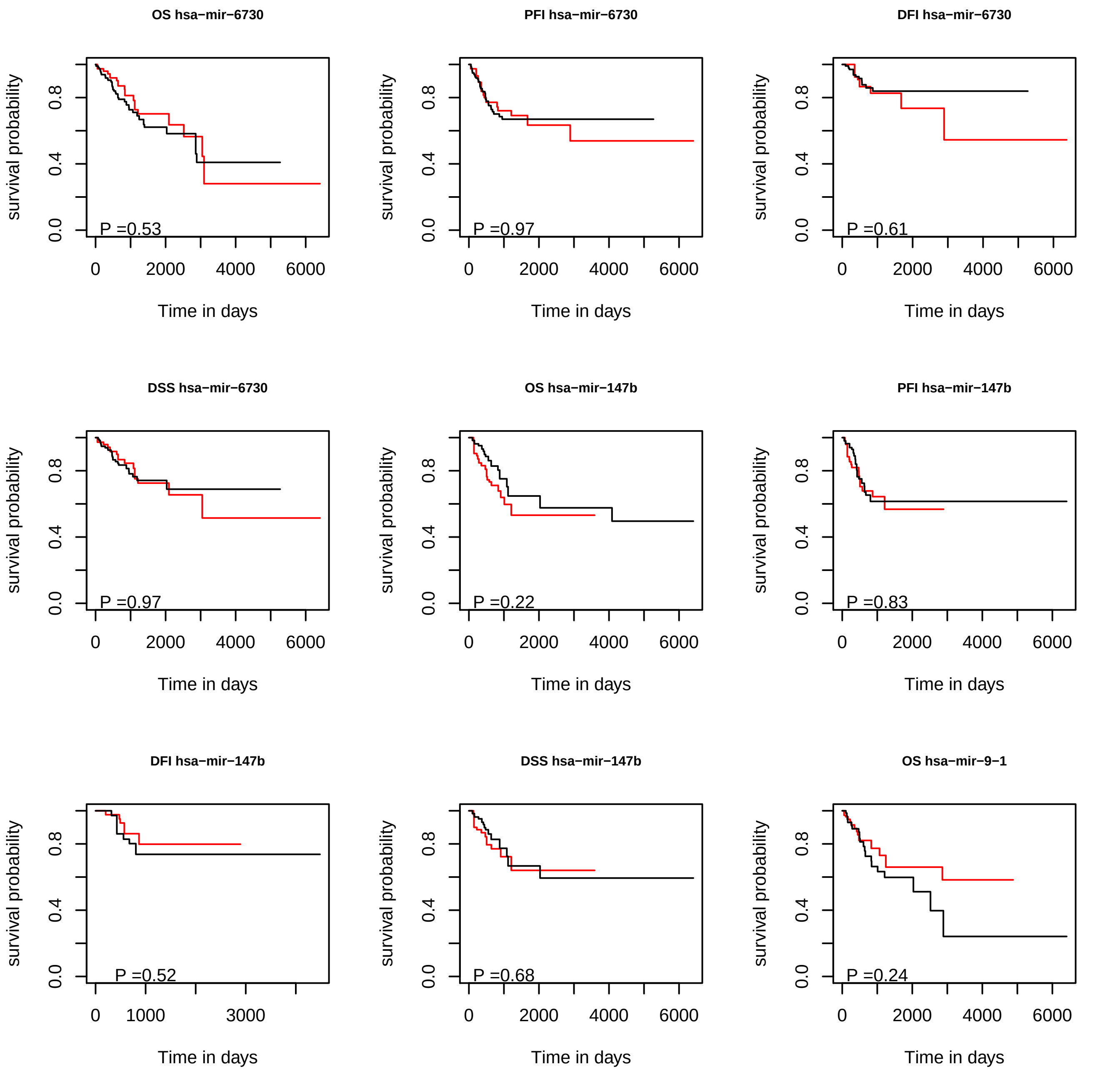
OS hsa−mir−6730
P =0.53
PFI hsa−mir−6730
P =0.97
DFI hsa−mir−6730
P =0.61
DSS hsa−mir−6730
P =0.97
OS hsa−mir−147b
P =0.22
PFI hsa−mir−147b
P =0.83
DFI hsa−mir−147b
P =0.52
DSS hsa−mir−147b
P =0.68
OS hsa−mir−9−1
P =0.24
survival probability
Time in days
0.0
0.4
0.8
0
1000
2000
3000
4000
6000

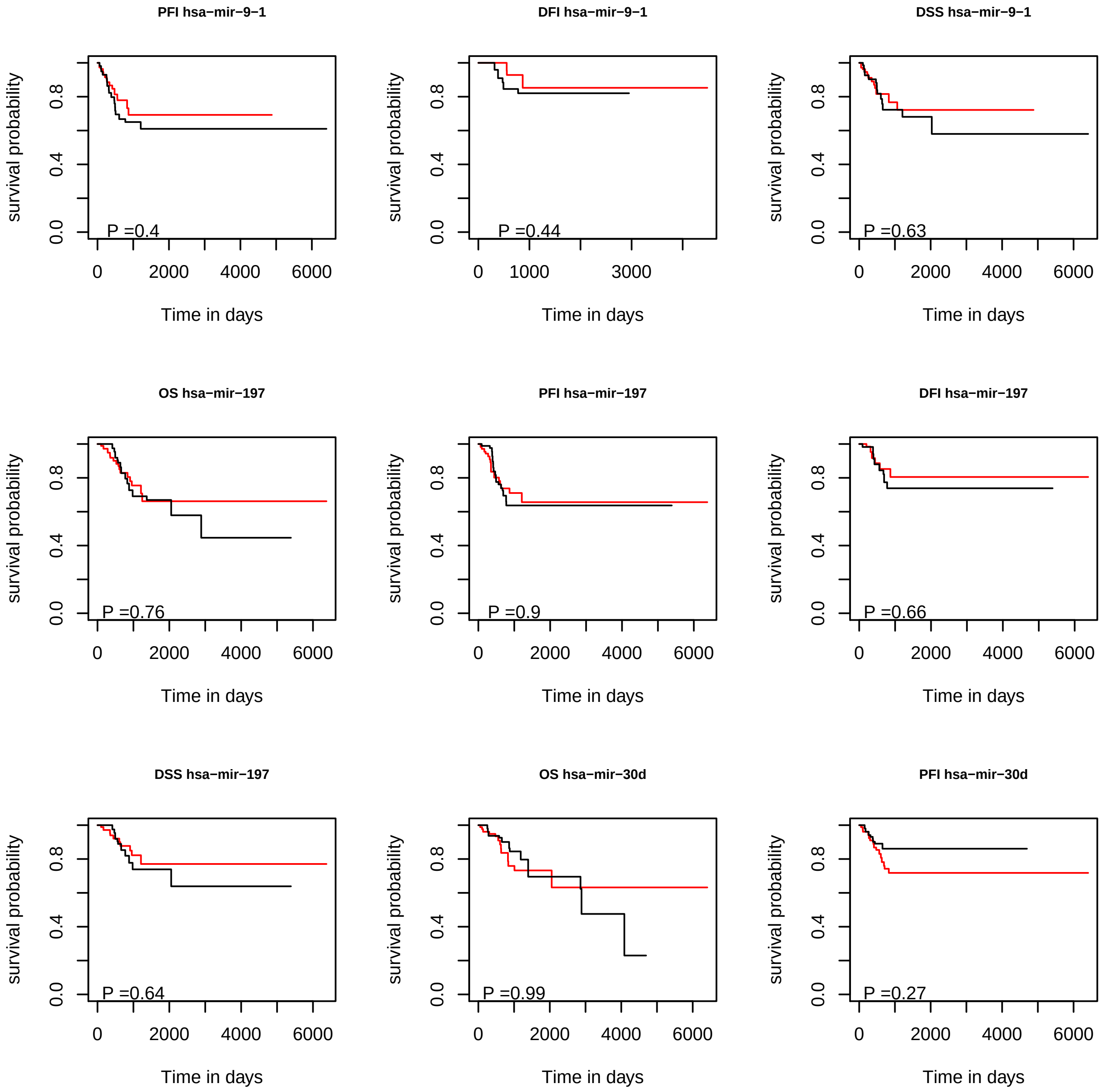
PFI hsa−mir−9−1
P =0.4
DFI hsa−mir−9−1
P =0.44
DSS hsa−mir−9−1
P =0.63
OS hsa−mir−197
P =0.76
PFI hsa−mir−197
P =0.9
DFI hsa−mir−197
P =0.66
DSS hsa−mir−197
P =0.64
OS hsa−mir−30d
P =0.99
PFI hsa−mir−30d
P =0.27
survival probability
Time in days

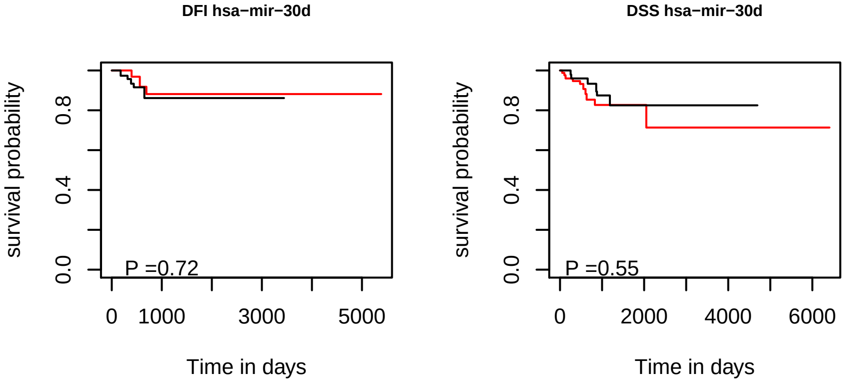

DFI hsa−mir−30d
survival probability
0.0
0.4
0.8
P =0.72
0
1000
3000
5000
Time in days
DSS hsa−mir−30d
survival probability
0.0
0.4
0.8
P =0.55
0
2000
4000
6000
Time in days
