# Supplementary material for: Computing microRNA-gene interaction networks in pan-cancer using miRDriver
Source: Sci Rep. 2022 Mar 8;12:3717. doi: 10.1038/s41598-022-07628-z (PMC8904490; doi:10.1038/s41598-022-07628-z)
Supplement: Supplementary file 14 — Supplementary Information 14. [file 41598_2022_7628_MOESM14_ESM.pdf]

Computing microRNA-gene interaction networks in pan-cancer using miRDriver

Banabithi Bose, Matthew Moravec, and Serdar Bozdag

Supplemental Figure S5

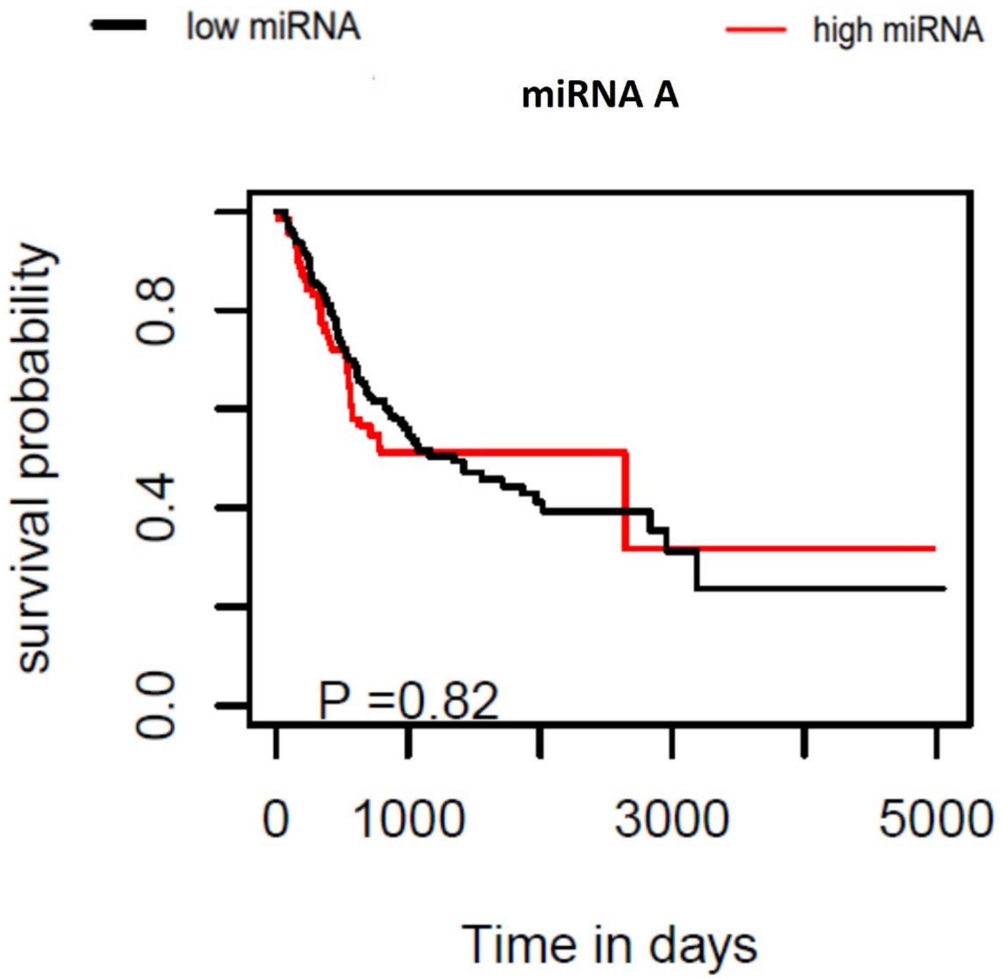

The *Adjusted Kaplan-Meier* survival plots for the computed miRNAs in high and low miRNA expression patient groups.

Supplemental Figure S5 Cancer Type: DLBC

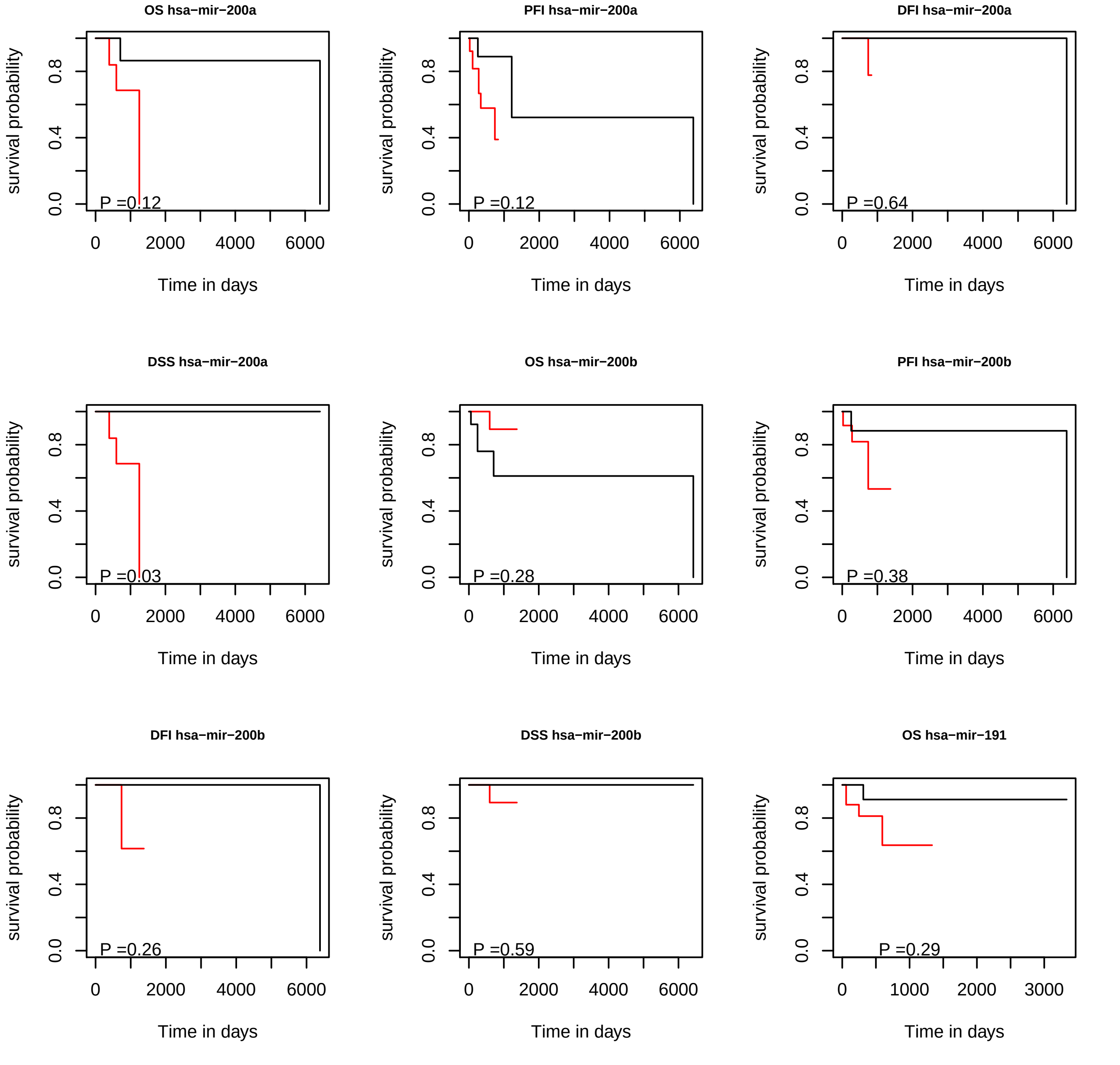

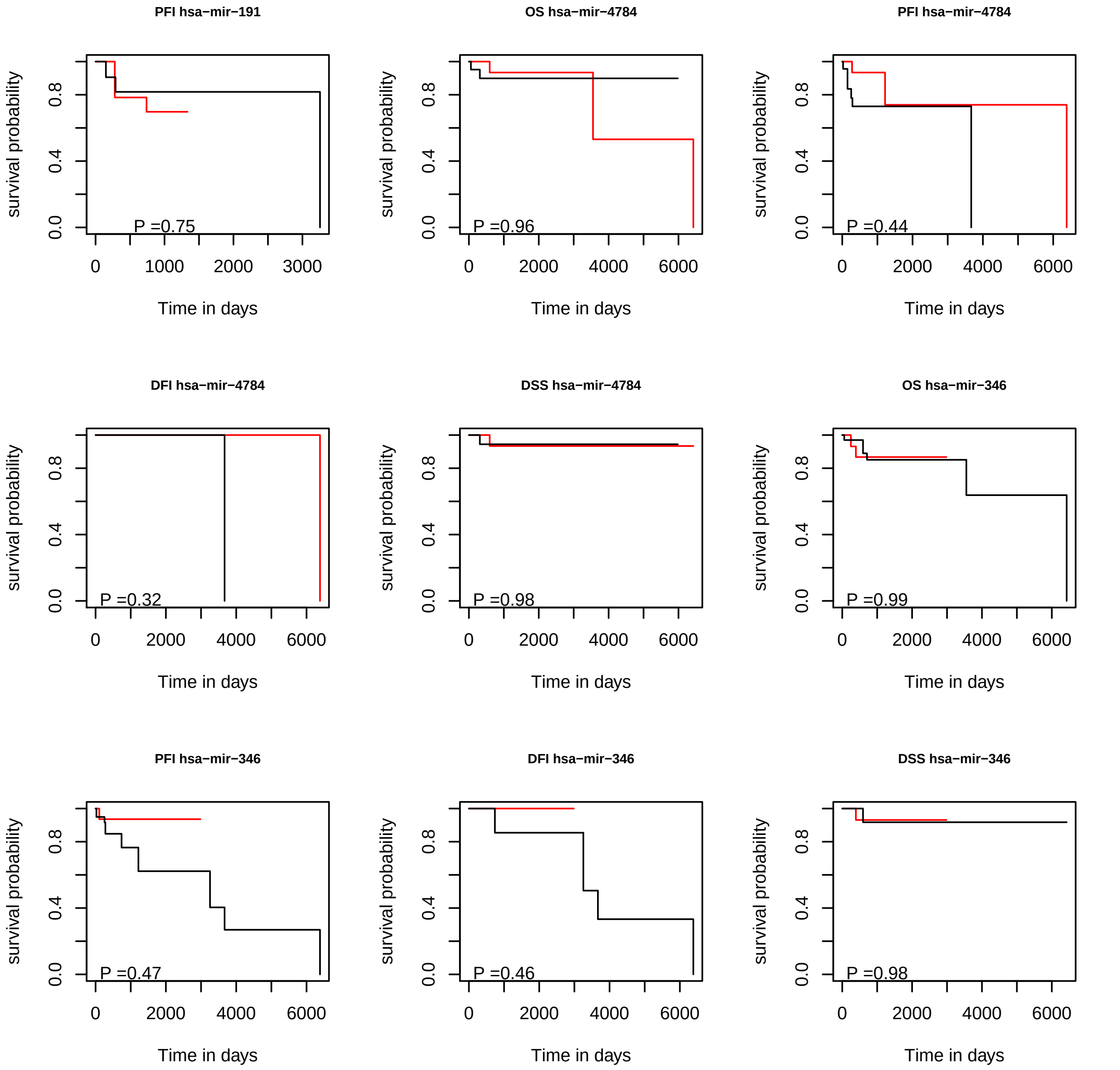
PFI hsa−mir−191
P =0.75
OS hsa−mir−4784
P =0.96
PFI hsa−mir−4784
P =0.44
DFI hsa−mir−4784
P =0.32
DSS hsa−mir−4784
P =0.98
OS hsa−mir−346
P =0.99
PFI hsa−mir−346
P =0.47
DFI hsa−mir−346
P =0.46
DSS hsa−mir−346
P =0.98
survival probability
0.0
0.4
0.8
Time in days
0
1000
2000
3000
4000
6000

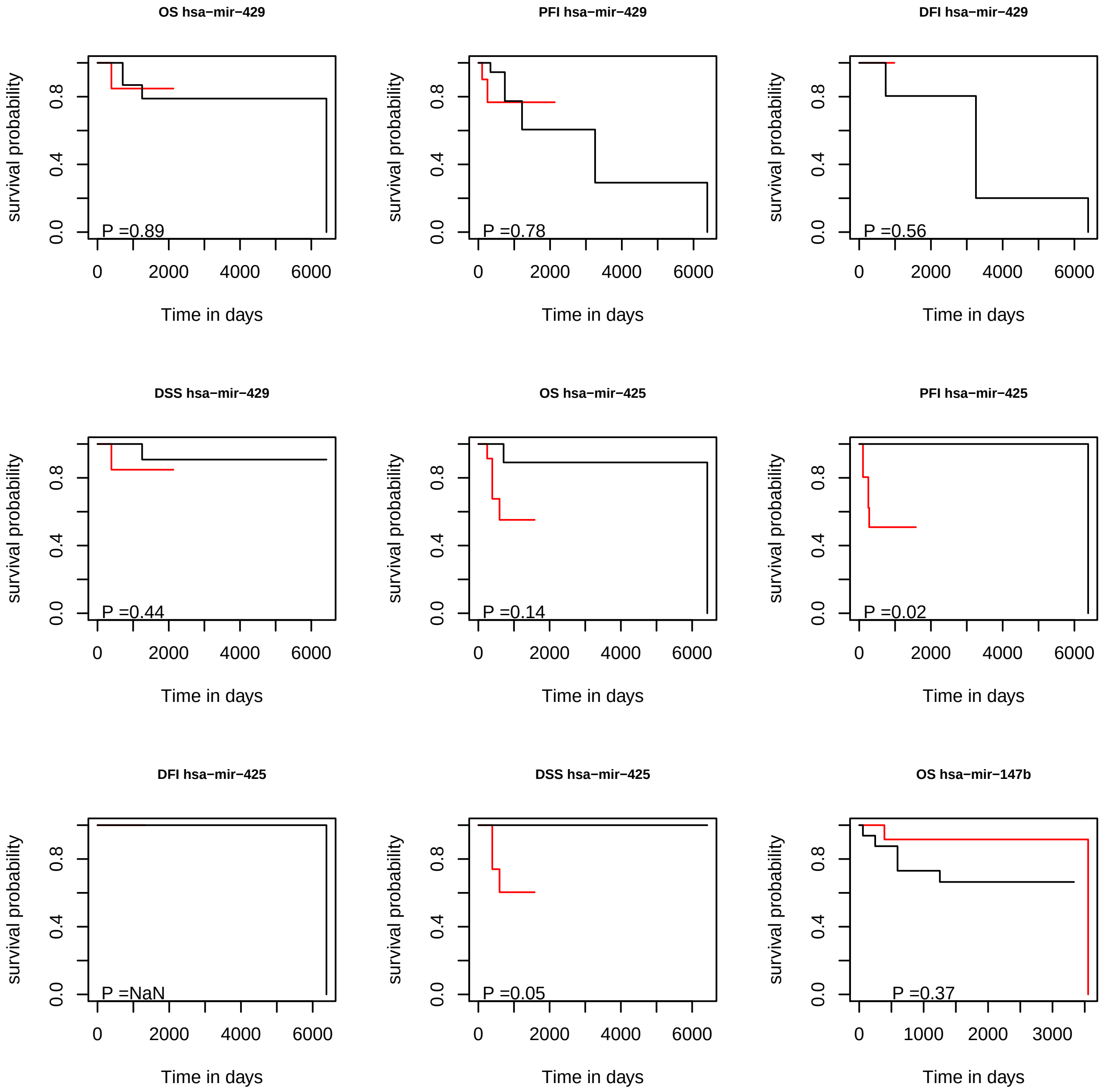
OS hsa−mir−429
P =0.89
PFI hsa−mir−429
P =0.78
DFI hsa−mir−429
P =0.56
DSS hsa−mir−429
P =0.44
OS hsa−mir−425
P =0.14
PFI hsa−mir−425
P =0.02
DFI hsa−mir−425
P =NaN
DSS hsa−mir−425
P =0.05
OS hsa−mir−147b
P =0.37
survival probability
Time in days
0.0
0.4
0.8
0
1000
2000
3000
4000
6000

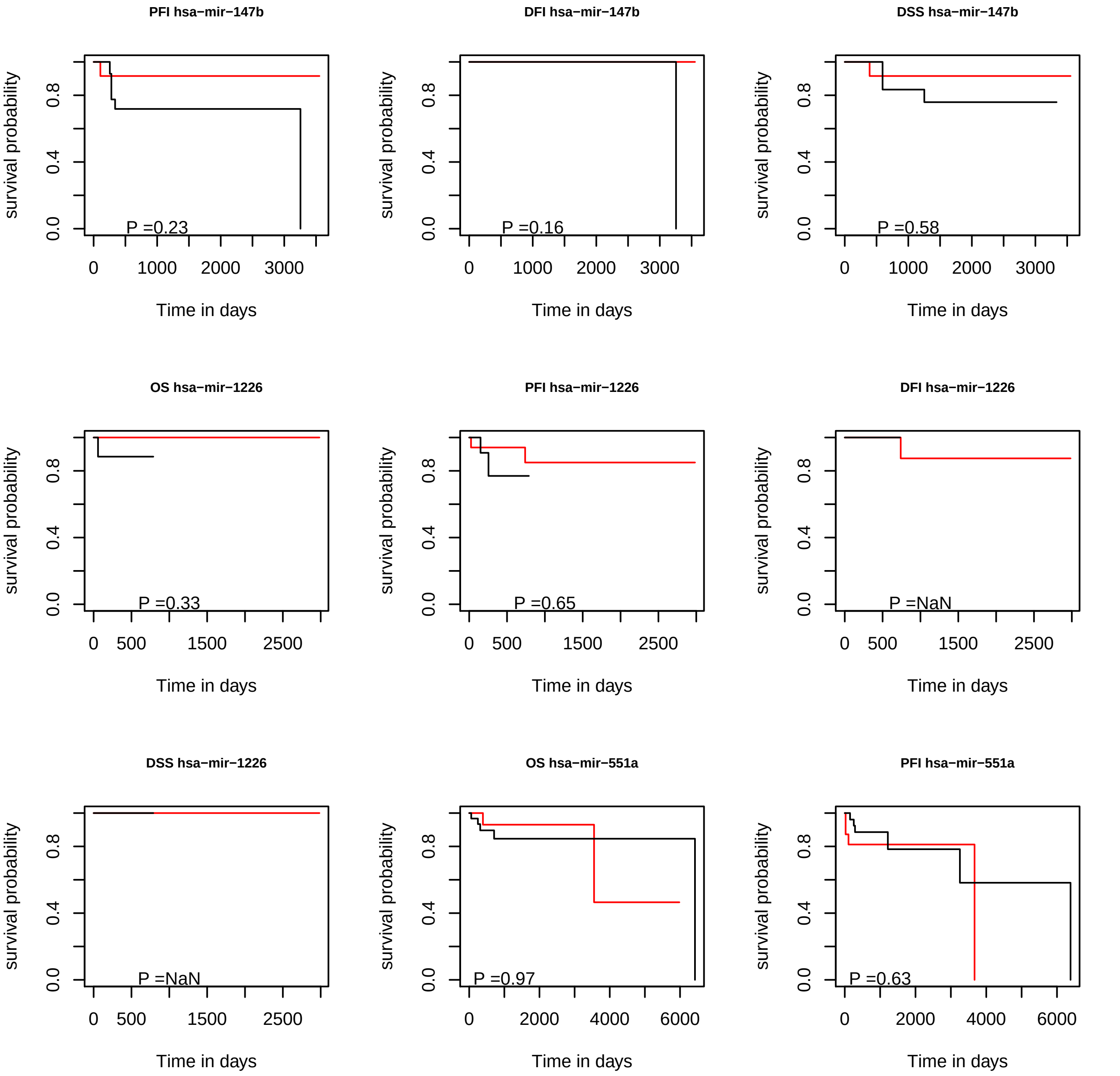
PFI hsa−mir−147b
P =0.23
DFI hsa−mir−147b
P =0.16
DSS hsa−mir−147b
P =0.58
OS hsa−mir−1226
P =0.33
PFI hsa−mir−1226
P =0.65
DFI hsa−mir−1226
P =NaN
DSS hsa−mir−1226
P =NaN
OS hsa−mir−551a
P =0.97
PFI hsa−mir−551a
P =0.63
survival probability
Time in days
0.0
0.4
0.8
0
500
1000
1500
2000
2500
3000
4000
6000

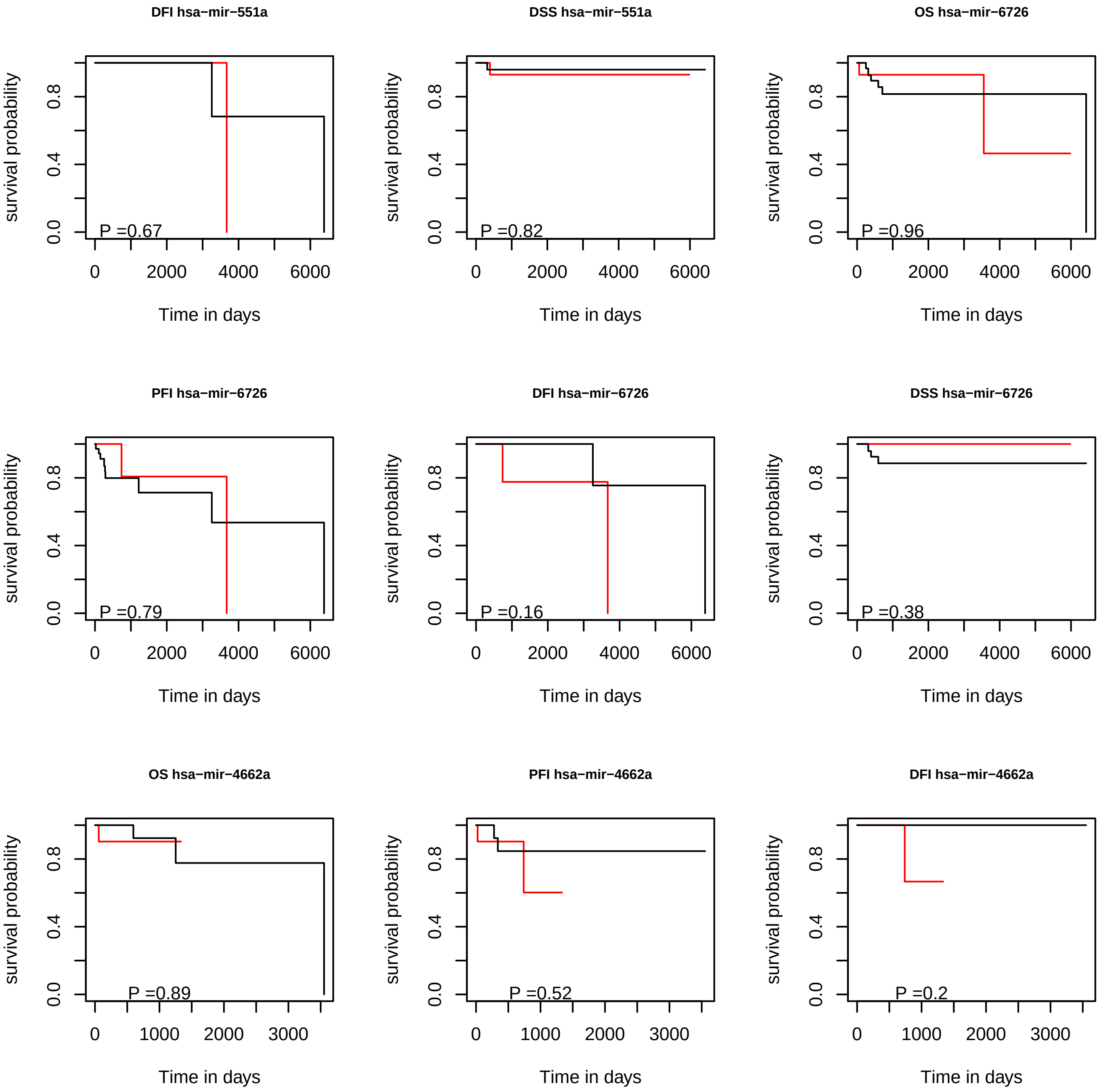
DFI hsa−mir−551a
DSS hsa−mir−551a
OS hsa−mir−6726
PFI hsa−mir−6726
DFI hsa−mir−6726
DSS hsa−mir−6726
OS hsa−mir−4662a
PFI hsa−mir−4662a
DFI hsa−mir−4662a
survival probability
Time in days
P =0.67
P =0.82
P =0.96
P =0.79
P =0.16
P =0.38
P =0.89
P =0.52
P =0.2
0.0
0.4
0.8
0
1000
2000
3000
4000
6000

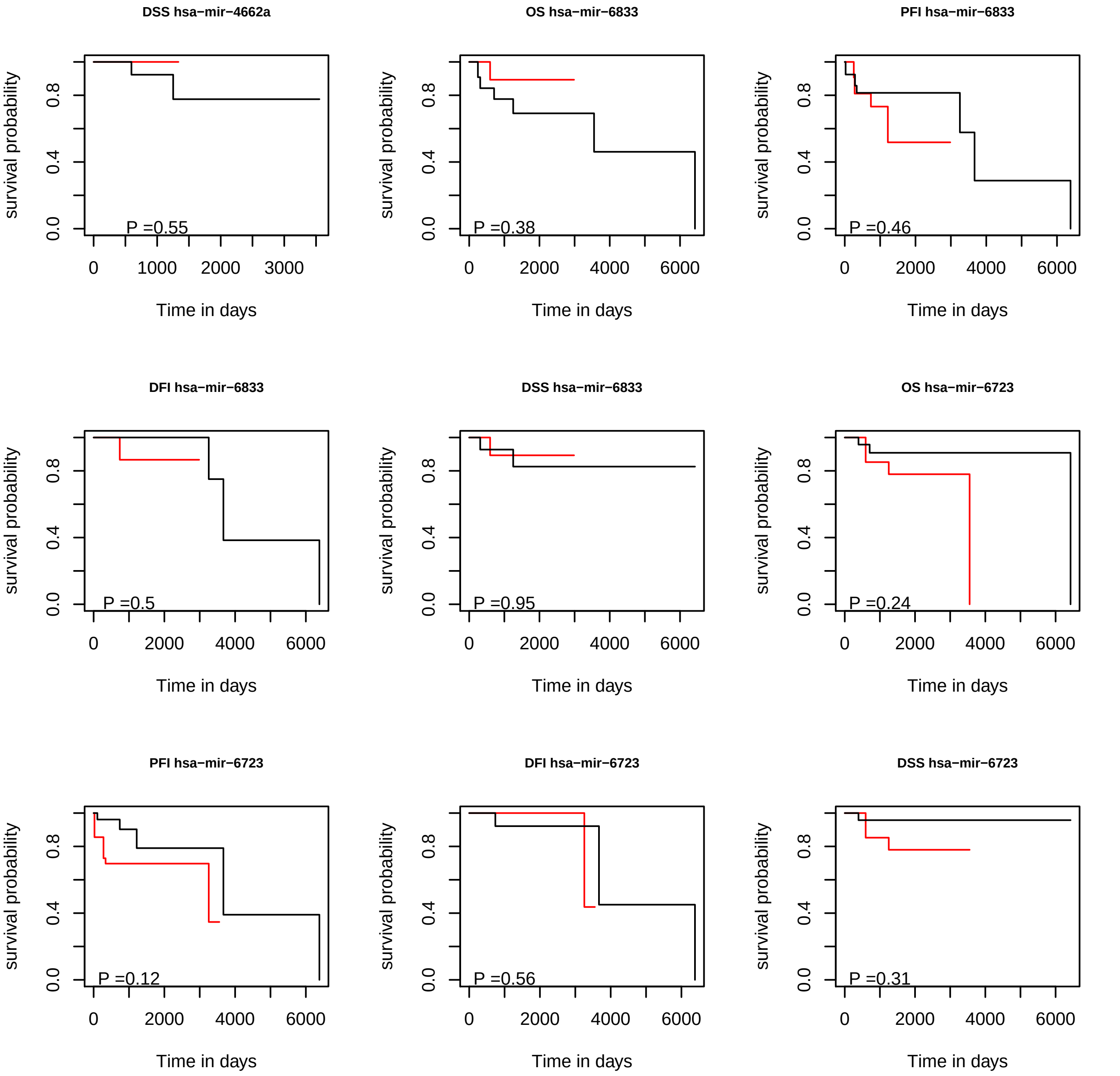
DSS hsa−mir−4662a
survival probability
0.0
0.4
0.8
P =0.55
0
1000
2000
3000
Time in days
OS hsa−mir−6833
survival probability
0.0
0.4
0.8
P =0.38
0
2000
4000
6000
Time in days
PFI hsa−mir−6833
survival probability
0.0
0.4
0.8
P =0.46
0
2000
4000
6000
Time in days
DFI hsa−mir−6833
survival probability
0.0
0.4
0.8
P =0.5
0
2000
4000
6000
Time in days
DSS hsa−mir−6833
survival probability
0.0
0.4
0.8
P =0.95
0
2000
4000
6000
Time in days
OS hsa−mir−6723
survival probability
0.0
0.4
0.8
P =0.24
0
2000
4000
6000
Time in days
PFI hsa−mir−6723
survival probability
0.0
0.4
0.8
P =0.12
0
2000
4000
6000
Time in days
DFI hsa−mir−6723
survival probability
0.0
0.4
0.8
P =0.56
0
2000
4000
6000
Time in days
DSS hsa−mir−6723
survival probability
0.0
0.4
0.8
P =0.31
0
2000
4000
6000
Time in days

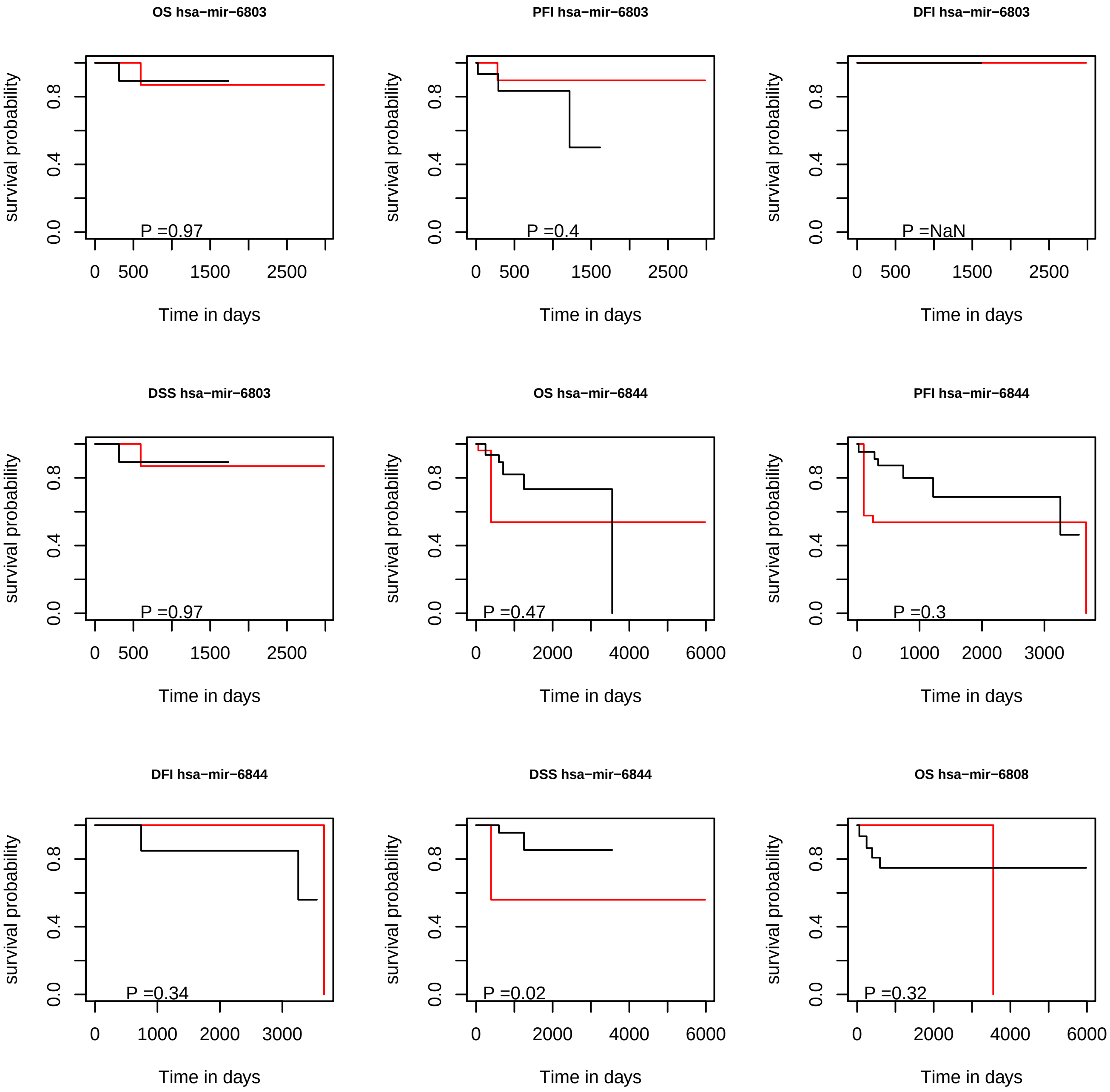
OS hsa−mir−6803
survival probability
0.0
0.4
0.8
0
500
1500
2500
Time in days
P =0.97
PFI hsa−mir−6803
survival probability
0.0
0.4
0.8
0
500
1500
2500
Time in days
P =0.4
DFI hsa−mir−6803
survival probability
0.0
0.4
0.8
0
500
1500
2500
Time in days
P =NaN
DSS hsa−mir−6803
survival probability
0.0
0.4
0.8
0
500
1500
2500
Time in days
P =0.97
OS hsa−mir−6844
survival probability
0.0
0.4
0.8
0
2000
4000
6000
Time in days
P =0.47
PFI hsa−mir−6844
survival probability
0.0
0.4
0.8
0
1000
2000
3000
Time in days
P =0.3
DFI hsa−mir−6844
survival probability
0.0
0.4
0.8
0
1000
2000
3000
Time in days
P =0.34
DSS hsa−mir−6844
survival probability
0.0
0.4
0.8
0
2000
4000
6000
Time in days
P =0.02
OS hsa−mir−6808
survival probability
0.0
0.4
0.8
0
2000
4000
6000
Time in days
P =0.32

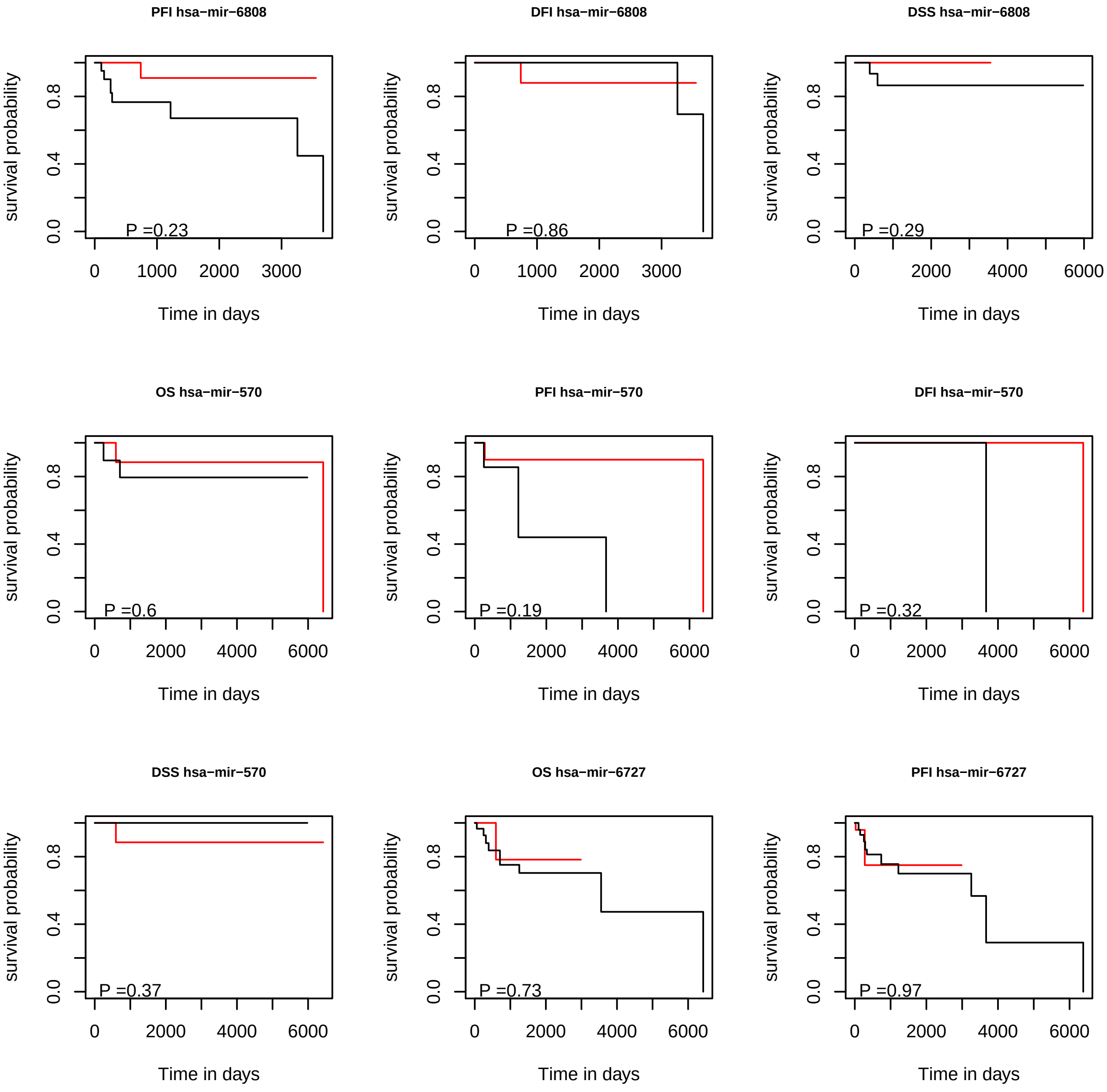
PFI hsa−mir−6808
P =0.23
DFI hsa−mir−6808
P =0.86
DSS hsa−mir−6808
P =0.29
OS hsa−mir−570
P =0.6
PFI hsa−mir−570
P =0.19
DFI hsa−mir−570
P =0.32
DSS hsa−mir−570
P =0.37
OS hsa−mir−6727
P =0.73
PFI hsa−mir−6727
P =0.97
survival probability
Time in days
0.0
0.4
0.8
0
1000
2000
3000
4000
6000

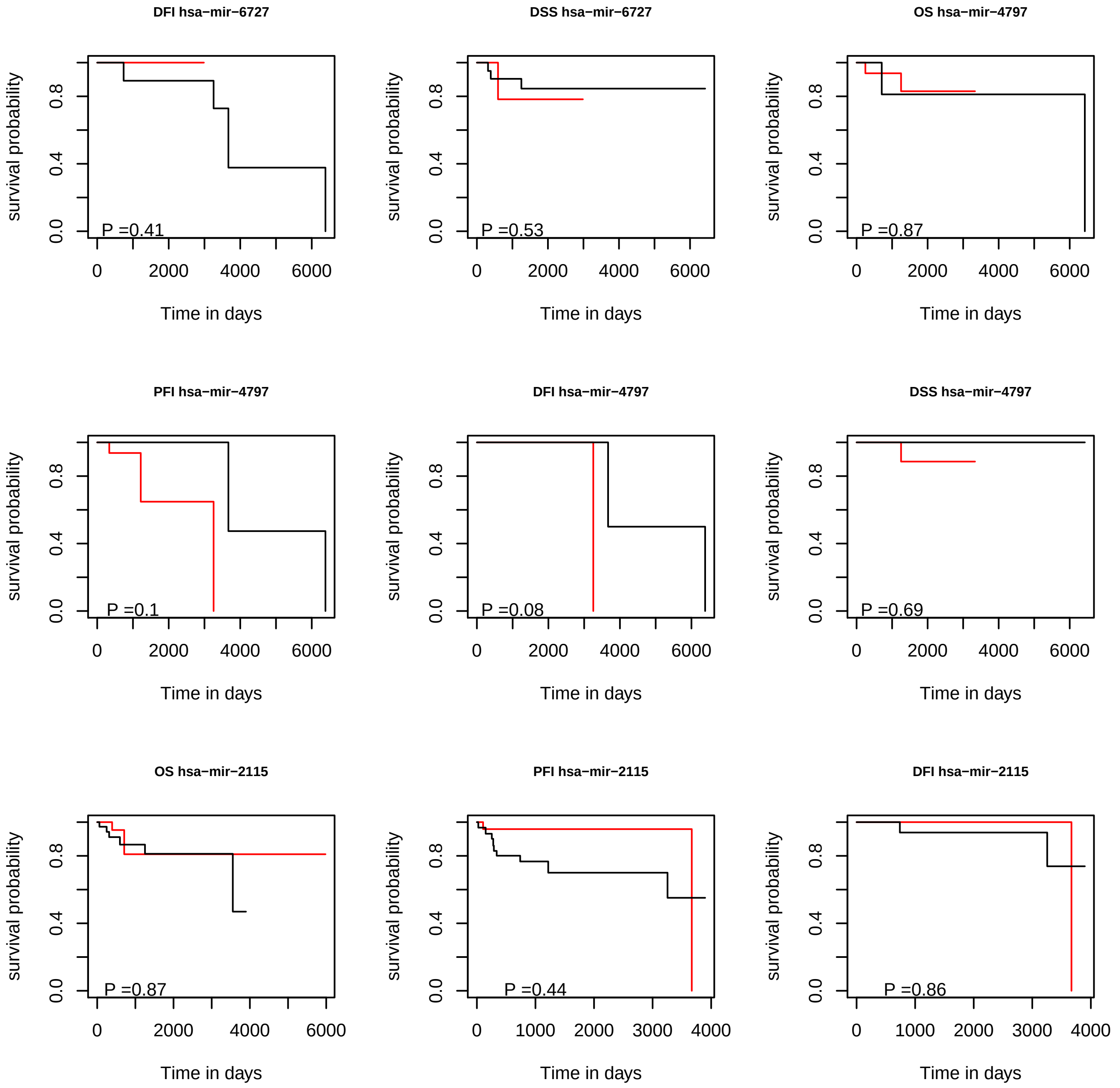
DFI hsa−mir−6727
survival probability
Time in days
P =0.41
DSS hsa−mir−6727
survival probability
Time in days
P =0.53
OS hsa−mir−4797
survival probability
Time in days
P =0.87
PFI hsa−mir−4797
survival probability
Time in days
P =0.1
DFI hsa−mir−4797
survival probability
Time in days
P =0.08
DSS hsa−mir−4797
survival probability
Time in days
P =0.69
OS hsa−mir−2115
survival probability
Time in days
P =0.87
PFI hsa−mir−2115
survival probability
Time in days
P =0.44
DFI hsa−mir−2115
survival probability
Time in days
P =0.86

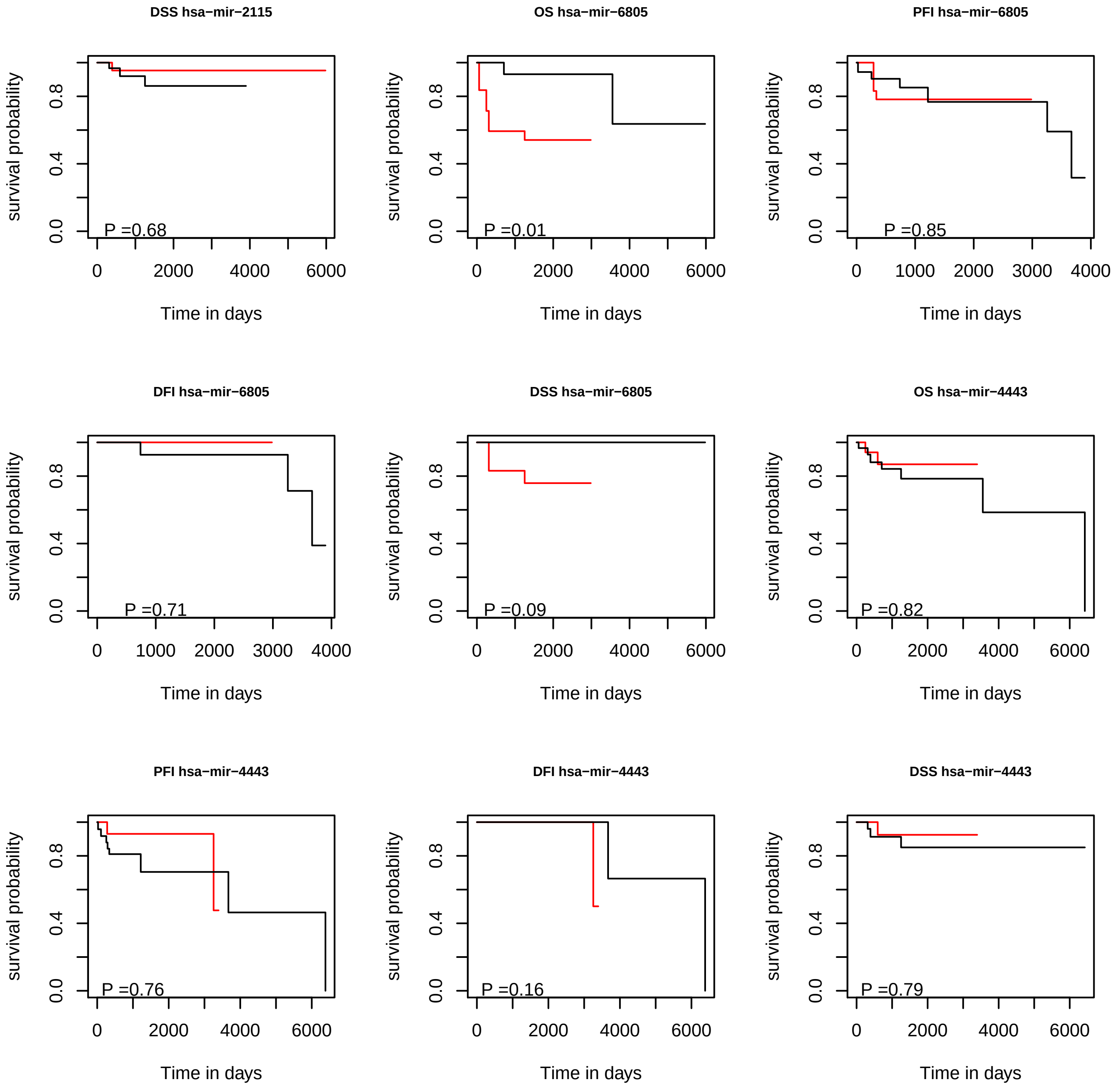
DSS hsa−mir−2115
survival probability
0.0
0.4
0.8
P =0.68
0
2000
4000
6000
Time in days
OS hsa−mir−6805
survival probability
0.0
0.4
0.8
P =0.01
0
2000
4000
6000
Time in days
PFI hsa−mir−6805
survival probability
0.0
0.4
0.8
P =0.85
0
1000
2000
3000
4000
Time in days
DFI hsa−mir−6805
survival probability
0.0
0.4
0.8
P =0.71
0
1000
2000
3000
4000
Time in days
DSS hsa−mir−6805
survival probability
0.0
0.4
0.8
P =0.09
0
2000
4000
6000
Time in days
OS hsa−mir−4443
survival probability
0.0
0.4
0.8
P =0.82
0
2000
4000
6000
Time in days
PFI hsa−mir−4443
survival probability
0.0
0.4
0.8
P =0.76
0
2000
4000
6000
Time in days
DFI hsa−mir−4443
survival probability
0.0
0.4
0.8
P =0.16
0
2000
4000
6000
Time in days
DSS hsa−mir−4443
survival probability
0.0
0.4
0.8
P =0.79
0
2000
4000
6000
Time in days

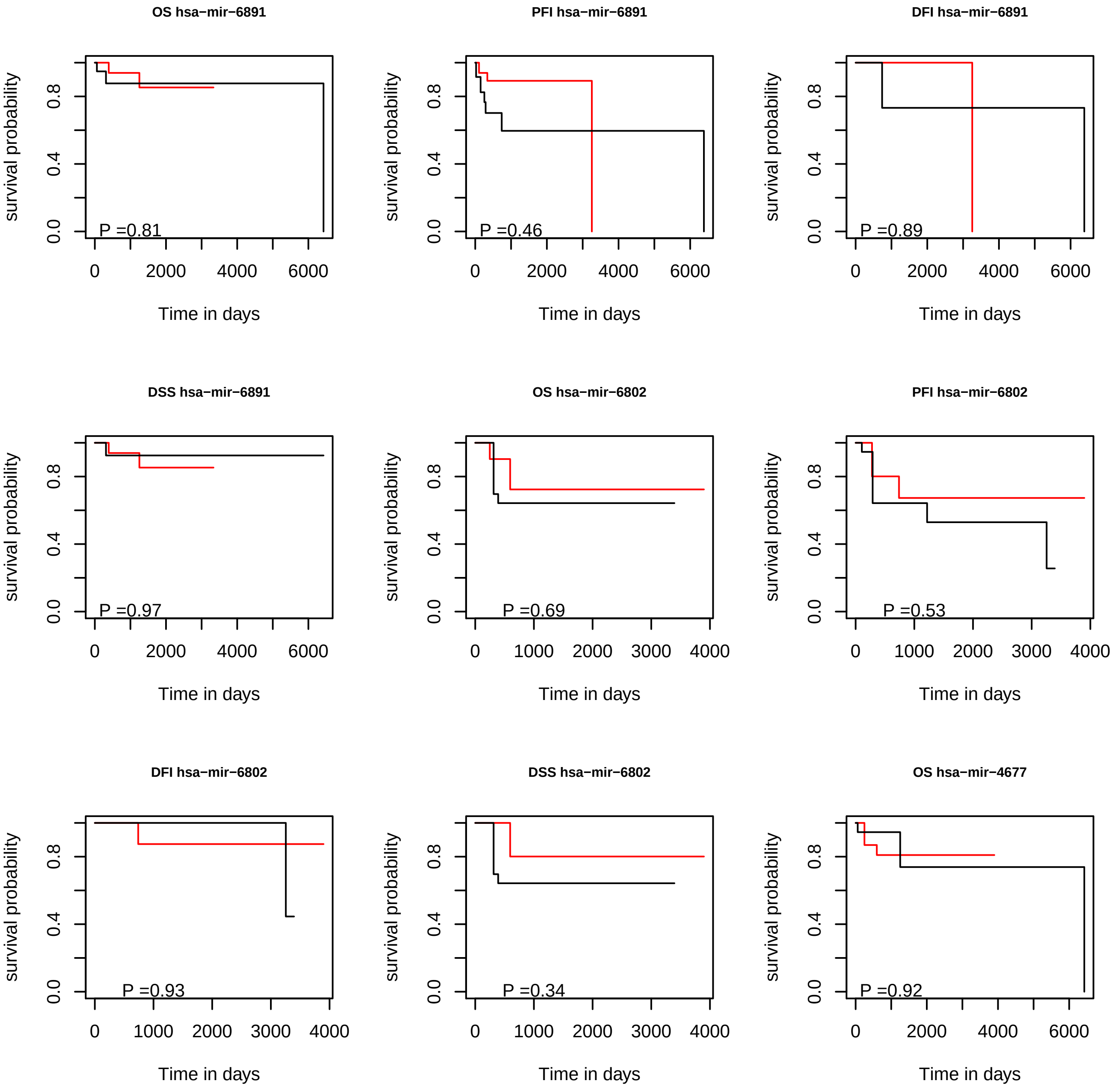
OS hsa−mir−6891
P =0.81
PFI hsa−mir−6891
P =0.46
DFI hsa−mir−6891
P =0.89
DSS hsa−mir−6891
P =0.97
OS hsa−mir−6802
P =0.69
PFI hsa−mir−6802
P =0.53
DFI hsa−mir−6802
P =0.93
DSS hsa−mir−6802
P =0.34
OS hsa−mir−4677
P =0.92
survival probability
Time in days

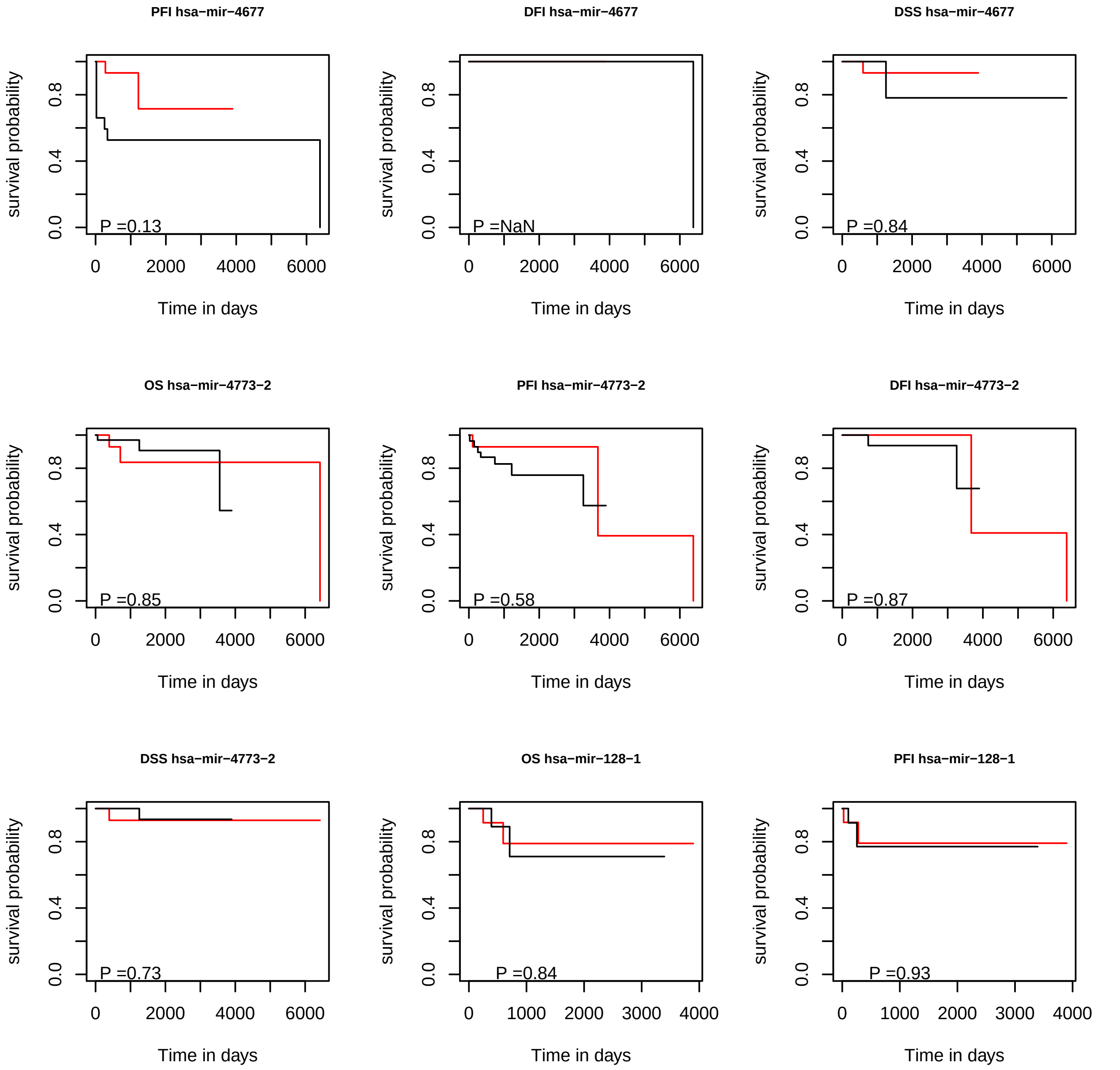
PFI hsa−mir−4677
P =0.13
DFI hsa−mir−4677
P =NaN
DSS hsa−mir−4677
P =0.84
OS hsa−mir−4773−2
P =0.85
PFI hsa−mir−4773−2
P =0.58
DFI hsa−mir−4773−2
P =0.87
DSS hsa−mir−4773−2
P =0.73
OS hsa−mir−128−1
P =0.84
PFI hsa−mir−128−1
P =0.93
survival probability
Time in days
0.0
0.4
0.8
0
1000
2000
3000
4000
6000

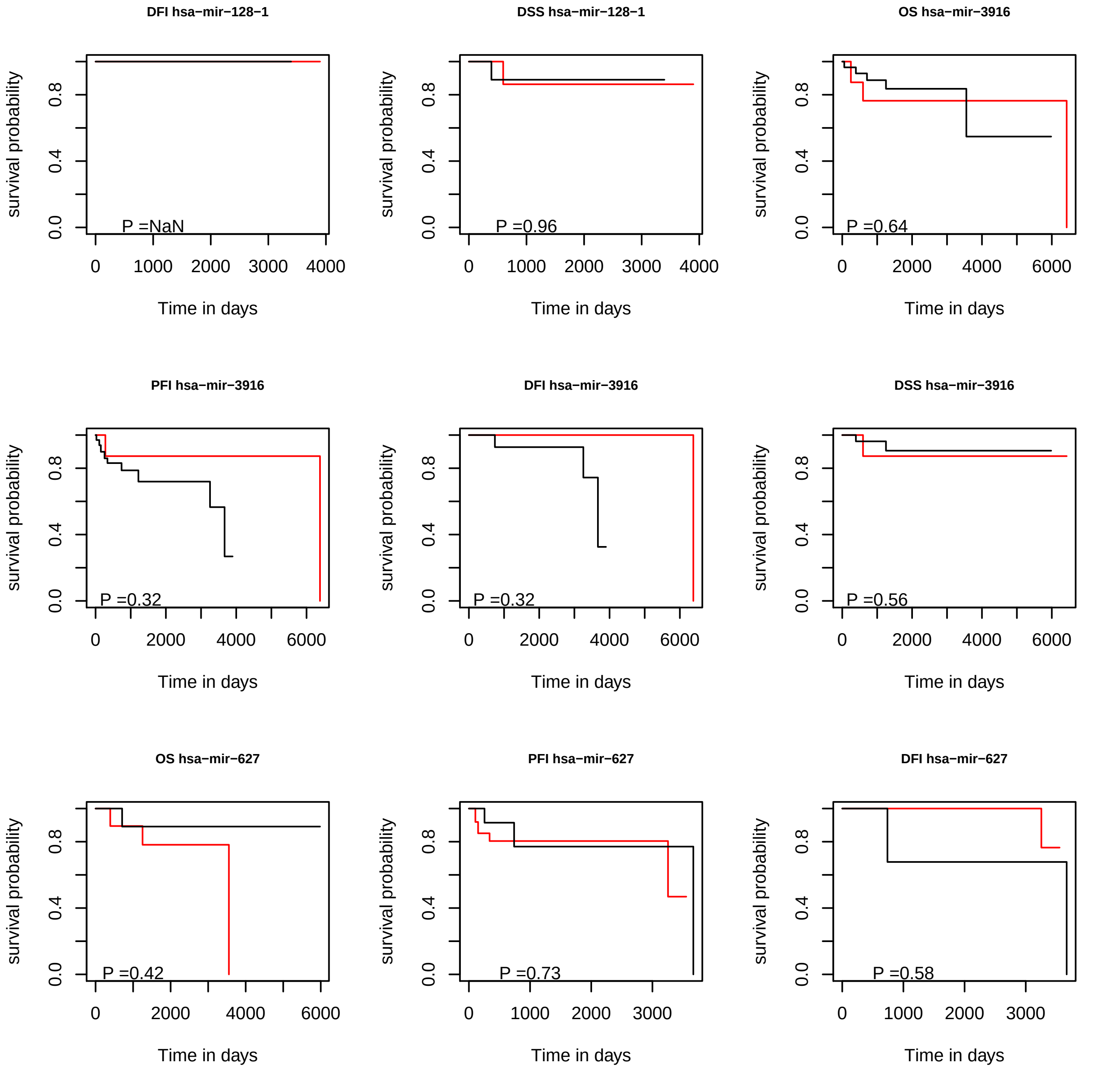
DFI hsa−mir−128−1
survival probability
0.0
0.4
0.8
P =NaN
0
1000
2000
3000
4000
Time in days
DSS hsa−mir−128−1
survival probability
0.0
0.4
0.8
P =0.96
0
1000
2000
3000
4000
Time in days
OS hsa−mir−3916
survival probability
0.0
0.4
0.8
P =0.64
0
2000
4000
6000
Time in days
PFI hsa−mir−3916
survival probability
0.0
0.4
0.8
P =0.32
0
2000
4000
6000
Time in days
DFI hsa−mir−3916
survival probability
0.0
0.4
0.8
P =0.32
0
2000
4000
6000
Time in days
DSS hsa−mir−3916
survival probability
0.0
0.4
0.8
P =0.56
0
2000
4000
6000
Time in days
OS hsa−mir−627
survival probability
0.0
0.4
0.8
P =0.42
0
2000
4000
6000
Time in days
PFI hsa−mir−627
survival probability
0.0
0.4
0.8
P =0.73
0
1000
2000
3000
Time in days
DFI hsa−mir−627
survival probability
0.0
0.4
0.8
P =0.58
0
1000
2000
3000
Time in days

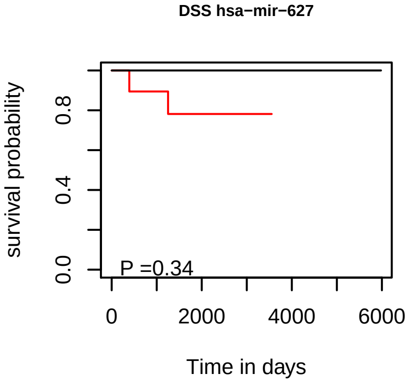
DSS hsa−mir−627
survival probability
0.0
0.4
0.8
P =0.34
0
2000
4000
6000
Time in days
