# Supplementary material for: Computing microRNA-gene interaction networks in pan-cancer using miRDriver
Source: Sci Rep. 2022 Mar 8;12:3717. doi: 10.1038/s41598-022-07628-z (PMC8904490; doi:10.1038/s41598-022-07628-z)
Supplement: Supplementary file 15 — Supplementary Information 15. [file 41598_2022_7628_MOESM15_ESM.pdf]

Computing microRNA-gene interaction networks in pan-cancer using miRDriver

Banabithi Bose, Matthew Moravec, and Serdar Bozdag

Supplemental Figure S6

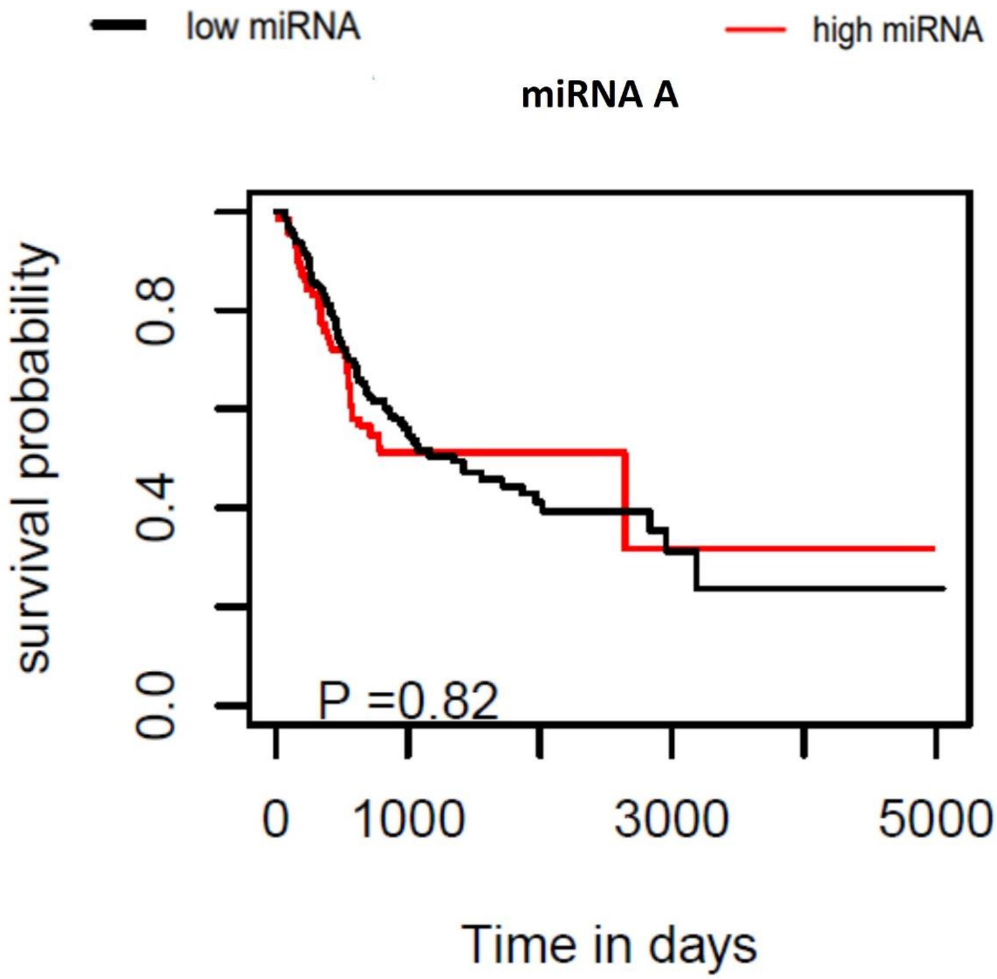

The *Adjusted Kaplan-Meier* survival plots for the computed miRNAs in high and low miRNA expression patient groups.

Supplemental Figure S6

Cancer Type: ESCA

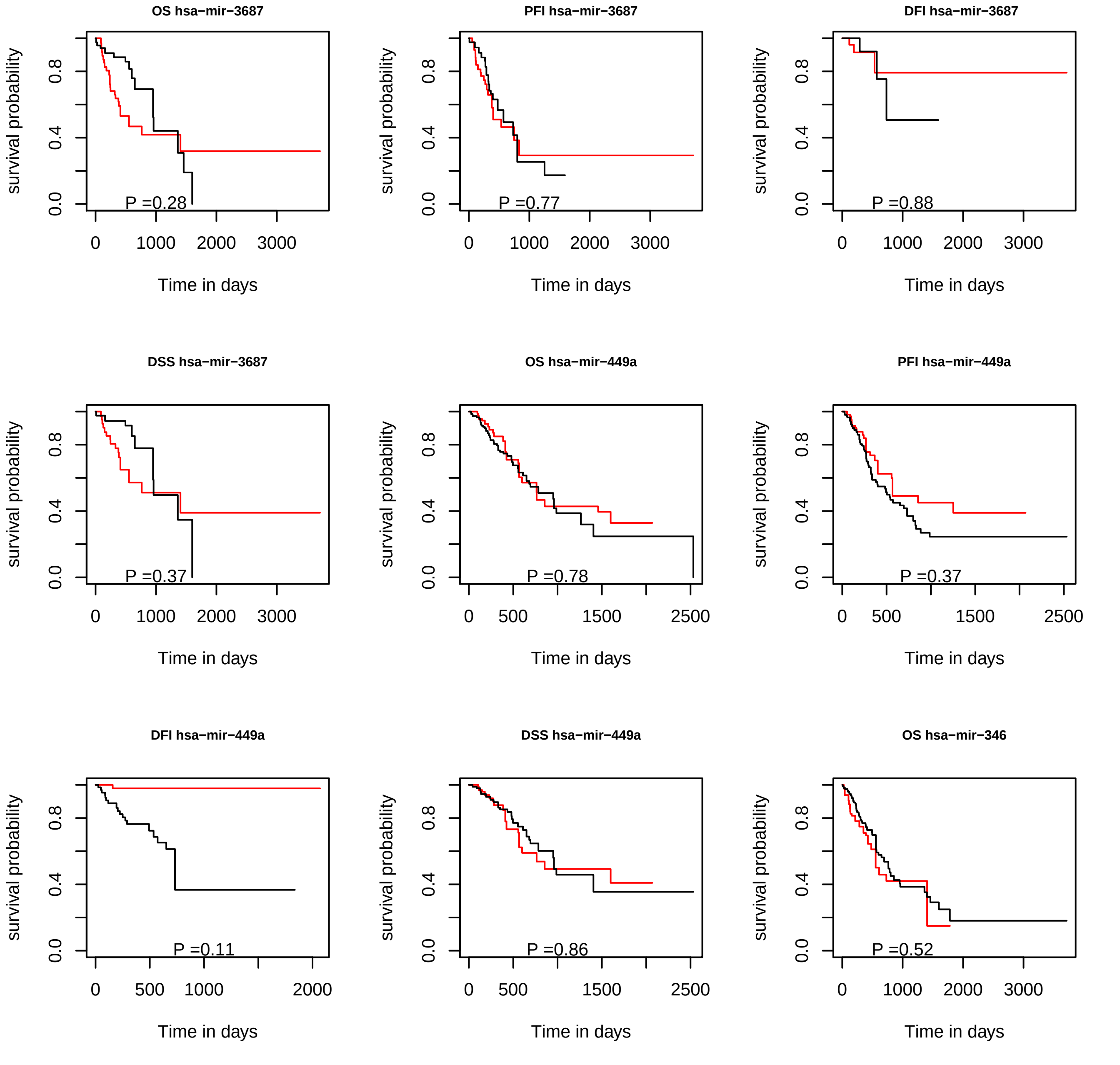

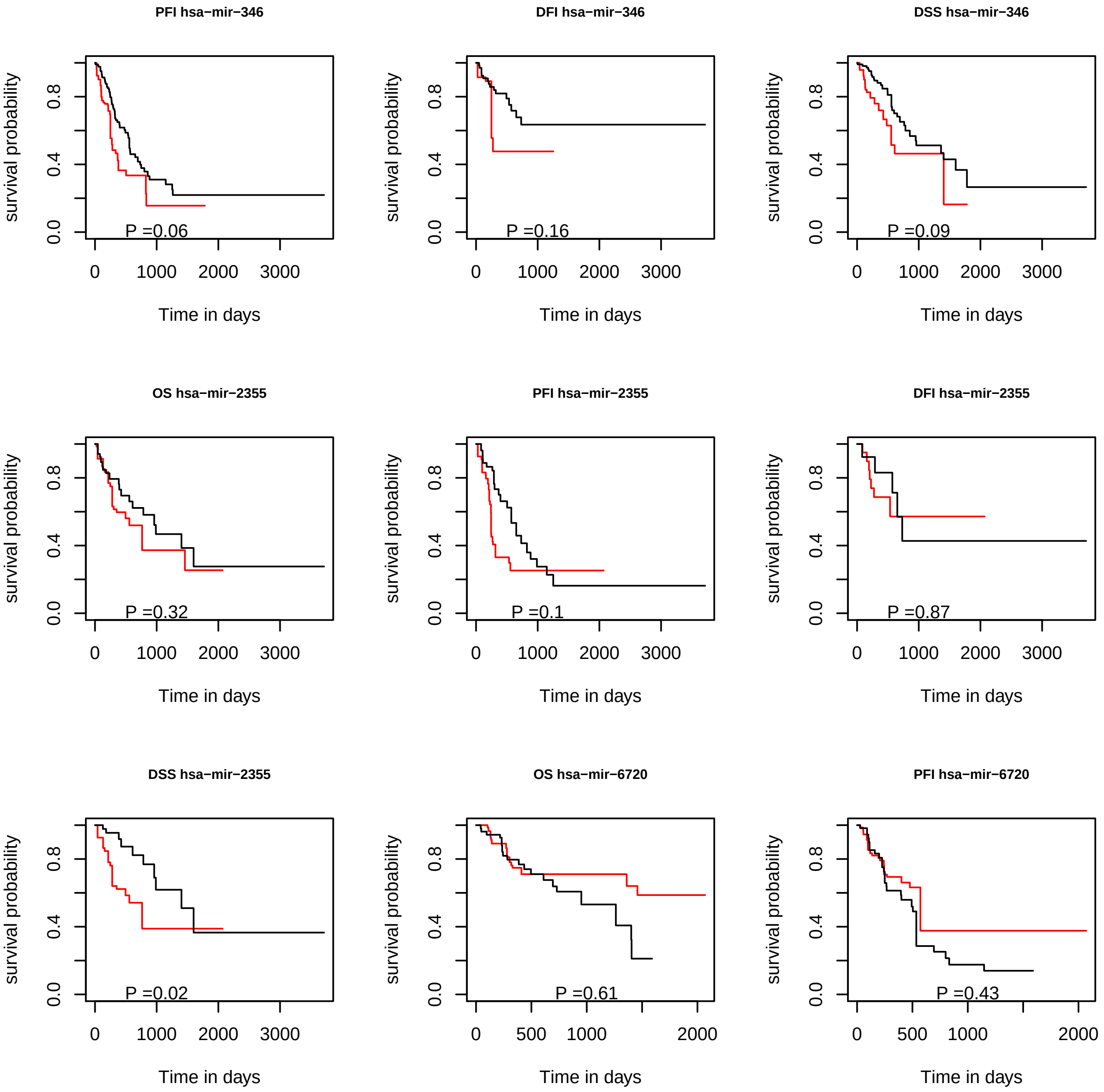
PFI hsa−mir−346
P =0.06
DFI hsa−mir−346
P =0.16
DSS hsa−mir−346
P =0.09
OS hsa−mir−2355
P =0.32
PFI hsa−mir−2355
P =0.1
DFI hsa−mir−2355
P =0.87
DSS hsa−mir−2355
P =0.02
OS hsa−mir−6720
P =0.61
PFI hsa−mir−6720
P =0.43
survival probability
Time in days

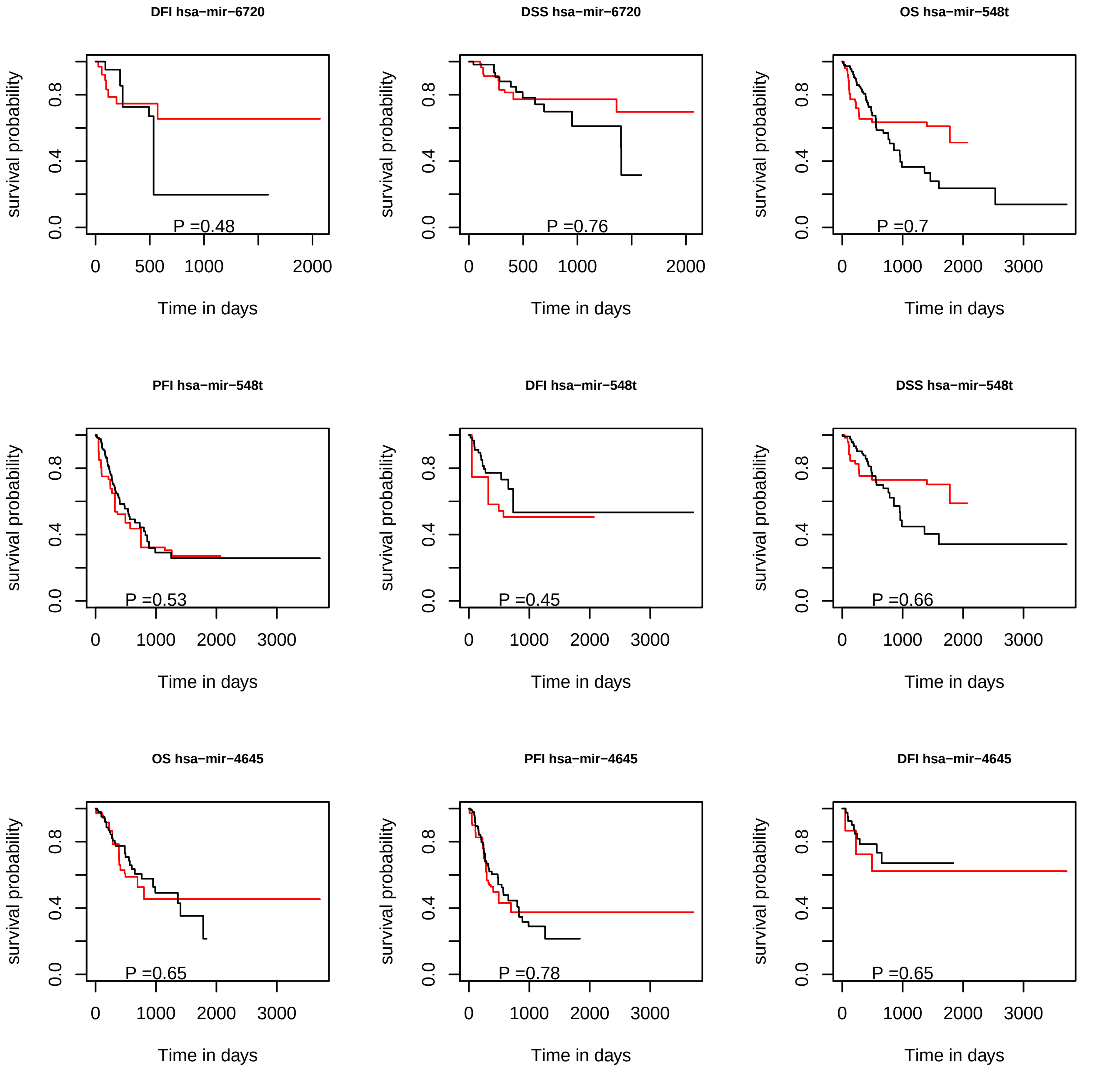
DFI hsa−mir−6720
P =0.48
DSS hsa−mir−6720
P =0.76
OS hsa−mir−548t
P =0.7
PFI hsa−mir−548t
P =0.53
DFI hsa−mir−548t
P =0.45
DSS hsa−mir−548t
P =0.66
OS hsa−mir−4645
P =0.65
PFI hsa−mir−4645
P =0.78
DFI hsa−mir−4645
P =0.65
survival probability
Time in days

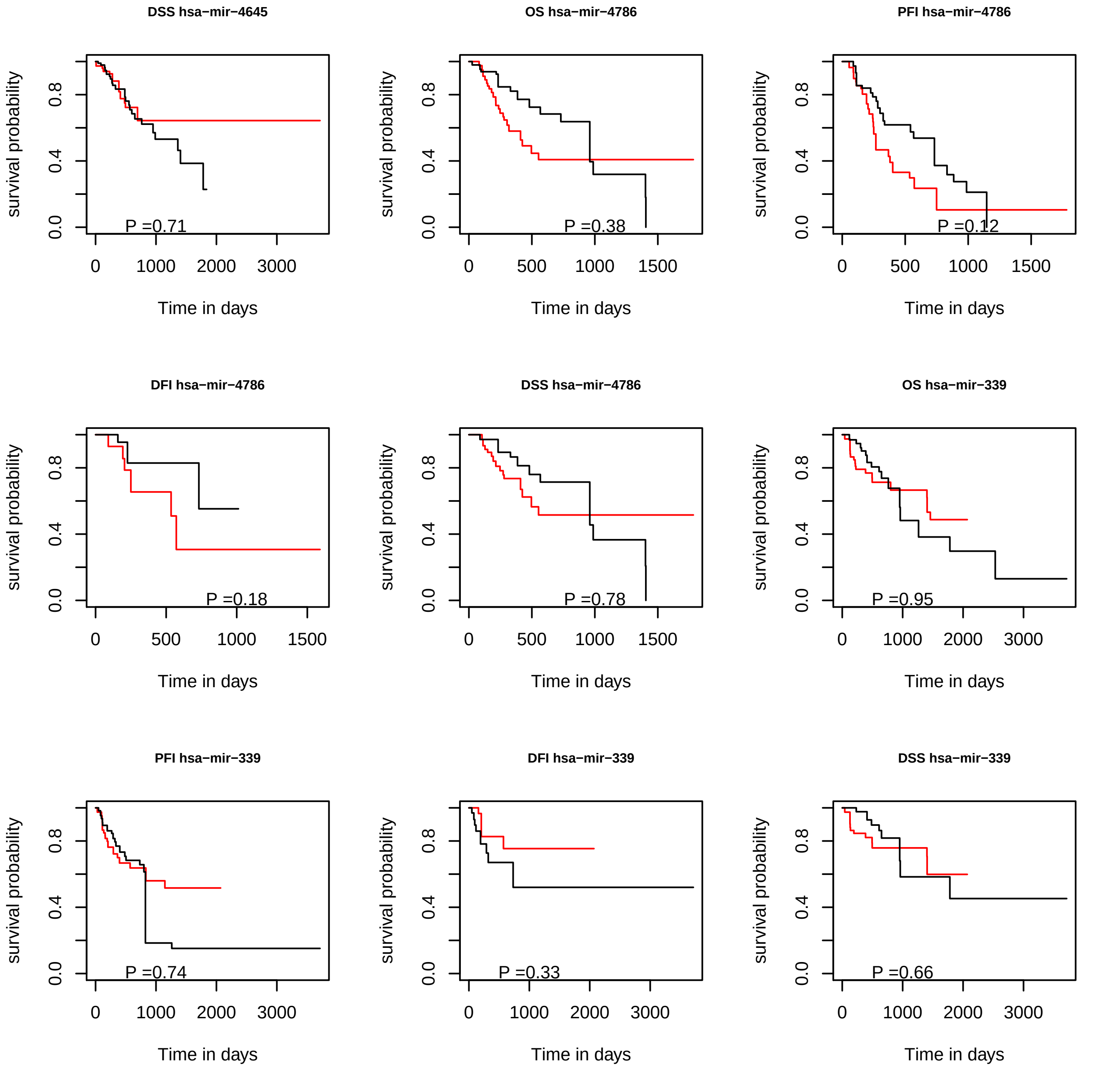
DSS hsa−mir−4645
P =0.71
OS hsa−mir−4786
P =0.38
PFI hsa−mir−4786
P =0.12
DFI hsa−mir−4786
P =0.18
DSS hsa−mir−4786
P =0.78
OS hsa−mir−339
P =0.95
PFI hsa−mir−339
P =0.74
DFI hsa−mir−339
P =0.33
DSS hsa−mir−339
P =0.66
survival probability
Time in days

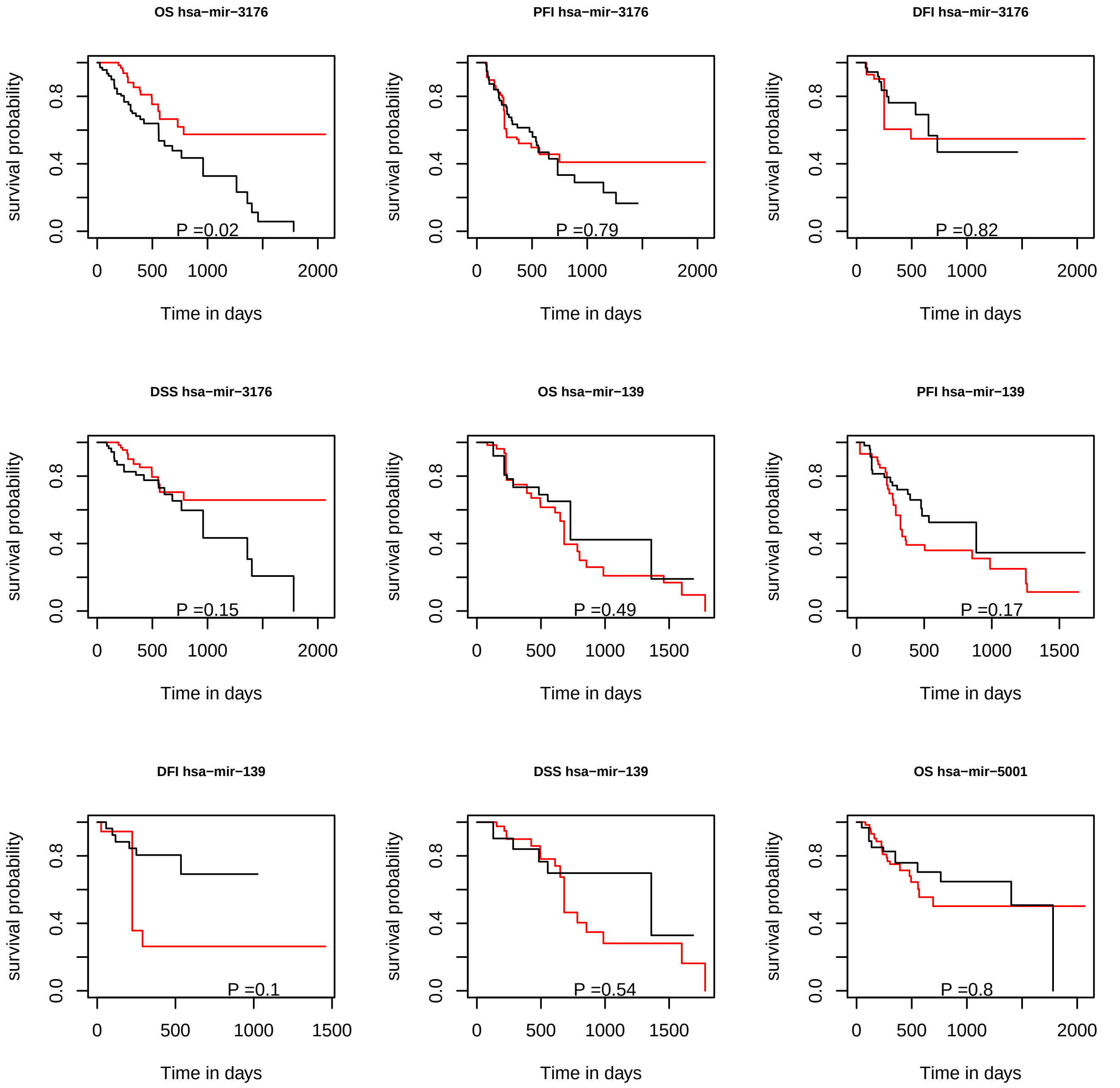
OS hsa−mir−3176
P =0.02
PFI hsa−mir−3176
P =0.79
DFI hsa−mir−3176
P =0.82
DSS hsa−mir−3176
P =0.15
OS hsa−mir−139
P =0.49
PFI hsa−mir−139
P =0.17
DFI hsa−mir−139
P =0.1
DSS hsa−mir−139
P =0.54
OS hsa−mir−5001
P =0.8
survival probability
Time in days
0.0
0.4
0.8
0
500
1000
1500
2000

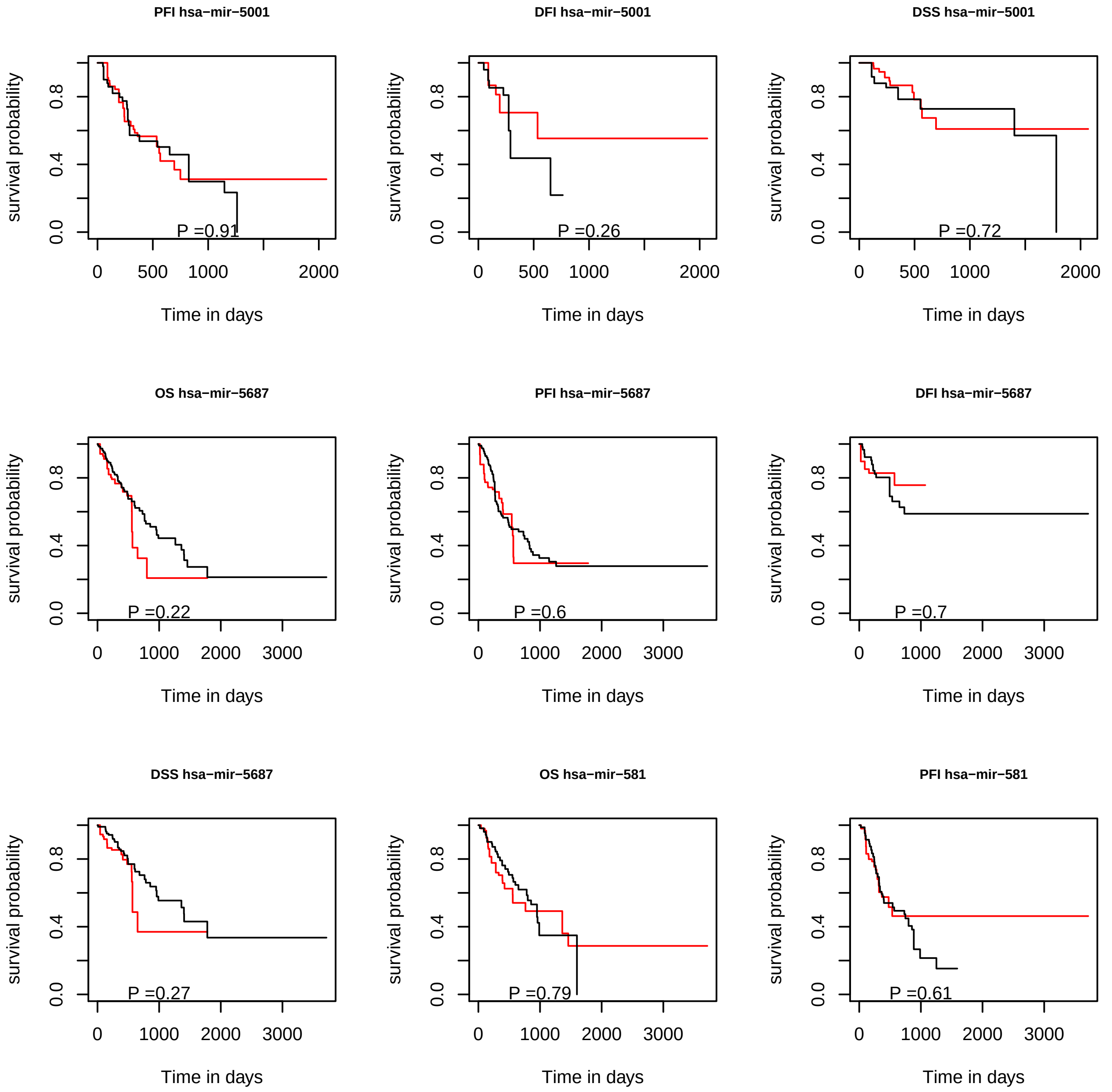
PFI hsa−mir−5001
P =0.91
DFI hsa−mir−5001
P =0.26
DSS hsa−mir−5001
P =0.72
OS hsa−mir−5687
P =0.22
PFI hsa−mir−5687
P =0.6
DFI hsa−mir−5687
P =0.7
DSS hsa−mir−5687
P =0.27
OS hsa−mir−581
P =0.79
PFI hsa−mir−581
P =0.61
survival probability
Time in days
0.0
0.4
0.8
0
500
1000
2000
3000

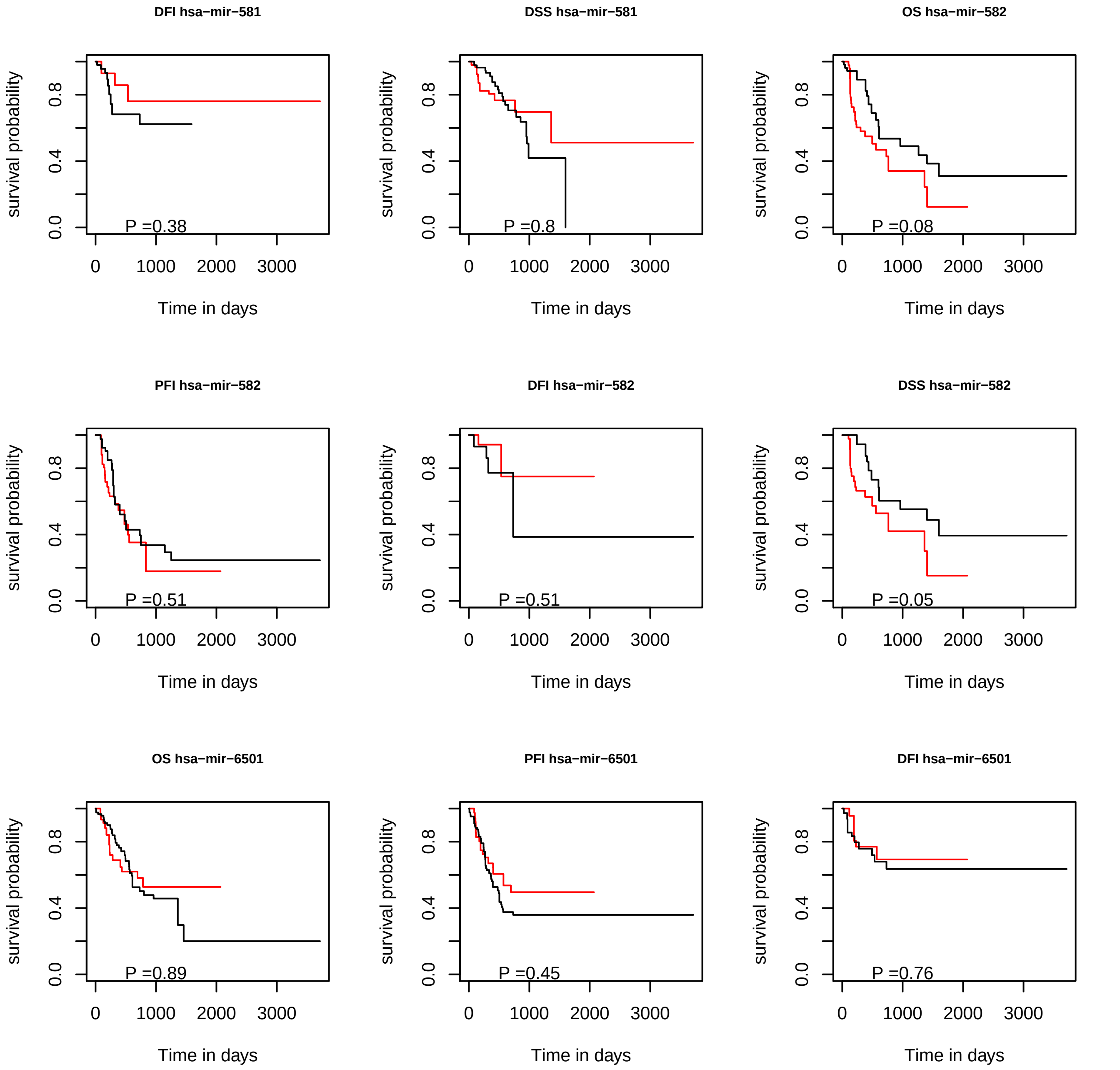
DFI hsa−mir−581
survival probability
Time in days
P =0.38
DSS hsa−mir−581
survival probability
Time in days
P =0.8
OS hsa−mir−582
survival probability
Time in days
P =0.08
PFI hsa−mir−582
survival probability
Time in days
P =0.51
DFI hsa−mir−582
survival probability
Time in days
P =0.51
DSS hsa−mir−582
survival probability
Time in days
P =0.05
OS hsa−mir−6501
survival probability
Time in days
P =0.89
PFI hsa−mir−6501
survival probability
Time in days
P =0.45
DFI hsa−mir−6501
survival probability
Time in days
P =0.76
0.0
0.4
0.8
0
1000
2000
3000

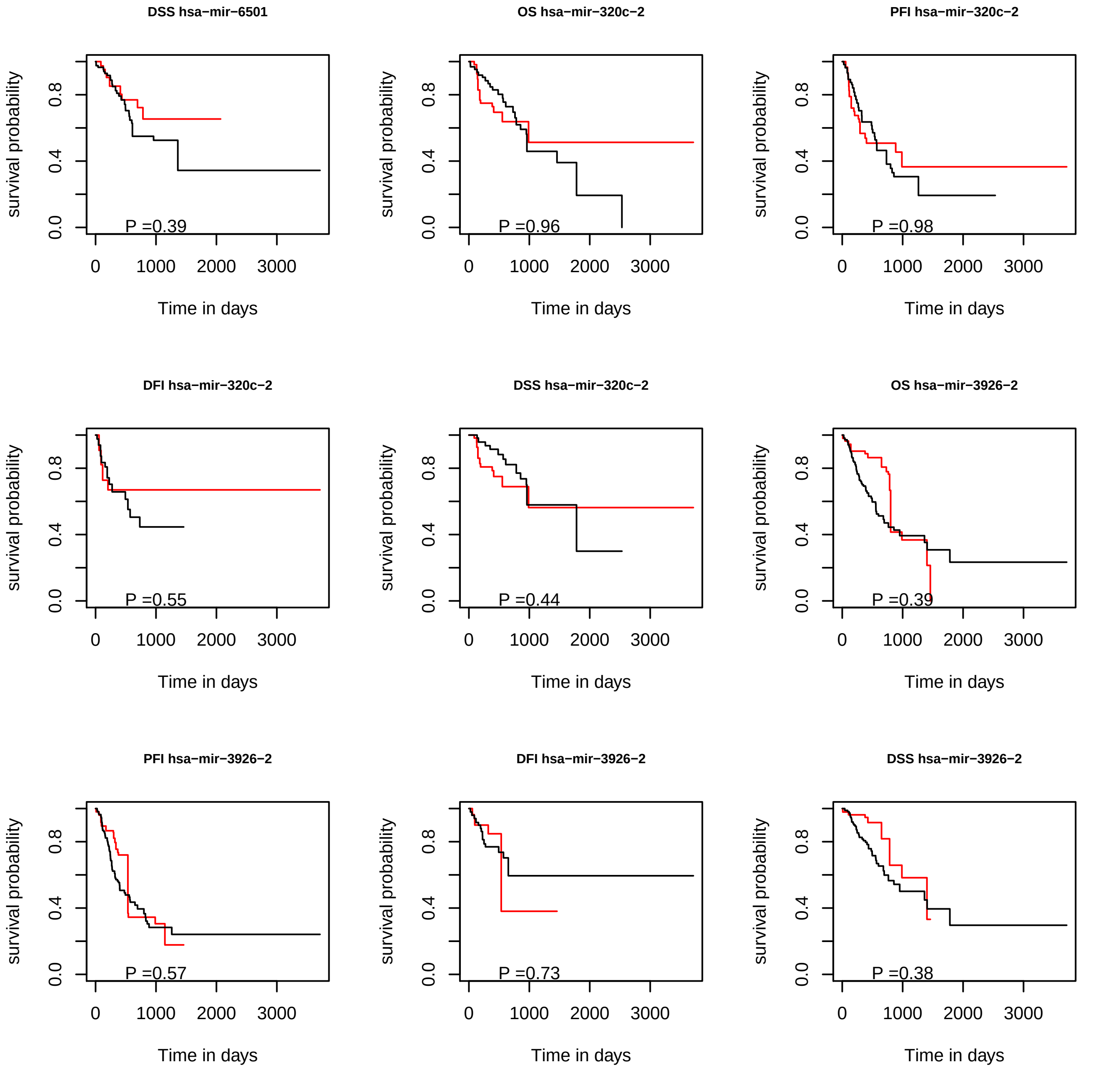

DSS hsa−mir−6501
P =0.39
OS hsa−mir−320c−2
P =0.96
PFI hsa−mir−320c−2
P =0.98
DFI hsa−mir−320c−2
P =0.55
DSS hsa−mir−320c−2
P =0.44
OS hsa−mir−3926−2
P =0.39
PFI hsa−mir−3926−2
P =0.57
DFI hsa−mir−3926−2
P =0.73
DSS hsa−mir−3926−2
P =0.38
survival probability
0.0
0.4
0.8
Time in days
0
1000
2000
3000

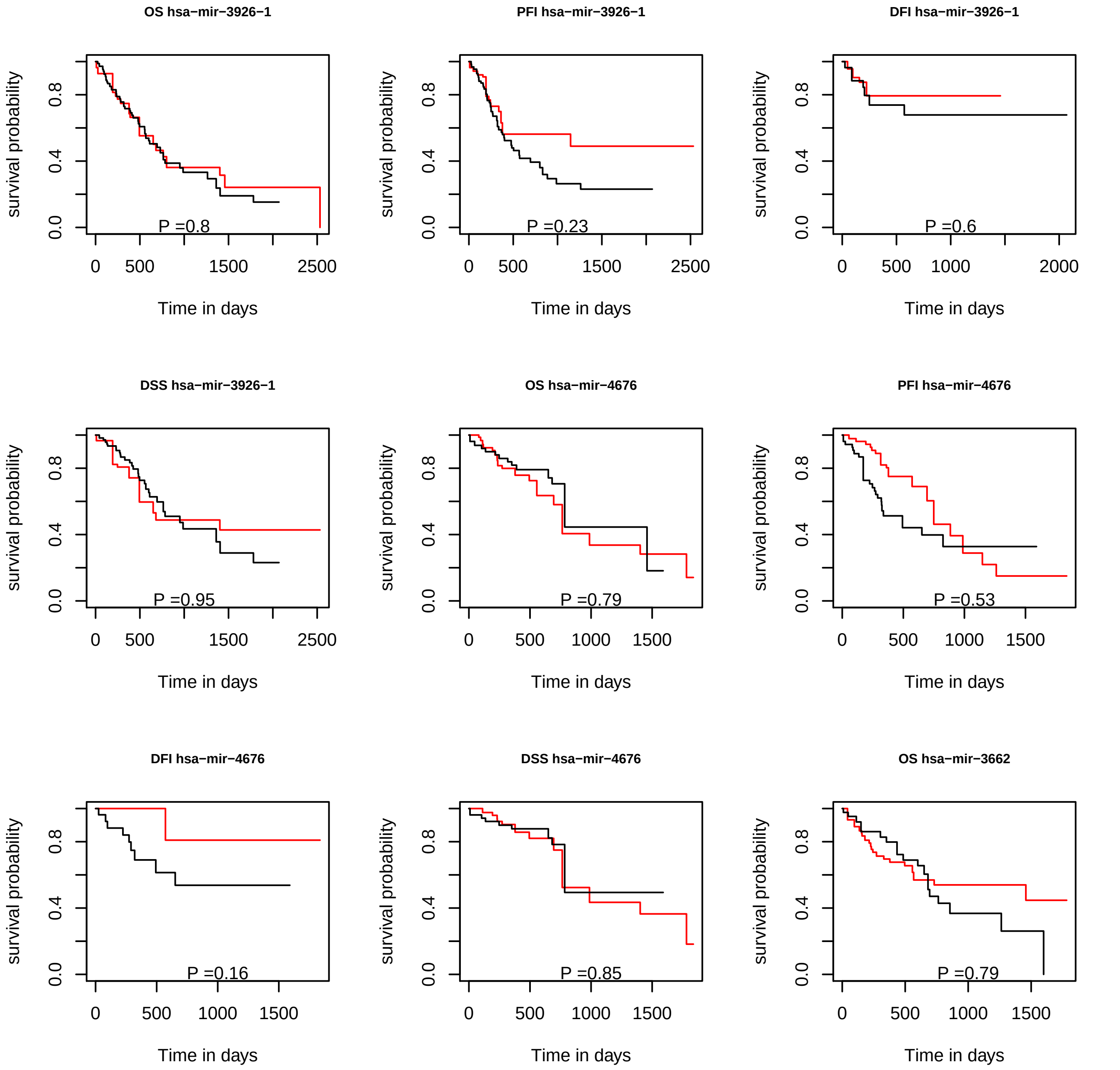
OS hsa−mir−3926−1
P =0.8
PFI hsa−mir−3926−1
P =0.23
DFI hsa−mir−3926−1
P =0.6
DSS hsa−mir−3926−1
P =0.95
OS hsa−mir−4676
P =0.79
PFI hsa−mir−4676
P =0.53
DFI hsa−mir−4676
P =0.16
DSS hsa−mir−4676
P =0.85
OS hsa−mir−3662
P =0.79
survival probability
Time in days
0.0
0.4
0.8
0
500
1000
1500
2000
2500

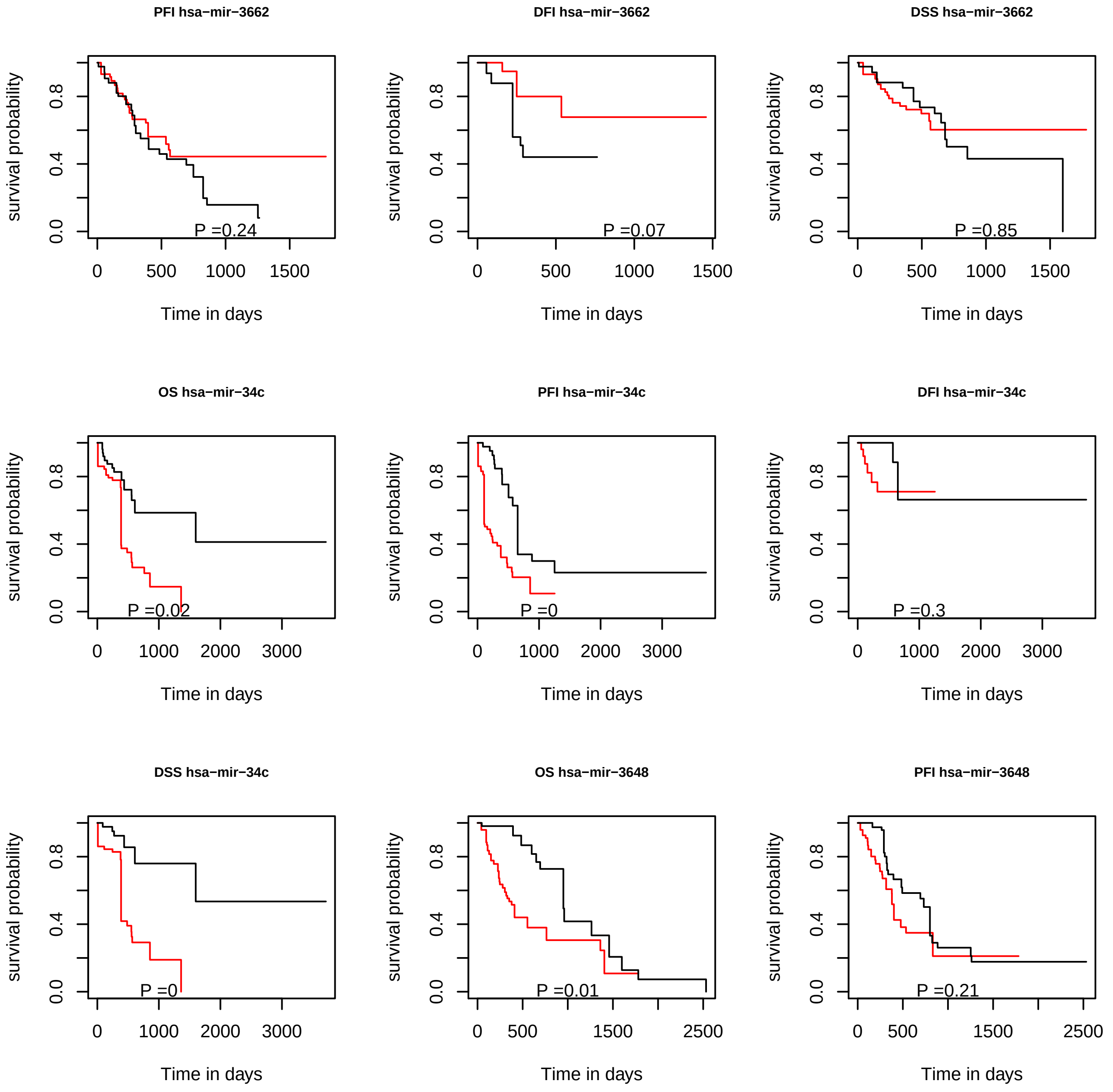
PFI hsa−mir−3662
survival probability
0.0
0.4
0.8
0
500
1000
1500
P =0.24
Time in days
DFI hsa−mir−3662
survival probability
0.0
0.4
0.8
0
500
1000
1500
P =0.07
Time in days
DSS hsa−mir−3662
survival probability
0.0
0.4
0.8
0
500
1000
1500
P =0.85
Time in days
OS hsa−mir−34c
survival probability
0.0
0.4
0.8
0
1000
2000
3000
P =0.02
Time in days
PFI hsa−mir−34c
survival probability
0.0
0.4
0.8
0
1000
2000
3000
P =0
Time in days
DFI hsa−mir−34c
survival probability
0.0
0.4
0.8
0
1000
2000
3000
P =0.3
Time in days
DSS hsa−mir−34c
survival probability
0.0
0.4
0.8
0
1000
2000
3000
P =0
Time in days
OS hsa−mir−3648
survival probability
0.0
0.4
0.8
0
500
1500
2500
P =0.01
Time in days
PFI hsa−mir−3648
survival probability
0.0
0.4
0.8
0
500
1500
2500
P =0.21
Time in days

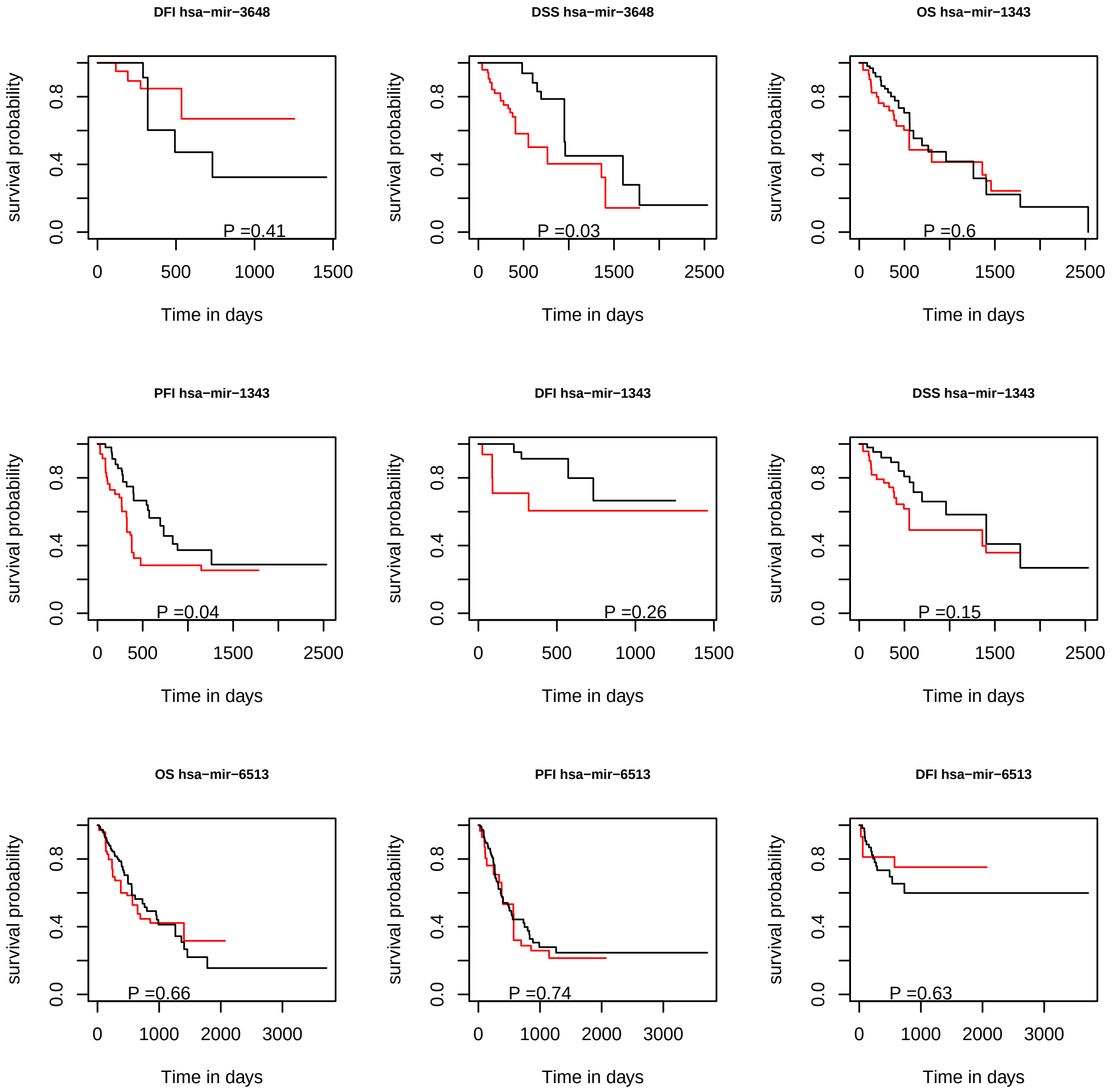
DFI hsa−mir−3648
survival probability
0.0
0.4
0.8
0
500
1000
1500
Time in days
P =0.41
DSS hsa−mir−3648
survival probability
0.0
0.4
0.8
0
500
1500
2500
Time in days
P =0.03
OS hsa−mir−1343
survival probability
0.0
0.4
0.8
0
500
1500
2500
Time in days
P =0.6
PFI hsa−mir−1343
survival probability
0.0
0.4
0.8
0
500
1500
2500
Time in days
P =0.04
DFI hsa−mir−1343
survival probability
0.0
0.4
0.8
0
500
1000
1500
Time in days
P =0.26
DSS hsa−mir−1343
survival probability
0.0
0.4
0.8
0
500
1500
2500
Time in days
P =0.15
OS hsa−mir−6513
survival probability
0.0
0.4
0.8
0
1000
2000
3000
Time in days
P =0.66
PFI hsa−mir−6513
survival probability
0.0
0.4
0.8
0
1000
2000
3000
Time in days
P =0.74
DFI hsa−mir−6513
survival probability
0.0
0.4
0.8
0
1000
2000
3000
Time in days
P =0.63

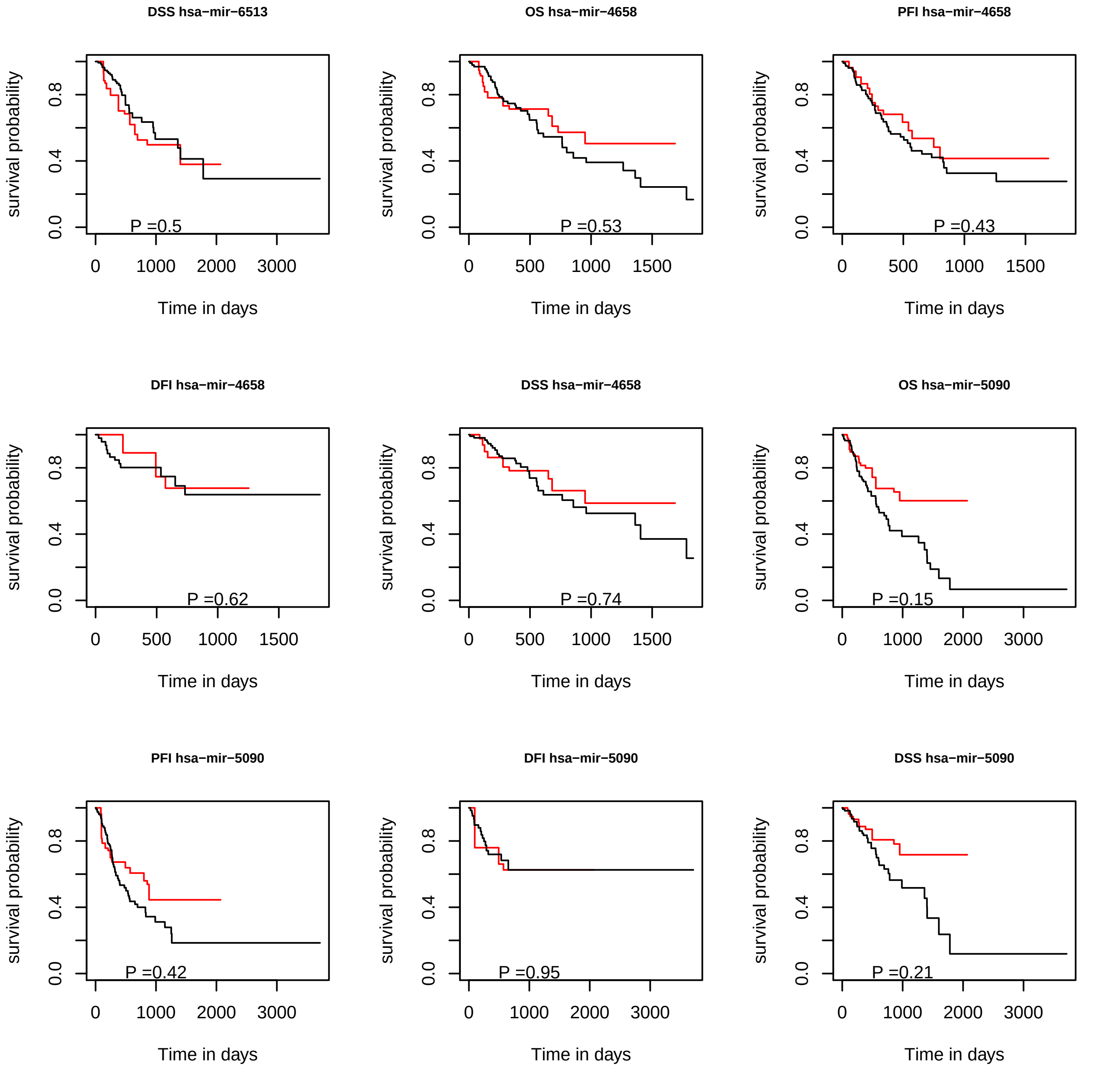
DSS hsa−mir−6513
P =0.5
OS hsa−mir−4658
P =0.53
PFI hsa−mir−4658
P =0.43
DFI hsa−mir−4658
P =0.62
DSS hsa−mir−4658
P =0.74
OS hsa−mir−5090
P =0.15
PFI hsa−mir−5090
P =0.42
DFI hsa−mir−5090
P =0.95
DSS hsa−mir−5090
P =0.21
survival probability
Time in days
0.0
0.4
0.8
0
500
1000
1500
2000
3000

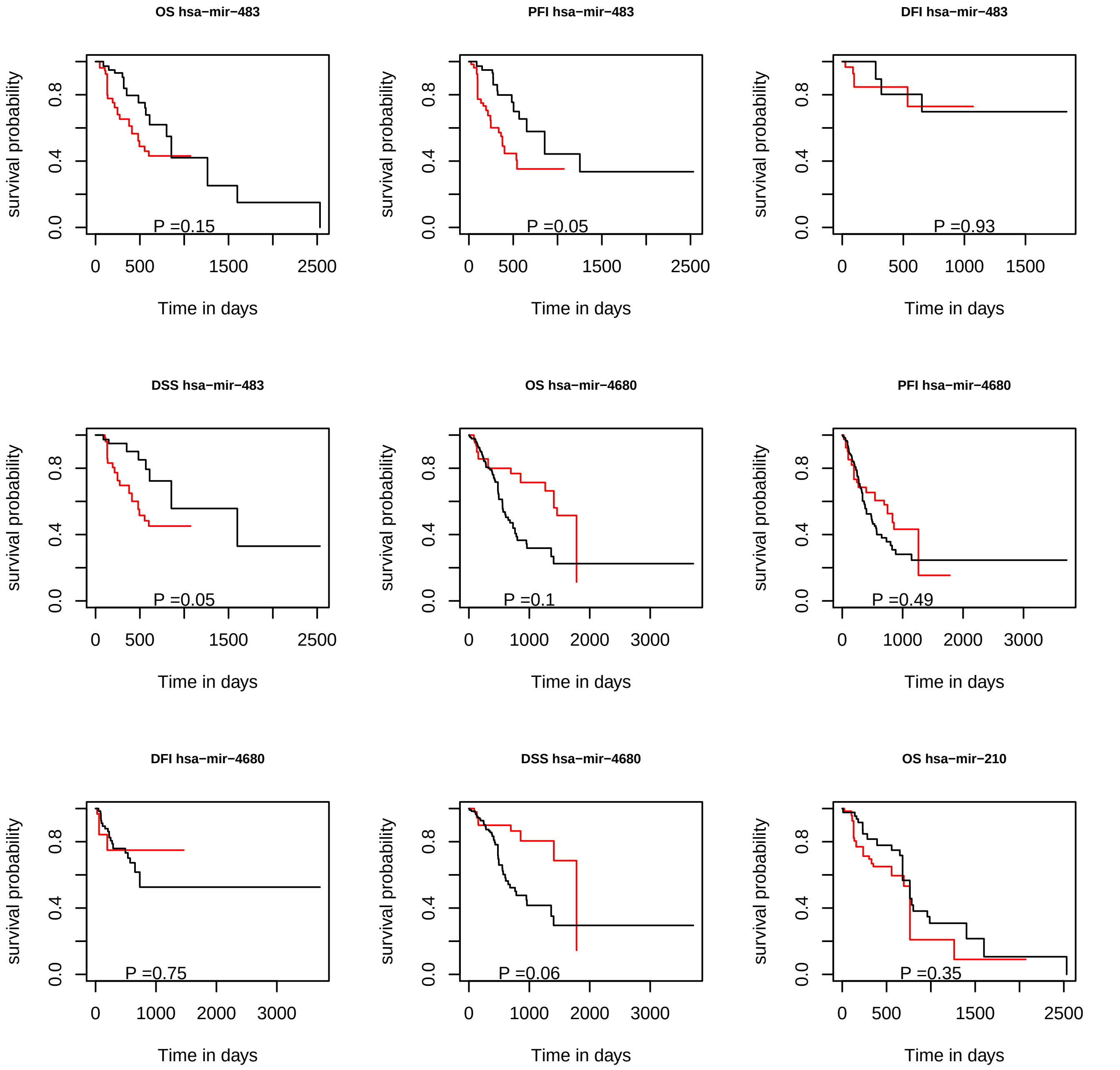
OS hsa−mir−483
PFI hsa−mir−483
DFI hsa−mir−483
DSS hsa−mir−483
OS hsa−mir−4680
PFI hsa−mir−4680
DFI hsa−mir−4680
DSS hsa−mir−4680
OS hsa−mir−210
survival probability
Time in days
P =0.15
P =0.05
P =0.93
P =0.05
P =0.1
P =0.49
P =0.75
P =0.06
P =0.35

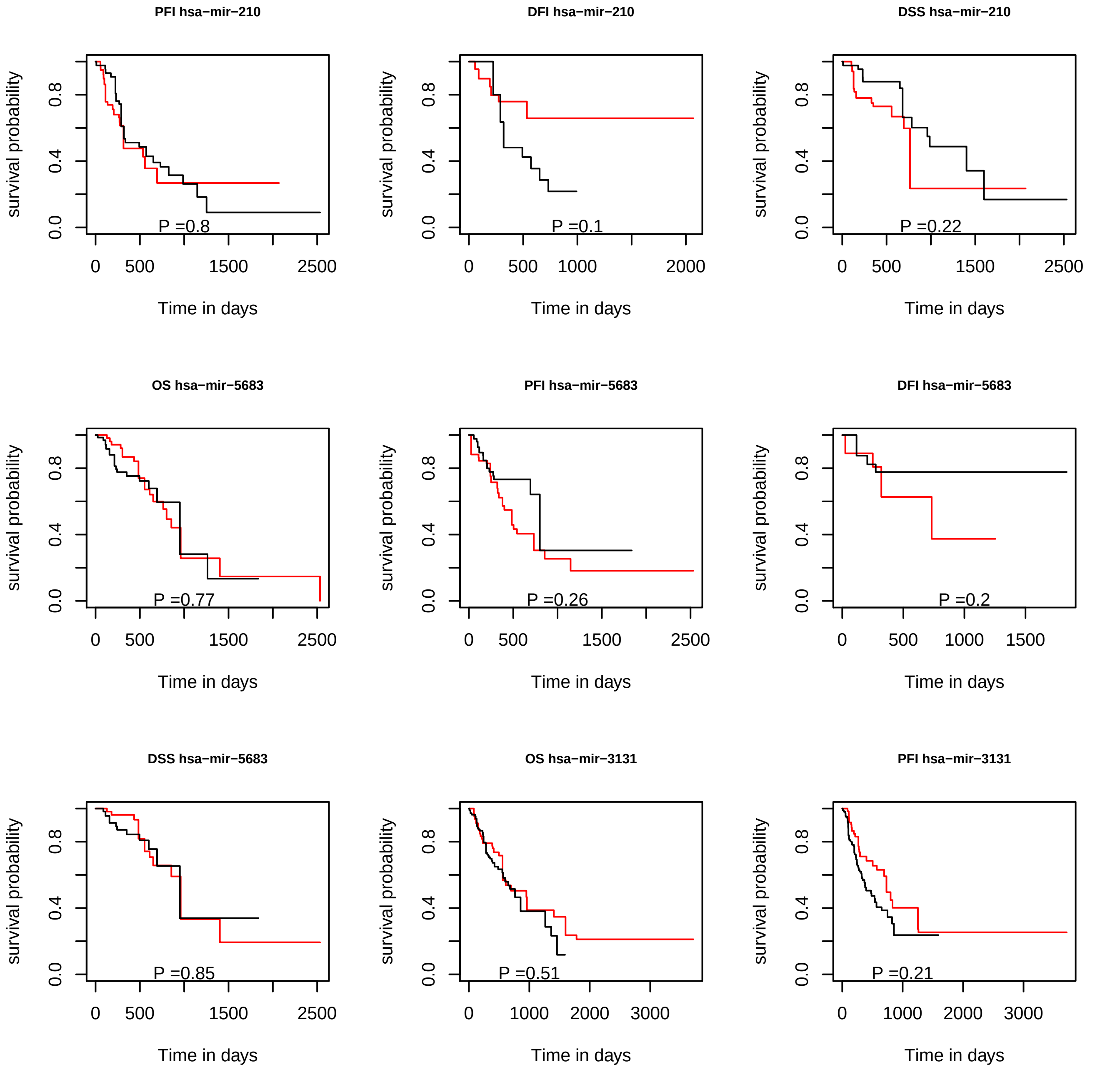
PFI hsa−mir−210
P =0.8
DFI hsa−mir−210
P =0.1
DSS hsa−mir−210
P =0.22
OS hsa−mir−5683
P =0.77
PFI hsa−mir−5683
P =0.26
DFI hsa−mir−5683
P =0.2
DSS hsa−mir−5683
P =0.85
OS hsa−mir−3131
P =0.51
PFI hsa−mir−3131
P =0.21
survival probability
Time in days
0.0
0.4
0.8
0
500
1000
1500
2000
2500
3000

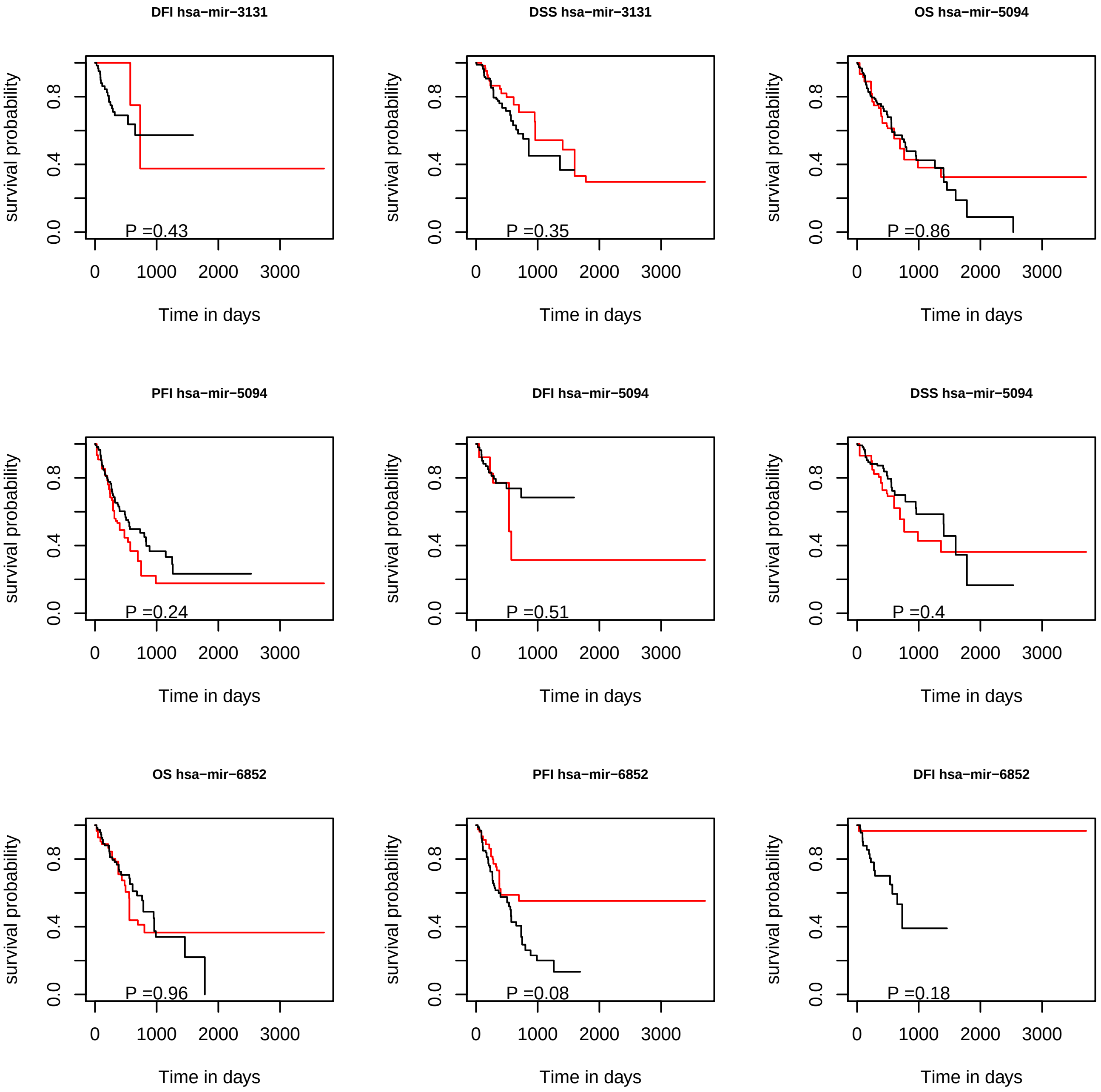
DFI hsa−mir−3131
P =0.43
DSS hsa−mir−3131
P =0.35
OS hsa−mir−5094
P =0.86
PFI hsa−mir−5094
P =0.24
DFI hsa−mir−5094
P =0.51
DSS hsa−mir−5094
P =0.4
OS hsa−mir−6852
P =0.96
PFI hsa−mir−6852
P =0.08
DFI hsa−mir−6852
P =0.18
survival probability
Time in days
0.0
0.4
0.8
0
1000
2000
3000

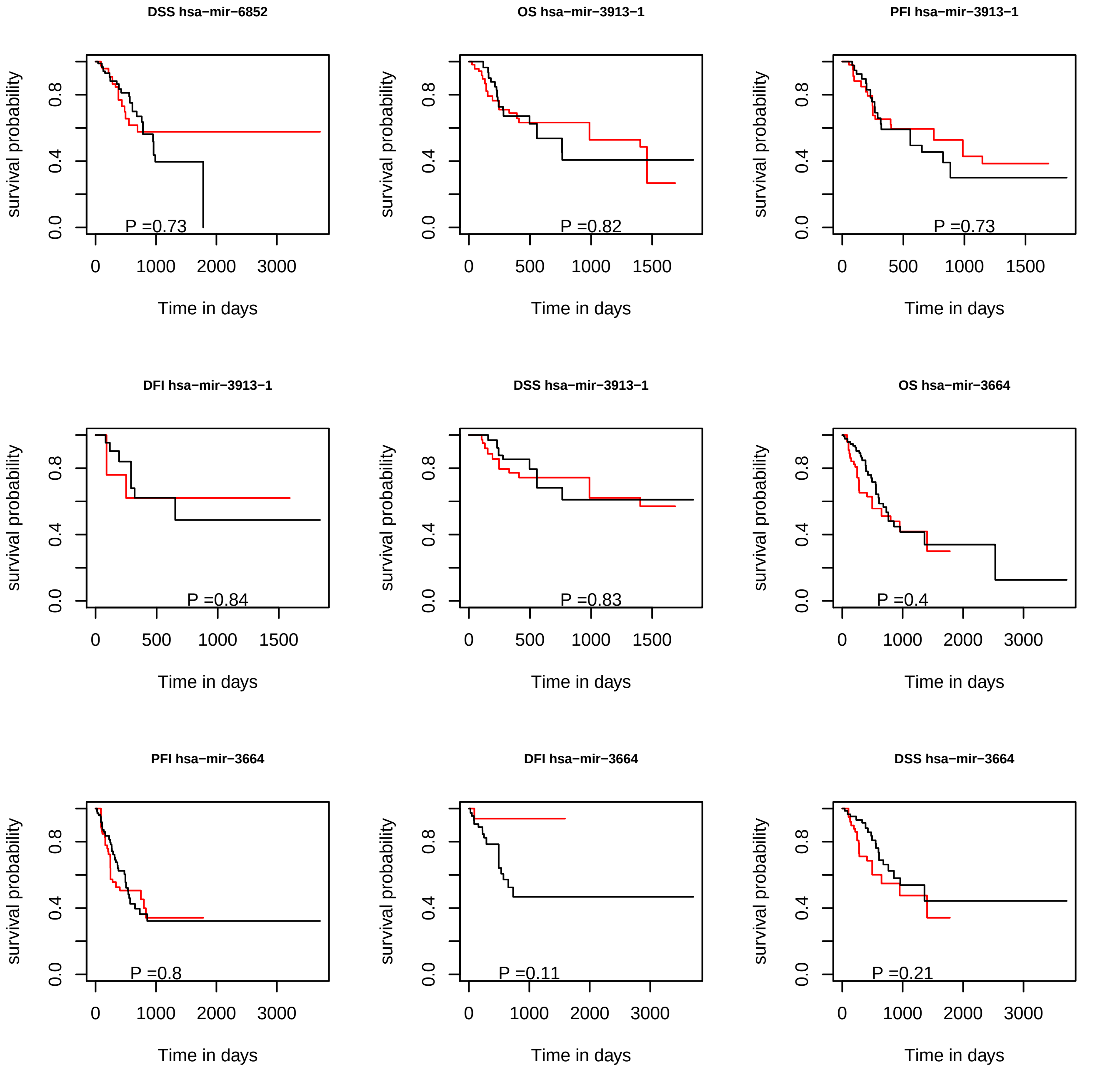
DSS hsa−mir−6852
P =0.73
OS hsa−mir−3913−1
P =0.82
PFI hsa−mir−3913−1
P =0.73
DFI hsa−mir−3913−1
P =0.84
DSS hsa−mir−3913−1
P =0.83
OS hsa−mir−3664
P =0.4
PFI hsa−mir−3664
P =0.8
DFI hsa−mir−3664
P =0.11
DSS hsa−mir−3664
P =0.21
survival probability
Time in days
0.0
0.4
0.8
0
500
1000
1500
2000
3000

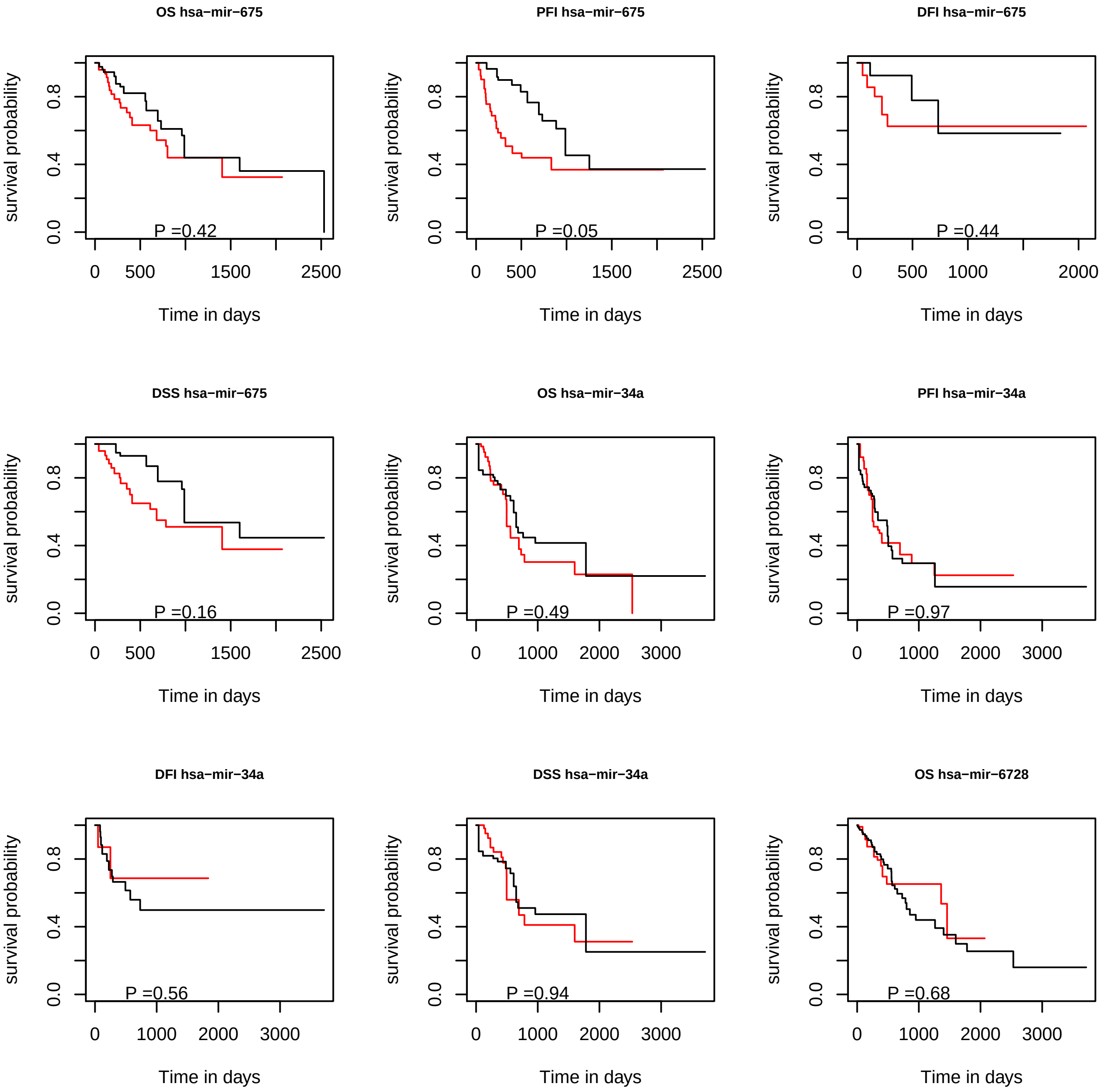
OS hsa−mir−675
P =0.42
PFI hsa−mir−675
P =0.05
DFI hsa−mir−675
P =0.44
DSS hsa−mir−675
P =0.16
OS hsa−mir−34a
P =0.49
PFI hsa−mir−34a
P =0.97
DFI hsa−mir−34a
P =0.56
DSS hsa−mir−34a
P =0.94
OS hsa−mir−6728
P =0.68
survival probability
Time in days
0.0
0.4
0.8
0
500
1000
1500
2000
2500
3000

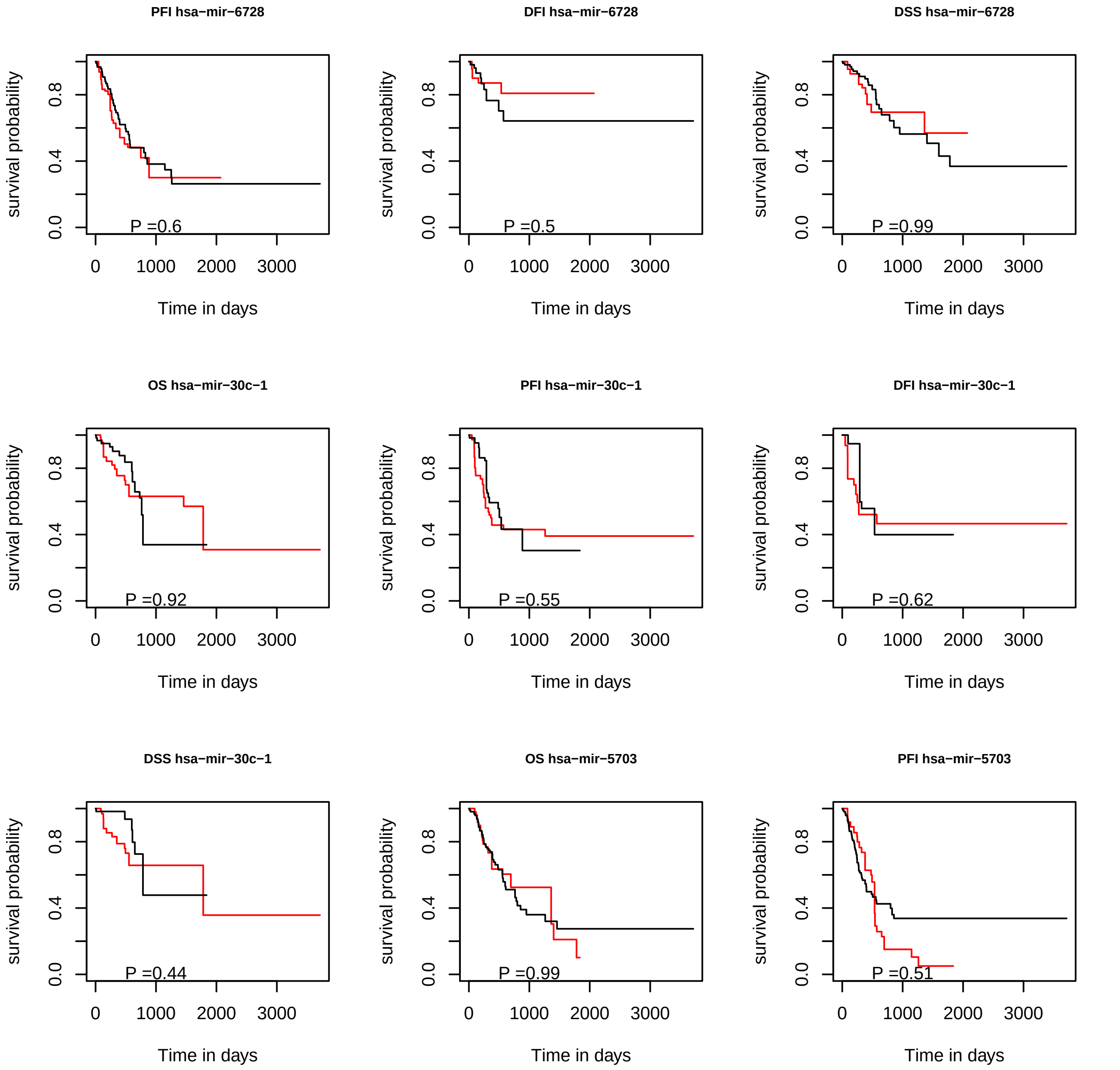
PFI hsa−mir−6728
P =0.6
DFI hsa−mir−6728
P =0.5
DSS hsa−mir−6728
P =0.99
OS hsa−mir−30c−1
P =0.92
PFI hsa−mir−30c−1
P =0.55
DFI hsa−mir−30c−1
P =0.62
DSS hsa−mir−30c−1
P =0.44
OS hsa−mir−5703
P =0.99
PFI hsa−mir−5703
P =0.51
survival probability
0.0
0.4
0.8
Time in days
0
1000
2000
3000

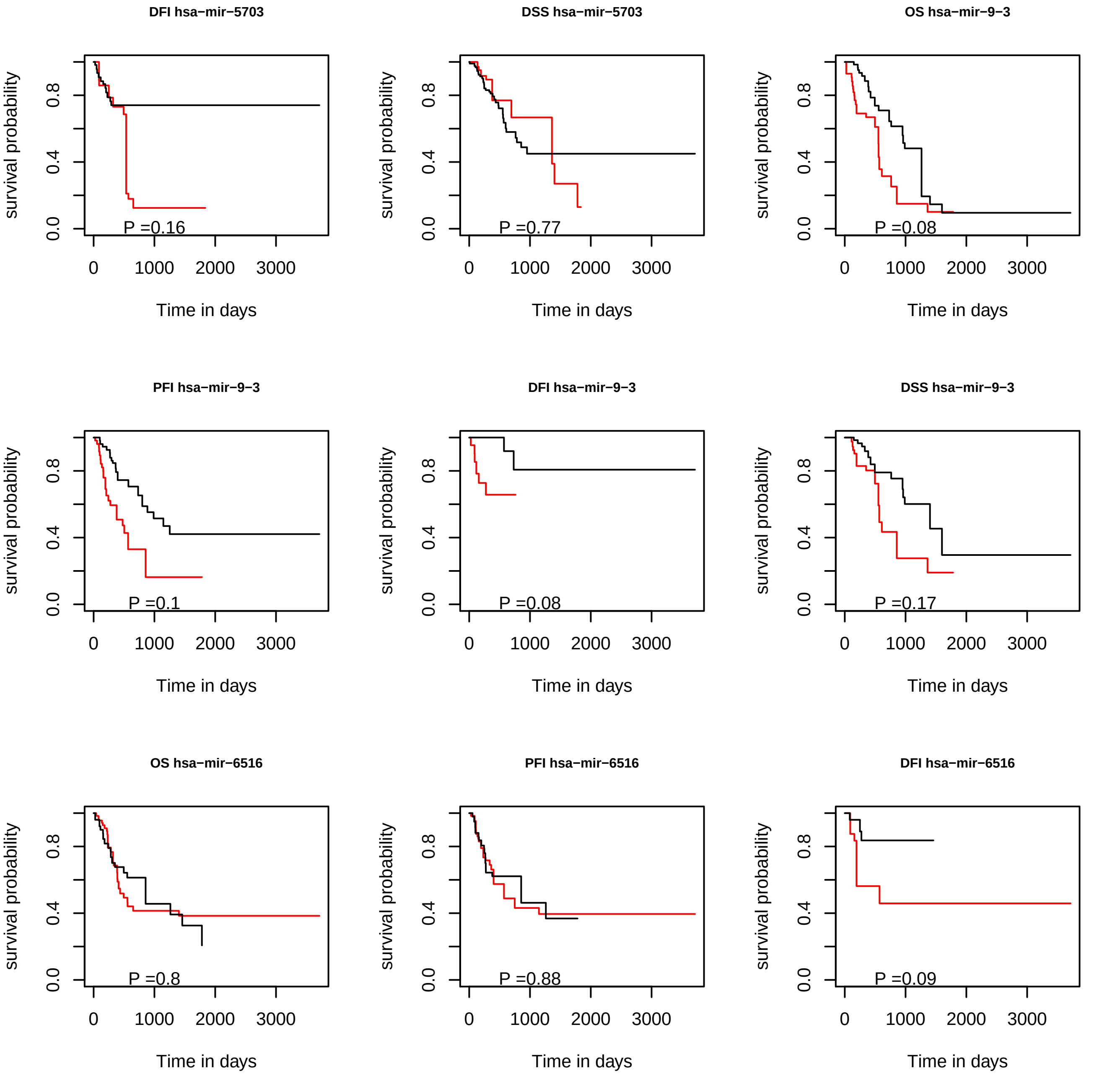
DFI hsa−mir−5703
P =0.16
DSS hsa−mir−5703
P =0.77
OS hsa−mir−9−3
P =0.08
PFI hsa−mir−9−3
P =0.1
DFI hsa−mir−9−3
P =0.08
DSS hsa−mir−9−3
P =0.17
OS hsa−mir−6516
P =0.8
PFI hsa−mir−6516
P =0.88
DFI hsa−mir−6516
P =0.09
survival probability
Time in days
0.0
0.4
0.8
0
1000
2000
3000

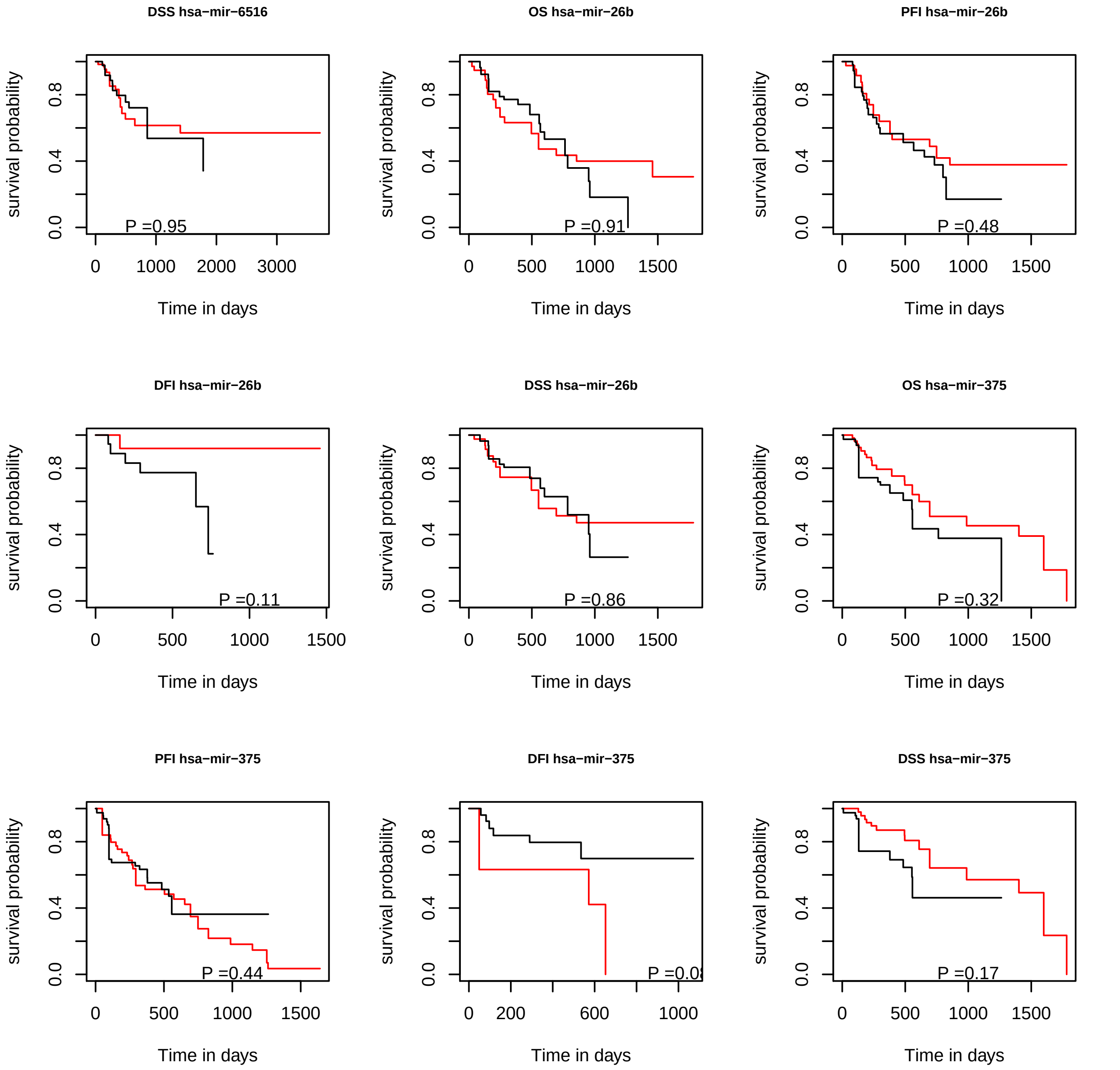
DSS hsa−mir−6516
survival probability
0.0
0.4
0.8
P =0.95
0
1000
2000
3000
Time in days
OS hsa−mir−26b
survival probability
0.0
0.4
0.8
P =0.91
0
500
1000
1500
Time in days
PFI hsa−mir−26b
survival probability
0.0
0.4
0.8
P =0.48
0
500
1000
1500
Time in days
DFI hsa−mir−26b
survival probability
0.0
0.4
0.8
P =0.11
0
500
1000
1500
Time in days
DSS hsa−mir−26b
survival probability
0.0
0.4
0.8
P =0.86
0
500
1000
1500
Time in days
OS hsa−mir−375
survival probability
0.0
0.4
0.8
P =0.32
0
500
1000
1500
Time in days
PFI hsa−mir−375
survival probability
0.0
0.4
0.8
P =0.44
0
500
1000
1500
Time in days
DFI hsa−mir−375
survival probability
0.0
0.4
0.8
P =0.0
0
200
600
1000
Time in days
DSS hsa−mir−375
survival probability
0.0
0.4
0.8
P =0.17
0
500
1000
1500
Time in days

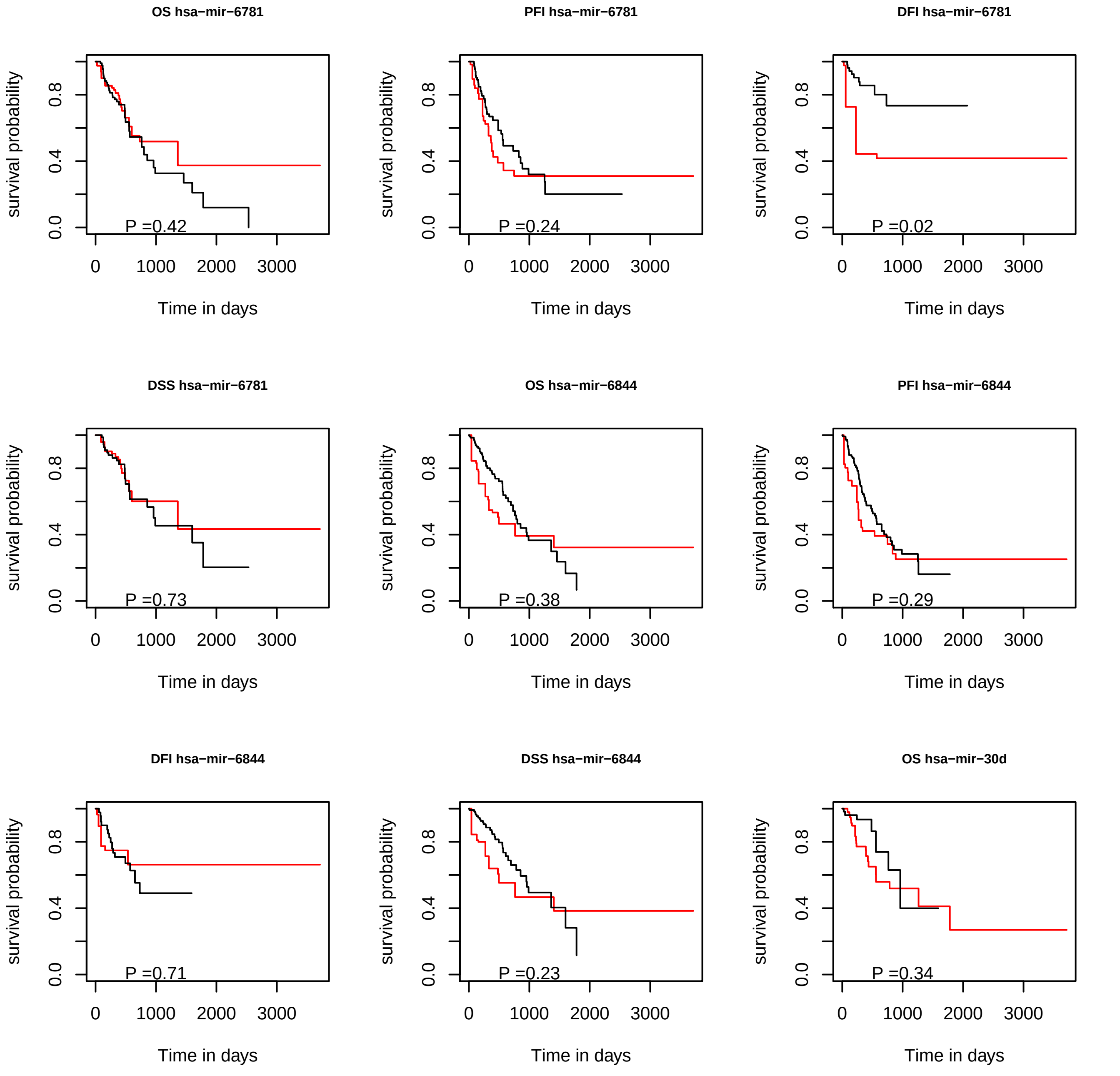
OS hsa−mir−6781
survival probability
0.0
0.4
0.8
P =0.42
0
1000
2000
3000
Time in days
PFI hsa−mir−6781
survival probability
0.0
0.4
0.8
P =0.24
0
1000
2000
3000
Time in days
DFI hsa−mir−6781
survival probability
0.0
0.4
0.8
P =0.02
0
1000
2000
3000
Time in days
DSS hsa−mir−6781
survival probability
0.0
0.4
0.8
P =0.73
0
1000
2000
3000
Time in days
OS hsa−mir−6844
survival probability
0.0
0.4
0.8
P =0.38
0
1000
2000
3000
Time in days
PFI hsa−mir−6844
survival probability
0.0
0.4
0.8
P =0.29
0
1000
2000
3000
Time in days
DFI hsa−mir−6844
survival probability
0.0
0.4
0.8
P =0.71
0
1000
2000
3000
Time in days
DSS hsa−mir−6844
survival probability
0.0
0.4
0.8
P =0.23
0
1000
2000
3000
Time in days
OS hsa−mir−30d
survival probability
0.0
0.4
0.8
P =0.34
0
1000
2000
3000
Time in days

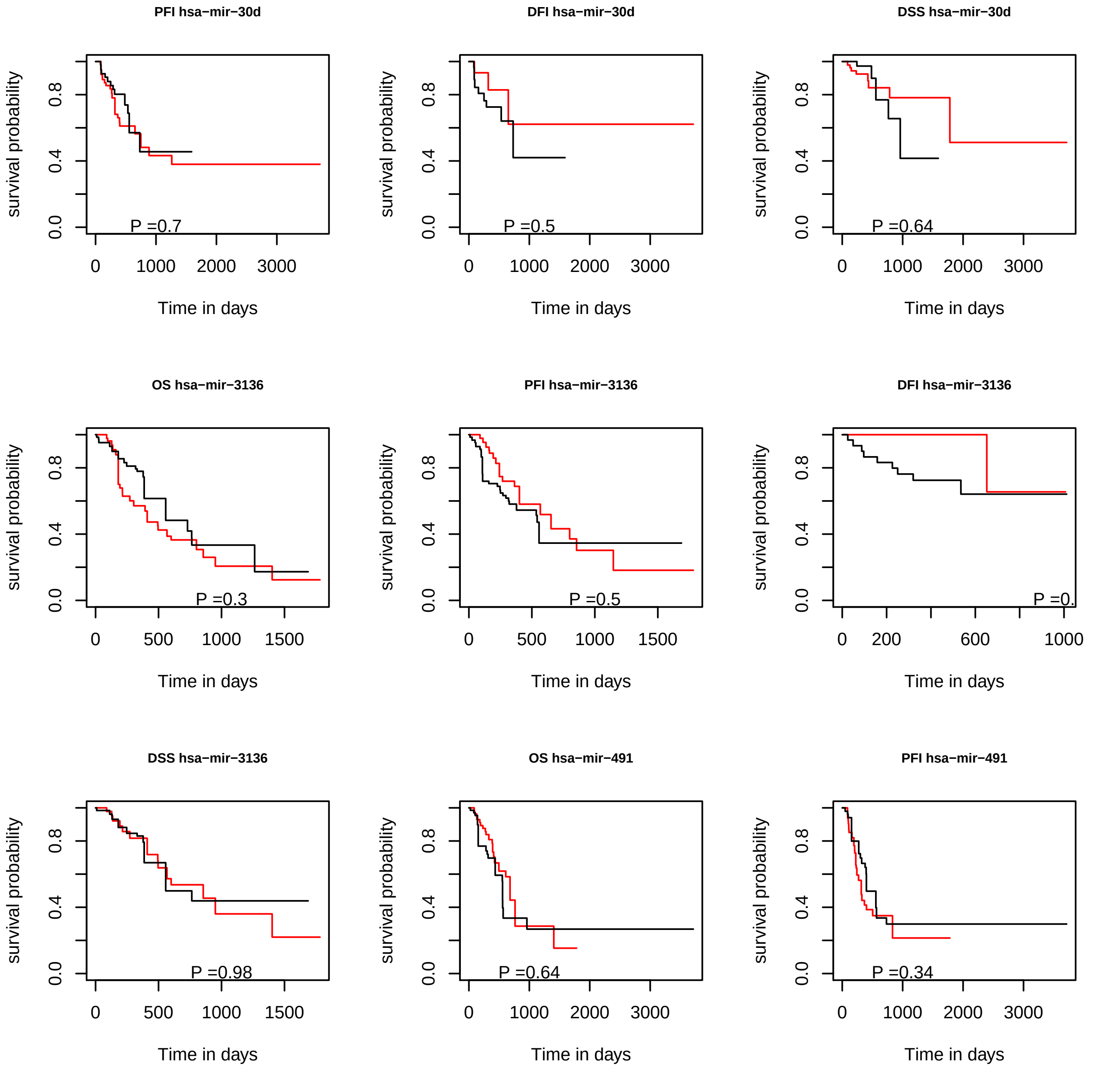
PFI hsa−mir−30d
P =0.7
DFI hsa−mir−30d
P =0.5
DSS hsa−mir−30d
P =0.64
OS hsa−mir−3136
P =0.3
PFI hsa−mir−3136
P =0.5
DFI hsa−mir−3136
P =0.
DSS hsa−mir−3136
P =0.98
OS hsa−mir−491
P =0.64
PFI hsa−mir−491
P =0.34
survival probability
Time in days

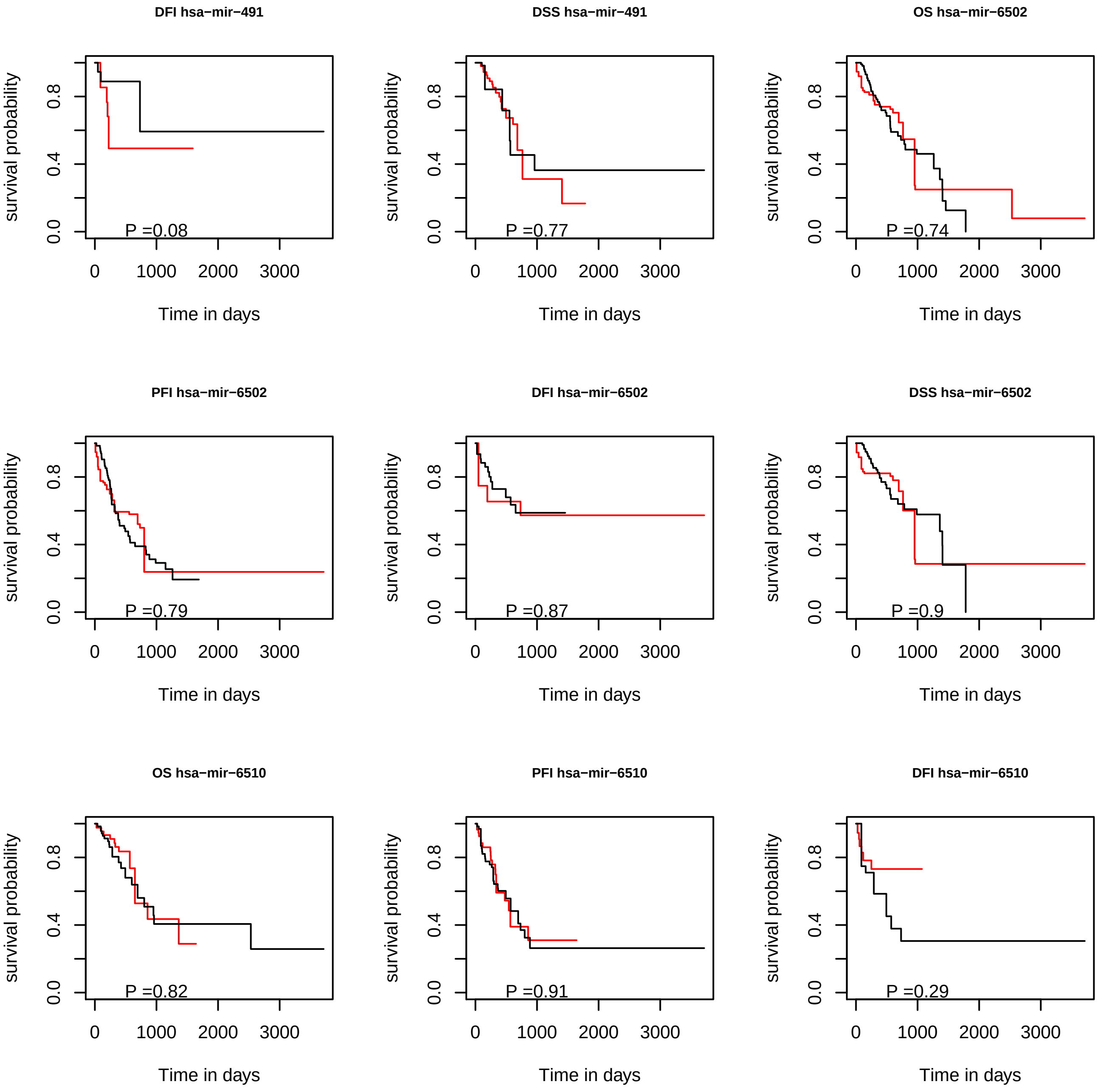
DFI hsa−mir−491
P =0.08
DSS hsa−mir−491
P =0.77
OS hsa−mir−6502
P =0.74
PFI hsa−mir−6502
P =0.79
DFI hsa−mir−6502
P =0.87
DSS hsa−mir−6502
P =0.9
OS hsa−mir−6510
P =0.82
PFI hsa−mir−6510
P =0.91
DFI hsa−mir−6510
P =0.29
survival probability
0.0
0.4
0.8
0
1000
2000
3000
Time in days

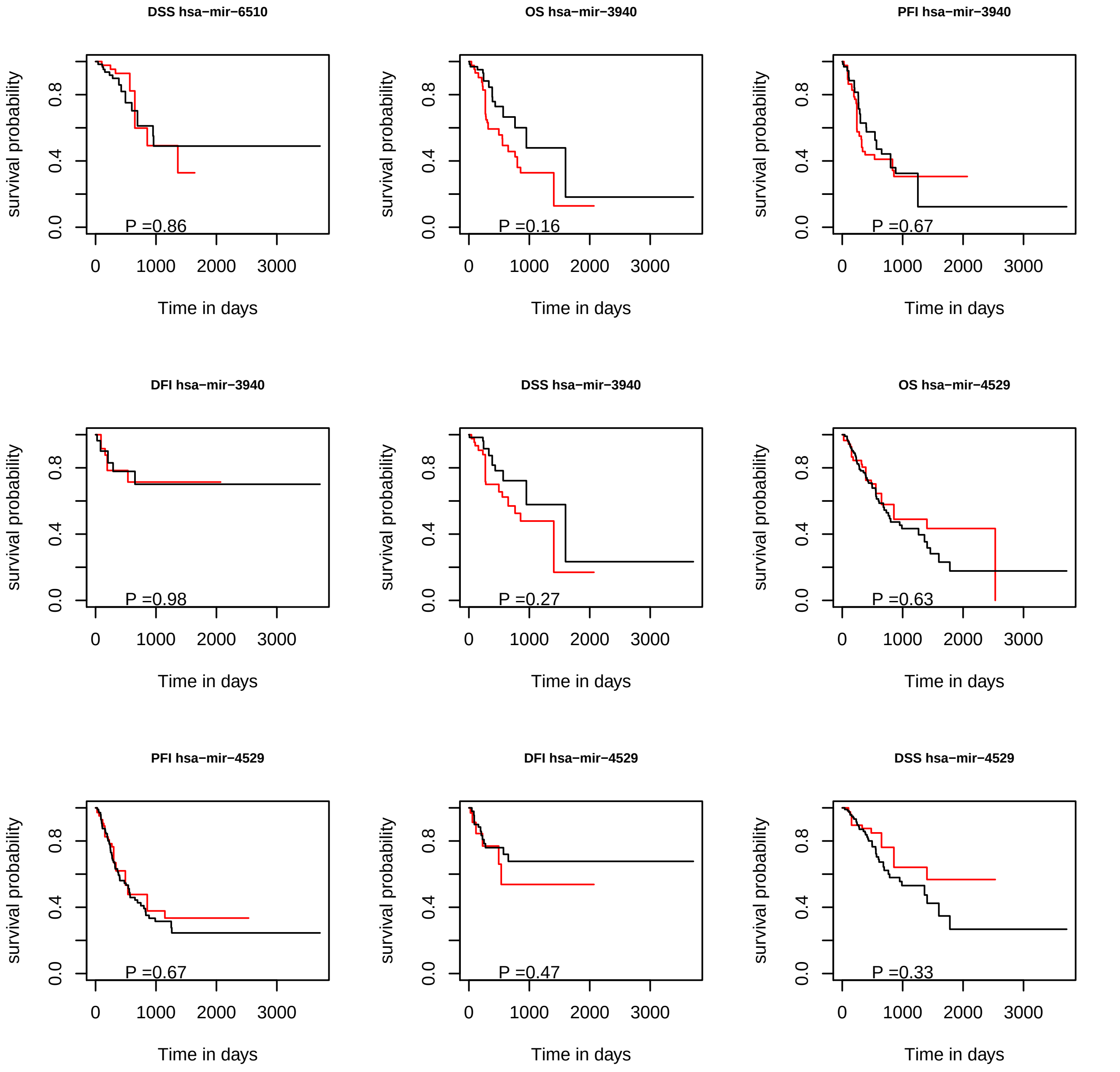
DSS hsa−mir−6510
P =0.86
OS hsa−mir−3940
P =0.16
PFI hsa−mir−3940
P =0.67
DFI hsa−mir−3940
P =0.98
DSS hsa−mir−3940
P =0.27
OS hsa−mir−4529
P =0.63
PFI hsa−mir−4529
P =0.67
DFI hsa−mir−4529
P =0.47
DSS hsa−mir−4529
P =0.33
survival probability
Time in days
0.0
0.4
0.8
0
1000
2000
3000

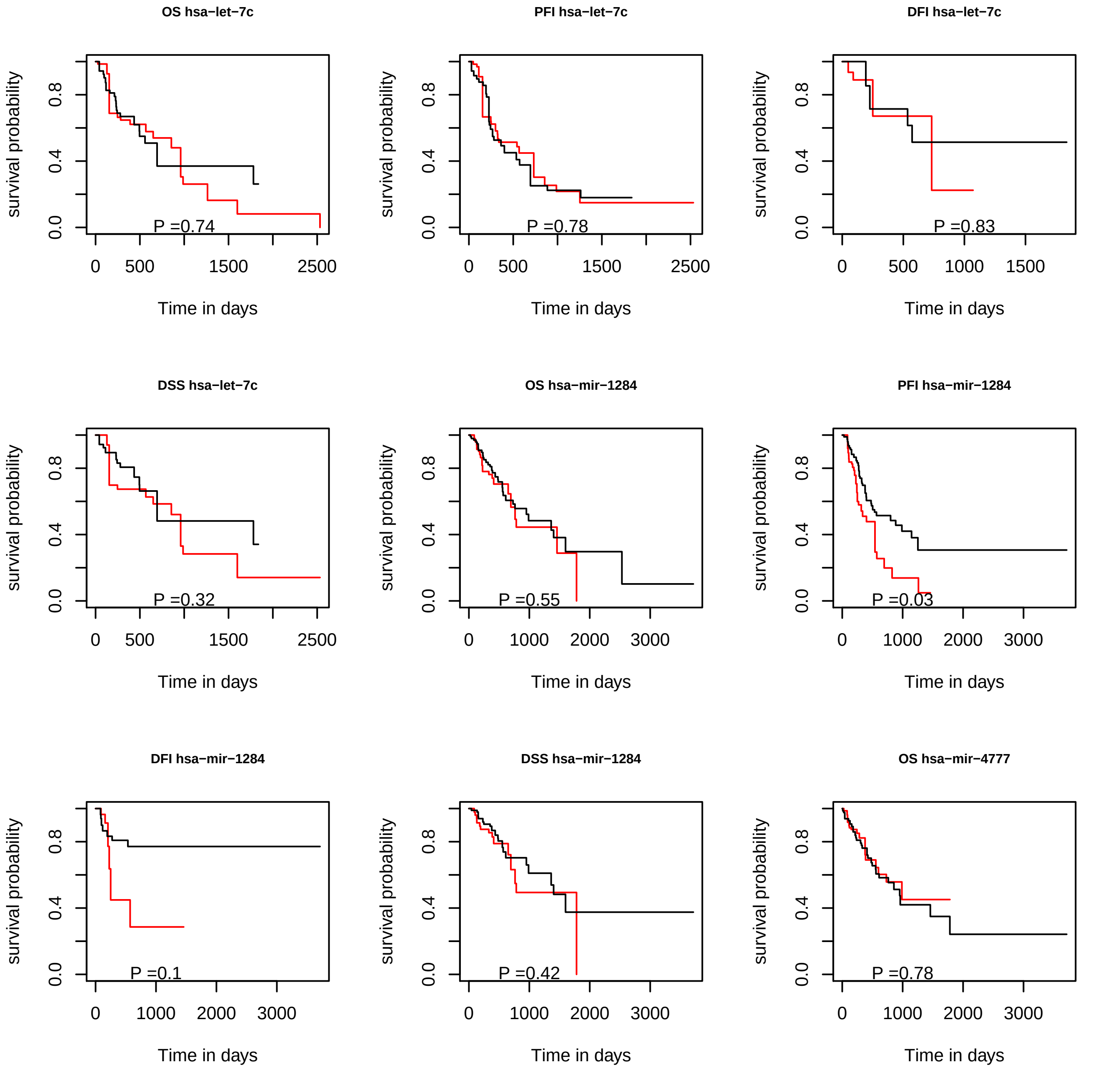
OS hsa−let−7c
survival probability
0.0
0.4
0.8
P =0.74
0
500
1500
2500
Time in days
PFI hsa−let−7c
survival probability
0.0
0.4
0.8
P =0.78
0
500
1500
2500
Time in days
DFI hsa−let−7c
survival probability
0.0
0.4
0.8
P =0.83
0
500
1000
1500
Time in days
DSS hsa−let−7c
survival probability
0.0
0.4
0.8
P =0.32
0
500
1500
2500
Time in days
OS hsa−mir−1284
survival probability
0.0
0.4
0.8
P =0.55
0
1000
2000
3000
Time in days
PFI hsa−mir−1284
survival probability
0.0
0.4
0.8
P =0.03
0
1000
2000
3000
Time in days
DFI hsa−mir−1284
survival probability
0.0
0.4
0.8
P =0.1
0
1000
2000
3000
Time in days
DSS hsa−mir−1284
survival probability
0.0
0.4
0.8
P =0.42
0
1000
2000
3000
Time in days
OS hsa−mir−4777
survival probability
0.0
0.4
0.8
P =0.78
0
1000
2000
3000
Time in days

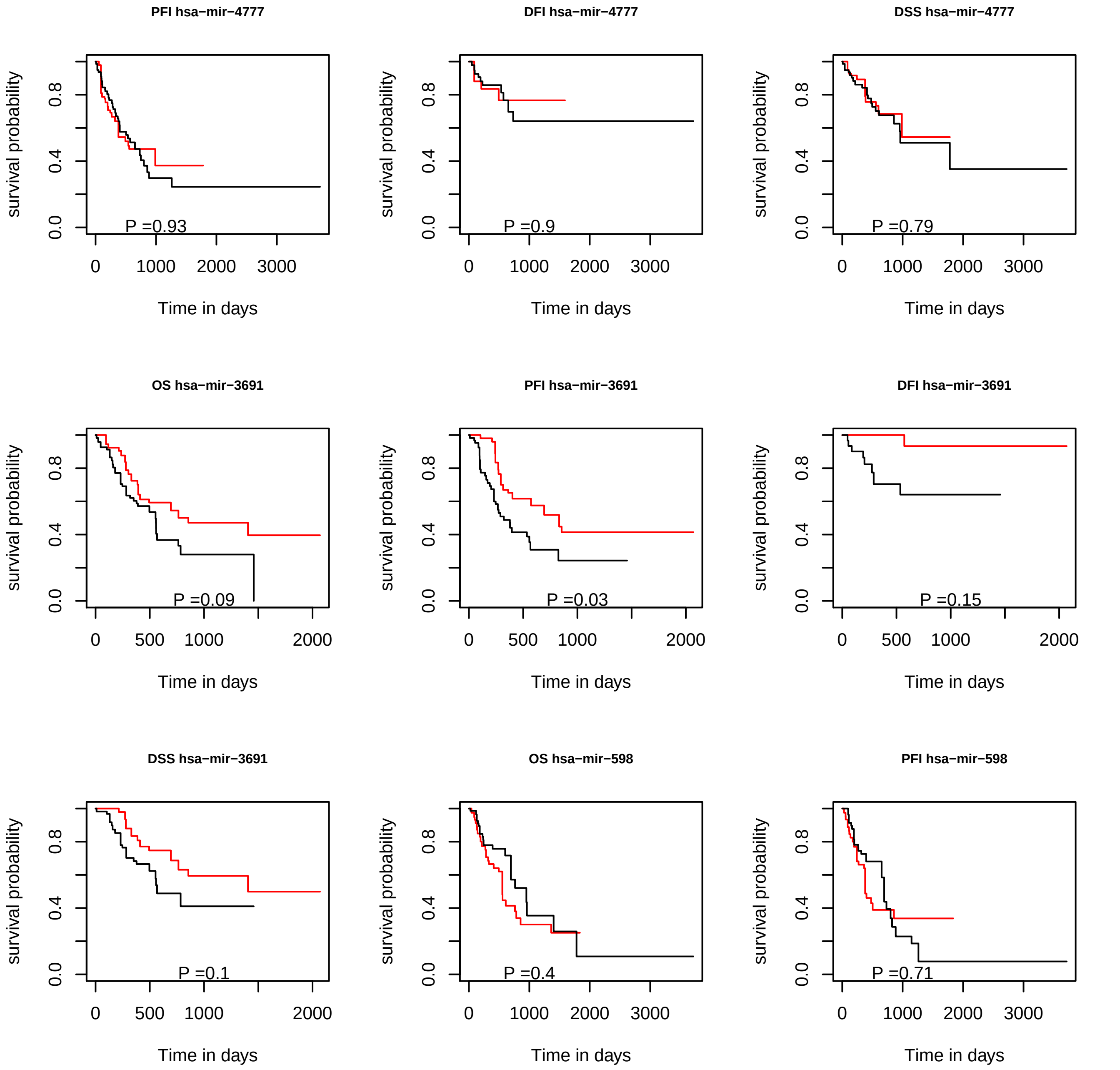
PFI hsa−mir−4777
P =0.93
DFI hsa−mir−4777
P =0.9
DSS hsa−mir−4777
P =0.79
OS hsa−mir−3691
P =0.09
PFI hsa−mir−3691
P =0.03
DFI hsa−mir−3691
P =0.15
DSS hsa−mir−3691
P =0.1
OS hsa−mir−598
P =0.4
PFI hsa−mir−598
P =0.71
survival probability
0.0
0.4
0.8
Time in days
0
500
1000
2000
3000

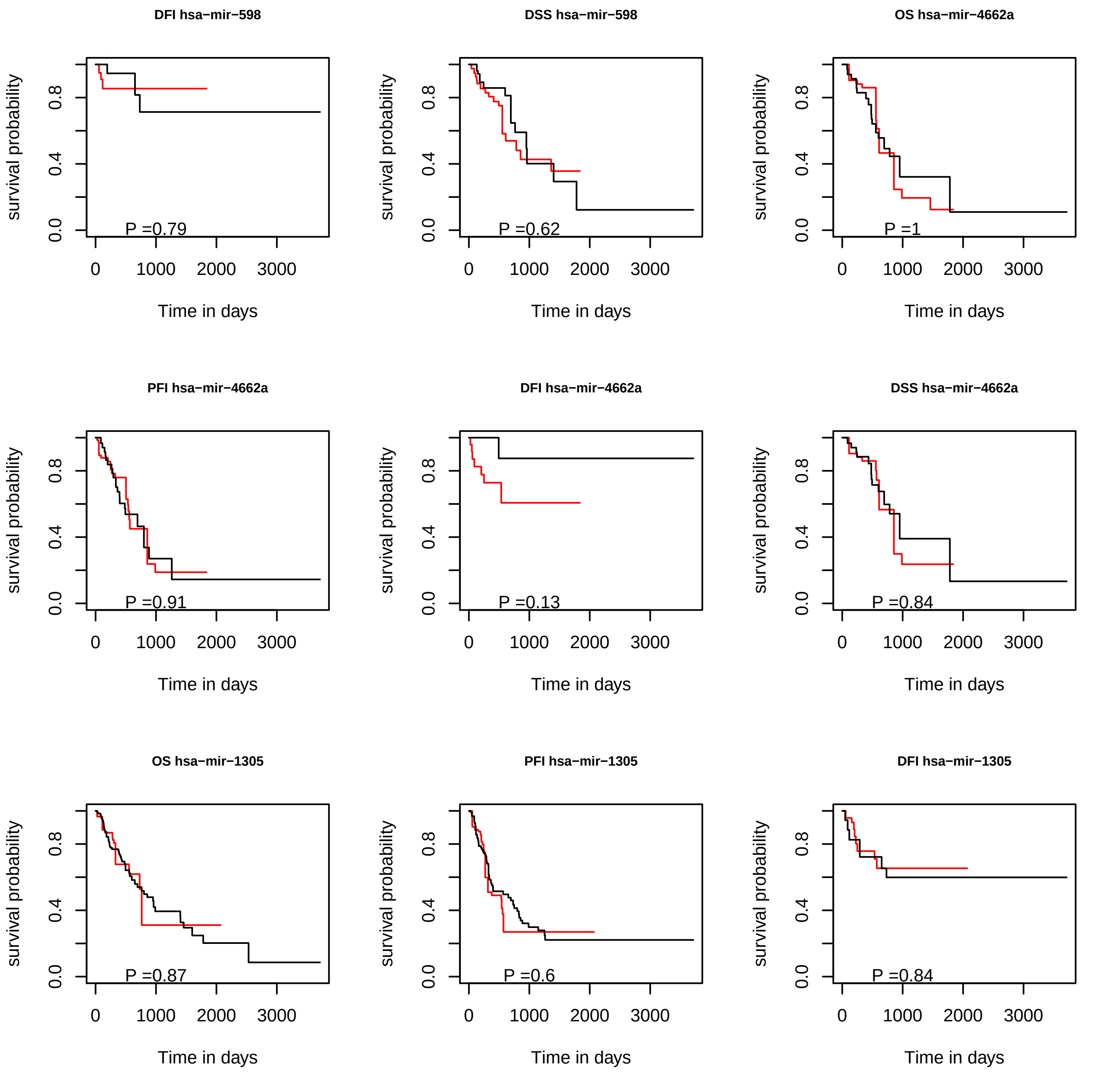
DFI hsa−mir−598
survival probability
0.0 0.4 0.8
P =0.79
0 1000 2000 3000
Time in days
DSS hsa−mir−598
survival probability
0.0 0.4 0.8
P =0.62
0 1000 2000 3000
Time in days
OS hsa−mir−4662a
survival probability
0.0 0.4 0.8
P =1
0 1000 2000 3000
Time in days
PFI hsa−mir−4662a
survival probability
0.0 0.4 0.8
P =0.91
0 1000 2000 3000
Time in days
DFI hsa−mir−4662a
survival probability
0.0 0.4 0.8
P =0.13
0 1000 2000 3000
Time in days
DSS hsa−mir−4662a
survival probability
0.0 0.4 0.8
P =0.84
0 1000 2000 3000
Time in days
OS hsa−mir−1305
survival probability
0.0 0.4 0.8
P =0.87
0 1000 2000 3000
Time in days
PFI hsa−mir−1305
survival probability
0.0 0.4 0.8
P =0.6
0 1000 2000 3000
Time in days
DFI hsa−mir−1305
survival probability
0.0 0.4 0.8
P =0.84
0 1000 2000 3000
Time in days

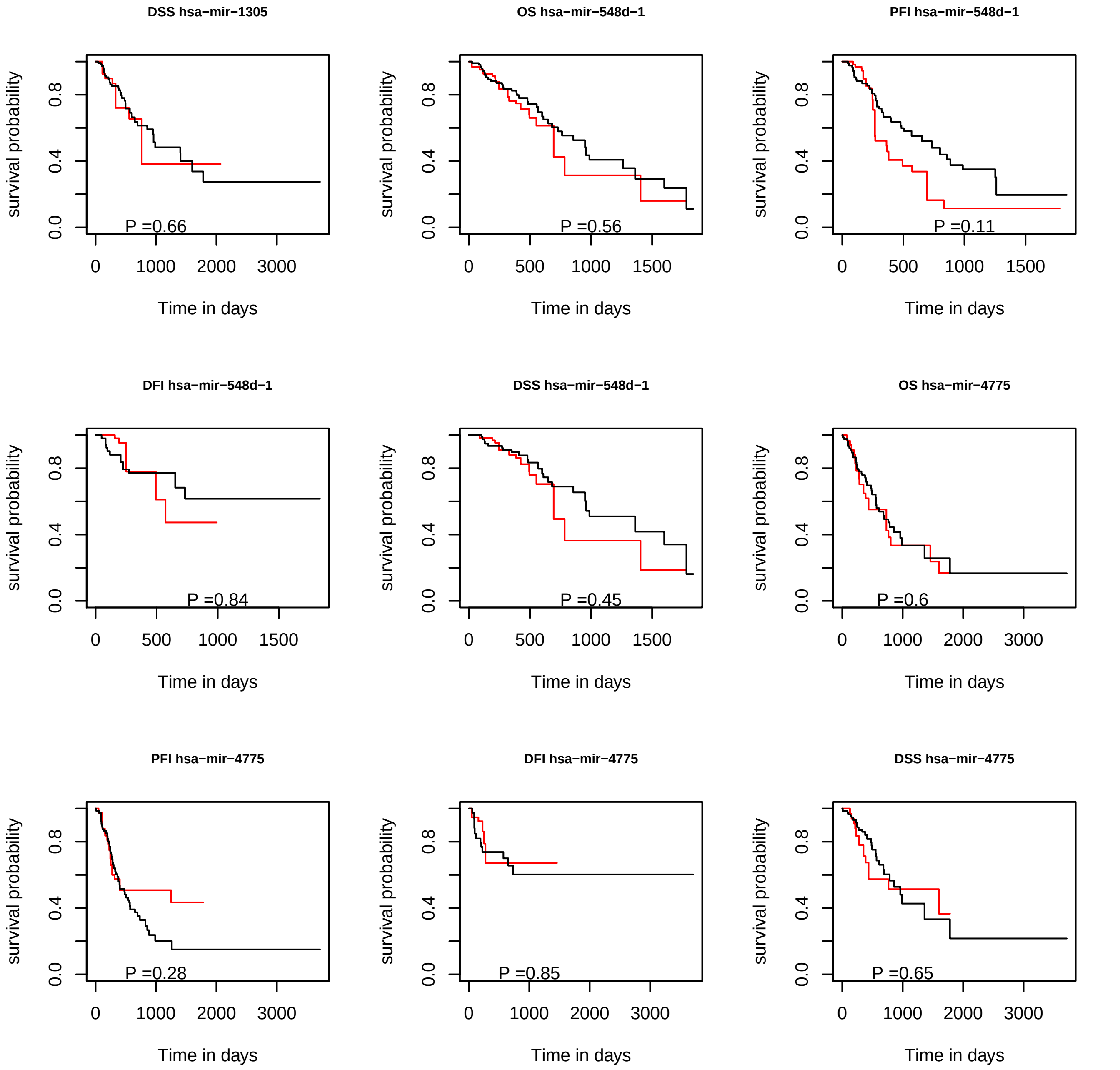
DSS hsa−mir−1305
P =0.66
OS hsa−mir−548d−1
P =0.56
PFI hsa−mir−548d−1
P =0.11
DFI hsa−mir−548d−1
P =0.84
DSS hsa−mir−548d−1
P =0.45
OS hsa−mir−4775
P =0.6
PFI hsa−mir−4775
P =0.28
DFI hsa−mir−4775
P =0.85
DSS hsa−mir−4775
P =0.65
survival probability
0.0
0.4
0.8
Time in days
0
500
1000
1500
2000
3000

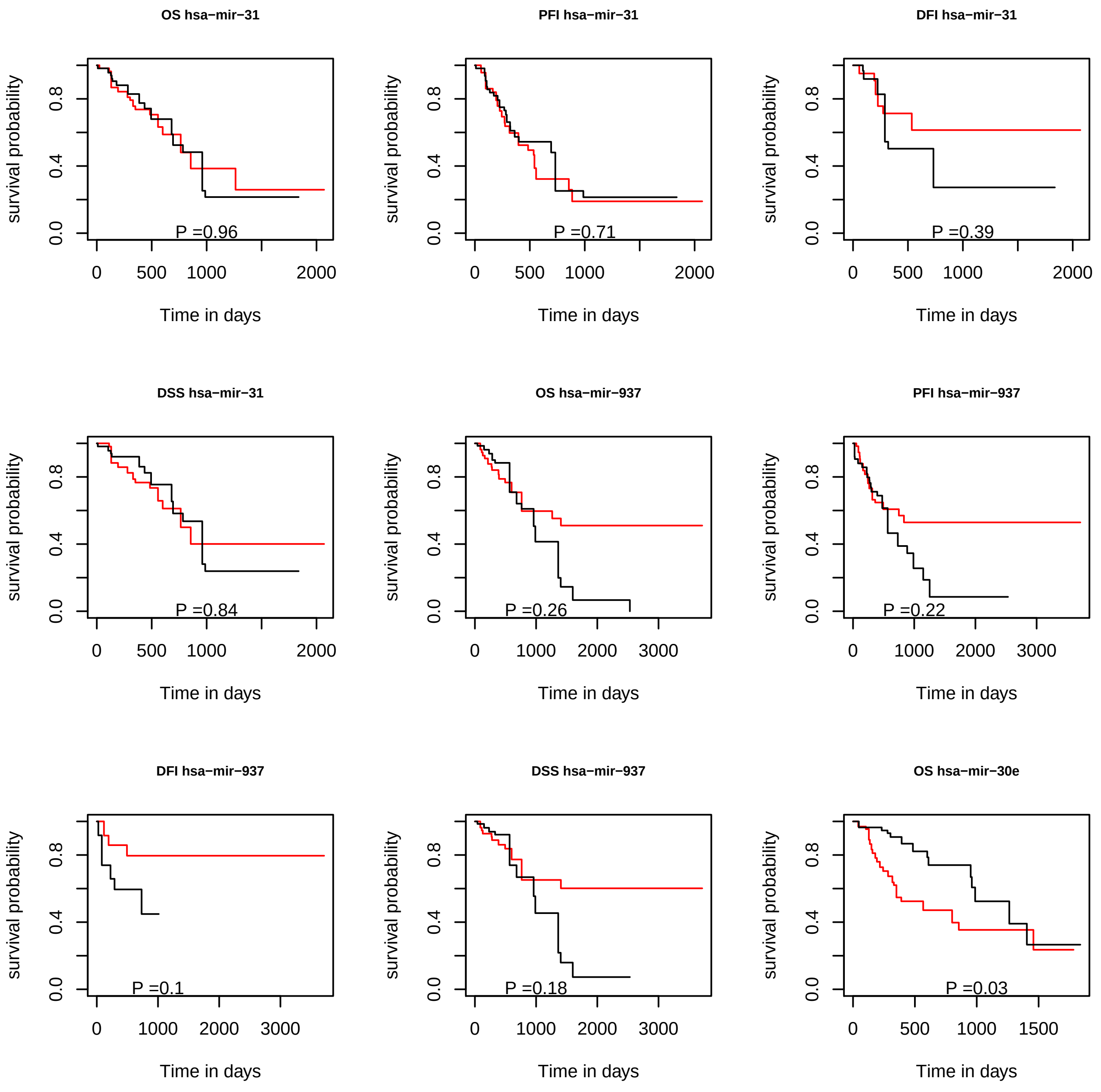

OS hsa−mir−31
P =0.96
PFI hsa−mir−31
P =0.71
DFI hsa−mir−31
P =0.39
DSS hsa−mir−31
P =0.84
OS hsa−mir−937
P =0.26
PFI hsa−mir−937
P =0.22
DFI hsa−mir−937
P =0.1
DSS hsa−mir−937
P =0.18
OS hsa−mir−30e
P =0.03
survival probability
Time in days
0.0
0.4
0.8
0
500
1000
1500
2000
3000

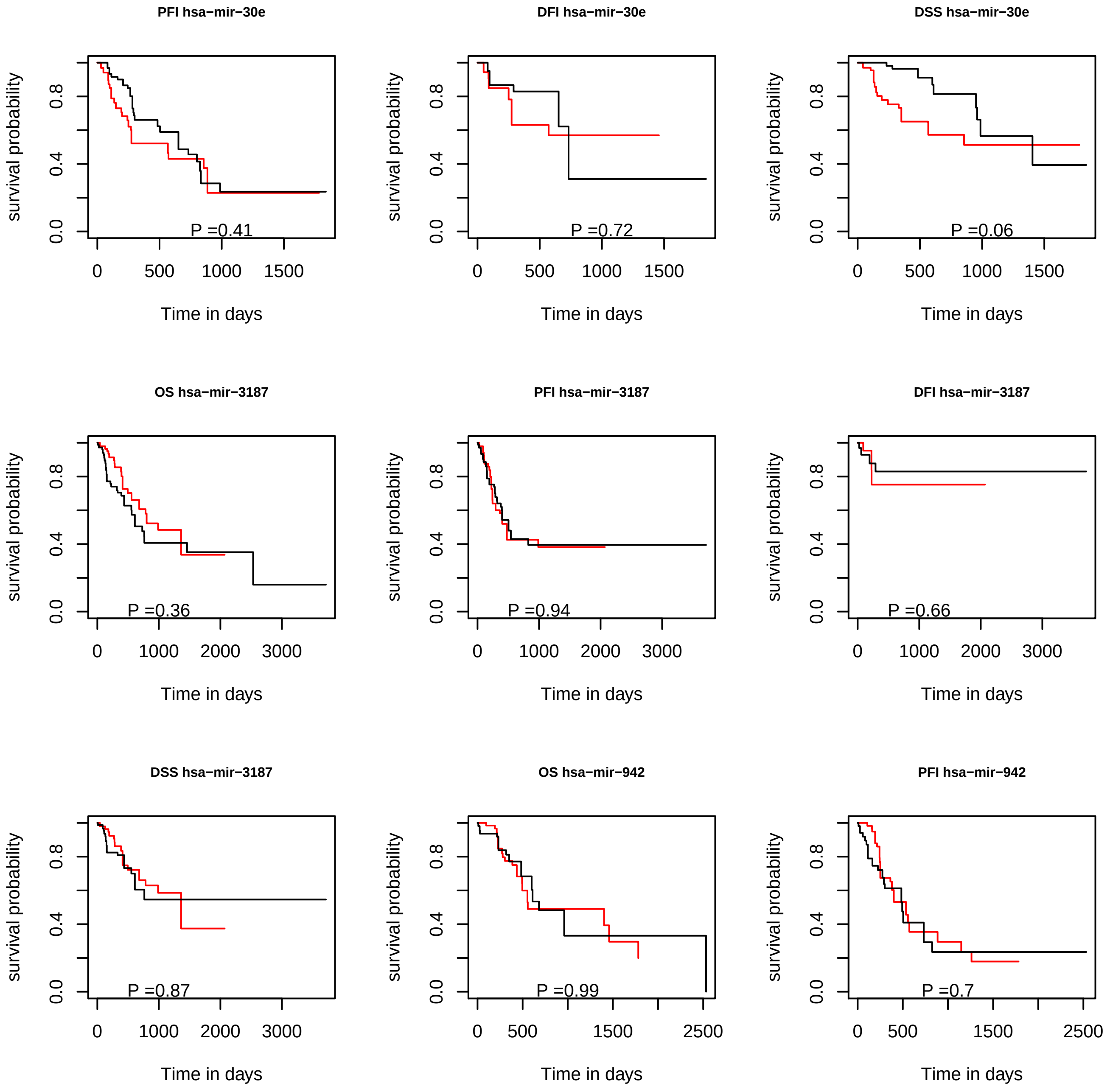
PFI hsa−mir−30e
survival probability
0.0
0.4
0.8
P =0.41
0
500
1000
1500
Time in days
DFI hsa−mir−30e
survival probability
0.0
0.4
0.8
P =0.72
0
500
1000
1500
Time in days
DSS hsa−mir−30e
survival probability
0.0
0.4
0.8
P =0.06
0
500
1000
1500
Time in days
OS hsa−mir−3187
survival probability
0.0
0.4
0.8
P =0.36
0
1000
2000
3000
Time in days
PFI hsa−mir−3187
survival probability
0.0
0.4
0.8
P =0.94
0
1000
2000
3000
Time in days
DFI hsa−mir−3187
survival probability
0.0
0.4
0.8
P =0.66
0
1000
2000
3000
Time in days
DSS hsa−mir−3187
survival probability
0.0
0.4
0.8
P =0.87
0
1000
2000
3000
Time in days
OS hsa−mir−942
survival probability
0.0
0.4
0.8
P =0.99
0
500
1500
2500
Time in days
PFI hsa−mir−942
survival probability
0.0
0.4
0.8
P =0.7
0
500
1500
2500
Time in days

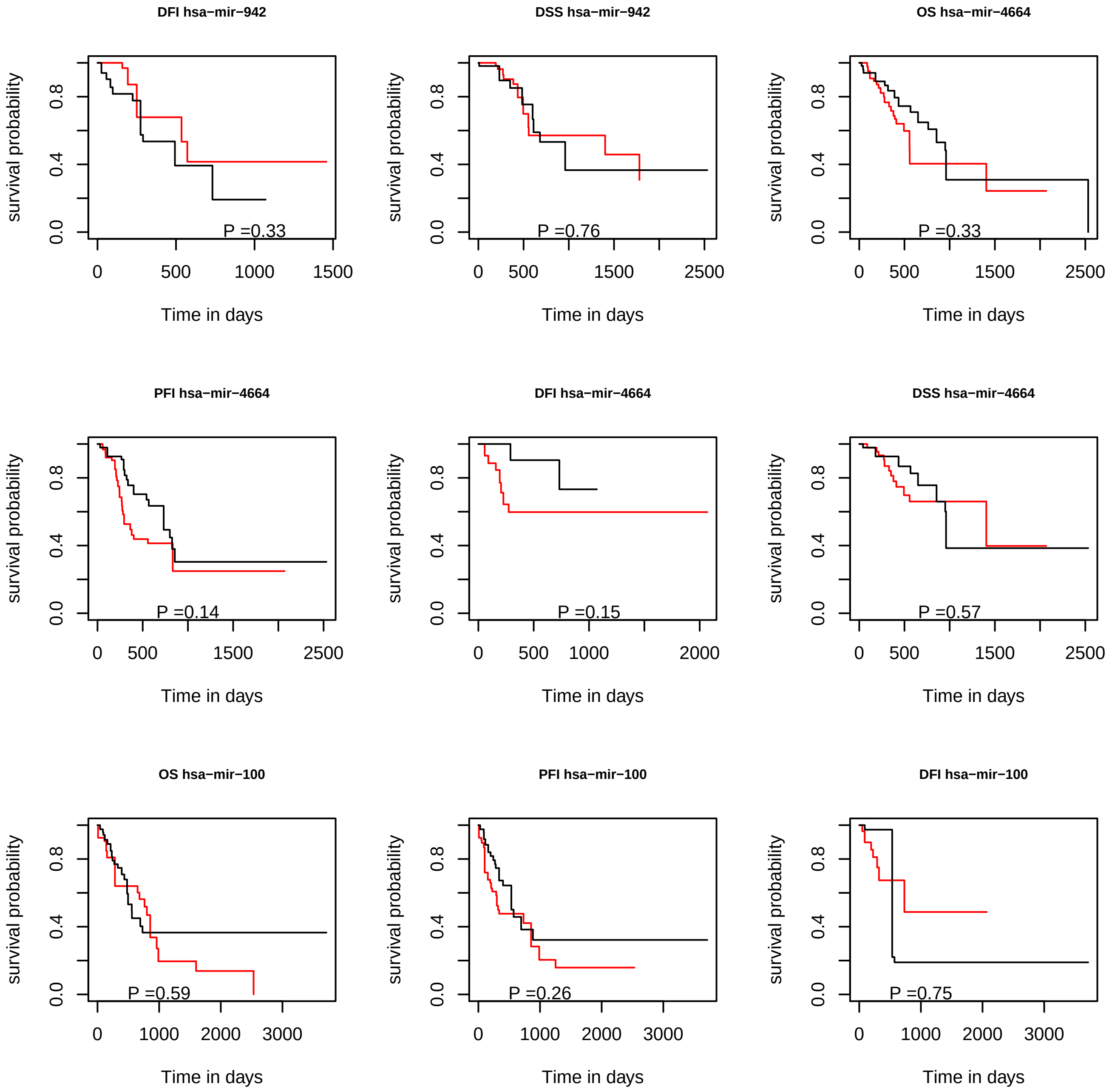

DFI hsa−mir−942
P =0.33
DSS hsa−mir−942
P =0.76
OS hsa−mir−4664
P =0.33
PFI hsa−mir−4664
P =0.14
DFI hsa−mir−4664
P =0.15
DSS hsa−mir−4664
P =0.57
OS hsa−mir−100
P =0.59
PFI hsa−mir−100
P =0.26
DFI hsa−mir−100
P =0.75
survival probability
0.0
0.4
0.8
Time in days
0
500
1000
1500
2000
2500
3000

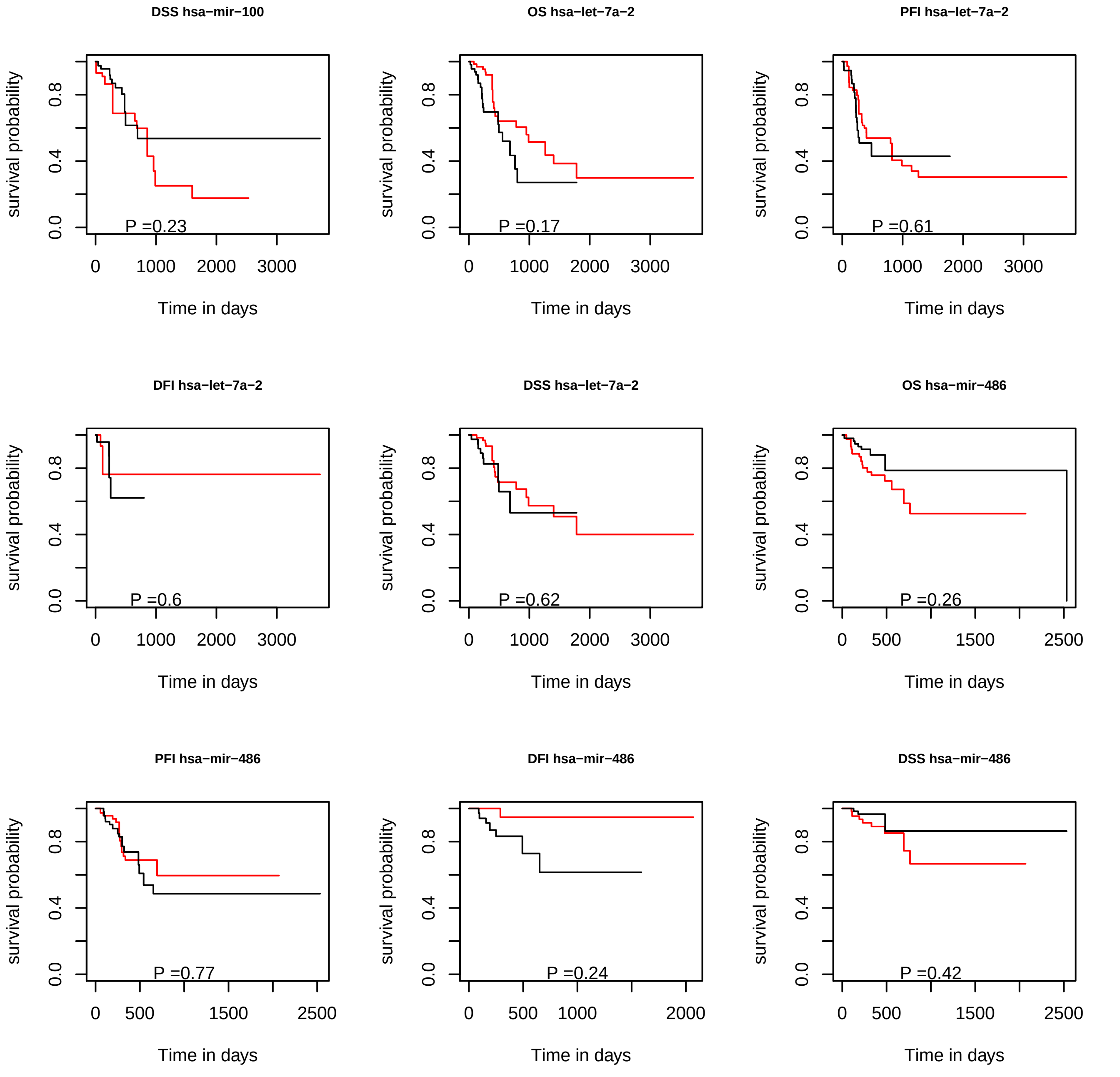
DSS hsa−mir−100
P =0.23
OS hsa−let−7a−2
P =0.17
PFI hsa−let−7a−2
P =0.61
DFI hsa−let−7a−2
P =0.6
DSS hsa−let−7a−2
P =0.62
OS hsa−mir−486
P =0.26
PFI hsa−mir−486
P =0.77
DFI hsa−mir−486
P =0.24
DSS hsa−mir−486
P =0.42
survival probability
Time in days
0.0
0.4
0.8
0
500
1000
1500
2000
2500
3000

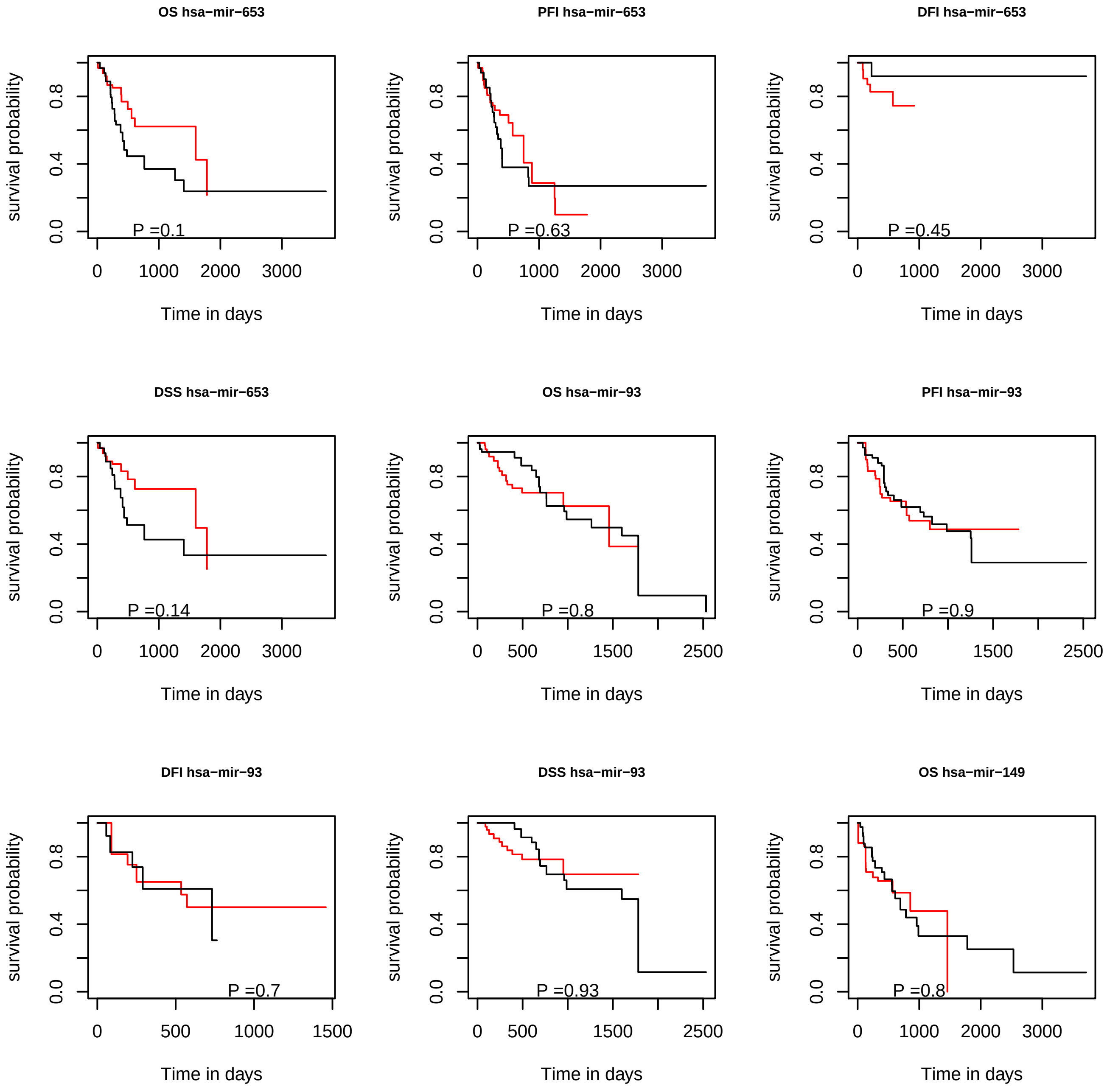
OS hsa−mir−653
PFI hsa−mir−653
DFI hsa−mir−653
DSS hsa−mir−653
OS hsa−mir−93
PFI hsa−mir−93
DFI hsa−mir−93
DSS hsa−mir−93
OS hsa−mir−149
P =0.1
P =0.63
P =0.45
P =0.14
P =0.8
P =0.9
P =0.7
P =0.93
P =0.8
survival probability
Time in days
0.0
0.4
0.8
0
500
1000
1500
2000
2500
3000

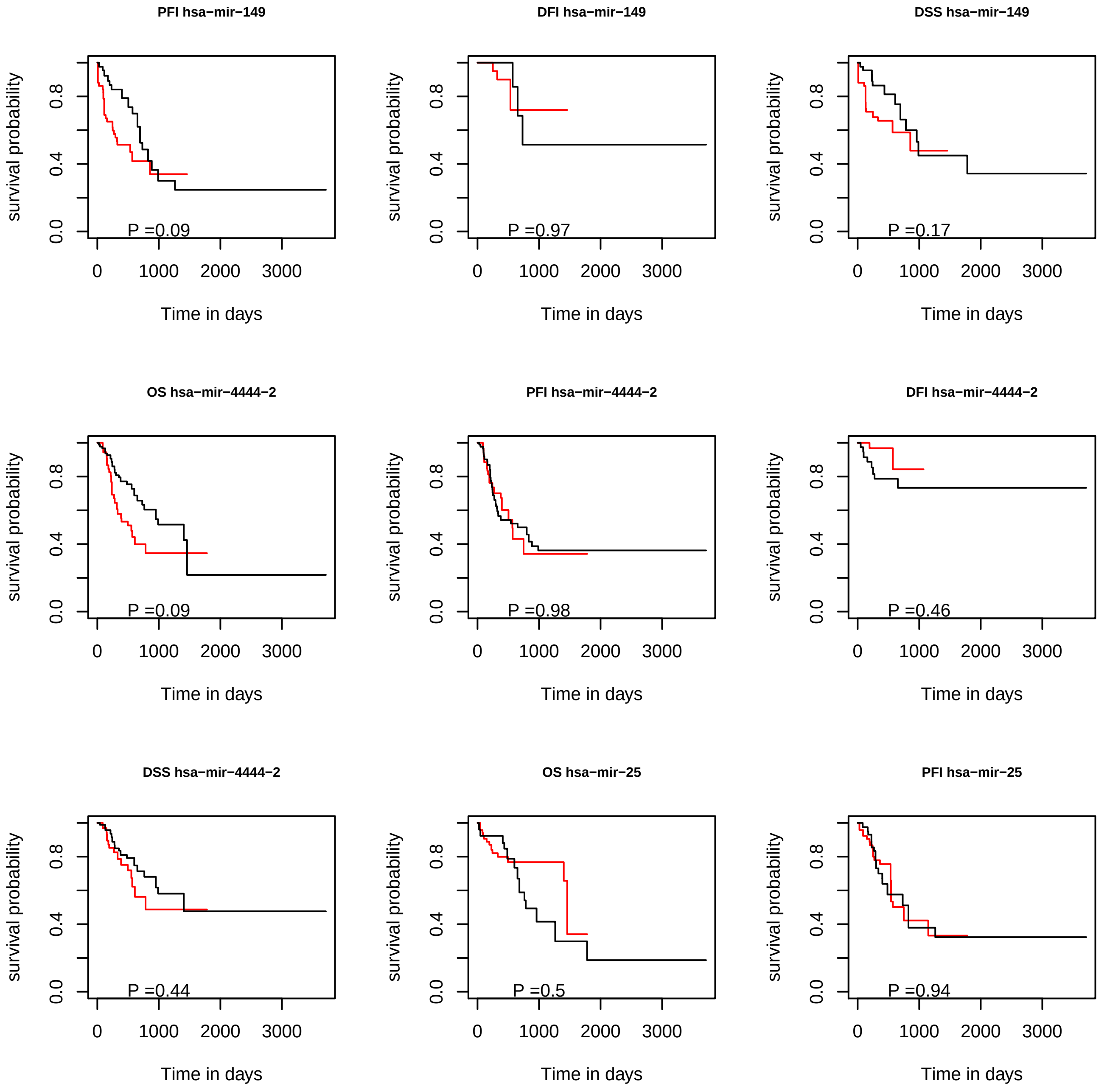
PFI hsa−mir−149
P =0.09
DFI hsa−mir−149
P =0.97
DSS hsa−mir−149
P =0.17
OS hsa−mir−4444−2
P =0.09
PFI hsa−mir−4444−2
P =0.98
DFI hsa−mir−4444−2
P =0.46
DSS hsa−mir−4444−2
P =0.44
OS hsa−mir−25
P =0.5
PFI hsa−mir−25
P =0.94
survival probability
0.0
0.4
0.8
Time in days
0
1000
2000
3000

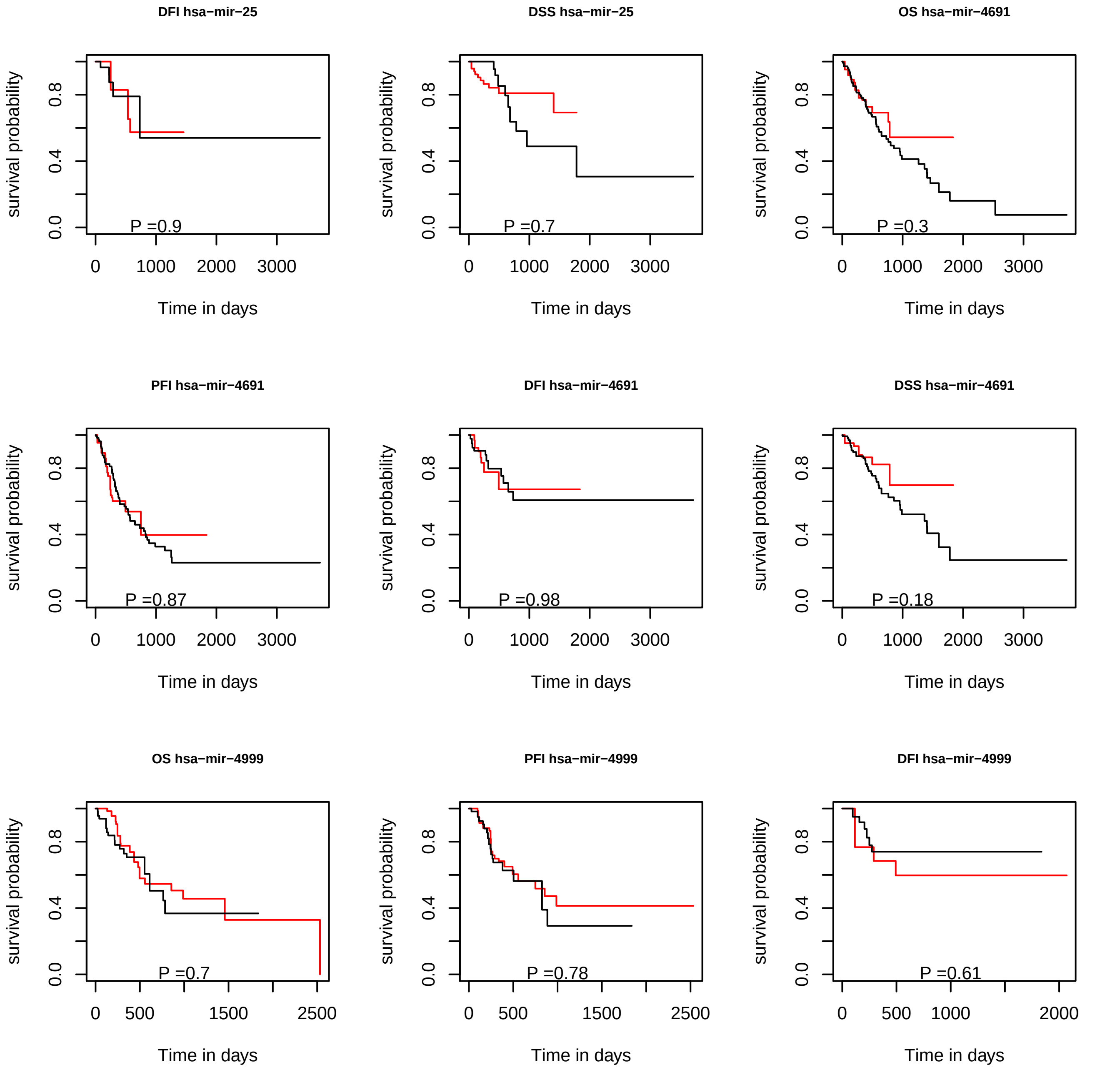
DFI hsa−mir−25
P =0.9
DSS hsa−mir−25
P =0.7
OS hsa−mir−4691
P =0.3
PFI hsa−mir−4691
P =0.87
DFI hsa−mir−4691
P =0.98
DSS hsa−mir−4691
P =0.18
OS hsa−mir−4999
P =0.7
PFI hsa−mir−4999
P =0.78
DFI hsa−mir−4999
P =0.61
survival probability
0.0
0.4
0.8
Time in days
0
500
1000
1500
2000
2500
3000

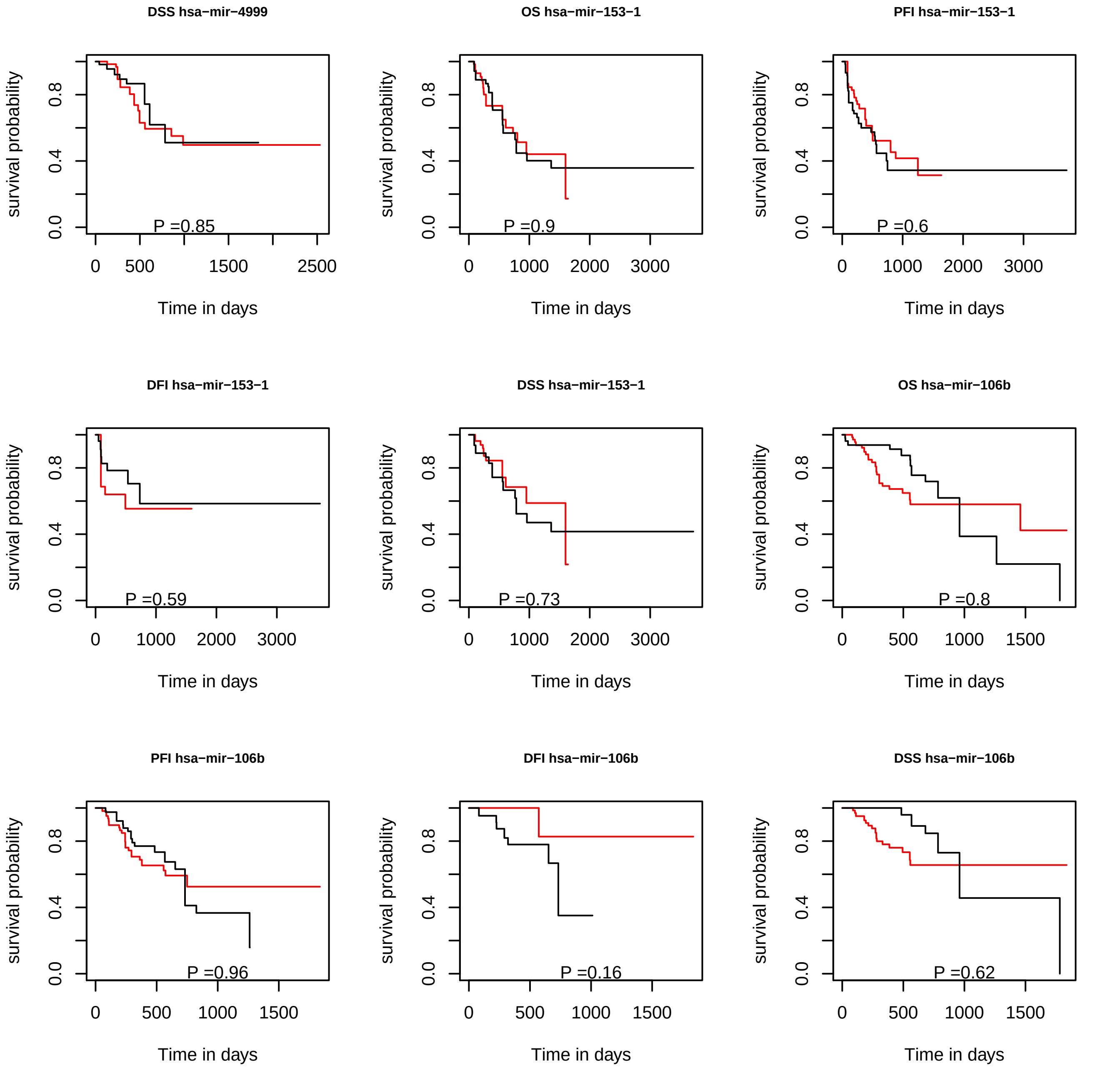
DSS hsa−mir−4999
P =0.85
OS hsa−mir−153−1
P =0.9
PFI hsa−mir−153−1
P =0.6
DFI hsa−mir−153−1
P =0.59
DSS hsa−mir−153−1
P =0.73
OS hsa−mir−106b
P =0.8
PFI hsa−mir−106b
P =0.96
DFI hsa−mir−106b
P =0.16
DSS hsa−mir−106b
P =0.62
survival probability
Time in days

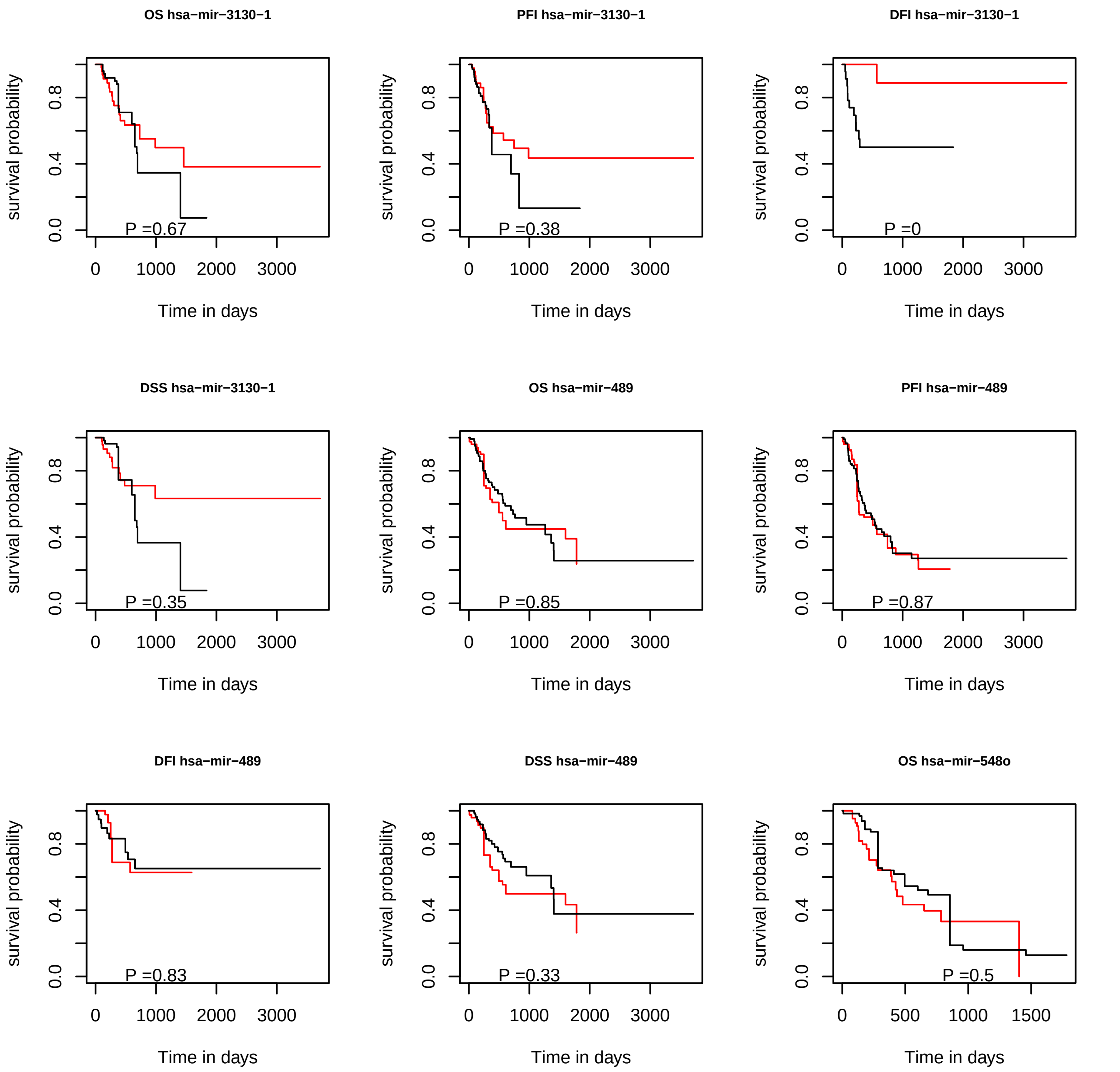
OS hsa−mir−3130−1
P =0.67
PFI hsa−mir−3130−1
P =0.38
DFI hsa−mir−3130−1
P =0
DSS hsa−mir−3130−1
P =0.35
OS hsa−mir−489
P =0.85
PFI hsa−mir−489
P =0.87
DFI hsa−mir−489
P =0.83
DSS hsa−mir−489
P =0.33
OS hsa−mir−548o
P =0.5
survival probability
Time in days

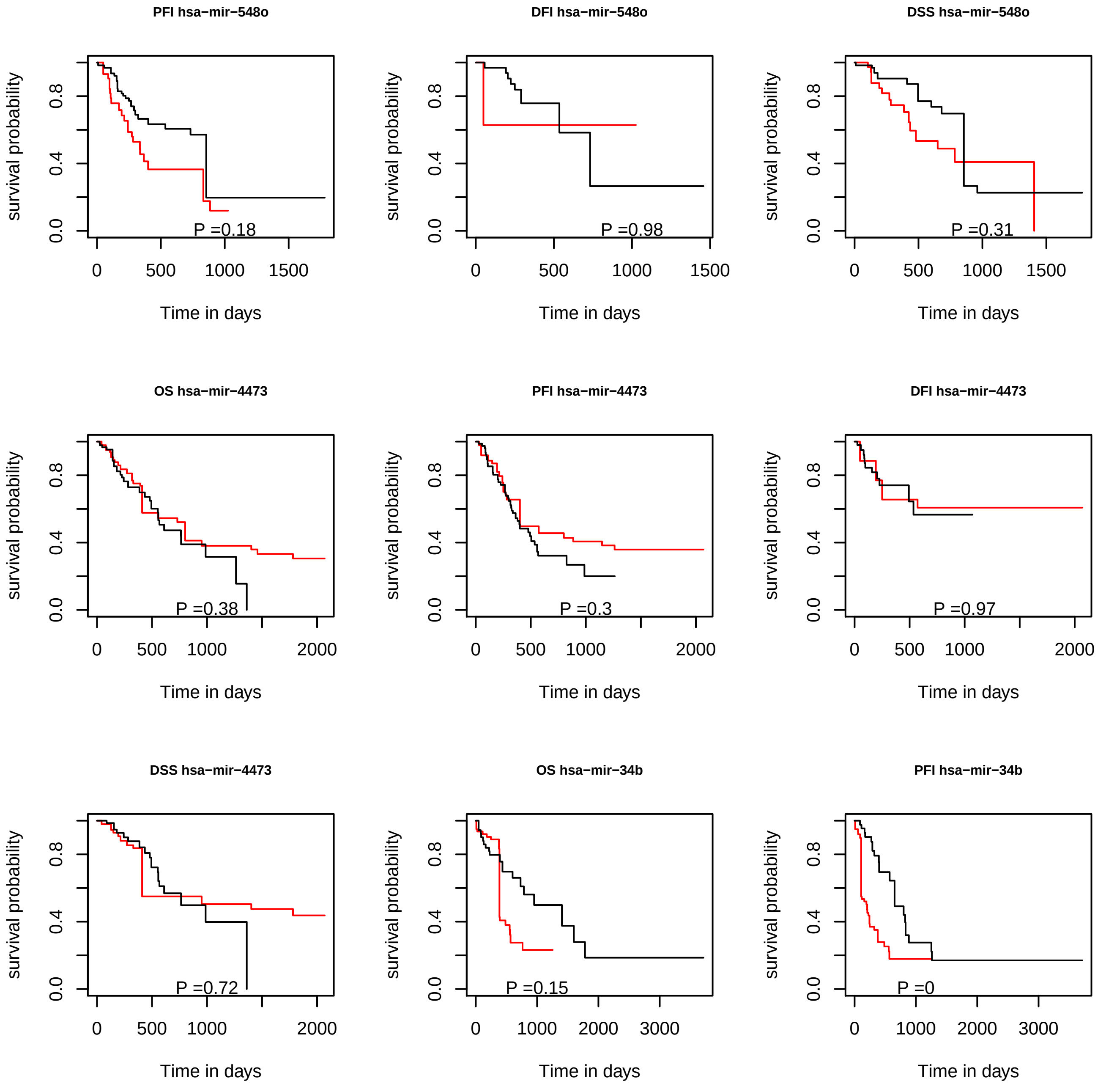
PFI hsa−mir−548o
survival probability
0.0
0.4
0.8
0
500
1000
1500
Time in days
P =0.18
DFI hsa−mir−548o
P =0.98
DSS hsa−mir−548o
P =0.31
OS hsa−mir−4473
2000
P =0.38
PFI hsa−mir−4473
P =0.3
DFI hsa−mir−4473
P =0.97
DSS hsa−mir−4473
P =0.72
OS hsa−mir−34b
3000
P =0.15
PFI hsa−mir−34b
P =0

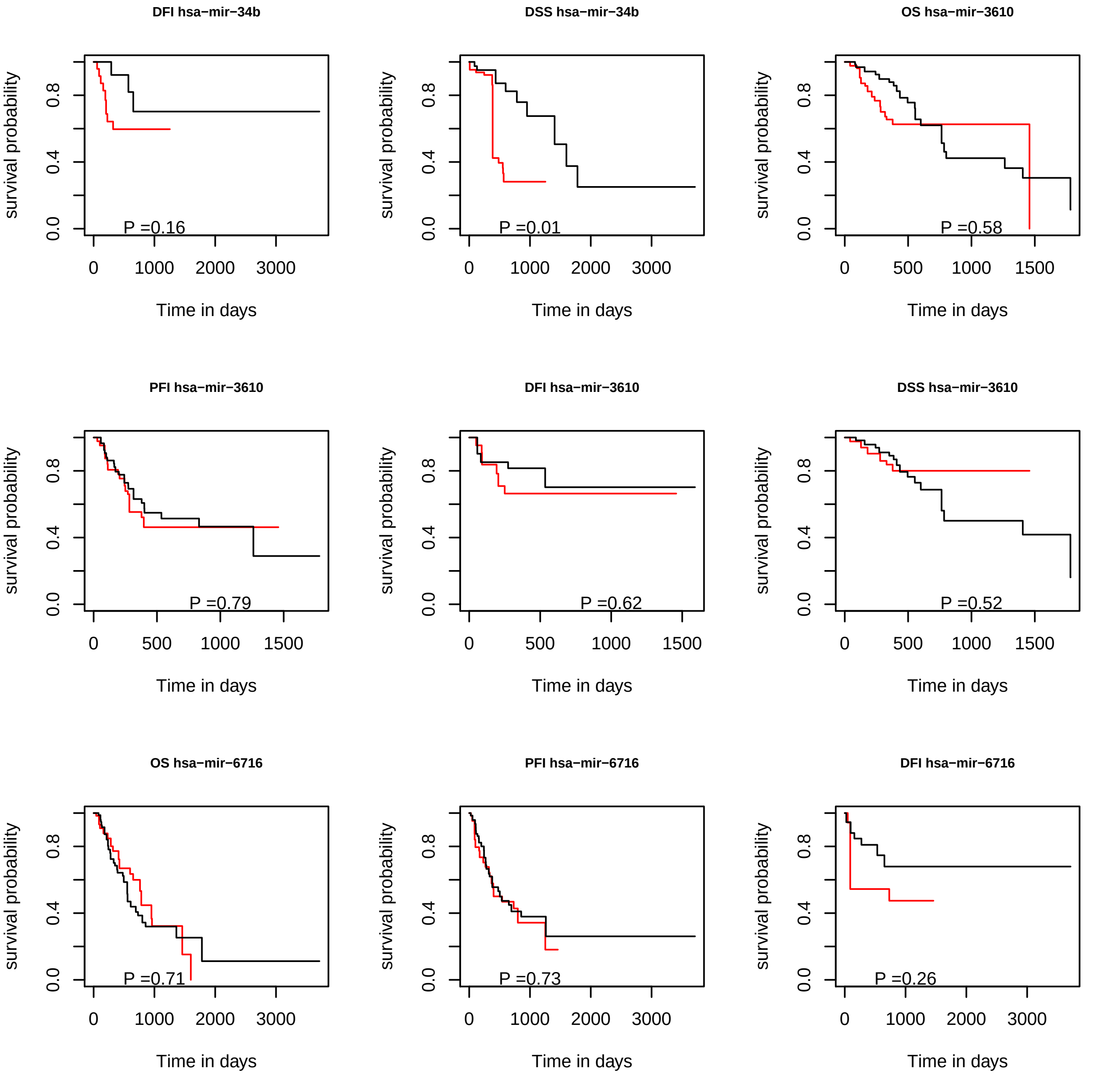
DFI hsa−mir−34b
P =0.16
DSS hsa−mir−34b
P =0.01
OS hsa−mir−3610
P =0.58
PFI hsa−mir−3610
P =0.79
DFI hsa−mir−3610
P =0.62
DSS hsa−mir−3610
P =0.52
OS hsa−mir−6716
P =0.71
PFI hsa−mir−6716
P =0.73
DFI hsa−mir−6716
P =0.26
survival probability
Time in days
0.0
0.4
0.8
0
500
1000
1500
2000
3000

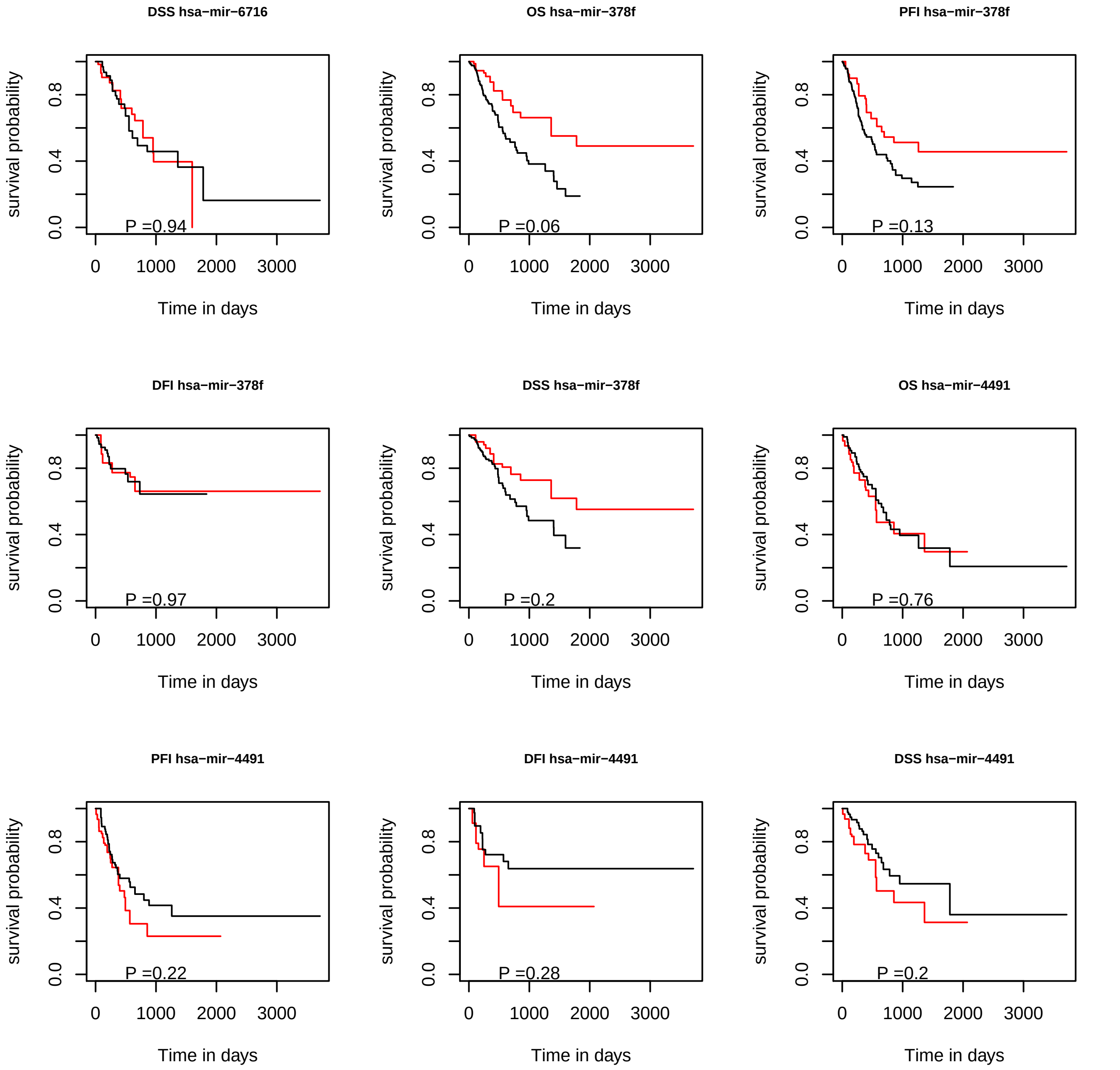

DSS hsa−mir−6716
P =0.94
OS hsa−mir−378f
P =0.06
PFI hsa−mir−378f
P =0.13
DFI hsa−mir−378f
P =0.97
DSS hsa−mir−378f
P =0.2
OS hsa−mir−4491
P =0.76
PFI hsa−mir−4491
P =0.22
DFI hsa−mir−4491
P =0.28
DSS hsa−mir−4491
P =0.2
survival probability
0.0
0.4
0.8
Time in days
0
1000
2000
3000

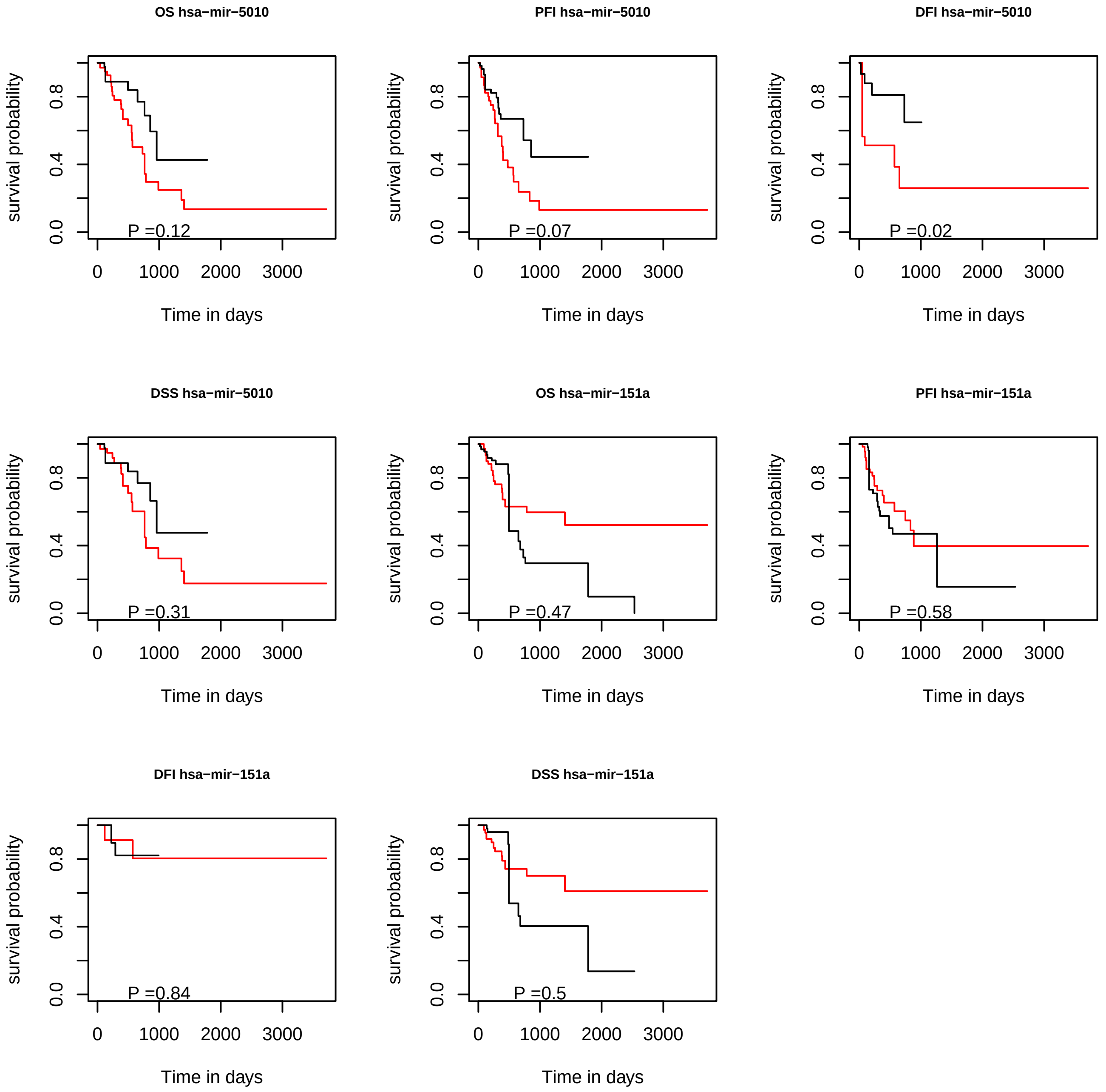
OS hsa−mir−5010
survival probability
0.0
0.4
0.8
P =0.12
0
1000
2000
3000
Time in days
PFI hsa−mir−5010
survival probability
0.0
0.4
0.8
P =0.07
0
1000
2000
3000
Time in days
DFI hsa−mir−5010
survival probability
0.0
0.4
0.8
P =0.02
0
1000
2000
3000
Time in days
DSS hsa−mir−5010
survival probability
0.0
0.4
0.8
P =0.31
0
1000
2000
3000
Time in days
OS hsa−mir−151a
survival probability
0.0
0.4
0.8
P =0.47
0
1000
2000
3000
Time in days
PFI hsa−mir−151a
survival probability
0.0
0.4
0.8
P =0.58
0
1000
2000
3000
Time in days
DFI hsa−mir−151a
survival probability
0.0
0.4
0.8
P =0.84
0
1000
2000
3000
Time in days
DSS hsa−mir−151a
survival probability
0.0
0.4
0.8
P =0.5
0
1000
2000
3000
Time in days
