# Supplementary material for: Computing microRNA-gene interaction networks in pan-cancer using miRDriver
Source: Sci Rep. 2022 Mar 8;12:3717. doi: 10.1038/s41598-022-07628-z (PMC8904490; doi:10.1038/s41598-022-07628-z)
Supplement: Supplementary file 16 — Supplementary Information 16. [file 41598_2022_7628_MOESM16_ESM.pdf]

Computing microRNA-gene interaction networks in pan-cancer using miRDriver

Banabithi Bose, Matthew Moravec, and Serdar Bozdag

Supplemental Figure S7

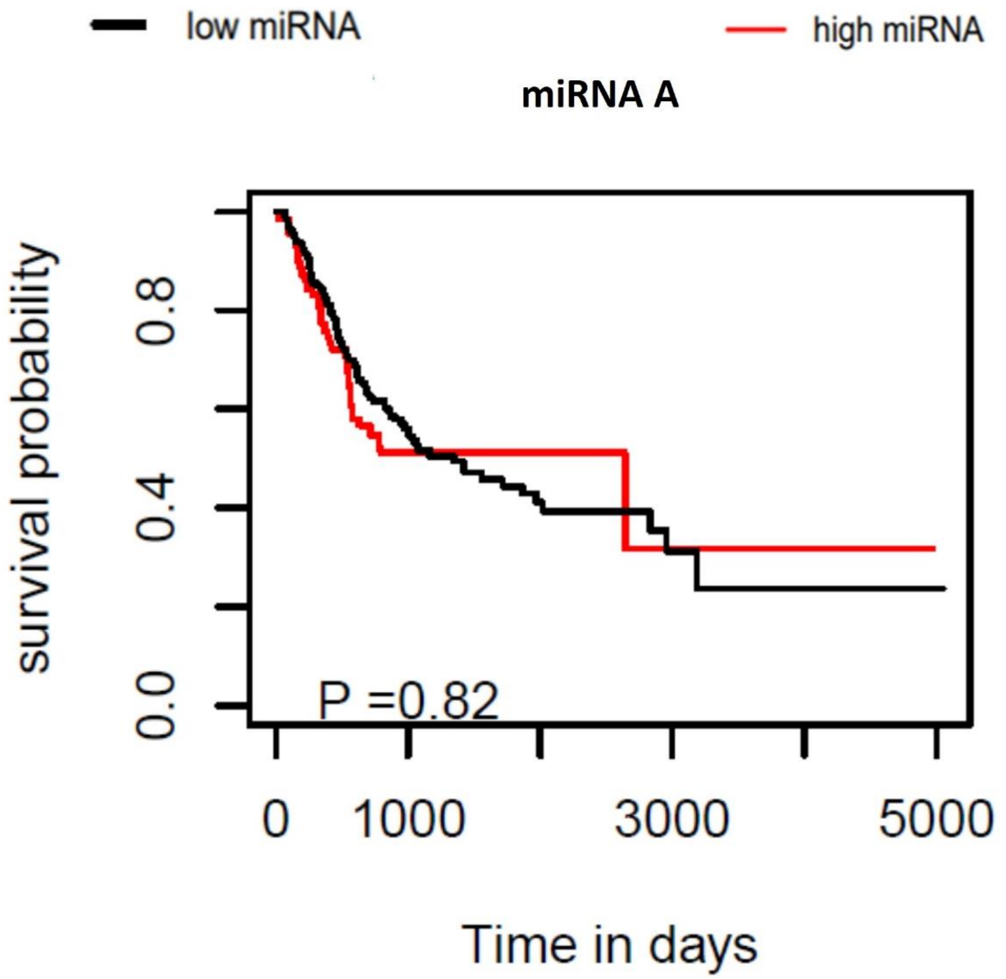

The *Adjusted Kaplan-Meier* survival plots for the computed miRNAs in high and low miRNA expression patient groups.

Supplemental Figure S7 Cancer Type: HNSC

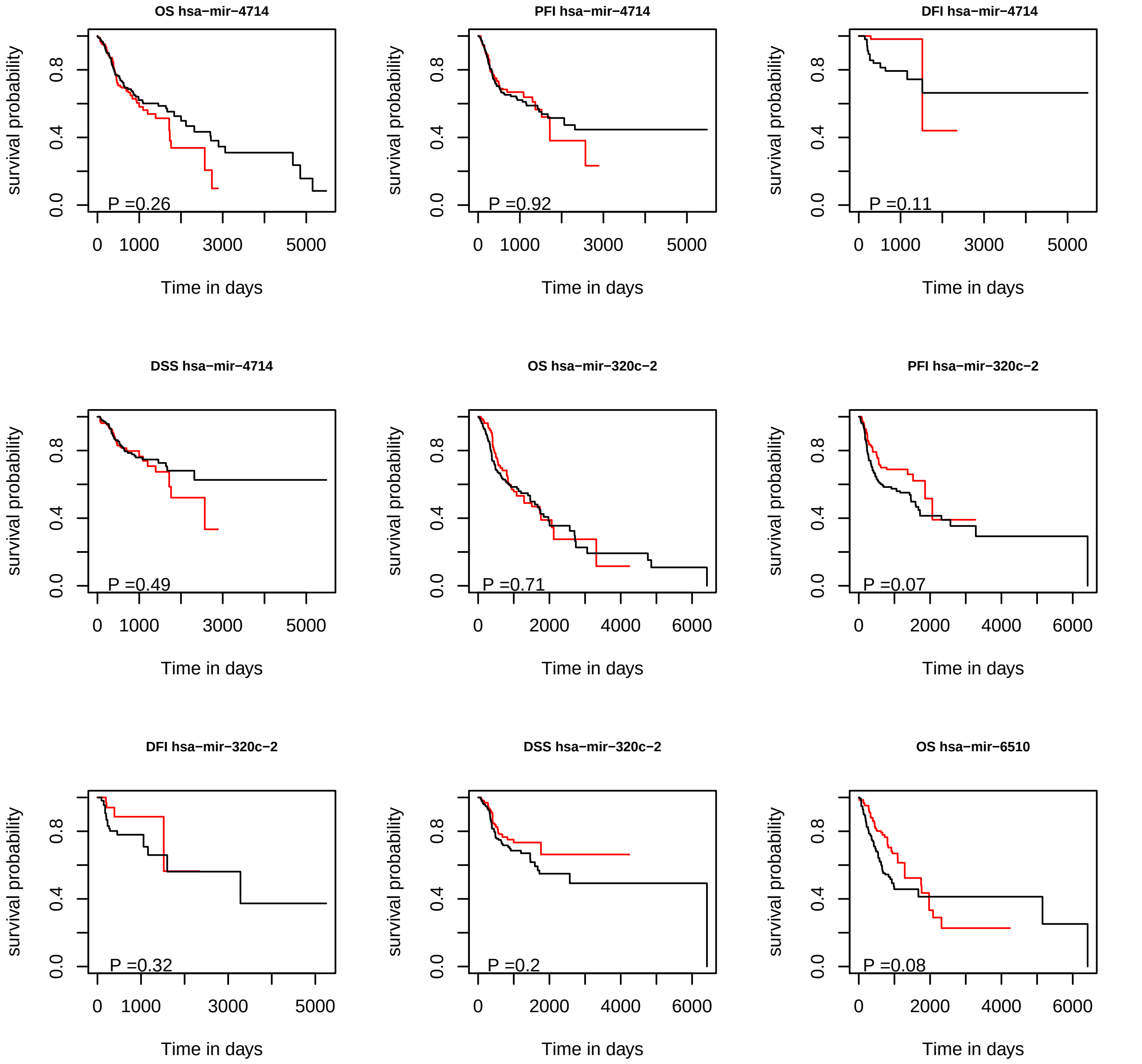

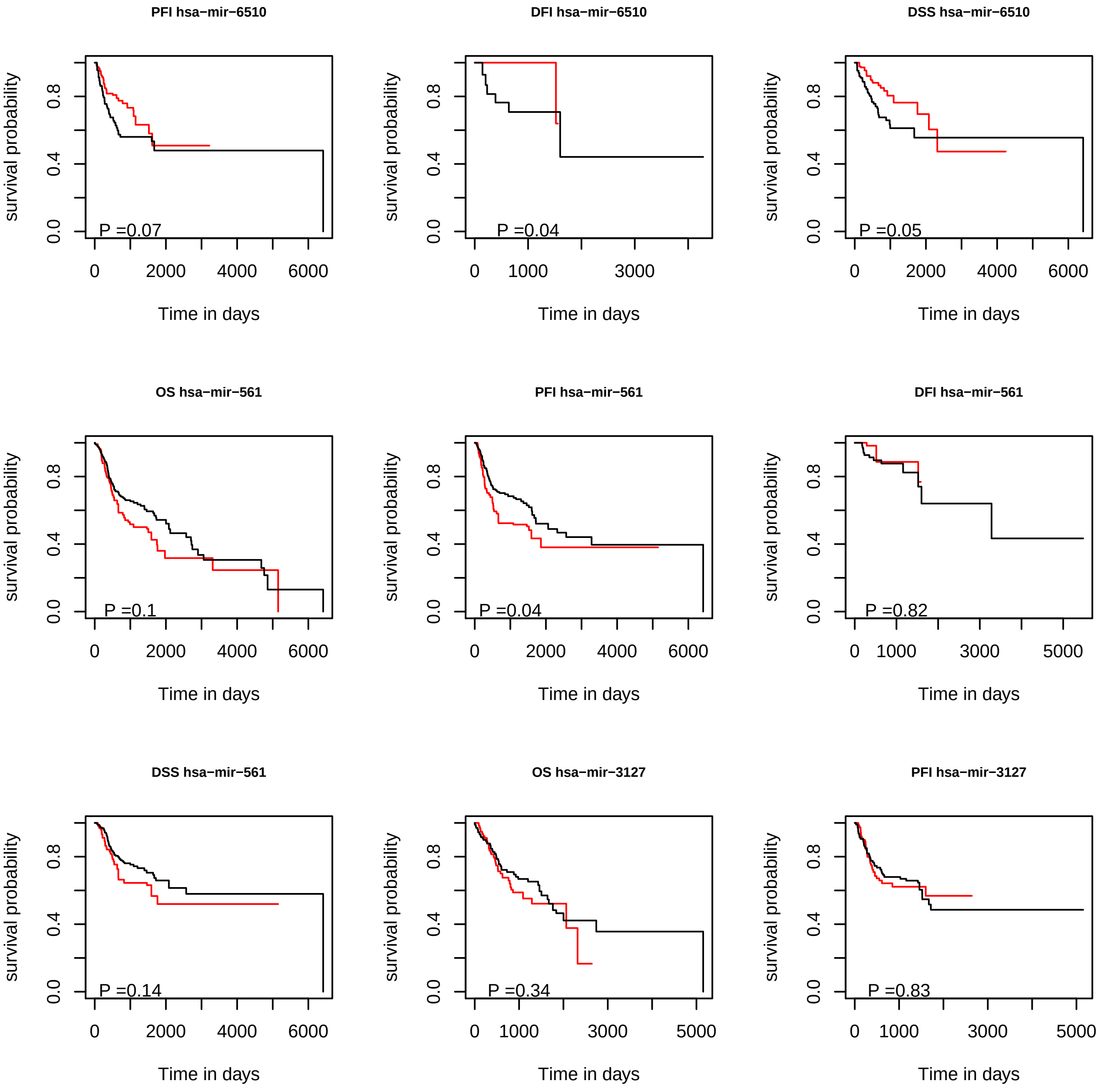
PFI hsa−mir−6510
survival probability
0.0
0.4
0.8
P =0.07
0
2000
4000
6000
Time in days
DFI hsa−mir−6510
survival probability
0.0
0.4
0.8
P =0.04
0
1000
3000
Time in days
DSS hsa−mir−6510
survival probability
0.0
0.4
0.8
P =0.05
0
2000
4000
6000
Time in days
OS hsa−mir−561
survival probability
0.0
0.4
0.8
P =0.1
0
2000
4000
6000
Time in days
PFI hsa−mir−561
survival probability
0.0
0.4
0.8
P =0.04
0
2000
4000
6000
Time in days
DFI hsa−mir−561
survival probability
0.0
0.4
0.8
P =0.82
0
1000
3000
5000
Time in days
DSS hsa−mir−561
survival probability
0.0
0.4
0.8
P =0.14
0
2000
4000
6000
Time in days
OS hsa−mir−3127
survival probability
0.0
0.4
0.8
P =0.34
0
1000
3000
5000
Time in days
PFI hsa−mir−3127
survival probability
0.0
0.4
0.8
P =0.83
0
1000
3000
5000
Time in days

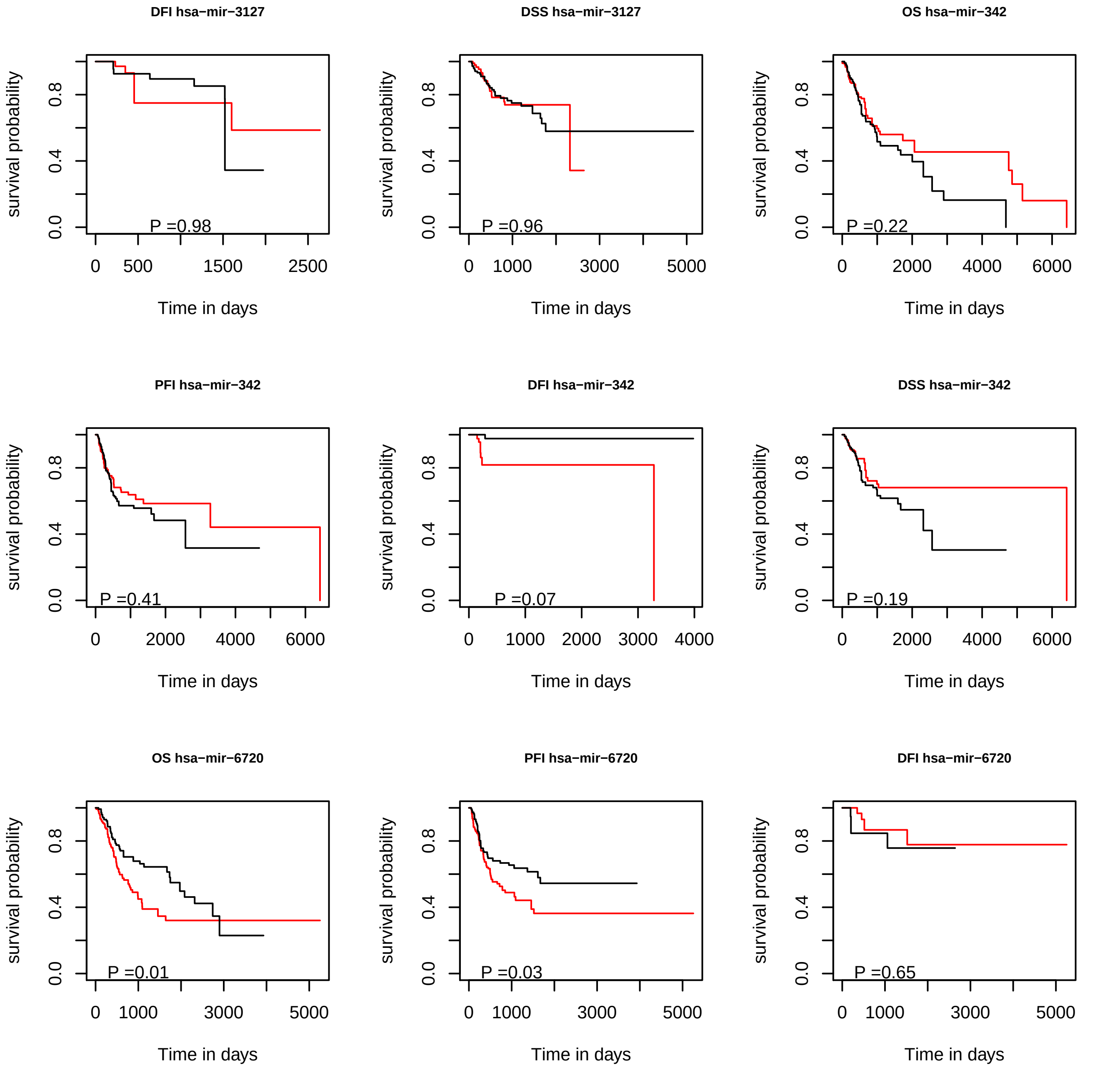
DFI hsa−mir−3127
P =0.98
DSS hsa−mir−3127
P =0.96
OS hsa−mir−342
P =0.22
PFI hsa−mir−342
P =0.41
DFI hsa−mir−342
P =0.07
DSS hsa−mir−342
P =0.19
OS hsa−mir−6720
P =0.01
PFI hsa−mir−6720
P =0.03
DFI hsa−mir−6720
P =0.65
survival probability
Time in days

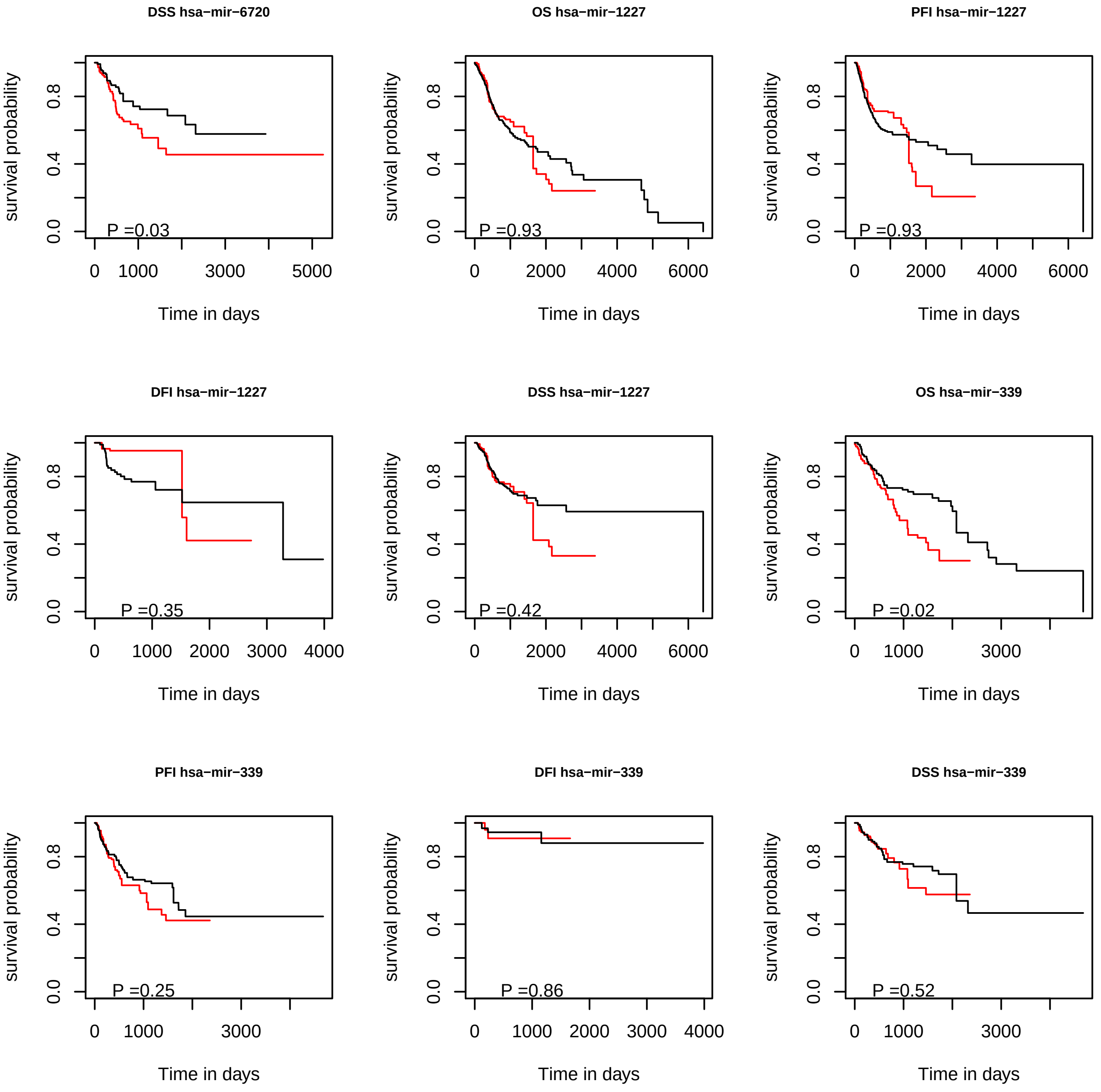

DSS hsa−mir−6720
P =0.03
OS hsa−mir−1227
P =0.93
PFI hsa−mir−1227
P =0.93
DFI hsa−mir−1227
P =0.35
DSS hsa−mir−1227
P =0.42
OS hsa−mir−339
P =0.02
PFI hsa−mir−339
P =0.25
DFI hsa−mir−339
P =0.86
DSS hsa−mir−339
P =0.52
survival probability
Time in days
0.0
0.4
0.8
0
1000
2000
3000
4000
5000
6000

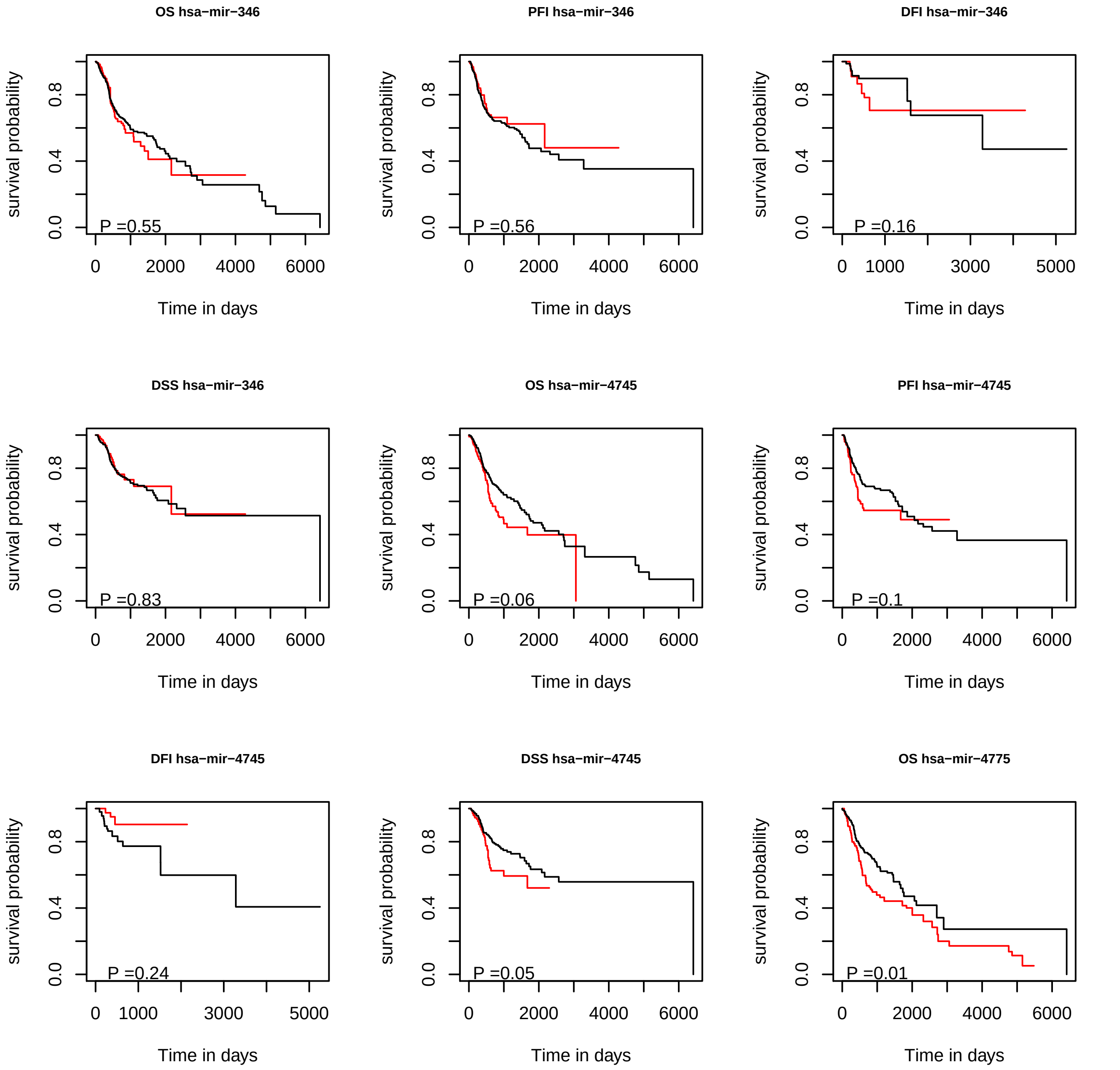
OS hsa−mir−346
PFI hsa−mir−346
DFI hsa−mir−346
DSS hsa−mir−346
OS hsa−mir−4745
PFI hsa−mir−4745
DFI hsa−mir−4745
DSS hsa−mir−4745
OS hsa−mir−4775
P =0.55
P =0.56
P =0.16
P =0.83
P =0.06
P =0.1
P =0.24
P =0.05
P =0.01
survival probability
0.0
0.4
0.8
Time in days
0
1000
2000
3000
4000
5000
6000

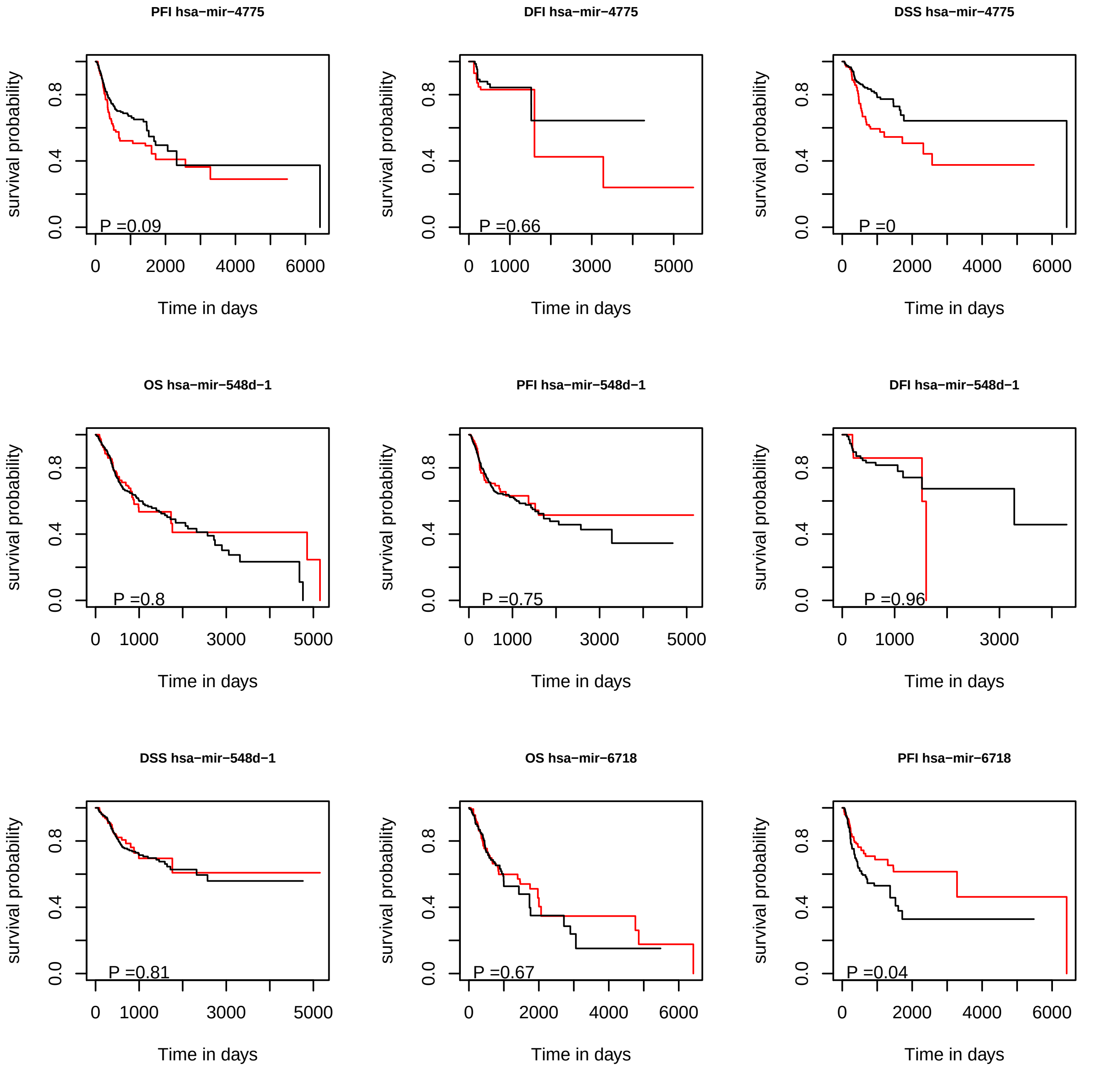
PFI hsa−mir−4775
P =0.09
DFI hsa−mir−4775
P =0.66
DSS hsa−mir−4775
P =0
OS hsa−mir−548d−1
P =0.8
PFI hsa−mir−548d−1
P =0.75
DFI hsa−mir−548d−1
P =0.96
DSS hsa−mir−548d−1
P =0.81
OS hsa−mir−6718
P =0.67
PFI hsa−mir−6718
P =0.04
survival probability
Time in days

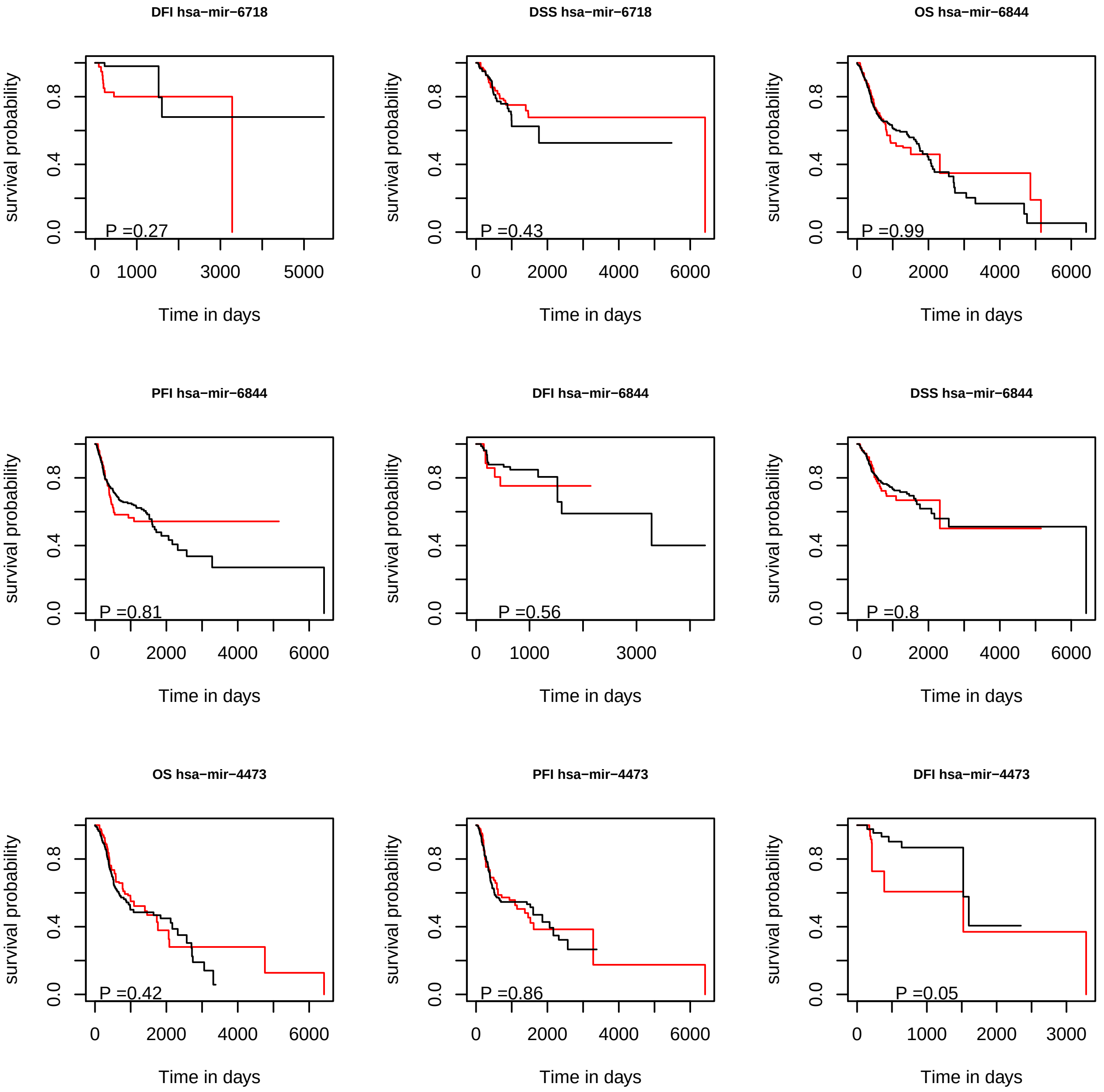
DFI hsa−mir−6718
P =0.27
DSS hsa−mir−6718
P =0.43
OS hsa−mir−6844
P =0.99
PFI hsa−mir−6844
P =0.81
DFI hsa−mir−6844
P =0.56
DSS hsa−mir−6844
P =0.8
OS hsa−mir−4473
P =0.42
PFI hsa−mir−4473
P =0.86
DFI hsa−mir−4473
P =0.05
survival probability
Time in days

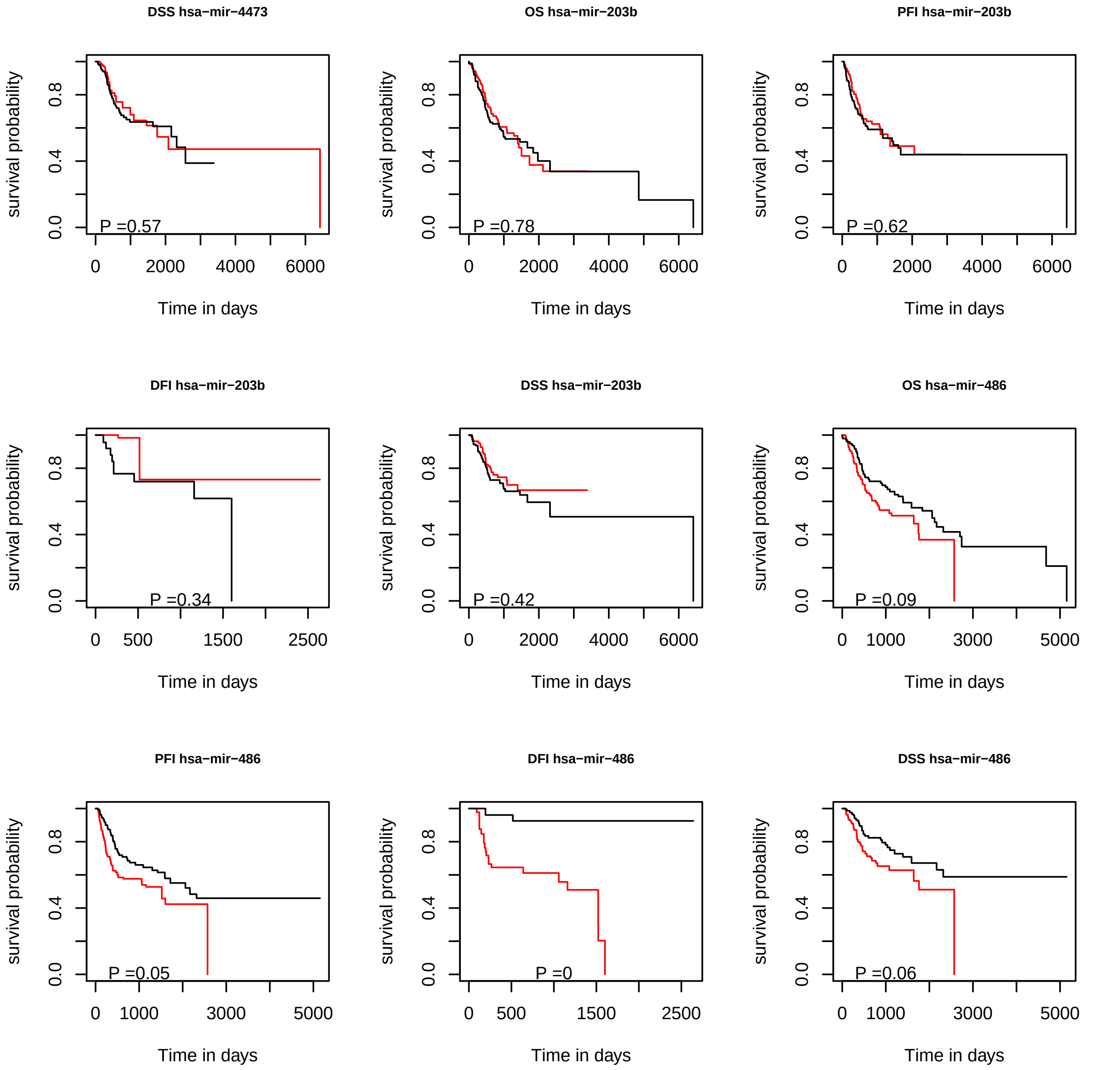
DSS hsa−mir−4473
P =0.57
OS hsa−mir−203b
P =0.78
PFI hsa−mir−203b
P =0.62
DFI hsa−mir−203b
P =0.34
DSS hsa−mir−203b
P =0.42
OS hsa−mir−486
P =0.09
PFI hsa−mir−486
P =0.05
DFI hsa−mir−486
P =0
DSS hsa−mir−486
P =0.06
survival probability
Time in days

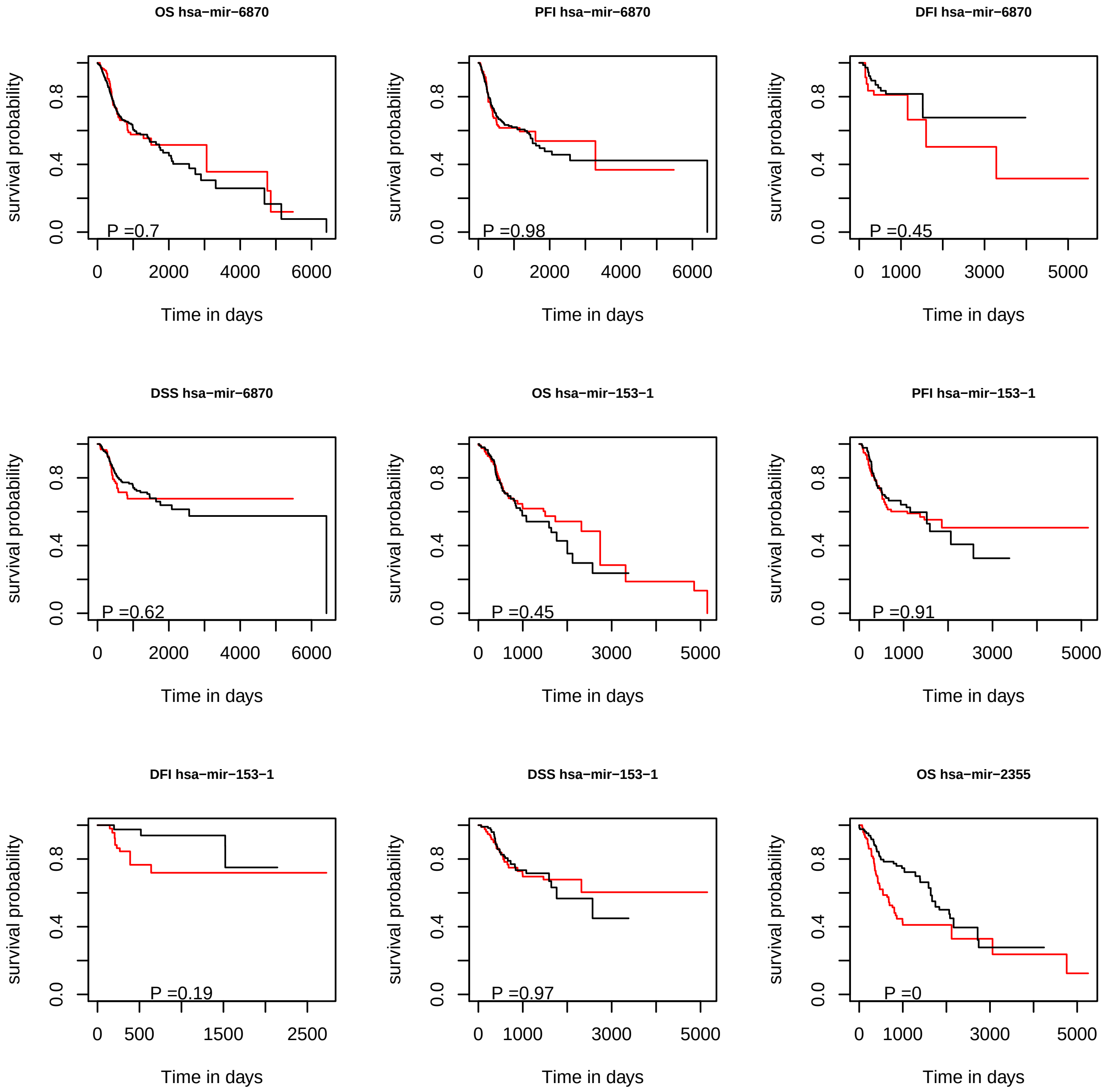
OS hsa−mir−6870
P =0.7
PFI hsa−mir−6870
P =0.98
DFI hsa−mir−6870
P =0.45
DSS hsa−mir−6870
P =0.62
OS hsa−mir−153−1
P =0.45
PFI hsa−mir−153−1
P =0.91
DFI hsa−mir−153−1
P =0.19
DSS hsa−mir−153−1
P =0.97
OS hsa−mir−2355
P =0
survival probability
Time in days

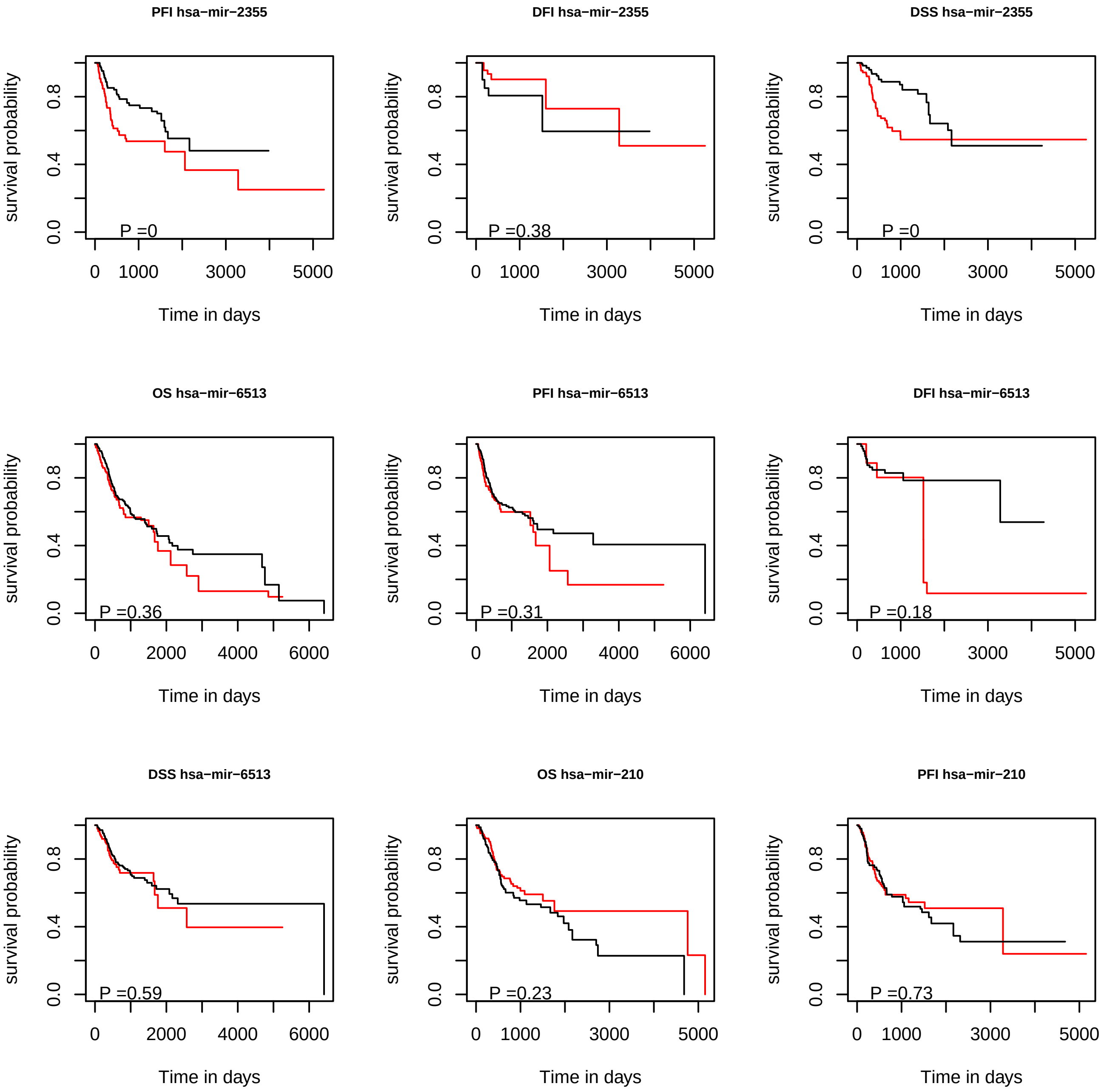
PFI hsa−mir−2355
P =0
DFI hsa−mir−2355
P =0.38
DSS hsa−mir−2355
P =0
OS hsa−mir−6513
P =0.36
PFI hsa−mir−6513
P =0.31
DFI hsa−mir−6513
P =0.18
DSS hsa−mir−6513
P =0.59
OS hsa−mir−210
P =0.23
PFI hsa−mir−210
P =0.73
survival probability
Time in days
0.0
0.4
0.8
0
1000
2000
3000
4000
5000
6000

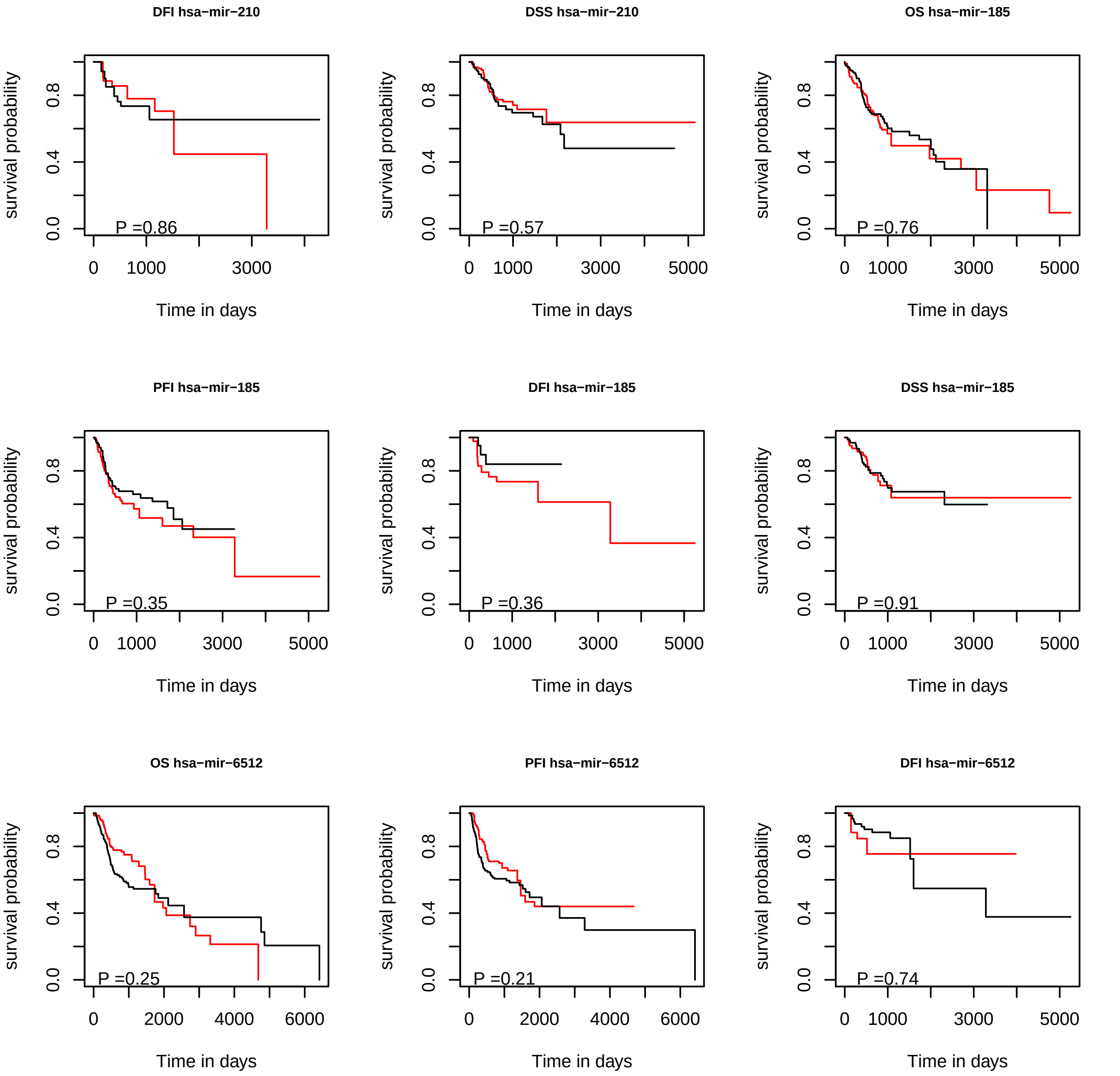
DFI hsa−mir−210
P =0.86
DSS hsa−mir−210
P =0.57
OS hsa−mir−185
P =0.76
PFI hsa−mir−185
P =0.35
DFI hsa−mir−185
P =0.36
DSS hsa−mir−185
P =0.91
OS hsa−mir−6512
P =0.25
PFI hsa−mir−6512
P =0.21
DFI hsa−mir−6512
P =0.74
survival probability
Time in days
0.0
0.4
0.8
0
1000
2000
3000
4000
5000
6000

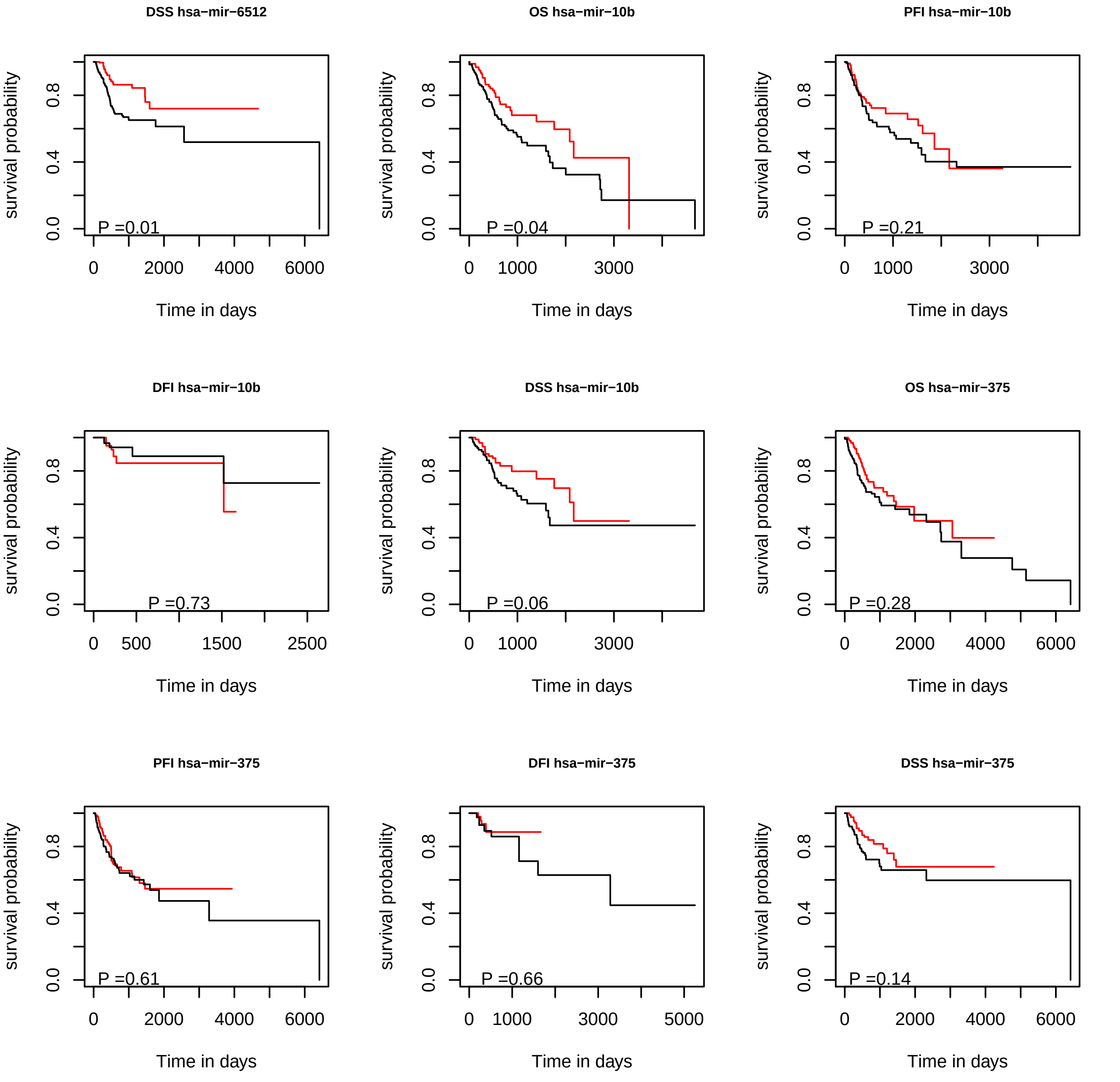
DSS hsa−mir−6512
P =0.01
OS hsa−mir−10b
P =0.04
PFI hsa−mir−10b
P =0.21
DFI hsa−mir−10b
P =0.73
DSS hsa−mir−10b
P =0.06
OS hsa−mir−375
P =0.28
PFI hsa−mir−375
P =0.61
DFI hsa−mir−375
P =0.66
DSS hsa−mir−375
P =0.14
survival probability
Time in days

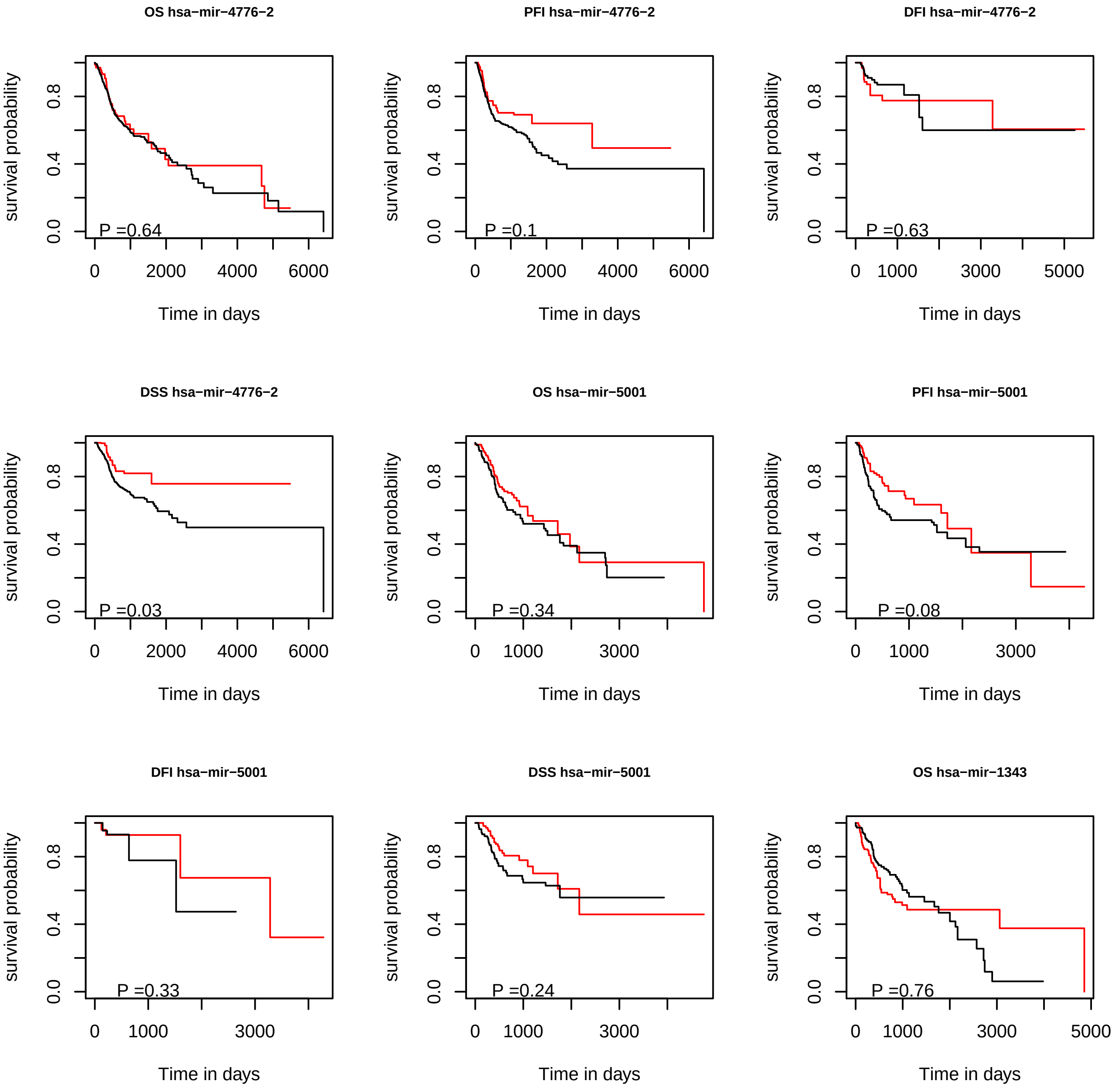
OS hsa−mir−4776−2
survival probability
0.0
0.4
0.8
P =0.64
0
2000
4000
6000
Time in days
PFI hsa−mir−4776−2
survival probability
P =0.1
0
2000
4000
6000
Time in days
DFI hsa−mir−4776−2
survival probability
P =0.63
0
1000
3000
5000
Time in days
DSS hsa−mir−4776−2
survival probability
P =0.03
0
2000
4000
6000
Time in days
OS hsa−mir−5001
survival probability
P =0.34
0
1000
3000
Time in days
PFI hsa−mir−5001
survival probability
P =0.08
0
1000
3000
Time in days
DFI hsa−mir−5001
survival probability
P =0.33
0
1000
3000
Time in days
DSS hsa−mir−5001
survival probability
P =0.24
0
1000
3000
Time in days
OS hsa−mir−1343
survival probability
P =0.76
0
1000
3000
5000
Time in days

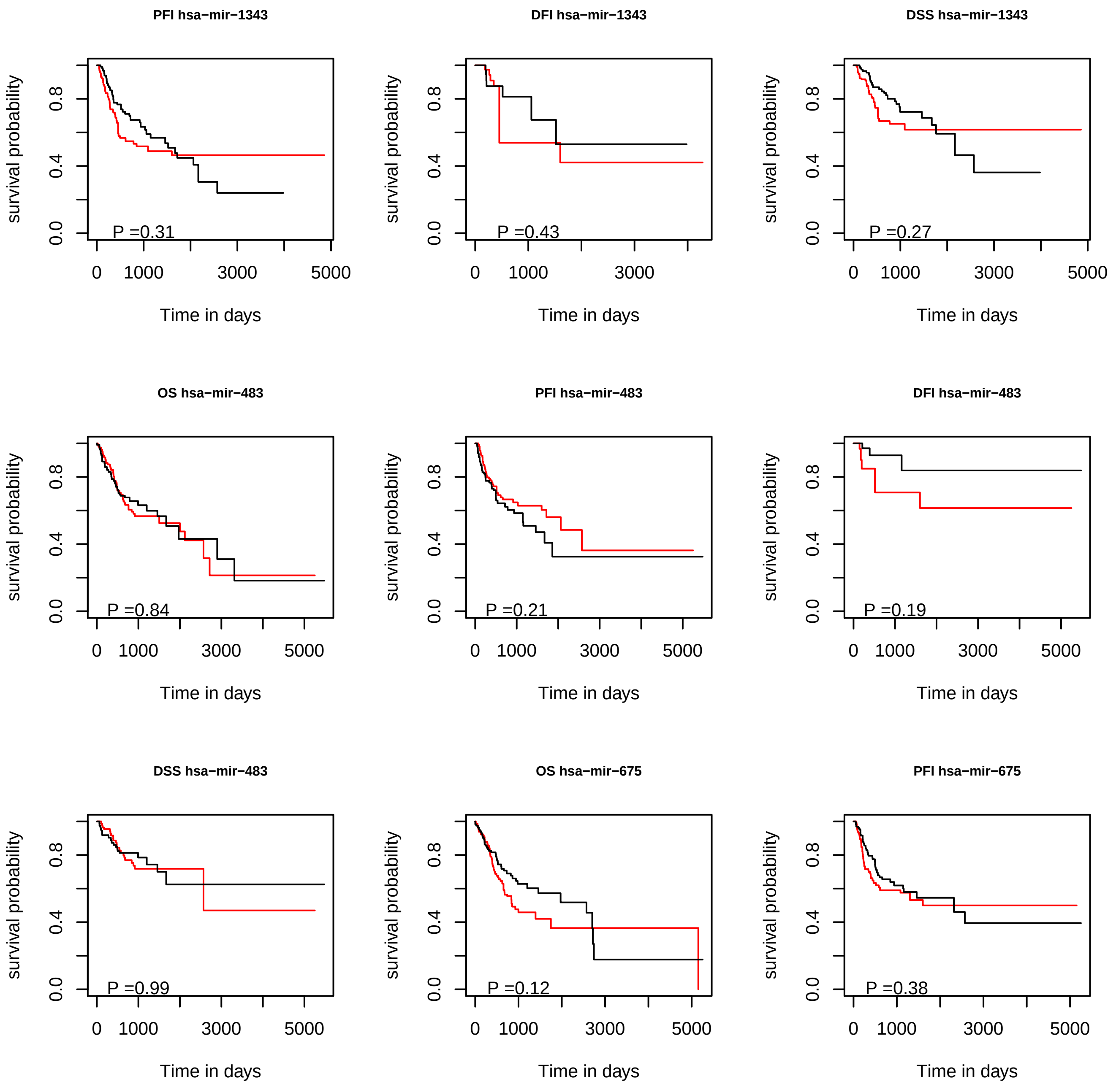
PFI hsa−mir−1343
P =0.31
DFI hsa−mir−1343
P =0.43
DSS hsa−mir−1343
P =0.27
OS hsa−mir−483
P =0.84
PFI hsa−mir−483
P =0.21
DFI hsa−mir−483
P =0.19
DSS hsa−mir−483
P =0.99
OS hsa−mir−675
P =0.12
PFI hsa−mir−675
P =0.38
survival probability
Time in days
0.0
0.4
0.8
0
1000
3000
5000

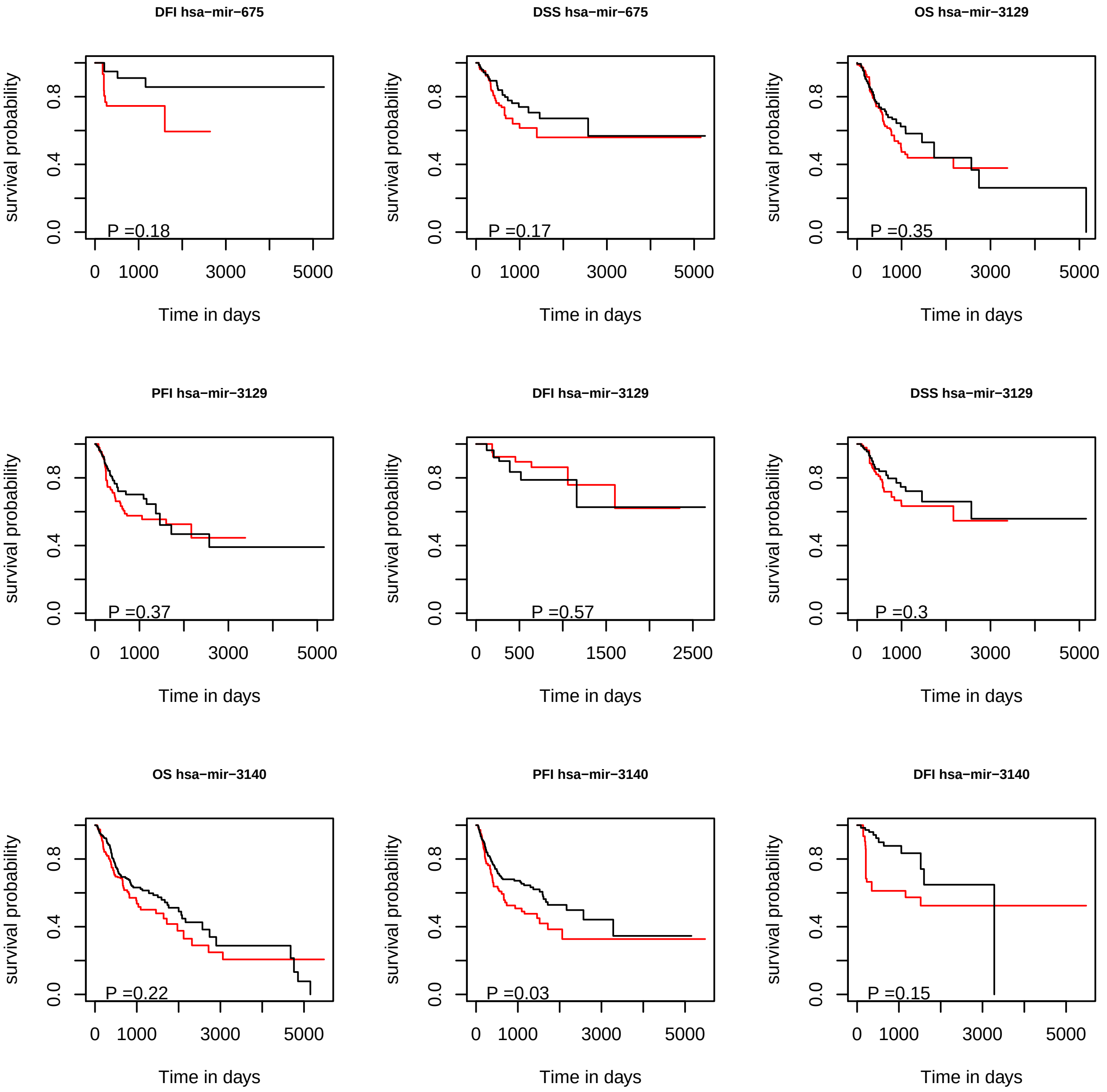
DFI hsa−mir−675
P =0.18
DSS hsa−mir−675
P =0.17
OS hsa−mir−3129
P =0.35
PFI hsa−mir−3129
P =0.37
DFI hsa−mir−3129
P =0.57
DSS hsa−mir−3129
P =0.3
OS hsa−mir−3140
P =0.22
PFI hsa−mir−3140
P =0.03
DFI hsa−mir−3140
P =0.15
survival probability
0.0
0.4
0.8
Time in days
0
500
1000
1500
2500
3000
5000

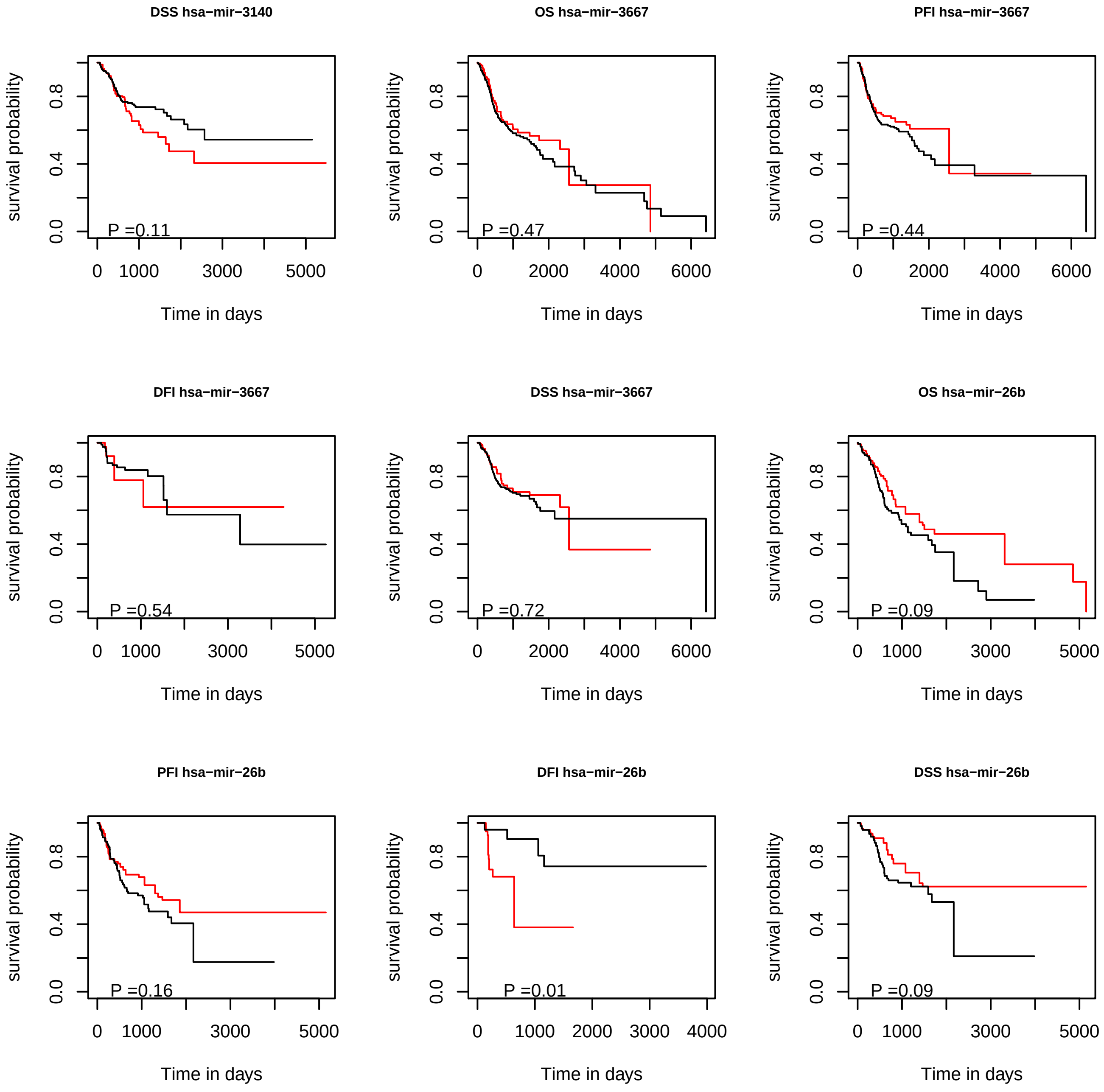
DSS hsa−mir−3140
P =0.11
OS hsa−mir−3667
P =0.47
PFI hsa−mir−3667
P =0.44
DFI hsa−mir−3667
P =0.54
DSS hsa−mir−3667
P =0.72
OS hsa−mir−26b
P =0.09
PFI hsa−mir−26b
P =0.16
DFI hsa−mir−26b
P =0.01
DSS hsa−mir−26b
P =0.09
survival probability
0.0
0.4
0.8
Time in days
0
1000
2000
3000
4000
5000
6000

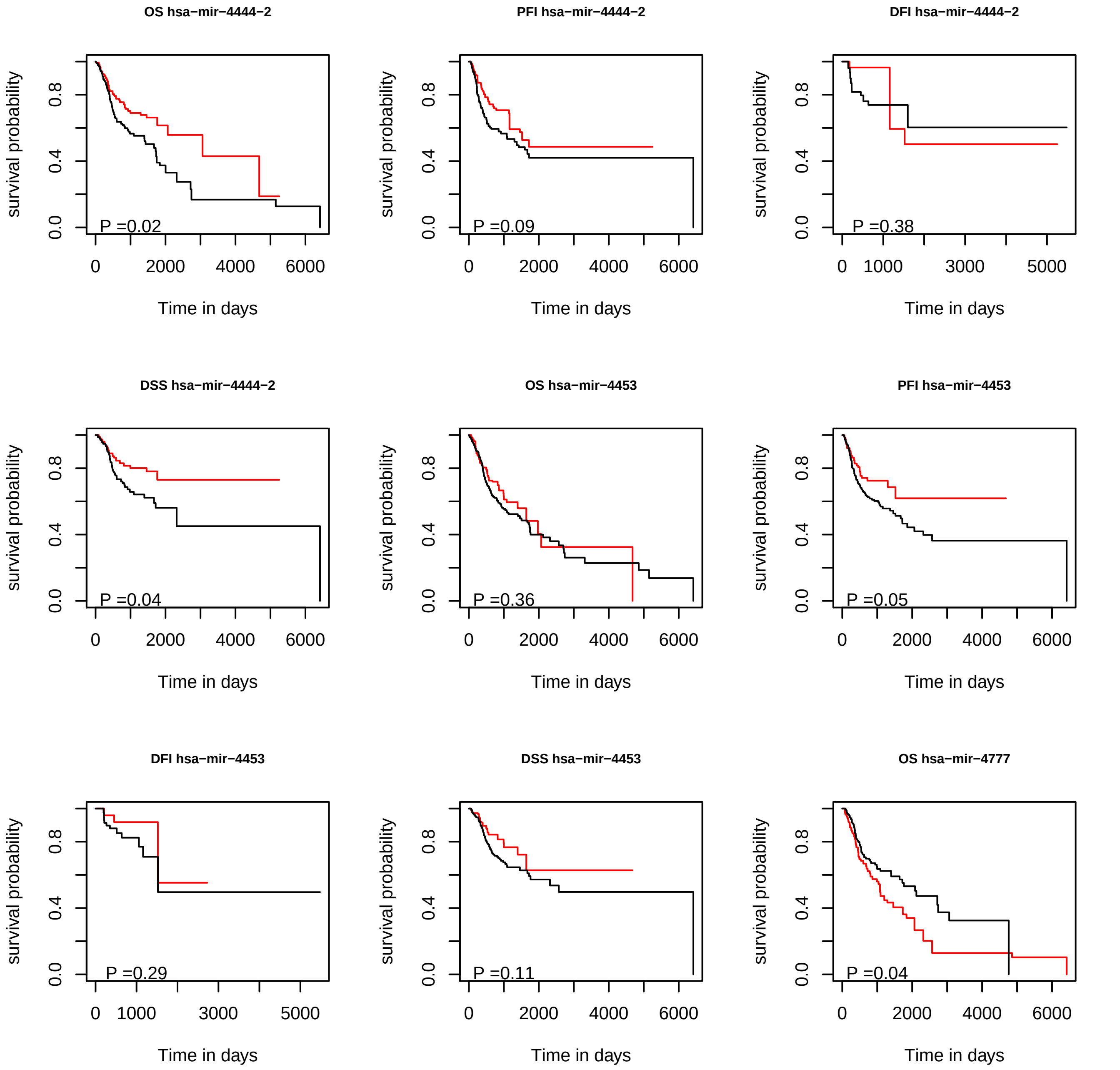
OS hsa−mir−4444−2
P =0.02
PFI hsa−mir−4444−2
P =0.09
DFI hsa−mir−4444−2
P =0.38
DSS hsa−mir−4444−2
P =0.04
OS hsa−mir−4453
P =0.36
PFI hsa−mir−4453
P =0.05
DFI hsa−mir−4453
P =0.29
DSS hsa−mir−4453
P =0.11
OS hsa−mir−4777
P =0.04
survival probability
Time in days
0.0
0.4
0.8
0
1000
2000
3000
4000
5000
6000

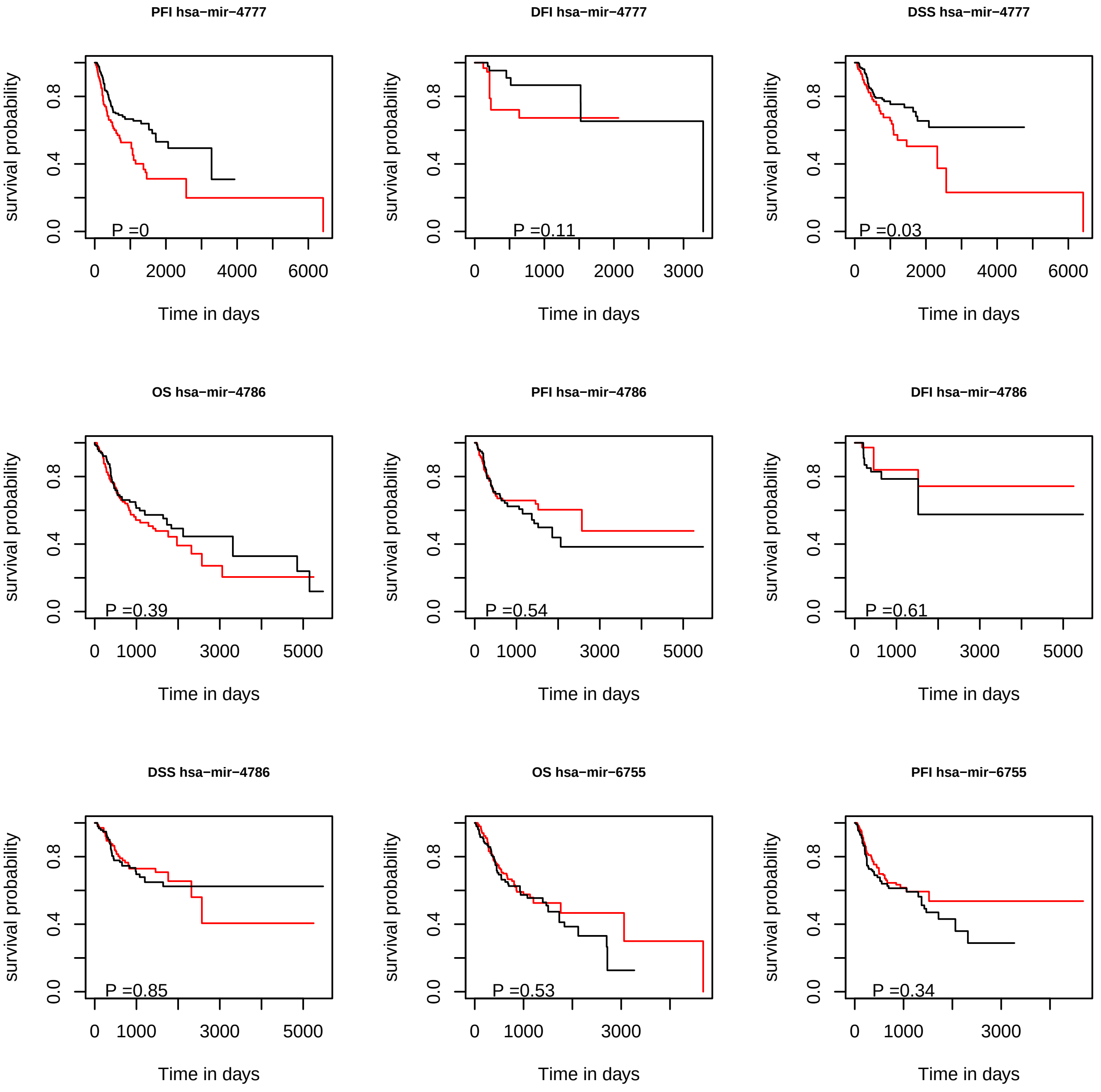
PFI hsa−mir−4777
survival probability
0.0
0.4
0.8
P =0
0
2000
4000
6000
Time in days
DFI hsa−mir−4777
survival probability
0.0
0.4
0.8
P =0.11
0
1000
2000
3000
Time in days
DSS hsa−mir−4777
survival probability
0.0
0.4
0.8
P =0.03
0
2000
4000
6000
Time in days
OS hsa−mir−4786
survival probability
0.0
0.4
0.8
P =0.39
0
1000
3000
5000
Time in days
PFI hsa−mir−4786
survival probability
0.0
0.4
0.8
P =0.54
0
1000
3000
5000
Time in days
DFI hsa−mir−4786
survival probability
0.0
0.4
0.8
P =0.61
0
1000
3000
5000
Time in days
DSS hsa−mir−4786
survival probability
0.0
0.4
0.8
P =0.85
0
1000
3000
5000
Time in days
OS hsa−mir−6755
survival probability
0.0
0.4
0.8
P =0.53
0
1000
3000
Time in days
PFI hsa−mir−6755
survival probability
0.0
0.4
0.8
P =0.34
0
1000
3000
Time in days

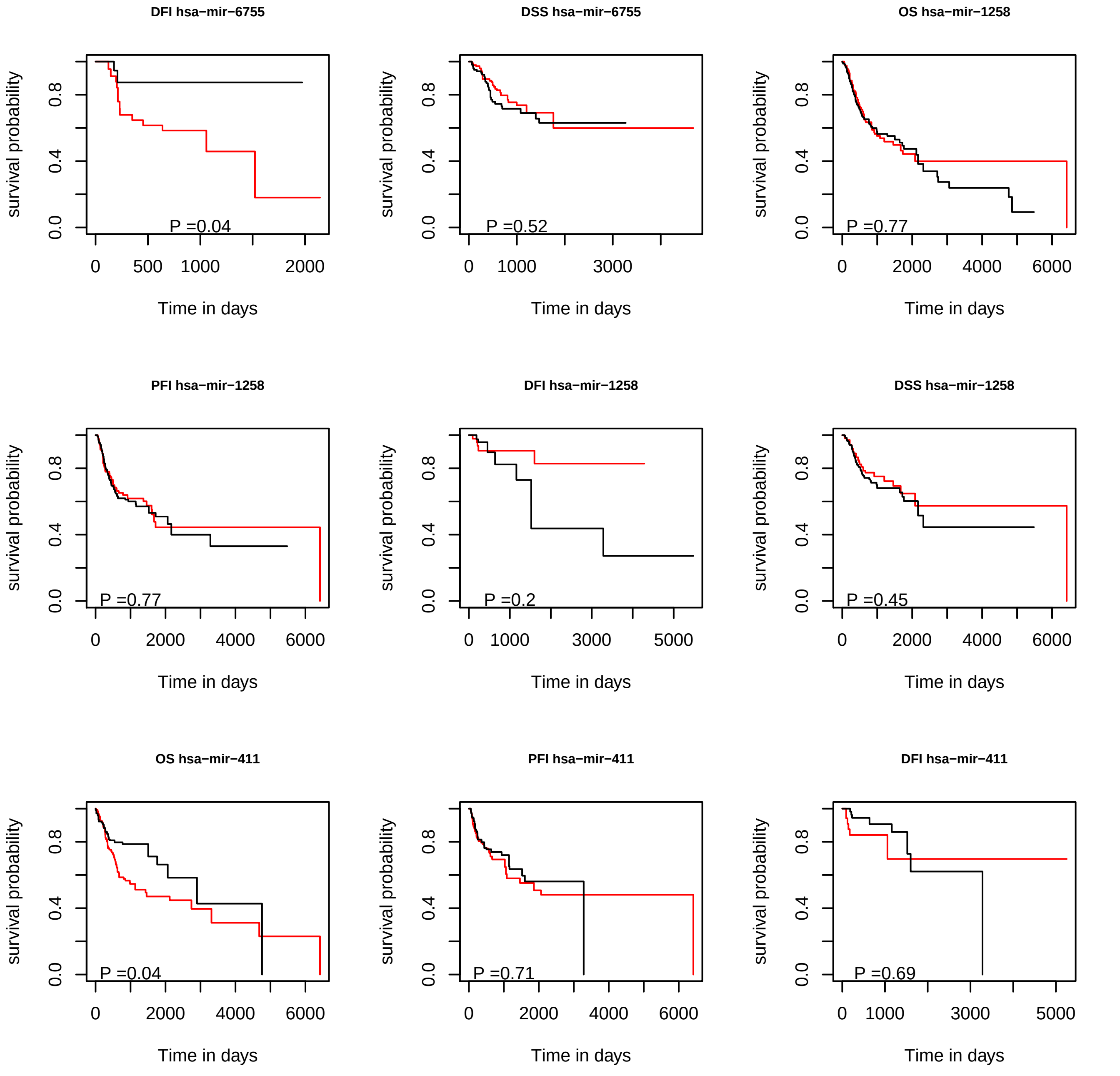
DFI hsa−mir−6755
survival probability
0.0
0.4
0.8
P =0.04
0
500
1000
2000
Time in days
DSS hsa−mir−6755
survival probability
0.0
0.4
0.8
P =0.52
0
1000
3000
Time in days
OS hsa−mir−1258
survival probability
0.0
0.4
0.8
P =0.77
0
2000
4000
6000
Time in days
PFI hsa−mir−1258
survival probability
0.0
0.4
0.8
P =0.77
0
2000
4000
6000
Time in days
DFI hsa−mir−1258
survival probability
0.0
0.4
0.8
P =0.2
0
1000
3000
5000
Time in days
DSS hsa−mir−1258
survival probability
0.0
0.4
0.8
P =0.45
0
2000
4000
6000
Time in days
OS hsa−mir−411
survival probability
0.0
0.4
0.8
P =0.04
0
2000
4000
6000
Time in days
PFI hsa−mir−411
survival probability
0.0
0.4
0.8
P =0.71
0
2000
4000
6000
Time in days
DFI hsa−mir−411
survival probability
0.0
0.4
0.8
P =0.69
0
1000
3000
5000
Time in days

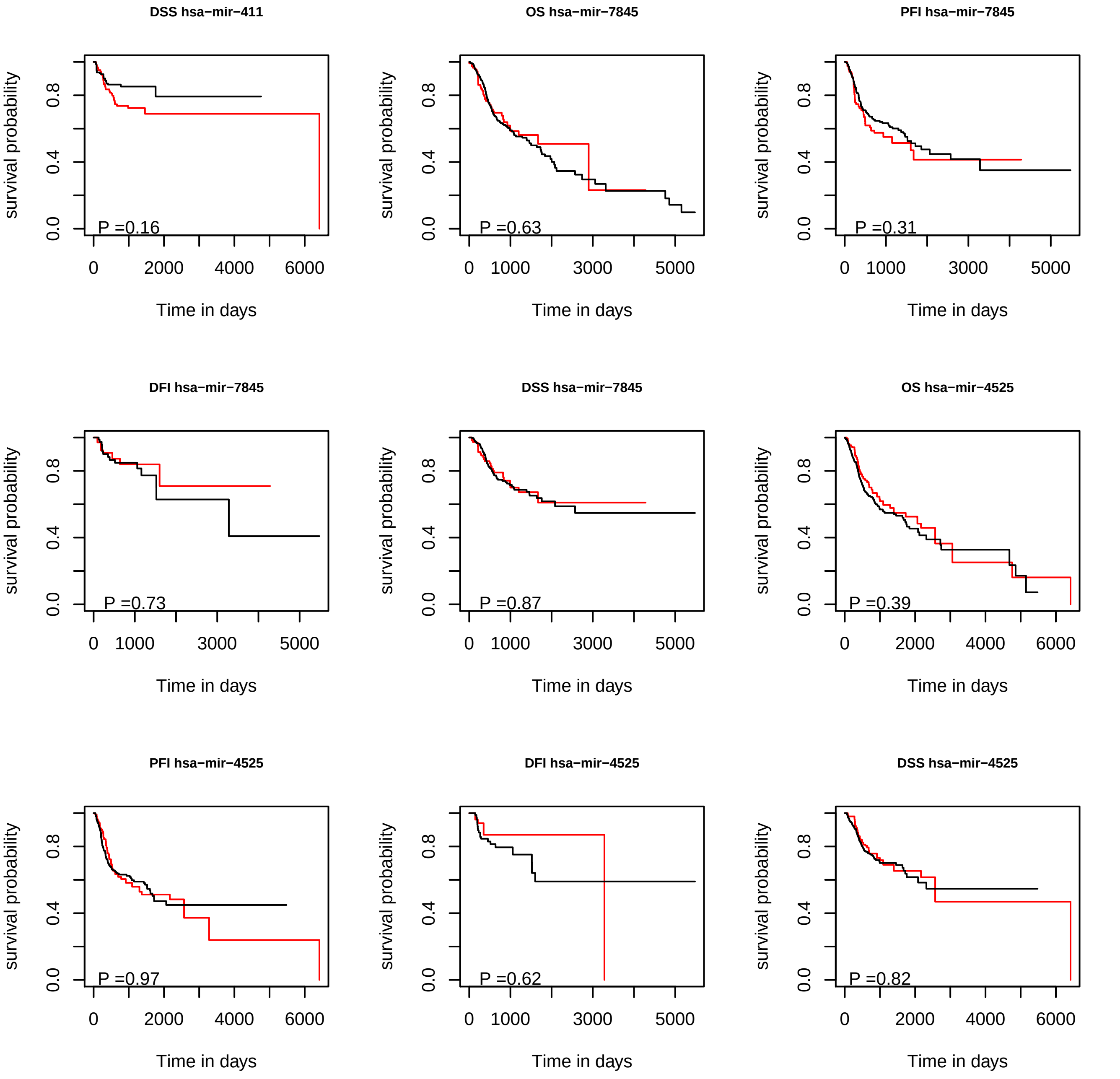

DSS hsa−mir−411
P =0.16
OS hsa−mir−7845
P =0.63
PFI hsa−mir−7845
P =0.31
DFI hsa−mir−7845
P =0.73
DSS hsa−mir−7845
P =0.87
OS hsa−mir−4525
P =0.39
PFI hsa−mir−4525
P =0.97
DFI hsa−mir−4525
P =0.62
DSS hsa−mir−4525
P =0.82
survival probability
Time in days
0.0
0.4
0.8
0
1000
2000
3000
4000
5000
6000

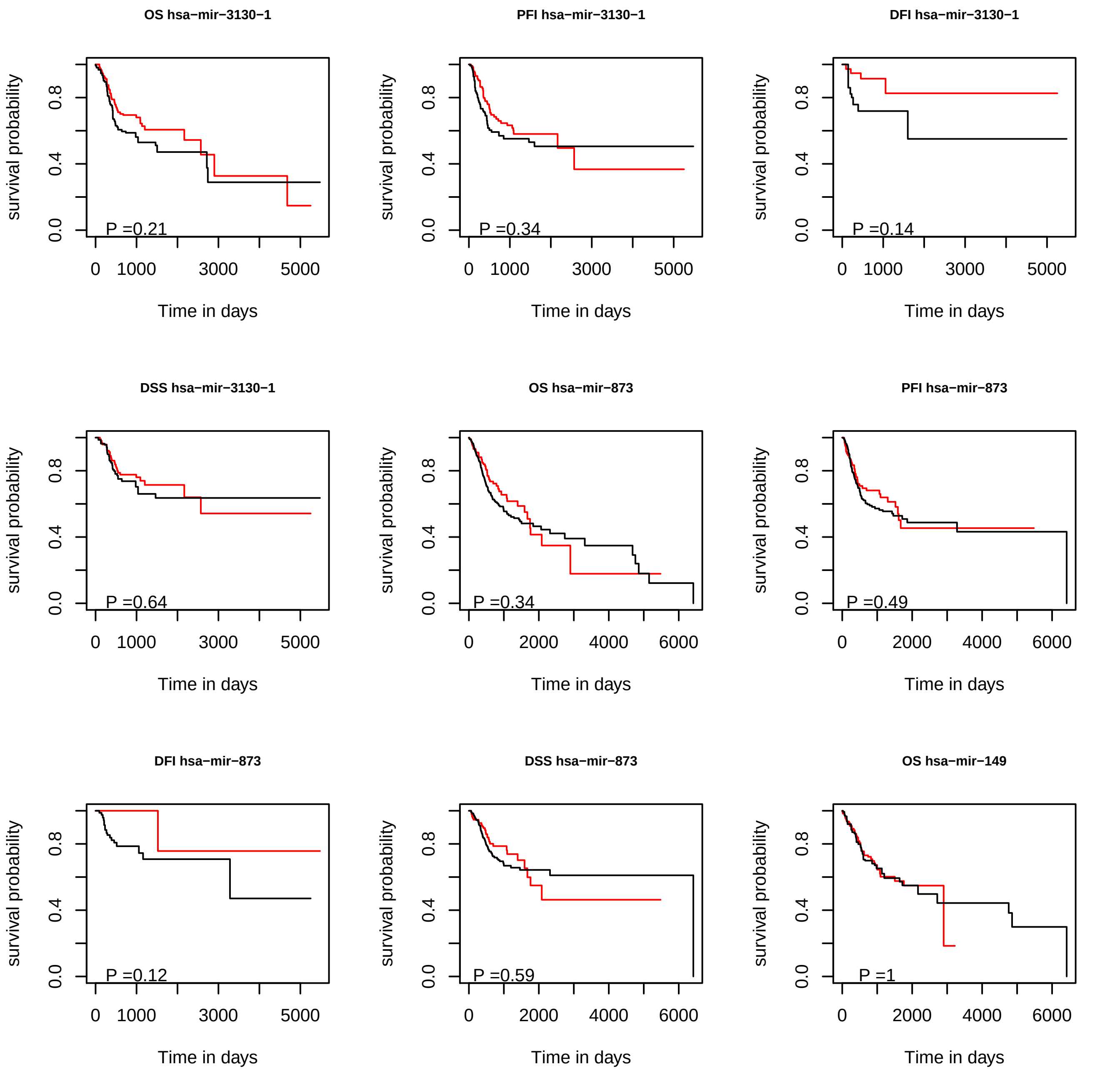
OS hsa−mir−3130−1
PFI hsa−mir−3130−1
DFI hsa−mir−3130−1
P =0.21
P =0.34
P =0.14
DSS hsa−mir−3130−1
OS hsa−mir−873
PFI hsa−mir−873
P =0.64
P =0.34
P =0.49
DFI hsa−mir−873
DSS hsa−mir−873
OS hsa−mir−149
P =0.12
P =0.59
P =1
survival probability
Time in days
0.0
0.4
0.8
0
1000
3000
5000
2000
4000
6000

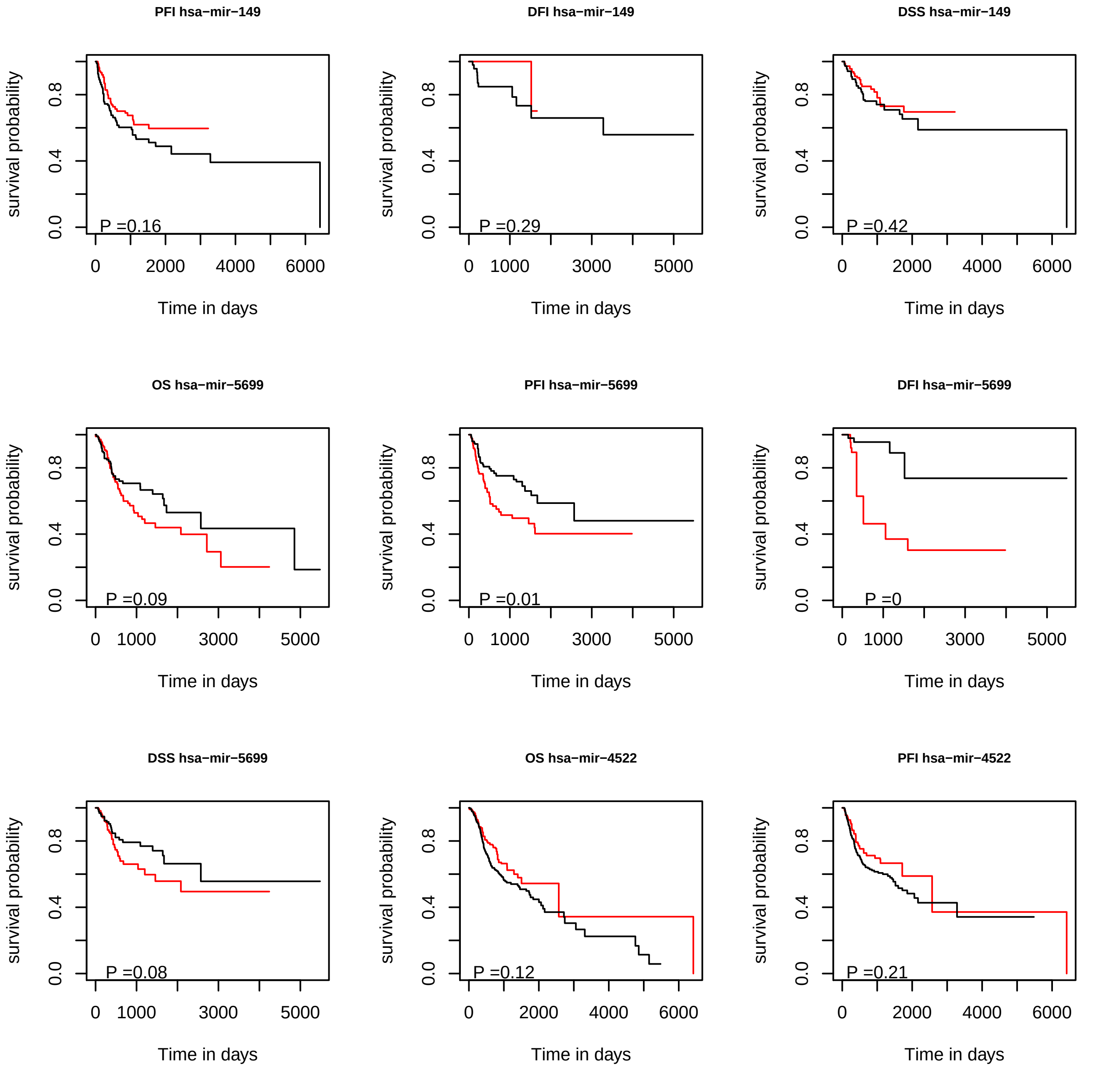
PFI hsa−mir−149
P =0.16
DFI hsa−mir−149
P =0.29
DSS hsa−mir−149
P =0.42
OS hsa−mir−5699
P =0.09
PFI hsa−mir−5699
P =0.01
DFI hsa−mir−5699
P =0
DSS hsa−mir−5699
P =0.08
OS hsa−mir−4522
P =0.12
PFI hsa−mir−4522
P =0.21
survival probability
Time in days
0.0
0.4
0.8
0
1000
2000
3000
4000
5000
6000

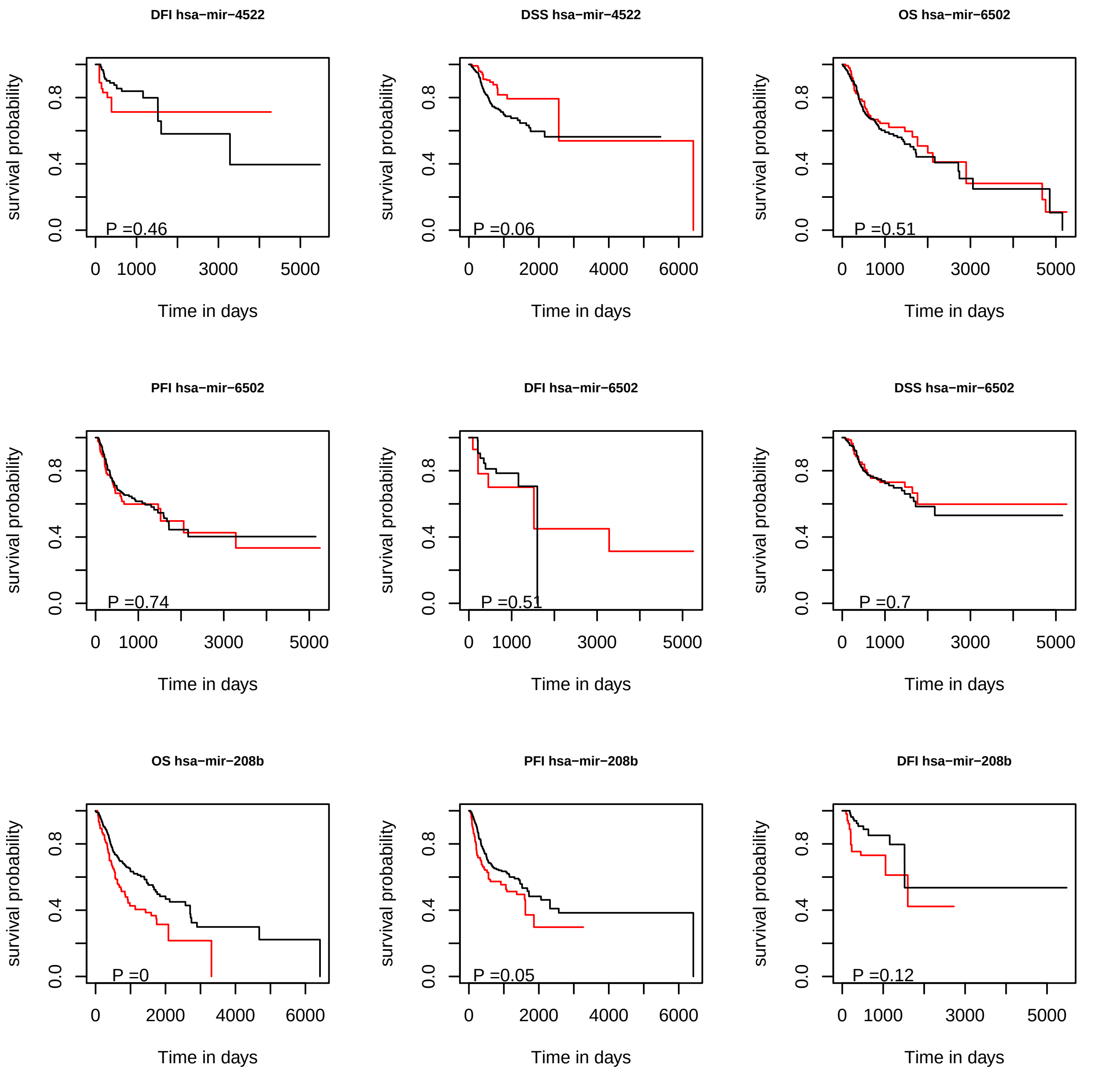

DFI hsa−mir−4522
P =0.46
DSS hsa−mir−4522
P =0.06
OS hsa−mir−6502
P =0.51
PFI hsa−mir−6502
P =0.74
DFI hsa−mir−6502
P =0.51
DSS hsa−mir−6502
P =0.7
OS hsa−mir−208b
P =0
PFI hsa−mir−208b
P =0.05
DFI hsa−mir−208b
P =0.12
survival probability
Time in days

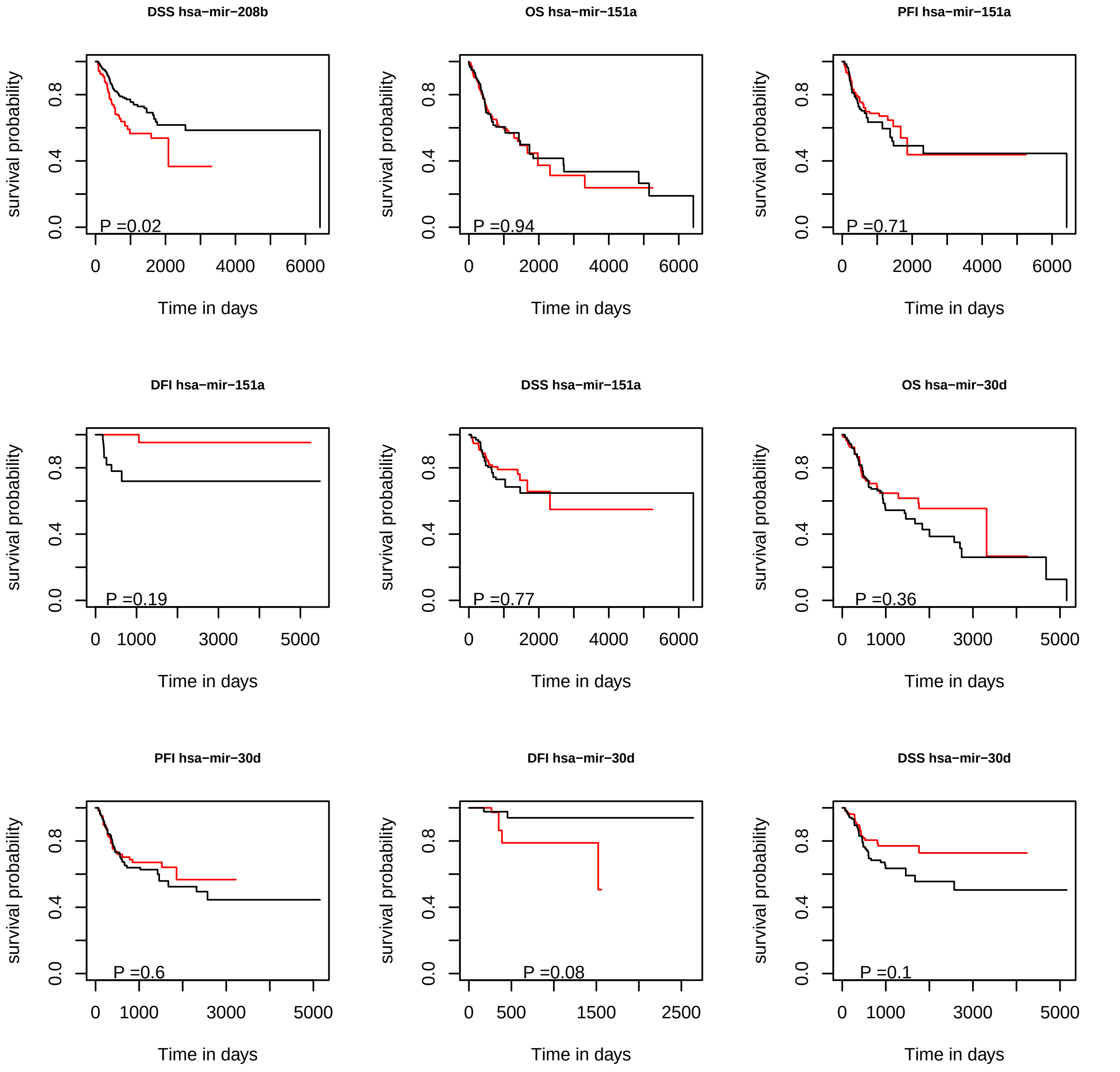
DSS hsa−mir−208b
P =0.02
OS hsa−mir−151a
P =0.94
PFI hsa−mir−151a
P =0.71
DFI hsa−mir−151a
P =0.19
DSS hsa−mir−151a
P =0.77
OS hsa−mir−30d
P =0.36
PFI hsa−mir−30d
P =0.6
DFI hsa−mir−30d
P =0.08
DSS hsa−mir−30d
P =0.1
survival probability
Time in days

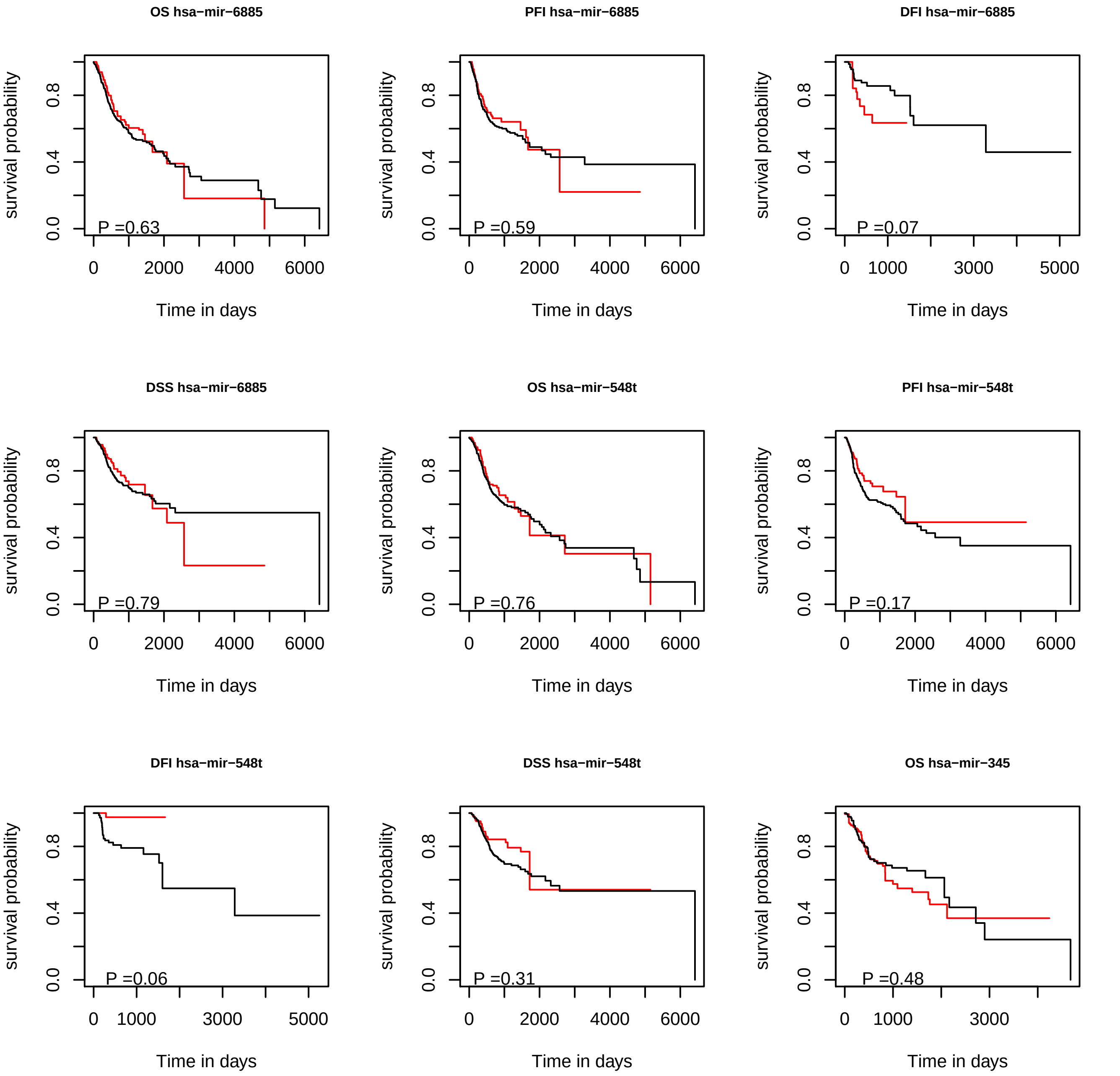
OS hsa−mir−6885
P =0.63
PFI hsa−mir−6885
P =0.59
DFI hsa−mir−6885
P =0.07
DSS hsa−mir−6885
P =0.79
OS hsa−mir−548t
P =0.76
PFI hsa−mir−548t
P =0.17
DFI hsa−mir−548t
P =0.06
DSS hsa−mir−548t
P =0.31
OS hsa−mir−345
P =0.48
survival probability
Time in days
0.0
0.4
0.8
0
1000
2000
3000
4000
5000
6000

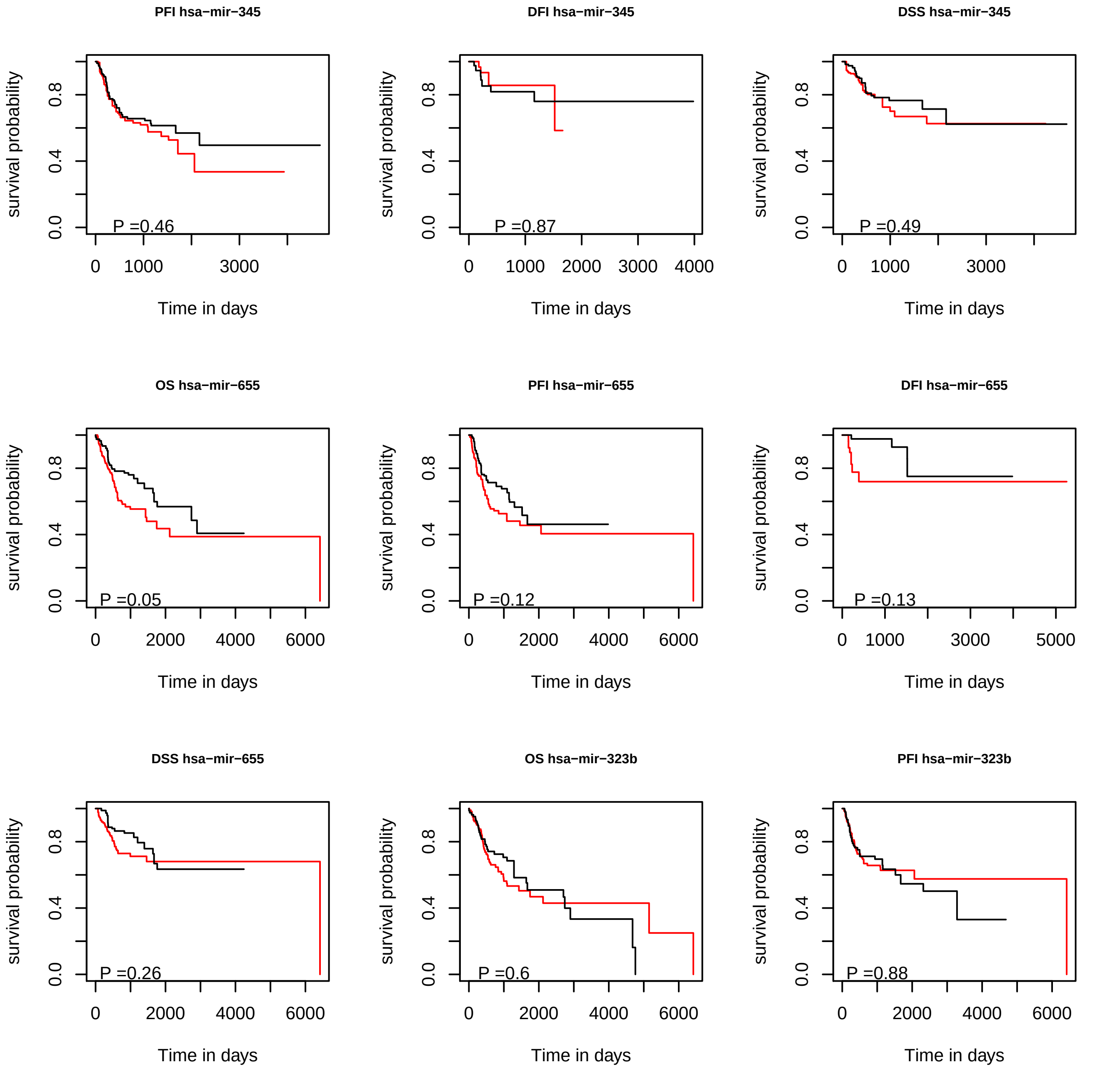

PFI hsa−mir−345
survival probability
0.0
0.4
0.8
P =0.46
0
1000
3000
Time in days
DFI hsa−mir−345
survival probability
0.0
0.4
0.8
P =0.87
0
1000
2000
3000
4000
Time in days
DSS hsa−mir−345
survival probability
0.0
0.4
0.8
P =0.49
0
1000
3000
Time in days
OS hsa−mir−655
survival probability
0.0
0.4
0.8
P =0.05
0
2000
4000
6000
Time in days
PFI hsa−mir−655
survival probability
0.0
0.4
0.8
P =0.12
0
2000
4000
6000
Time in days
DFI hsa−mir−655
survival probability
0.0
0.4
0.8
P =0.13
0
1000
3000
5000
Time in days
DSS hsa−mir−655
survival probability
0.0
0.4
0.8
P =0.26
0
2000
4000
6000
Time in days
OS hsa−mir−323b
survival probability
0.0
0.4
0.8
P =0.6
0
2000
4000
6000
Time in days
PFI hsa−mir−323b
survival probability
0.0
0.4
0.8
P =0.88
0
2000
4000
6000
Time in days

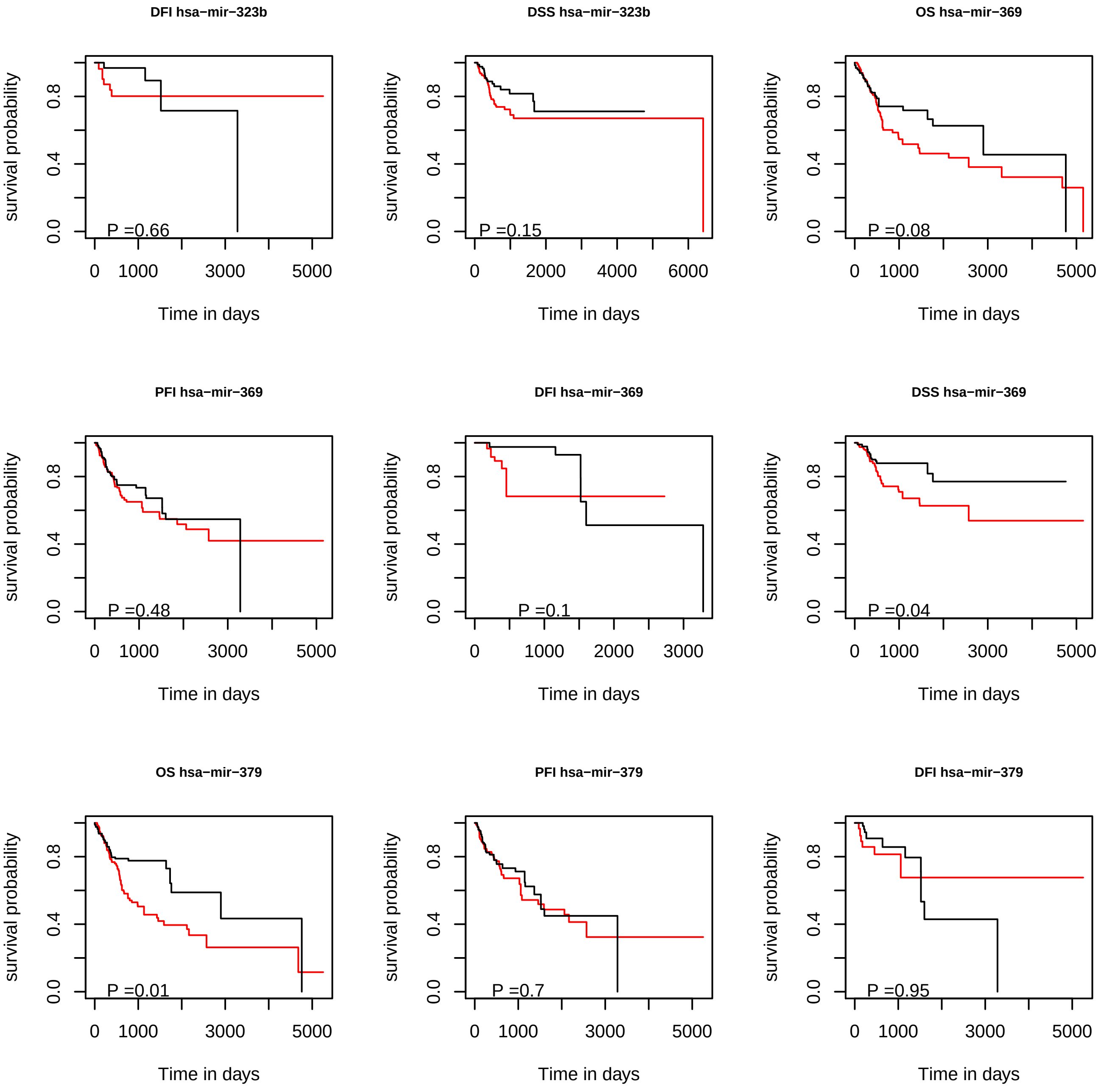
DFI hsa−mir−323b
DSS hsa−mir−323b
OS hsa−mir−369
PFI hsa−mir−369
DFI hsa−mir−369
DSS hsa−mir−369
OS hsa−mir−379
PFI hsa−mir−379
DFI hsa−mir−379
survival probability
0.0
0.4
0.8
Time in days
0
1000
2000
3000
4000
5000
6000
P =0.66
P =0.15
P =0.08
P =0.48
P =0.1
P =0.04
P =0.01
P =0.7
P =0.95

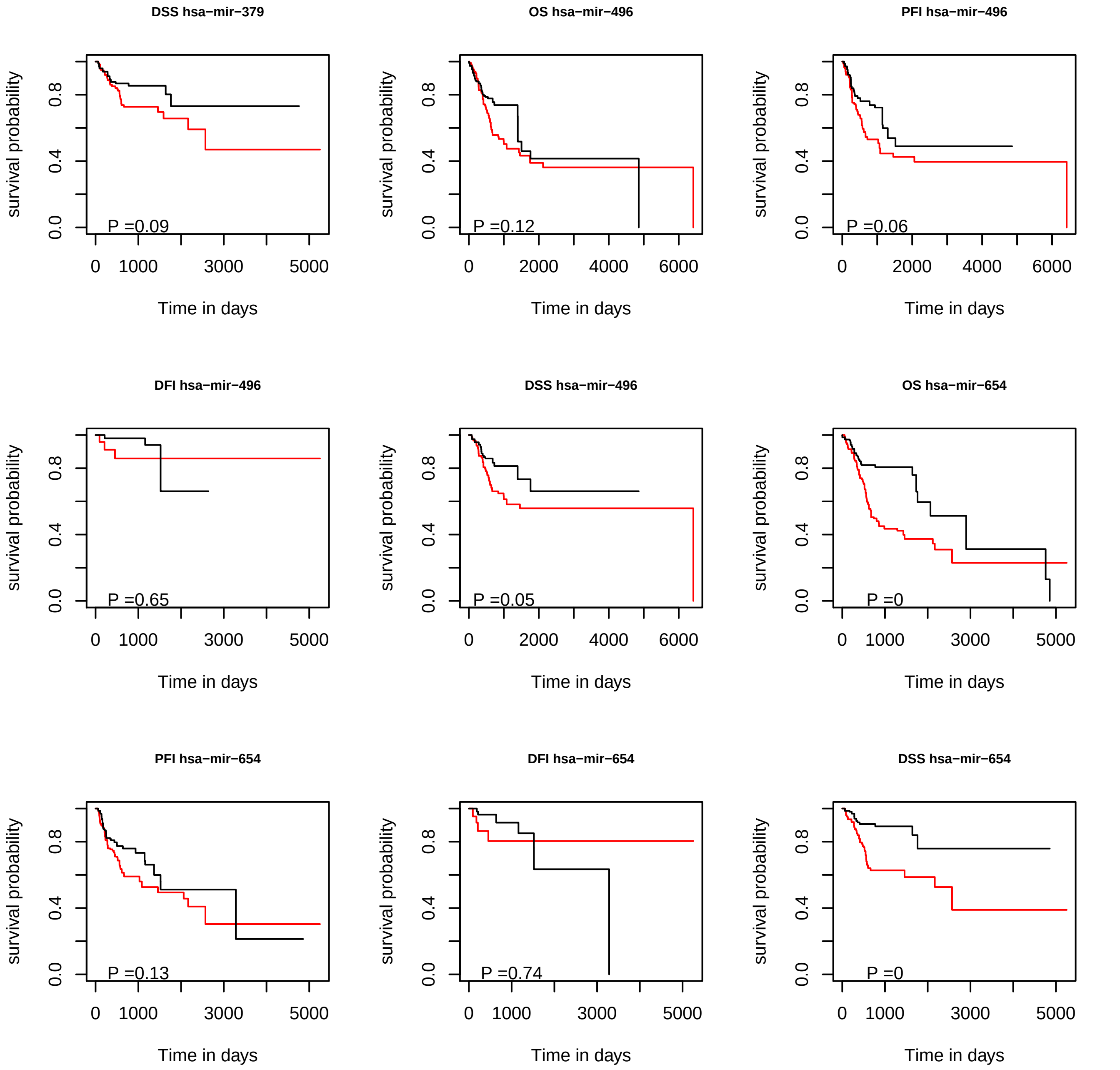
DSS hsa−mir−379
P =0.09
OS hsa−mir−496
P =0.12
PFI hsa−mir−496
P =0.06
DFI hsa−mir−496
P =0.65
DSS hsa−mir−496
P =0.05
OS hsa−mir−654
P =0
PFI hsa−mir−654
P =0.13
DFI hsa−mir−654
P =0.74
DSS hsa−mir−654
P =0
survival probability
0.0
0.4
0.8
Time in days
0
1000
3000
5000
2000
4000
6000

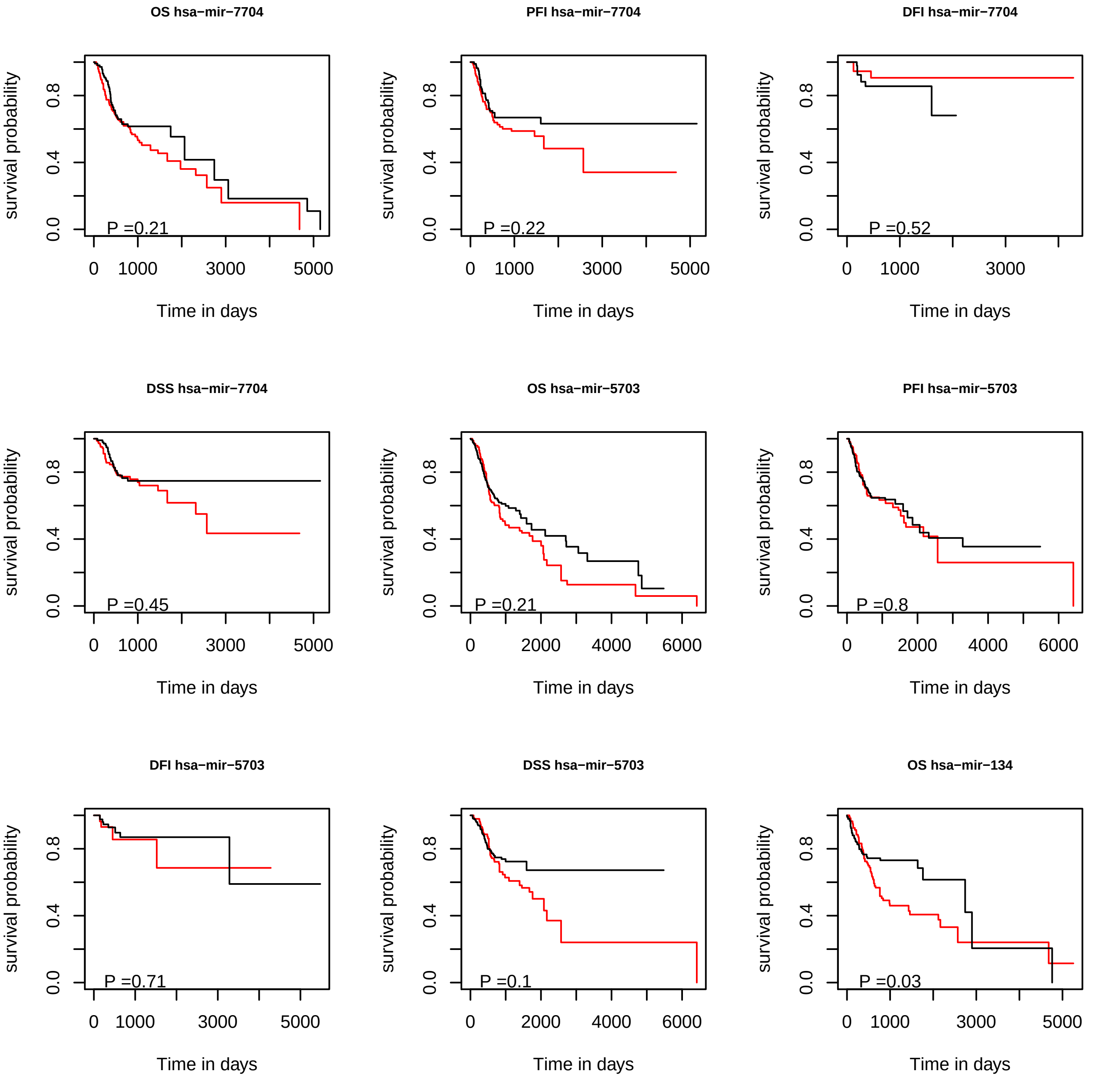

OS hsa−mir−7704
P =0.21
PFI hsa−mir−7704
P =0.22
DFI hsa−mir−7704
P =0.52
DSS hsa−mir−7704
P =0.45
OS hsa−mir−5703
P =0.21
PFI hsa−mir−5703
P =0.8
DFI hsa−mir−5703
P =0.71
DSS hsa−mir−5703
P =0.1
OS hsa−mir−134
P =0.03
survival probability
Time in days

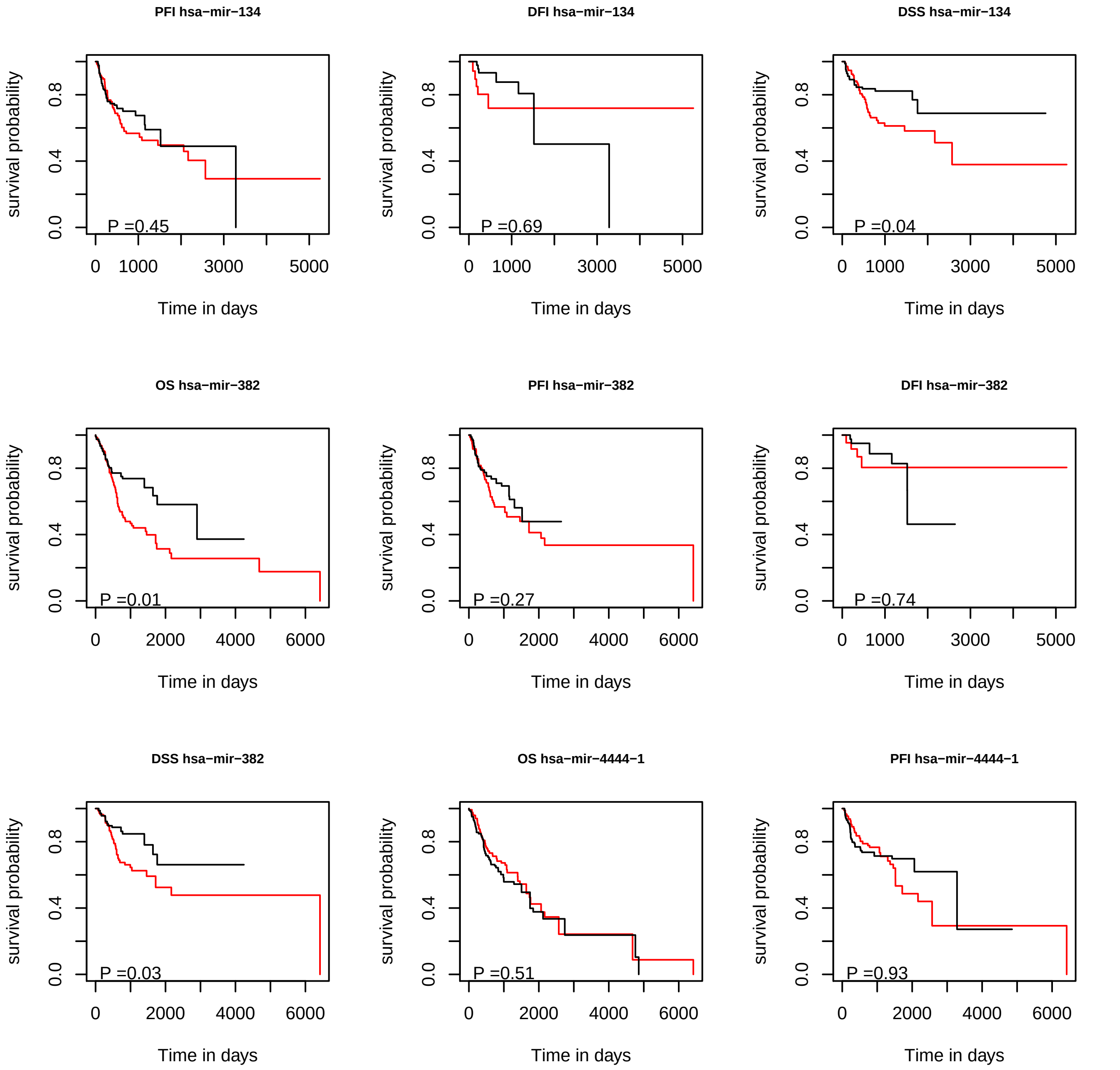
PFI hsa−mir−134
P =0.45
DFI hsa−mir−134
P =0.69
DSS hsa−mir−134
P =0.04
OS hsa−mir−382
P =0.01
PFI hsa−mir−382
P =0.27
DFI hsa−mir−382
P =0.74
DSS hsa−mir−382
P =0.03
OS hsa−mir−4444−1
P =0.51
PFI hsa−mir−4444−1
P =0.93
survival probability
Time in days

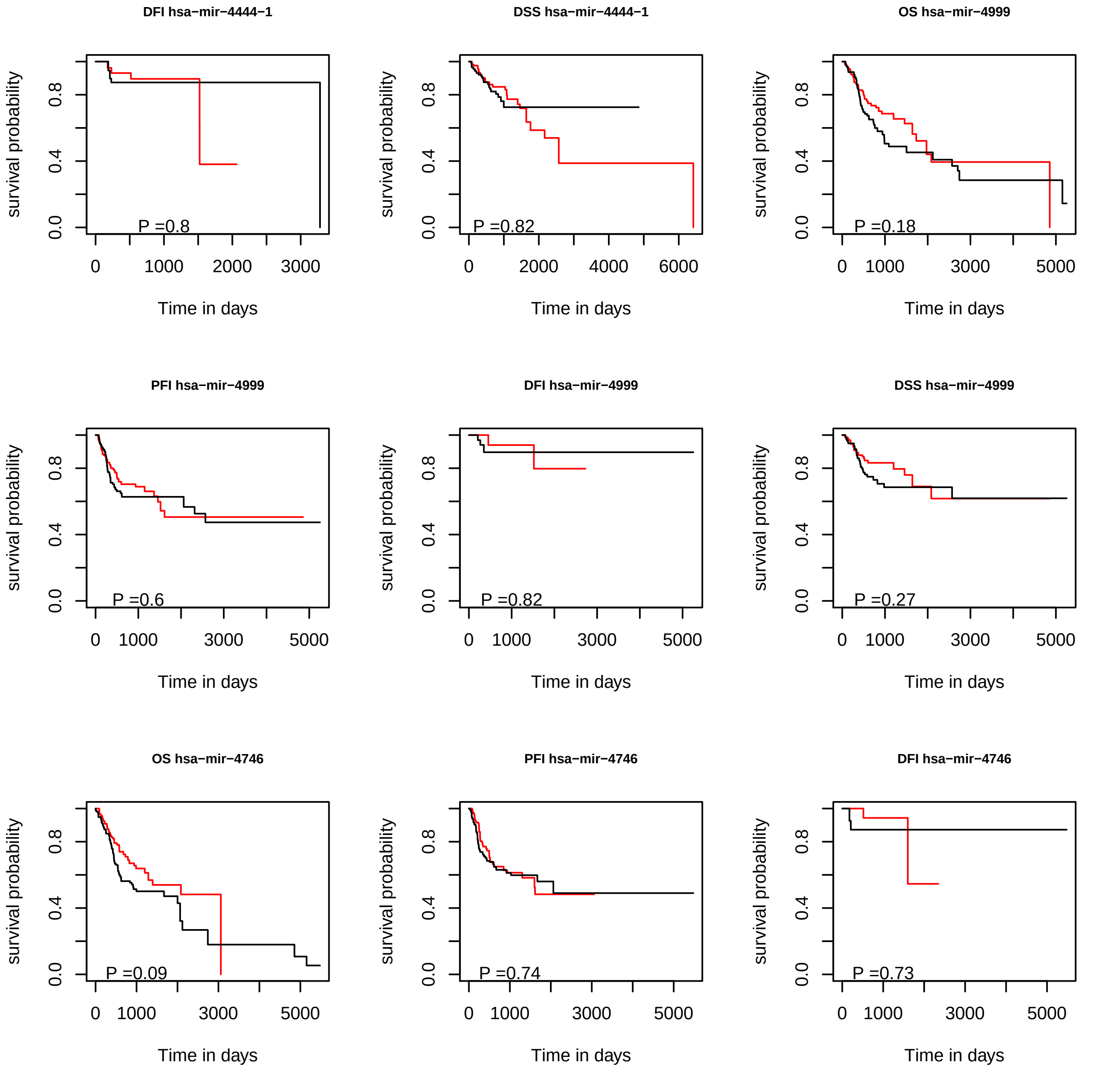
DFI hsa−mir−4444−1
P =0.8
DSS hsa−mir−4444−1
P =0.82
OS hsa−mir−4999
P =0.18
PFI hsa−mir−4999
P =0.6
DFI hsa−mir−4999
P =0.82
DSS hsa−mir−4999
P =0.27
OS hsa−mir−4746
P =0.09
PFI hsa−mir−4746
P =0.74
DFI hsa−mir−4746
P =0.73
survival probability
Time in days
0.0
0.4
0.8
0
1000
2000
3000
4000
5000
6000

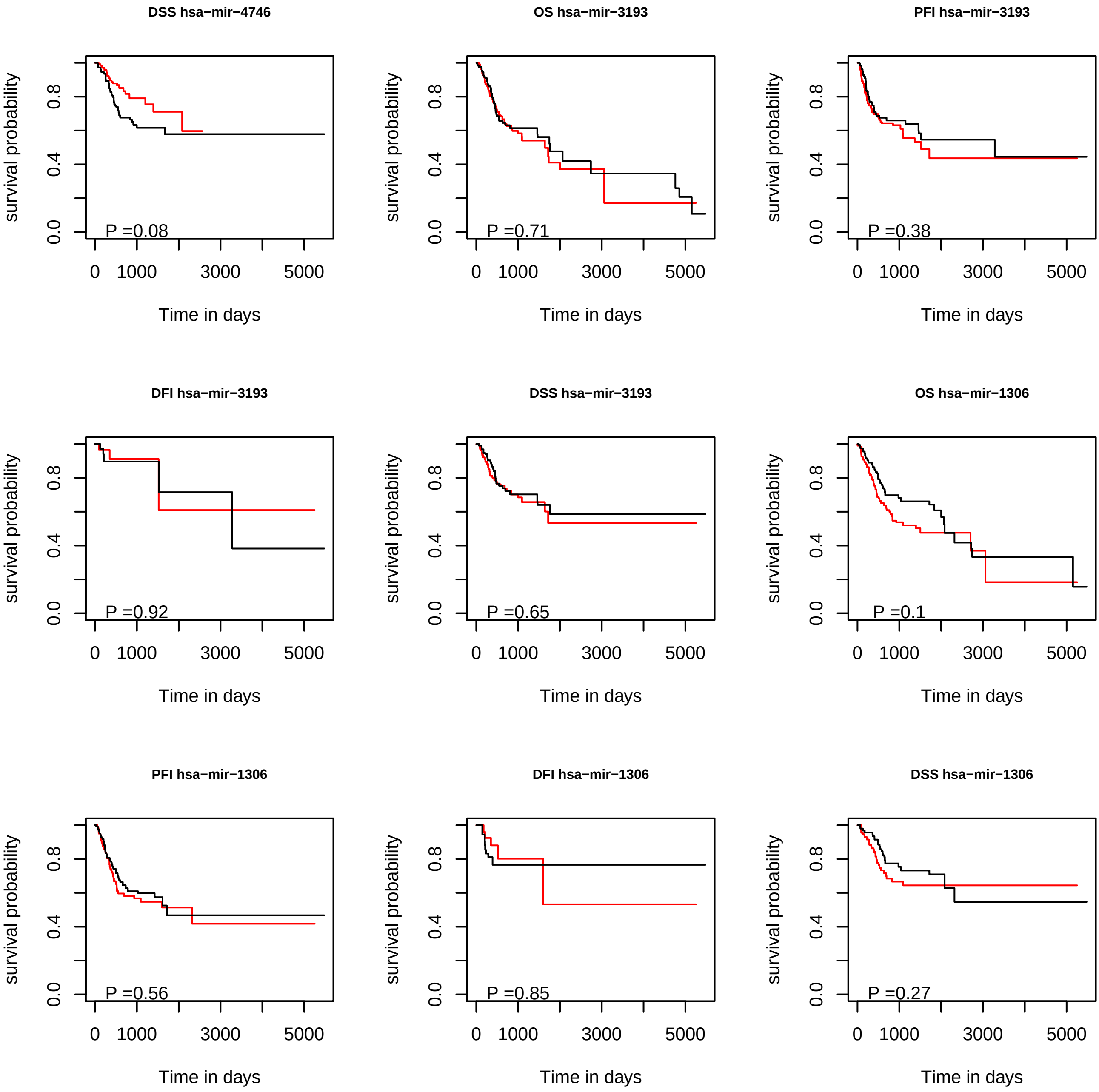
DSS hsa−mir−4746
P =0.08
OS hsa−mir−3193
P =0.71
PFI hsa−mir−3193
P =0.38
DFI hsa−mir−3193
P =0.92
DSS hsa−mir−3193
P =0.65
OS hsa−mir−1306
P =0.1
PFI hsa−mir−1306
P =0.56
DFI hsa−mir−1306
P =0.85
DSS hsa−mir−1306
P =0.27
survival probability
0.0
0.4
0.8
Time in days
0
1000
3000
5000

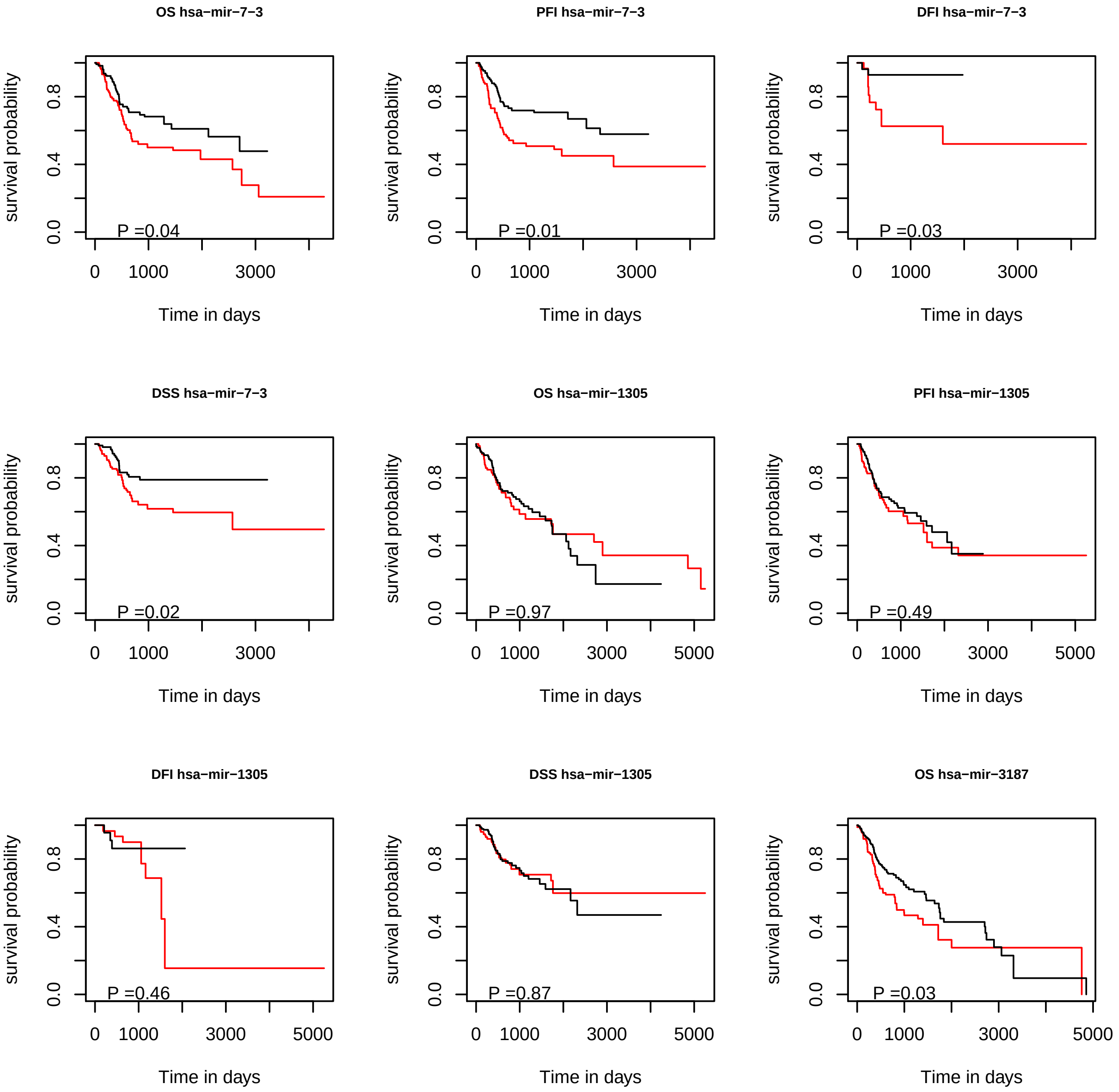
OS hsa−mir−7−3
P =0.04
PFI hsa−mir−7−3
P =0.01
DFI hsa−mir−7−3
P =0.03
DSS hsa−mir−7−3
P =0.02
OS hsa−mir−1305
P =0.97
PFI hsa−mir−1305
P =0.49
DFI hsa−mir−1305
P =0.46
DSS hsa−mir−1305
P =0.87
OS hsa−mir−3187
P =0.03
survival probability
Time in days

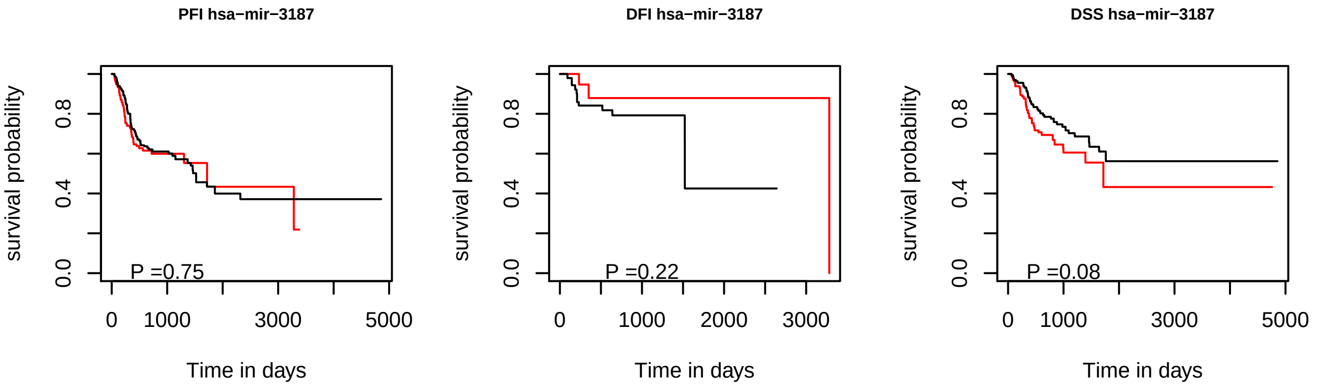
PFI hsa−mir−3187
survival probability
0.0
0.4
0.8
P =0.75
0
1000
3000
5000
Time in days
DFI hsa−mir−3187
survival probability
0.0
0.4
0.8
P =0.22
0
1000
2000
3000
Time in days
DSS hsa−mir−3187
survival probability
0.0
0.4
0.8
P =0.08
0
1000
3000
5000
Time in days
