# Supplementary material for: Computing microRNA-gene interaction networks in pan-cancer using miRDriver
Source: Sci Rep. 2022 Mar 8;12:3717. doi: 10.1038/s41598-022-07628-z (PMC8904490; doi:10.1038/s41598-022-07628-z)
Supplement: Supplementary file 17 — Supplementary Information 17. [file 41598_2022_7628_MOESM17_ESM.pdf]

Computing microRNA-gene interaction networks in pan-cancer using miRDriver

Banabithi Bose, Matthew Moravec, and Serdar Bozdag

Supplemental Figure S8

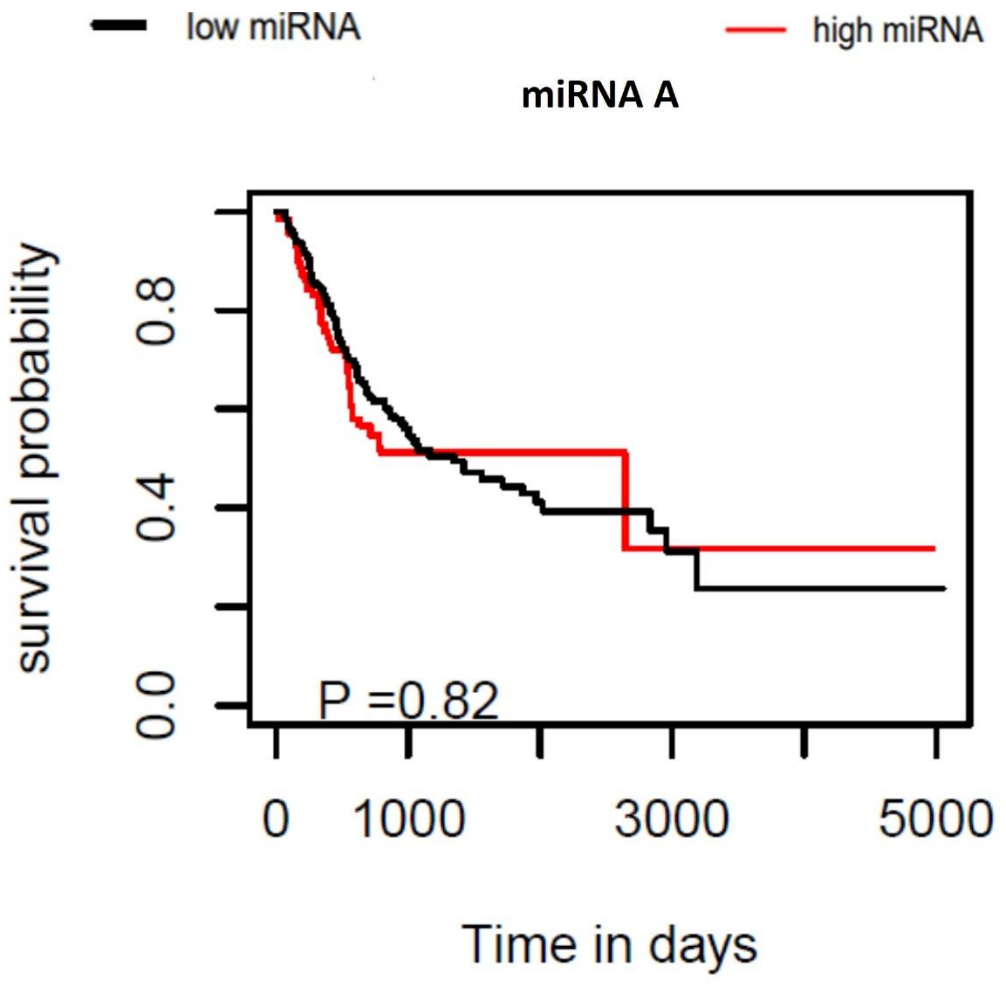

The *Adjusted Kaplan-Meier* survival plots for the computed miRNAs in high and low miRNA expression patient groups.

Supplemental Figure S8 Cancer Type: KIRC

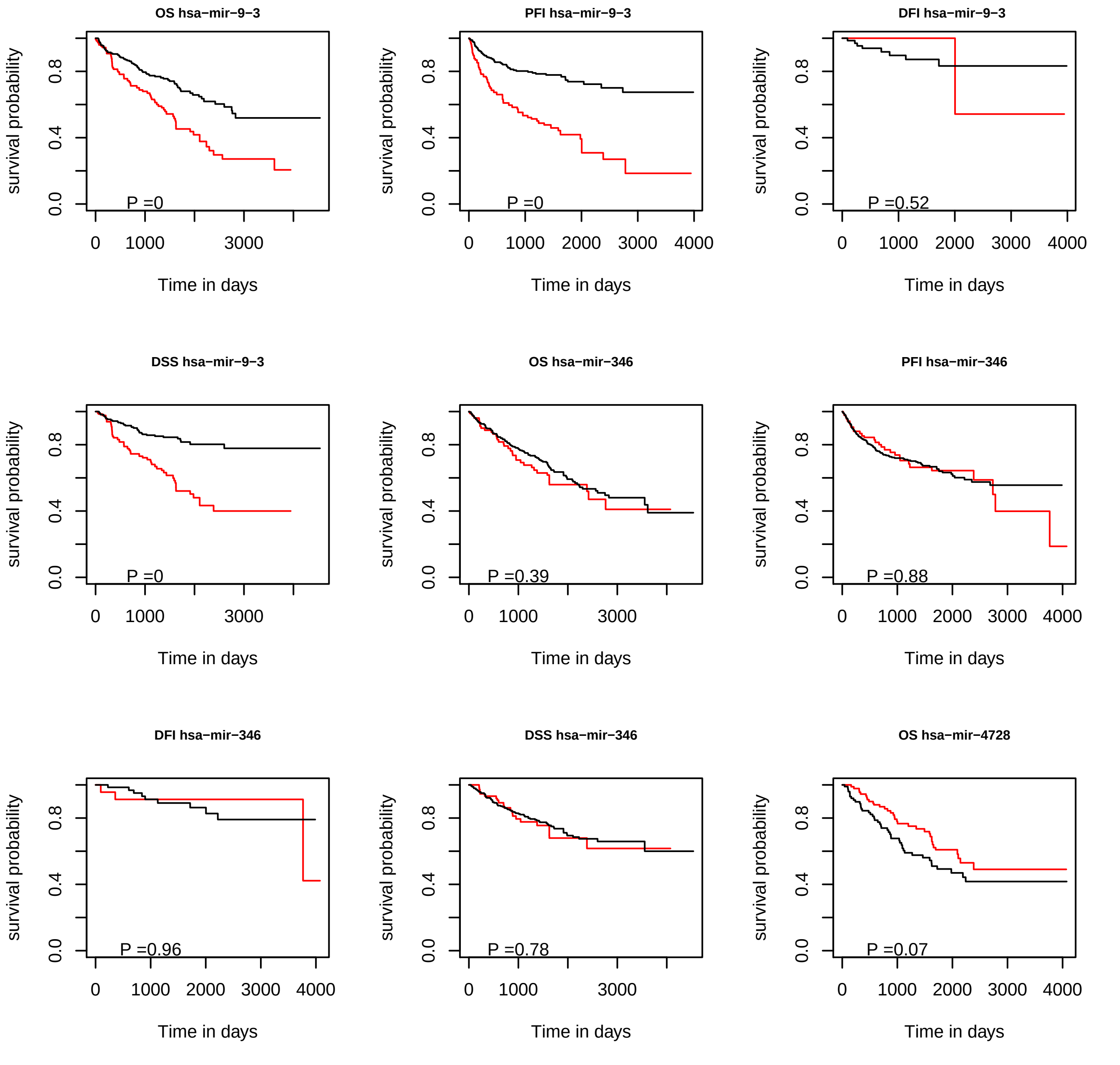

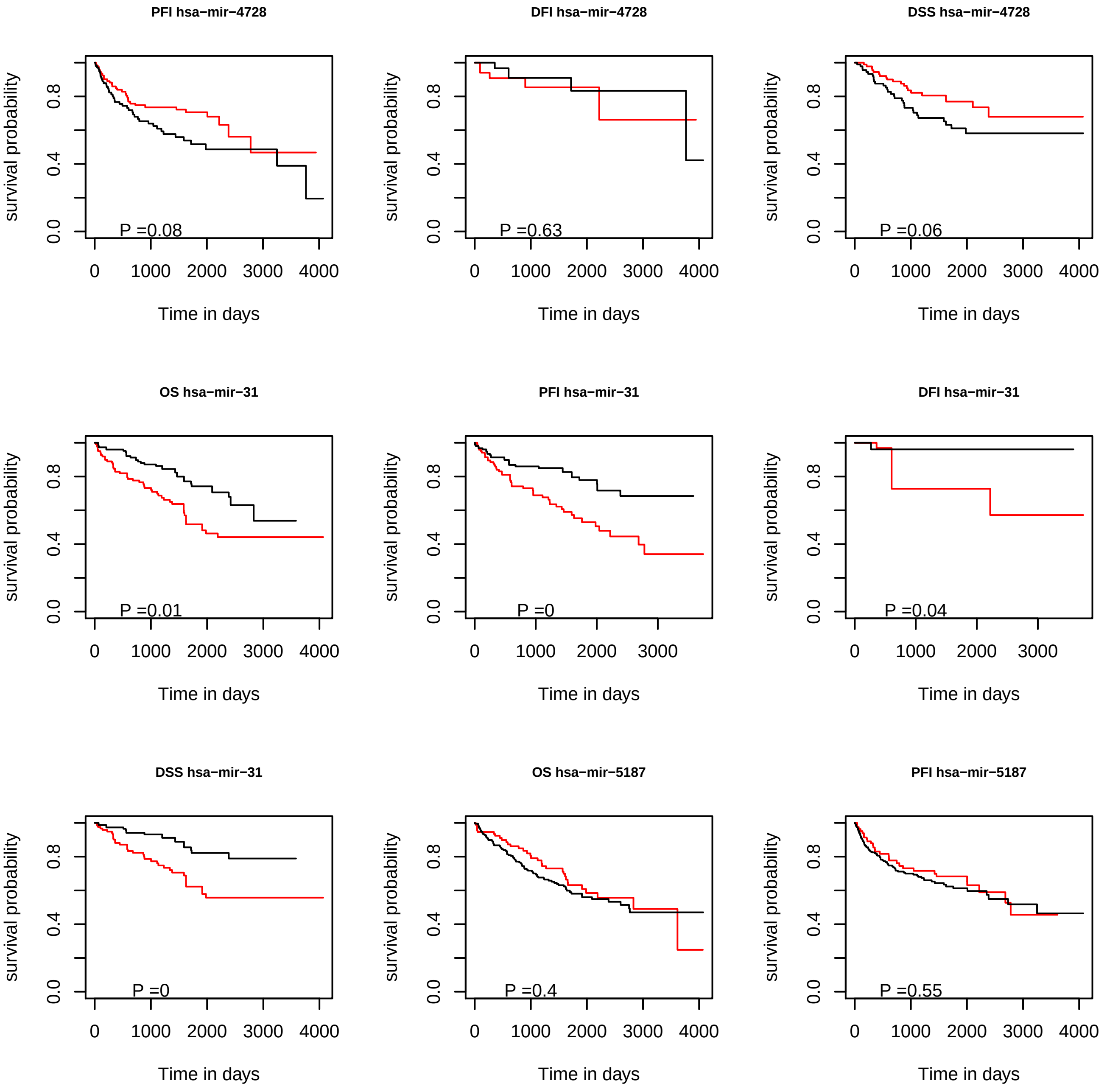

PFI hsa−mir−4728
P =0.08
DFI hsa−mir−4728
P =0.63
DSS hsa−mir−4728
P =0.06
OS hsa−mir−31
P =0.01
PFI hsa−mir−31
P =0
DFI hsa−mir−31
P =0.04
DSS hsa−mir−31
P =0
OS hsa−mir−5187
P =0.4
PFI hsa−mir−5187
P =0.55
survival probability
0.0
0.4
0.8
Time in days
0
1000
2000
3000
4000

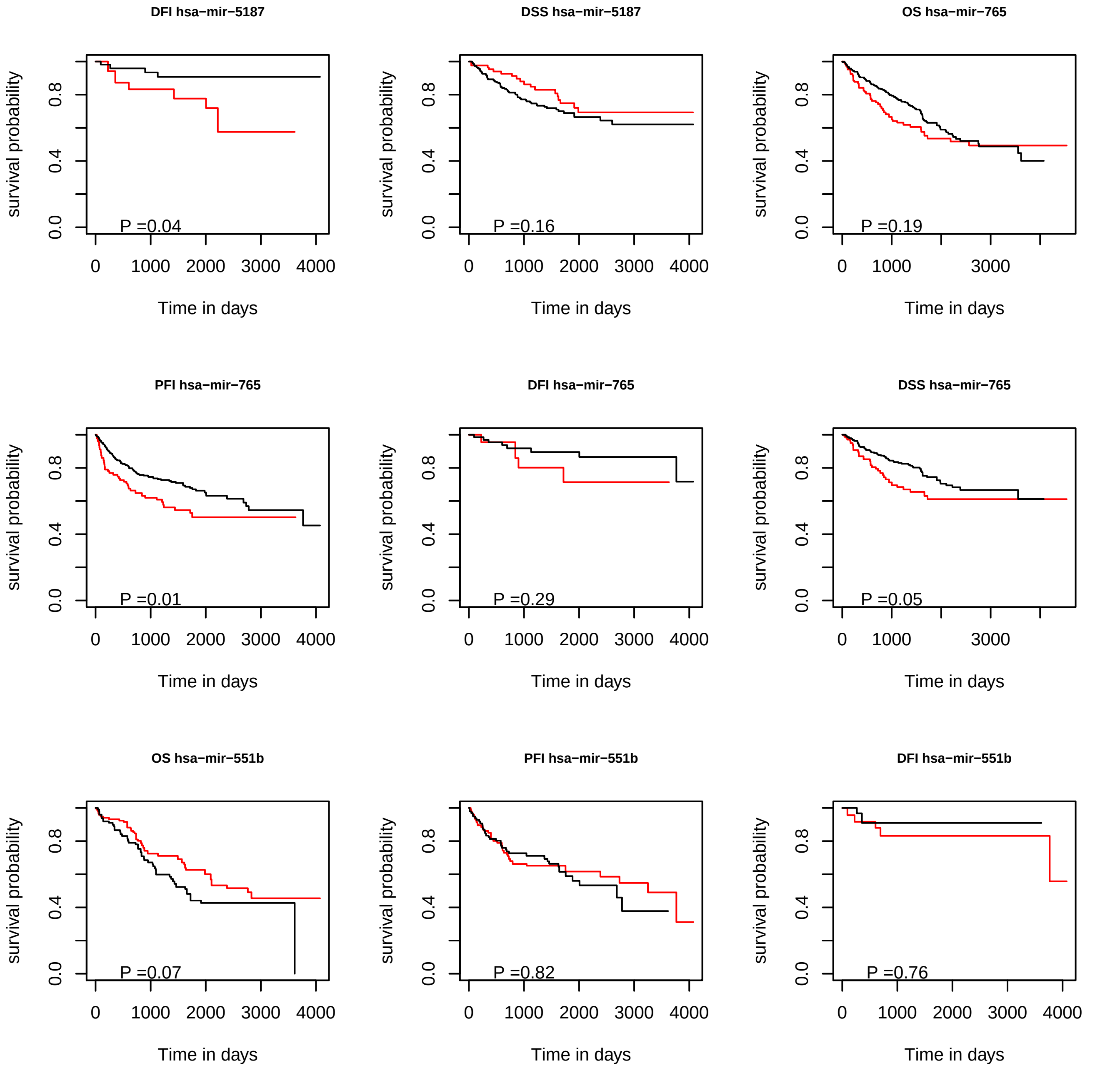
DFI hsa−mir−5187
survival probability
0.0 0.4 0.8
P =0.04
0 1000 2000 3000 4000
Time in days
DSS hsa−mir−5187
survival probability
0.0 0.4 0.8
P =0.16
0 1000 2000 3000 4000
Time in days
OS hsa−mir−765
survival probability
0.0 0.4 0.8
P =0.19
0 1000 3000
Time in days
PFI hsa−mir−765
survival probability
0.0 0.4 0.8
P =0.01
0 1000 2000 3000 4000
Time in days
DFI hsa−mir−765
survival probability
0.0 0.4 0.8
P =0.29
0 1000 2000 3000 4000
Time in days
DSS hsa−mir−765
survival probability
0.0 0.4 0.8
P =0.05
0 1000 3000
Time in days
OS hsa−mir−551b
survival probability
0.0 0.4 0.8
P =0.07
0 1000 2000 3000 4000
Time in days
PFI hsa−mir−551b
survival probability
0.0 0.4 0.8
P =0.82
0 1000 2000 3000 4000
Time in days
DFI hsa−mir−551b
survival probability
0.0 0.4 0.8
P =0.76
0 1000 2000 3000 4000
Time in days

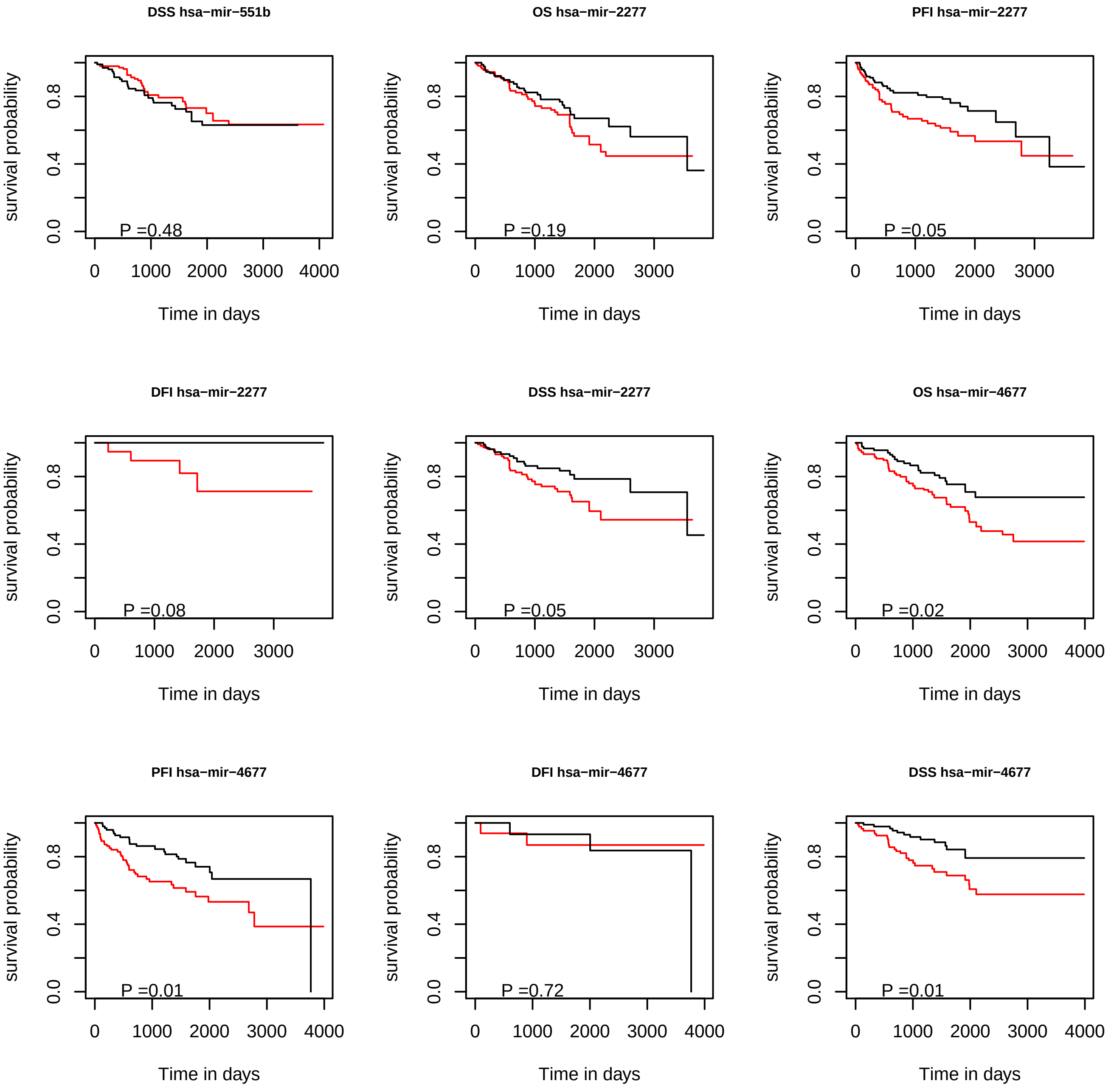
DSS hsa−mir−551b
P =0.48
OS hsa−mir−2277
P =0.19
PFI hsa−mir−2277
P =0.05
DFI hsa−mir−2277
P =0.08
DSS hsa−mir−2277
P =0.05
OS hsa−mir−4677
P =0.02
PFI hsa−mir−4677
P =0.01
DFI hsa−mir−4677
P =0.72
DSS hsa−mir−4677
P =0.01
survival probability
Time in days
0.0
0.4
0.8
0
1000
2000
3000
4000

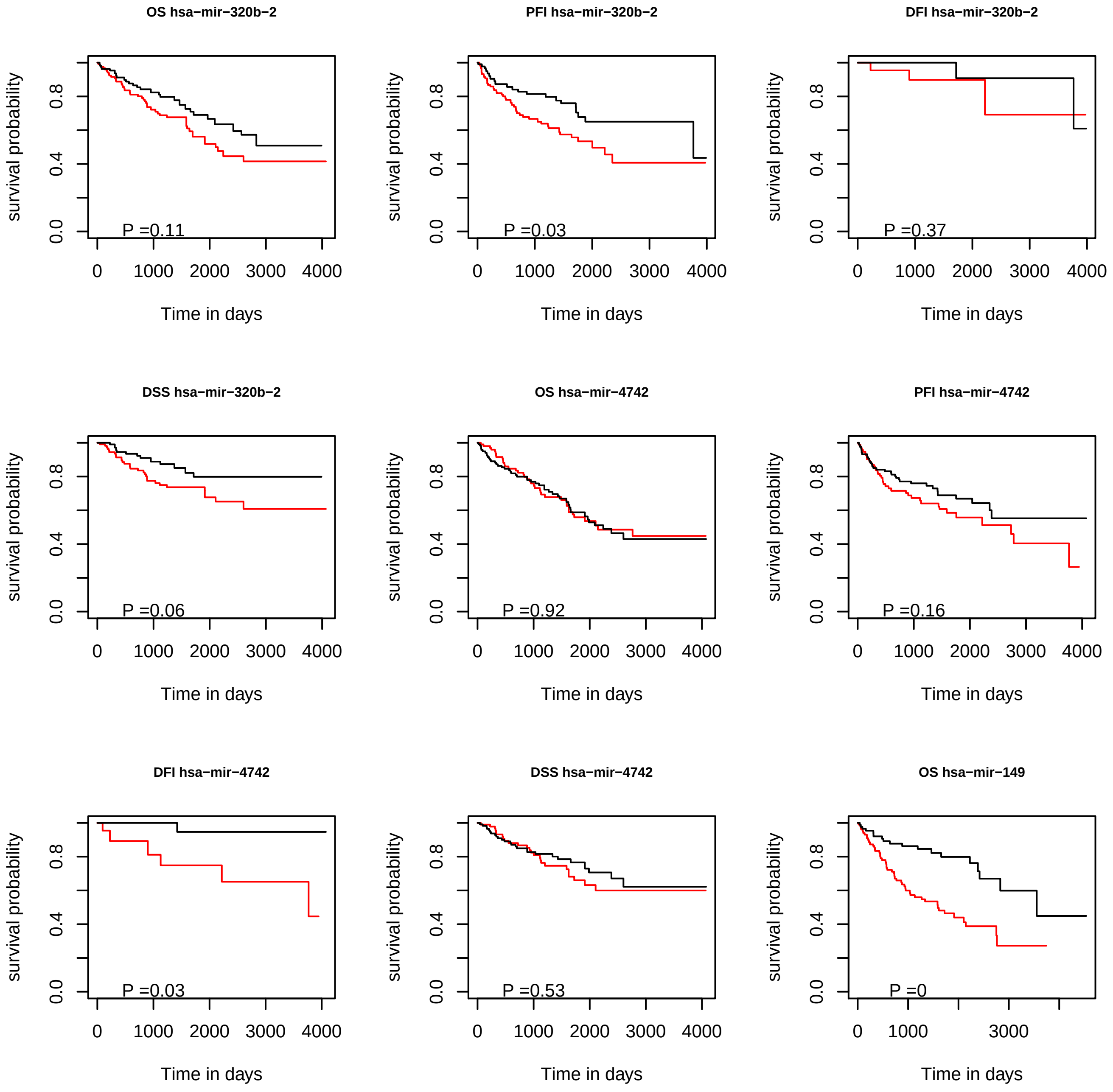
OS hsa−mir−320b−2
P =0.11
PFI hsa−mir−320b−2
P =0.03
DFI hsa−mir−320b−2
P =0.37
DSS hsa−mir−320b−2
P =0.06
OS hsa−mir−4742
P =0.92
PFI hsa−mir−4742
P =0.16
DFI hsa−mir−4742
P =0.03
DSS hsa−mir−4742
P =0.53
OS hsa−mir−149
P =0
survival probability
0.0
0.4
0.8
0
1000
2000
3000
4000
Time in days

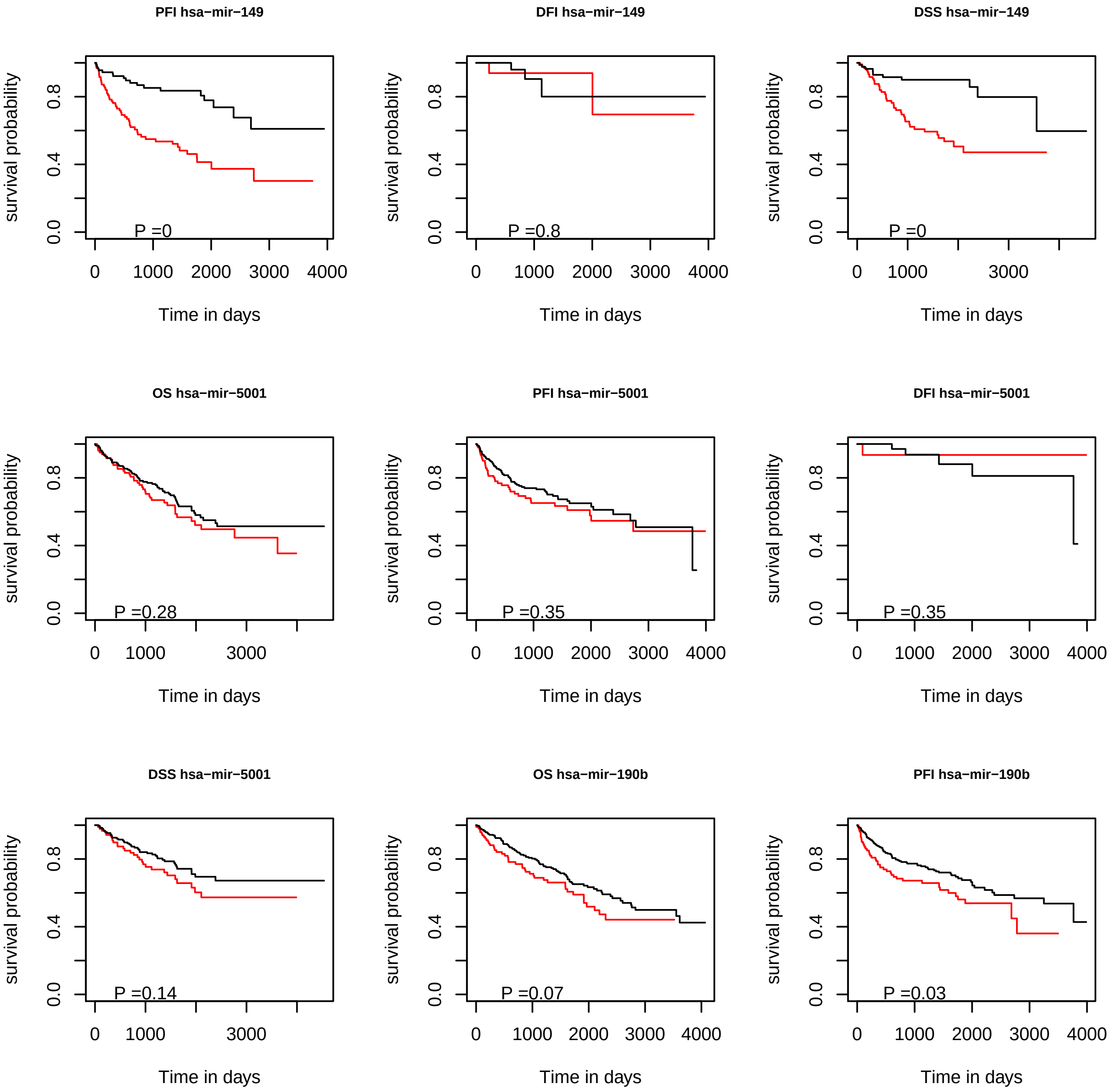
PFI hsa−mir−149
survival probability
0.0 0.4 0.8
P =0
0 1000 2000 3000 4000
Time in days
DFI hsa−mir−149
survival probability
0.0 0.4 0.8
P =0.8
0 1000 2000 3000 4000
Time in days
DSS hsa−mir−149
survival probability
0.0 0.4 0.8
P =0
0 1000 3000
Time in days
OS hsa−mir−5001
survival probability
0.0 0.4 0.8
P =0.28
0 1000 3000
Time in days
PFI hsa−mir−5001
survival probability
0.0 0.4 0.8
P =0.35
0 1000 2000 3000 4000
Time in days
DFI hsa−mir−5001
survival probability
0.0 0.4 0.8
P =0.35
0 1000 2000 3000 4000
Time in days
DSS hsa−mir−5001
survival probability
0.0 0.4 0.8
P =0.14
0 1000 3000
Time in days
OS hsa−mir−190b
survival probability
0.0 0.4 0.8
P =0.07
0 1000 2000 3000 4000
Time in days
PFI hsa−mir−190b
survival probability
0.0 0.4 0.8
P =0.03
0 1000 2000 3000 4000
Time in days

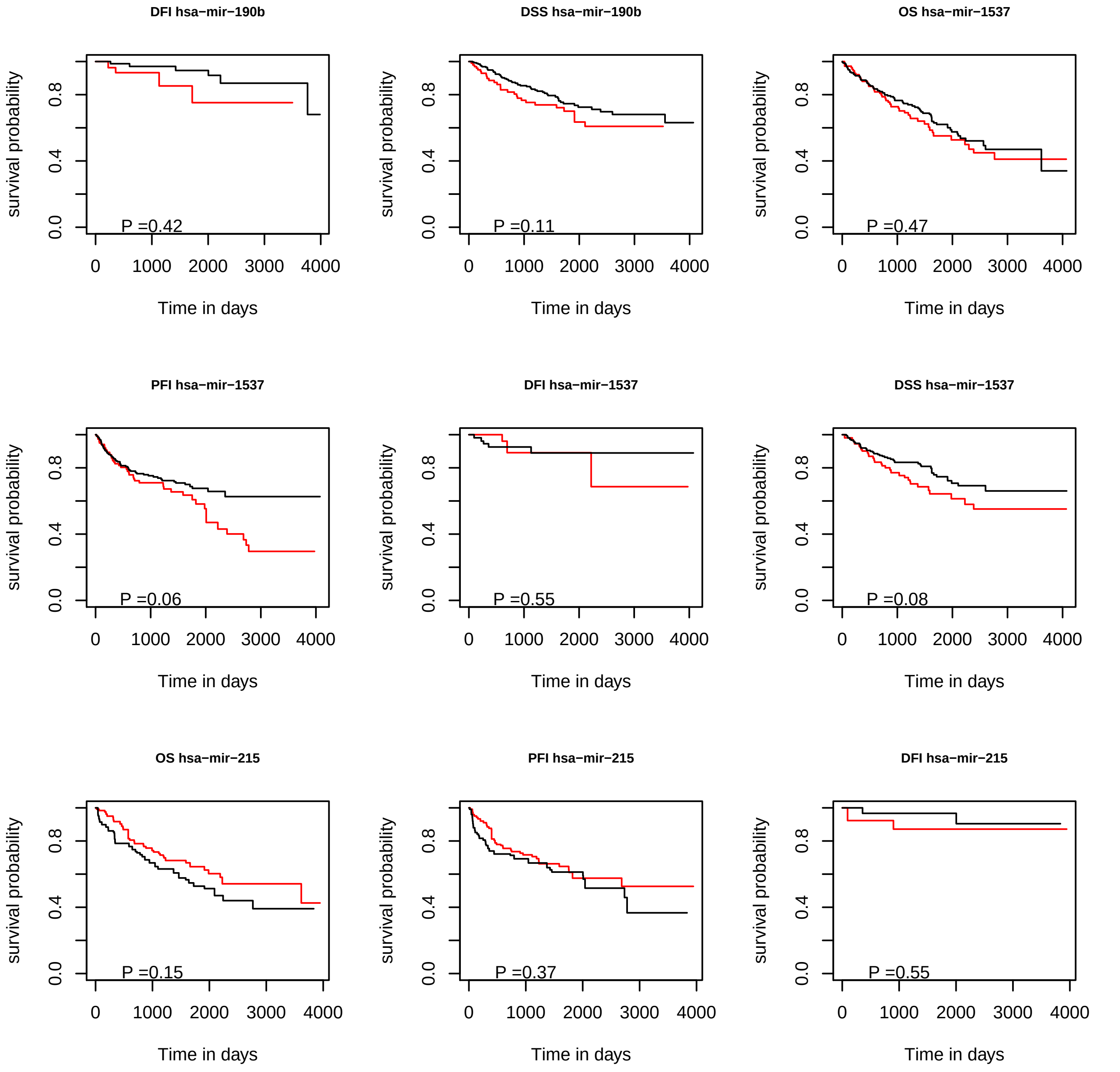
DFI hsa−mir−190b
P =0.42
DSS hsa−mir−190b
P =0.11
OS hsa−mir−1537
P =0.47
PFI hsa−mir−1537
P =0.06
DFI hsa−mir−1537
P =0.55
DSS hsa−mir−1537
P =0.08
OS hsa−mir−215
P =0.15
PFI hsa−mir−215
P =0.37
DFI hsa−mir−215
P =0.55
survival probability
Time in days
0.0
0.4
0.8
0
1000
2000
3000
4000

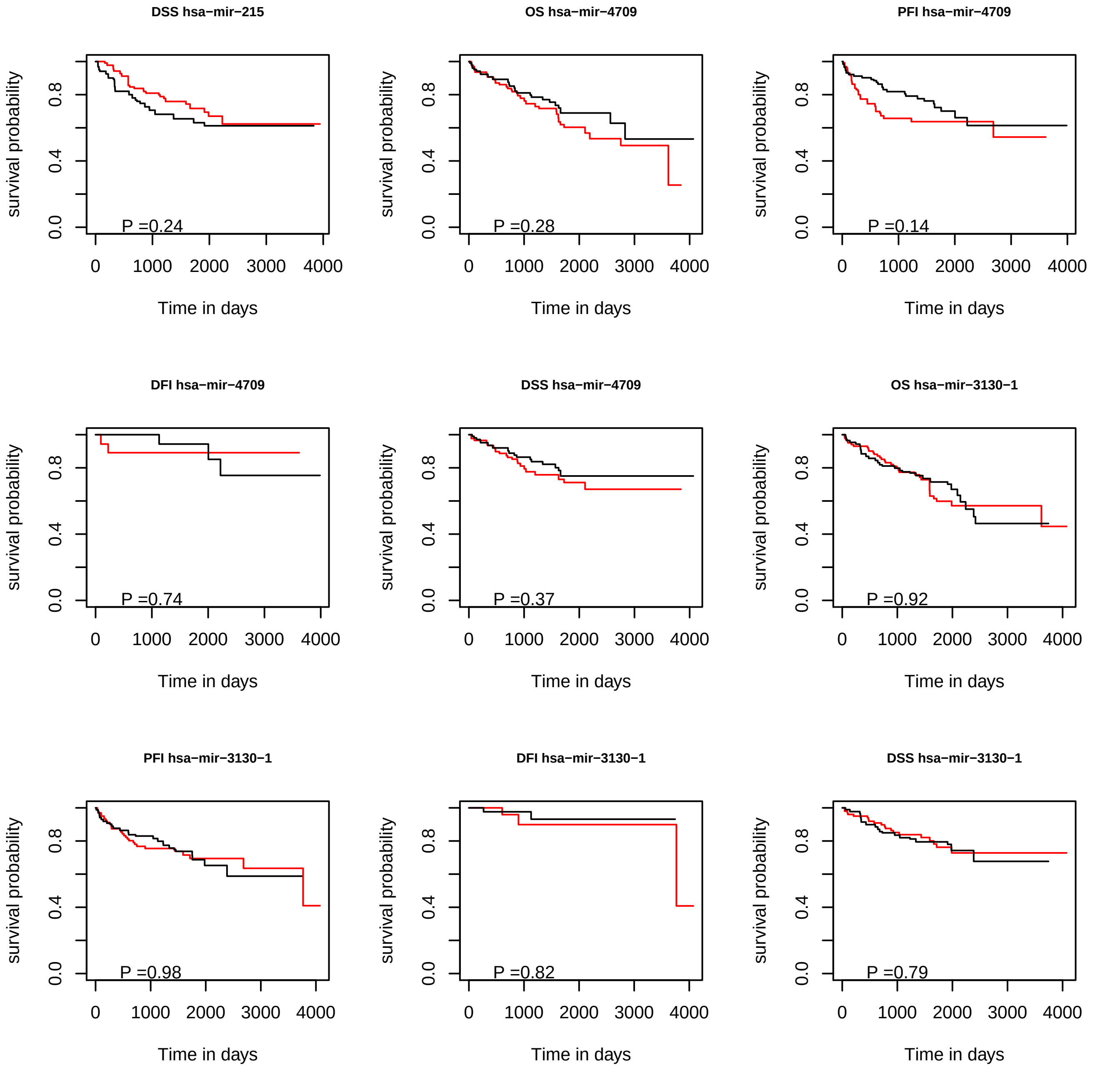
DSS hsa−mir−215
P =0.24
OS hsa−mir−4709
P =0.28
PFI hsa−mir−4709
P =0.14
DFI hsa−mir−4709
P =0.74
DSS hsa−mir−4709
P =0.37
OS hsa−mir−3130−1
P =0.92
PFI hsa−mir−3130−1
P =0.98
DFI hsa−mir−3130−1
P =0.82
DSS hsa−mir−3130−1
P =0.79
survival probability
0.0
0.4
0.8
Time in days
0
1000
2000
3000
4000

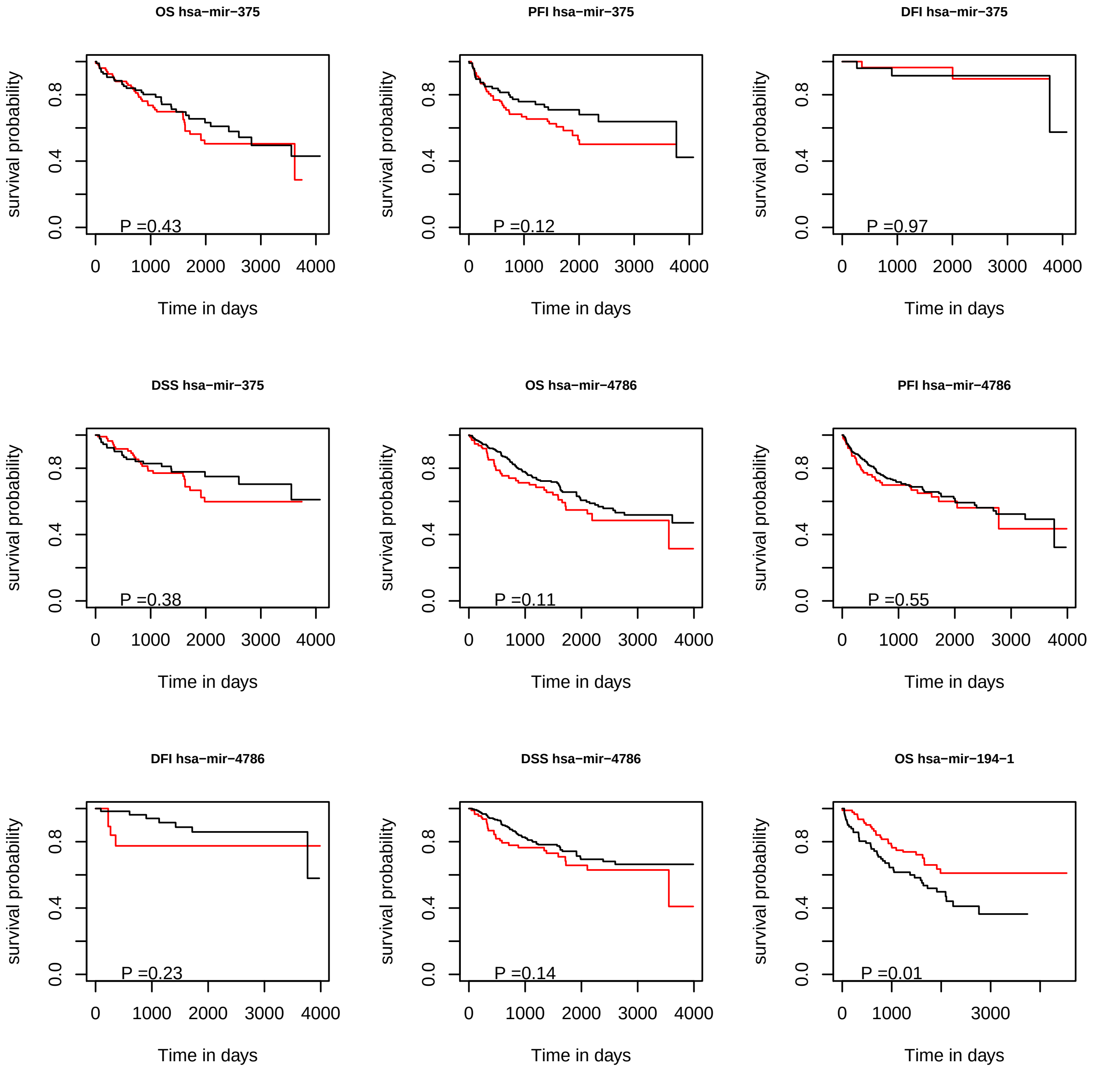

OS hsa−mir−375
P =0.43
PFI hsa−mir−375
P =0.12
DFI hsa−mir−375
P =0.97
DSS hsa−mir−375
P =0.38
OS hsa−mir−4786
P =0.11
PFI hsa−mir−4786
P =0.55
DFI hsa−mir−4786
P =0.23
DSS hsa−mir−4786
P =0.14
OS hsa−mir−194−1
P =0.01
survival probability
0.0
0.4
0.8
Time in days
0
1000
2000
3000
4000

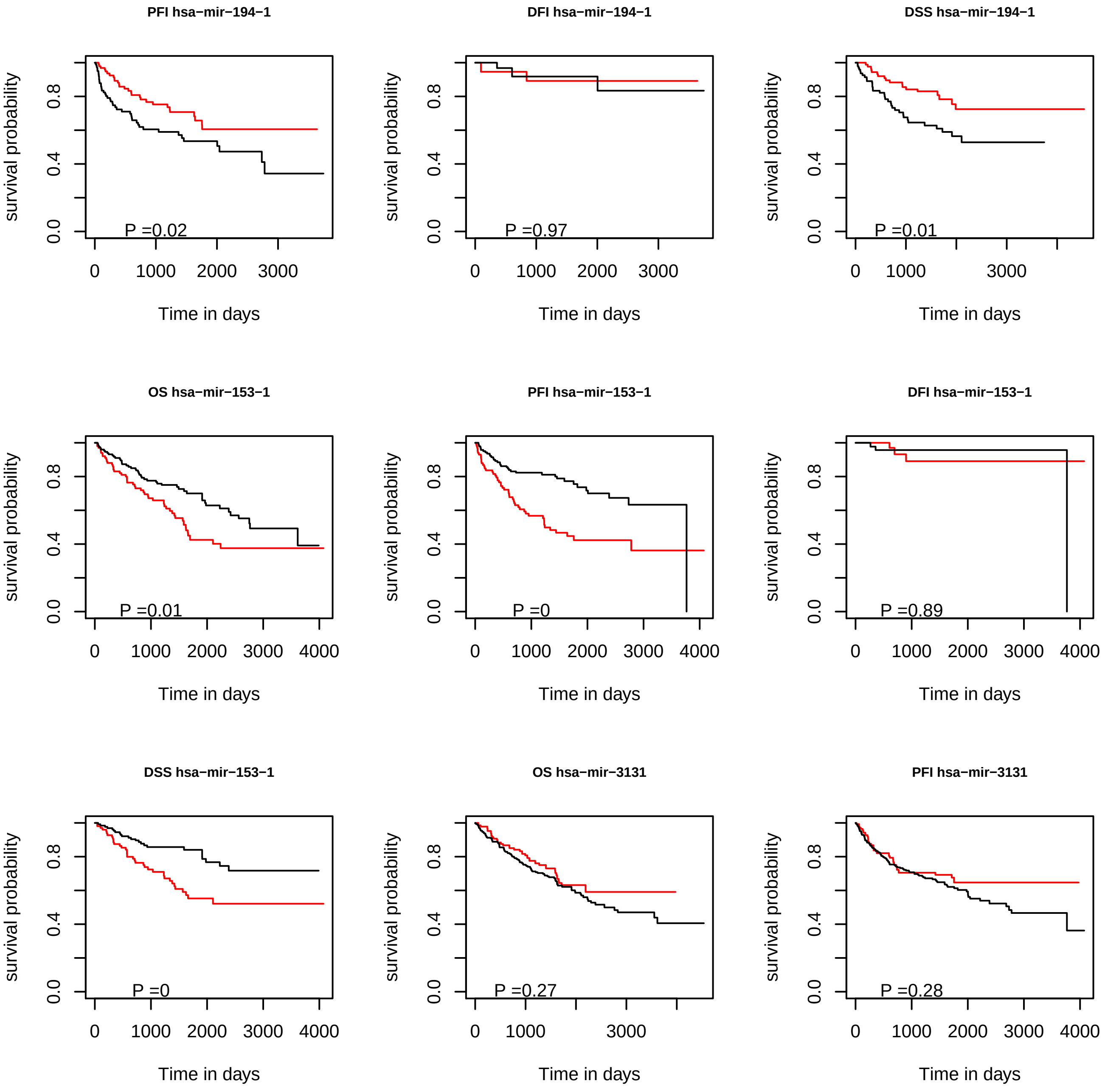
PFI hsa−mir−194−1
P =0.02
DFI hsa−mir−194−1
P =0.97
DSS hsa−mir−194−1
P =0.01
OS hsa−mir−153−1
P =0.01
PFI hsa−mir−153−1
P =0
DFI hsa−mir−153−1
P =0.89
DSS hsa−mir−153−1
P =0
OS hsa−mir−3131
P =0.27
PFI hsa−mir−3131
P =0.28
survival probability
Time in days
0.0
0.4
0.8
0
1000
2000
3000
4000

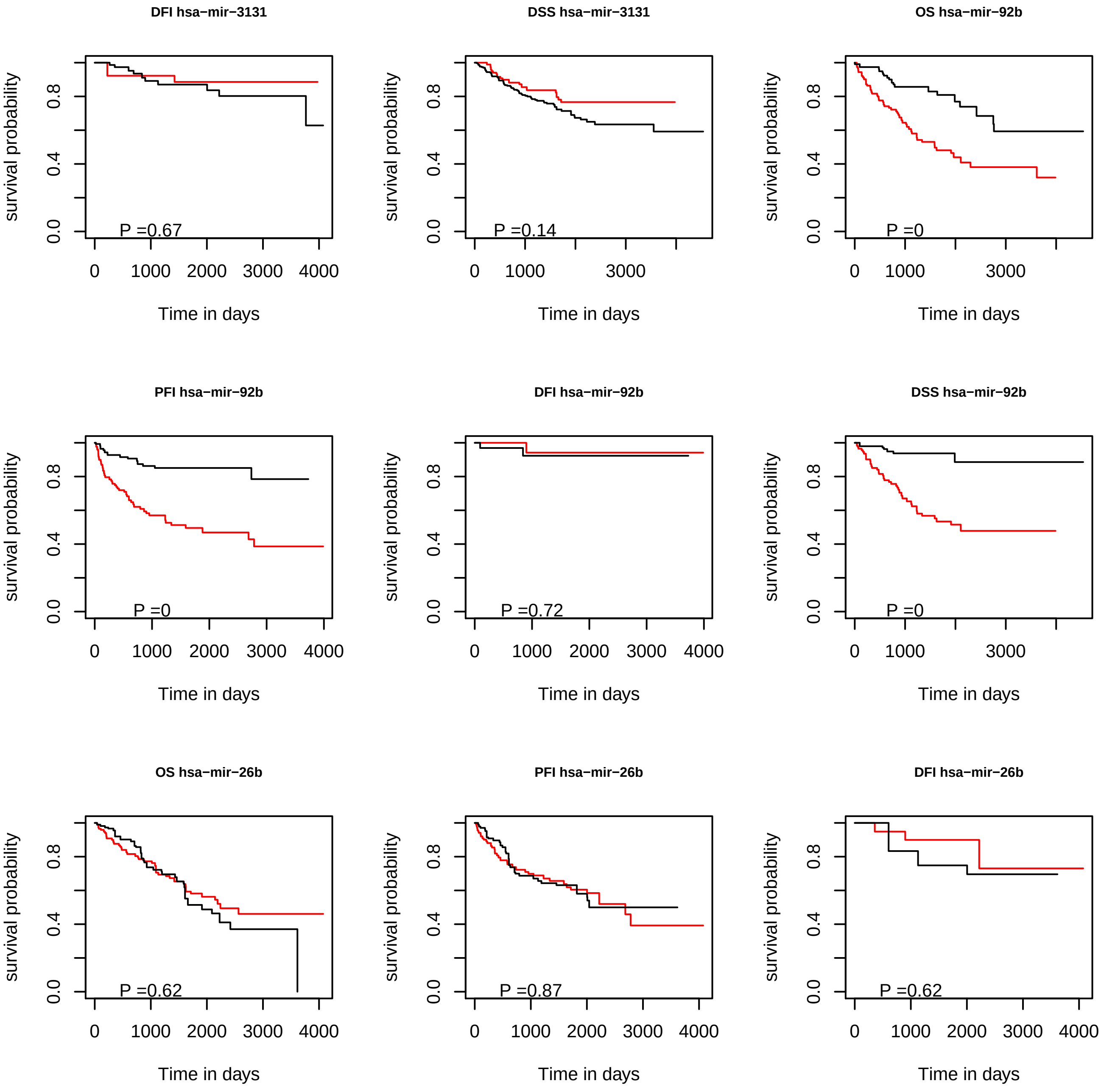
DFI hsa−mir−3131
P =0.67
DSS hsa−mir−3131
P =0.14
OS hsa−mir−92b
P =0
PFI hsa−mir−92b
P =0
DFI hsa−mir−92b
P =0.72
DSS hsa−mir−92b
P =0
OS hsa−mir−26b
P =0.62
PFI hsa−mir−26b
P =0.87
DFI hsa−mir−26b
P =0.62
survival probability
Time in days
0.0
0.4
0.8
0
1000
2000
3000
4000

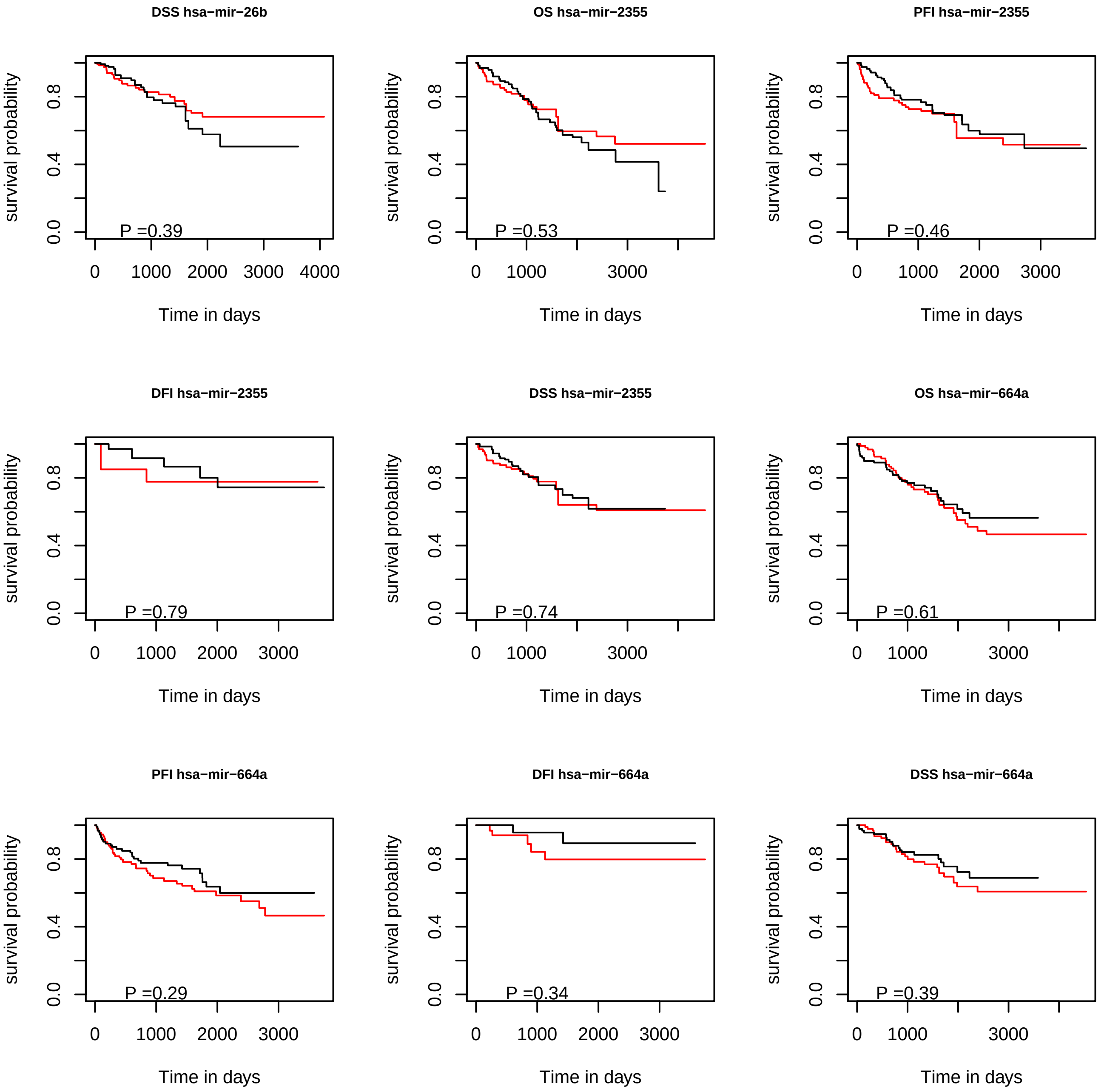
DSS hsa−mir−26b
survival probability
0.0
0.4
0.8
P =0.39
0
1000
2000
3000
4000
Time in days
OS hsa−mir−2355
survival probability
0.0
0.4
0.8
P =0.53
0
1000
3000
Time in days
PFI hsa−mir−2355
survival probability
0.0
0.4
0.8
P =0.46
0
1000
2000
3000
Time in days
DFI hsa−mir−2355
survival probability
0.0
0.4
0.8
P =0.79
0
1000
2000
3000
Time in days
DSS hsa−mir−2355
survival probability
0.0
0.4
0.8
P =0.74
0
1000
3000
Time in days
OS hsa−mir−664a
survival probability
0.0
0.4
0.8
P =0.61
0
1000
3000
Time in days
PFI hsa−mir−664a
survival probability
0.0
0.4
0.8
P =0.29
0
1000
2000
3000
Time in days
DFI hsa−mir−664a
survival probability
0.0
0.4
0.8
P =0.34
0
1000
2000
3000
Time in days
DSS hsa−mir−664a
survival probability
0.0
0.4
0.8
P =0.39
0
1000
3000
Time in days

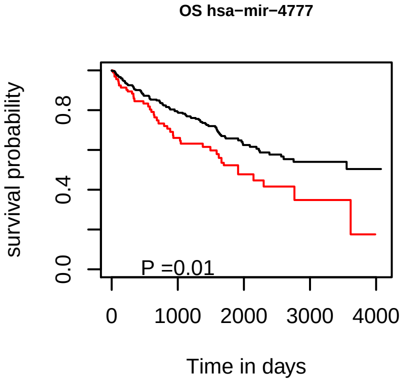
OS hsa−mir−4777
survival probability
0.0
0.4
0.8
P =0.01
0
1000
2000
3000
4000
Time in days

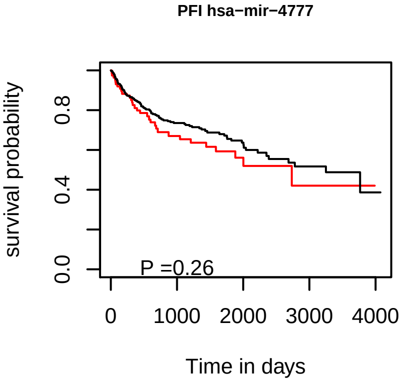
PFI hsa−mir−4777
survival probability
0.0
0.4
0.8
P =0.26
0
1000
2000
3000
4000
Time in days

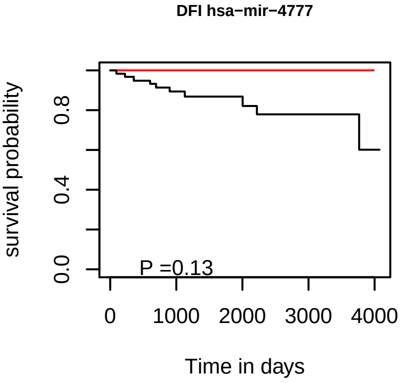
DFI hsa−mir−4777
survival probability
0.0
0.4
0.8
P =0.13
0
1000
2000
3000
4000
Time in days

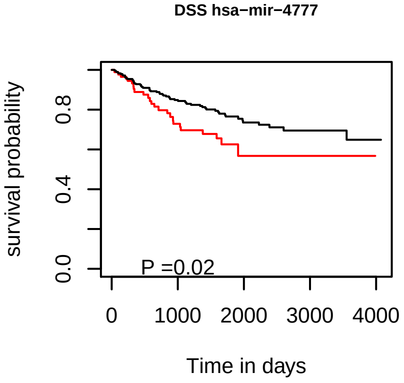
DSS hsa−mir−4777
survival probability
0.0
0.4
0.8
P =0.02
0
1000
2000
3000
4000
Time in days
