# Supplementary material for: Computing microRNA-gene interaction networks in pan-cancer using miRDriver
Source: Sci Rep. 2022 Mar 8;12:3717. doi: 10.1038/s41598-022-07628-z (PMC8904490; doi:10.1038/s41598-022-07628-z)
Supplement: Supplementary file 18 — Supplementary Information 18. [file 41598_2022_7628_MOESM18_ESM.pdf]

Computing microRNA-gene interaction networks in pan-cancer using miRDriver

Banabithi Bose, Matthew Moravec, and Serdar Bozdag

Supplemental Figure S9

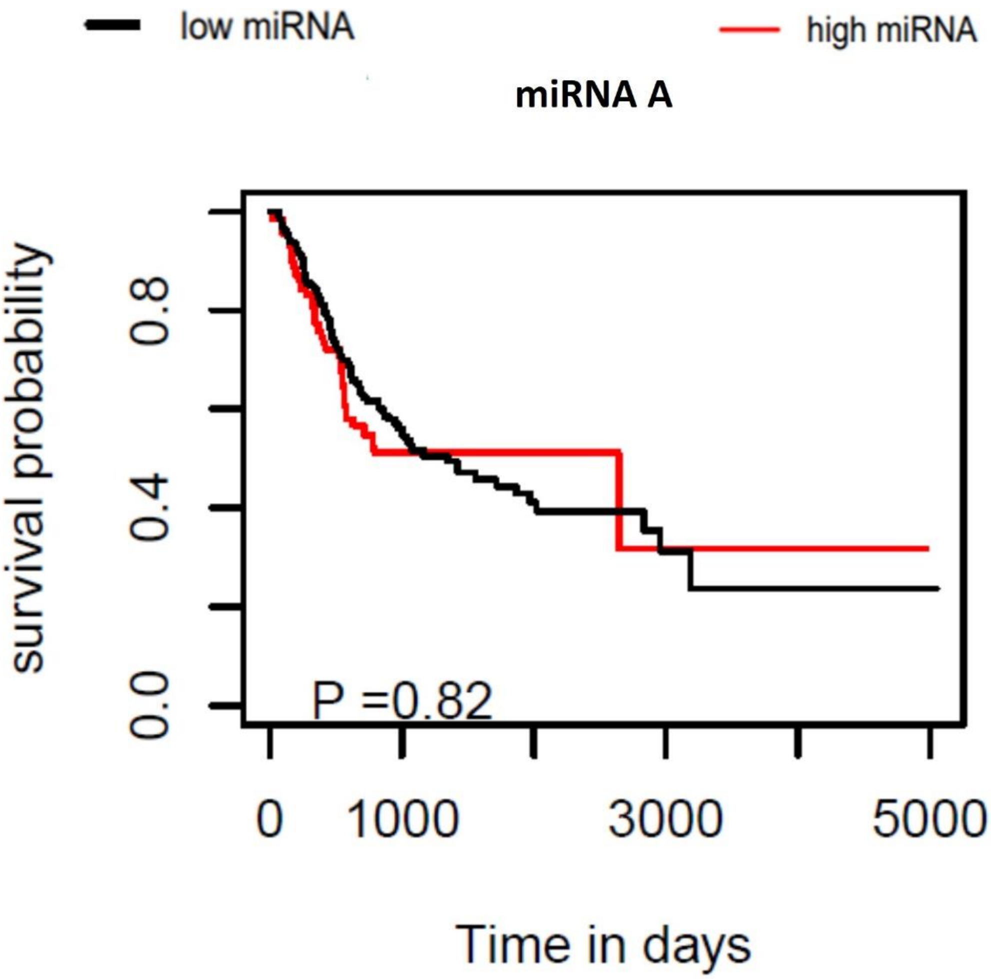

The *Adjusted Kaplan-Meier* survival plots for the computed miRNAs in high and low miRNA expression patient groups.

Supplemental Figure S9 Cancer Type: LAML

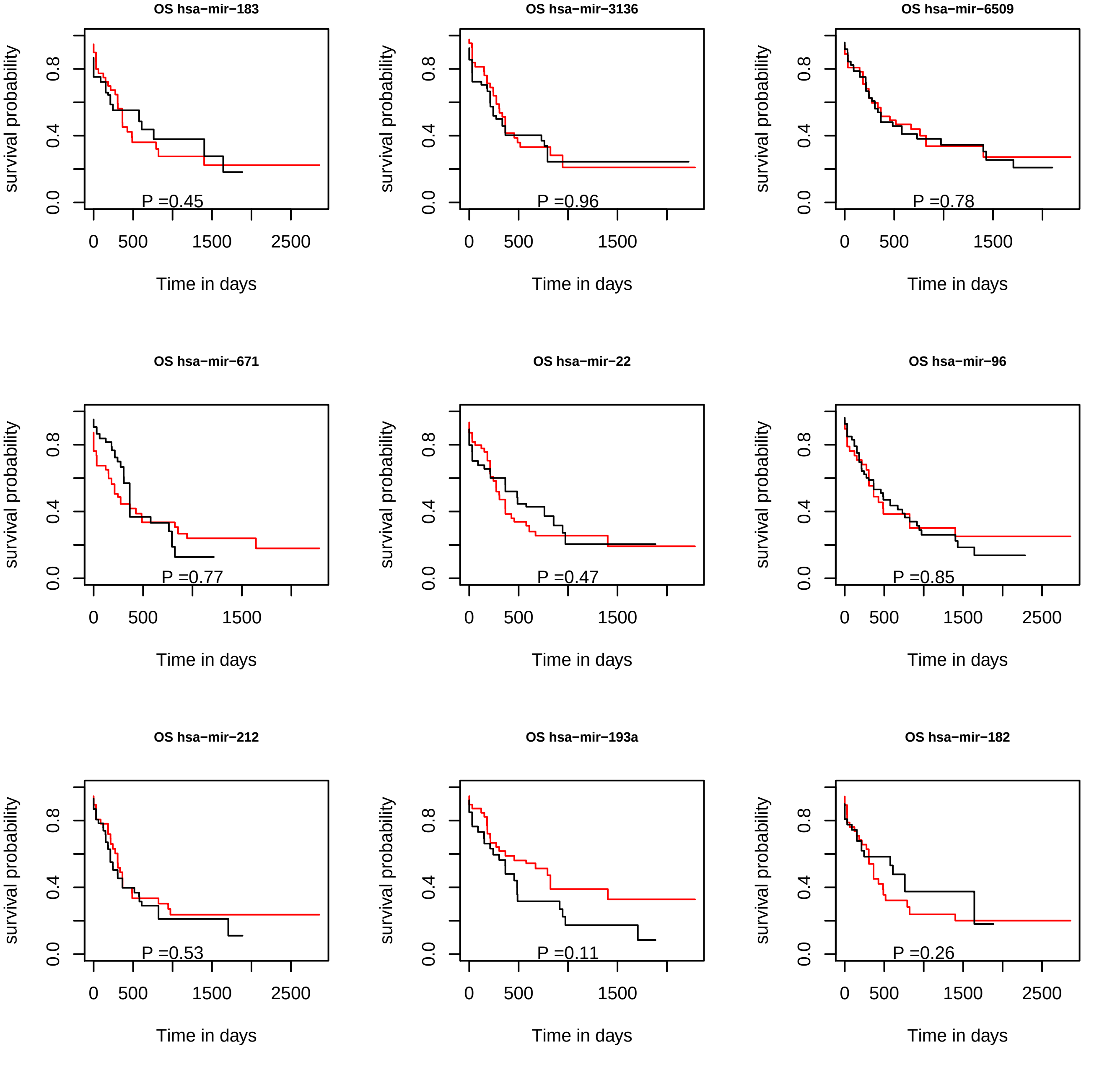

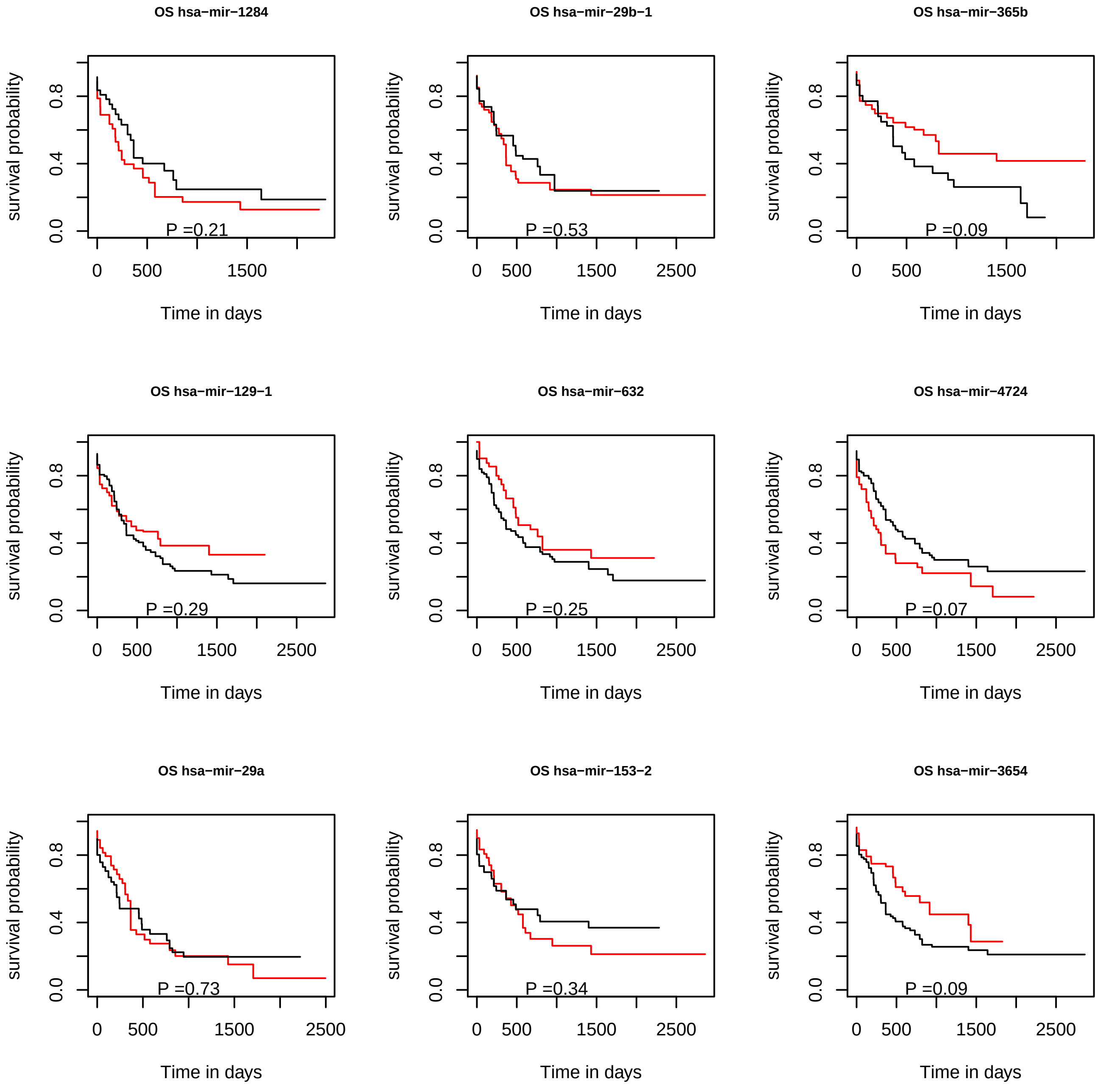
OS hsa−mir−1284
survival probability
0.0
0.4
0.8
P =0.21
0
500
1500
Time in days
OS hsa−mir−29b−1
survival probability
0.0
0.4
0.8
P =0.53
0
500
1500
2500
Time in days
OS hsa−mir−365b
survival probability
0.0
0.4
0.8
P =0.09
0
500
1500
Time in days
OS hsa−mir−129−1
survival probability
0.0
0.4
0.8
P =0.29
0
500
1500
2500
Time in days
OS hsa−mir−632
survival probability
0.0
0.4
0.8
P =0.25
0
500
1500
2500
Time in days
OS hsa−mir−4724
survival probability
0.0
0.4
0.8
P =0.07
0
500
1500
2500
Time in days
OS hsa−mir−29a
survival probability
0.0
0.4
0.8
P =0.73
0
500
1500
2500
Time in days
OS hsa−mir−153−2
survival probability
0.0
0.4
0.8
P =0.34
0
500
1500
2500
Time in days
OS hsa−mir−3654
survival probability
0.0
0.4
0.8
P =0.09
0
500
1500
2500
Time in days

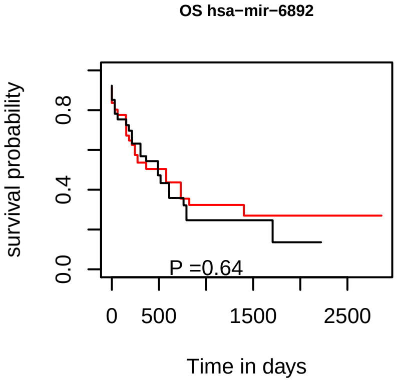
OS hsa−mir−6892
survival probability
0.0
0.4
0.8
P =0.64
0
500
1500
2500
Time in days

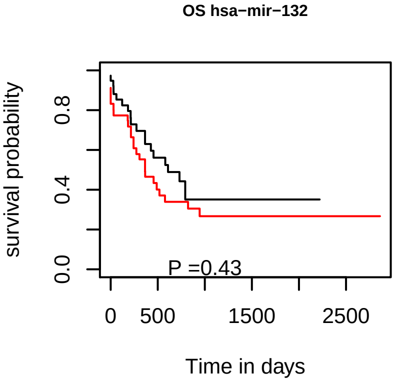
OS hsa−mir−132
survival probability
0.0
0.4
0.8
P =0.43
0
500
1500
2500
Time in days

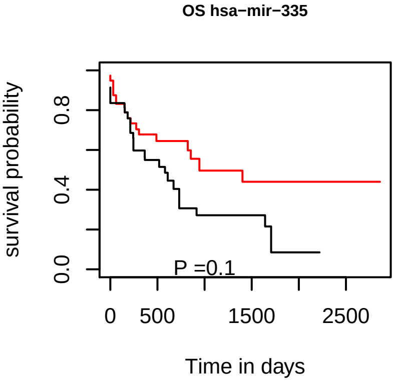
OS hsa−mir−335
survival probability
0.0
0.4
0.8
P =0.1
0
500
1500
2500
Time in days
