# Supplementary material for: Computing microRNA-gene interaction networks in pan-cancer using miRDriver
Source: Sci Rep. 2022 Mar 8;12:3717. doi: 10.1038/s41598-022-07628-z (PMC8904490; doi:10.1038/s41598-022-07628-z)
Supplement: Supplementary file 19 — Supplementary Information 19. [file 41598_2022_7628_MOESM19_ESM.pdf]

Computing microRNA-gene interaction networks in pan-cancer using miRDriver

Banabithi Bose, Matthew Moravec, and Serdar Bozdag

Supplemental Figure S10

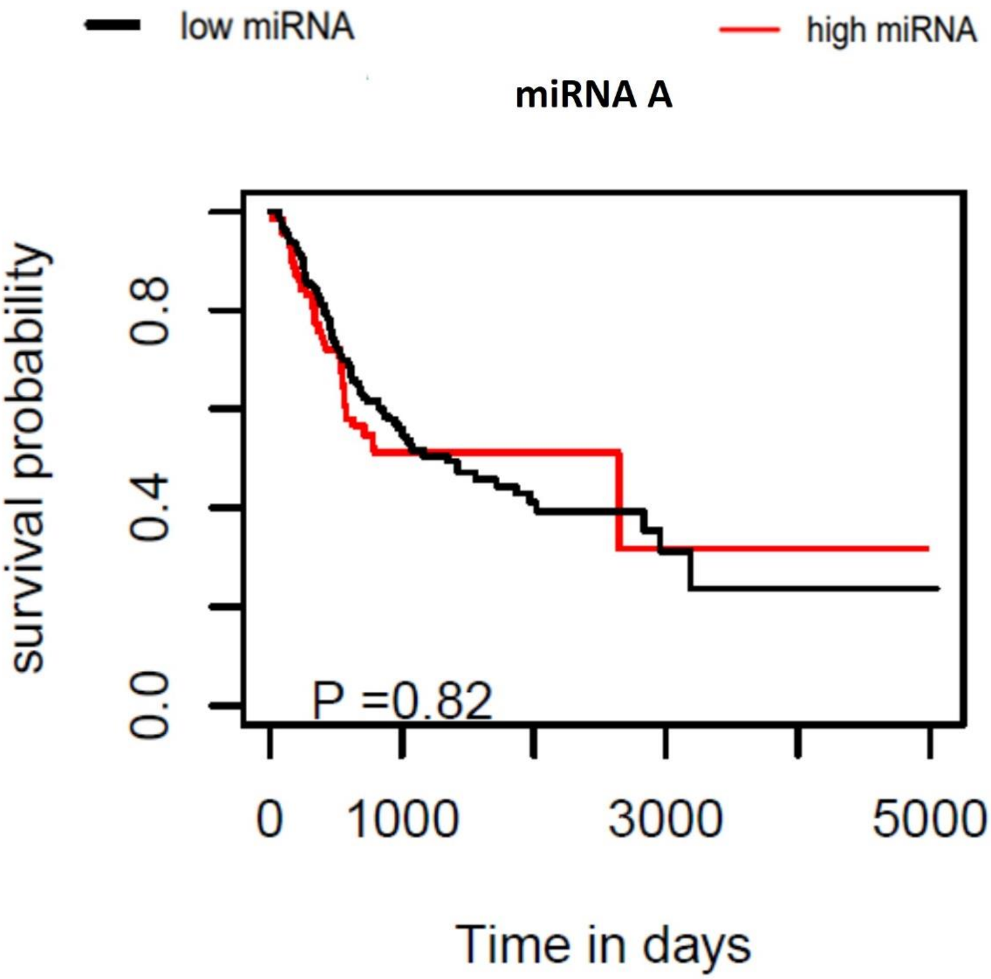

The *Adjusted Kaplan-Meier* survival plots for the computed miRNAs in high and low miRNA expression patient groups.

Supplemental Figure S10 Cancer Type: LGG

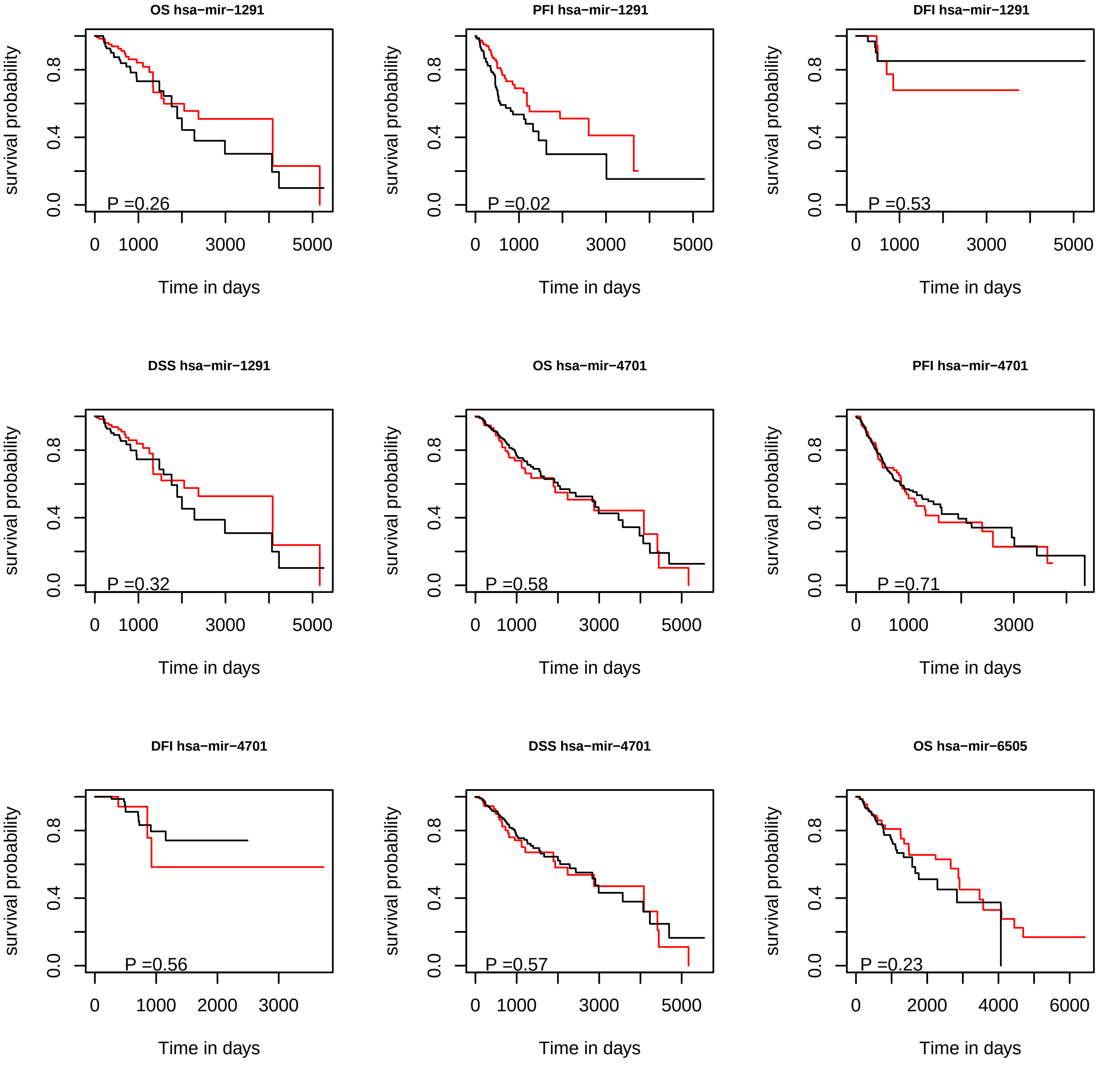

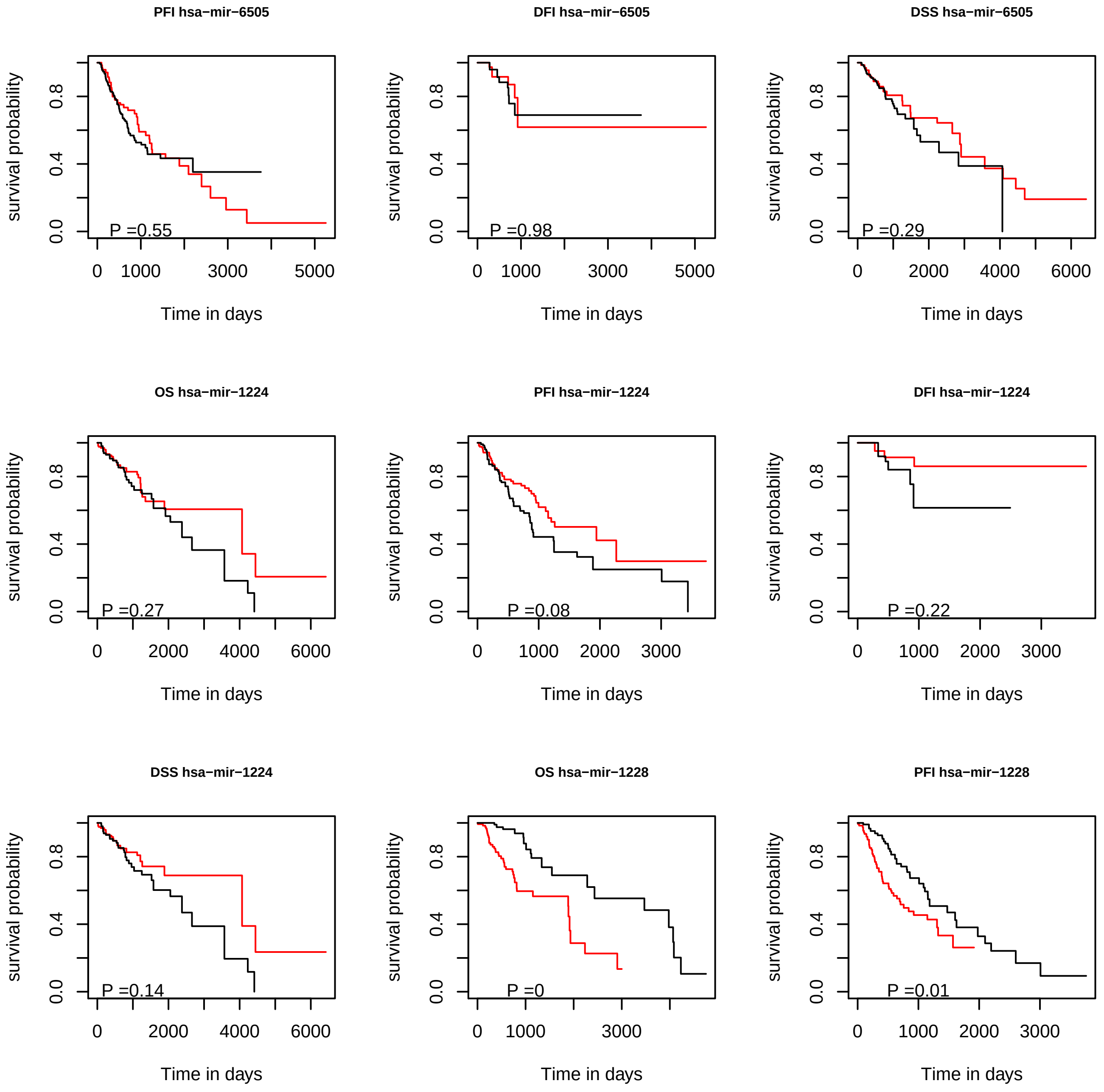
PFI hsa−mir−6505
survival probability
0.0
0.4
0.8
P =0.55
0
1000
3000
5000
Time in days
DFI hsa−mir−6505
P =0.98
DSS hsa−mir−6505
P =0.29
2000
4000
6000
OS hsa−mir−1224
P =0.27
PFI hsa−mir−1224
P =0.08
DFI hsa−mir−1224
P =0.22
DSS hsa−mir−1224
P =0.14
OS hsa−mir−1228
P =0
PFI hsa−mir−1228
P =0.01

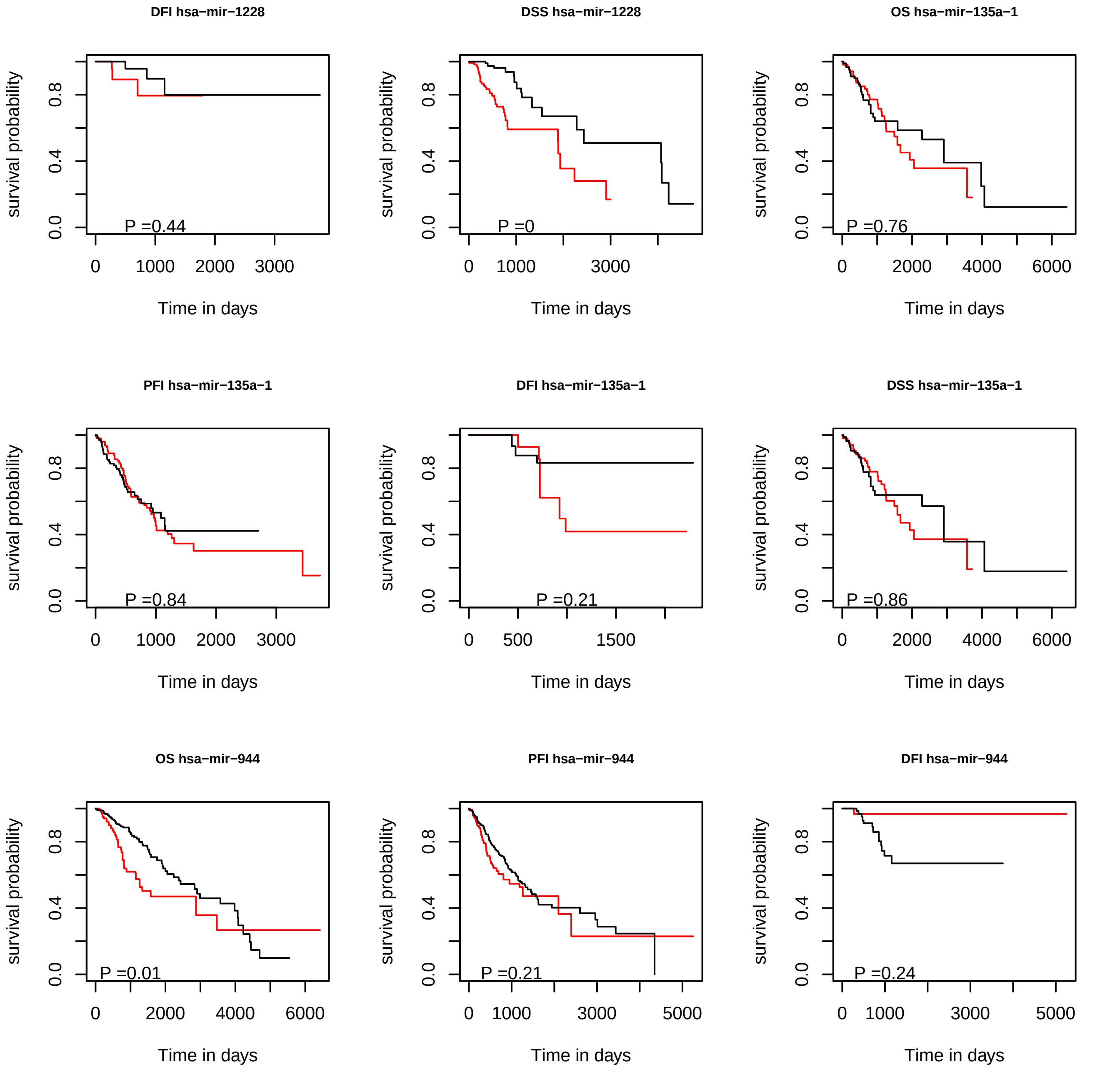
DFI hsa−mir−1228
P =0.44
DSS hsa−mir−1228
P =0
OS hsa−mir−135a−1
P =0.76
PFI hsa−mir−135a−1
P =0.84
DFI hsa−mir−135a−1
P =0.21
DSS hsa−mir−135a−1
P =0.86
OS hsa−mir−944
P =0.01
PFI hsa−mir−944
P =0.21
DFI hsa−mir−944
P =0.24
survival probability
Time in days

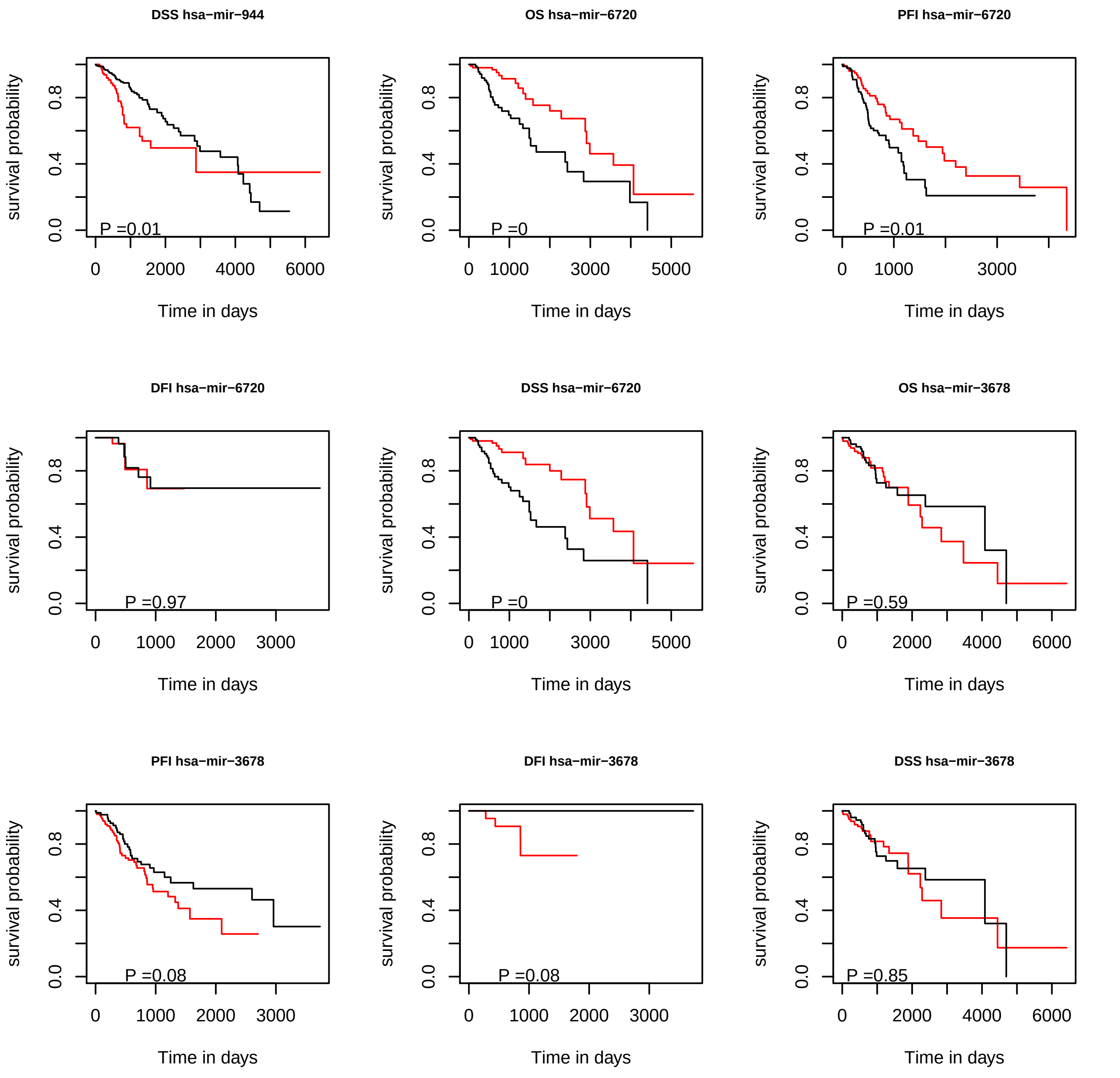
DSS hsa−mir−944
P =0.01
OS hsa−mir−6720
P =0
PFI hsa−mir−6720
P =0.01
DFI hsa−mir−6720
P =0.97
DSS hsa−mir−6720
P =0
OS hsa−mir−3678
P =0.59
PFI hsa−mir−3678
P =0.08
DFI hsa−mir−3678
P =0.08
DSS hsa−mir−3678
P =0.85
survival probability
Time in days
0.0
0.4
0.8
0
1000
2000
3000
4000
5000
6000

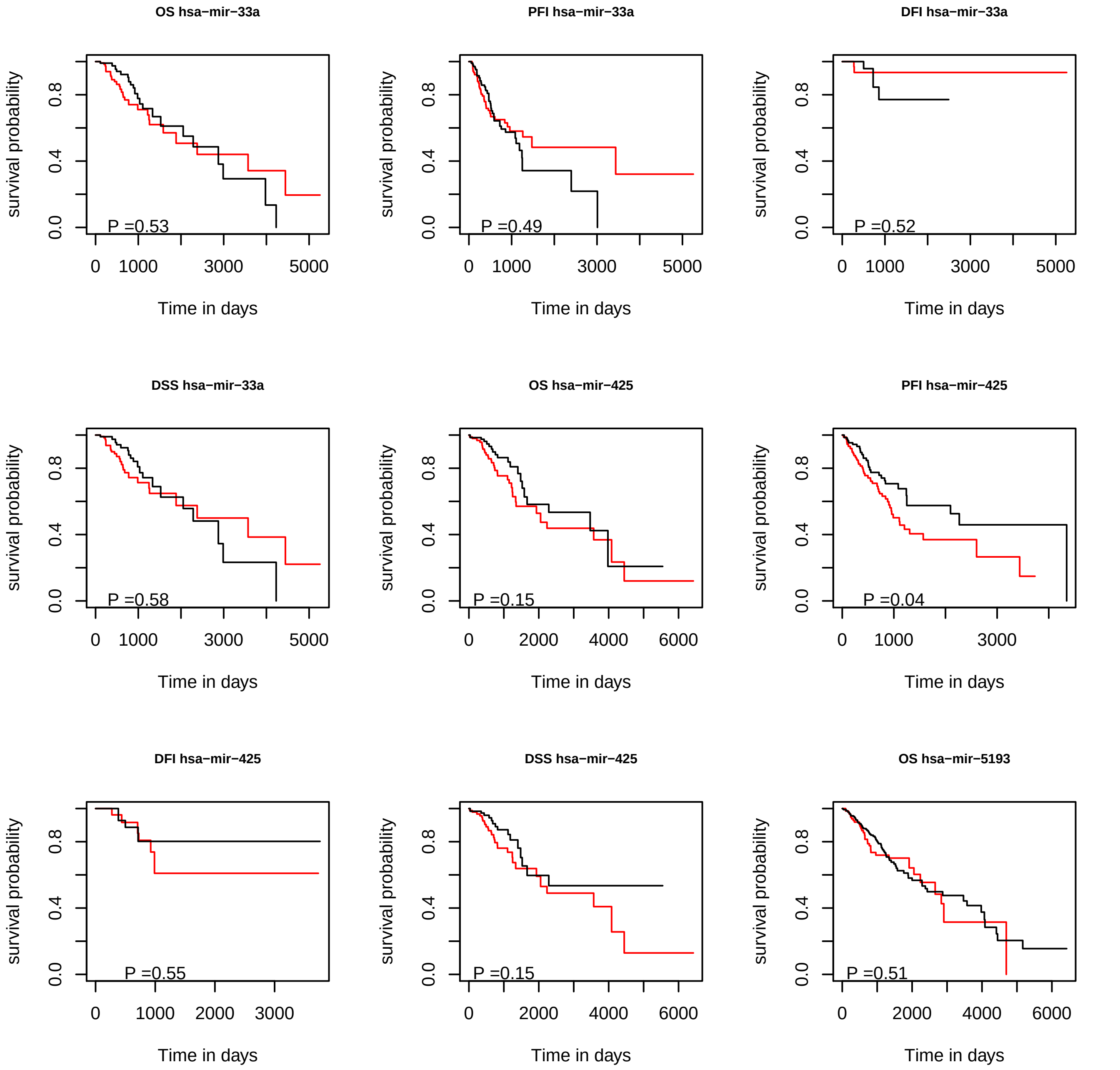
OS hsa−mir−33a
P =0.53
PFI hsa−mir−33a
P =0.49
DFI hsa−mir−33a
P =0.52
DSS hsa−mir−33a
P =0.58
OS hsa−mir−425
P =0.15
PFI hsa−mir−425
P =0.04
DFI hsa−mir−425
P =0.55
DSS hsa−mir−425
P =0.15
OS hsa−mir−5193
P =0.51
survival probability
Time in days

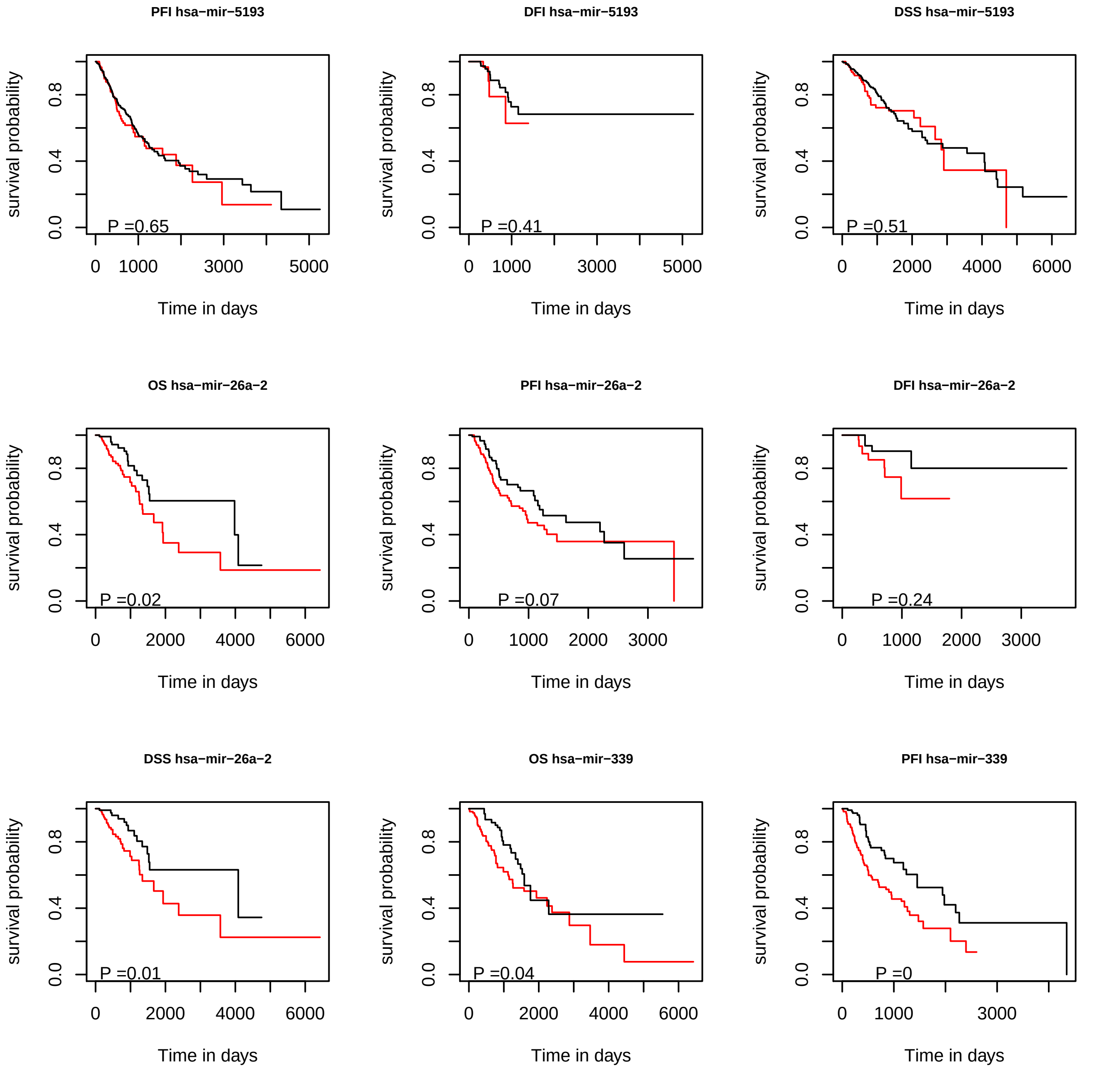
PFI hsa−mir−5193
survival probability
0.0 0.4 0.8
P =0.65
0 1000 3000 5000
Time in days
DFI hsa−mir−5193
survival probability
0.0 0.4 0.8
P =0.41
0 1000 3000 5000
Time in days
DSS hsa−mir−5193
survival probability
0.0 0.4 0.8
P =0.51
0 2000 4000 6000
Time in days
OS hsa−mir−26a−2
survival probability
0.0 0.4 0.8
P =0.02
0 2000 4000 6000
Time in days
PFI hsa−mir−26a−2
survival probability
0.0 0.4 0.8
P =0.07
0 1000 2000 3000
Time in days
DFI hsa−mir−26a−2
survival probability
0.0 0.4 0.8
P =0.24
0 1000 2000 3000
Time in days
DSS hsa−mir−26a−2
survival probability
0.0 0.4 0.8
P =0.01
0 2000 4000 6000
Time in days
OS hsa−mir−339
survival probability
0.0 0.4 0.8
P =0.04
0 2000 4000 6000
Time in days
PFI hsa−mir−339
survival probability
0.0 0.4 0.8
P =0
0 1000 3000
Time in days

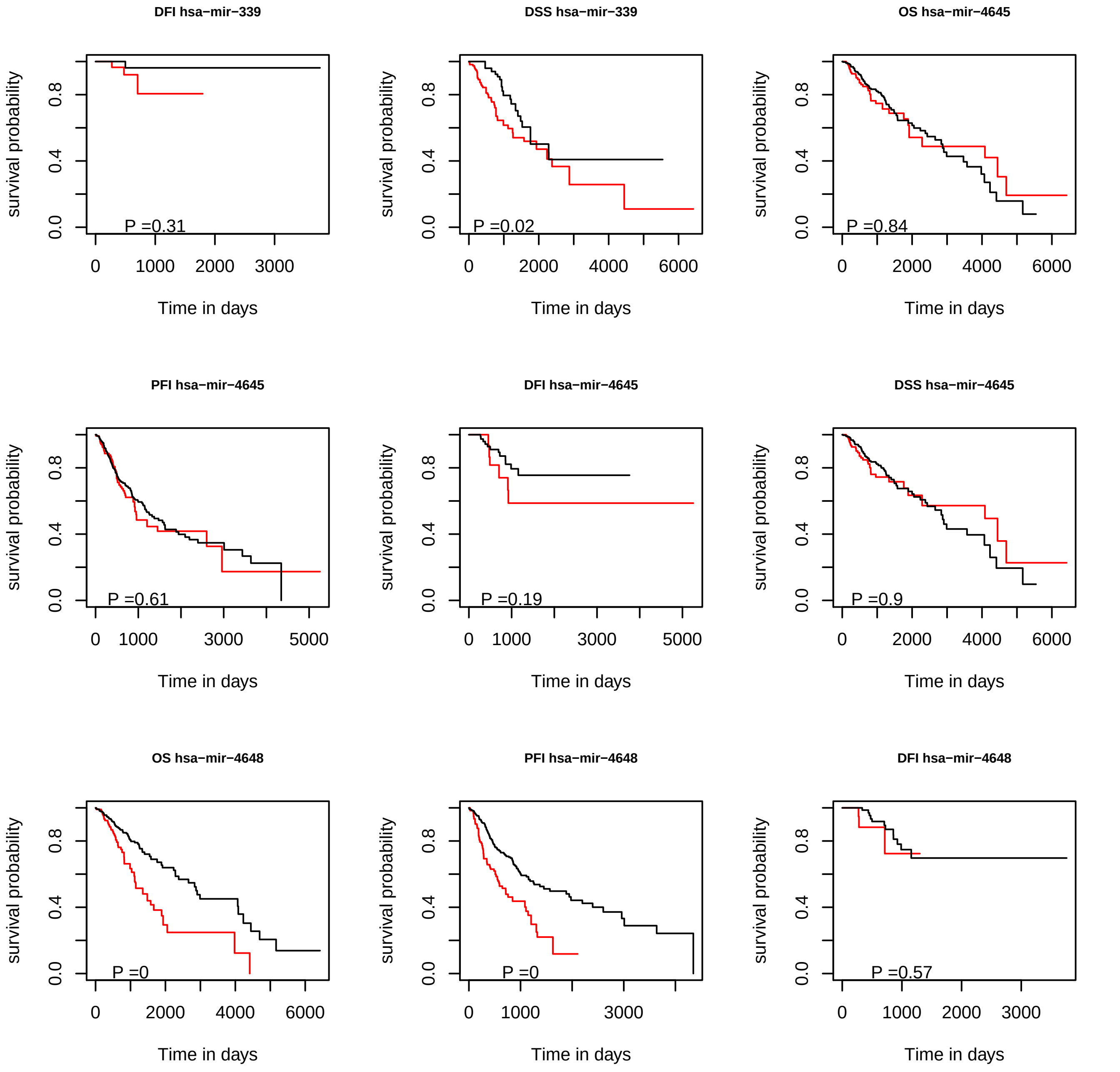

DFI hsa−mir−339
P =0.31
DSS hsa−mir−339
P =0.02
OS hsa−mir−4645
P =0.84
PFI hsa−mir−4645
P =0.61
DFI hsa−mir−4645
P =0.19
DSS hsa−mir−4645
P =0.9
OS hsa−mir−4648
P =0
PFI hsa−mir−4648
P =0
DFI hsa−mir−4648
P =0.57
survival probability
Time in days
0.0
0.4
0.8
0
1000
2000
3000
4000
5000
6000

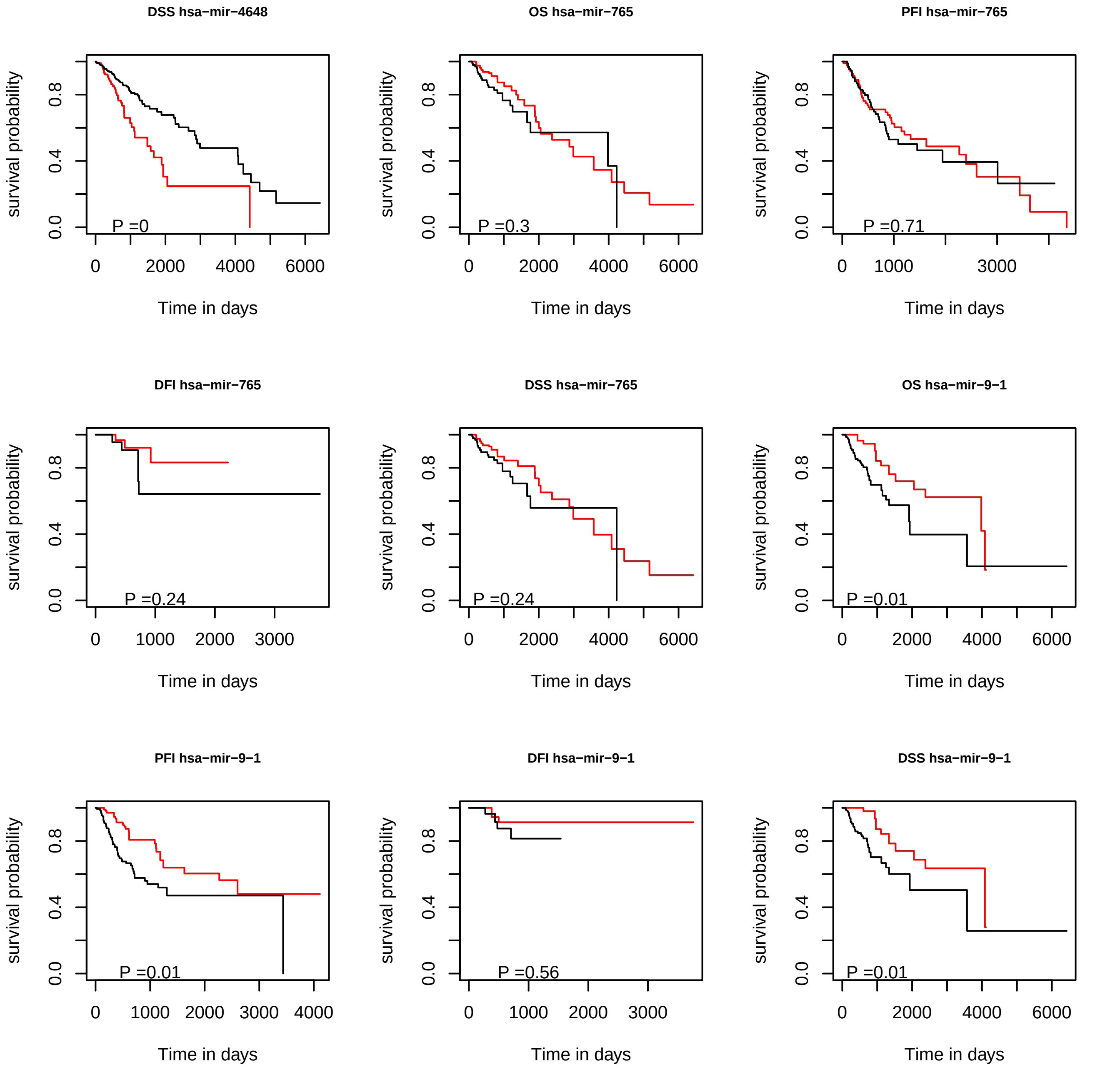
DSS hsa−mir−4648
survival probability
0.0
0.4
0.8
P =0
0
2000
4000
6000
Time in days
OS hsa−mir−765
survival probability
0.0
0.4
0.8
P =0.3
0
2000
4000
6000
Time in days
PFI hsa−mir−765
survival probability
0.0
0.4
0.8
P =0.71
0
1000
3000
Time in days
DFI hsa−mir−765
survival probability
0.0
0.4
0.8
P =0.24
0
1000
2000
3000
Time in days
DSS hsa−mir−765
survival probability
0.0
0.4
0.8
P =0.24
0
2000
4000
6000
Time in days
OS hsa−mir−9−1
survival probability
0.0
0.4
0.8
P =0.01
0
2000
4000
6000
Time in days
PFI hsa−mir−9−1
survival probability
0.0
0.4
0.8
P =0.01
0
1000
2000
3000
4000
Time in days
DFI hsa−mir−9−1
survival probability
0.0
0.4
0.8
P =0.56
0
1000
2000
3000
Time in days
DSS hsa−mir−9−1
survival probability
0.0
0.4
0.8
P =0.01
0
2000
4000
6000
Time in days

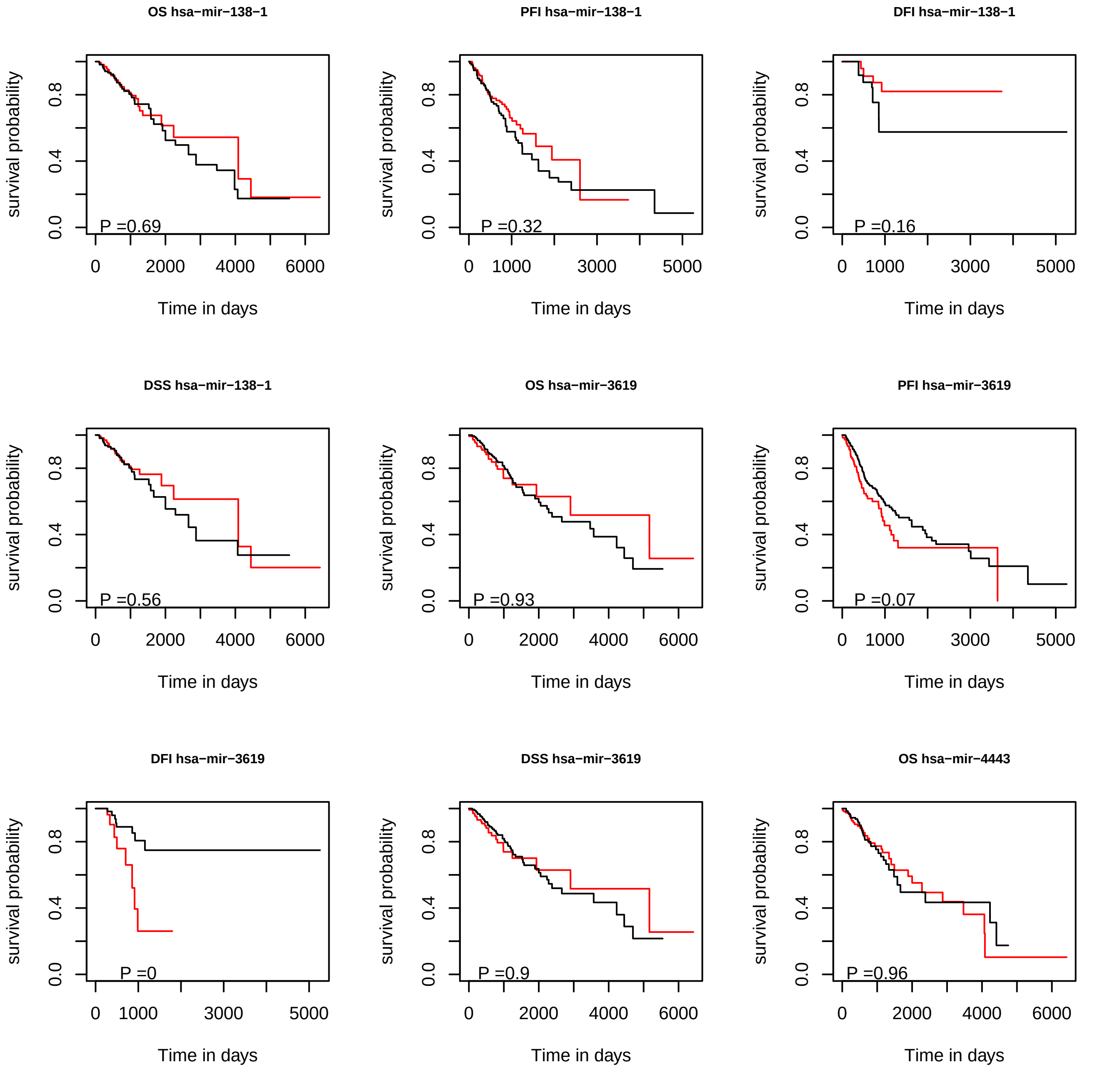
OS hsa−mir−138−1
survival probability
0.0
0.4
0.8
P =0.69
0
2000
4000
6000
Time in days
PFI hsa−mir−138−1
survival probability
0.0
0.4
0.8
P =0.32
0
1000
3000
5000
Time in days
DFI hsa−mir−138−1
survival probability
0.0
0.4
0.8
P =0.16
0
1000
3000
5000
Time in days
DSS hsa−mir−138−1
survival probability
0.0
0.4
0.8
P =0.56
0
2000
4000
6000
Time in days
OS hsa−mir−3619
survival probability
0.0
0.4
0.8
P =0.93
0
2000
4000
6000
Time in days
PFI hsa−mir−3619
survival probability
0.0
0.4
0.8
P =0.07
0
1000
3000
5000
Time in days
DFI hsa−mir−3619
survival probability
0.0
0.4
0.8
P =0
0
1000
3000
5000
Time in days
DSS hsa−mir−3619
survival probability
0.0
0.4
0.8
P =0.9
0
2000
4000
6000
Time in days
OS hsa−mir−4443
survival probability
0.0
0.4
0.8
P =0.96
0
2000
4000
6000
Time in days

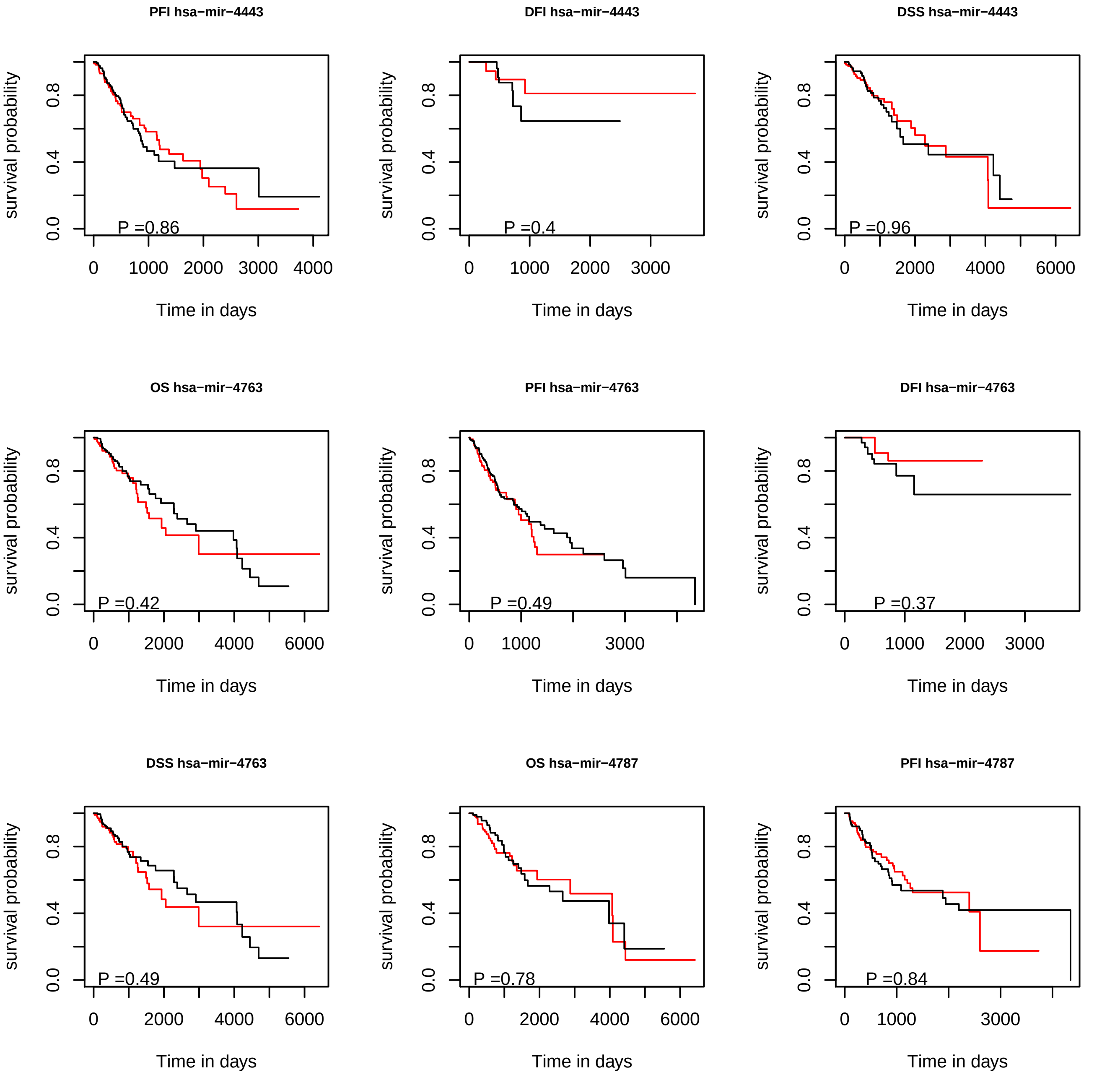
PFI hsa−mir−4443
P =0.86
DFI hsa−mir−4443
P =0.4
DSS hsa−mir−4443
P =0.96
OS hsa−mir−4763
P =0.42
PFI hsa−mir−4763
P =0.49
DFI hsa−mir−4763
P =0.37
DSS hsa−mir−4763
P =0.49
OS hsa−mir−4787
P =0.78
PFI hsa−mir−4787
P =0.84
survival probability
Time in days

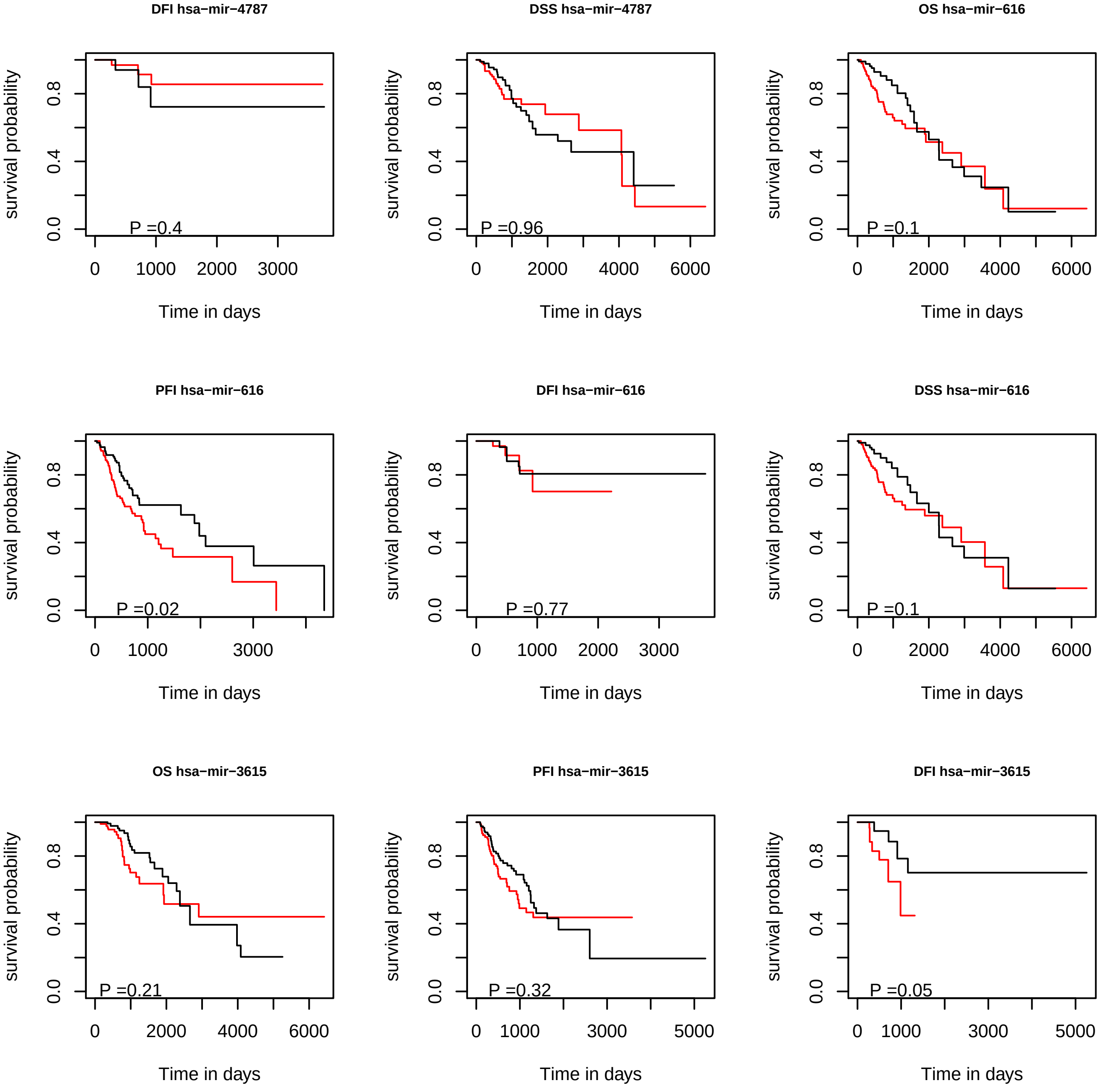
DFI hsa−mir−4787
P =0.4
DSS hsa−mir−4787
P =0.96
OS hsa−mir−616
P =0.1
PFI hsa−mir−616
P =0.02
DFI hsa−mir−616
P =0.77
DSS hsa−mir−616
P =0.1
OS hsa−mir−3615
P =0.21
PFI hsa−mir−3615
P =0.32
DFI hsa−mir−3615
P =0.05
survival probability
Time in days
0.0
0.4
0.8
0
1000
2000
3000
4000
5000
6000

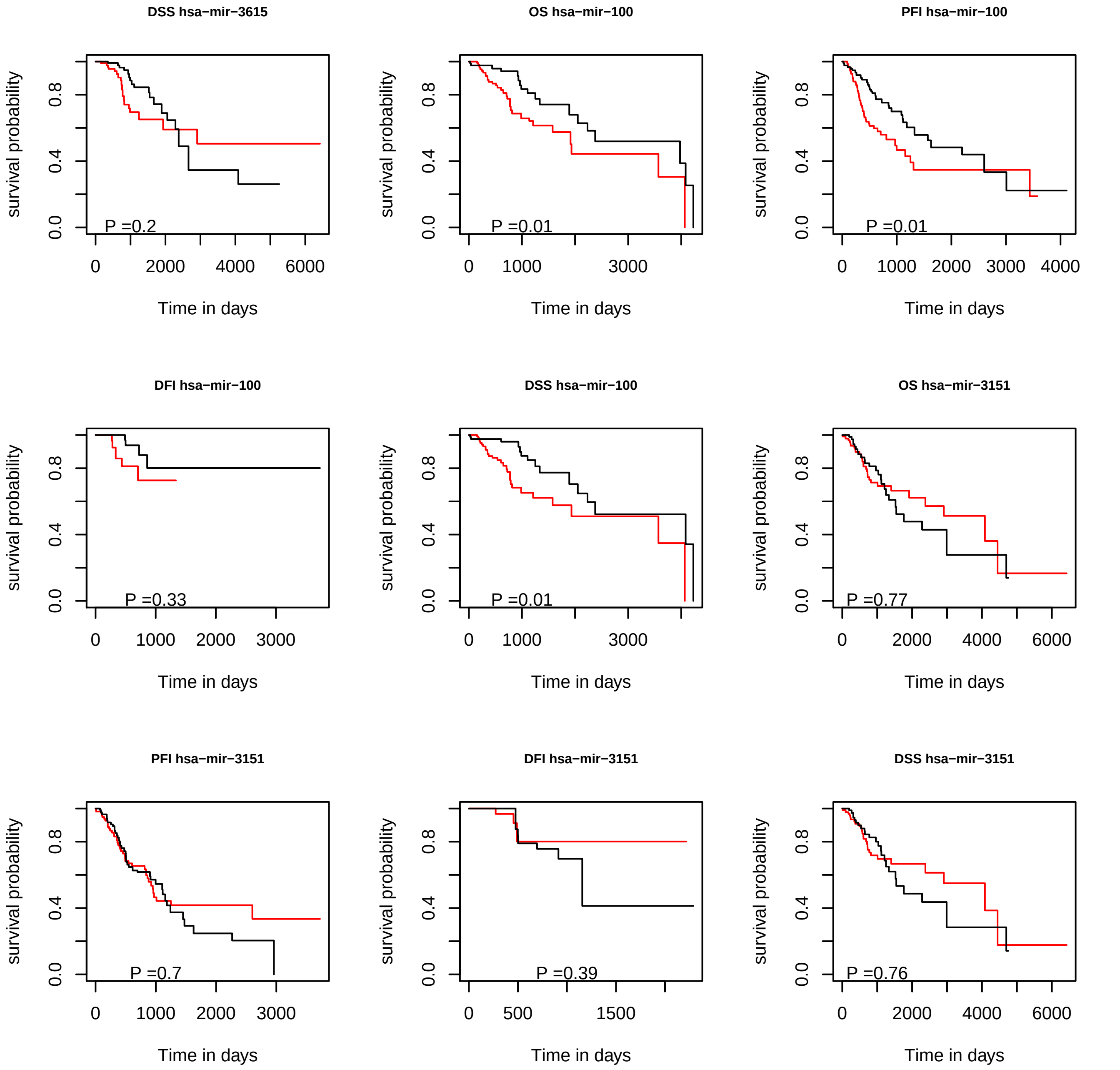
DSS hsa−mir−3615
P =0.2
OS hsa−mir−100
P =0.01
PFI hsa−mir−100
P =0.01
DFI hsa−mir−100
P =0.33
DSS hsa−mir−100
P =0.01
OS hsa−mir−3151
P =0.77
PFI hsa−mir−3151
P =0.7
DFI hsa−mir−3151
P =0.39
DSS hsa−mir−3151
P =0.76
survival probability
Time in days

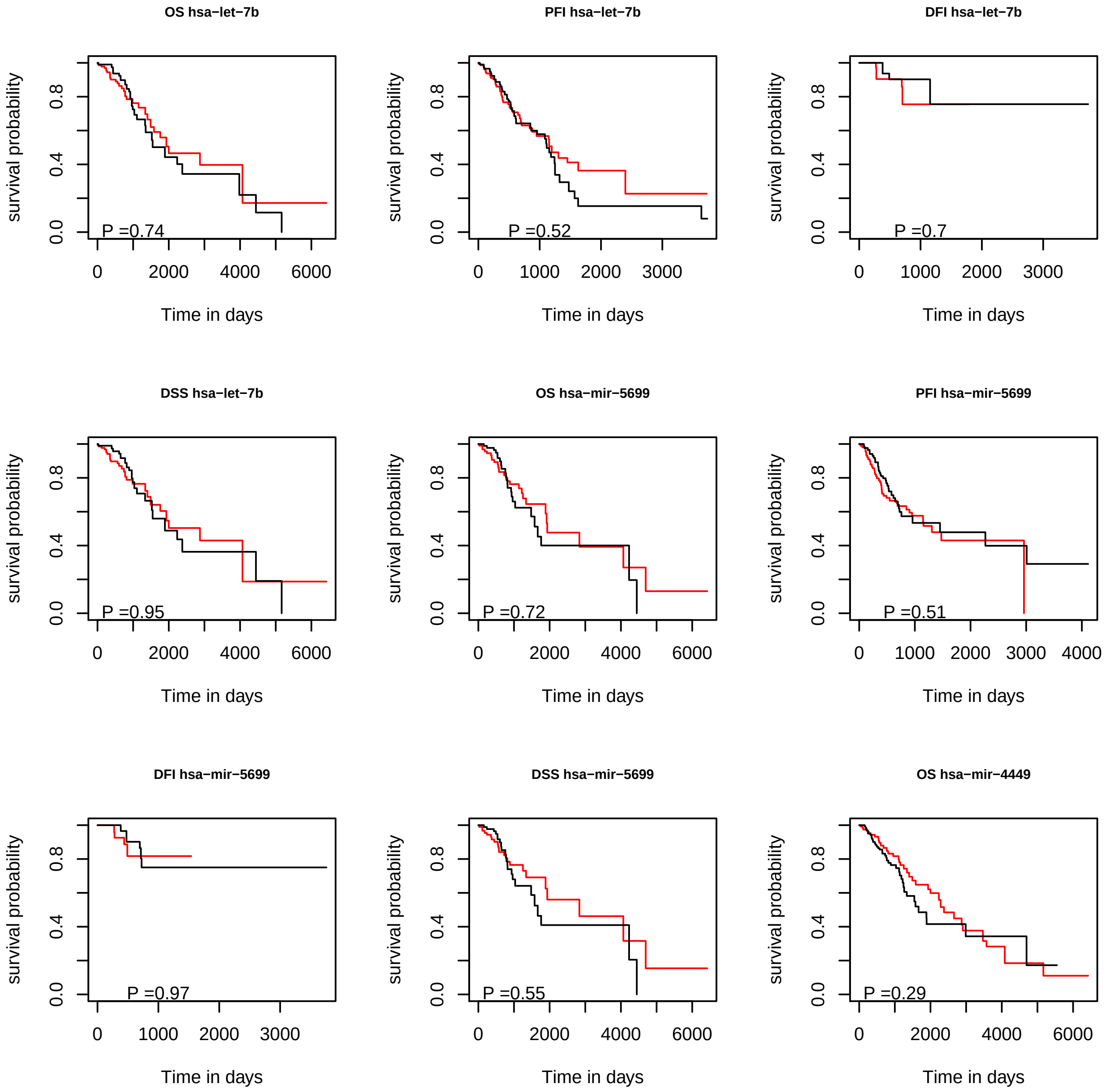
OS hsa−let−7b
P =0.74
PFI hsa−let−7b
P =0.52
DFI hsa−let−7b
P =0.7
DSS hsa−let−7b
P =0.95
OS hsa−mir−5699
P =0.72
PFI hsa−mir−5699
P =0.51
DFI hsa−mir−5699
P =0.97
DSS hsa−mir−5699
P =0.55
OS hsa−mir−4449
P =0.29
survival probability
Time in days

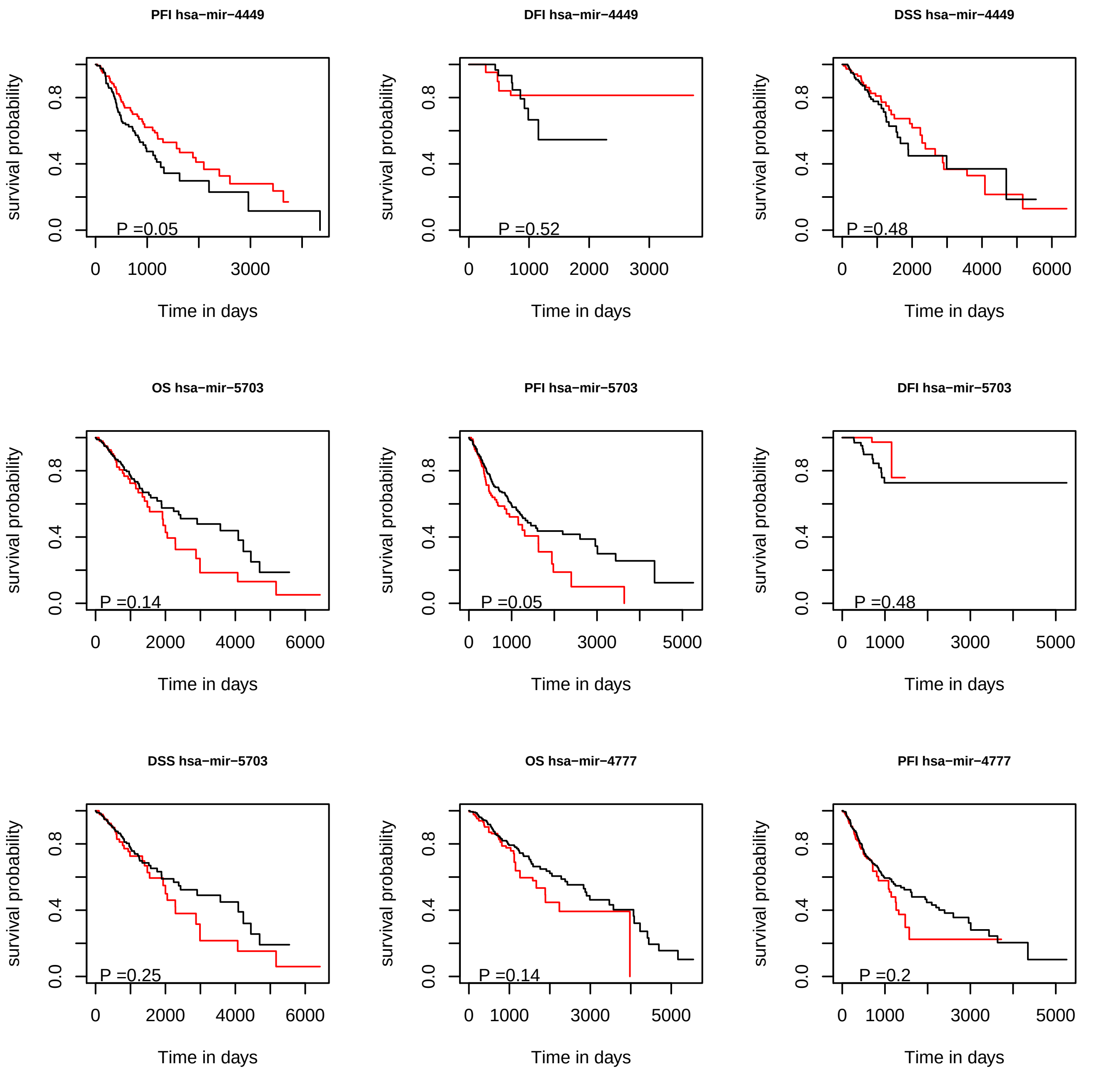
PFI hsa−mir−4449
survival probability
0.0 0.4 0.8
P =0.05
0 1000 3000
Time in days
DFI hsa−mir−4449
survival probability
0.0 0.4 0.8
P =0.52
0 1000 2000 3000
Time in days
DSS hsa−mir−4449
survival probability
0.0 0.4 0.8
P =0.48
0 2000 4000 6000
Time in days
OS hsa−mir−5703
survival probability
0.0 0.4 0.8
P =0.14
0 2000 4000 6000
Time in days
PFI hsa−mir−5703
survival probability
0.0 0.4 0.8
P =0.05
0 1000 3000 5000
Time in days
DFI hsa−mir−5703
survival probability
0.0 0.4 0.8
P =0.48
0 1000 3000 5000
Time in days
DSS hsa−mir−5703
survival probability
0.0 0.4 0.8
P =0.25
0 2000 4000 6000
Time in days
OS hsa−mir−4777
survival probability
0.0 0.4 0.8
P =0.14
0 1000 3000 5000
Time in days
PFI hsa−mir−4777
survival probability
0.0 0.4 0.8
P =0.2
0 1000 3000 5000
Time in days

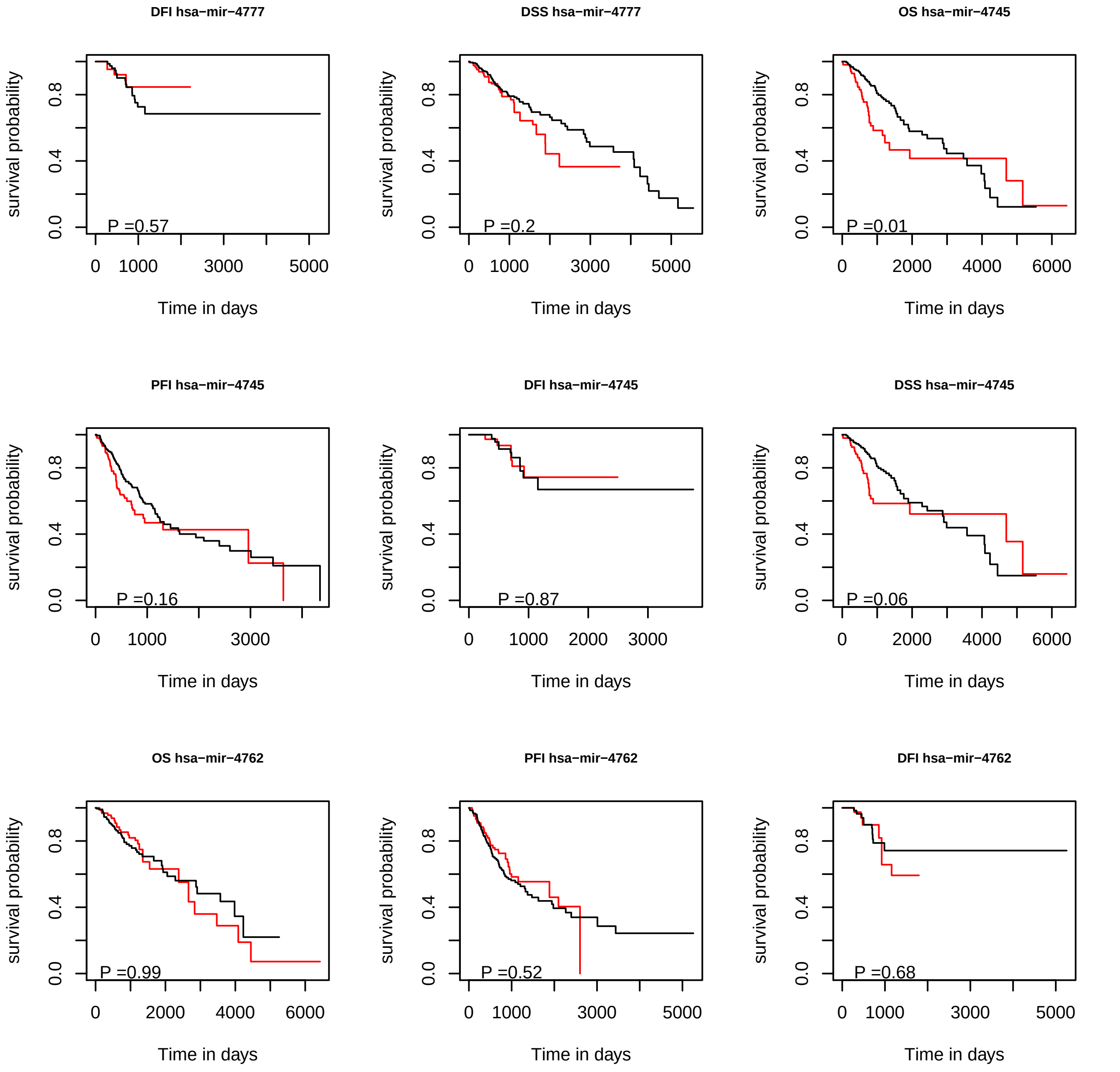
DFI hsa−mir−4777
survival probability
0.0
0.4
0.8
P =0.57
0
1000
3000
5000
Time in days
DSS hsa−mir−4777
survival probability
0.0
0.4
0.8
P =0.2
0
1000
3000
5000
Time in days
OS hsa−mir−4745
survival probability
0.0
0.4
0.8
P =0.01
0
2000
4000
6000
Time in days
PFI hsa−mir−4745
survival probability
0.0
0.4
0.8
P =0.16
0
1000
3000
Time in days
DFI hsa−mir−4745
survival probability
0.0
0.4
0.8
P =0.87
0
1000
2000
3000
Time in days
DSS hsa−mir−4745
survival probability
0.0
0.4
0.8
P =0.06
0
2000
4000
6000
Time in days
OS hsa−mir−4762
survival probability
0.0
0.4
0.8
P =0.99
0
2000
4000
6000
Time in days
PFI hsa−mir−4762
survival probability
0.0
0.4
0.8
P =0.52
0
1000
3000
5000
Time in days
DFI hsa−mir−4762
survival probability
0.0
0.4
0.8
P =0.68
0
1000
3000
5000
Time in days

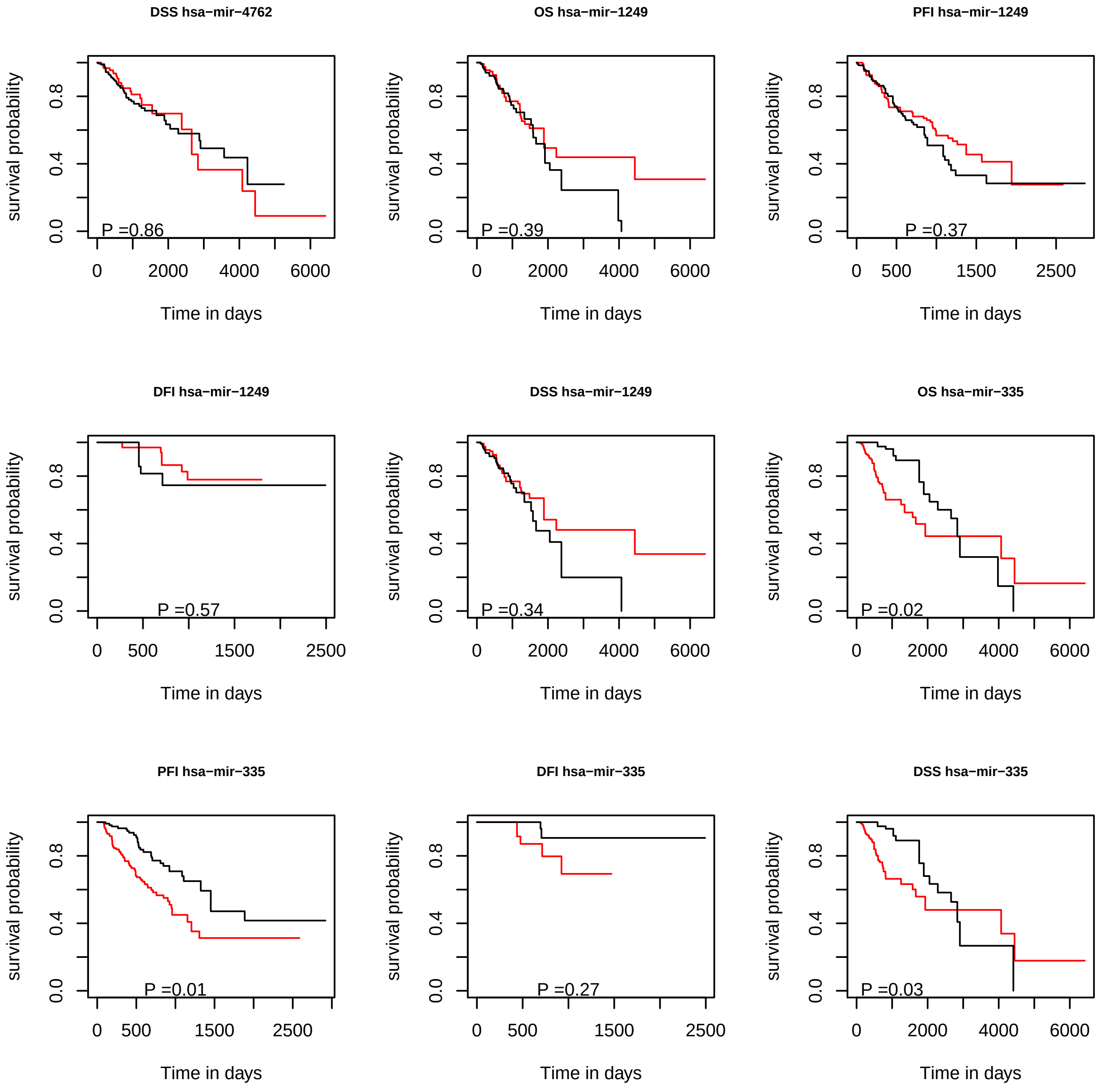
DSS hsa−mir−4762
P =0.86
OS hsa−mir−1249
P =0.39
PFI hsa−mir−1249
P =0.37
DFI hsa−mir−1249
P =0.57
DSS hsa−mir−1249
P =0.34
OS hsa−mir−335
P =0.02
PFI hsa−mir−335
P =0.01
DFI hsa−mir−335
P =0.27
DSS hsa−mir−335
P =0.03
survival probability
Time in days
0.0
0.4
0.8
0
500
1500
2500
2000
4000
6000

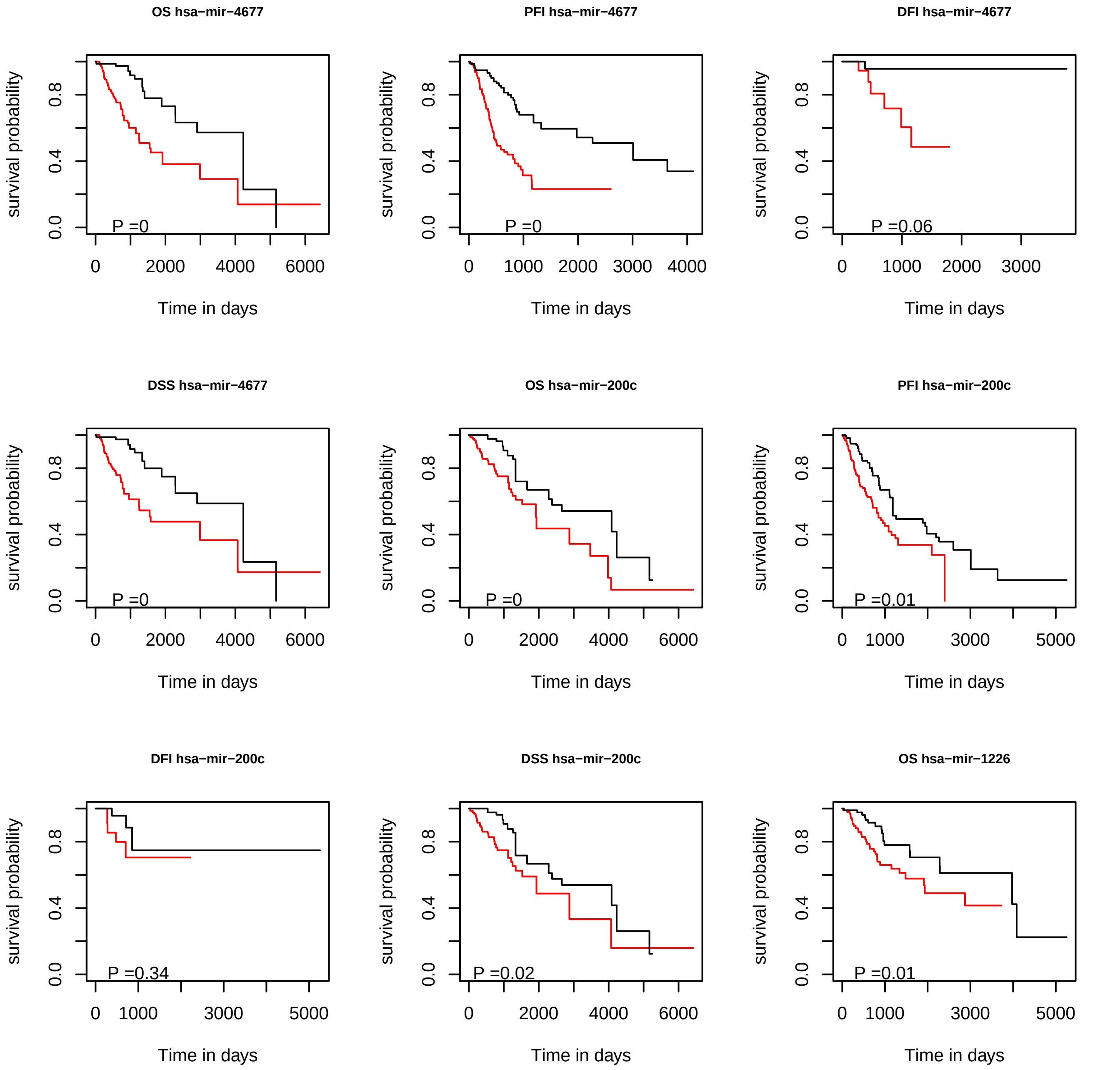
OS hsa−mir−4677
survival probability
0.0 0.4 0.8
P =0
0 2000 4000 6000
Time in days
PFI hsa−mir−4677
survival probability
0.0 0.4 0.8
P =0
0 1000 2000 3000 4000
Time in days
DFI hsa−mir−4677
survival probability
0.0 0.4 0.8
P =0.06
0 1000 2000 3000
Time in days
DSS hsa−mir−4677
survival probability
0.0 0.4 0.8
P =0
0 2000 4000 6000
Time in days
OS hsa−mir−200c
survival probability
0.0 0.4 0.8
P =0
0 2000 4000 6000
Time in days
PFI hsa−mir−200c
survival probability
0.0 0.4 0.8
P =0.01
0 1000 3000 5000
Time in days
DFI hsa−mir−200c
survival probability
0.0 0.4 0.8
P =0.34
0 1000 3000 5000
Time in days
DSS hsa−mir−200c
survival probability
0.0 0.4 0.8
P =0.02
0 2000 4000 6000
Time in days
OS hsa−mir−1226
survival probability
0.0 0.4 0.8
P =0.01
0 1000 3000 5000
Time in days

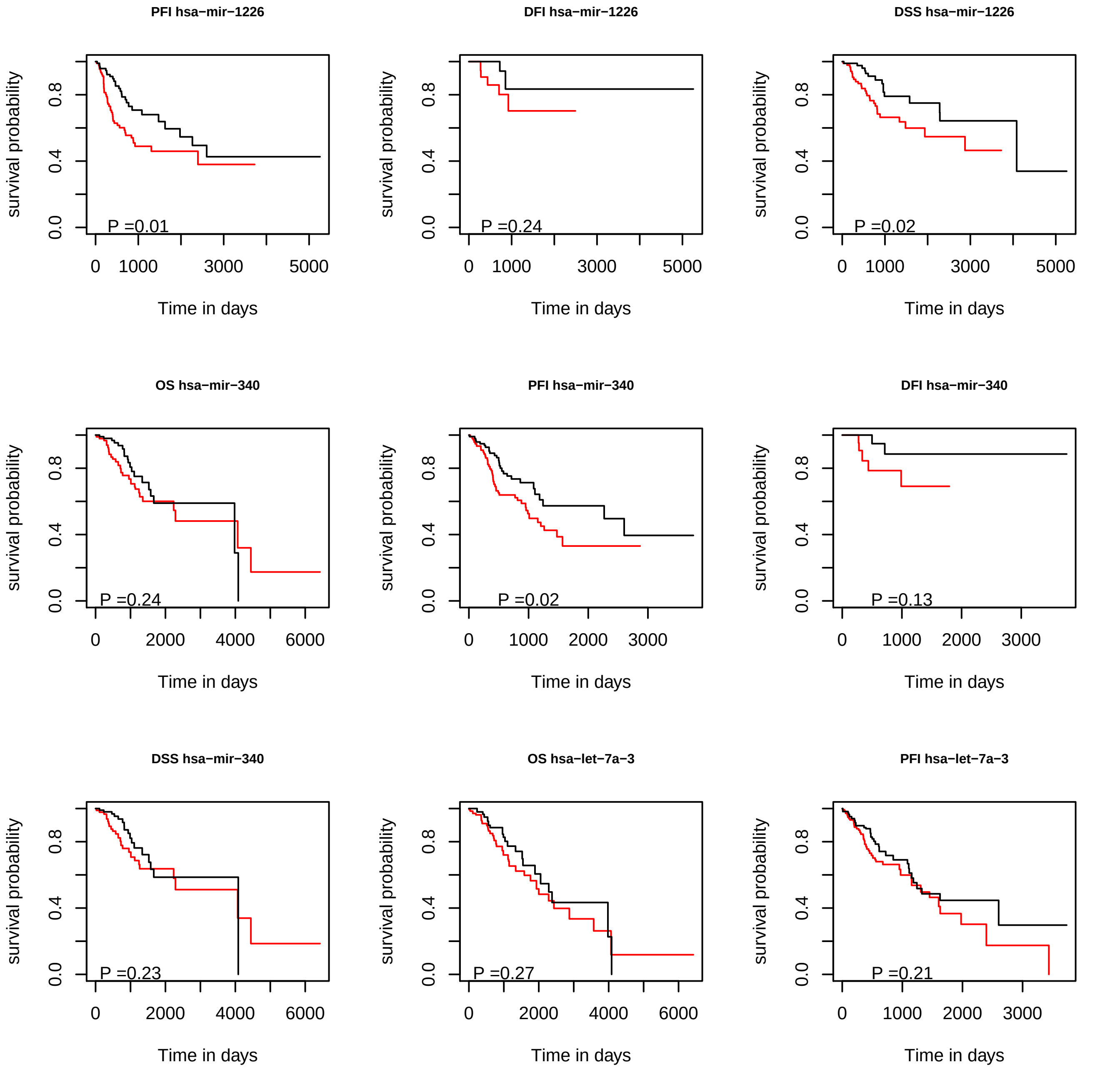
PFI hsa−mir−1226
survival probability
0.0
0.4
0.8
P =0.01
0
1000
3000
5000
Time in days
DFI hsa−mir−1226
survival probability
0.0
0.4
0.8
P =0.24
0
1000
3000
5000
Time in days
DSS hsa−mir−1226
survival probability
0.0
0.4
0.8
P =0.02
0
1000
3000
5000
Time in days
OS hsa−mir−340
survival probability
0.0
0.4
0.8
P =0.24
0
2000
4000
6000
Time in days
PFI hsa−mir−340
survival probability
0.0
0.4
0.8
P =0.02
0
1000
2000
3000
Time in days
DFI hsa−mir−340
survival probability
0.0
0.4
0.8
P =0.13
0
1000
2000
3000
Time in days
DSS hsa−mir−340
survival probability
0.0
0.4
0.8
P =0.23
0
2000
4000
6000
Time in days
OS hsa−let−7a−3
survival probability
0.0
0.4
0.8
P =0.27
0
2000
4000
6000
Time in days
PFI hsa−let−7a−3
survival probability
0.0
0.4
0.8
P =0.21
0
1000
2000
3000
Time in days

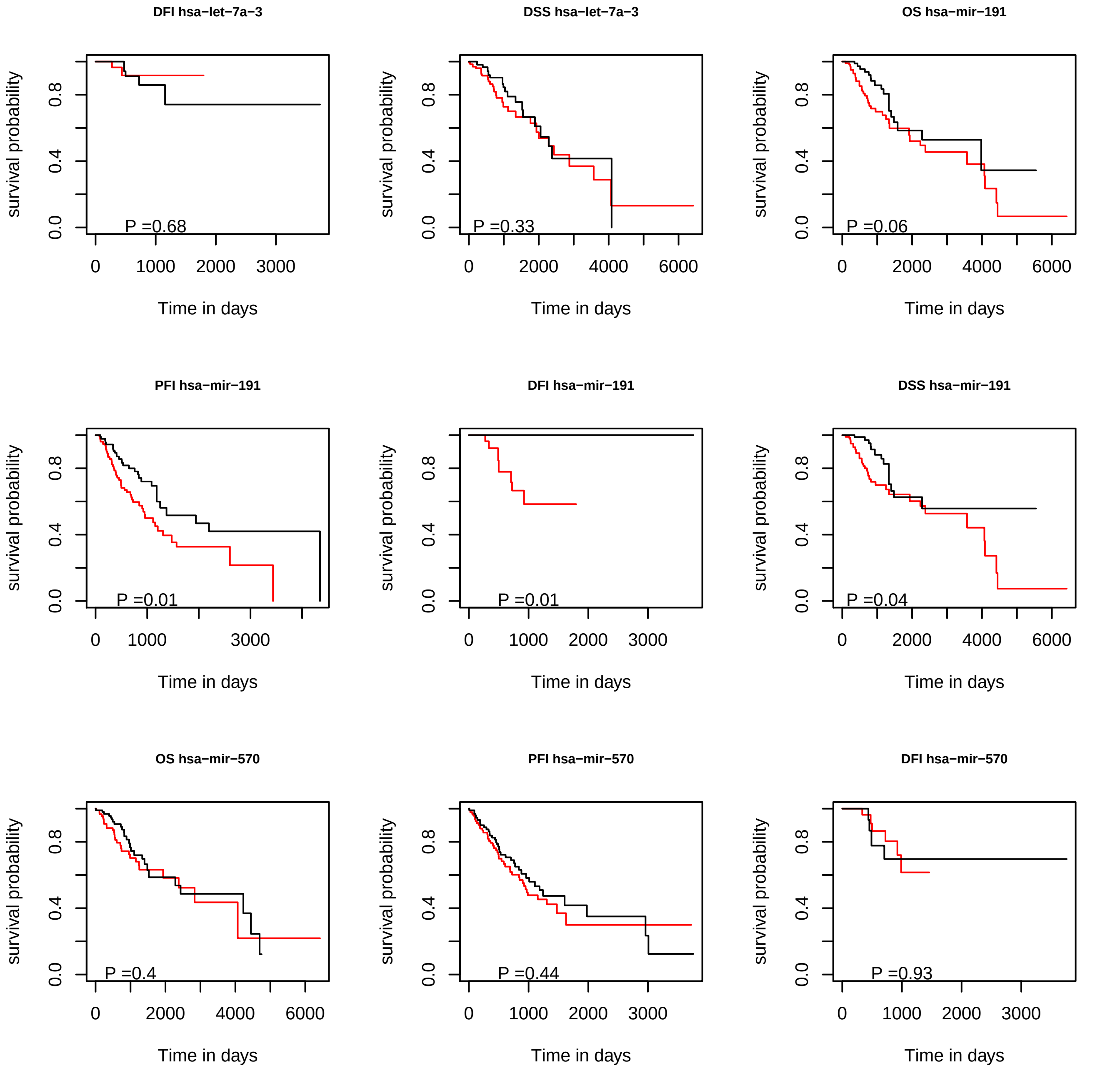
DFI hsa−let−7a−3
P =0.68
DSS hsa−let−7a−3
P =0.33
OS hsa−mir−191
P =0.06
PFI hsa−mir−191
P =0.01
DFI hsa−mir−191
P =0.01
DSS hsa−mir−191
P =0.04
OS hsa−mir−570
P =0.4
PFI hsa−mir−570
P =0.44
DFI hsa−mir−570
P =0.93
survival probability
Time in days
0.0
0.4
0.8
0
1000
2000
3000
4000
6000

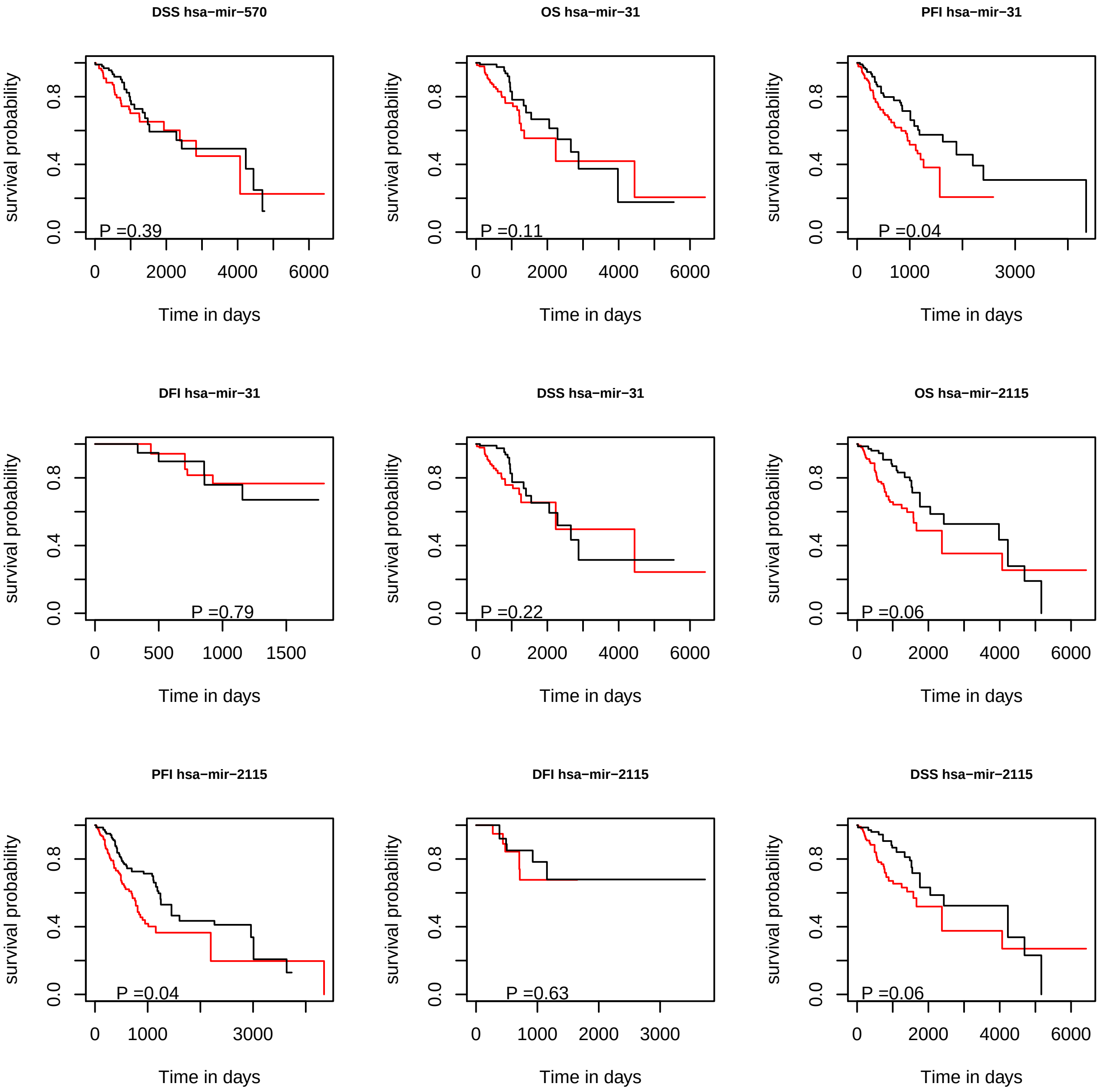
DSS hsa−mir−570
P =0.39
OS hsa−mir−31
P =0.11
PFI hsa−mir−31
P =0.04
DFI hsa−mir−31
P =0.79
DSS hsa−mir−31
P =0.22
OS hsa−mir−2115
P =0.06
PFI hsa−mir−2115
P =0.04
DFI hsa−mir−2115
P =0.63
DSS hsa−mir−2115
P =0.06
survival probability
Time in days
0.0
0.4
0.8
0
500
1000
1500
2000
3000
4000
6000
