# Supplementary material for: Computing microRNA-gene interaction networks in pan-cancer using miRDriver
Source: Sci Rep. 2022 Mar 8;12:3717. doi: 10.1038/s41598-022-07628-z (PMC8904490; doi:10.1038/s41598-022-07628-z)
Supplement: Supplementary file 20 — Supplementary Information 20. [file 41598_2022_7628_MOESM20_ESM.pdf]

Computing microRNA-gene interaction networks in pan-cancer using miRDriver

Banabithi Bose, Matthew Moravec, and Serdar Bozdag

Supplemental Figure S11

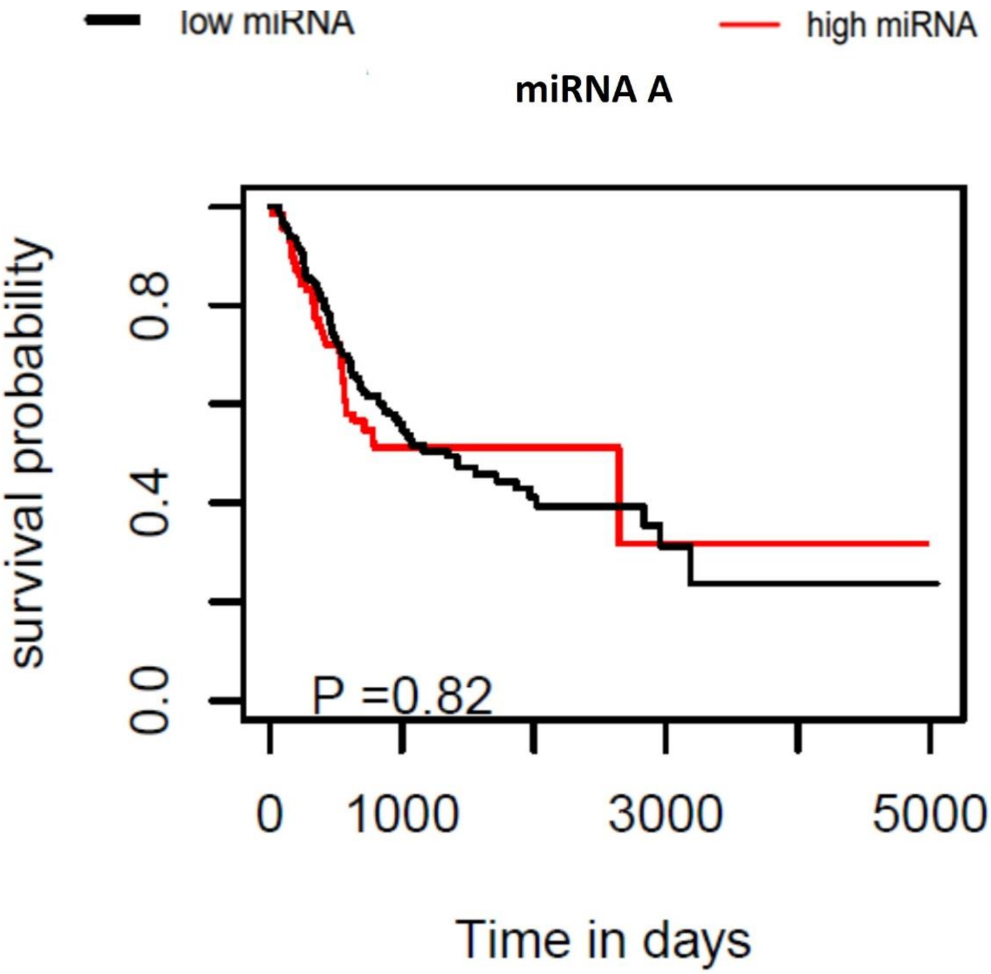

The *Adjusted Kaplan-Meier* survival plots for the computed miRNAs in high and low miRNA expression patient groups.

Supplemental Figure S11

Cancer Type: LIHC

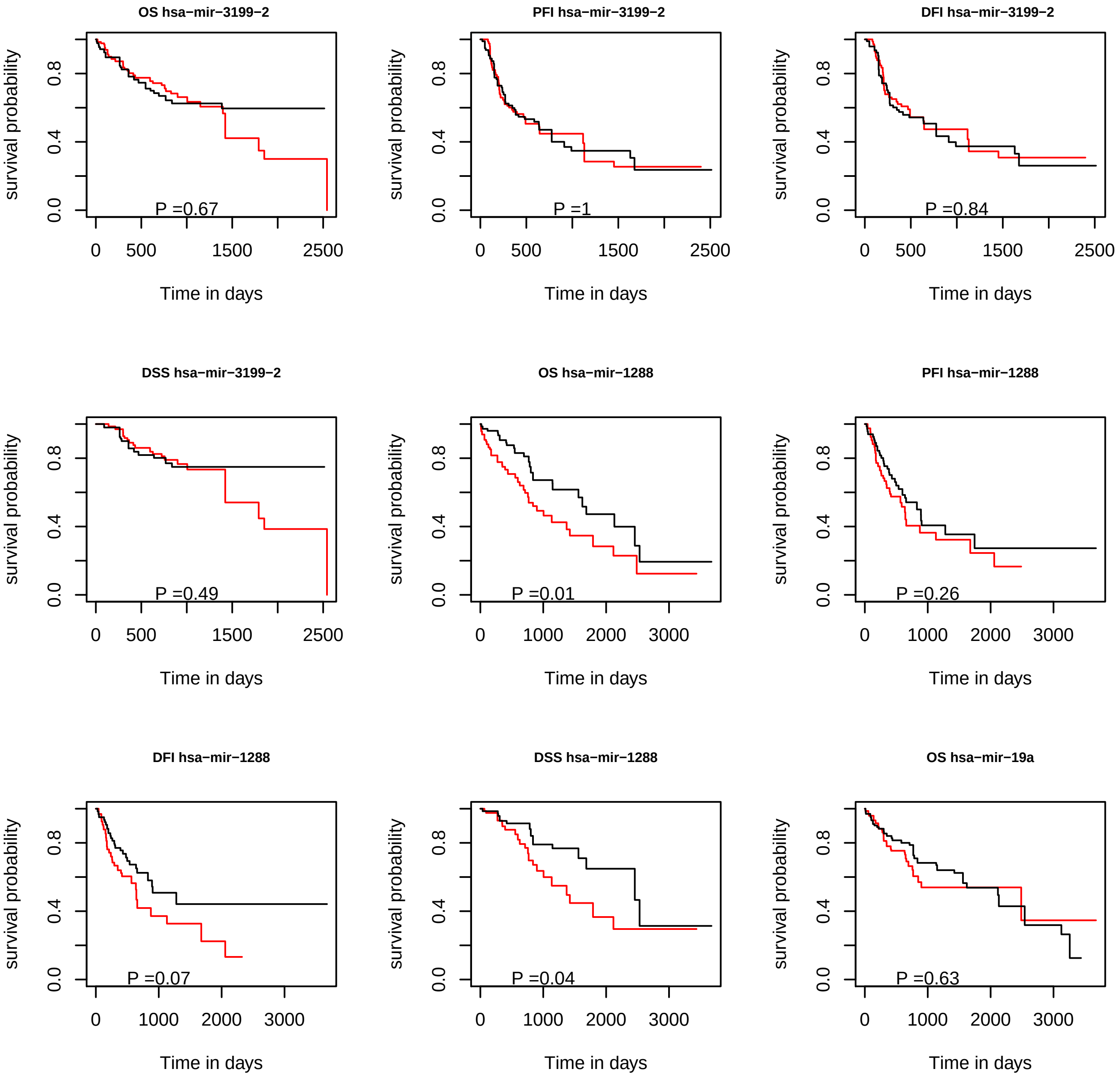

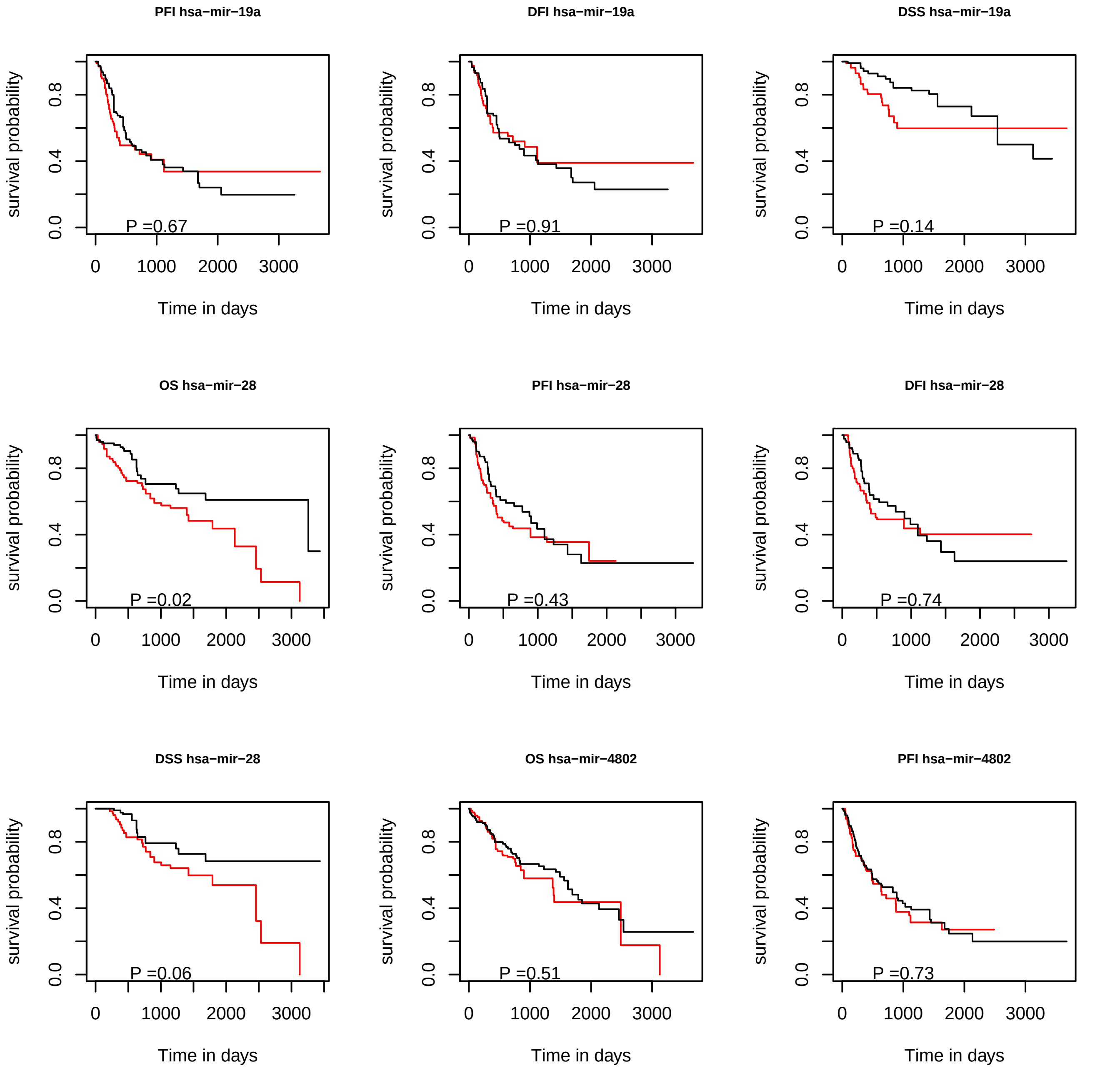
PFI hsa−mir−19a
P =0.67
DFI hsa−mir−19a
P =0.91
DSS hsa−mir−19a
P =0.14
OS hsa−mir−28
P =0.02
PFI hsa−mir−28
P =0.43
DFI hsa−mir−28
P =0.74
DSS hsa−mir−28
P =0.06
OS hsa−mir−4802
P =0.51
PFI hsa−mir−4802
P =0.73
survival probability
0.0
0.4
0.8
Time in days
0
1000
2000
3000

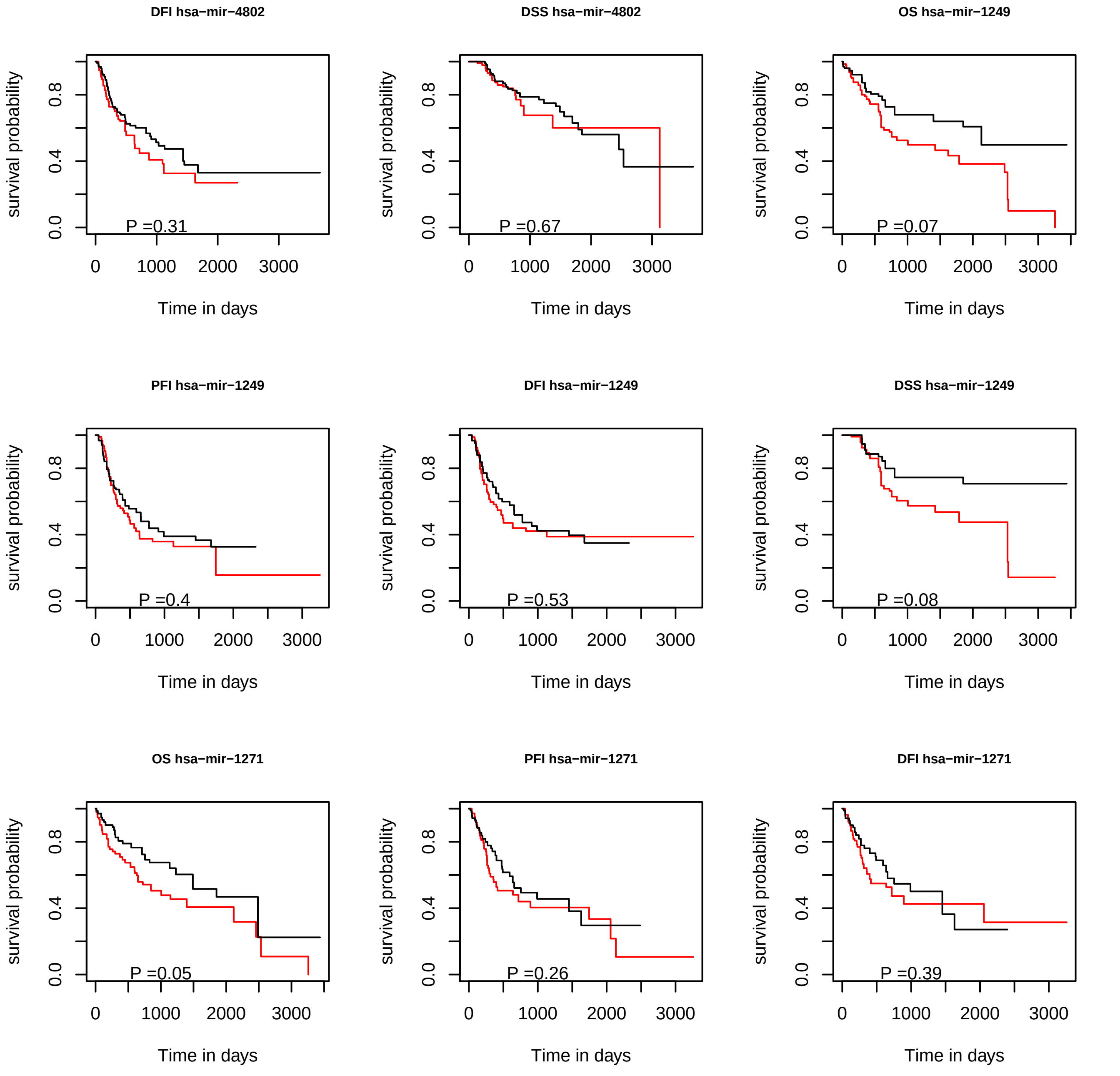
DFI hsa−mir−4802
survival probability
0.0 0.4 0.8
P =0.31
0 1000 2000 3000
Time in days
DSS hsa−mir−4802
survival probability
0.0 0.4 0.8
P =0.67
0 1000 2000 3000
Time in days
OS hsa−mir−1249
survival probability
0.0 0.4 0.8
P =0.07
0 1000 2000 3000
Time in days
PFI hsa−mir−1249
survival probability
0.0 0.4 0.8
P =0.4
0 1000 2000 3000
Time in days
DFI hsa−mir−1249
survival probability
0.0 0.4 0.8
P =0.53
0 1000 2000 3000
Time in days
DSS hsa−mir−1249
survival probability
0.0 0.4 0.8
P =0.08
0 1000 2000 3000
Time in days
OS hsa−mir−1271
survival probability
0.0 0.4 0.8
P =0.05
0 1000 2000 3000
Time in days
PFI hsa−mir−1271
survival probability
0.0 0.4 0.8
P =0.26
0 1000 2000 3000
Time in days
DFI hsa−mir−1271
survival probability
0.0 0.4 0.8
P =0.39
0 1000 2000 3000
Time in days

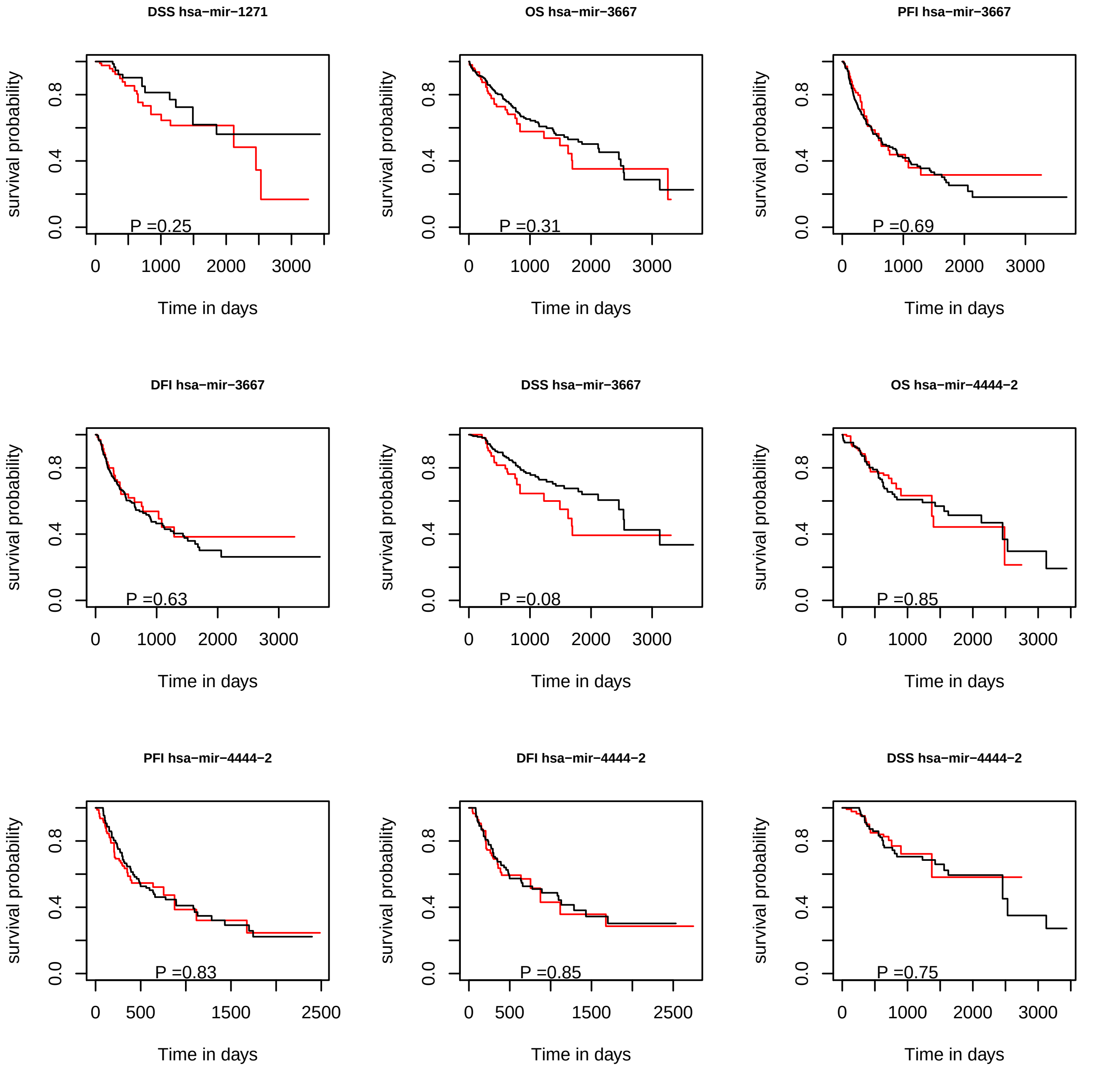
DSS hsa−mir−1271
survival probability
0.0
0.4
0.8
P =0.25
0
1000
2000
3000
Time in days
OS hsa−mir−3667
survival probability
0.0
0.4
0.8
P =0.31
0
1000
2000
3000
Time in days
PFI hsa−mir−3667
survival probability
0.0
0.4
0.8
P =0.69
0
1000
2000
3000
Time in days
DFI hsa−mir−3667
survival probability
0.0
0.4
0.8
P =0.63
0
1000
2000
3000
Time in days
DSS hsa−mir−3667
survival probability
0.0
0.4
0.8
P =0.08
0
1000
2000
3000
Time in days
OS hsa−mir−4444−2
survival probability
0.0
0.4
0.8
P =0.85
0
1000
2000
3000
Time in days
PFI hsa−mir−4444−2
survival probability
0.0
0.4
0.8
P =0.83
0
500
1500
2500
Time in days
DFI hsa−mir−4444−2
survival probability
0.0
0.4
0.8
P =0.85
0
500
1500
2500
Time in days
DSS hsa−mir−4444−2
survival probability
0.0
0.4
0.8
P =0.75
0
1000
2000
3000
Time in days

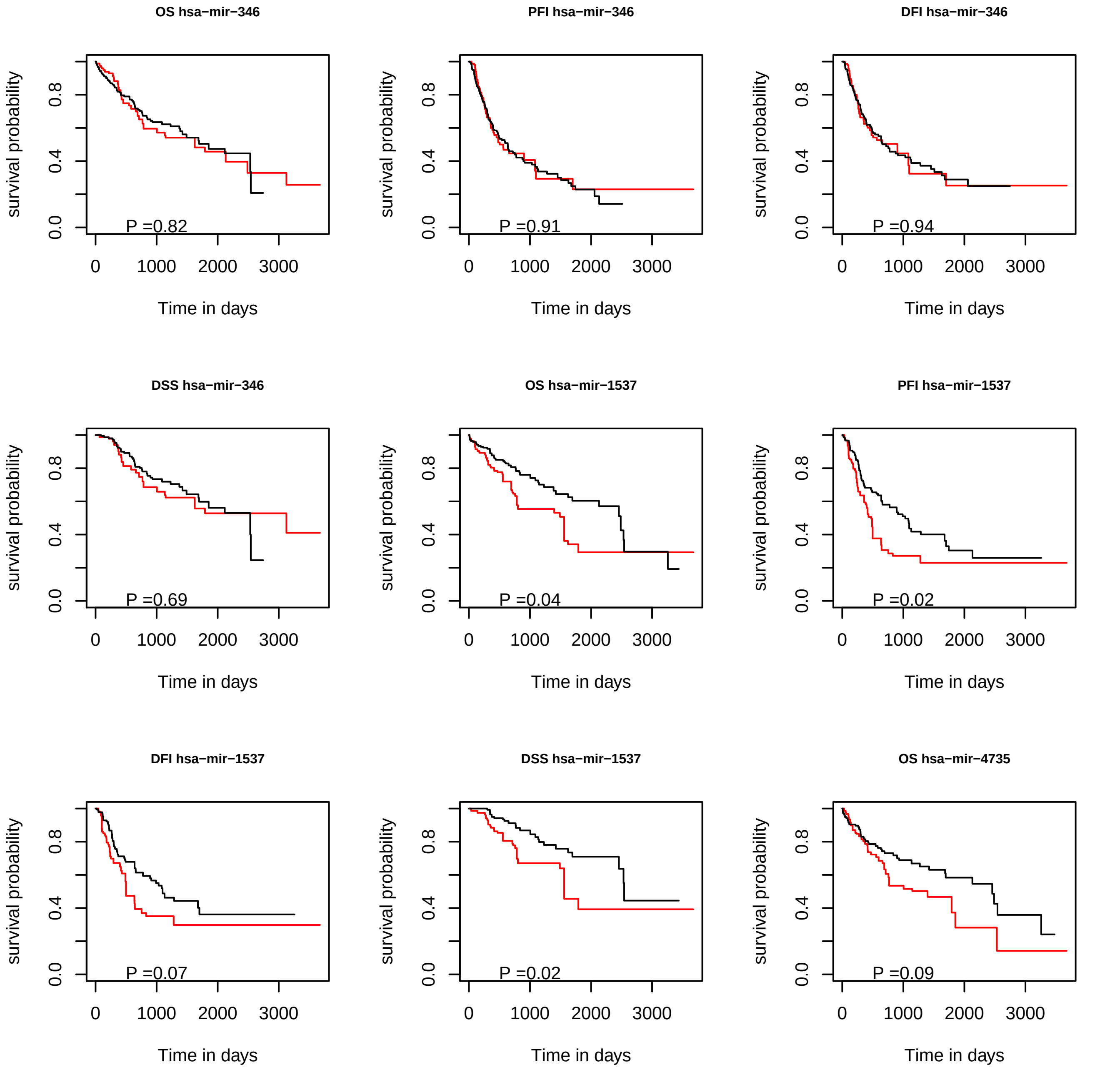
OS hsa−mir−346
P =0.82
PFI hsa−mir−346
P =0.91
DFI hsa−mir−346
P =0.94
DSS hsa−mir−346
P =0.69
OS hsa−mir−1537
P =0.04
PFI hsa−mir−1537
P =0.02
DFI hsa−mir−1537
P =0.07
DSS hsa−mir−1537
P =0.02
OS hsa−mir−4735
P =0.09
survival probability
0.0
0.4
0.8
Time in days
0
1000
2000
3000

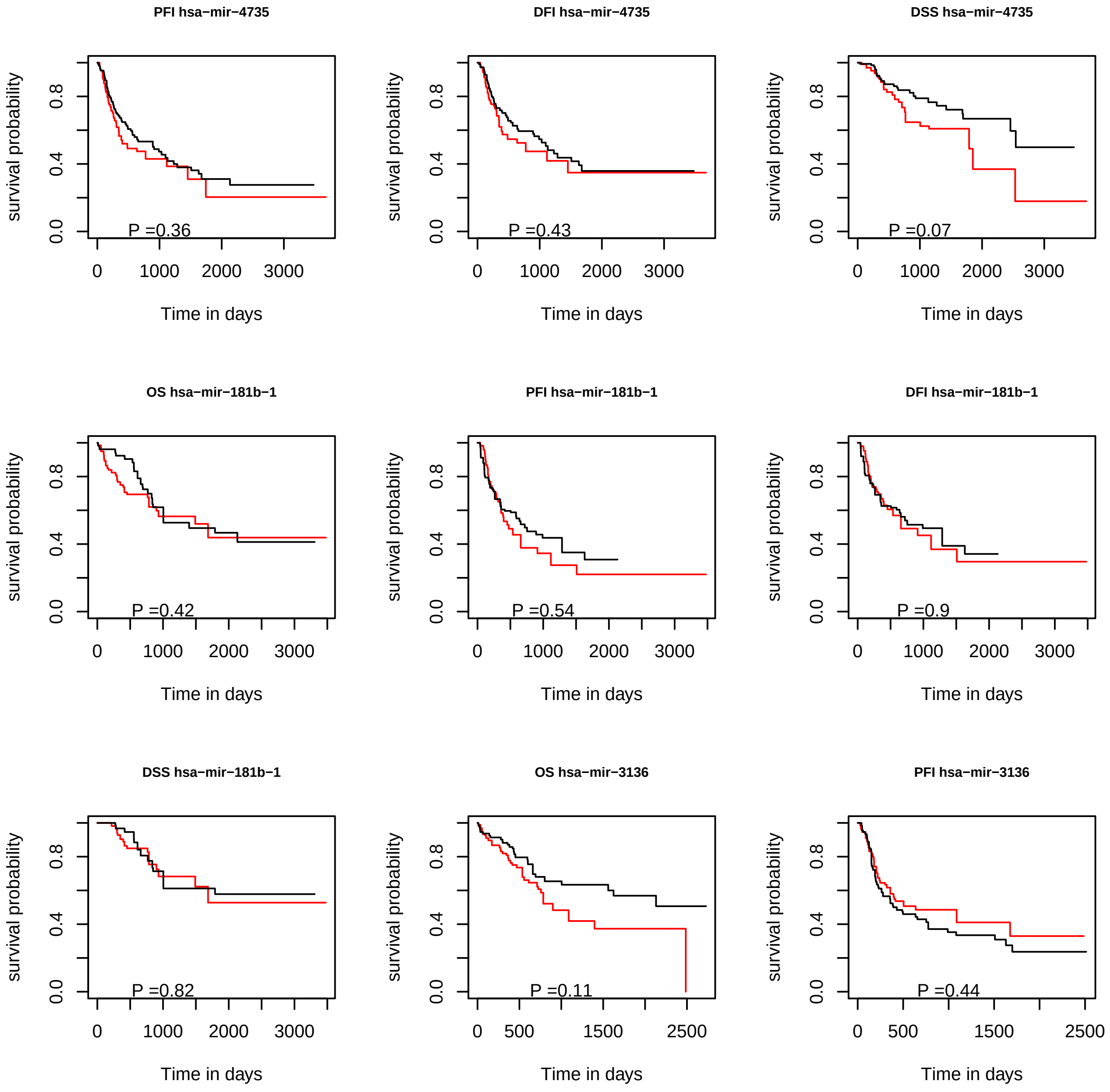

PFI hsa−mir−4735
P =0.36
DFI hsa−mir−4735
P =0.43
DSS hsa−mir−4735
P =0.07
OS hsa−mir−181b−1
P =0.42
PFI hsa−mir−181b−1
P =0.54
DFI hsa−mir−181b−1
P =0.9
DSS hsa−mir−181b−1
P =0.82
OS hsa−mir−3136
P =0.11
PFI hsa−mir−3136
P =0.44
survival probability
0.0
0.4
0.8
Time in days
0
500
1000
1500
2000
2500
3000

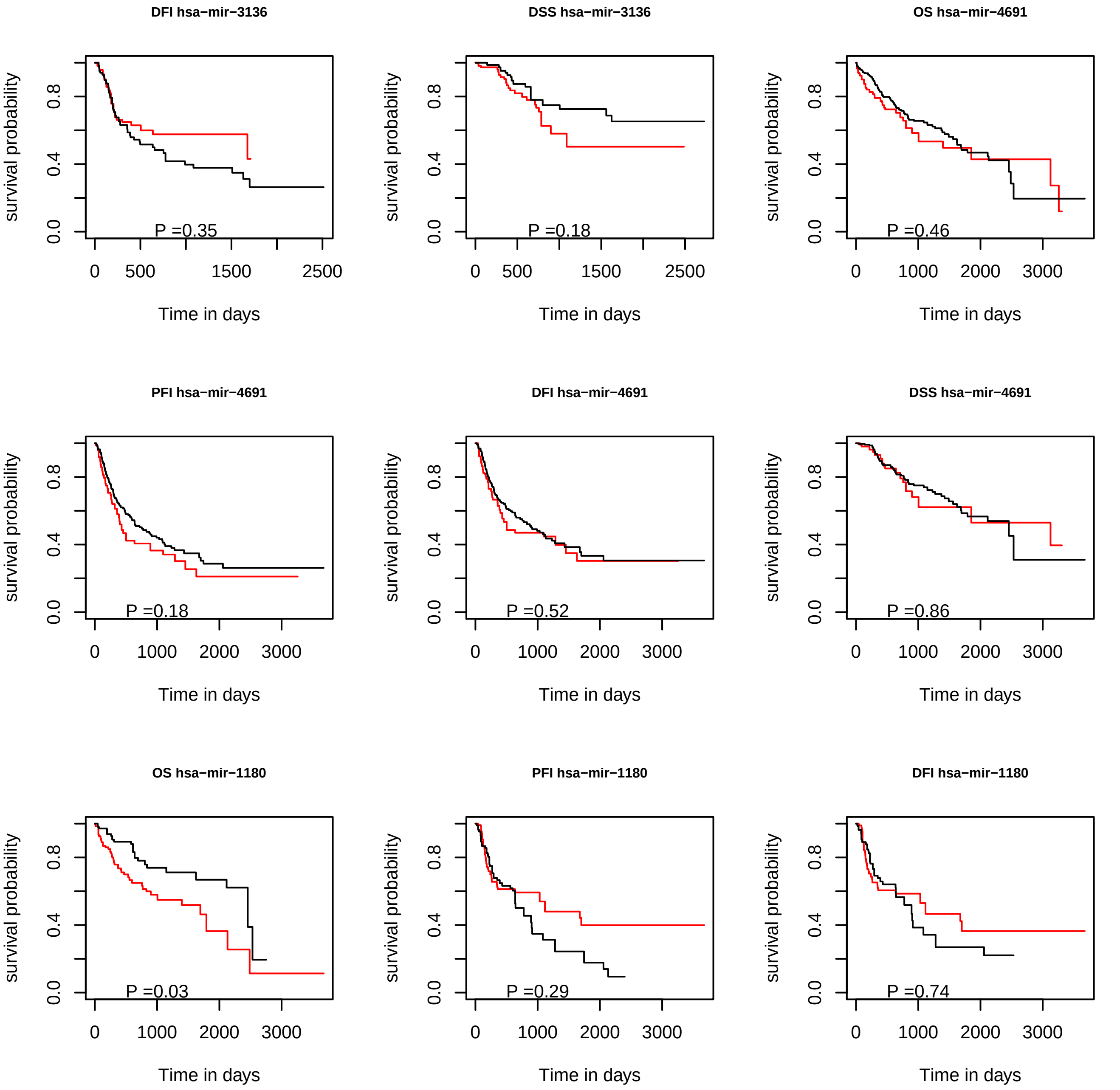
DFI hsa−mir−3136
P =0.35
DSS hsa−mir−3136
P =0.18
OS hsa−mir−4691
P =0.46
PFI hsa−mir−4691
P =0.18
DFI hsa−mir−4691
P =0.52
DSS hsa−mir−4691
P =0.86
OS hsa−mir−1180
P =0.03
PFI hsa−mir−1180
P =0.29
DFI hsa−mir−1180
P =0.74
survival probability
Time in days
0.0
0.4
0.8
0
500
1500
2500
1000
2000
3000

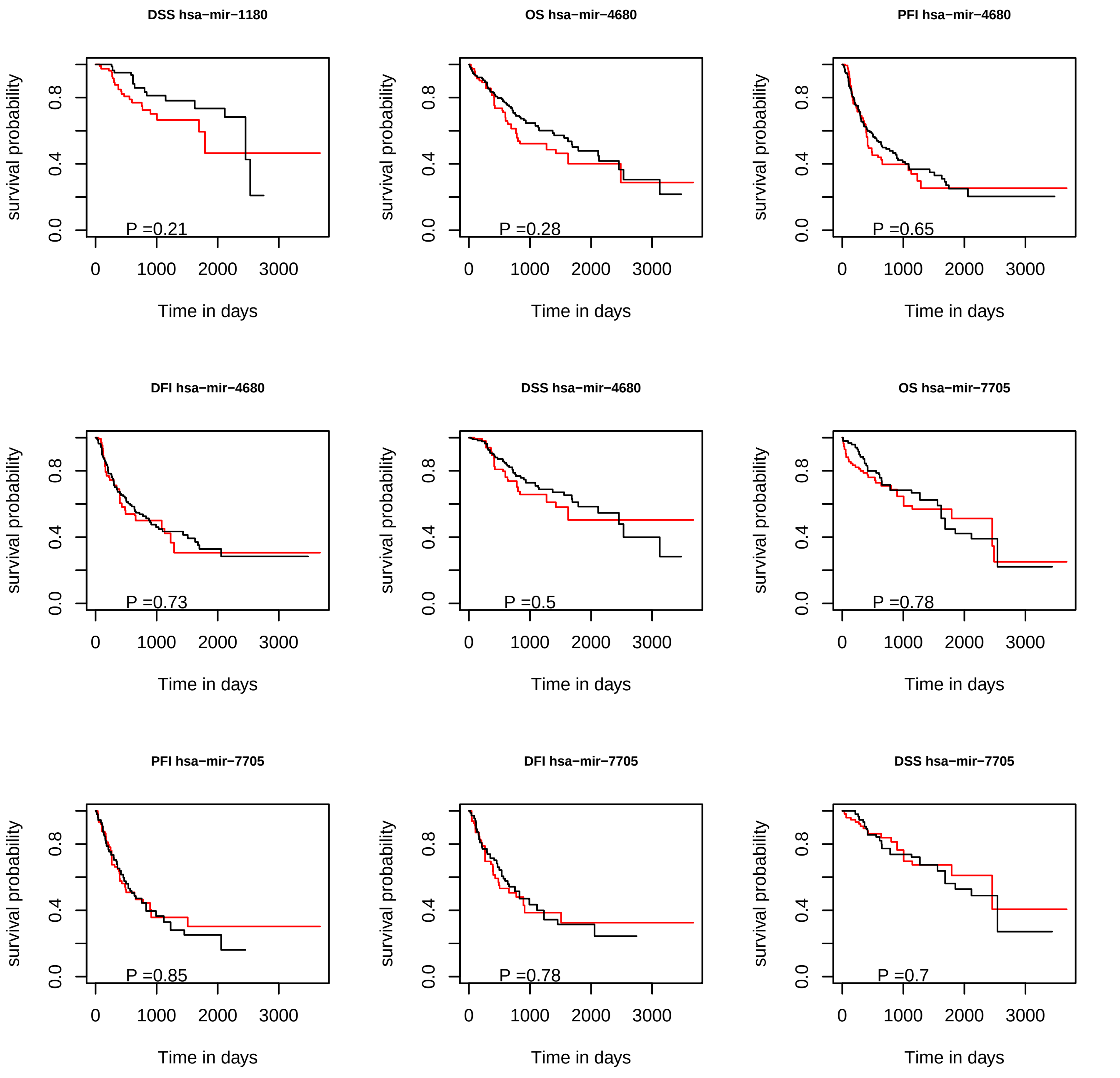
DSS hsa−mir−1180
P =0.21
OS hsa−mir−4680
P =0.28
PFI hsa−mir−4680
P =0.65
DFI hsa−mir−4680
P =0.73
DSS hsa−mir−4680
P =0.5
OS hsa−mir−7705
P =0.78
PFI hsa−mir−7705
P =0.85
DFI hsa−mir−7705
P =0.78
DSS hsa−mir−7705
P =0.7
survival probability
Time in days
0.0
0.4
0.8
0
1000
2000
3000

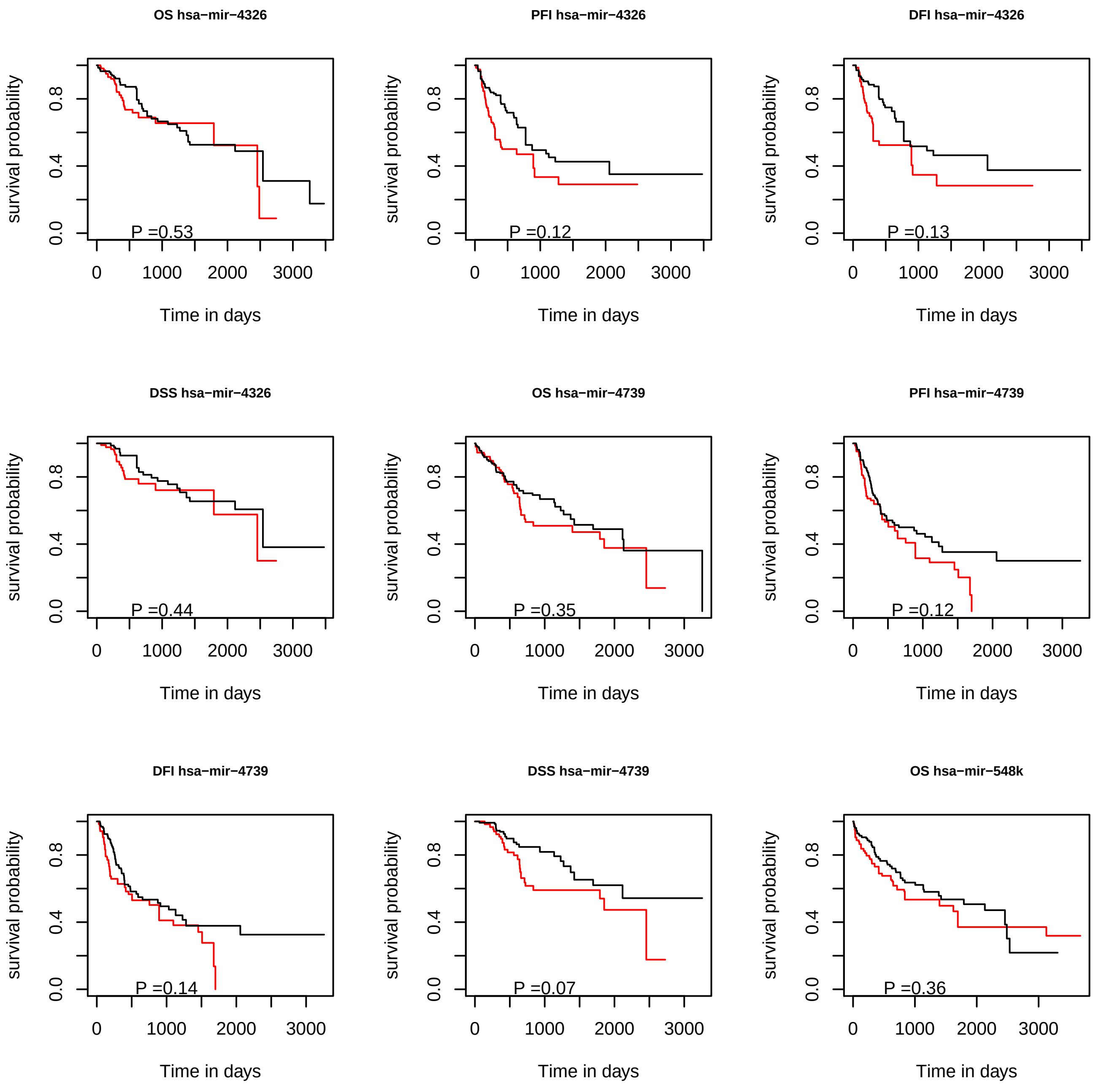
OS hsa−mir−4326
P =0.53
PFI hsa−mir−4326
P =0.12
DFI hsa−mir−4326
P =0.13
DSS hsa−mir−4326
P =0.44
OS hsa−mir−4739
P =0.35
PFI hsa−mir−4739
P =0.12
DFI hsa−mir−4739
P =0.14
DSS hsa−mir−4739
P =0.07
OS hsa−mir−548k
P =0.36
survival probability
0.0
0.4
0.8
Time in days
0
1000
2000
3000

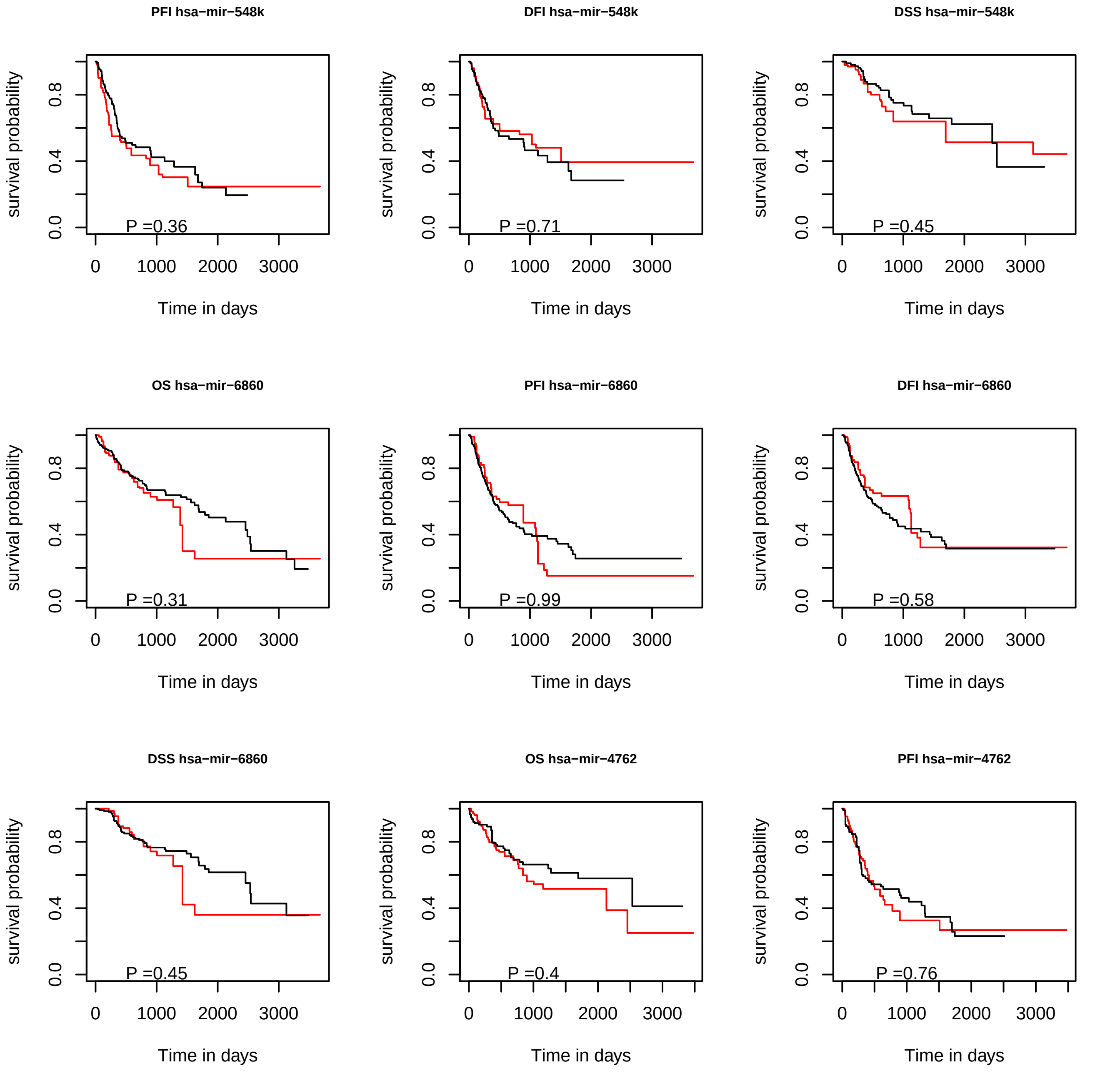
PFI hsa−mir−548k
P =0.36
DFI hsa−mir−548k
P =0.71
DSS hsa−mir−548k
P =0.45
OS hsa−mir−6860
P =0.31
PFI hsa−mir−6860
P =0.99
DFI hsa−mir−6860
P =0.58
DSS hsa−mir−6860
P =0.45
OS hsa−mir−4762
P =0.4
PFI hsa−mir−4762
P =0.76
survival probability
0.0
0.4
0.8
0
1000
2000
3000
Time in days

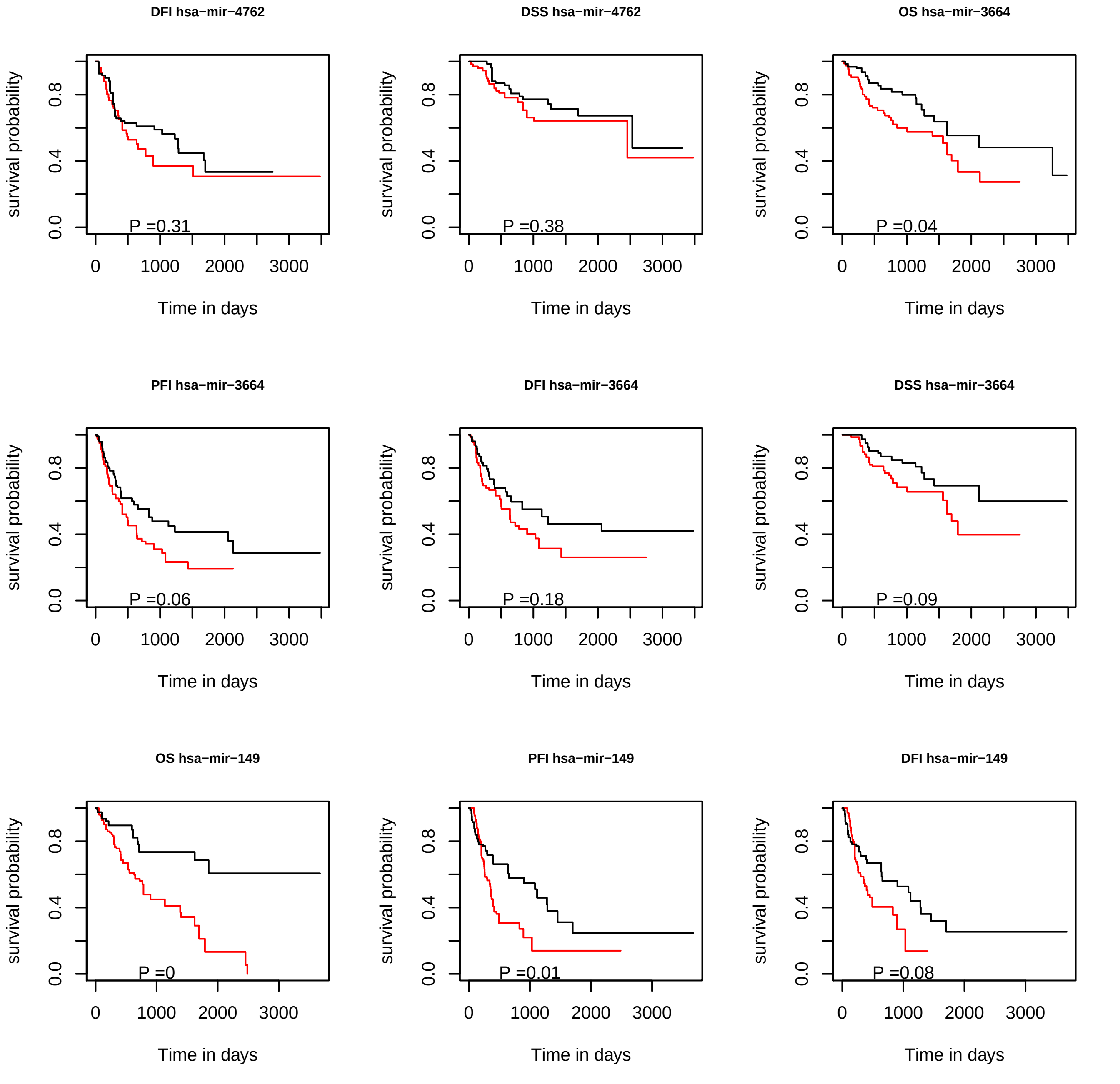
DFI hsa−mir−4762
P =0.31
DSS hsa−mir−4762
P =0.38
OS hsa−mir−3664
P =0.04
PFI hsa−mir−3664
P =0.06
DFI hsa−mir−3664
P =0.18
DSS hsa−mir−3664
P =0.09
OS hsa−mir−149
P =0
PFI hsa−mir−149
P =0.01
DFI hsa−mir−149
P =0.08
survival probability
0.0
0.4
0.8
Time in days
0
1000
2000
3000

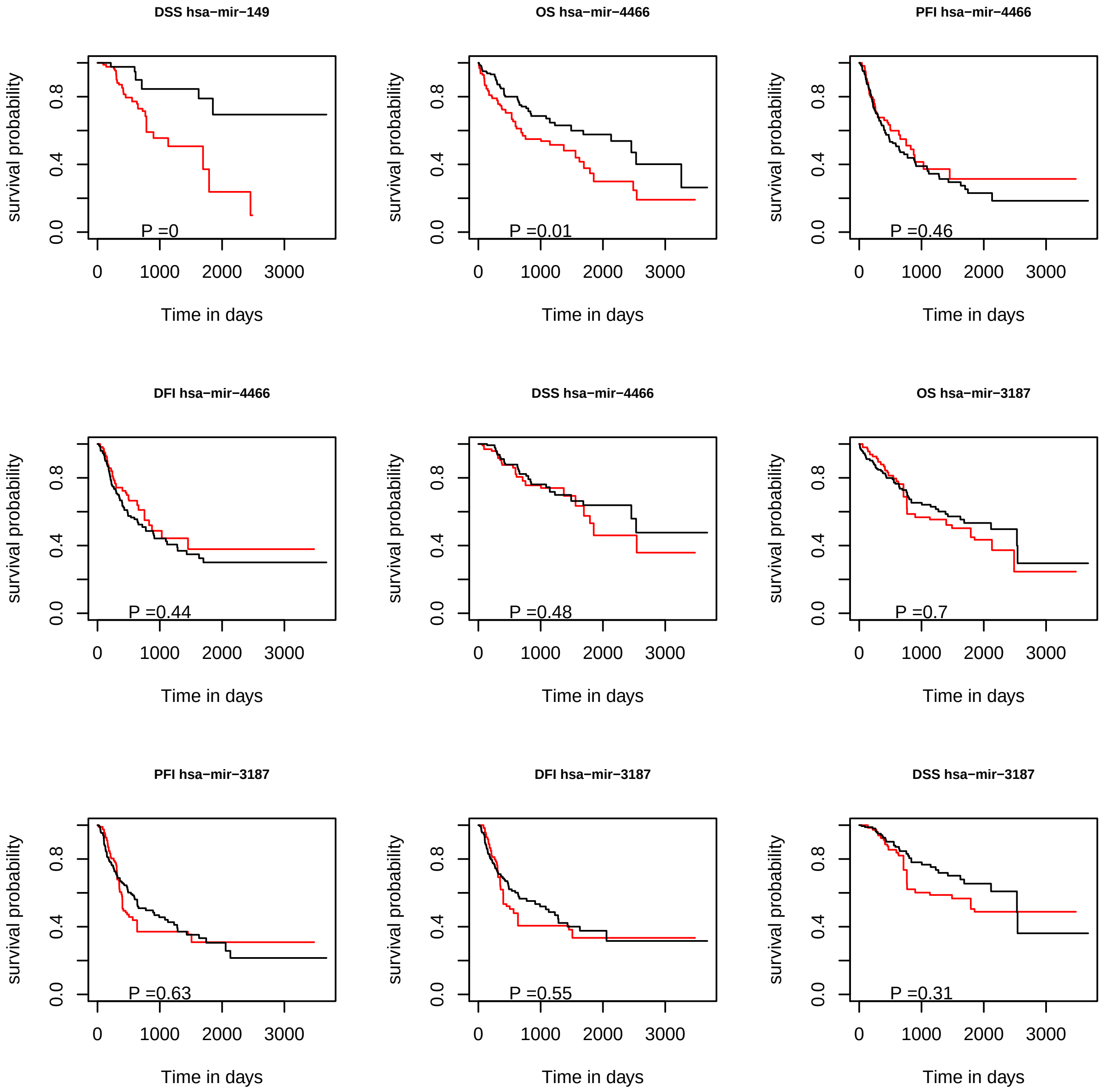
DSS hsa−mir−149
P =0
OS hsa−mir−4466
P =0.01
PFI hsa−mir−4466
P =0.46
DFI hsa−mir−4466
P =0.44
DSS hsa−mir−4466
P =0.48
OS hsa−mir−3187
P =0.7
PFI hsa−mir−3187
P =0.63
DFI hsa−mir−3187
P =0.55
DSS hsa−mir−3187
P =0.31
survival probability
0.0
0.4
0.8
0
1000
2000
3000
Time in days

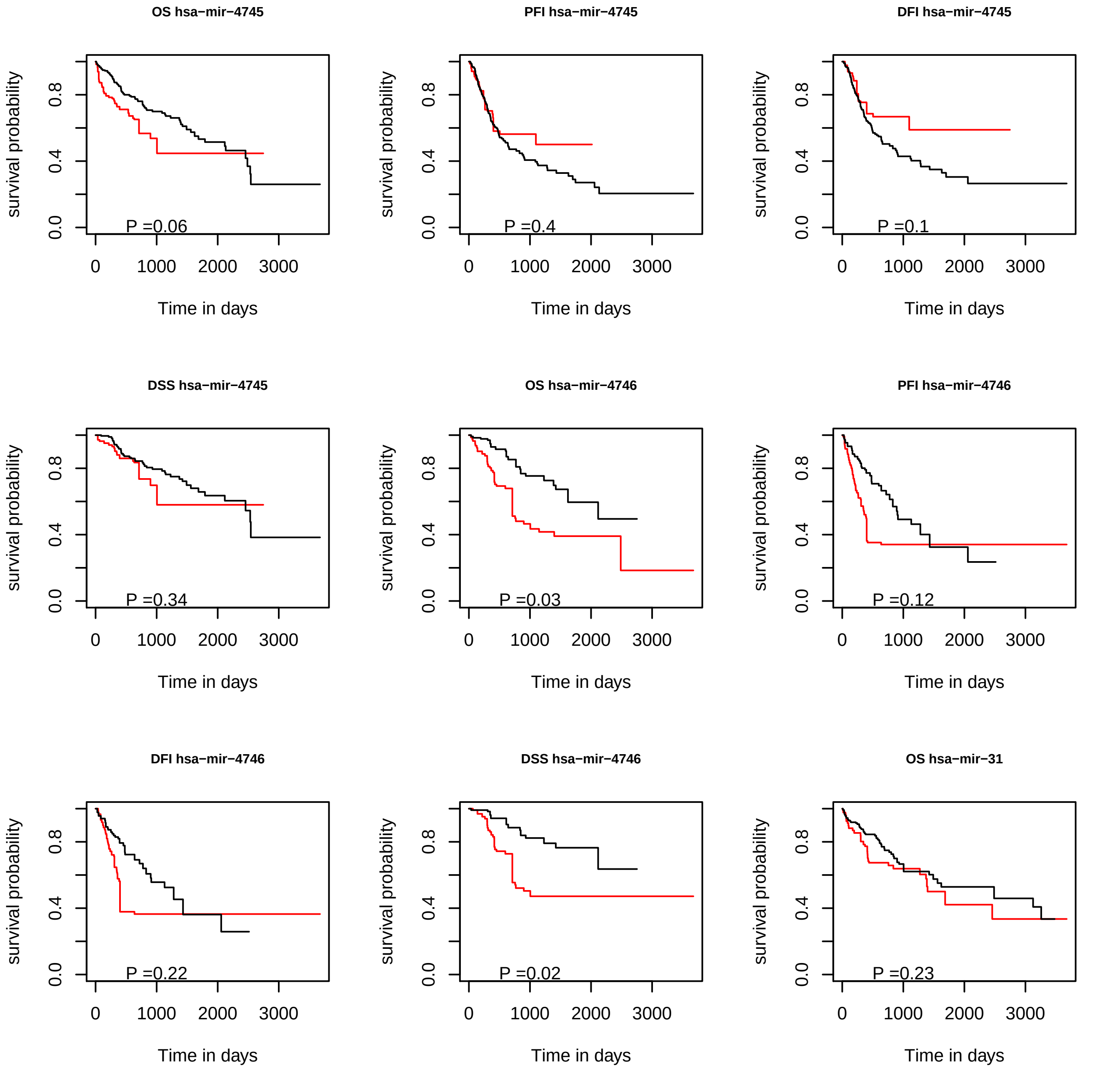
OS hsa−mir−4745
P =0.06
PFI hsa−mir−4745
P =0.4
DFI hsa−mir−4745
P =0.1
DSS hsa−mir−4745
P =0.34
OS hsa−mir−4746
P =0.03
PFI hsa−mir−4746
P =0.12
DFI hsa−mir−4746
P =0.22
DSS hsa−mir−4746
P =0.02
OS hsa−mir−31
P =0.23
survival probability
Time in days
0.0
0.4
0.8
0
1000
2000
3000

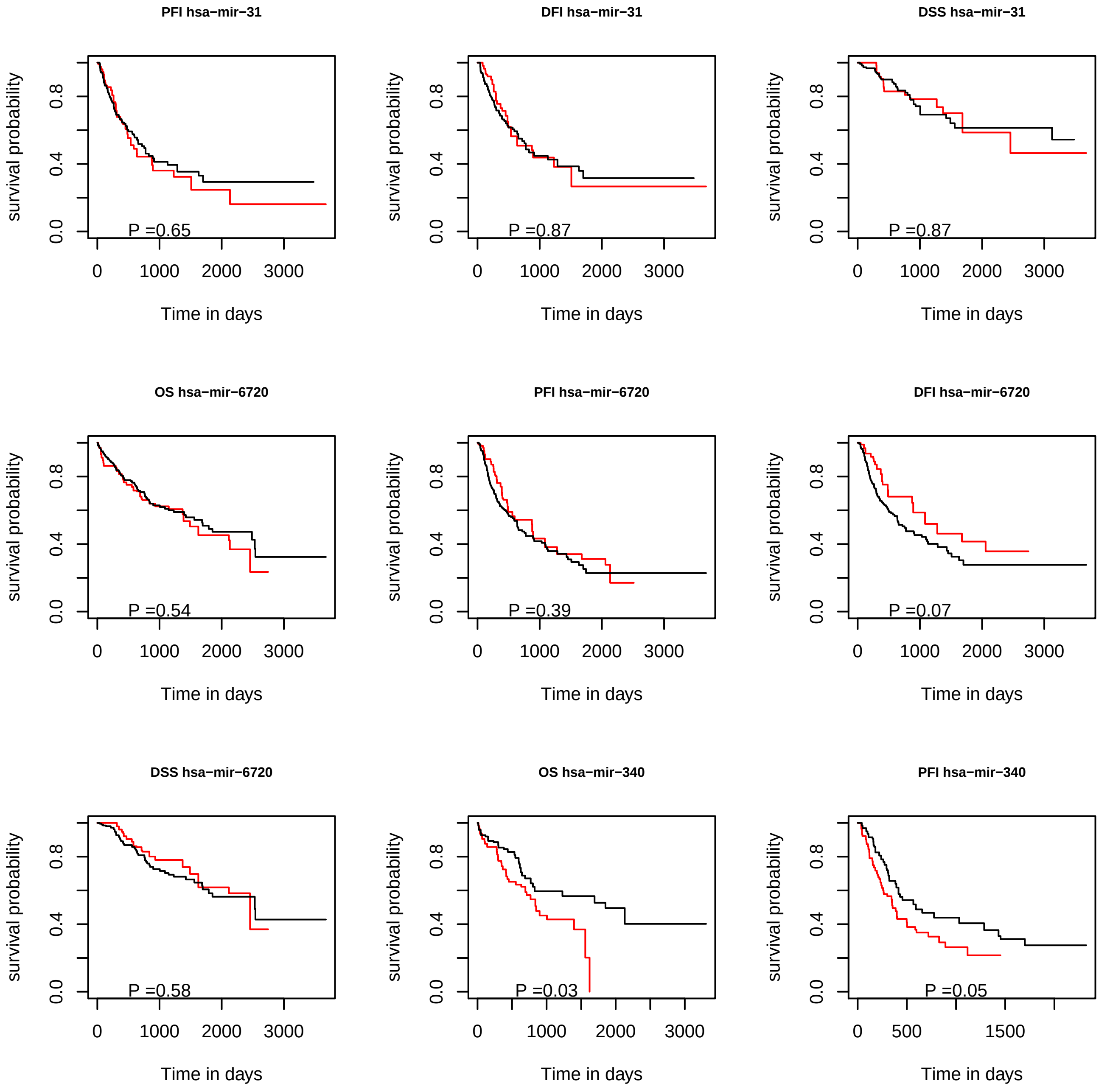
PFI hsa−mir−31
P =0.65
DFI hsa−mir−31
P =0.87
DSS hsa−mir−31
P =0.87
OS hsa−mir−6720
P =0.54
PFI hsa−mir−6720
P =0.39
DFI hsa−mir−6720
P =0.07
DSS hsa−mir−6720
P =0.58
OS hsa−mir−340
P =0.03
PFI hsa−mir−340
P =0.05
survival probability
Time in days
0.0
0.4
0.8
0
500
1000
1500
2000
3000

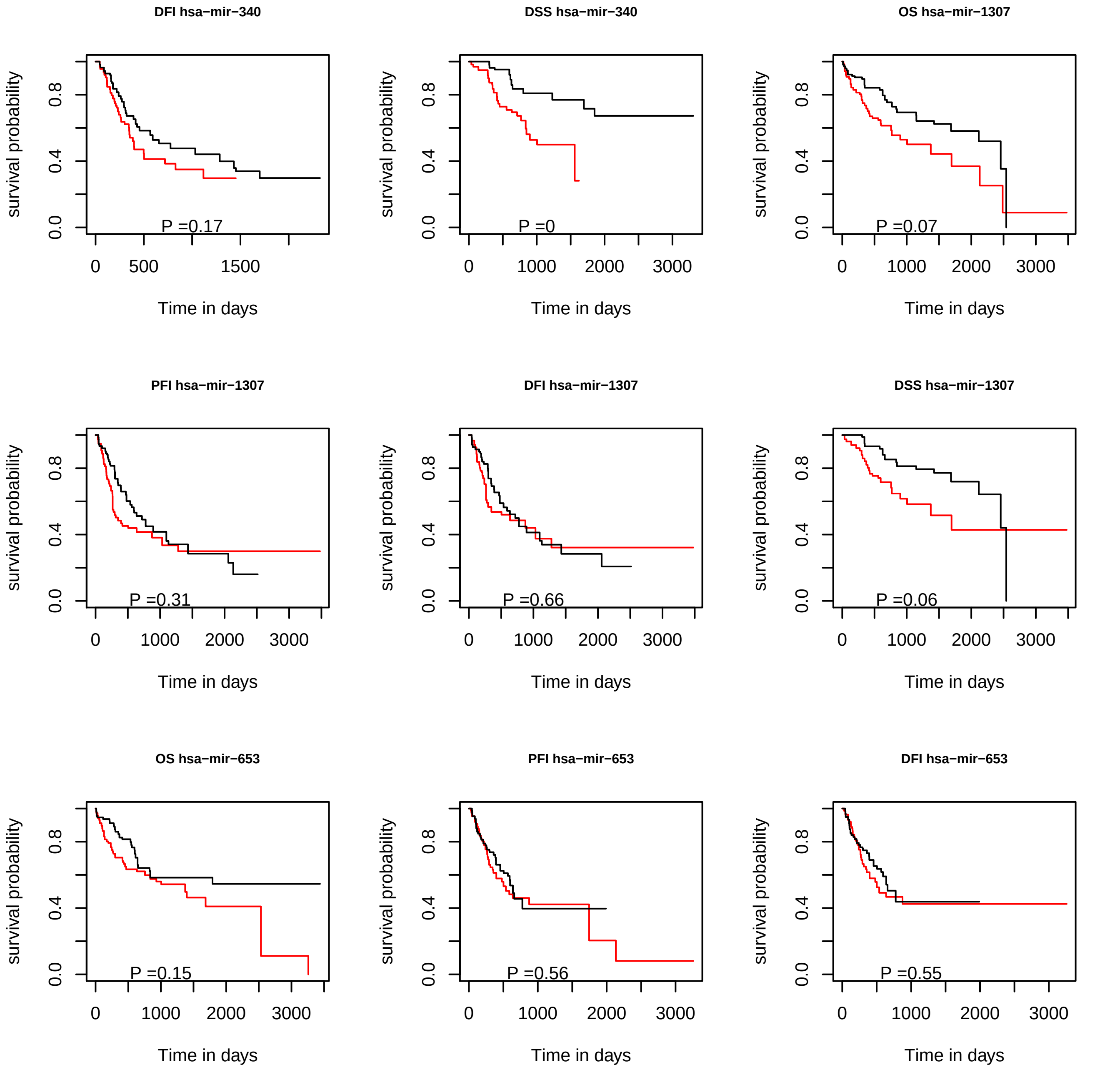
DFI hsa−mir−340
survival probability
0.0 0.4 0.8
P =0.17
0 500 1500
Time in days
DSS hsa−mir−340
survival probability
0.0 0.4 0.8
P =0
0 1000 2000 3000
Time in days
OS hsa−mir−1307
survival probability
0.0 0.4 0.8
P =0.07
0 1000 2000 3000
Time in days
PFI hsa−mir−1307
survival probability
0.0 0.4 0.8
P =0.31
0 1000 2000 3000
Time in days
DFI hsa−mir−1307
survival probability
0.0 0.4 0.8
P =0.66
0 1000 2000 3000
Time in days
DSS hsa−mir−1307
survival probability
0.0 0.4 0.8
P =0.06
0 1000 2000 3000
Time in days
OS hsa−mir−653
survival probability
0.0 0.4 0.8
P =0.15
0 1000 2000 3000
Time in days
PFI hsa−mir−653
survival probability
0.0 0.4 0.8
P =0.56
0 1000 2000 3000
Time in days
DFI hsa−mir−653
survival probability
0.0 0.4 0.8
P =0.55
0 1000 2000 3000
Time in days

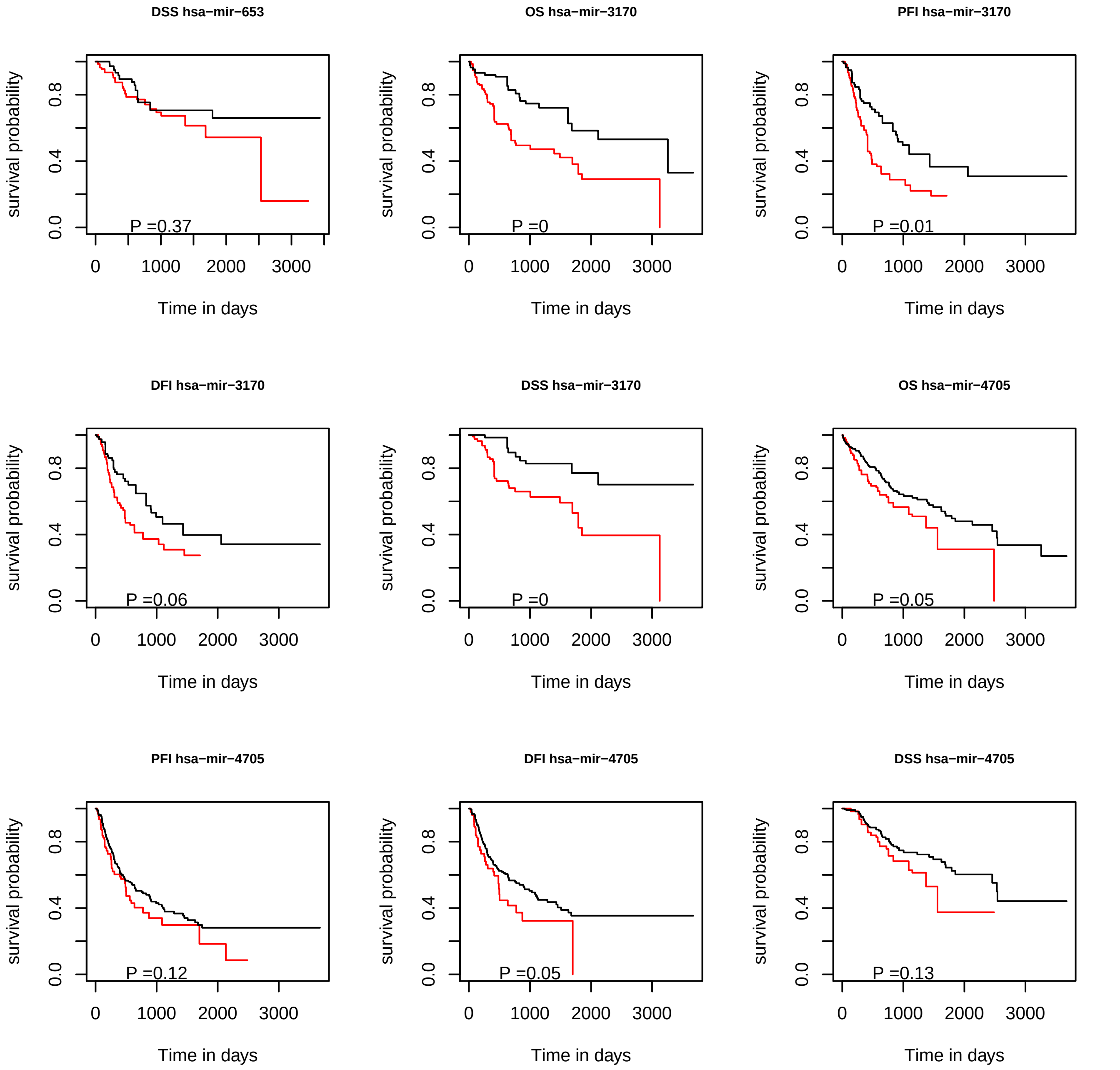
DSS hsa−mir−653
P =0.37
OS hsa−mir−3170
P =0
PFI hsa−mir−3170
P =0.01
DFI hsa−mir−3170
P =0.06
DSS hsa−mir−3170
P =0
OS hsa−mir−4705
P =0.05
PFI hsa−mir−4705
P =0.12
DFI hsa−mir−4705
P =0.05
DSS hsa−mir−4705
P =0.13
survival probability
0.0
0.4
0.8
0
1000
2000
3000
Time in days

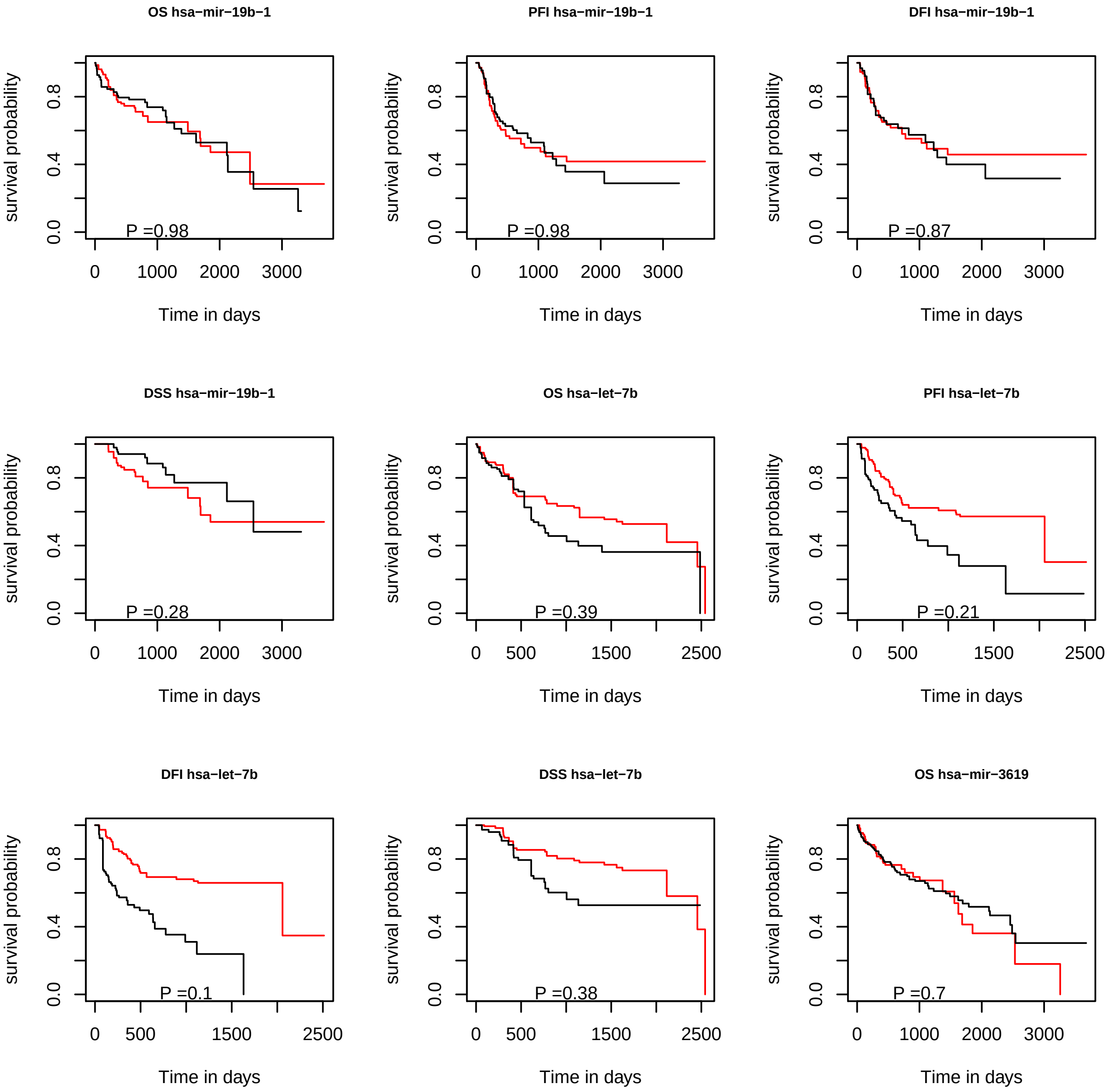
OS hsa−mir−19b−1
PFI hsa−mir−19b−1
DFI hsa−mir−19b−1
DSS hsa−mir−19b−1
OS hsa−let−7b
PFI hsa−let−7b
DFI hsa−let−7b
DSS hsa−let−7b
OS hsa−mir−3619
survival probability
Time in days
0.0
0.4
0.8
0
500
1000
1500
2000
2500
3000
P =0.98
P =0.98
P =0.87
P =0.28
P =0.39
P =0.21
P =0.1
P =0.38
P =0.7

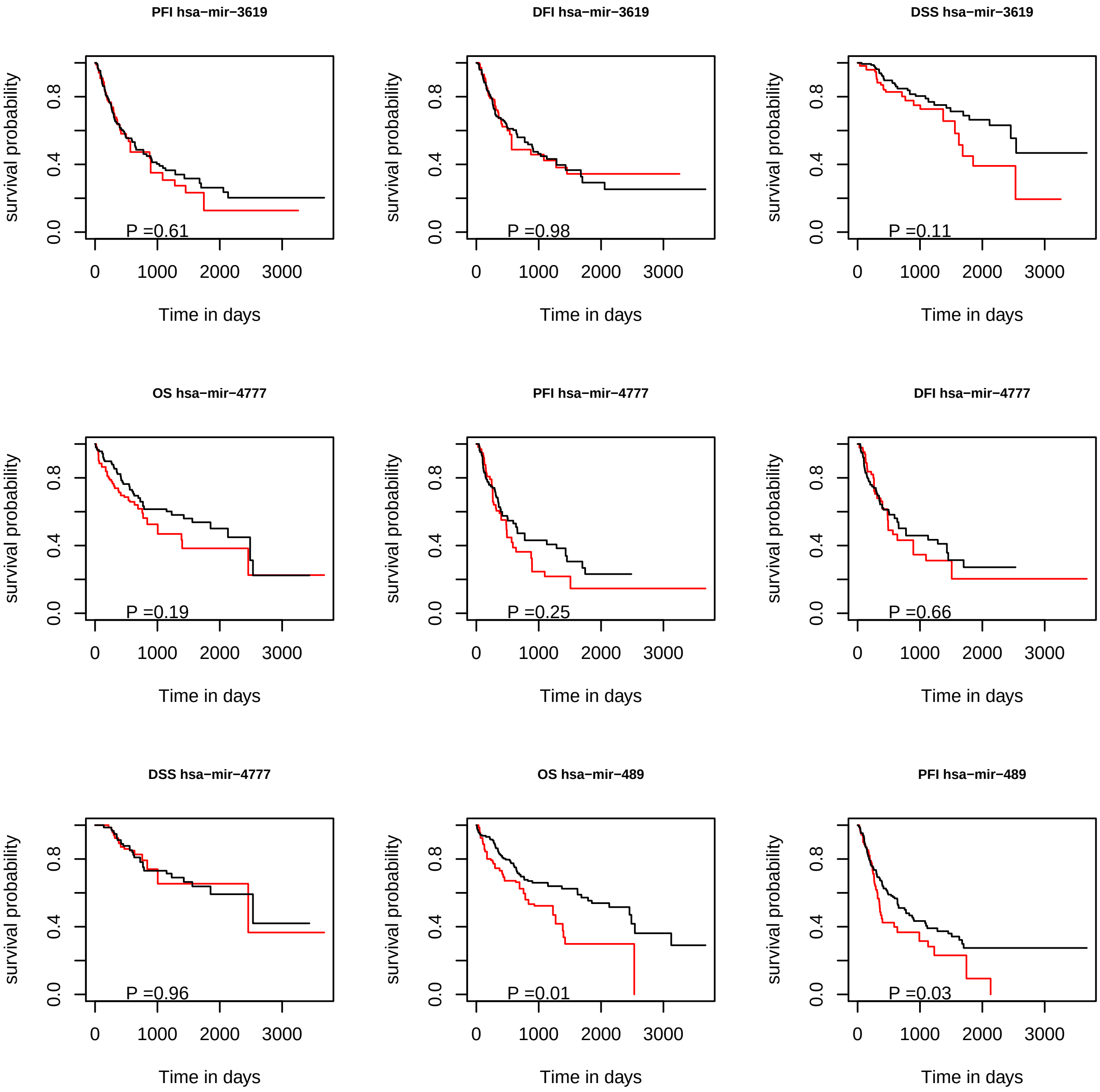

PFI hsa−mir−3619
P =0.61
DFI hsa−mir−3619
P =0.98
DSS hsa−mir−3619
P =0.11
OS hsa−mir−4777
P =0.19
PFI hsa−mir−4777
P =0.25
DFI hsa−mir−4777
P =0.66
DSS hsa−mir−4777
P =0.96
OS hsa−mir−489
P =0.01
PFI hsa−mir−489
P =0.03
survival probability
0.0
0.4
0.8
0
1000
2000
3000
Time in days

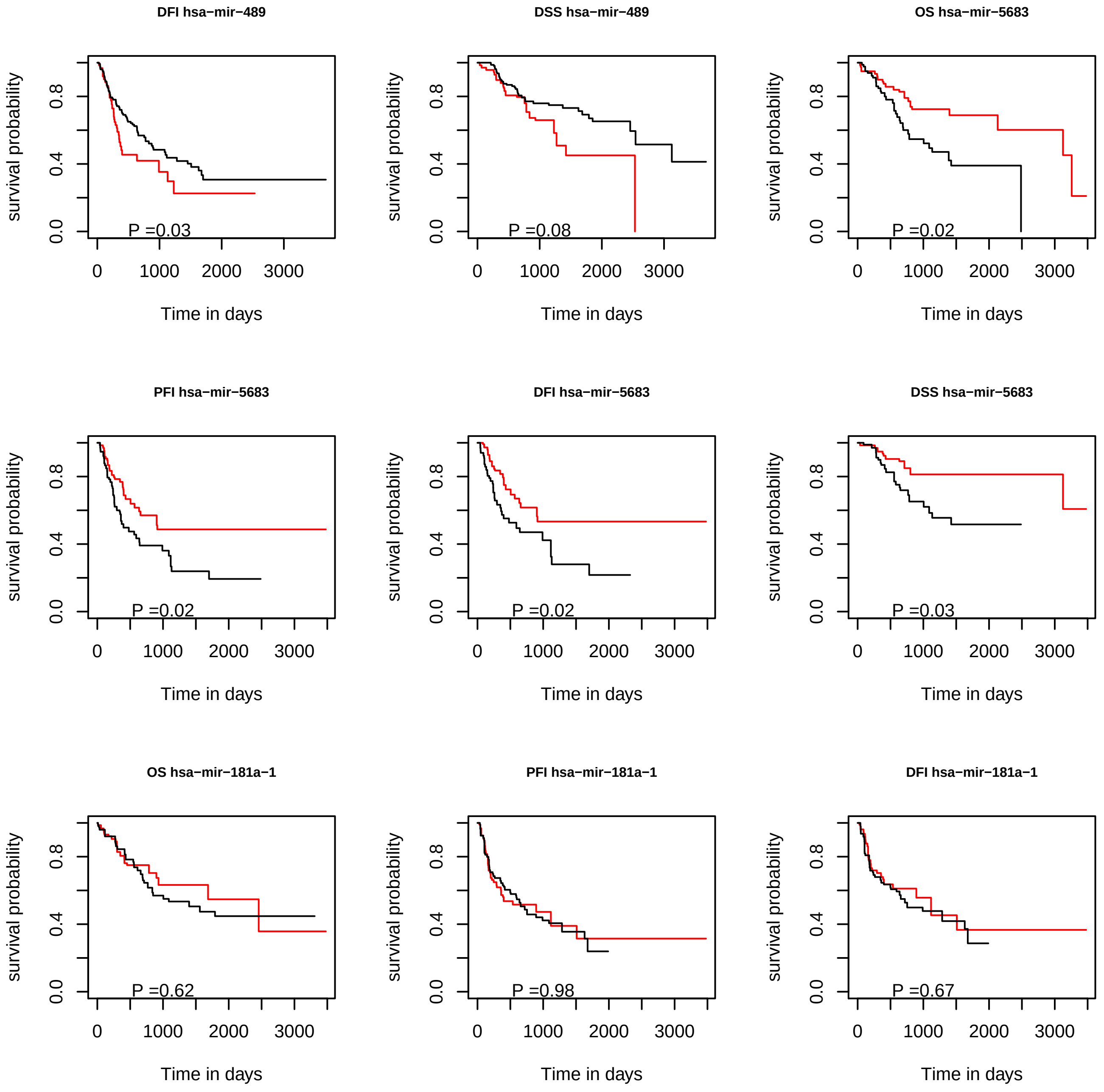
DFI hsa−mir−489
P =0.03
DSS hsa−mir−489
P =0.08
OS hsa−mir−5683
P =0.02
PFI hsa−mir−5683
P =0.02
DFI hsa−mir−5683
P =0.02
DSS hsa−mir−5683
P =0.03
OS hsa−mir−181a−1
P =0.62
PFI hsa−mir−181a−1
P =0.98
DFI hsa−mir−181a−1
P =0.67
survival probability
Time in days
0.0
0.4
0.8
0
1000
2000
3000

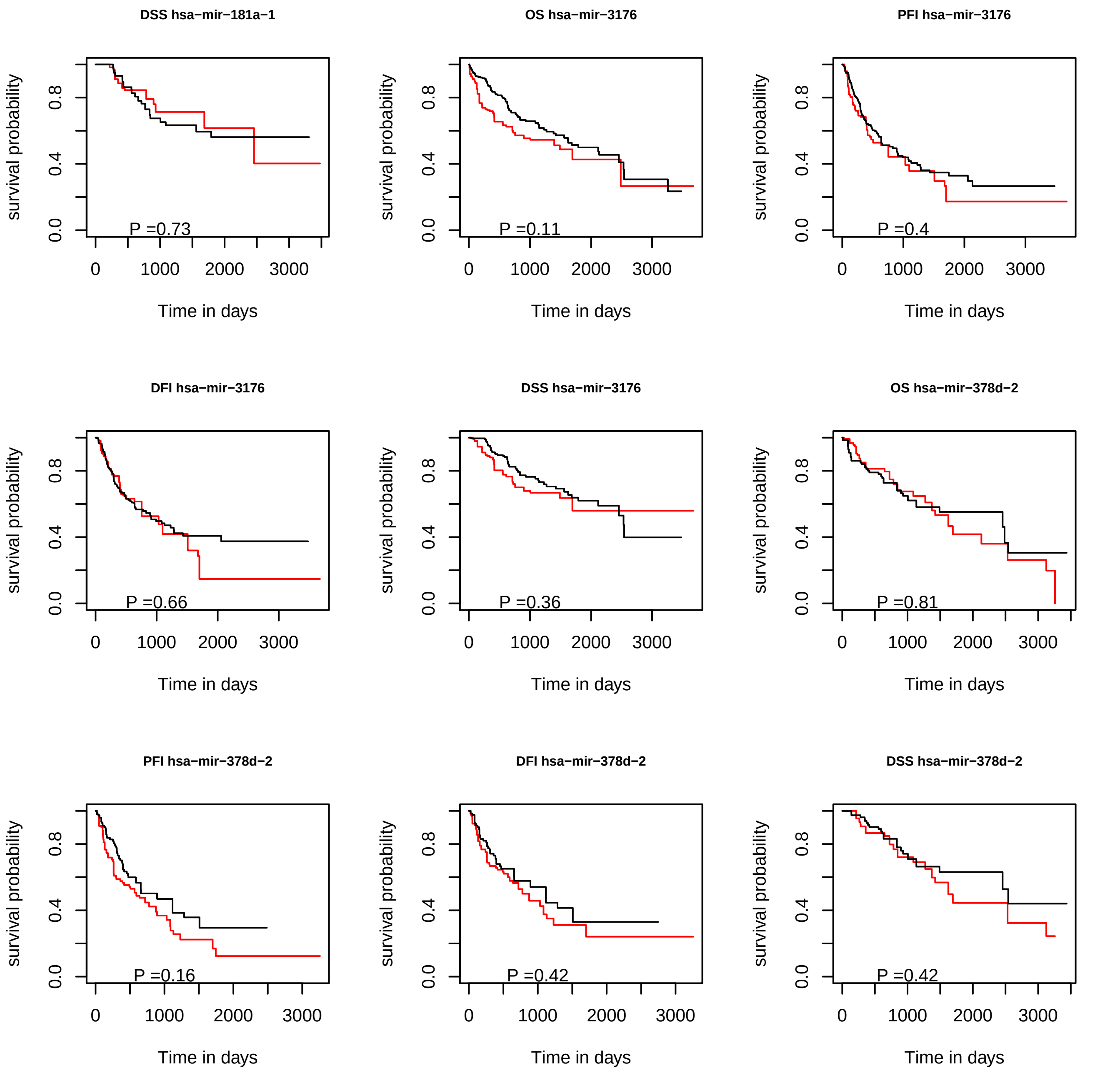
DSS hsa−mir−181a−1
P =0.73
OS hsa−mir−3176
P =0.11
PFI hsa−mir−3176
P =0.4
DFI hsa−mir−3176
P =0.66
DSS hsa−mir−3176
P =0.36
OS hsa−mir−378d−2
P =0.81
PFI hsa−mir−378d−2
P =0.16
DFI hsa−mir−378d−2
P =0.42
DSS hsa−mir−378d−2
P =0.42
survival probability
0.0
0.4
0.8
Time in days
0
1000
2000
3000

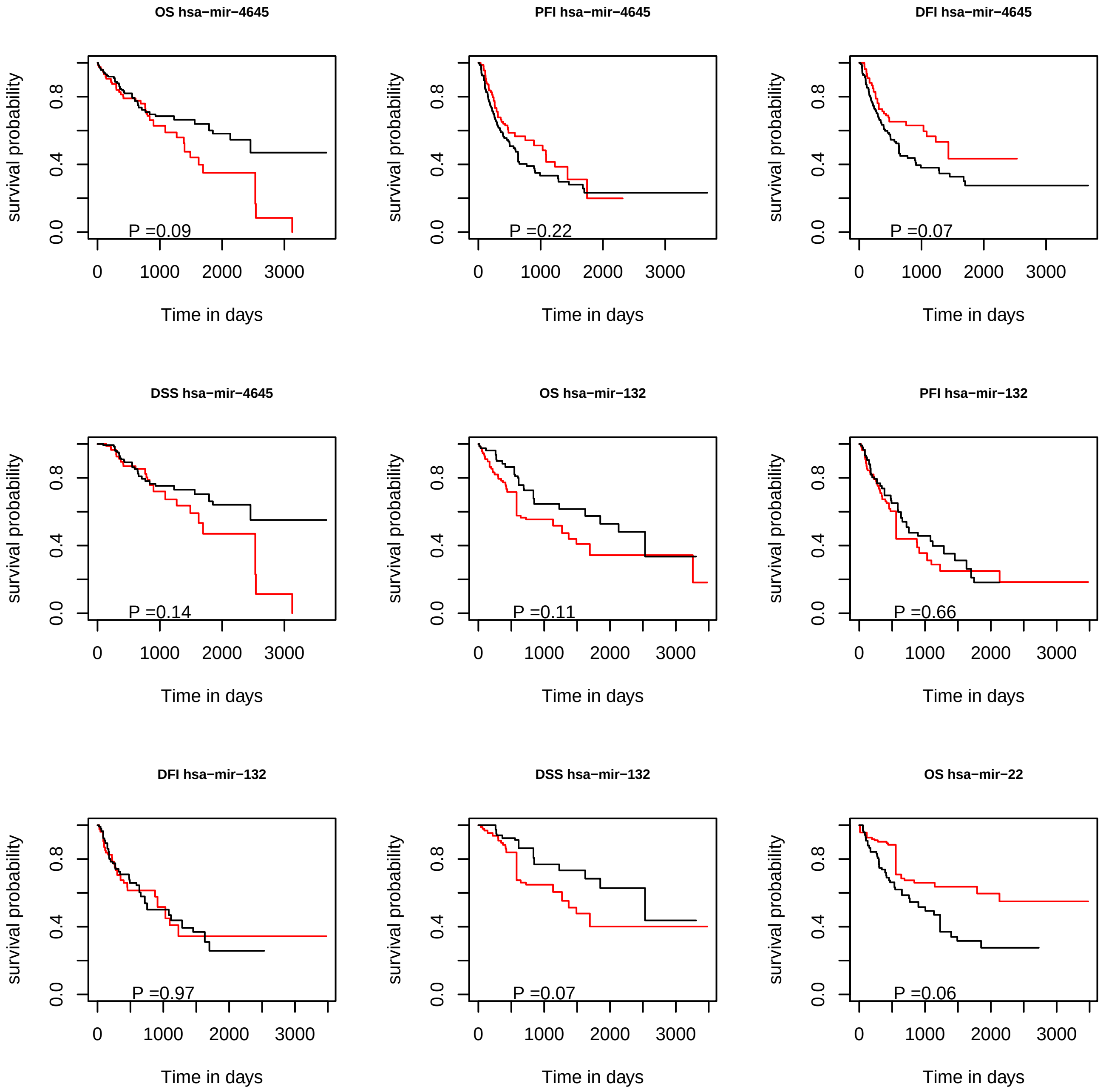
OS hsa−mir−4645
P =0.09
PFI hsa−mir−4645
P =0.22
DFI hsa−mir−4645
P =0.07
DSS hsa−mir−4645
P =0.14
OS hsa−mir−132
P =0.11
PFI hsa−mir−132
P =0.66
DFI hsa−mir−132
P =0.97
DSS hsa−mir−132
P =0.07
OS hsa−mir−22
P =0.06
survival probability
Time in days
0.0
0.4
0.8
0
1000
2000
3000

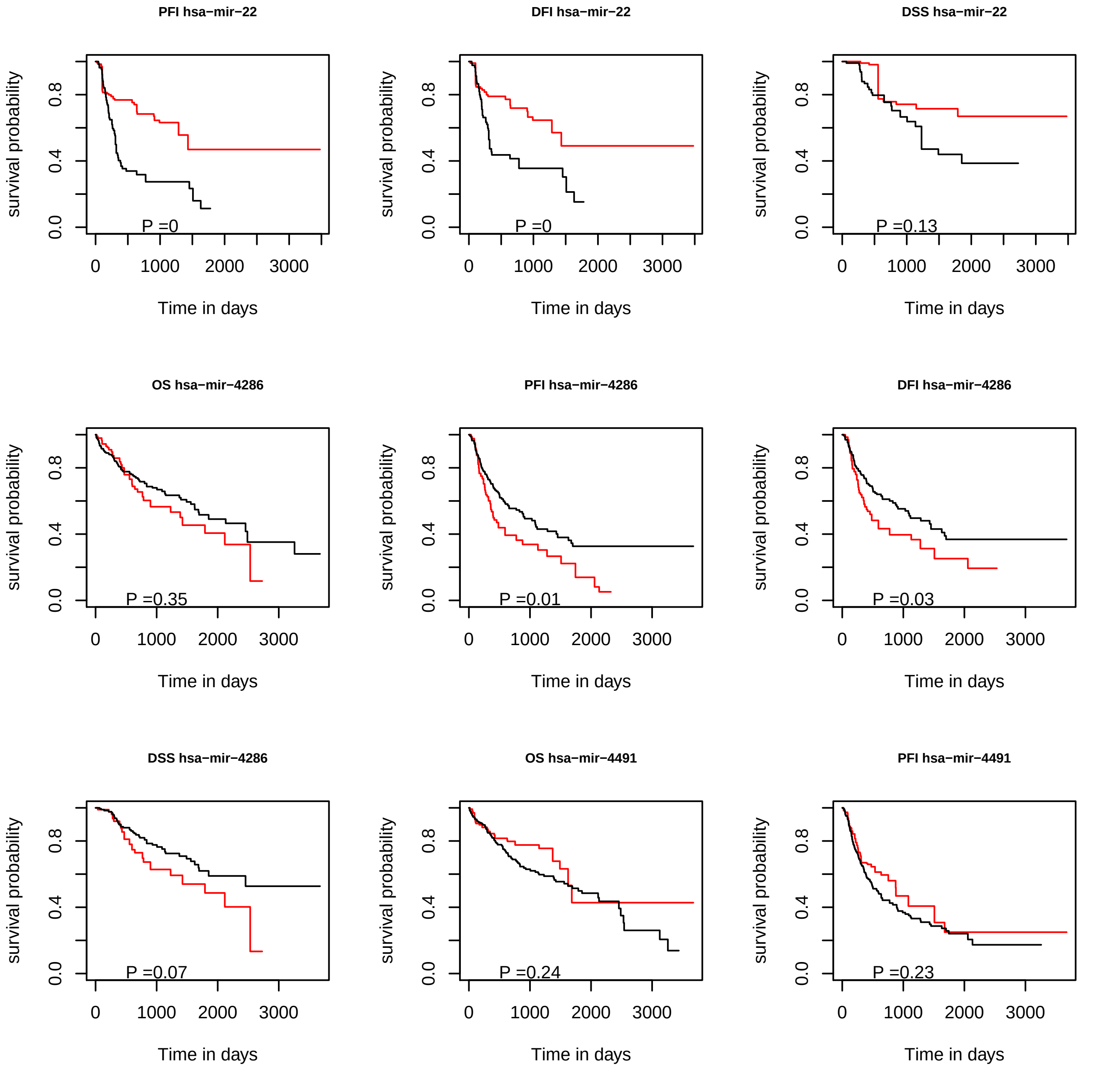
PFI hsa−mir−22
survival probability
0.0
0.4
0.8
P =0
0
1000
2000
3000
Time in days
DFI hsa−mir−22
survival probability
0.0
0.4
0.8
P =0
0
1000
2000
3000
Time in days
DSS hsa−mir−22
survival probability
0.0
0.4
0.8
P =0.13
0
1000
2000
3000
Time in days
OS hsa−mir−4286
survival probability
0.0
0.4
0.8
P =0.35
0
1000
2000
3000
Time in days
PFI hsa−mir−4286
survival probability
0.0
0.4
0.8
P =0.01
0
1000
2000
3000
Time in days
DFI hsa−mir−4286
survival probability
0.0
0.4
0.8
P =0.03
0
1000
2000
3000
Time in days
DSS hsa−mir−4286
survival probability
0.0
0.4
0.8
P =0.07
0
1000
2000
3000
Time in days
OS hsa−mir−4491
survival probability
0.0
0.4
0.8
P =0.24
0
1000
2000
3000
Time in days
PFI hsa−mir−4491
survival probability
0.0
0.4
0.8
P =0.23
0
1000
2000
3000
Time in days

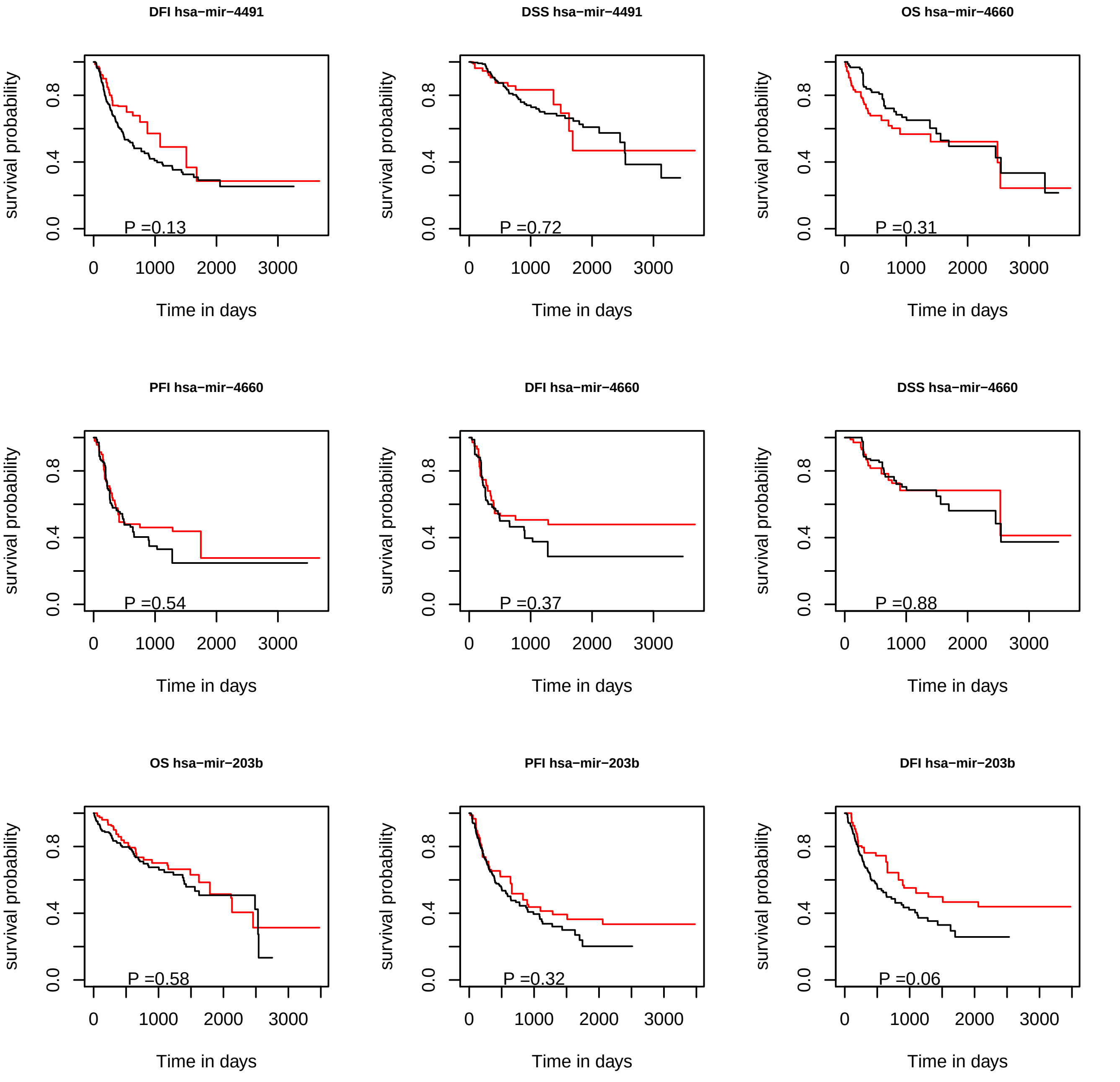
DFI hsa−mir−4491
P =0.13
DSS hsa−mir−4491
P =0.72
OS hsa−mir−4660
P =0.31
PFI hsa−mir−4660
P =0.54
DFI hsa−mir−4660
P =0.37
DSS hsa−mir−4660
P =0.88
OS hsa−mir−203b
P =0.58
PFI hsa−mir−203b
P =0.32
DFI hsa−mir−203b
P =0.06
survival probability
0.0
0.4
0.8
Time in days
0
1000
2000
3000

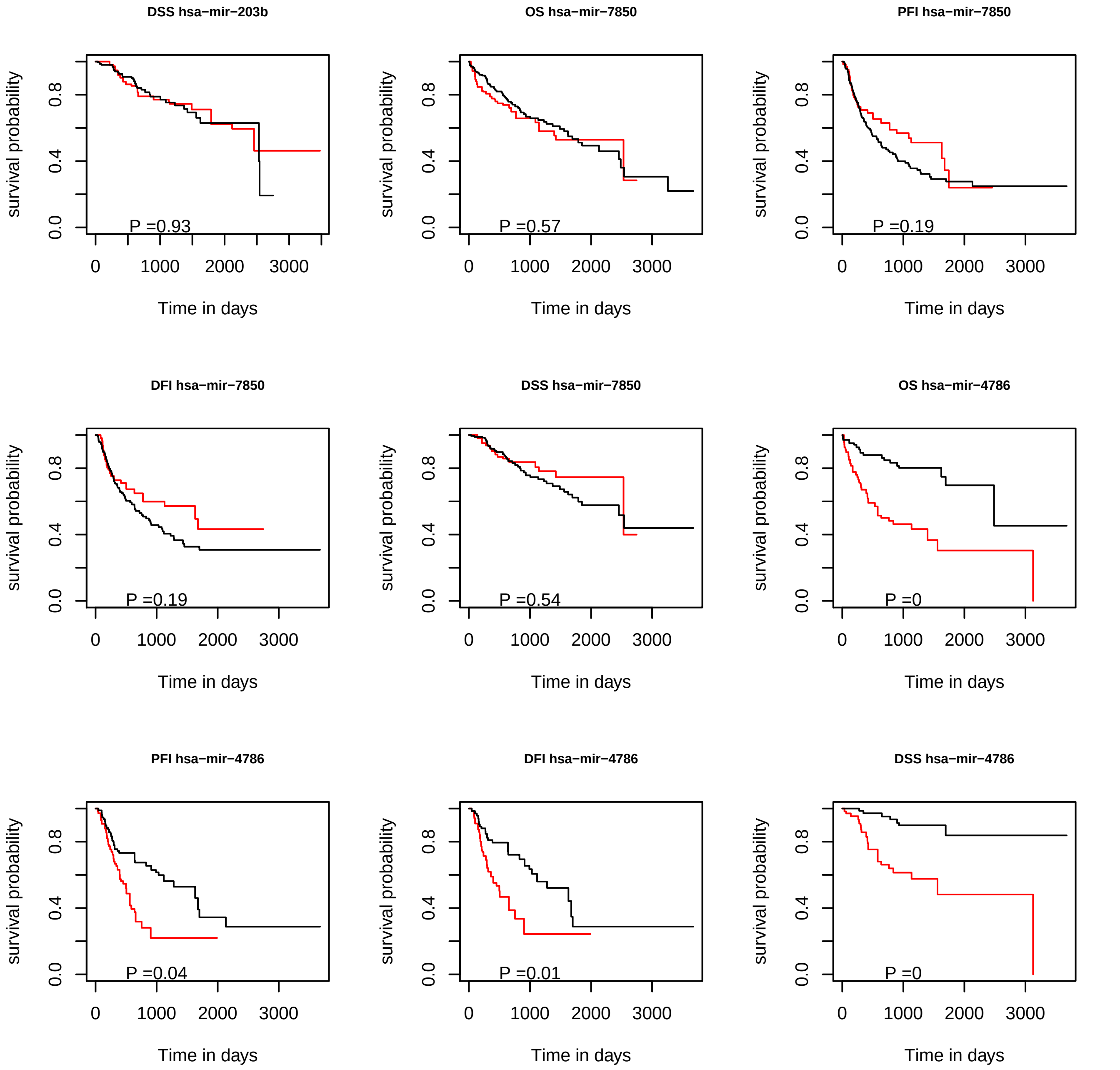

DSS hsa−mir−203b
P =0.93
OS hsa−mir−7850
P =0.57
PFI hsa−mir−7850
P =0.19
DFI hsa−mir−7850
P =0.19
DSS hsa−mir−7850
P =0.54
OS hsa−mir−4786
P =0
PFI hsa−mir−4786
P =0.04
DFI hsa−mir−4786
P =0.01
DSS hsa−mir−4786
P =0
survival probability
0.0
0.4
0.8
Time in days
0
1000
2000
3000

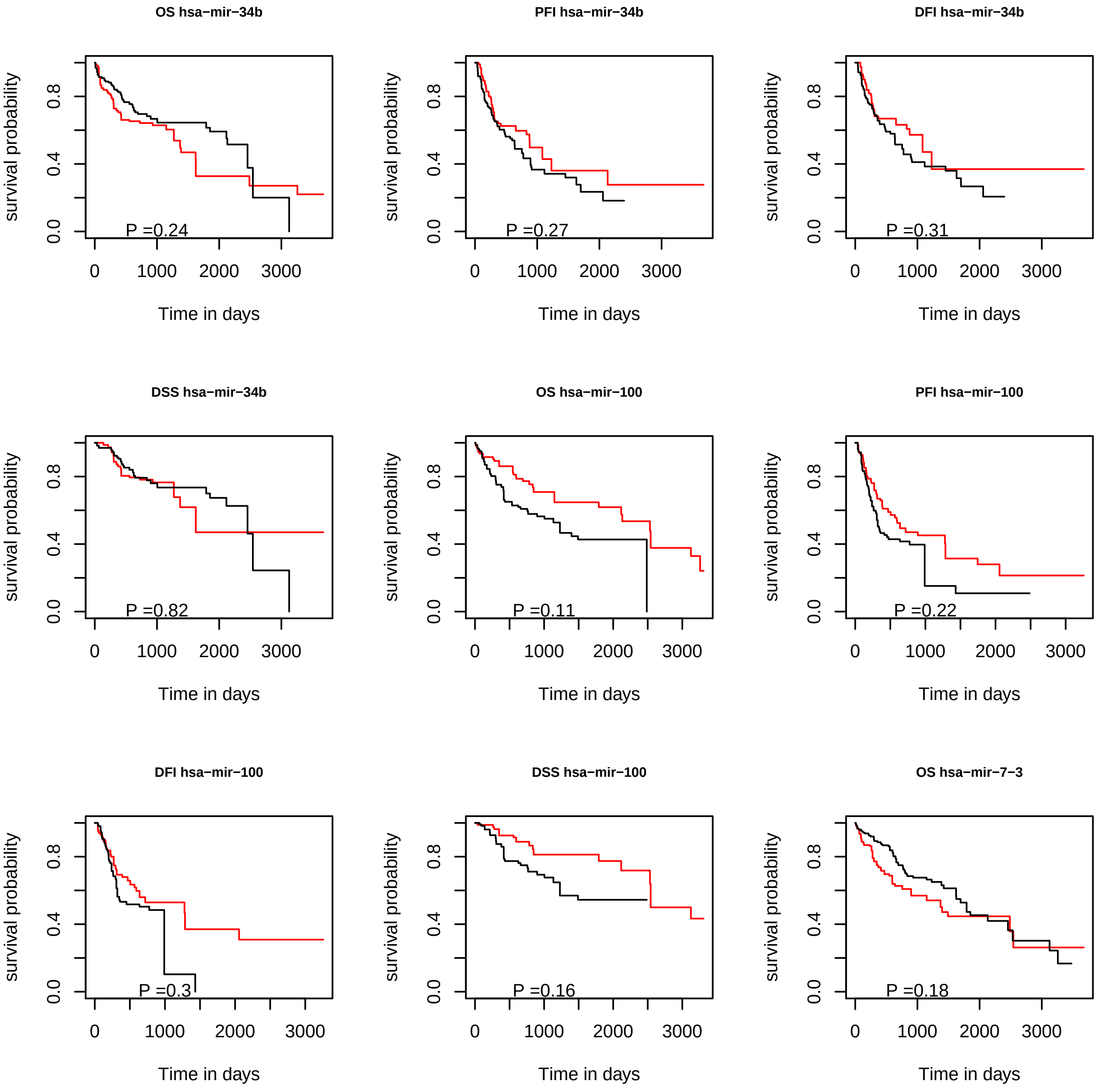
OS hsa−mir−34b
PFI hsa−mir−34b
DFI hsa−mir−34b
DSS hsa−mir−34b
OS hsa−mir−100
PFI hsa−mir−100
DFI hsa−mir−100
DSS hsa−mir−100
OS hsa−mir−7−3
survival probability
Time in days
0.0
0.4
0.8
0
1000
2000
3000
P =0.24
P =0.27
P =0.31
P =0.82
P =0.11
P =0.22
P =0.3
P =0.16
P =0.18

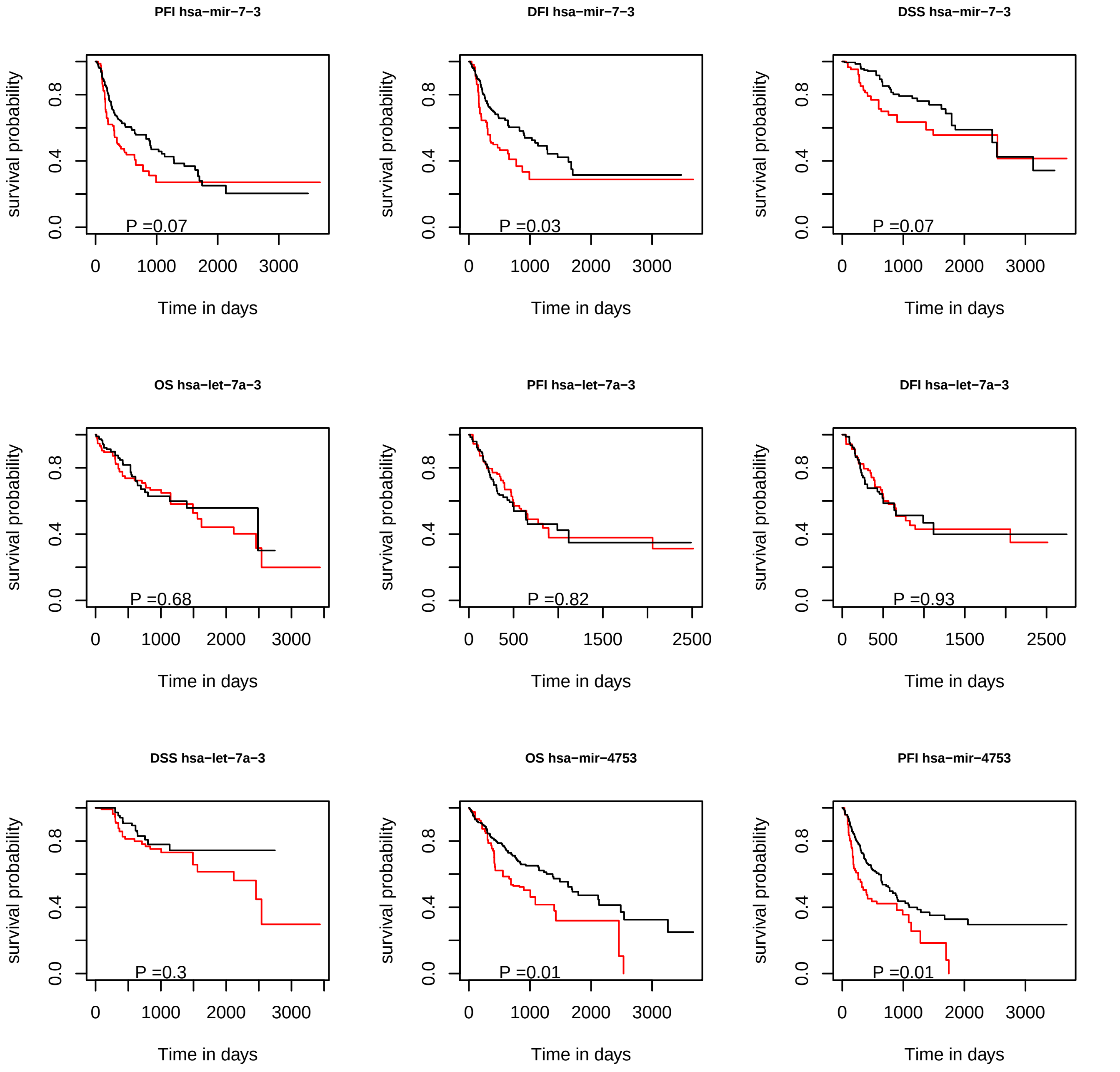
PFI hsa−mir−7−3
P =0.07
DFI hsa−mir−7−3
P =0.03
DSS hsa−mir−7−3
P =0.07
OS hsa−let−7a−3
P =0.68
PFI hsa−let−7a−3
P =0.82
DFI hsa−let−7a−3
P =0.93
DSS hsa−let−7a−3
P =0.3
OS hsa−mir−4753
P =0.01
PFI hsa−mir−4753
P =0.01
survival probability
Time in days

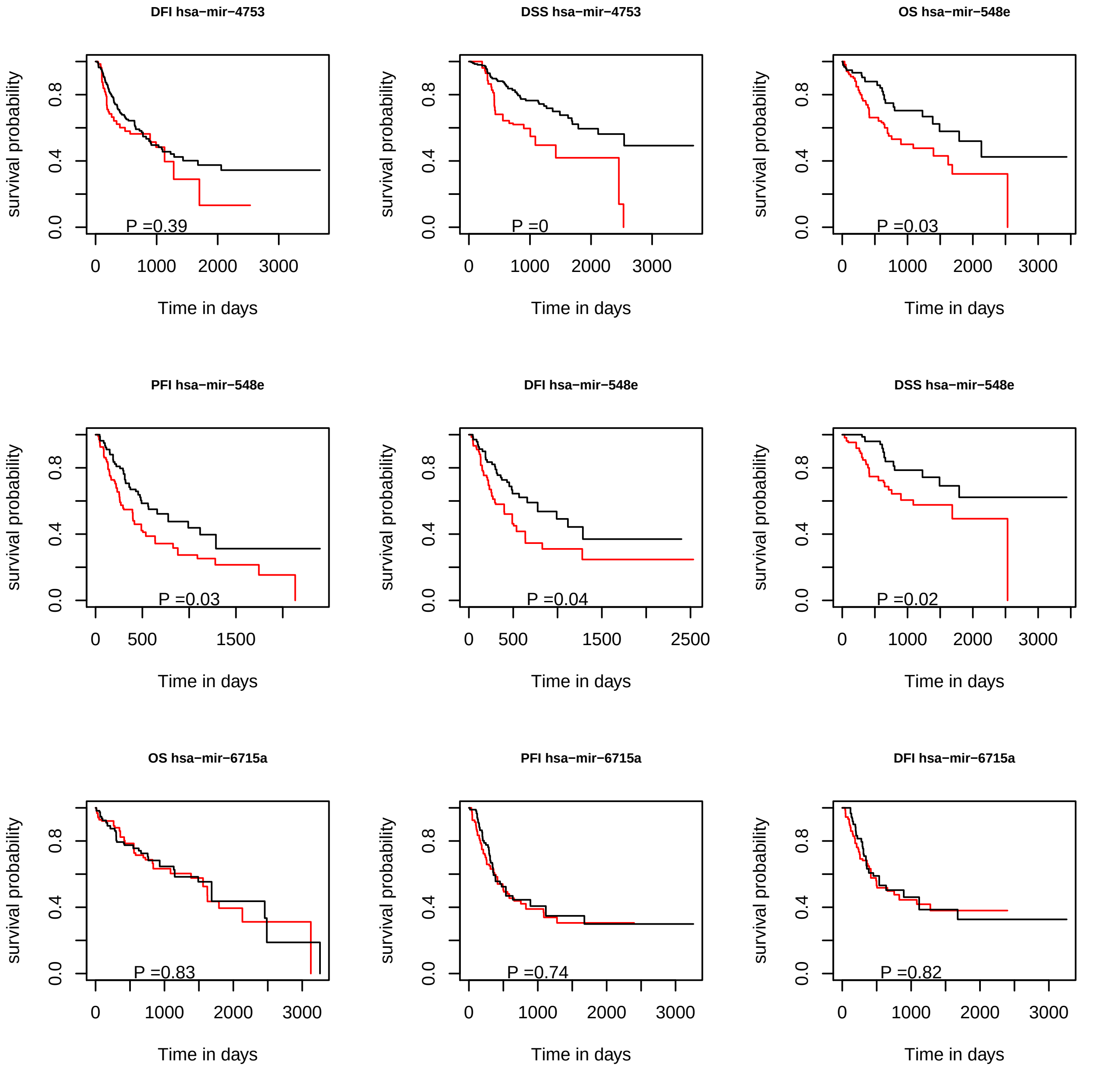

DFI hsa−mir−4753
P =0.39
DSS hsa−mir−4753
P =0
OS hsa−mir−548e
P =0.03
PFI hsa−mir−548e
P =0.03
DFI hsa−mir−548e
P =0.04
DSS hsa−mir−548e
P =0.02
OS hsa−mir−6715a
P =0.83
PFI hsa−mir−6715a
P =0.74
DFI hsa−mir−6715a
P =0.82
survival probability
Time in days

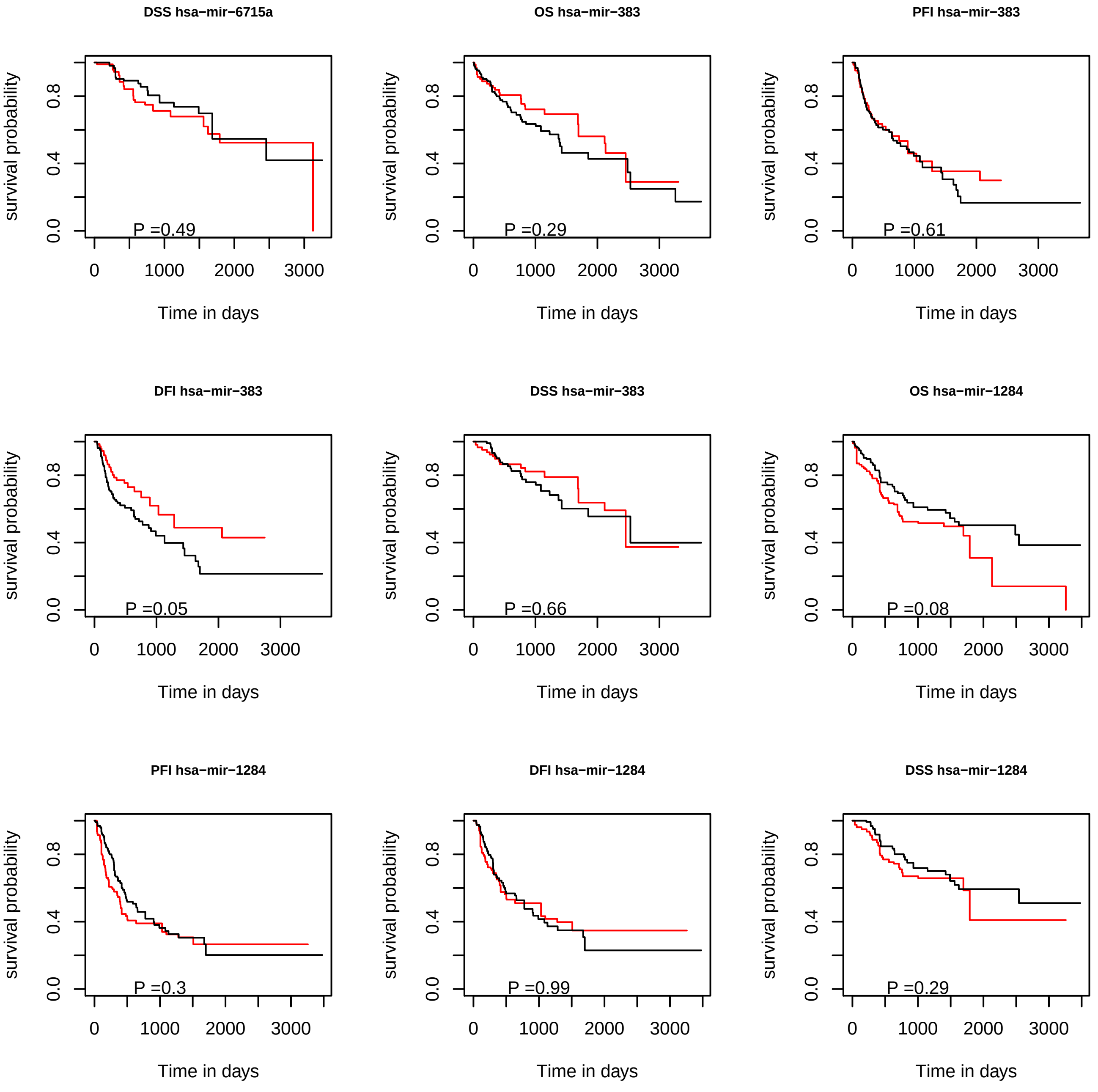

DSS hsa−mir−6715a
P =0.49
OS hsa−mir−383
P =0.29
PFI hsa−mir−383
P =0.61
DFI hsa−mir−383
P =0.05
DSS hsa−mir−383
P =0.66
OS hsa−mir−1284
P =0.08
PFI hsa−mir−1284
P =0.3
DFI hsa−mir−1284
P =0.99
DSS hsa−mir−1284
P =0.29
survival probability
0.0
0.4
0.8
Time in days
0
1000
2000
3000

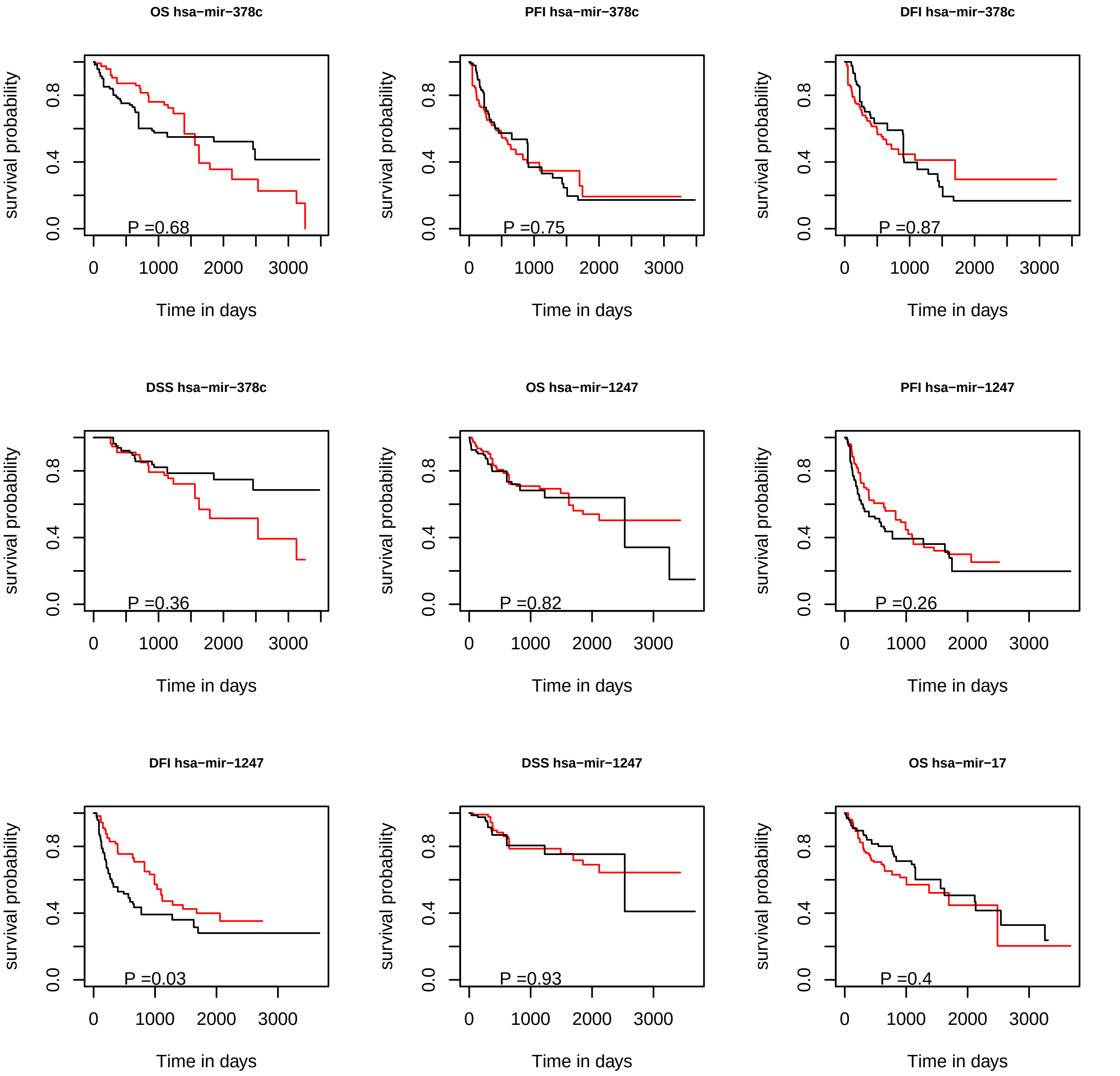
OS hsa−mir−378c
P =0.68
PFI hsa−mir−378c
P =0.75
DFI hsa−mir−378c
P =0.87
DSS hsa−mir−378c
P =0.36
OS hsa−mir−1247
P =0.82
PFI hsa−mir−1247
P =0.26
DFI hsa−mir−1247
P =0.03
DSS hsa−mir−1247
P =0.93
OS hsa−mir−17
P =0.4
survival probability
0.0
0.4
0.8
Time in days
0
1000
2000
3000

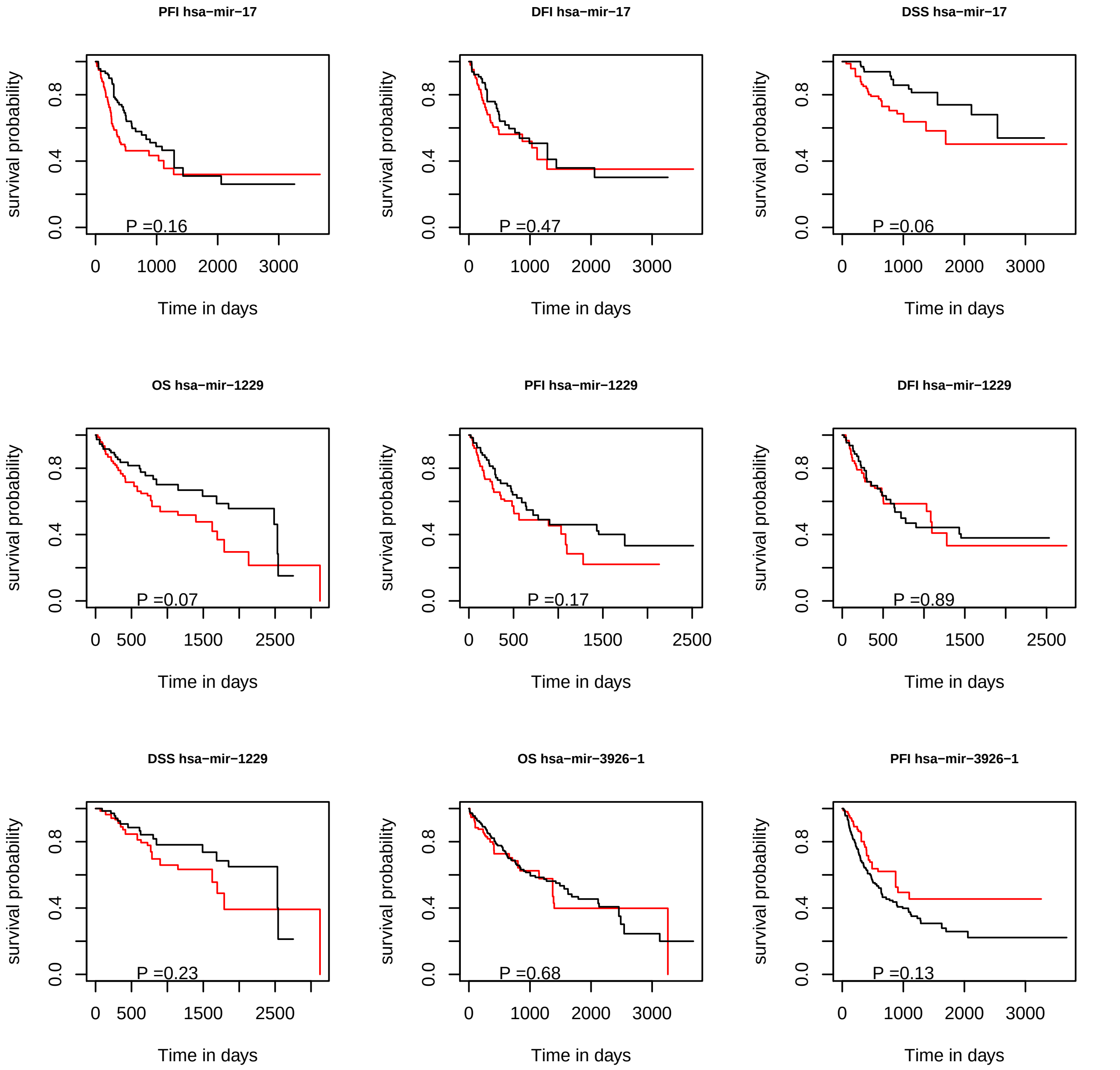
PFI hsa−mir−17
P =0.16
DFI hsa−mir−17
P =0.47
DSS hsa−mir−17
P =0.06
OS hsa−mir−1229
P =0.07
PFI hsa−mir−1229
P =0.17
DFI hsa−mir−1229
P =0.89
DSS hsa−mir−1229
P =0.23
OS hsa−mir−3926−1
P =0.68
PFI hsa−mir−3926−1
P =0.13
survival probability
Time in days
0.0
0.4
0.8
0
500
1000
1500
2000
2500
3000

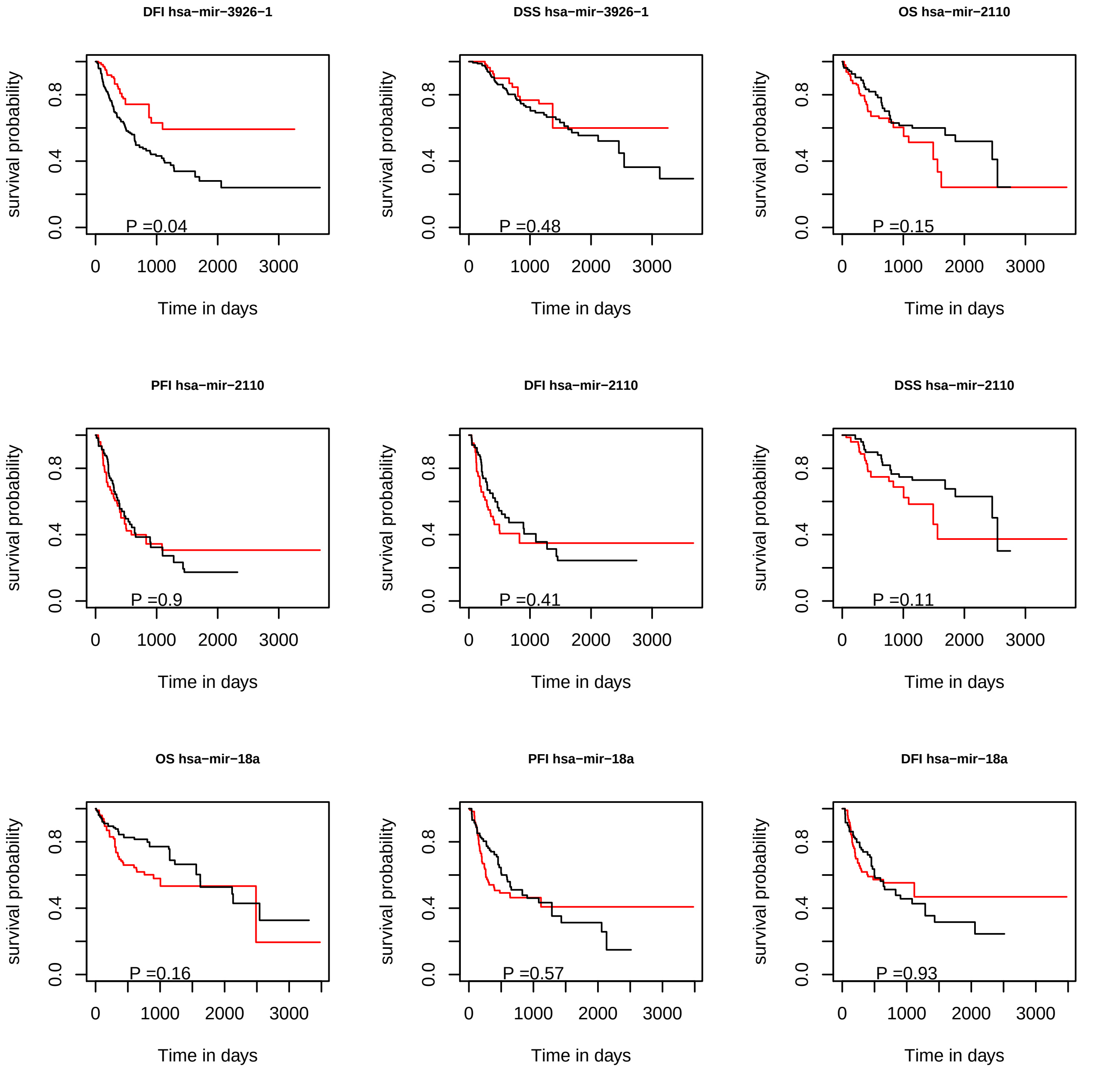
DFI hsa−mir−3926−1
P =0.04
DSS hsa−mir−3926−1
P =0.48
OS hsa−mir−2110
P =0.15
PFI hsa−mir−2110
P =0.9
DFI hsa−mir−2110
P =0.41
DSS hsa−mir−2110
P =0.11
OS hsa−mir−18a
P =0.16
PFI hsa−mir−18a
P =0.57
DFI hsa−mir−18a
P =0.93
survival probability
0.0
0.4
0.8
Time in days
0
1000
2000
3000

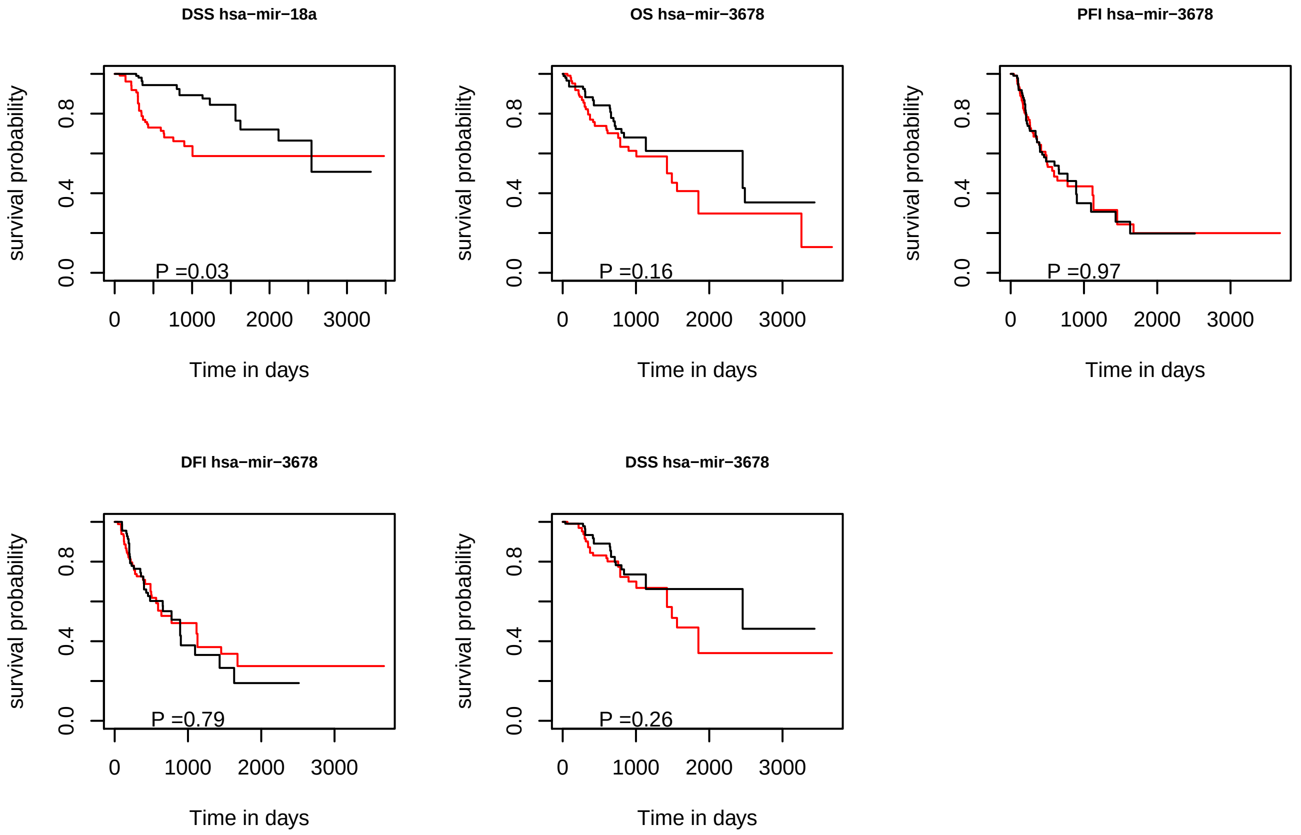
DSS hsa−mir−18a
P =0.03
OS hsa−mir−3678
P =0.16
PFI hsa−mir−3678
P =0.97
DFI hsa−mir−3678
P =0.79
DSS hsa−mir−3678
P =0.26
survival probability
Time in days
0.0
0.4
0.8
0
1000
2000
3000
