# Supplementary material for: Computing microRNA-gene interaction networks in pan-cancer using miRDriver
Source: Sci Rep. 2022 Mar 8;12:3717. doi: 10.1038/s41598-022-07628-z (PMC8904490; doi:10.1038/s41598-022-07628-z)
Supplement: Supplementary file 21 — Supplementary Information 21. [file 41598_2022_7628_MOESM21_ESM.pdf]

Computing microRNA-gene interaction networks in pan-cancer using miRDriver

Banabithi Bose, Matthew Moravec, and Serdar Bozdag

Supplemental Figure S12

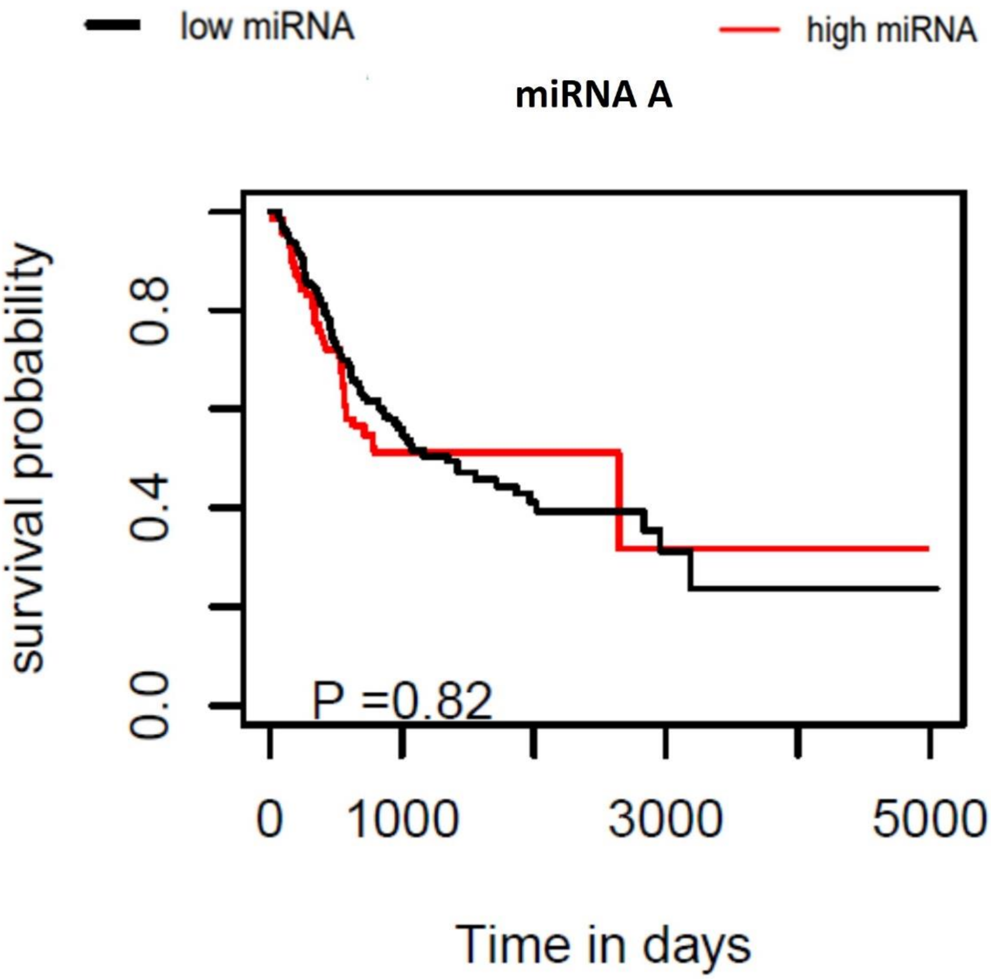

The *Adjusted Kaplan-Meier* survival plots for the computed miRNAs in high and low miRNA expression patient groups.

Supplemental Figure S12 Cancer Type: LUAD

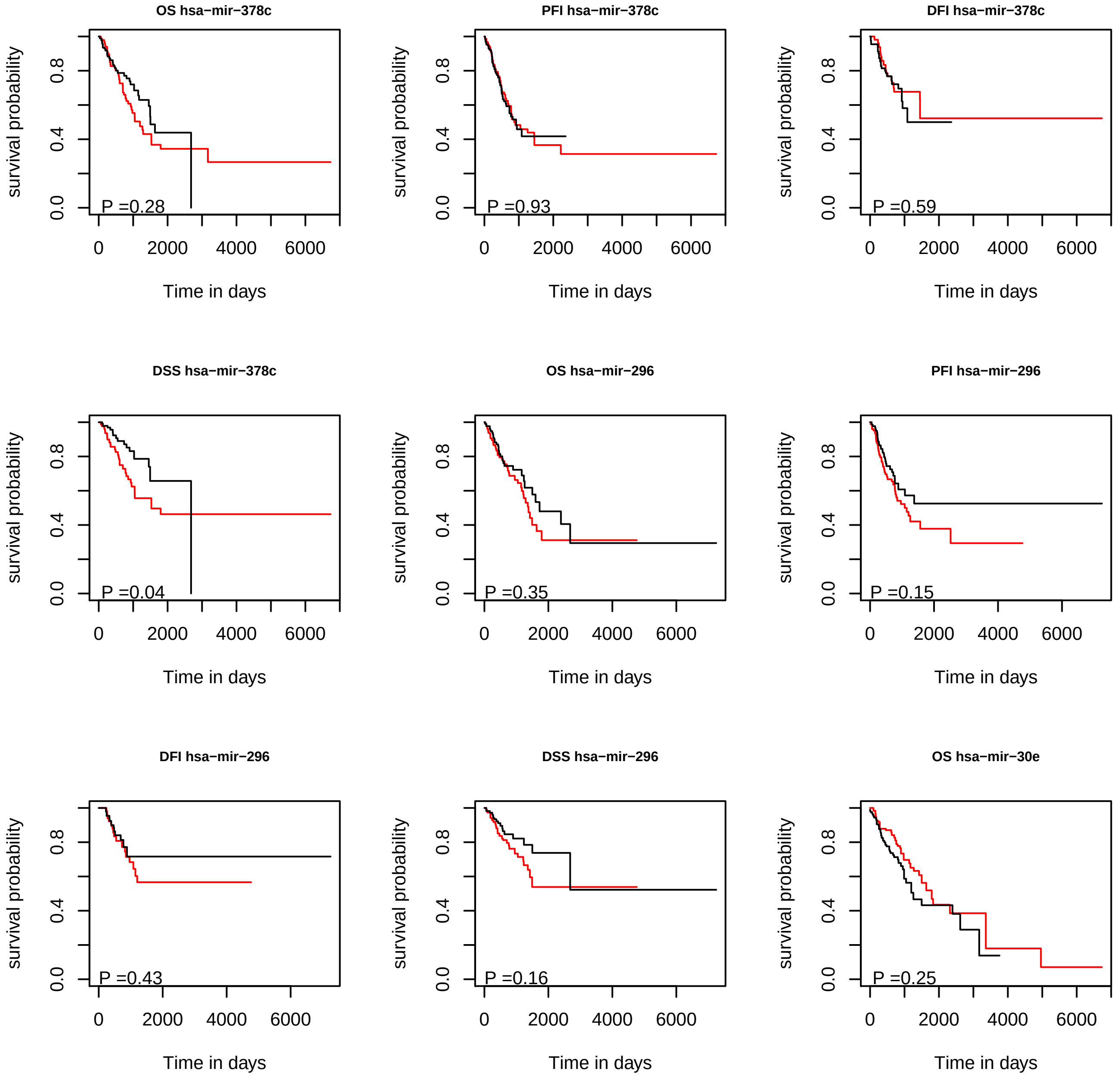

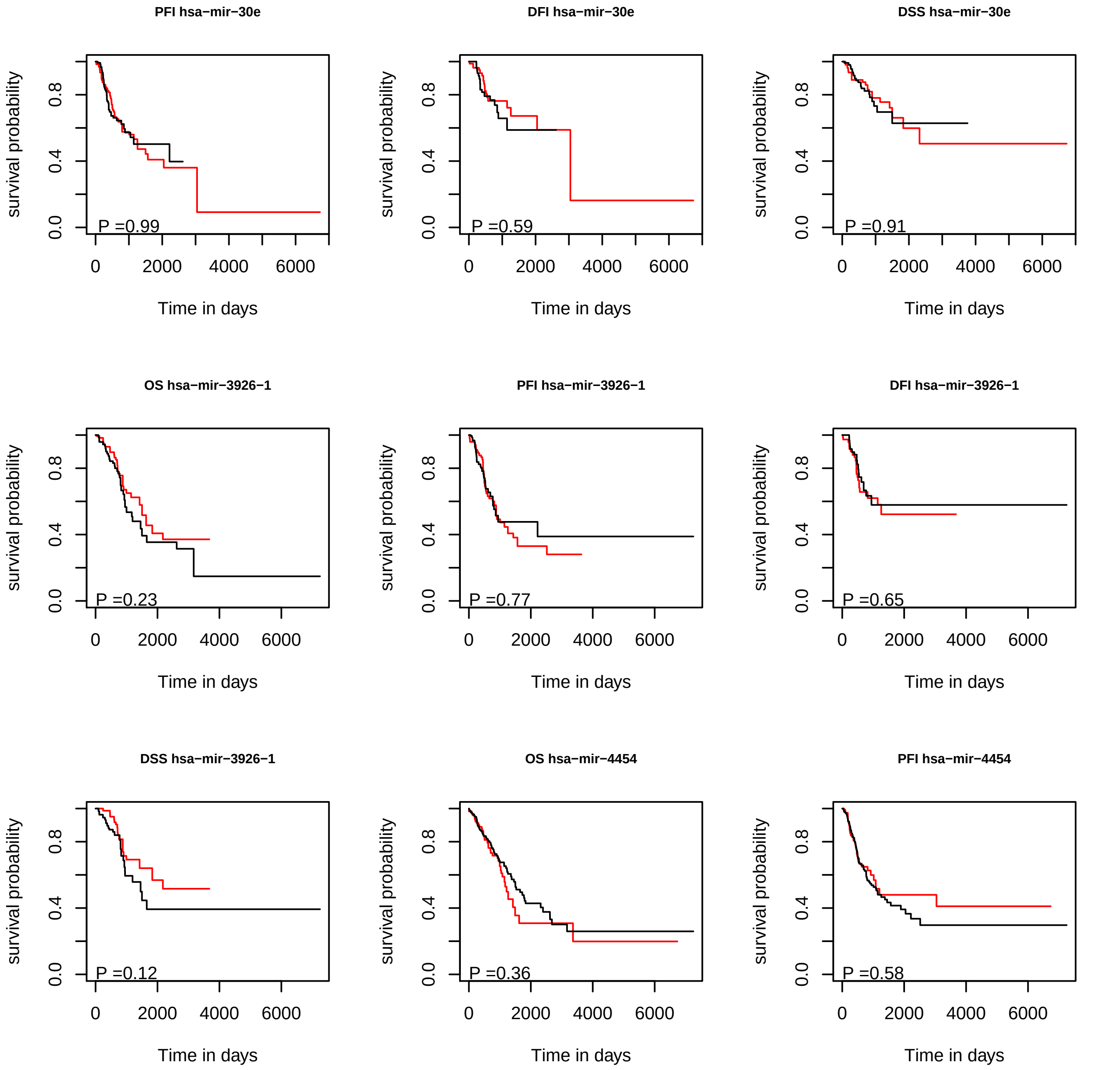
PFI hsa−mir−30e
P =0.99
DFI hsa−mir−30e
P =0.59
DSS hsa−mir−30e
P =0.91
OS hsa−mir−3926−1
P =0.23
PFI hsa−mir−3926−1
P =0.77
DFI hsa−mir−3926−1
P =0.65
DSS hsa−mir−3926−1
P =0.12
OS hsa−mir−4454
P =0.36
PFI hsa−mir−4454
P =0.58
survival probability
0.0
0.4
0.8
Time in days
0
2000
4000
6000

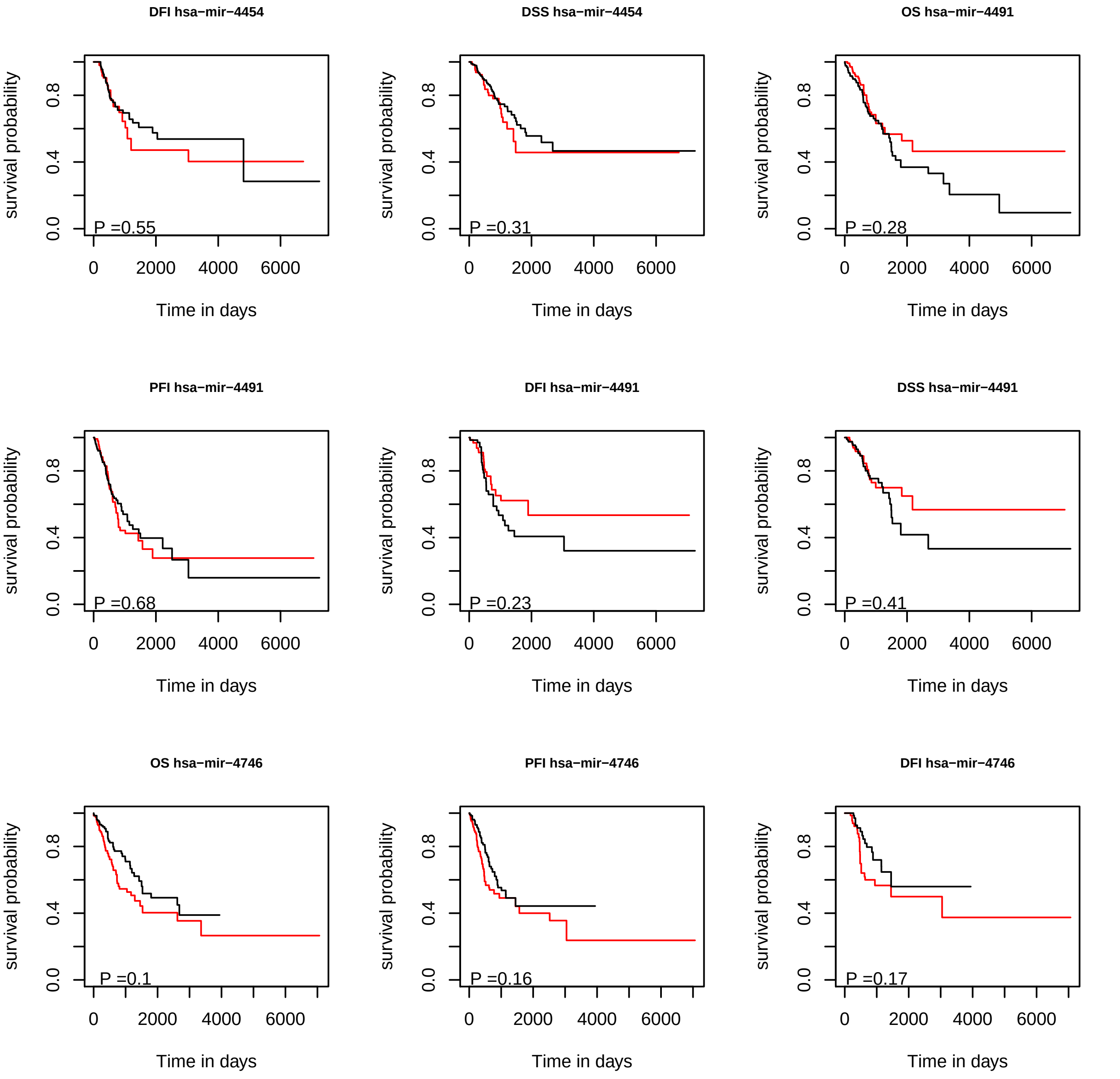
DFI hsa−mir−4454
P =0.55
DSS hsa−mir−4454
P =0.31
OS hsa−mir−4491
P =0.28
PFI hsa−mir−4491
P =0.68
DFI hsa−mir−4491
P =0.23
DSS hsa−mir−4491
P =0.41
OS hsa−mir−4746
P =0.1
PFI hsa−mir−4746
P =0.16
DFI hsa−mir−4746
P =0.17
survival probability
Time in days
0.0
0.4
0.8
0
2000
4000
6000

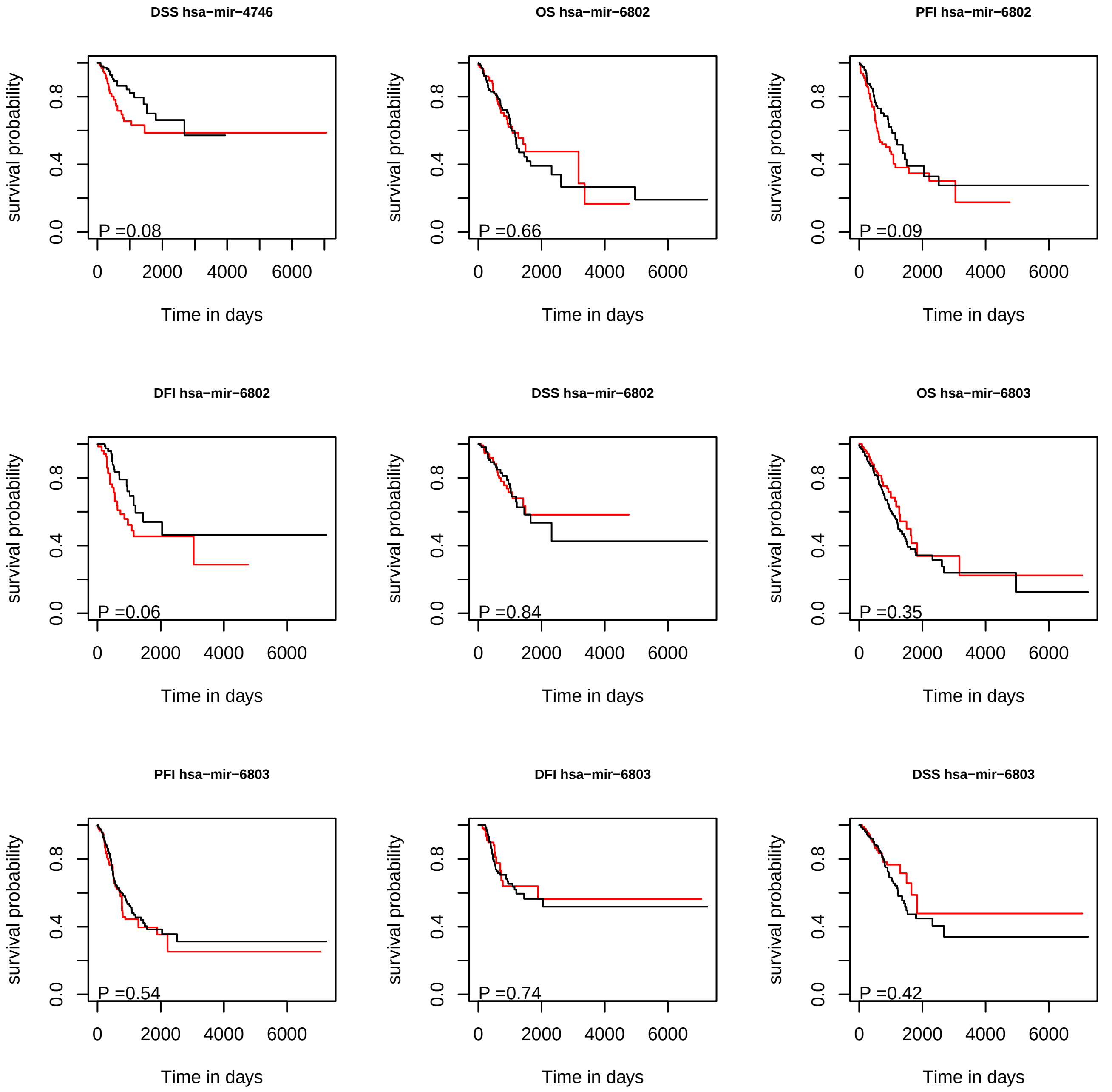
DSS hsa−mir−4746
P =0.08
OS hsa−mir−6802
P =0.66
PFI hsa−mir−6802
P =0.09
DFI hsa−mir−6802
P =0.06
DSS hsa−mir−6802
P =0.84
OS hsa−mir−6803
P =0.35
PFI hsa−mir−6803
P =0.54
DFI hsa−mir−6803
P =0.74
DSS hsa−mir−6803
P =0.42
survival probability
Time in days
0.0
0.4
0.8
0
2000
4000
6000

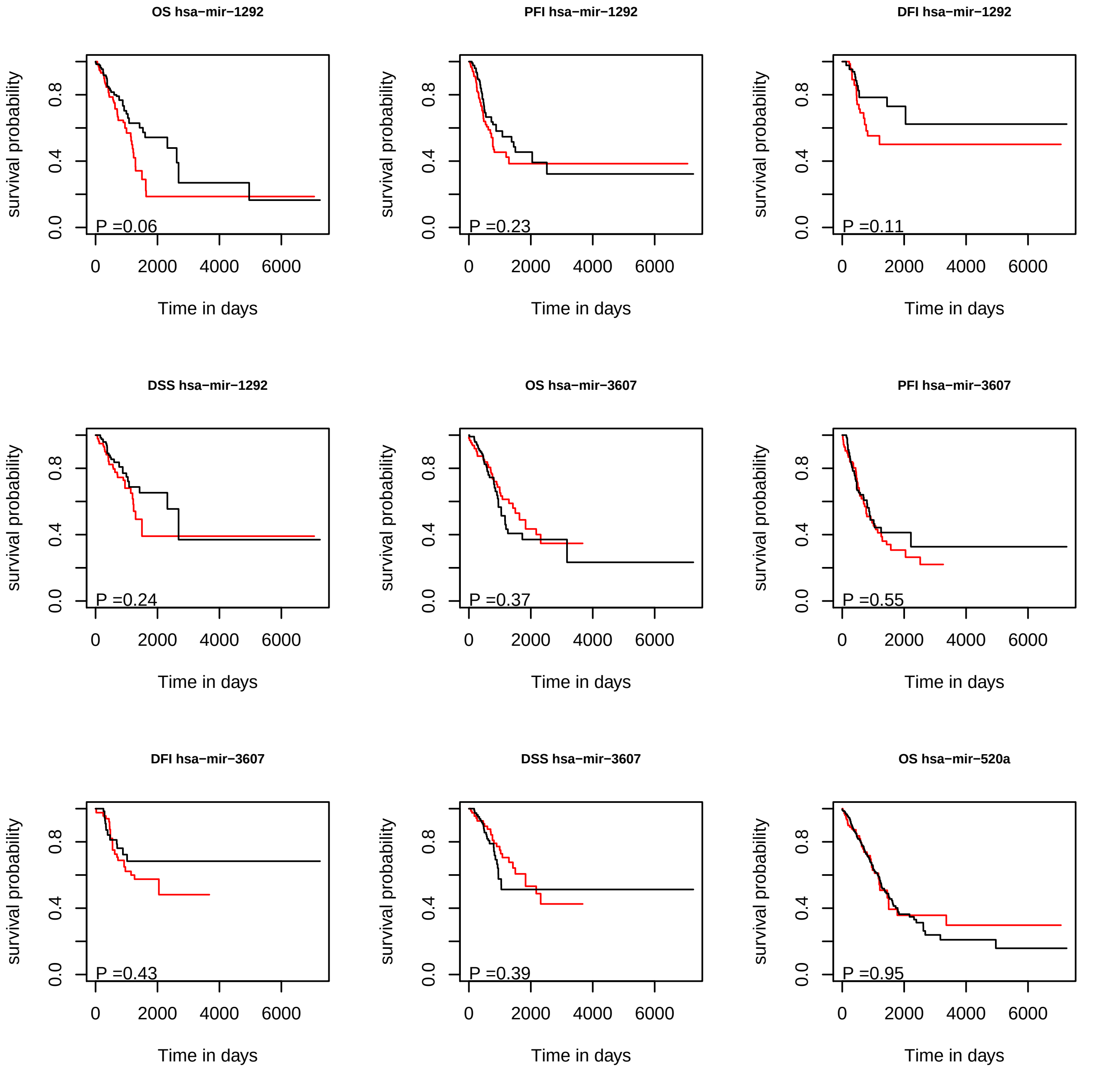
OS hsa−mir−1292
P =0.06
PFI hsa−mir−1292
P =0.23
DFI hsa−mir−1292
P =0.11
DSS hsa−mir−1292
P =0.24
OS hsa−mir−3607
P =0.37
PFI hsa−mir−3607
P =0.55
DFI hsa−mir−3607
P =0.43
DSS hsa−mir−3607
P =0.39
OS hsa−mir−520a
P =0.95
survival probability
0.0
0.4
0.8
0
2000
4000
6000
Time in days

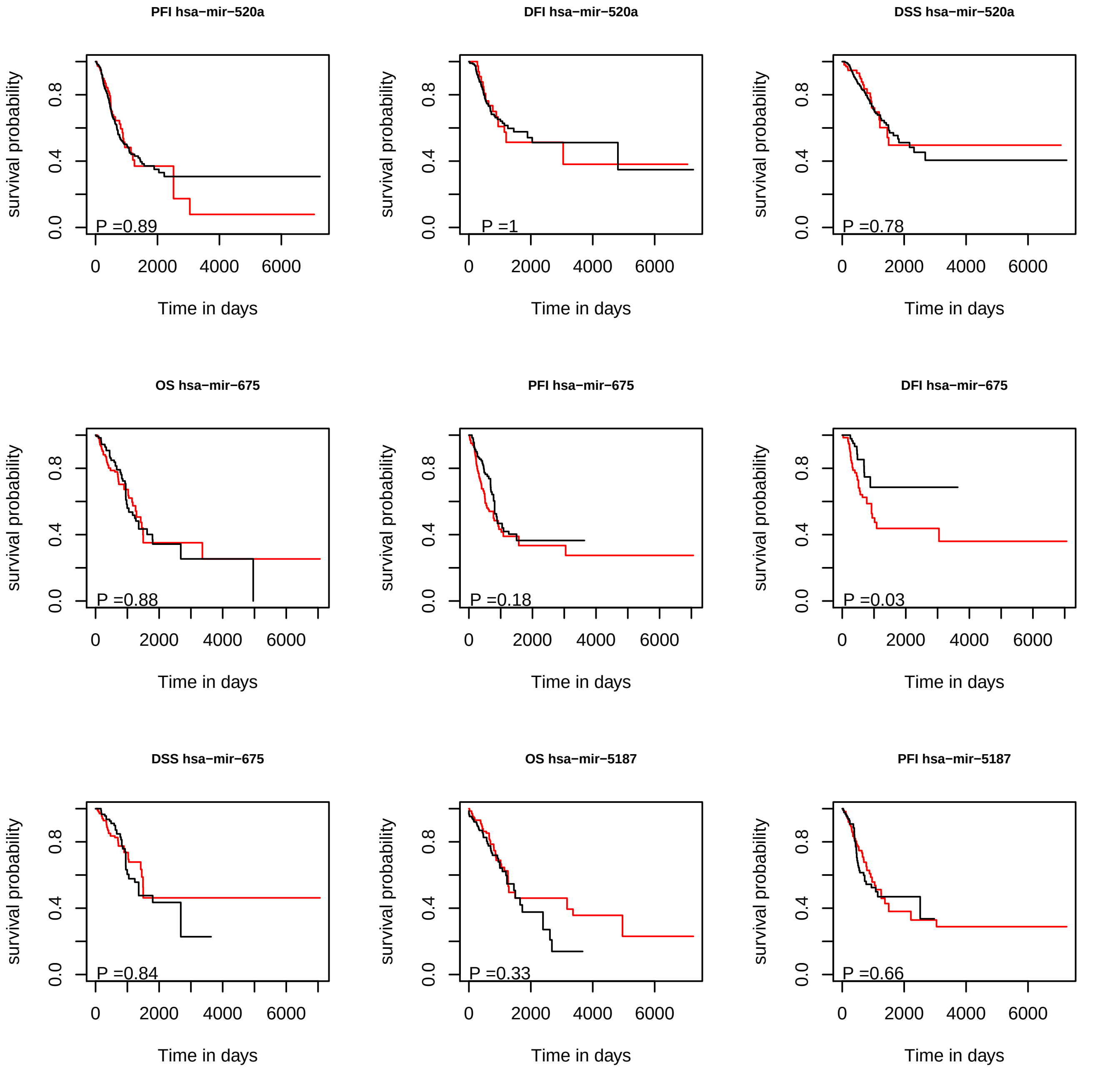
PFI hsa−mir−520a
P =0.89
DFI hsa−mir−520a
P =1
DSS hsa−mir−520a
P =0.78
OS hsa−mir−675
P =0.88
PFI hsa−mir−675
P =0.18
DFI hsa−mir−675
P =0.03
DSS hsa−mir−675
P =0.84
OS hsa−mir−5187
P =0.33
PFI hsa−mir−5187
P =0.66
survival probability
Time in days
0.0
0.4
0.8
0
2000
4000
6000

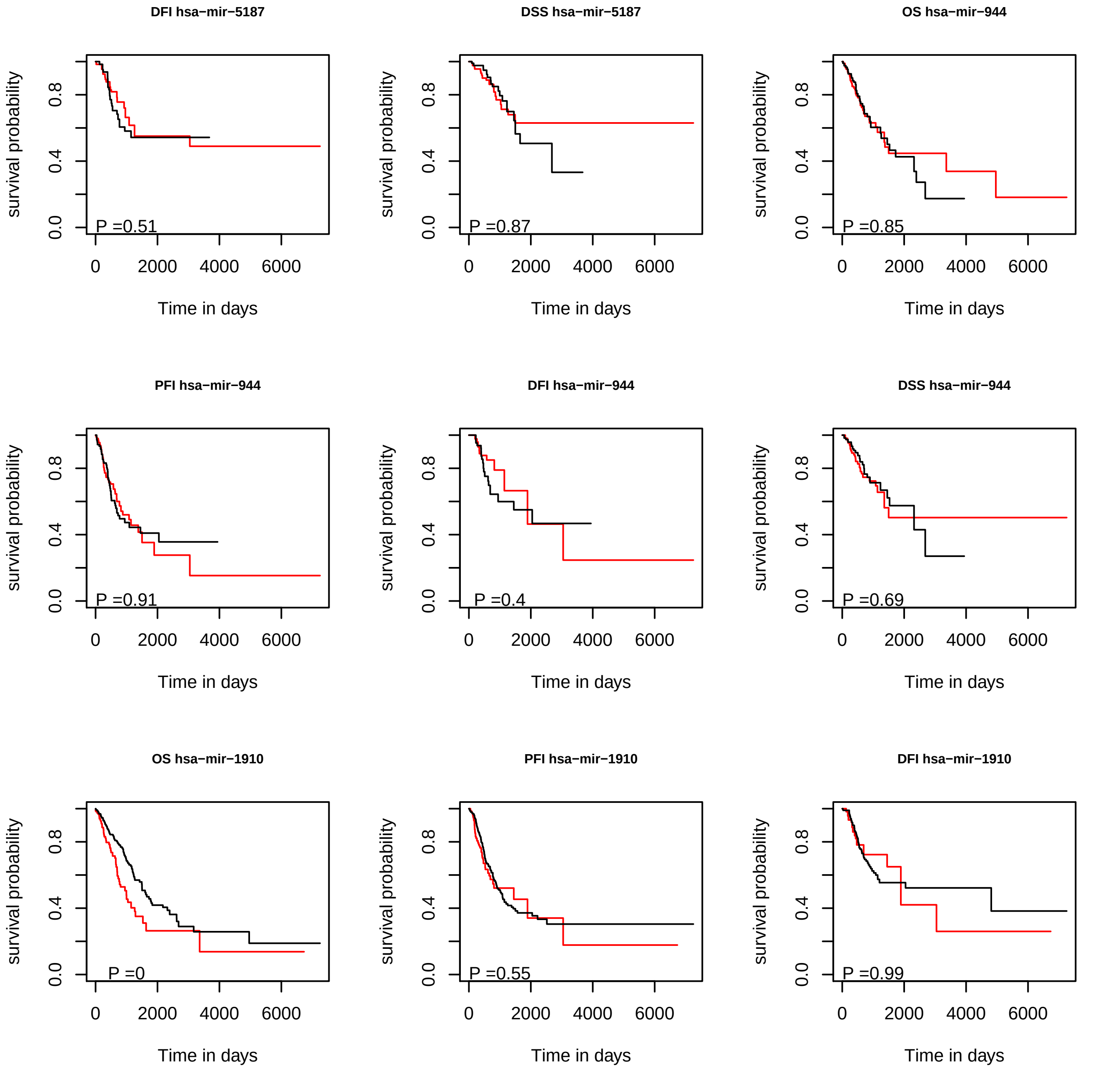
DFI hsa−mir−5187
P =0.51
DSS hsa−mir−5187
P =0.87
OS hsa−mir−944
P =0.85
PFI hsa−mir−944
P =0.91
DFI hsa−mir−944
P =0.4
DSS hsa−mir−944
P =0.69
OS hsa−mir−1910
P =0
PFI hsa−mir−1910
P =0.55
DFI hsa−mir−1910
P =0.99
survival probability
0.0
0.4
0.8
Time in days
0
2000
4000
6000

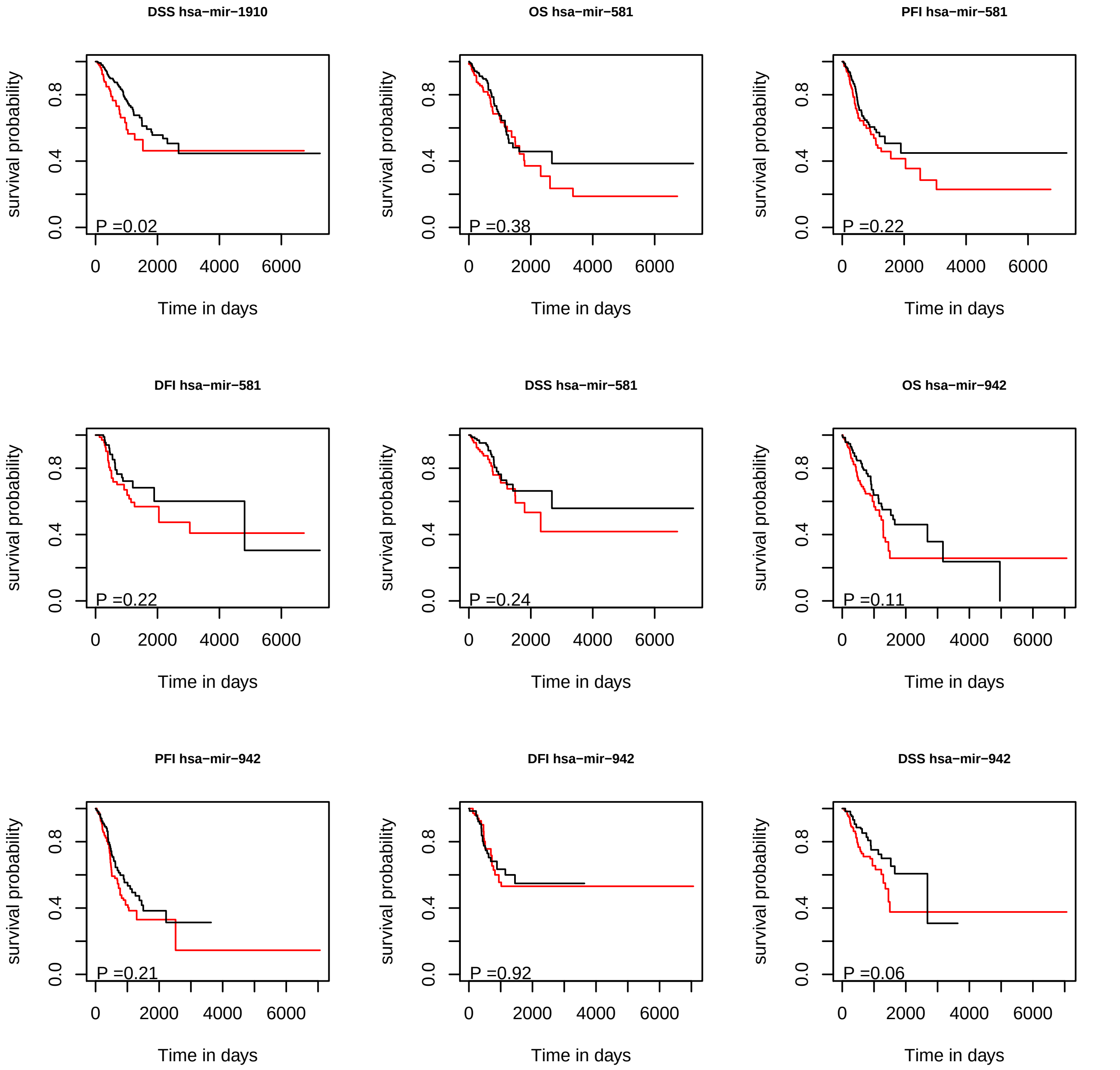
DSS hsa−mir−1910
P =0.02
OS hsa−mir−581
P =0.38
PFI hsa−mir−581
P =0.22
DFI hsa−mir−581
P =0.22
DSS hsa−mir−581
P =0.24
OS hsa−mir−942
P =0.11
PFI hsa−mir−942
P =0.21
DFI hsa−mir−942
P =0.92
DSS hsa−mir−942
P =0.06
survival probability
0.0
0.4
0.8
Time in days
0
2000
4000
6000

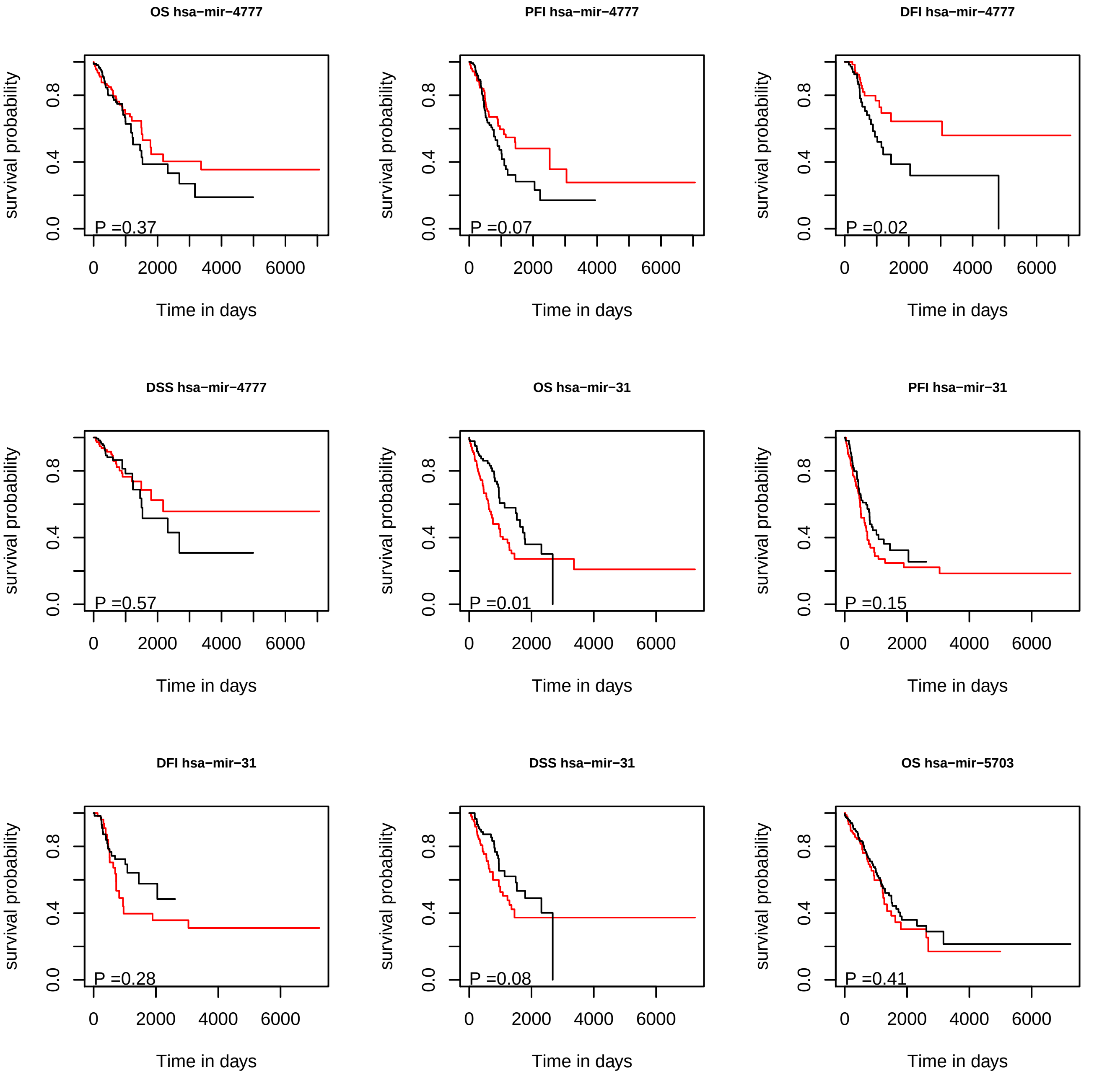
OS hsa−mir−4777
P =0.37
PFI hsa−mir−4777
P =0.07
DFI hsa−mir−4777
P =0.02
DSS hsa−mir−4777
P =0.57
OS hsa−mir−31
P =0.01
PFI hsa−mir−31
P =0.15
DFI hsa−mir−31
P =0.28
DSS hsa−mir−31
P =0.08
OS hsa−mir−5703
P =0.41
survival probability
0.0
0.4
0.8
Time in days
0
2000
4000
6000

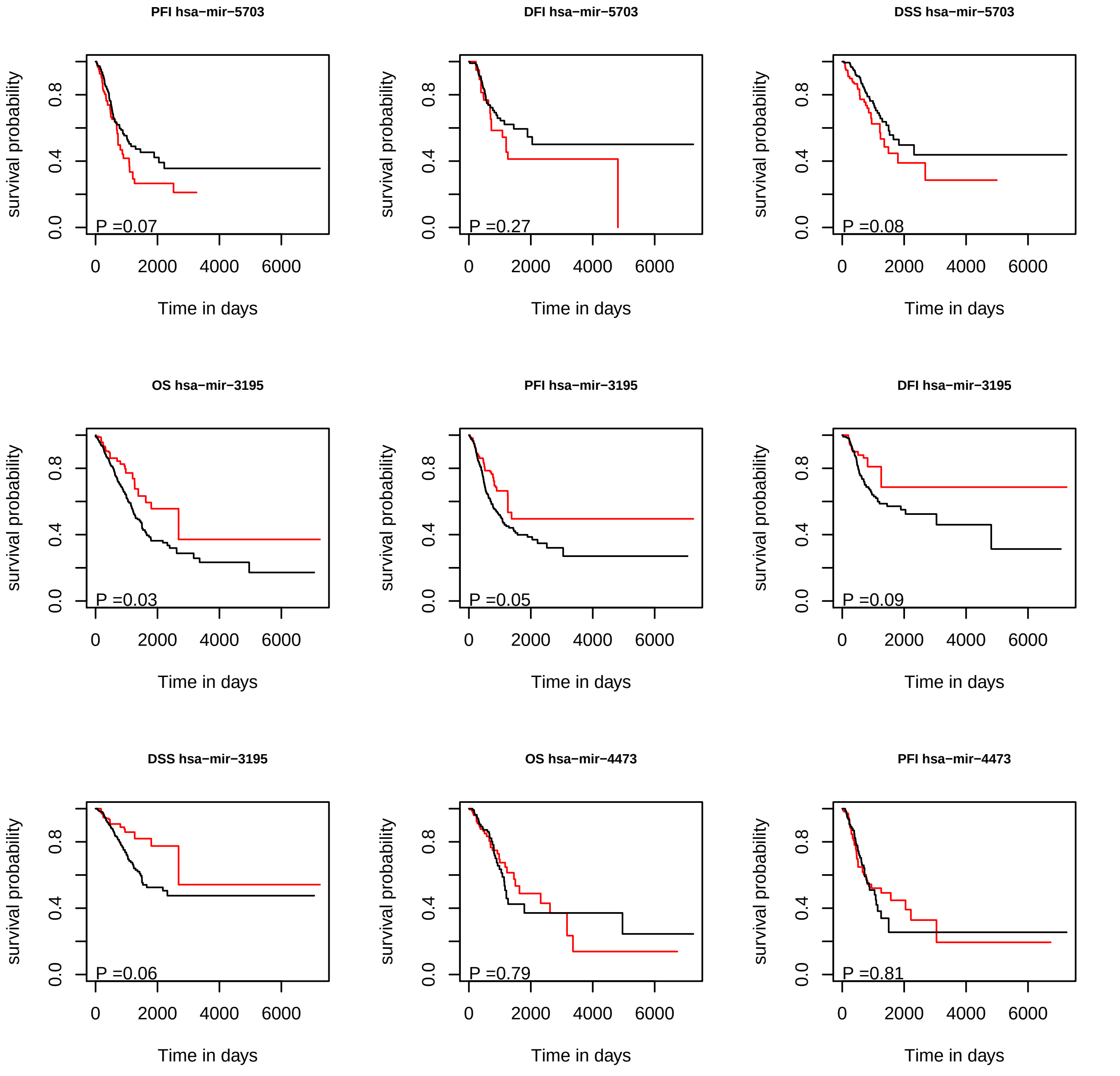
PFI hsa−mir−5703
P =0.07
DFI hsa−mir−5703
P =0.27
DSS hsa−mir−5703
P =0.08
OS hsa−mir−3195
P =0.03
PFI hsa−mir−3195
P =0.05
DFI hsa−mir−3195
P =0.09
DSS hsa−mir−3195
P =0.06
OS hsa−mir−4473
P =0.79
PFI hsa−mir−4473
P =0.81
survival probability
Time in days
0.0
0.4
0.8
0
2000
4000
6000

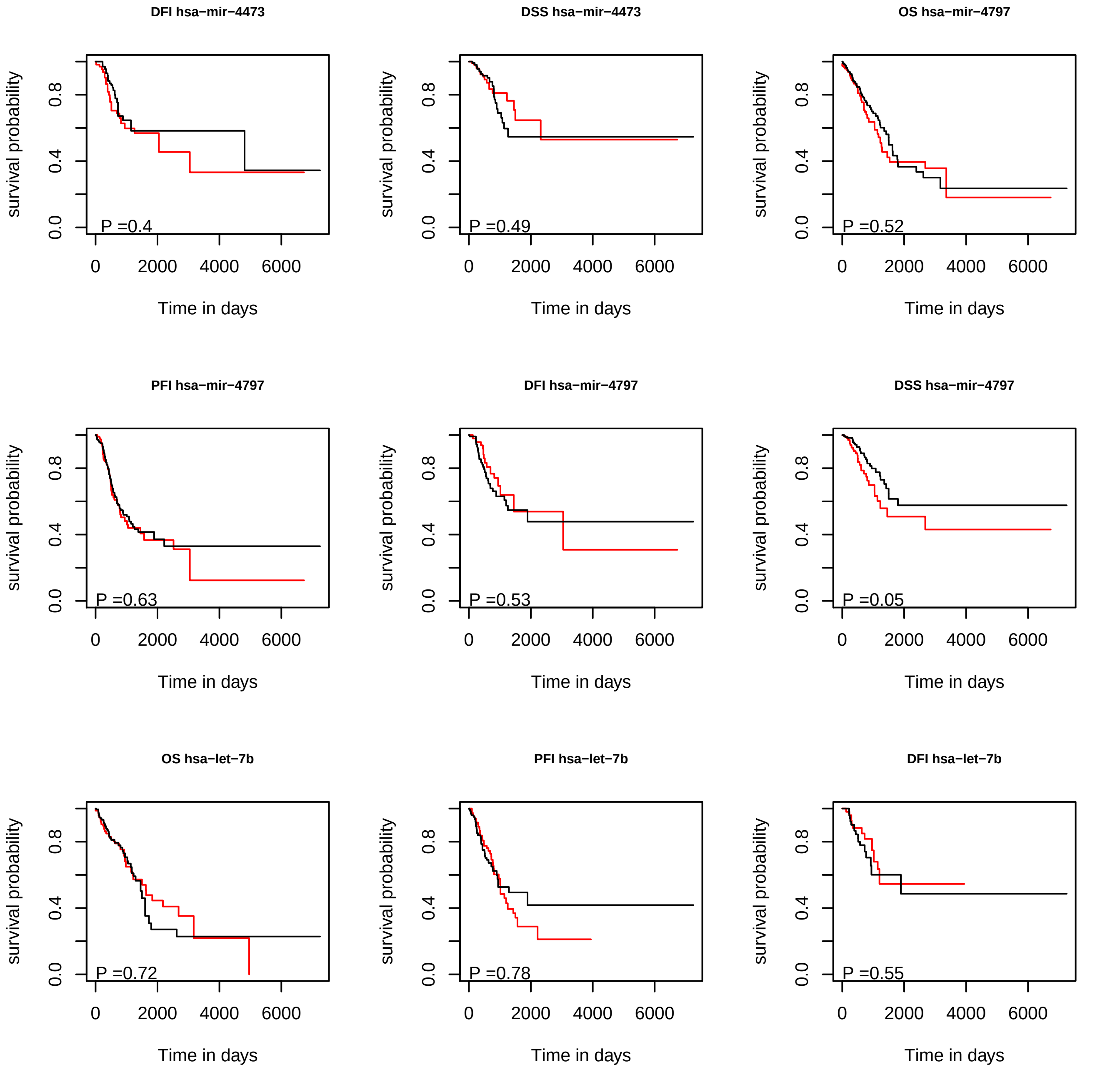
DFI hsa−mir−4473
survival probability
0.0
0.4
0.8
P =0.4
0
2000
4000
6000
Time in days
DSS hsa−mir−4473
survival probability
0.0
0.4
0.8
P =0.49
0
2000
4000
6000
Time in days
OS hsa−mir−4797
survival probability
0.0
0.4
0.8
P =0.52
0
2000
4000
6000
Time in days
PFI hsa−mir−4797
survival probability
0.0
0.4
0.8
P =0.63
0
2000
4000
6000
Time in days
DFI hsa−mir−4797
survival probability
0.0
0.4
0.8
P =0.53
0
2000
4000
6000
Time in days
DSS hsa−mir−4797
survival probability
0.0
0.4
0.8
P =0.05
0
2000
4000
6000
Time in days
OS hsa−let−7b
survival probability
0.0
0.4
0.8
P =0.72
0
2000
4000
6000
Time in days
PFI hsa−let−7b
survival probability
0.0
0.4
0.8
P =0.78
0
2000
4000
6000
Time in days
DFI hsa−let−7b
survival probability
0.0
0.4
0.8
P =0.55
0
2000
4000
6000
Time in days

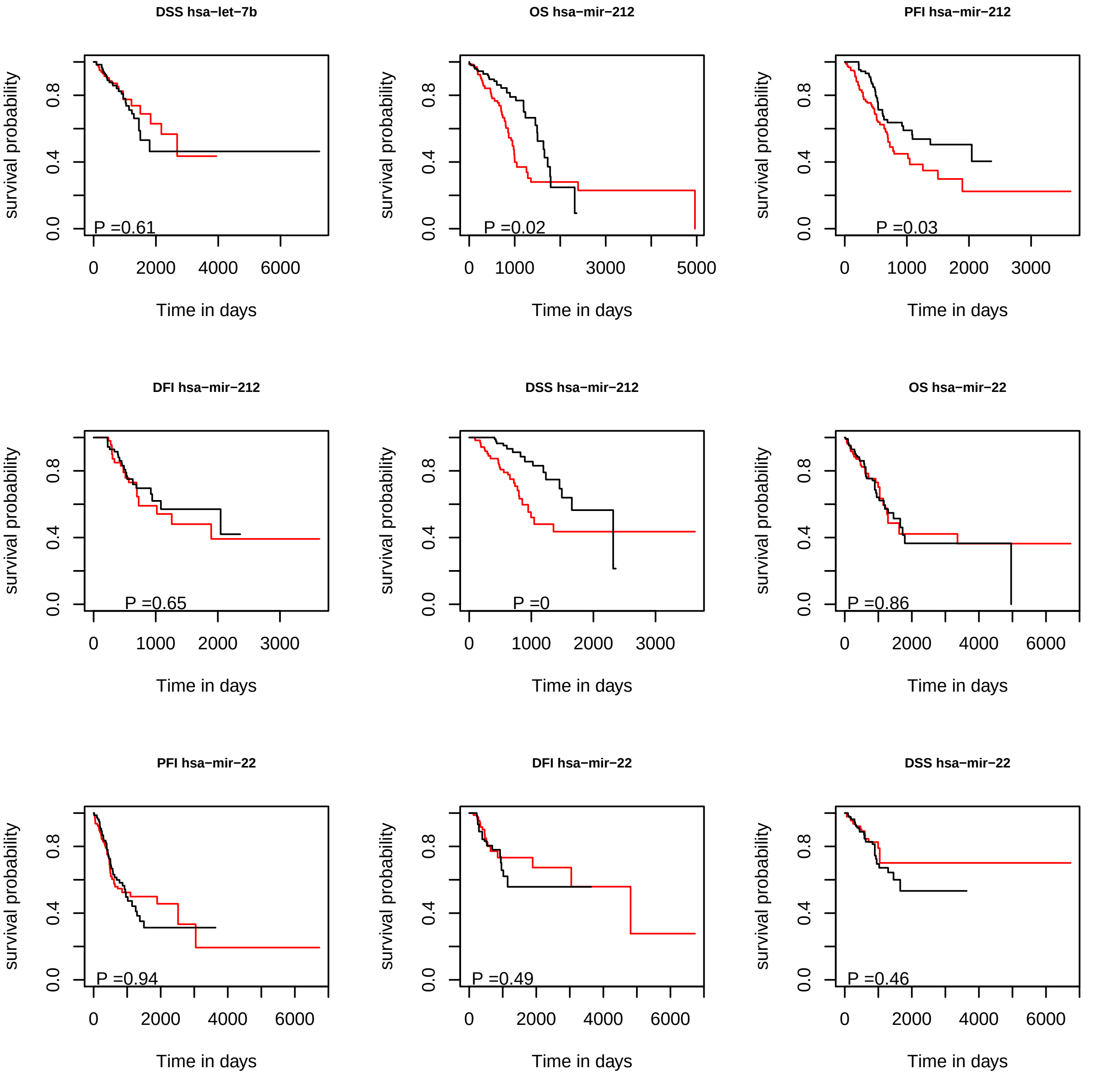
DSS hsa−let−7b
P =0.61
OS hsa−mir−212
P =0.02
PFI hsa−mir−212
P =0.03
DFI hsa−mir−212
P =0.65
DSS hsa−mir−212
P =0
OS hsa−mir−22
P =0.86
PFI hsa−mir−22
P =0.94
DFI hsa−mir−22
P =0.49
DSS hsa−mir−22
P =0.46
survival probability
Time in days
0.0
0.4
0.8
0
1000
2000
3000
4000
5000
6000

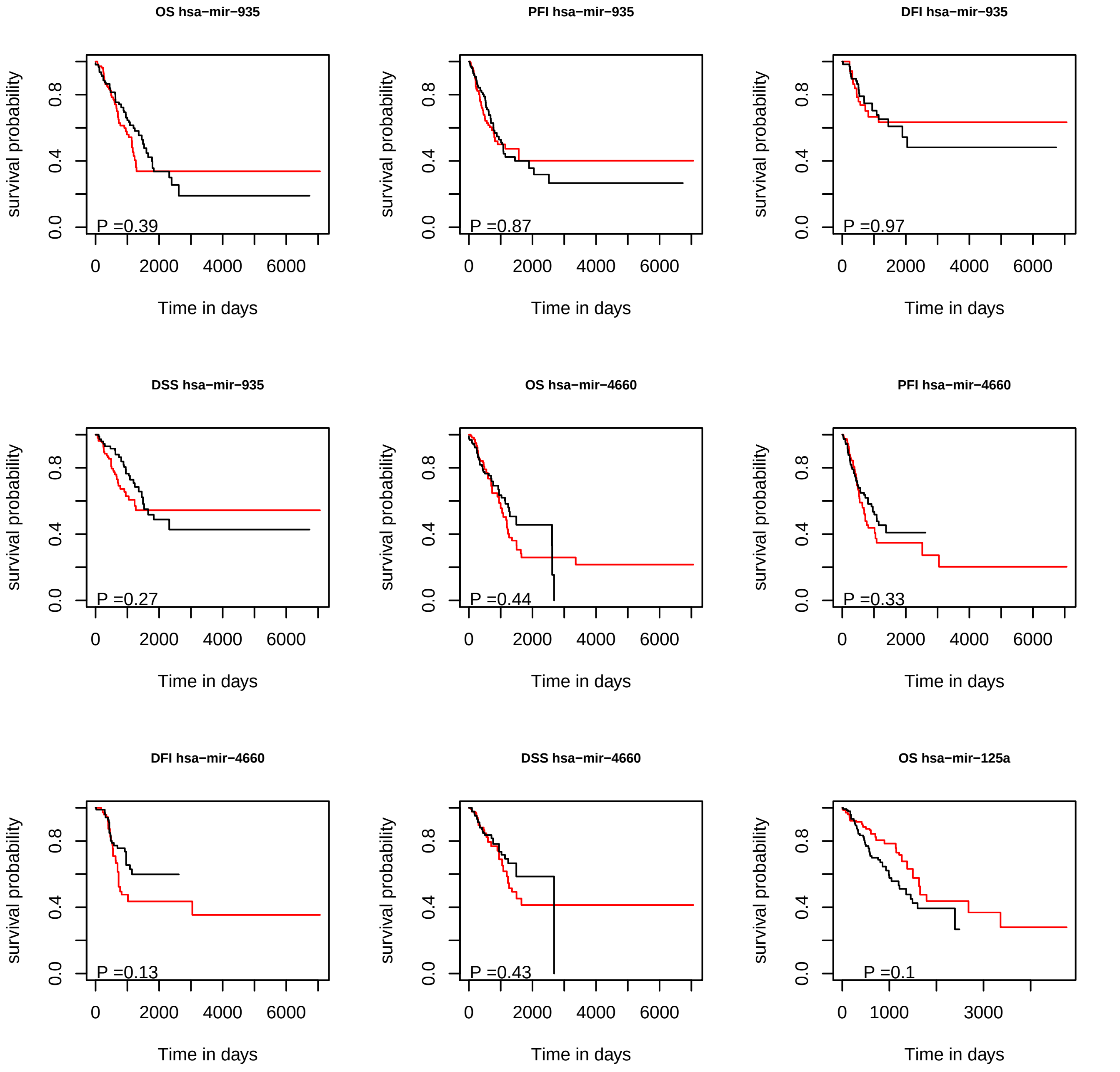
OS hsa−mir−935
P =0.39
PFI hsa−mir−935
P =0.87
DFI hsa−mir−935
P =0.97
DSS hsa−mir−935
P =0.27
OS hsa−mir−4660
P =0.44
PFI hsa−mir−4660
P =0.33
DFI hsa−mir−4660
P =0.13
DSS hsa−mir−4660
P =0.43
OS hsa−mir−125a
P =0.1
survival probability
0.0
0.4
0.8
0
1000
2000
3000
4000
6000
Time in days

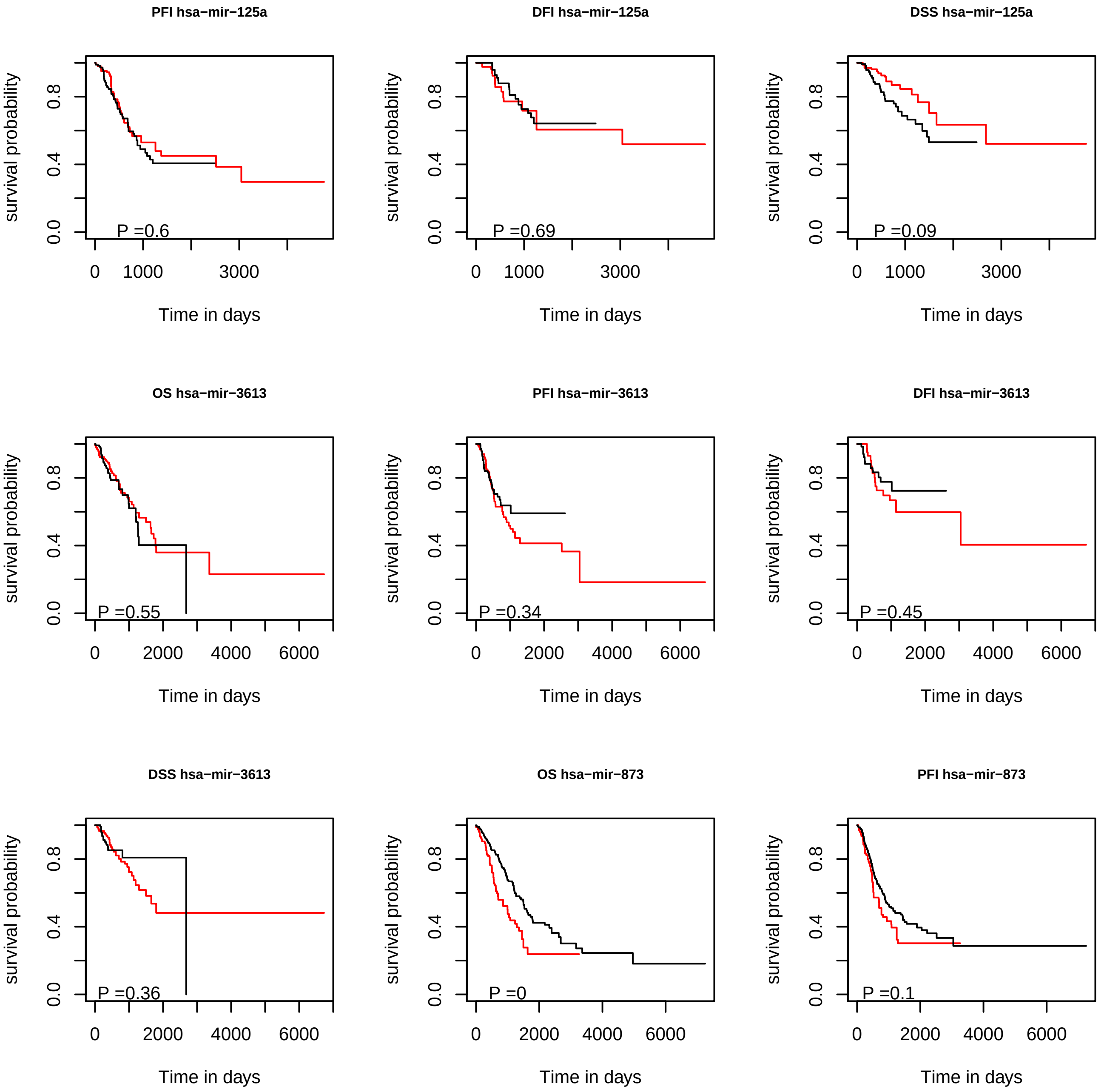
PFI hsa−mir−125a
P =0.6
DFI hsa−mir−125a
P =0.69
DSS hsa−mir−125a
P =0.09
OS hsa−mir−3613
P =0.55
PFI hsa−mir−3613
P =0.34
DFI hsa−mir−3613
P =0.45
DSS hsa−mir−3613
P =0.36
OS hsa−mir−873
P =0
PFI hsa−mir−873
P =0.1
survival probability
Time in days
0.0
0.4
0.8
0
1000
2000
3000
4000
6000

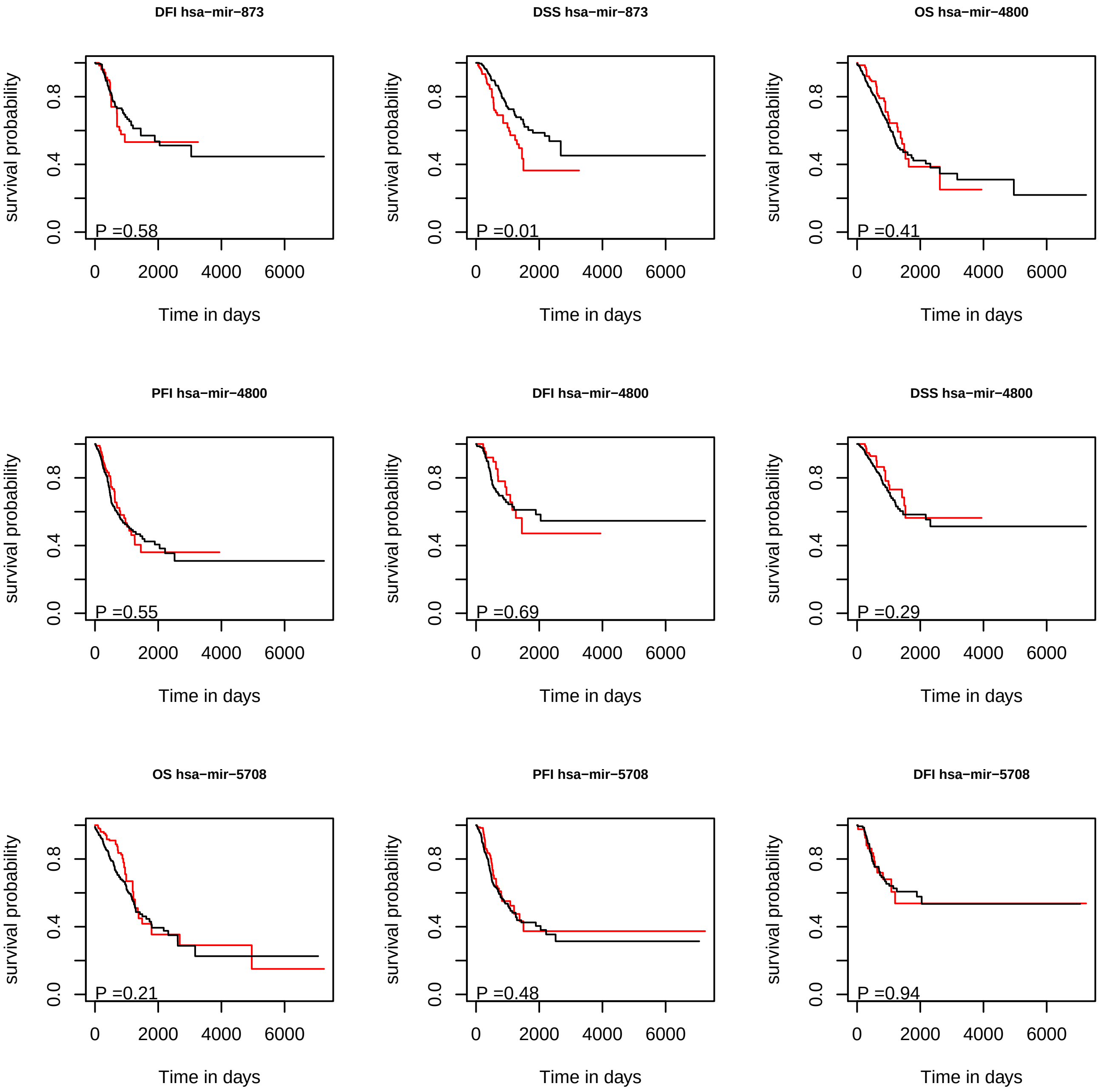
DFI hsa−mir−873
P =0.58
DSS hsa−mir−873
P =0.01
OS hsa−mir−4800
P =0.41
PFI hsa−mir−4800
P =0.55
DFI hsa−mir−4800
P =0.69
DSS hsa−mir−4800
P =0.29
OS hsa−mir−5708
P =0.21
PFI hsa−mir−5708
P =0.48
DFI hsa−mir−5708
P =0.94
survival probability
0.0
0.4
0.8
0
2000
4000
6000
Time in days

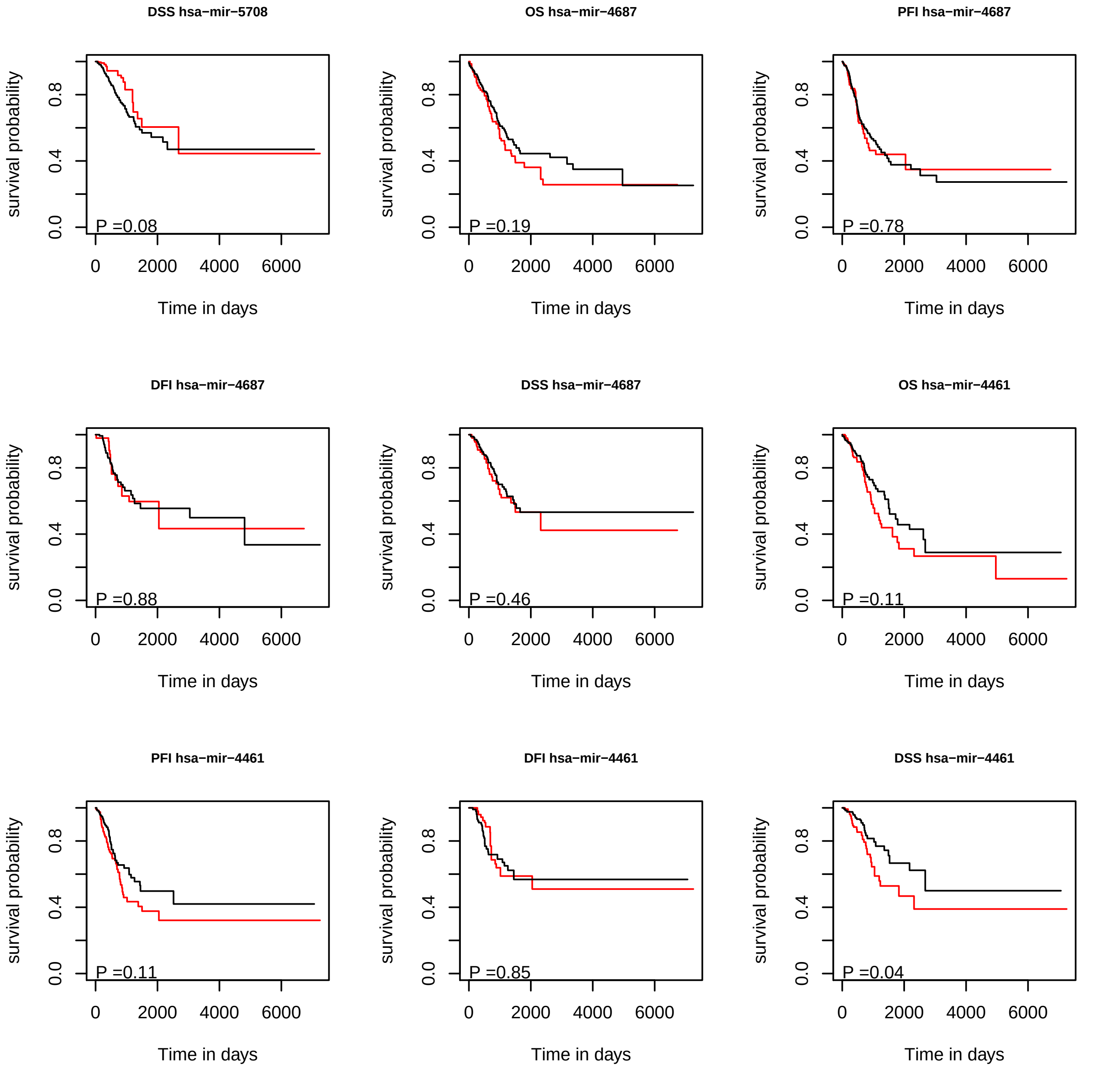
DSS hsa−mir−5708
P =0.08
OS hsa−mir−4687
P =0.19
PFI hsa−mir−4687
P =0.78
DFI hsa−mir−4687
P =0.88
DSS hsa−mir−4687
P =0.46
OS hsa−mir−4461
P =0.11
PFI hsa−mir−4461
P =0.11
DFI hsa−mir−4461
P =0.85
DSS hsa−mir−4461
P =0.04
survival probability
0.0
0.4
0.8
Time in days
0
2000
4000
6000

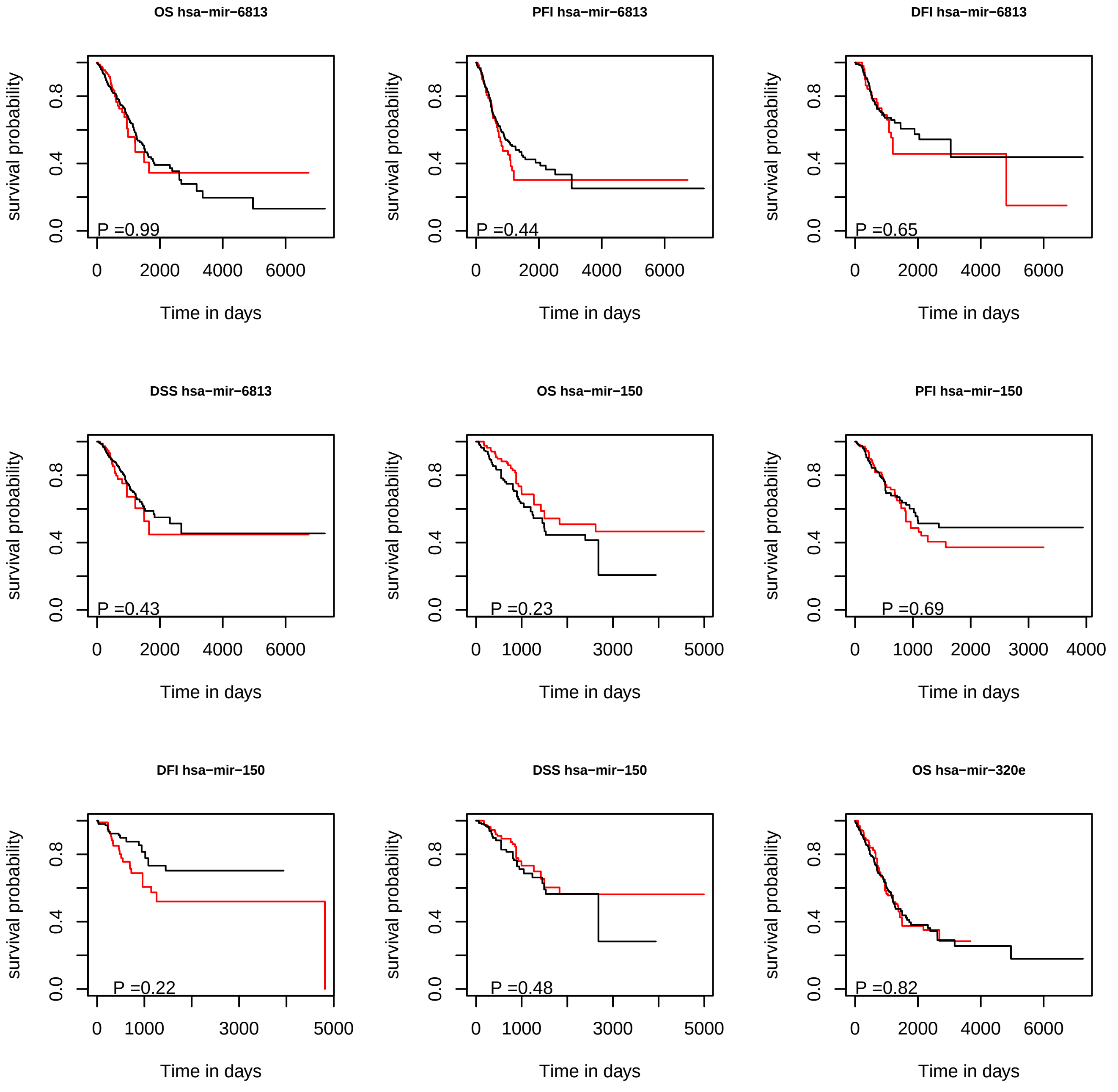
OS hsa−mir−6813
P =0.99
PFI hsa−mir−6813
P =0.44
DFI hsa−mir−6813
P =0.65
DSS hsa−mir−6813
P =0.43
OS hsa−mir−150
P =0.23
PFI hsa−mir−150
P =0.69
DFI hsa−mir−150
P =0.22
DSS hsa−mir−150
P =0.48
OS hsa−mir−320e
P =0.82
survival probability
Time in days
0.0
0.4
0.8
0
1000
2000
3000
4000
5000
6000

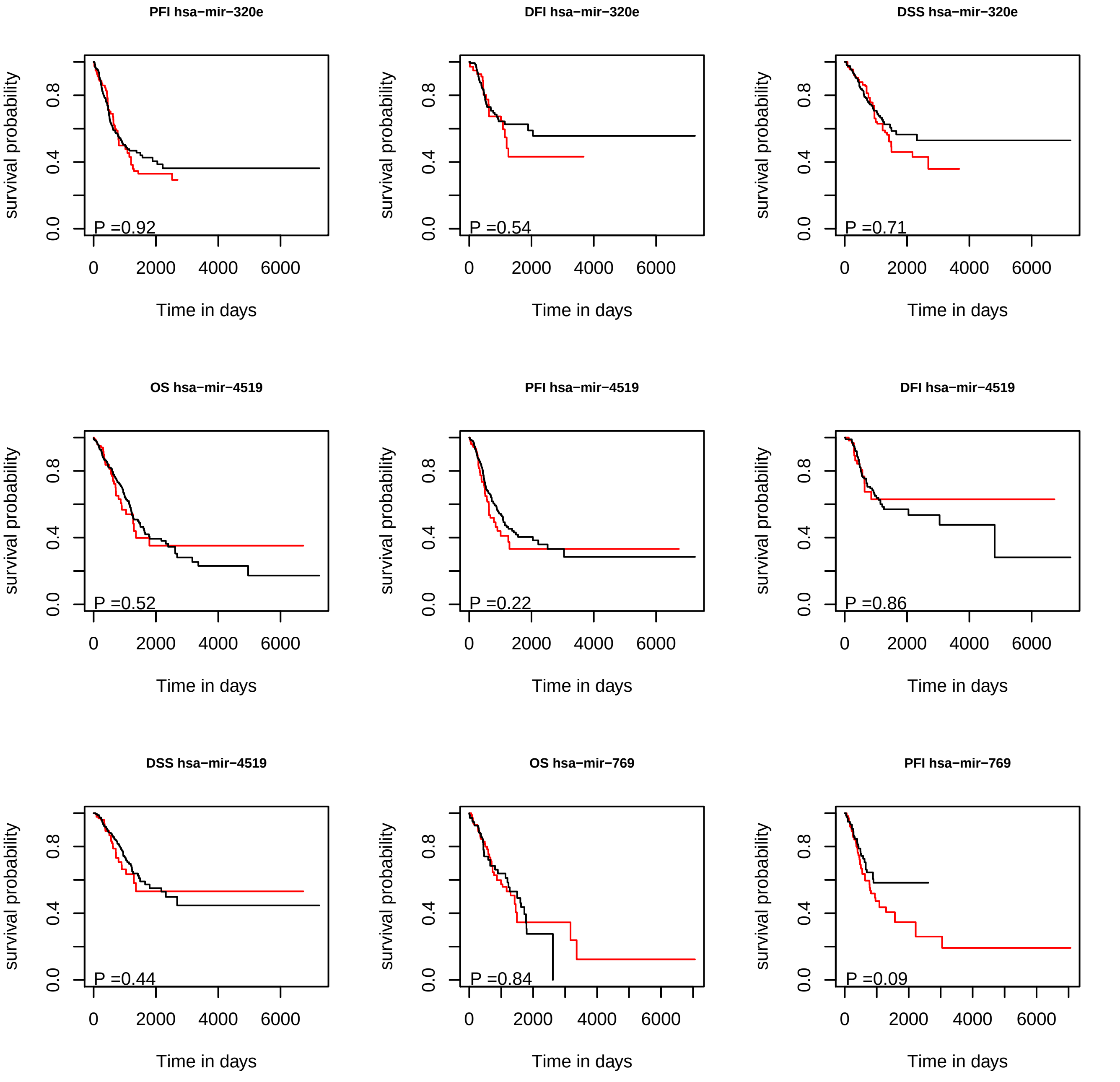
PFI hsa−mir−320e
P =0.92
DFI hsa−mir−320e
P =0.54
DSS hsa−mir−320e
P =0.71
OS hsa−mir−4519
P =0.52
PFI hsa−mir−4519
P =0.22
DFI hsa−mir−4519
P =0.86
DSS hsa−mir−4519
P =0.44
OS hsa−mir−769
P =0.84
PFI hsa−mir−769
P =0.09
survival probability
0.0
0.4
0.8
Time in days
0
2000
4000
6000

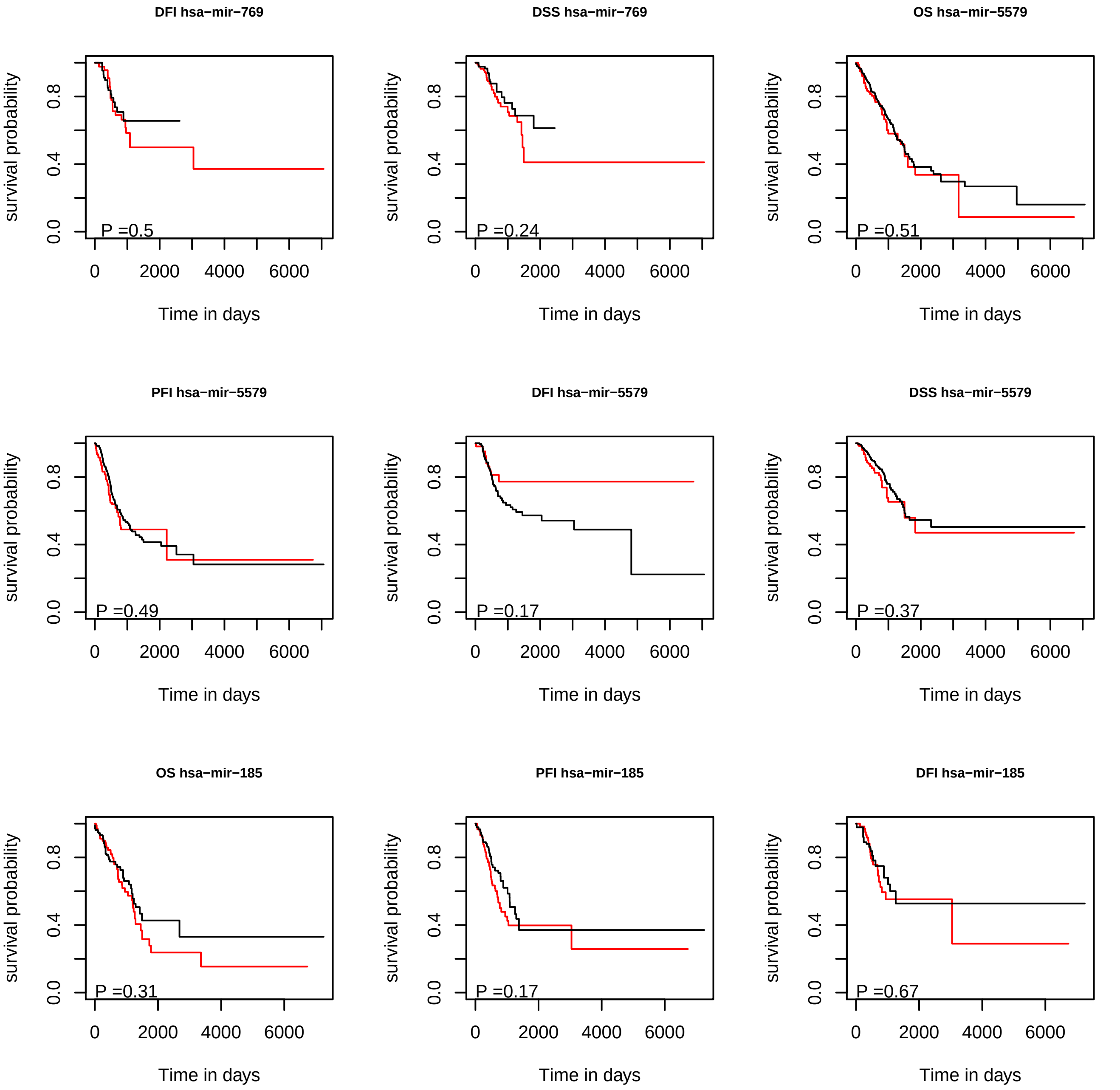
DFI hsa−mir−769
P =0.5
DSS hsa−mir−769
P =0.24
OS hsa−mir−5579
P =0.51
PFI hsa−mir−5579
P =0.49
DFI hsa−mir−5579
P =0.17
DSS hsa−mir−5579
P =0.37
OS hsa−mir−185
P =0.31
PFI hsa−mir−185
P =0.17
DFI hsa−mir−185
P =0.67
survival probability
0.0
0.4
0.8
Time in days
0
2000
4000
6000

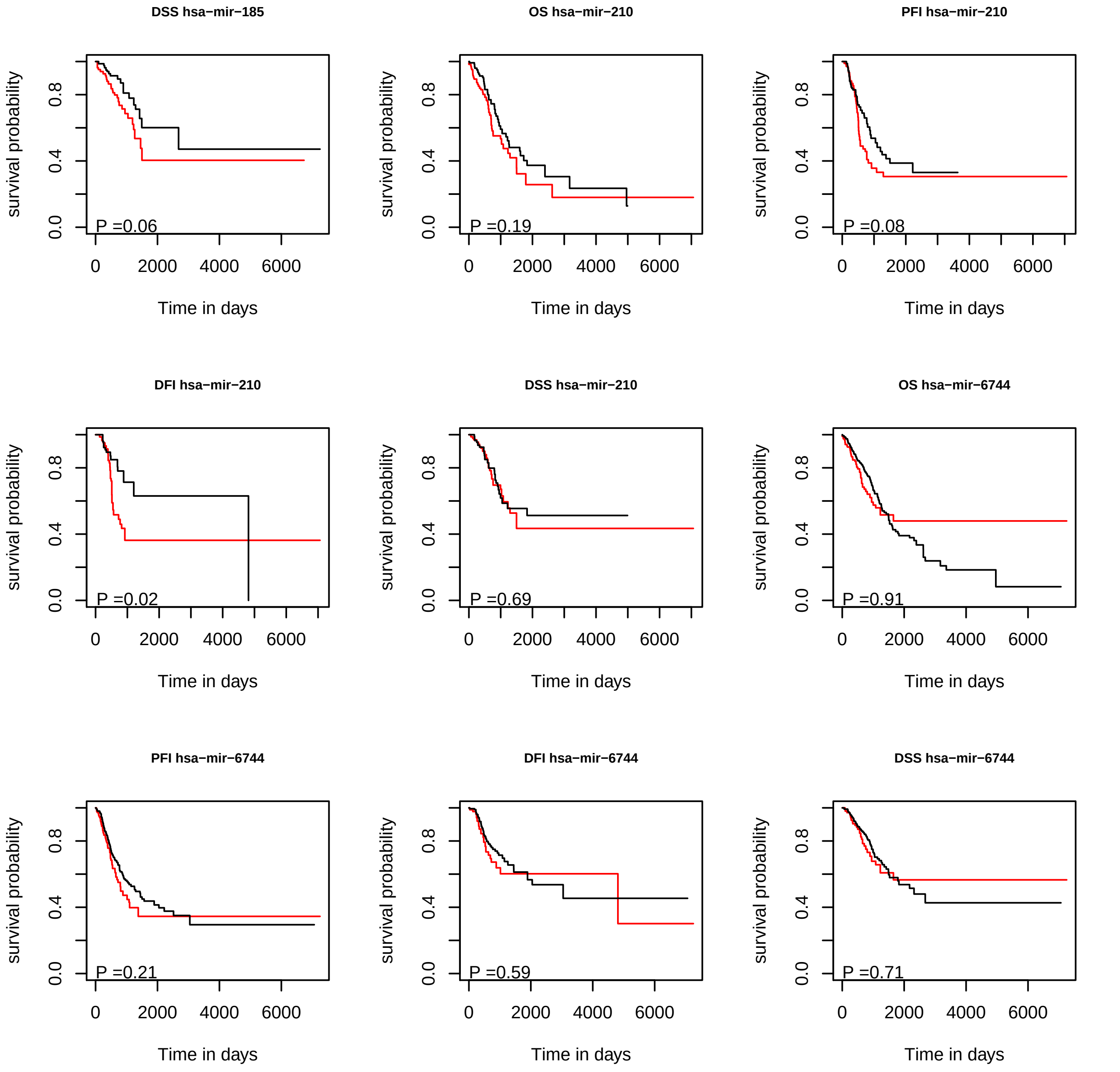
DSS hsa−mir−185
P =0.06
OS hsa−mir−210
P =0.19
PFI hsa−mir−210
P =0.08
DFI hsa−mir−210
P =0.02
DSS hsa−mir−210
P =0.69
OS hsa−mir−6744
P =0.91
PFI hsa−mir−6744
P =0.21
DFI hsa−mir−6744
P =0.59
DSS hsa−mir−6744
P =0.71
survival probability
0.0
0.4
0.8
0
2000
4000
6000
Time in days

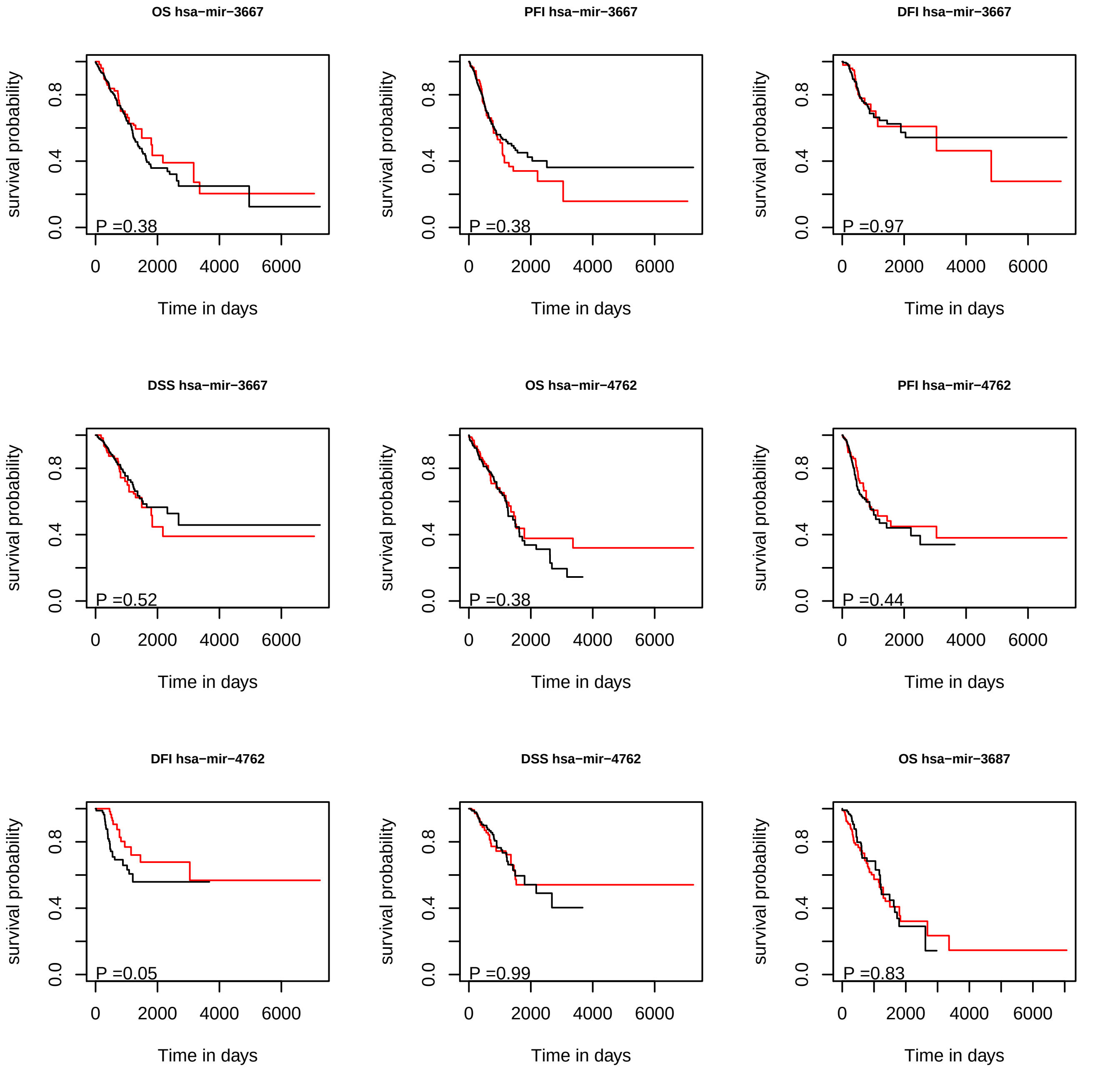

OS hsa−mir−3667
P =0.38
PFI hsa−mir−3667
P =0.38
DFI hsa−mir−3667
P =0.97
DSS hsa−mir−3667
P =0.52
OS hsa−mir−4762
P =0.38
PFI hsa−mir−4762
P =0.44
DFI hsa−mir−4762
P =0.05
DSS hsa−mir−4762
P =0.99
OS hsa−mir−3687
P =0.83
survival probability
0.0
0.4
0.8
0
2000
4000
6000
Time in days

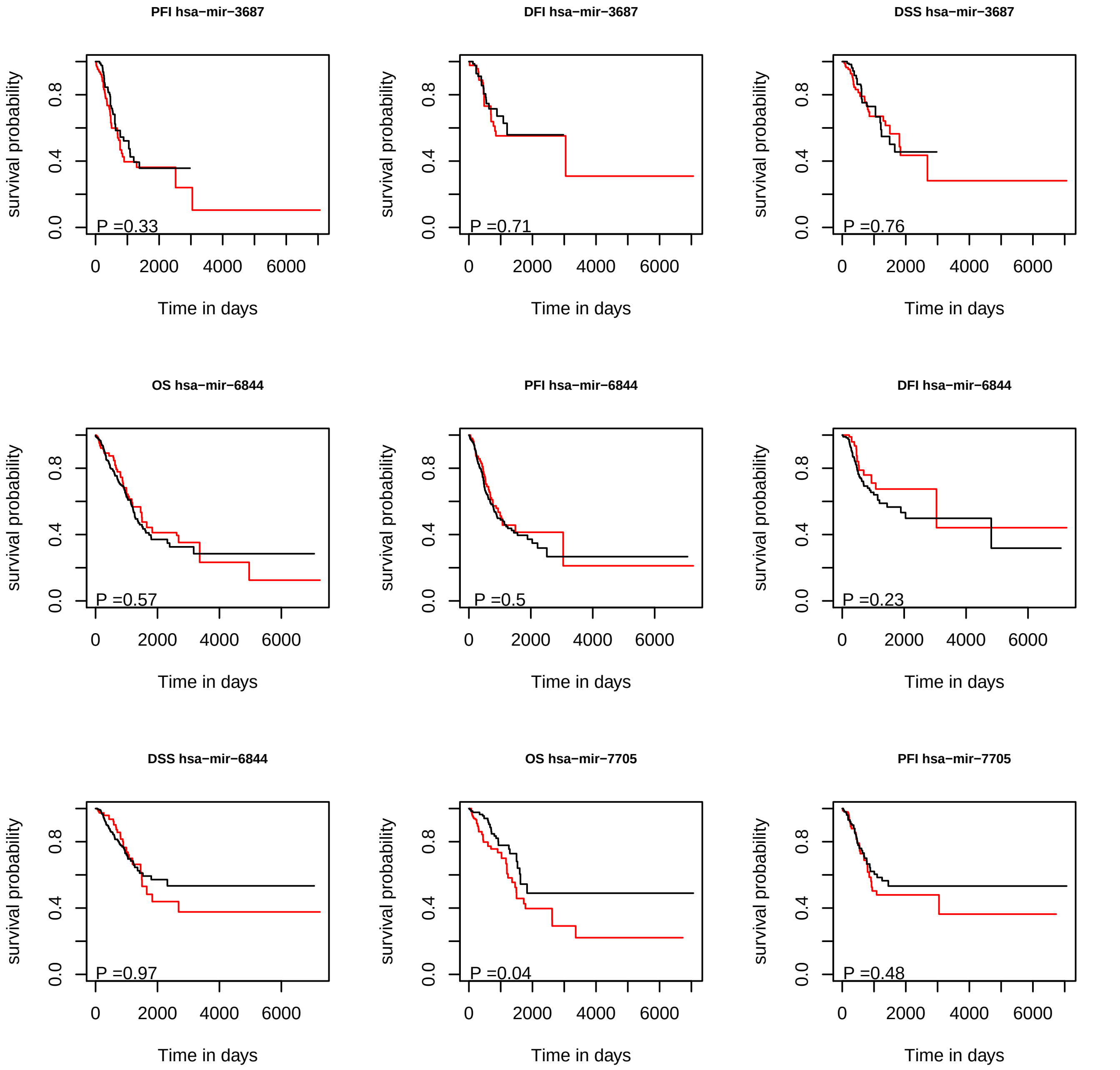
PFI hsa−mir−3687
P =0.33
DFI hsa−mir−3687
P =0.71
DSS hsa−mir−3687
P =0.76
OS hsa−mir−6844
P =0.57
PFI hsa−mir−6844
P =0.5
DFI hsa−mir−6844
P =0.23
DSS hsa−mir−6844
P =0.97
OS hsa−mir−7705
P =0.04
PFI hsa−mir−7705
P =0.48
survival probability
0.0
0.4
0.8
Time in days
0
2000
4000
6000

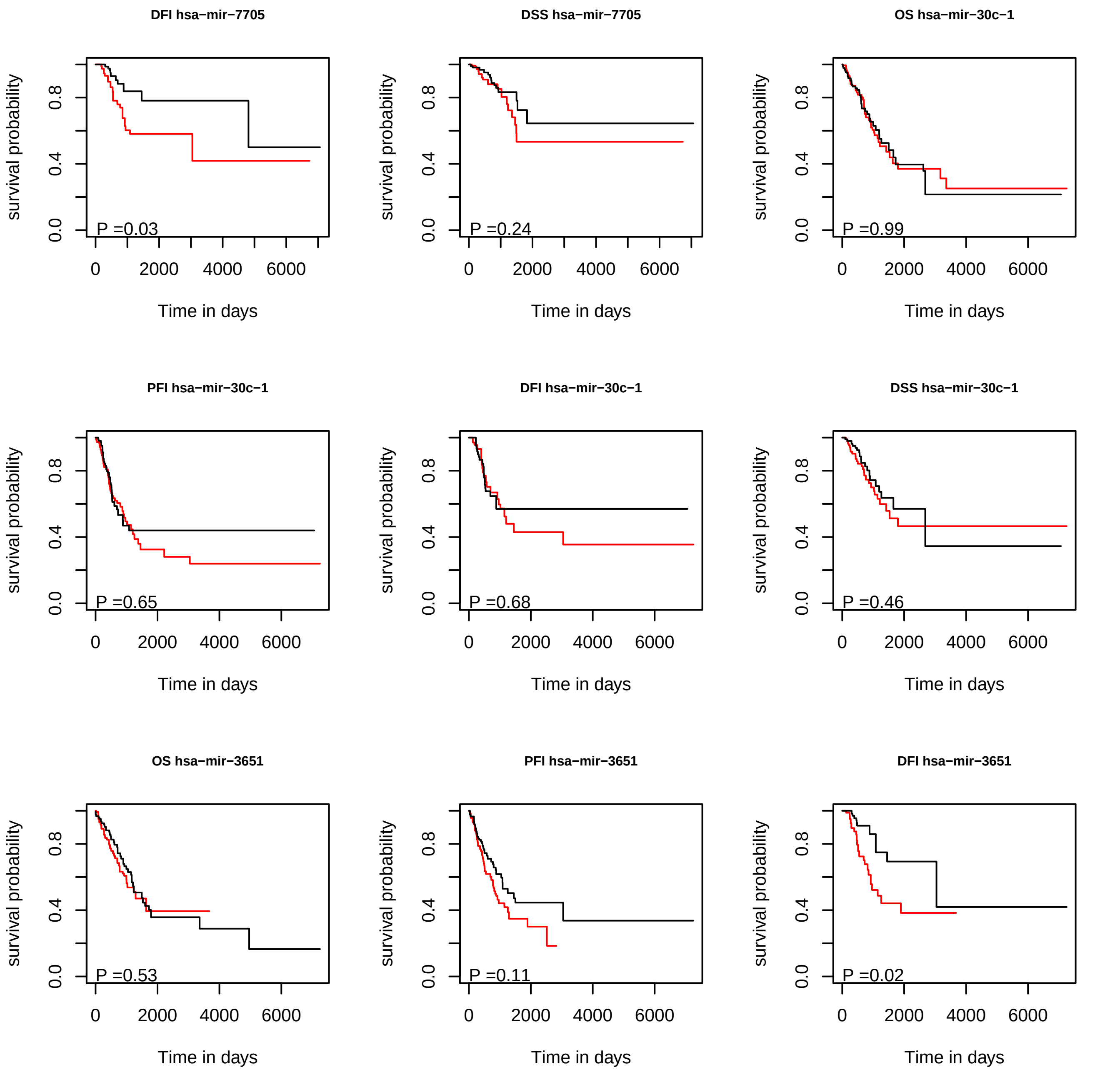
DFI hsa−mir−7705
P =0.03
DSS hsa−mir−7705
P =0.24
OS hsa−mir−30c−1
P =0.99
PFI hsa−mir−30c−1
P =0.65
DFI hsa−mir−30c−1
P =0.68
DSS hsa−mir−30c−1
P =0.46
OS hsa−mir−3651
P =0.53
PFI hsa−mir−3651
P =0.11
DFI hsa−mir−3651
P =0.02
survival probability
Time in days
0.0
0.4
0.8
0
2000
4000
6000

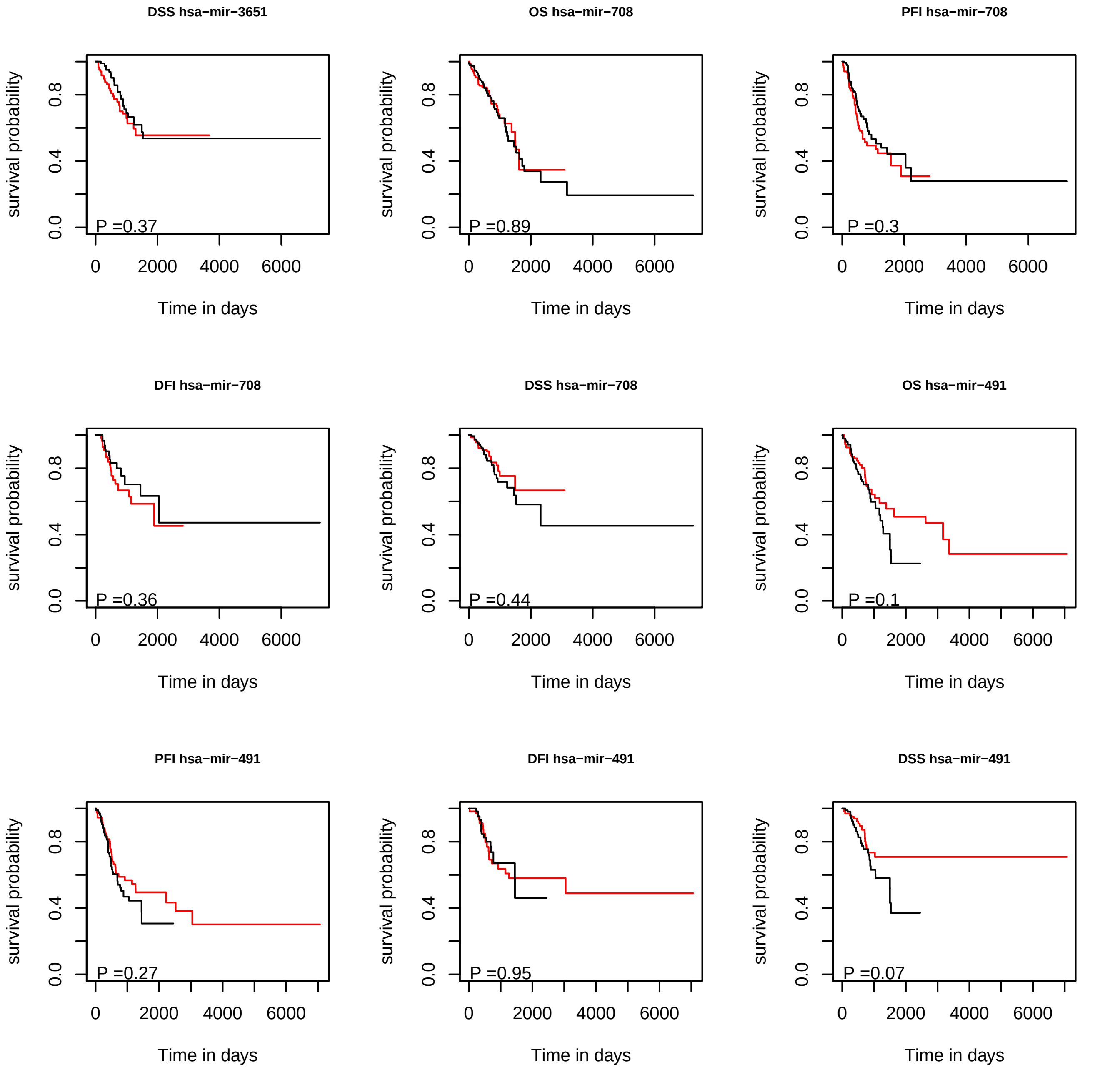

DSS hsa−mir−3651
P =0.37
OS hsa−mir−708
P =0.89
PFI hsa−mir−708
P =0.3
DFI hsa−mir−708
P =0.36
DSS hsa−mir−708
P =0.44
OS hsa−mir−491
P =0.1
PFI hsa−mir−491
P =0.27
DFI hsa−mir−491
P =0.95
DSS hsa−mir−491
P =0.07
survival probability
0.0
0.4
0.8
Time in days
0
2000
4000
6000

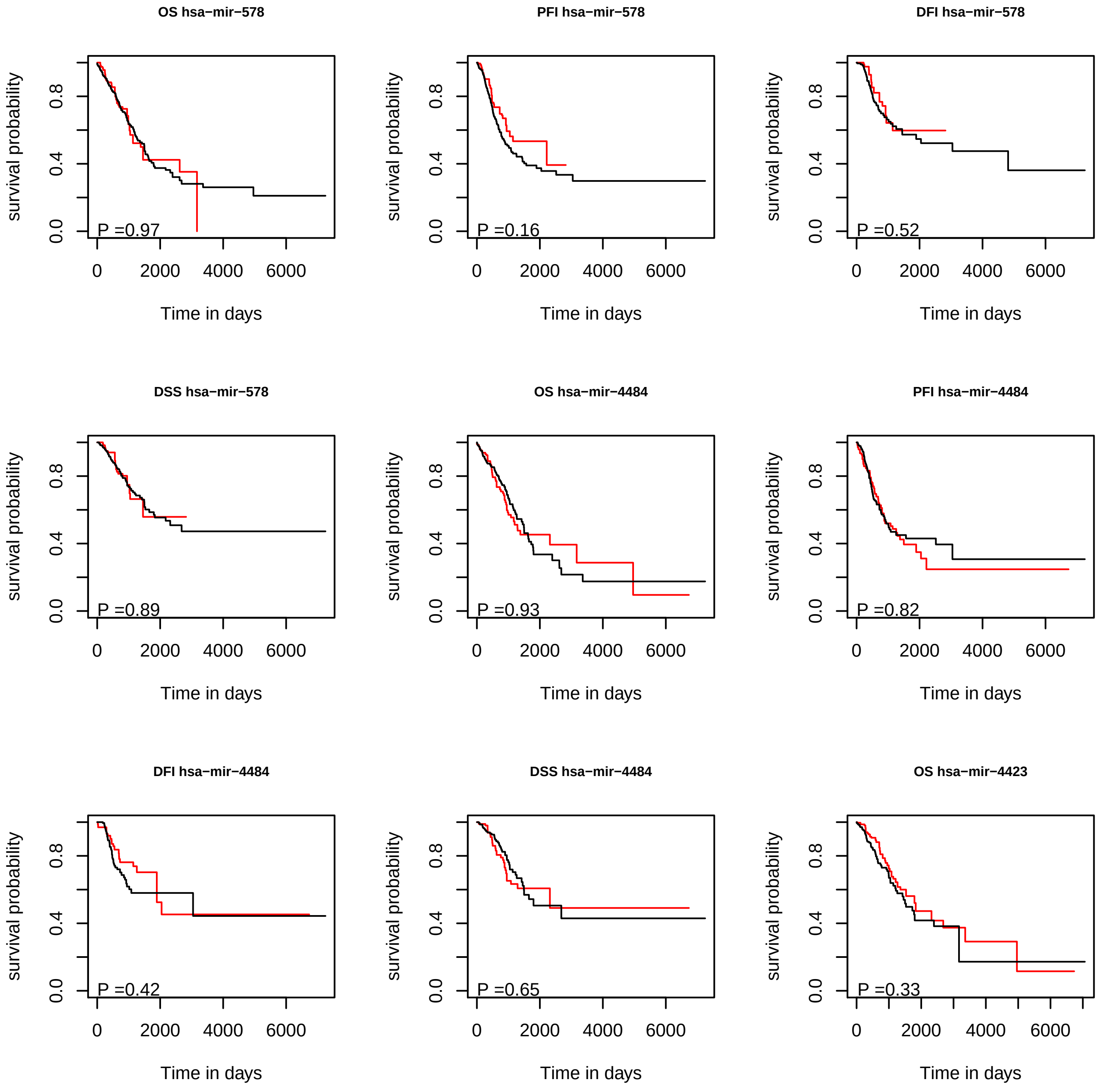
OS hsa−mir−578
P =0.97
PFI hsa−mir−578
P =0.16
DFI hsa−mir−578
P =0.52
DSS hsa−mir−578
P =0.89
OS hsa−mir−4484
P =0.93
PFI hsa−mir−4484
P =0.82
DFI hsa−mir−4484
P =0.42
DSS hsa−mir−4484
P =0.65
OS hsa−mir−4423
P =0.33
survival probability
0.0
0.4
0.8
Time in days
0
2000
4000
6000

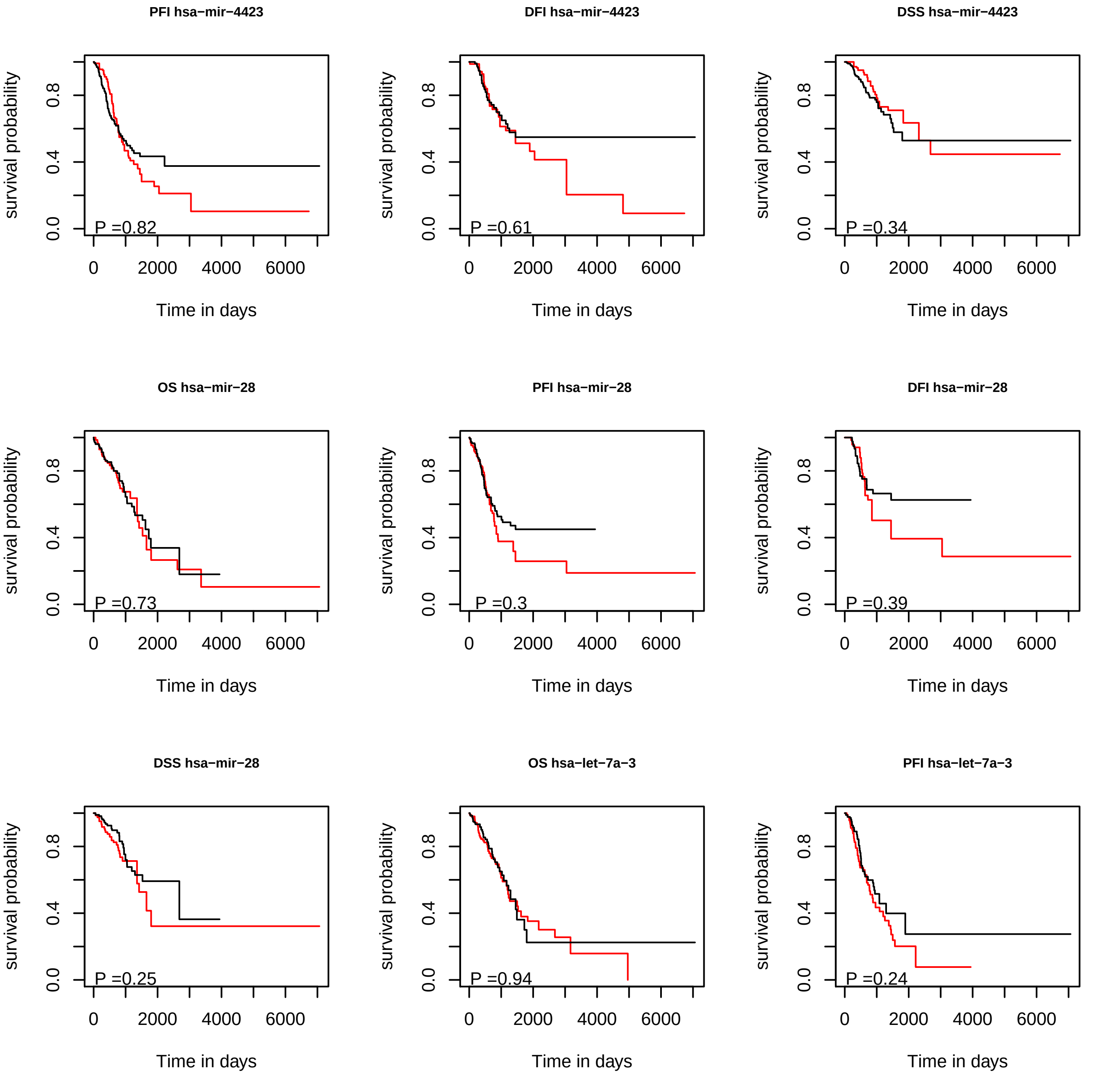
PFI hsa−mir−4423
DFI hsa−mir−4423
DSS hsa−mir−4423
OS hsa−mir−28
PFI hsa−mir−28
DFI hsa−mir−28
DSS hsa−mir−28
OS hsa−let−7a−3
PFI hsa−let−7a−3
survival probability
Time in days
0.0
0.4
0.8
0
2000
4000
6000
P =0.82
P =0.61
P =0.34
P =0.73
P =0.3
P =0.39
P =0.25
P =0.94
P =0.24

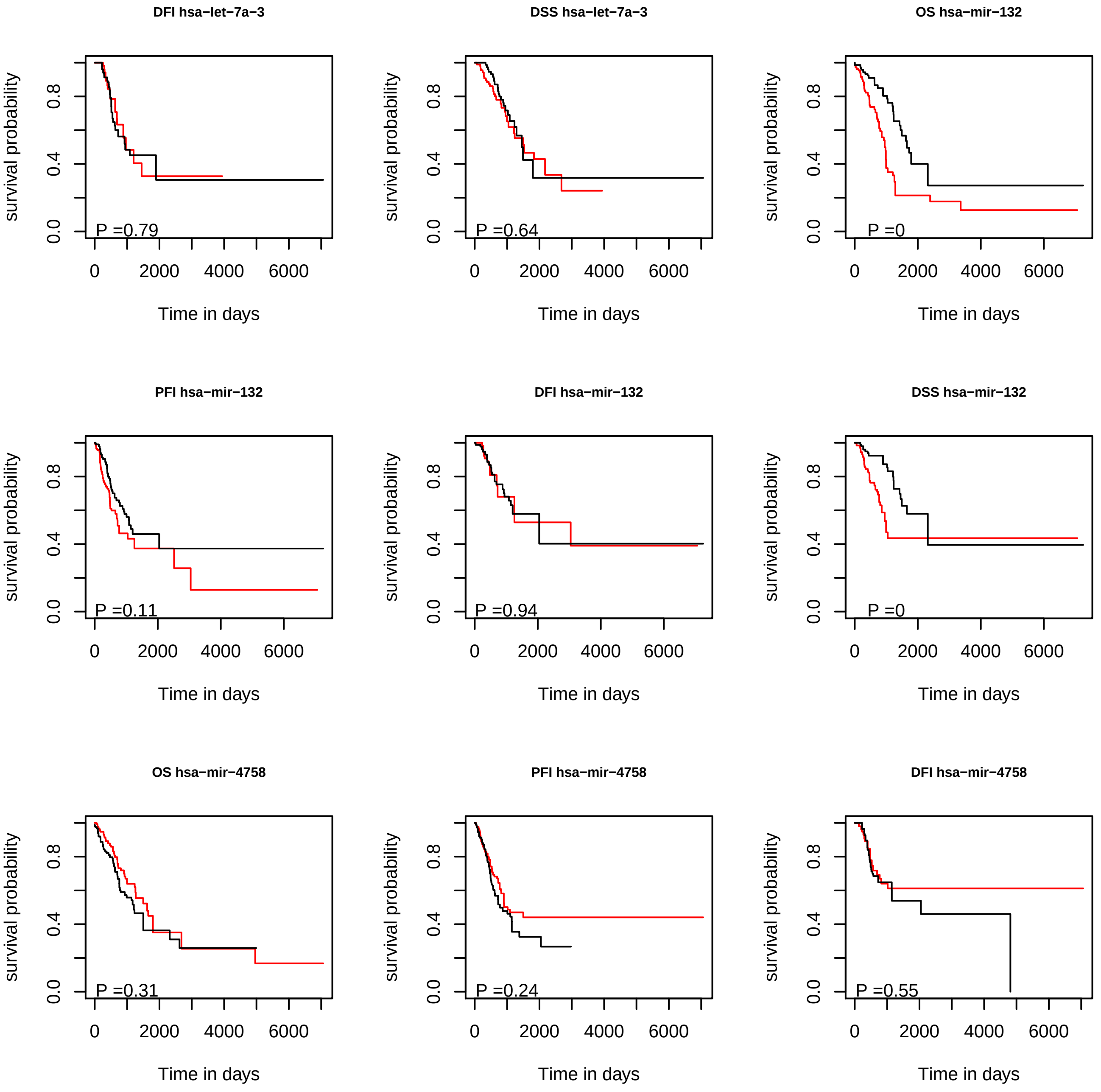
DFI hsa−let−7a−3
P =0.79
DSS hsa−let−7a−3
P =0.64
OS hsa−mir−132
P =0
PFI hsa−mir−132
P =0.11
DFI hsa−mir−132
P =0.94
DSS hsa−mir−132
P =0
OS hsa−mir−4758
P =0.31
PFI hsa−mir−4758
P =0.24
DFI hsa−mir−4758
P =0.55
survival probability
0.0
0.4
0.8
Time in days
0
2000
4000
6000

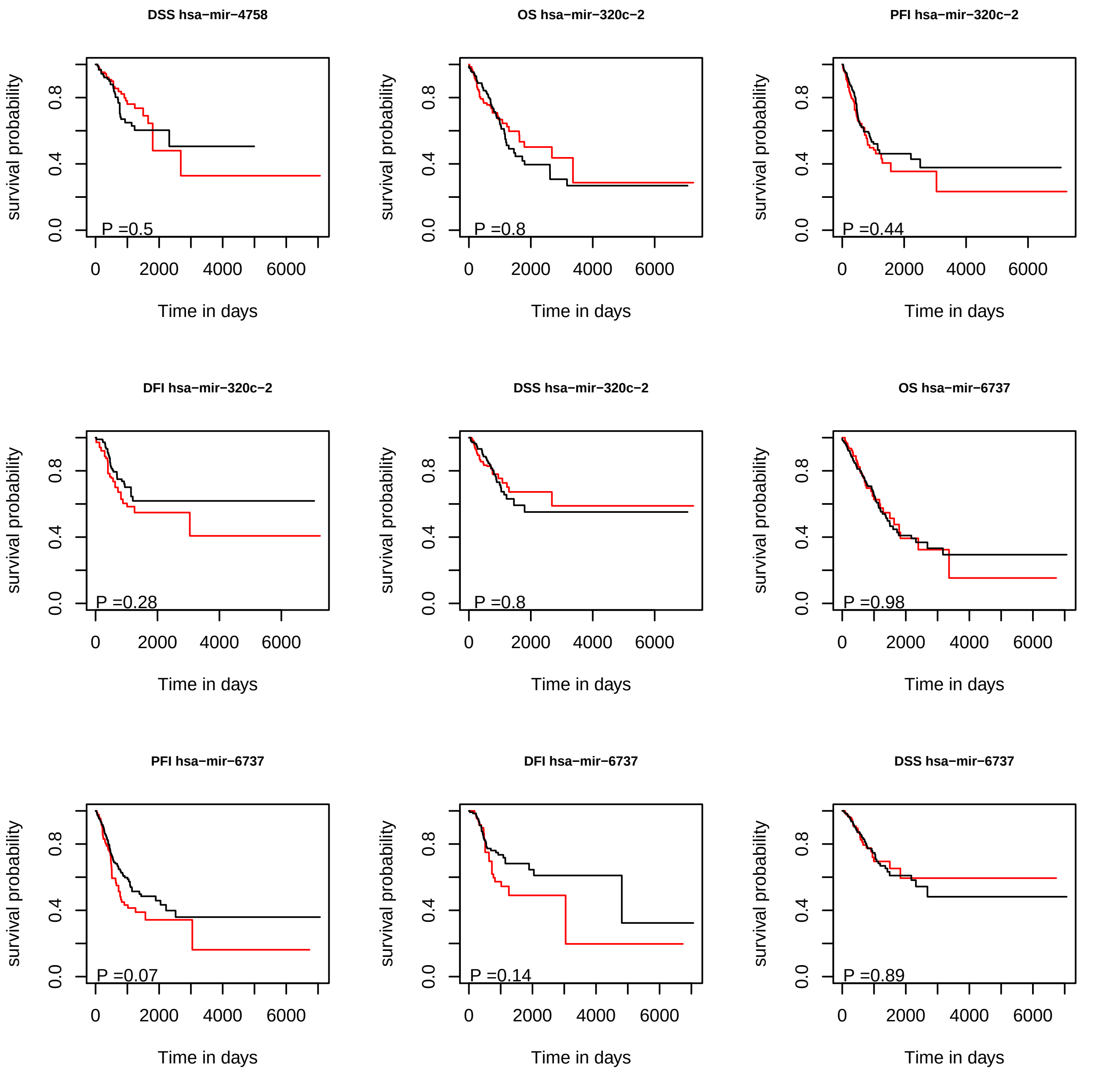
DSS hsa−mir−4758
survival probability
Time in days
P =0.5
OS hsa−mir−320c−2
survival probability
Time in days
P =0.8
PFI hsa−mir−320c−2
survival probability
Time in days
P =0.44
DFI hsa−mir−320c−2
survival probability
Time in days
P =0.28
DSS hsa−mir−320c−2
survival probability
Time in days
P =0.8
OS hsa−mir−6737
survival probability
Time in days
P =0.98
PFI hsa−mir−6737
survival probability
Time in days
P =0.07
DFI hsa−mir−6737
survival probability
Time in days
P =0.14
DSS hsa−mir−6737
survival probability
Time in days
P =0.89
0.0
0.4
0.8
0
2000
4000
6000

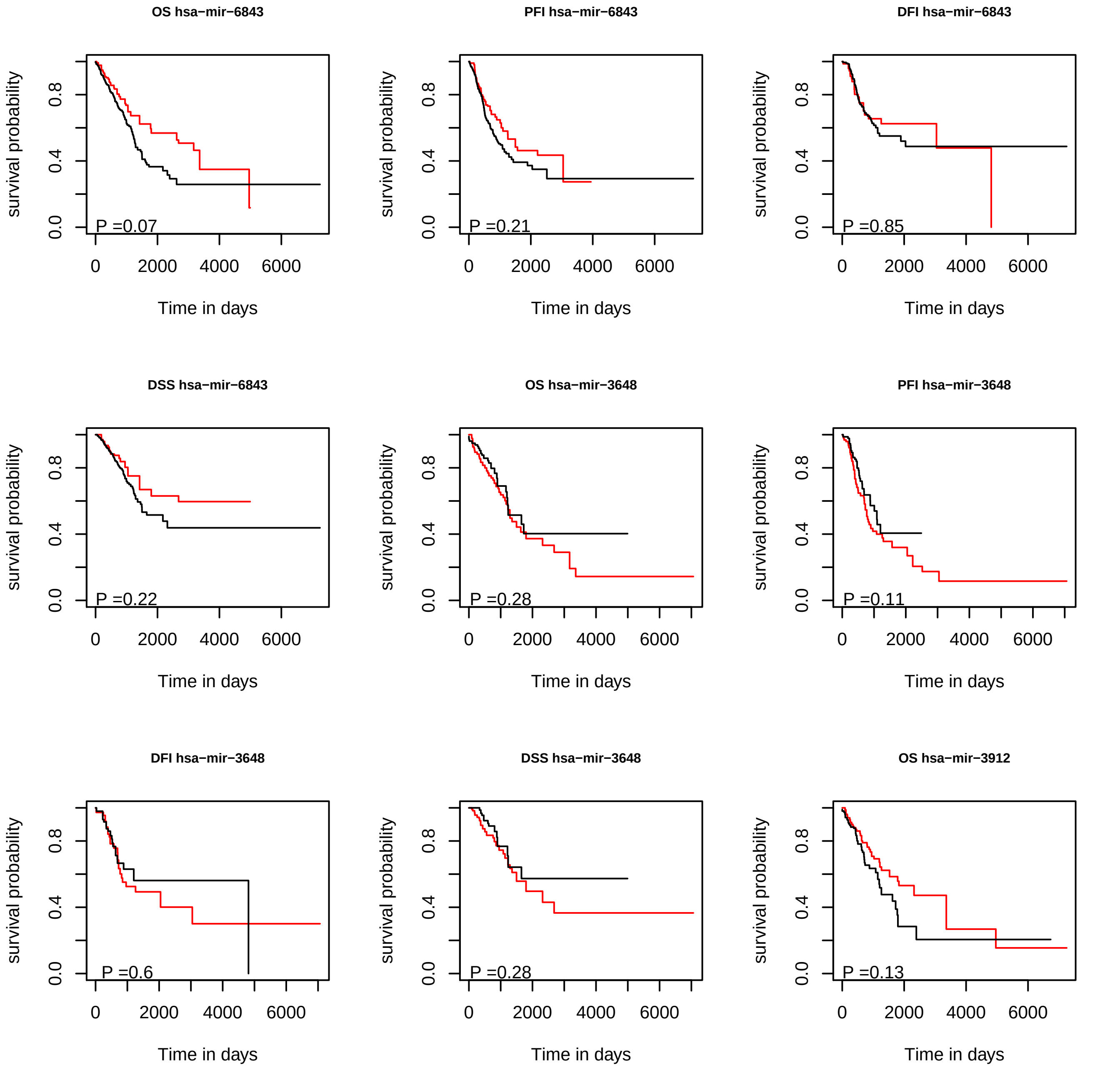
OS hsa−mir−6843
P =0.07
PFI hsa−mir−6843
P =0.21
DFI hsa−mir−6843
P =0.85
DSS hsa−mir−6843
P =0.22
OS hsa−mir−3648
P =0.28
PFI hsa−mir−3648
P =0.11
DFI hsa−mir−3648
P =0.6
DSS hsa−mir−3648
P =0.28
OS hsa−mir−3912
P =0.13
survival probability
0.0
0.4
0.8
Time in days
0
2000
4000
6000

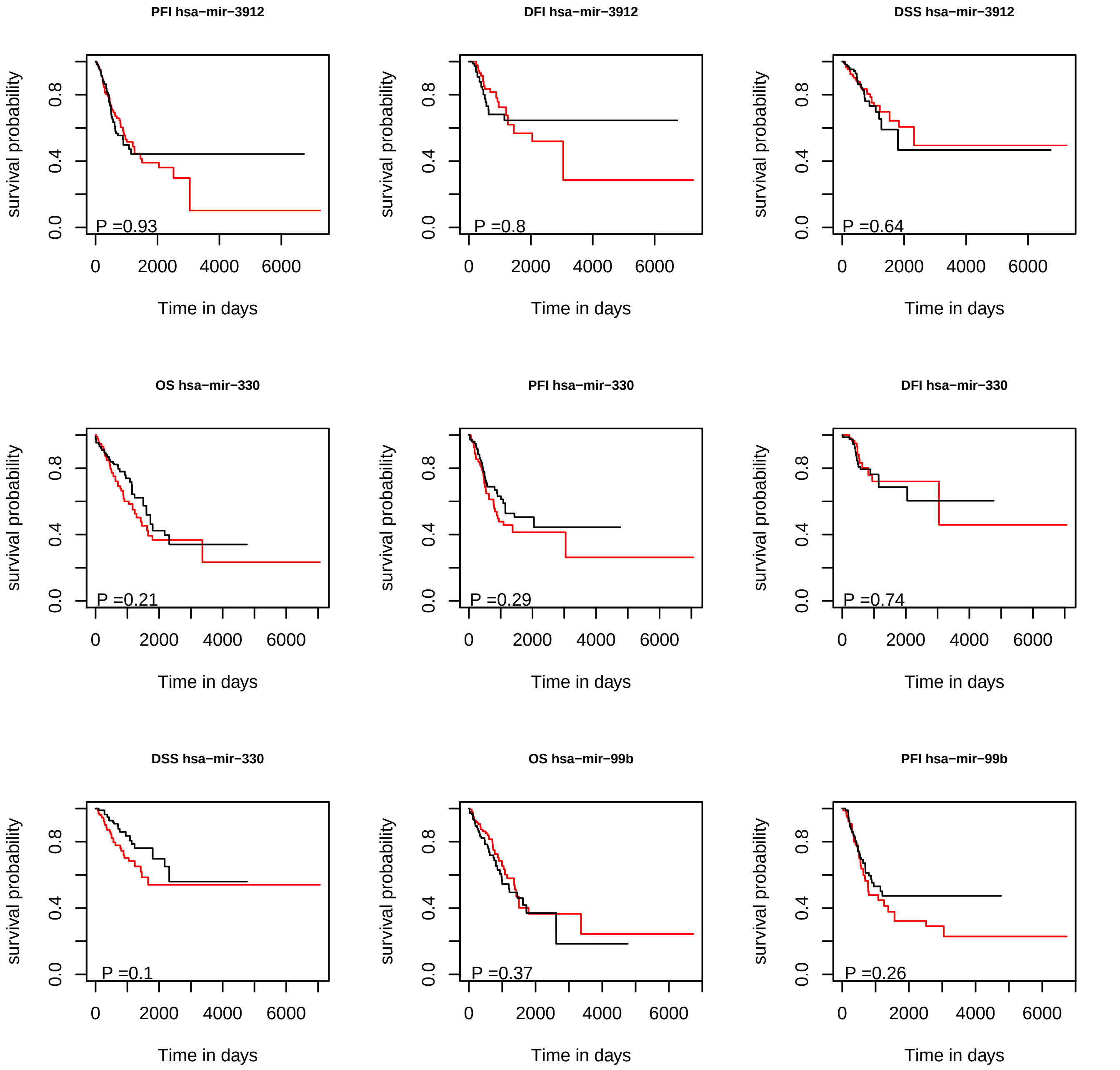
PFI hsa−mir−3912
survival probability
0.0
0.4
0.8
P =0.93
0
2000
4000
6000
Time in days
DFI hsa−mir−3912
P =0.8
DSS hsa−mir−3912
P =0.64
OS hsa−mir−330
P =0.21
PFI hsa−mir−330
P =0.29
DFI hsa−mir−330
P =0.74
DSS hsa−mir−330
P =0.1
OS hsa−mir−99b
P =0.37
PFI hsa−mir−99b
P =0.26

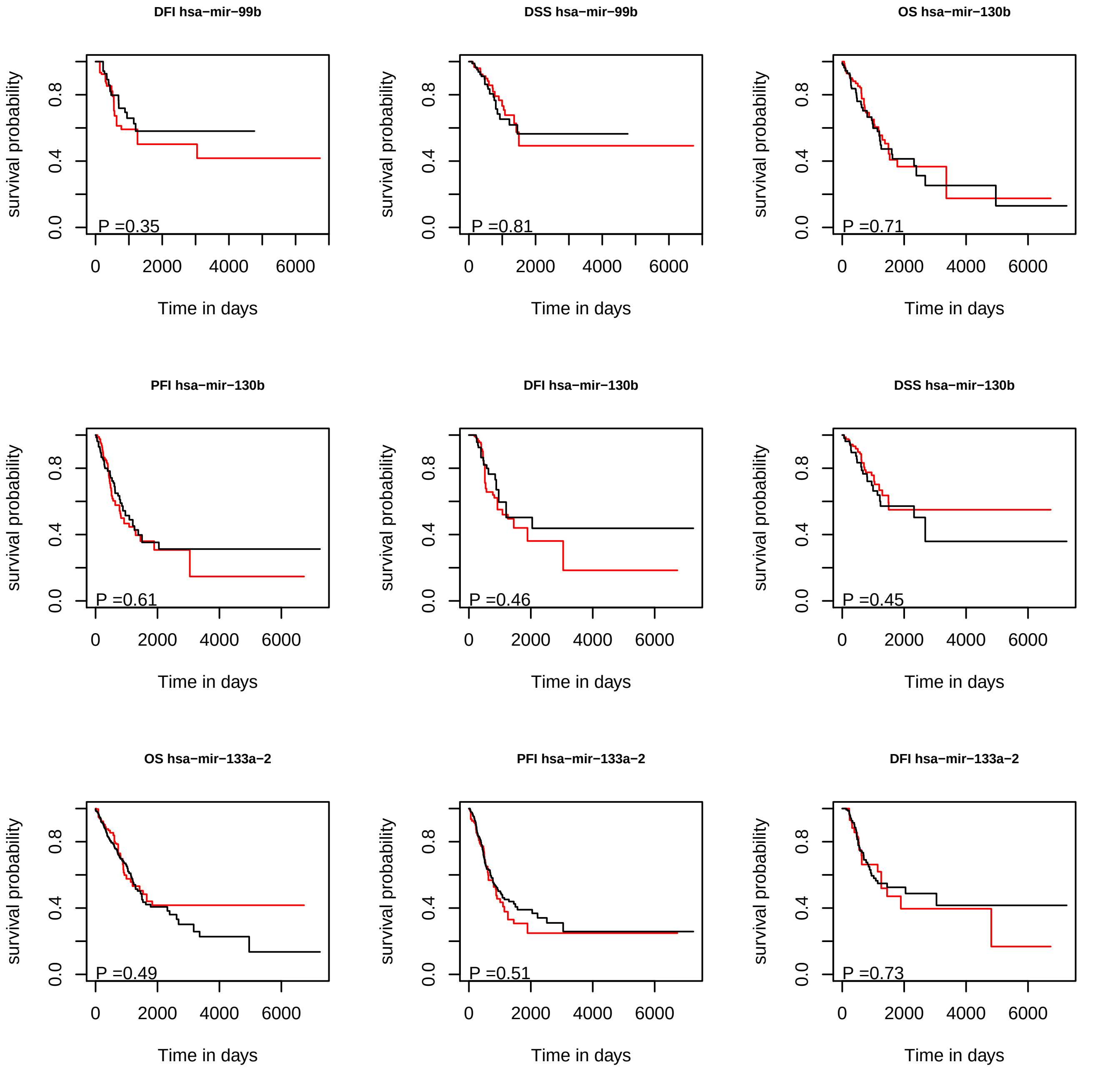
DFI hsa−mir−99b
P =0.35
DSS hsa−mir−99b
P =0.81
OS hsa−mir−130b
P =0.71
PFI hsa−mir−130b
P =0.61
DFI hsa−mir−130b
P =0.46
DSS hsa−mir−130b
P =0.45
OS hsa−mir−133a−2
P =0.49
PFI hsa−mir−133a−2
P =0.51
DFI hsa−mir−133a−2
P =0.73
survival probability
0.0
0.4
0.8
Time in days
0
2000
4000
6000

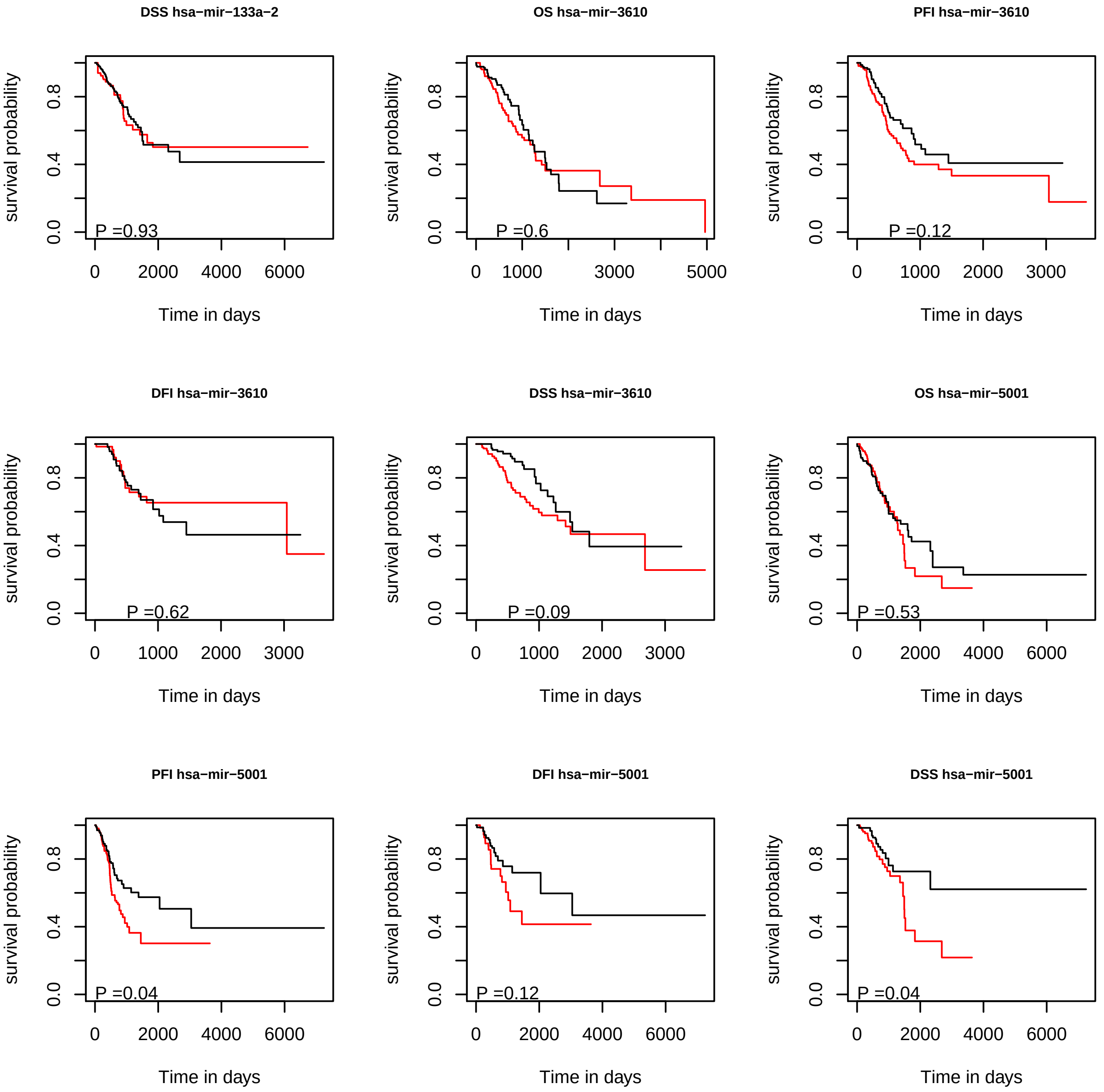
DSS hsa−mir−133a−2
survival probability
Time in days
P =0.93
OS hsa−mir−3610
survival probability
Time in days
P =0.6
PFI hsa−mir−3610
survival probability
Time in days
P =0.12
DFI hsa−mir−3610
survival probability
Time in days
P =0.62
DSS hsa−mir−3610
survival probability
Time in days
P =0.09
OS hsa−mir−5001
survival probability
Time in days
P =0.53
PFI hsa−mir−5001
survival probability
Time in days
P =0.04
DFI hsa−mir−5001
survival probability
Time in days
P =0.12
DSS hsa−mir−5001
survival probability
Time in days
P =0.04

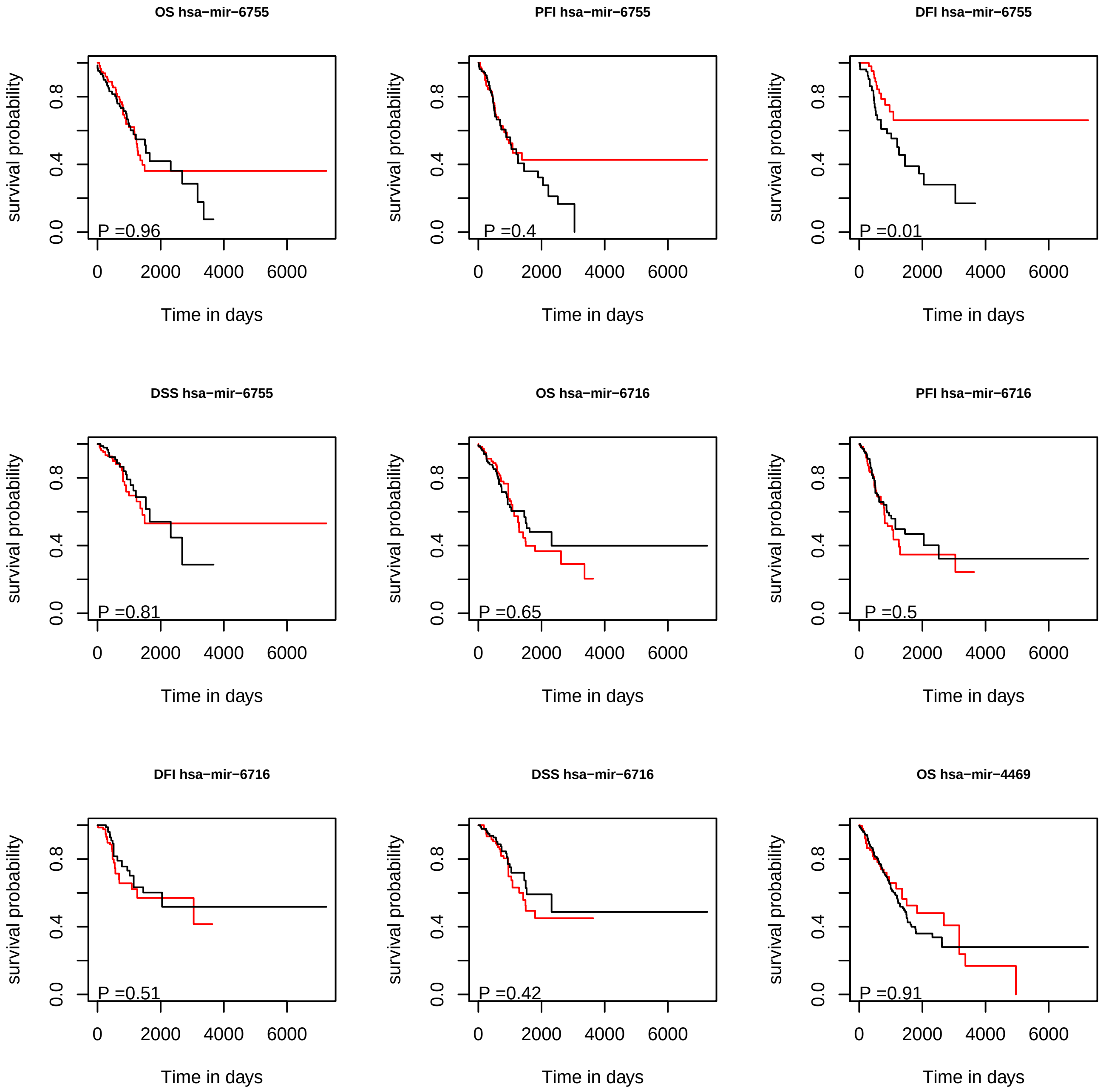
OS hsa−mir−6755
P =0.96
PFI hsa−mir−6755
P =0.4
DFI hsa−mir−6755
P =0.01
DSS hsa−mir−6755
P =0.81
OS hsa−mir−6716
P =0.65
PFI hsa−mir−6716
P =0.5
DFI hsa−mir−6716
P =0.51
DSS hsa−mir−6716
P =0.42
OS hsa−mir−4469
P =0.91
survival probability
0.0
0.4
0.8
0
2000
4000
6000
Time in days

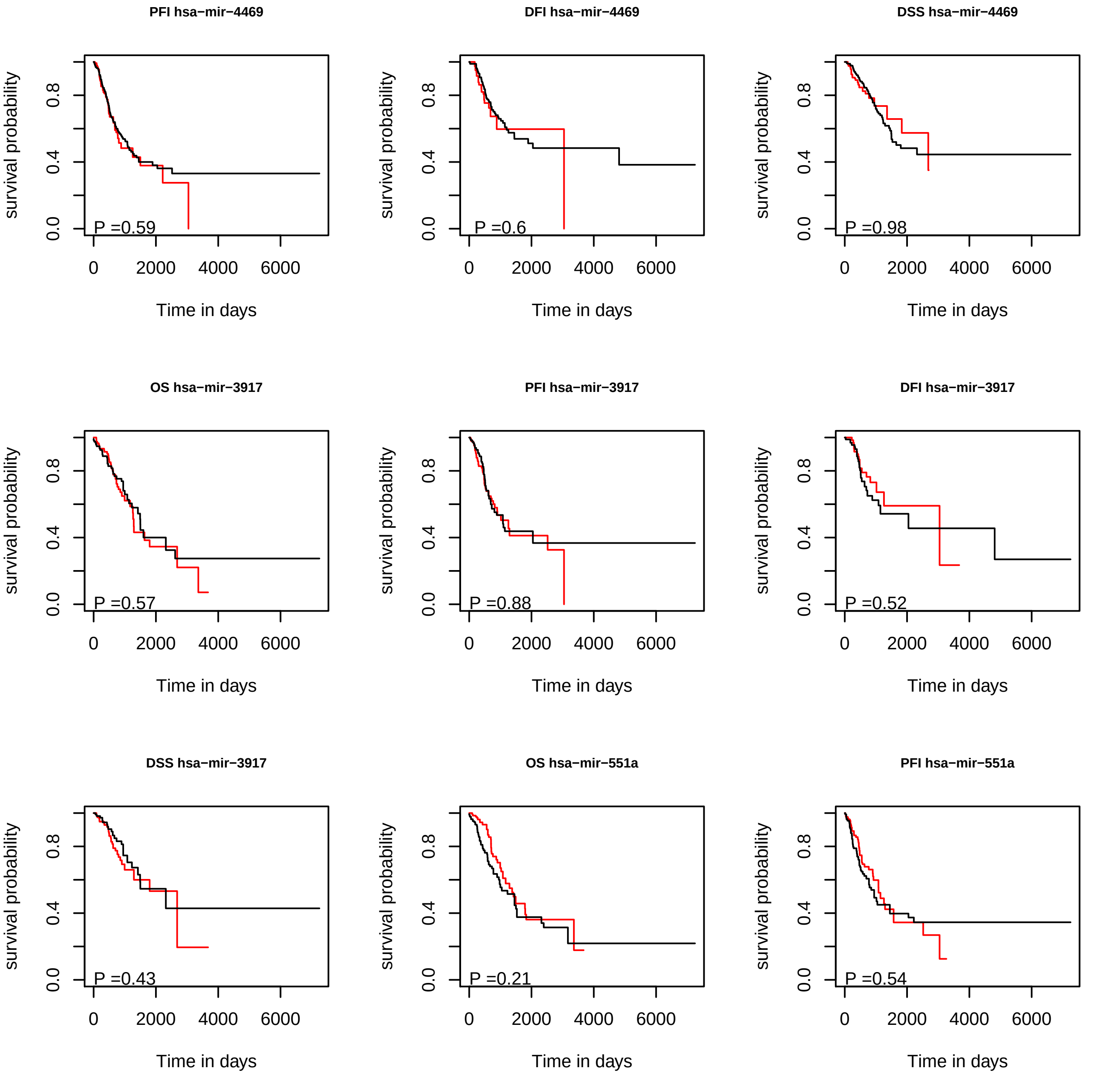
PFI hsa−mir−4469
DFI hsa−mir−4469
DSS hsa−mir−4469
OS hsa−mir−3917
PFI hsa−mir−3917
DFI hsa−mir−3917
DSS hsa−mir−3917
OS hsa−mir−551a
PFI hsa−mir−551a
survival probability
Time in days
0.0
0.4
0.8
0
2000
4000
6000
P =0.59
P =0.6
P =0.98
P =0.57
P =0.88
P =0.52
P =0.43
P =0.21
P =0.54

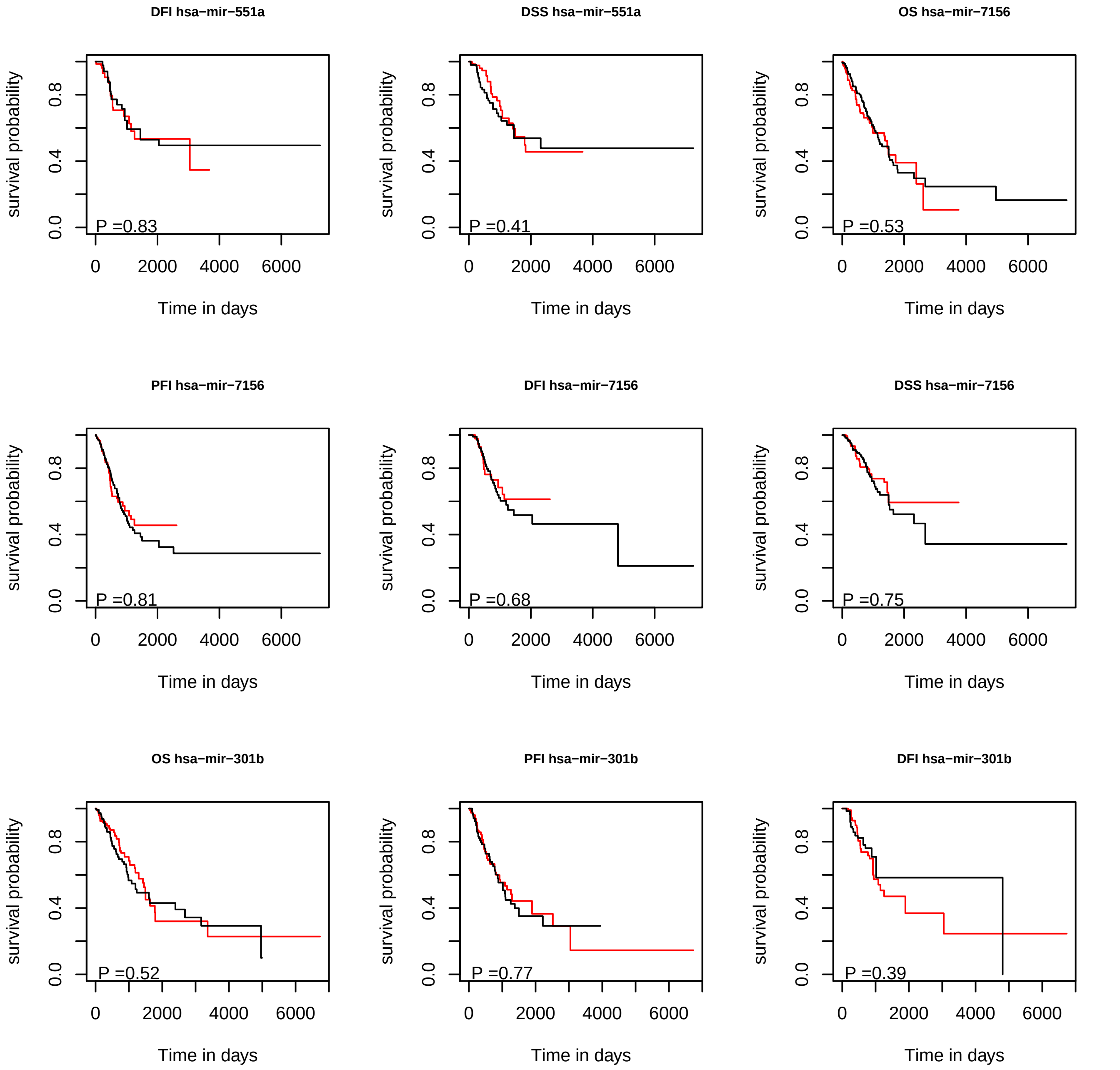
DFI hsa−mir−551a
P =0.83
DSS hsa−mir−551a
P =0.41
OS hsa−mir−7156
P =0.53
PFI hsa−mir−7156
P =0.81
DFI hsa−mir−7156
P =0.68
DSS hsa−mir−7156
P =0.75
OS hsa−mir−301b
P =0.52
PFI hsa−mir−301b
P =0.77
DFI hsa−mir−301b
P =0.39
survival probability
0.0
0.4
0.8
0
2000
4000
6000
Time in days

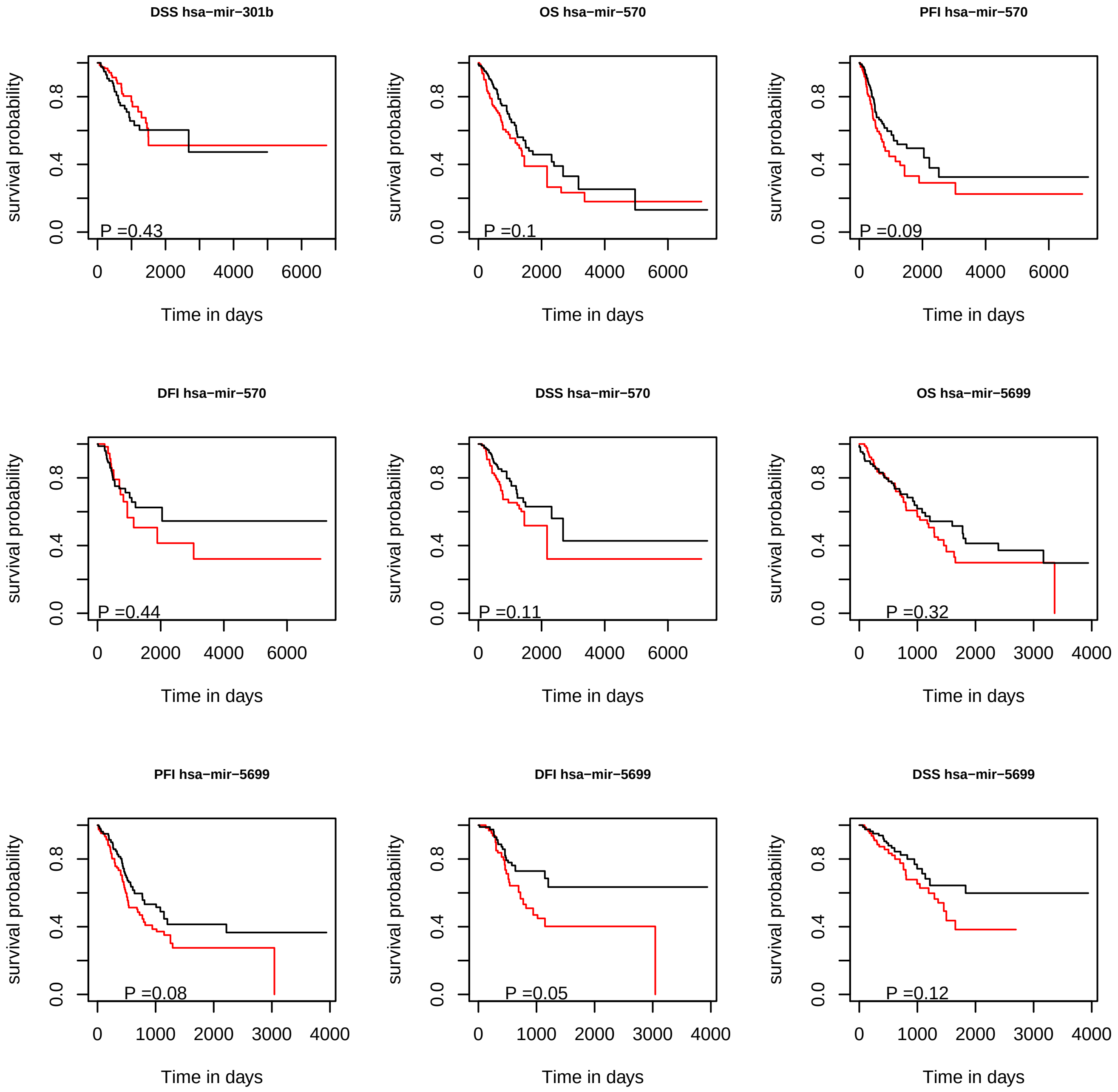
DSS hsa−mir−301b
P =0.43
OS hsa−mir−570
P =0.1
PFI hsa−mir−570
P =0.09
DFI hsa−mir−570
P =0.44
DSS hsa−mir−570
P =0.11
OS hsa−mir−5699
P =0.32
PFI hsa−mir−5699
P =0.08
DFI hsa−mir−5699
P =0.05
DSS hsa−mir−5699
P =0.12
survival probability
0.0
0.4
0.8
Time in days
0
1000
2000
3000
4000
6000

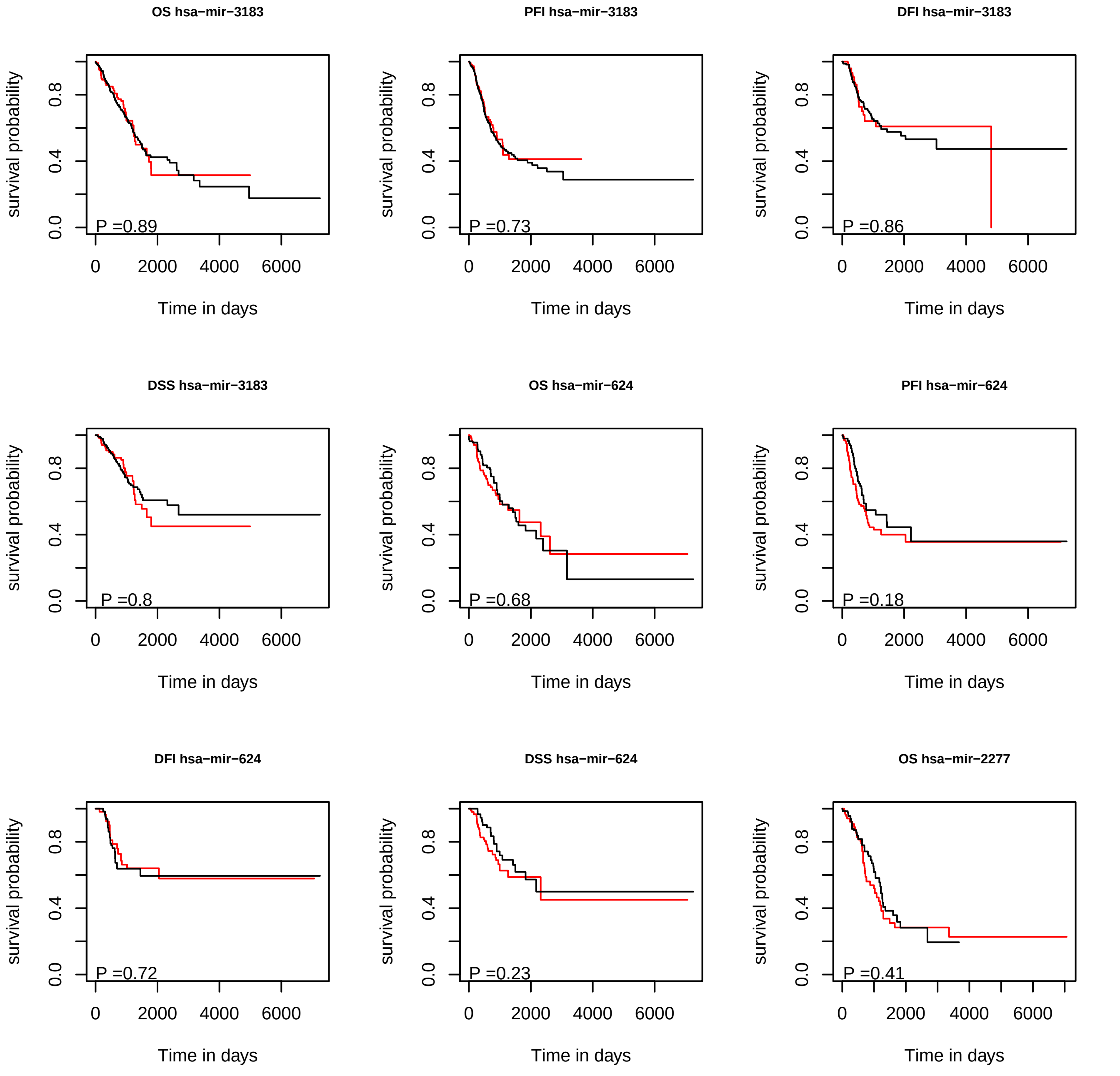
OS hsa−mir−3183
survival probability
0.0
0.4
0.8
P =0.89
0
2000
4000
6000
Time in days
PFI hsa−mir−3183
survival probability
P =0.73
Time in days
DFI hsa−mir−3183
survival probability
P =0.86
Time in days
DSS hsa−mir−3183
survival probability
P =0.8
Time in days
OS hsa−mir−624
survival probability
P =0.68
Time in days
PFI hsa−mir−624
survival probability
P =0.18
Time in days
DFI hsa−mir−624
survival probability
P =0.72
Time in days
DSS hsa−mir−624
survival probability
P =0.23
Time in days
OS hsa−mir−2277
survival probability
P =0.41
Time in days

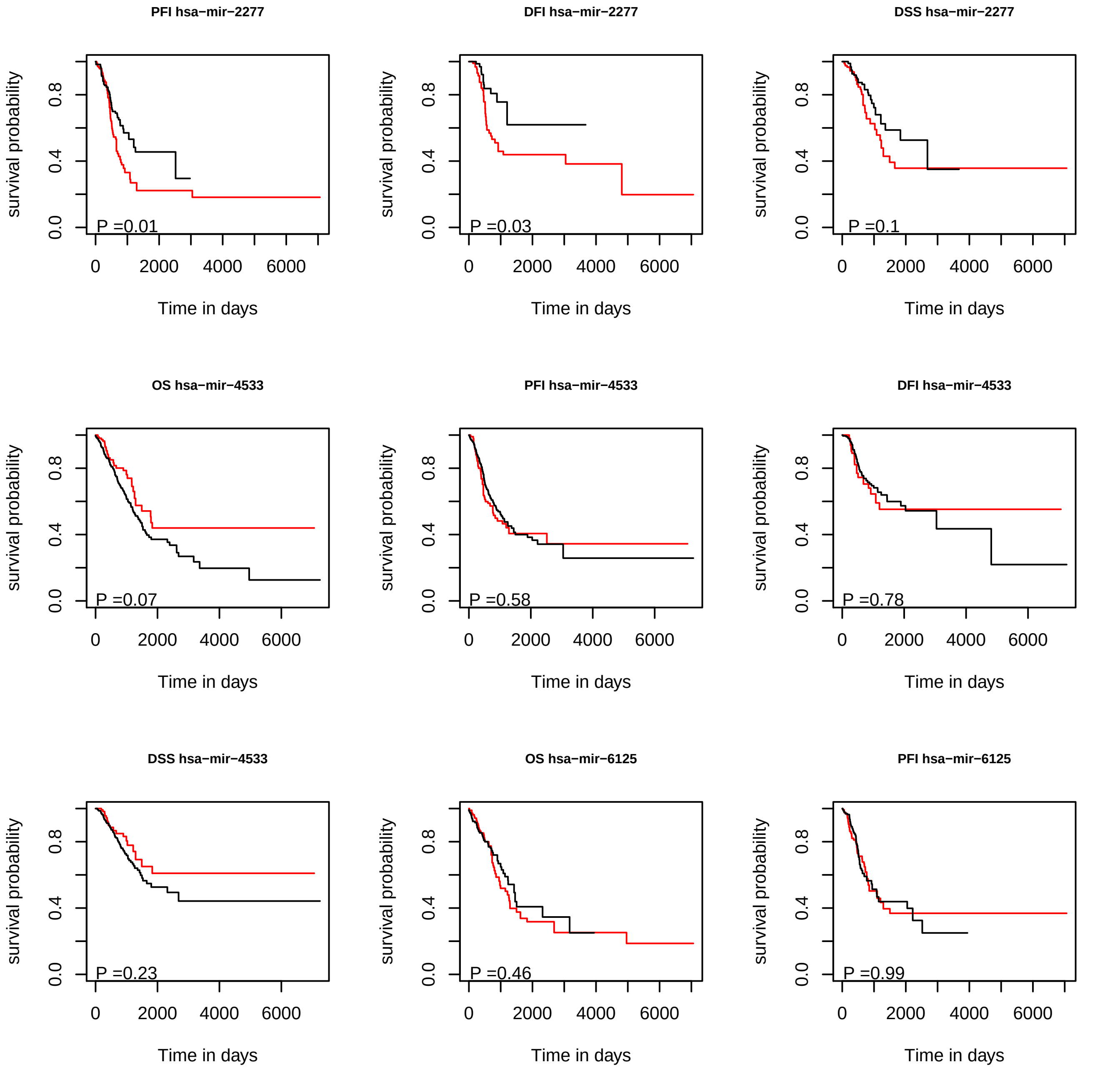
PFI hsa−mir−2277
P =0.01
DFI hsa−mir−2277
P =0.03
DSS hsa−mir−2277
P =0.1
OS hsa−mir−4533
P =0.07
PFI hsa−mir−4533
P =0.58
DFI hsa−mir−4533
P =0.78
DSS hsa−mir−4533
P =0.23
OS hsa−mir−6125
P =0.46
PFI hsa−mir−6125
P =0.99
survival probability
0.0
0.4
0.8
Time in days
0
2000
4000
6000

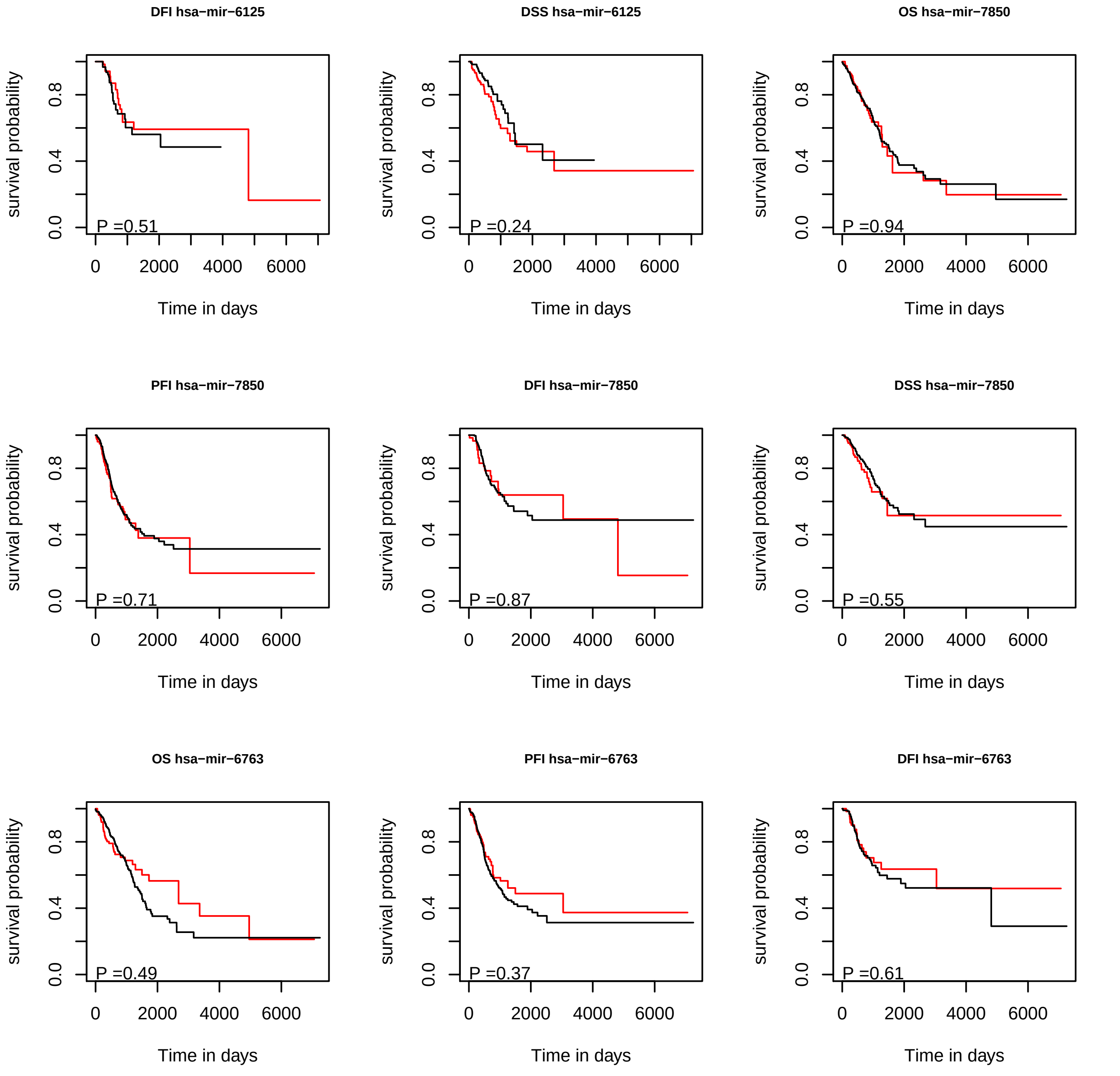
DFI hsa−mir−6125
P =0.51
DSS hsa−mir−6125
P =0.24
OS hsa−mir−7850
P =0.94
PFI hsa−mir−7850
P =0.71
DFI hsa−mir−7850
P =0.87
DSS hsa−mir−7850
P =0.55
OS hsa−mir−6763
P =0.49
PFI hsa−mir−6763
P =0.37
DFI hsa−mir−6763
P =0.61
survival probability
Time in days
0.0
0.4
0.8
0
2000
4000
6000

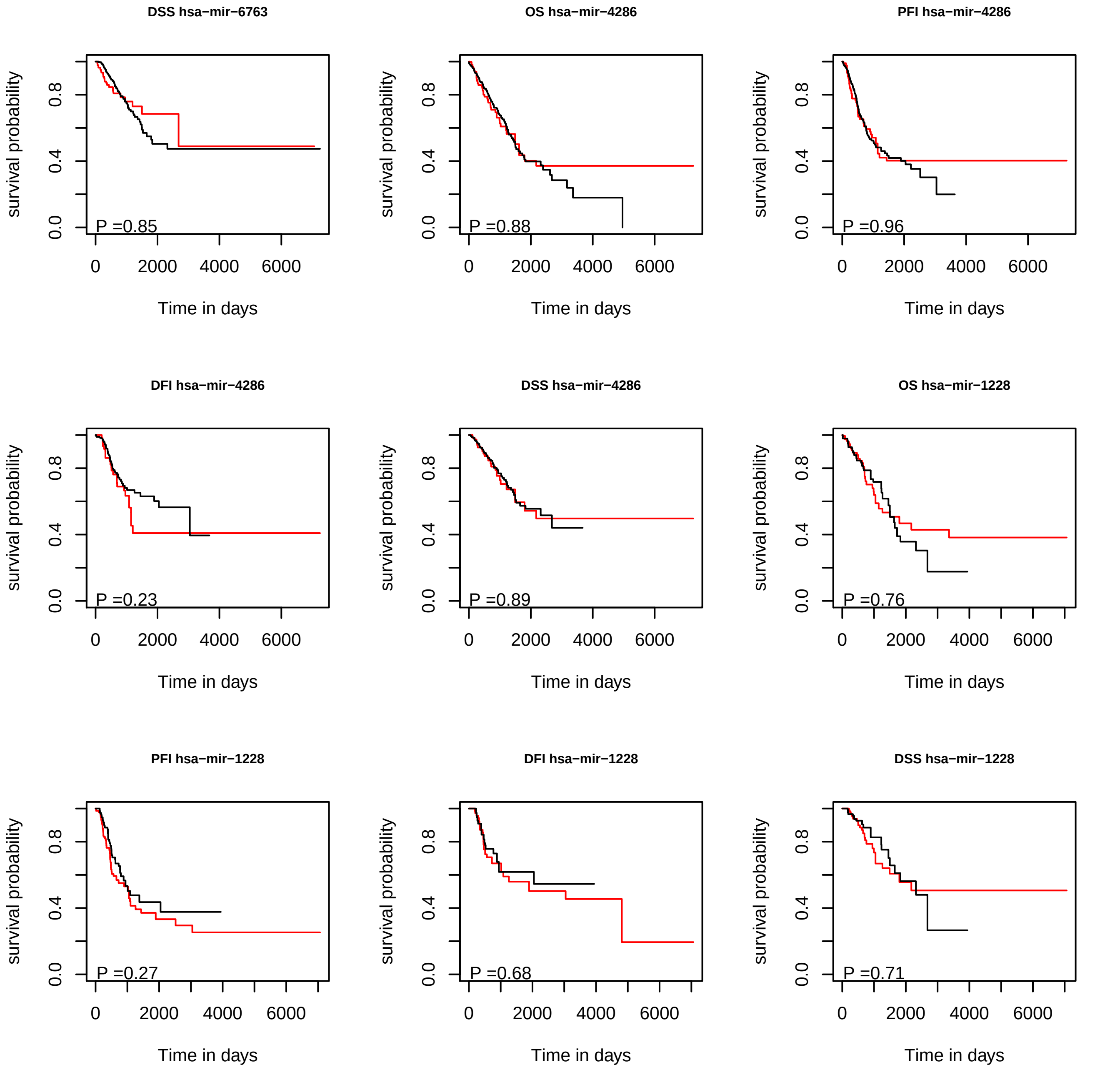

DSS hsa−mir−6763
P =0.85
OS hsa−mir−4286
P =0.88
PFI hsa−mir−4286
P =0.96
DFI hsa−mir−4286
P =0.23
DSS hsa−mir−4286
P =0.89
OS hsa−mir−1228
P =0.76
PFI hsa−mir−1228
P =0.27
DFI hsa−mir−1228
P =0.68
DSS hsa−mir−1228
P =0.71
survival probability
0.0
0.4
0.8
Time in days
0
2000
4000
6000

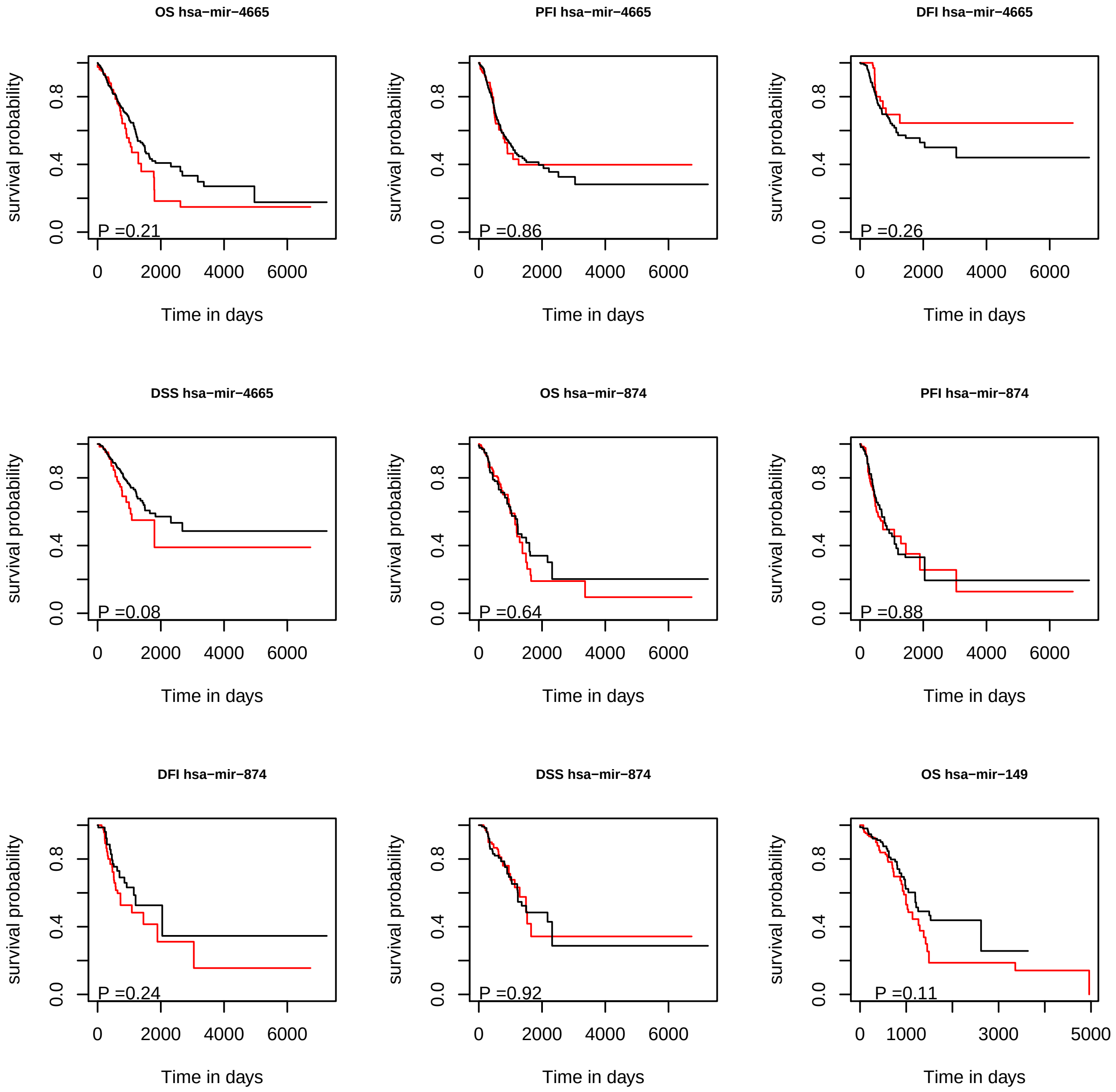
OS hsa−mir−4665
P =0.21
PFI hsa−mir−4665
P =0.86
DFI hsa−mir−4665
P =0.26
DSS hsa−mir−4665
P =0.08
OS hsa−mir−874
P =0.64
PFI hsa−mir−874
P =0.88
DFI hsa−mir−874
P =0.24
DSS hsa−mir−874
P =0.92
OS hsa−mir−149
P =0.11
survival probability
0.0
0.4
0.8
0
2000
4000
6000
1000
3000
5000
Time in days

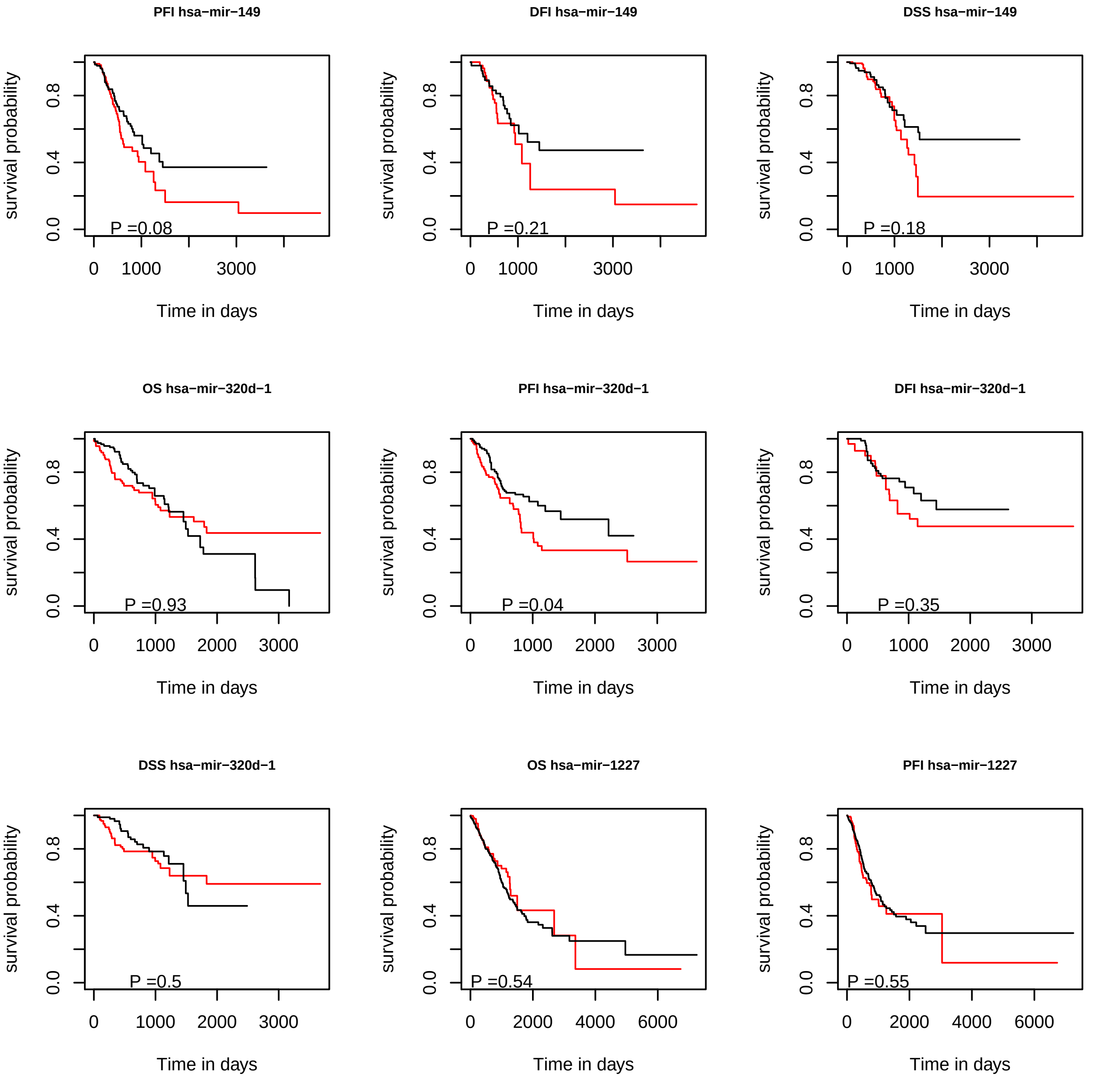
PFI hsa−mir−149
P =0.08
DFI hsa−mir−149
P =0.21
DSS hsa−mir−149
P =0.18
OS hsa−mir−320d−1
P =0.93
PFI hsa−mir−320d−1
P =0.04
DFI hsa−mir−320d−1
P =0.35
DSS hsa−mir−320d−1
P =0.5
OS hsa−mir−1227
P =0.54
PFI hsa−mir−1227
P =0.55
survival probability
0.0
0.4
0.8
Time in days
0
1000
2000
3000
4000
6000

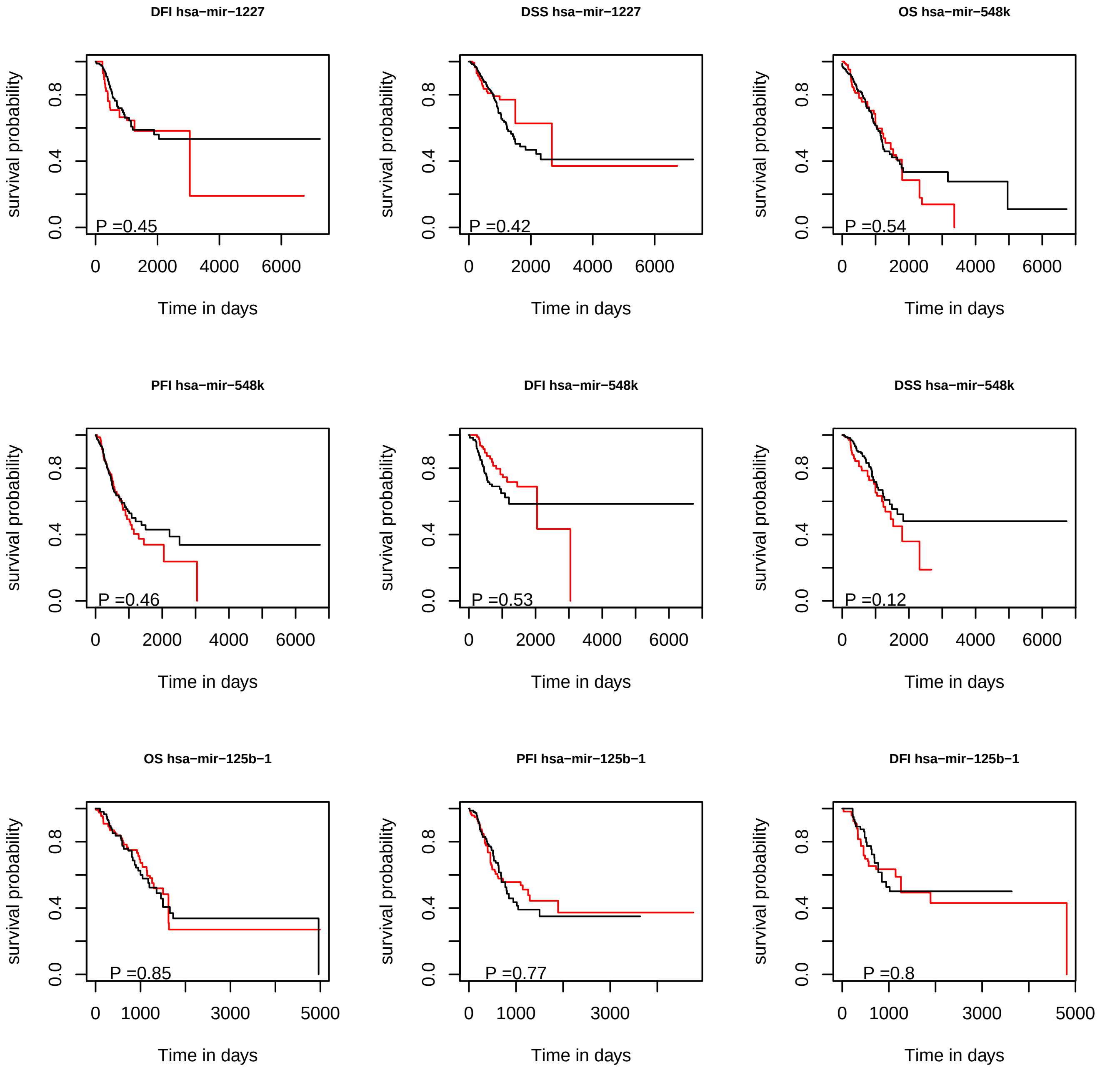
DFI hsa−mir−1227
P =0.45
DSS hsa−mir−1227
P =0.42
OS hsa−mir−548k
P =0.54
PFI hsa−mir−548k
P =0.46
DFI hsa−mir−548k
P =0.53
DSS hsa−mir−548k
P =0.12
OS hsa−mir−125b−1
P =0.85
PFI hsa−mir−125b−1
P =0.77
DFI hsa−mir−125b−1
P =0.8
survival probability
0.0
0.4
0.8
Time in days
0
1000
2000
3000
4000
5000
6000

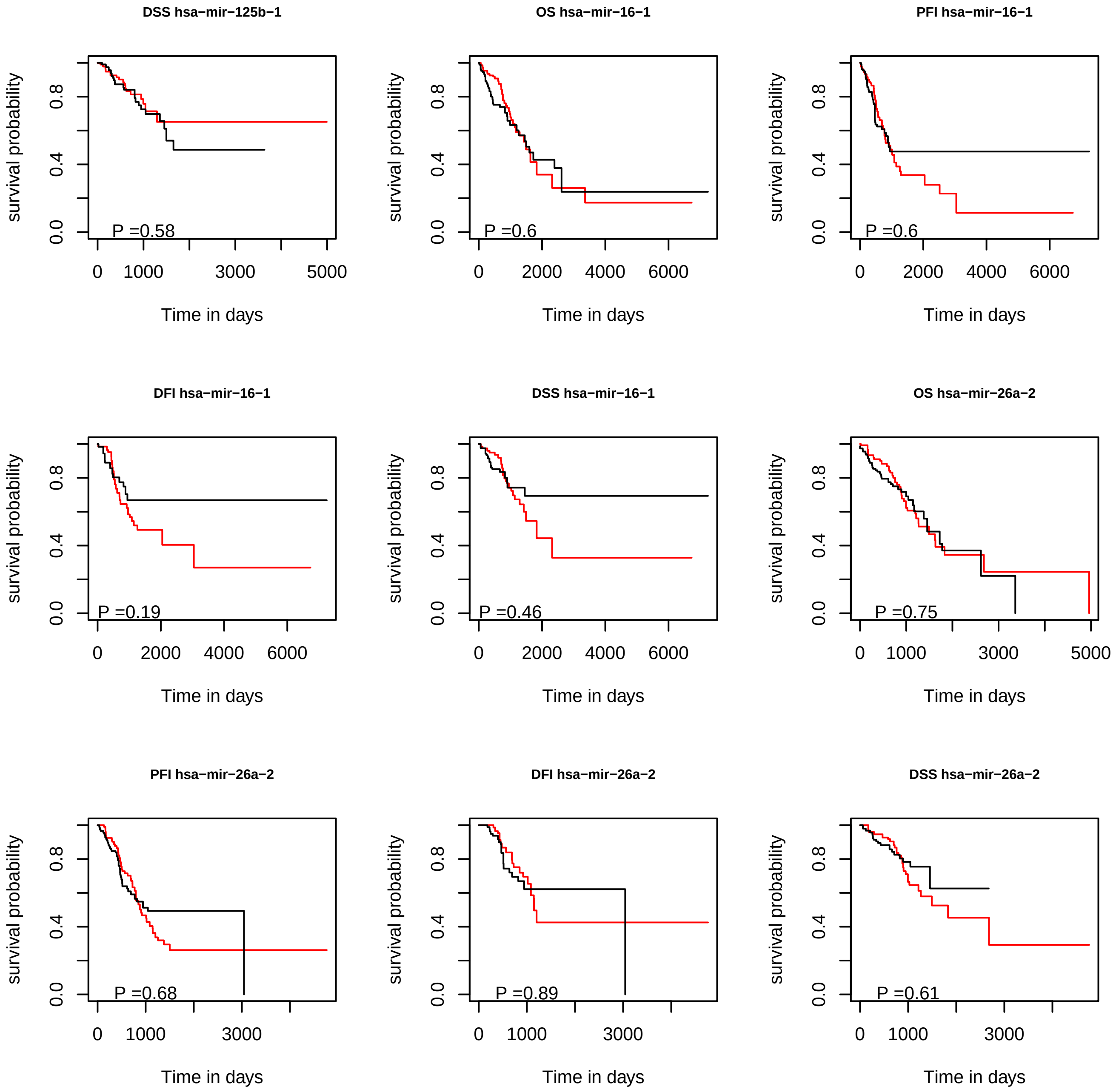
DSS hsa−mir−125b−1
P =0.58
OS hsa−mir−16−1
P =0.6
PFI hsa−mir−16−1
P =0.6
DFI hsa−mir−16−1
P =0.19
DSS hsa−mir−16−1
P =0.46
OS hsa−mir−26a−2
P =0.75
PFI hsa−mir−26a−2
P =0.68
DFI hsa−mir−26a−2
P =0.89
DSS hsa−mir−26a−2
P =0.61
survival probability
Time in days
0.0
0.4
0.8
0
1000
2000
3000
4000
5000
6000

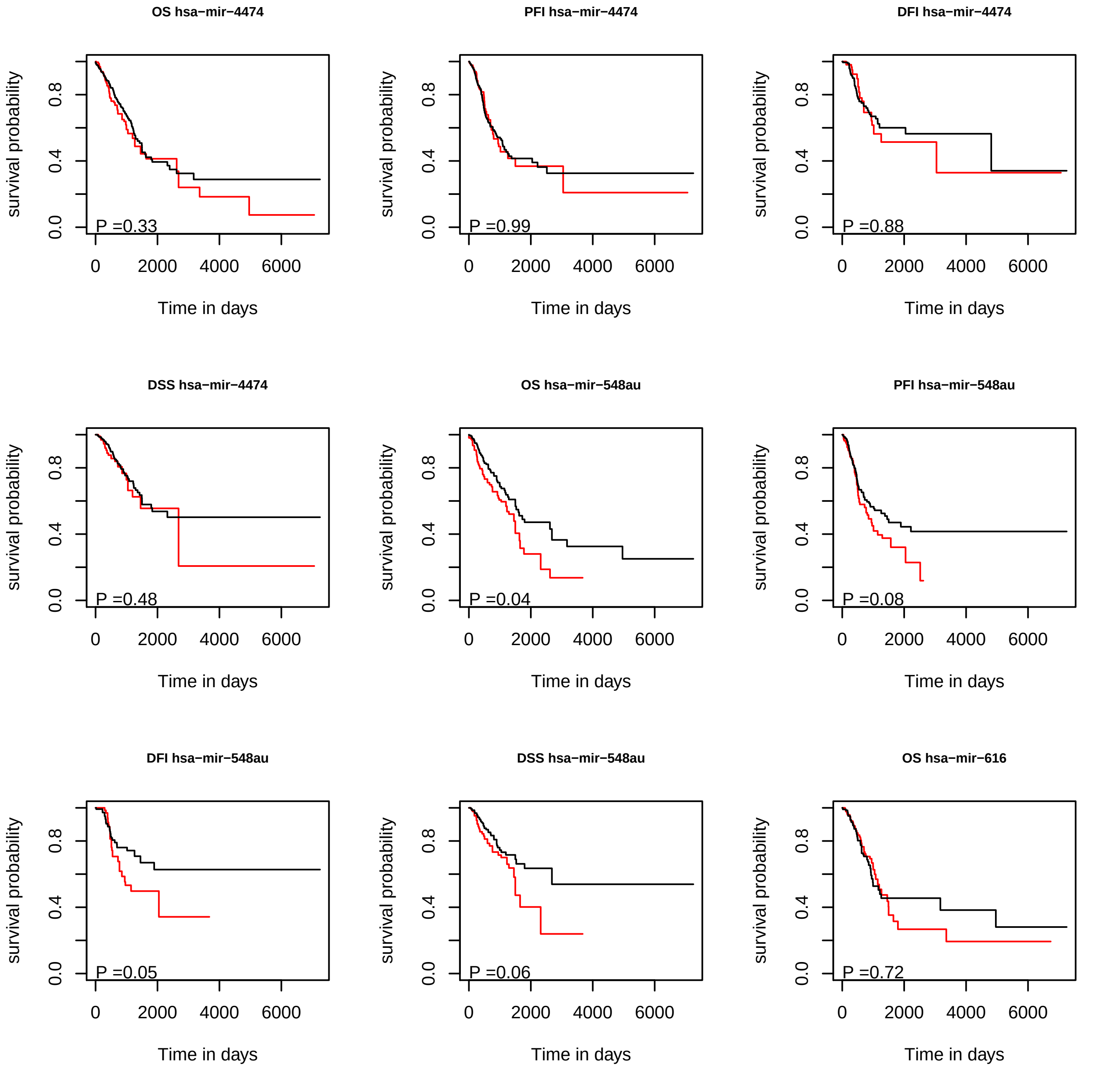
OS hsa−mir−4474
P =0.33
PFI hsa−mir−4474
P =0.99
DFI hsa−mir−4474
P =0.88
DSS hsa−mir−4474
P =0.48
OS hsa−mir−548au
P =0.04
PFI hsa−mir−548au
P =0.08
DFI hsa−mir−548au
P =0.05
DSS hsa−mir−548au
P =0.06
OS hsa−mir−616
P =0.72
survival probability
0.0
0.4
0.8
Time in days
0
2000
4000
6000

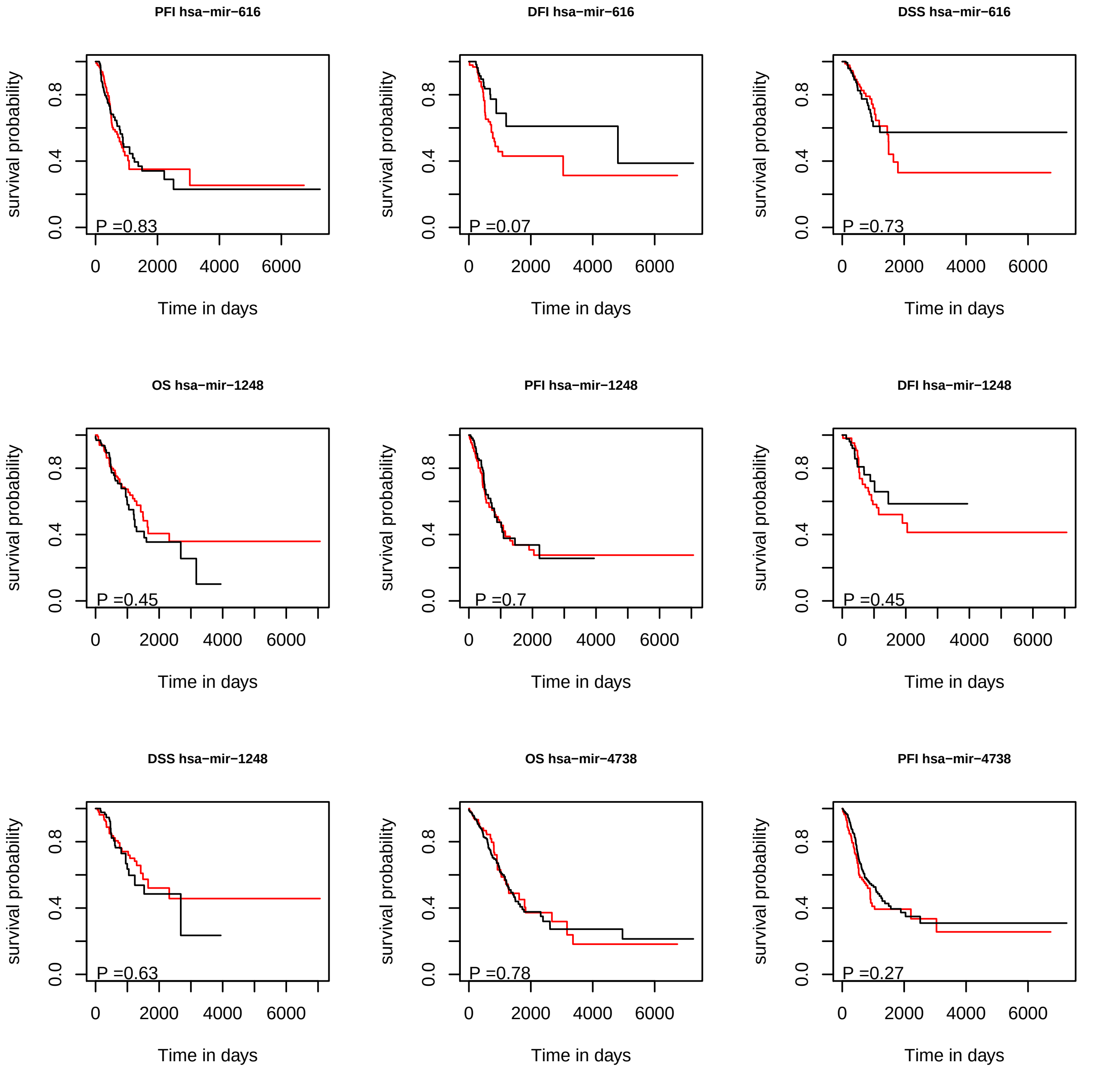
PFI hsa−mir−616
P =0.83
DFI hsa−mir−616
P =0.07
DSS hsa−mir−616
P =0.73
OS hsa−mir−1248
P =0.45
PFI hsa−mir−1248
P =0.7
DFI hsa−mir−1248
P =0.45
DSS hsa−mir−1248
P =0.63
OS hsa−mir−4738
P =0.78
PFI hsa−mir−4738
P =0.27
survival probability
Time in days
0.0
0.4
0.8
0
2000
4000
6000

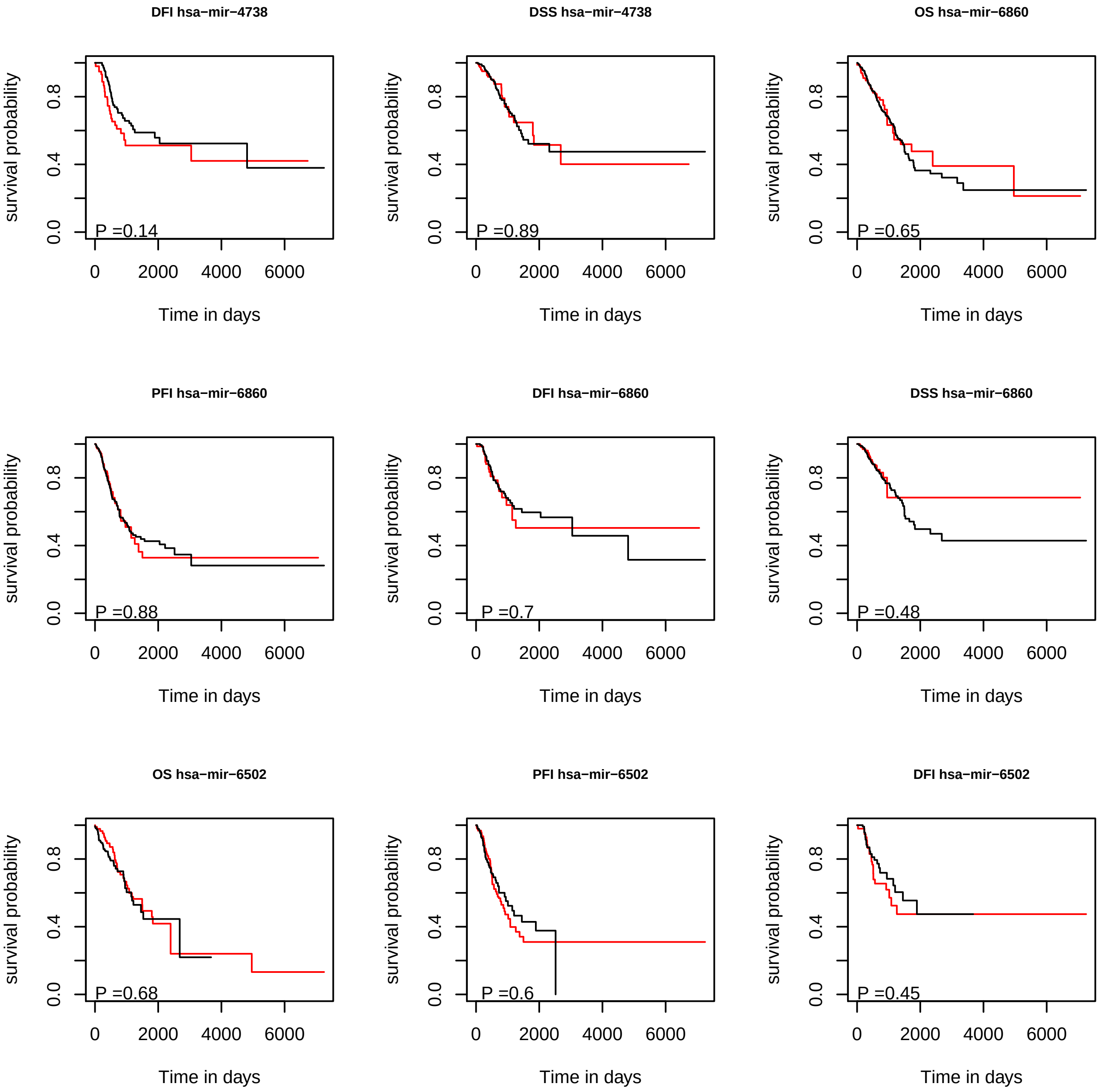
DFI hsa−mir−4738
P =0.14
DSS hsa−mir−4738
P =0.89
OS hsa−mir−6860
P =0.65
PFI hsa−mir−6860
P =0.88
DFI hsa−mir−6860
P =0.7
DSS hsa−mir−6860
P =0.48
OS hsa−mir−6502
P =0.68
PFI hsa−mir−6502
P =0.6
DFI hsa−mir−6502
P =0.45
survival probability
Time in days
0.0
0.4
0.8
0
2000
4000
6000

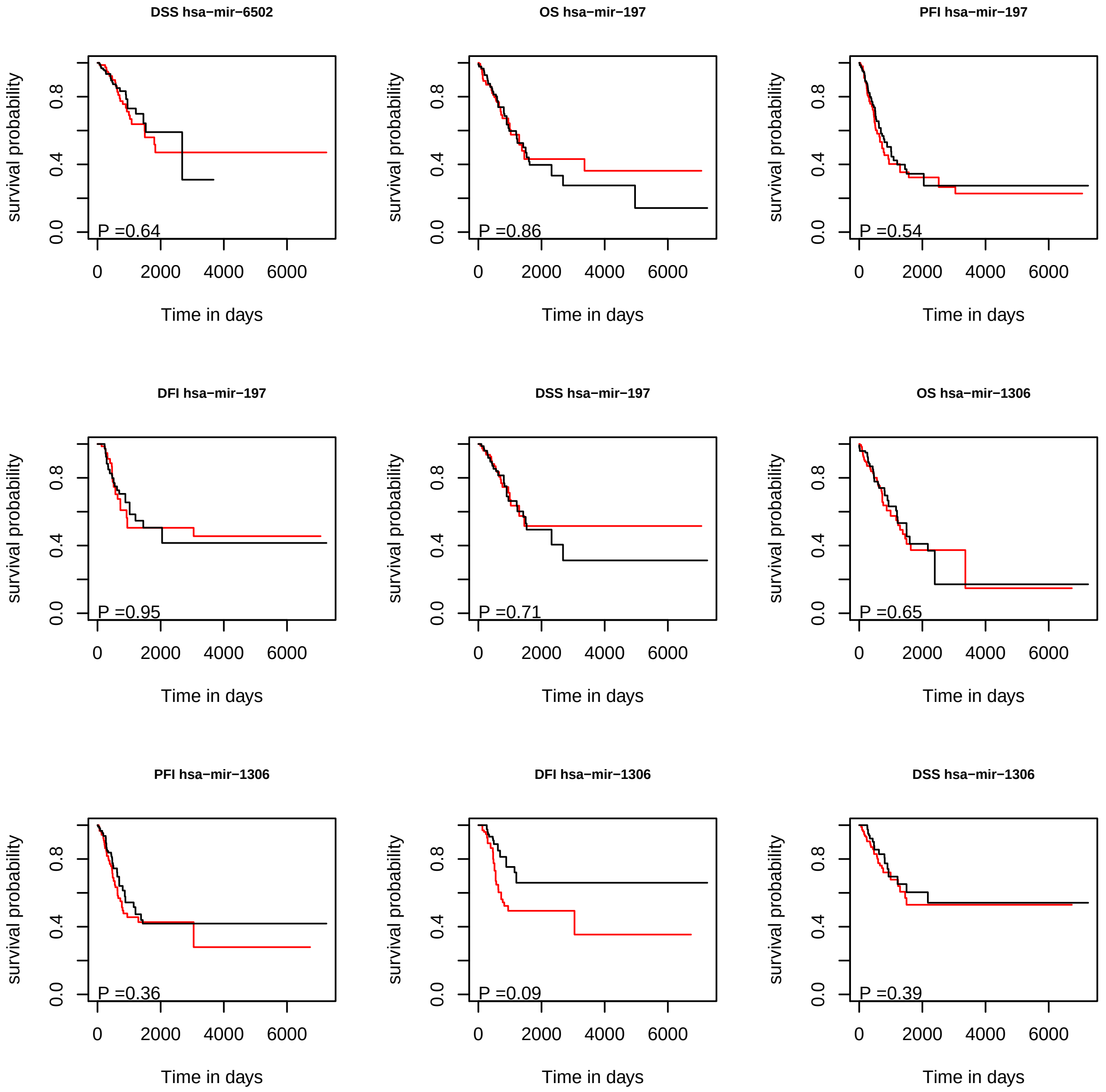
DSS hsa−mir−6502
P =0.64
OS hsa−mir−197
P =0.86
PFI hsa−mir−197
P =0.54
DFI hsa−mir−197
P =0.95
DSS hsa−mir−197
P =0.71
OS hsa−mir−1306
P =0.65
PFI hsa−mir−1306
P =0.36
DFI hsa−mir−1306
P =0.09
DSS hsa−mir−1306
P =0.39
survival probability
0.0
0.4
0.8
0
2000
4000
6000
Time in days

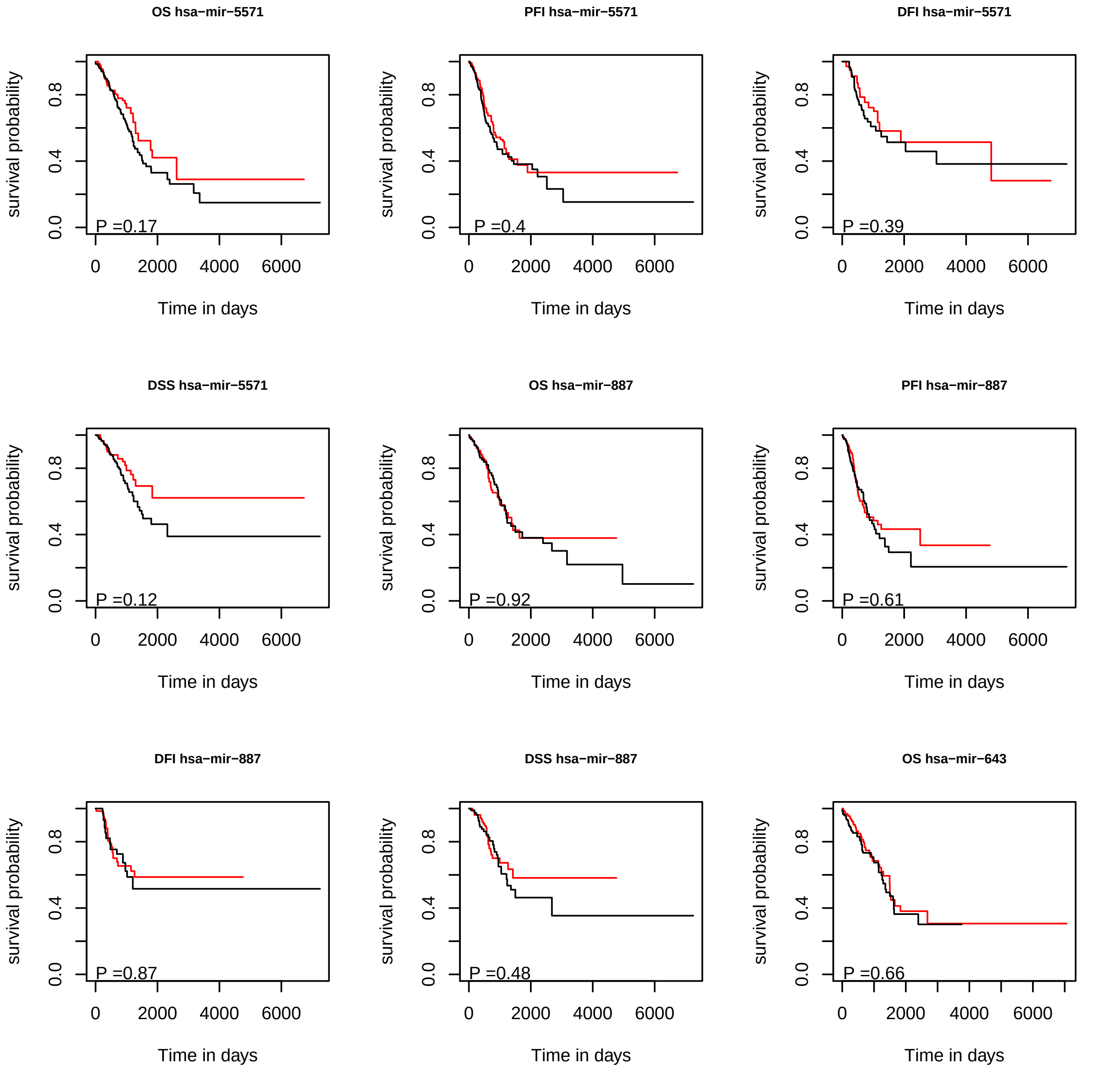
OS hsa−mir−5571
P =0.17
PFI hsa−mir−5571
P =0.4
DFI hsa−mir−5571
P =0.39
DSS hsa−mir−5571
P =0.12
OS hsa−mir−887
P =0.92
PFI hsa−mir−887
P =0.61
DFI hsa−mir−887
P =0.87
DSS hsa−mir−887
P =0.48
OS hsa−mir−643
P =0.66
survival probability
Time in days
0.0
0.4
0.8
0
2000
4000
6000

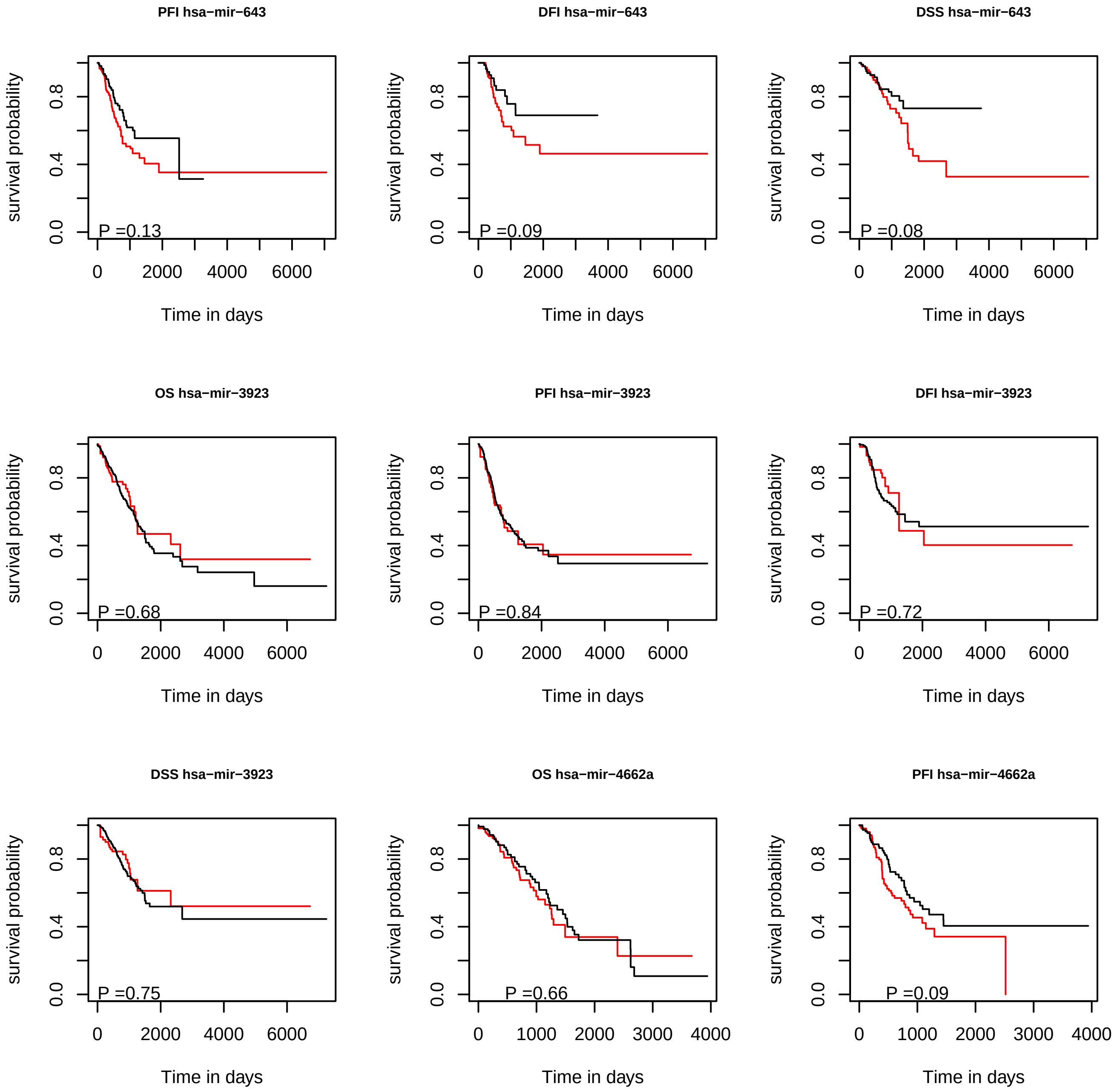
PFI hsa−mir−643
P =0.13
DFI hsa−mir−643
P =0.09
DSS hsa−mir−643
P =0.08
OS hsa−mir−3923
P =0.68
PFI hsa−mir−3923
P =0.84
DFI hsa−mir−3923
P =0.72
DSS hsa−mir−3923
P =0.75
OS hsa−mir−4662a
P =0.66
PFI hsa−mir−4662a
P =0.09
survival probability
Time in days
0.0
0.4
0.8
0
1000
2000
3000
4000
6000

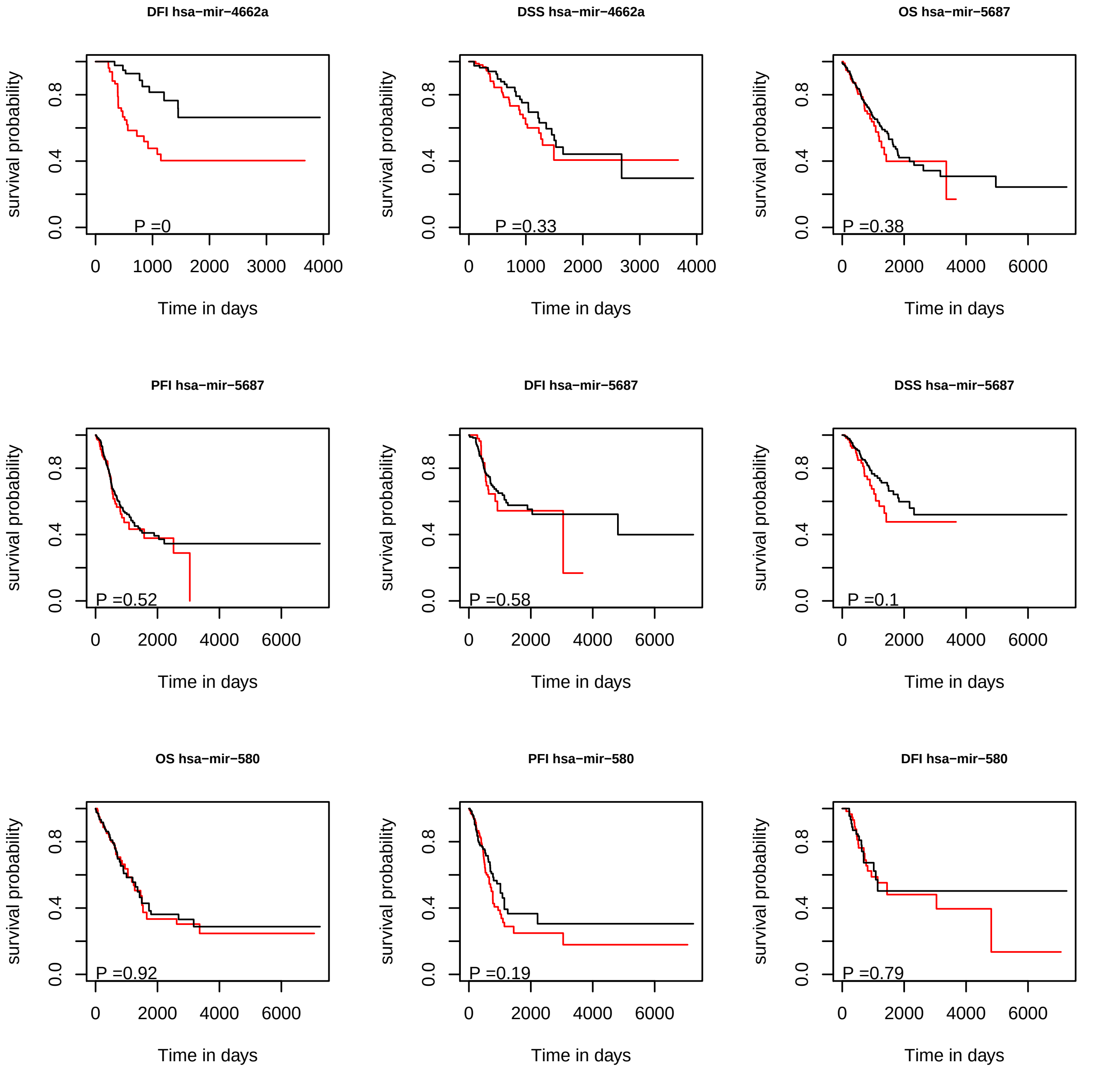

DFI hsa−mir−4662a
P =0
DSS hsa−mir−4662a
P =0.33
OS hsa−mir−5687
P =0.38
PFI hsa−mir−5687
P =0.52
DFI hsa−mir−5687
P =0.58
DSS hsa−mir−5687
P =0.1
OS hsa−mir−580
P =0.92
PFI hsa−mir−580
P =0.19
DFI hsa−mir−580
P =0.79
survival probability
Time in days
0.0
0.4
0.8
0
1000
2000
3000
4000
6000

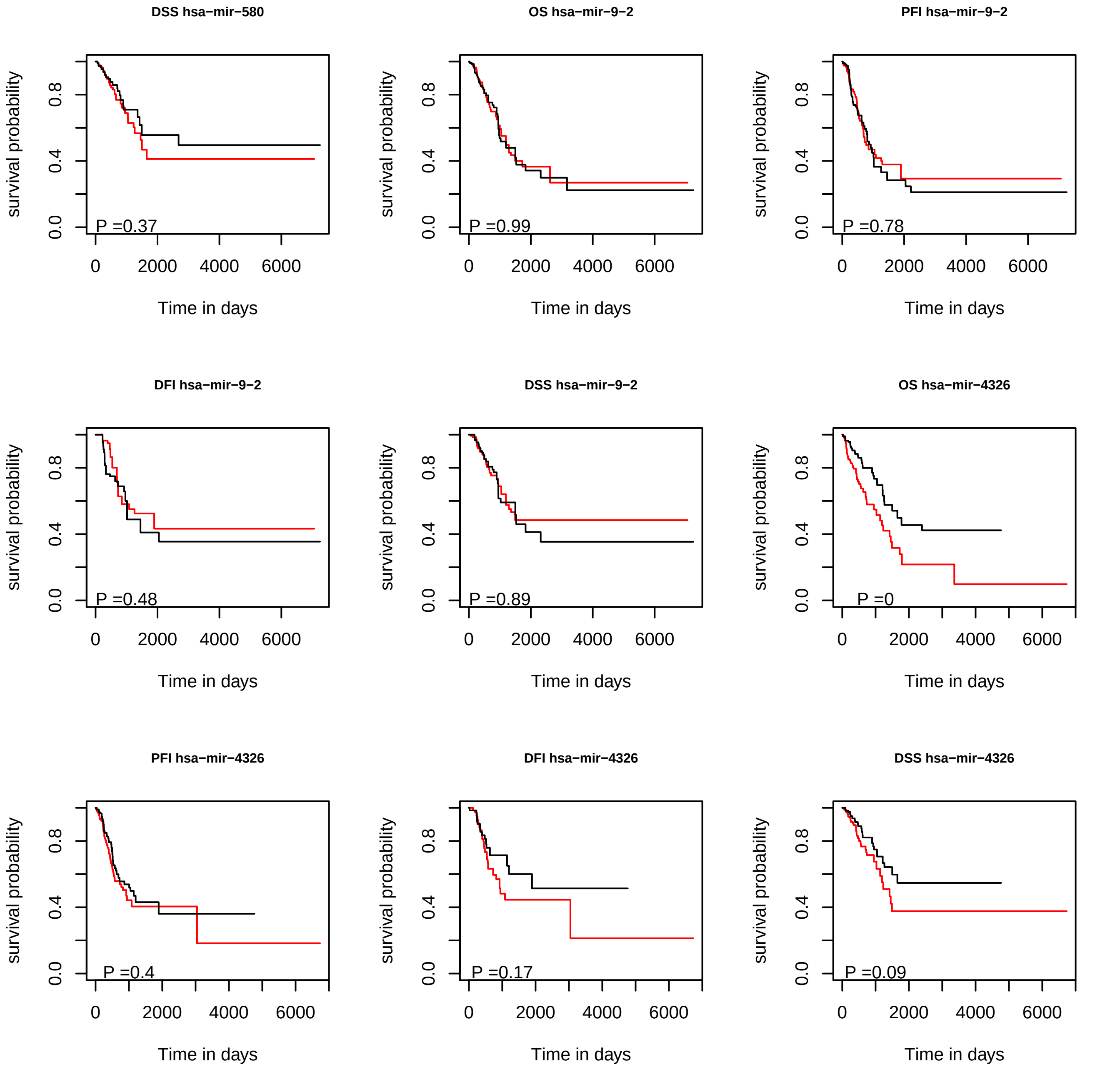
DSS hsa−mir−580
P =0.37
OS hsa−mir−9−2
P =0.99
PFI hsa−mir−9−2
P =0.78
DFI hsa−mir−9−2
P =0.48
DSS hsa−mir−9−2
P =0.89
OS hsa−mir−4326
P =0
PFI hsa−mir−4326
P =0.4
DFI hsa−mir−4326
P =0.17
DSS hsa−mir−4326
P =0.09
survival probability
0.0
0.4
0.8
Time in days
0
2000
4000
6000

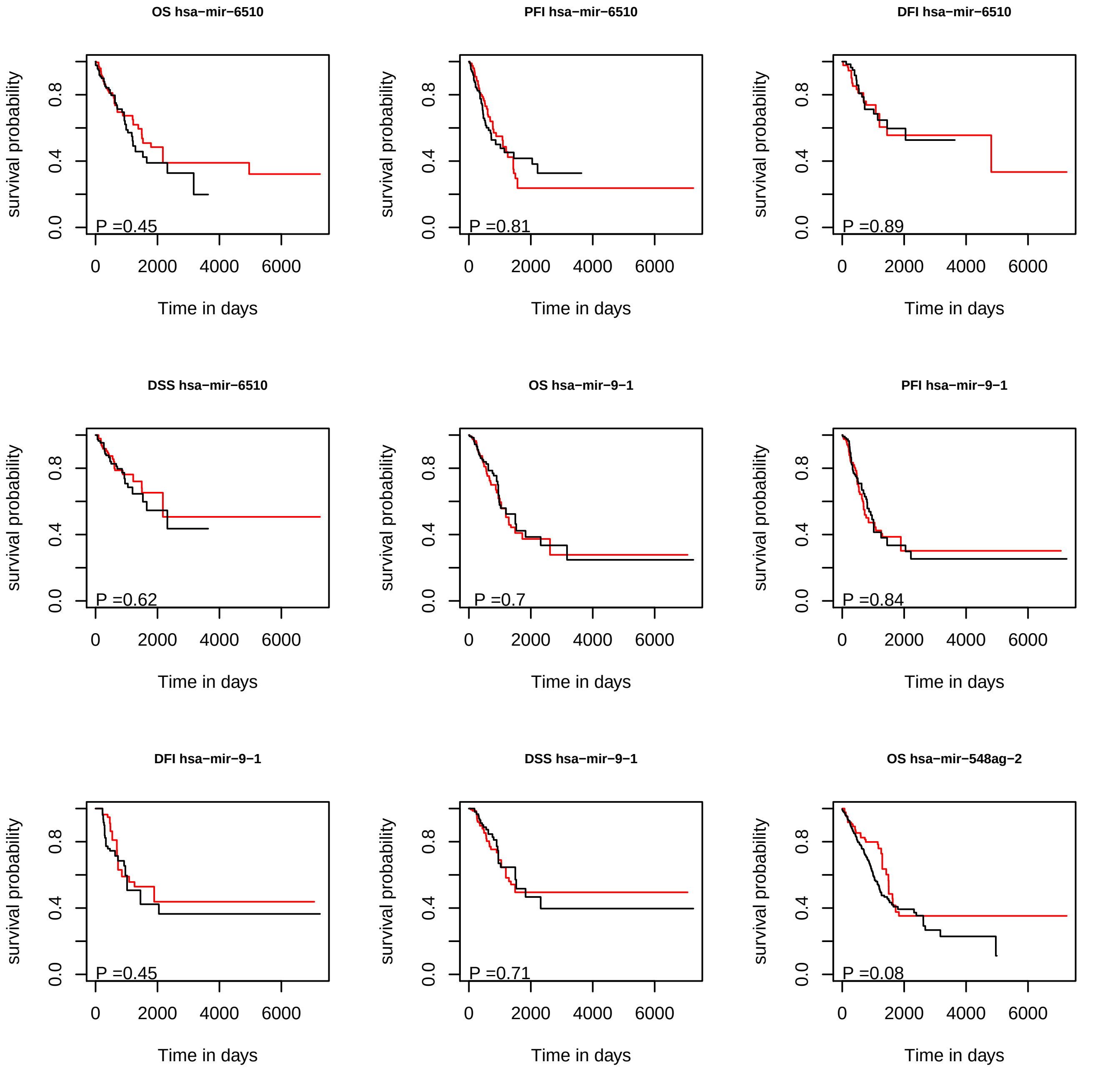
OS hsa−mir−6510
P =0.45
PFI hsa−mir−6510
P =0.81
DFI hsa−mir−6510
P =0.89
DSS hsa−mir−6510
P =0.62
OS hsa−mir−9−1
P =0.7
PFI hsa−mir−9−1
P =0.84
DFI hsa−mir−9−1
P =0.45
DSS hsa−mir−9−1
P =0.71
OS hsa−mir−548ag−2
P =0.08
survival probability
Time in days
0.0
0.4
0.8
0
2000
4000
6000

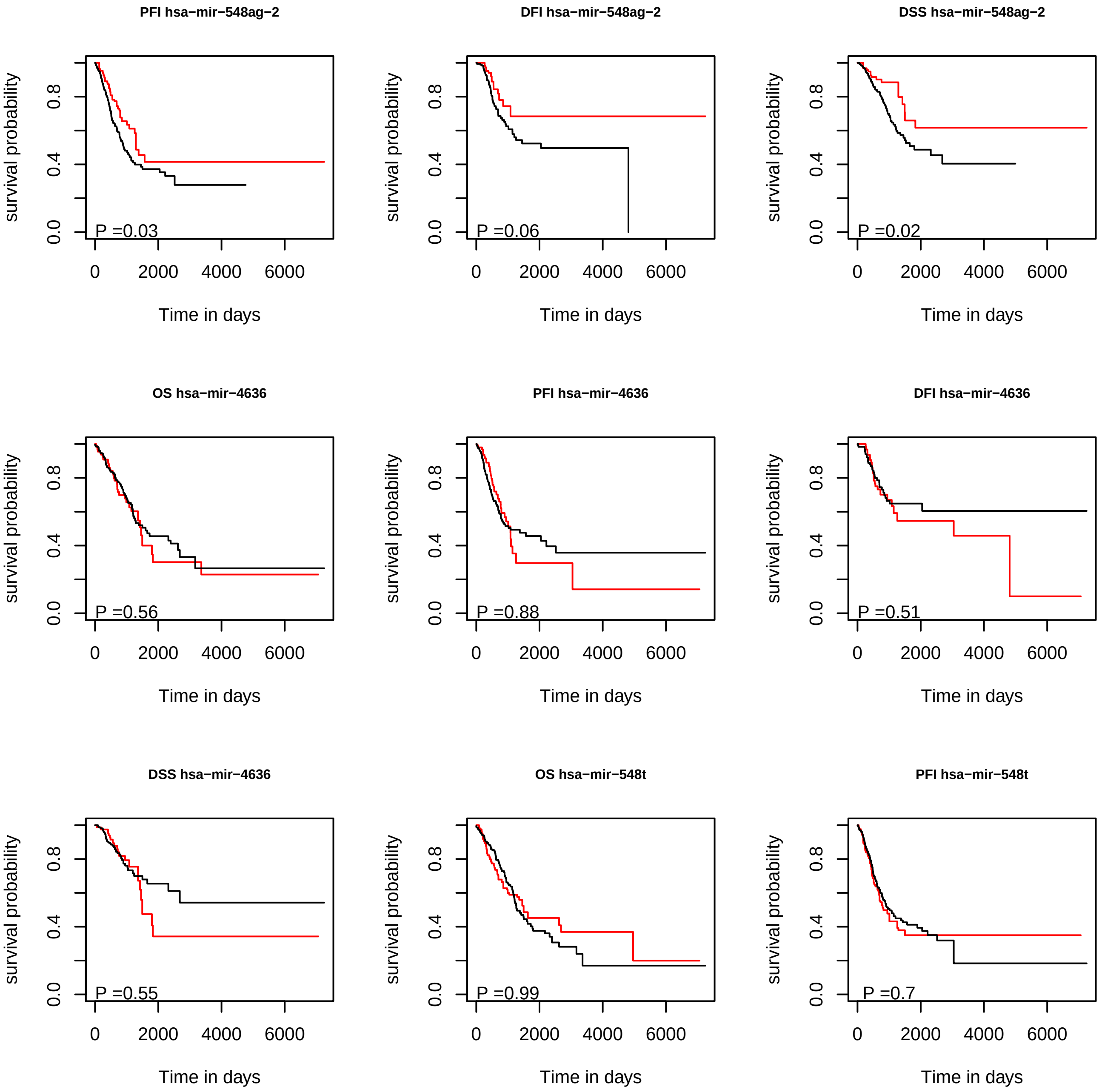
PFI hsa−mir−548ag−2
DFI hsa−mir−548ag−2
DSS hsa−mir−548ag−2
OS hsa−mir−4636
PFI hsa−mir−4636
DFI hsa−mir−4636
DSS hsa−mir−4636
OS hsa−mir−548t
PFI hsa−mir−548t
survival probability
Time in days
0.0
0.4
0.8
0
2000
4000
6000
P =0.03
P =0.06
P =0.02
P =0.56
P =0.88
P =0.51
P =0.55
P =0.99
P =0.7

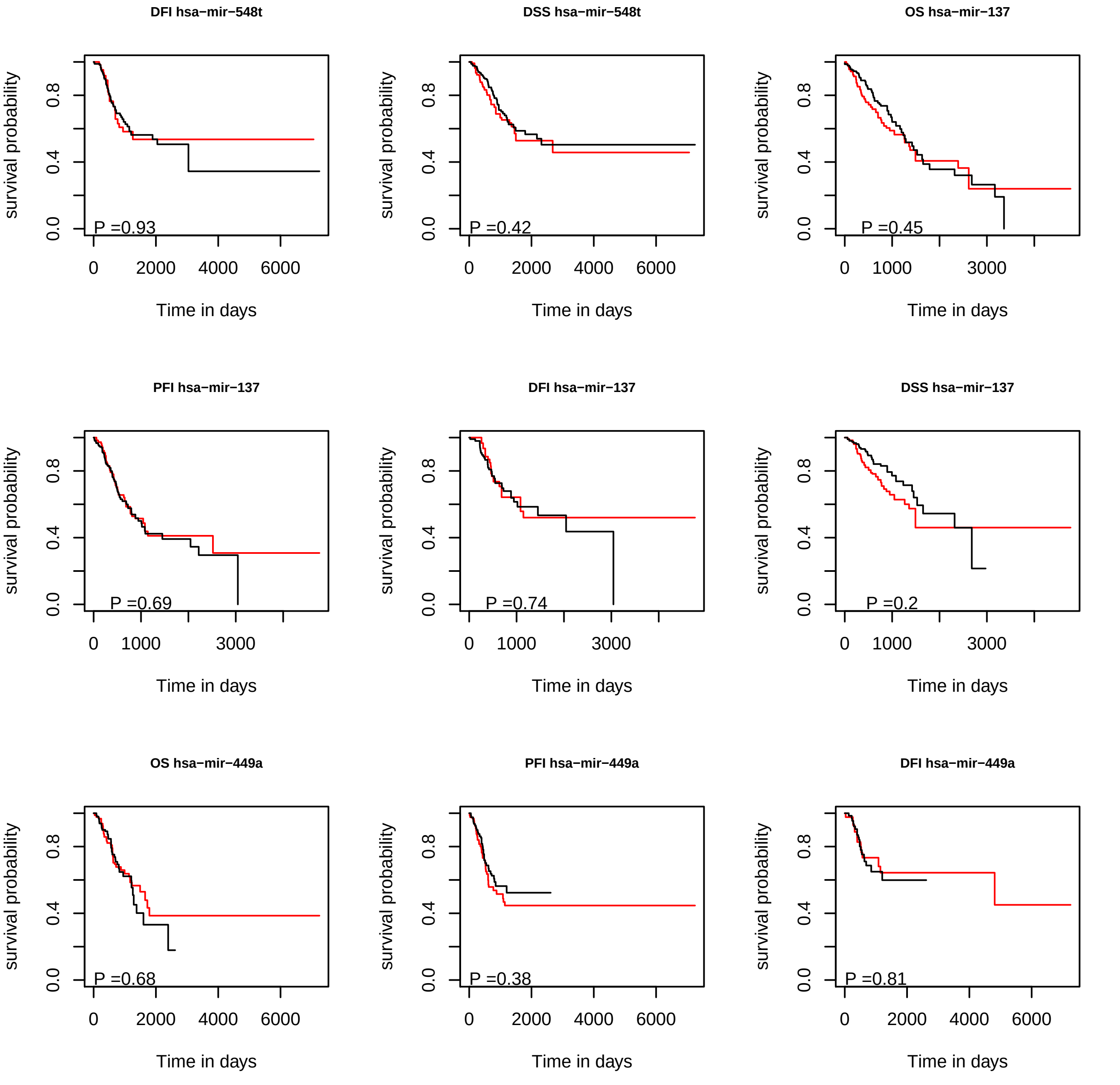
DFI hsa−mir−548t
P =0.93
DSS hsa−mir−548t
P =0.42
OS hsa−mir−137
P =0.45
PFI hsa−mir−137
P =0.69
DFI hsa−mir−137
P =0.74
DSS hsa−mir−137
P =0.2
OS hsa−mir−449a
P =0.68
PFI hsa−mir−449a
P =0.38
DFI hsa−mir−449a
P =0.81
survival probability
Time in days
0.0
0.4
0.8
0
1000
2000
3000
4000
6000

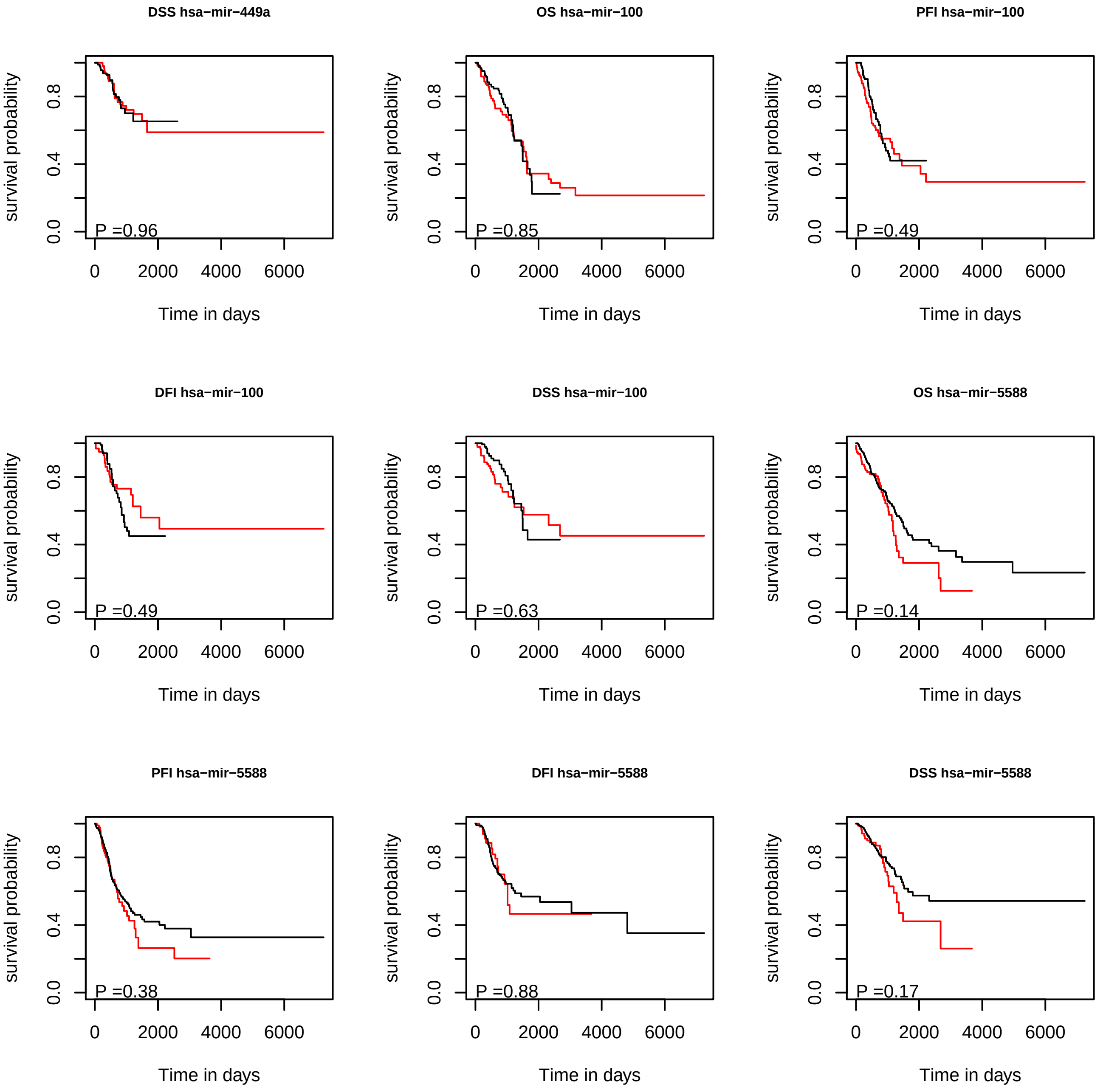
DSS hsa−mir−449a
survival probability
0.0 0.4 0.8
P =0.96
0 2000 4000 6000
Time in days
OS hsa−mir−100
survival probability
0.0 0.4 0.8
P =0.85
0 2000 4000 6000
Time in days
PFI hsa−mir−100
survival probability
0.0 0.4 0.8
P =0.49
0 2000 4000 6000
Time in days
DFI hsa−mir−100
survival probability
0.0 0.4 0.8
P =0.49
0 2000 4000 6000
Time in days
DSS hsa−mir−100
survival probability
0.0 0.4 0.8
P =0.63
0 2000 4000 6000
Time in days
OS hsa−mir−5588
survival probability
0.0 0.4 0.8
P =0.14
0 2000 4000 6000
Time in days
PFI hsa−mir−5588
survival probability
0.0 0.4 0.8
P =0.38
0 2000 4000 6000
Time in days
DFI hsa−mir−5588
survival probability
0.0 0.4 0.8
P =0.88
0 2000 4000 6000
Time in days
DSS hsa−mir−5588
survival probability
0.0 0.4 0.8
P =0.17
0 2000 4000 6000
Time in days

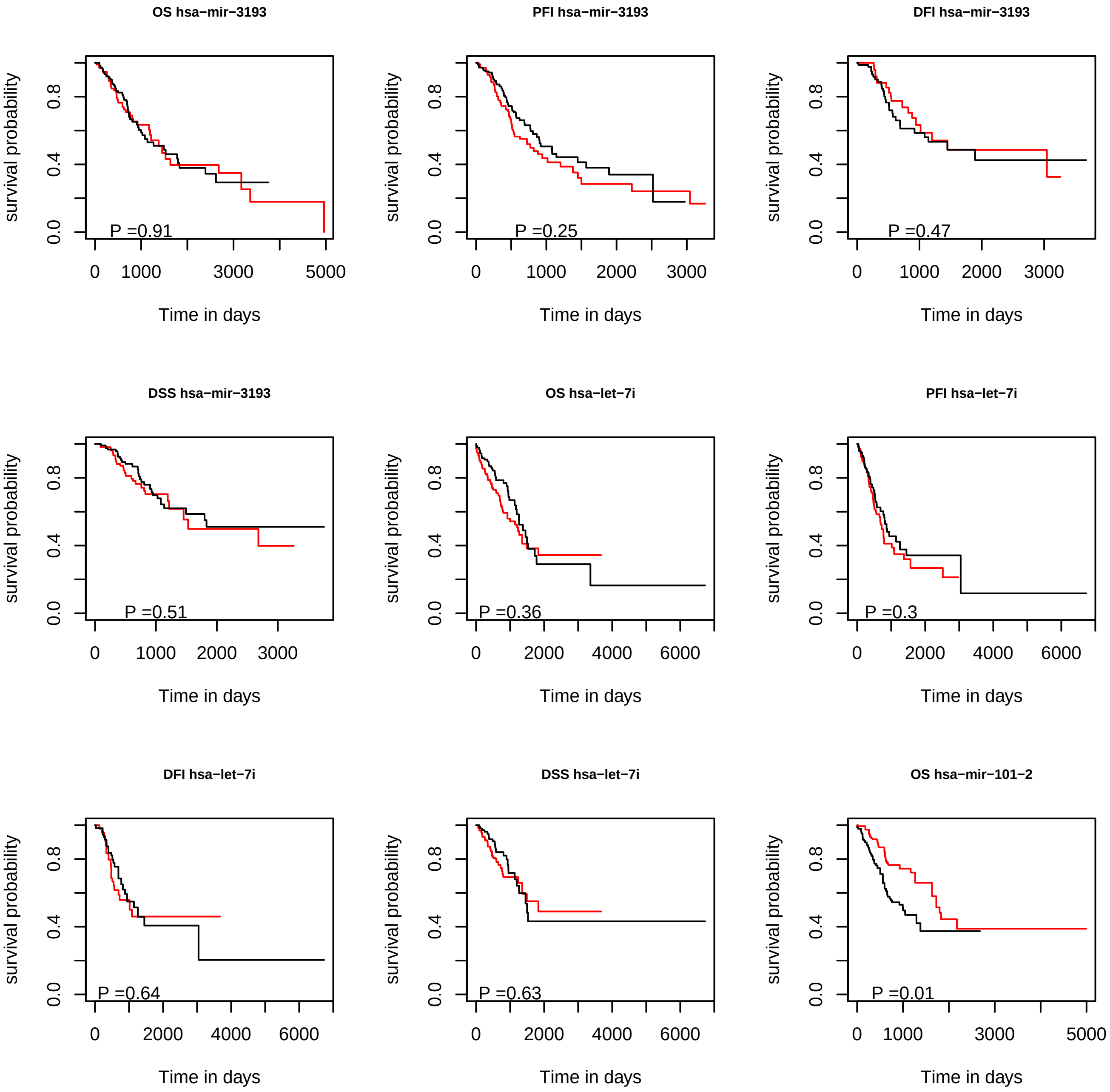
OS hsa−mir−3193
survival probability
P =0.91
Time in days
PFI hsa−mir−3193
survival probability
P =0.25
Time in days
DFI hsa−mir−3193
survival probability
P =0.47
Time in days
DSS hsa−mir−3193
survival probability
P =0.51
Time in days
OS hsa−let−7i
survival probability
P =0.36
Time in days
PFI hsa−let−7i
survival probability
P =0.3
Time in days
DFI hsa−let−7i
survival probability
P =0.64
Time in days
DSS hsa−let−7i
survival probability
P =0.63
Time in days
OS hsa−mir−101−2
survival probability
P =0.01
Time in days

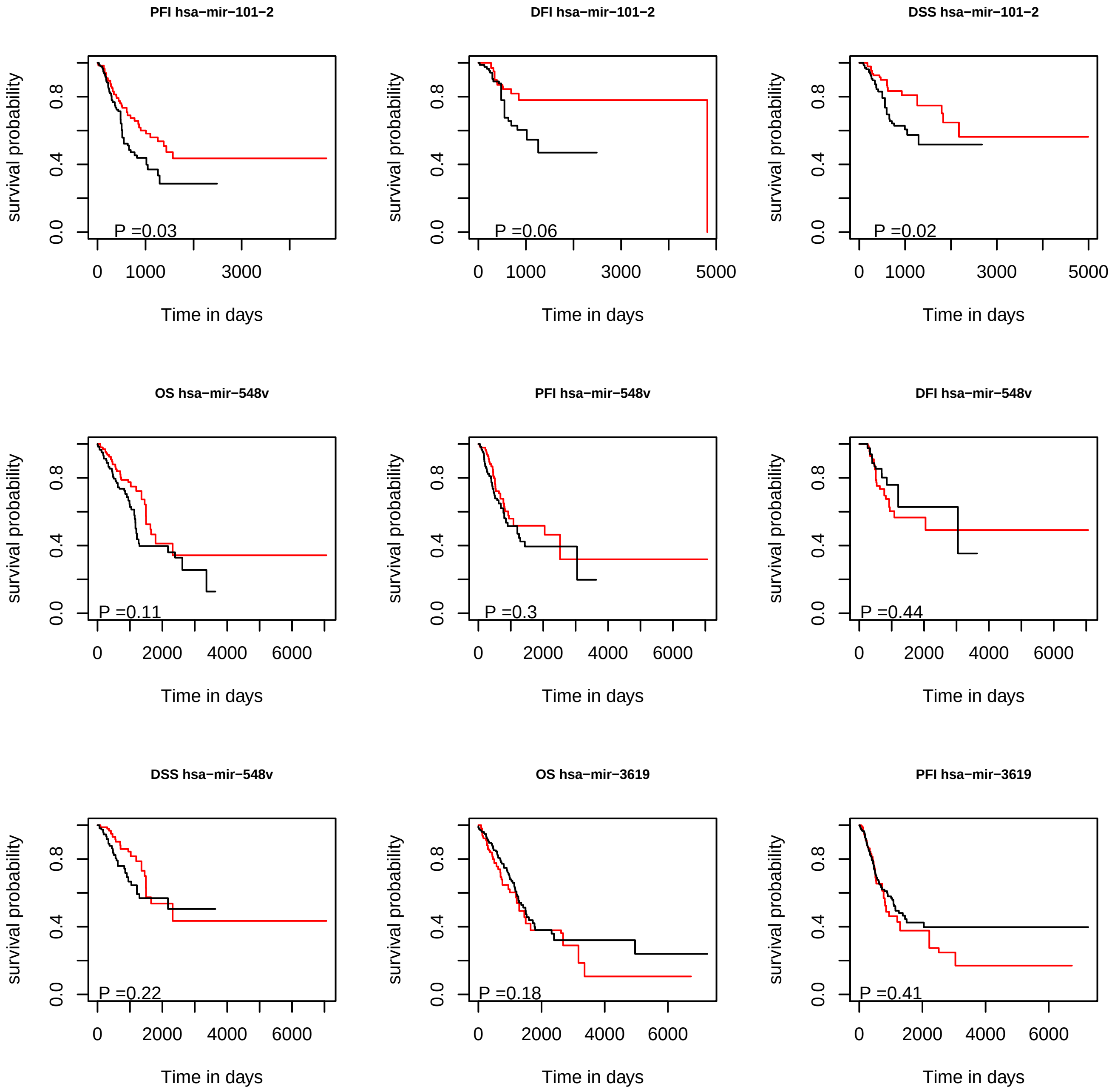
PFI hsa−mir−101−2
survival probability
0.0 0.4 0.8
P =0.03
0 1000 3000
Time in days
DFI hsa−mir−101−2
survival probability
0.0 0.4 0.8
P =0.06
0 1000 3000 5000
Time in days
DSS hsa−mir−101−2
survival probability
0.0 0.4 0.8
P =0.02
0 1000 3000 5000
Time in days
OS hsa−mir−548v
survival probability
0.0 0.4 0.8
P =0.11
0 2000 4000 6000
Time in days
PFI hsa−mir−548v
survival probability
0.0 0.4 0.8
P =0.3
0 2000 4000 6000
Time in days
DFI hsa−mir−548v
survival probability
0.0 0.4 0.8
P =0.44
0 2000 4000 6000
Time in days
DSS hsa−mir−548v
survival probability
0.0 0.4 0.8
P =0.22
0 2000 4000 6000
Time in days
OS hsa−mir−3619
survival probability
0.0 0.4 0.8
P =0.18
0 2000 4000 6000
Time in days
PFI hsa−mir−3619
survival probability
0.0 0.4 0.8
P =0.41
0 2000 4000 6000
Time in days

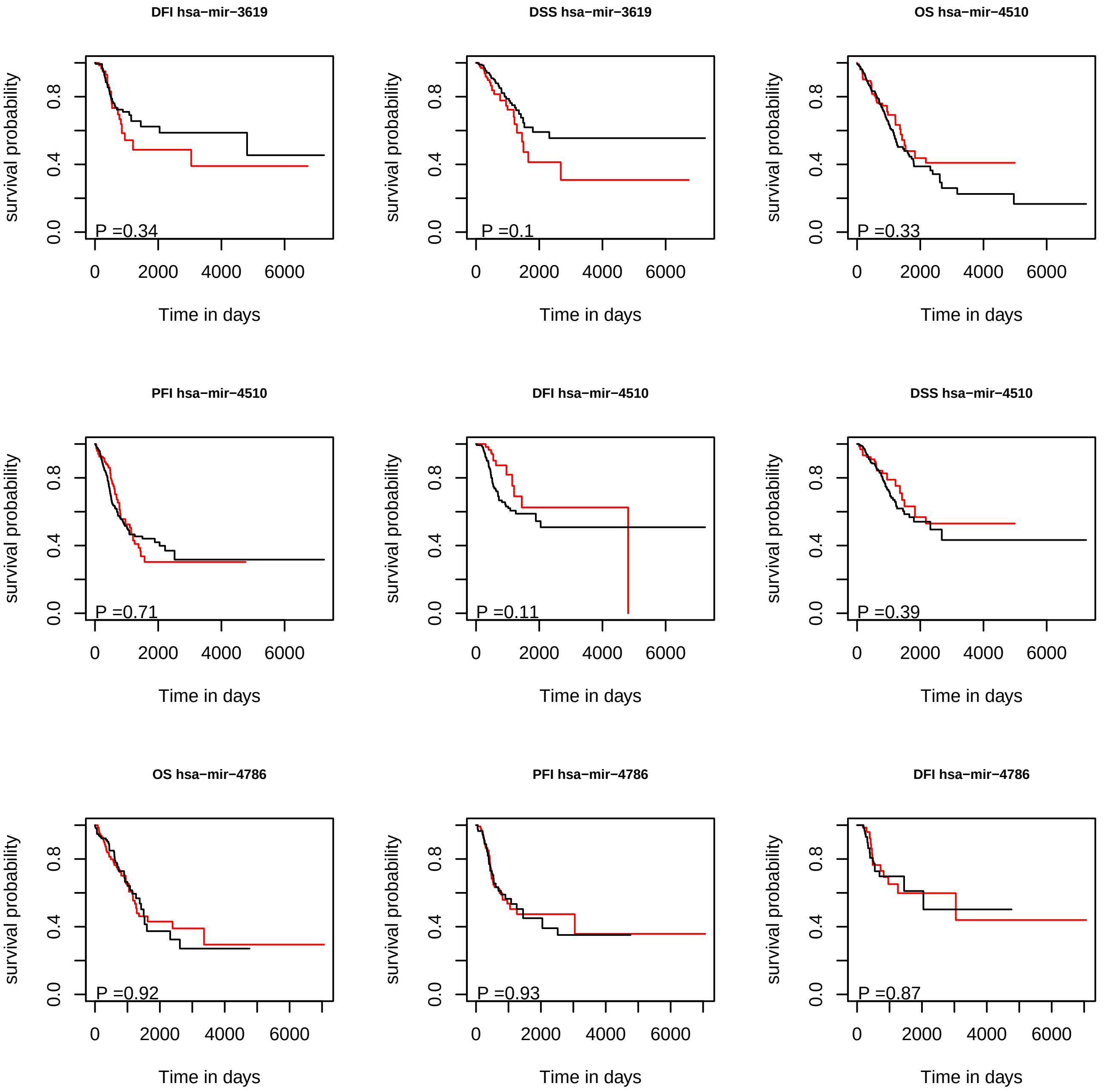
DFI hsa−mir−3619
P =0.34
DSS hsa−mir−3619
P =0.1
OS hsa−mir−4510
P =0.33
PFI hsa−mir−4510
P =0.71
DFI hsa−mir−4510
P =0.11
DSS hsa−mir−4510
P =0.39
OS hsa−mir−4786
P =0.92
PFI hsa−mir−4786
P =0.93
DFI hsa−mir−4786
P =0.87
survival probability
0.0
0.4
0.8
0
2000
4000
6000
Time in days

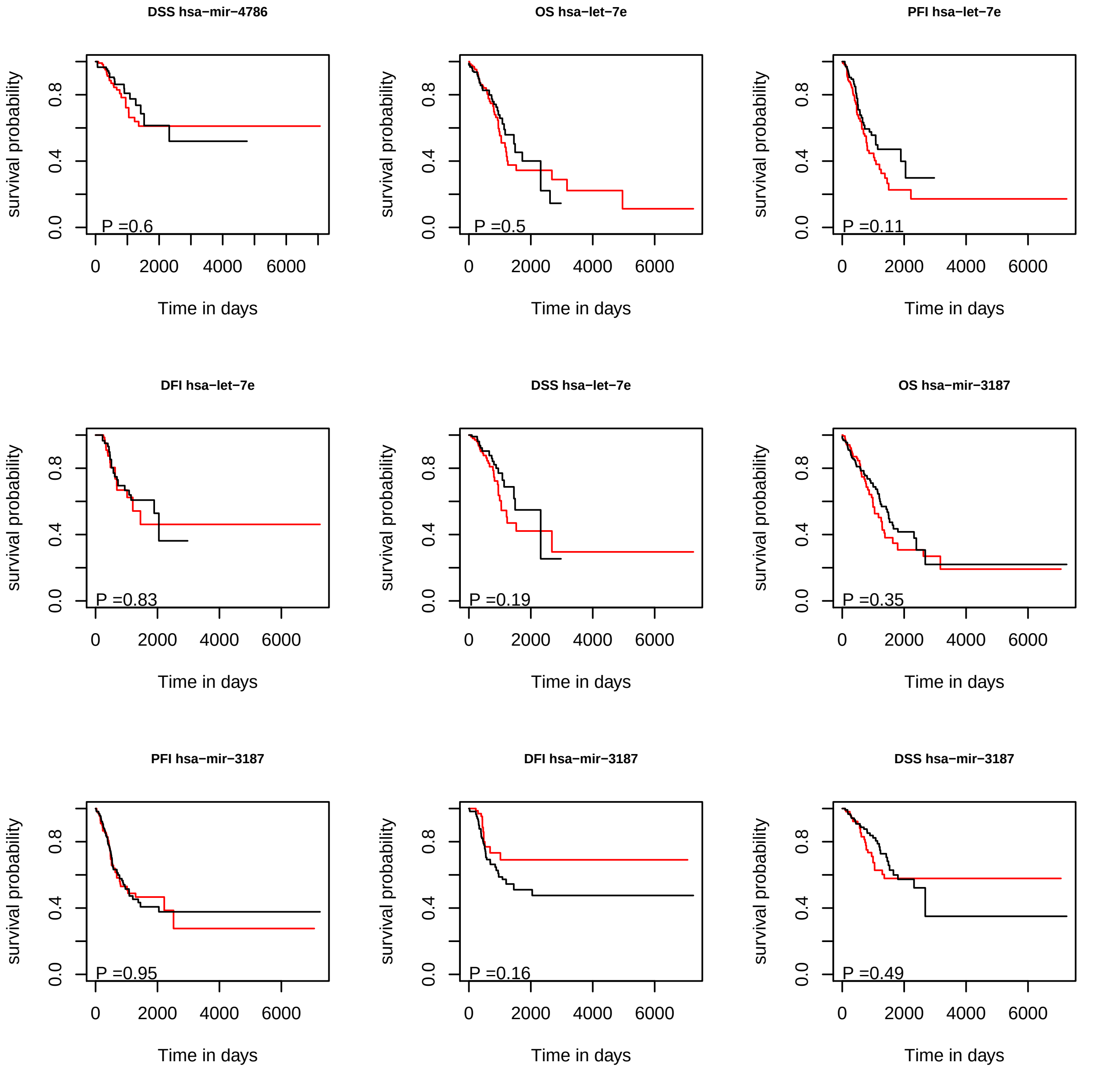
DSS hsa−mir−4786
P =0.6
OS hsa−let−7e
P =0.5
PFI hsa−let−7e
P =0.11
DFI hsa−let−7e
P =0.83
DSS hsa−let−7e
P =0.19
OS hsa−mir−3187
P =0.35
PFI hsa−mir−3187
P =0.95
DFI hsa−mir−3187
P =0.16
DSS hsa−mir−3187
P =0.49
survival probability
0.0
0.4
0.8
Time in days
0
2000
4000
6000

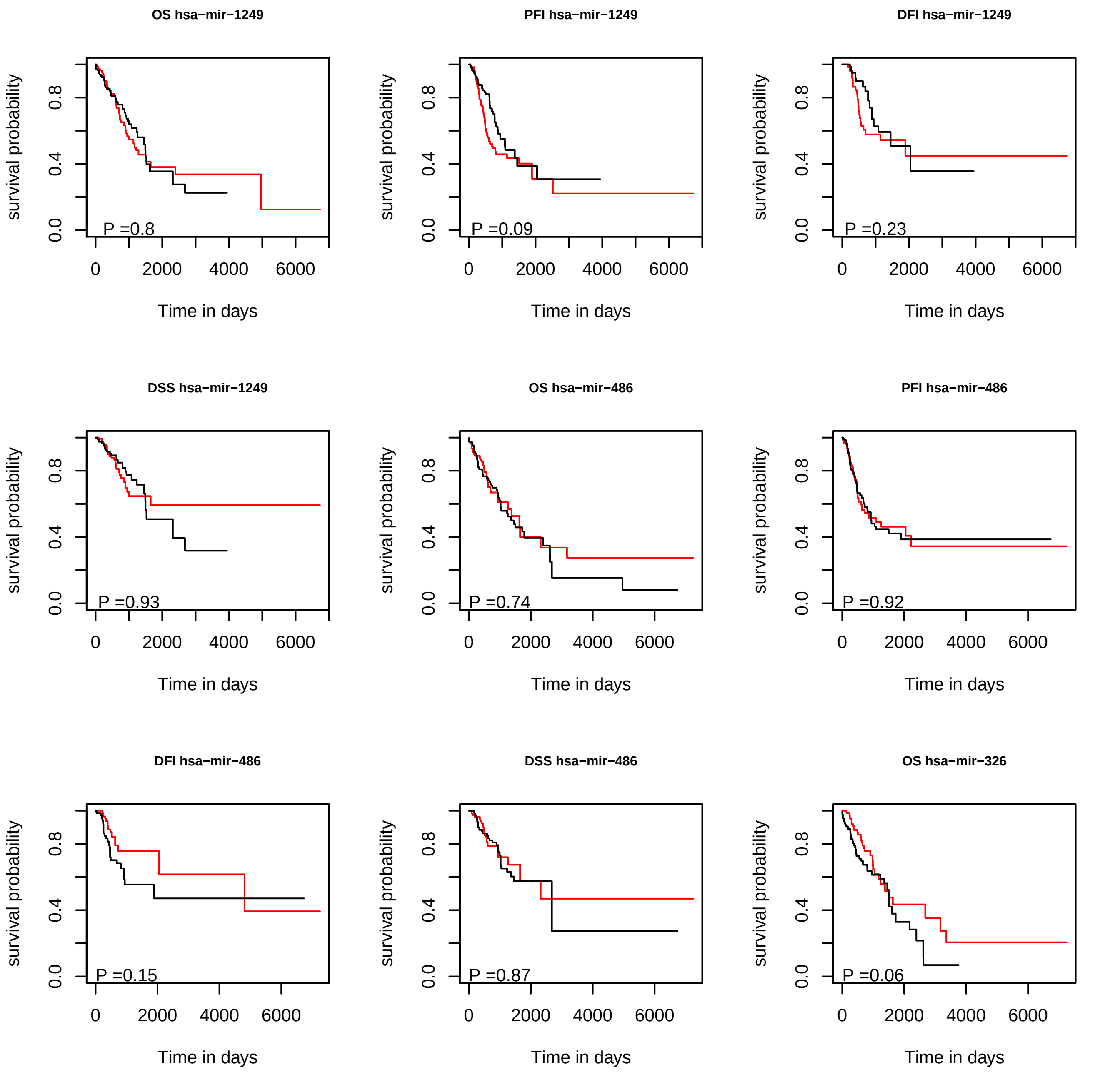
OS hsa−mir−1249
P =0.8
PFI hsa−mir−1249
P =0.09
DFI hsa−mir−1249
P =0.23
DSS hsa−mir−1249
P =0.93
OS hsa−mir−486
P =0.74
PFI hsa−mir−486
P =0.92
DFI hsa−mir−486
P =0.15
DSS hsa−mir−486
P =0.87
OS hsa−mir−326
P =0.06
survival probability
0.0
0.4
0.8
0
2000
4000
6000
Time in days

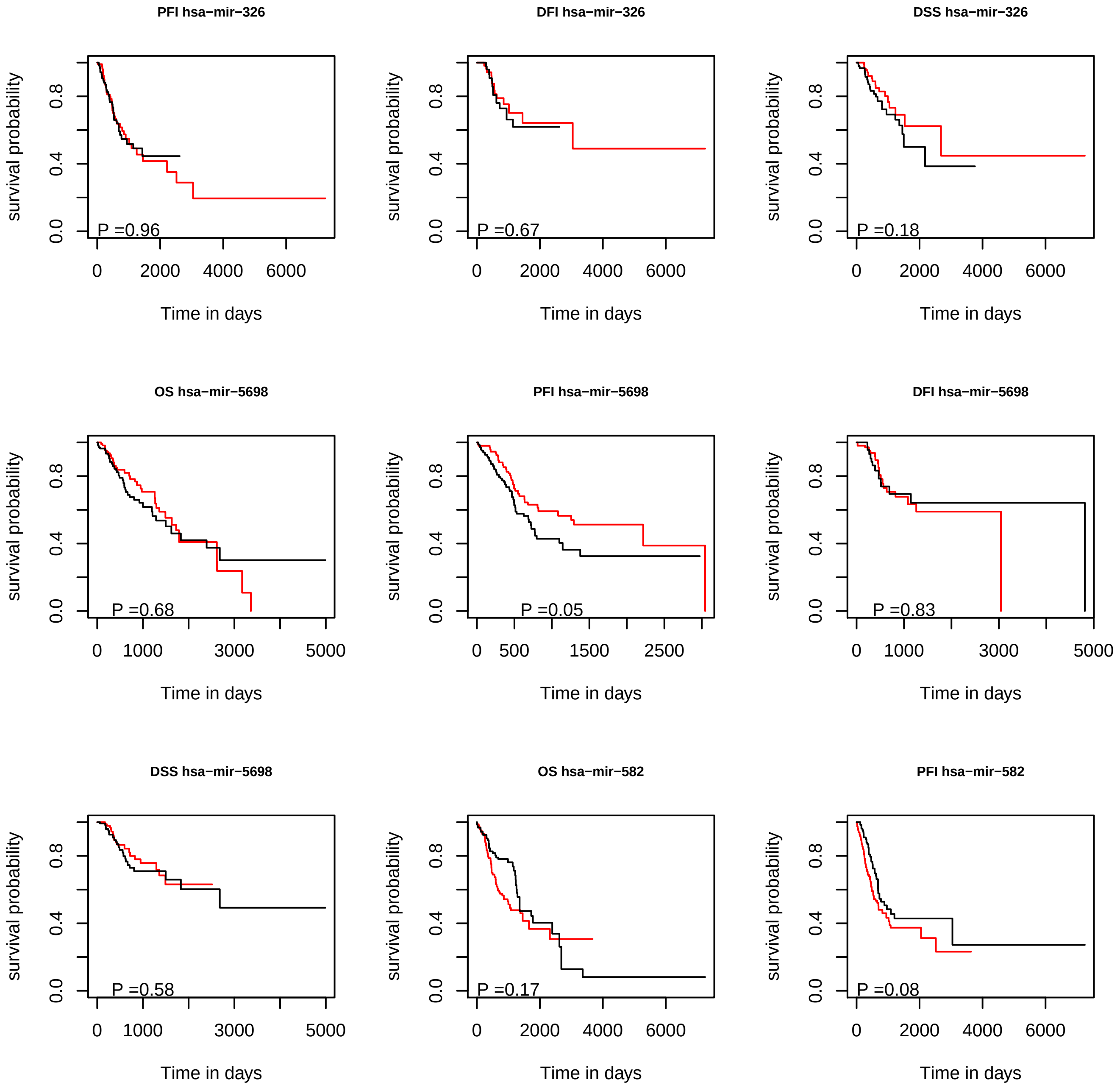
PFI hsa−mir−326
survival probability
0.0
0.4
0.8
P =0.96
0
2000
4000
6000
Time in days
DFI hsa−mir−326
survival probability
0.0
0.4
0.8
P =0.67
0
2000
4000
6000
Time in days
DSS hsa−mir−326
survival probability
0.0
0.4
0.8
P =0.18
0
2000
4000
6000
Time in days
OS hsa−mir−5698
survival probability
0.0
0.4
0.8
P =0.68
0
1000
3000
5000
Time in days
PFI hsa−mir−5698
survival probability
0.0
0.4
0.8
P =0.05
0
500
1500
2500
Time in days
DFI hsa−mir−5698
survival probability
0.0
0.4
0.8
P =0.83
0
1000
3000
5000
Time in days
DSS hsa−mir−5698
survival probability
0.0
0.4
0.8
P =0.58
0
1000
3000
5000
Time in days
OS hsa−mir−582
survival probability
0.0
0.4
0.8
P =0.17
0
2000
4000
6000
Time in days
PFI hsa−mir−582
survival probability
0.0
0.4
0.8
P =0.08
0
2000
4000
6000
Time in days

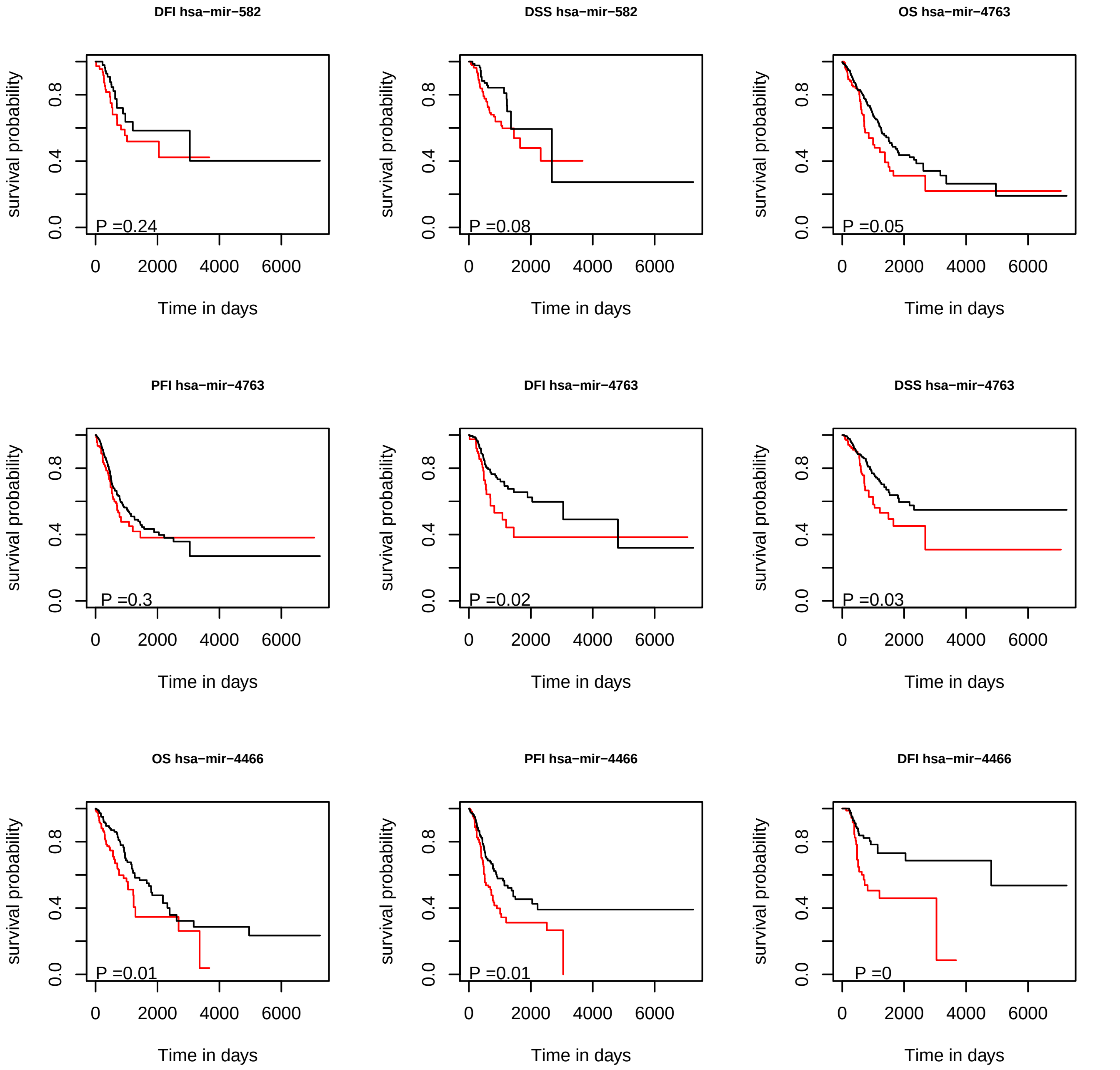
DFI hsa−mir−582
P =0.24
DSS hsa−mir−582
P =0.08
OS hsa−mir−4763
P =0.05
PFI hsa−mir−4763
P =0.3
DFI hsa−mir−4763
P =0.02
DSS hsa−mir−4763
P =0.03
OS hsa−mir−4466
P =0.01
PFI hsa−mir−4466
P =0.01
DFI hsa−mir−4466
P =0
survival probability
0.0
0.4
0.8
0
2000
4000
6000
Time in days

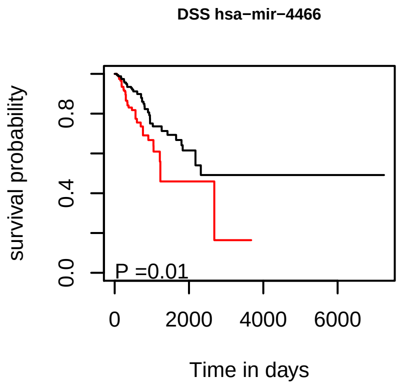
DSS hsa−mir−4466
survival probability
0.0
0.4
0.8
P =0.01
0
2000
4000
6000
Time in days
