# Supplementary material for: Computing microRNA-gene interaction networks in pan-cancer using miRDriver
Source: Sci Rep. 2022 Mar 8;12:3717. doi: 10.1038/s41598-022-07628-z (PMC8904490; doi:10.1038/s41598-022-07628-z)
Supplement: Supplementary file 22 — Supplementary Information 22. [file 41598_2022_7628_MOESM22_ESM.pdf]

Computing microRNA-gene interaction networks in pan-cancer using miRDriver

Banabithi Bose, Matthew Moravec, and Serdar Bozdag

Supplemental Figure S13

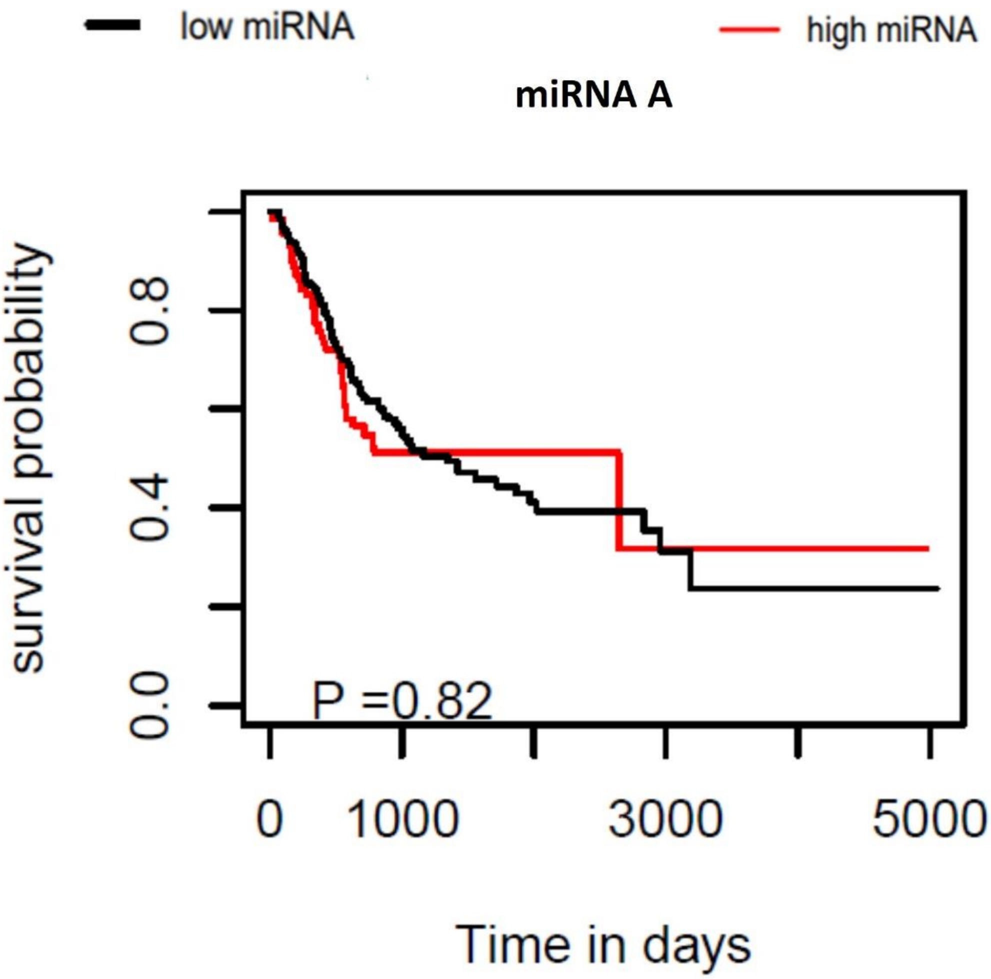

The *Adjusted Kaplan-Meier* survival plots for the computed miRNAs in high and low miRNA expression patient groups.

Supplemental Figure S13

Cancer Type: LUSC

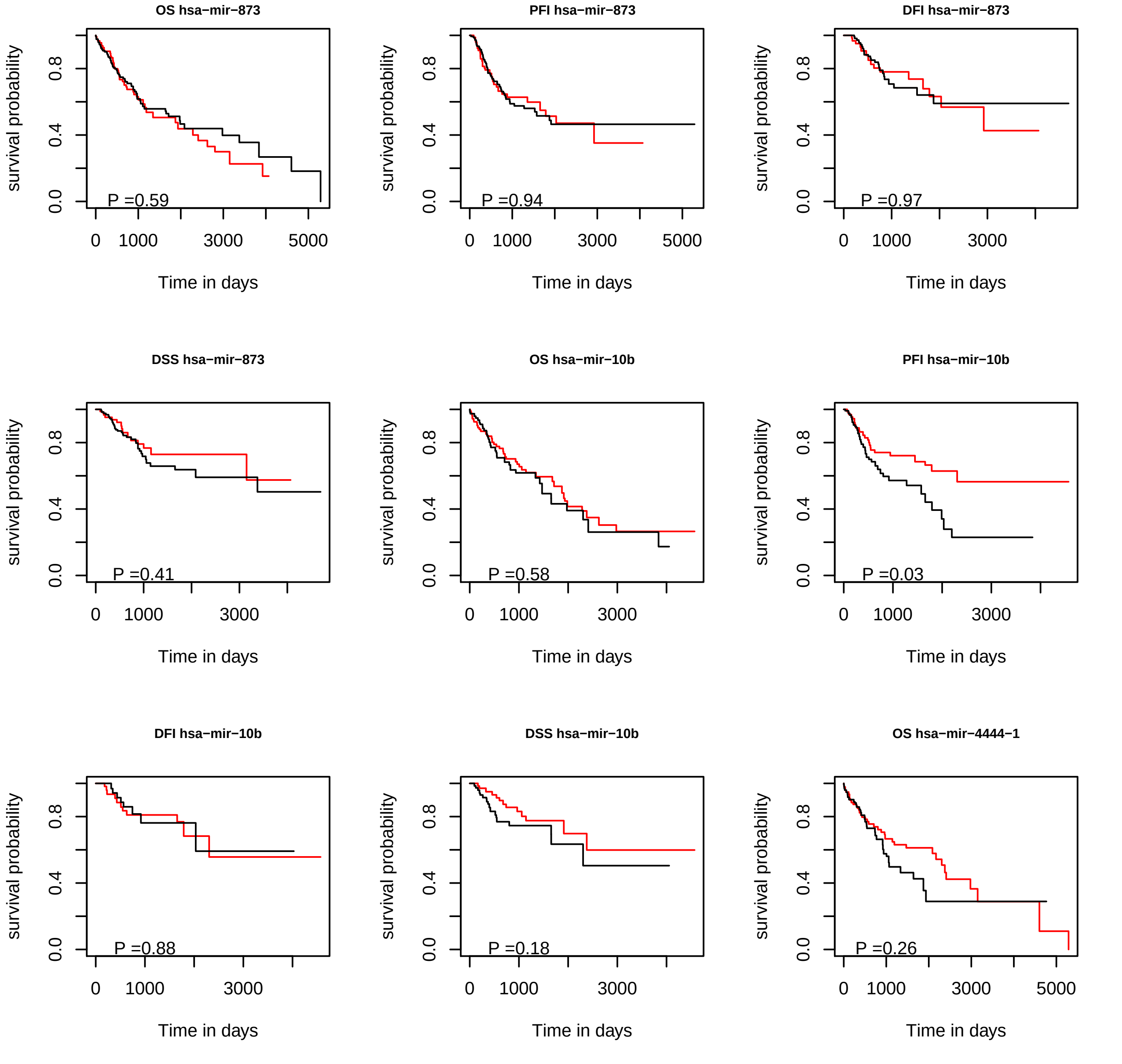

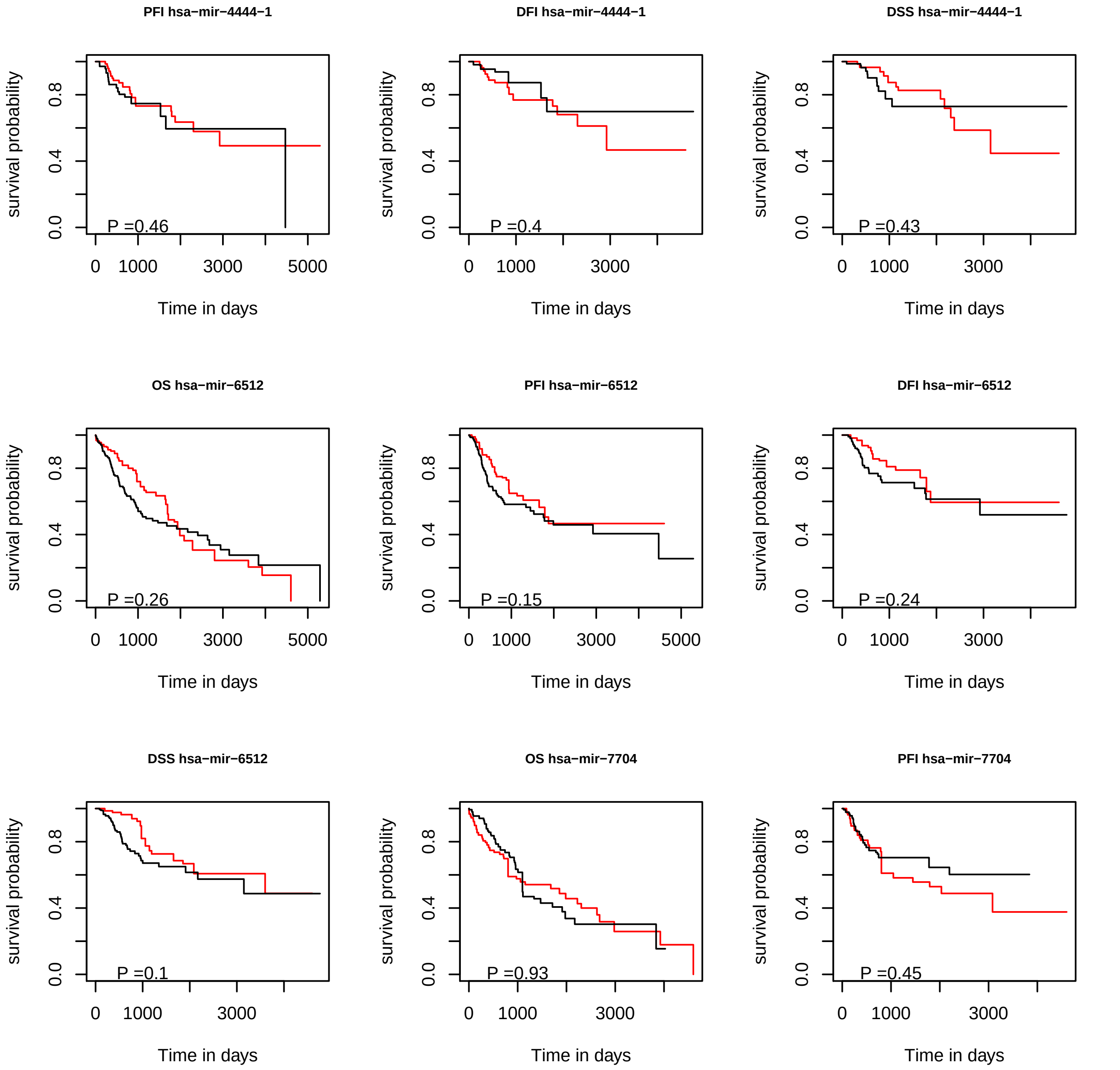
PFI hsa−mir−4444−1
P =0.46
DFI hsa−mir−4444−1
P =0.4
DSS hsa−mir−4444−1
P =0.43
OS hsa−mir−6512
P =0.26
PFI hsa−mir−6512
P =0.15
DFI hsa−mir−6512
P =0.24
DSS hsa−mir−6512
P =0.1
OS hsa−mir−7704
P =0.93
PFI hsa−mir−7704
P =0.45
survival probability
Time in days
0.0
0.4
0.8
0
1000
3000
5000

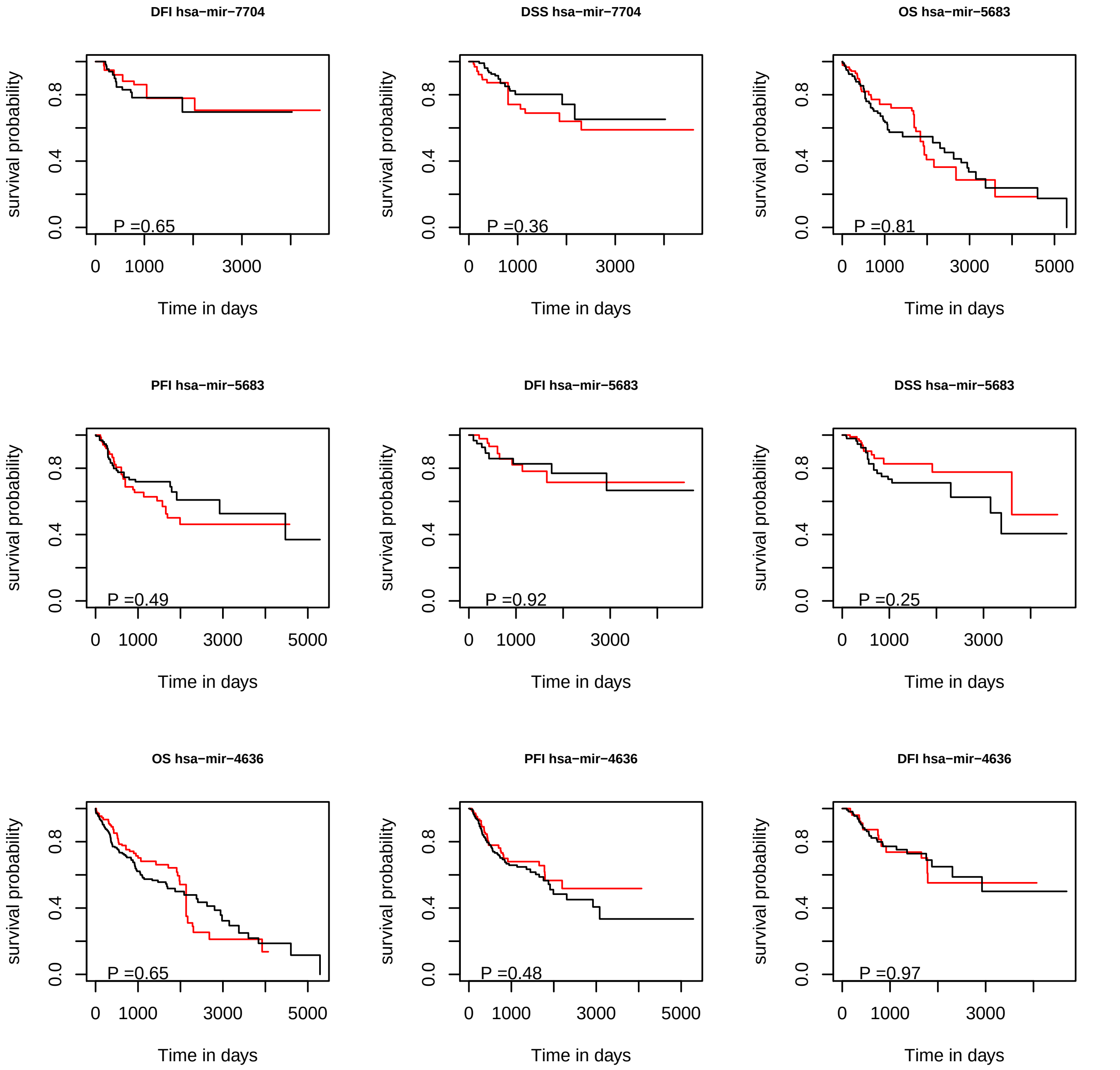
DFI hsa−mir−7704
survival probability
0.0
0.4
0.8
P =0.65
0
1000
3000
Time in days
DSS hsa−mir−7704
survival probability
0.0
0.4
0.8
P =0.36
0
1000
3000
Time in days
OS hsa−mir−5683
survival probability
0.0
0.4
0.8
P =0.81
0
1000
3000
5000
Time in days
PFI hsa−mir−5683
survival probability
0.0
0.4
0.8
P =0.49
0
1000
3000
5000
Time in days
DFI hsa−mir−5683
survival probability
0.0
0.4
0.8
P =0.92
0
1000
3000
Time in days
DSS hsa−mir−5683
survival probability
0.0
0.4
0.8
P =0.25
0
1000
3000
Time in days
OS hsa−mir−4636
survival probability
0.0
0.4
0.8
P =0.65
0
1000
3000
5000
Time in days
PFI hsa−mir−4636
survival probability
0.0
0.4
0.8
P =0.48
0
1000
3000
5000
Time in days
DFI hsa−mir−4636
survival probability
0.0
0.4
0.8
P =0.97
0
1000
3000
Time in days

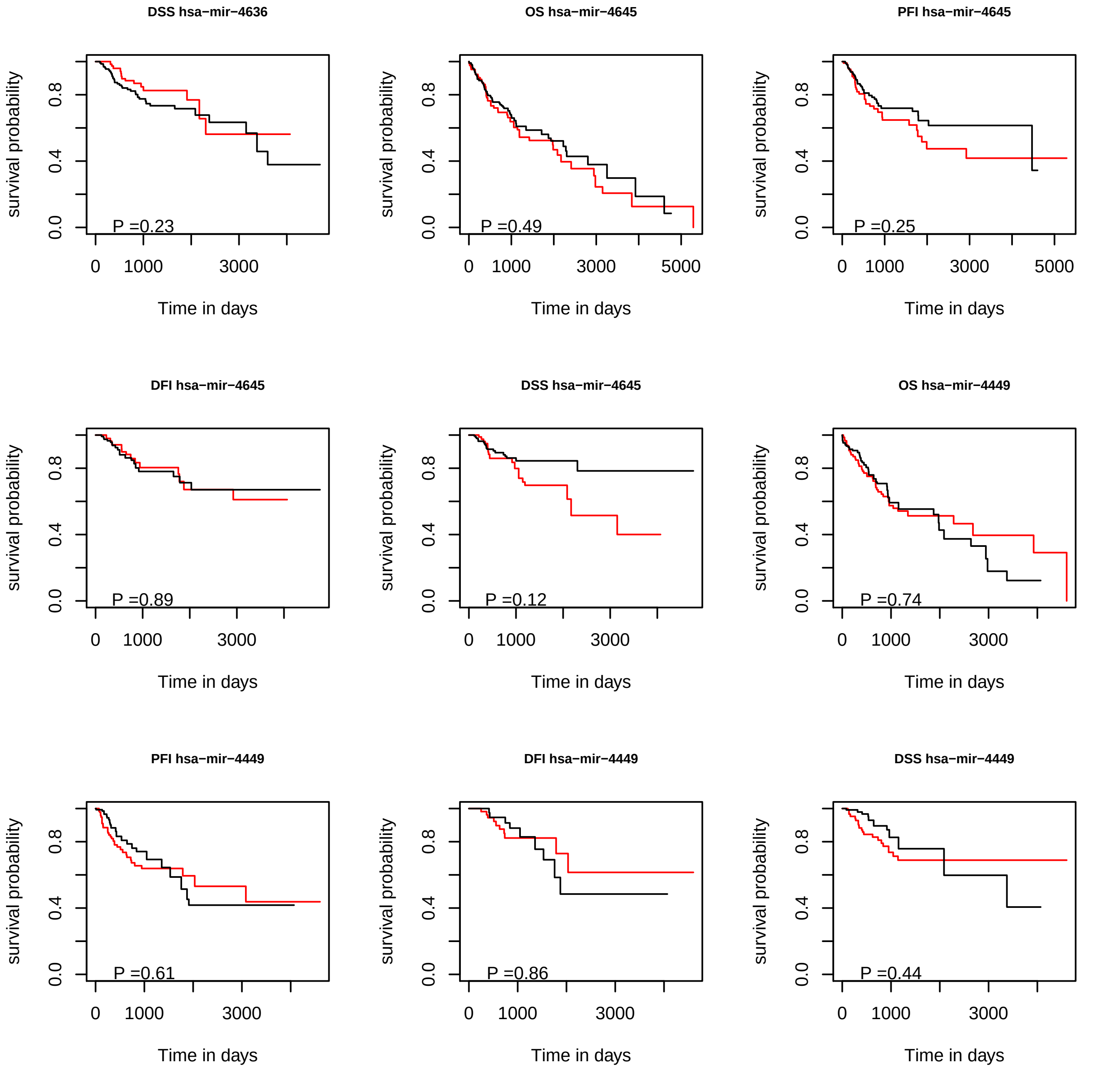
DSS hsa−mir−4636
P =0.23
OS hsa−mir−4645
P =0.49
PFI hsa−mir−4645
P =0.25
DFI hsa−mir−4645
P =0.89
DSS hsa−mir−4645
P =0.12
OS hsa−mir−4449
P =0.74
PFI hsa−mir−4449
P =0.61
DFI hsa−mir−4449
P =0.86
DSS hsa−mir−4449
P =0.44
survival probability
Time in days

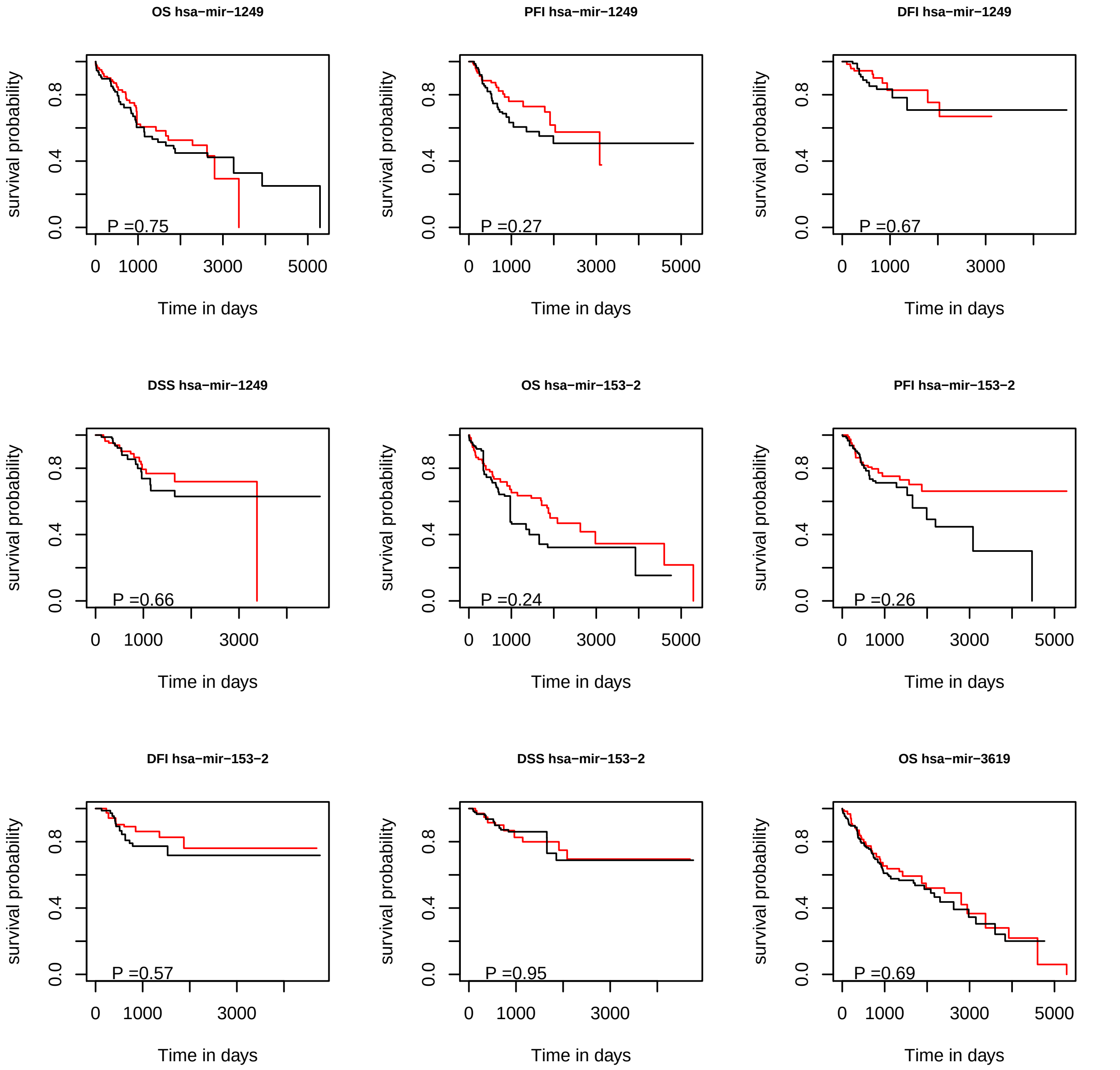
OS hsa−mir−1249
P =0.75
PFI hsa−mir−1249
P =0.27
DFI hsa−mir−1249
P =0.67
DSS hsa−mir−1249
P =0.66
OS hsa−mir−153−2
P =0.24
PFI hsa−mir−153−2
P =0.26
DFI hsa−mir−153−2
P =0.57
DSS hsa−mir−153−2
P =0.95
OS hsa−mir−3619
P =0.69
survival probability
0.0
0.4
0.8
Time in days
0
1000
3000
5000

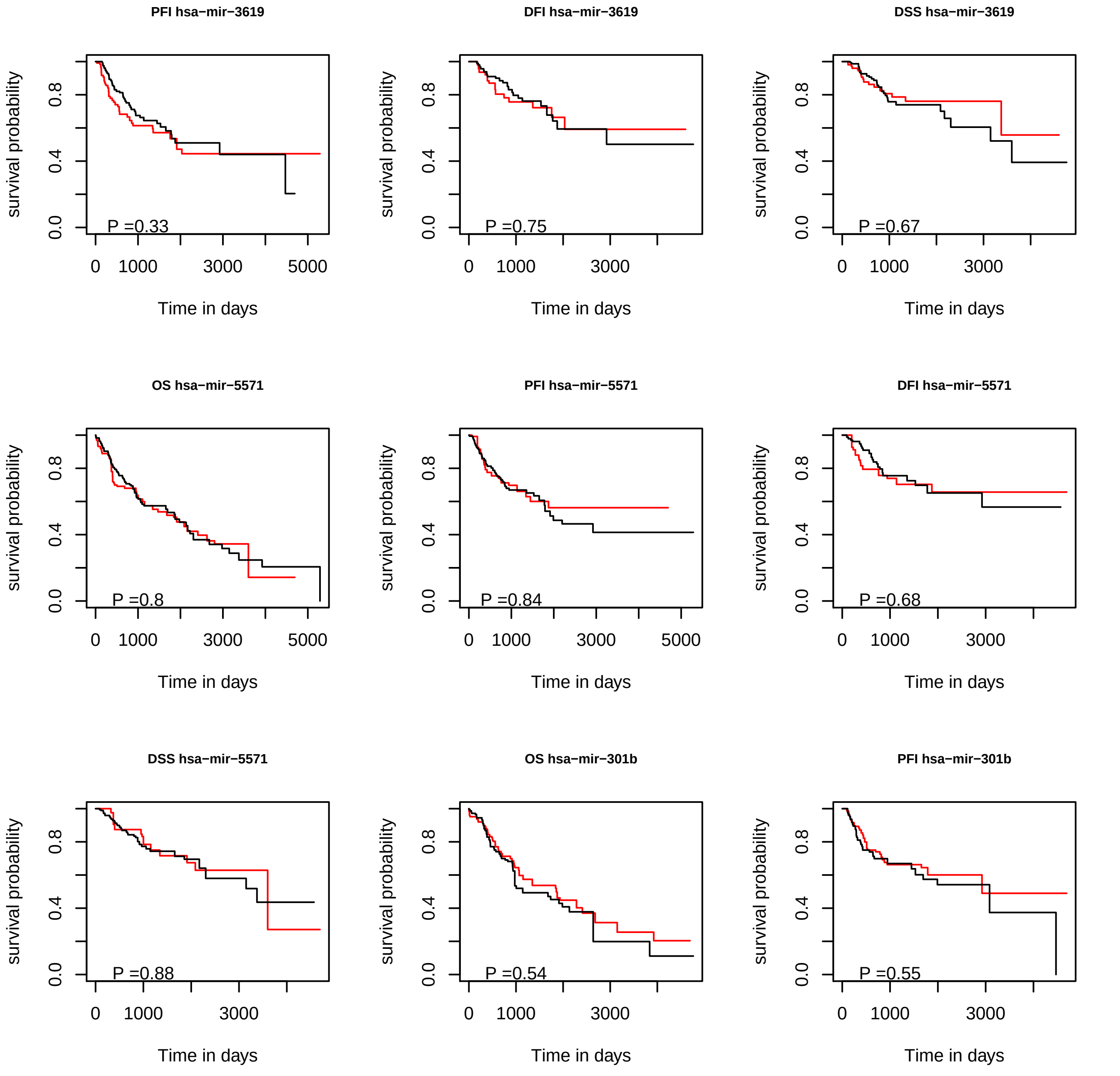
PFI hsa−mir−3619
survival probability
0.0 0.4 0.8
P =0.33
0 1000 3000 5000
Time in days
DFI hsa−mir−3619
survival probability
0.0 0.4 0.8
P =0.75
0 1000 3000
Time in days
DSS hsa−mir−3619
survival probability
0.0 0.4 0.8
P =0.67
0 1000 3000
Time in days
OS hsa−mir−5571
survival probability
0.0 0.4 0.8
P =0.8
0 1000 3000 5000
Time in days
PFI hsa−mir−5571
survival probability
0.0 0.4 0.8
P =0.84
0 1000 3000 5000
Time in days
DFI hsa−mir−5571
survival probability
0.0 0.4 0.8
P =0.68
0 1000 3000
Time in days
DSS hsa−mir−5571
survival probability
0.0 0.4 0.8
P =0.88
0 1000 3000
Time in days
OS hsa−mir−301b
survival probability
0.0 0.4 0.8
P =0.54
0 1000 3000
Time in days
PFI hsa−mir−301b
survival probability
0.0 0.4 0.8
P =0.55
0 1000 3000
Time in days

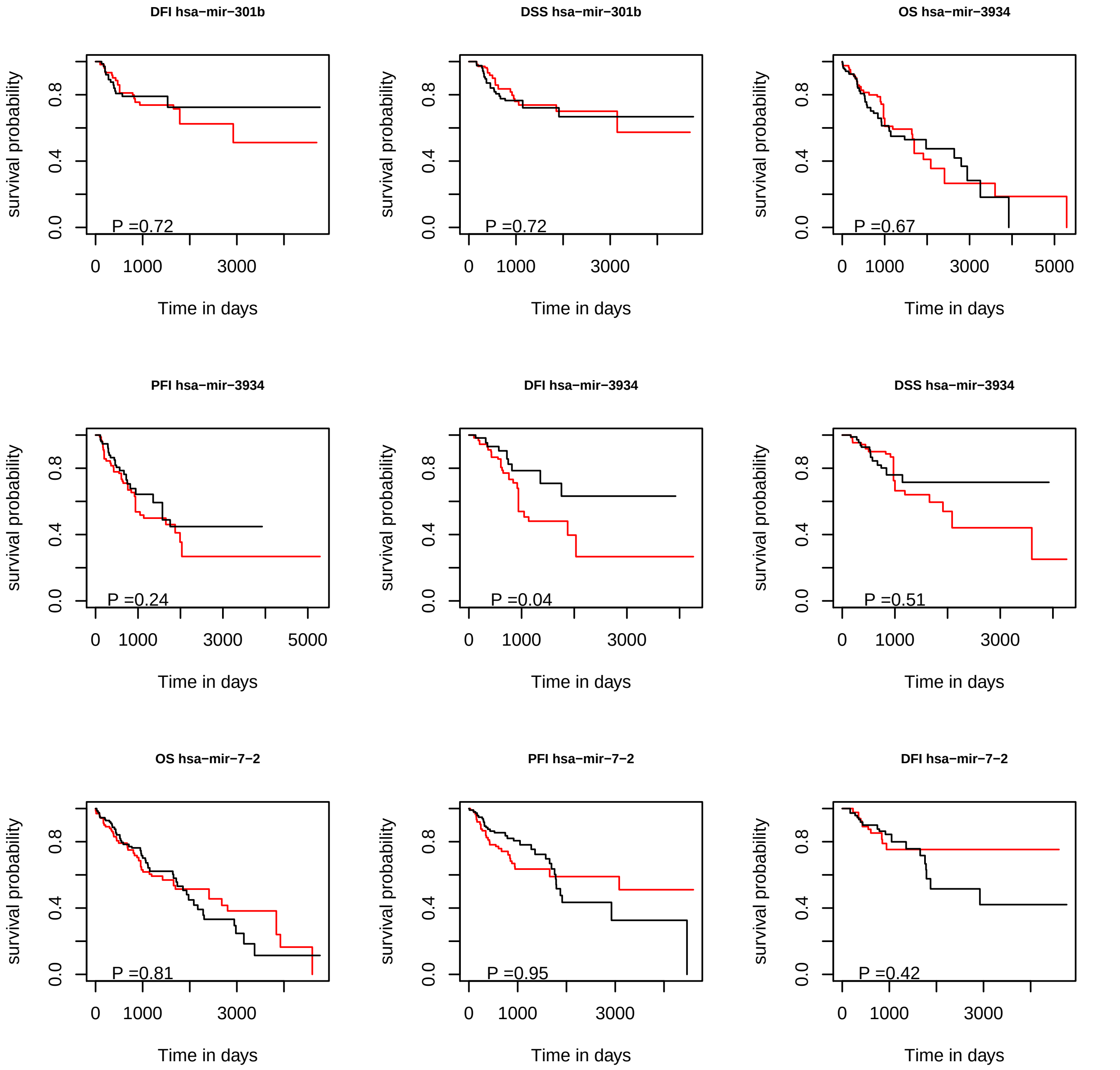
DFI hsa−mir−301b
P =0.72
DSS hsa−mir−301b
P =0.72
OS hsa−mir−3934
P =0.67
PFI hsa−mir−3934
P =0.24
DFI hsa−mir−3934
P =0.04
DSS hsa−mir−3934
P =0.51
OS hsa−mir−7−2
P =0.81
PFI hsa−mir−7−2
P =0.95
DFI hsa−mir−7−2
P =0.42
survival probability
Time in days
0.0
0.4
0.8
0
1000
3000
5000

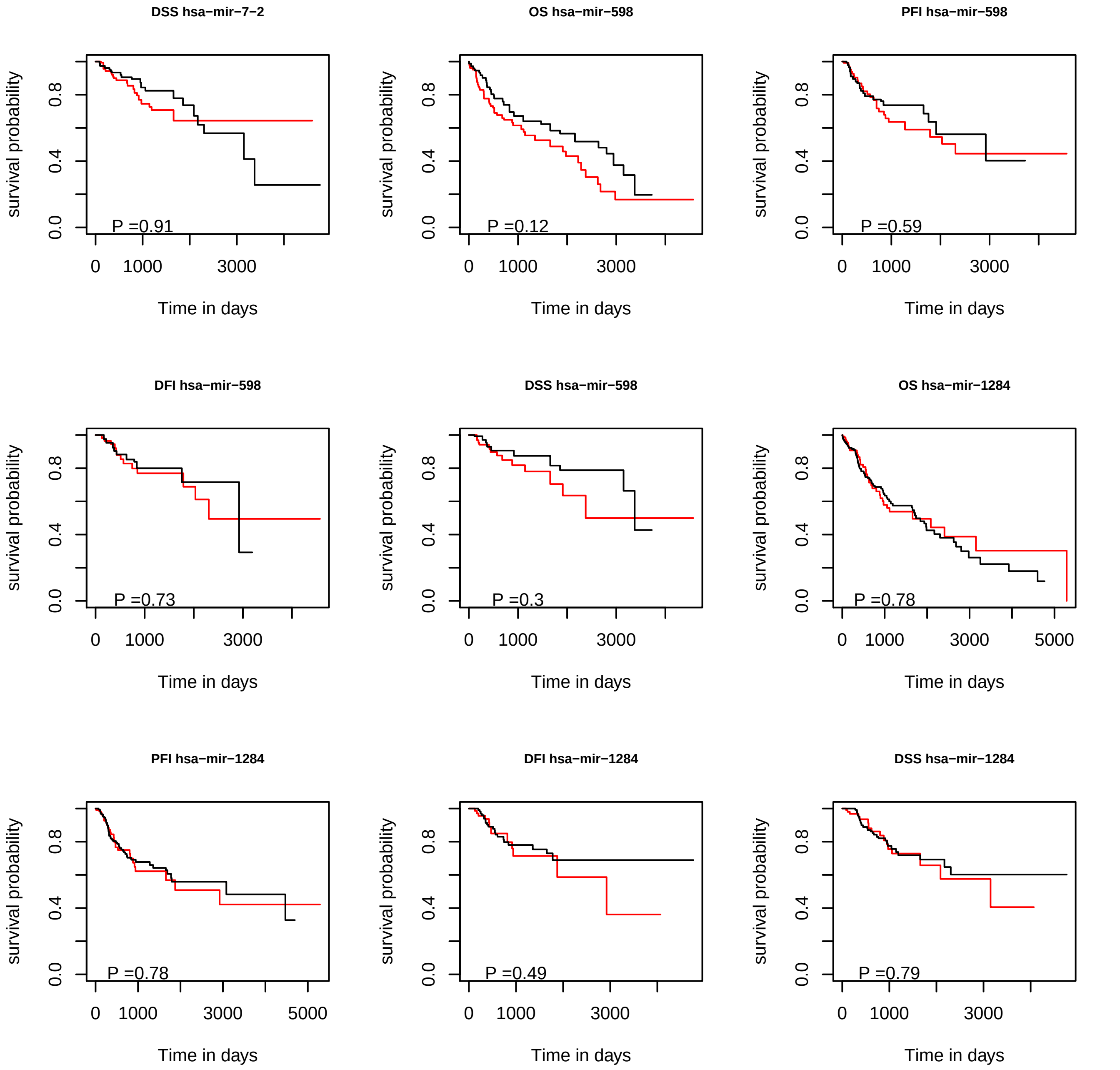

DSS hsa−mir−7−2
P =0.91
OS hsa−mir−598
P =0.12
PFI hsa−mir−598
P =0.59
DFI hsa−mir−598
P =0.73
DSS hsa−mir−598
P =0.3
OS hsa−mir−1284
P =0.78
PFI hsa−mir−1284
P =0.78
DFI hsa−mir−1284
P =0.49
DSS hsa−mir−1284
P =0.79
survival probability
0.0
0.4
0.8
Time in days
0
1000
3000
5000

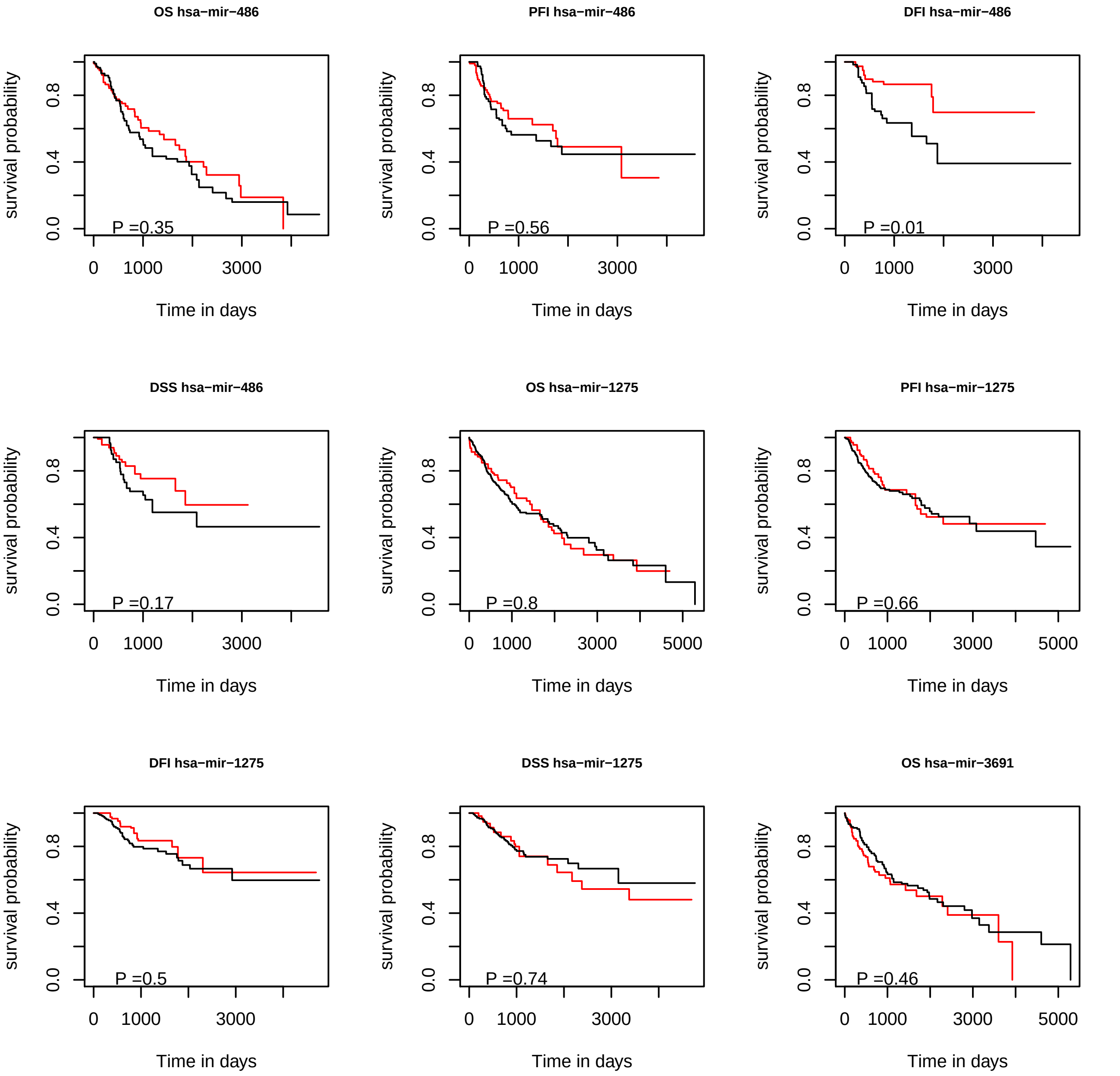

OS hsa−mir−486
P =0.35
PFI hsa−mir−486
P =0.56
DFI hsa−mir−486
P =0.01
DSS hsa−mir−486
P =0.17
OS hsa−mir−1275
P =0.8
PFI hsa−mir−1275
P =0.66
DFI hsa−mir−1275
P =0.5
DSS hsa−mir−1275
P =0.74
OS hsa−mir−3691
P =0.46
survival probability
Time in days
0.0
0.4
0.8
0
1000
3000
5000

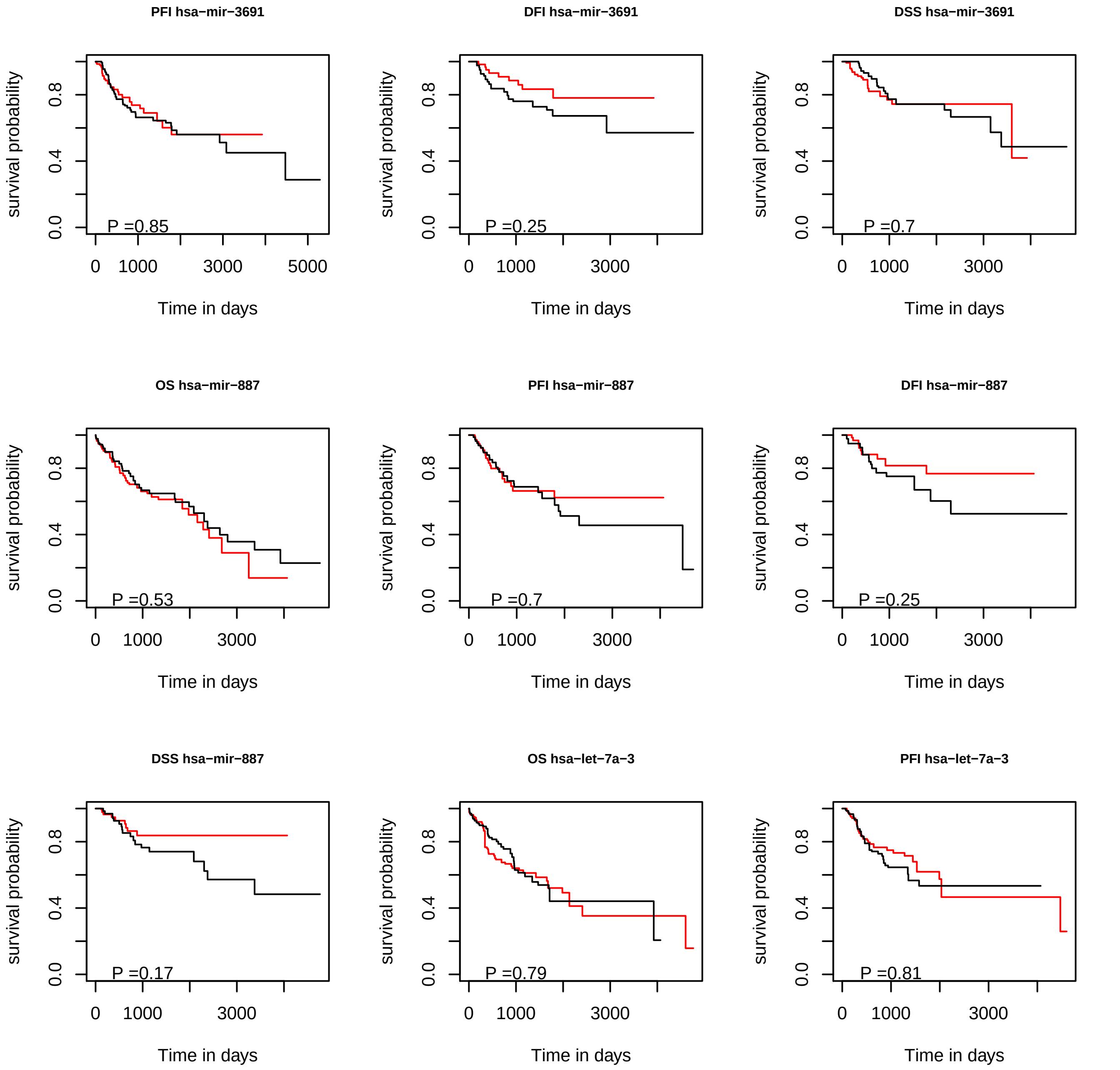
PFI hsa−mir−3691
P =0.85
DFI hsa−mir−3691
P =0.25
DSS hsa−mir−3691
P =0.7
OS hsa−mir−887
P =0.53
PFI hsa−mir−887
P =0.7
DFI hsa−mir−887
P =0.25
DSS hsa−mir−887
P =0.17
OS hsa−let−7a−3
P =0.79
PFI hsa−let−7a−3
P =0.81
survival probability
Time in days
0.0
0.4
0.8
0
1000
3000
5000

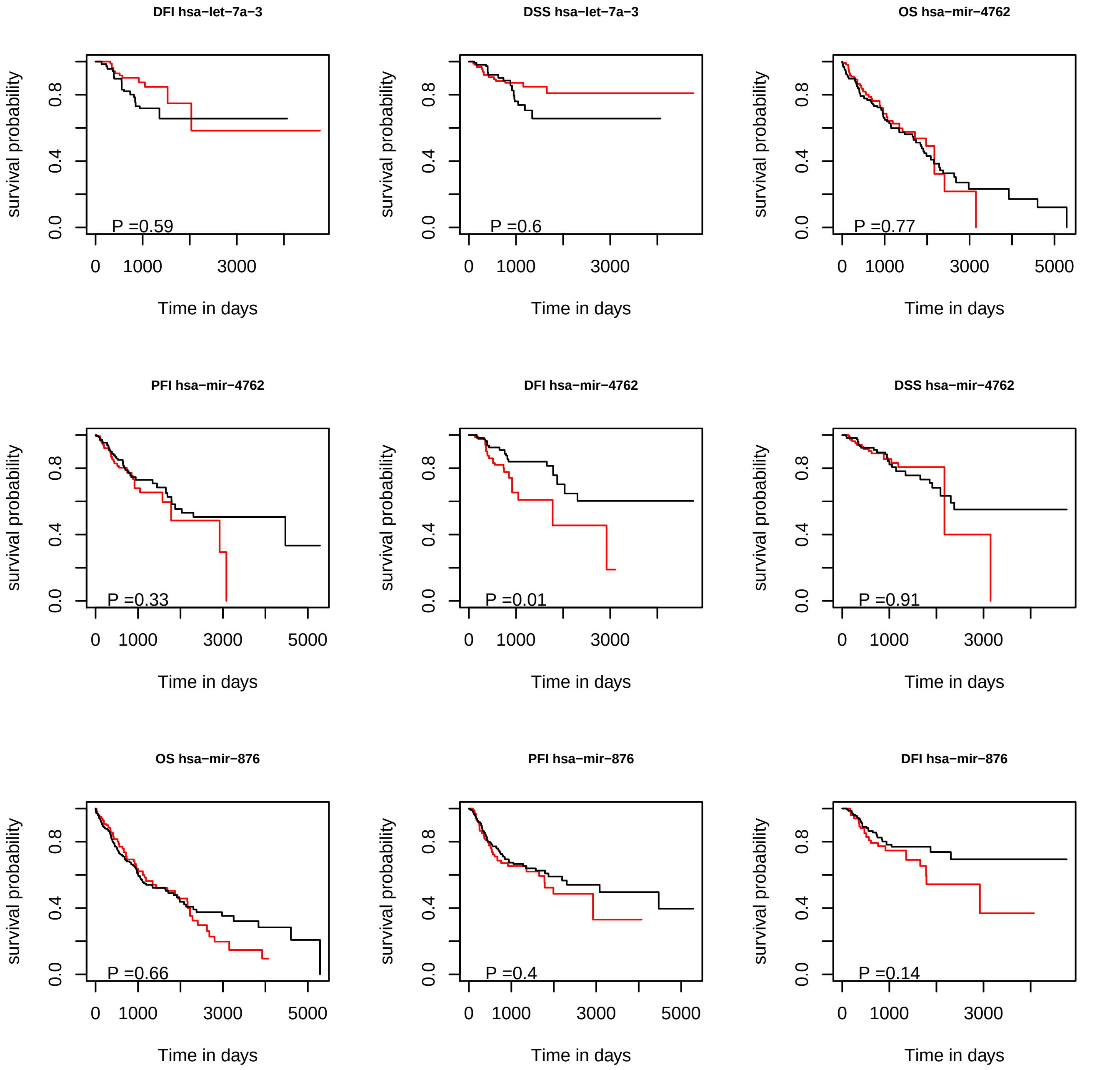
DFI hsa−let−7a−3
P =0.59
DSS hsa−let−7a−3
P =0.6
OS hsa−mir−4762
P =0.77
PFI hsa−mir−4762
P =0.33
DFI hsa−mir−4762
P =0.01
DSS hsa−mir−4762
P =0.91
OS hsa−mir−876
P =0.66
PFI hsa−mir−876
P =0.4
DFI hsa−mir−876
P =0.14
survival probability
Time in days
0.0
0.4
0.8
0
1000
3000
5000

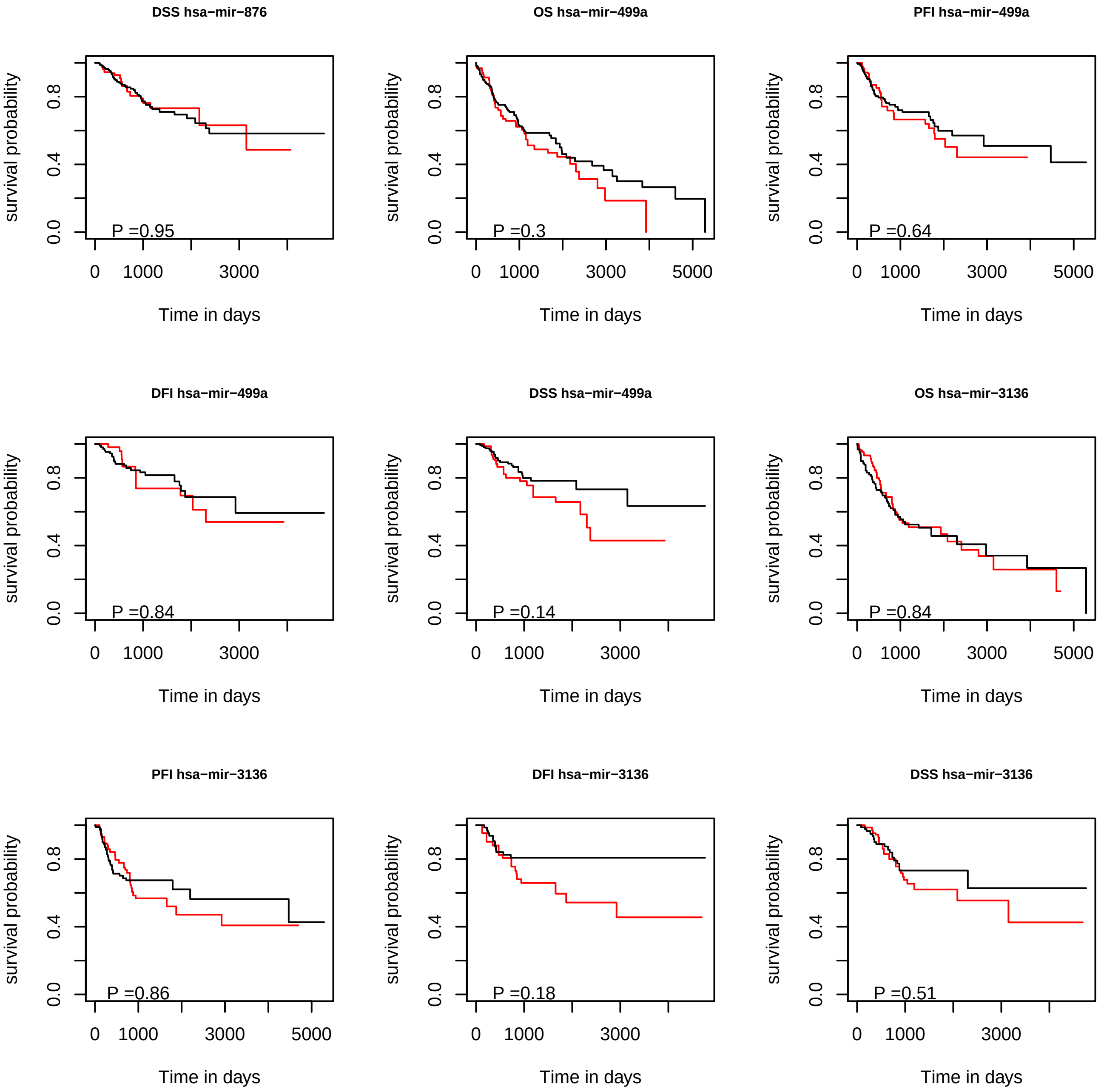
DSS hsa−mir−876
P =0.95
OS hsa−mir−499a
P =0.3
PFI hsa−mir−499a
P =0.64
DFI hsa−mir−499a
P =0.84
DSS hsa−mir−499a
P =0.14
OS hsa−mir−3136
P =0.84
PFI hsa−mir−3136
P =0.86
DFI hsa−mir−3136
P =0.18
DSS hsa−mir−3136
P =0.51
survival probability
Time in days
0.0
0.4
0.8
0
1000
3000
5000

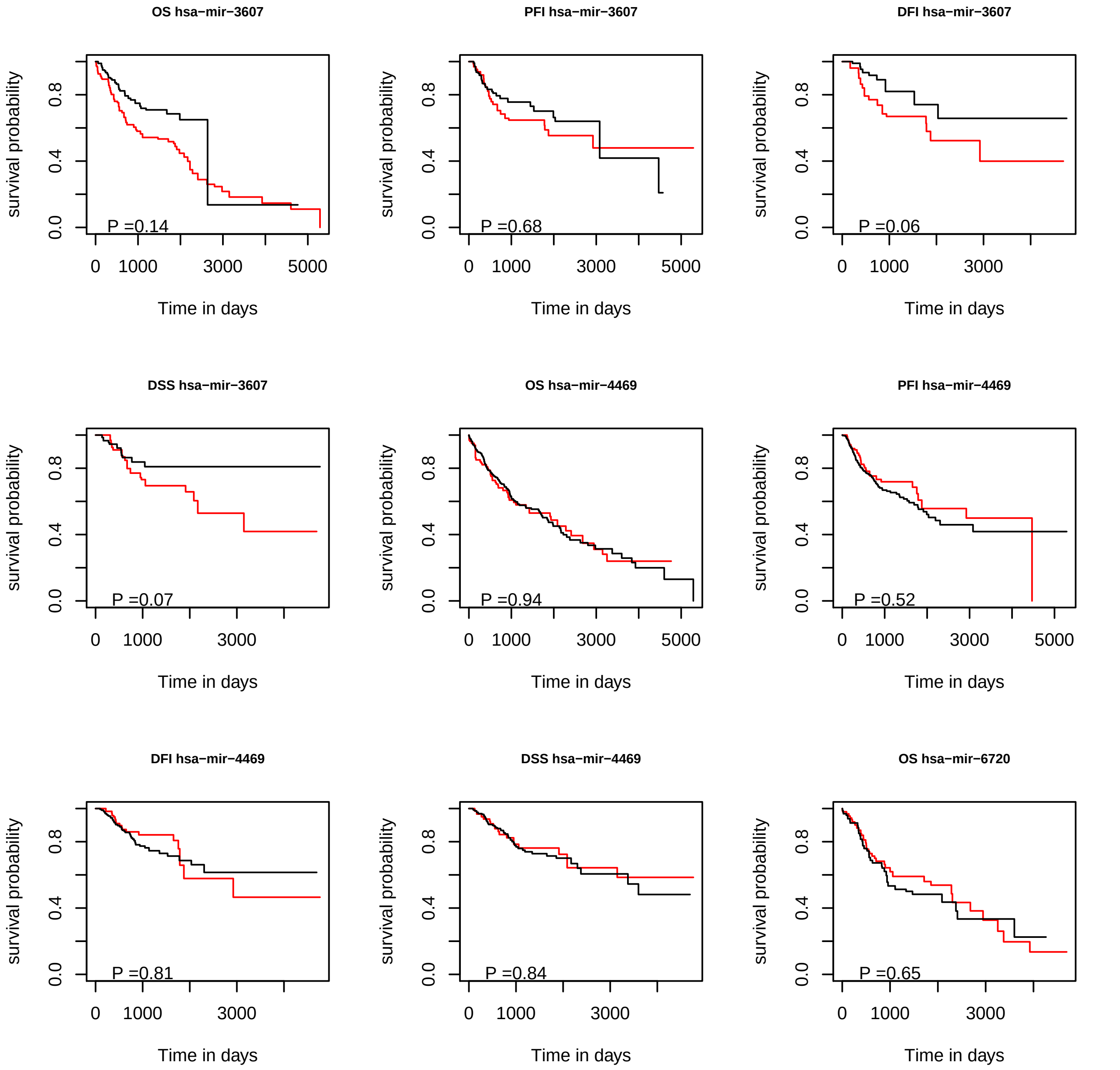
OS hsa−mir−3607
P =0.14
PFI hsa−mir−3607
P =0.68
DFI hsa−mir−3607
P =0.06
DSS hsa−mir−3607
P =0.07
OS hsa−mir−4469
P =0.94
PFI hsa−mir−4469
P =0.52
DFI hsa−mir−4469
P =0.81
DSS hsa−mir−4469
P =0.84
OS hsa−mir−6720
P =0.65
survival probability
0.0
0.4
0.8
Time in days
0
1000
3000
5000

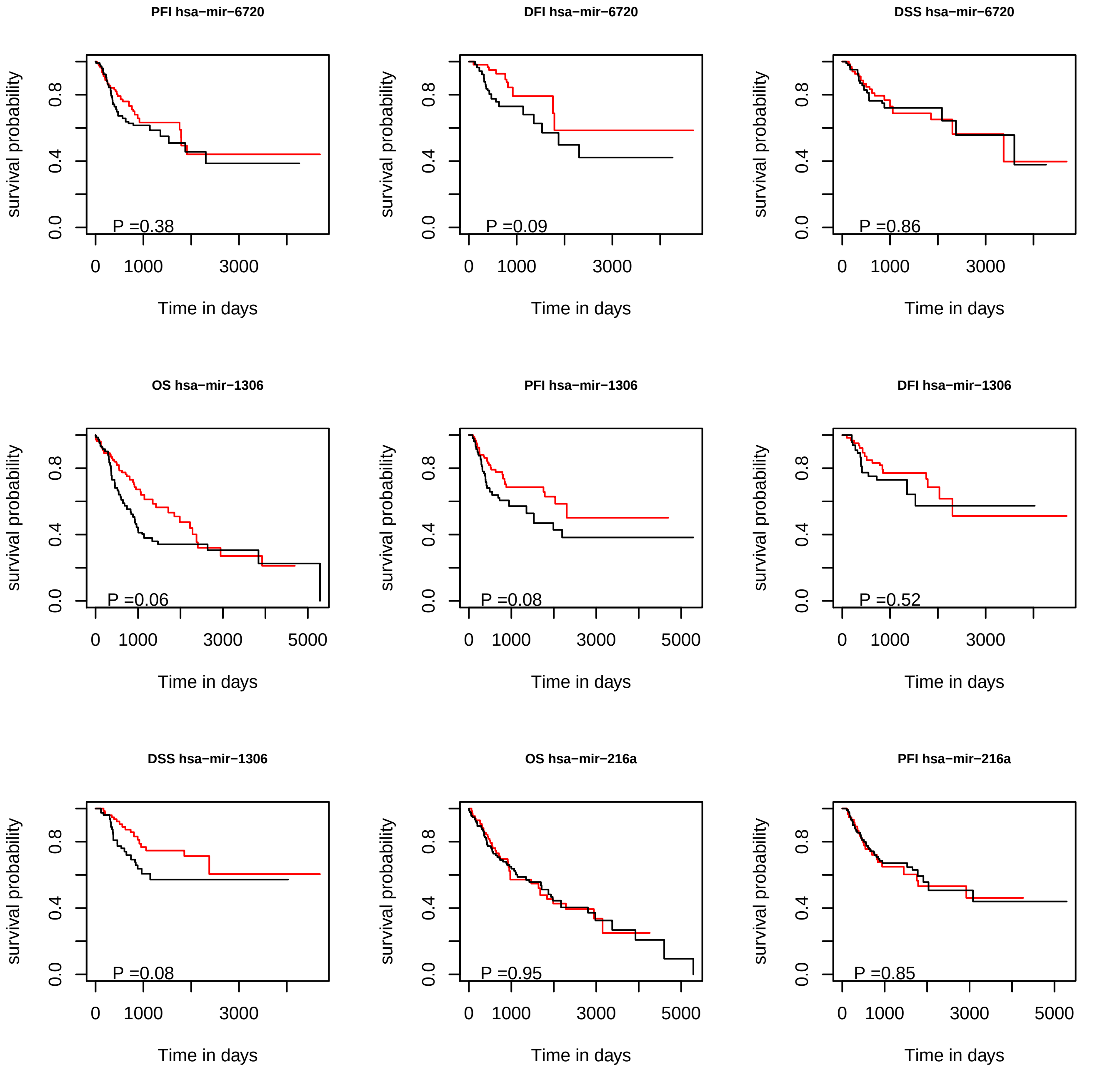
PFI hsa−mir−6720
P =0.38
DFI hsa−mir−6720
P =0.09
DSS hsa−mir−6720
P =0.86
OS hsa−mir−1306
P =0.06
PFI hsa−mir−1306
P =0.08
DFI hsa−mir−1306
P =0.52
DSS hsa−mir−1306
P =0.08
OS hsa−mir−216a
P =0.95
PFI hsa−mir−216a
P =0.85
survival probability
Time in days

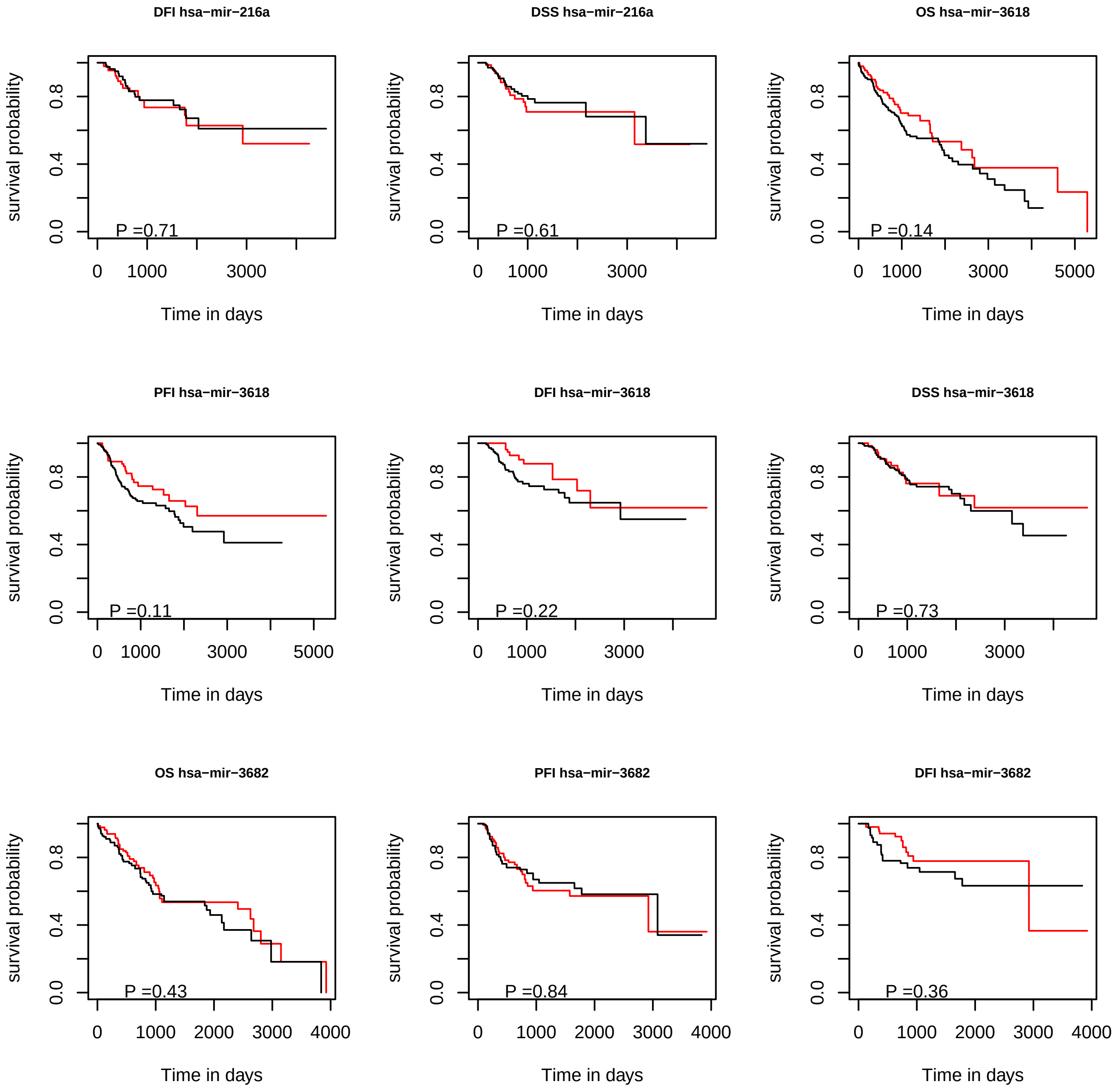
DFI hsa−mir−216a
P =0.71
DSS hsa−mir−216a
P =0.61
OS hsa−mir−3618
P =0.14
PFI hsa−mir−3618
P =0.11
DFI hsa−mir−3618
P =0.22
DSS hsa−mir−3618
P =0.73
OS hsa−mir−3682
P =0.43
PFI hsa−mir−3682
P =0.84
DFI hsa−mir−3682
P =0.36
survival probability
Time in days

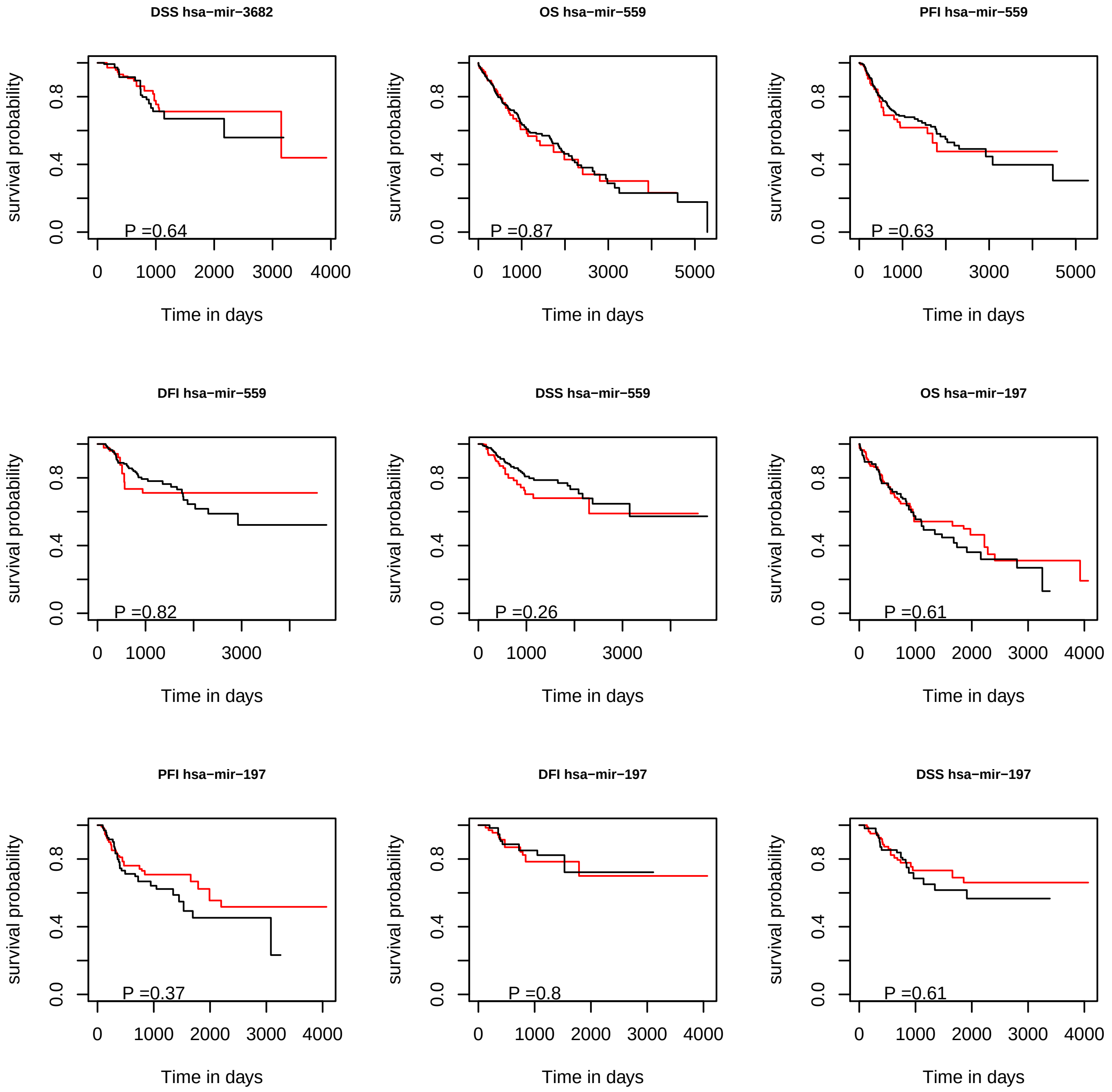
DSS hsa−mir−3682
P =0.64
OS hsa−mir−559
P =0.87
PFI hsa−mir−559
P =0.63
DFI hsa−mir−559
P =0.82
DSS hsa−mir−559
P =0.26
OS hsa−mir−197
P =0.61
PFI hsa−mir−197
P =0.37
DFI hsa−mir−197
P =0.8
DSS hsa−mir−197
P =0.61
survival probability
Time in days
0.0
0.4
0.8
0
1000
2000
3000
4000
5000

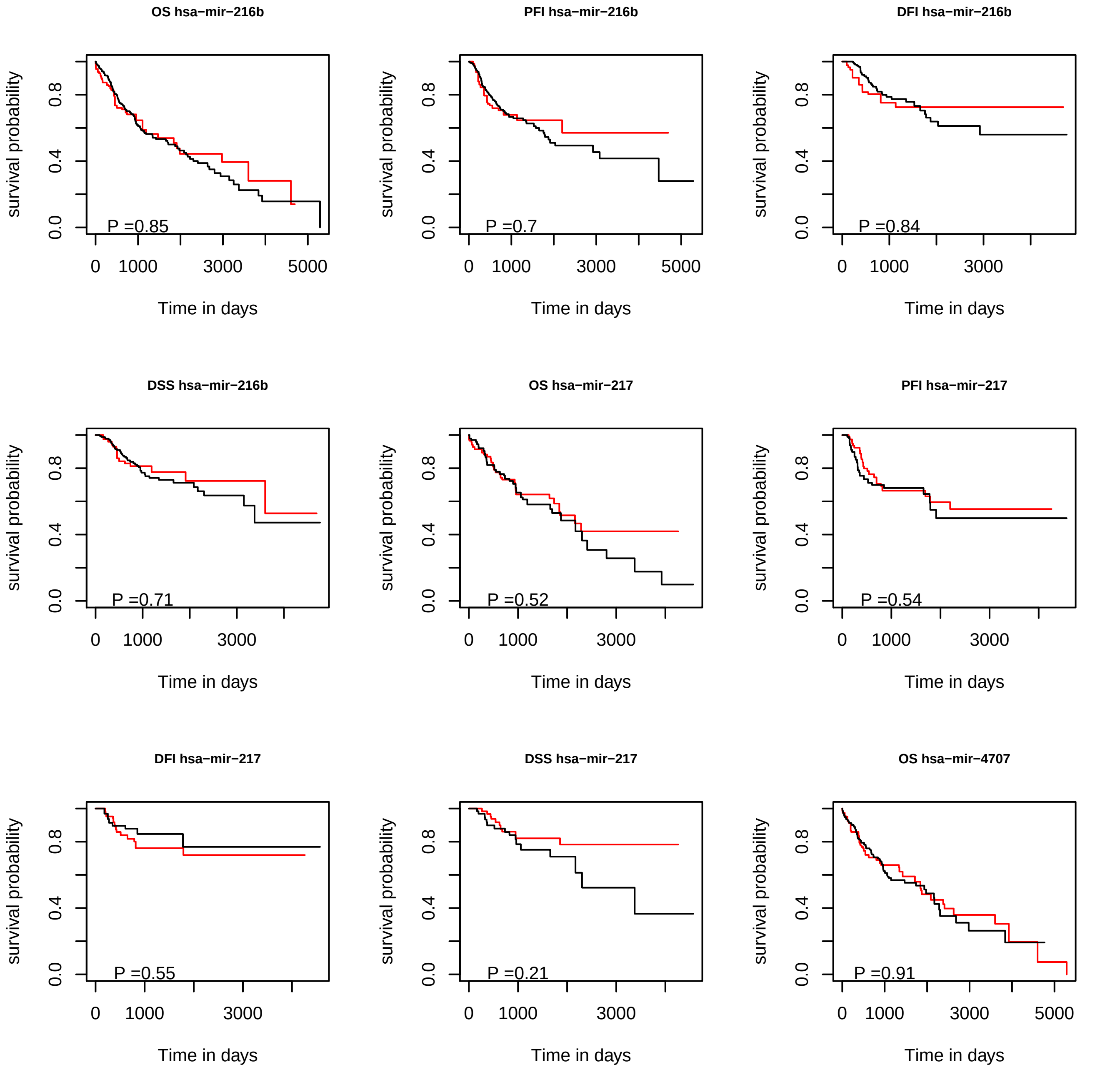
OS hsa−mir−216b
P =0.85
PFI hsa−mir−216b
P =0.7
DFI hsa−mir−216b
P =0.84
DSS hsa−mir−216b
P =0.71
OS hsa−mir−217
P =0.52
PFI hsa−mir−217
P =0.54
DFI hsa−mir−217
P =0.55
DSS hsa−mir−217
P =0.21
OS hsa−mir−4707
P =0.91
survival probability
Time in days
0.0
0.4
0.8
0
1000
3000
5000

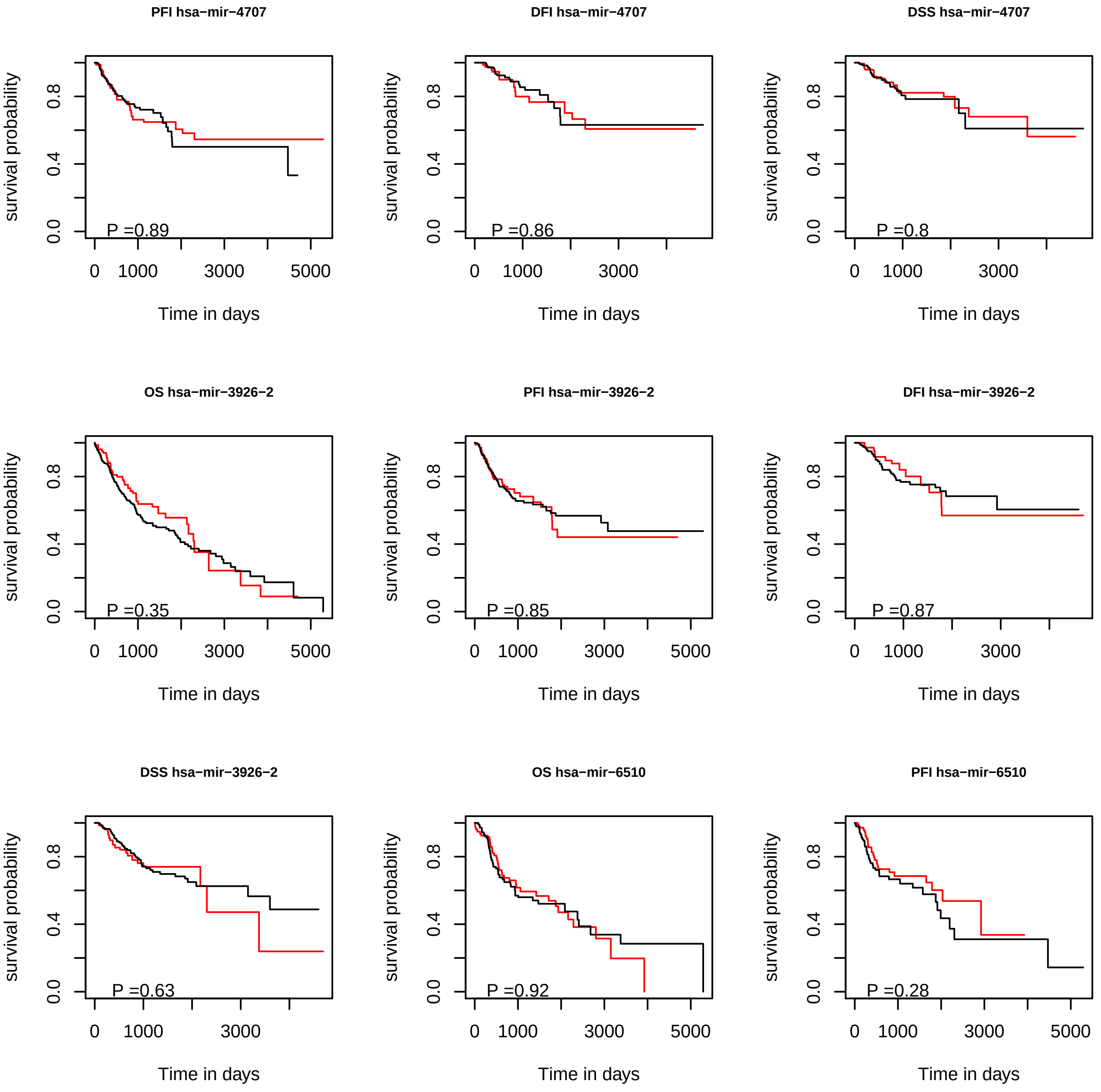
PFI hsa−mir−4707
P =0.89
DFI hsa−mir−4707
P =0.86
DSS hsa−mir−4707
P =0.8
OS hsa−mir−3926−2
P =0.35
PFI hsa−mir−3926−2
P =0.85
DFI hsa−mir−3926−2
P =0.87
DSS hsa−mir−3926−2
P =0.63
OS hsa−mir−6510
P =0.92
PFI hsa−mir−6510
P =0.28
survival probability
Time in days
0.0
0.4
0.8
0
1000
3000
5000

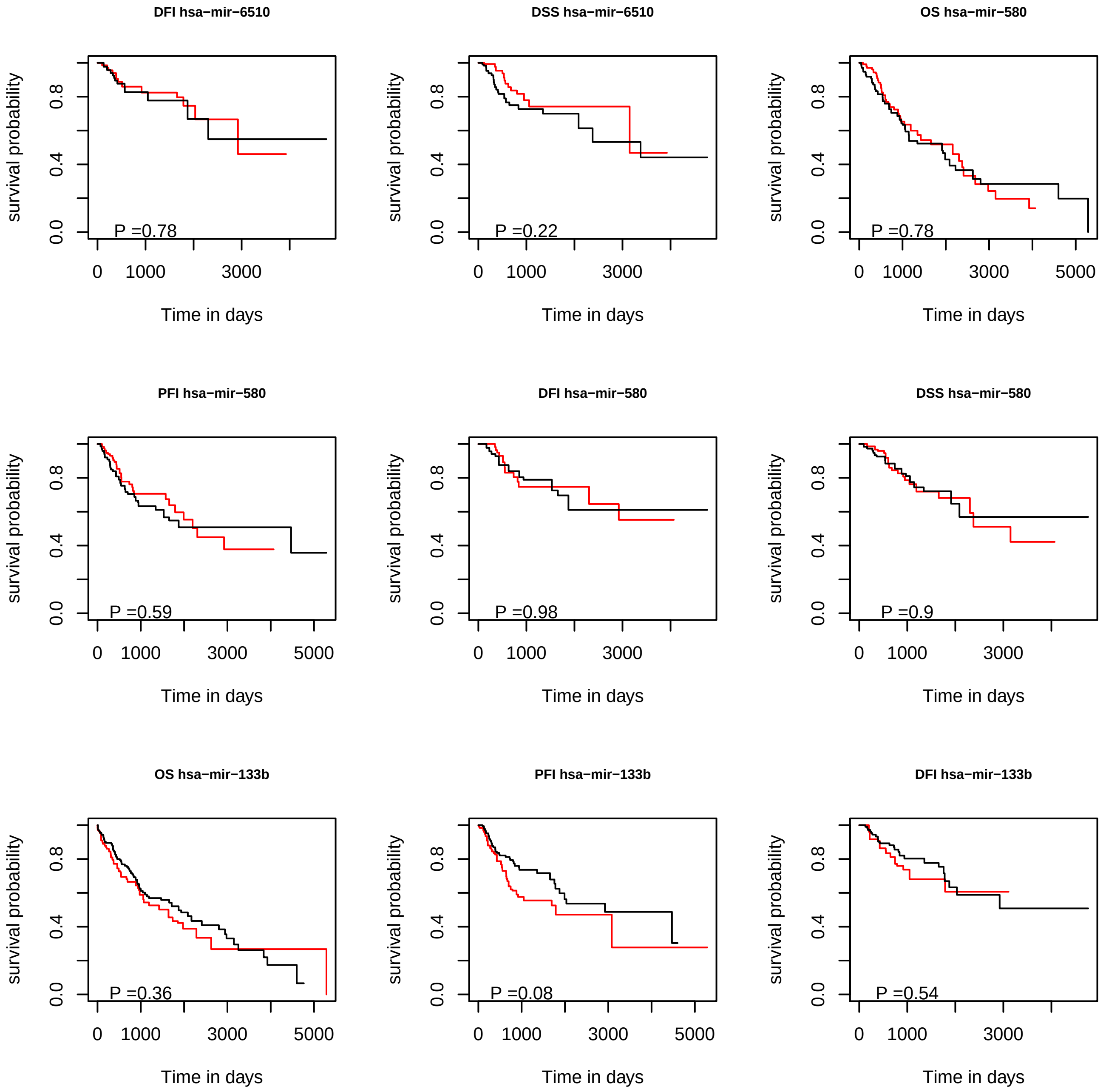
DFI hsa−mir−6510
P =0.78
DSS hsa−mir−6510
P =0.22
OS hsa−mir−580
P =0.78
PFI hsa−mir−580
P =0.59
DFI hsa−mir−580
P =0.98
DSS hsa−mir−580
P =0.9
OS hsa−mir−133b
P =0.36
PFI hsa−mir−133b
P =0.08
DFI hsa−mir−133b
P =0.54
survival probability
Time in days
0.0
0.4
0.8
0
1000
3000
5000

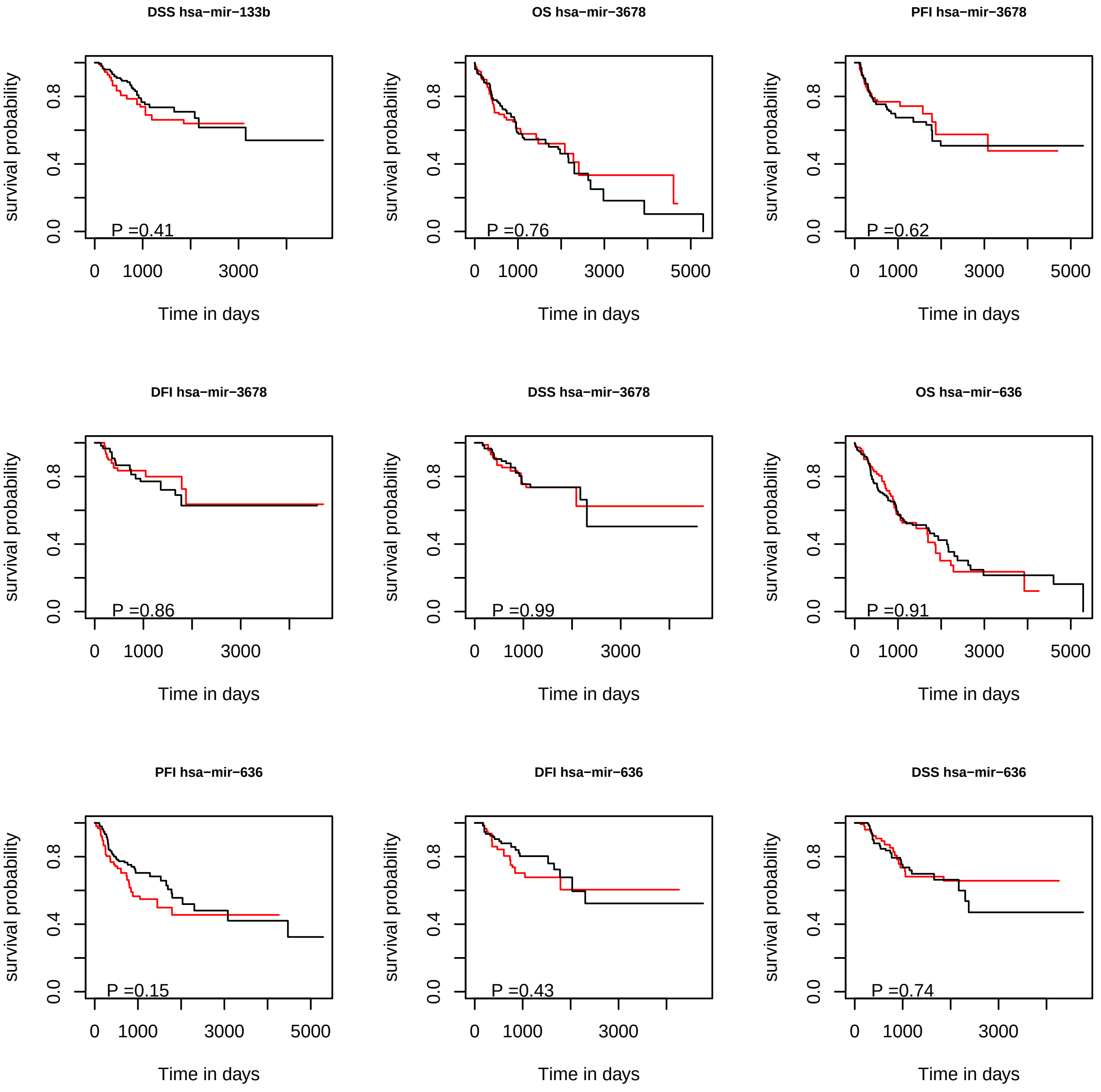
DSS hsa−mir−133b
P =0.41
OS hsa−mir−3678
P =0.76
PFI hsa−mir−3678
P =0.62
DFI hsa−mir−3678
P =0.86
DSS hsa−mir−3678
P =0.99
OS hsa−mir−636
P =0.91
PFI hsa−mir−636
P =0.15
DFI hsa−mir−636
P =0.43
DSS hsa−mir−636
P =0.74
survival probability
Time in days
0.0
0.4
0.8
0
1000
3000
5000

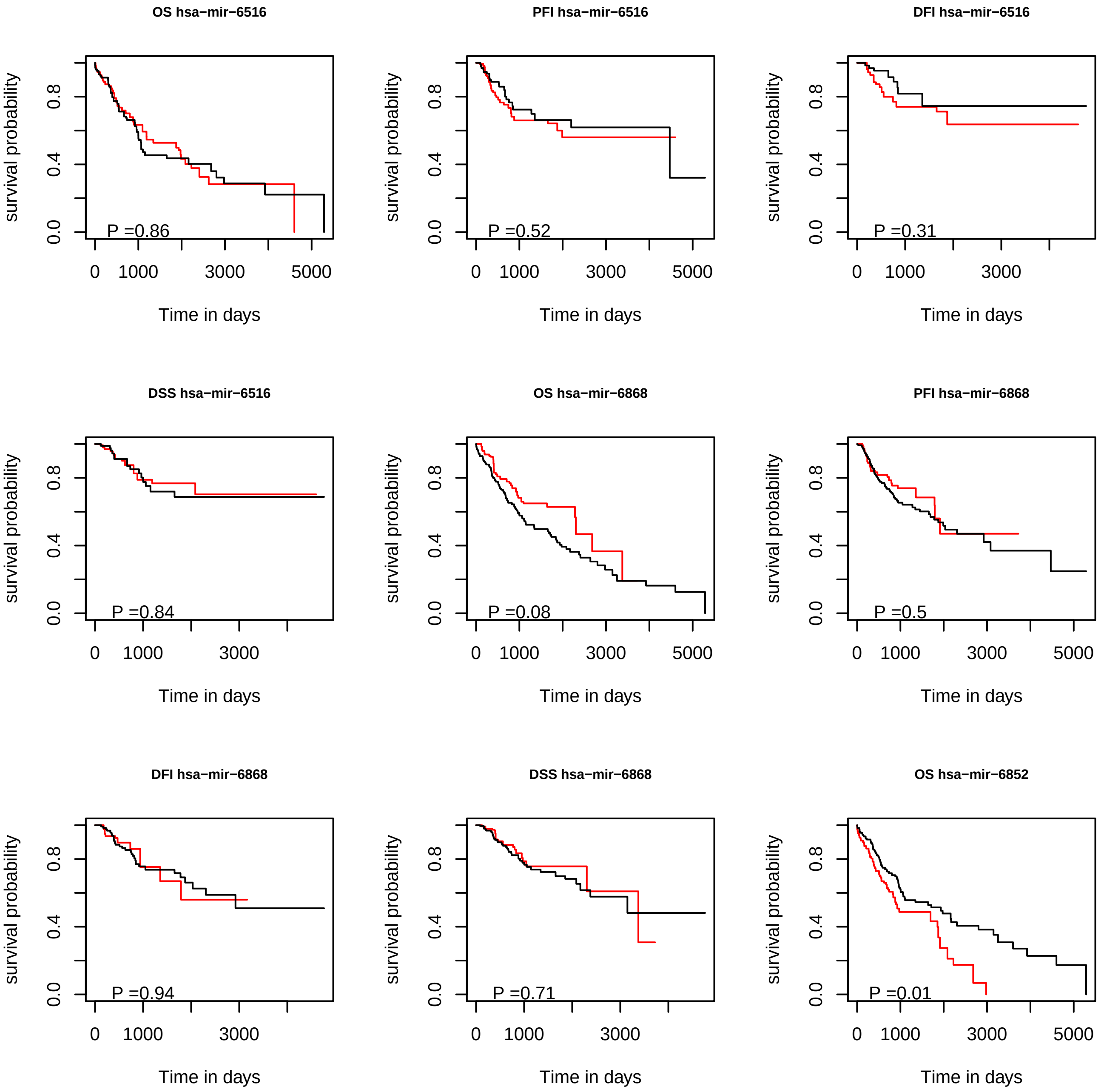
OS hsa−mir−6516
survival probability
0.0
0.4
0.8
P =0.86
0
1000
3000
5000
Time in days
PFI hsa−mir−6516
survival probability
0.0
0.4
0.8
P =0.52
0
1000
3000
5000
Time in days
DFI hsa−mir−6516
survival probability
0.0
0.4
0.8
P =0.31
0
1000
3000
Time in days
DSS hsa−mir−6516
survival probability
0.0
0.4
0.8
P =0.84
0
1000
3000
Time in days
OS hsa−mir−6868
survival probability
0.0
0.4
0.8
P =0.08
0
1000
3000
5000
Time in days
PFI hsa−mir−6868
survival probability
0.0
0.4
0.8
P =0.5
0
1000
3000
5000
Time in days
DFI hsa−mir−6868
survival probability
0.0
0.4
0.8
P =0.94
0
1000
3000
Time in days
DSS hsa−mir−6868
survival probability
0.0
0.4
0.8
P =0.71
0
1000
3000
Time in days
OS hsa−mir−6852
survival probability
0.0
0.4
0.8
P =0.01
0
1000
3000
5000
Time in days

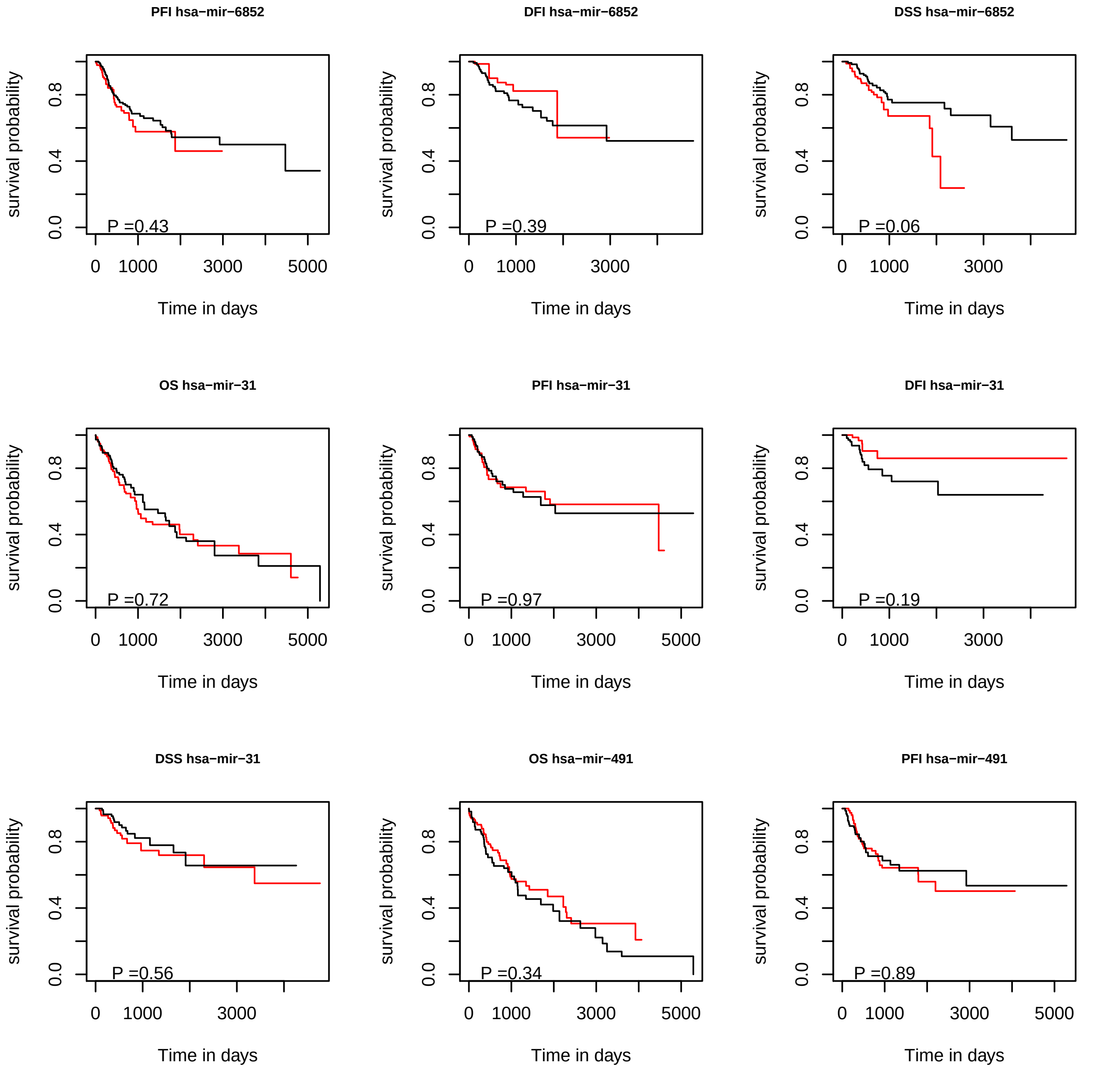

PFI hsa−mir−6852
survival probability
0.0
0.4
0.8
P =0.43
0
1000
3000
5000
Time in days
DFI hsa−mir−6852
survival probability
0.0
0.4
0.8
P =0.39
0
1000
3000
Time in days
DSS hsa−mir−6852
survival probability
0.0
0.4
0.8
P =0.06
0
1000
3000
Time in days
OS hsa−mir−31
survival probability
0.0
0.4
0.8
P =0.72
0
1000
3000
5000
Time in days
PFI hsa−mir−31
survival probability
0.0
0.4
0.8
P =0.97
0
1000
3000
5000
Time in days
DFI hsa−mir−31
survival probability
0.0
0.4
0.8
P =0.19
0
1000
3000
Time in days
DSS hsa−mir−31
survival probability
0.0
0.4
0.8
P =0.56
0
1000
3000
Time in days
OS hsa−mir−491
survival probability
0.0
0.4
0.8
P =0.34
0
1000
3000
5000
Time in days
PFI hsa−mir−491
survival probability
0.0
0.4
0.8
P =0.89
0
1000
3000
5000
Time in days

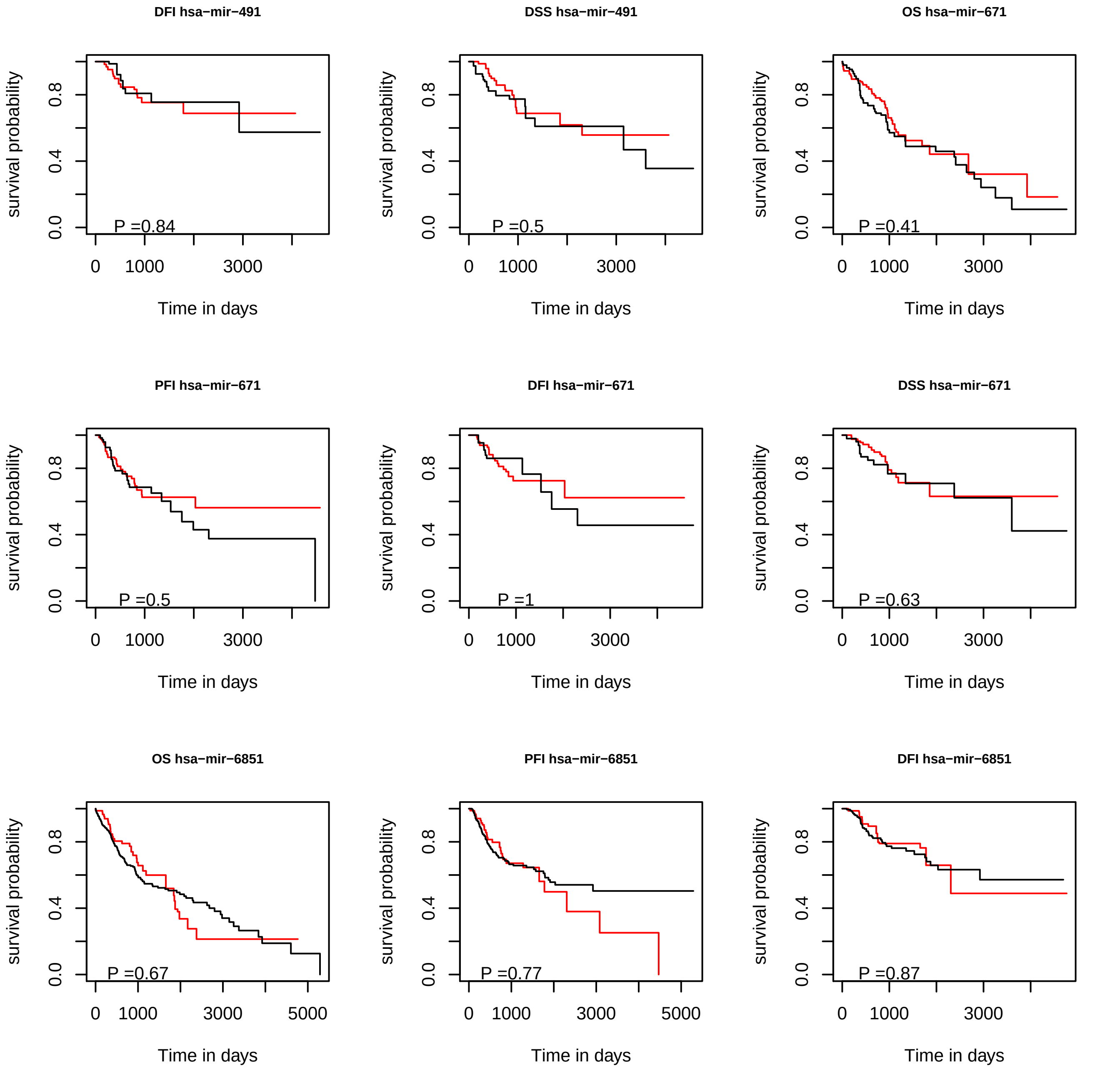
DFI hsa−mir−491
survival probability
0.0
0.4
0.8
P =0.84
0
1000
3000
Time in days
DSS hsa−mir−491
survival probability
0.0
0.4
0.8
P =0.5
0
1000
3000
Time in days
OS hsa−mir−671
survival probability
0.0
0.4
0.8
P =0.41
0
1000
3000
Time in days
PFI hsa−mir−671
survival probability
0.0
0.4
0.8
P =0.5
0
1000
3000
Time in days
DFI hsa−mir−671
survival probability
0.0
0.4
0.8
P =1
0
1000
3000
Time in days
DSS hsa−mir−671
survival probability
0.0
0.4
0.8
P =0.63
0
1000
3000
Time in days
OS hsa−mir−6851
survival probability
0.0
0.4
0.8
P =0.67
0
1000
3000
5000
Time in days
PFI hsa−mir−6851
survival probability
0.0
0.4
0.8
P =0.77
0
1000
3000
5000
Time in days
DFI hsa−mir−6851
survival probability
0.0
0.4
0.8
P =0.87
0
1000
3000
Time in days

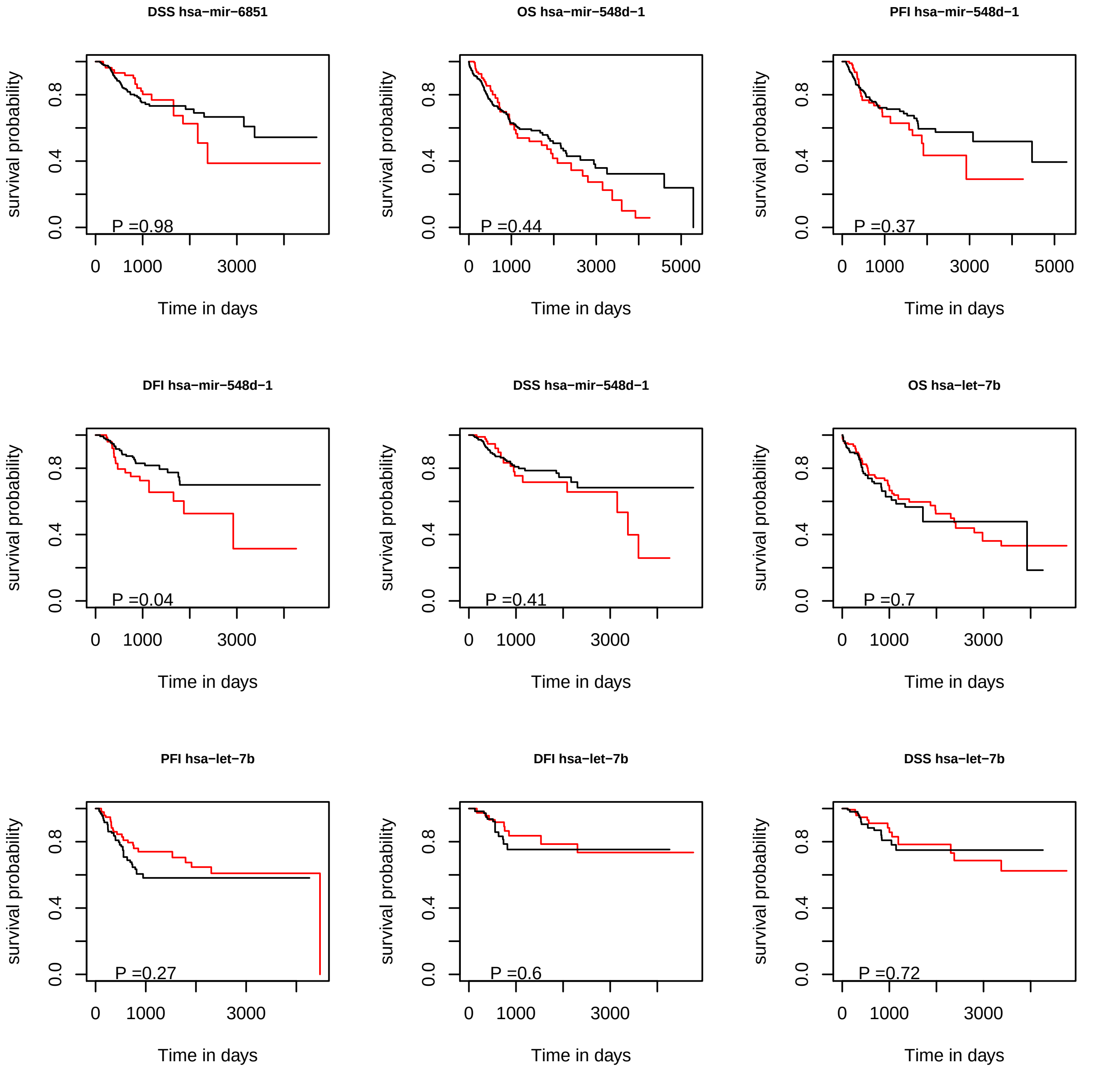
DSS hsa−mir−6851
P =0.98
OS hsa−mir−548d−1
P =0.44
PFI hsa−mir−548d−1
P =0.37
DFI hsa−mir−548d−1
P =0.04
DSS hsa−mir−548d−1
P =0.41
OS hsa−let−7b
P =0.7
PFI hsa−let−7b
P =0.27
DFI hsa−let−7b
P =0.6
DSS hsa−let−7b
P =0.72
survival probability
0.0
0.4
0.8
0
1000
3000
5000
Time in days

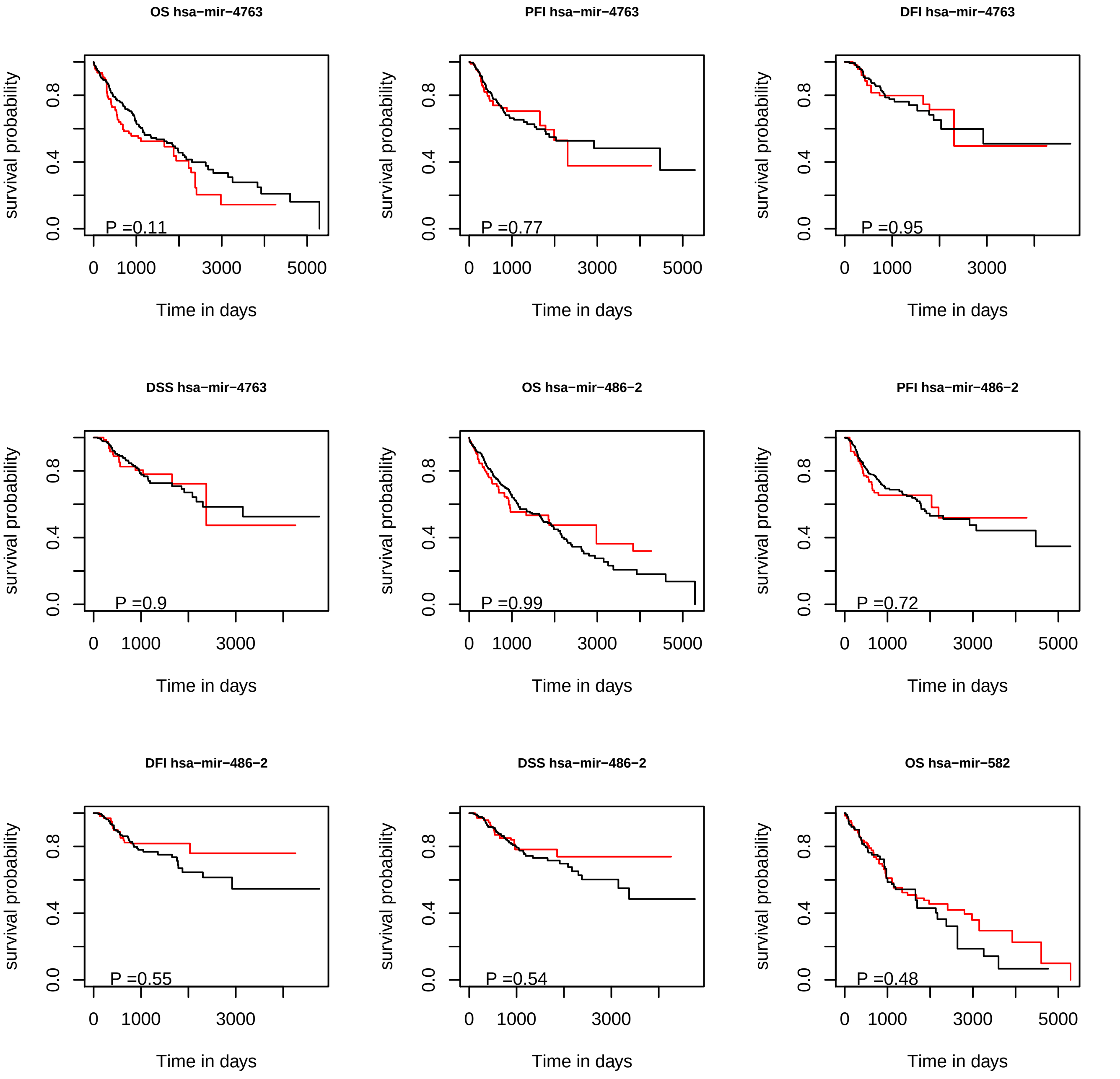
OS hsa−mir−4763
P =0.11
PFI hsa−mir−4763
P =0.77
DFI hsa−mir−4763
P =0.95
DSS hsa−mir−4763
P =0.9
OS hsa−mir−486−2
P =0.99
PFI hsa−mir−486−2
P =0.72
DFI hsa−mir−486−2
P =0.55
DSS hsa−mir−486−2
P =0.54
OS hsa−mir−582
P =0.48
survival probability
0.0
0.4
0.8
Time in days
0
1000
3000
5000

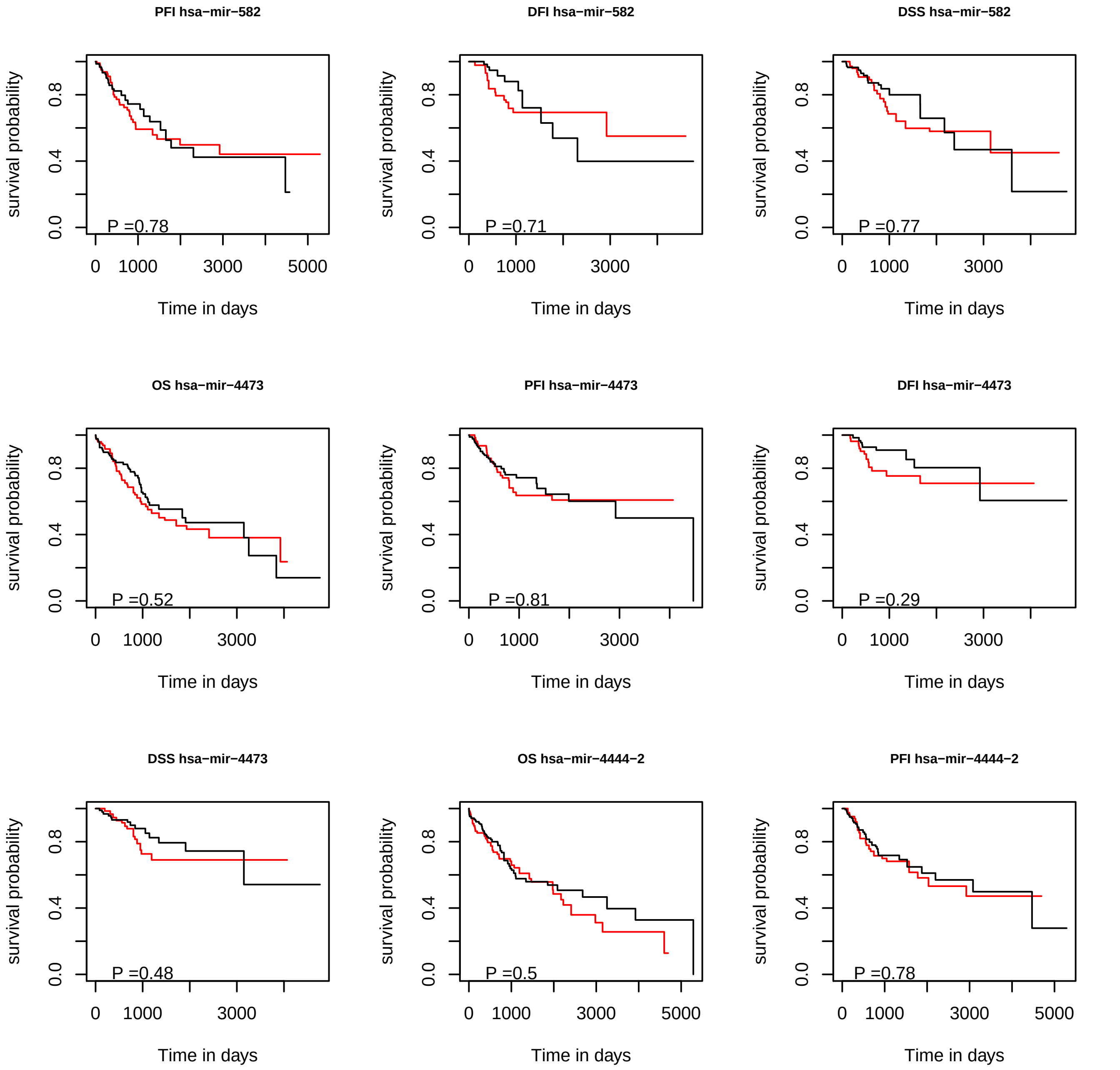
PFI hsa−mir−582
P =0.78
DFI hsa−mir−582
P =0.71
DSS hsa−mir−582
P =0.77
OS hsa−mir−4473
P =0.52
PFI hsa−mir−4473
P =0.81
DFI hsa−mir−4473
P =0.29
DSS hsa−mir−4473
P =0.48
OS hsa−mir−4444−2
P =0.5
PFI hsa−mir−4444−2
P =0.78
survival probability
0.0
0.4
0.8
Time in days
0
1000
3000
5000

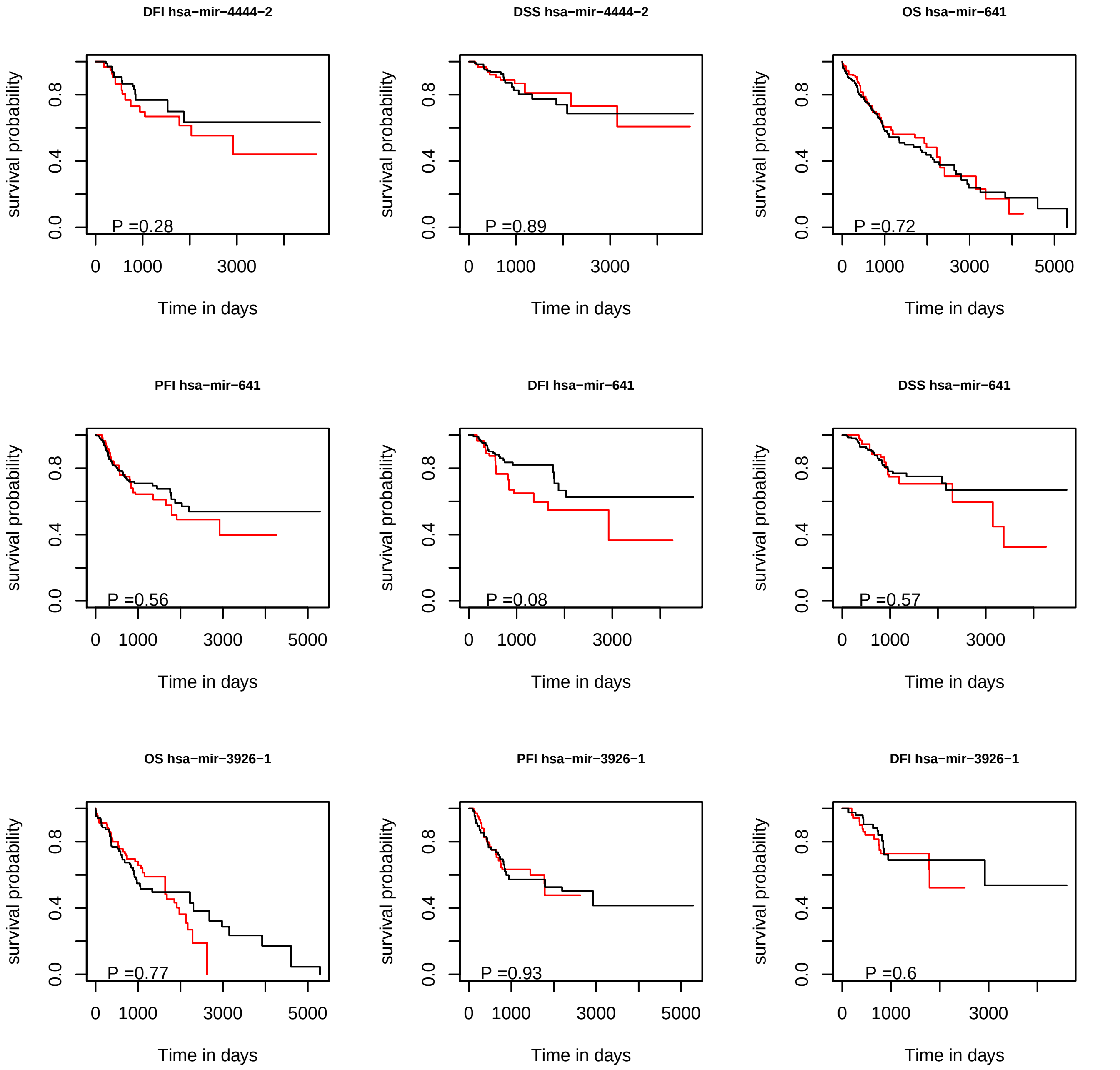
DFI hsa−mir−4444−2
P =0.28
DSS hsa−mir−4444−2
P =0.89
OS hsa−mir−641
P =0.72
PFI hsa−mir−641
P =0.56
DFI hsa−mir−641
P =0.08
DSS hsa−mir−641
P =0.57
OS hsa−mir−3926−1
P =0.77
PFI hsa−mir−3926−1
P =0.93
DFI hsa−mir−3926−1
P =0.6
survival probability
Time in days
0.0
0.4
0.8
0
1000
3000
5000

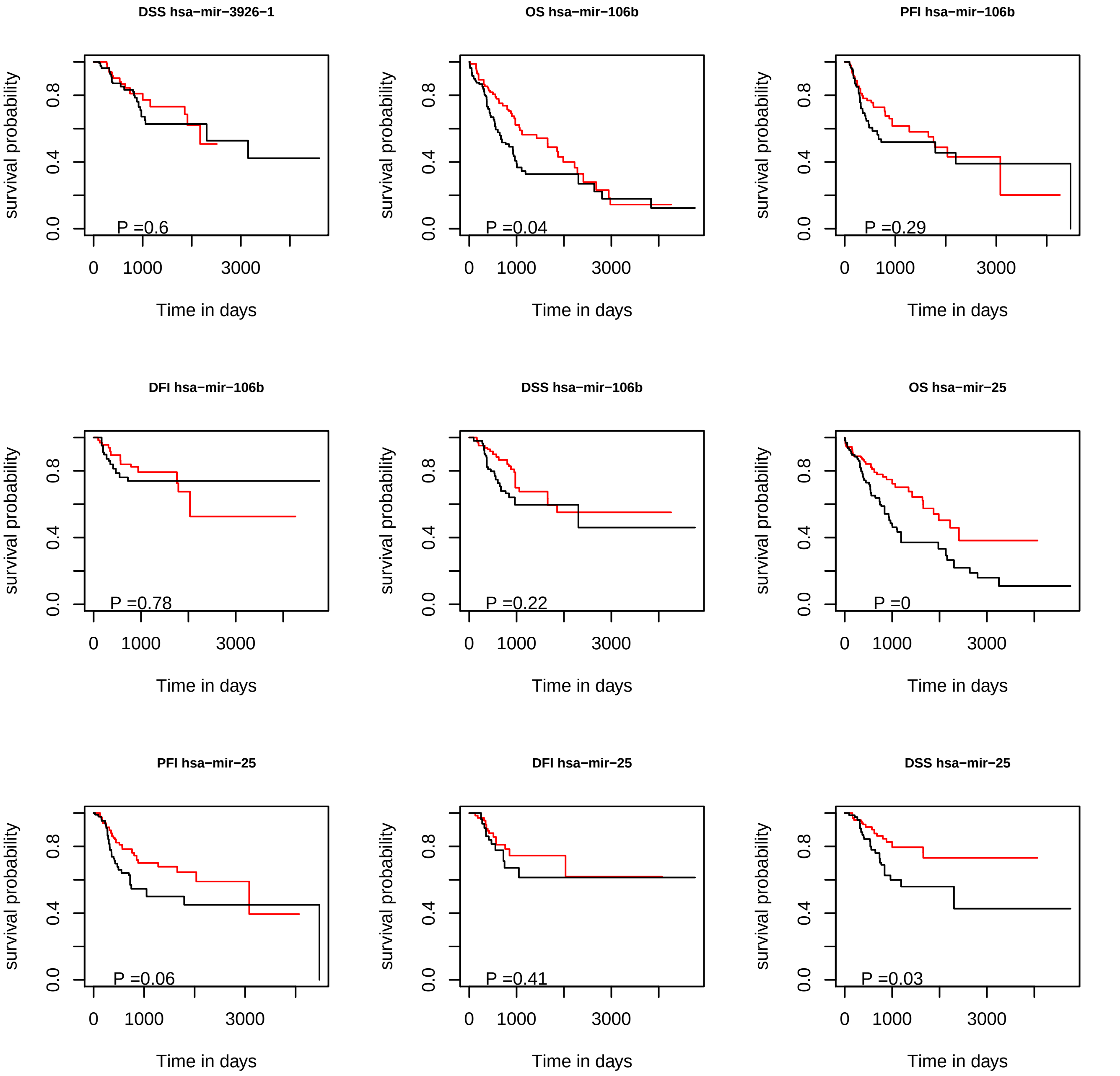
DSS hsa−mir−3926−1
P =0.6
OS hsa−mir−106b
P =0.04
PFI hsa−mir−106b
P =0.29
DFI hsa−mir−106b
P =0.78
DSS hsa−mir−106b
P =0.22
OS hsa−mir−25
P =0
PFI hsa−mir−25
P =0.06
DFI hsa−mir−25
P =0.41
DSS hsa−mir−25
P =0.03
survival probability
0.0
0.4
0.8
Time in days
0
1000
3000

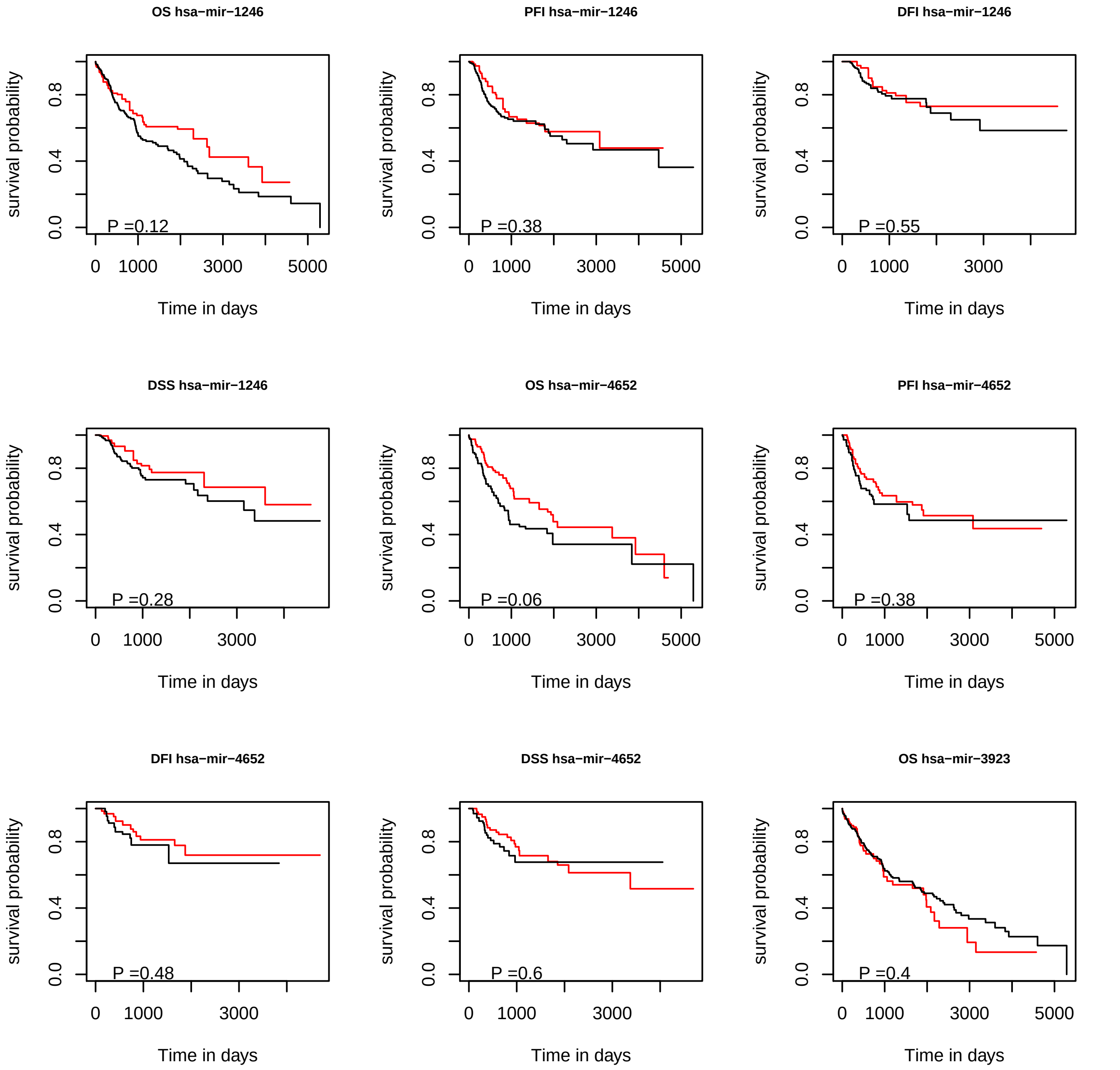
OS hsa−mir−1246
P =0.12
PFI hsa−mir−1246
P =0.38
DFI hsa−mir−1246
P =0.55
DSS hsa−mir−1246
P =0.28
OS hsa−mir−4652
P =0.06
PFI hsa−mir−4652
P =0.38
DFI hsa−mir−4652
P =0.48
DSS hsa−mir−4652
P =0.6
OS hsa−mir−3923
P =0.4
survival probability
Time in days

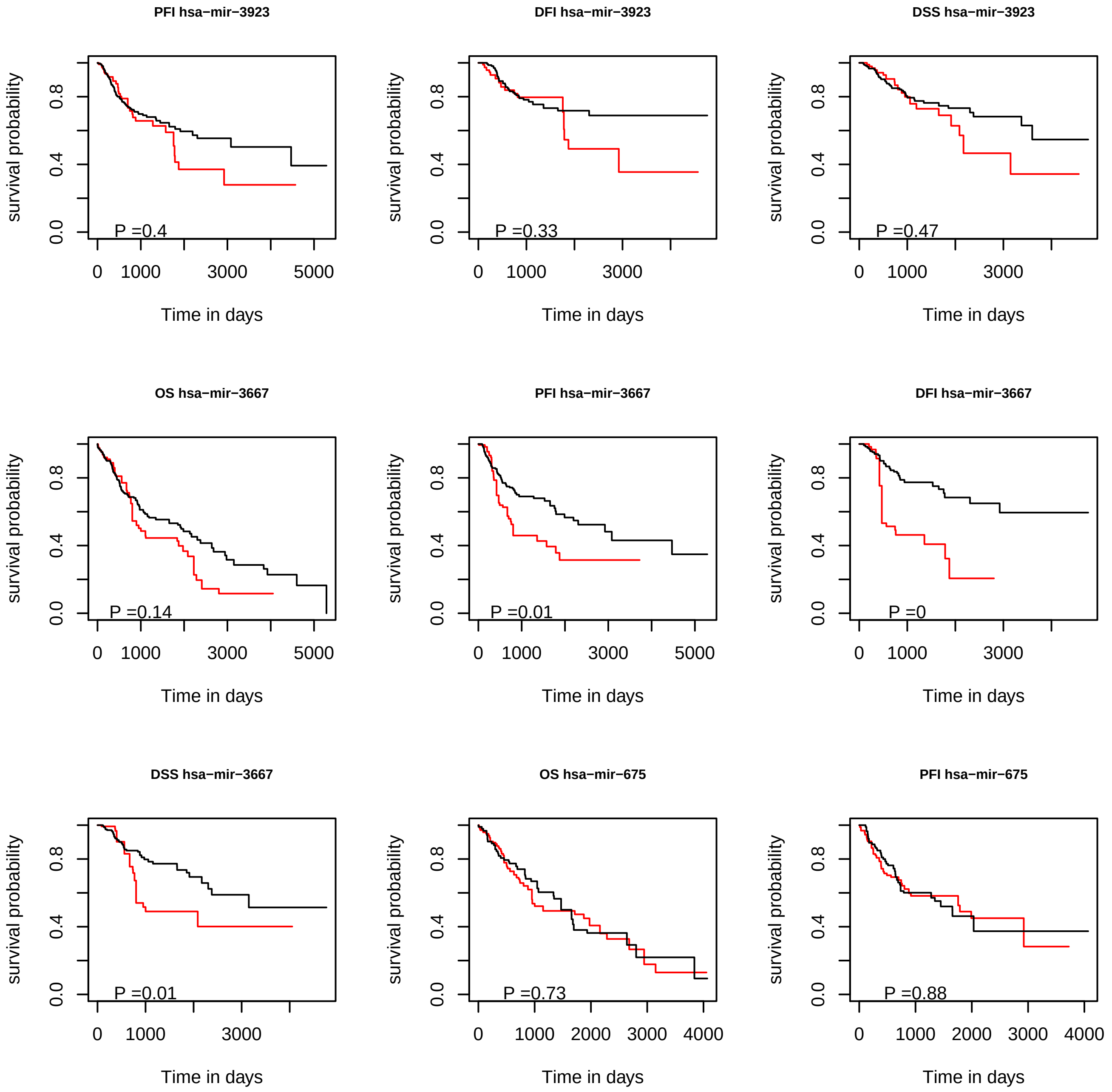
PFI hsa−mir−3923
P =0.4
DFI hsa−mir−3923
P =0.33
DSS hsa−mir−3923
P =0.47
OS hsa−mir−3667
P =0.14
PFI hsa−mir−3667
P =0.01
DFI hsa−mir−3667
P =0
DSS hsa−mir−3667
P =0.01
OS hsa−mir−675
P =0.73
PFI hsa−mir−675
P =0.88
survival probability
Time in days

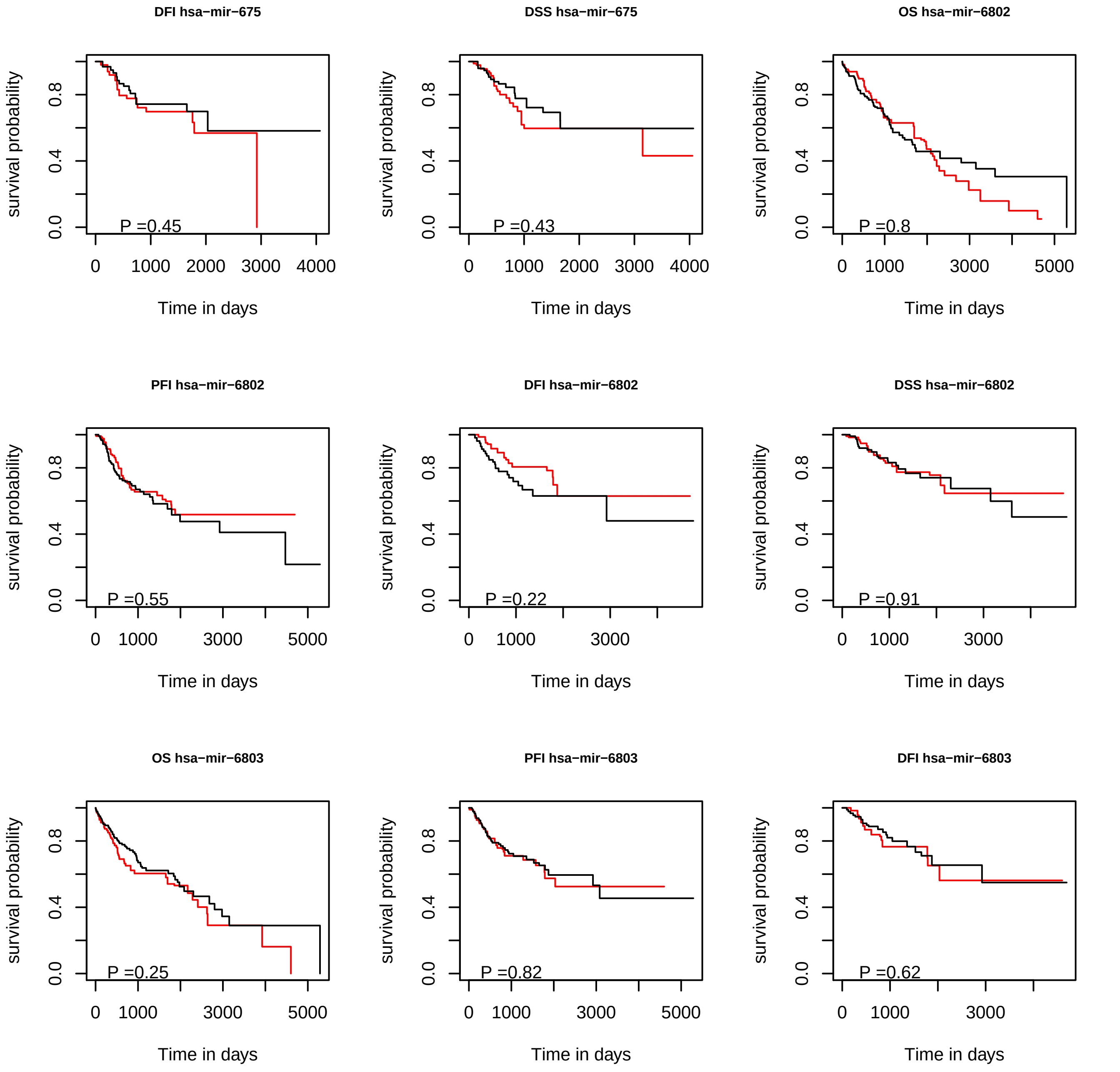
DFI hsa−mir−675
P =0.45
DSS hsa−mir−675
P =0.43
OS hsa−mir−6802
P =0.8
PFI hsa−mir−6802
P =0.55
DFI hsa−mir−6802
P =0.22
DSS hsa−mir−6802
P =0.91
OS hsa−mir−6803
P =0.25
PFI hsa−mir−6803
P =0.82
DFI hsa−mir−6803
P =0.62
survival probability
Time in days
0.0
0.4
0.8
0
1000
2000
3000
4000
5000

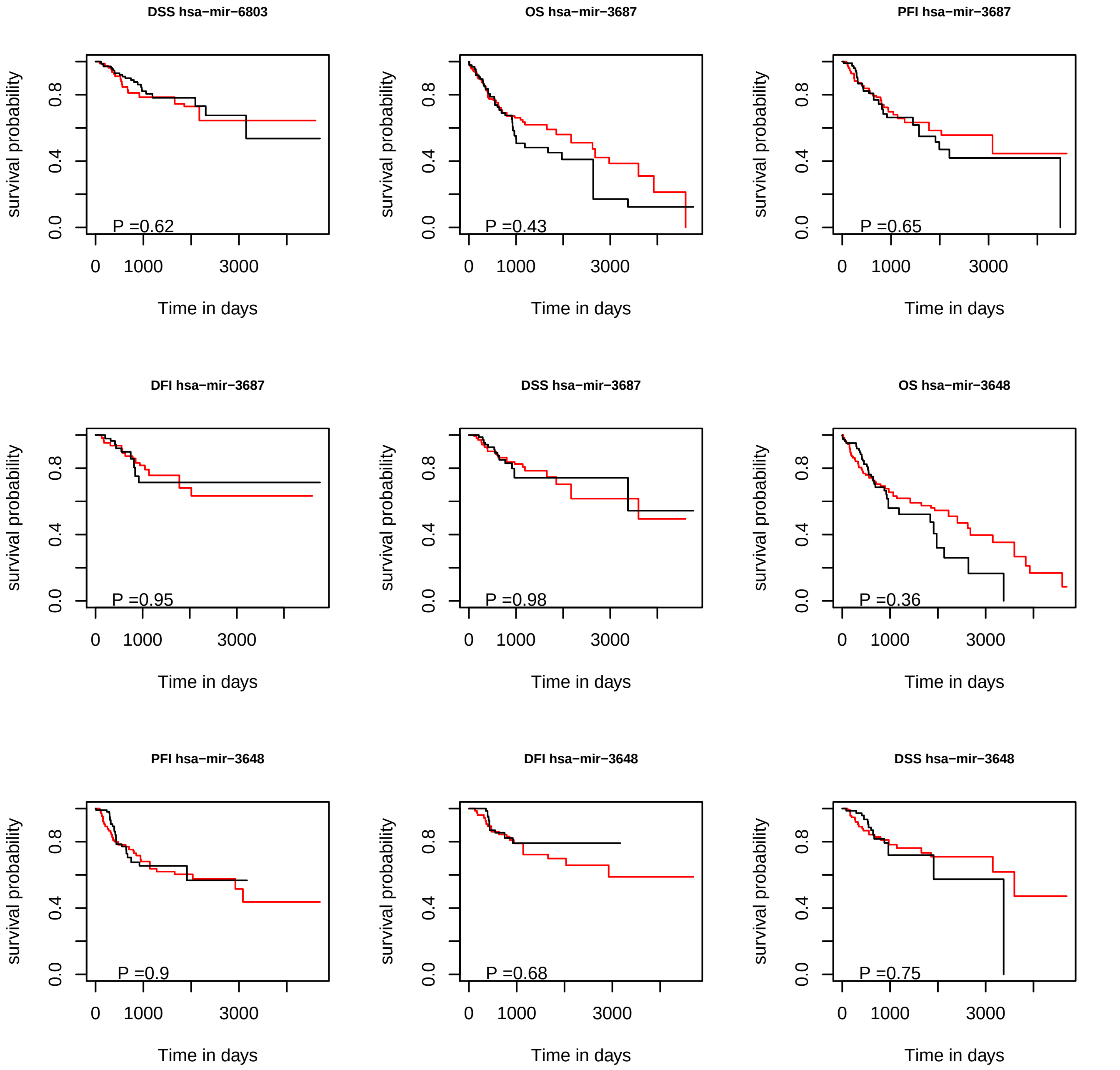
DSS hsa−mir−6803
P =0.62
OS hsa−mir−3687
P =0.43
PFI hsa−mir−3687
P =0.65
DFI hsa−mir−3687
P =0.95
DSS hsa−mir−3687
P =0.98
OS hsa−mir−3648
P =0.36
PFI hsa−mir−3648
P =0.9
DFI hsa−mir−3648
P =0.68
DSS hsa−mir−3648
P =0.75
survival probability
Time in days
0.0
0.4
0.8
0
1000
3000

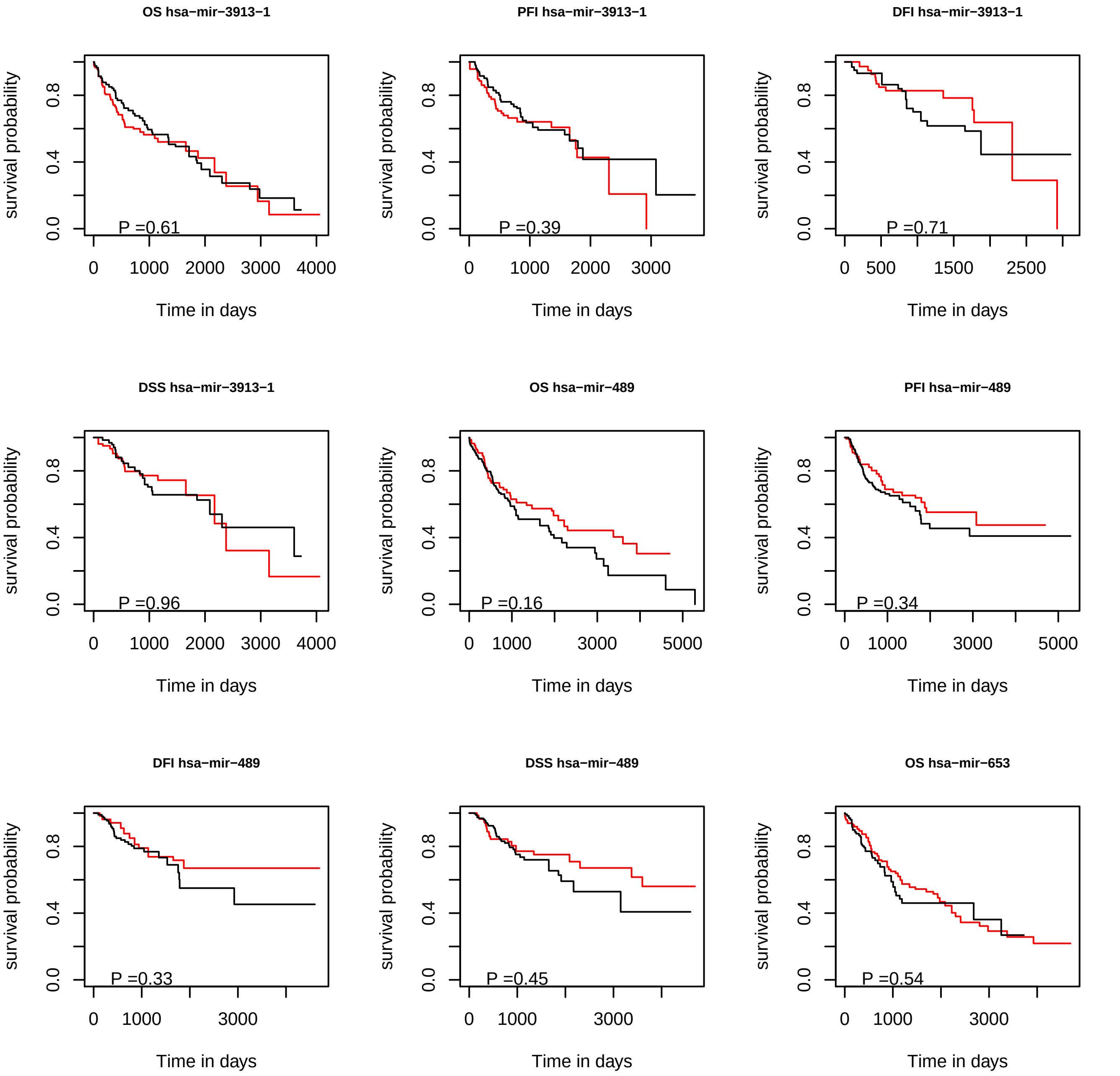

OS hsa−mir−3913−1
P =0.61
PFI hsa−mir−3913−1
P =0.39
DFI hsa−mir−3913−1
P =0.71
DSS hsa−mir−3913−1
P =0.96
OS hsa−mir−489
P =0.16
PFI hsa−mir−489
P =0.34
DFI hsa−mir−489
P =0.33
DSS hsa−mir−489
P =0.45
OS hsa−mir−653
P =0.54
survival probability
Time in days
0.0
0.4
0.8
0
500
1000
1500
2000
2500
3000
4000
5000

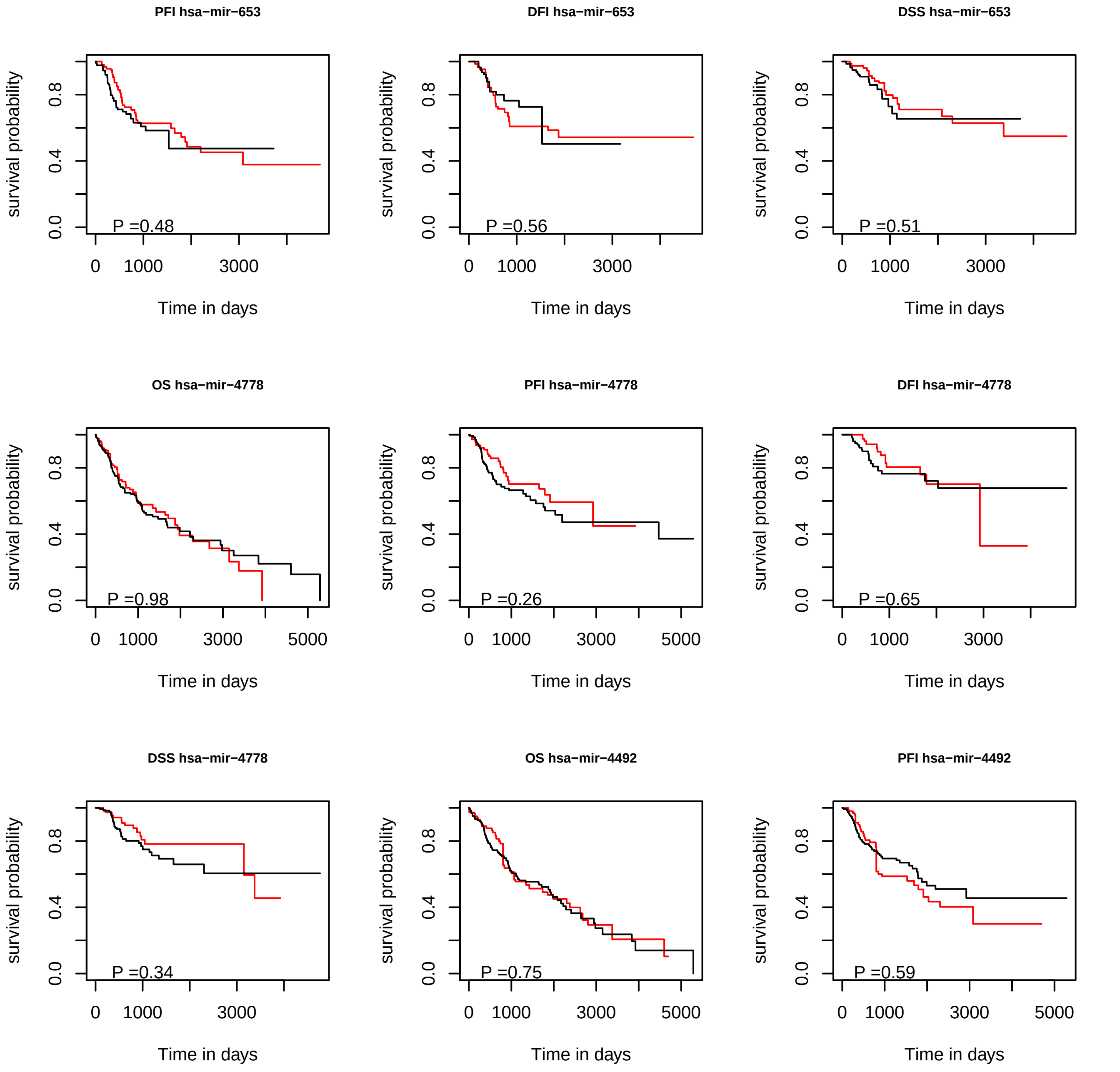
PFI hsa−mir−653
P =0.48
DFI hsa−mir−653
P =0.56
DSS hsa−mir−653
P =0.51
OS hsa−mir−4778
P =0.98
PFI hsa−mir−4778
P =0.26
DFI hsa−mir−4778
P =0.65
DSS hsa−mir−4778
P =0.34
OS hsa−mir−4492
P =0.75
PFI hsa−mir−4492
P =0.59
survival probability
Time in days
0.0
0.4
0.8
0
1000
3000
5000

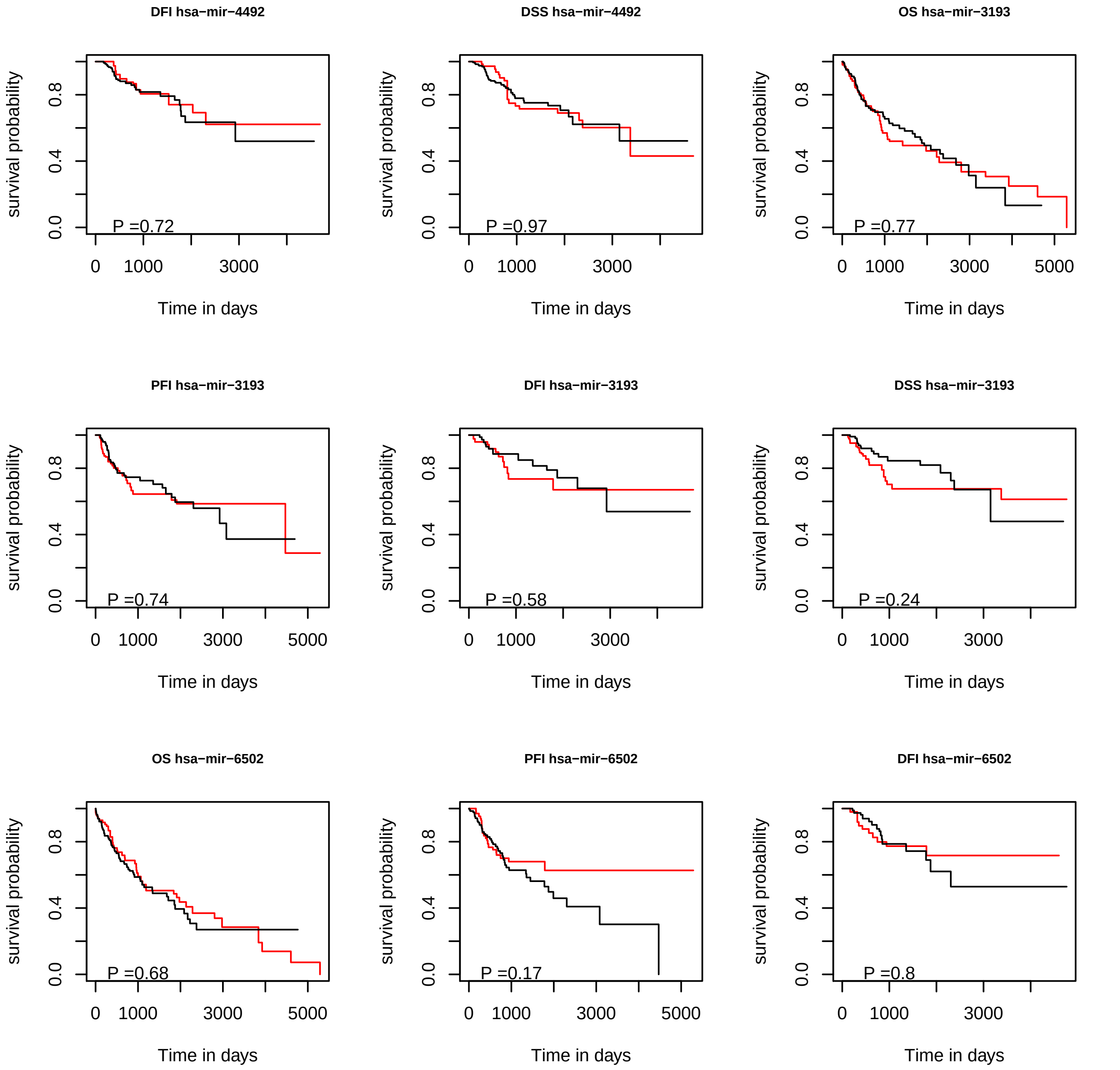
DFI hsa−mir−4492
P =0.72
DSS hsa−mir−4492
P =0.97
OS hsa−mir−3193
P =0.77
PFI hsa−mir−3193
P =0.74
DFI hsa−mir−3193
P =0.58
DSS hsa−mir−3193
P =0.24
OS hsa−mir−6502
P =0.68
PFI hsa−mir−6502
P =0.17
DFI hsa−mir−6502
P =0.8
survival probability
0.0
0.4
0.8
Time in days
0
1000
3000
5000

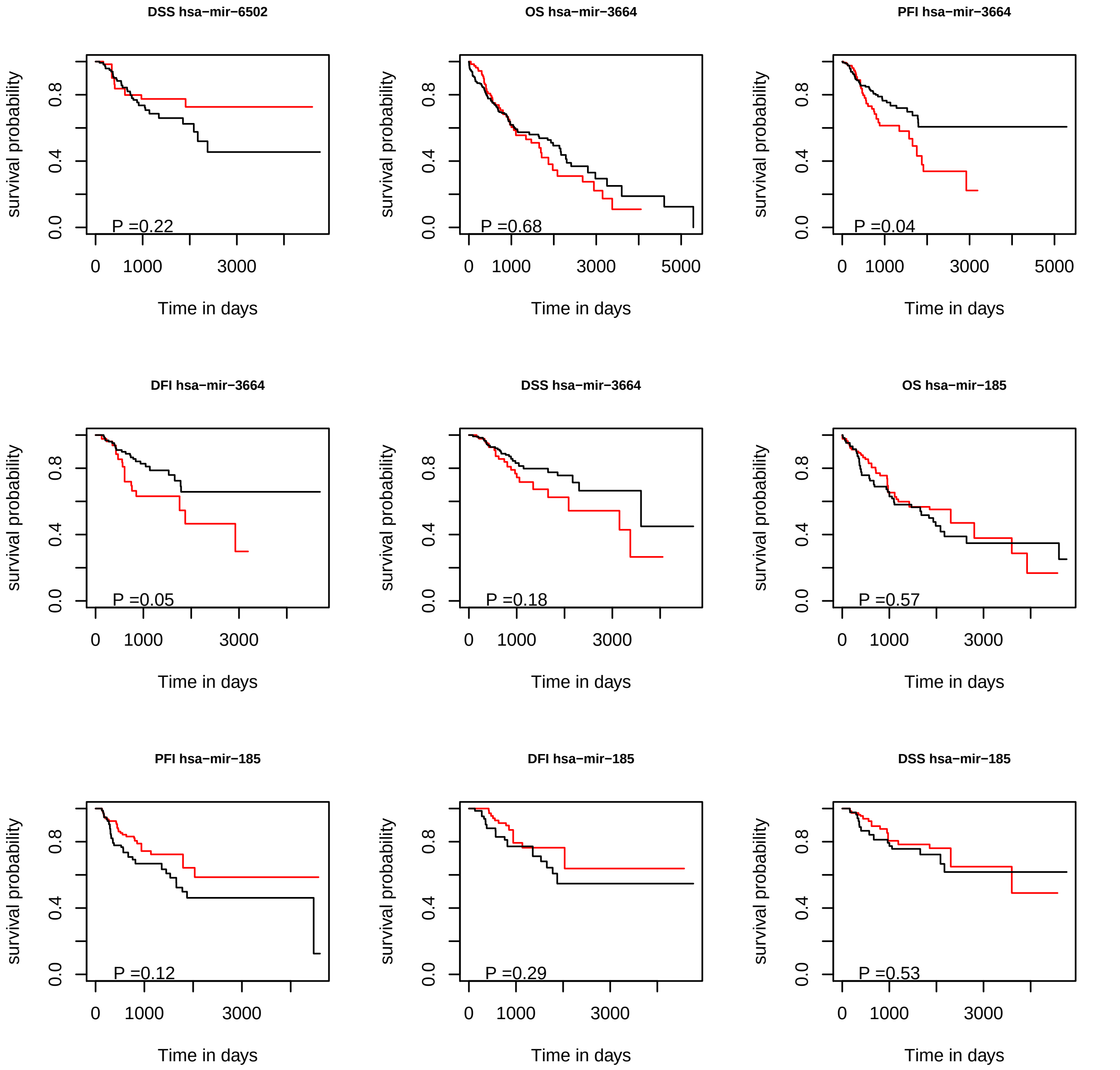
DSS hsa−mir−6502
P =0.22
OS hsa−mir−3664
P =0.68
PFI hsa−mir−3664
P =0.04
DFI hsa−mir−3664
P =0.05
DSS hsa−mir−3664
P =0.18
OS hsa−mir−185
P =0.57
PFI hsa−mir−185
P =0.12
DFI hsa−mir−185
P =0.29
DSS hsa−mir−185
P =0.53
survival probability
0.0
0.4
0.8
Time in days
0
1000
3000
5000

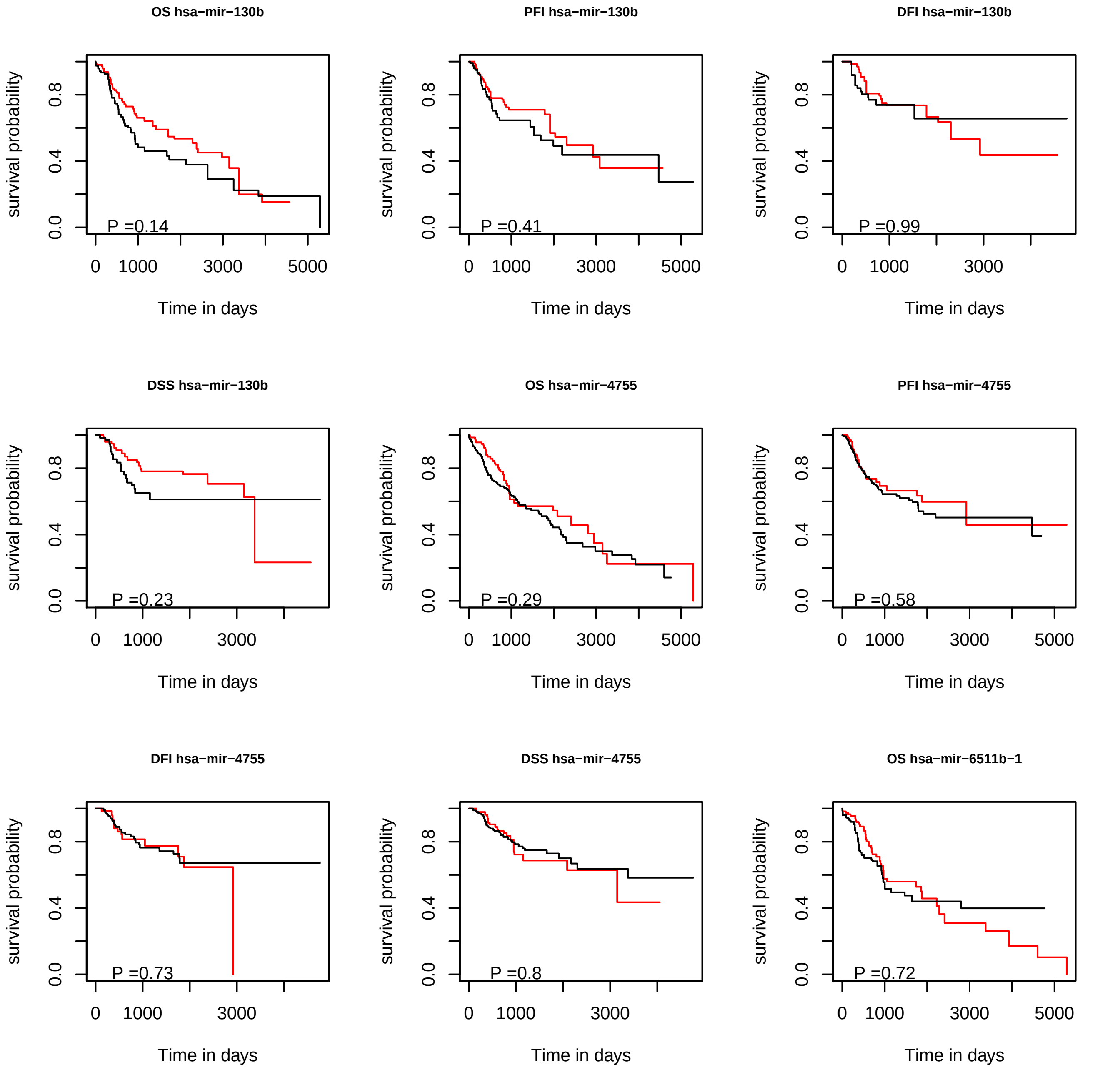

OS hsa−mir−130b
P =0.14
PFI hsa−mir−130b
P =0.41
DFI hsa−mir−130b
P =0.99
DSS hsa−mir−130b
P =0.23
OS hsa−mir−4755
P =0.29
PFI hsa−mir−4755
P =0.58
DFI hsa−mir−4755
P =0.73
DSS hsa−mir−4755
P =0.8
OS hsa−mir−6511b−1
P =0.72
survival probability
Time in days
0.0
0.4
0.8
0
1000
3000
5000

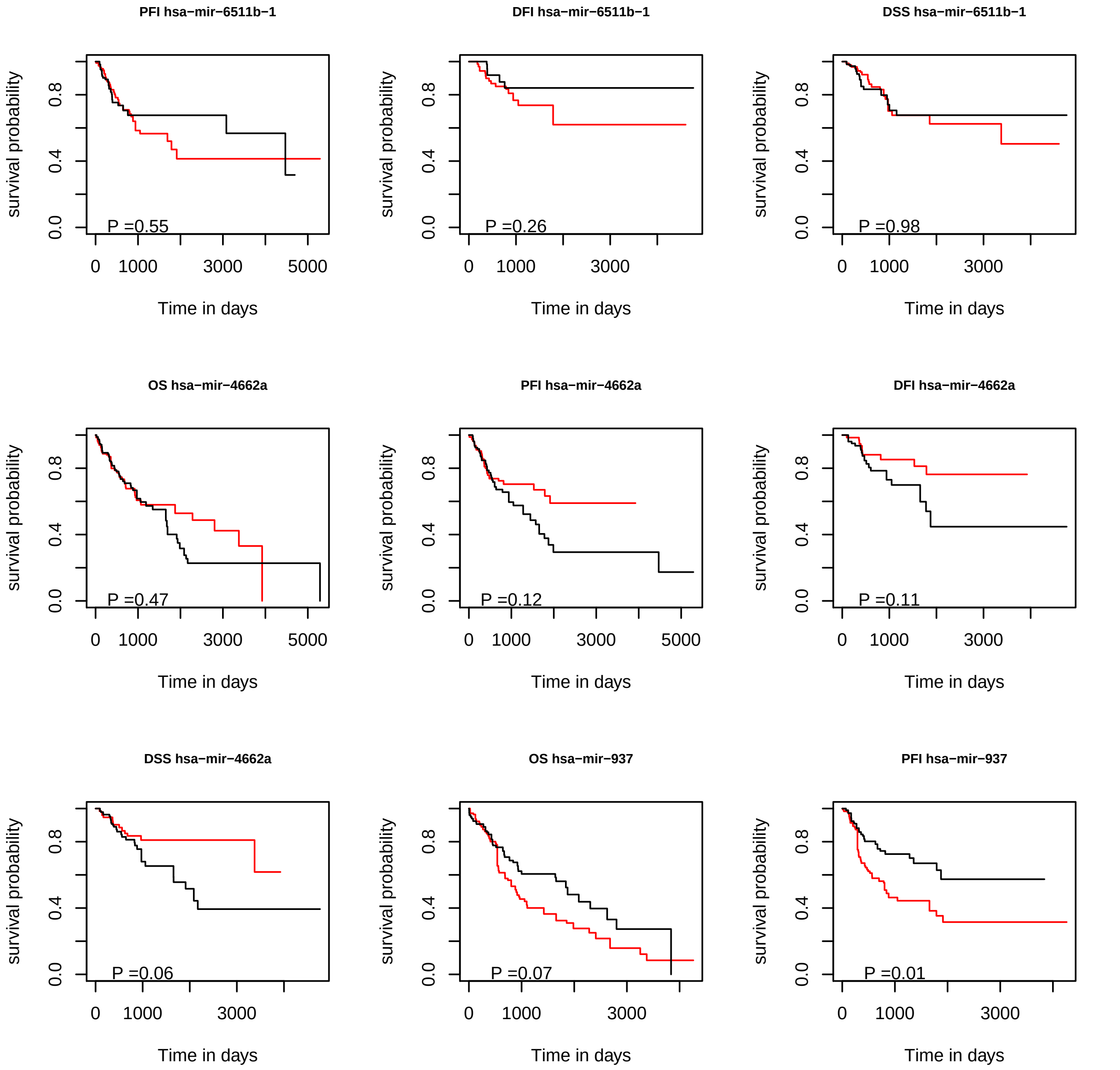
PFI hsa−mir−6511b−1
P =0.55
DFI hsa−mir−6511b−1
P =0.26
DSS hsa−mir−6511b−1
P =0.98
OS hsa−mir−4662a
P =0.47
PFI hsa−mir−4662a
P =0.12
DFI hsa−mir−4662a
P =0.11
DSS hsa−mir−4662a
P =0.06
OS hsa−mir−937
P =0.07
PFI hsa−mir−937
P =0.01
survival probability
Time in days

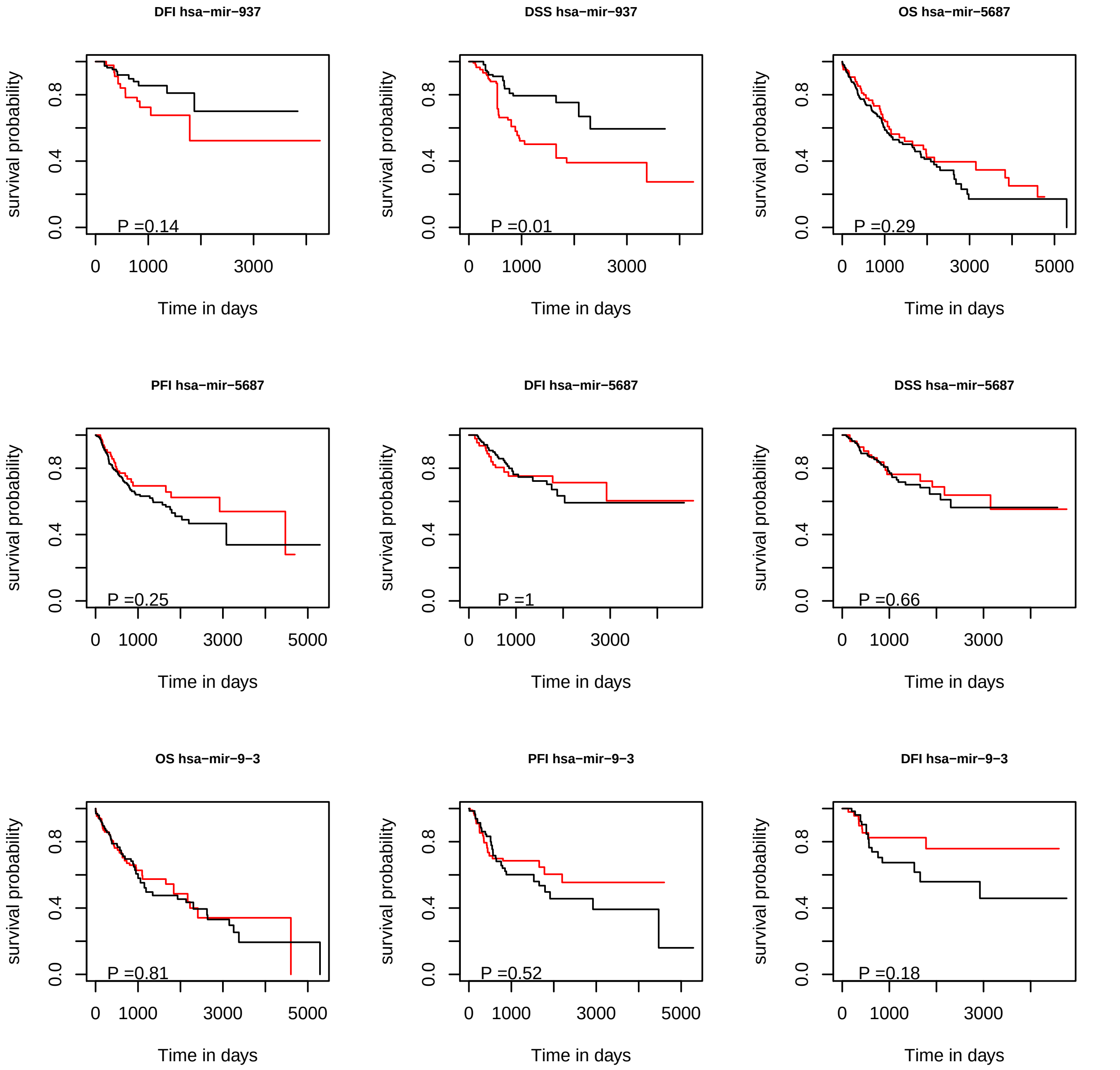
DFI hsa−mir−937
survival probability
0.0
0.4
0.8
P =0.14
0
1000
3000
Time in days
DSS hsa−mir−937
survival probability
0.0
0.4
0.8
P =0.01
0
1000
3000
Time in days
OS hsa−mir−5687
survival probability
0.0
0.4
0.8
P =0.29
0
1000
3000
5000
Time in days
PFI hsa−mir−5687
survival probability
0.0
0.4
0.8
P =0.25
0
1000
3000
5000
Time in days
DFI hsa−mir−5687
survival probability
0.0
0.4
0.8
P =1
0
1000
3000
Time in days
DSS hsa−mir−5687
survival probability
0.0
0.4
0.8
P =0.66
0
1000
3000
Time in days
OS hsa−mir−9−3
survival probability
0.0
0.4
0.8
P =0.81
0
1000
3000
5000
Time in days
PFI hsa−mir−9−3
survival probability
0.0
0.4
0.8
P =0.52
0
1000
3000
5000
Time in days
DFI hsa−mir−9−3
survival probability
0.0
0.4
0.8
P =0.18
0
1000
3000
Time in days

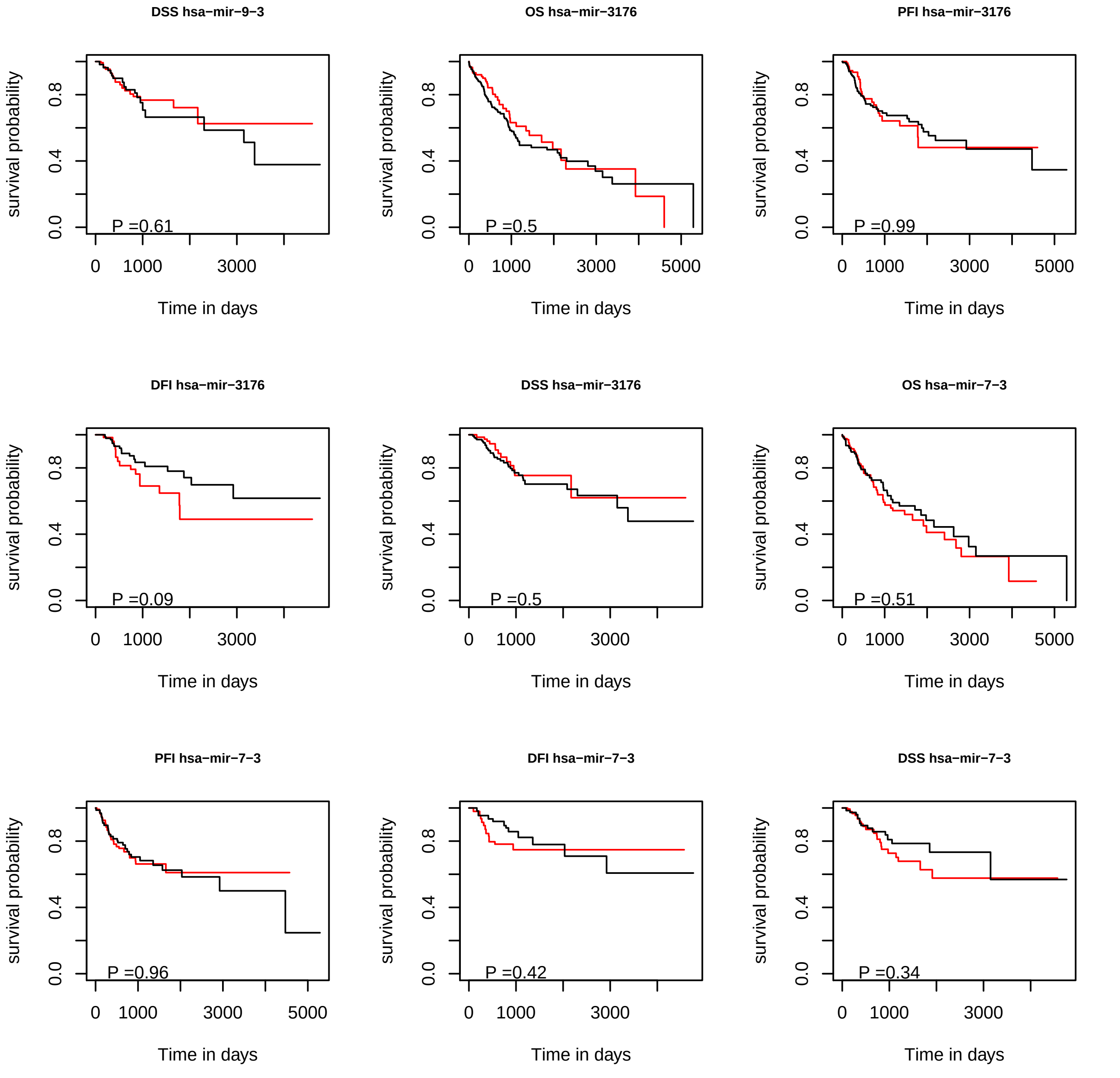
DSS hsa−mir−9−3
P =0.61
OS hsa−mir−3176
P =0.5
PFI hsa−mir−3176
P =0.99
DFI hsa−mir−3176
P =0.09
DSS hsa−mir−3176
P =0.5
OS hsa−mir−7−3
P =0.51
PFI hsa−mir−7−3
P =0.96
DFI hsa−mir−7−3
P =0.42
DSS hsa−mir−7−3
P =0.34
survival probability
0.0
0.4
0.8
Time in days
0
1000
3000
5000

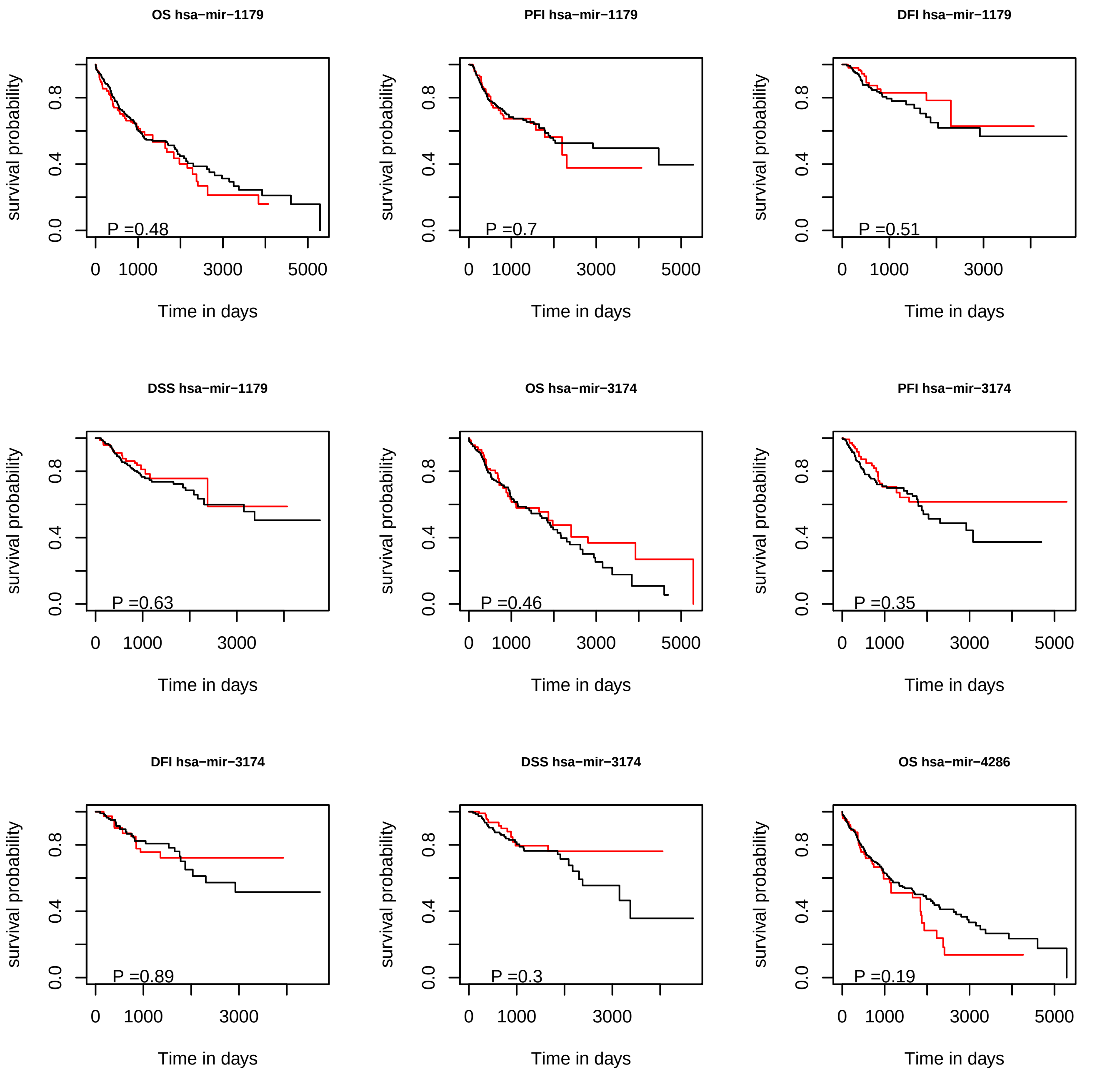
OS hsa−mir−1179
P =0.48
PFI hsa−mir−1179
P =0.7
DFI hsa−mir−1179
P =0.51
DSS hsa−mir−1179
P =0.63
OS hsa−mir−3174
P =0.46
PFI hsa−mir−3174
P =0.35
DFI hsa−mir−3174
P =0.89
DSS hsa−mir−3174
P =0.3
OS hsa−mir−4286
P =0.19
survival probability
0.0
0.4
0.8
Time in days
0
1000
3000
5000

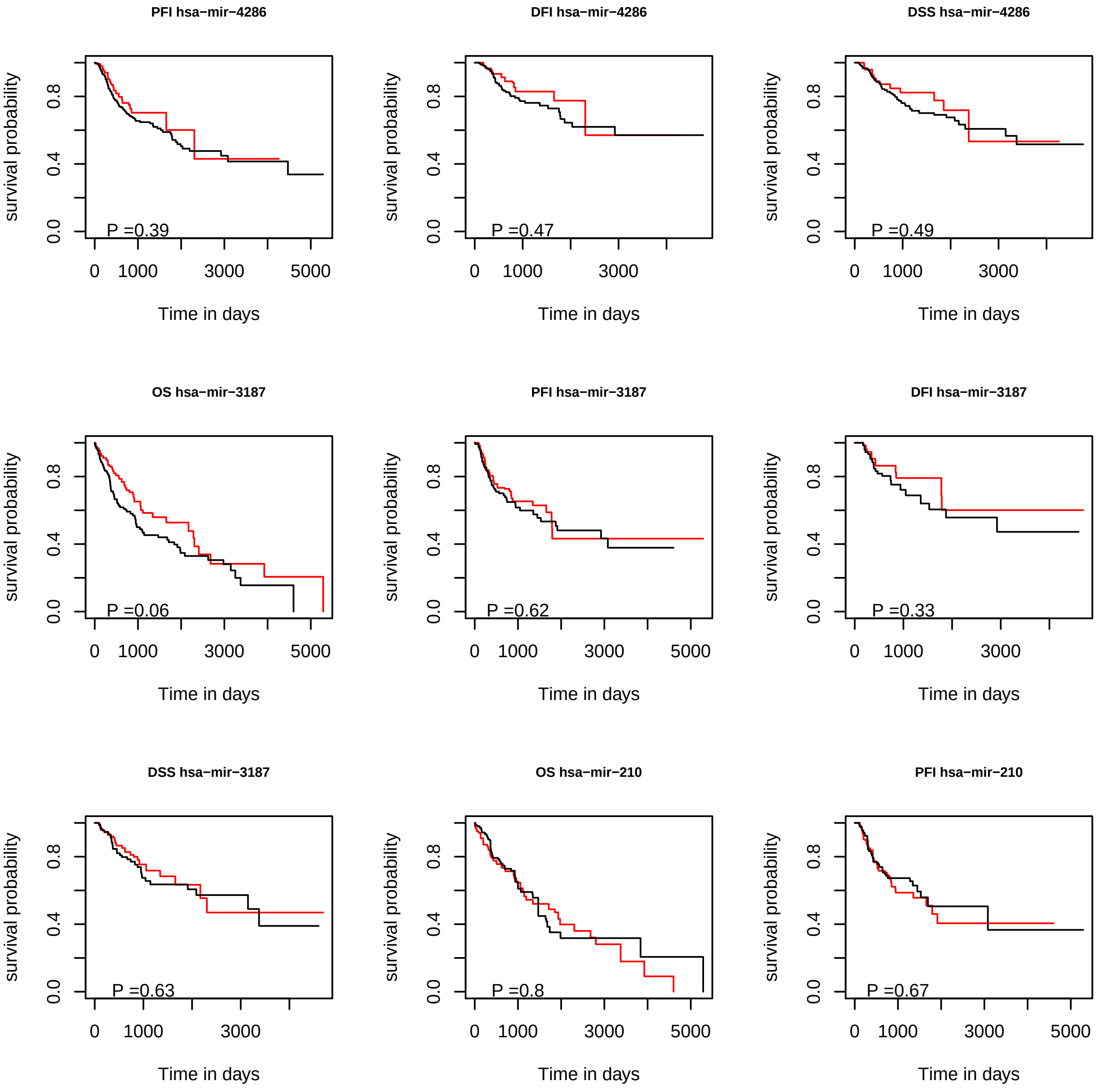
PFI hsa−mir−4286
P =0.39
DFI hsa−mir−4286
P =0.47
DSS hsa−mir−4286
P =0.49
OS hsa−mir−3187
P =0.06
PFI hsa−mir−3187
P =0.62
DFI hsa−mir−3187
P =0.33
DSS hsa−mir−3187
P =0.63
OS hsa−mir−210
P =0.8
PFI hsa−mir−210
P =0.67
survival probability
Time in days
0.0
0.4
0.8
0
1000
3000
5000

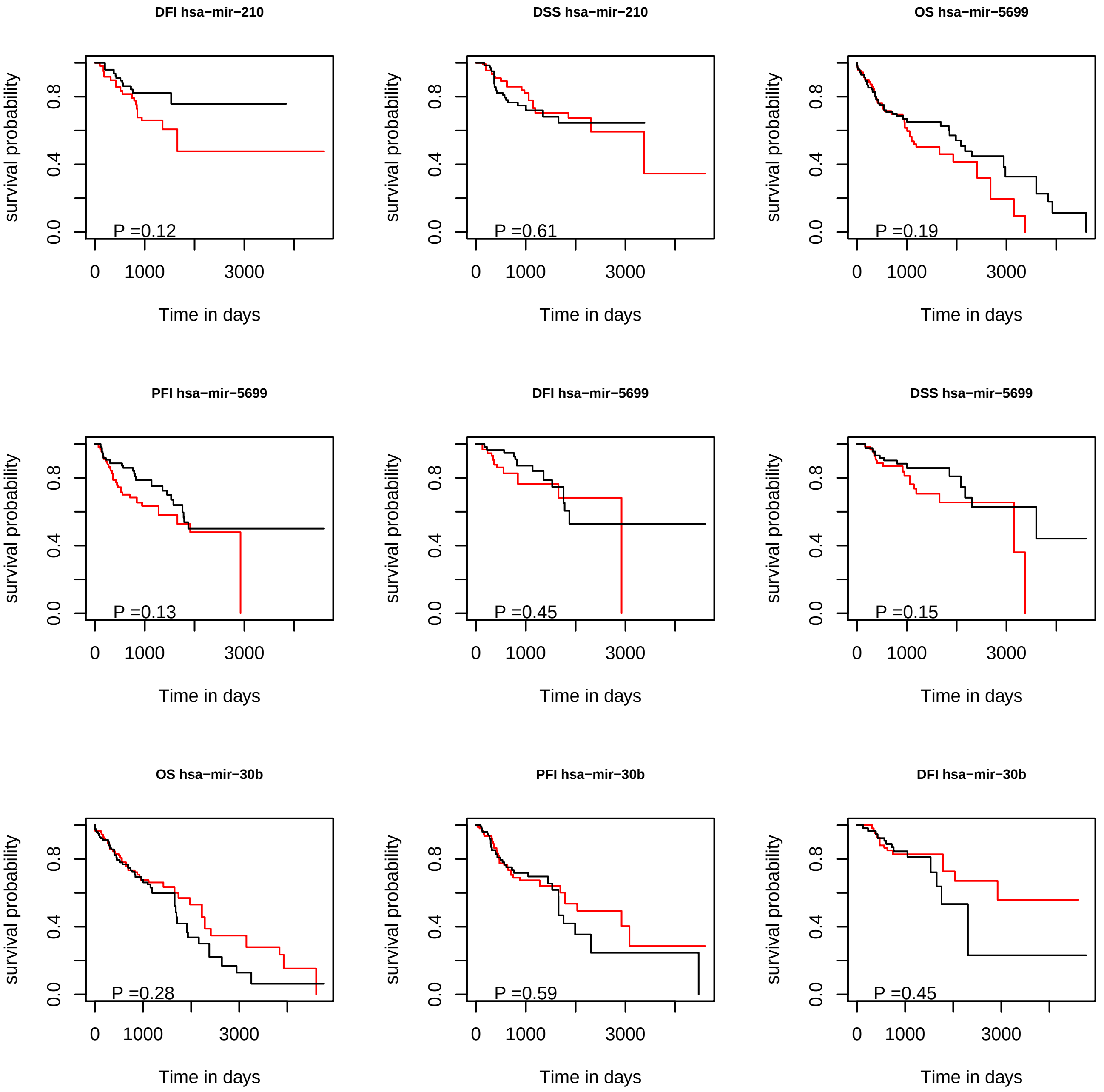

DFI hsa−mir−210
P =0.12
DSS hsa−mir−210
P =0.61
OS hsa−mir−5699
P =0.19
PFI hsa−mir−5699
P =0.13
DFI hsa−mir−5699
P =0.45
DSS hsa−mir−5699
P =0.15
OS hsa−mir−30b
P =0.28
PFI hsa−mir−30b
P =0.59
DFI hsa−mir−30b
P =0.45
survival probability
0.0
0.4
0.8
0
1000
3000
Time in days

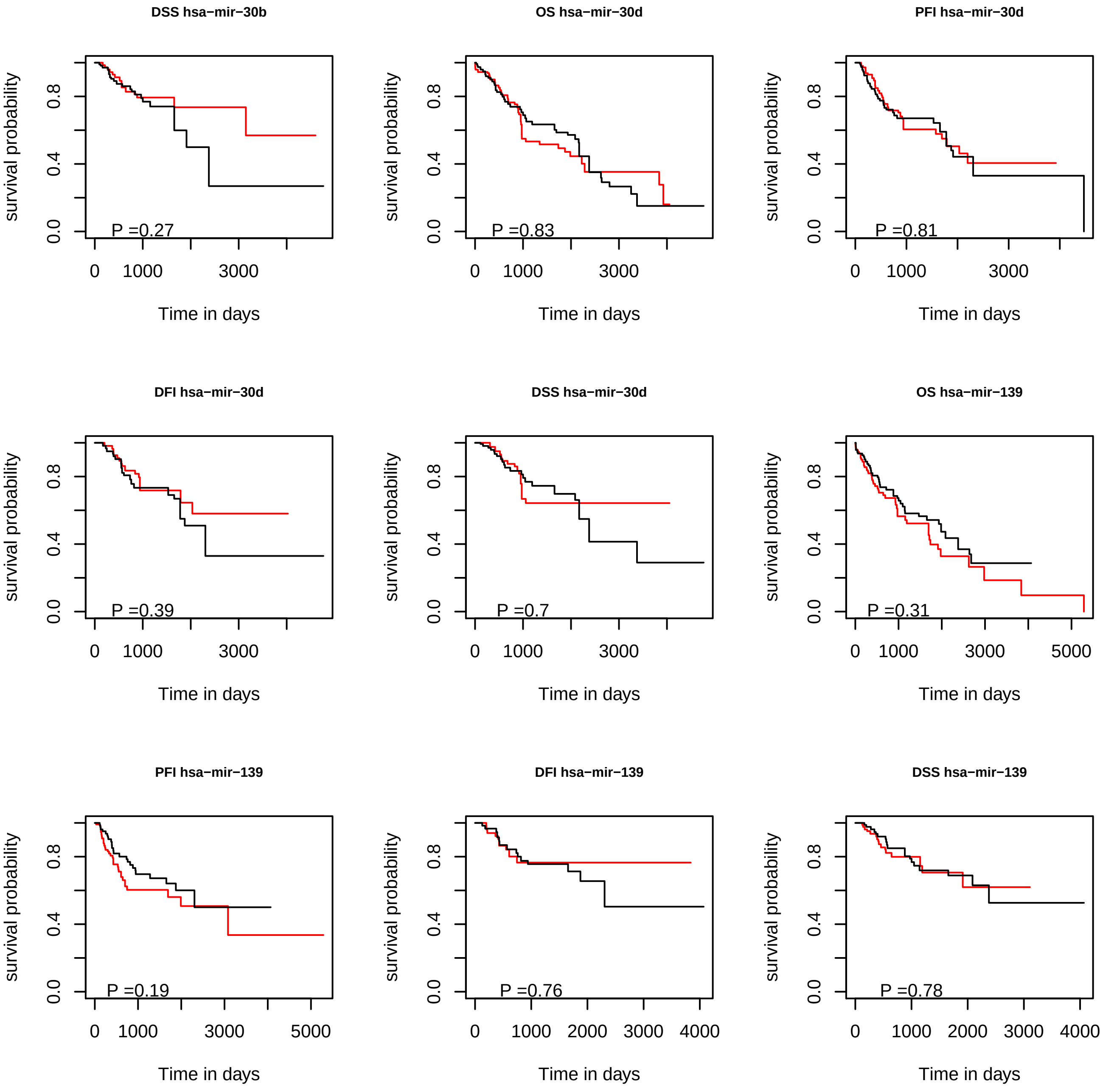
DSS hsa−mir−30b
P =0.27
OS hsa−mir−30d
P =0.83
PFI hsa−mir−30d
P =0.81
DFI hsa−mir−30d
P =0.39
DSS hsa−mir−30d
P =0.7
OS hsa−mir−139
P =0.31
PFI hsa−mir−139
P =0.19
DFI hsa−mir−139
P =0.76
DSS hsa−mir−139
P =0.78
survival probability
Time in days
0.0
0.4
0.8
0
1000
2000
3000
4000
5000

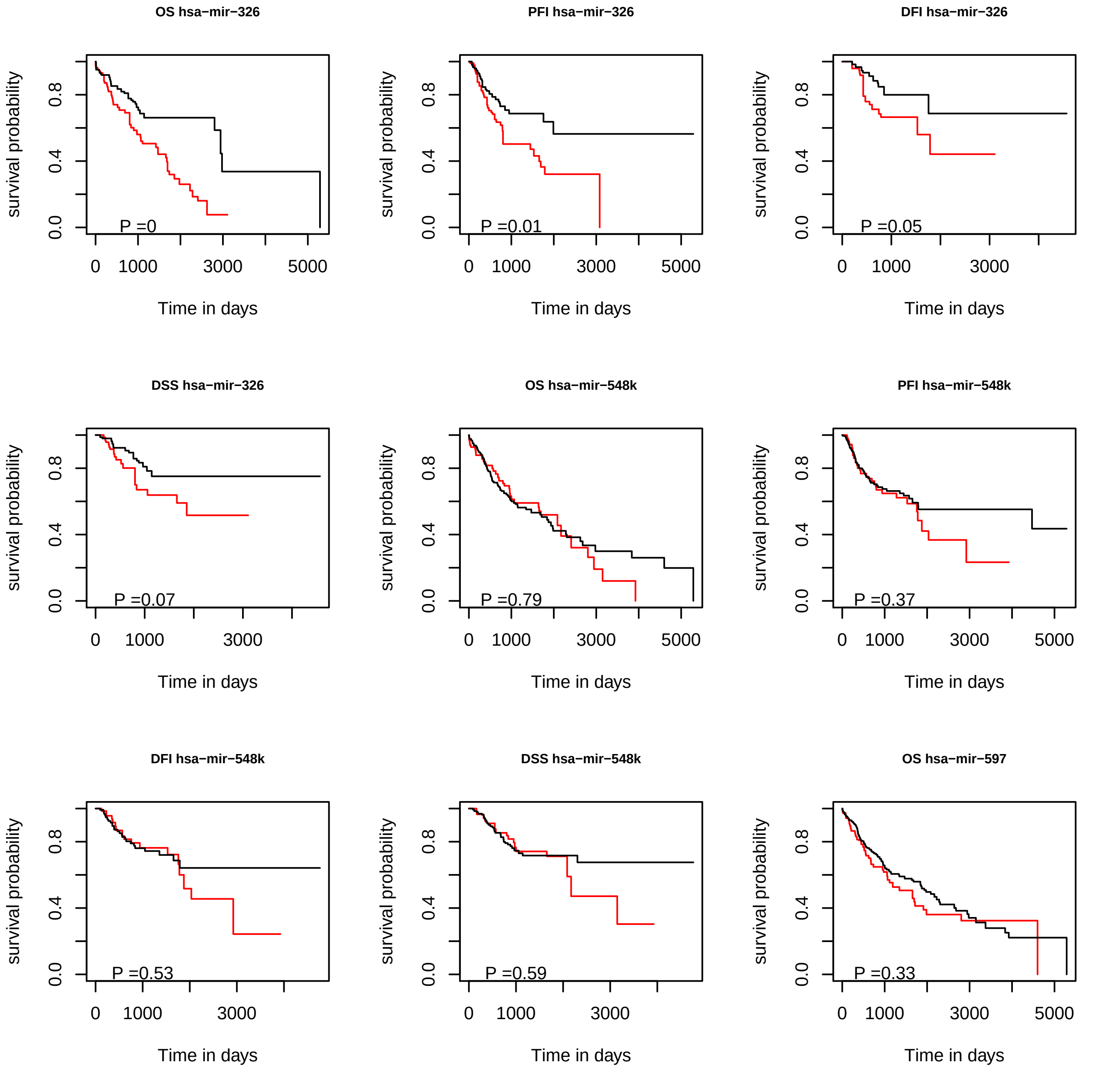
OS hsa−mir−326
P =0
PFI hsa−mir−326
P =0.01
DFI hsa−mir−326
P =0.05
DSS hsa−mir−326
P =0.07
OS hsa−mir−548k
P =0.79
PFI hsa−mir−548k
P =0.37
DFI hsa−mir−548k
P =0.53
DSS hsa−mir−548k
P =0.59
OS hsa−mir−597
P =0.33
survival probability
Time in days
0.0
0.4
0.8
0
1000
3000
5000

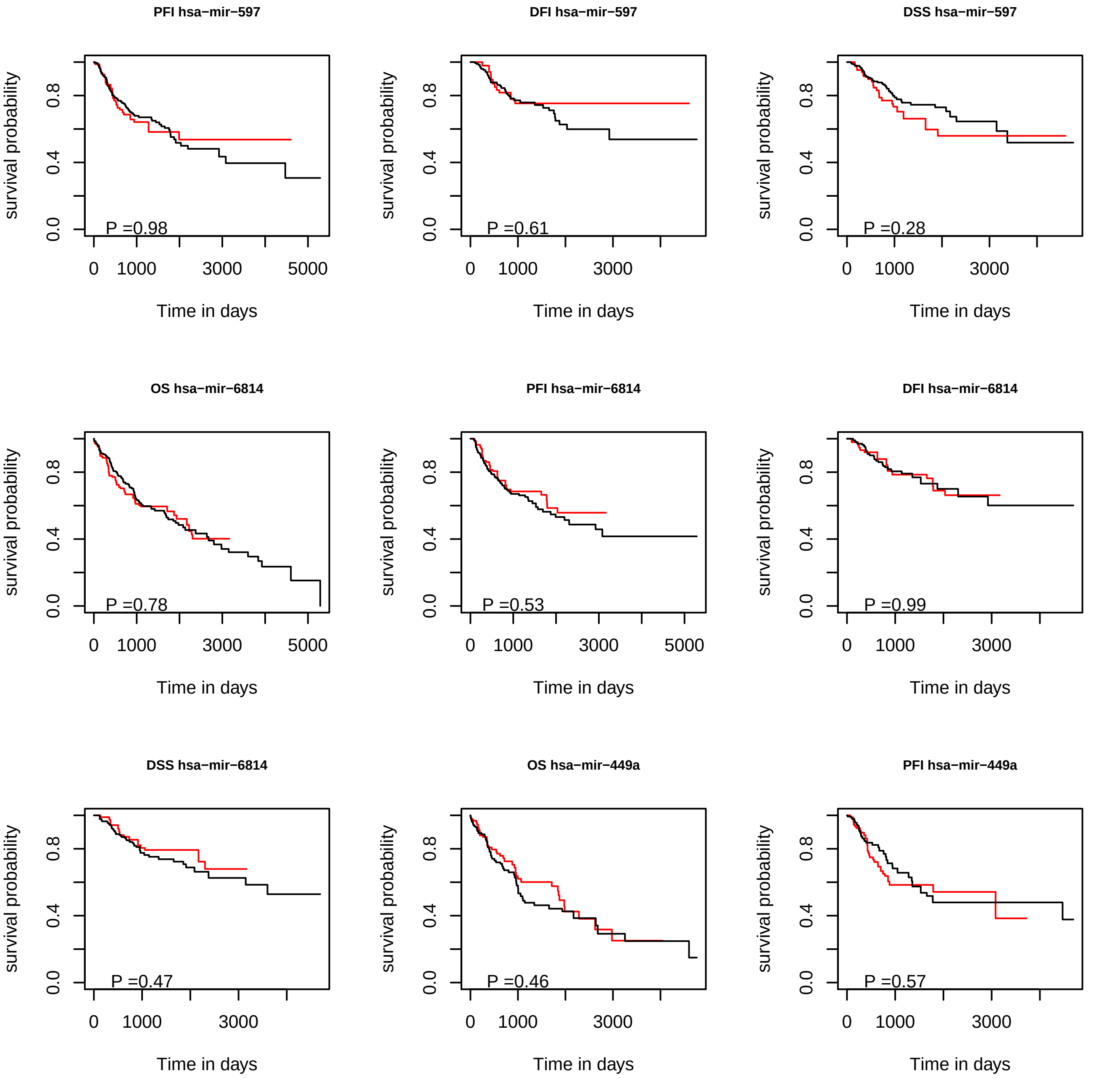

PFI hsa−mir−597
P =0.98
DFI hsa−mir−597
P =0.61
DSS hsa−mir−597
P =0.28
OS hsa−mir−6814
P =0.78
PFI hsa−mir−6814
P =0.53
DFI hsa−mir−6814
P =0.99
DSS hsa−mir−6814
P =0.47
OS hsa−mir−449a
P =0.46
PFI hsa−mir−449a
P =0.57
survival probability
Time in days
0.0
0.4
0.8
0
1000
3000
5000

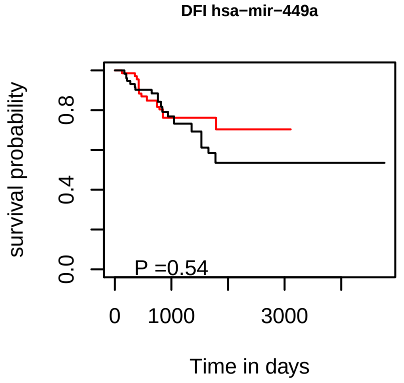
DFI hsa−mir−449a
survival probability
0.0
0.4
0.8
P =0.54
0
1000
3000
Time in days

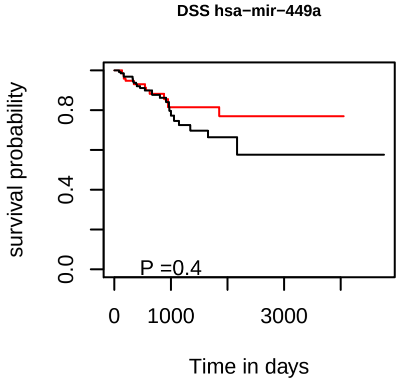
DSS hsa−mir−449a
survival probability
0.0
0.4
0.8
P =0.4
0
1000
3000
Time in days

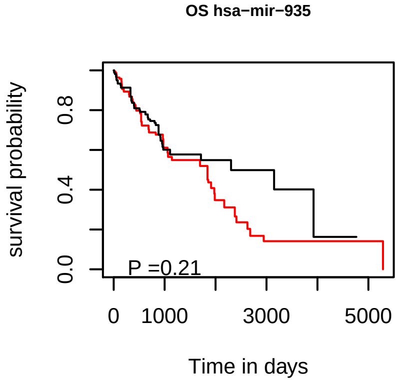
OS hsa−mir−935
survival probability
0.0
0.4
0.8
P =0.21
0
1000
3000
5000
Time in days

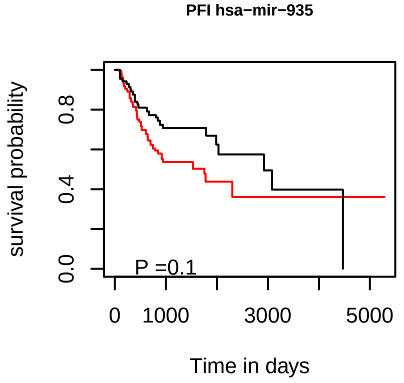
PFI hsa−mir−935
survival probability
0.0
0.4
0.8
P =0.1
0
1000
3000
5000
Time in days

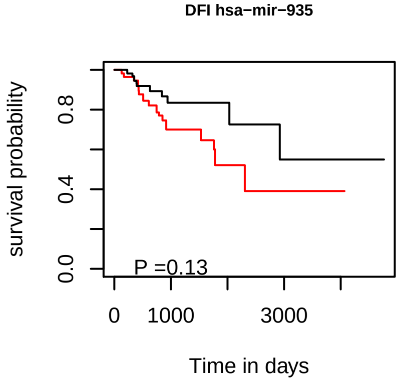
DFI hsa−mir−935
survival probability
0.0
0.4
0.8
P =0.13
0
1000
3000
Time in days

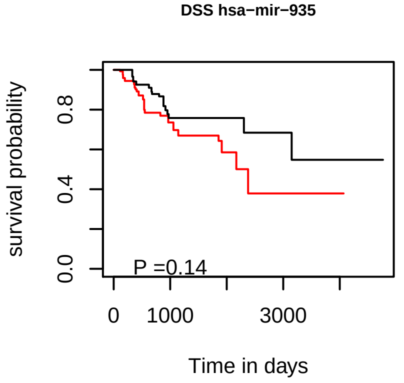
DSS hsa−mir−935
survival probability
0.0
0.4
0.8
P =0.14
0
1000
3000
Time in days
