# Supplementary material for: Computing microRNA-gene interaction networks in pan-cancer using miRDriver
Source: Sci Rep. 2022 Mar 8;12:3717. doi: 10.1038/s41598-022-07628-z (PMC8904490; doi:10.1038/s41598-022-07628-z)
Supplement: Supplementary file 23 — Supplementary Information 23. [file 41598_2022_7628_MOESM23_ESM.pdf]

Computing microRNA-gene interaction networks in pan-cancer using miRDriver

Banabithi Bose, Matthew Moravec, and Serdar Bozdag

Supplemental Figure S14

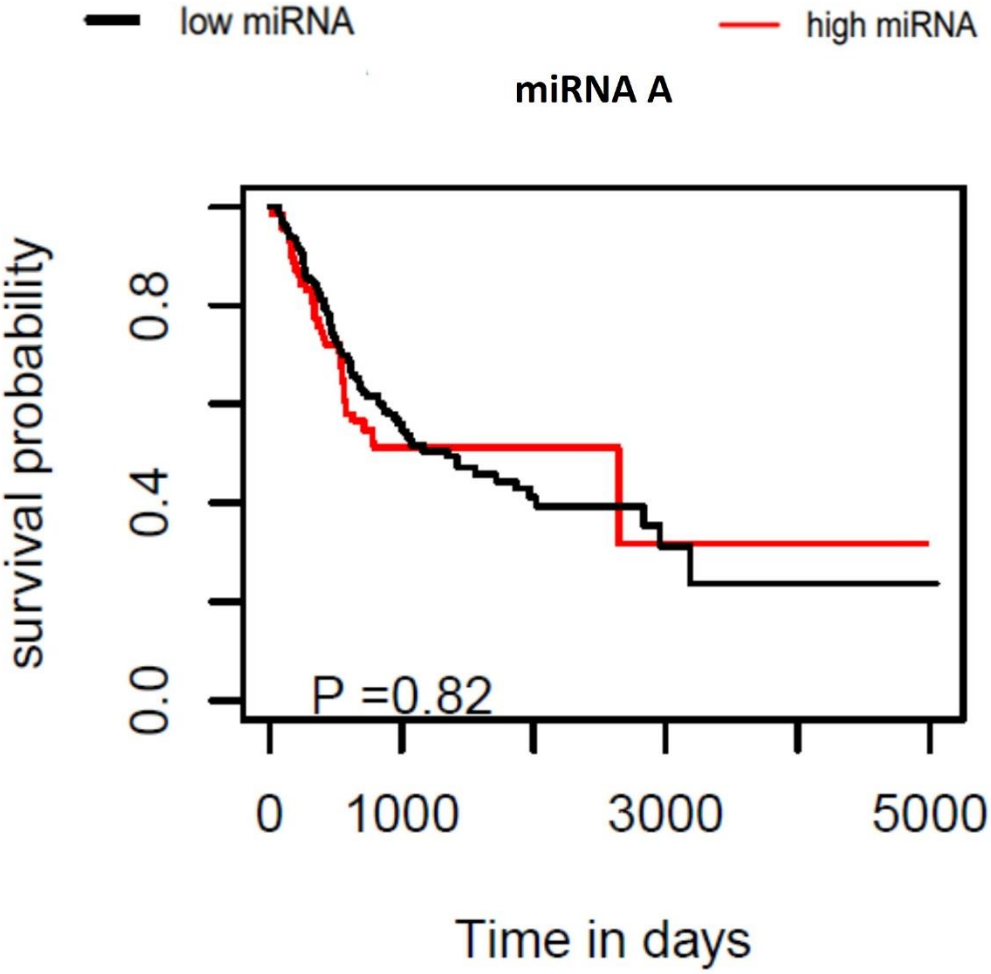

The *Adjusted Kaplan-Meier* survival plots for the computed miRNAs in high and low miRNA expression patient groups.

Supplemental Figure S14

Cancer Type: OV

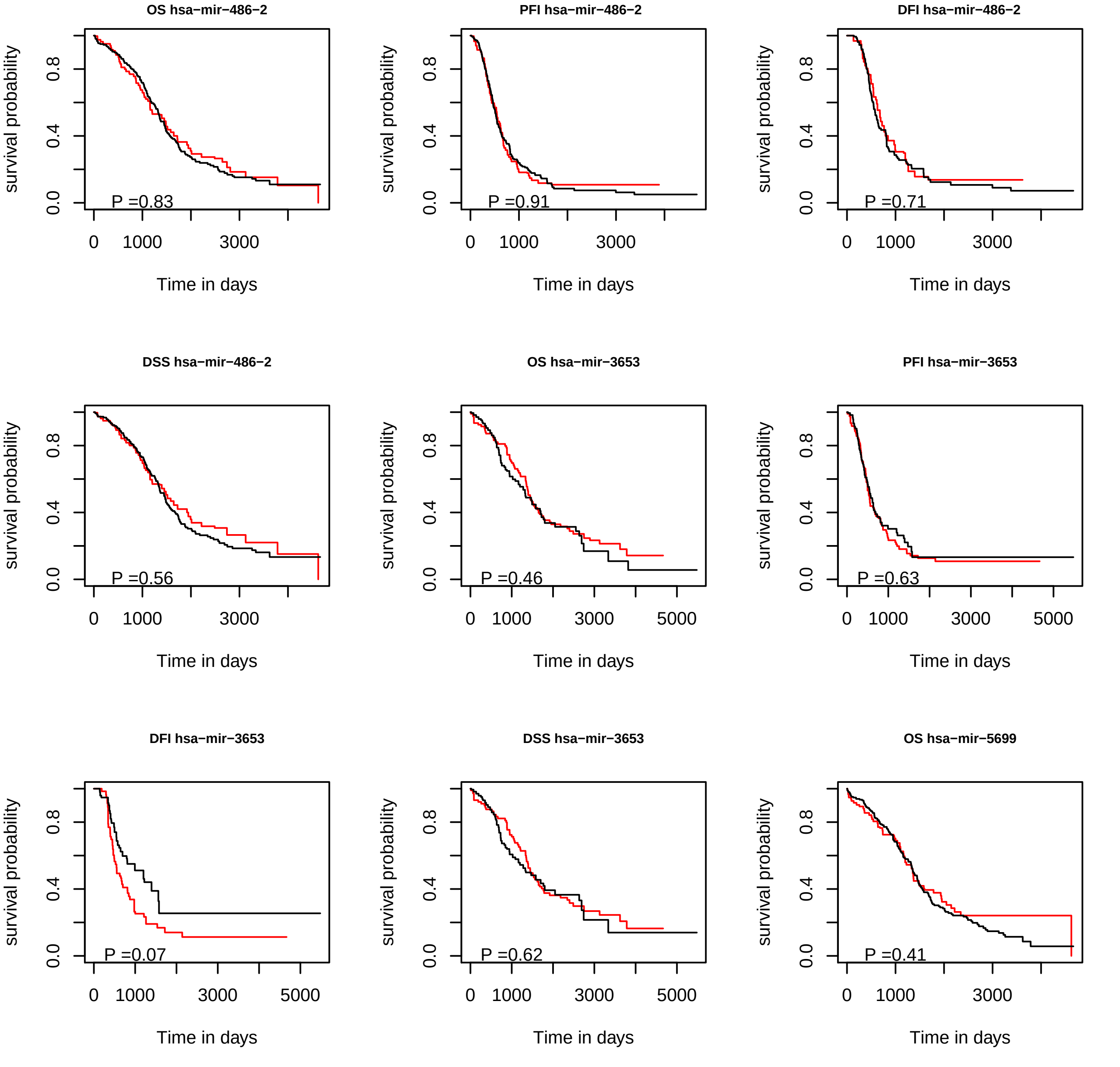

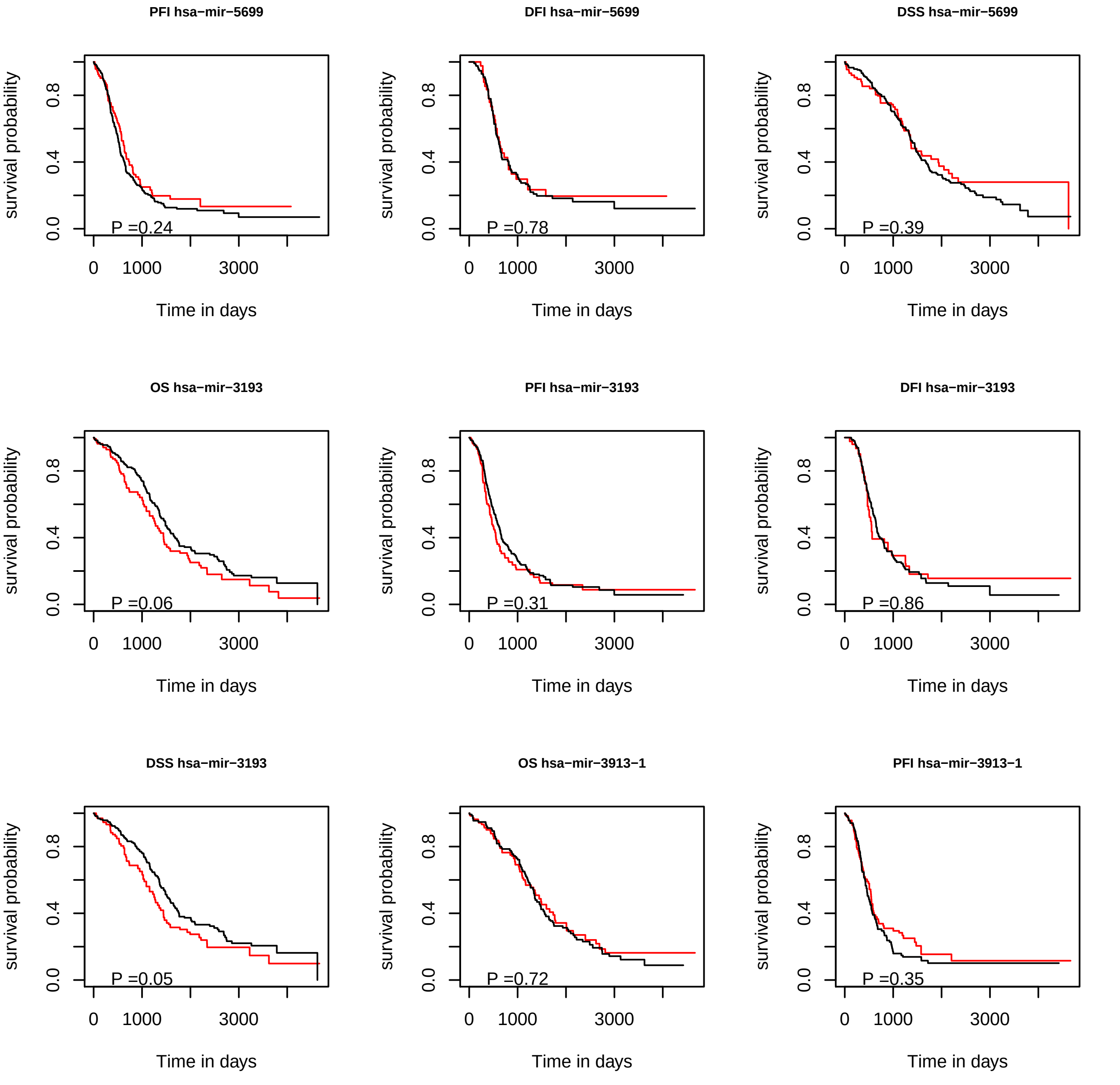

PFI hsa−mir−5699
P =0.24
DFI hsa−mir−5699
P =0.78
DSS hsa−mir−5699
P =0.39
OS hsa−mir−3193
P =0.06
PFI hsa−mir−3193
P =0.31
DFI hsa−mir−3193
P =0.86
DSS hsa−mir−3193
P =0.05
OS hsa−mir−3913−1
P =0.72
PFI hsa−mir−3913−1
P =0.35
survival probability
0.0
0.4
0.8
0
1000
3000
Time in days

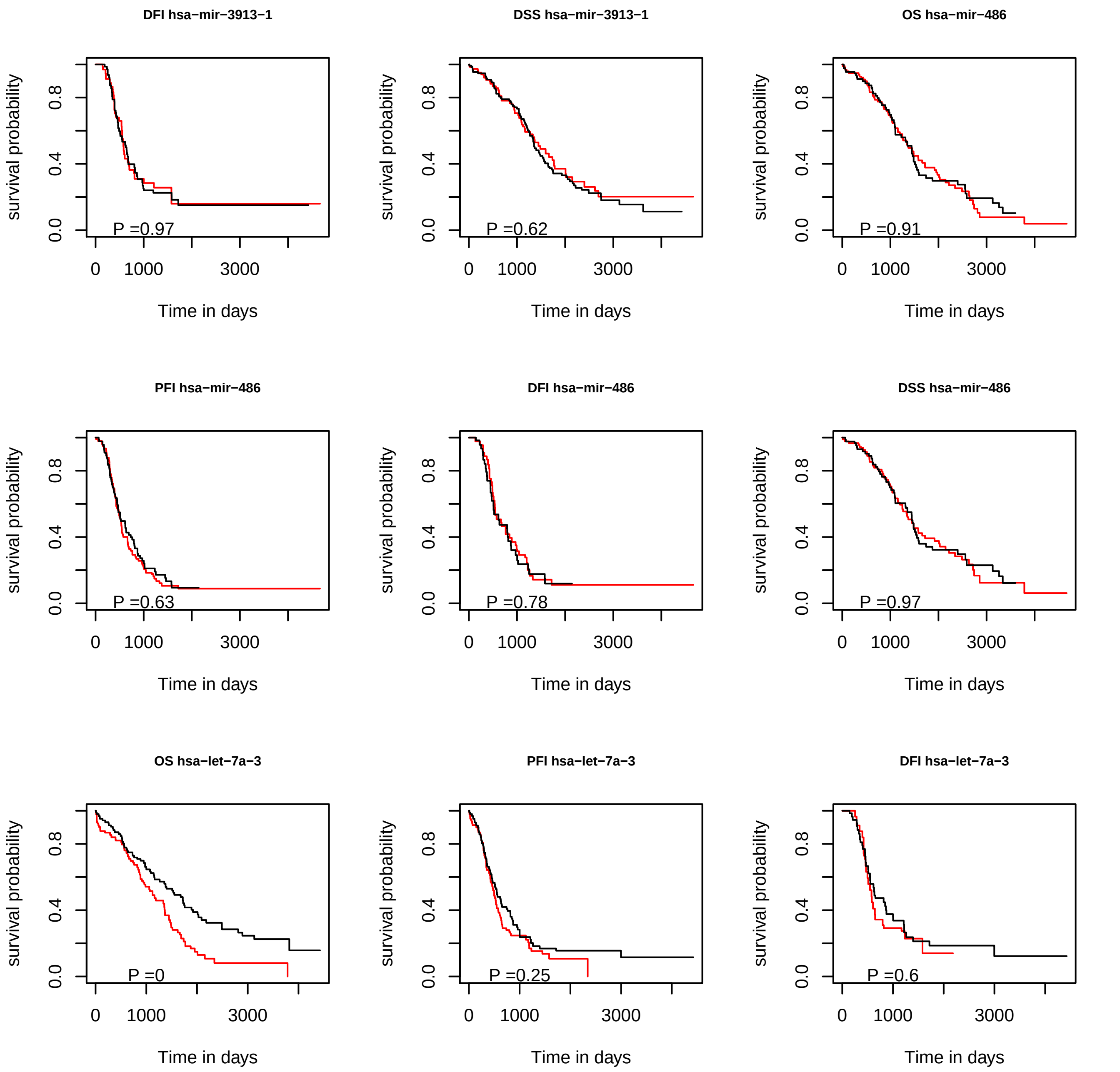

DFI hsa−mir−3913−1
P =0.97
DSS hsa−mir−3913−1
P =0.62
OS hsa−mir−486
P =0.91
PFI hsa−mir−486
P =0.63
DFI hsa−mir−486
P =0.78
DSS hsa−mir−486
P =0.97
OS hsa−let−7a−3
P =0
PFI hsa−let−7a−3
P =0.25
DFI hsa−let−7a−3
P =0.6
survival probability
0.0
0.4
0.8
0
1000
3000
Time in days

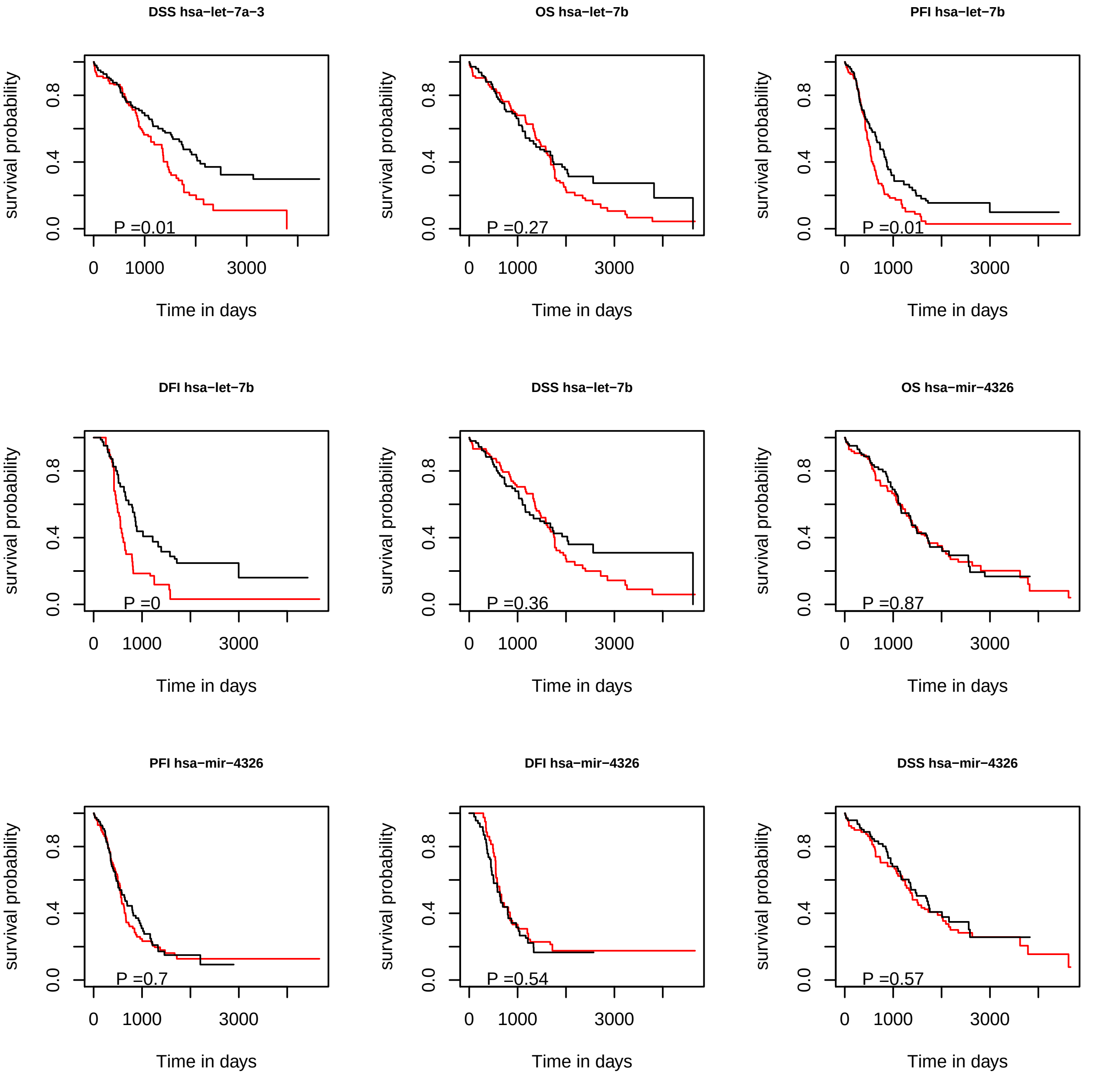
DSS hsa−let−7a−3
survival probability
0.0
0.4
0.8
P =0.01
0
1000
3000
Time in days
OS hsa−let−7b
survival probability
0.0
0.4
0.8
P =0.27
0
1000
3000
Time in days
PFI hsa−let−7b
survival probability
0.0
0.4
0.8
P =0.01
0
1000
3000
Time in days
DFI hsa−let−7b
survival probability
0.0
0.4
0.8
P =0
0
1000
3000
Time in days
DSS hsa−let−7b
survival probability
0.0
0.4
0.8
P =0.36
0
1000
3000
Time in days
OS hsa−mir−4326
survival probability
0.0
0.4
0.8
P =0.87
0
1000
3000
Time in days
PFI hsa−mir−4326
survival probability
0.0
0.4
0.8
P =0.7
0
1000
3000
Time in days
DFI hsa−mir−4326
survival probability
0.0
0.4
0.8
P =0.54
0
1000
3000
Time in days
DSS hsa−mir−4326
survival probability
0.0
0.4
0.8
P =0.57
0
1000
3000
Time in days

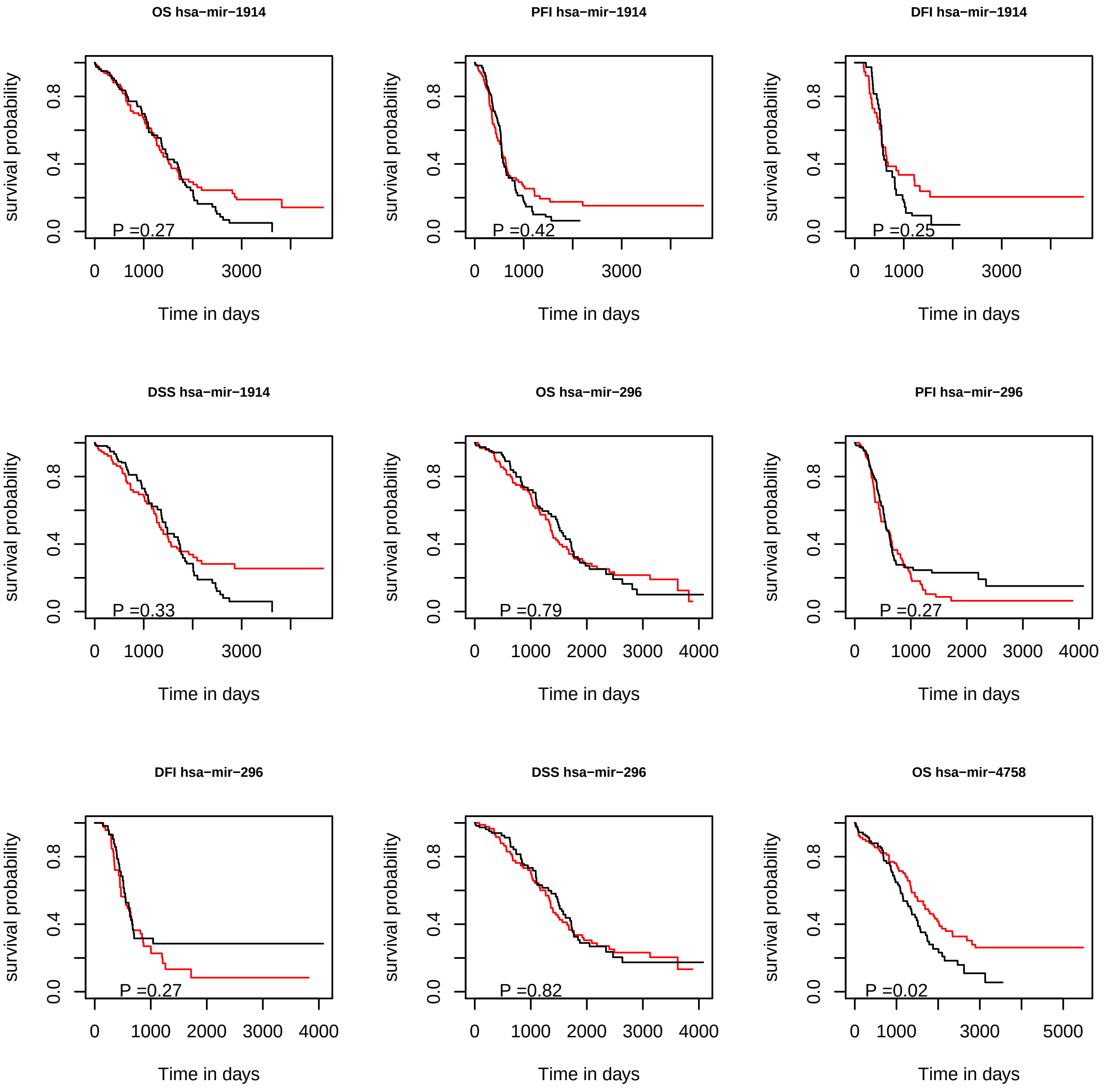
OS hsa−mir−1914
P =0.27
PFI hsa−mir−1914
P =0.42
DFI hsa−mir−1914
P =0.25
DSS hsa−mir−1914
P =0.33
OS hsa−mir−296
P =0.79
PFI hsa−mir−296
P =0.27
DFI hsa−mir−296
P =0.27
DSS hsa−mir−296
P =0.82
OS hsa−mir−4758
P =0.02
survival probability
Time in days

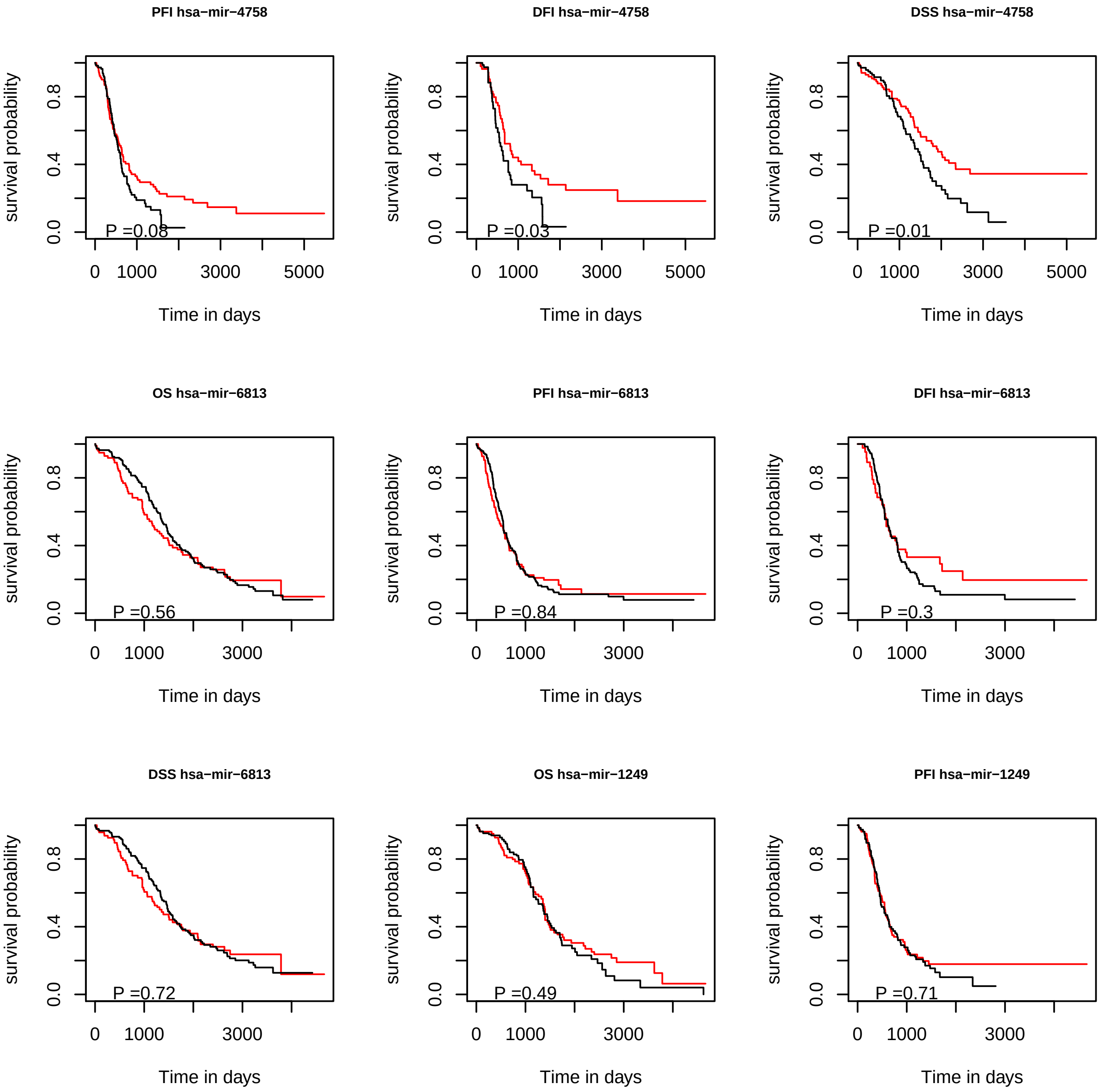

PFI hsa−mir−4758
P =0.08
DFI hsa−mir−4758
P =0.03
DSS hsa−mir−4758
P =0.01
OS hsa−mir−6813
P =0.56
PFI hsa−mir−6813
P =0.84
DFI hsa−mir−6813
P =0.3
DSS hsa−mir−6813
P =0.72
OS hsa−mir−1249
P =0.49
PFI hsa−mir−1249
P =0.71
survival probability
Time in days

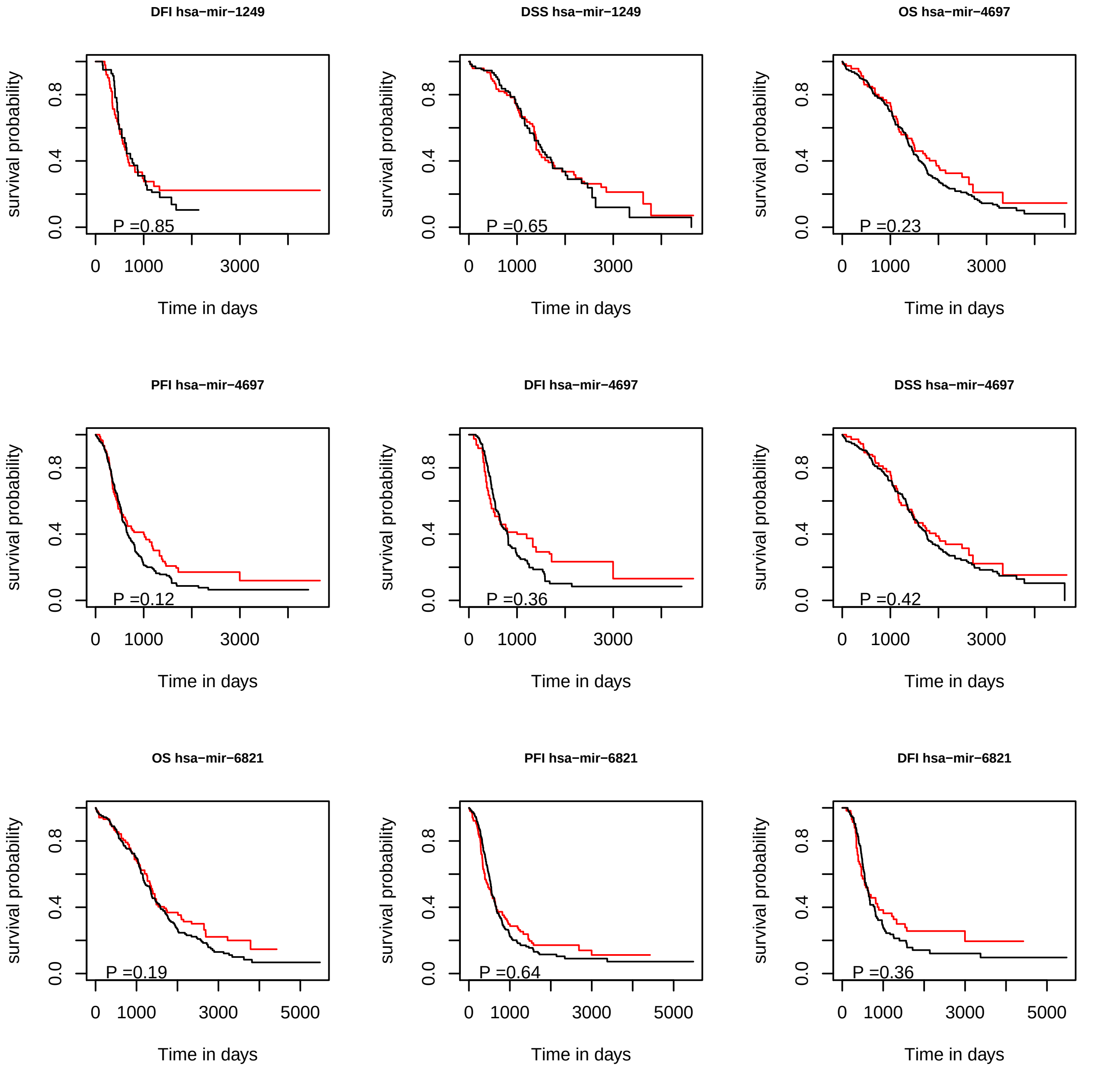
DFI hsa−mir−1249
survival probability
0.0
0.4
0.8
P =0.85
0
1000
3000
Time in days
DSS hsa−mir−1249
survival probability
0.0
0.4
0.8
P =0.65
0
1000
3000
Time in days
OS hsa−mir−4697
survival probability
0.0
0.4
0.8
P =0.23
0
1000
3000
Time in days
PFI hsa−mir−4697
survival probability
0.0
0.4
0.8
P =0.12
0
1000
3000
Time in days
DFI hsa−mir−4697
survival probability
0.0
0.4
0.8
P =0.36
0
1000
3000
Time in days
DSS hsa−mir−4697
survival probability
0.0
0.4
0.8
P =0.42
0
1000
3000
Time in days
OS hsa−mir−6821
survival probability
0.0
0.4
0.8
P =0.19
0
1000
3000
5000
Time in days
PFI hsa−mir−6821
survival probability
0.0
0.4
0.8
P =0.64
0
1000
3000
5000
Time in days
DFI hsa−mir−6821
survival probability
0.0
0.4
0.8
P =0.36
0
1000
3000
5000
Time in days

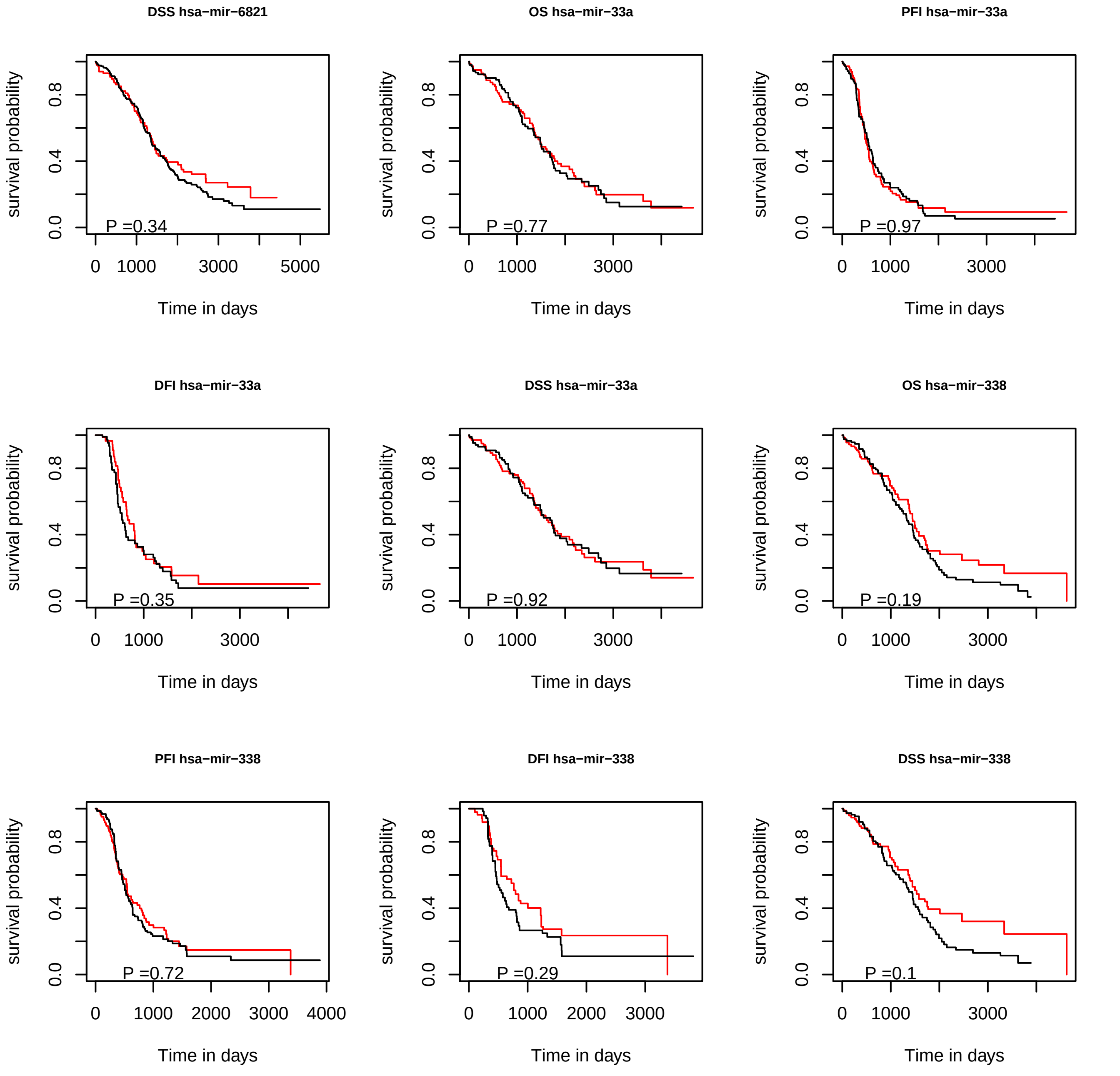
DSS hsa−mir−6821
P =0.34
OS hsa−mir−33a
P =0.77
PFI hsa−mir−33a
P =0.97
DFI hsa−mir−33a
P =0.35
DSS hsa−mir−33a
P =0.92
OS hsa−mir−338
P =0.19
PFI hsa−mir−338
P =0.72
DFI hsa−mir−338
P =0.29
DSS hsa−mir−338
P =0.1
survival probability
Time in days

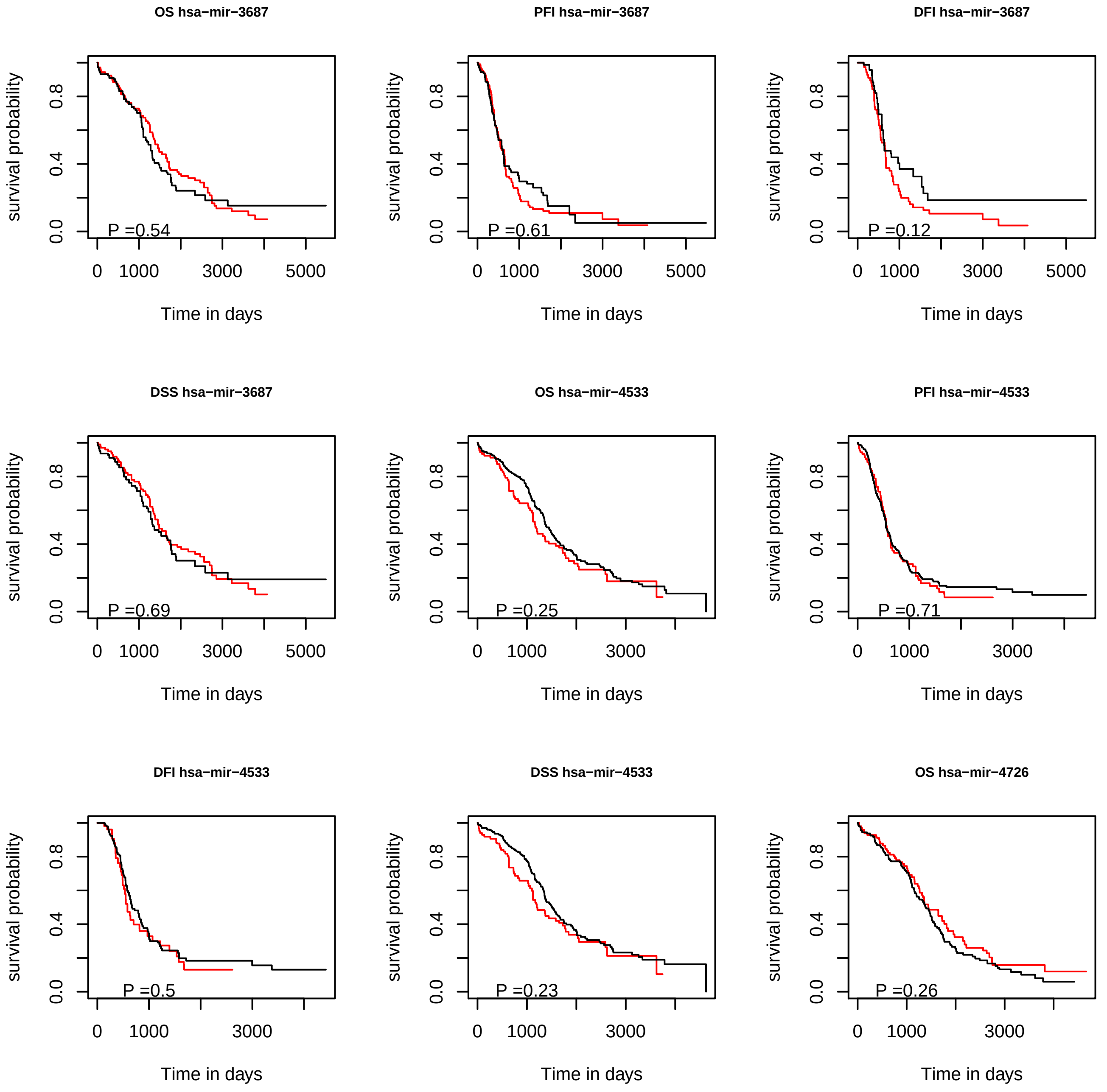
OS hsa−mir−3687
P =0.54
PFI hsa−mir−3687
P =0.61
DFI hsa−mir−3687
P =0.12
DSS hsa−mir−3687
P =0.69
OS hsa−mir−4533
P =0.25
PFI hsa−mir−4533
P =0.71
DFI hsa−mir−4533
P =0.5
DSS hsa−mir−4533
P =0.23
OS hsa−mir−4726
P =0.26
survival probability
0.0
0.4
0.8
Time in days
0
1000
3000
5000

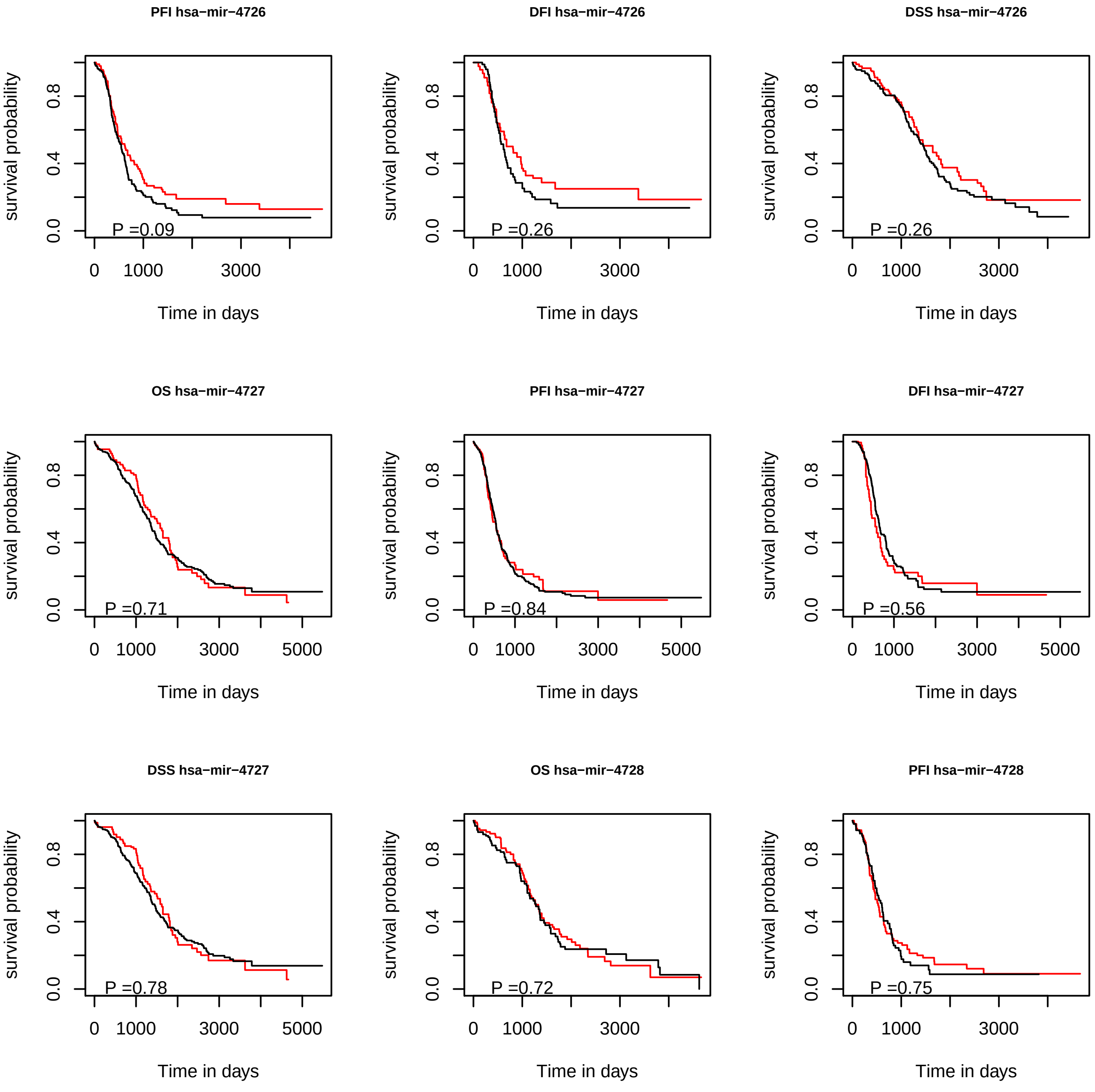

PFI hsa−mir−4726
P =0.09
DFI hsa−mir−4726
P =0.26
DSS hsa−mir−4726
P =0.26
OS hsa−mir−4727
P =0.71
PFI hsa−mir−4727
P =0.84
DFI hsa−mir−4727
P =0.56
DSS hsa−mir−4727
P =0.78
OS hsa−mir−4728
P =0.72
PFI hsa−mir−4728
P =0.75
survival probability
Time in days
0.0
0.4
0.8
0
1000
3000
5000

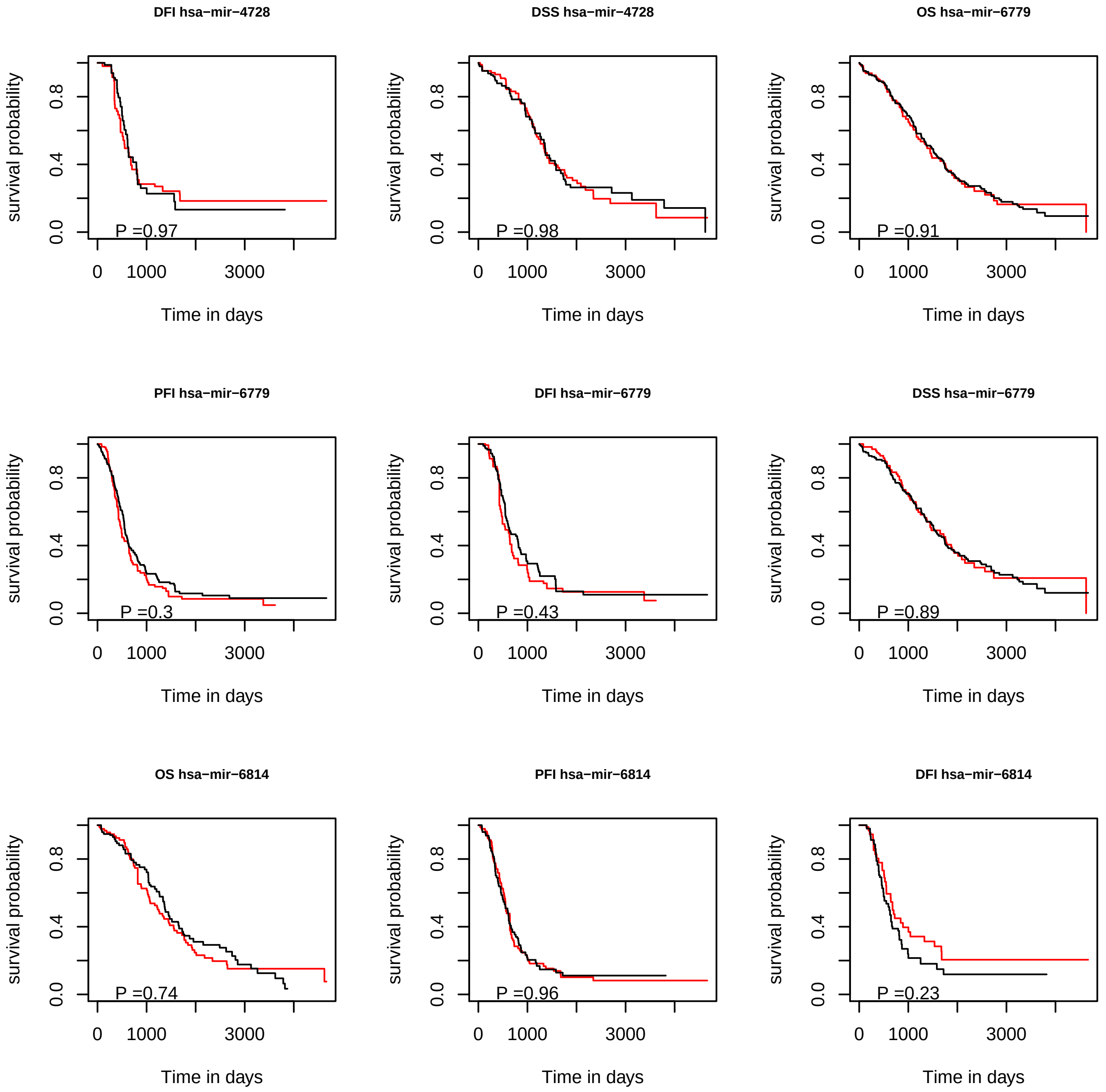
DFI hsa−mir−4728
P =0.97
DSS hsa−mir−4728
P =0.98
OS hsa−mir−6779
P =0.91
PFI hsa−mir−6779
P =0.3
DFI hsa−mir−6779
P =0.43
DSS hsa−mir−6779
P =0.89
OS hsa−mir−6814
P =0.74
PFI hsa−mir−6814
P =0.96
DFI hsa−mir−6814
P =0.23
survival probability
0.0
0.4
0.8
0
1000
3000
Time in days

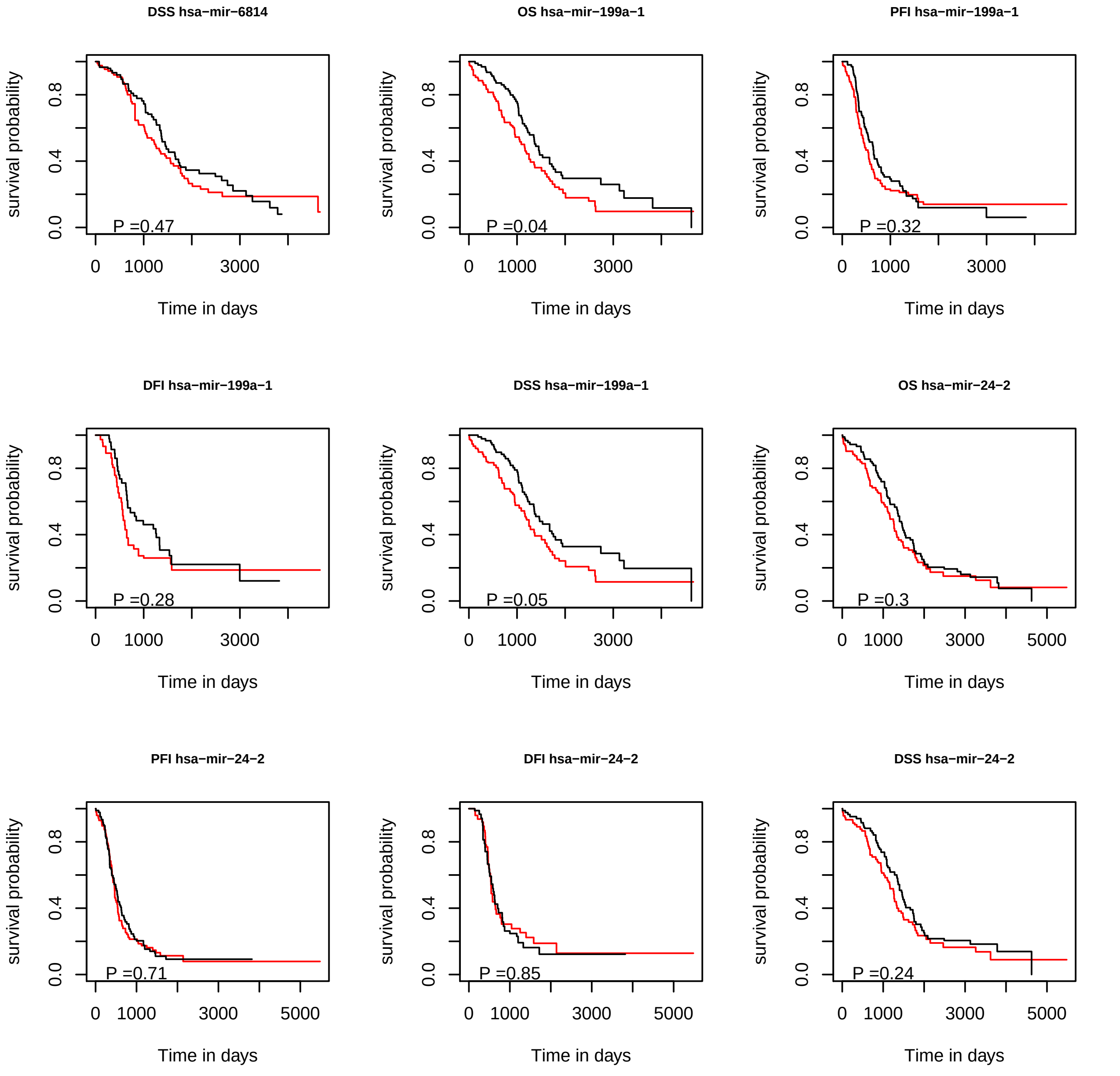

DSS hsa−mir−6814
P =0.47
OS hsa−mir−199a−1
P =0.04
PFI hsa−mir−199a−1
P =0.32
DFI hsa−mir−199a−1
P =0.28
DSS hsa−mir−199a−1
P =0.05
OS hsa−mir−24−2
P =0.3
PFI hsa−mir−24−2
P =0.71
DFI hsa−mir−24−2
P =0.85
DSS hsa−mir−24−2
P =0.24
survival probability
0.0
0.4
0.8
Time in days
0
1000
3000
5000

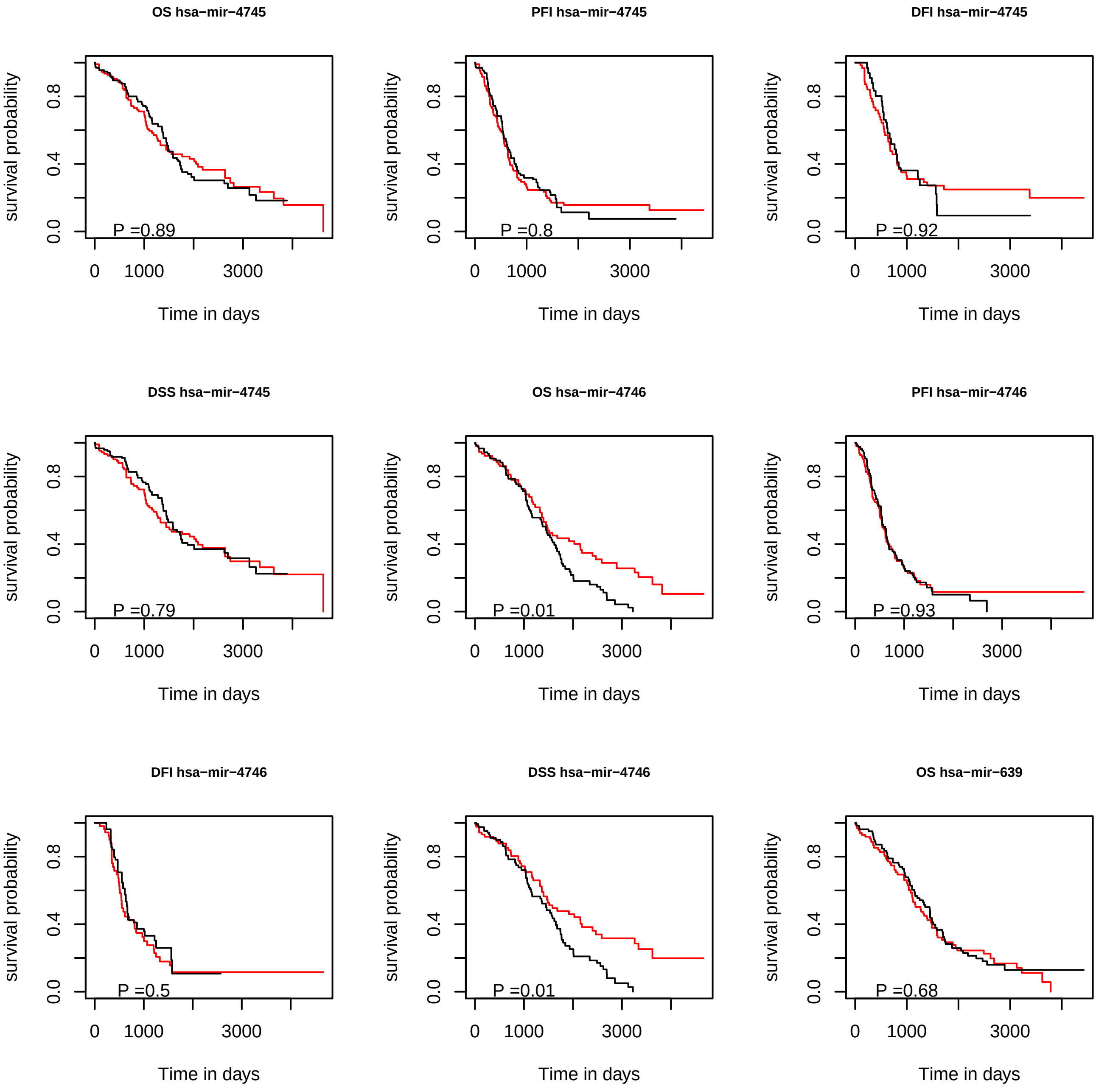
OS hsa−mir−4745
P =0.89
PFI hsa−mir−4745
P =0.8
DFI hsa−mir−4745
P =0.92
DSS hsa−mir−4745
P =0.79
OS hsa−mir−4746
P =0.01
PFI hsa−mir−4746
P =0.93
DFI hsa−mir−4746
P =0.5
DSS hsa−mir−4746
P =0.01
OS hsa−mir−639
P =0.68
survival probability
Time in days
0.0
0.4
0.8
0
1000
3000

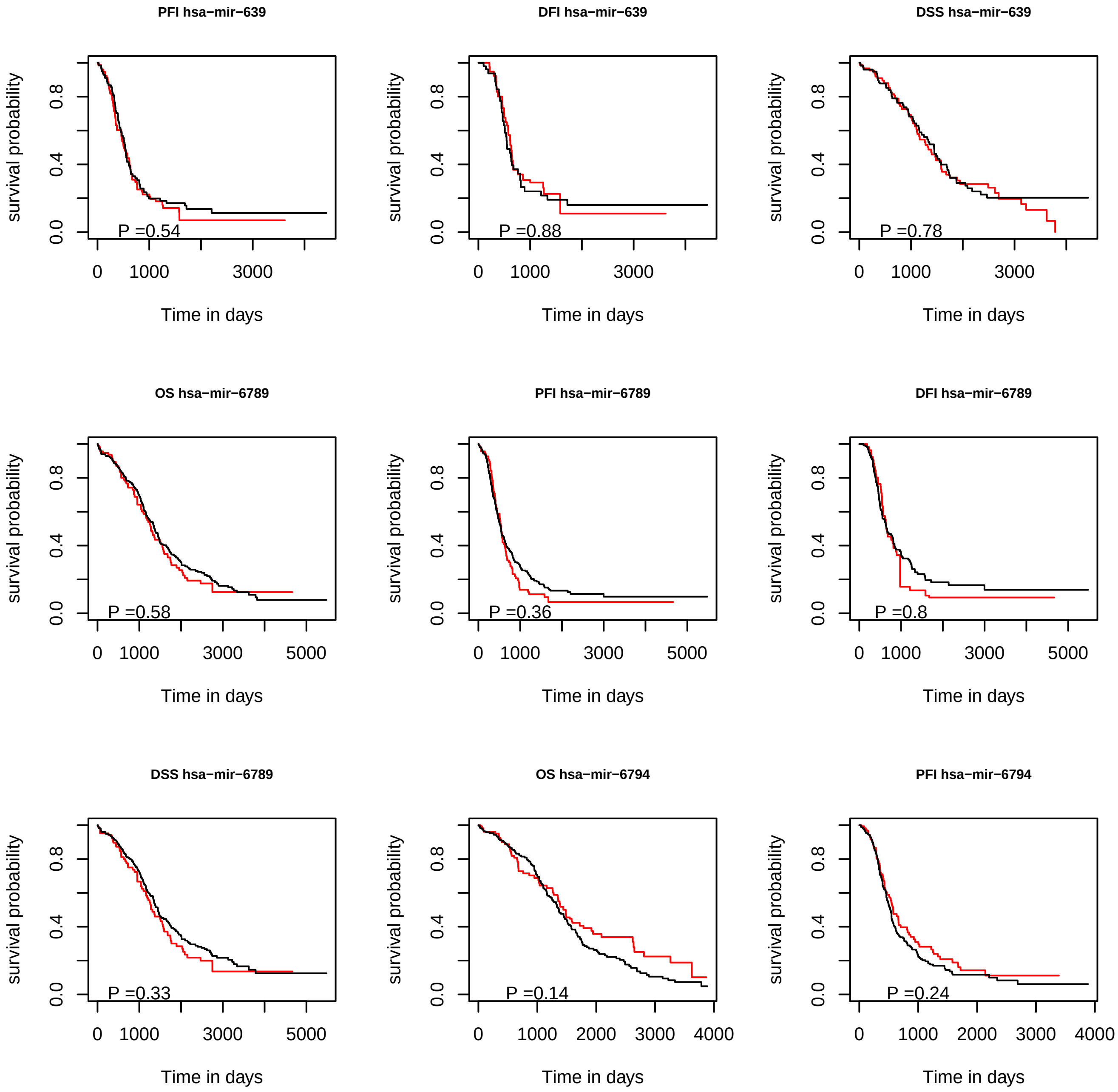

PFI hsa−mir−639
P =0.54
DFI hsa−mir−639
P =0.88
DSS hsa−mir−639
P =0.78
OS hsa−mir−6789
P =0.58
PFI hsa−mir−6789
P =0.36
DFI hsa−mir−6789
P =0.8
DSS hsa−mir−6789
P =0.33
OS hsa−mir−6794
P =0.14
PFI hsa−mir−6794
P =0.24
survival probability
Time in days
0.0
0.4
0.8
0
1000
2000
3000
4000
5000

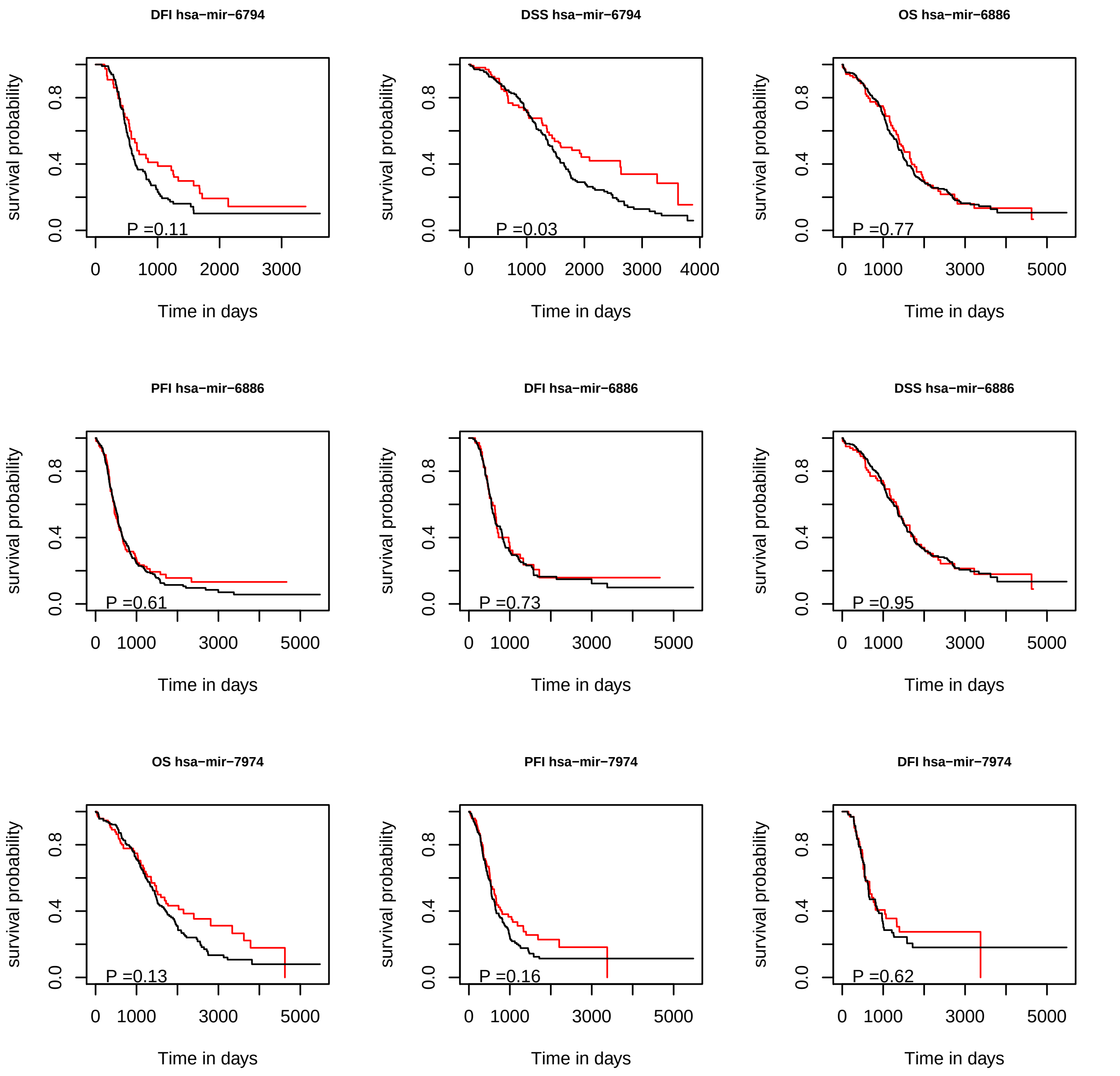
DFI hsa−mir−6794
P =0.11
DSS hsa−mir−6794
P =0.03
OS hsa−mir−6886
P =0.77
PFI hsa−mir−6886
P =0.61
DFI hsa−mir−6886
P =0.73
DSS hsa−mir−6886
P =0.95
OS hsa−mir−7974
P =0.13
PFI hsa−mir−7974
P =0.16
DFI hsa−mir−7974
P =0.62
survival probability
Time in days

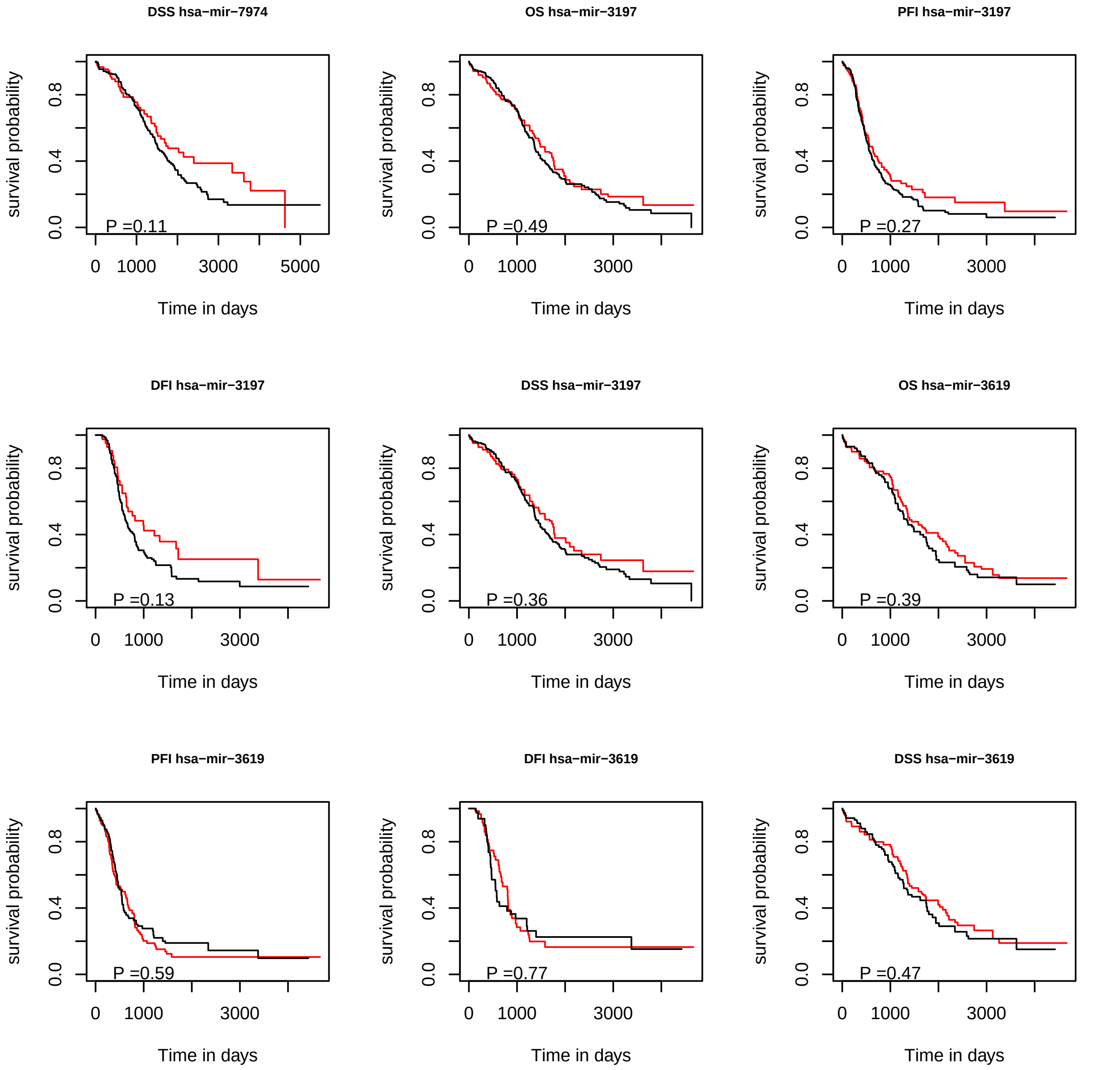
DSS hsa−mir−7974
survival probability
0.0
0.4
0.8
P =0.11
0
1000
3000
5000
Time in days
OS hsa−mir−3197
survival probability
0.0
0.4
0.8
P =0.49
0
1000
3000
Time in days
PFI hsa−mir−3197
survival probability
0.0
0.4
0.8
P =0.27
0
1000
3000
Time in days
DFI hsa−mir−3197
survival probability
0.0
0.4
0.8
P =0.13
0
1000
3000
Time in days
DSS hsa−mir−3197
survival probability
0.0
0.4
0.8
P =0.36
0
1000
3000
Time in days
OS hsa−mir−3619
survival probability
0.0
0.4
0.8
P =0.39
0
1000
3000
Time in days
PFI hsa−mir−3619
survival probability
0.0
0.4
0.8
P =0.59
0
1000
3000
Time in days
DFI hsa−mir−3619
survival probability
0.0
0.4
0.8
P =0.77
0
1000
3000
Time in days
DSS hsa−mir−3619
survival probability
0.0
0.4
0.8
P =0.47
0
1000
3000
Time in days

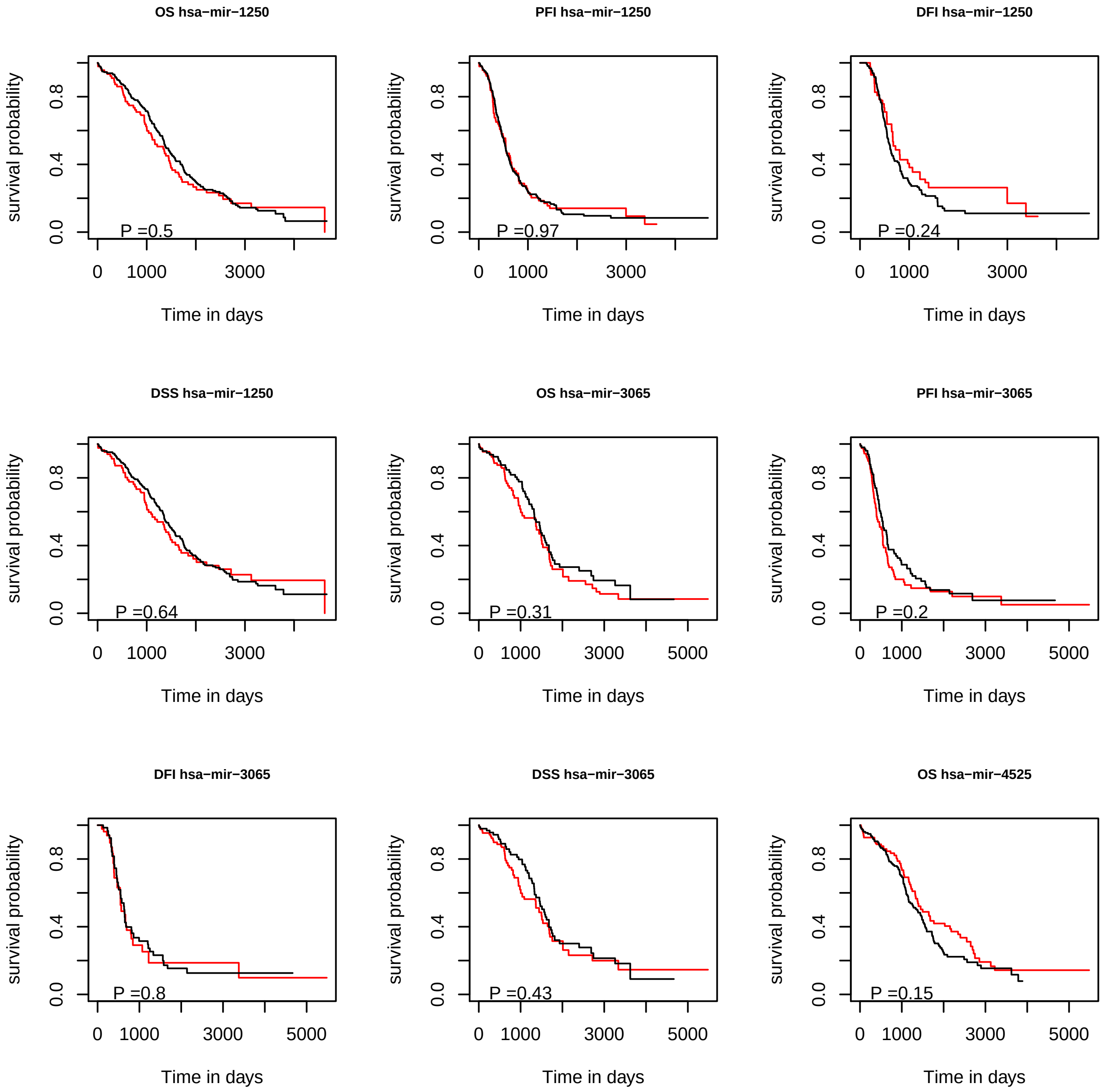
OS hsa−mir−1250
P =0.5
PFI hsa−mir−1250
P =0.97
DFI hsa−mir−1250
P =0.24
DSS hsa−mir−1250
P =0.64
OS hsa−mir−3065
P =0.31
PFI hsa−mir−3065
P =0.2
DFI hsa−mir−3065
P =0.8
DSS hsa−mir−3065
P =0.43
OS hsa−mir−4525
P =0.15
survival probability
Time in days
0.0
0.4
0.8
0
1000
3000
5000

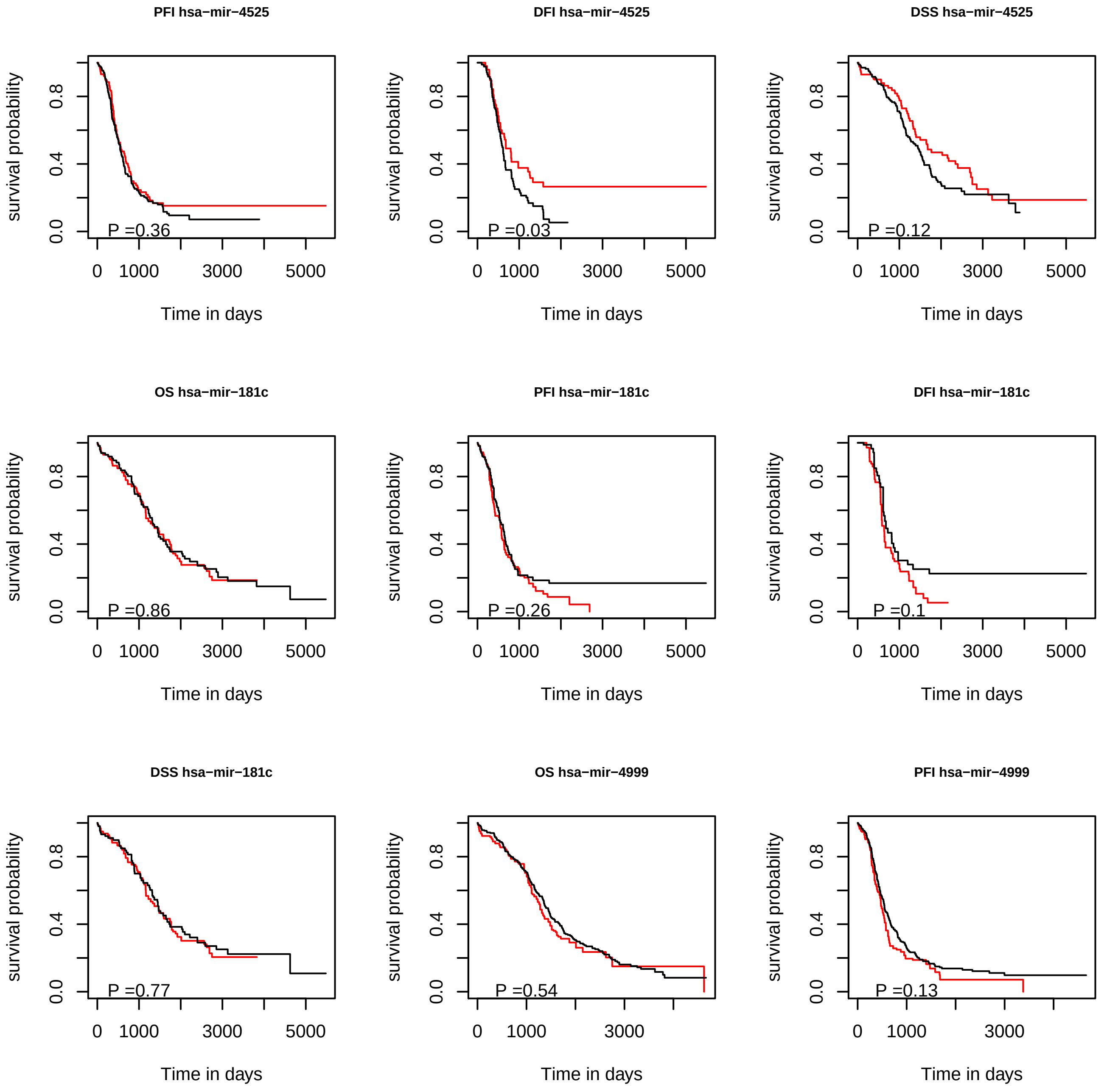
PFI hsa−mir−4525
P =0.36
DFI hsa−mir−4525
P =0.03
DSS hsa−mir−4525
P =0.12
OS hsa−mir−181c
P =0.86
PFI hsa−mir−181c
P =0.26
DFI hsa−mir−181c
P =0.1
DSS hsa−mir−181c
P =0.77
OS hsa−mir−4999
P =0.54
PFI hsa−mir−4999
P =0.13
survival probability
Time in days
0.0
0.4
0.8
0
1000
3000
5000

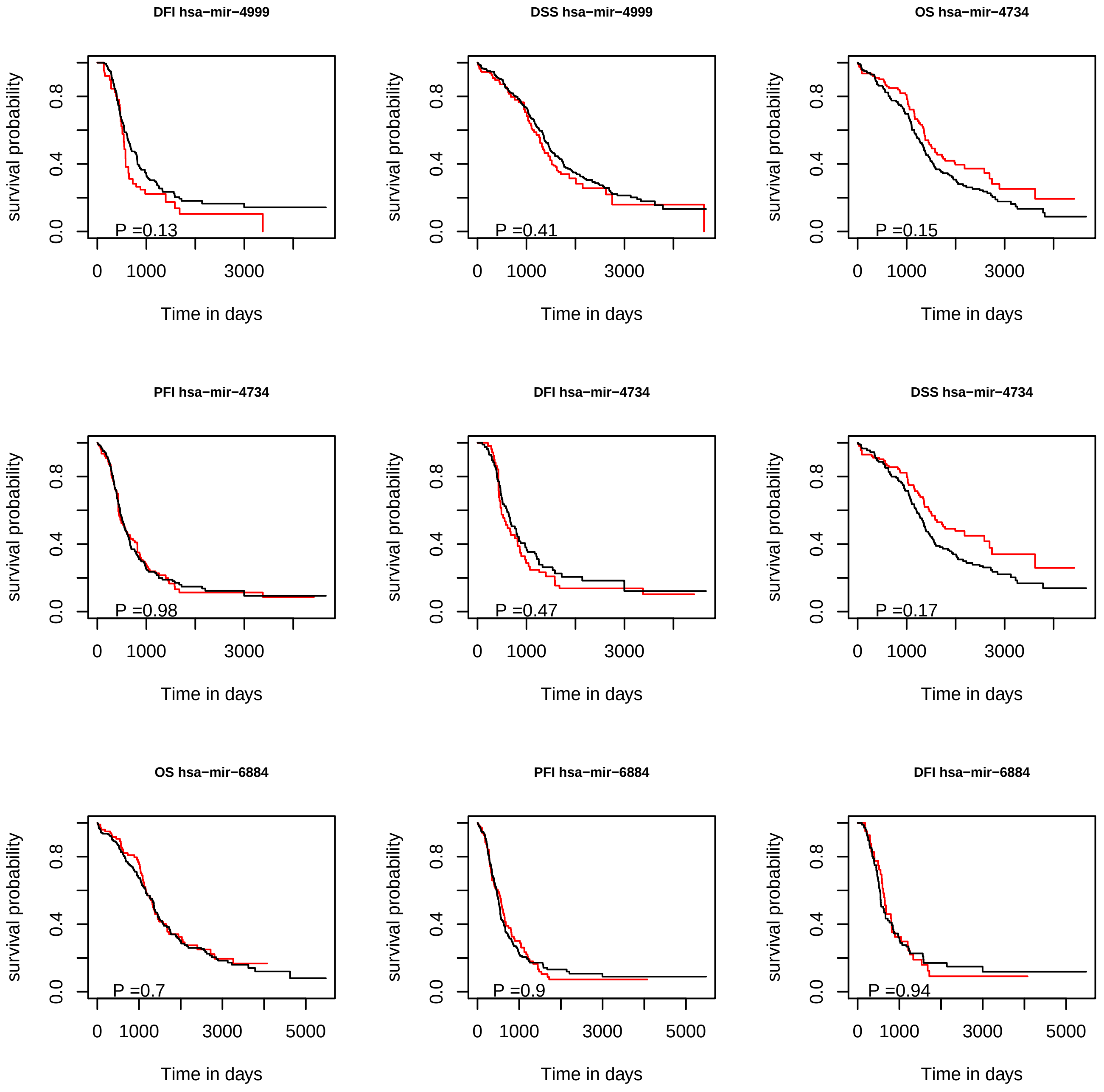
DFI hsa−mir−4999
P =0.13
DSS hsa−mir−4999
P =0.41
OS hsa−mir−4734
P =0.15
PFI hsa−mir−4734
P =0.98
DFI hsa−mir−4734
P =0.47
DSS hsa−mir−4734
P =0.17
OS hsa−mir−6884
P =0.7
PFI hsa−mir−6884
P =0.9
DFI hsa−mir−6884
P =0.94
survival probability
0.0
0.4
0.8
Time in days
0
1000
3000
5000

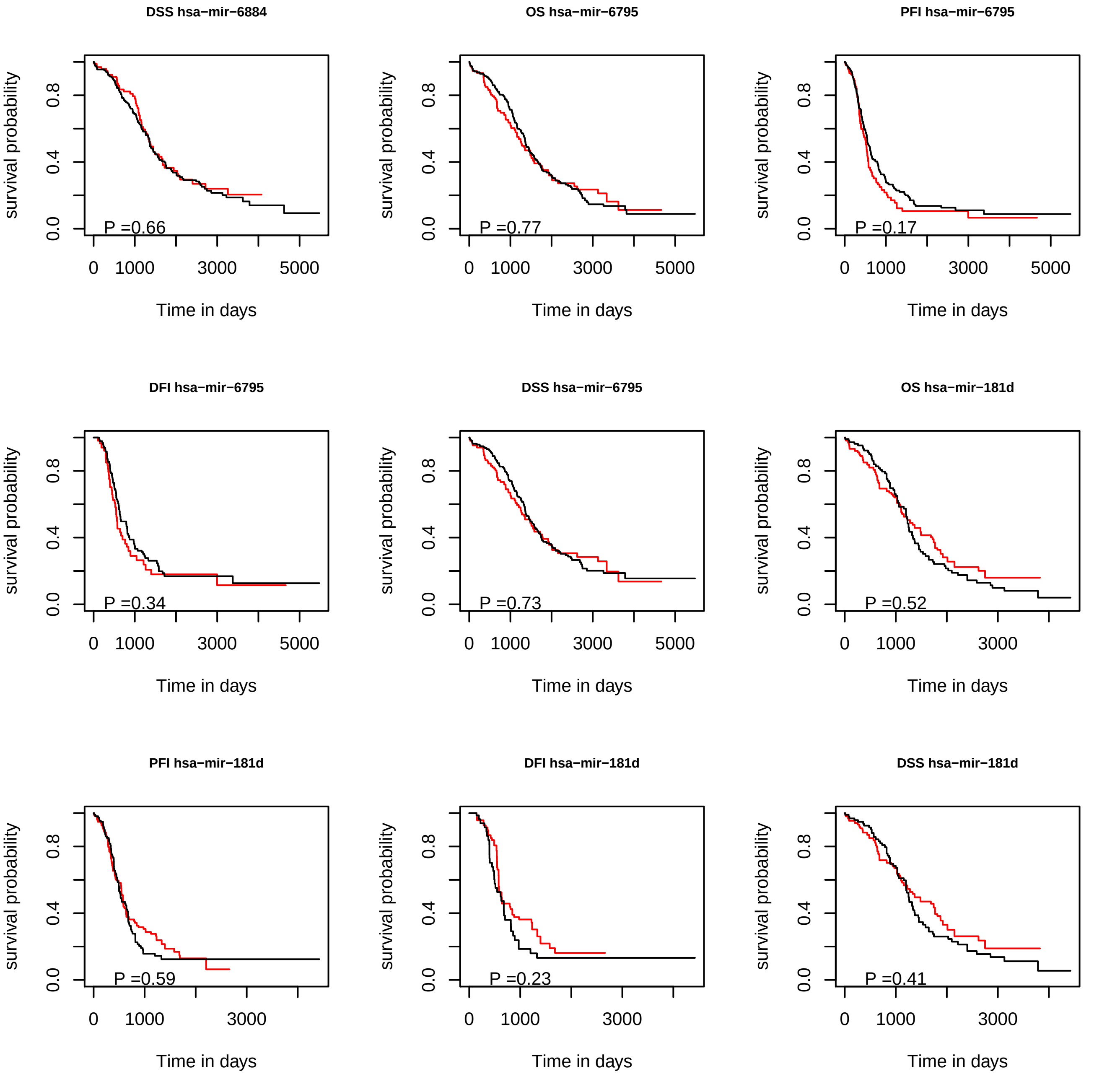

DSS hsa−mir−6884
P =0.66
OS hsa−mir−6795
P =0.77
PFI hsa−mir−6795
P =0.17
DFI hsa−mir−6795
P =0.34
DSS hsa−mir−6795
P =0.73
OS hsa−mir−181d
P =0.52
PFI hsa−mir−181d
P =0.59
DFI hsa−mir−181d
P =0.23
DSS hsa−mir−181d
P =0.41
survival probability
Time in days

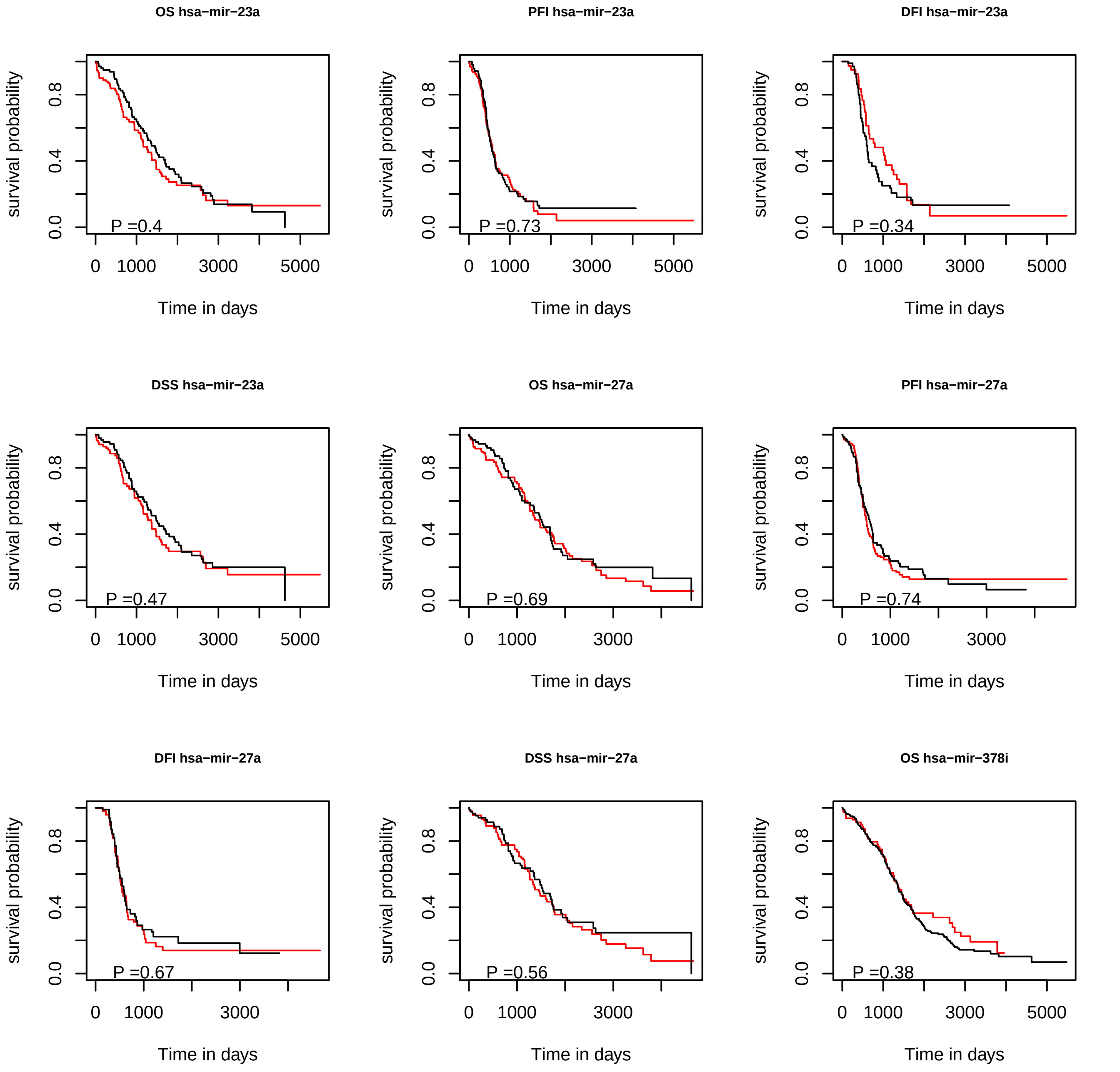
OS hsa−mir−23a
P =0.4
PFI hsa−mir−23a
P =0.73
DFI hsa−mir−23a
P =0.34
DSS hsa−mir−23a
P =0.47
OS hsa−mir−27a
P =0.69
PFI hsa−mir−27a
P =0.74
DFI hsa−mir−27a
P =0.67
DSS hsa−mir−27a
P =0.56
OS hsa−mir−378i
P =0.38
survival probability
Time in days
0.0
0.4
0.8
0
1000
3000
5000

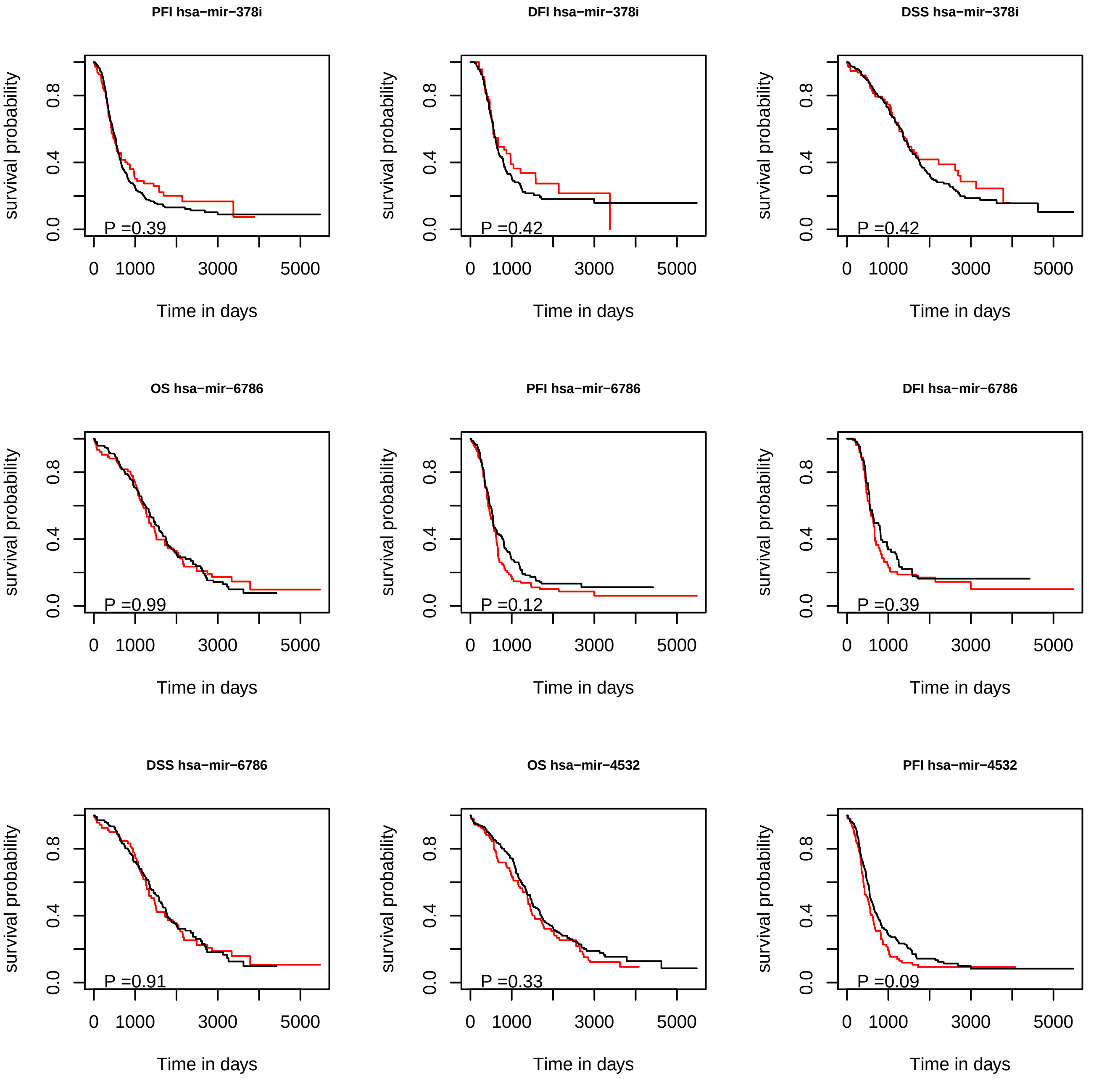
PFI hsa−mir−378i
P =0.39
DFI hsa−mir−378i
P =0.42
DSS hsa−mir−378i
P =0.42
OS hsa−mir−6786
P =0.99
PFI hsa−mir−6786
P =0.12
DFI hsa−mir−6786
P =0.39
DSS hsa−mir−6786
P =0.91
OS hsa−mir−4532
P =0.33
PFI hsa−mir−4532
P =0.09
survival probability
0.0
0.4
0.8
Time in days
0
1000
3000
5000

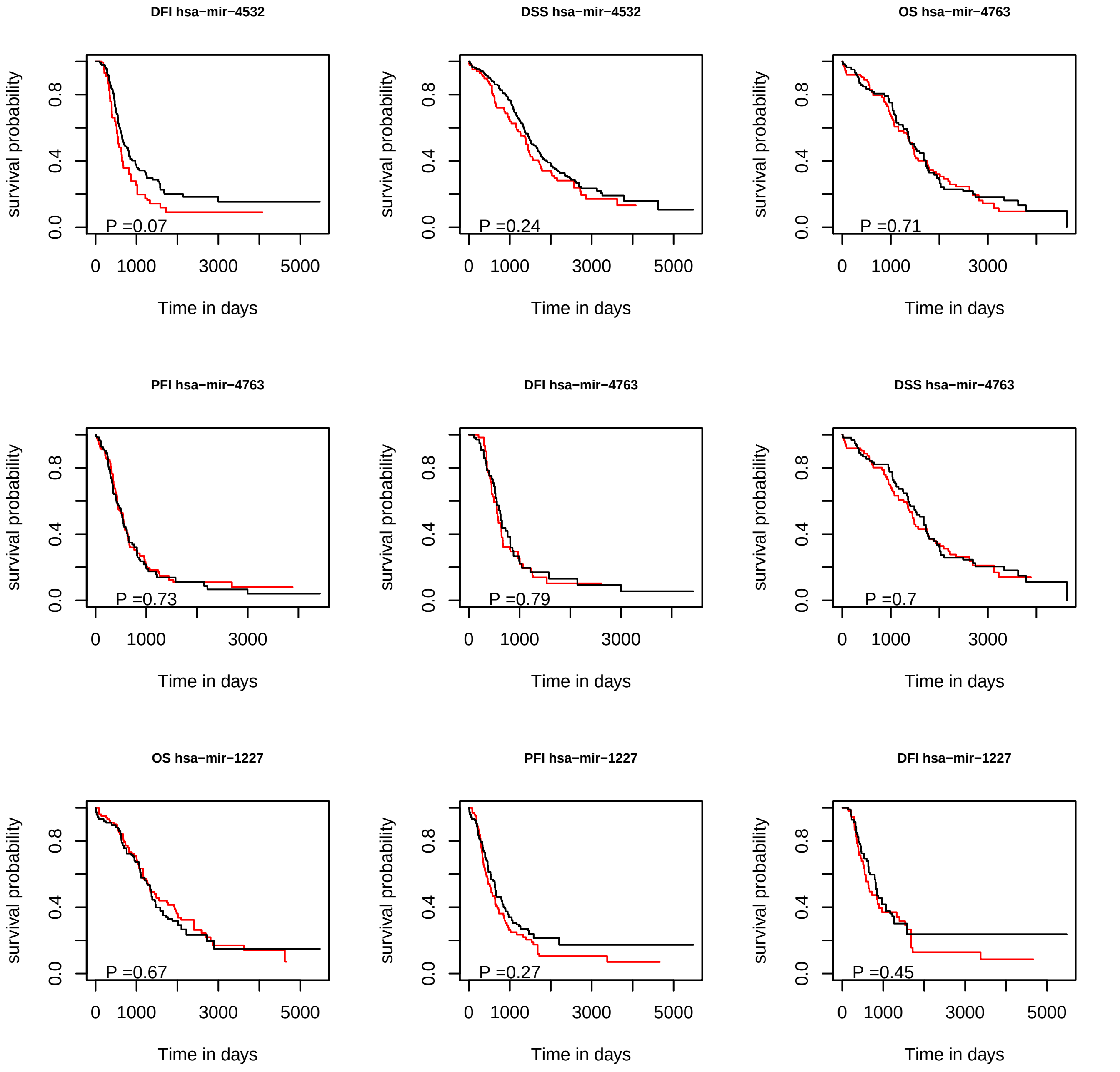
DFI hsa−mir−4532
P =0.07
DSS hsa−mir−4532
P =0.24
OS hsa−mir−4763
P =0.71
PFI hsa−mir−4763
P =0.73
DFI hsa−mir−4763
P =0.79
DSS hsa−mir−4763
P =0.7
OS hsa−mir−1227
P =0.67
PFI hsa−mir−1227
P =0.27
DFI hsa−mir−1227
P =0.45
survival probability
Time in days
0.0
0.4
0.8
0
1000
3000
5000

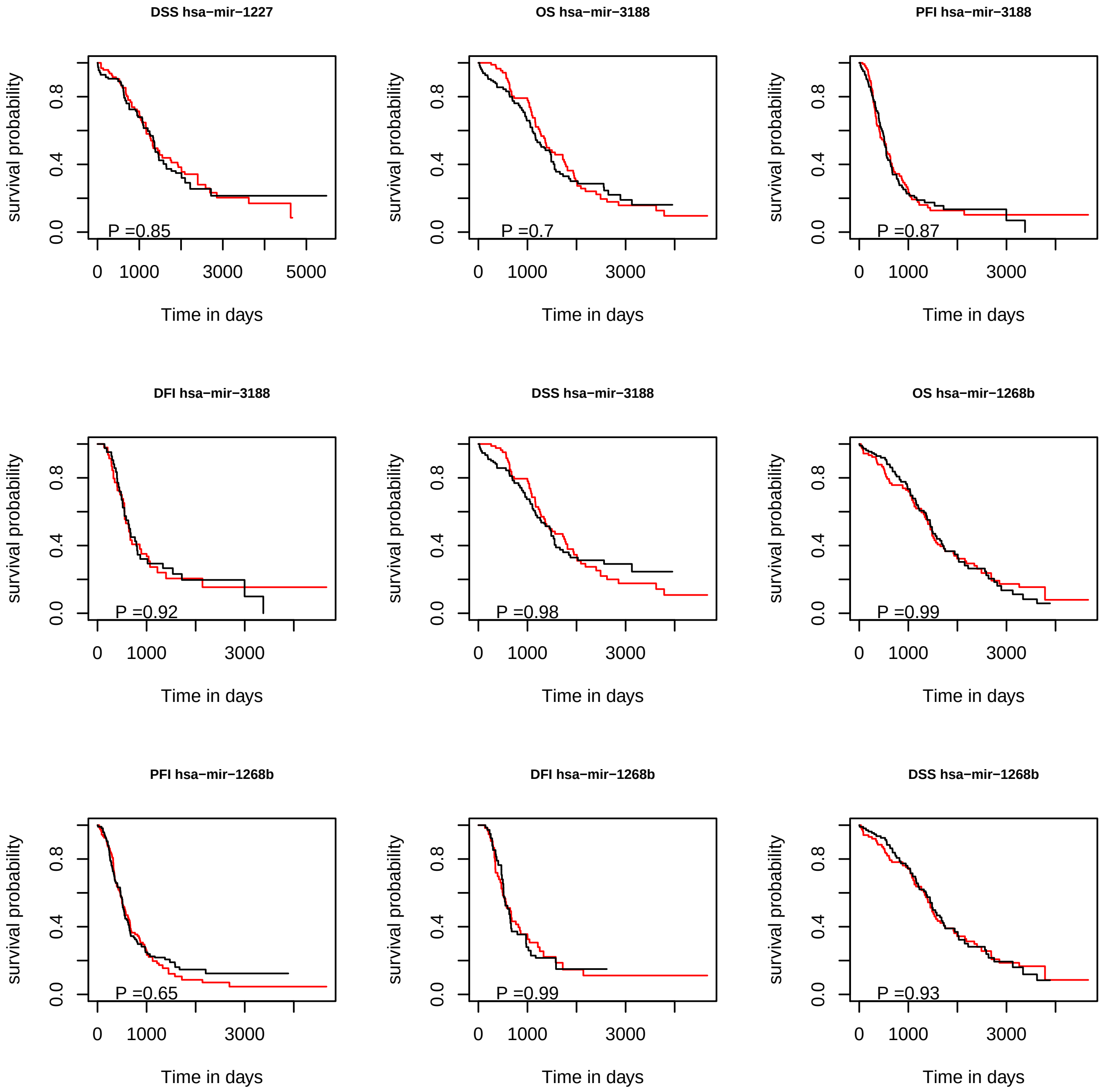
DSS hsa−mir−1227
P =0.85
OS hsa−mir−3188
P =0.7
PFI hsa−mir−3188
P =0.87
DFI hsa−mir−3188
P =0.92
DSS hsa−mir−3188
P =0.98
OS hsa−mir−1268b
P =0.99
PFI hsa−mir−1268b
P =0.65
DFI hsa−mir−1268b
P =0.99
DSS hsa−mir−1268b
P =0.93
survival probability
Time in days
0.0
0.4
0.8
0
1000
3000
5000

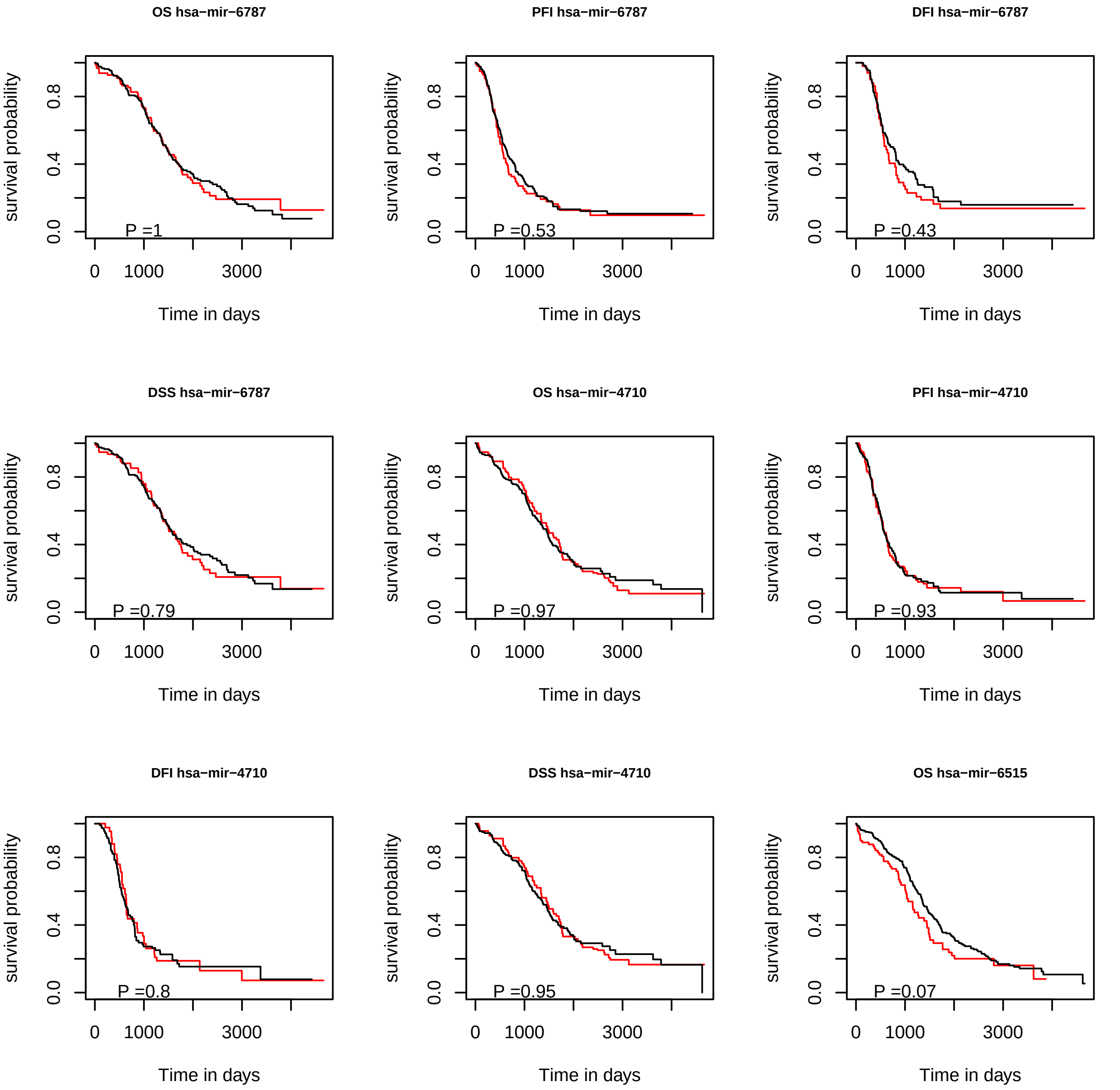
OS hsa−mir−6787
P =1
PFI hsa−mir−6787
P =0.53
DFI hsa−mir−6787
P =0.43
DSS hsa−mir−6787
P =0.79
OS hsa−mir−4710
P =0.97
PFI hsa−mir−4710
P =0.93
DFI hsa−mir−4710
P =0.8
DSS hsa−mir−4710
P =0.95
OS hsa−mir−6515
P =0.07
survival probability
Time in days
0.0
0.4
0.8
0
1000
3000

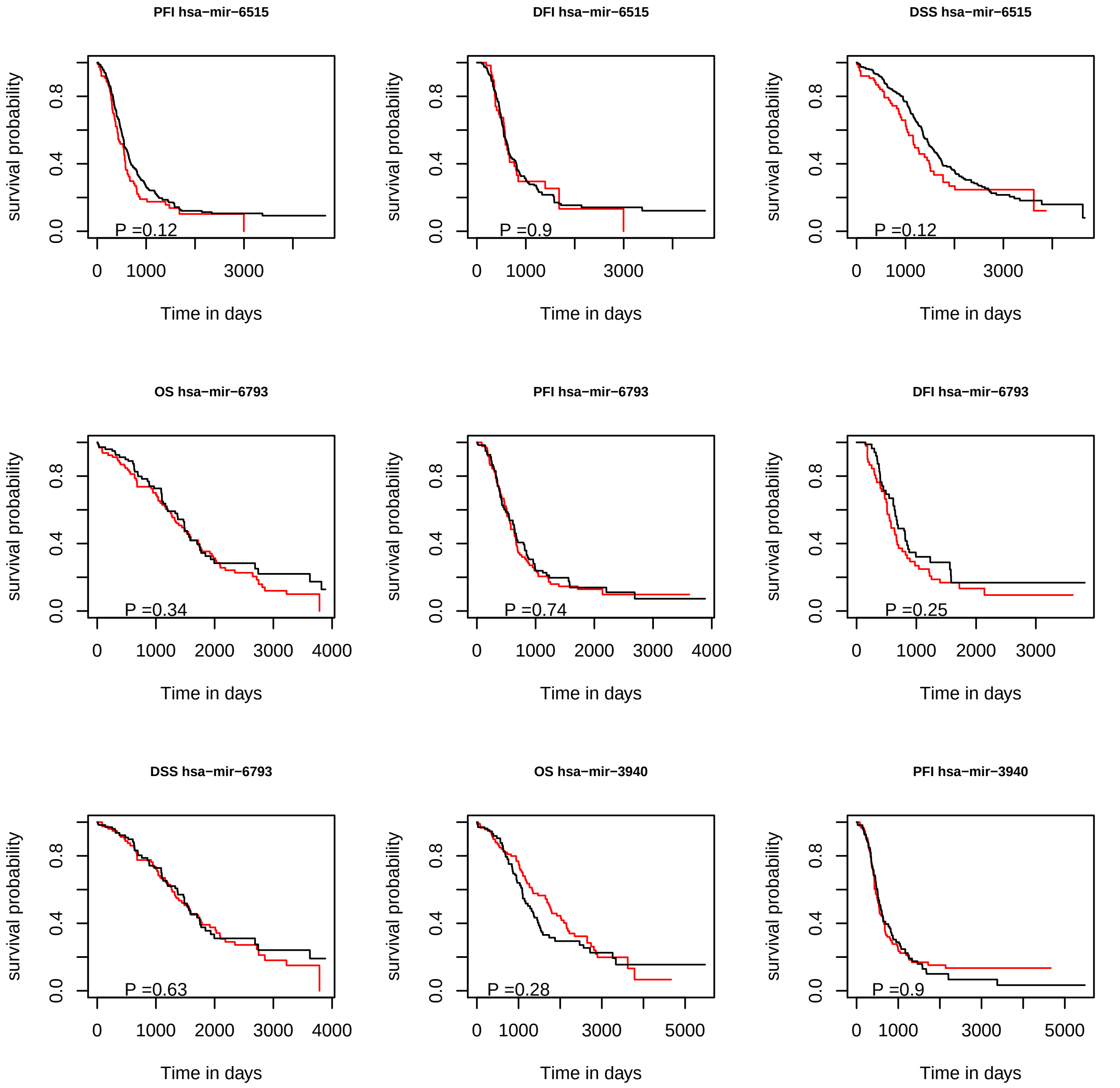
PFI hsa−mir−6515
P =0.12
DFI hsa−mir−6515
P =0.9
DSS hsa−mir−6515
P =0.12
OS hsa−mir−6793
P =0.34
PFI hsa−mir−6793
P =0.74
DFI hsa−mir−6793
P =0.25
DSS hsa−mir−6793
P =0.63
OS hsa−mir−3940
P =0.28
PFI hsa−mir−3940
P =0.9
survival probability
Time in days
0.0
0.4
0.8
0
1000
2000
3000
4000
5000

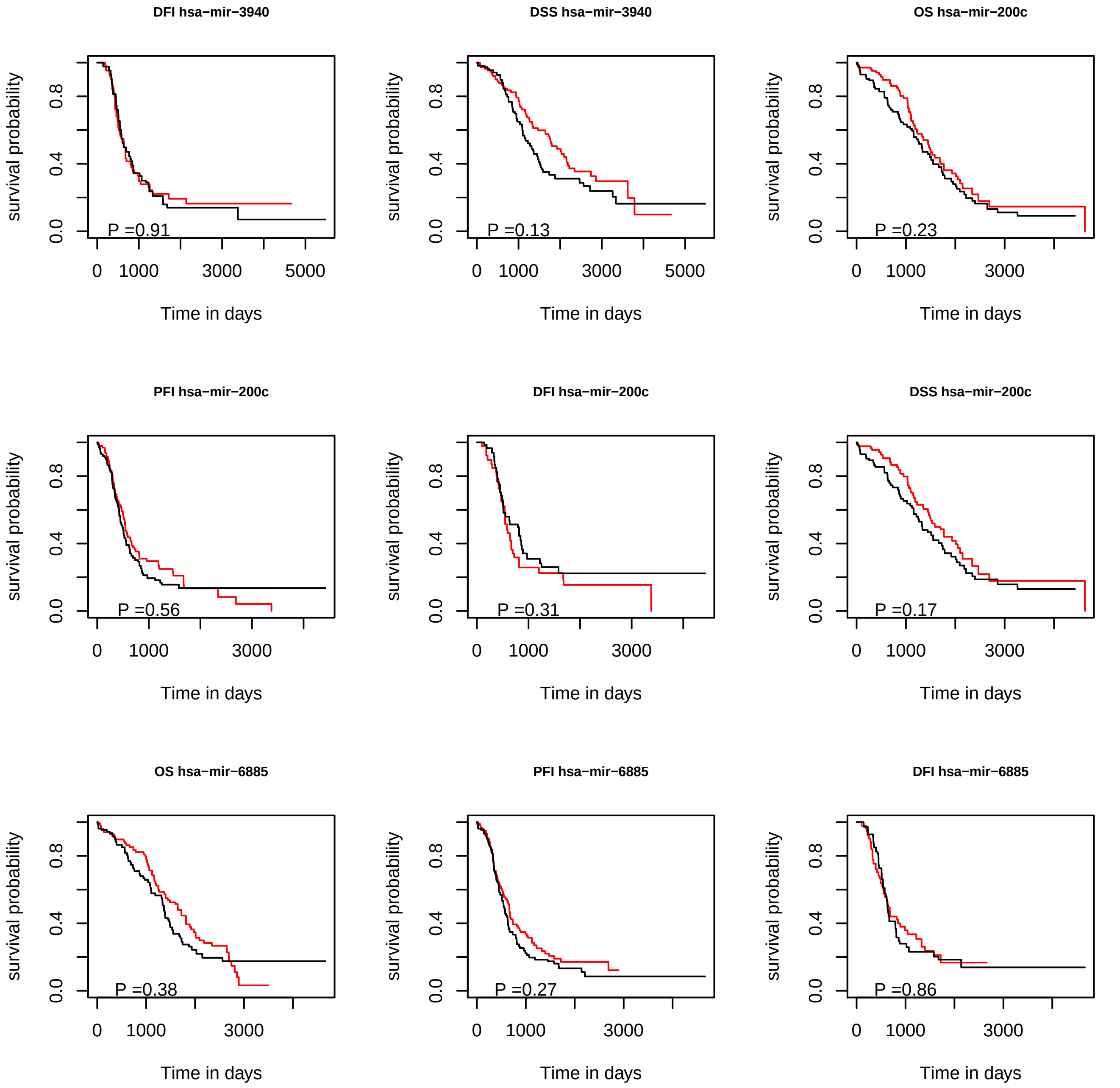
DFI hsa−mir−3940
P =0.91
DSS hsa−mir−3940
P =0.13
OS hsa−mir−200c
P =0.23
PFI hsa−mir−200c
P =0.56
DFI hsa−mir−200c
P =0.31
DSS hsa−mir−200c
P =0.17
OS hsa−mir−6885
P =0.38
PFI hsa−mir−6885
P =0.27
DFI hsa−mir−6885
P =0.86
survival probability
Time in days
0.0
0.4
0.8
0
1000
3000
5000

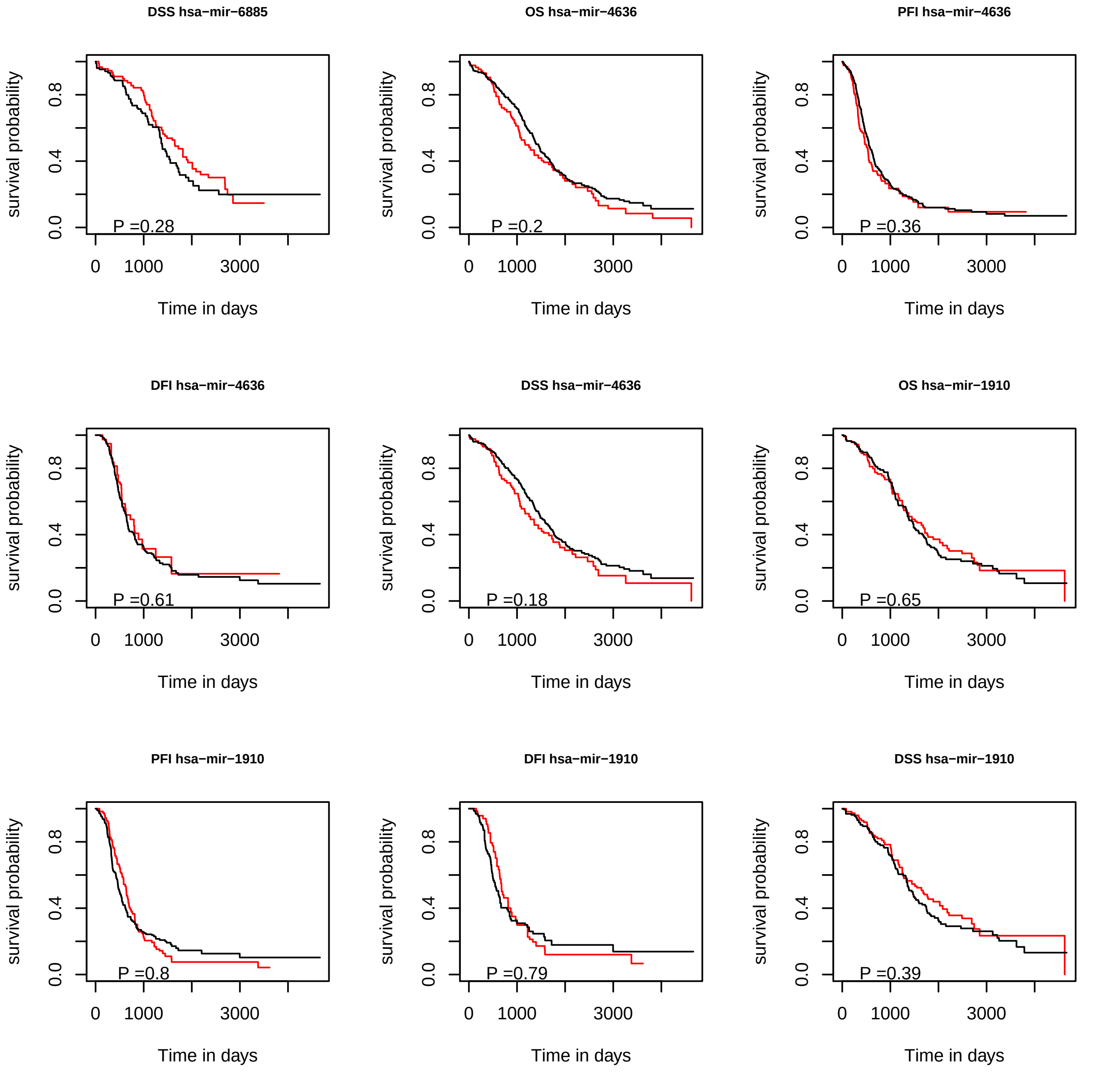

DSS hsa−mir−6885
P =0.28
OS hsa−mir−4636
P =0.2
PFI hsa−mir−4636
P =0.36
DFI hsa−mir−4636
P =0.61
DSS hsa−mir−4636
P =0.18
OS hsa−mir−1910
P =0.65
PFI hsa−mir−1910
P =0.8
DFI hsa−mir−1910
P =0.79
DSS hsa−mir−1910
P =0.39
survival probability
0.0
0.4
0.8
Time in days
0
1000
3000

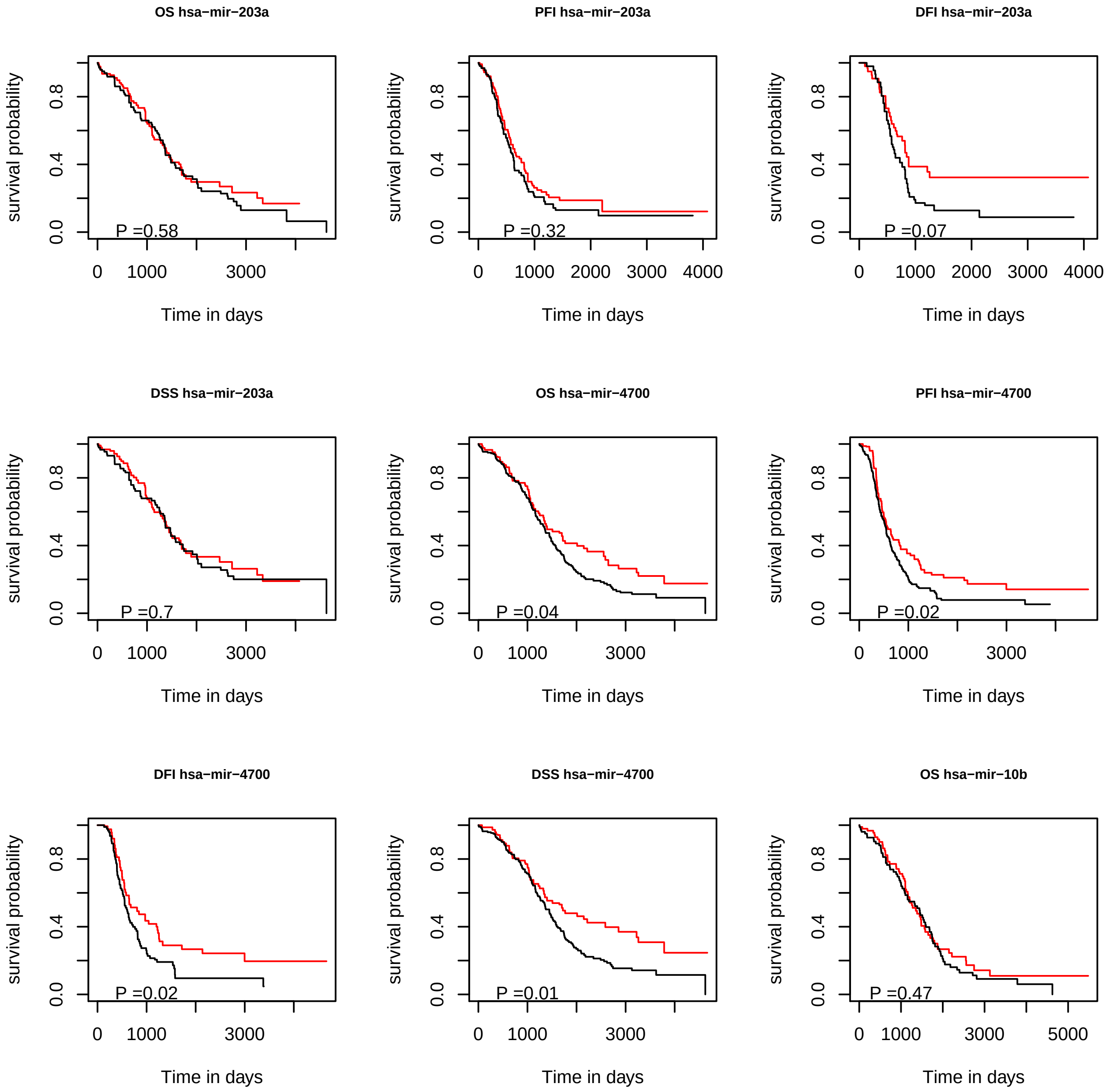
OS hsa−mir−203a
P =0.58
PFI hsa−mir−203a
P =0.32
DFI hsa−mir−203a
P =0.07
DSS hsa−mir−203a
P =0.7
OS hsa−mir−4700
P =0.04
PFI hsa−mir−4700
P =0.02
DFI hsa−mir−4700
P =0.02
DSS hsa−mir−4700
P =0.01
OS hsa−mir−10b
P =0.47
survival probability
Time in days
0.0
0.4
0.8
0
1000
2000
3000
4000
5000

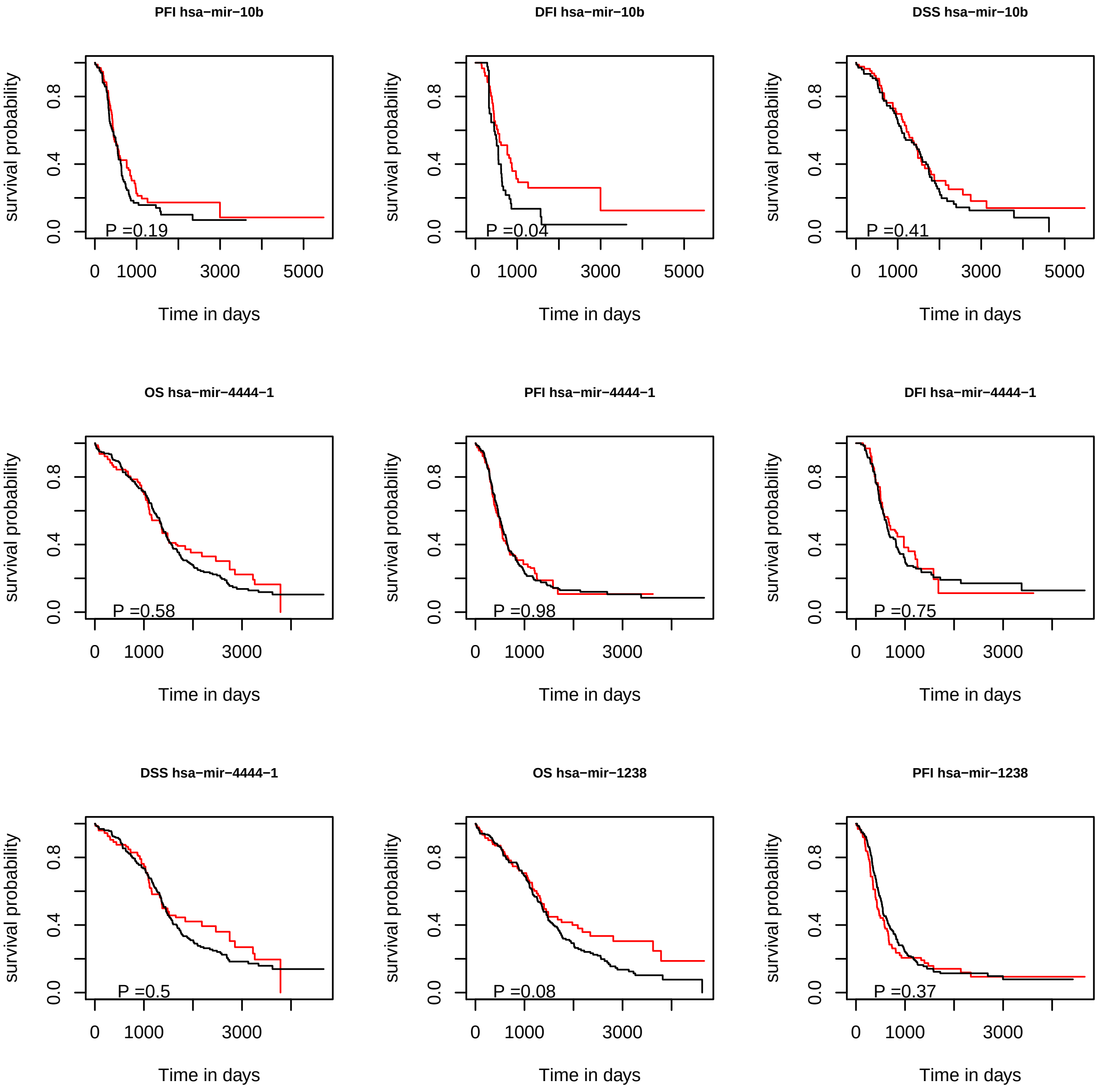
PFI hsa−mir−10b
P =0.19
DFI hsa−mir−10b
P =0.04
DSS hsa−mir−10b
P =0.41
OS hsa−mir−4444−1
P =0.58
PFI hsa−mir−4444−1
P =0.98
DFI hsa−mir−4444−1
P =0.75
DSS hsa−mir−4444−1
P =0.5
OS hsa−mir−1238
P =0.08
PFI hsa−mir−1238
P =0.37
survival probability
Time in days

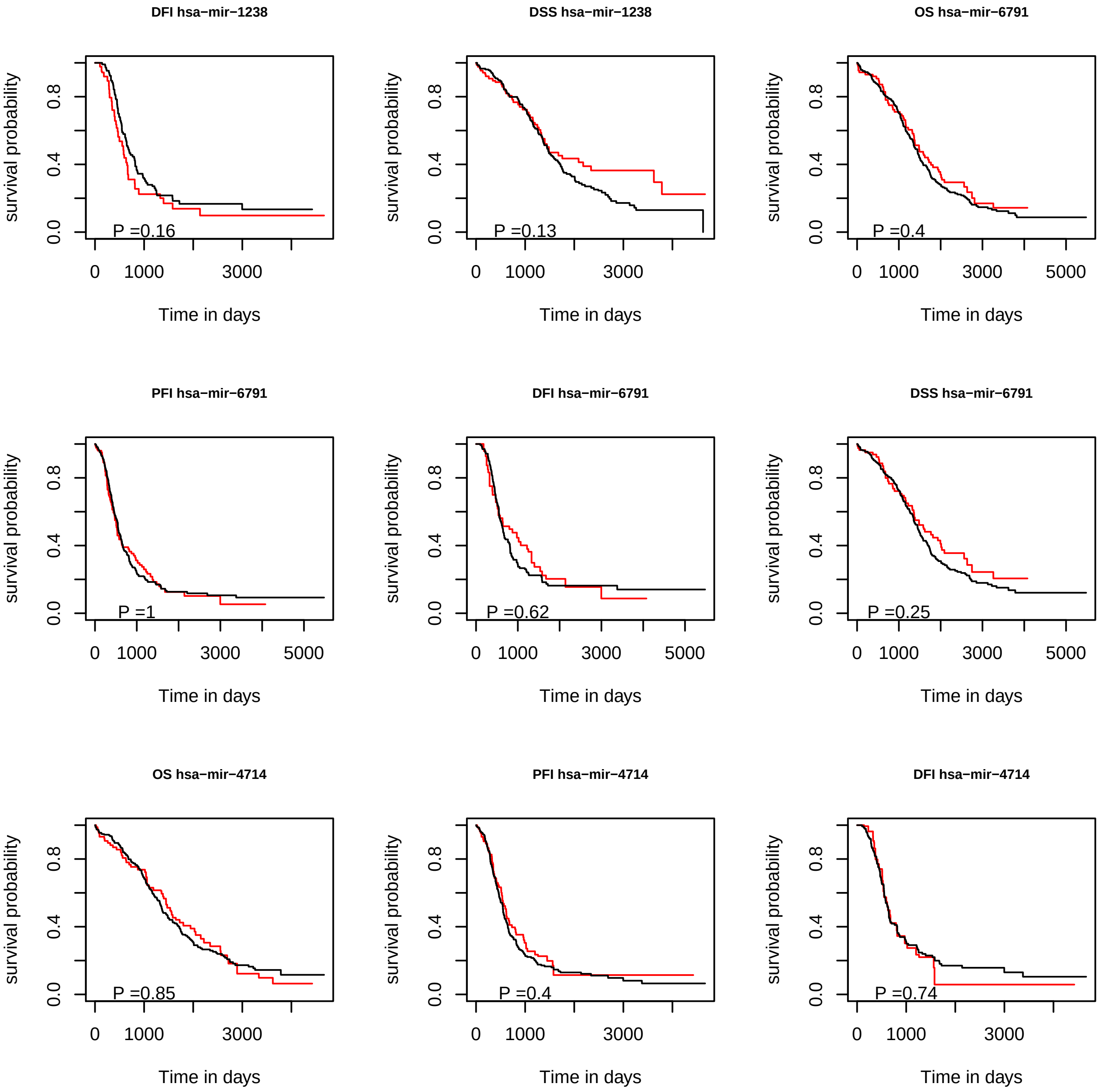
DFI hsa−mir−1238
P =0.16
DSS hsa−mir−1238
P =0.13
OS hsa−mir−6791
P =0.4
PFI hsa−mir−6791
P =1
DFI hsa−mir−6791
P =0.62
DSS hsa−mir−6791
P =0.25
OS hsa−mir−4714
P =0.85
PFI hsa−mir−4714
P =0.4
DFI hsa−mir−4714
P =0.74
survival probability
0.0
0.4
0.8
Time in days
0
1000
3000
5000

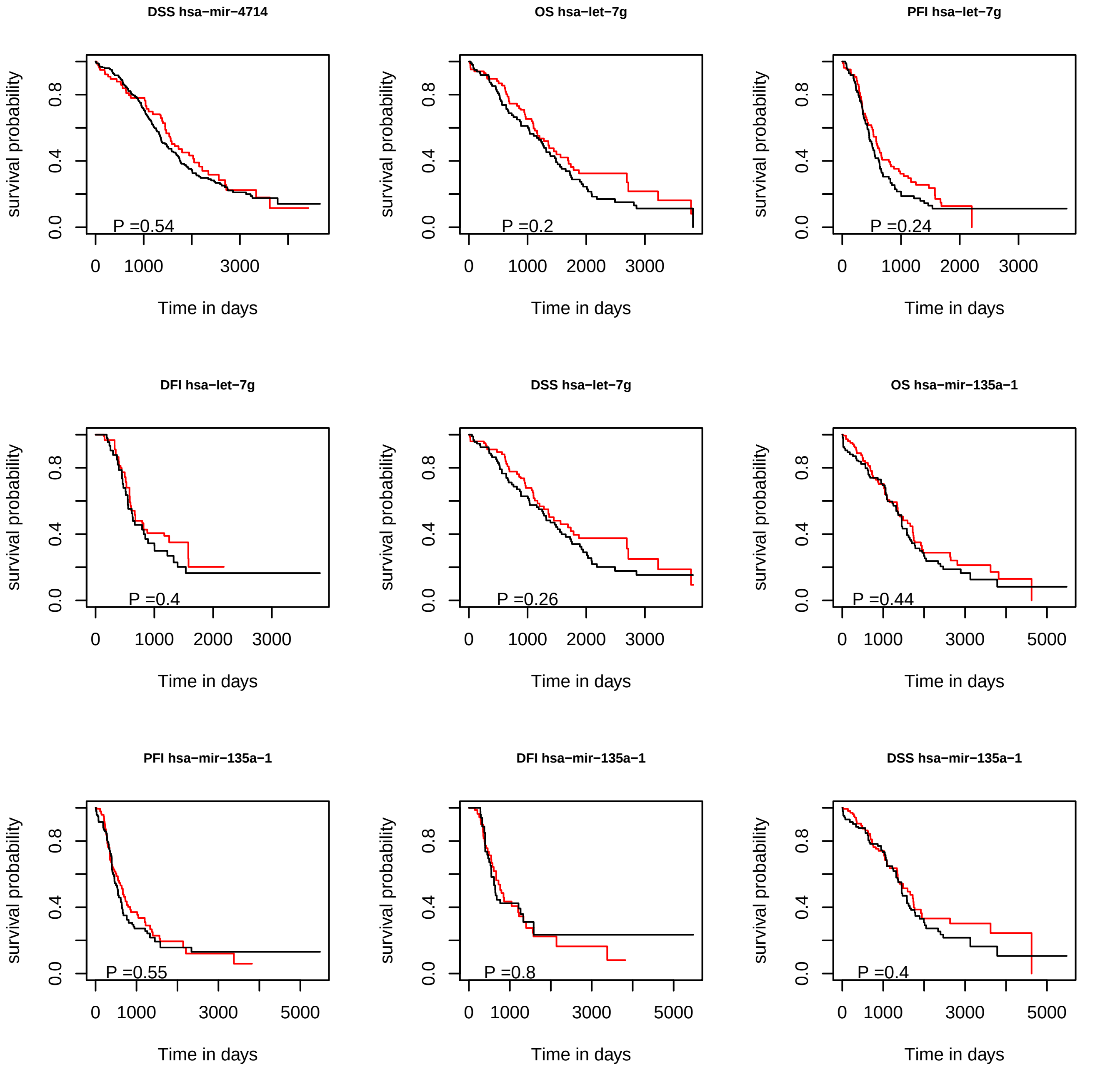
DSS hsa−mir−4714
P =0.54
OS hsa−let−7g
P =0.2
PFI hsa−let−7g
P =0.24
DFI hsa−let−7g
P =0.4
DSS hsa−let−7g
P =0.26
OS hsa−mir−135a−1
P =0.44
PFI hsa−mir−135a−1
P =0.55
DFI hsa−mir−135a−1
P =0.8
DSS hsa−mir−135a−1
P =0.4
survival probability
Time in days

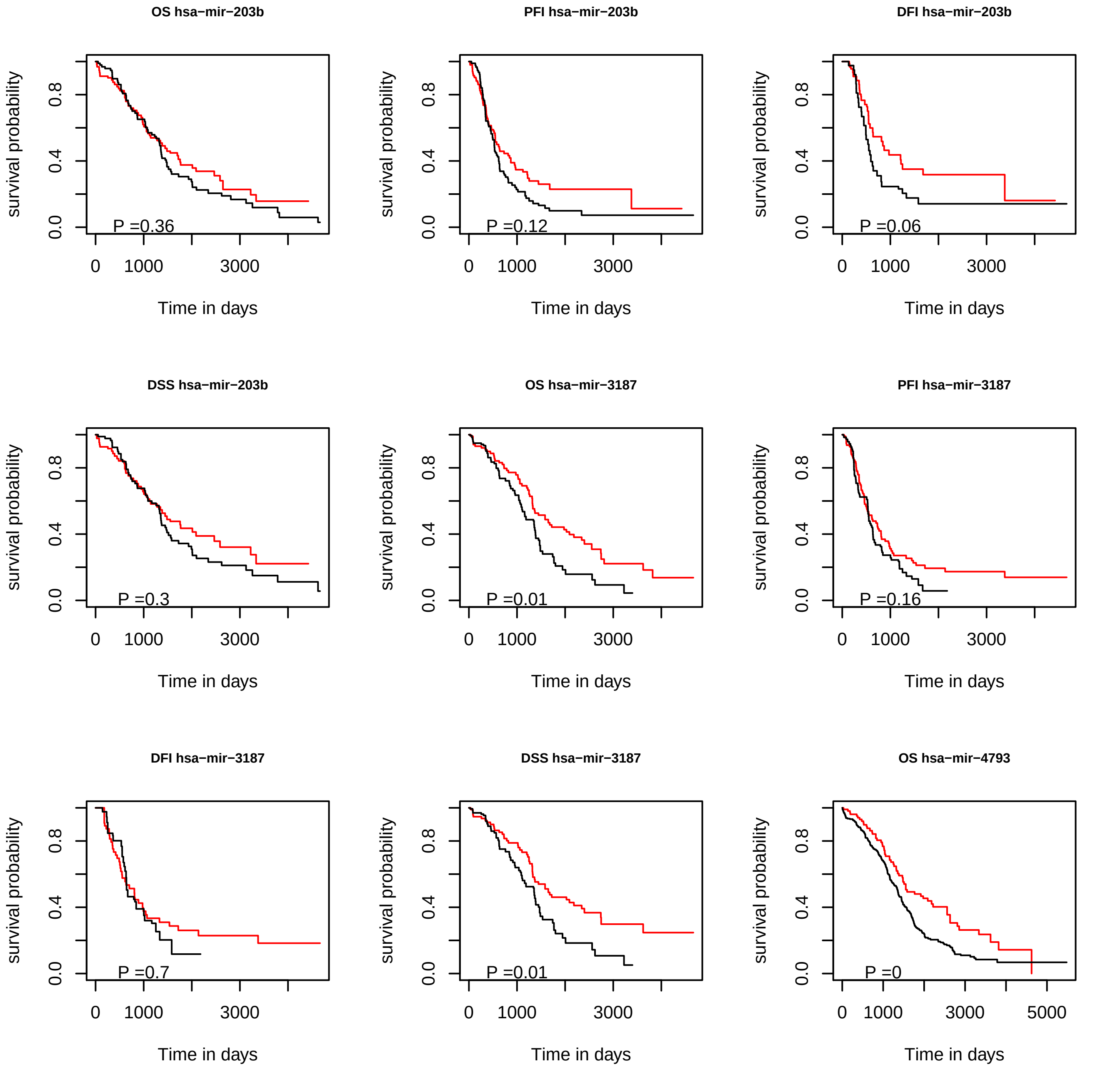
OS hsa−mir−203b
survival probability
0.0
0.4
0.8
P =0.36
0
1000
3000
Time in days
PFI hsa−mir−203b
survival probability
0.0
0.4
0.8
P =0.12
0
1000
3000
Time in days
DFI hsa−mir−203b
survival probability
0.0
0.4
0.8
P =0.06
0
1000
3000
Time in days
DSS hsa−mir−203b
survival probability
0.0
0.4
0.8
P =0.3
0
1000
3000
Time in days
OS hsa−mir−3187
survival probability
0.0
0.4
0.8
P =0.01
0
1000
3000
Time in days
PFI hsa−mir−3187
survival probability
0.0
0.4
0.8
P =0.16
0
1000
3000
Time in days
DFI hsa−mir−3187
survival probability
0.0
0.4
0.8
P =0.7
0
1000
3000
Time in days
DSS hsa−mir−3187
survival probability
0.0
0.4
0.8
P =0.01
0
1000
3000
Time in days
OS hsa−mir−4793
survival probability
0.0
0.4
0.8
P =0
0
1000
3000
5000
Time in days

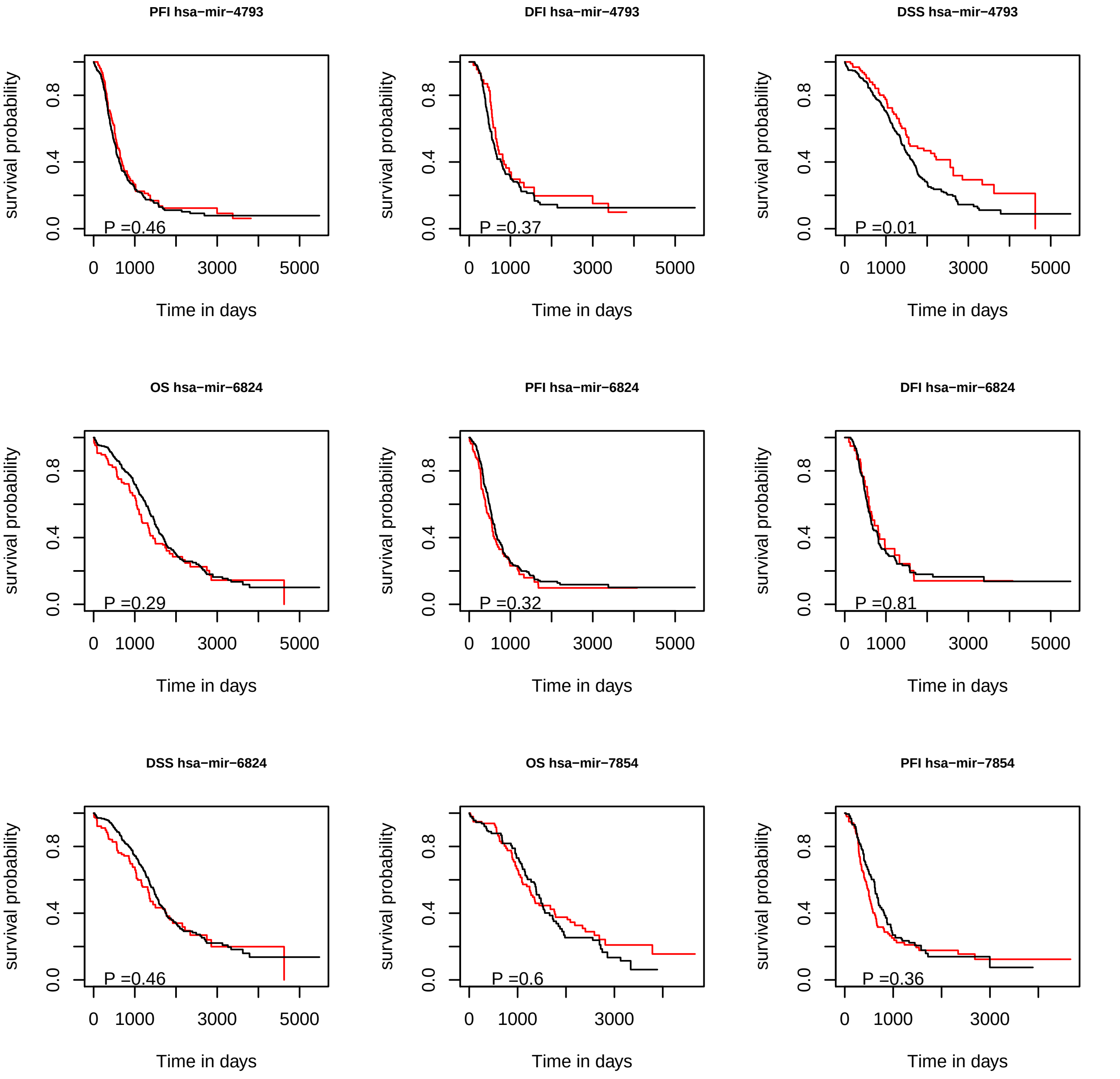
PFI hsa−mir−4793
P =0.46
DFI hsa−mir−4793
P =0.37
DSS hsa−mir−4793
P =0.01
OS hsa−mir−6824
P =0.29
PFI hsa−mir−6824
P =0.32
DFI hsa−mir−6824
P =0.81
DSS hsa−mir−6824
P =0.46
OS hsa−mir−7854
P =0.6
PFI hsa−mir−7854
P =0.36
survival probability
Time in days
0.0
0.4
0.8
0
1000
3000
5000

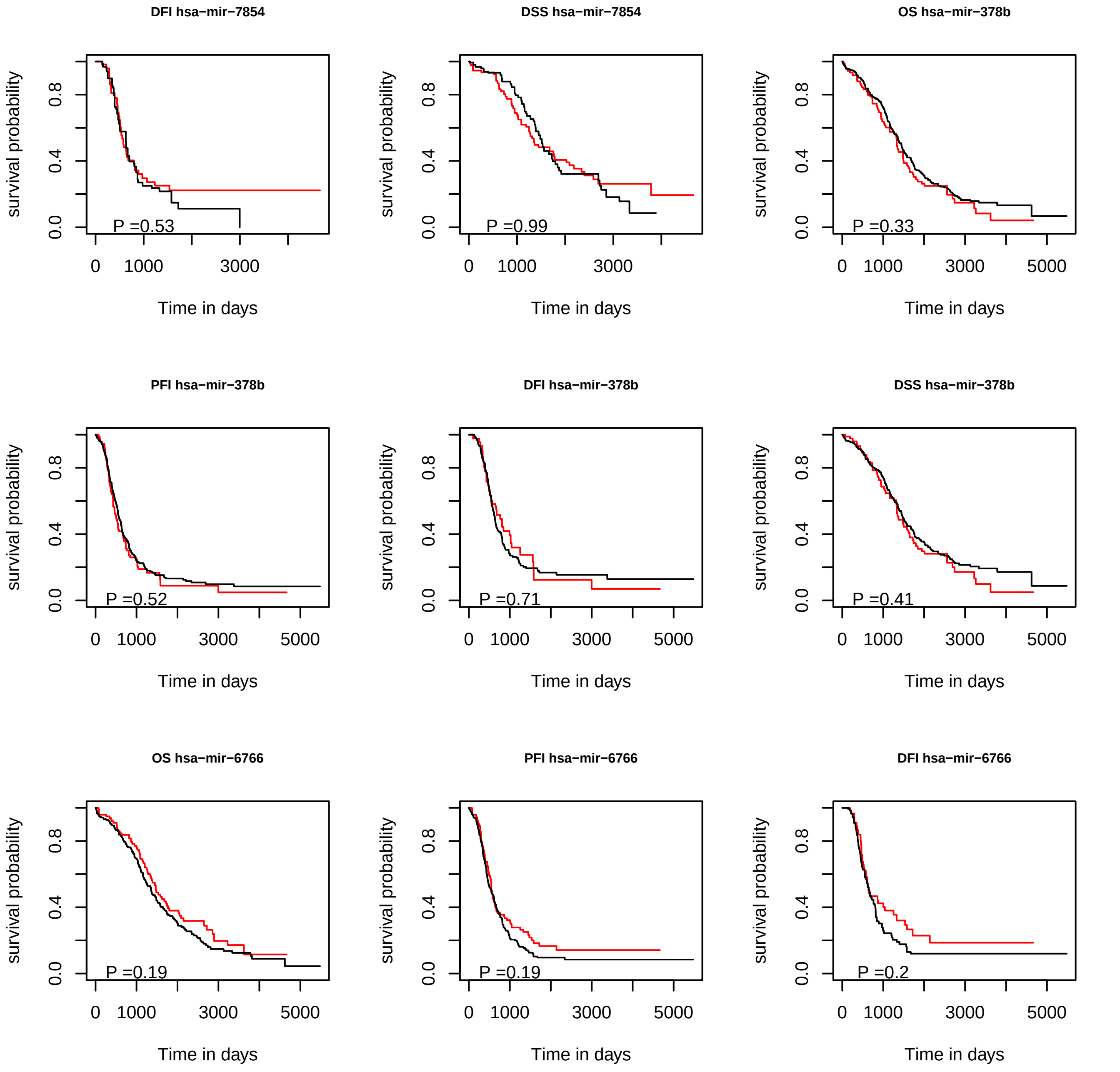
DFI hsa−mir−7854
P =0.53
DSS hsa−mir−7854
P =0.99
OS hsa−mir−378b
P =0.33
PFI hsa−mir−378b
P =0.52
DFI hsa−mir−378b
P =0.71
DSS hsa−mir−378b
P =0.41
OS hsa−mir−6766
P =0.19
PFI hsa−mir−6766
P =0.19
DFI hsa−mir−6766
P =0.2
survival probability
0.0
0.4
0.8
Time in days
0
1000
3000
5000

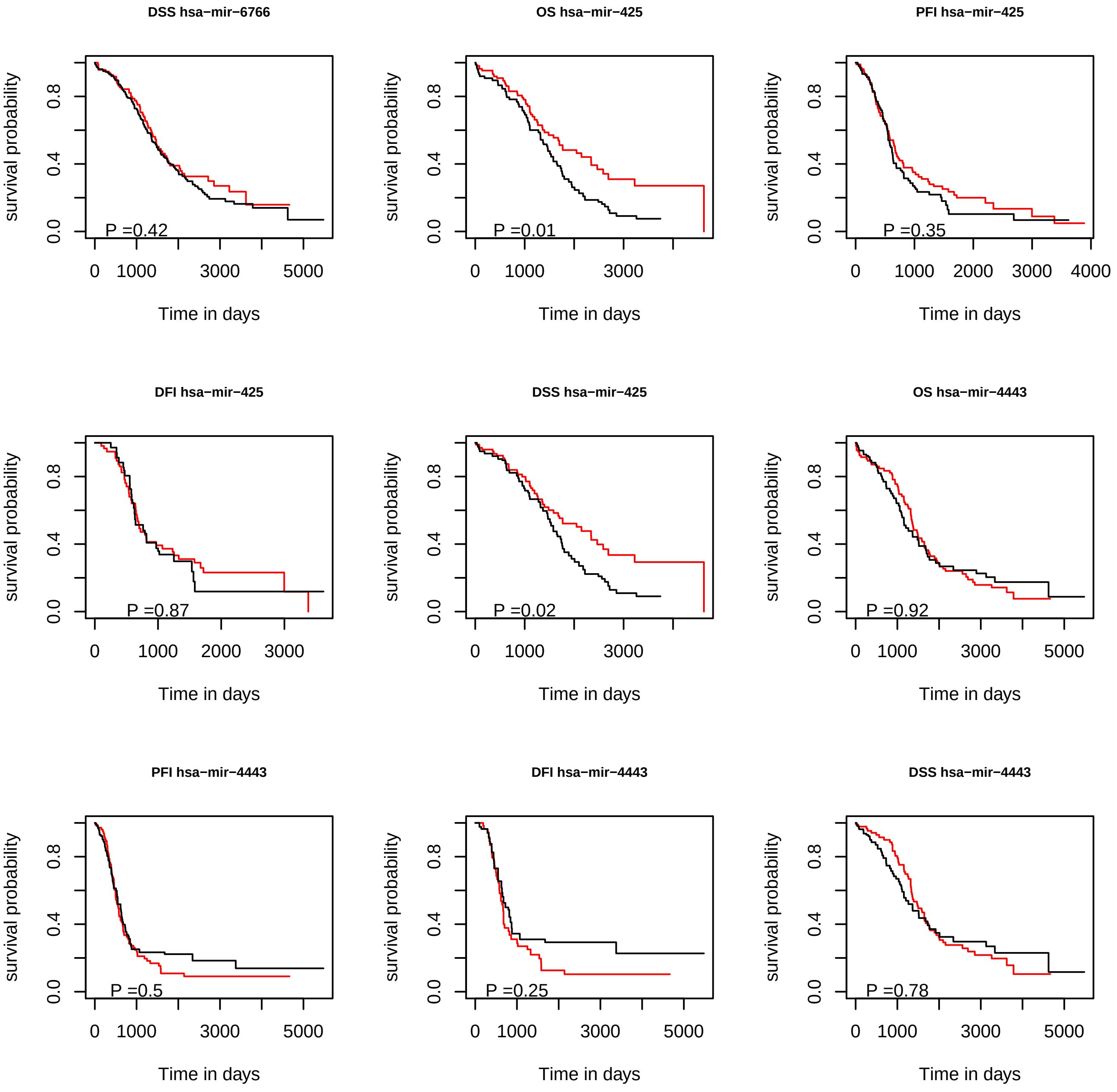

DSS hsa−mir−6766
P =0.42
OS hsa−mir−425
P =0.01
PFI hsa−mir−425
P =0.35
DFI hsa−mir−425
P =0.87
DSS hsa−mir−425
P =0.02
OS hsa−mir−4443
P =0.92
PFI hsa−mir−4443
P =0.5
DFI hsa−mir−4443
P =0.25
DSS hsa−mir−4443
P =0.78
survival probability
Time in days

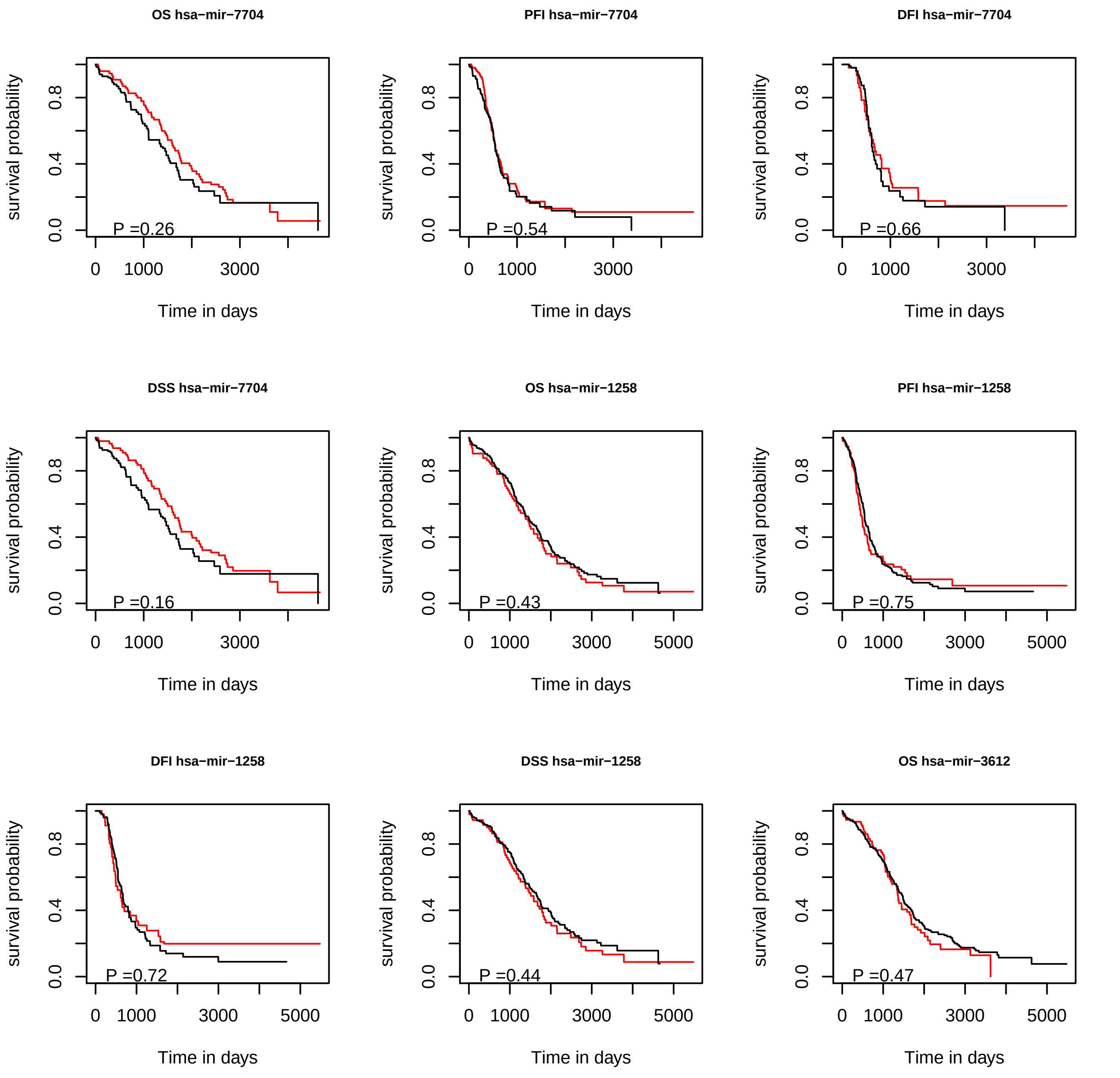

OS hsa−mir−7704
P =0.26
PFI hsa−mir−7704
P =0.54
DFI hsa−mir−7704
P =0.66
DSS hsa−mir−7704
P =0.16
OS hsa−mir−1258
P =0.43
PFI hsa−mir−1258
P =0.75
DFI hsa−mir−1258
P =0.72
DSS hsa−mir−1258
P =0.44
OS hsa−mir−3612
P =0.47
survival probability
0.0
0.4
0.8
0
1000
3000
5000
Time in days

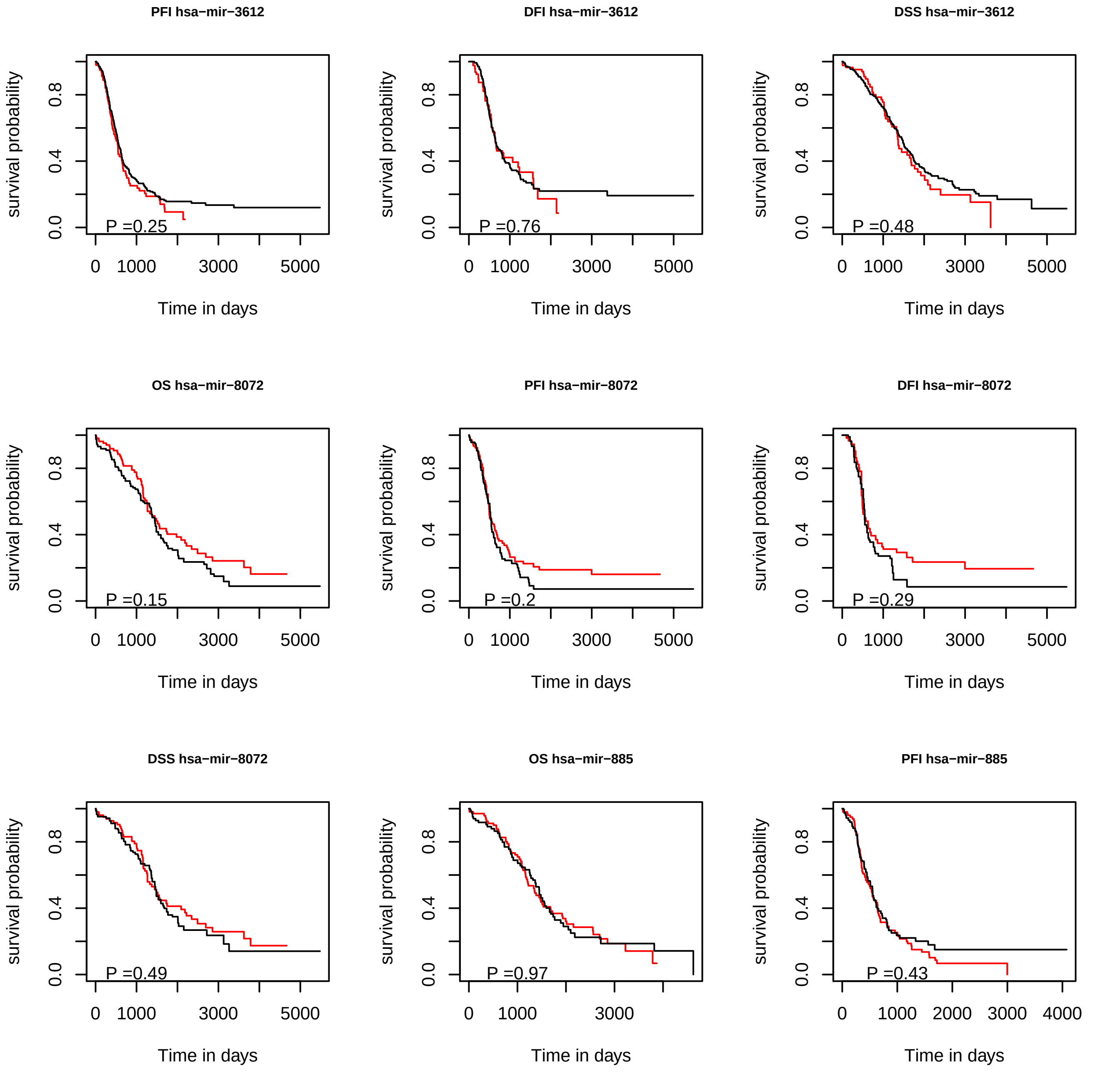
PFI hsa−mir−3612
P =0.25
DFI hsa−mir−3612
P =0.76
DSS hsa−mir−3612
P =0.48
OS hsa−mir−8072
P =0.15
PFI hsa−mir−8072
P =0.2
DFI hsa−mir−8072
P =0.29
DSS hsa−mir−8072
P =0.49
OS hsa−mir−885
P =0.97
PFI hsa−mir−885
P =0.43
survival probability
Time in days
0.0
0.4
0.8
0
1000
2000
3000
4000
5000

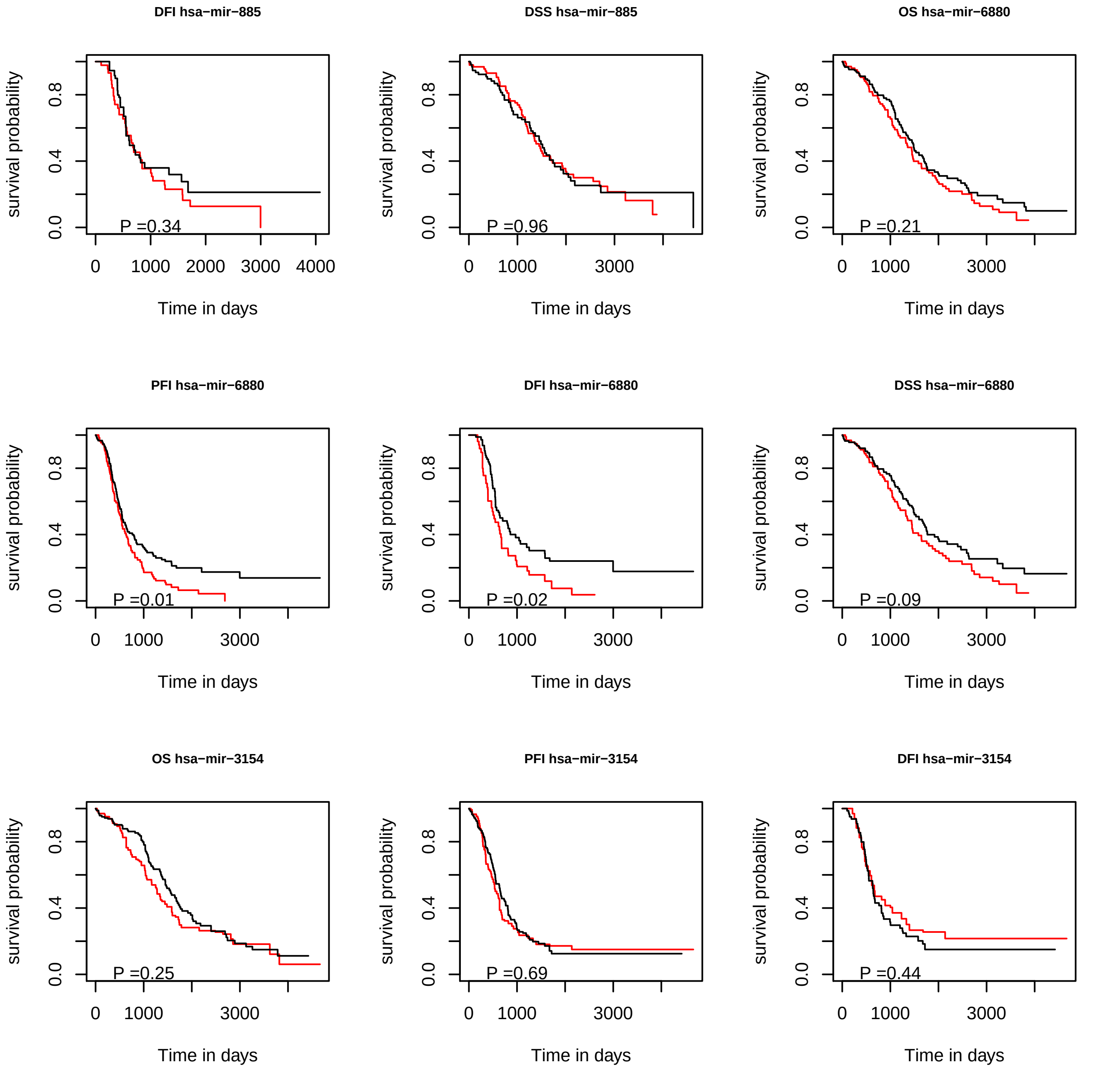
DFI hsa−mir−885
P =0.34
DSS hsa−mir−885
P =0.96
OS hsa−mir−6880
P =0.21
PFI hsa−mir−6880
P =0.01
DFI hsa−mir−6880
P =0.02
DSS hsa−mir−6880
P =0.09
OS hsa−mir−3154
P =0.25
PFI hsa−mir−3154
P =0.69
DFI hsa−mir−3154
P =0.44
survival probability
Time in days
0.0
0.4
0.8
0
1000
2000
3000
4000

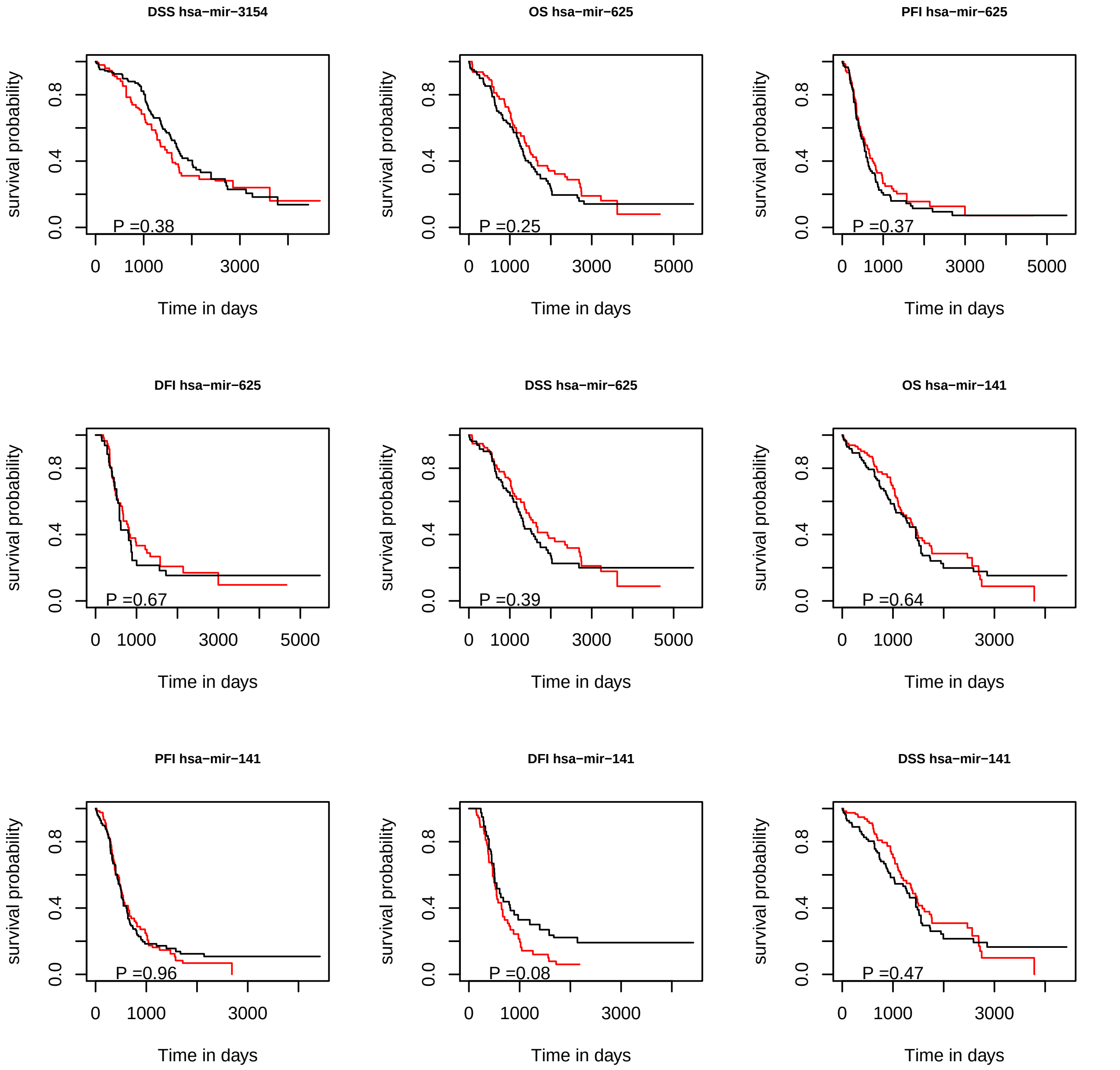
DSS hsa−mir−3154
P =0.38
OS hsa−mir−625
P =0.25
PFI hsa−mir−625
P =0.37
DFI hsa−mir−625
P =0.67
DSS hsa−mir−625
P =0.39
OS hsa−mir−141
P =0.64
PFI hsa−mir−141
P =0.96
DFI hsa−mir−141
P =0.08
DSS hsa−mir−141
P =0.47
survival probability
Time in days
0.0
0.4
0.8
0
1000
3000
5000

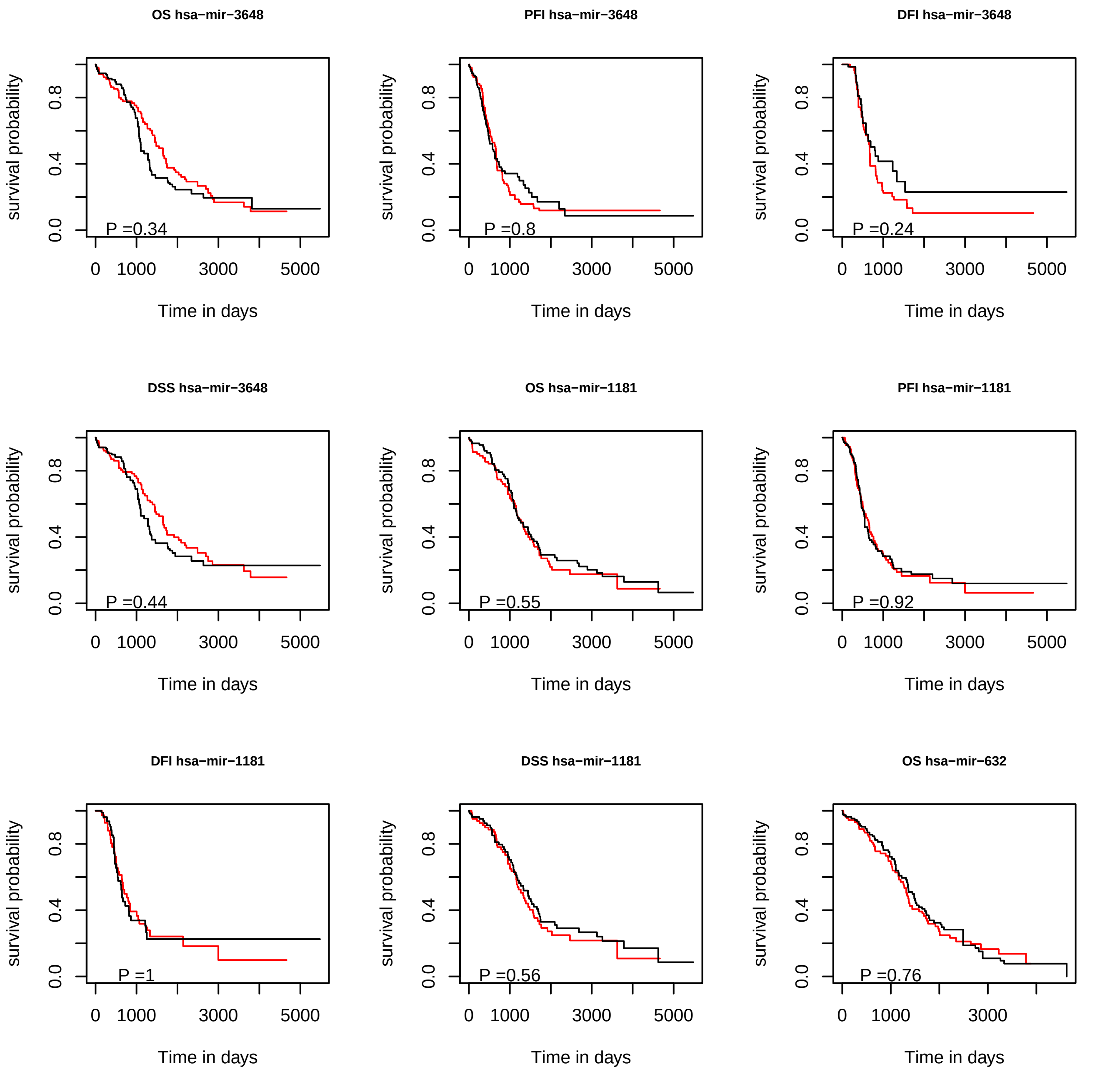
OS hsa−mir−3648
P =0.34
PFI hsa−mir−3648
P =0.8
DFI hsa−mir−3648
P =0.24
DSS hsa−mir−3648
P =0.44
OS hsa−mir−1181
P =0.55
PFI hsa−mir−1181
P =0.92
DFI hsa−mir−1181
P =1
DSS hsa−mir−1181
P =0.56
OS hsa−mir−632
P =0.76
survival probability
Time in days
0.0
0.4
0.8
0
1000
3000
5000

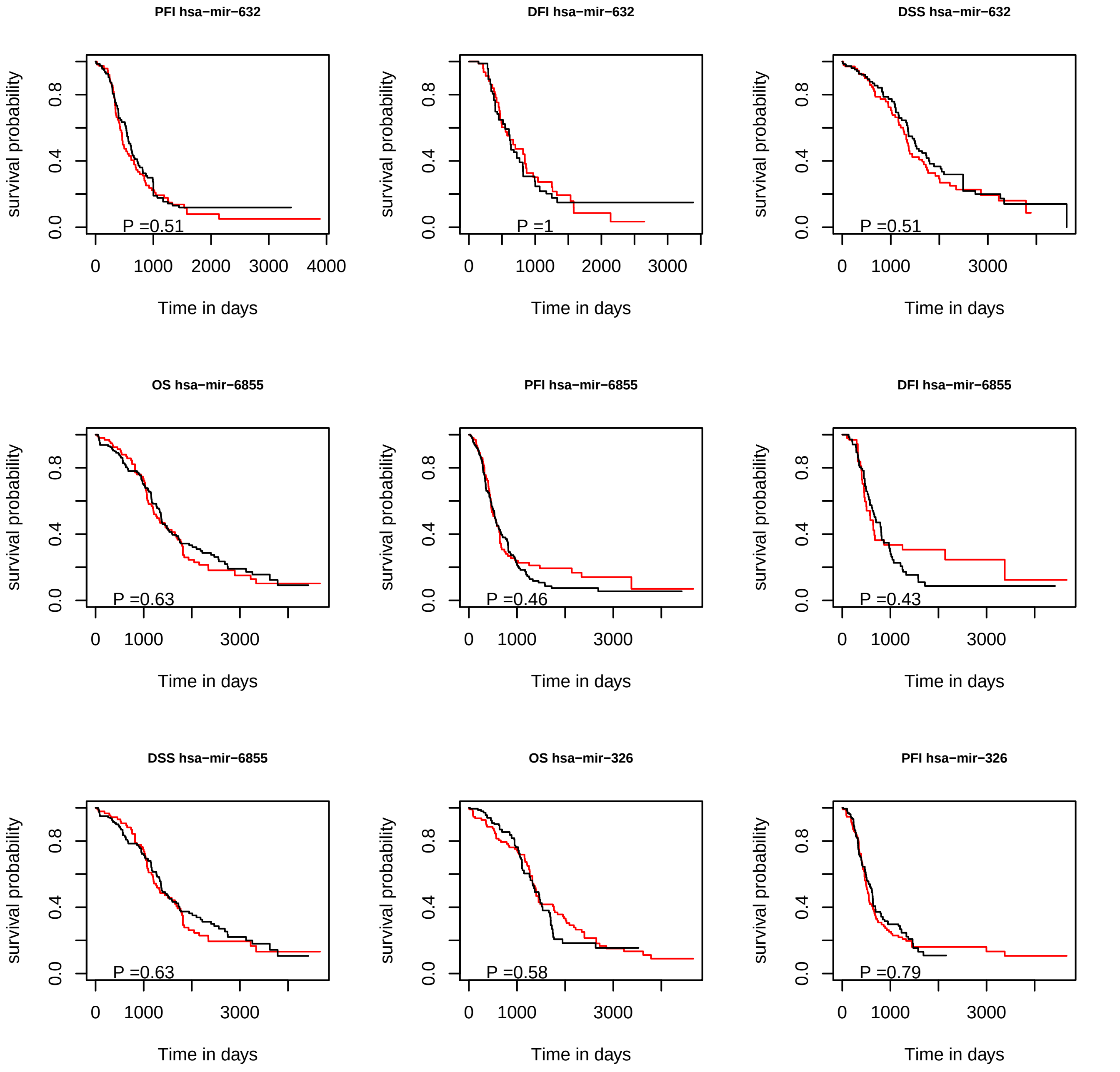
PFI hsa−mir−632
P =0.51
DFI hsa−mir−632
P =1
DSS hsa−mir−632
P =0.51
OS hsa−mir−6855
P =0.63
PFI hsa−mir−6855
P =0.46
DFI hsa−mir−6855
P =0.43
DSS hsa−mir−6855
P =0.63
OS hsa−mir−326
P =0.58
PFI hsa−mir−326
P =0.79
survival probability
Time in days
0.0
0.4
0.8
0
1000
2000
3000
4000

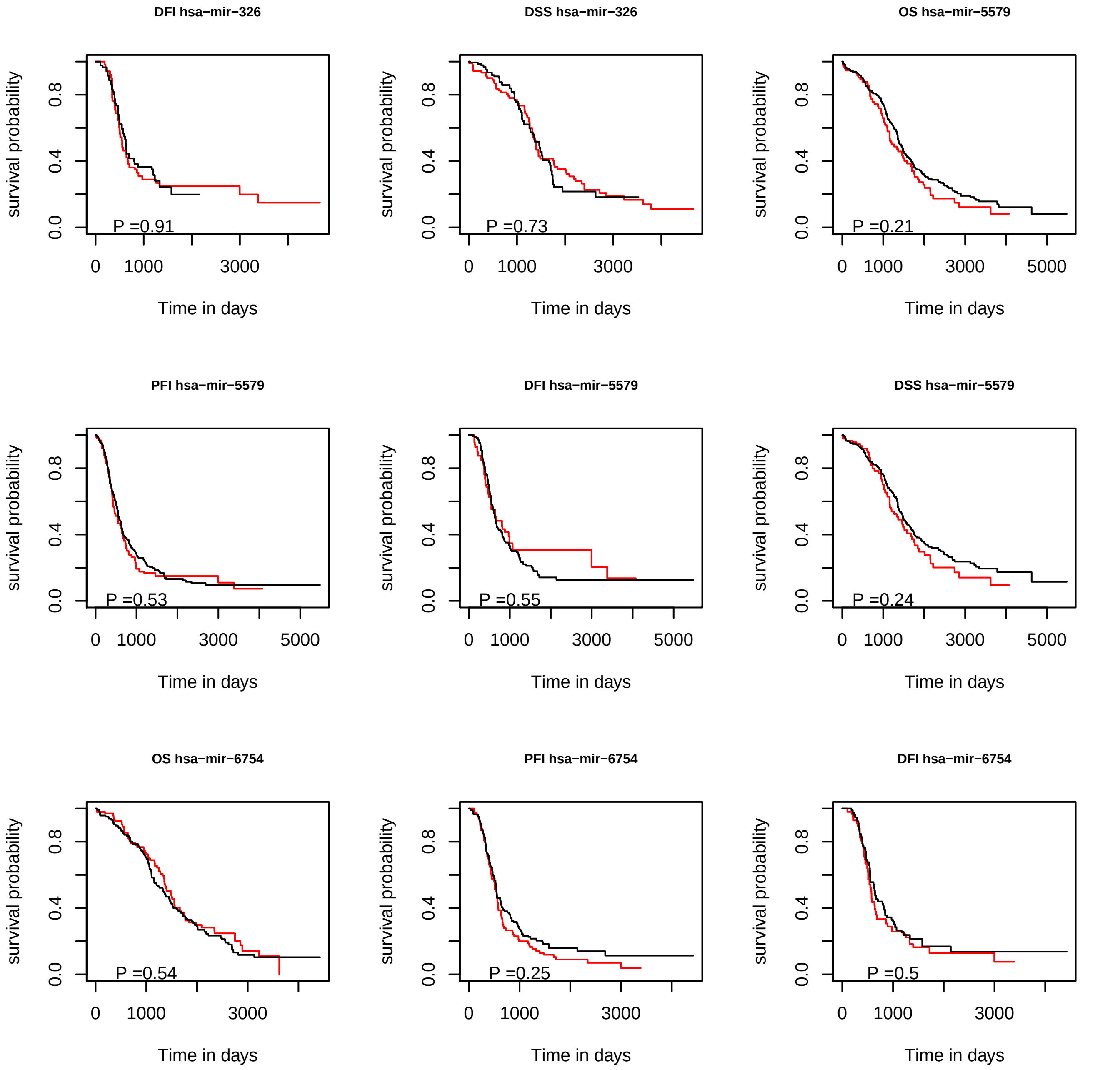
DFI hsa−mir−326
P =0.91
DSS hsa−mir−326
P =0.73
OS hsa−mir−5579
P =0.21
PFI hsa−mir−5579
P =0.53
DFI hsa−mir−5579
P =0.55
DSS hsa−mir−5579
P =0.24
OS hsa−mir−6754
P =0.54
PFI hsa−mir−6754
P =0.25
DFI hsa−mir−6754
P =0.5
survival probability
0.0
0.4
0.8
Time in days
0
1000
3000
5000

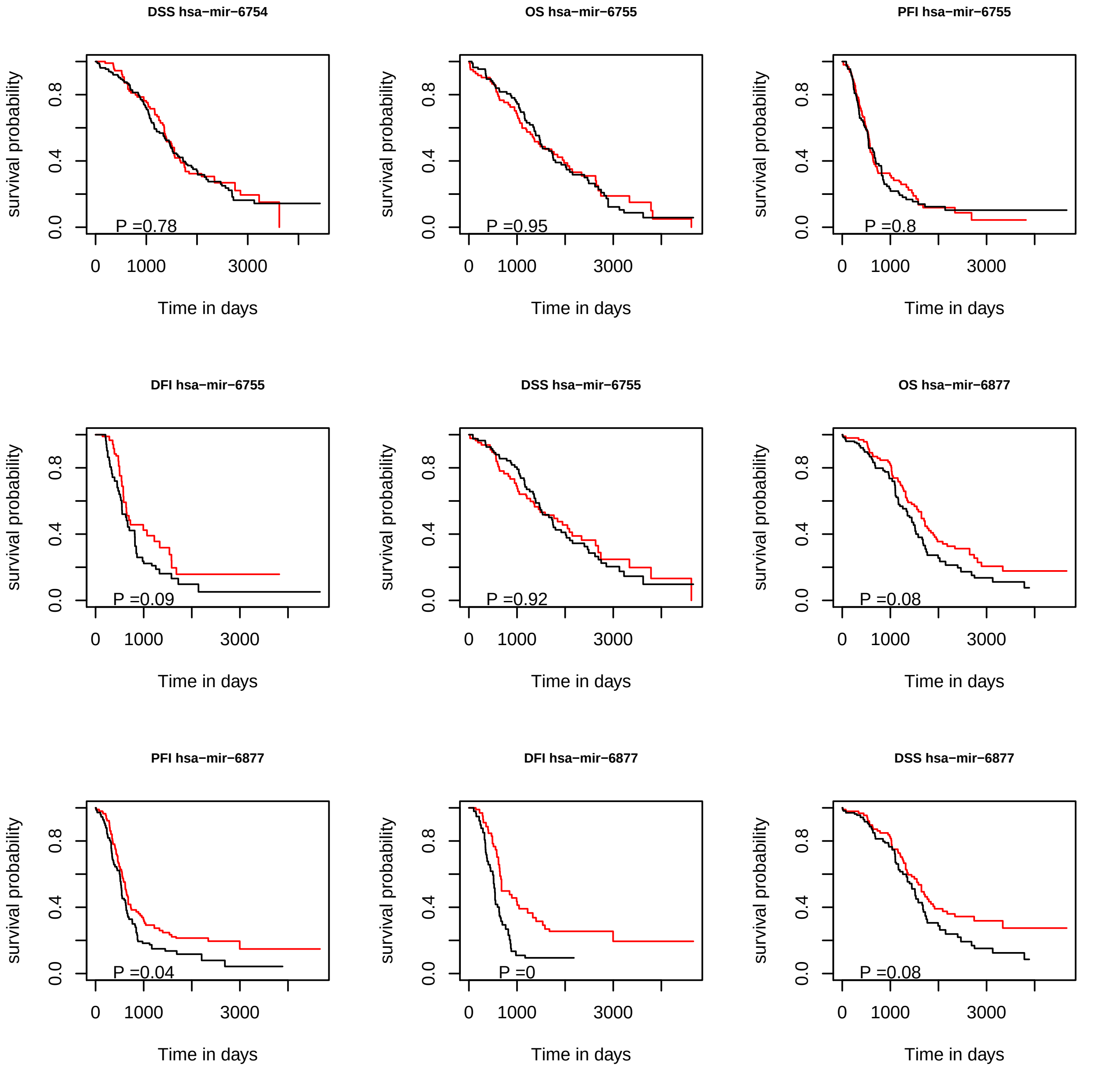
DSS hsa−mir−6754
P =0.78
OS hsa−mir−6755
P =0.95
PFI hsa−mir−6755
P =0.8
DFI hsa−mir−6755
P =0.09
DSS hsa−mir−6755
P =0.92
OS hsa−mir−6877
P =0.08
PFI hsa−mir−6877
P =0.04
DFI hsa−mir−6877
P =0
DSS hsa−mir−6877
P =0.08
survival probability
0.0
0.4
0.8
0
1000
3000
Time in days

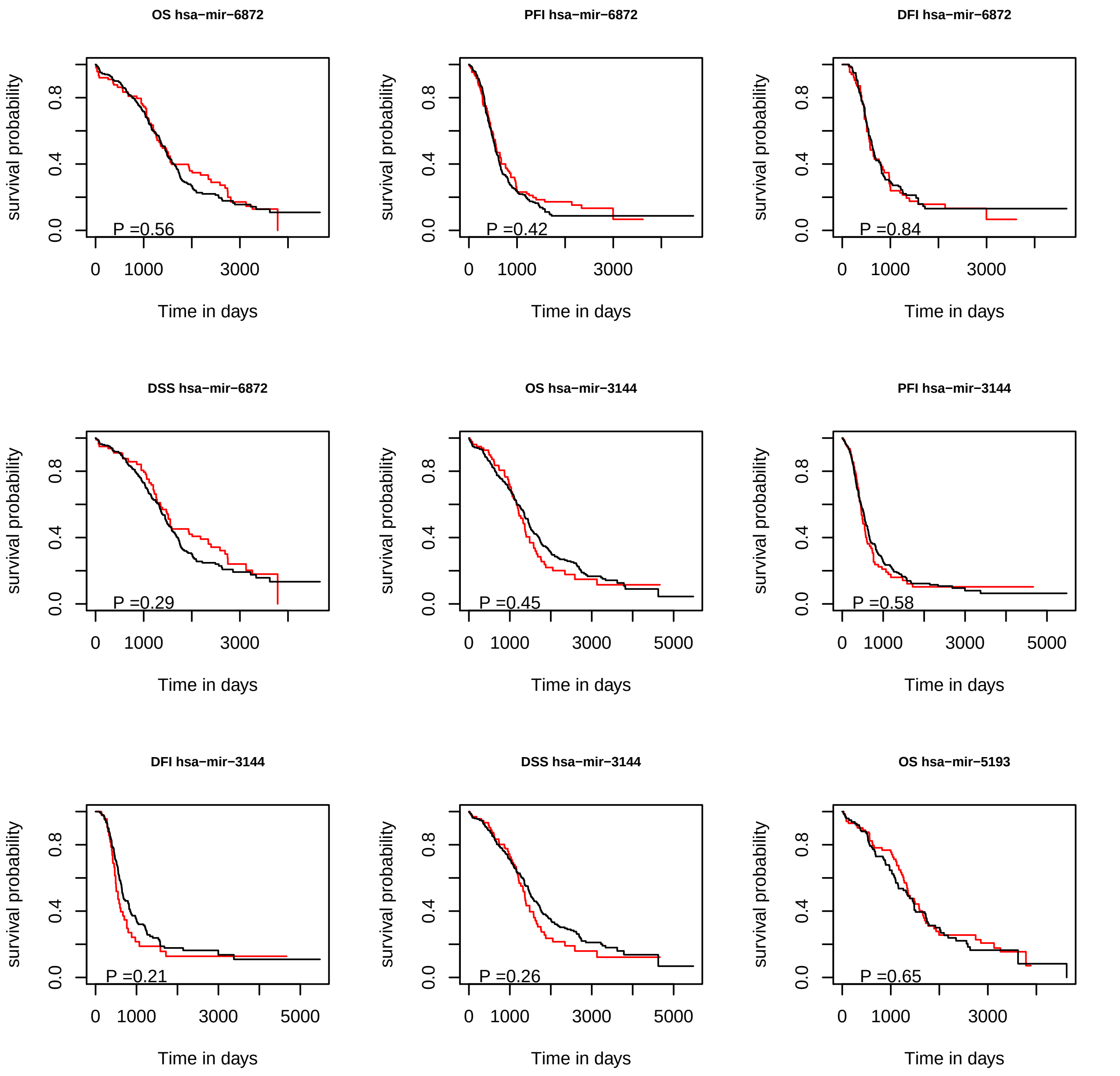
OS hsa−mir−6872
P =0.56
PFI hsa−mir−6872
P =0.42
DFI hsa−mir−6872
P =0.84
DSS hsa−mir−6872
P =0.29
OS hsa−mir−3144
P =0.45
PFI hsa−mir−3144
P =0.58
DFI hsa−mir−3144
P =0.21
DSS hsa−mir−3144
P =0.26
OS hsa−mir−5193
P =0.65
survival probability
Time in days
0.0
0.4
0.8
0
1000
3000
5000

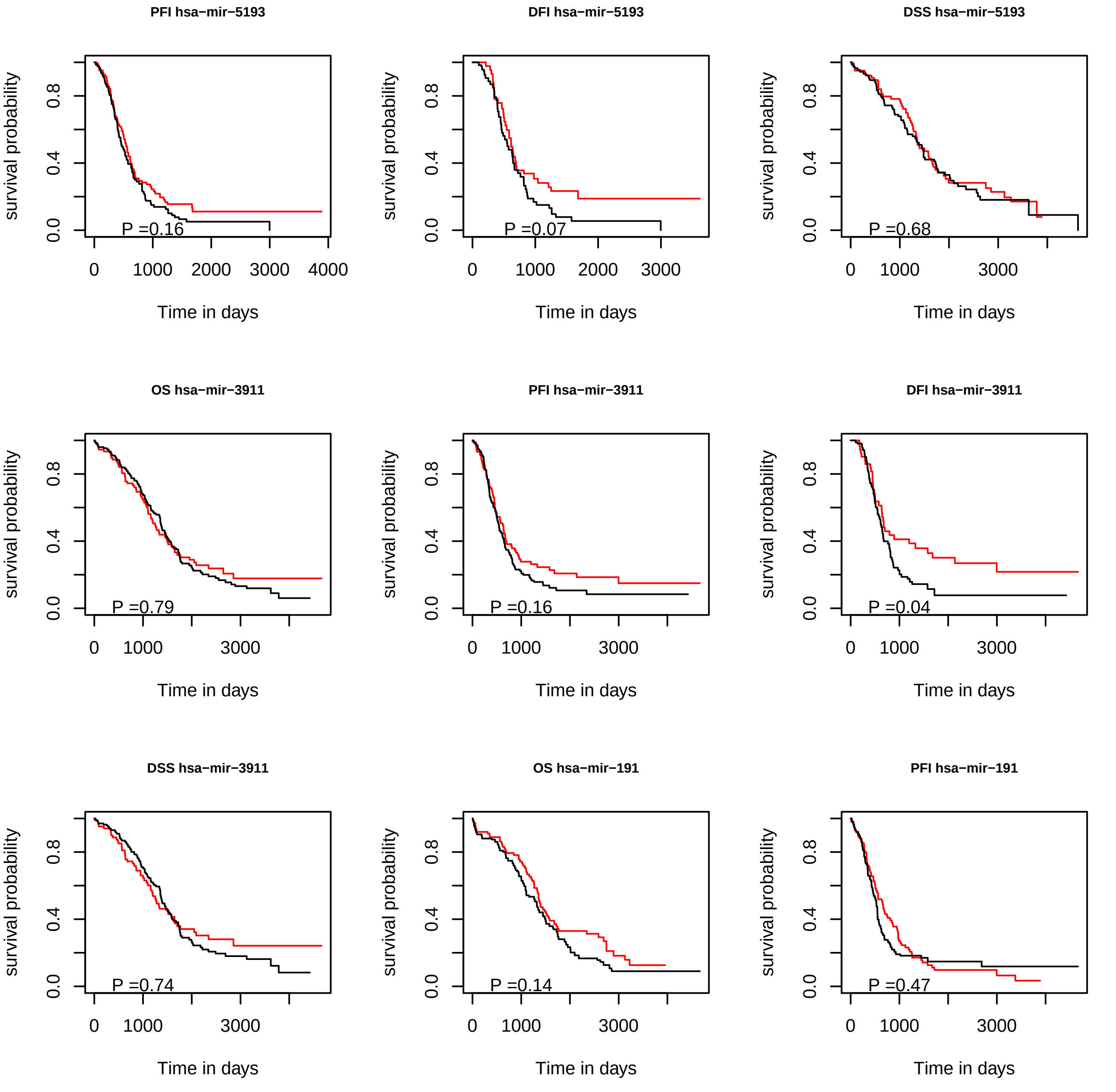

PFI hsa−mir−5193
P =0.16
DFI hsa−mir−5193
P =0.07
DSS hsa−mir−5193
P =0.68
OS hsa−mir−3911
P =0.79
PFI hsa−mir−3911
P =0.16
DFI hsa−mir−3911
P =0.04
DSS hsa−mir−3911
P =0.74
OS hsa−mir−191
P =0.14
PFI hsa−mir−191
P =0.47
survival probability
0.0
0.4
0.8
Time in days
0
1000
2000
3000
4000

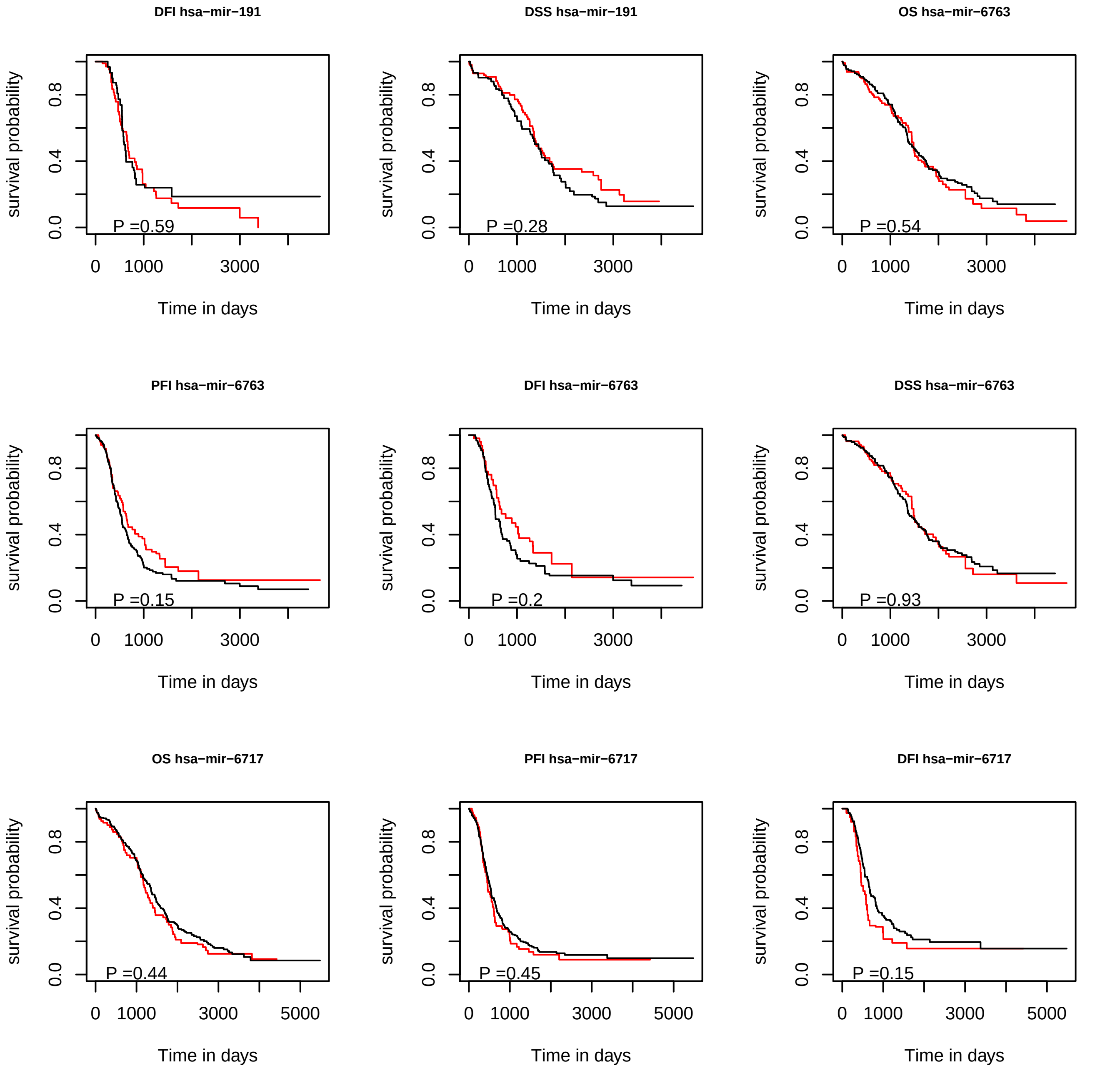
DFI hsa−mir−191
P =0.59
DSS hsa−mir−191
P =0.28
OS hsa−mir−6763
P =0.54
PFI hsa−mir−6763
P =0.15
DFI hsa−mir−6763
P =0.2
DSS hsa−mir−6763
P =0.93
OS hsa−mir−6717
P =0.44
PFI hsa−mir−6717
P =0.45
DFI hsa−mir−6717
P =0.15
survival probability
0.0
0.4
0.8
Time in days
0
1000
3000
5000

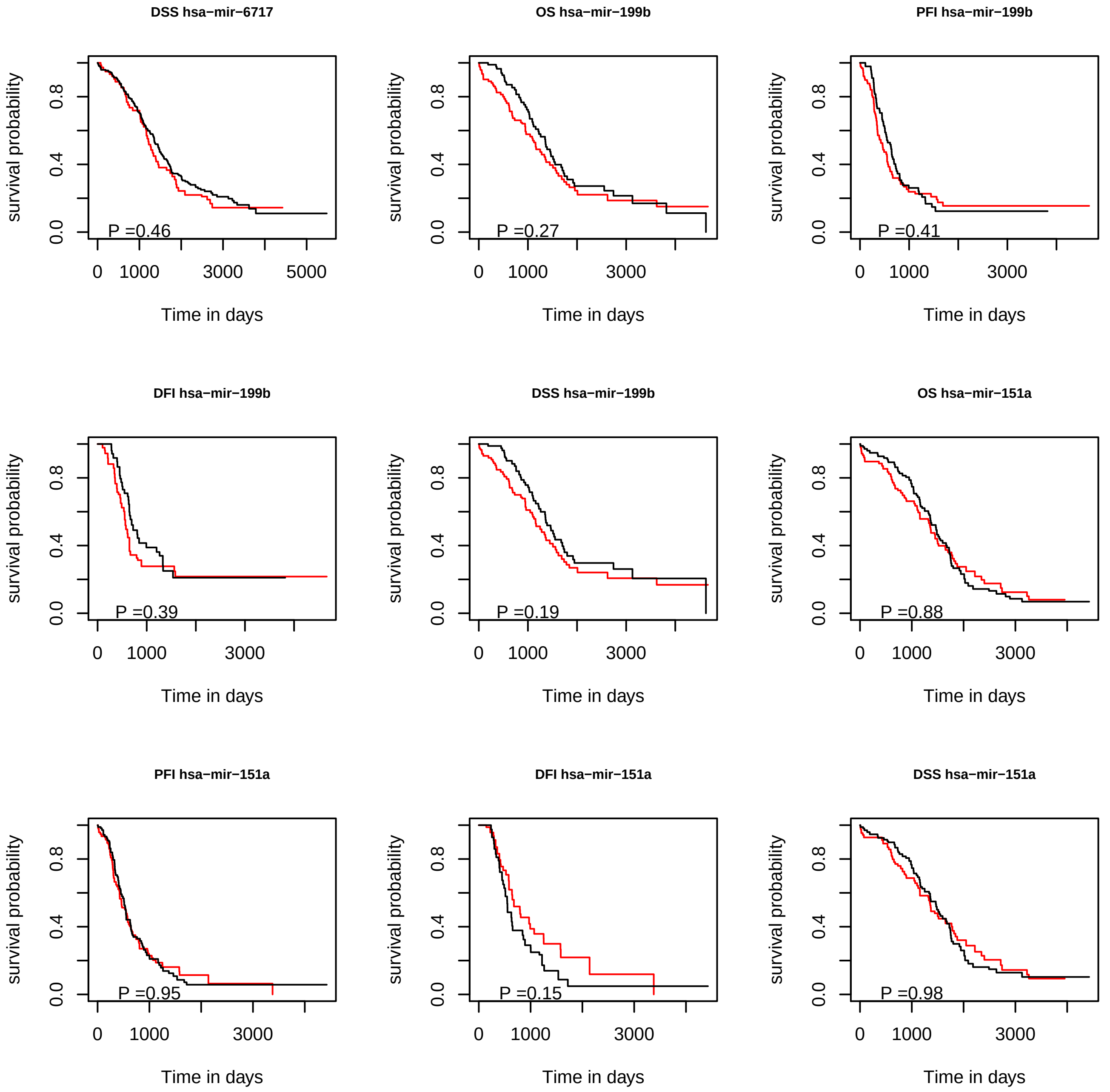
DSS hsa−mir−6717
P =0.46
OS hsa−mir−199b
P =0.27
PFI hsa−mir−199b
P =0.41
DFI hsa−mir−199b
P =0.39
DSS hsa−mir−199b
P =0.19
OS hsa−mir−151a
P =0.88
PFI hsa−mir−151a
P =0.95
DFI hsa−mir−151a
P =0.15
DSS hsa−mir−151a
P =0.98
survival probability
Time in days

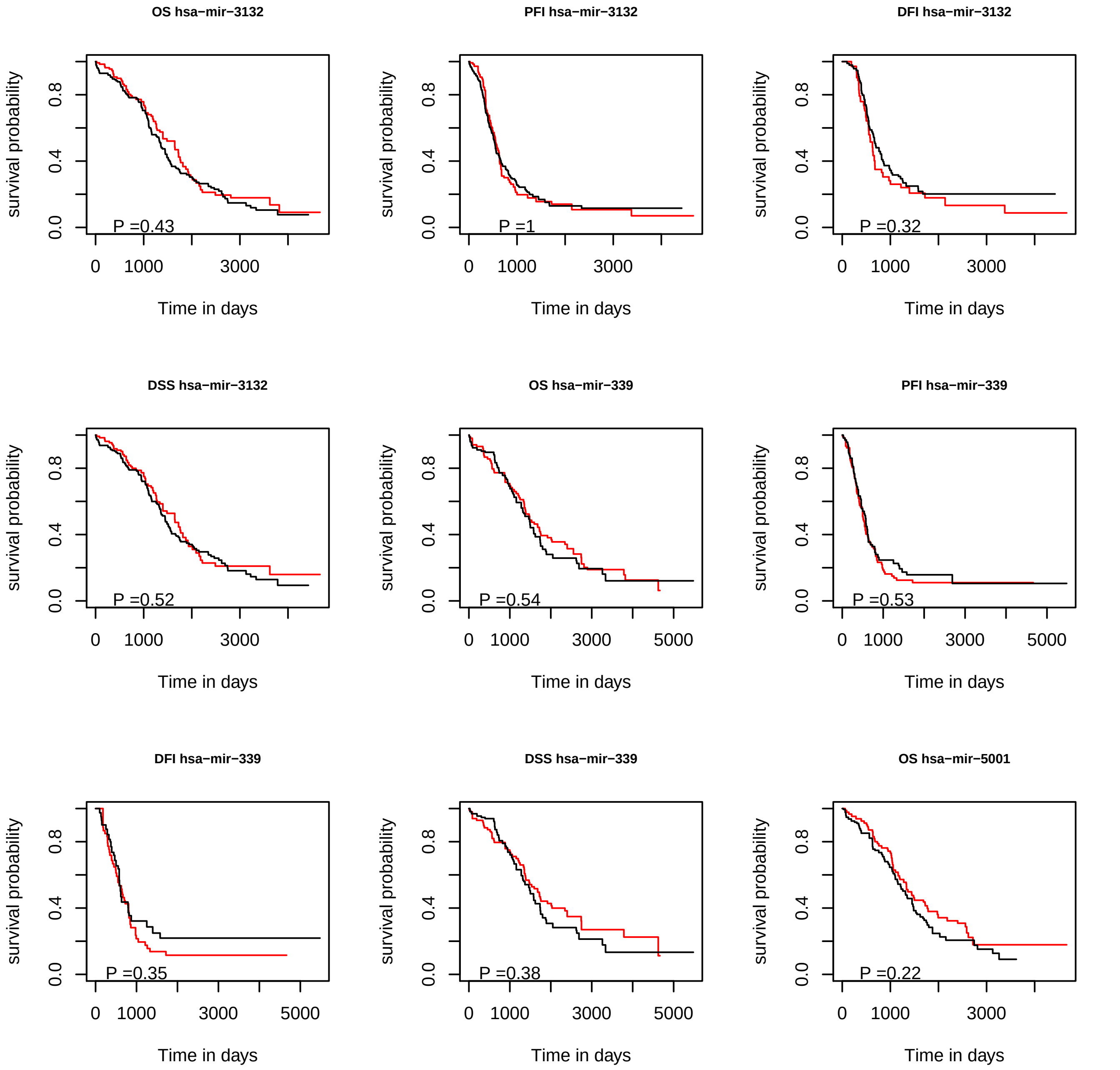
OS hsa−mir−3132
survival probability
Time in days
P =0.43
PFI hsa−mir−3132
survival probability
Time in days
P =1
DFI hsa−mir−3132
survival probability
Time in days
P =0.32
DSS hsa−mir−3132
survival probability
Time in days
P =0.52
OS hsa−mir−339
survival probability
Time in days
P =0.54
PFI hsa−mir−339
survival probability
Time in days
P =0.53
DFI hsa−mir−339
survival probability
Time in days
P =0.35
DSS hsa−mir−339
survival probability
Time in days
P =0.38
OS hsa−mir−5001
survival probability
Time in days
P =0.22

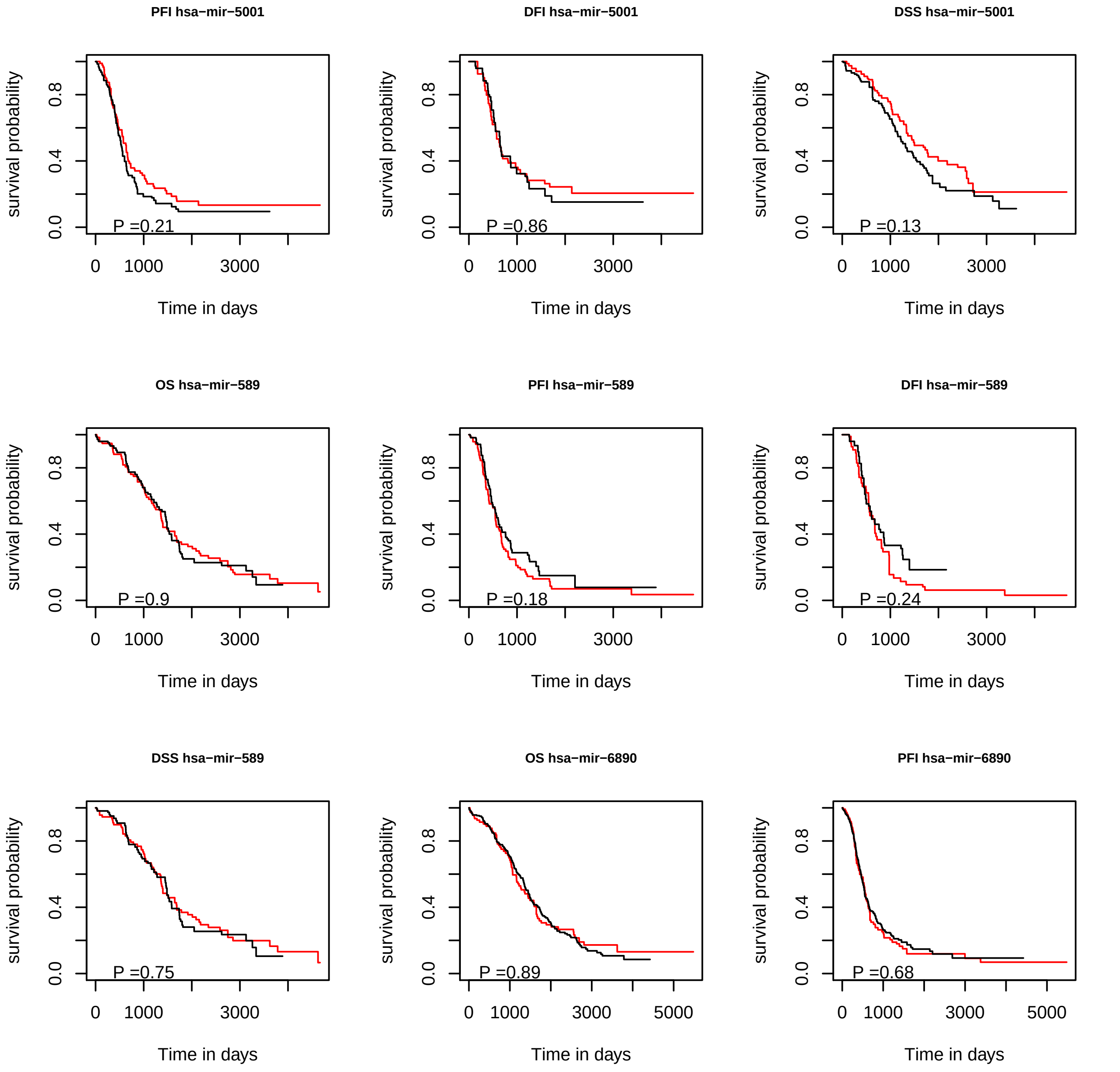
PFI hsa−mir−5001
P =0.21
DFI hsa−mir−5001
P =0.86
DSS hsa−mir−5001
P =0.13
OS hsa−mir−589
P =0.9
PFI hsa−mir−589
P =0.18
DFI hsa−mir−589
P =0.24
DSS hsa−mir−589
P =0.75
OS hsa−mir−6890
P =0.89
PFI hsa−mir−6890
P =0.68
survival probability
0.0
0.4
0.8
Time in days
0
1000
3000
5000

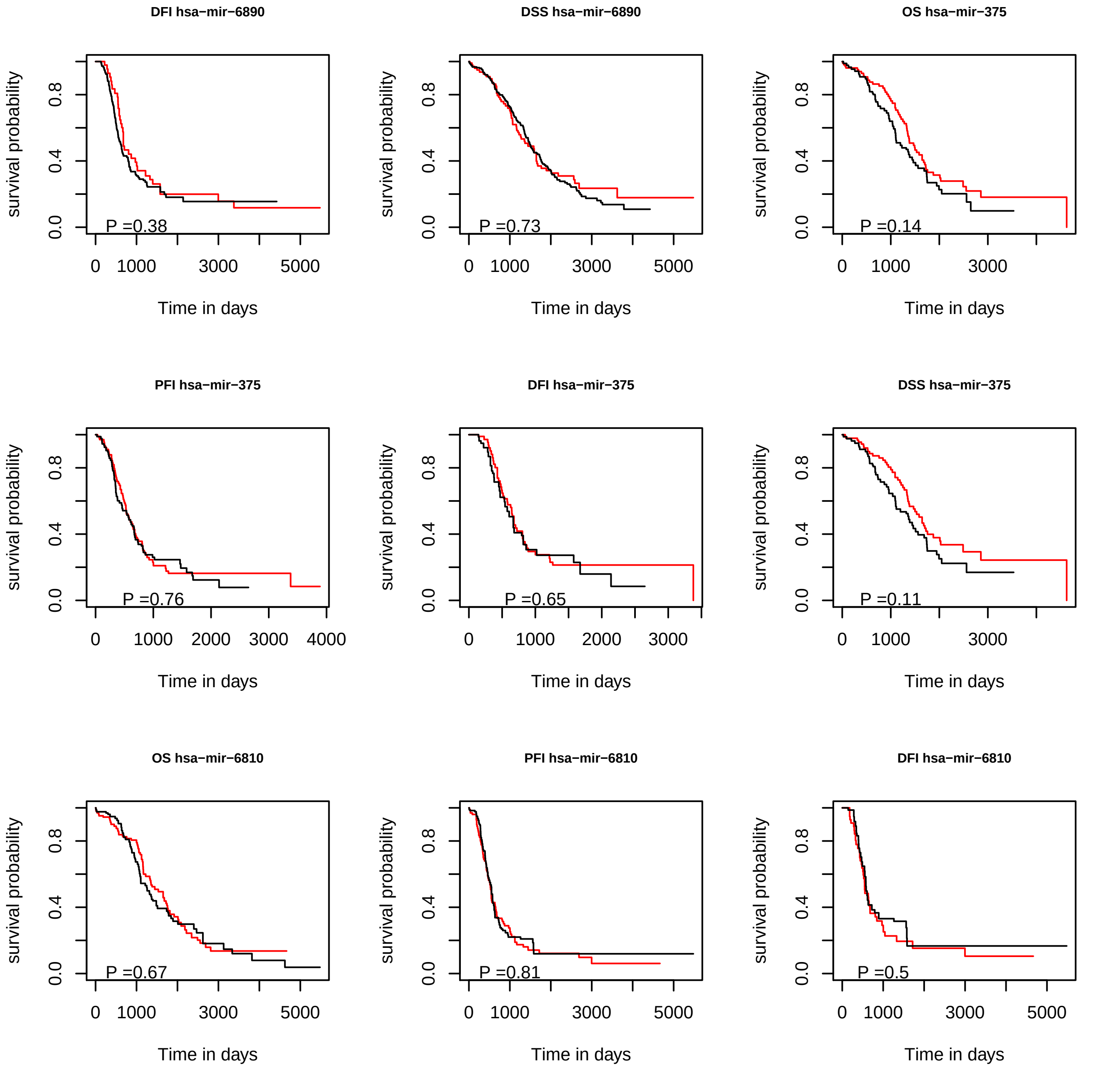
DFI hsa−mir−6890
P =0.38
DSS hsa−mir−6890
P =0.73
OS hsa−mir−375
P =0.14
PFI hsa−mir−375
P =0.76
DFI hsa−mir−375
P =0.65
DSS hsa−mir−375
P =0.11
OS hsa−mir−6810
P =0.67
PFI hsa−mir−6810
P =0.81
DFI hsa−mir−6810
P =0.5
survival probability
Time in days
0.0
0.4
0.8
0
1000
2000
3000
4000
5000

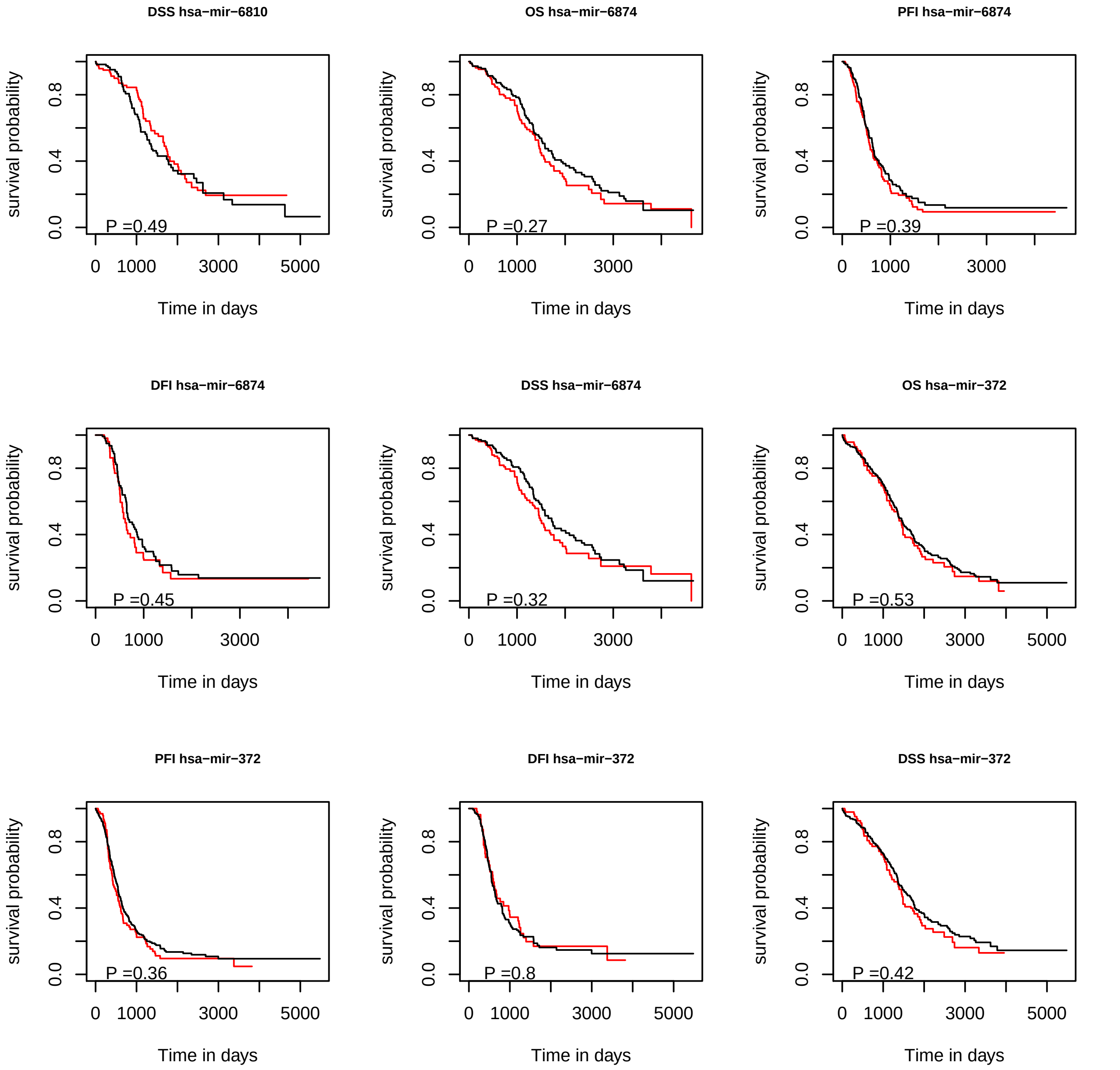
DSS hsa−mir−6810
P =0.49
OS hsa−mir−6874
P =0.27
PFI hsa−mir−6874
P =0.39
DFI hsa−mir−6874
P =0.45
DSS hsa−mir−6874
P =0.32
OS hsa−mir−372
P =0.53
PFI hsa−mir−372
P =0.36
DFI hsa−mir−372
P =0.8
DSS hsa−mir−372
P =0.42
survival probability
Time in days

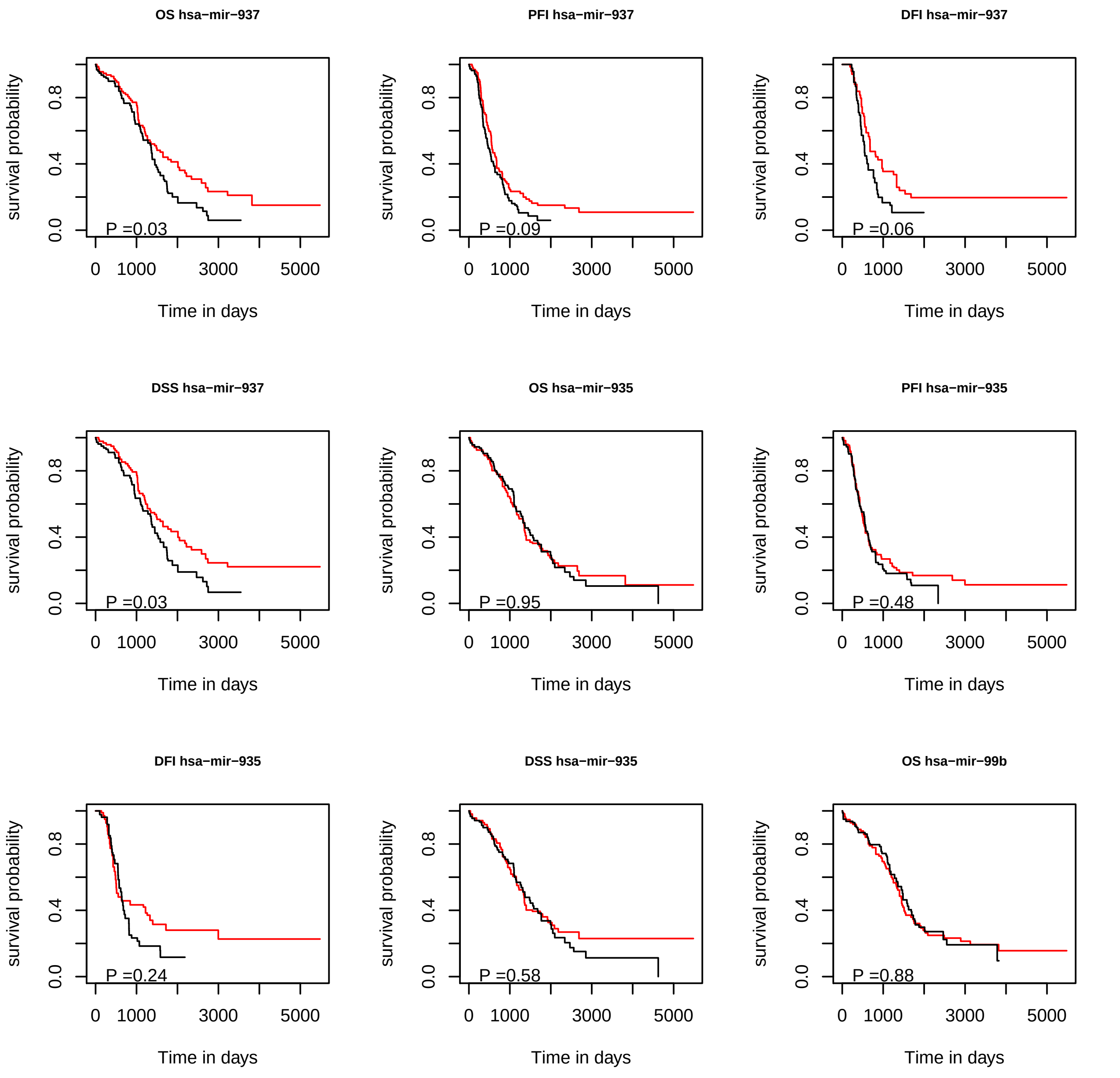
OS hsa−mir−937
survival probability
P =0.03
Time in days
PFI hsa−mir−937
survival probability
P =0.09
Time in days
DFI hsa−mir−937
survival probability
P =0.06
Time in days
DSS hsa−mir−937
survival probability
P =0.03
Time in days
OS hsa−mir−935
survival probability
P =0.95
Time in days
PFI hsa−mir−935
survival probability
P =0.48
Time in days
DFI hsa−mir−935
survival probability
P =0.24
Time in days
DSS hsa−mir−935
survival probability
P =0.58
Time in days
OS hsa−mir−99b
survival probability
P =0.88
Time in days

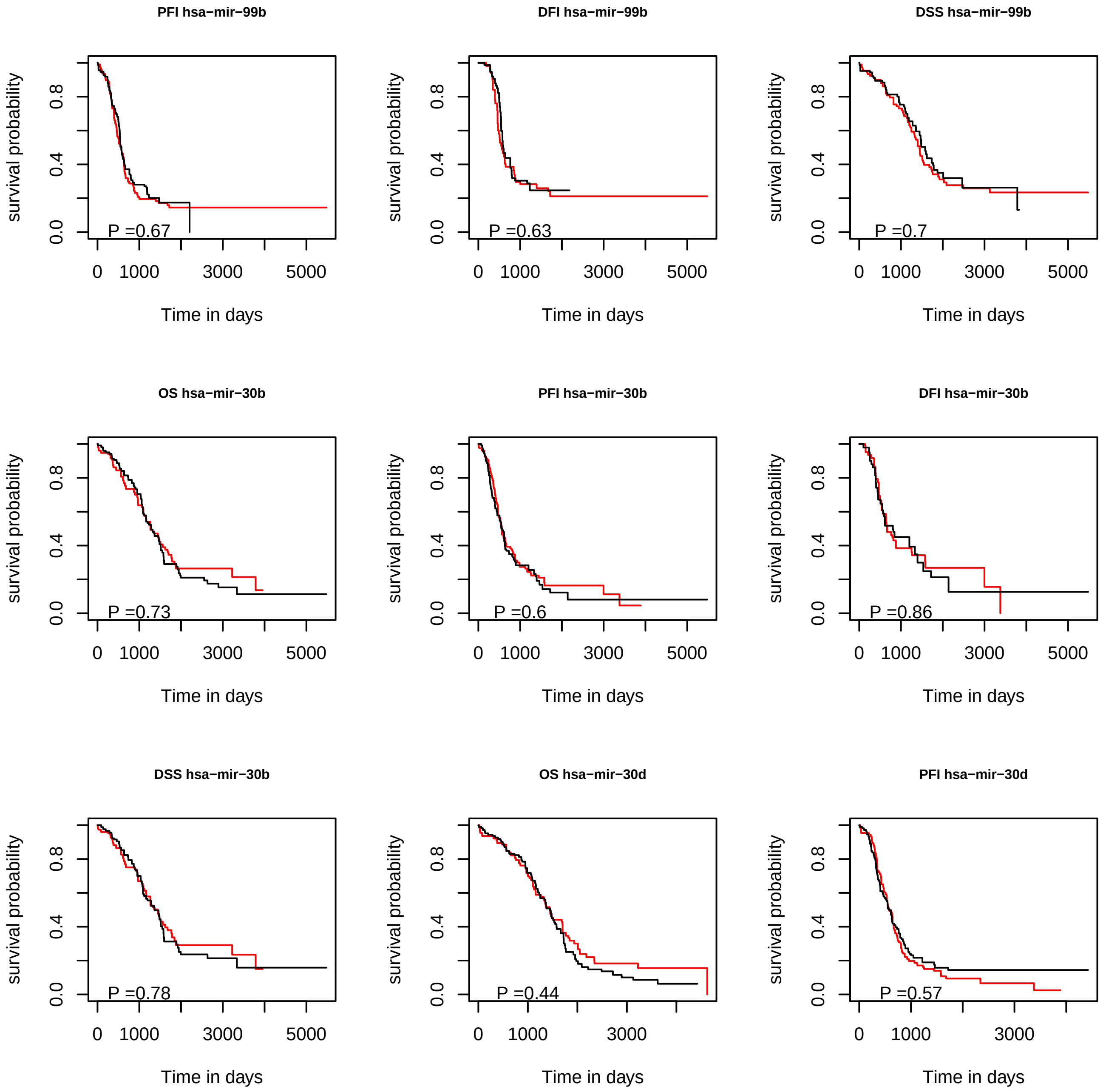
PFI hsa−mir−99b
DFI hsa−mir−99b
DSS hsa−mir−99b
P =0.67
P =0.63
P =0.7
OS hsa−mir−30b
PFI hsa−mir−30b
DFI hsa−mir−30b
P =0.73
P =0.6
P =0.86
DSS hsa−mir−30b
OS hsa−mir−30d
PFI hsa−mir−30d
P =0.78
P =0.44
P =0.57
survival probability
Time in days
0.0
0.4
0.8
0
1000
3000
5000

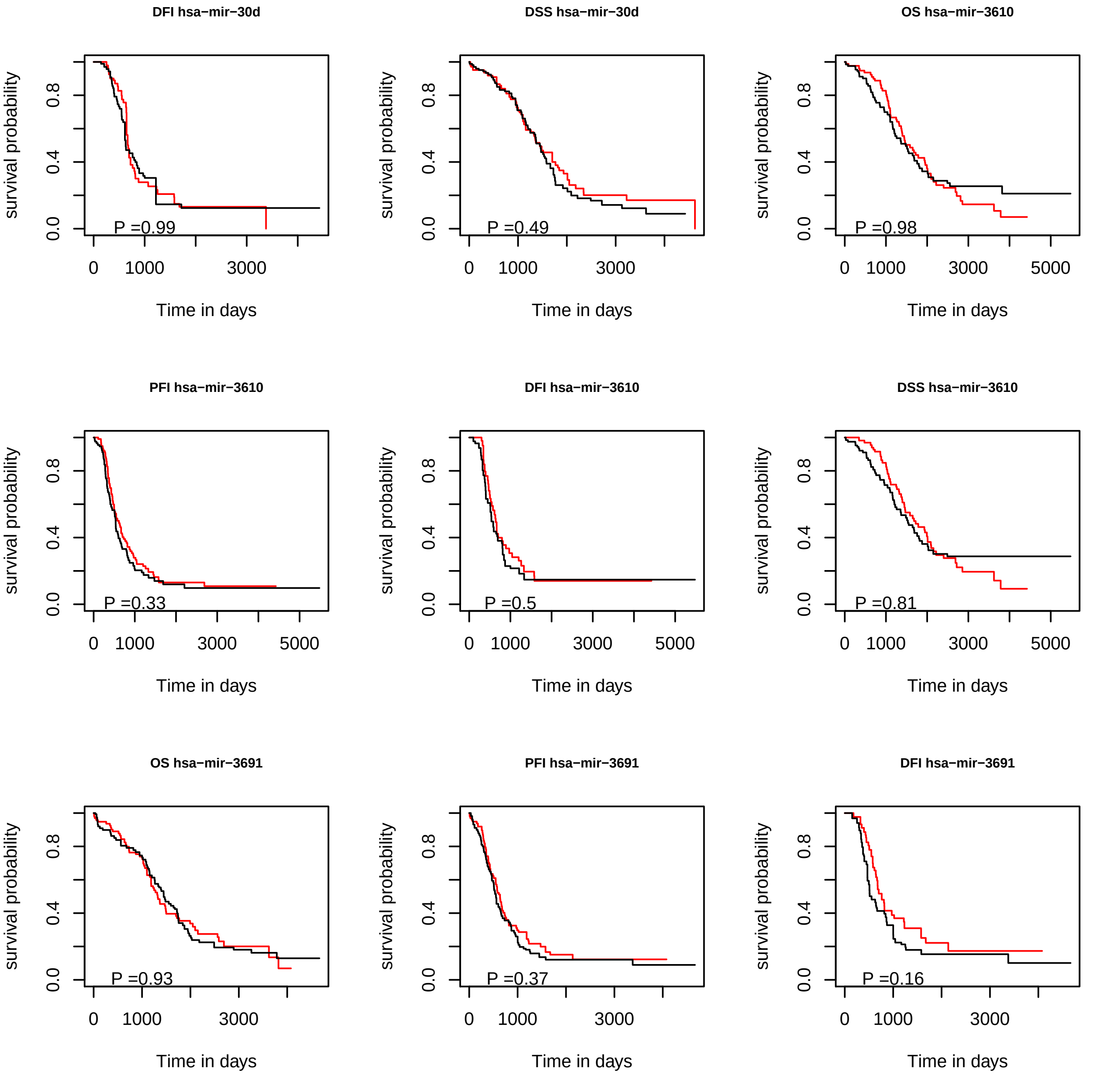
DFI hsa−mir−30d
survival probability
0.0
0.4
0.8
P =0.99
0
1000
3000
Time in days
DSS hsa−mir−30d
survival probability
0.0
0.4
0.8
P =0.49
0
1000
3000
Time in days
OS hsa−mir−3610
survival probability
0.0
0.4
0.8
P =0.98
0
1000
3000
5000
Time in days
PFI hsa−mir−3610
survival probability
0.0
0.4
0.8
P =0.33
0
1000
3000
5000
Time in days
DFI hsa−mir−3610
survival probability
0.0
0.4
0.8
P =0.5
0
1000
3000
5000
Time in days
DSS hsa−mir−3610
survival probability
0.0
0.4
0.8
P =0.81
0
1000
3000
5000
Time in days
OS hsa−mir−3691
survival probability
0.0
0.4
0.8
P =0.93
0
1000
3000
Time in days
PFI hsa−mir−3691
survival probability
0.0
0.4
0.8
P =0.37
0
1000
3000
Time in days
DFI hsa−mir−3691
survival probability
0.0
0.4
0.8
P =0.16
0
1000
3000
Time in days

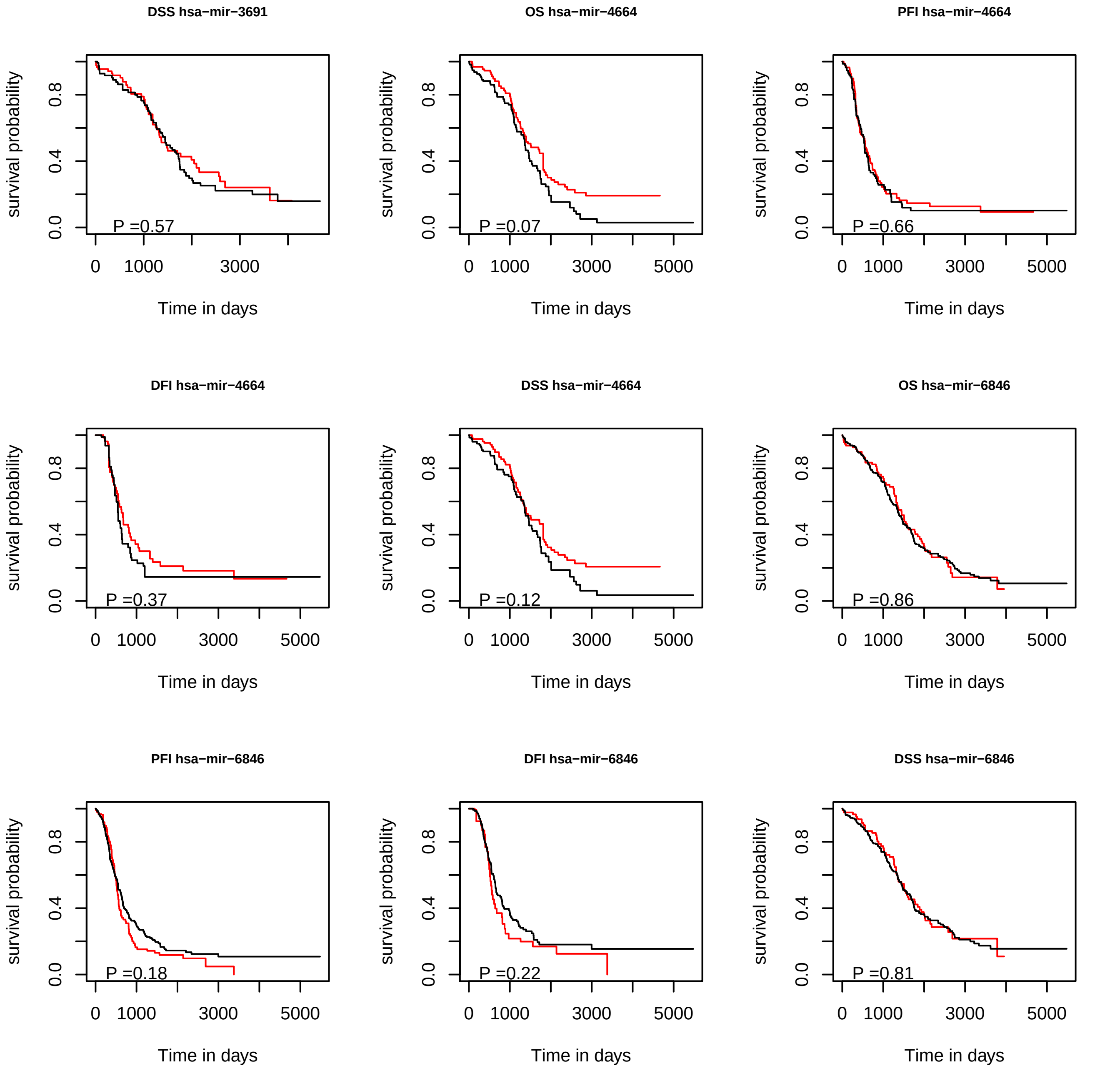
DSS hsa−mir−3691
P =0.57
OS hsa−mir−4664
P =0.07
PFI hsa−mir−4664
P =0.66
DFI hsa−mir−4664
P =0.37
DSS hsa−mir−4664
P =0.12
OS hsa−mir−6846
P =0.86
PFI hsa−mir−6846
P =0.18
DFI hsa−mir−6846
P =0.22
DSS hsa−mir−6846
P =0.81
survival probability
Time in days

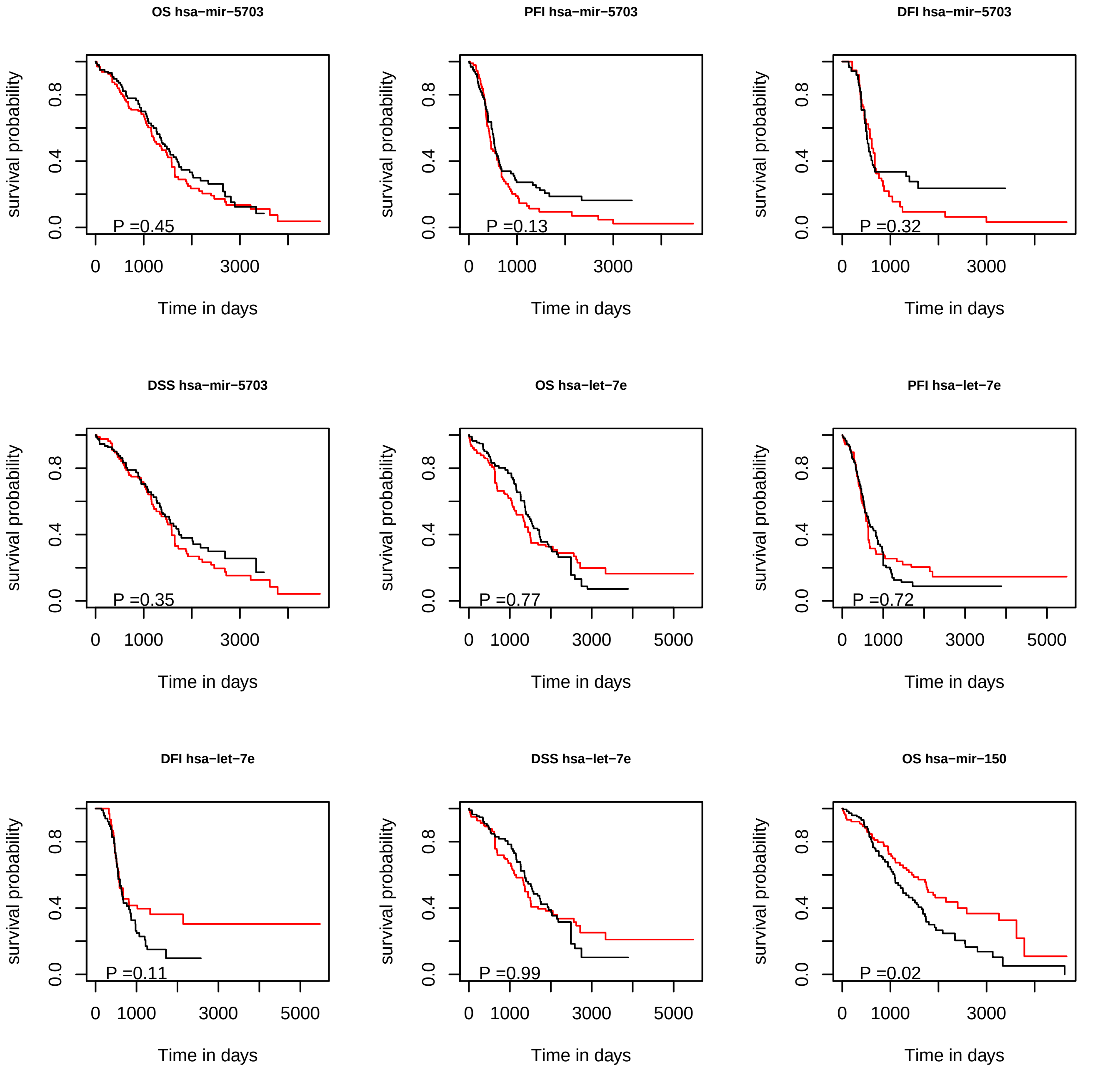
OS hsa−mir−5703
P =0.45
PFI hsa−mir−5703
P =0.13
DFI hsa−mir−5703
P =0.32
DSS hsa−mir−5703
P =0.35
OS hsa−let−7e
P =0.77
PFI hsa−let−7e
P =0.72
DFI hsa−let−7e
P =0.11
DSS hsa−let−7e
P =0.99
OS hsa−mir−150
P =0.02
survival probability
Time in days
0.0
0.4
0.8
0
1000
3000
5000

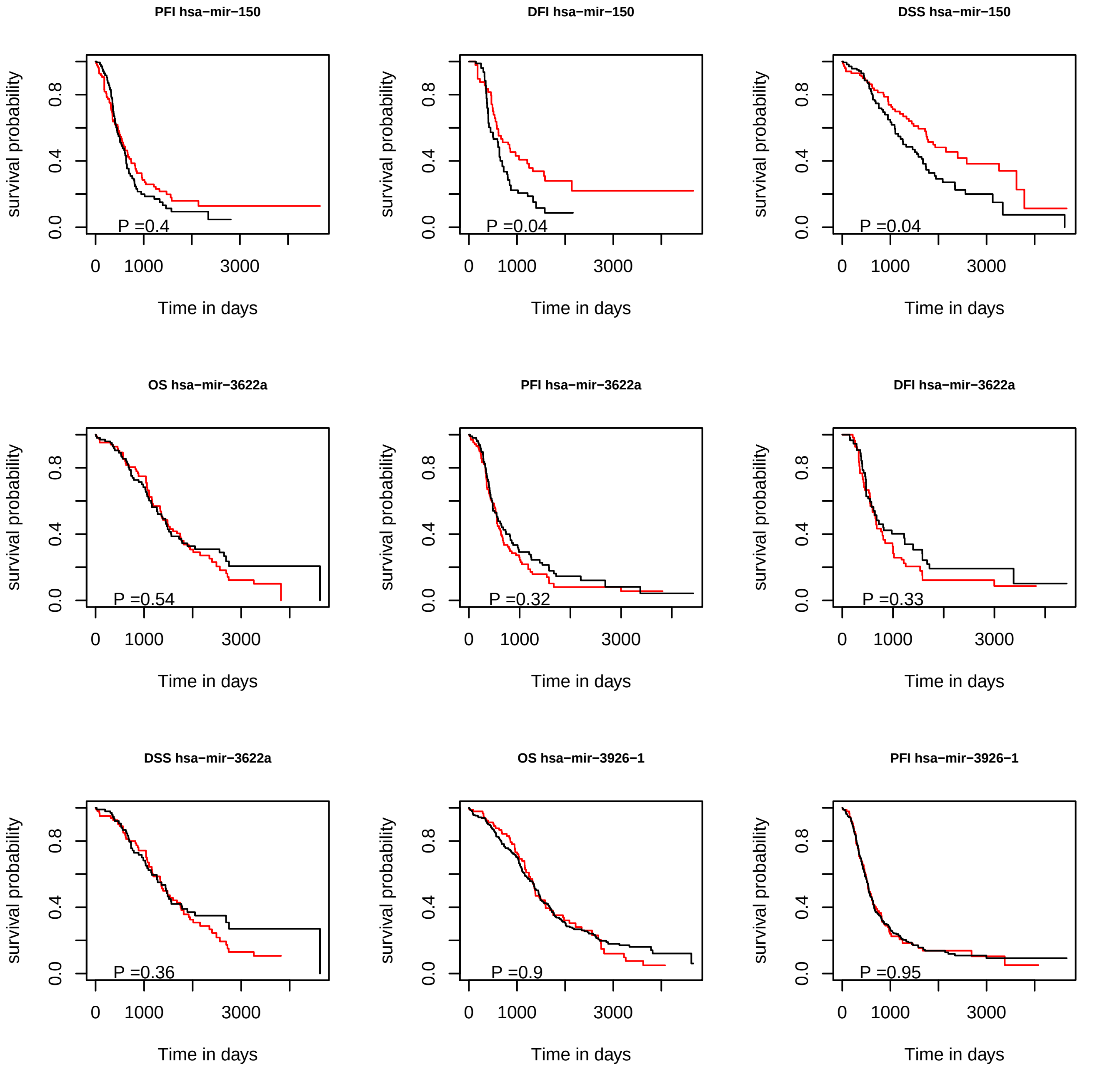
PFI hsa−mir−150
survival probability
0.0
0.4
0.8
P =0.4
0
1000
3000
Time in days
DFI hsa−mir−150
survival probability
0.0
0.4
0.8
P =0.04
0
1000
3000
Time in days
DSS hsa−mir−150
survival probability
0.0
0.4
0.8
P =0.04
0
1000
3000
Time in days
OS hsa−mir−3622a
survival probability
0.0
0.4
0.8
P =0.54
0
1000
3000
Time in days
PFI hsa−mir−3622a
survival probability
0.0
0.4
0.8
P =0.32
0
1000
3000
Time in days
DFI hsa−mir−3622a
survival probability
0.0
0.4
0.8
P =0.33
0
1000
3000
Time in days
DSS hsa−mir−3622a
survival probability
0.0
0.4
0.8
P =0.36
0
1000
3000
Time in days
OS hsa−mir−3926−1
survival probability
0.0
0.4
0.8
P =0.9
0
1000
3000
Time in days
PFI hsa−mir−3926−1
survival probability
0.0
0.4
0.8
P =0.95
0
1000
3000
Time in days

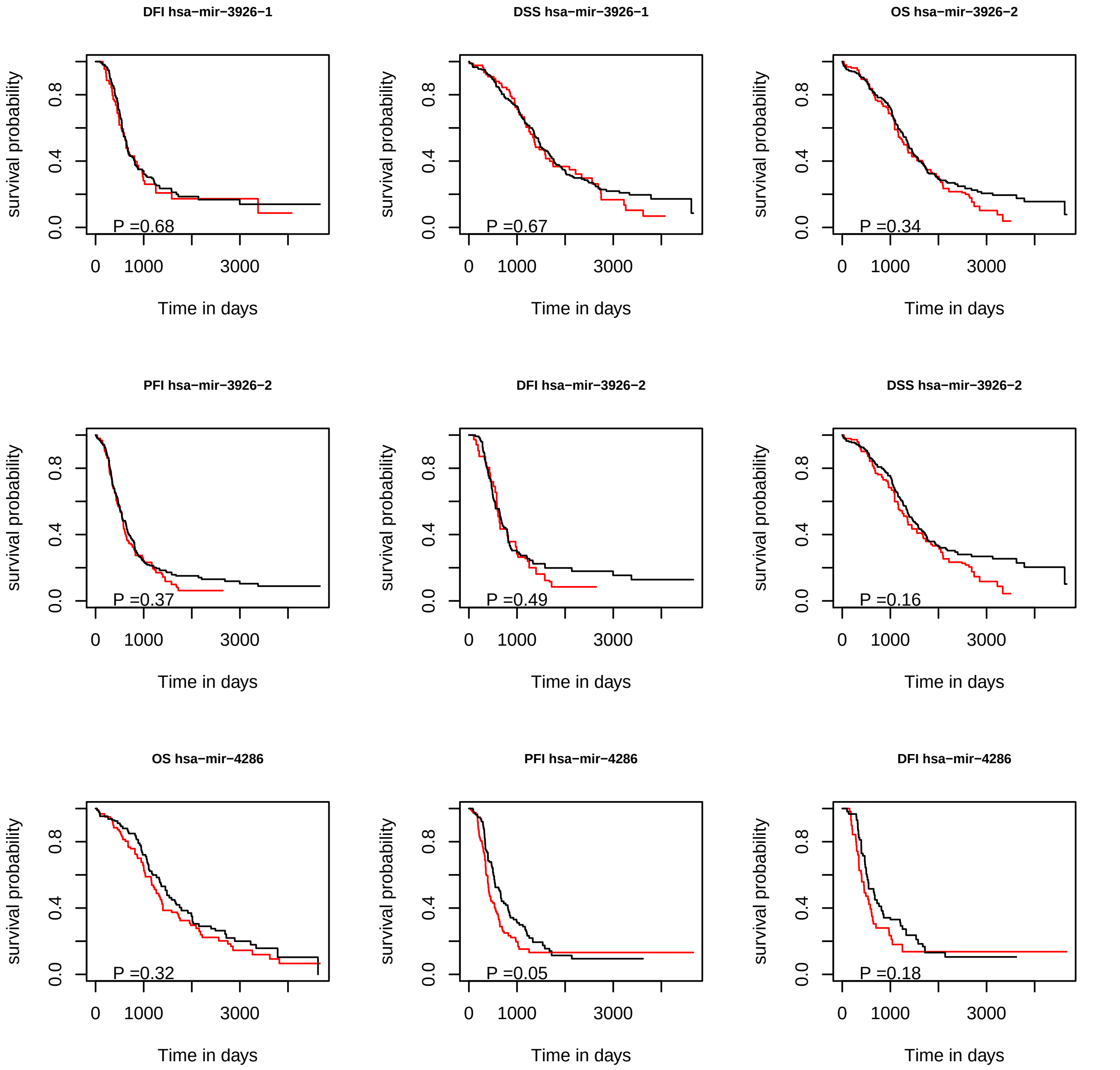

DFI hsa−mir−3926−1
P =0.68
DSS hsa−mir−3926−1
P =0.67
OS hsa−mir−3926−2
P =0.34
PFI hsa−mir−3926−2
P =0.37
DFI hsa−mir−3926−2
P =0.49
DSS hsa−mir−3926−2
P =0.16
OS hsa−mir−4286
P =0.32
PFI hsa−mir−4286
P =0.05
DFI hsa−mir−4286
P =0.18
survival probability
Time in days
0.0
0.4
0.8
0
1000
3000

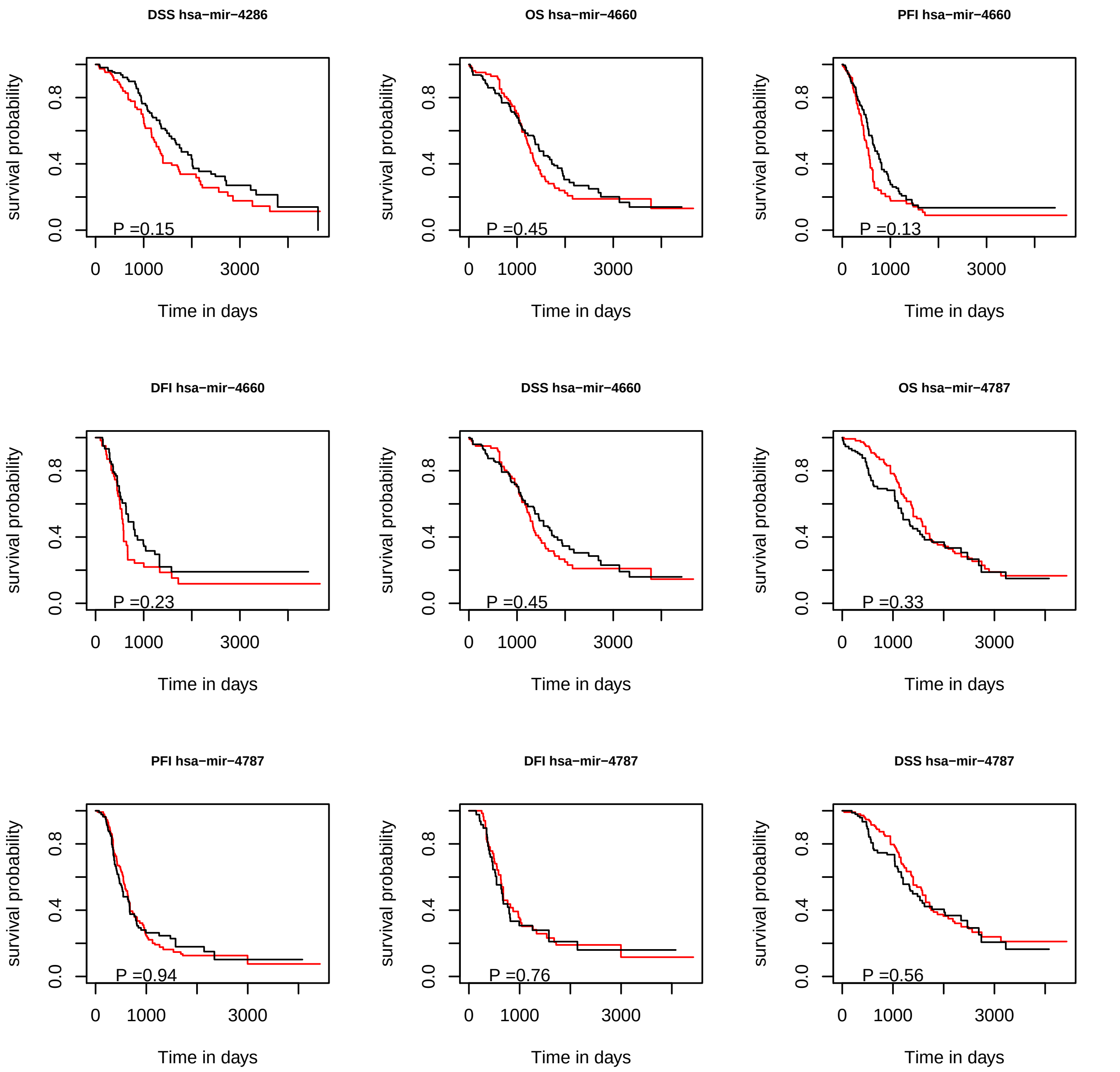
DSS hsa−mir−4286
P =0.15
OS hsa−mir−4660
P =0.45
PFI hsa−mir−4660
P =0.13
DFI hsa−mir−4660
P =0.23
DSS hsa−mir−4660
P =0.45
OS hsa−mir−4787
P =0.33
PFI hsa−mir−4787
P =0.94
DFI hsa−mir−4787
P =0.76
DSS hsa−mir−4787
P =0.56
survival probability
0.0
0.4
0.8
Time in days
0
1000
3000

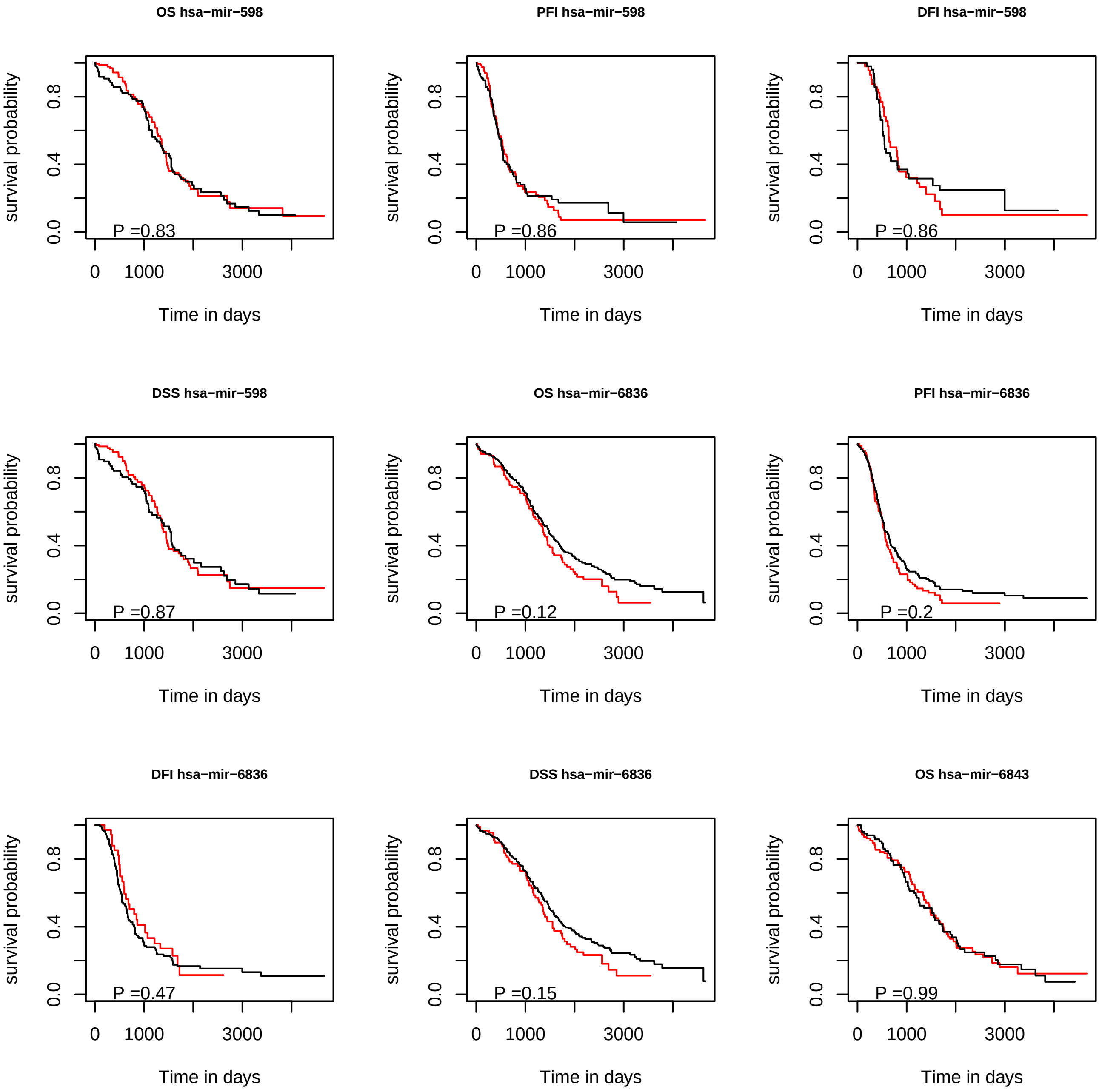

OS hsa−mir−598
P =0.83
PFI hsa−mir−598
P =0.86
DFI hsa−mir−598
P =0.86
DSS hsa−mir−598
P =0.87
OS hsa−mir−6836
P =0.12
PFI hsa−mir−6836
P =0.2
DFI hsa−mir−6836
P =0.47
DSS hsa−mir−6836
P =0.15
OS hsa−mir−6843
P =0.99
survival probability
Time in days
0.0
0.4
0.8
0
1000
3000

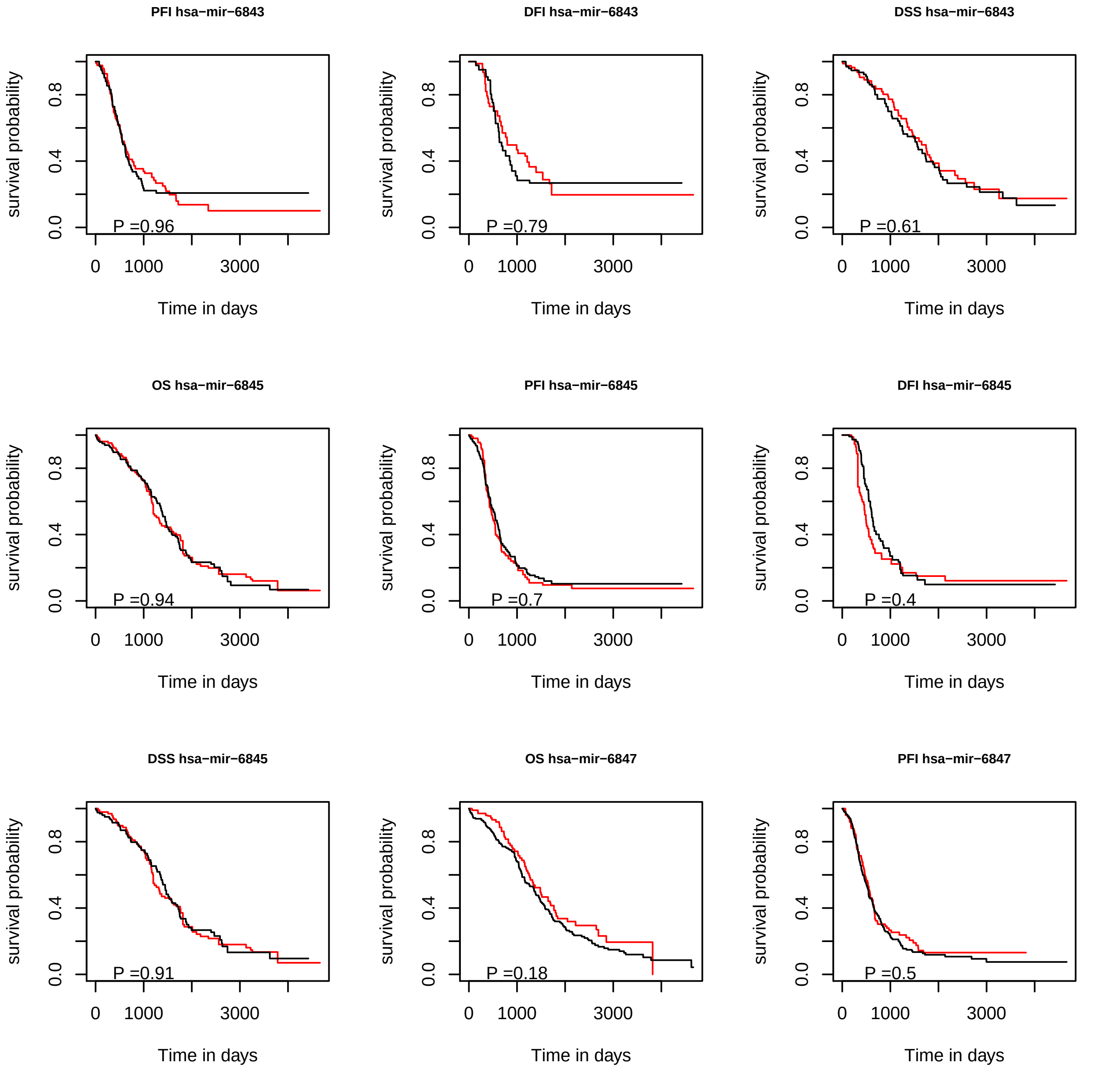
PFI hsa−mir−6843
P =0.96
DFI hsa−mir−6843
P =0.79
DSS hsa−mir−6843
P =0.61
OS hsa−mir−6845
P =0.94
PFI hsa−mir−6845
P =0.7
DFI hsa−mir−6845
P =0.4
DSS hsa−mir−6845
P =0.91
OS hsa−mir−6847
P =0.18
PFI hsa−mir−6847
P =0.5
survival probability
Time in days
0.0
0.4
0.8
0
1000
3000

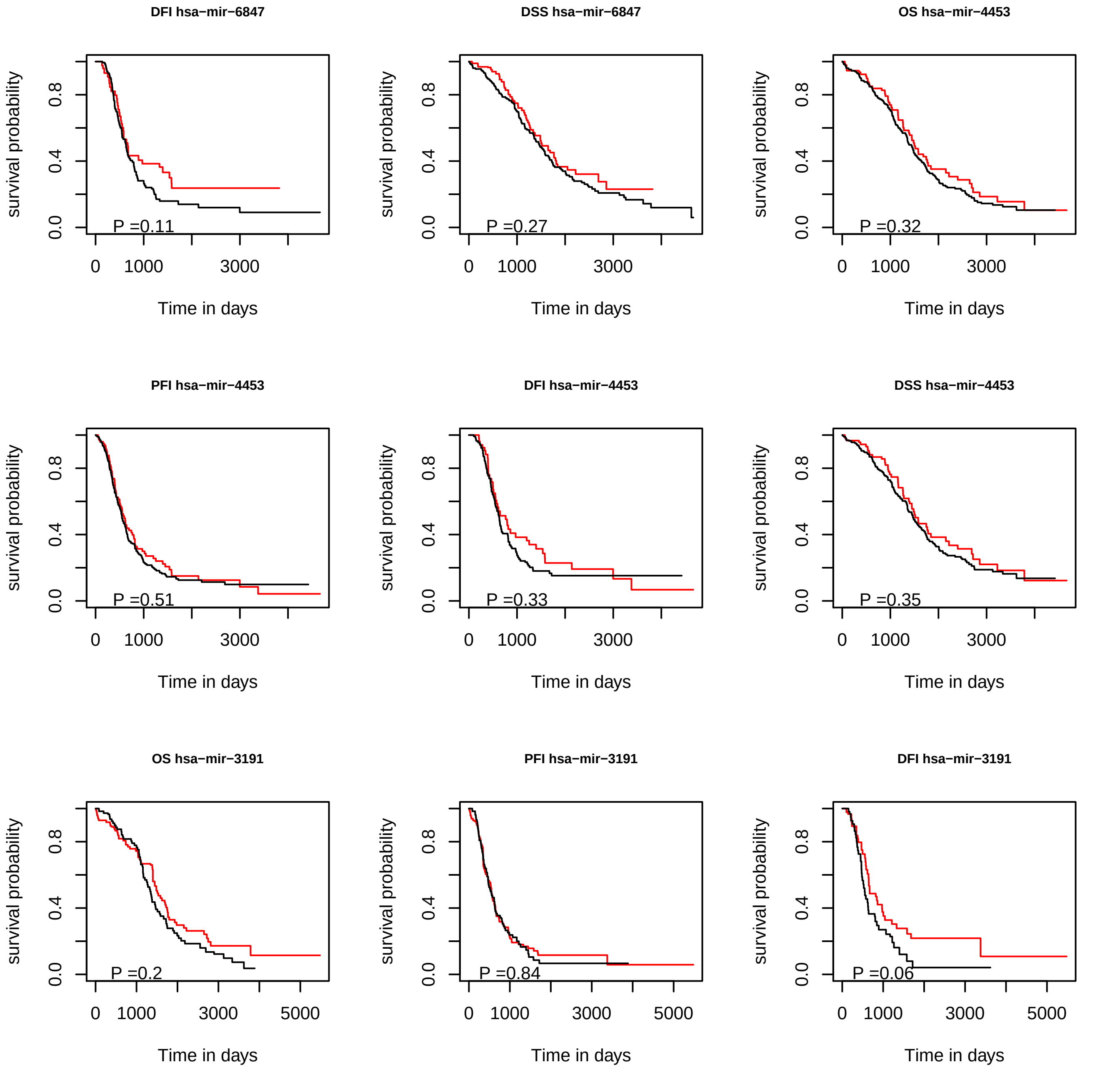
DFI hsa−mir−6847
P =0.11
DSS hsa−mir−6847
P =0.27
OS hsa−mir−4453
P =0.32
PFI hsa−mir−4453
P =0.51
DFI hsa−mir−4453
P =0.33
DSS hsa−mir−4453
P =0.35
OS hsa−mir−3191
P =0.2
PFI hsa−mir−3191
P =0.84
DFI hsa−mir−3191
P =0.06
survival probability
0.0
0.4
0.8
Time in days
0
1000
3000
5000

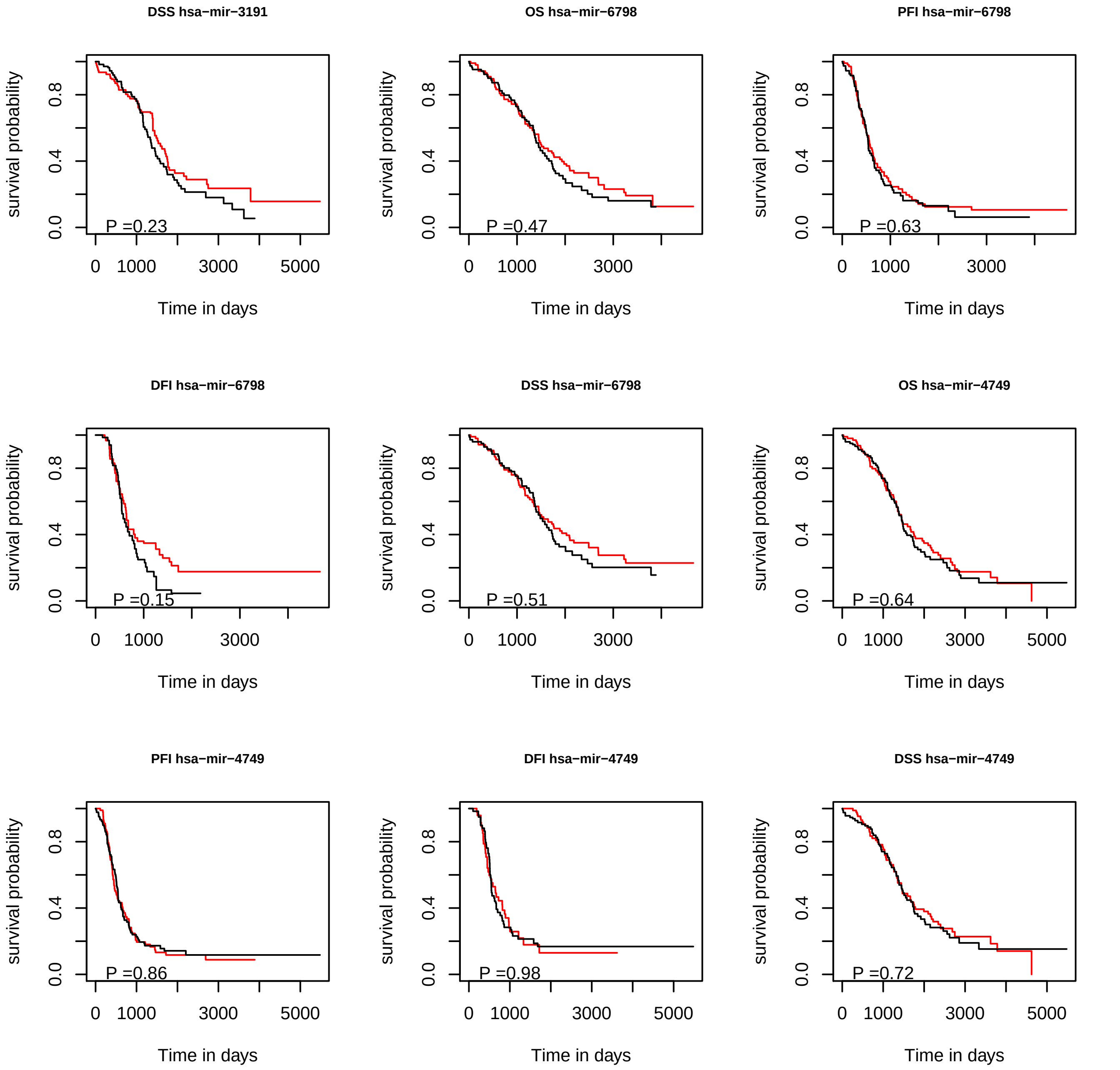
DSS hsa−mir−3191
P =0.23
OS hsa−mir−6798
P =0.47
PFI hsa−mir−6798
P =0.63
DFI hsa−mir−6798
P =0.15
DSS hsa−mir−6798
P =0.51
OS hsa−mir−4749
P =0.64
PFI hsa−mir−4749
P =0.86
DFI hsa−mir−4749
P =0.98
DSS hsa−mir−4749
P =0.72
survival probability
Time in days
0.0
0.4
0.8
0
1000
3000
5000

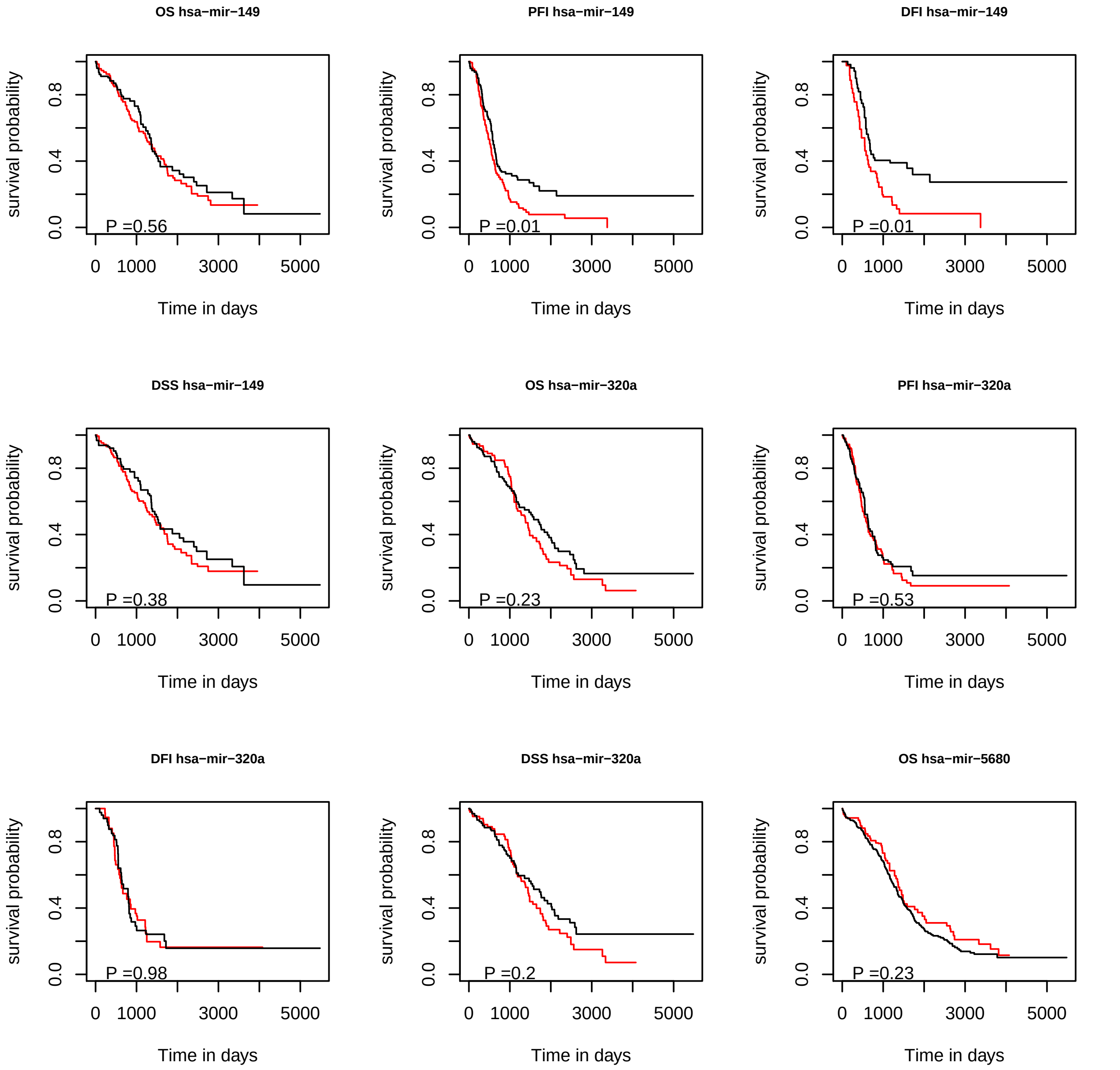
OS hsa−mir−149
P =0.56
PFI hsa−mir−149
P =0.01
DFI hsa−mir−149
P =0.01
DSS hsa−mir−149
P =0.38
OS hsa−mir−320a
P =0.23
PFI hsa−mir−320a
P =0.53
DFI hsa−mir−320a
P =0.98
DSS hsa−mir−320a
P =0.2
OS hsa−mir−5680
P =0.23
survival probability
0.0
0.4
0.8
0
1000
3000
5000
Time in days

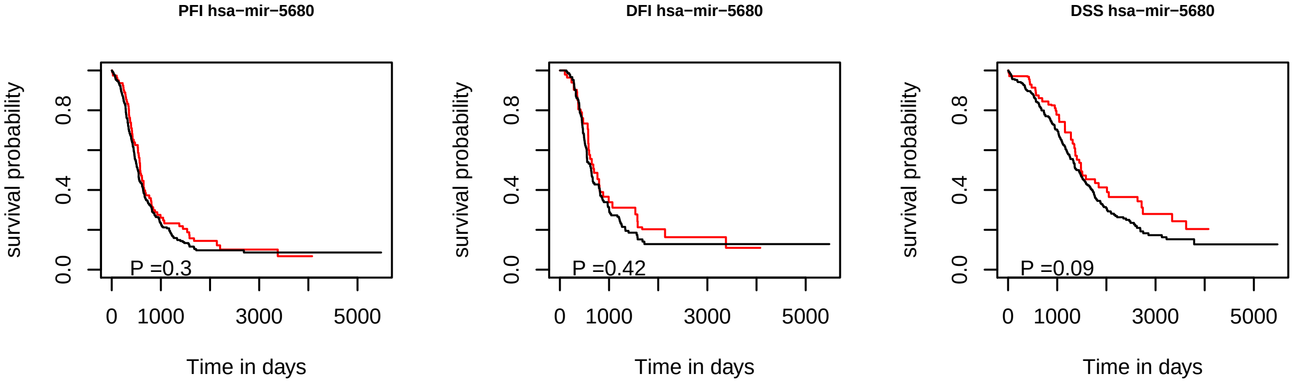

PFI hsa−mir−5680
survival probability
0.0
0.4
0.8
P =0.3
0
1000
3000
5000
Time in days
DFI hsa−mir−5680
survival probability
0.0
0.4
0.8
P =0.42
0
1000
3000
5000
Time in days
DSS hsa−mir−5680
survival probability
0.0
0.4
0.8
P =0.09
0
1000
3000
5000
Time in days
