# Supplementary material for: Computing microRNA-gene interaction networks in pan-cancer using miRDriver
Source: Sci Rep. 2022 Mar 8;12:3717. doi: 10.1038/s41598-022-07628-z (PMC8904490; doi:10.1038/s41598-022-07628-z)
Supplement: Supplementary file 24 — Supplementary Information 24. [file 41598_2022_7628_MOESM24_ESM.pdf]

Computing microRNA-gene interaction networks in pan-cancer using miRDriver

Banabithi Bose, Matthew Moravec, and Serdar Bozdag

Supplemental Figure S15

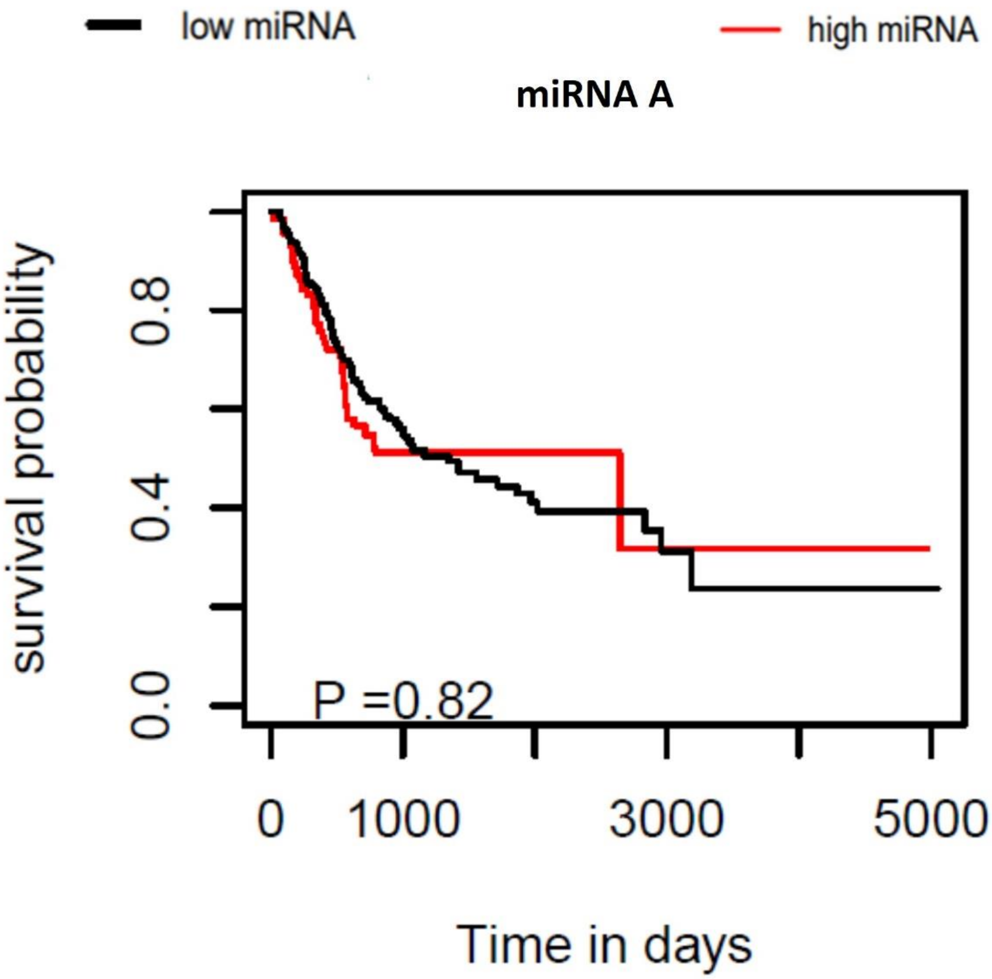

The *Adjusted Kaplan-Meier* survival plots for the computed miRNAs in high and low miRNA expression patient groups.

Supplemental Figure S15 Cancer Type: PAAD

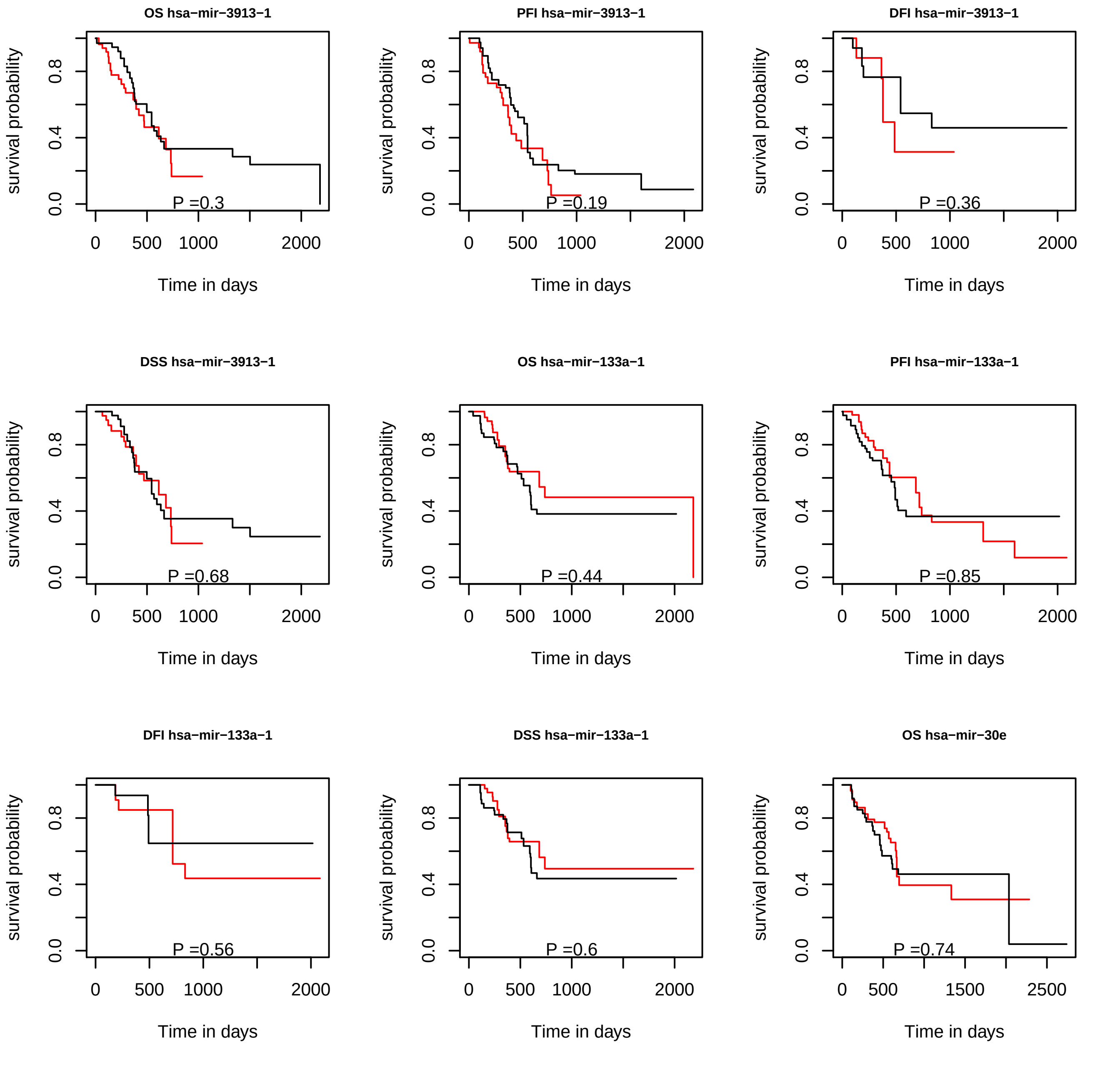

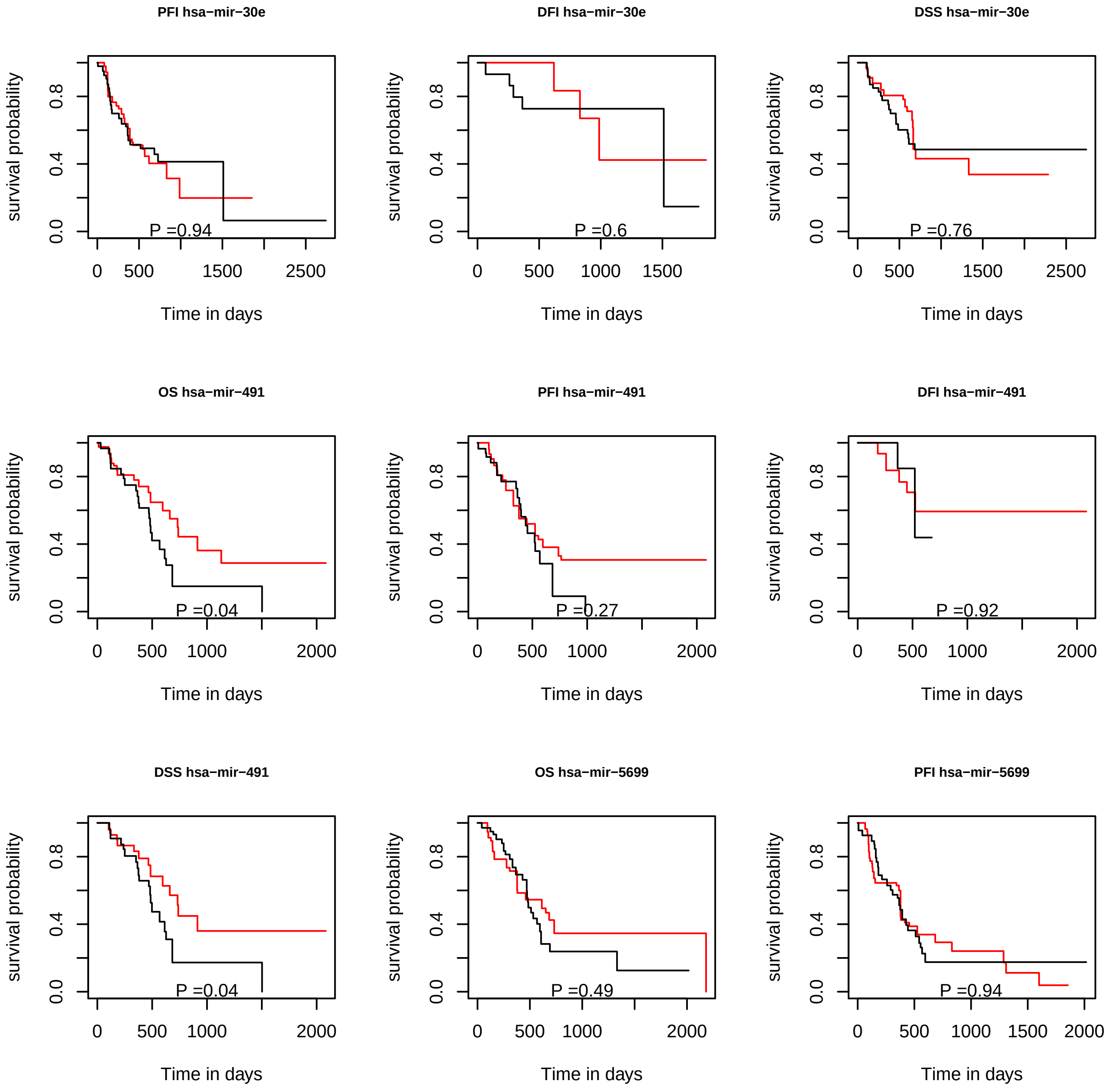
PFI hsa−mir−30e
P =0.94
DFI hsa−mir−30e
P =0.6
DSS hsa−mir−30e
P =0.76
OS hsa−mir−491
P =0.04
PFI hsa−mir−491
P =0.27
DFI hsa−mir−491
P =0.92
DSS hsa−mir−491
P =0.04
OS hsa−mir−5699
P =0.49
PFI hsa−mir−5699
P =0.94
survival probability
Time in days

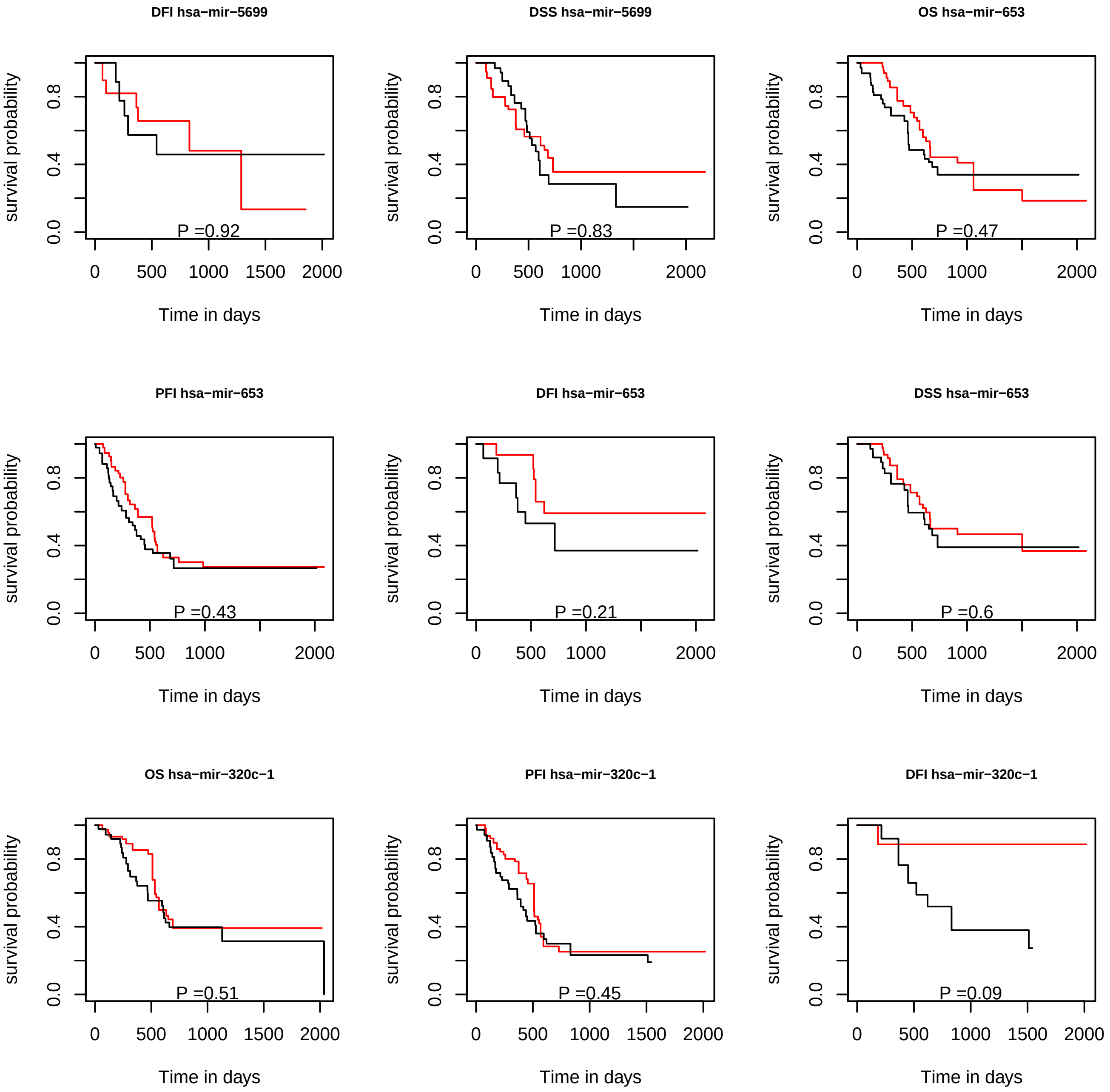
DFI hsa−mir−5699
survival probability
0.0 0.4 0.8
P =0.92
0 500 1000 1500 2000
Time in days
DSS hsa−mir−5699
survival probability
0.0 0.4 0.8
P =0.83
0 500 1000 2000
Time in days
OS hsa−mir−653
survival probability
0.0 0.4 0.8
P =0.47
0 500 1000 2000
Time in days
PFI hsa−mir−653
survival probability
0.0 0.4 0.8
P =0.43
0 500 1000 2000
Time in days
DFI hsa−mir−653
survival probability
0.0 0.4 0.8
P =0.21
0 500 1000 2000
Time in days
DSS hsa−mir−653
survival probability
0.0 0.4 0.8
P =0.6
0 500 1000 2000
Time in days
OS hsa−mir−320c−1
survival probability
0.0 0.4 0.8
P =0.51
0 500 1000 1500 2000
Time in days
PFI hsa−mir−320c−1
survival probability
0.0 0.4 0.8
P =0.45
0 500 1000 1500 2000
Time in days
DFI hsa−mir−320c−1
survival probability
0.0 0.4 0.8
P =0.09
0 500 1000 1500 2000
Time in days

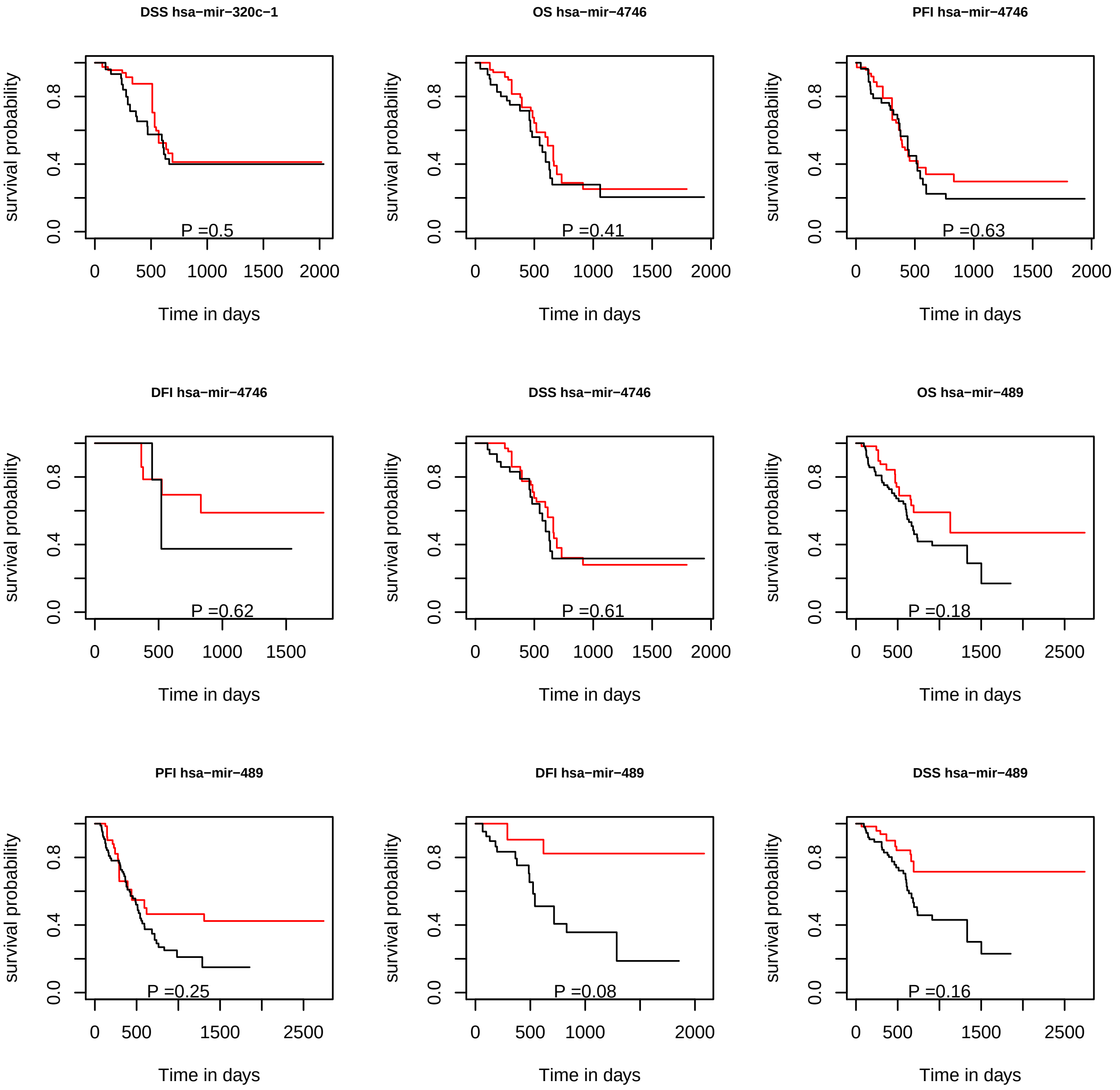
DSS hsa−mir−320c−1
survival probability
0.0
0.4
0.8
P =0.5
0
500
1000
1500
2000
Time in days
OS hsa−mir−4746
survival probability
0.0
0.4
0.8
P =0.41
0
500
1000
1500
2000
Time in days
PFI hsa−mir−4746
survival probability
0.0
0.4
0.8
P =0.63
0
500
1000
1500
2000
Time in days
DFI hsa−mir−4746
survival probability
0.0
0.4
0.8
P =0.62
0
500
1000
1500
Time in days
DSS hsa−mir−4746
survival probability
0.0
0.4
0.8
P =0.61
0
500
1000
1500
2000
Time in days
OS hsa−mir−489
survival probability
0.0
0.4
0.8
P =0.18
0
500
1500
2500
Time in days
PFI hsa−mir−489
survival probability
0.0
0.4
0.8
P =0.25
0
500
1500
2500
Time in days
DFI hsa−mir−489
survival probability
0.0
0.4
0.8
P =0.08
0
500
1000
2000
Time in days
DSS hsa−mir−489
survival probability
0.0
0.4
0.8
P =0.16
0
500
1500
2500
Time in days

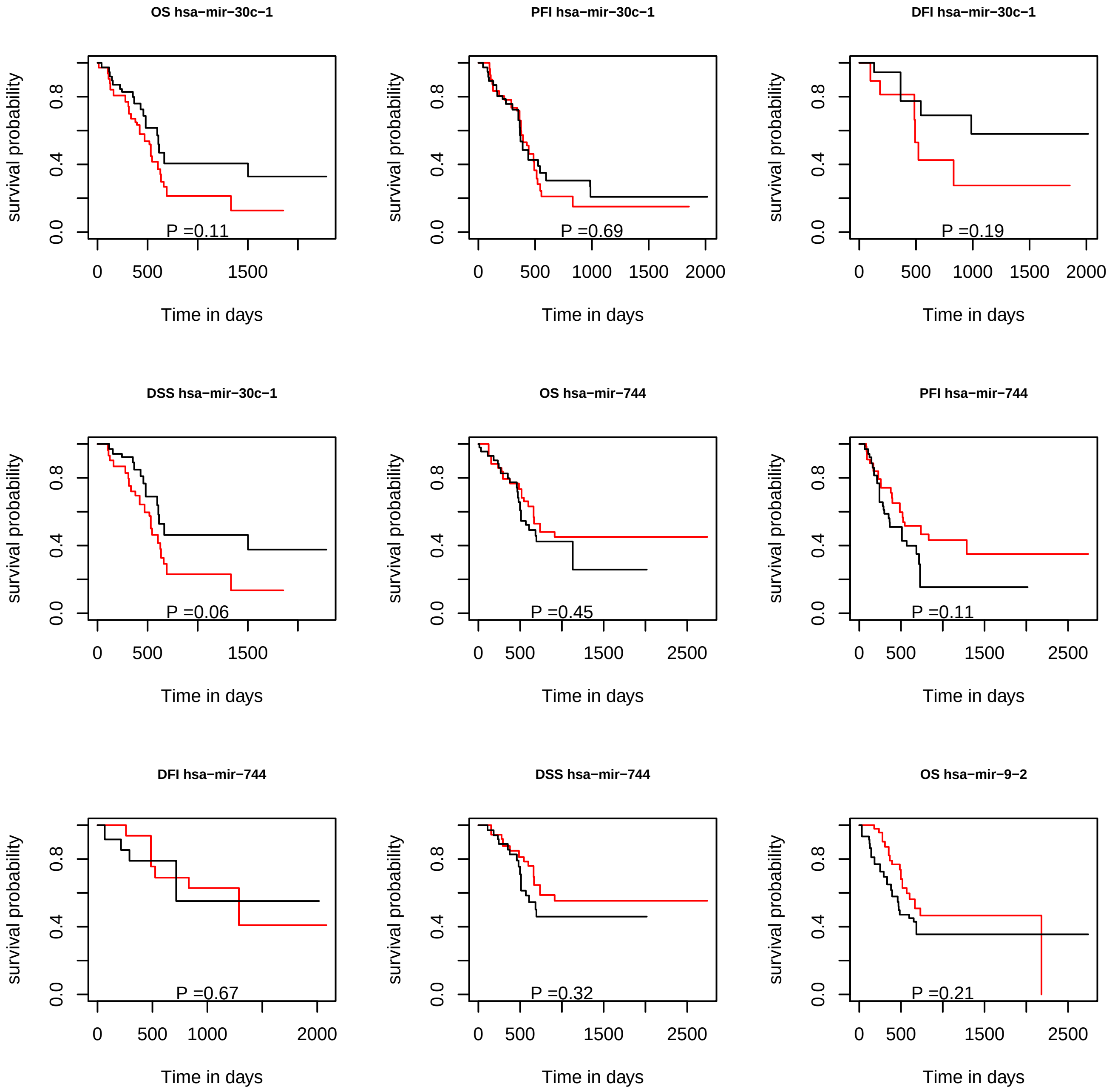
OS hsa−mir−30c−1
P =0.11
PFI hsa−mir−30c−1
P =0.69
DFI hsa−mir−30c−1
P =0.19
DSS hsa−mir−30c−1
P =0.06
OS hsa−mir−744
P =0.45
PFI hsa−mir−744
P =0.11
DFI hsa−mir−744
P =0.67
DSS hsa−mir−744
P =0.32
OS hsa−mir−9−2
P =0.21
survival probability
Time in days
0.0
0.4
0.8
0
500
1000
1500
2000
2500

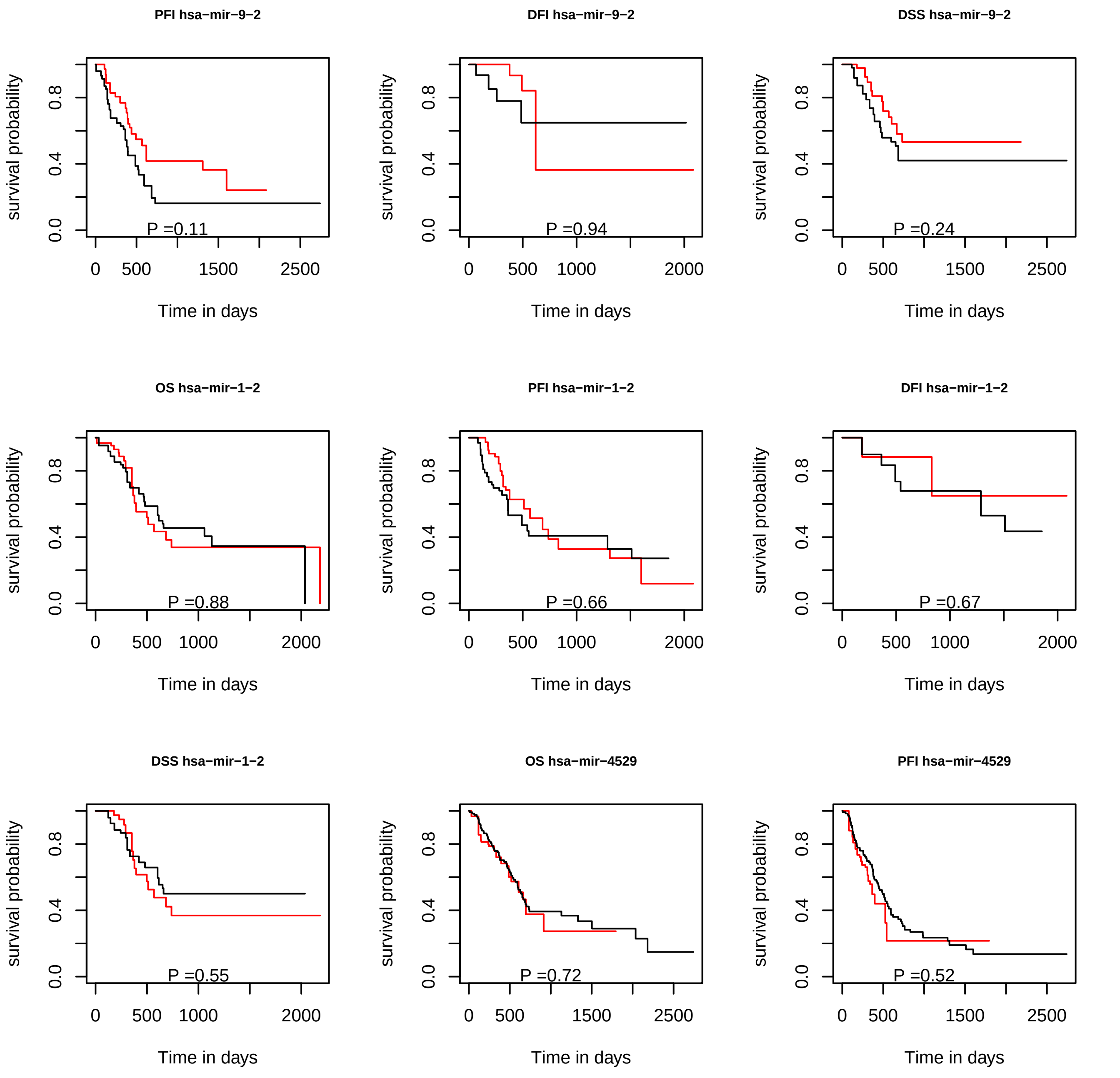
PFI hsa−mir−9−2
survival probability
0.0 0.4 0.8
0 500 1500 2500
Time in days
P =0.11
DFI hsa−mir−9−2
survival probability
0.0 0.4 0.8
0 500 1000 2000
Time in days
P =0.94
DSS hsa−mir−9−2
survival probability
0.0 0.4 0.8
0 500 1500 2500
Time in days
P =0.24
OS hsa−mir−1−2
survival probability
0.0 0.4 0.8
0 500 1000 2000
Time in days
P =0.88
PFI hsa−mir−1−2
survival probability
0.0 0.4 0.8
0 500 1000 2000
Time in days
P =0.66
DFI hsa−mir−1−2
survival probability
0.0 0.4 0.8
0 500 1000 2000
Time in days
P =0.67
DSS hsa−mir−1−2
survival probability
0.0 0.4 0.8
0 500 1000 2000
Time in days
P =0.55
OS hsa−mir−4529
survival probability
0.0 0.4 0.8
0 500 1500 2500
Time in days
P =0.72
PFI hsa−mir−4529
survival probability
0.0 0.4 0.8
0 500 1500 2500
Time in days
P =0.52

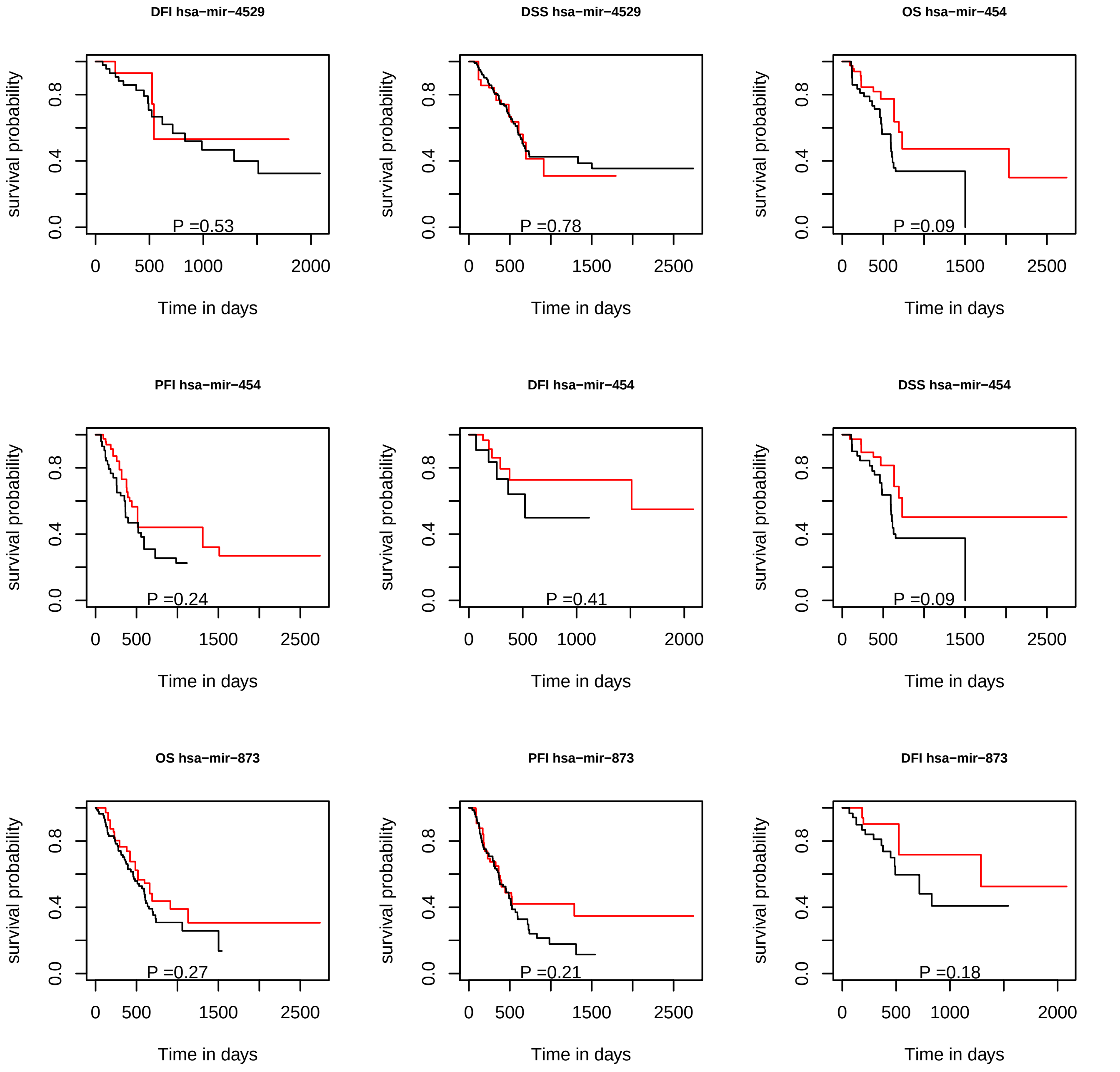

DFI hsa−mir−4529
P =0.53
DSS hsa−mir−4529
P =0.78
OS hsa−mir−454
P =0.09
PFI hsa−mir−454
P =0.24
DFI hsa−mir−454
P =0.41
DSS hsa−mir−454
P =0.09
OS hsa−mir−873
P =0.27
PFI hsa−mir−873
P =0.21
DFI hsa−mir−873
P =0.18
survival probability
Time in days

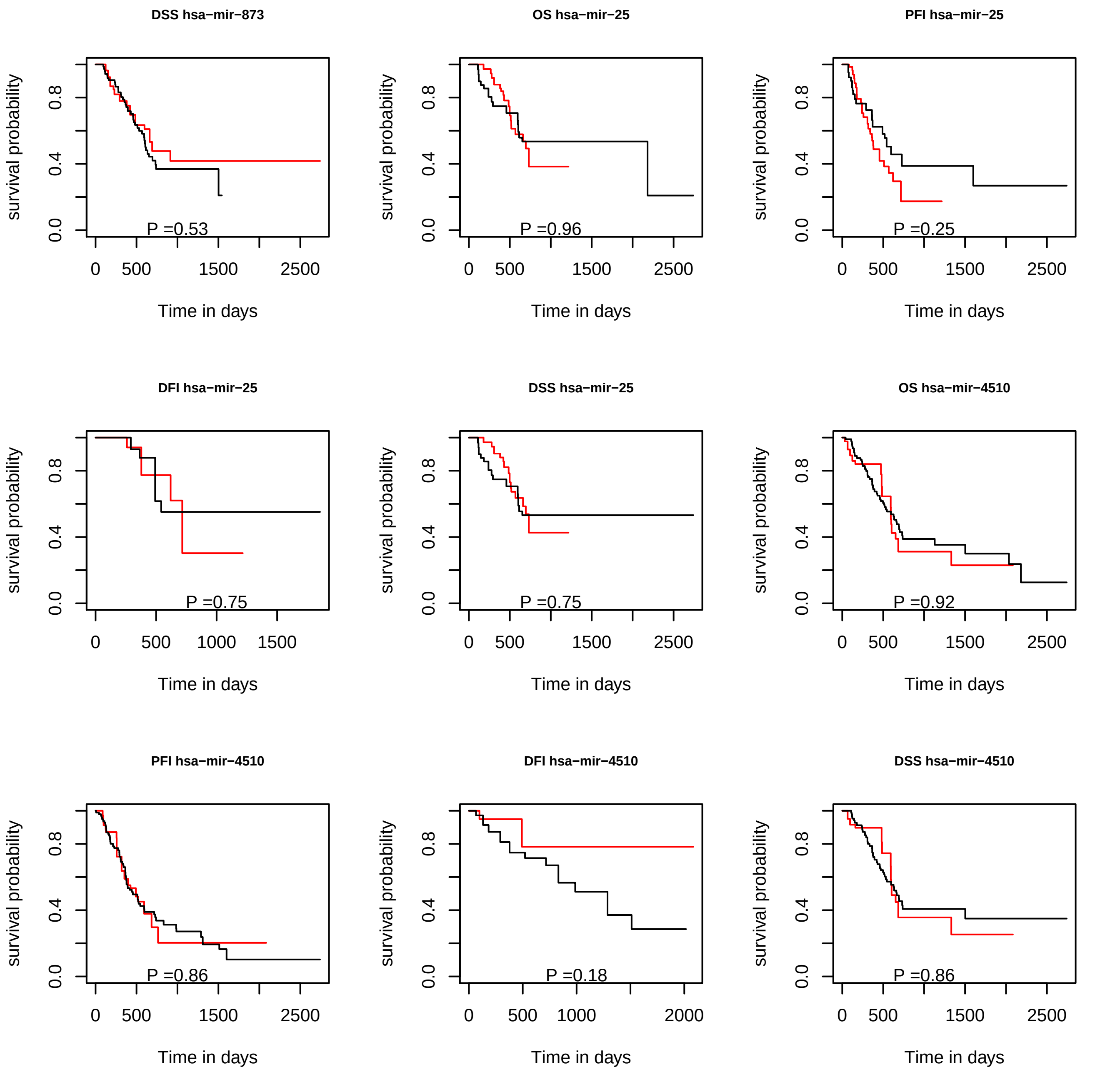
DSS hsa−mir−873
P =0.53
OS hsa−mir−25
P =0.96
PFI hsa−mir−25
P =0.25
DFI hsa−mir−25
P =0.75
DSS hsa−mir−25
P =0.75
OS hsa−mir−4510
P =0.92
PFI hsa−mir−4510
P =0.86
DFI hsa−mir−4510
P =0.18
DSS hsa−mir−4510
P =0.86
survival probability
Time in days

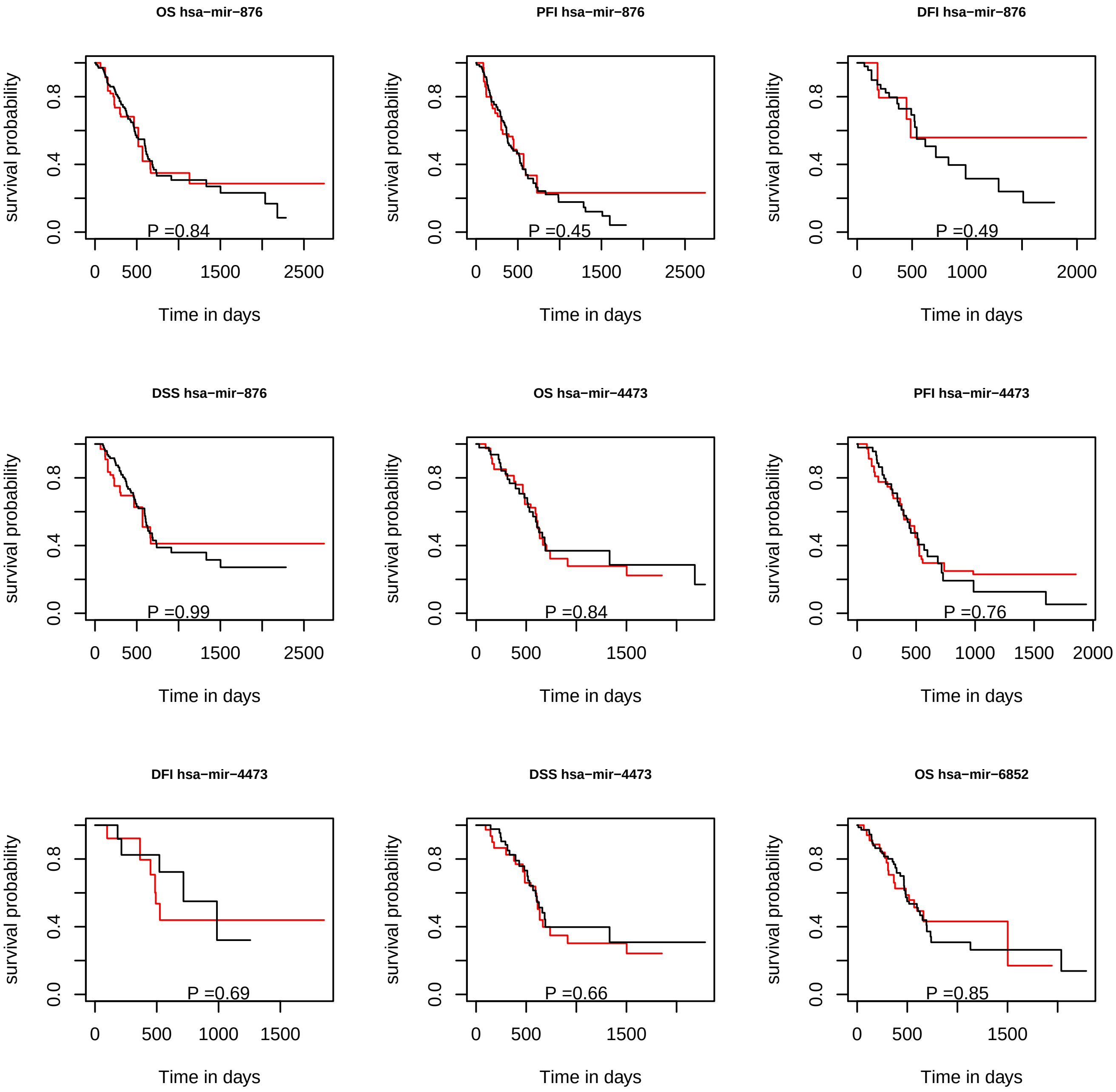
OS hsa−mir−876
P =0.84
PFI hsa−mir−876
P =0.45
DFI hsa−mir−876
P =0.49
DSS hsa−mir−876
P =0.99
OS hsa−mir−4473
P =0.84
PFI hsa−mir−4473
P =0.76
DFI hsa−mir−4473
P =0.69
DSS hsa−mir−4473
P =0.66
OS hsa−mir−6852
P =0.85
survival probability
Time in days

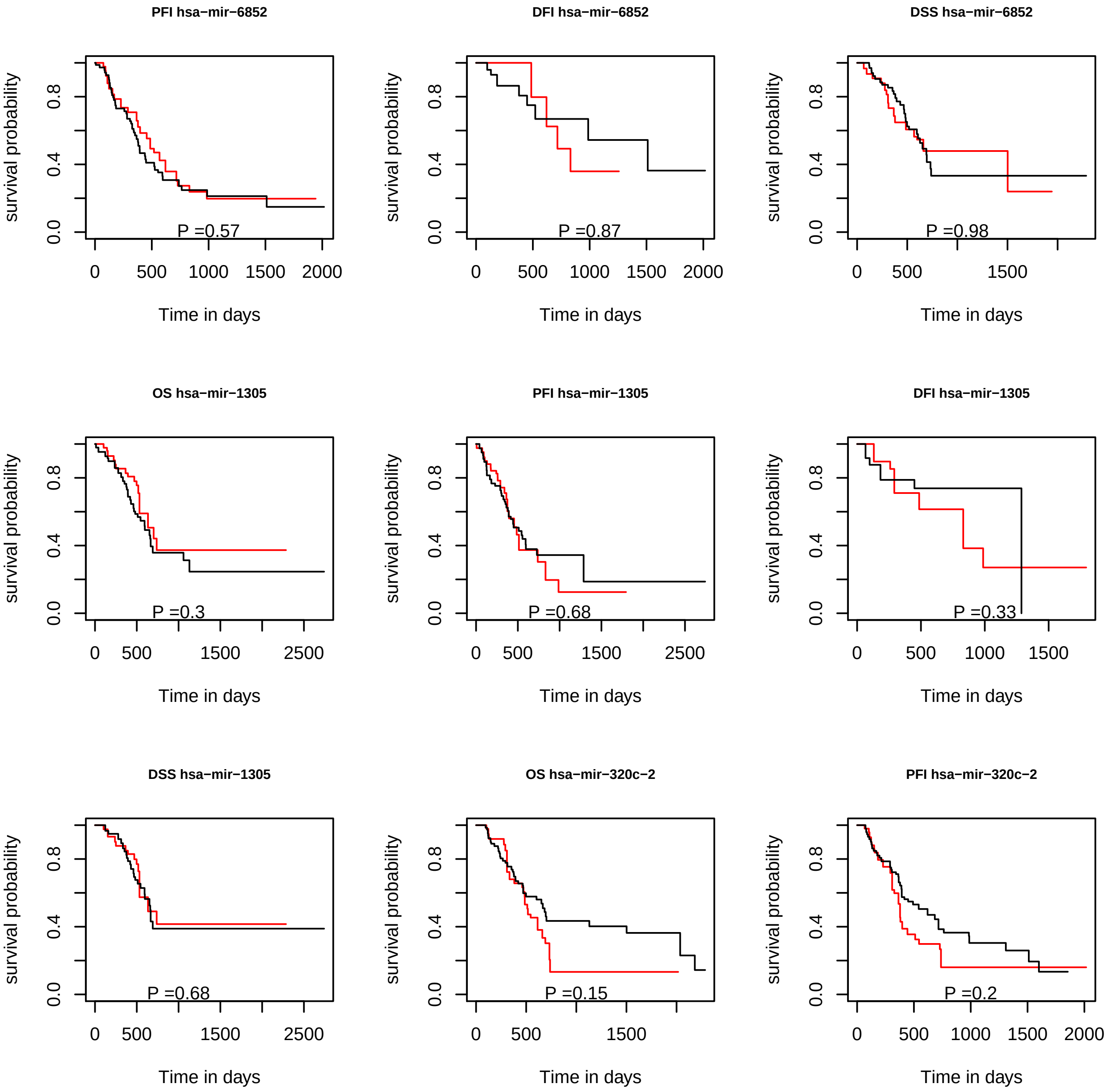
PFI hsa−mir−6852
P =0.57
DFI hsa−mir−6852
P =0.87
DSS hsa−mir−6852
P =0.98
OS hsa−mir−1305
P =0.3
PFI hsa−mir−1305
P =0.68
DFI hsa−mir−1305
P =0.33
DSS hsa−mir−1305
P =0.68
OS hsa−mir−320c−2
P =0.15
PFI hsa−mir−320c−2
P =0.2
survival probability
Time in days

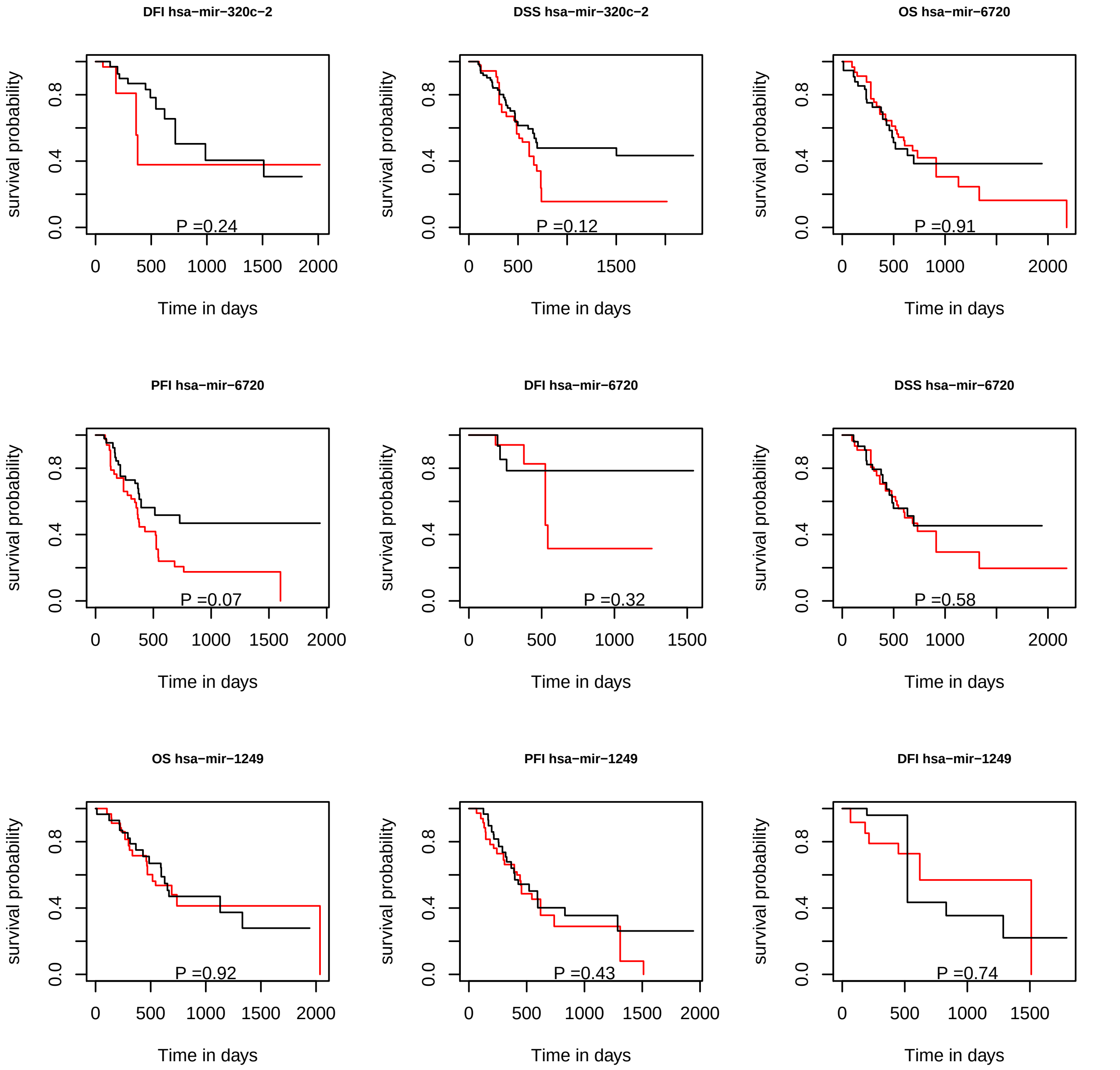

DFI hsa−mir−320c−2
P =0.24
DSS hsa−mir−320c−2
P =0.12
OS hsa−mir−6720
P =0.91
PFI hsa−mir−6720
P =0.07
DFI hsa−mir−6720
P =0.32
DSS hsa−mir−6720
P =0.58
OS hsa−mir−1249
P =0.92
PFI hsa−mir−1249
P =0.43
DFI hsa−mir−1249
P =0.74
survival probability
Time in days

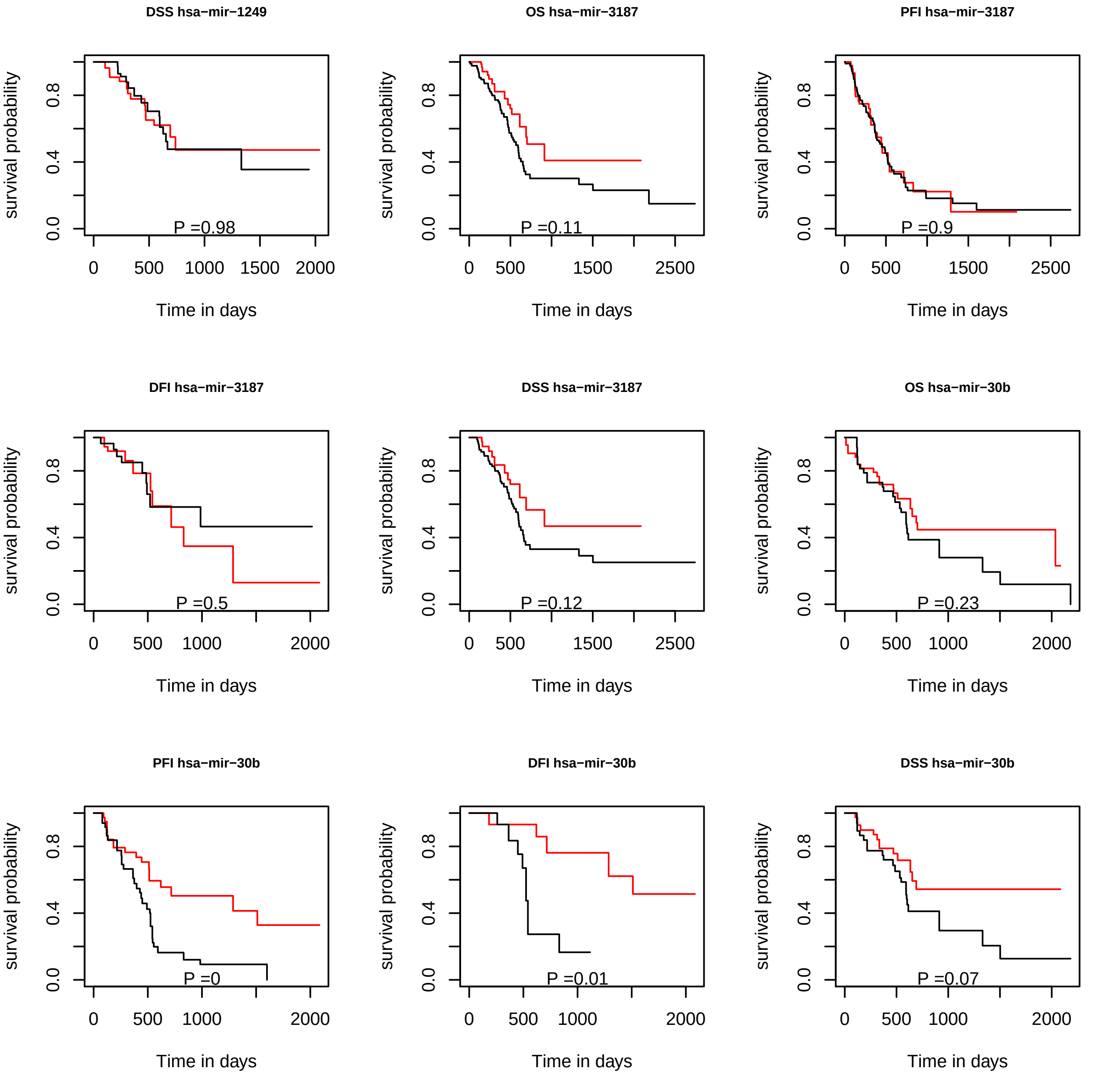
DSS hsa−mir−1249
P =0.98
OS hsa−mir−3187
P =0.11
PFI hsa−mir−3187
P =0.9
DFI hsa−mir−3187
P =0.5
DSS hsa−mir−3187
P =0.12
OS hsa−mir−30b
P =0.23
PFI hsa−mir−30b
P =0
DFI hsa−mir−30b
P =0.01
DSS hsa−mir−30b
P =0.07
survival probability
Time in days

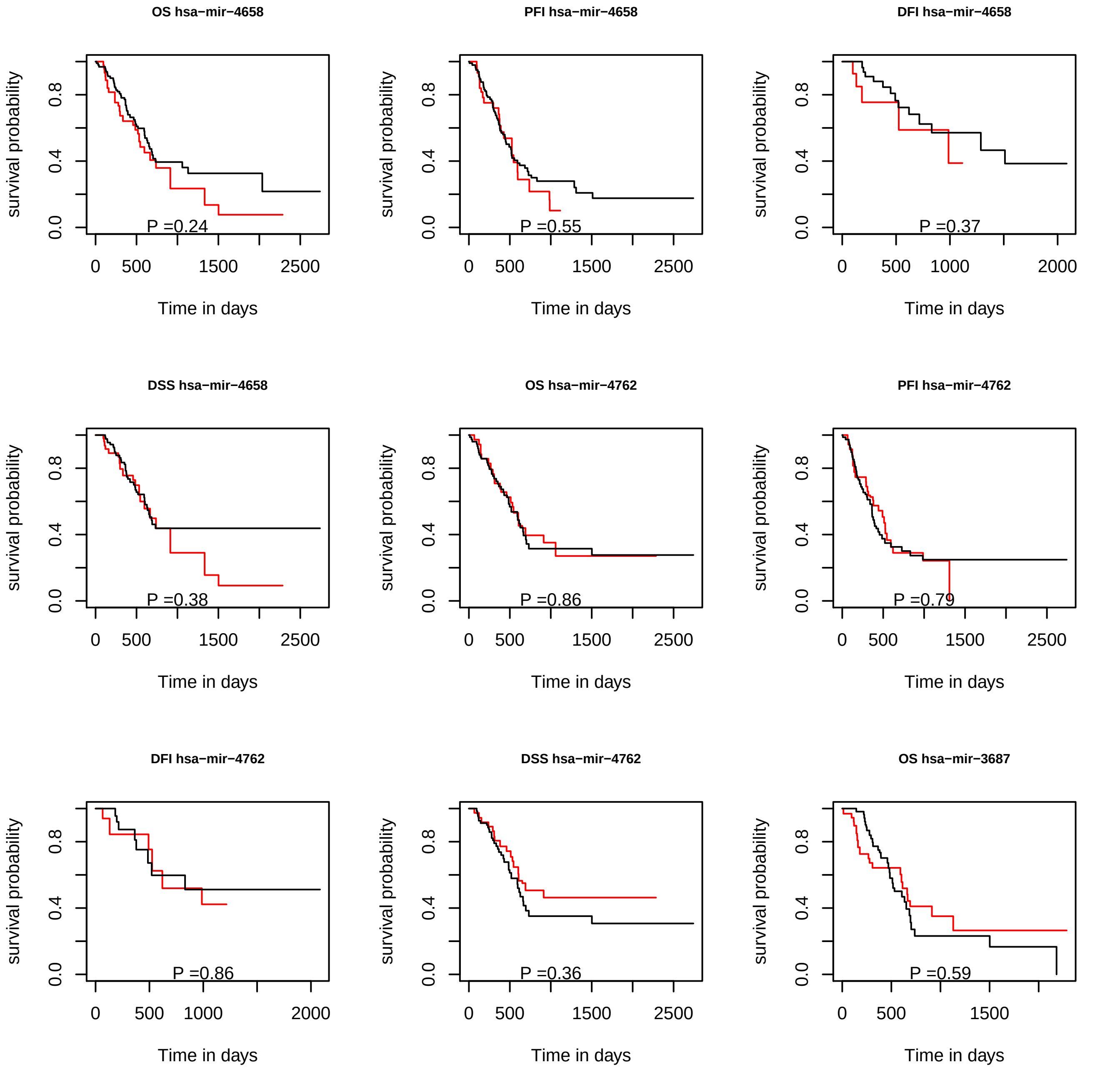
OS hsa−mir−4658
P =0.24
PFI hsa−mir−4658
P =0.55
DFI hsa−mir−4658
P =0.37
DSS hsa−mir−4658
P =0.38
OS hsa−mir−4762
P =0.86
PFI hsa−mir−4762
P =0.79
DFI hsa−mir−4762
P =0.86
DSS hsa−mir−4762
P =0.36
OS hsa−mir−3687
P =0.59
survival probability
Time in days
0.0
0.4
0.8
0
500
1000
1500
2000
2500

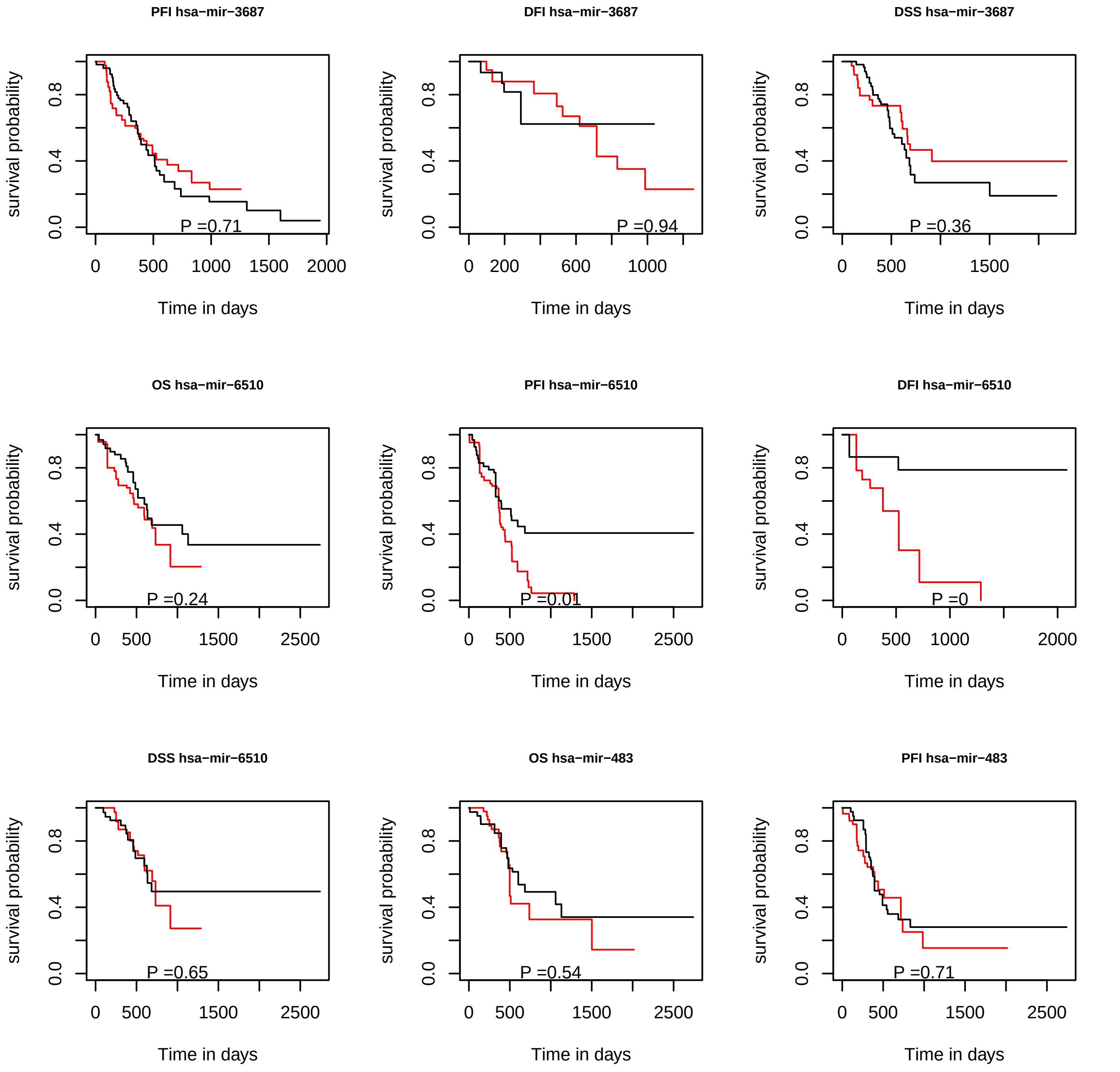
PFI hsa−mir−3687
P =0.71
DFI hsa−mir−3687
P =0.94
DSS hsa−mir−3687
P =0.36
OS hsa−mir−6510
P =0.24
PFI hsa−mir−6510
P =0.01
DFI hsa−mir−6510
P =0
DSS hsa−mir−6510
P =0.65
OS hsa−mir−483
P =0.54
PFI hsa−mir−483
P =0.71
survival probability
Time in days
0.0
0.4
0.8
0
200
500
600
1000
1500
2000
2500

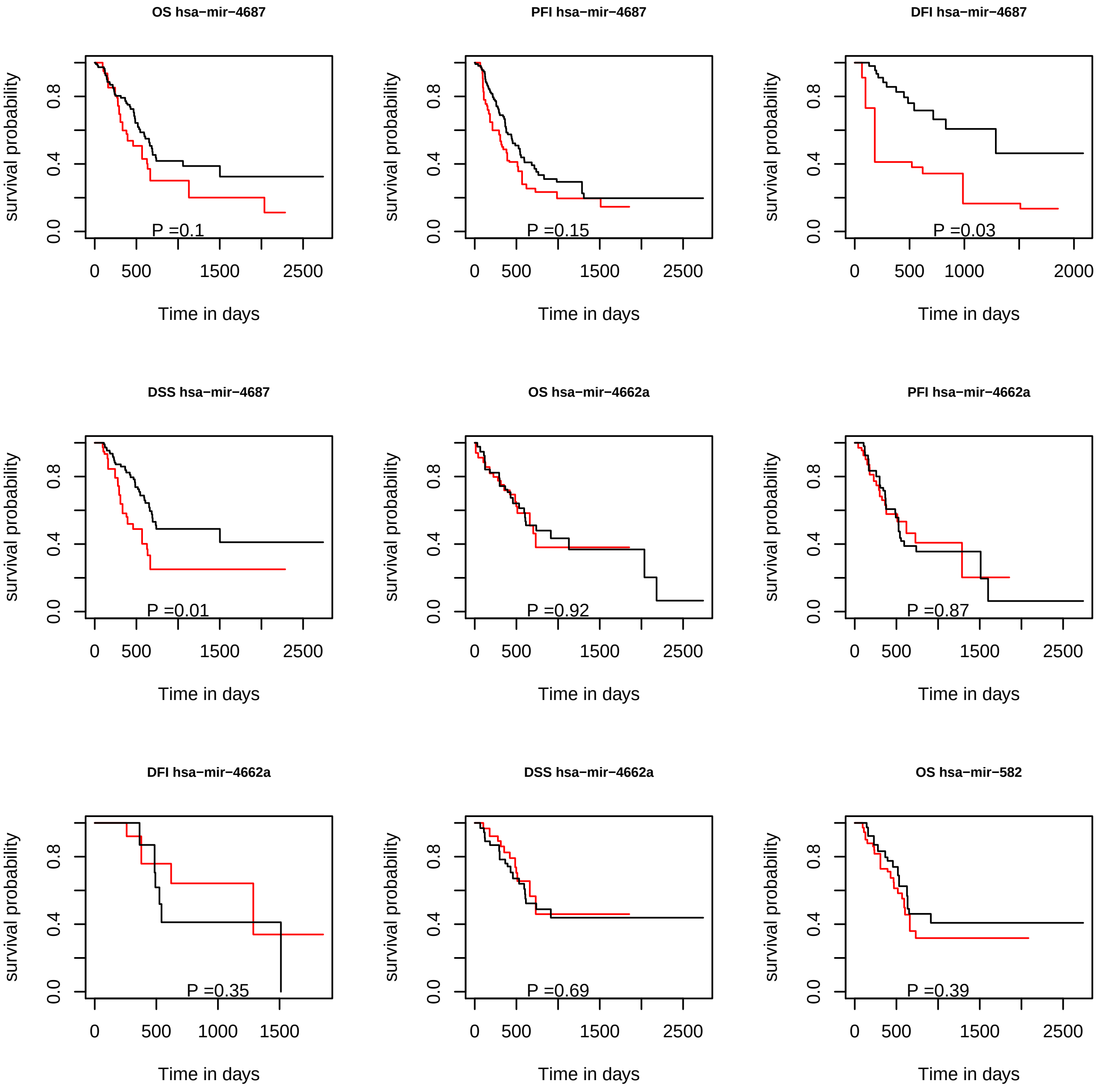
OS hsa−mir−4687
survival probability
0.0 0.4 0.8
0 500 1500 2500
Time in days
P =0.1
PFI hsa−mir−4687
survival probability
0.0 0.4 0.8
0 500 1500 2500
Time in days
P =0.15
DFI hsa−mir−4687
survival probability
0.0 0.4 0.8
0 500 1000 2000
Time in days
P =0.03
DSS hsa−mir−4687
survival probability
0.0 0.4 0.8
0 500 1500 2500
Time in days
P =0.01
OS hsa−mir−4662a
survival probability
0.0 0.4 0.8
0 500 1500 2500
Time in days
P =0.92
PFI hsa−mir−4662a
survival probability
0.0 0.4 0.8
0 500 1500 2500
Time in days
P =0.87
DFI hsa−mir−4662a
survival probability
0.0 0.4 0.8
0 500 1000 1500
Time in days
P =0.35
DSS hsa−mir−4662a
survival probability
0.0 0.4 0.8
0 500 1500 2500
Time in days
P =0.69
OS hsa−mir−582
survival probability
0.0 0.4 0.8
0 500 1500 2500
Time in days
P =0.39

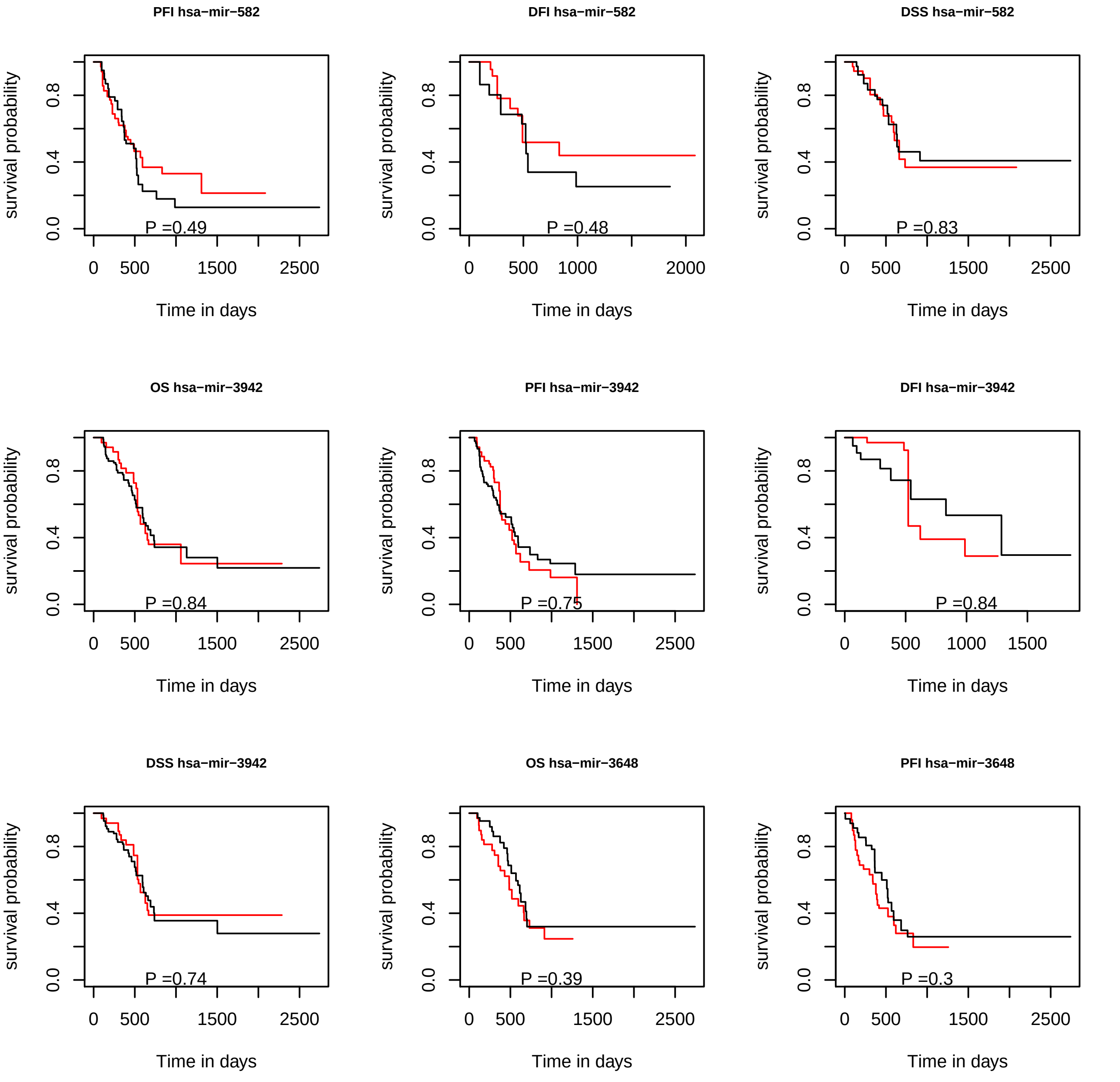
PFI hsa−mir−582
P =0.49
DFI hsa−mir−582
P =0.48
DSS hsa−mir−582
P =0.83
OS hsa−mir−3942
P =0.84
PFI hsa−mir−3942
P =0.75
DFI hsa−mir−3942
P =0.84
DSS hsa−mir−3942
P =0.74
OS hsa−mir−3648
P =0.39
PFI hsa−mir−3648
P =0.3
survival probability
Time in days
0.0
0.4
0.8
0
500
1000
1500
2000
2500

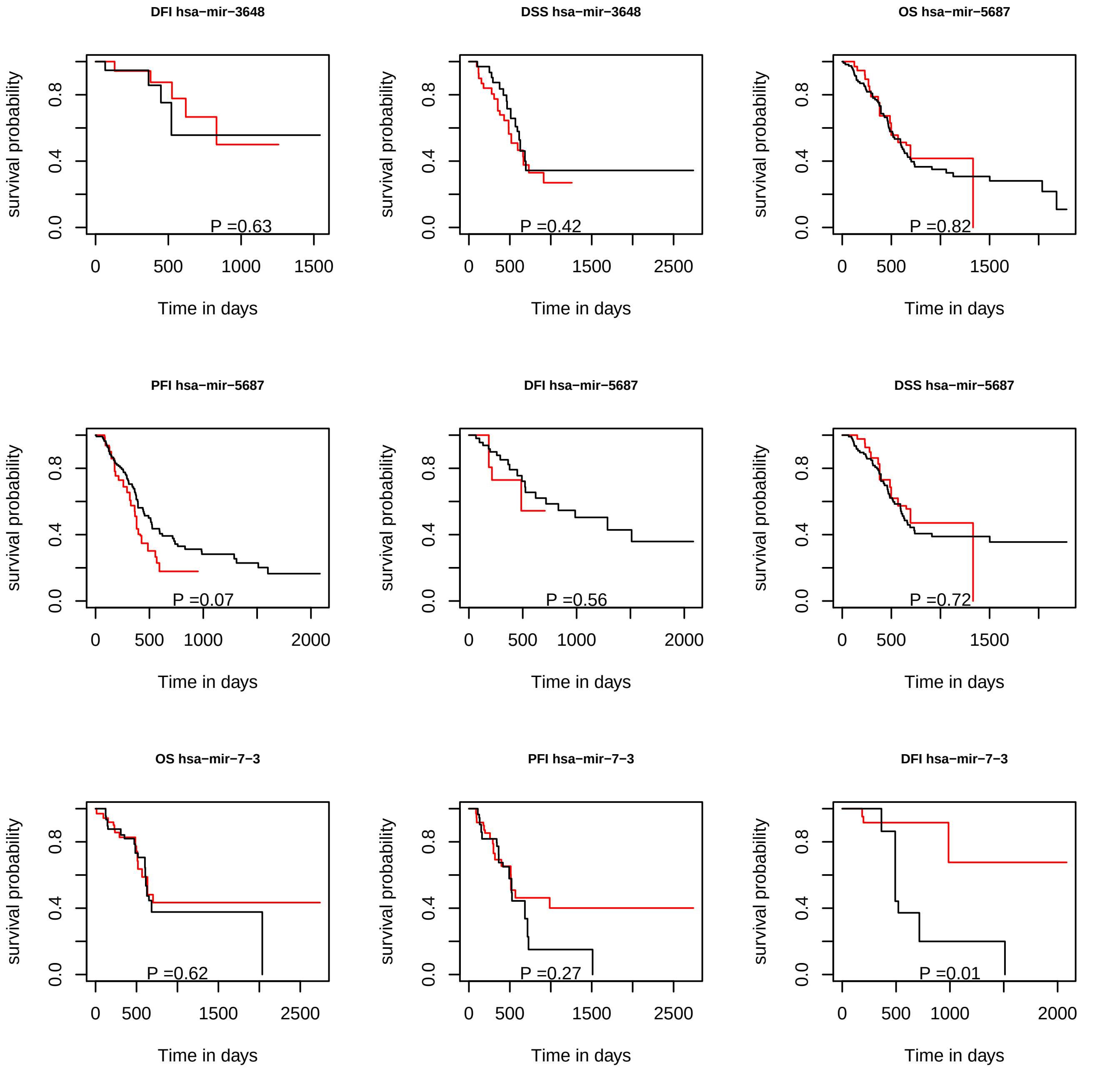
DFI hsa−mir−3648
P =0.63
DSS hsa−mir−3648
P =0.42
OS hsa−mir−5687
P =0.82
PFI hsa−mir−5687
P =0.07
DFI hsa−mir−5687
P =0.56
DSS hsa−mir−5687
P =0.72
OS hsa−mir−7−3
P =0.62
PFI hsa−mir−7−3
P =0.27
DFI hsa−mir−7−3
P =0.01
survival probability
Time in days

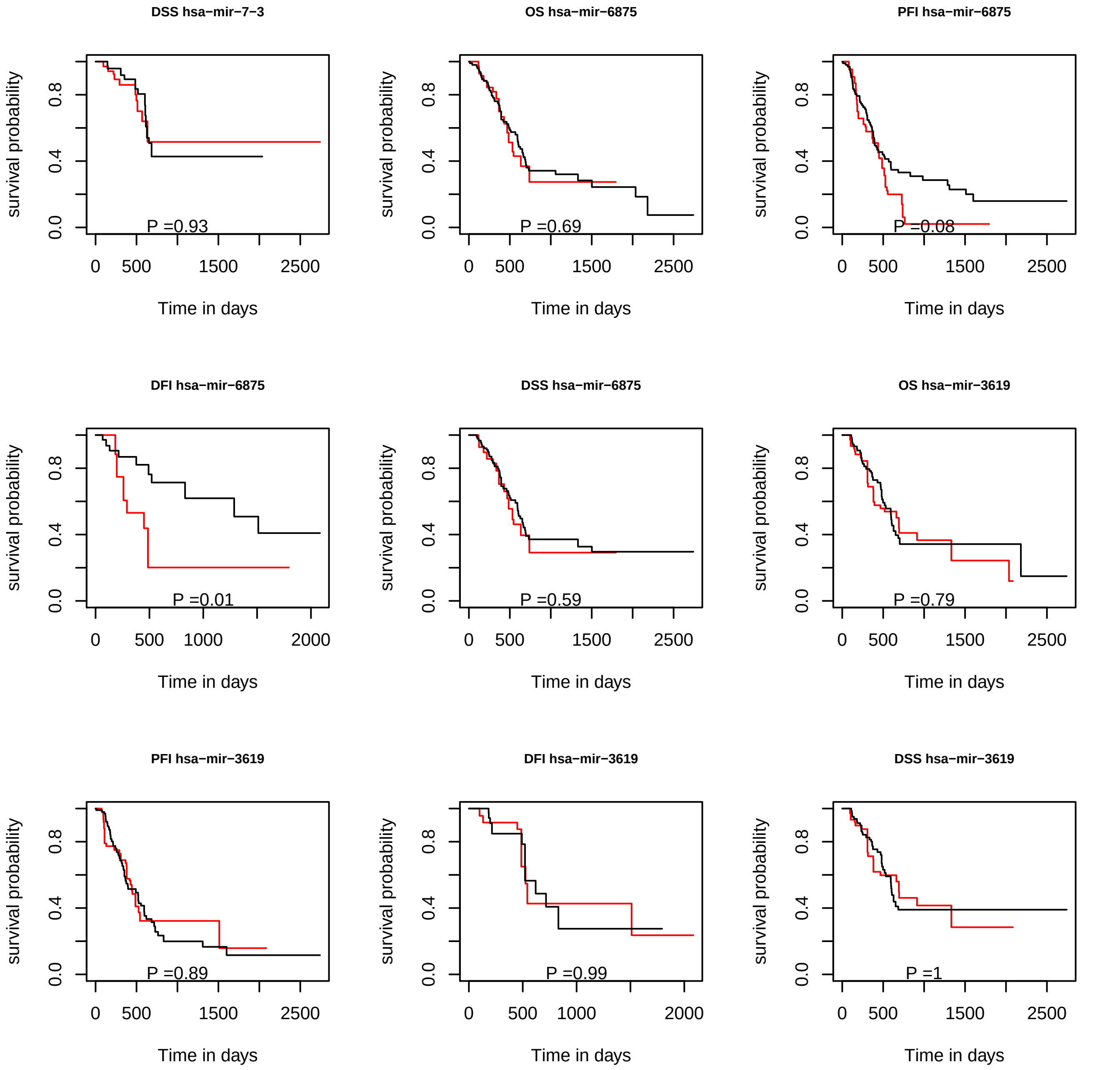
DSS hsa−mir−7−3
P =0.93
OS hsa−mir−6875
P =0.69
PFI hsa−mir−6875
P =0.08
DFI hsa−mir−6875
P =0.01
DSS hsa−mir−6875
P =0.59
OS hsa−mir−3619
P =0.79
PFI hsa−mir−3619
P =0.89
DFI hsa−mir−3619
P =0.99
DSS hsa−mir−3619
P =1
survival probability
0.0
0.4
0.8
Time in days
0
500
1000
1500
2000
2500

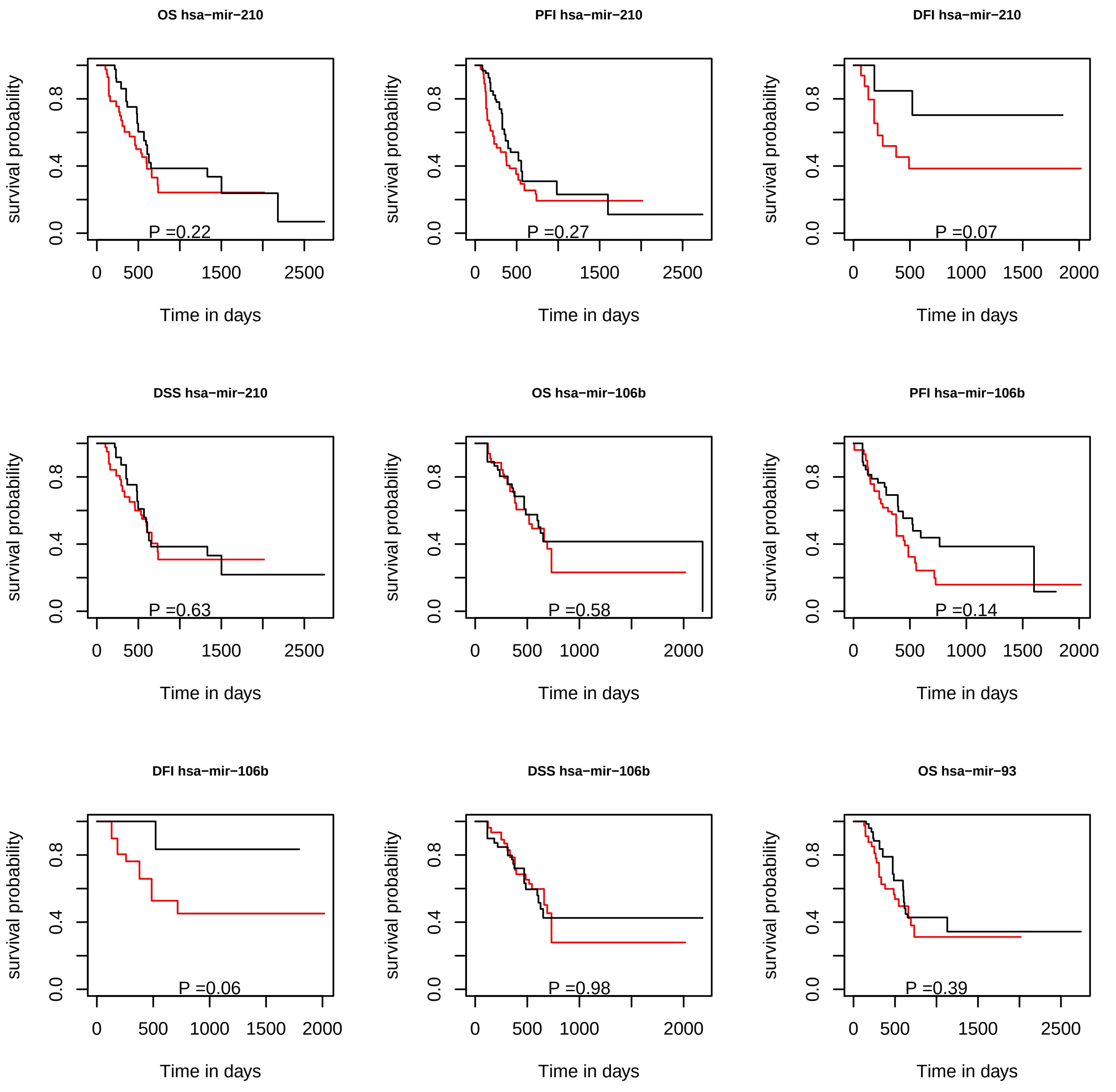
OS hsa−mir−210
P =0.22
PFI hsa−mir−210
P =0.27
DFI hsa−mir−210
P =0.07
DSS hsa−mir−210
P =0.63
OS hsa−mir−106b
P =0.58
PFI hsa−mir−106b
P =0.14
DFI hsa−mir−106b
P =0.06
DSS hsa−mir−106b
P =0.98
OS hsa−mir−93
P =0.39
survival probability
0.0
0.4
0.8
Time in days
0
500
1000
1500
2000
2500

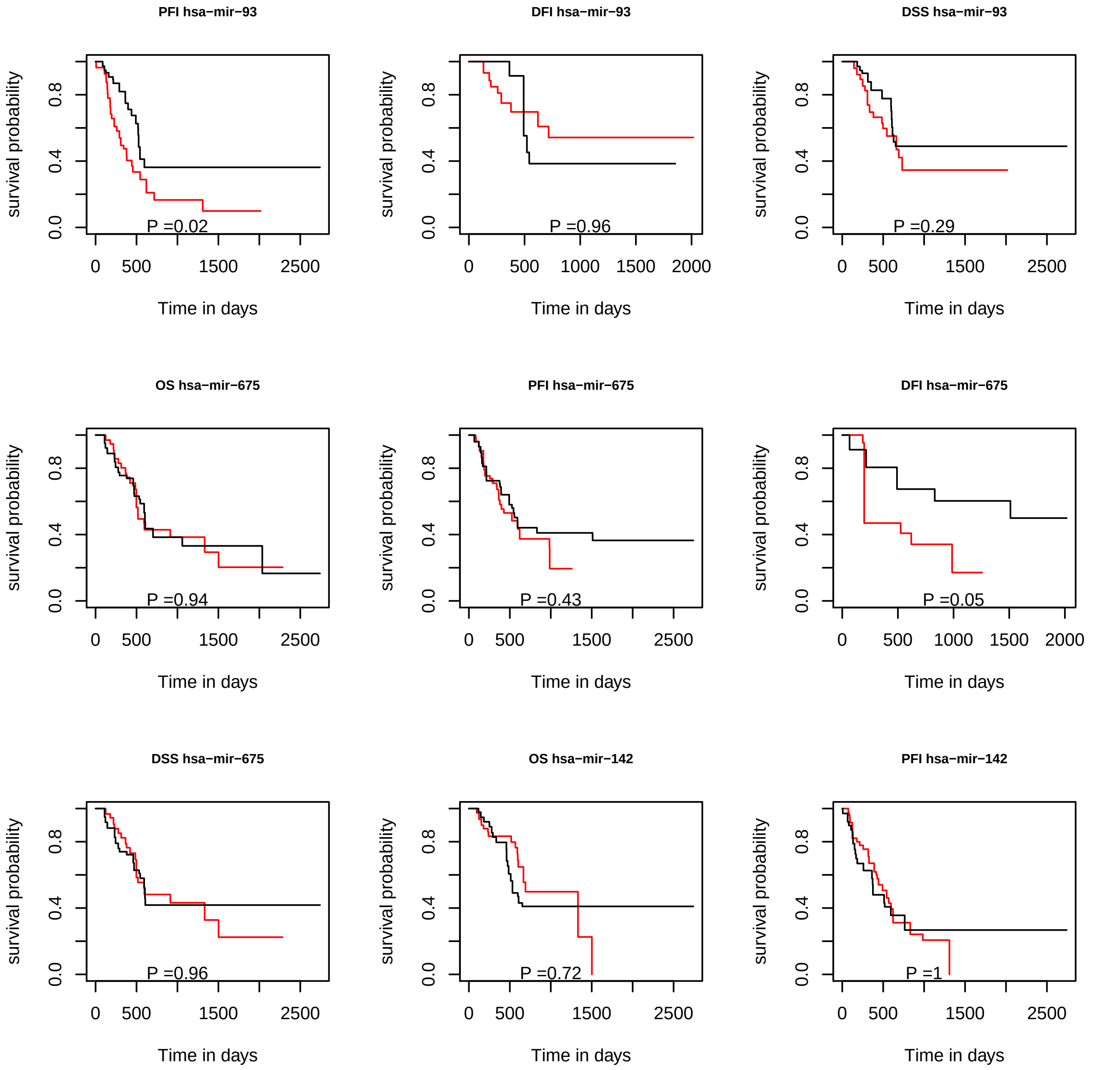
PFI hsa−mir−93
survival probability
0.0
0.4
0.8
P =0.02
0
500
1500
2500
Time in days
DFI hsa−mir−93
survival probability
0.0
0.4
0.8
P =0.96
0
500
1000
1500
2000
Time in days
DSS hsa−mir−93
survival probability
0.0
0.4
0.8
P =0.29
0
500
1500
2500
Time in days
OS hsa−mir−675
survival probability
0.0
0.4
0.8
P =0.94
0
500
1500
2500
Time in days
PFI hsa−mir−675
survival probability
0.0
0.4
0.8
P =0.43
0
500
1500
2500
Time in days
DFI hsa−mir−675
survival probability
0.0
0.4
0.8
P =0.05
0
500
1000
1500
2000
Time in days
DSS hsa−mir−675
survival probability
0.0
0.4
0.8
P =0.96
0
500
1500
2500
Time in days
OS hsa−mir−142
survival probability
0.0
0.4
0.8
P =0.72
0
500
1500
2500
Time in days
PFI hsa−mir−142
survival probability
0.0
0.4
0.8
P =1
0
500
1500
2500
Time in days

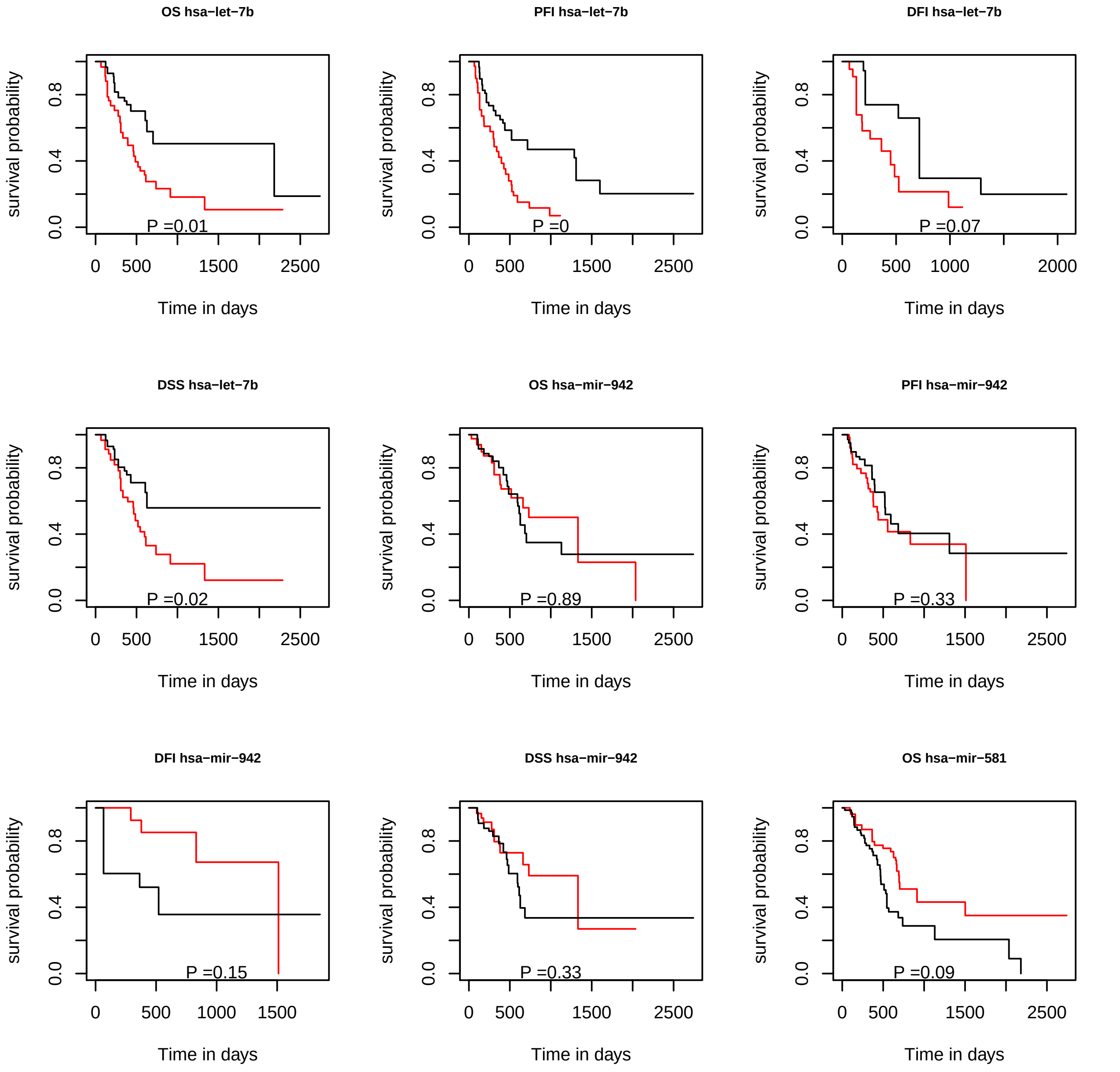
OS hsa−let−7b
survival probability
Time in days
P =0.01
PFI hsa−let−7b
survival probability
Time in days
P =0
DFI hsa−let−7b
survival probability
Time in days
P =0.07
DSS hsa−let−7b
survival probability
Time in days
P =0.02
OS hsa−mir−942
survival probability
Time in days
P =0.89
PFI hsa−mir−942
survival probability
Time in days
P =0.33
DFI hsa−mir−942
survival probability
Time in days
P =0.15
DSS hsa−mir−942
survival probability
Time in days
P =0.33
OS hsa−mir−581
survival probability
Time in days
P =0.09

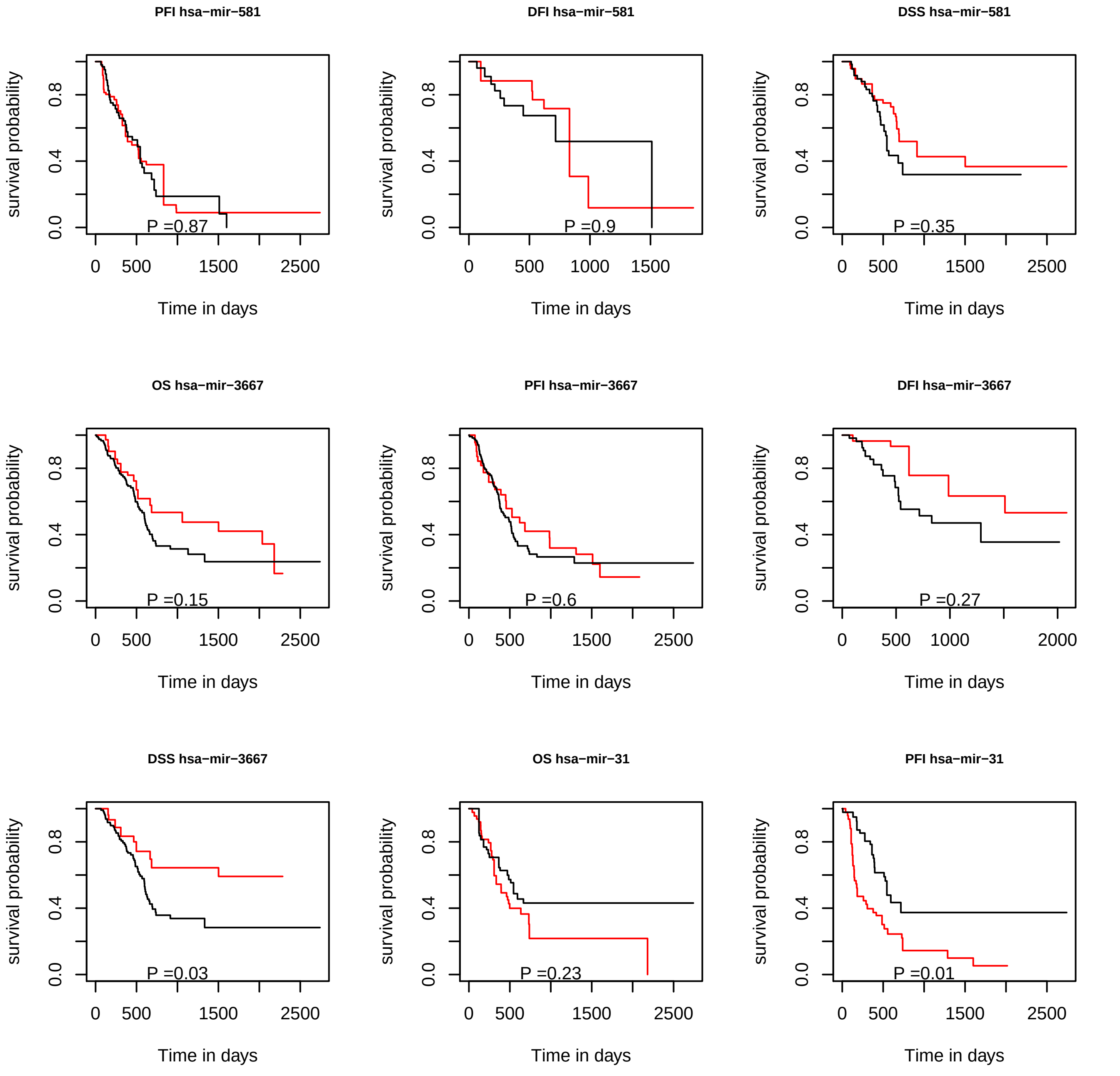
PFI hsa−mir−581
P =0.87
DFI hsa−mir−581
P =0.9
DSS hsa−mir−581
P =0.35
OS hsa−mir−3667
P =0.15
PFI hsa−mir−3667
P =0.6
DFI hsa−mir−3667
P =0.27
DSS hsa−mir−3667
P =0.03
OS hsa−mir−31
P =0.23
PFI hsa−mir−31
P =0.01
survival probability
Time in days
0.0
0.4
0.8
0
500
1000
1500
2000
2500

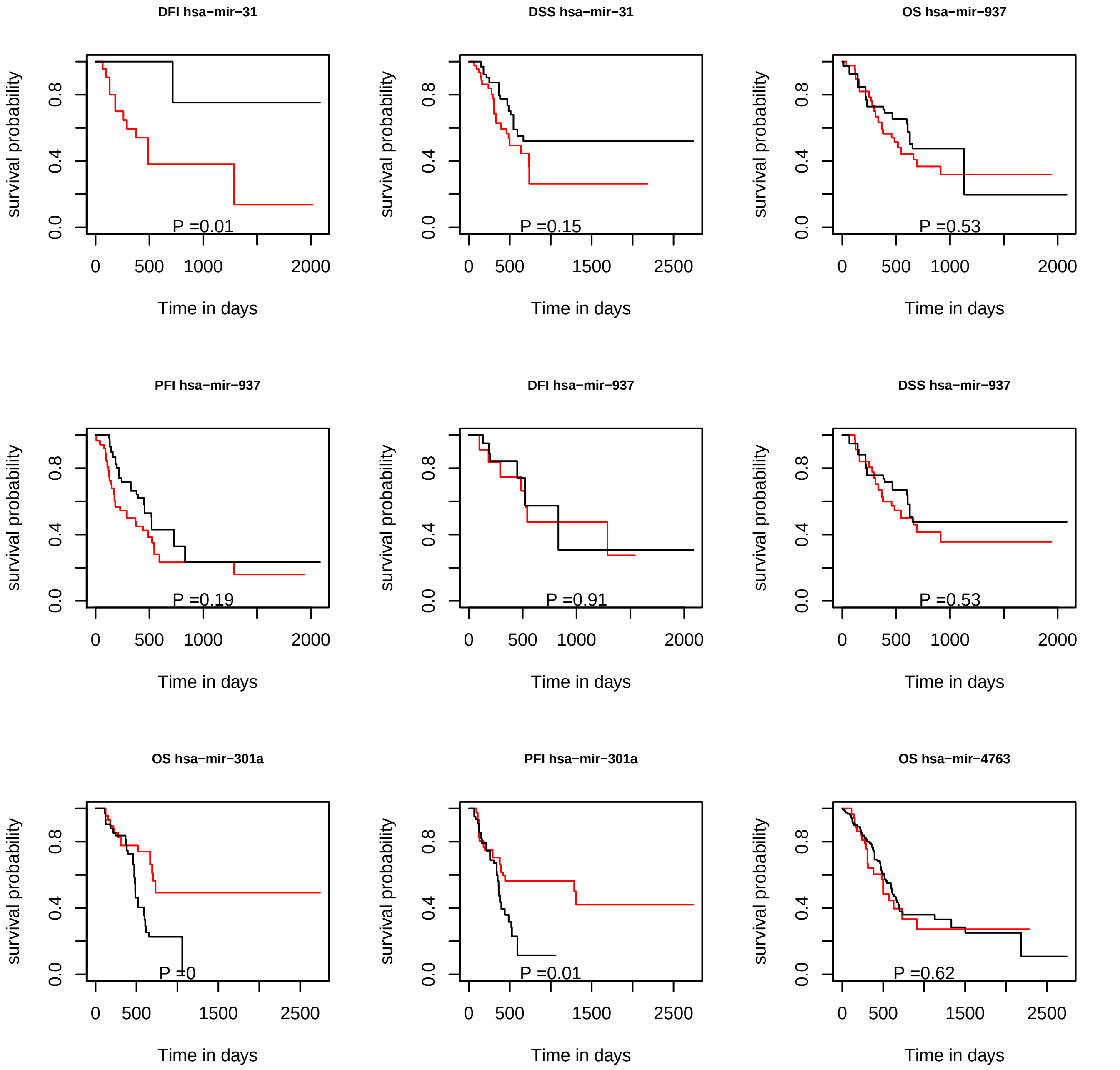
DFI hsa−mir−31
P =0.01
DSS hsa−mir−31
P =0.15
OS hsa−mir−937
P =0.53
PFI hsa−mir−937
P =0.19
DFI hsa−mir−937
P =0.91
DSS hsa−mir−937
P =0.53
OS hsa−mir−301a
P =0
PFI hsa−mir−301a
P =0.01
OS hsa−mir−4763
P =0.62
survival probability
Time in days
0.0
0.4
0.8
0
500
1000
1500
2000
2500

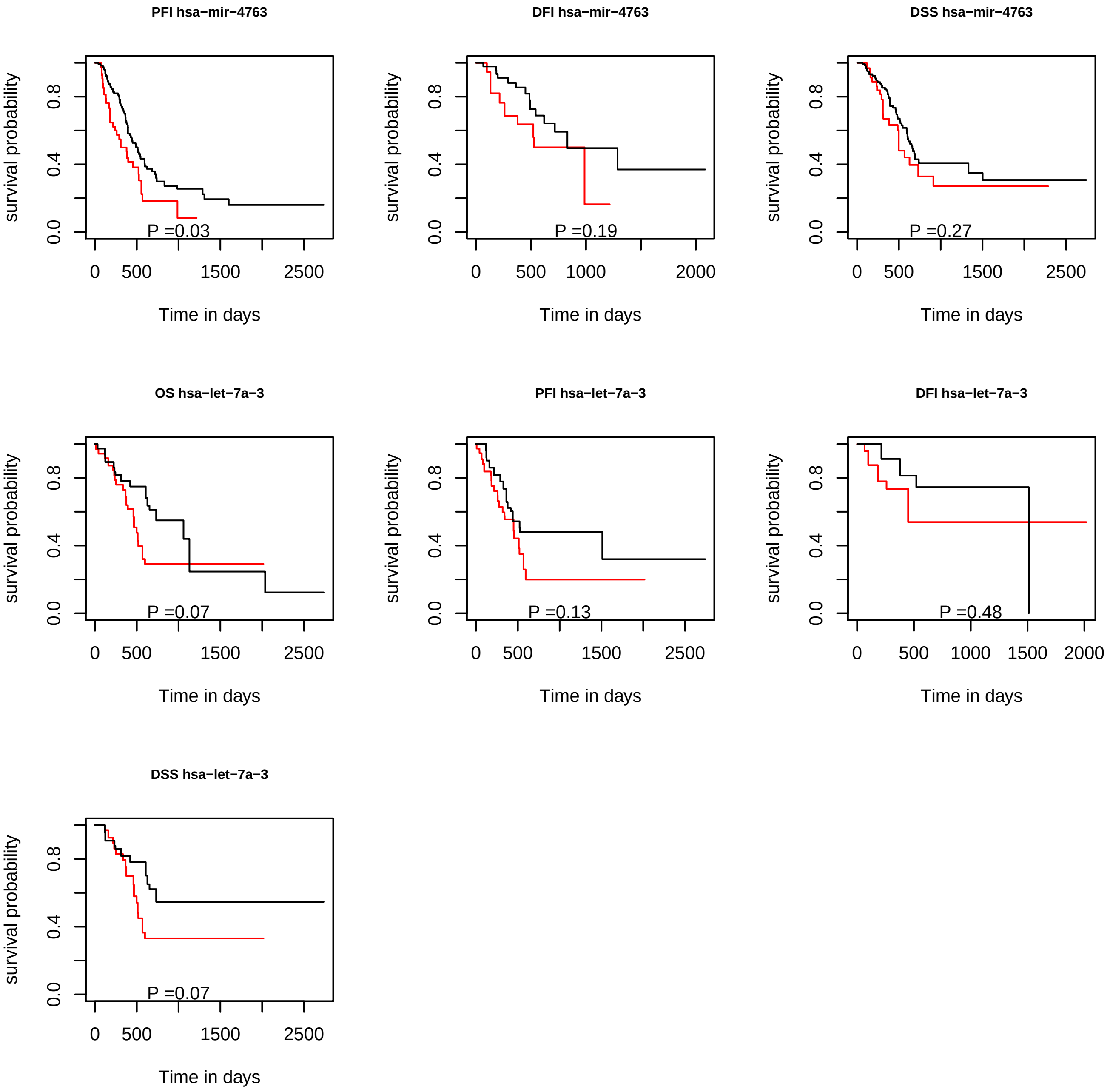
PFI hsa−mir−4763
P =0.03
DFI hsa−mir−4763
P =0.19
DSS hsa−mir−4763
P =0.27
OS hsa−let−7a−3
P =0.07
PFI hsa−let−7a−3
P =0.13
DFI hsa−let−7a−3
P =0.48
DSS hsa−let−7a−3
P =0.07
survival probability
0.0
0.4
0.8
Time in days
0
500
1000
1500
2000
2500
