# Supplementary material for: Computing microRNA-gene interaction networks in pan-cancer using miRDriver
Source: Sci Rep. 2022 Mar 8;12:3717. doi: 10.1038/s41598-022-07628-z (PMC8904490; doi:10.1038/s41598-022-07628-z)
Supplement: Supplementary file 25 — Supplementary Information 25. [file 41598_2022_7628_MOESM25_ESM.pdf]

Computing microRNA-gene interaction networks in pan-cancer using miRDriver

Banabithi Bose, Matthew Moravec, and Serdar Bozdag

Supplemental Figure S16

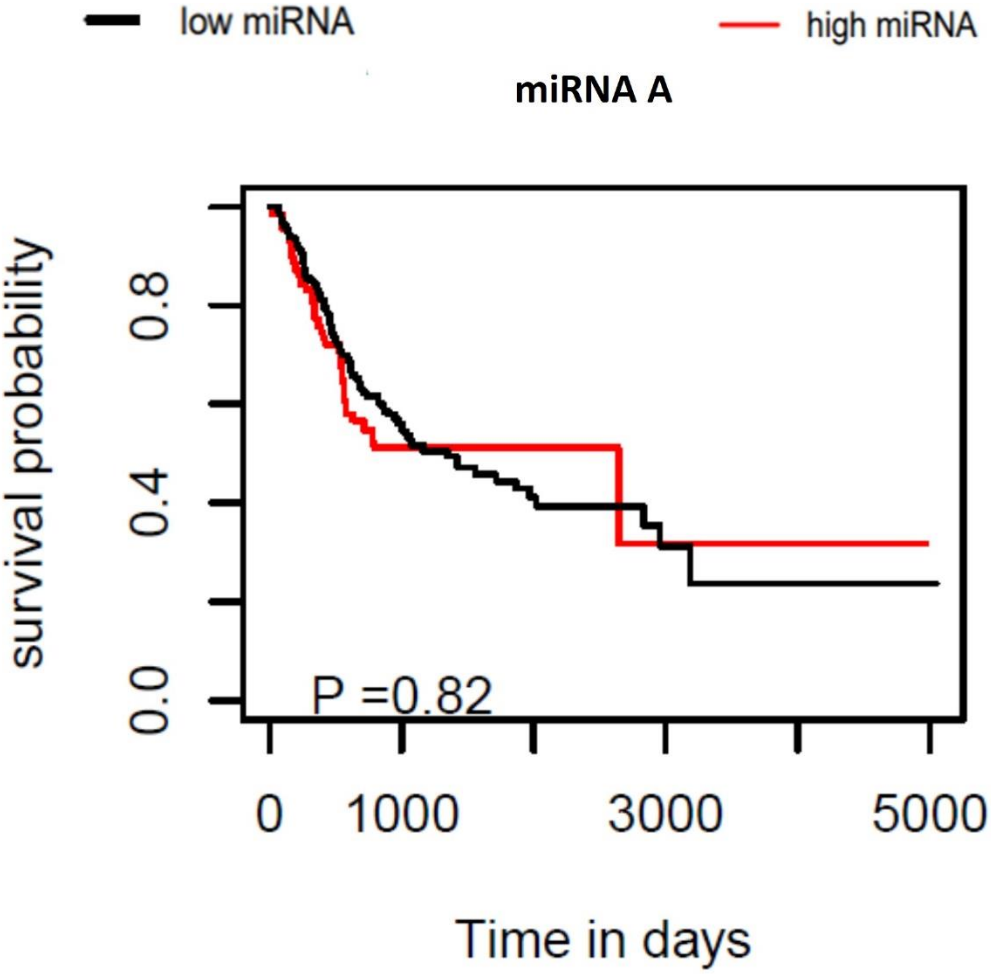

The *Adjusted Kaplan-Meier* survival plots for the computed miRNAs in high and low miRNA expression patient groups.

Supplemental Figure S16 Cancer Type: PRAD

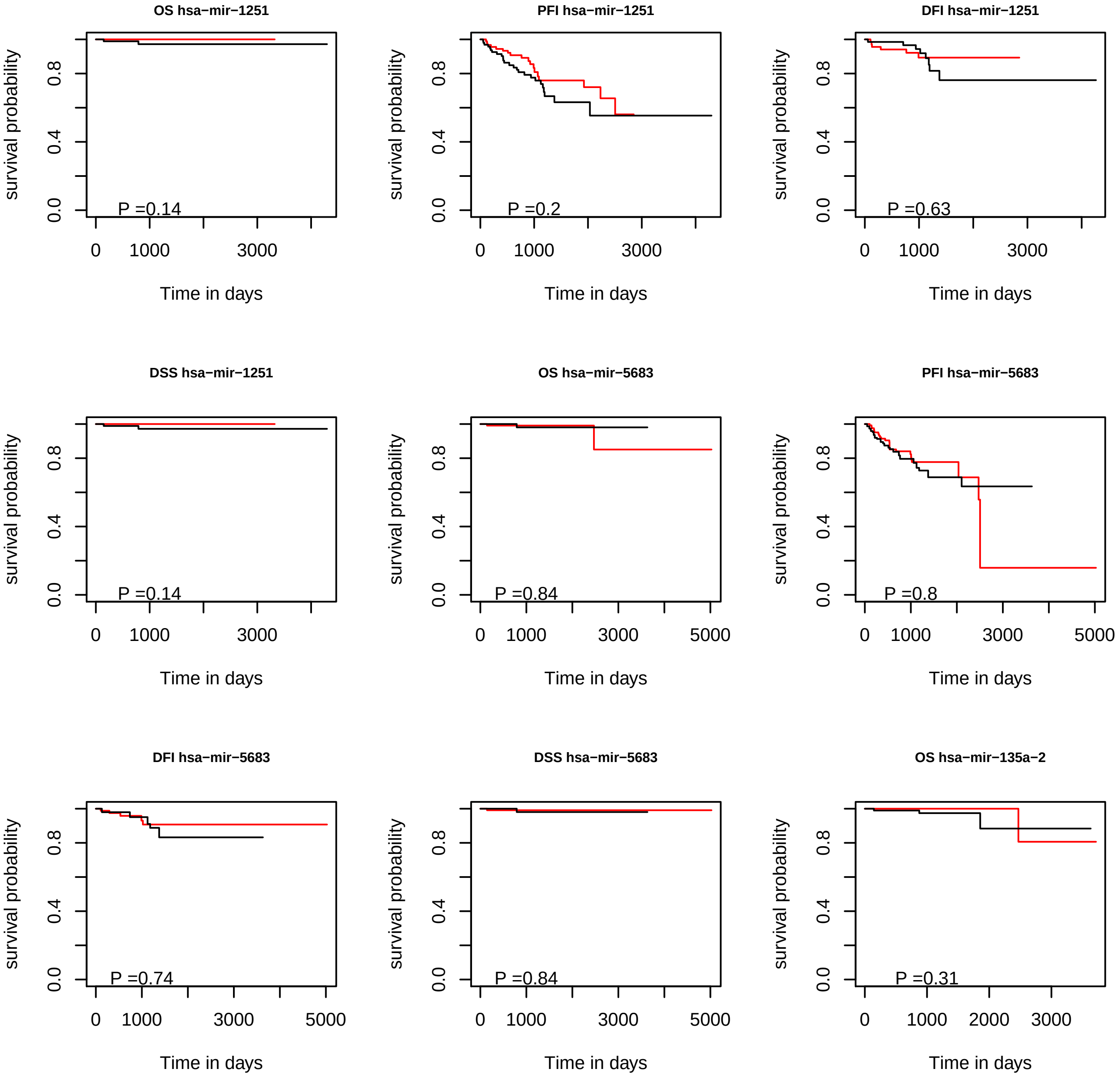

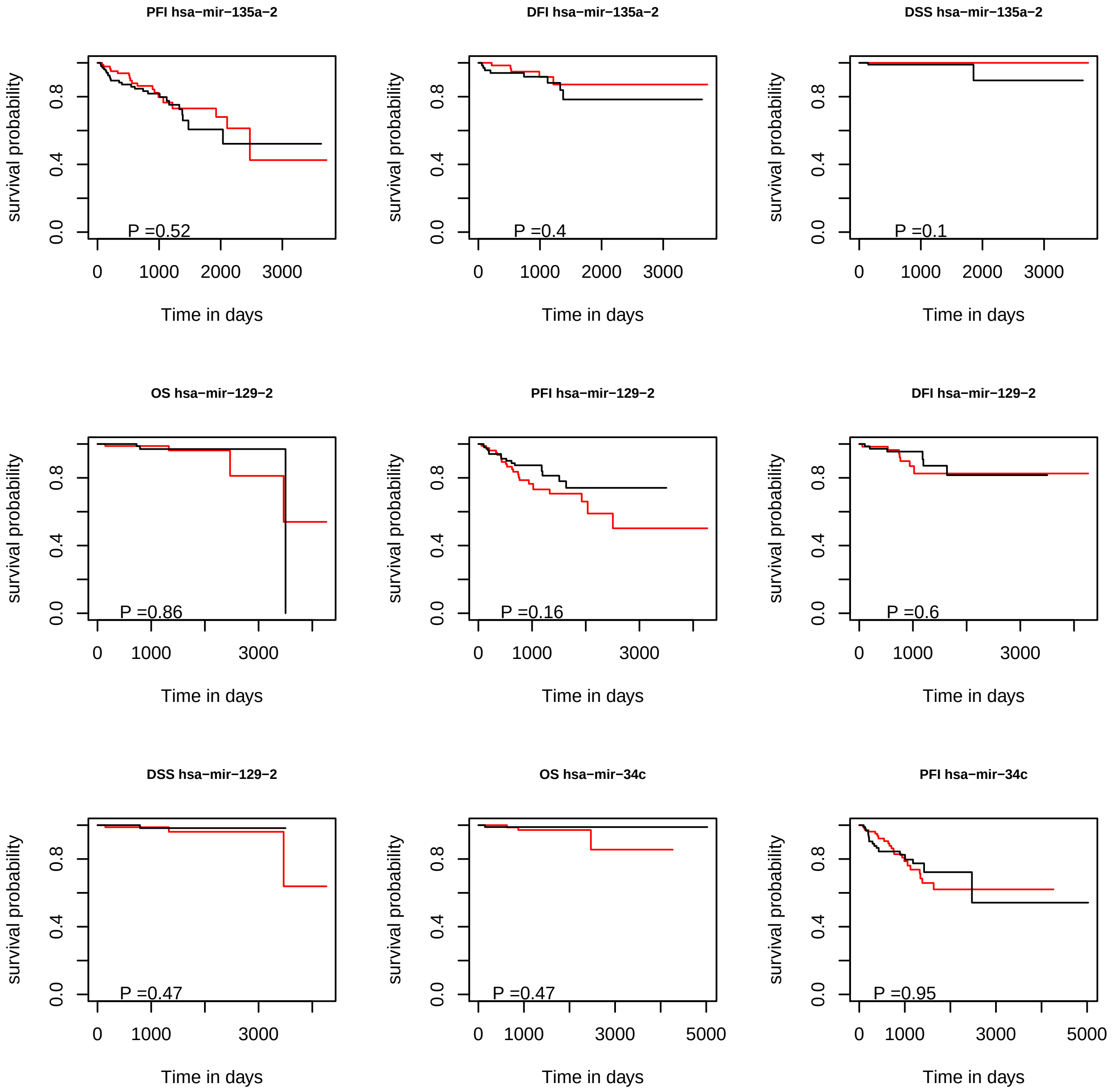
PFI hsa−mir−135a−2
P =0.52
DFI hsa−mir−135a−2
P =0.4
DSS hsa−mir−135a−2
P =0.1
OS hsa−mir−129−2
P =0.86
PFI hsa−mir−129−2
P =0.16
DFI hsa−mir−129−2
P =0.6
DSS hsa−mir−129−2
P =0.47
OS hsa−mir−34c
P =0.47
PFI hsa−mir−34c
P =0.95
survival probability
Time in days
0.0
0.4
0.8
0
1000
2000
3000
5000

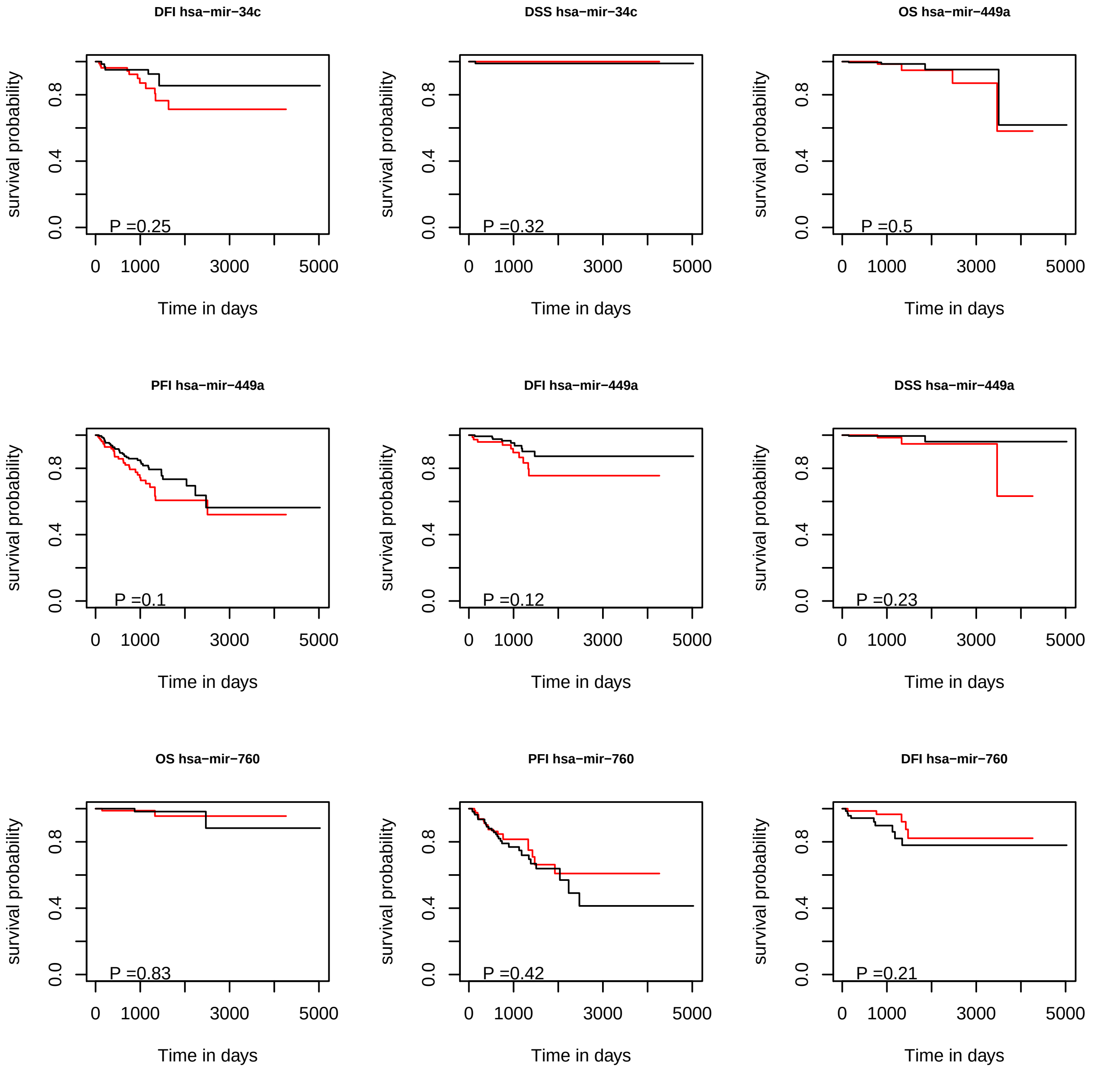
DFI hsa−mir−34c
P =0.25
DSS hsa−mir−34c
P =0.32
OS hsa−mir−449a
P =0.5
PFI hsa−mir−449a
P =0.1
DFI hsa−mir−449a
P =0.12
DSS hsa−mir−449a
P =0.23
OS hsa−mir−760
P =0.83
PFI hsa−mir−760
P =0.42
DFI hsa−mir−760
P =0.21
survival probability
0.0
0.4
0.8
Time in days
0
1000
3000
5000

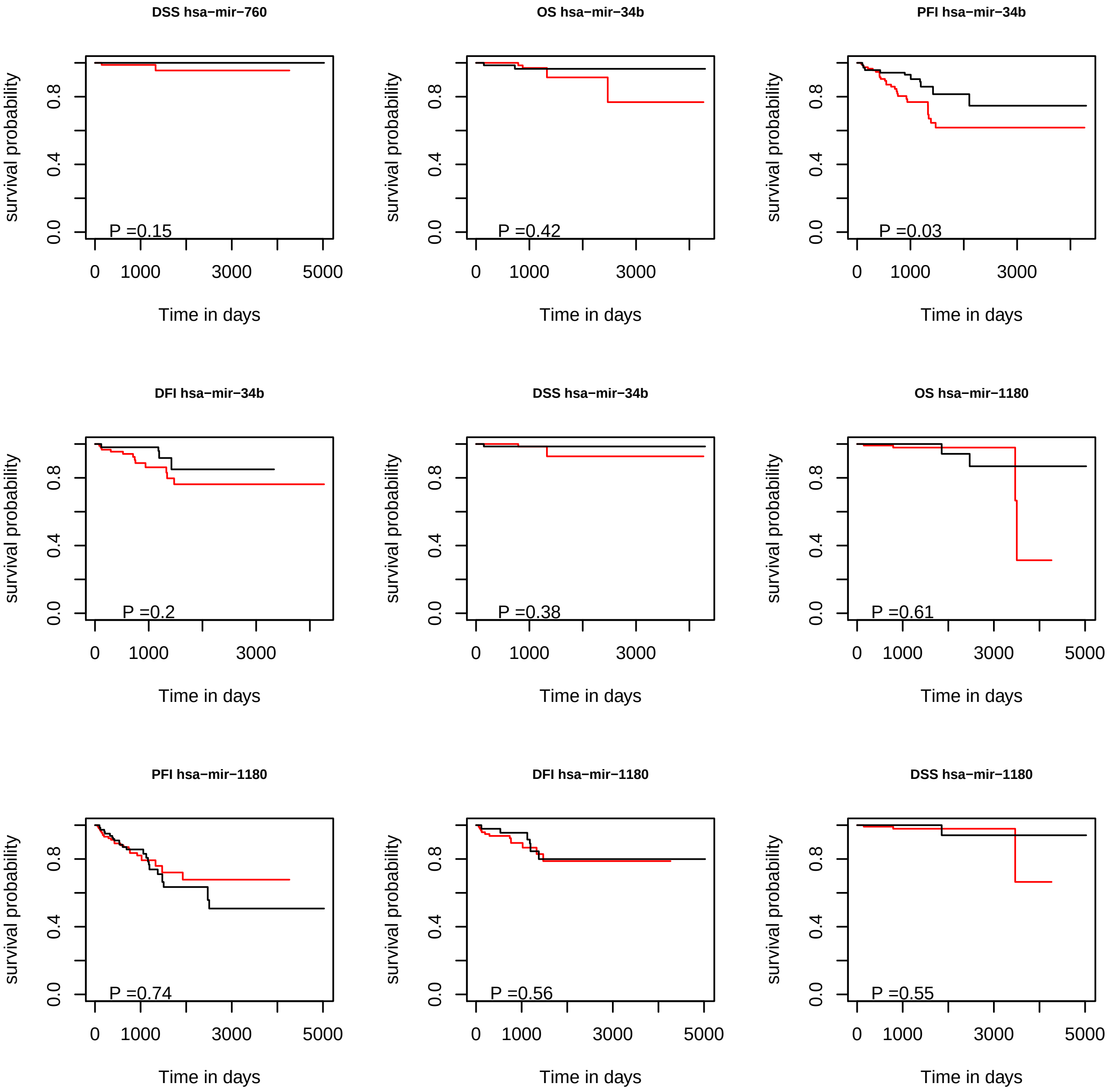
DSS hsa−mir−760
P =0.15
OS hsa−mir−34b
P =0.42
PFI hsa−mir−34b
P =0.03
DFI hsa−mir−34b
P =0.2
DSS hsa−mir−34b
P =0.38
OS hsa−mir−1180
P =0.61
PFI hsa−mir−1180
P =0.74
DFI hsa−mir−1180
P =0.56
DSS hsa−mir−1180
P =0.55
survival probability
Time in days

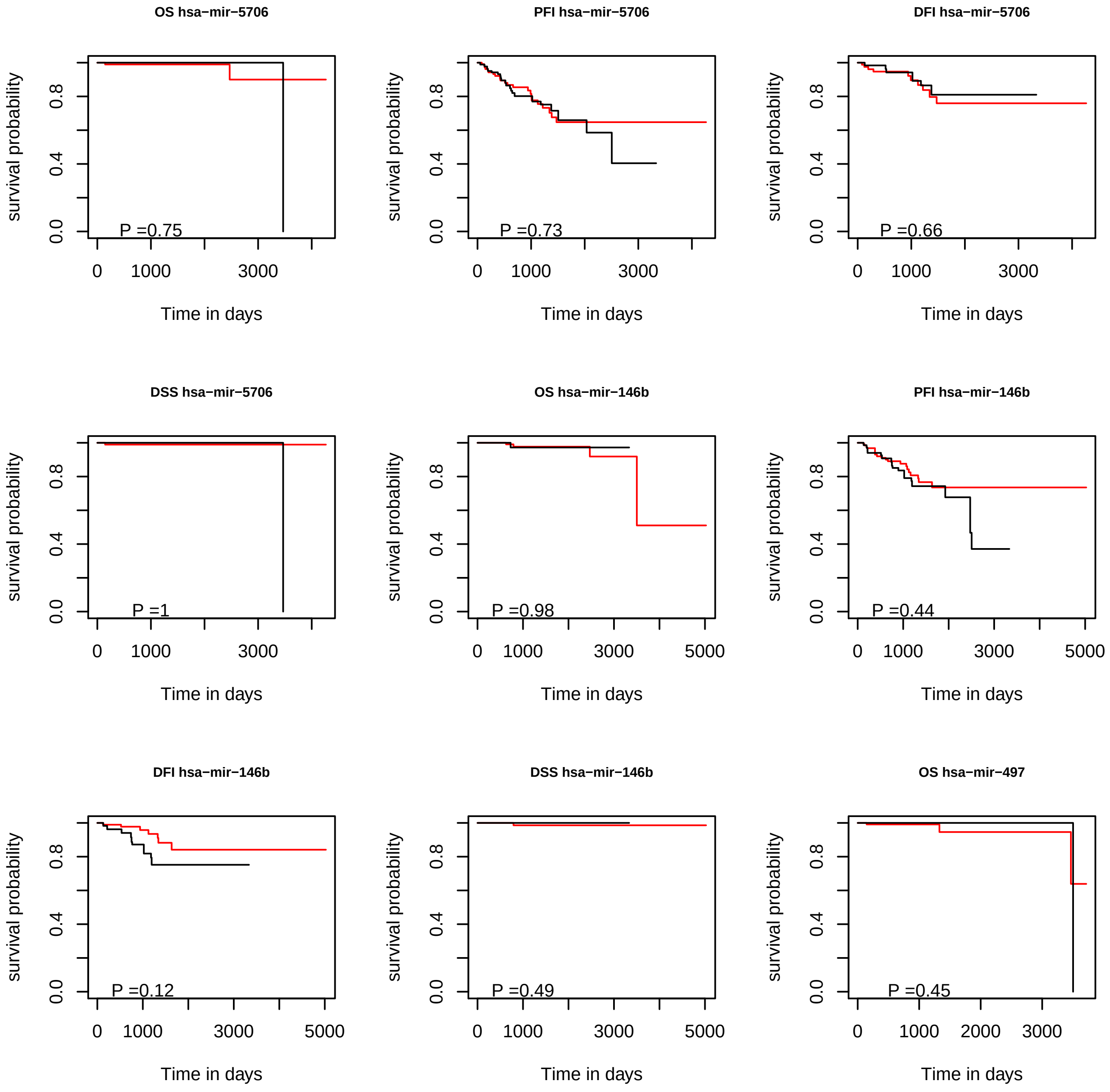
OS hsa−mir−5706
P =0.75
PFI hsa−mir−5706
P =0.73
DFI hsa−mir−5706
P =0.66
DSS hsa−mir−5706
P =1
OS hsa−mir−146b
P =0.98
PFI hsa−mir−146b
P =0.44
DFI hsa−mir−146b
P =0.12
DSS hsa−mir−146b
P =0.49
OS hsa−mir−497
P =0.45
survival probability
Time in days
0.0
0.4
0.8
0
1000
2000
3000
5000

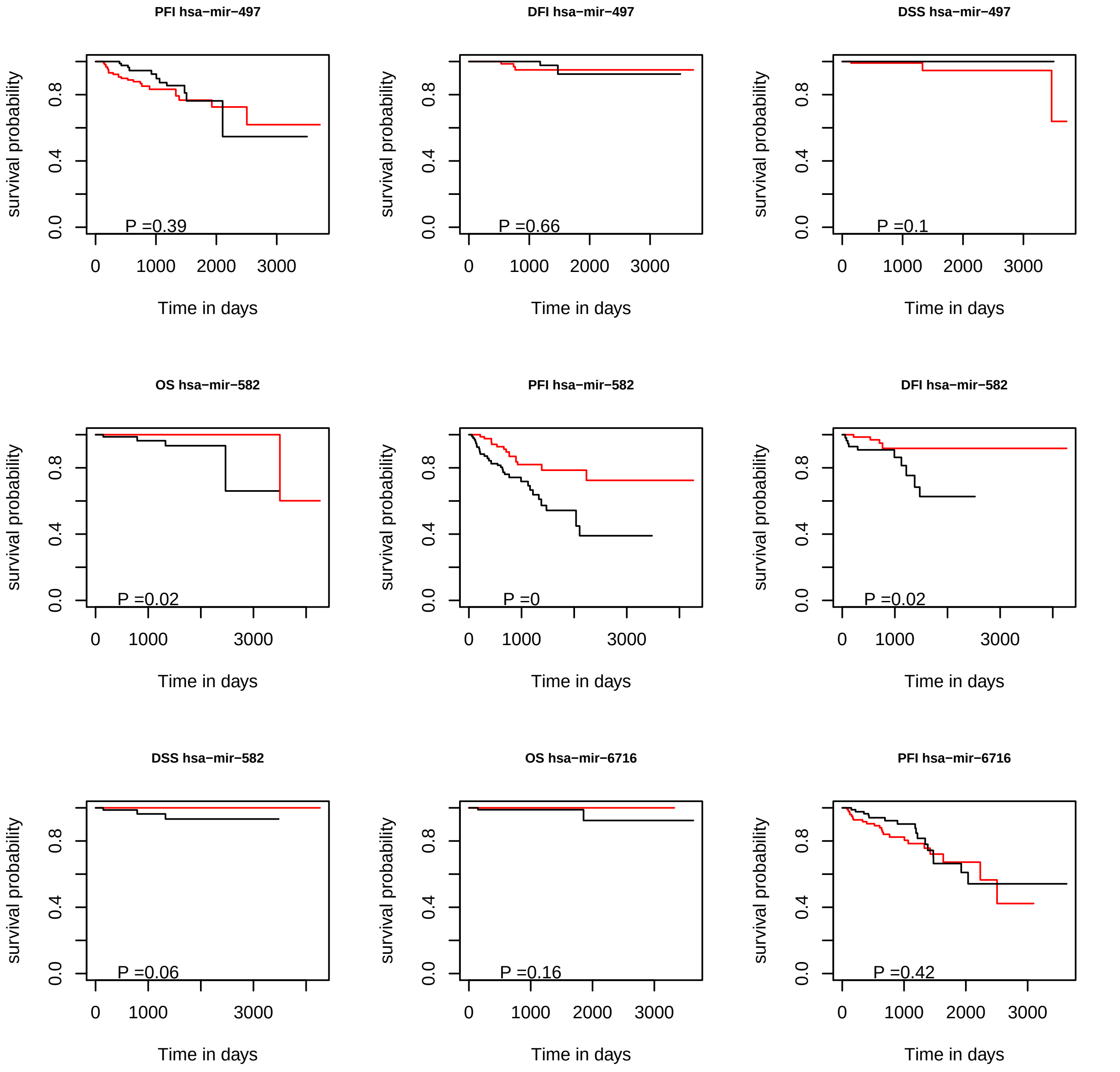
PFI hsa−mir−497
P =0.39
DFI hsa−mir−497
P =0.66
DSS hsa−mir−497
P =0.1
OS hsa−mir−582
P =0.02
PFI hsa−mir−582
P =0
DFI hsa−mir−582
P =0.02
DSS hsa−mir−582
P =0.06
OS hsa−mir−6716
P =0.16
PFI hsa−mir−6716
P =0.42
survival probability
Time in days
0.0
0.4
0.8
0
1000
2000
3000

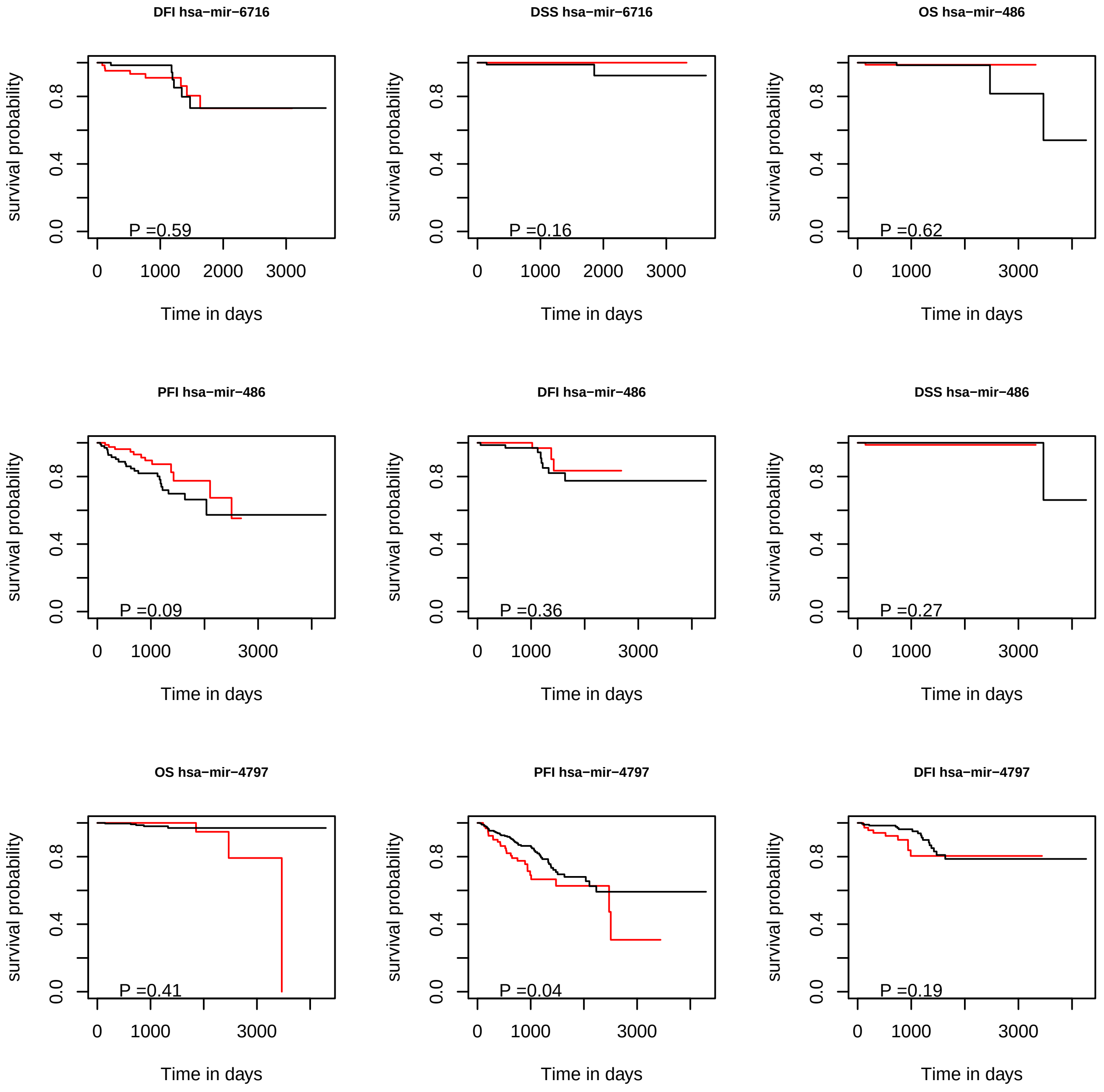
DFI hsa−mir−6716
survival probability
0.0
0.4
0.8
P =0.59
0
1000
2000
3000
Time in days
DSS hsa−mir−6716
survival probability
0.0
0.4
0.8
P =0.16
0
1000
2000
3000
Time in days
OS hsa−mir−486
survival probability
0.0
0.4
0.8
P =0.62
0
1000
3000
Time in days
PFI hsa−mir−486
survival probability
0.0
0.4
0.8
P =0.09
0
1000
3000
Time in days
DFI hsa−mir−486
survival probability
0.0
0.4
0.8
P =0.36
0
1000
3000
Time in days
DSS hsa−mir−486
survival probability
0.0
0.4
0.8
P =0.27
0
1000
3000
Time in days
OS hsa−mir−4797
survival probability
0.0
0.4
0.8
P =0.41
0
1000
3000
Time in days
PFI hsa−mir−4797
survival probability
0.0
0.4
0.8
P =0.04
0
1000
3000
Time in days
DFI hsa−mir−4797
survival probability
0.0
0.4
0.8
P =0.19
0
1000
3000
Time in days

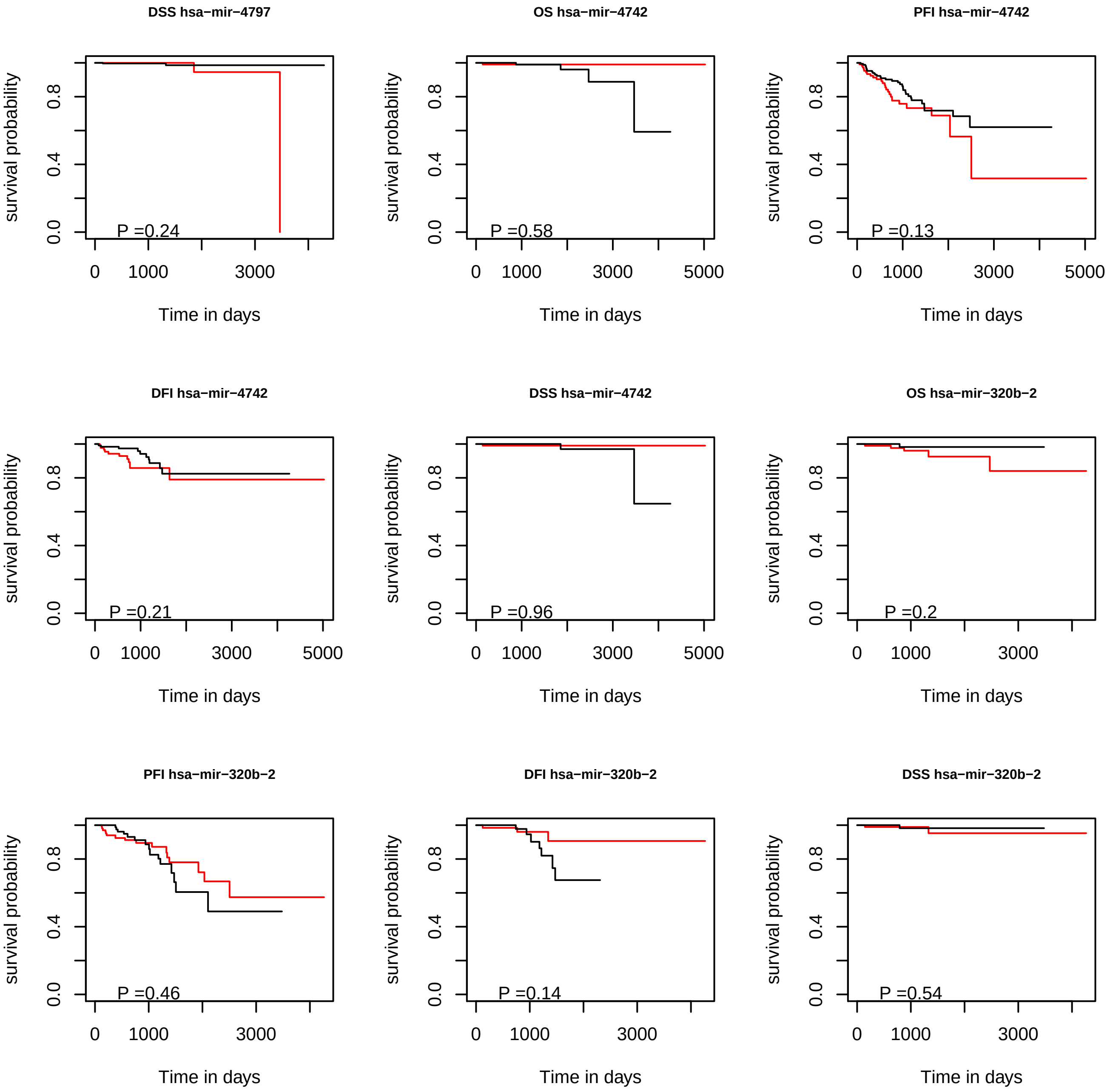
DSS hsa−mir−4797
P =0.24
OS hsa−mir−4742
P =0.58
PFI hsa−mir−4742
P =0.13
DFI hsa−mir−4742
P =0.21
DSS hsa−mir−4742
P =0.96
OS hsa−mir−320b−2
P =0.2
PFI hsa−mir−320b−2
P =0.46
DFI hsa−mir−320b−2
P =0.14
DSS hsa−mir−320b−2
P =0.54
survival probability
Time in days
0.0
0.4
0.8
0
1000
3000
5000

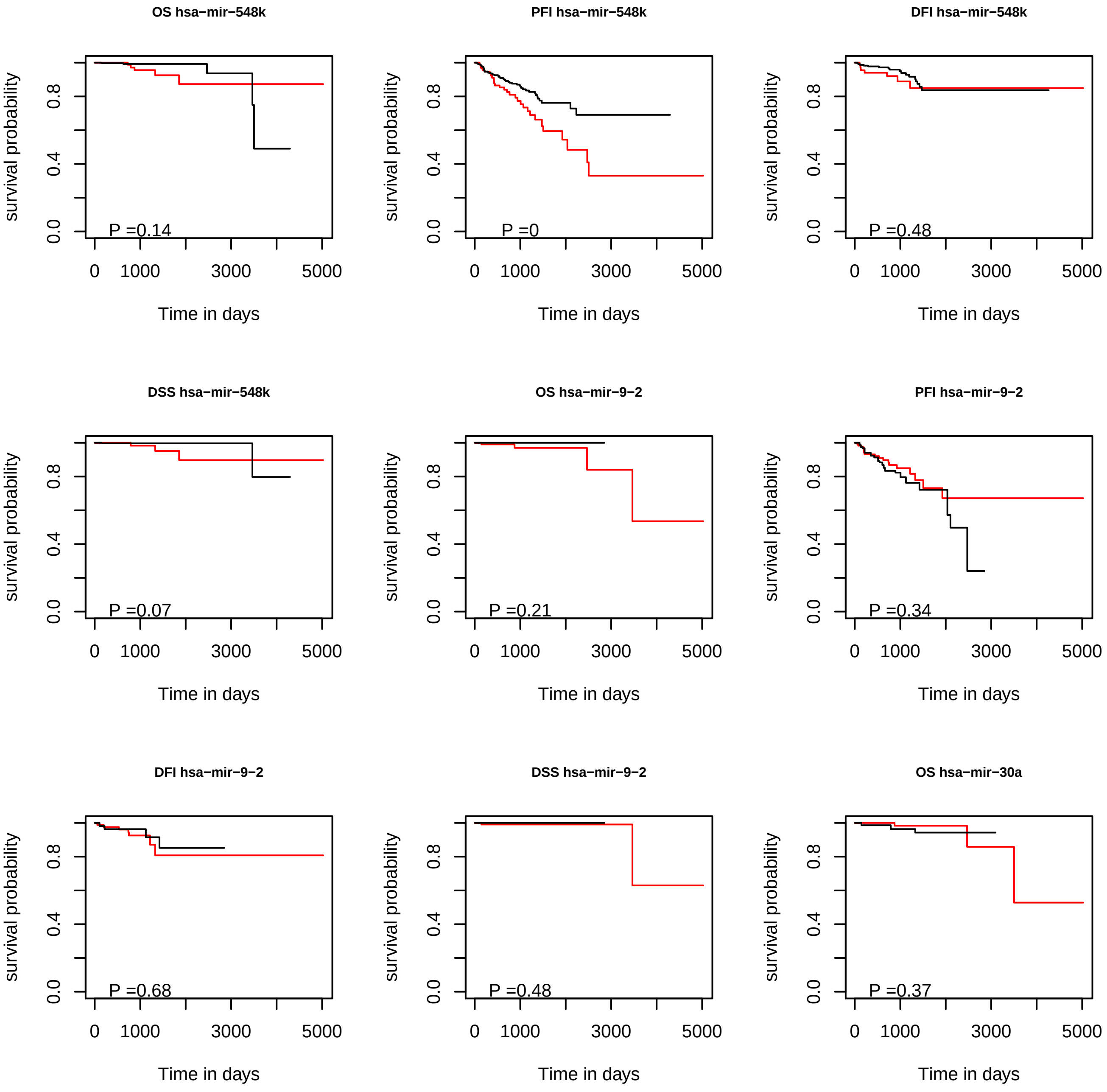

OS hsa−mir−548k
P =0.14
PFI hsa−mir−548k
P =0
DFI hsa−mir−548k
P =0.48
DSS hsa−mir−548k
P =0.07
OS hsa−mir−9−2
P =0.21
PFI hsa−mir−9−2
P =0.34
DFI hsa−mir−9−2
P =0.68
DSS hsa−mir−9−2
P =0.48
OS hsa−mir−30a
P =0.37
survival probability
Time in days
0.0
0.4
0.8
0
1000
3000
5000

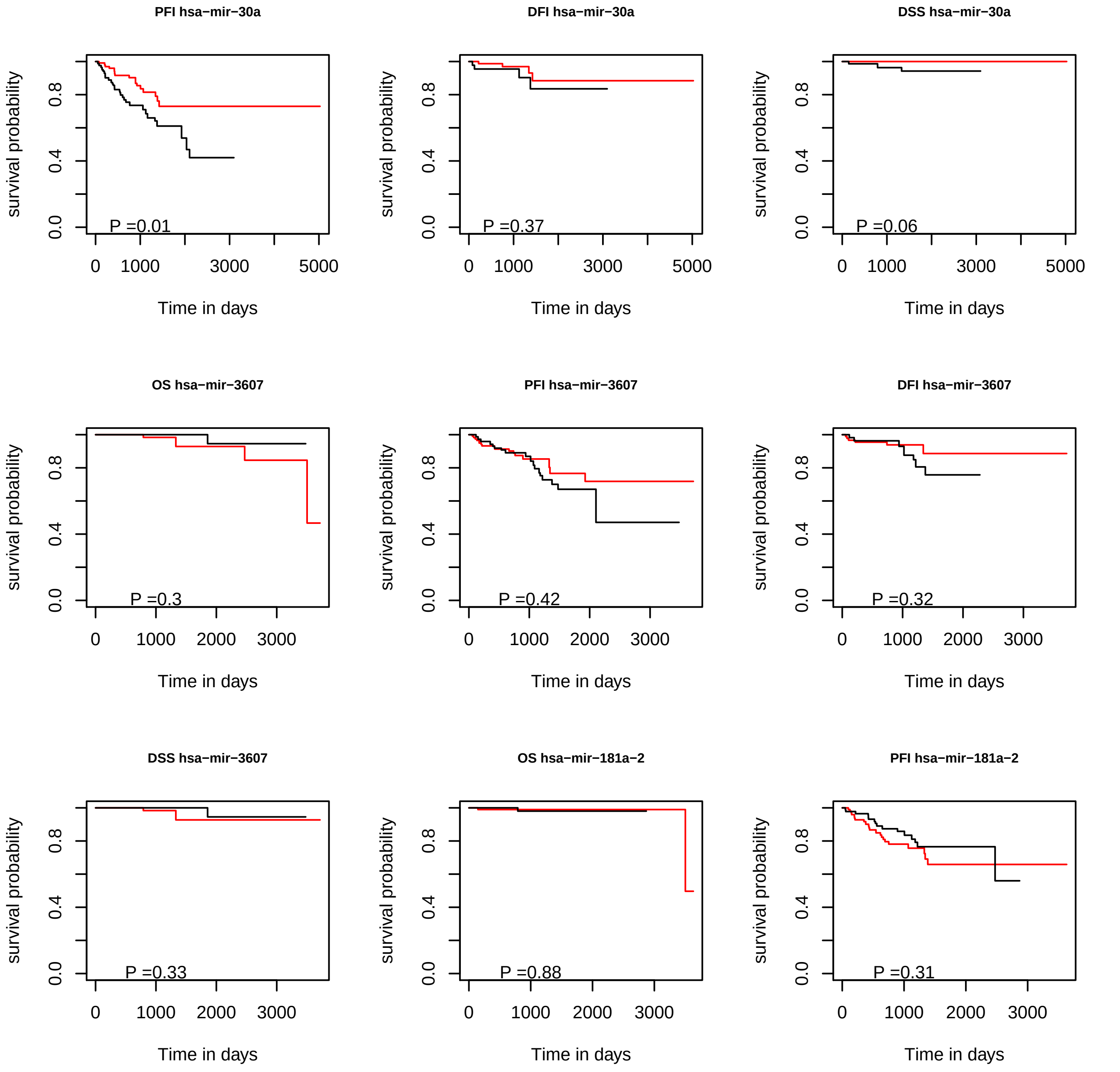
PFI hsa−mir−30a
P =0.01
DFI hsa−mir−30a
P =0.37
DSS hsa−mir−30a
P =0.06
OS hsa−mir−3607
P =0.3
PFI hsa−mir−3607
P =0.42
DFI hsa−mir−3607
P =0.32
DSS hsa−mir−3607
P =0.33
OS hsa−mir−181a−2
P =0.88
PFI hsa−mir−181a−2
P =0.31
survival probability
0.0
0.4
0.8
Time in days
0
1000
2000
3000
5000

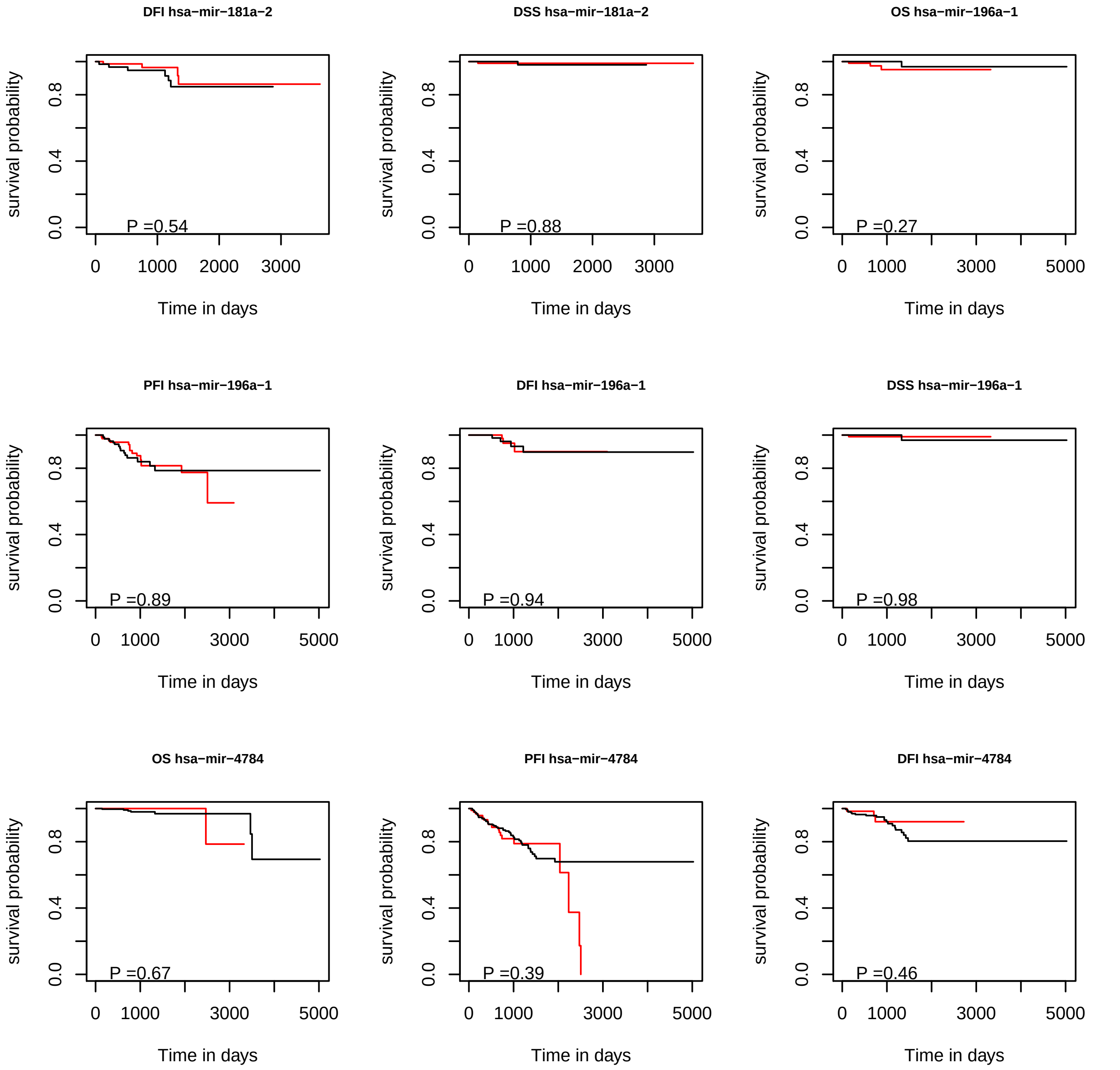
DFI hsa−mir−181a−2
P =0.54
DSS hsa−mir−181a−2
P =0.88
OS hsa−mir−196a−1
P =0.27
PFI hsa−mir−196a−1
P =0.89
DFI hsa−mir−196a−1
P =0.94
DSS hsa−mir−196a−1
P =0.98
OS hsa−mir−4784
P =0.67
PFI hsa−mir−4784
P =0.39
DFI hsa−mir−4784
P =0.46
survival probability
0.0
0.4
0.8
Time in days
0
1000
2000
3000
5000

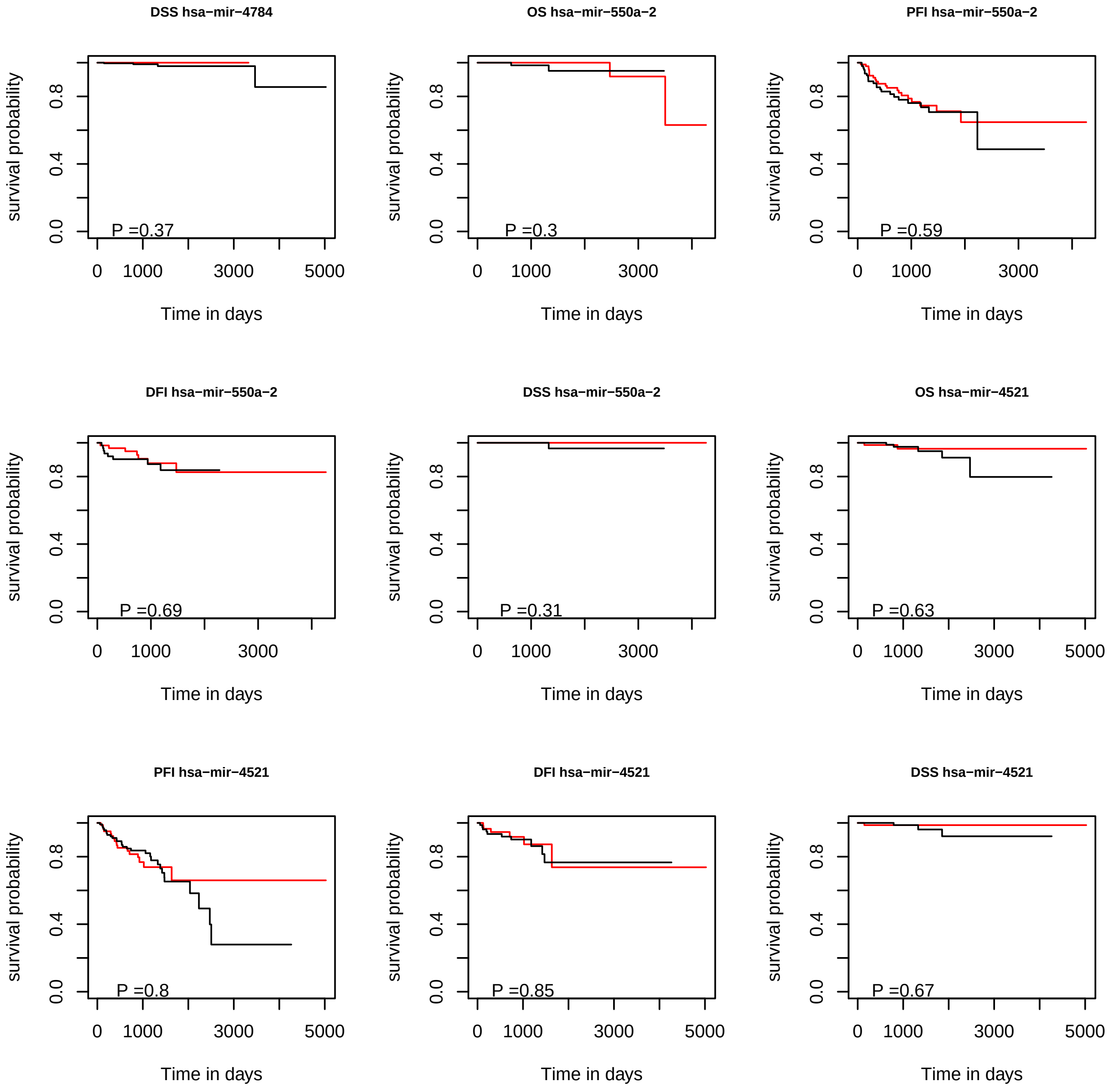
DSS hsa−mir−4784
P =0.37
OS hsa−mir−550a−2
P =0.3
PFI hsa−mir−550a−2
P =0.59
DFI hsa−mir−550a−2
P =0.69
DSS hsa−mir−550a−2
P =0.31
OS hsa−mir−4521
P =0.63
PFI hsa−mir−4521
P =0.8
DFI hsa−mir−4521
P =0.85
DSS hsa−mir−4521
P =0.67
survival probability
Time in days
0.0
0.4
0.8
0
1000
3000
5000

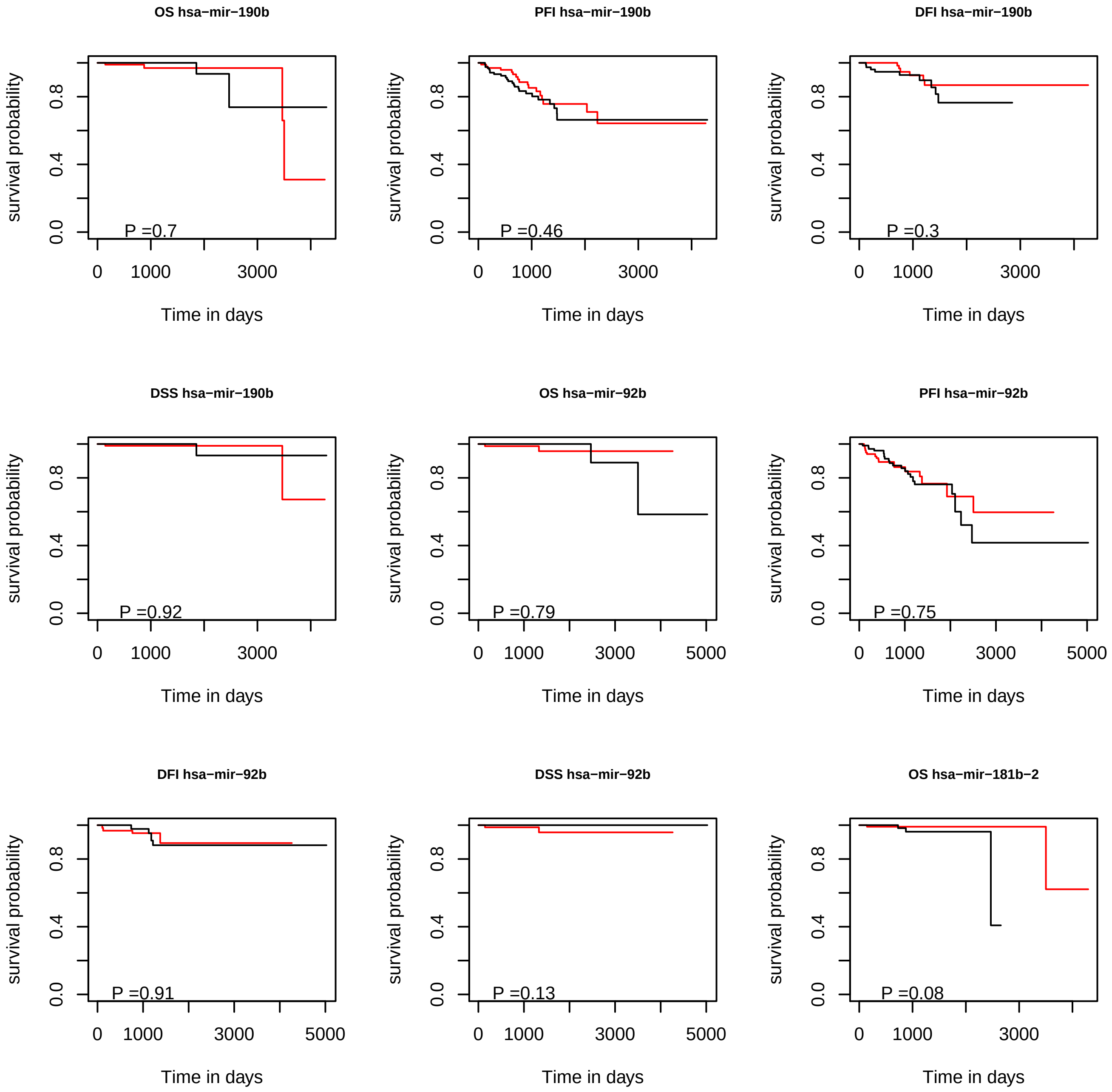
OS hsa−mir−190b
P =0.7
PFI hsa−mir−190b
P =0.46
DFI hsa−mir−190b
P =0.3
DSS hsa−mir−190b
P =0.92
OS hsa−mir−92b
P =0.79
PFI hsa−mir−92b
P =0.75
DFI hsa−mir−92b
P =0.91
DSS hsa−mir−92b
P =0.13
OS hsa−mir−181b−2
P =0.08
survival probability
0.0
0.4
0.8
Time in days
0
1000
3000
5000

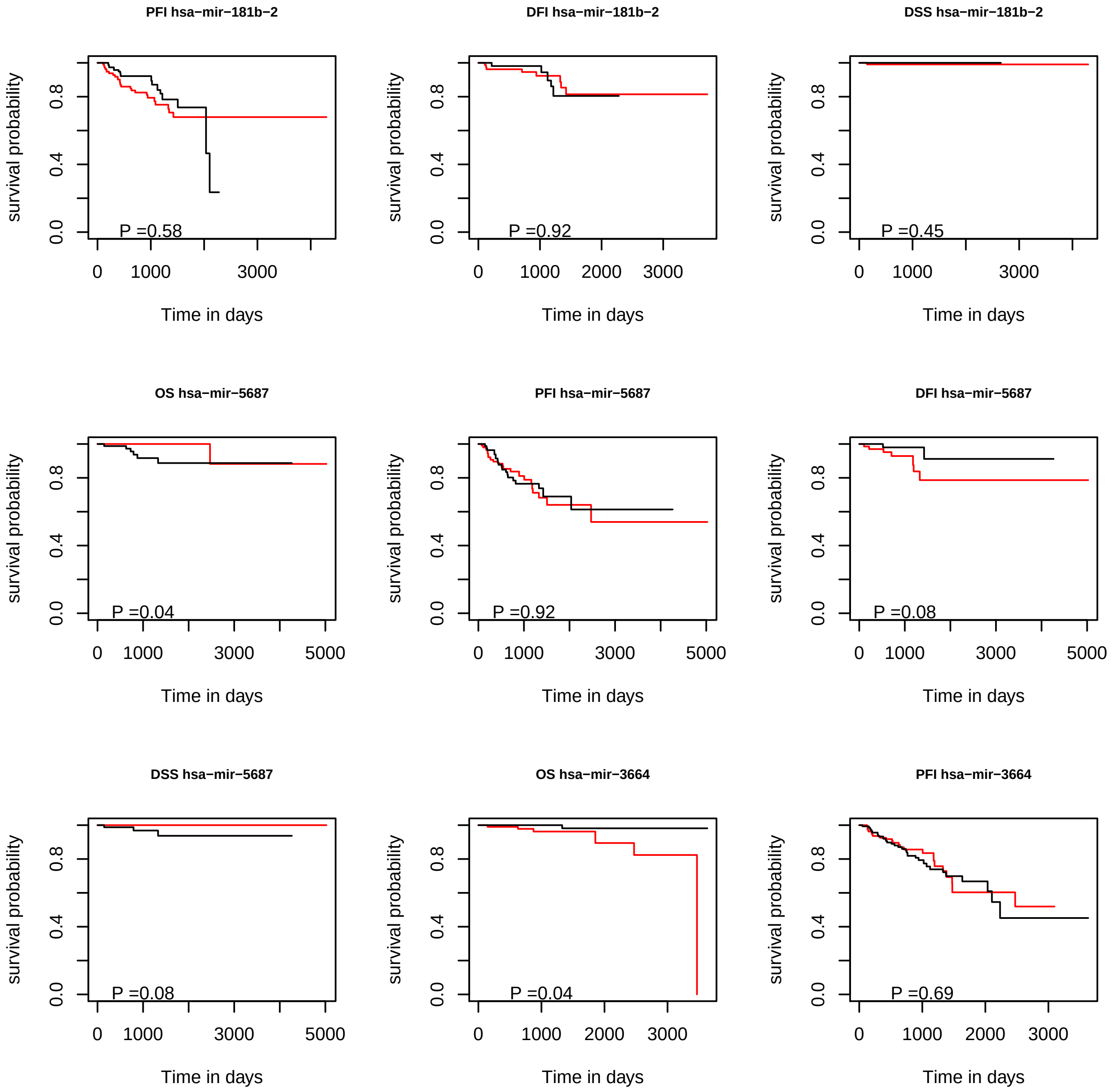
PFI hsa−mir−181b−2
P =0.58
DFI hsa−mir−181b−2
P =0.92
DSS hsa−mir−181b−2
P =0.45
OS hsa−mir−5687
P =0.04
PFI hsa−mir−5687
P =0.92
DFI hsa−mir−5687
P =0.08
DSS hsa−mir−5687
P =0.08
OS hsa−mir−3664
P =0.04
PFI hsa−mir−3664
P =0.69
survival probability
Time in days
0.0
0.4
0.8
0
1000
2000
3000
5000

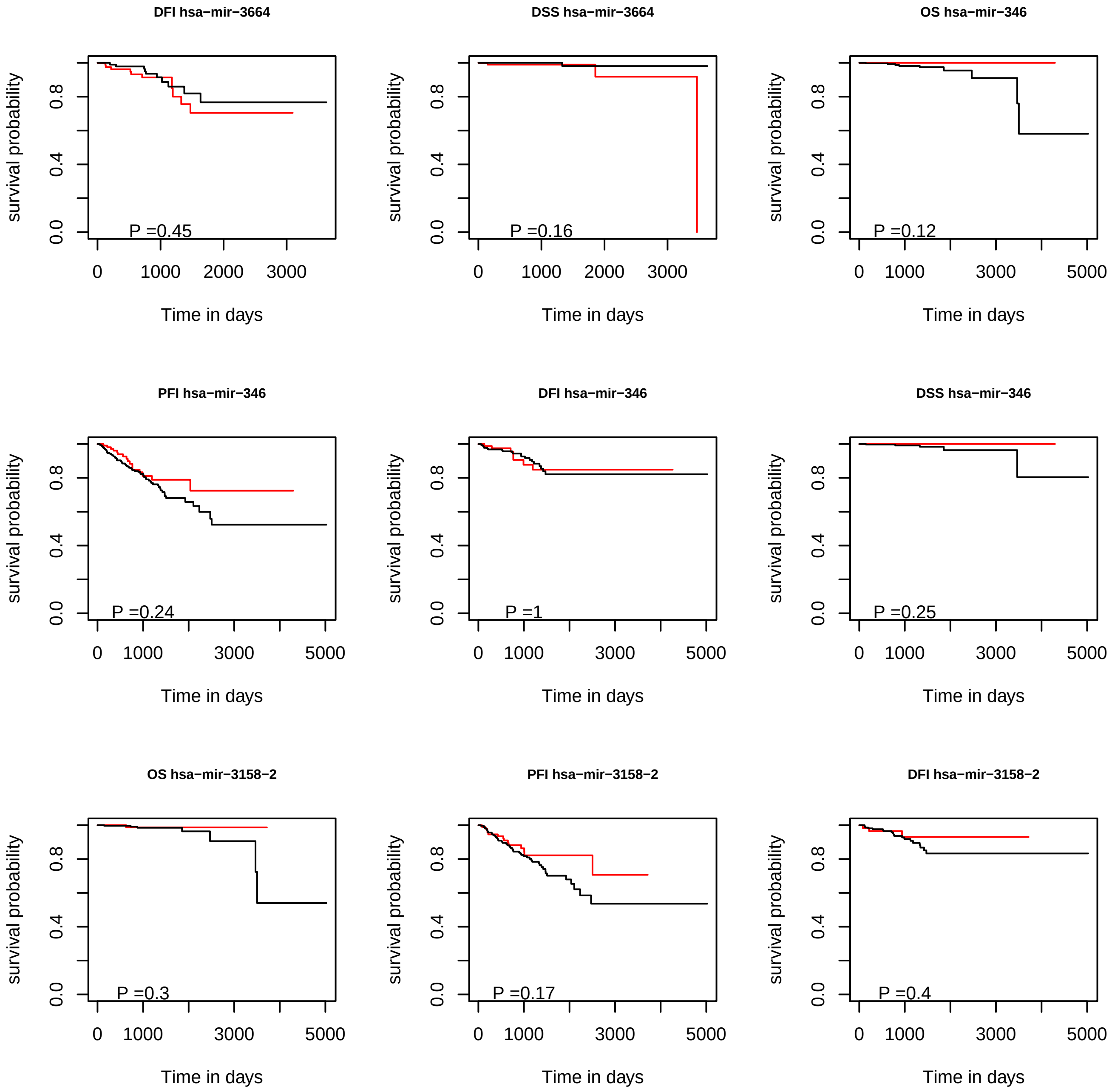
DFI hsa−mir−3664
P =0.45
DSS hsa−mir−3664
P =0.16
OS hsa−mir−346
P =0.12
PFI hsa−mir−346
P =0.24
DFI hsa−mir−346
P =1
DSS hsa−mir−346
P =0.25
OS hsa−mir−3158−2
P =0.3
PFI hsa−mir−3158−2
P =0.17
DFI hsa−mir−3158−2
P =0.4
survival probability
Time in days

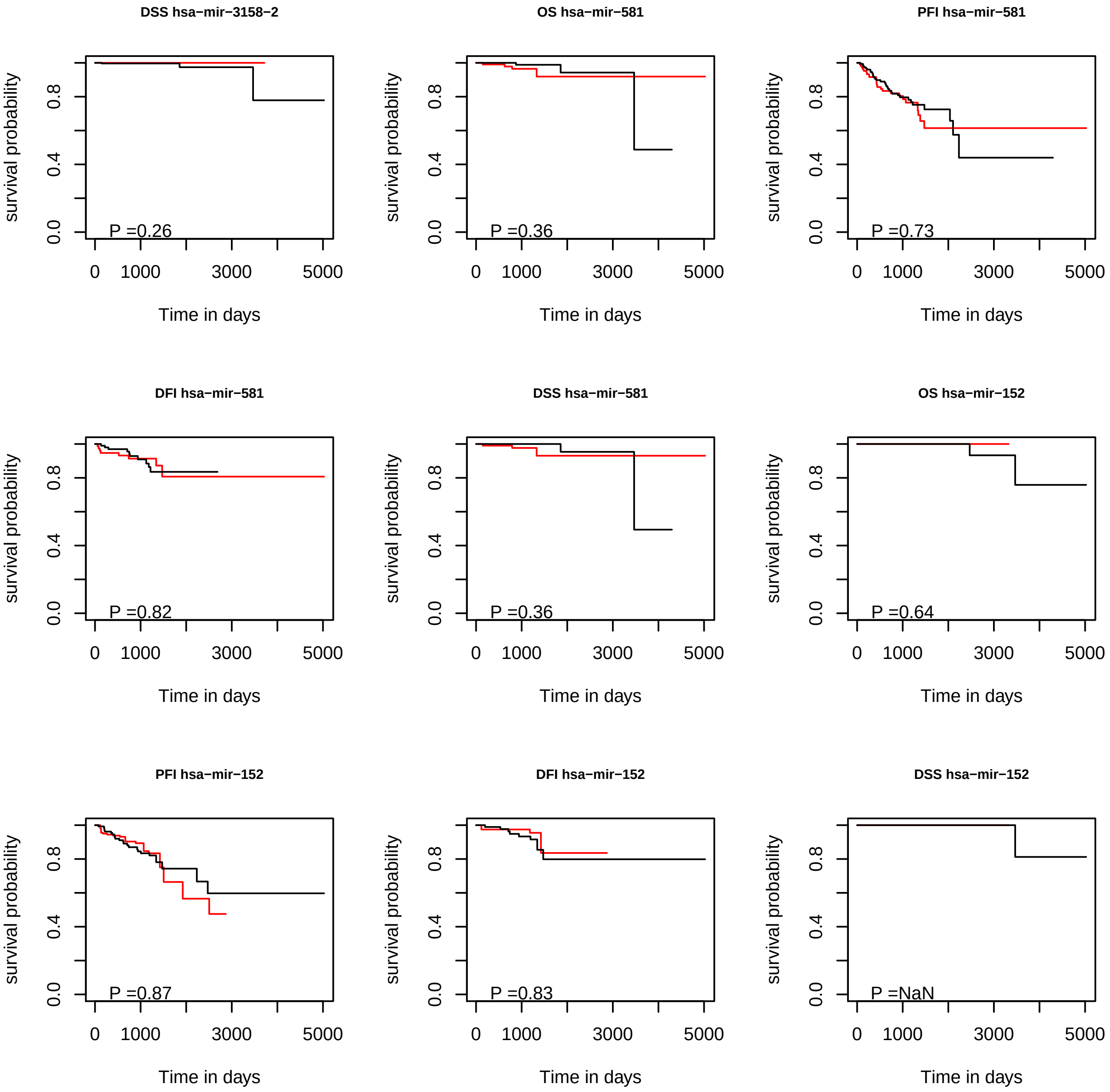
DSS hsa−mir−3158−2
P =0.26
OS hsa−mir−581
P =0.36
PFI hsa−mir−581
P =0.73
DFI hsa−mir−581
P =0.82
DSS hsa−mir−581
P =0.36
OS hsa−mir−152
P =0.64
PFI hsa−mir−152
P =0.87
DFI hsa−mir−152
P =0.83
DSS hsa−mir−152
P =NaN
survival probability
0.0
0.4
0.8
Time in days
0
1000
3000
5000

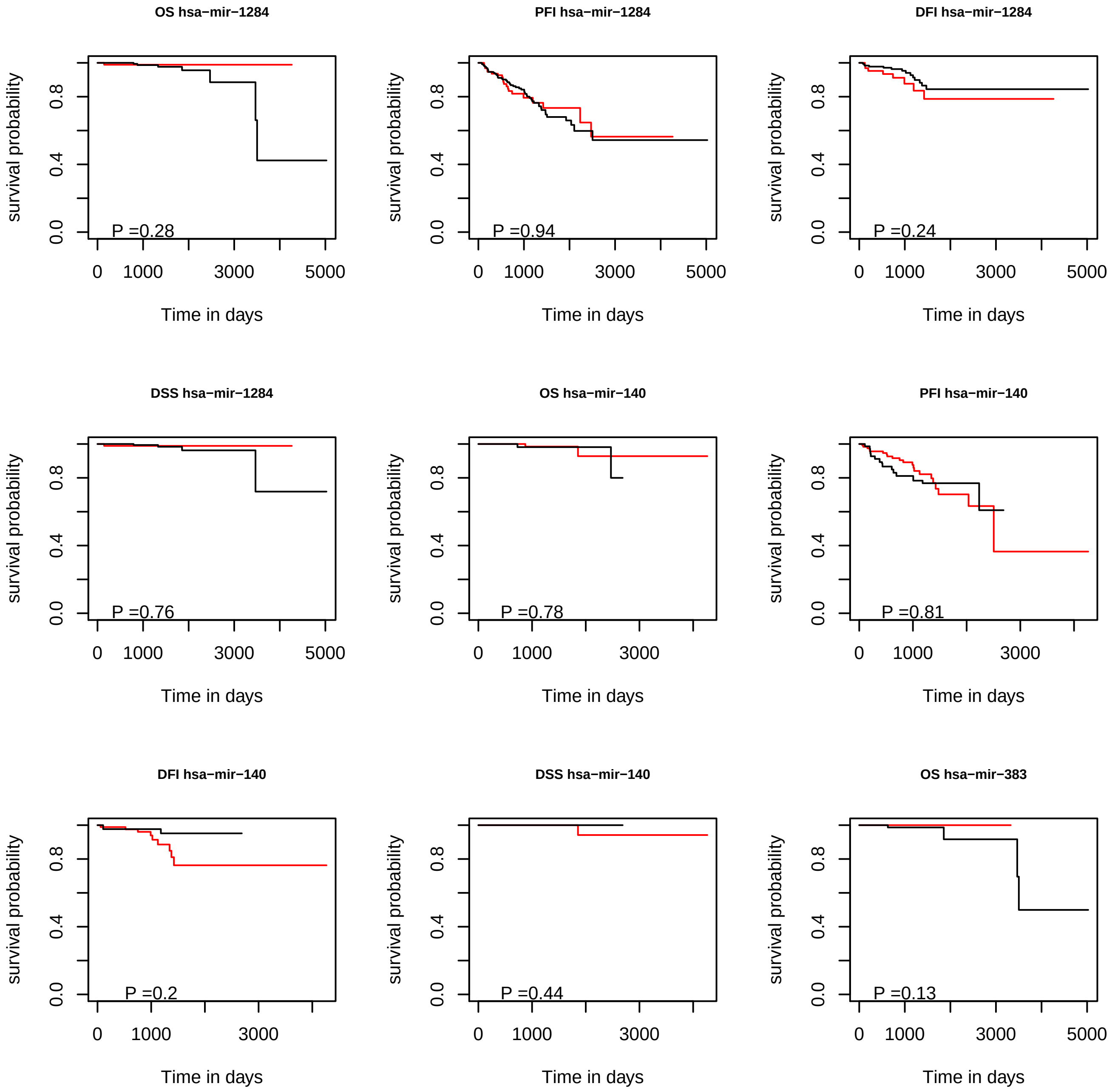
OS hsa−mir−1284
P =0.28
PFI hsa−mir−1284
P =0.94
DFI hsa−mir−1284
P =0.24
DSS hsa−mir−1284
P =0.76
OS hsa−mir−140
P =0.78
PFI hsa−mir−140
P =0.81
DFI hsa−mir−140
P =0.2
DSS hsa−mir−140
P =0.44
OS hsa−mir−383
P =0.13
survival probability
0.0
0.4
0.8
Time in days
0
1000
3000
5000

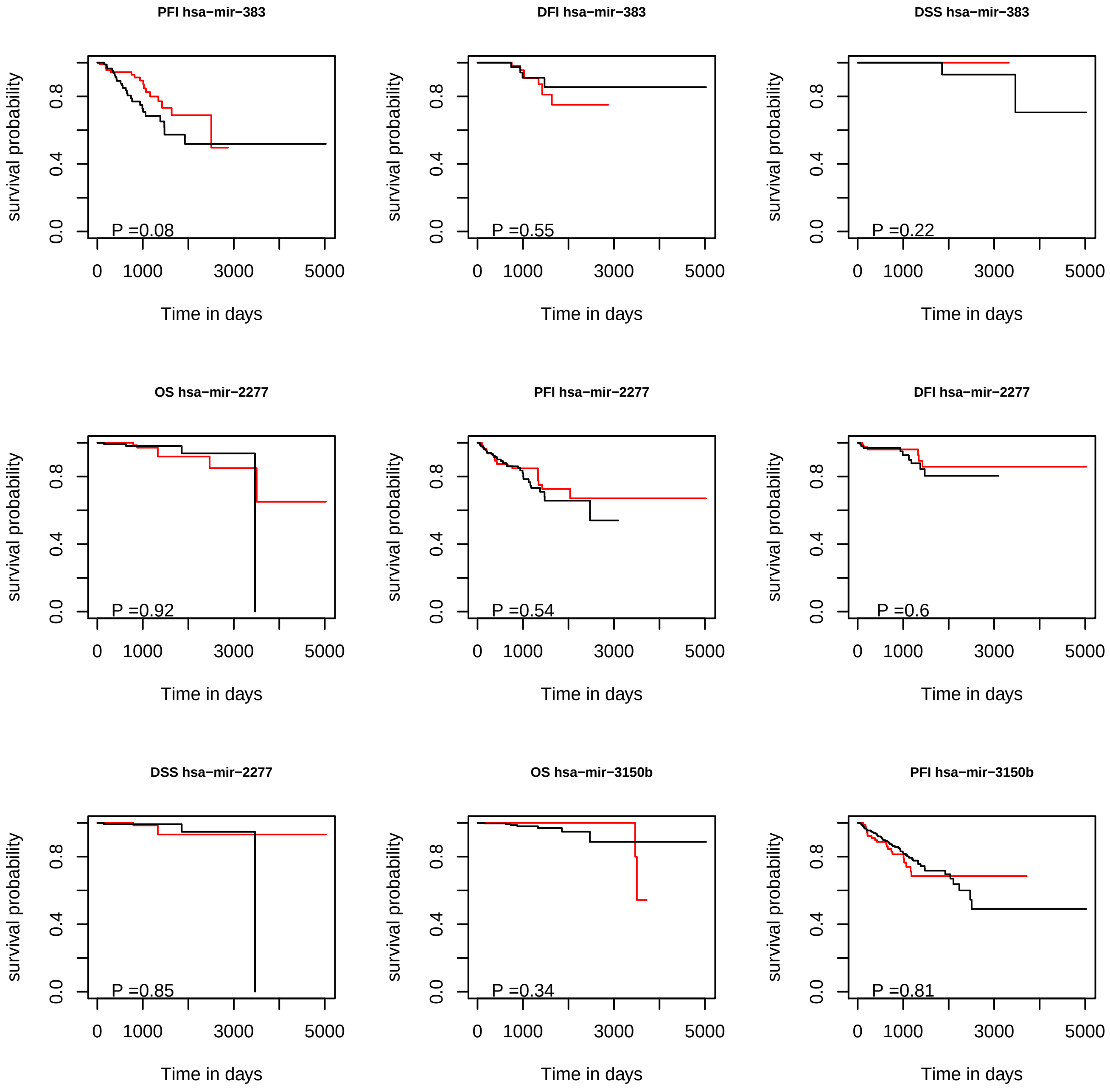
PFI hsa−mir−383
P =0.08
DFI hsa−mir−383
P =0.55
DSS hsa−mir−383
P =0.22
OS hsa−mir−2277
P =0.92
PFI hsa−mir−2277
P =0.54
DFI hsa−mir−2277
P =0.6
DSS hsa−mir−2277
P =0.85
OS hsa−mir−3150b
P =0.34
PFI hsa−mir−3150b
P =0.81
survival probability
0.0
0.4
0.8
Time in days
0
1000
3000
5000

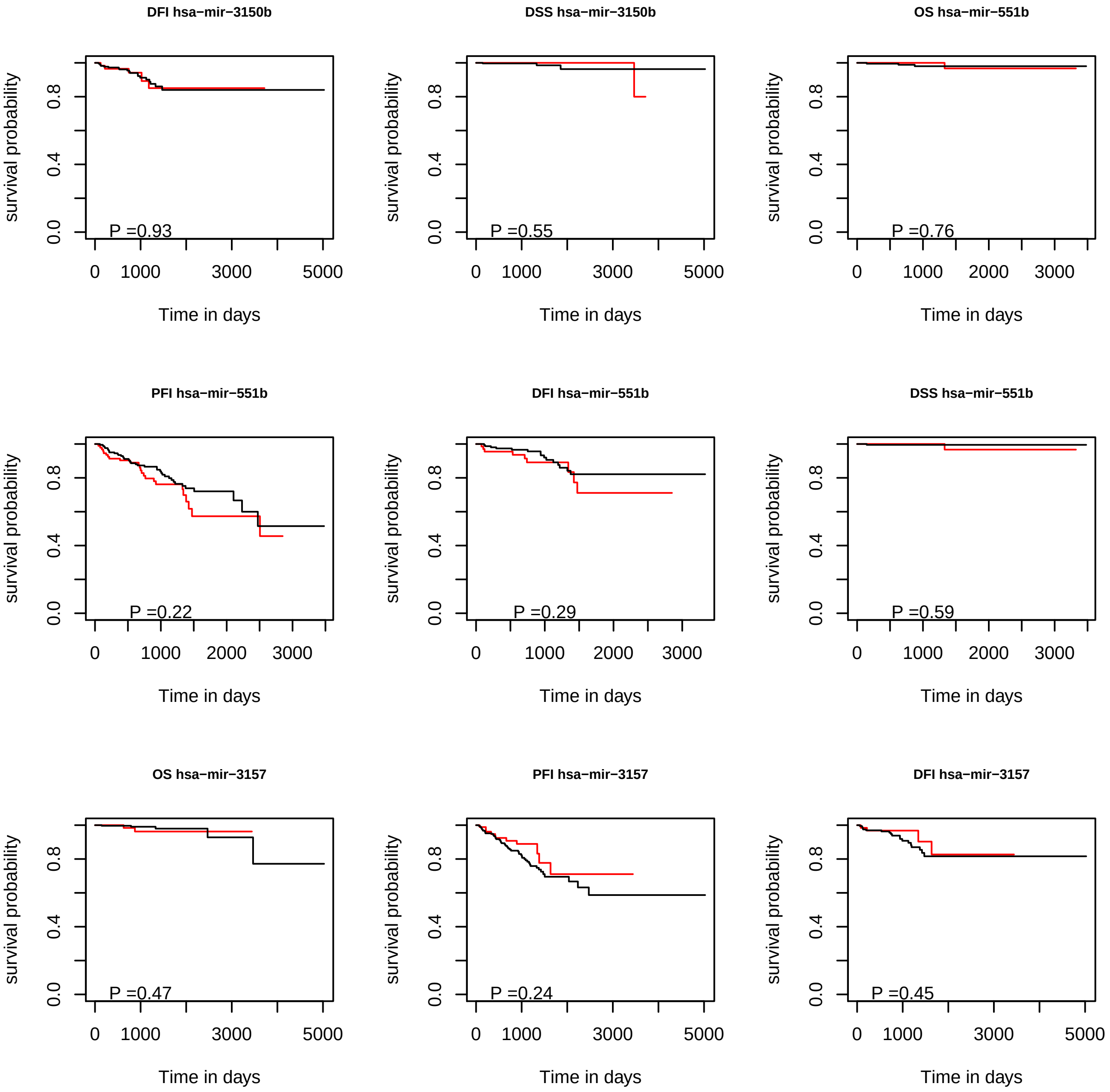
DFI hsa−mir−3150b
P =0.93
DSS hsa−mir−3150b
P =0.55
OS hsa−mir−551b
P =0.76
PFI hsa−mir−551b
P =0.22
DFI hsa−mir−551b
P =0.29
DSS hsa−mir−551b
P =0.59
OS hsa−mir−3157
P =0.47
PFI hsa−mir−3157
P =0.24
DFI hsa−mir−3157
P =0.45
survival probability
Time in days

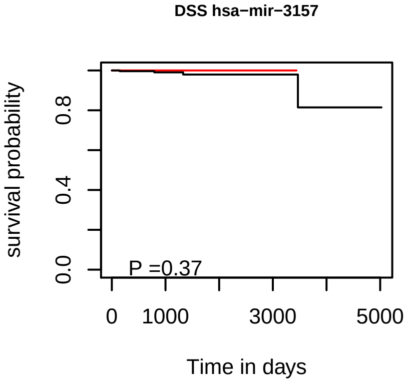
DSS hsa−mir−3157
survival probability
0.0
0.4
0.8
P =0.37
0
1000
3000
5000
Time in days
