# Supplementary material for: Computing microRNA-gene interaction networks in pan-cancer using miRDriver
Source: Sci Rep. 2022 Mar 8;12:3717. doi: 10.1038/s41598-022-07628-z (PMC8904490; doi:10.1038/s41598-022-07628-z)
Supplement: Supplementary file 27 — Supplementary Information 27. [file 41598_2022_7628_MOESM27_ESM.pdf]

Computing microRNA-gene interaction networks in pan-cancer using miRDriver

Banabithi Bose, Matthew Moravec, and Serdar Bozdag

Supplemental Figure S18

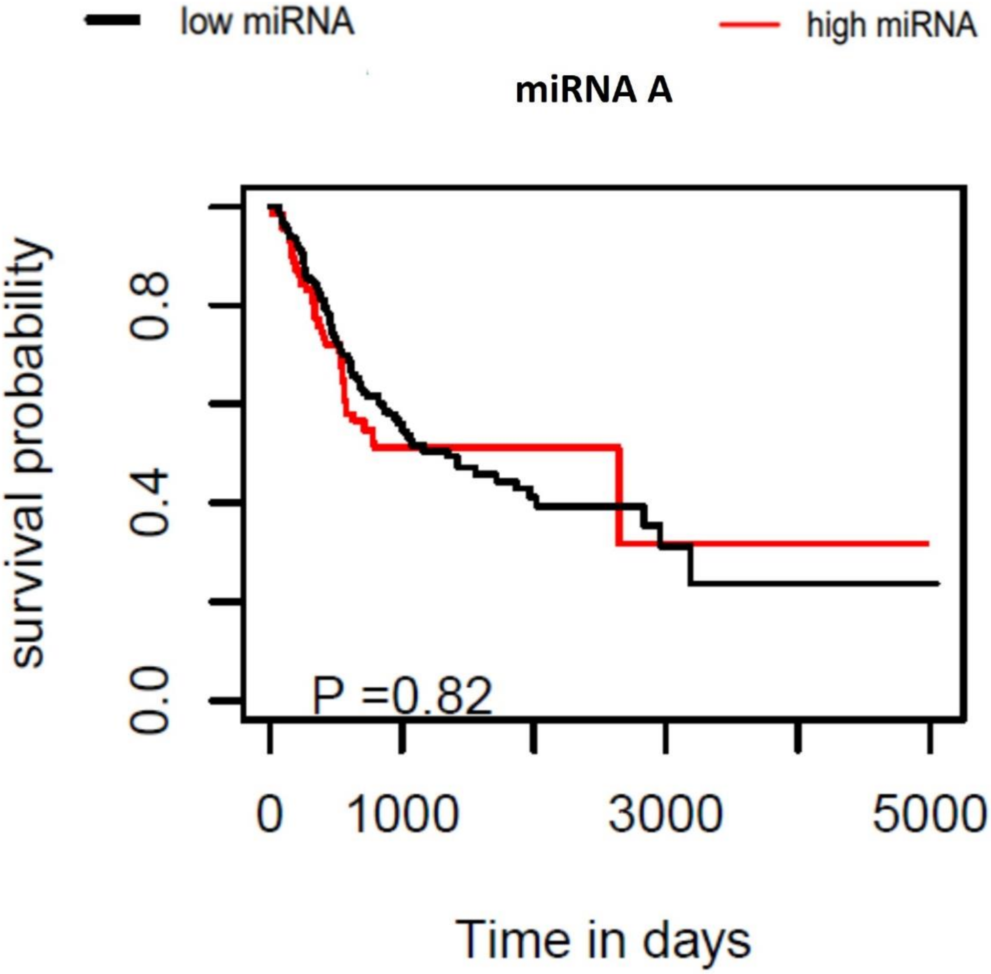

The *Adjusted Kaplan-Meier* survival plots for the computed miRNAs in high and low miRNA expression patient groups.

Supplemental Figure S18

Cancer Type: UCEC

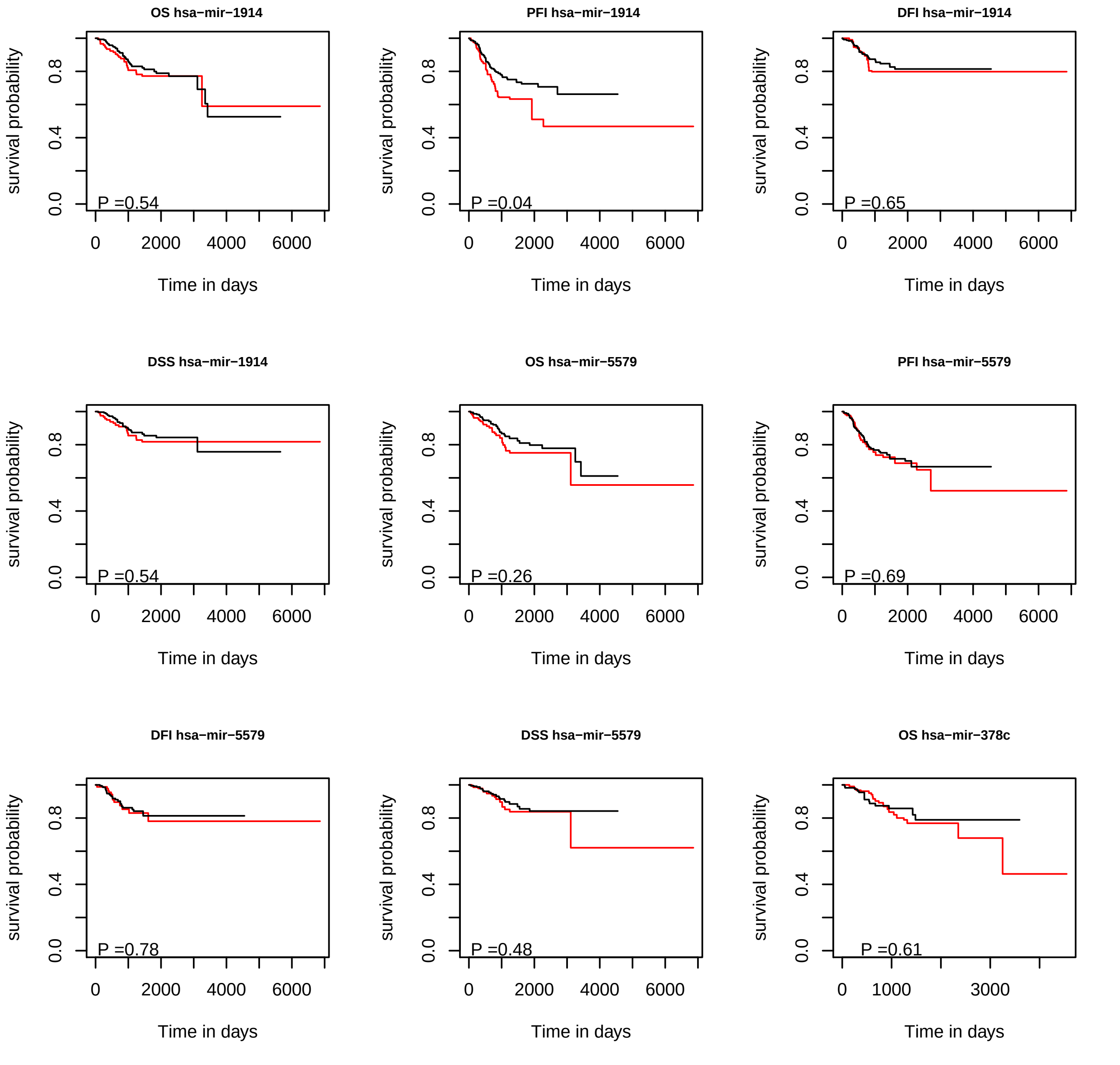

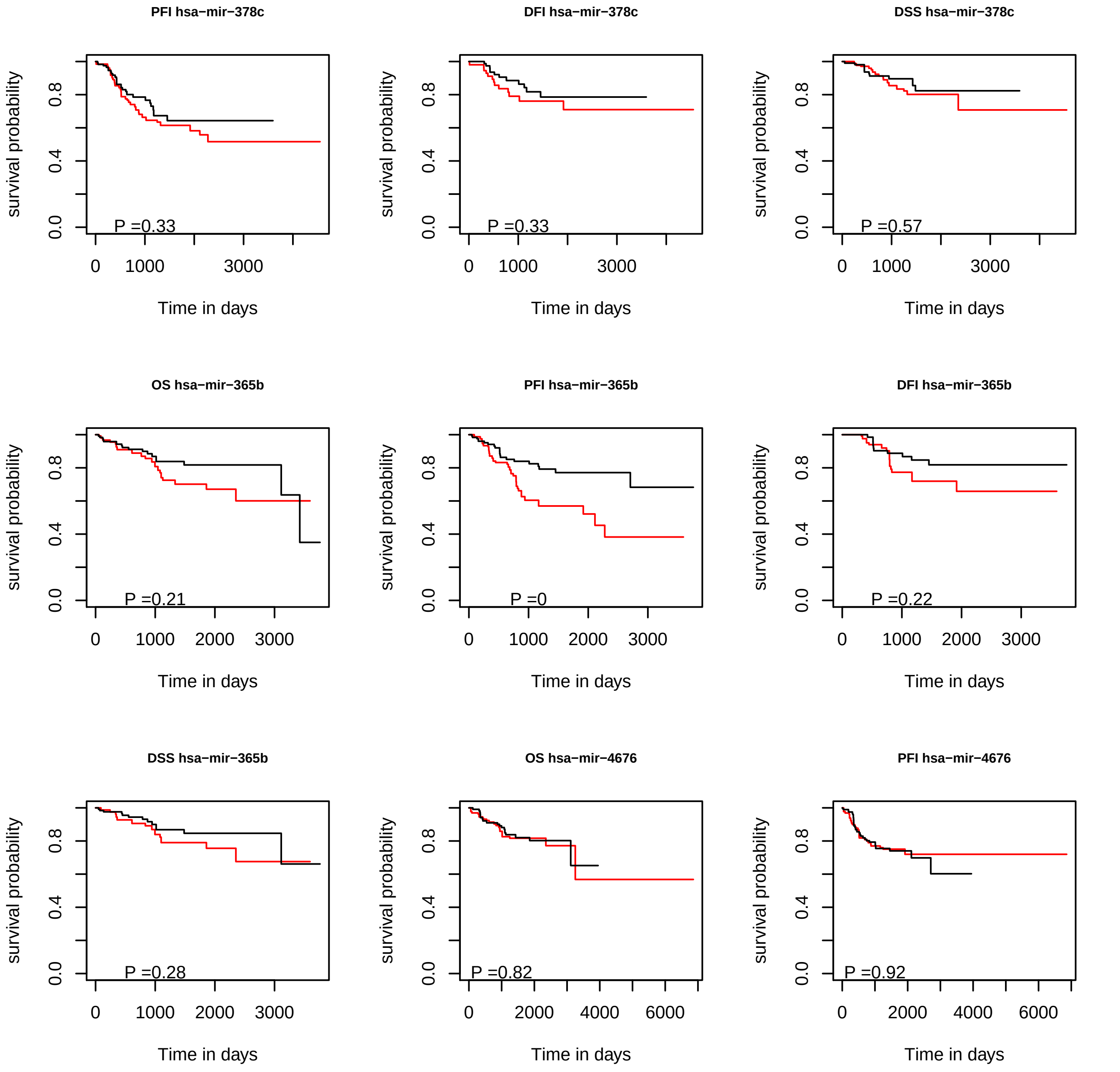

PFI hsa−mir−378c
DFI hsa−mir−378c
DSS hsa−mir−378c
OS hsa−mir−365b
PFI hsa−mir−365b
DFI hsa−mir−365b
DSS hsa−mir−365b
OS hsa−mir−4676
PFI hsa−mir−4676
survival probability
Time in days
P =0.33
P =0.33
P =0.57
P =0.21
P =0
P =0.22
P =0.28
P =0.82
P =0.92
0.0
0.4
0.8
0
1000
2000
3000
4000
6000

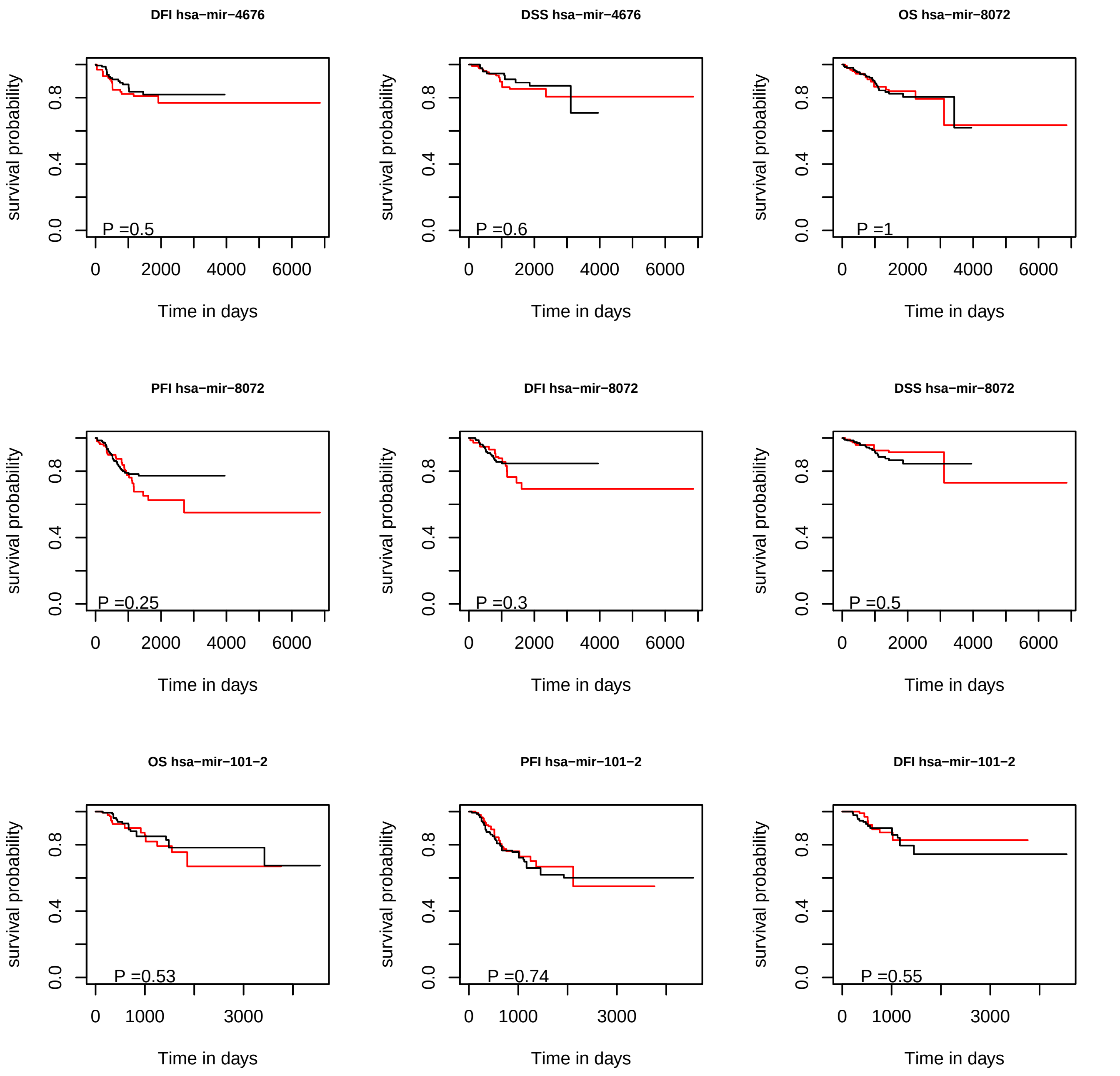
DFI hsa−mir−4676
P =0.5
DSS hsa−mir−4676
P =0.6
OS hsa−mir−8072
P =1
PFI hsa−mir−8072
P =0.25
DFI hsa−mir−8072
P =0.3
DSS hsa−mir−8072
P =0.5
OS hsa−mir−101−2
P =0.53
PFI hsa−mir−101−2
P =0.74
DFI hsa−mir−101−2
P =0.55
survival probability
Time in days
0.0
0.4
0.8
0
1000
2000
3000
4000
6000

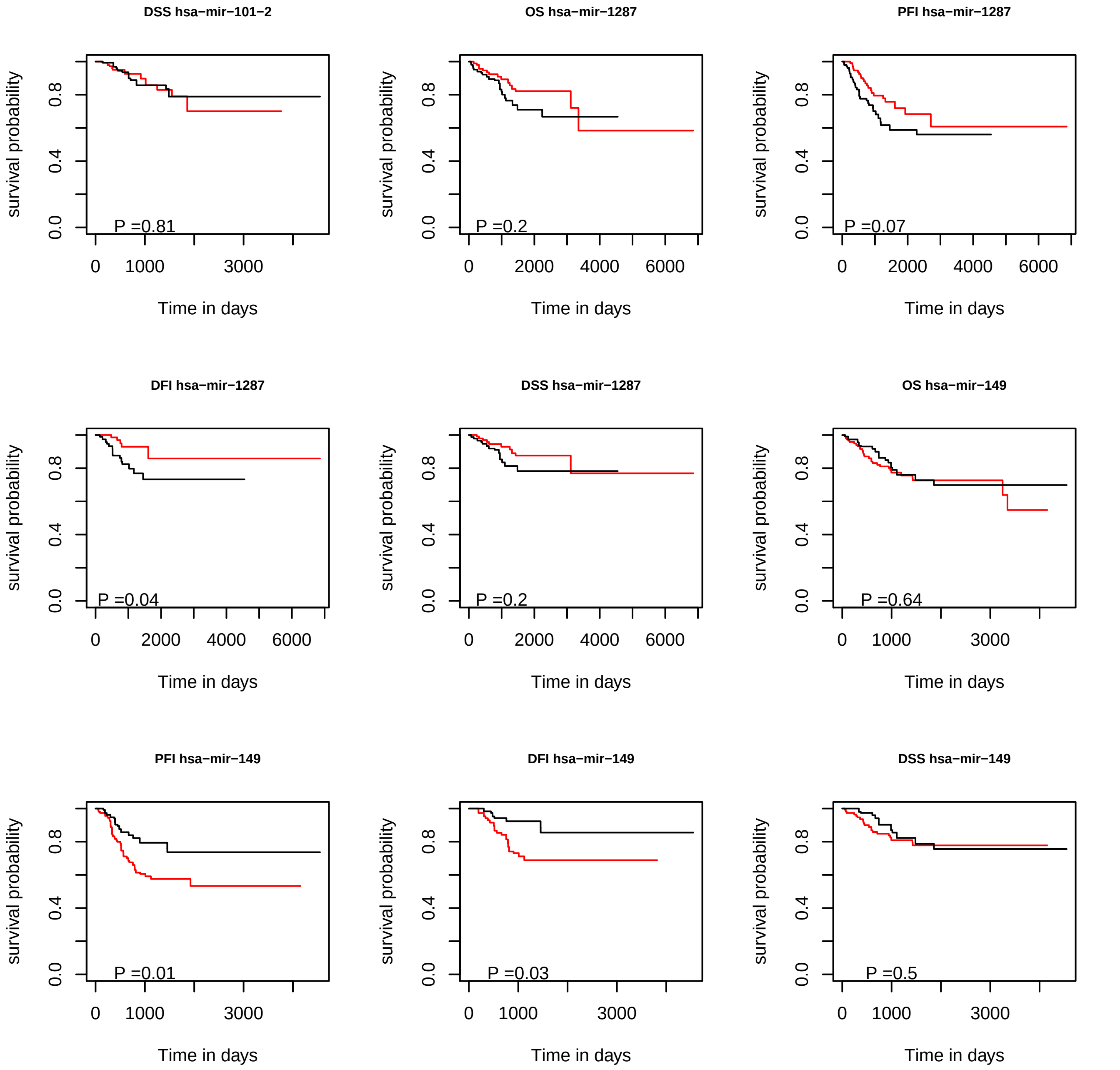

DSS hsa−mir−101−2
P =0.81
OS hsa−mir−1287
P =0.2
PFI hsa−mir−1287
P =0.07
DFI hsa−mir−1287
P =0.04
DSS hsa−mir−1287
P =0.2
OS hsa−mir−149
P =0.64
PFI hsa−mir−149
P =0.01
DFI hsa−mir−149
P =0.03
DSS hsa−mir−149
P =0.5
survival probability
Time in days
0.0
0.4
0.8
0
1000
2000
3000
4000
6000

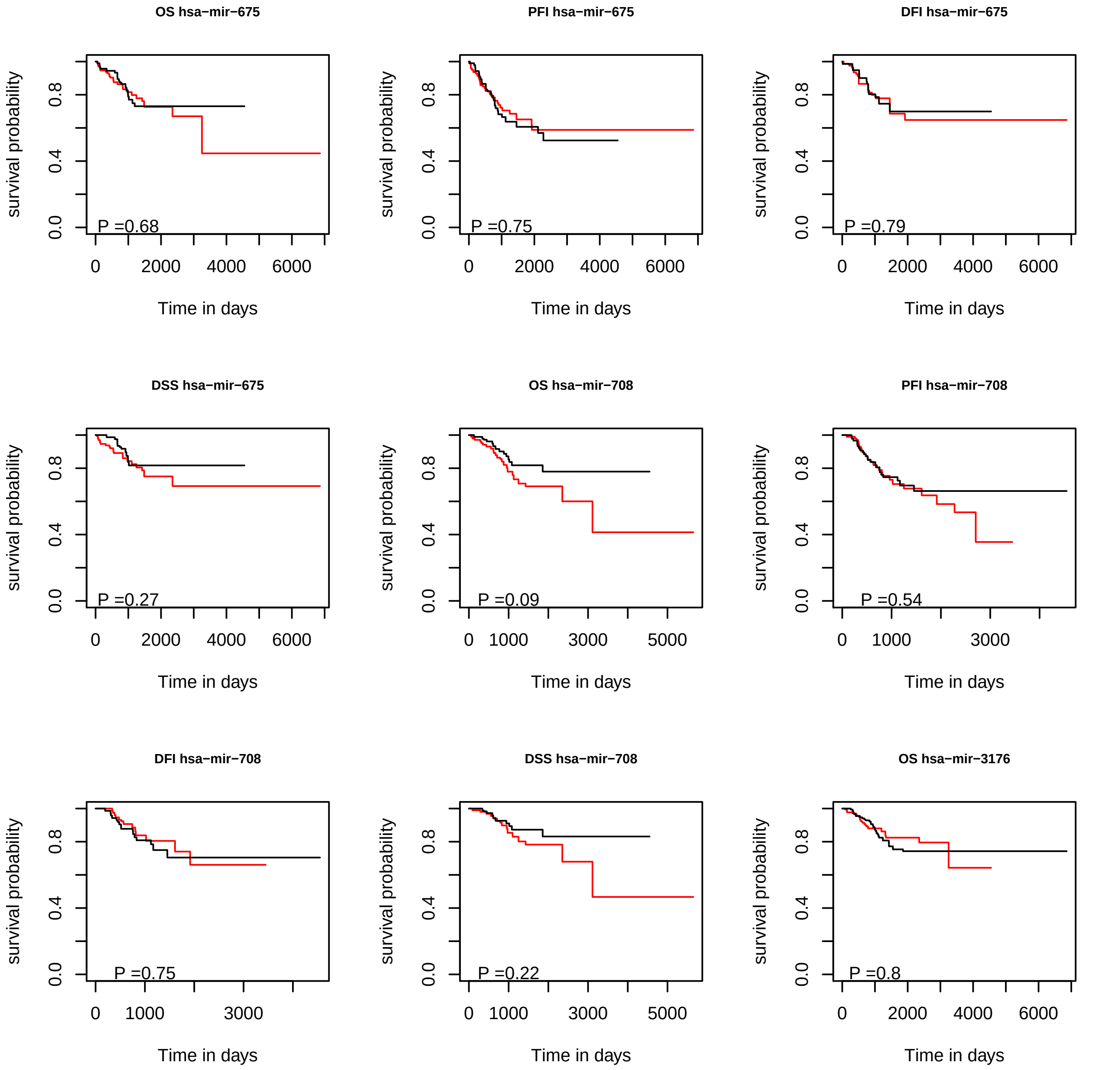
OS hsa−mir−675
P =0.68
PFI hsa−mir−675
P =0.75
DFI hsa−mir−675
P =0.79
DSS hsa−mir−675
P =0.27
OS hsa−mir−708
P =0.09
PFI hsa−mir−708
P =0.54
DFI hsa−mir−708
P =0.75
DSS hsa−mir−708
P =0.22
OS hsa−mir−3176
P =0.8
survival probability
Time in days
0.0
0.4
0.8
0
1000
2000
3000
4000
5000
6000

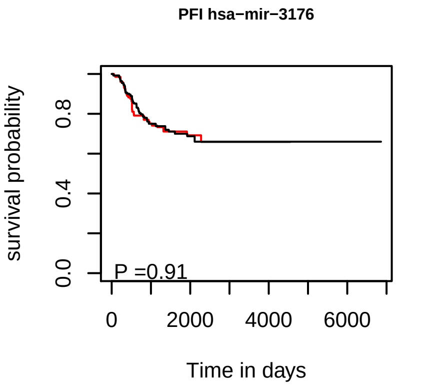
PFI hsa−mir−3176
survival probability
0.0 0.4 0.8
P =0.91
0 2000 4000 6000
Time in days
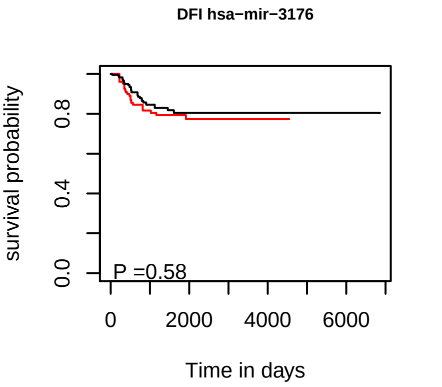
DFI hsa−mir−3176
survival probability
0.0 0.4 0.8
P =0.58
0 2000 4000 6000
Time in days
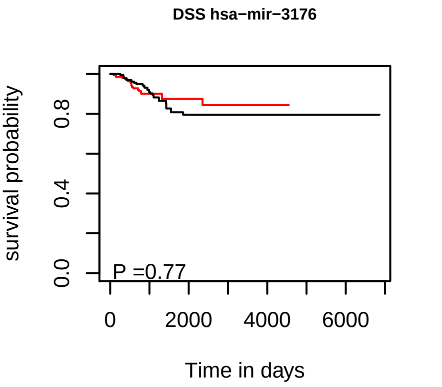
DSS hsa−mir−3176
survival probability
0.0 0.4 0.8
P =0.77
0 2000 4000 6000
Time in days
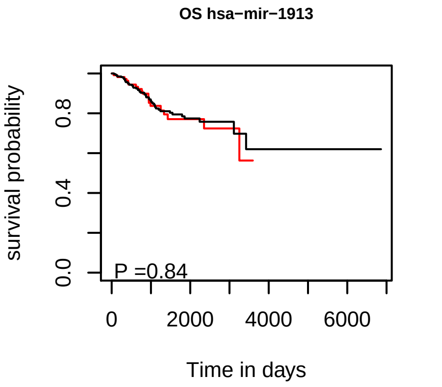
OS hsa−mir−1913
survival probability
0.0 0.4 0.8
P =0.84
0 2000 4000 6000
Time in days
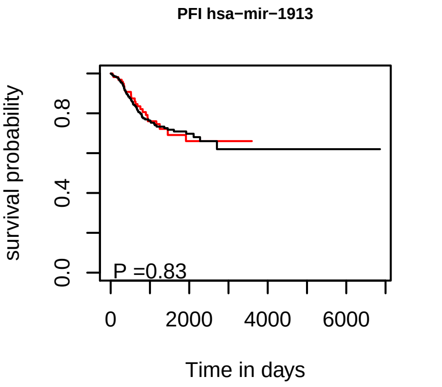
PFI hsa−mir−1913
survival probability
0.0 0.4 0.8
P =0.83
0 2000 4000 6000
Time in days
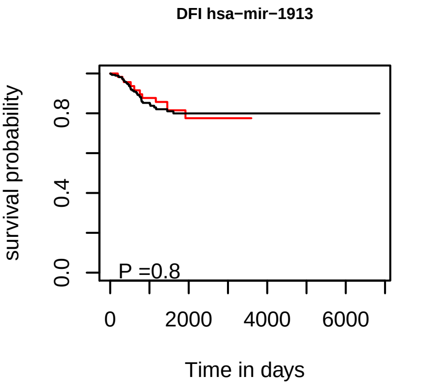
DFI hsa−mir−1913
survival probability
0.0 0.4 0.8
P =0.8
0 2000 4000 6000
Time in days
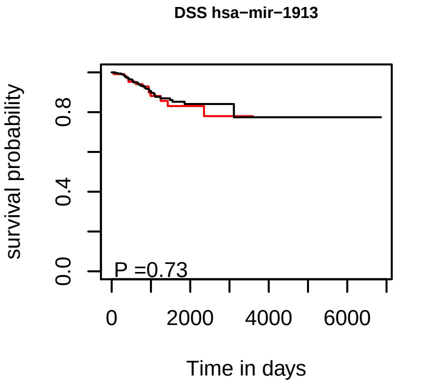
DSS hsa−mir−1913
survival probability
0.0 0.4 0.8
P =0.73
0 2000 4000 6000
Time in days
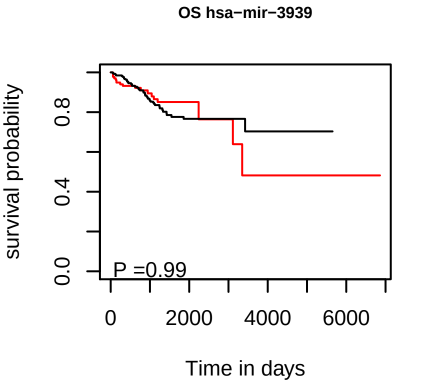
OS hsa−mir−3939
survival probability
0.0 0.4 0.8
P =0.99
0 2000 4000 6000
Time in days
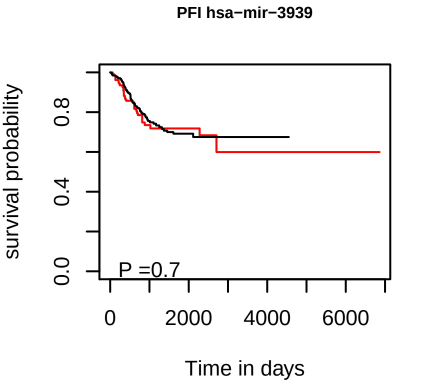
PFI hsa−mir−3939
survival probability
0.0 0.4 0.8
P =0.7
0 2000 4000 6000
Time in days

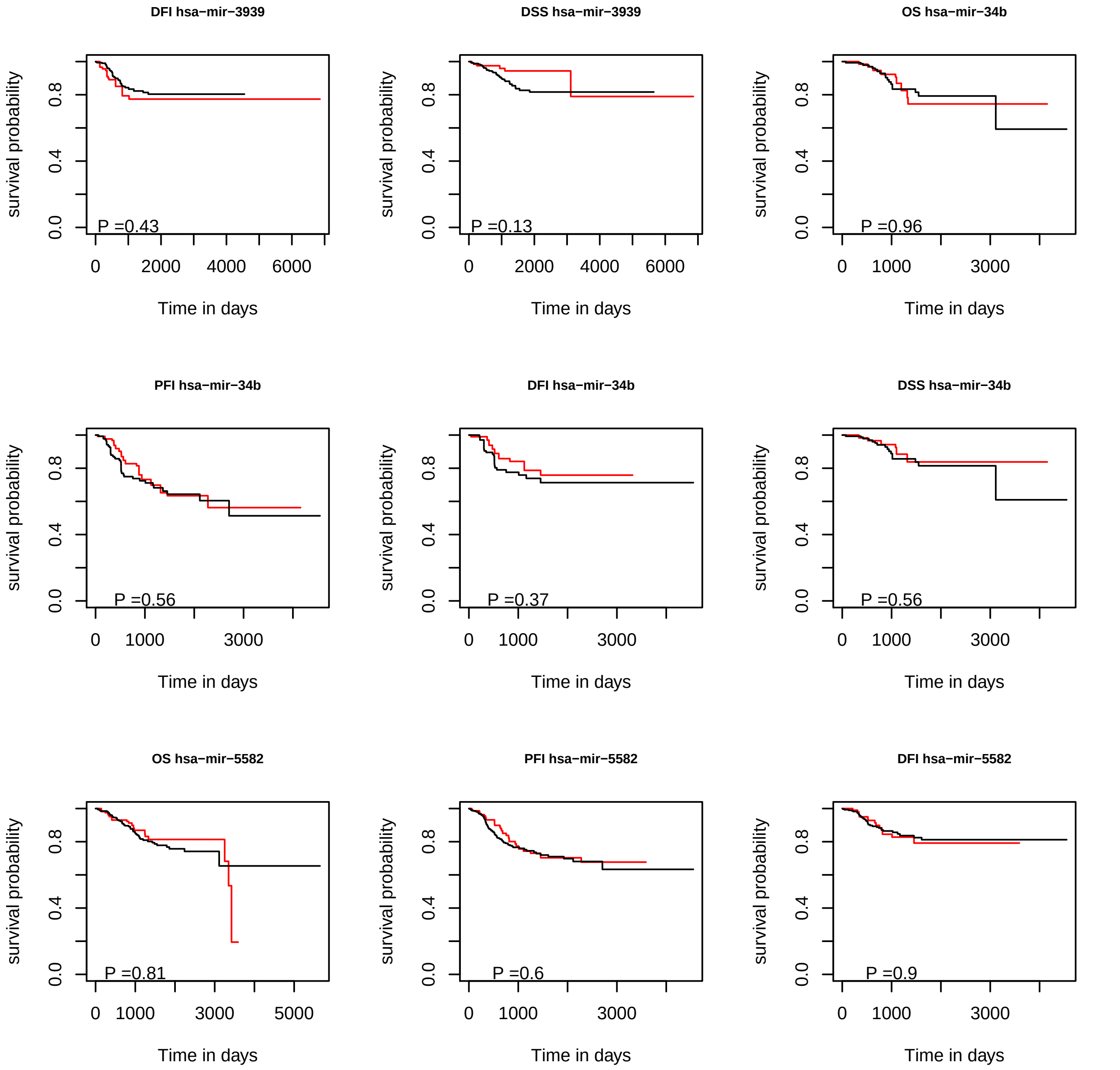
DFI hsa−mir−3939
P =0.43
DSS hsa−mir−3939
P =0.13
OS hsa−mir−34b
P =0.96
PFI hsa−mir−34b
P =0.56
DFI hsa−mir−34b
P =0.37
DSS hsa−mir−34b
P =0.56
OS hsa−mir−5582
P =0.81
PFI hsa−mir−5582
P =0.6
DFI hsa−mir−5582
P =0.9
survival probability
Time in days
0.0
0.4
0.8
0
1000
2000
3000
4000
5000
6000

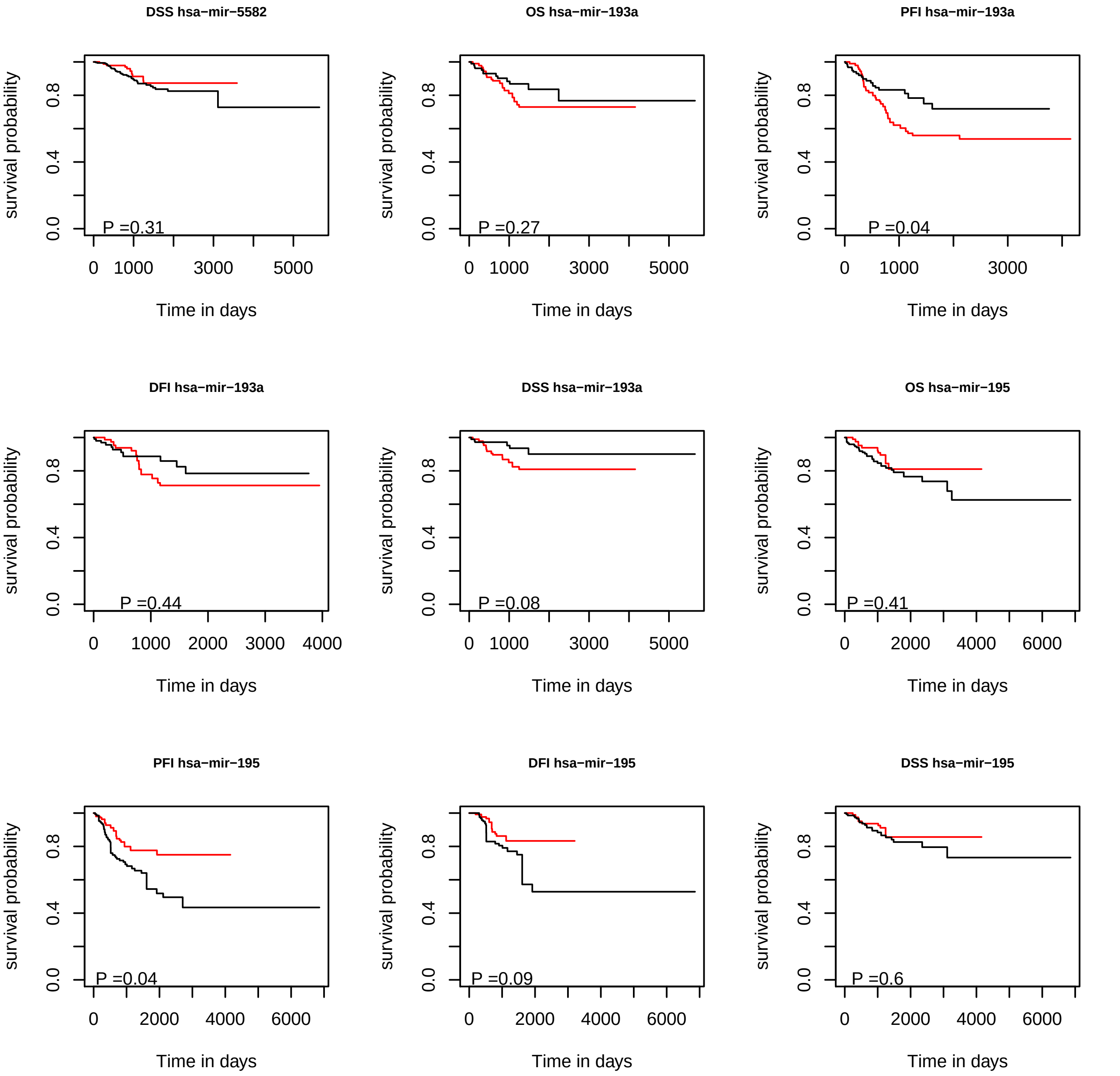
DSS hsa−mir−5582
P =0.31
OS hsa−mir−193a
P =0.27
PFI hsa−mir−193a
P =0.04
DFI hsa−mir−193a
P =0.44
DSS hsa−mir−193a
P =0.08
OS hsa−mir−195
P =0.41
PFI hsa−mir−195
P =0.04
DFI hsa−mir−195
P =0.09
DSS hsa−mir−195
P =0.6
survival probability
0.0
0.4
0.8
Time in days
0
1000
2000
3000
4000
5000
6000

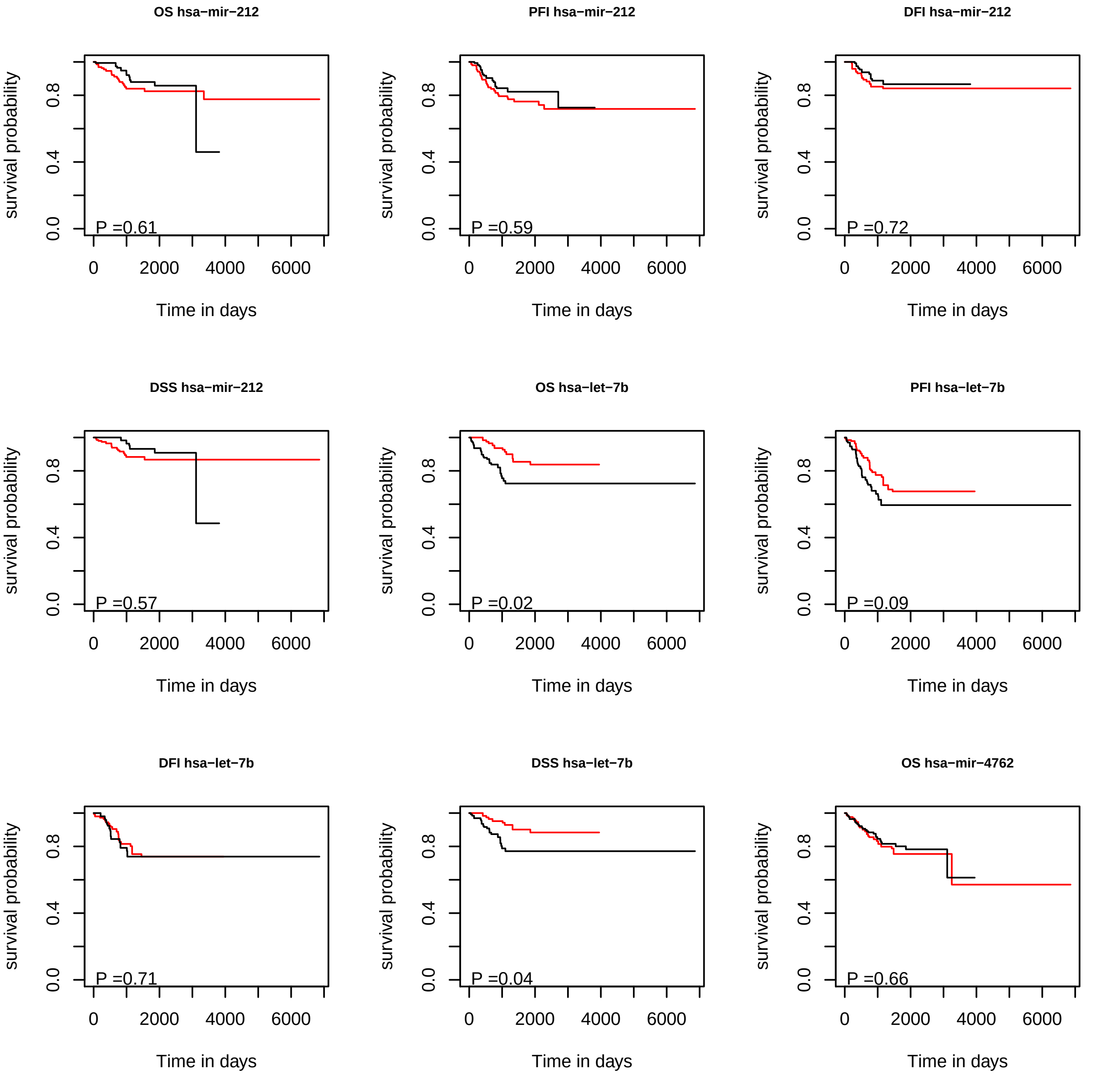
OS hsa−mir−212
P =0.61
PFI hsa−mir−212
P =0.59
DFI hsa−mir−212
P =0.72
DSS hsa−mir−212
P =0.57
OS hsa−let−7b
P =0.02
PFI hsa−let−7b
P =0.09
DFI hsa−let−7b
P =0.71
DSS hsa−let−7b
P =0.04
OS hsa−mir−4762
P =0.66
survival probability
0.0
0.4
0.8
Time in days
0
2000
4000
6000

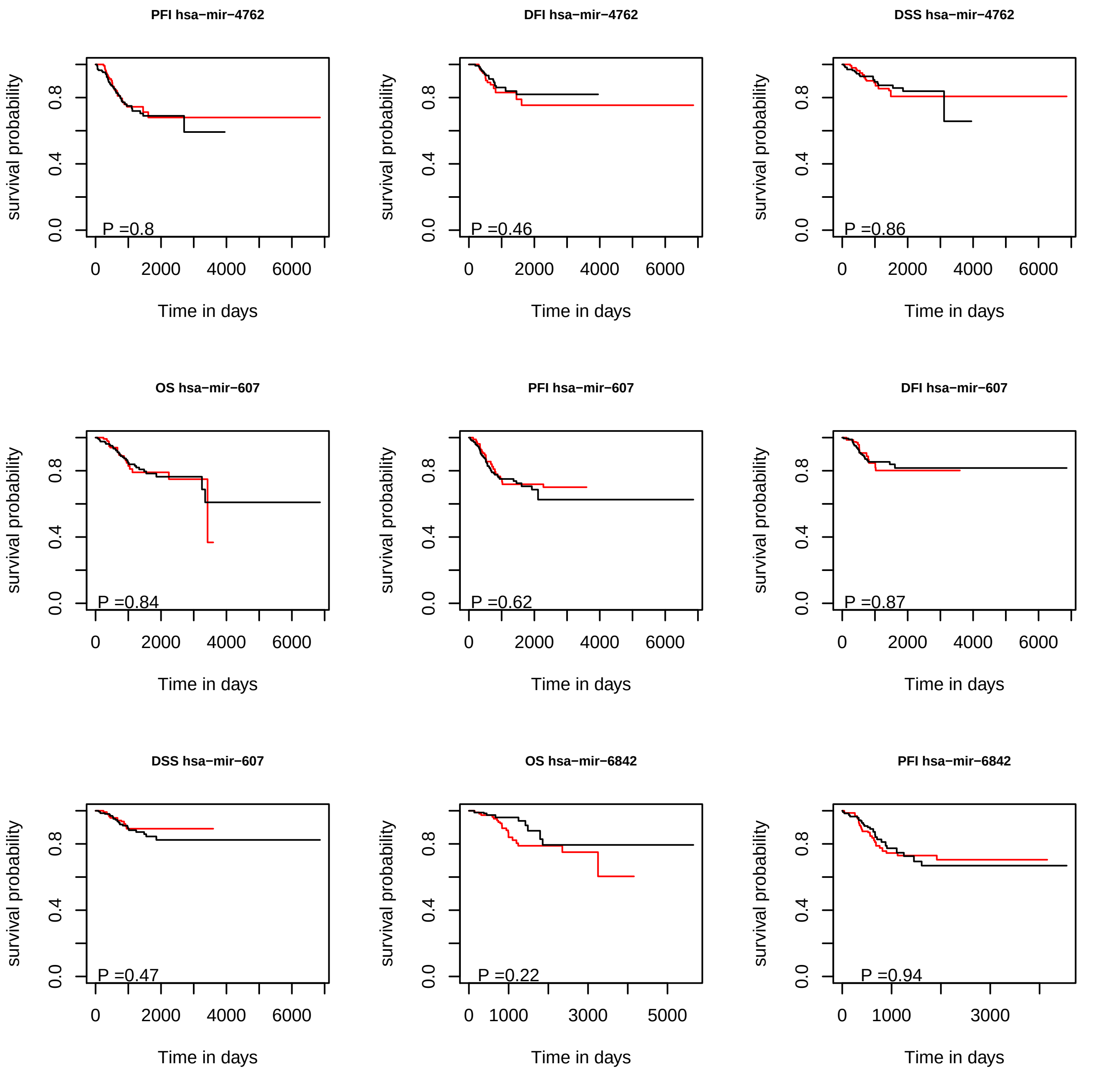

PFI hsa−mir−4762
P =0.8
DFI hsa−mir−4762
P =0.46
DSS hsa−mir−4762
P =0.86
OS hsa−mir−607
P =0.84
PFI hsa−mir−607
P =0.62
DFI hsa−mir−607
P =0.87
DSS hsa−mir−607
P =0.47
OS hsa−mir−6842
P =0.22
PFI hsa−mir−6842
P =0.94
survival probability
Time in days

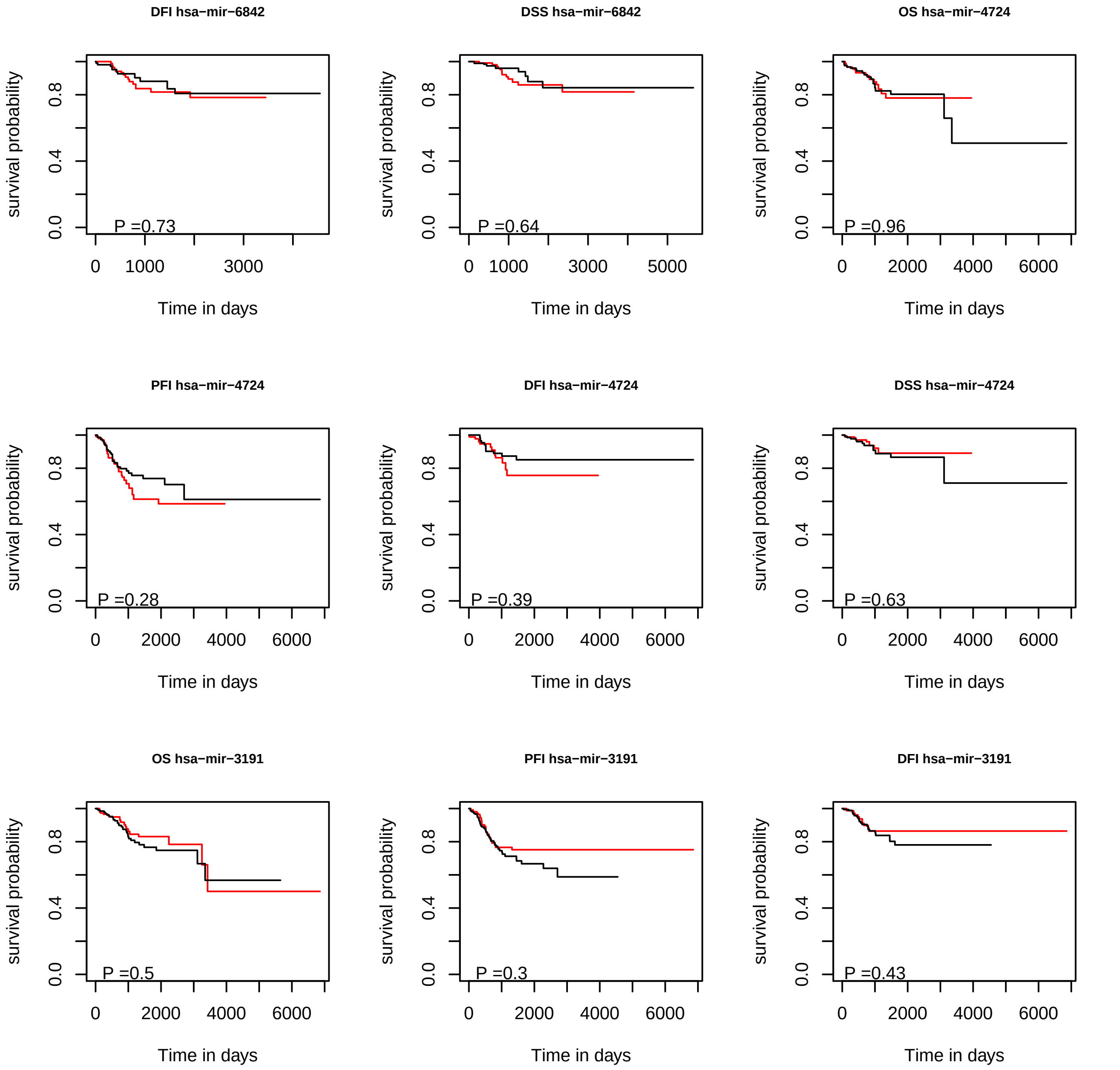
DFI hsa−mir−6842
P =0.73
DSS hsa−mir−6842
P =0.64
OS hsa−mir−4724
P =0.96
PFI hsa−mir−4724
P =0.28
DFI hsa−mir−4724
P =0.39
DSS hsa−mir−4724
P =0.63
OS hsa−mir−3191
P =0.5
PFI hsa−mir−3191
P =0.3
DFI hsa−mir−3191
P =0.43
survival probability
Time in days

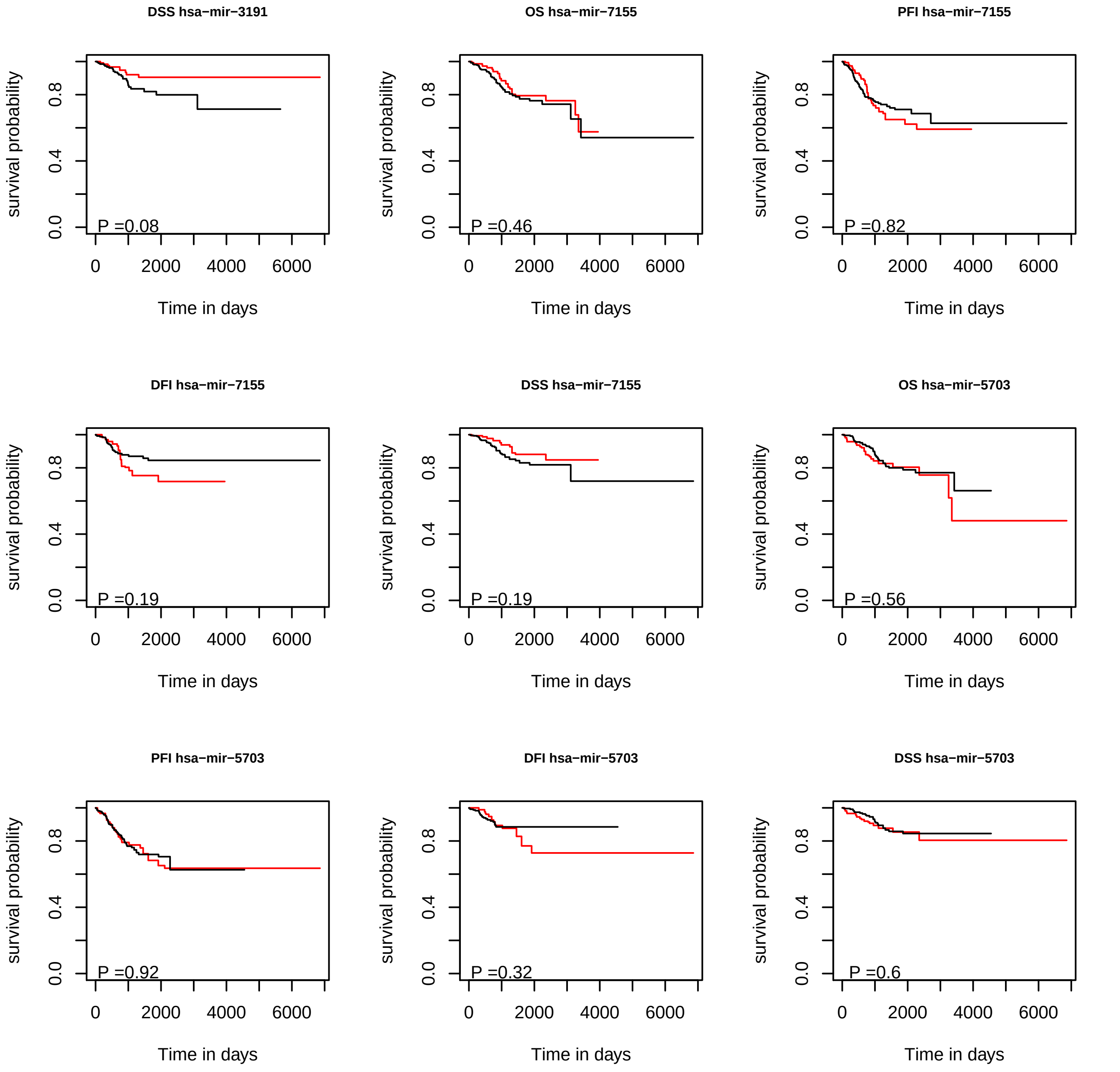
DSS hsa−mir−3191
P =0.08
OS hsa−mir−7155
P =0.46
PFI hsa−mir−7155
P =0.82
DFI hsa−mir−7155
P =0.19
DSS hsa−mir−7155
P =0.19
OS hsa−mir−5703
P =0.56
PFI hsa−mir−5703
P =0.92
DFI hsa−mir−5703
P =0.32
DSS hsa−mir−5703
P =0.6
survival probability
0.0
0.4
0.8
Time in days
0
2000
4000
6000

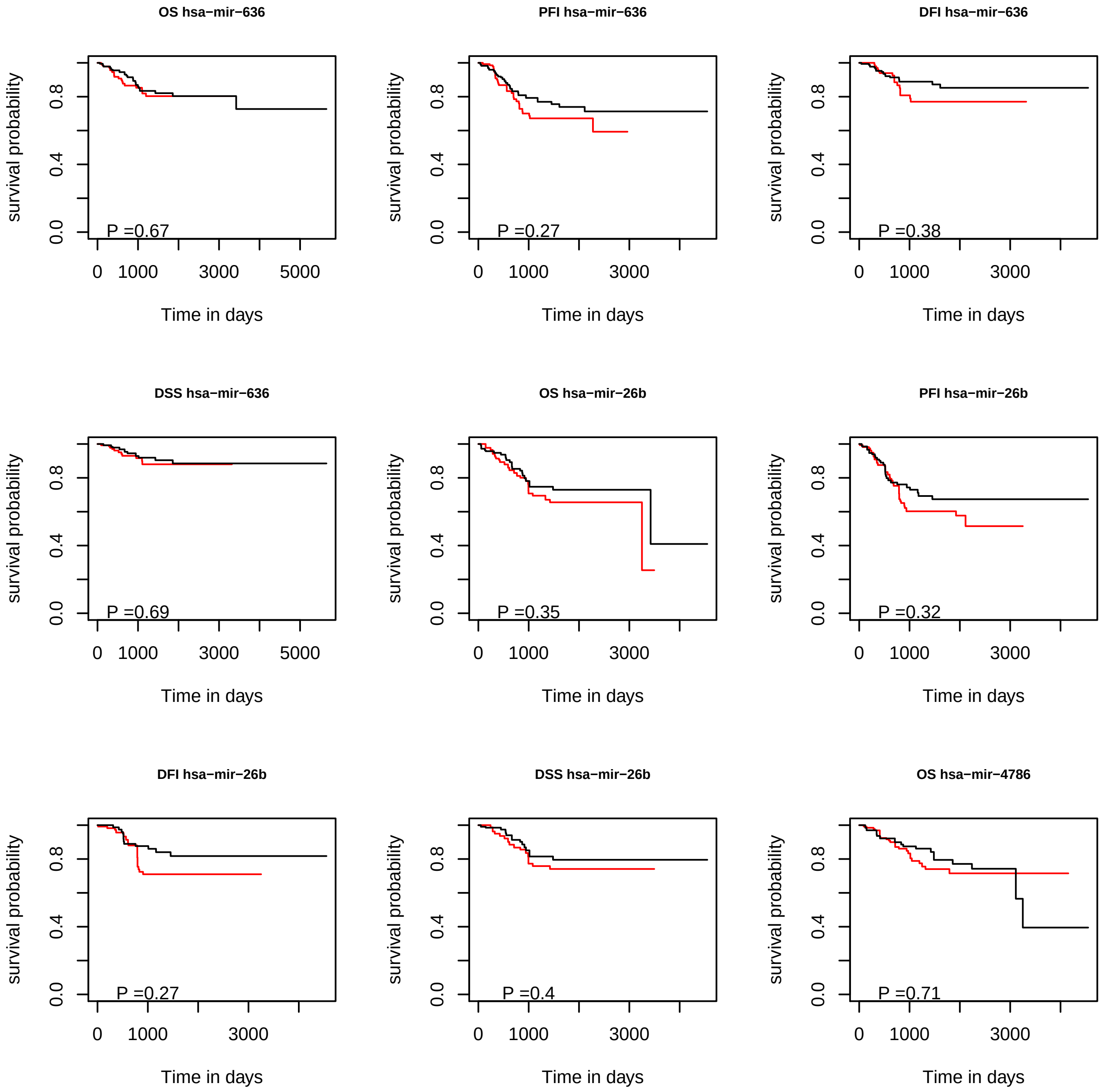
OS hsa−mir−636
PFI hsa−mir−636
DFI hsa−mir−636
DSS hsa−mir−636
OS hsa−mir−26b
PFI hsa−mir−26b
DFI hsa−mir−26b
DSS hsa−mir−26b
OS hsa−mir−4786
survival probability
Time in days
P =0.67
P =0.27
P =0.38
P =0.69
P =0.35
P =0.32
P =0.27
P =0.4
P =0.71
0.0
0.4
0.8
0
1000
3000
5000

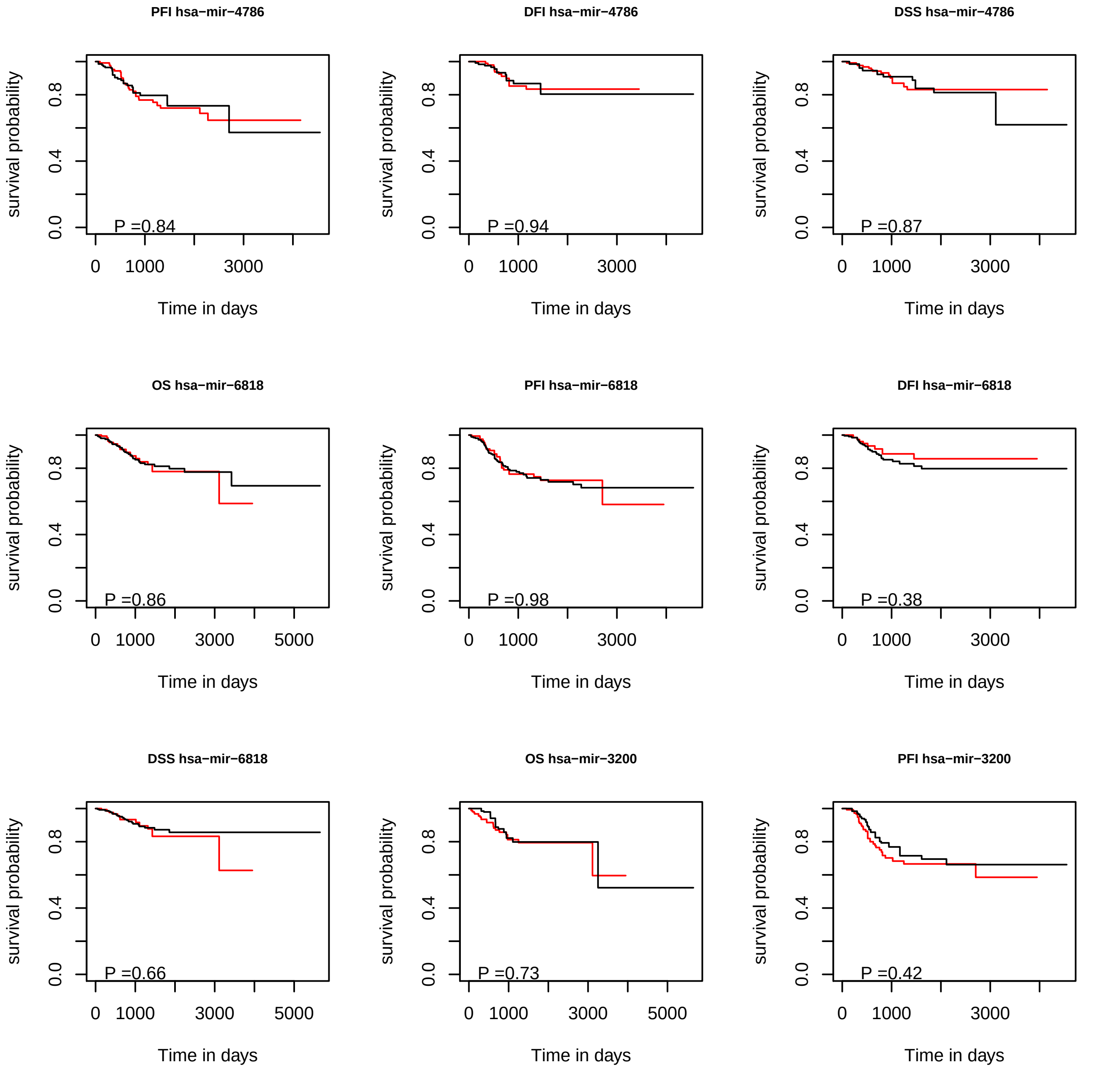
PFI hsa−mir−4786
survival probability
0.0
0.4
0.8
0
1000
3000
Time in days
P =0.84
DFI hsa−mir−4786
P =0.94
DSS hsa−mir−4786
P =0.87
OS hsa−mir−6818
5000
P =0.86
PFI hsa−mir−6818
P =0.98
DFI hsa−mir−6818
P =0.38
DSS hsa−mir−6818
P =0.66
OS hsa−mir−3200
P =0.73
PFI hsa−mir−3200
P =0.42

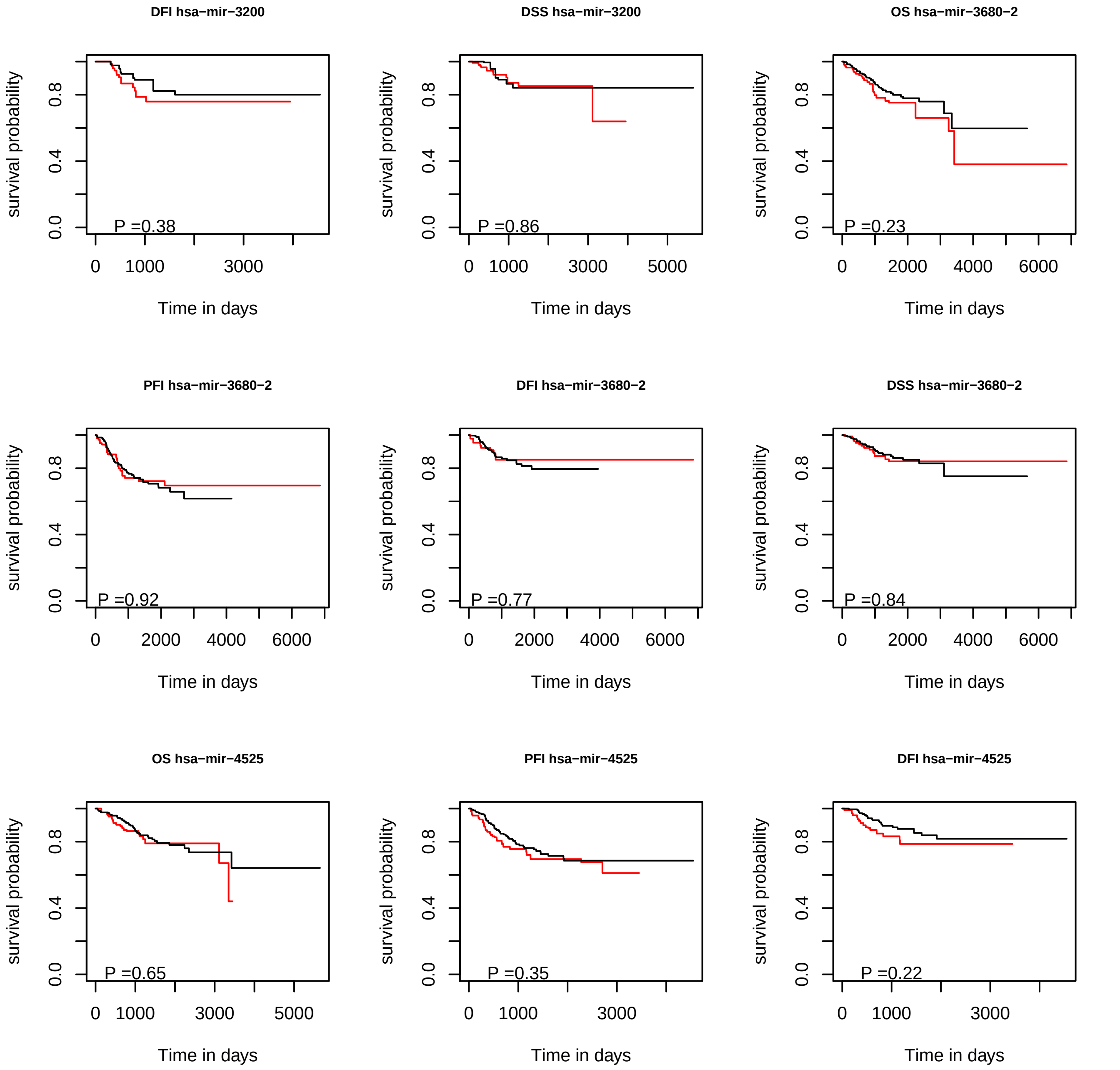
DFI hsa−mir−3200
P =0.38
DSS hsa−mir−3200
P =0.86
OS hsa−mir−3680−2
P =0.23
PFI hsa−mir−3680−2
P =0.92
DFI hsa−mir−3680−2
P =0.77
DSS hsa−mir−3680−2
P =0.84
OS hsa−mir−4525
P =0.65
PFI hsa−mir−4525
P =0.35
DFI hsa−mir−4525
P =0.22
survival probability
Time in days

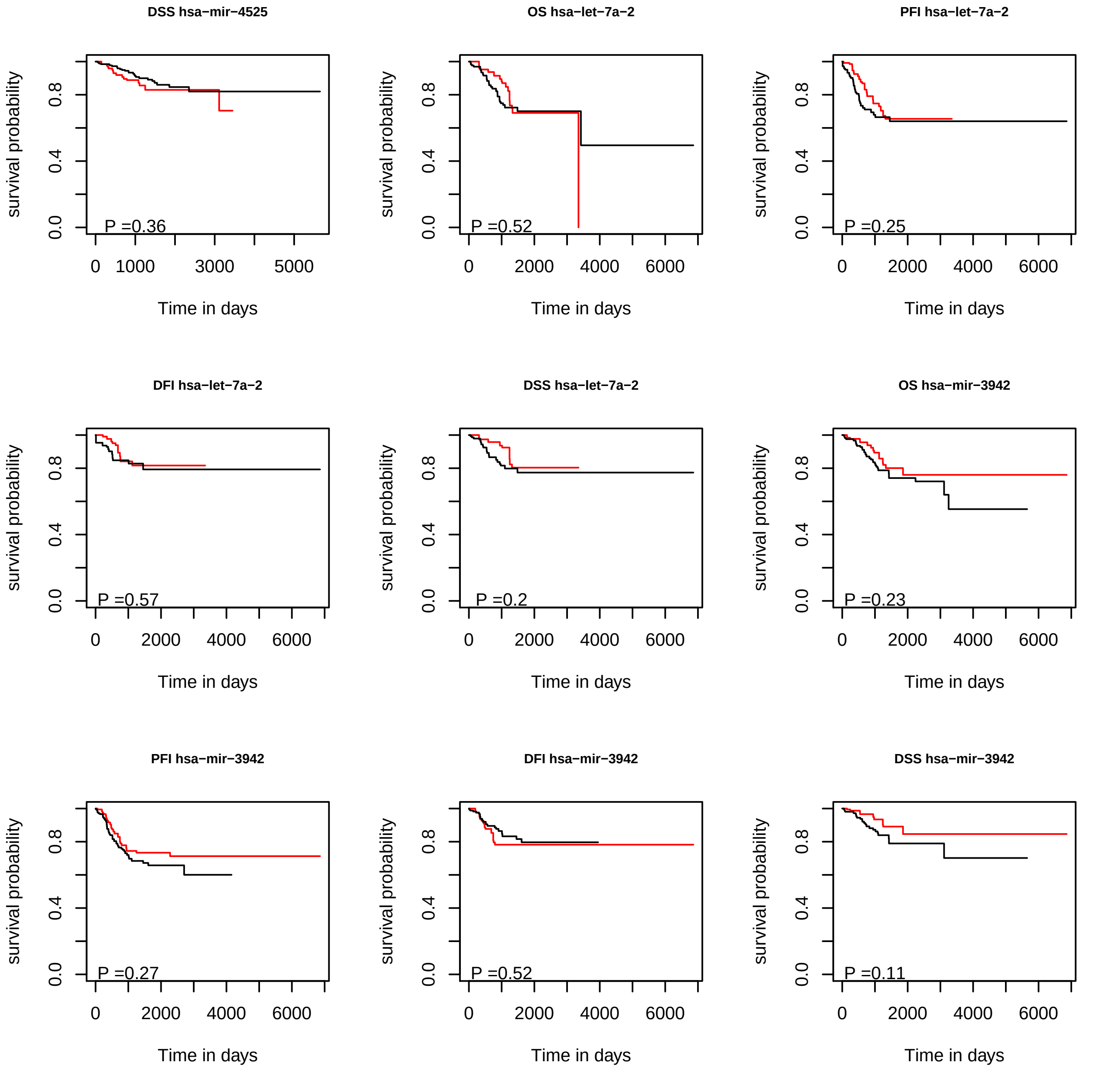

DSS hsa−mir−4525
P =0.36
OS hsa−let−7a−2
P =0.52
PFI hsa−let−7a−2
P =0.25
DFI hsa−let−7a−2
P =0.57
DSS hsa−let−7a−2
P =0.2
OS hsa−mir−3942
P =0.23
PFI hsa−mir−3942
P =0.27
DFI hsa−mir−3942
P =0.52
DSS hsa−mir−3942
P =0.11
survival probability
Time in days
0.0
0.4
0.8
0
1000
3000
5000
2000
4000
6000

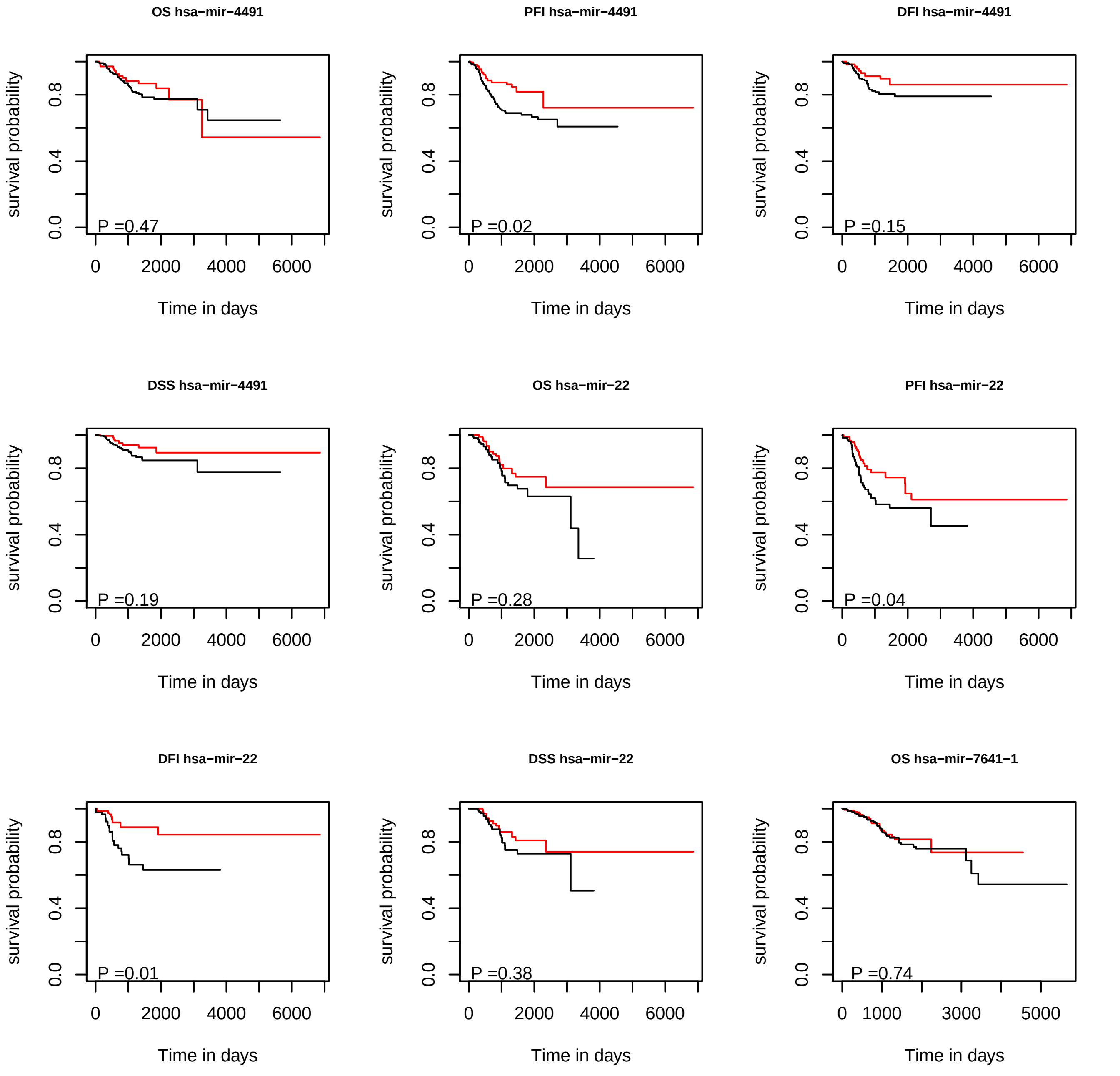

OS hsa−mir−4491
P =0.47
PFI hsa−mir−4491
P =0.02
DFI hsa−mir−4491
P =0.15
DSS hsa−mir−4491
P =0.19
OS hsa−mir−22
P =0.28
PFI hsa−mir−22
P =0.04
DFI hsa−mir−22
P =0.01
DSS hsa−mir−22
P =0.38
OS hsa−mir−7641−1
P =0.74
survival probability
0.0
0.4
0.8
Time in days
0
2000
4000
6000
1000
3000
5000

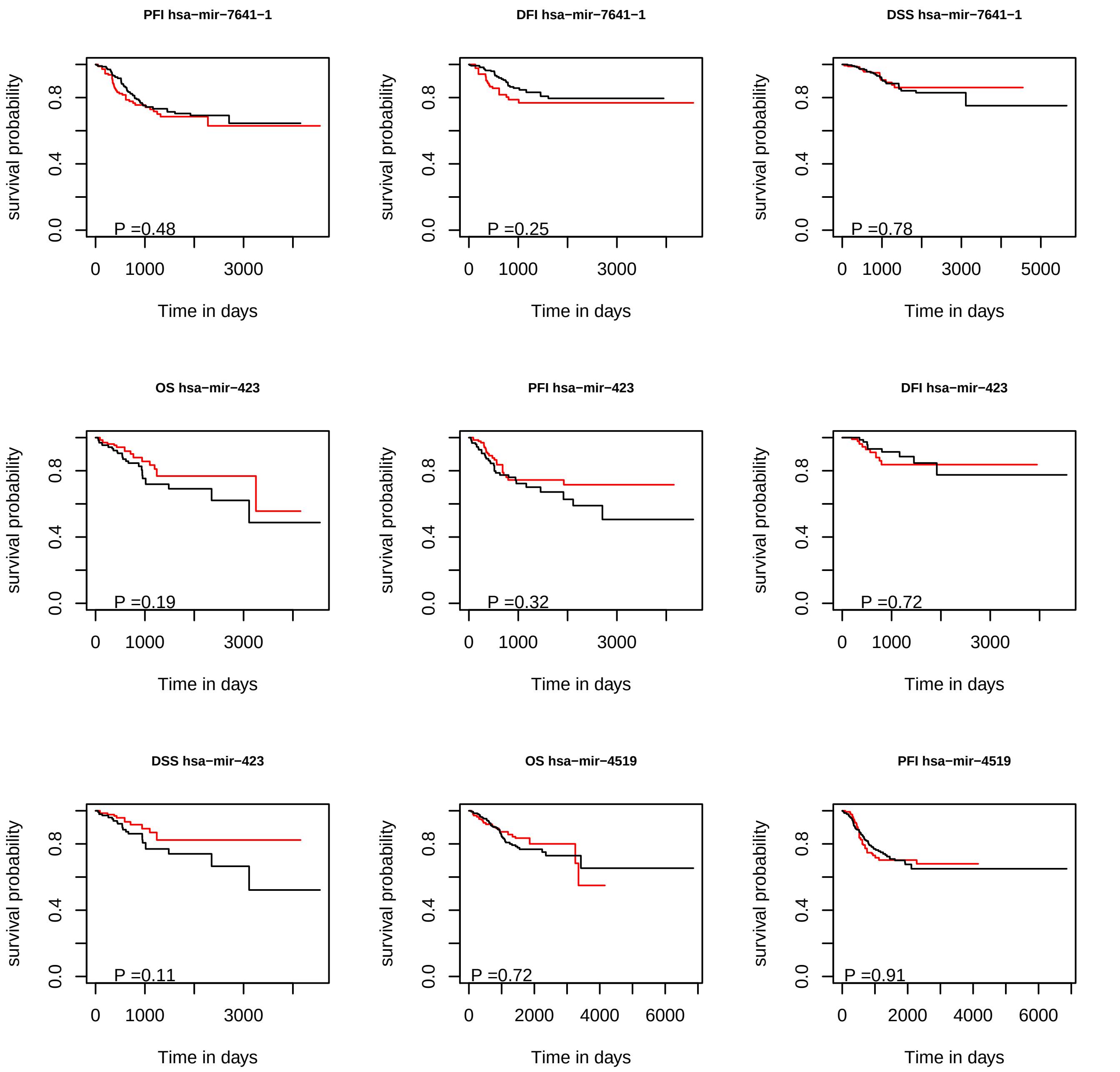
PFI hsa−mir−7641−1
P =0.48
DFI hsa−mir−7641−1
P =0.25
DSS hsa−mir−7641−1
P =0.78
OS hsa−mir−423
P =0.19
PFI hsa−mir−423
P =0.32
DFI hsa−mir−423
P =0.72
DSS hsa−mir−423
P =0.11
OS hsa−mir−4519
P =0.72
PFI hsa−mir−4519
P =0.91
survival probability
Time in days

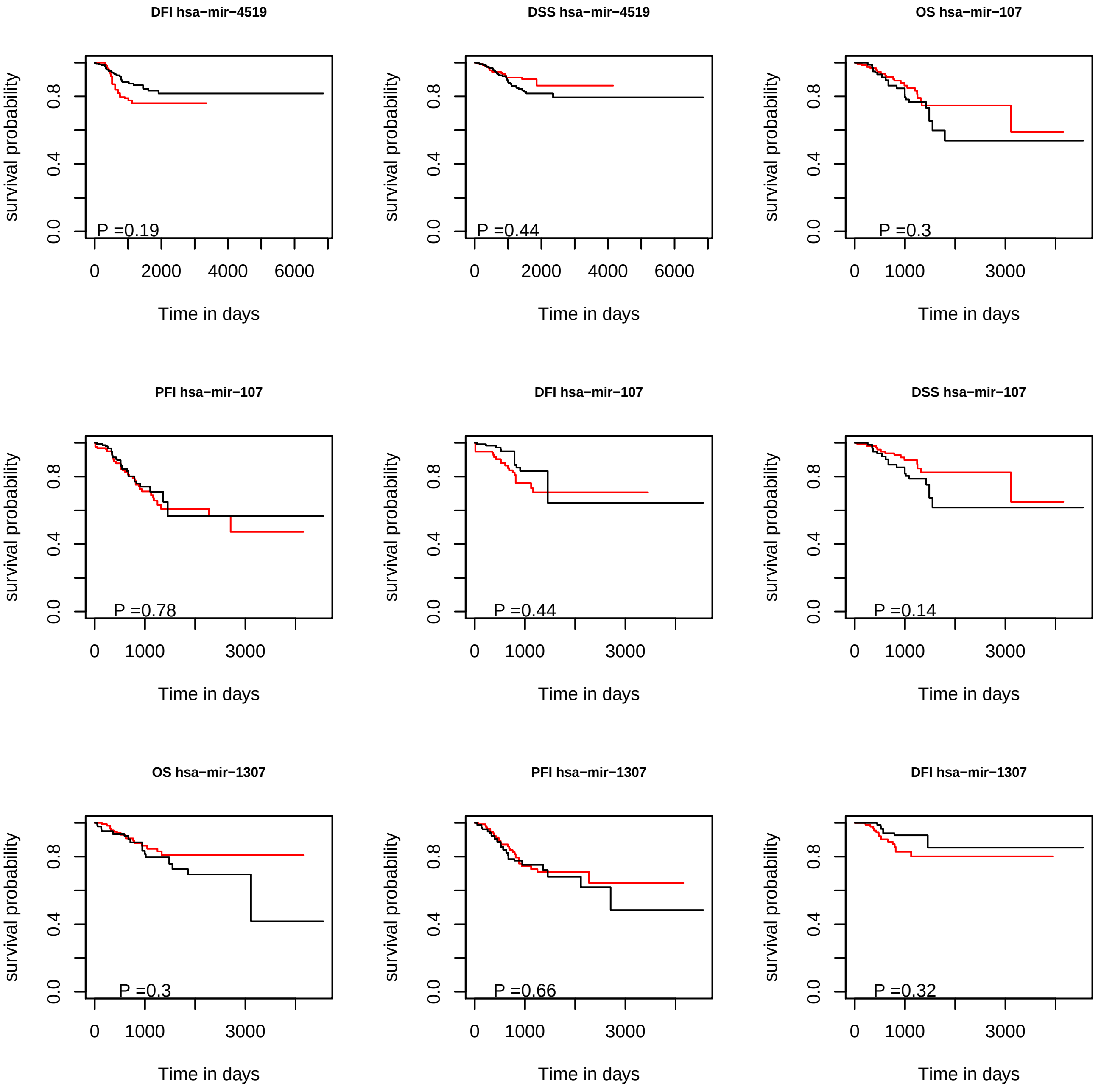
DFI hsa−mir−4519
survival probability
0.0
0.4
0.8
P =0.19
0
2000
4000
6000
Time in days
DSS hsa−mir−4519
survival probability
0.0
0.4
0.8
P =0.44
0
2000
4000
6000
Time in days
OS hsa−mir−107
survival probability
0.0
0.4
0.8
P =0.3
0
1000
3000
Time in days
PFI hsa−mir−107
survival probability
0.0
0.4
0.8
P =0.78
0
1000
3000
Time in days
DFI hsa−mir−107
survival probability
0.0
0.4
0.8
P =0.44
0
1000
3000
Time in days
DSS hsa−mir−107
survival probability
0.0
0.4
0.8
P =0.14
0
1000
3000
Time in days
OS hsa−mir−1307
survival probability
0.0
0.4
0.8
P =0.3
0
1000
3000
Time in days
PFI hsa−mir−1307
survival probability
0.0
0.4
0.8
P =0.66
0
1000
3000
Time in days
DFI hsa−mir−1307
survival probability
0.0
0.4
0.8
P =0.32
0
1000
3000
Time in days

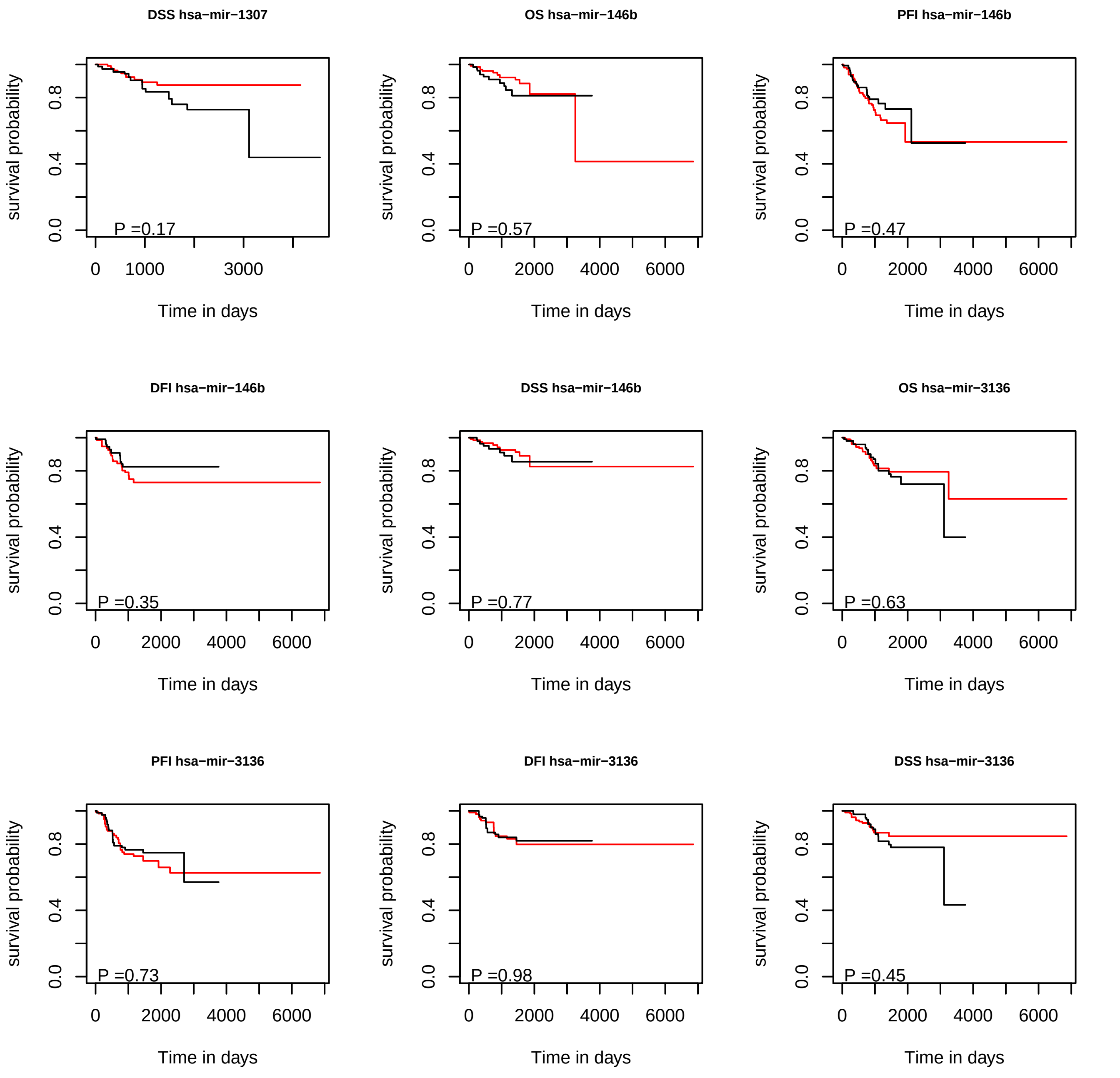
DSS hsa−mir−1307
P =0.17
OS hsa−mir−146b
P =0.57
PFI hsa−mir−146b
P =0.47
DFI hsa−mir−146b
P =0.35
DSS hsa−mir−146b
P =0.77
OS hsa−mir−3136
P =0.63
PFI hsa−mir−3136
P =0.73
DFI hsa−mir−3136
P =0.98
DSS hsa−mir−3136
P =0.45
survival probability
Time in days
0.0
0.4
0.8
0
1000
3000
2000
4000
6000

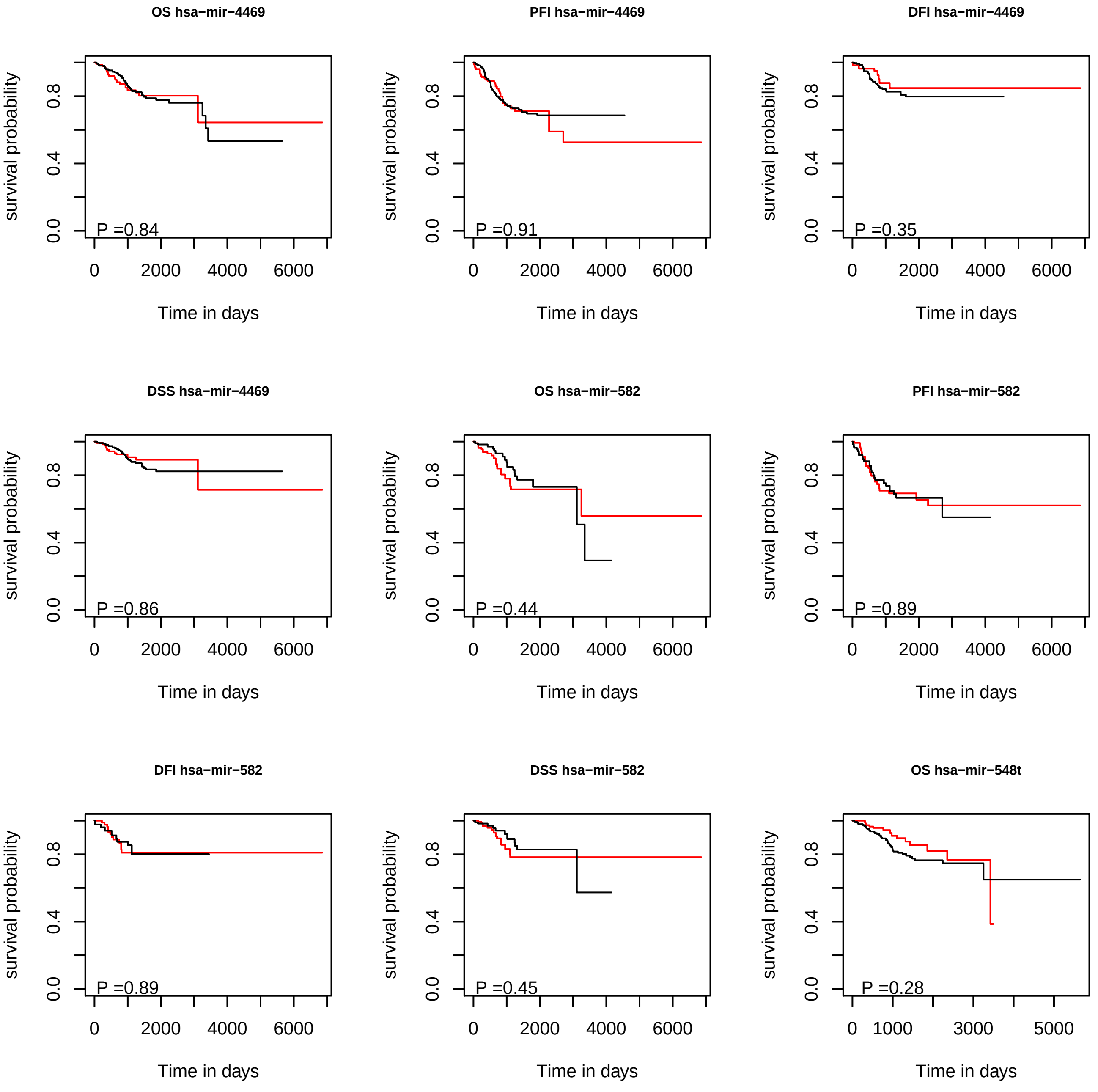

OS hsa−mir−4469
P =0.84
PFI hsa−mir−4469
P =0.91
DFI hsa−mir−4469
P =0.35
DSS hsa−mir−4469
P =0.86
OS hsa−mir−582
P =0.44
PFI hsa−mir−582
P =0.89
DFI hsa−mir−582
P =0.89
DSS hsa−mir−582
P =0.45
OS hsa−mir−548t
P =0.28
survival probability
0.0
0.4
0.8
Time in days
0
2000
4000
6000
1000
3000
5000

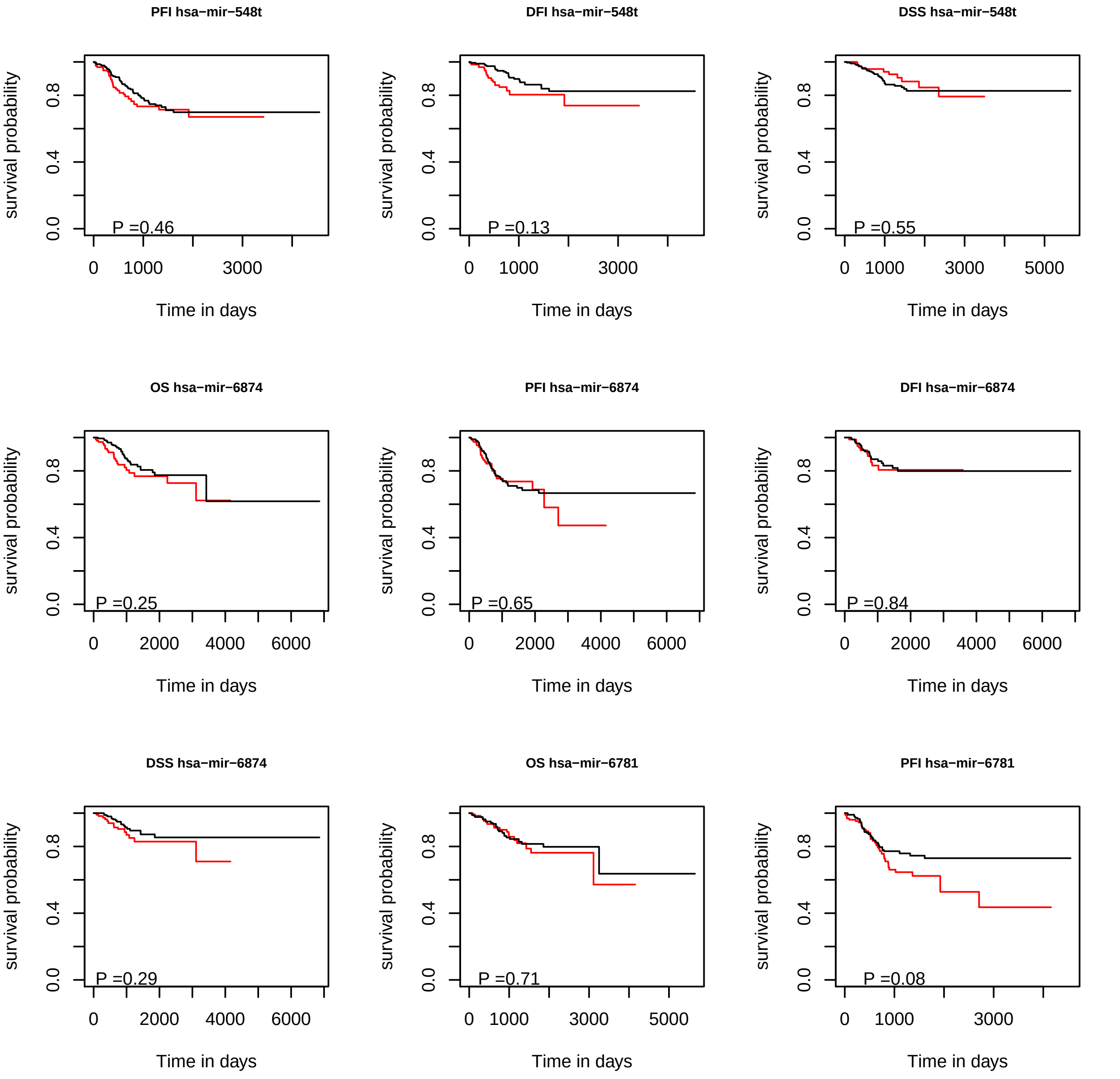
PFI hsa−mir−548t
P =0.46
DFI hsa−mir−548t
P =0.13
DSS hsa−mir−548t
P =0.55
OS hsa−mir−6874
P =0.25
PFI hsa−mir−6874
P =0.65
DFI hsa−mir−6874
P =0.84
DSS hsa−mir−6874
P =0.29
OS hsa−mir−6781
P =0.71
PFI hsa−mir−6781
P =0.08
survival probability
Time in days
0.0
0.4
0.8
0
1000
2000
3000
4000
5000
6000

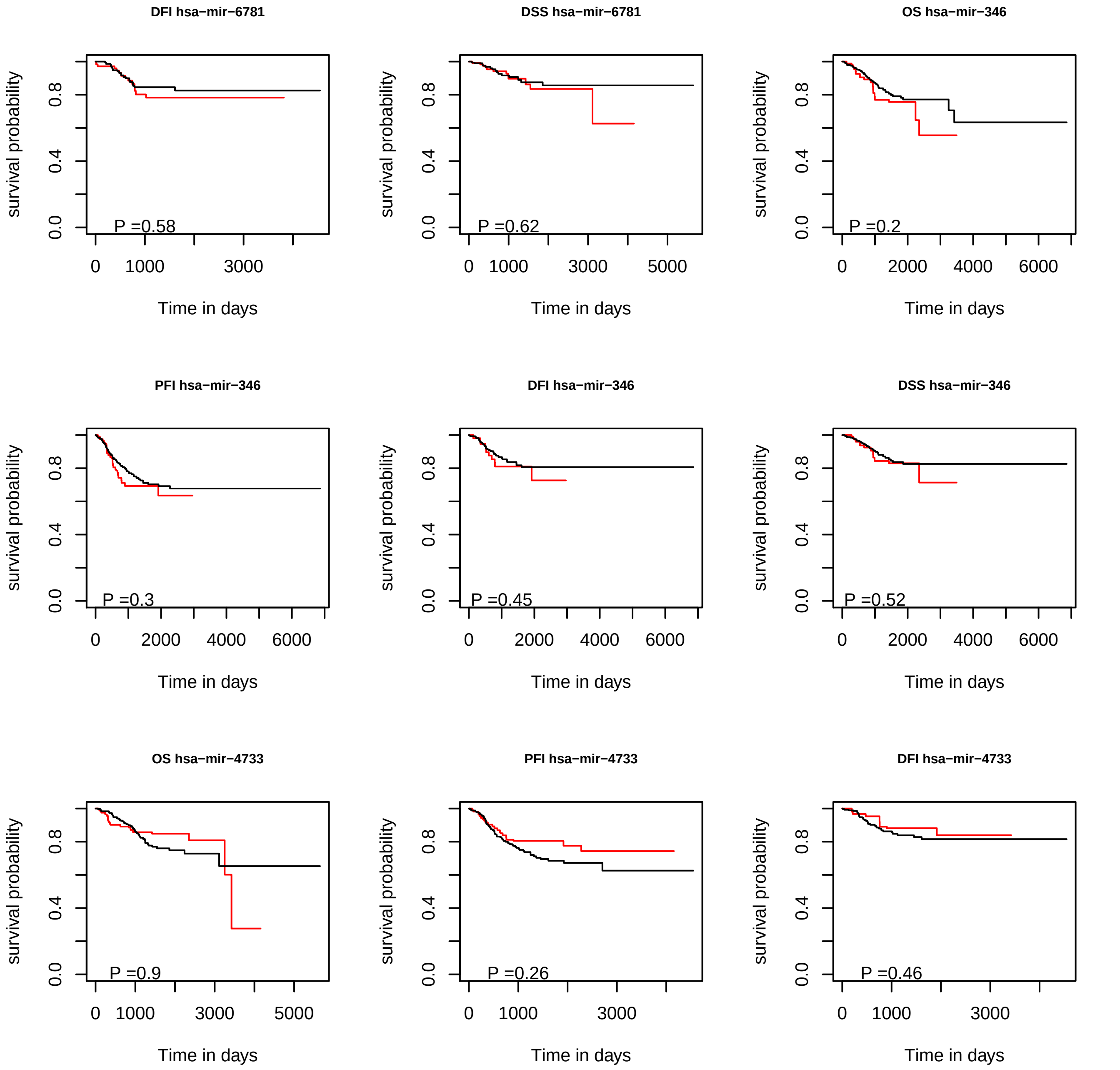
DFI hsa−mir−6781
P =0.58
DSS hsa−mir−6781
P =0.62
OS hsa−mir−346
P =0.2
PFI hsa−mir−346
P =0.3
DFI hsa−mir−346
P =0.45
DSS hsa−mir−346
P =0.52
OS hsa−mir−4733
P =0.9
PFI hsa−mir−4733
P =0.26
DFI hsa−mir−4733
P =0.46
survival probability
Time in days

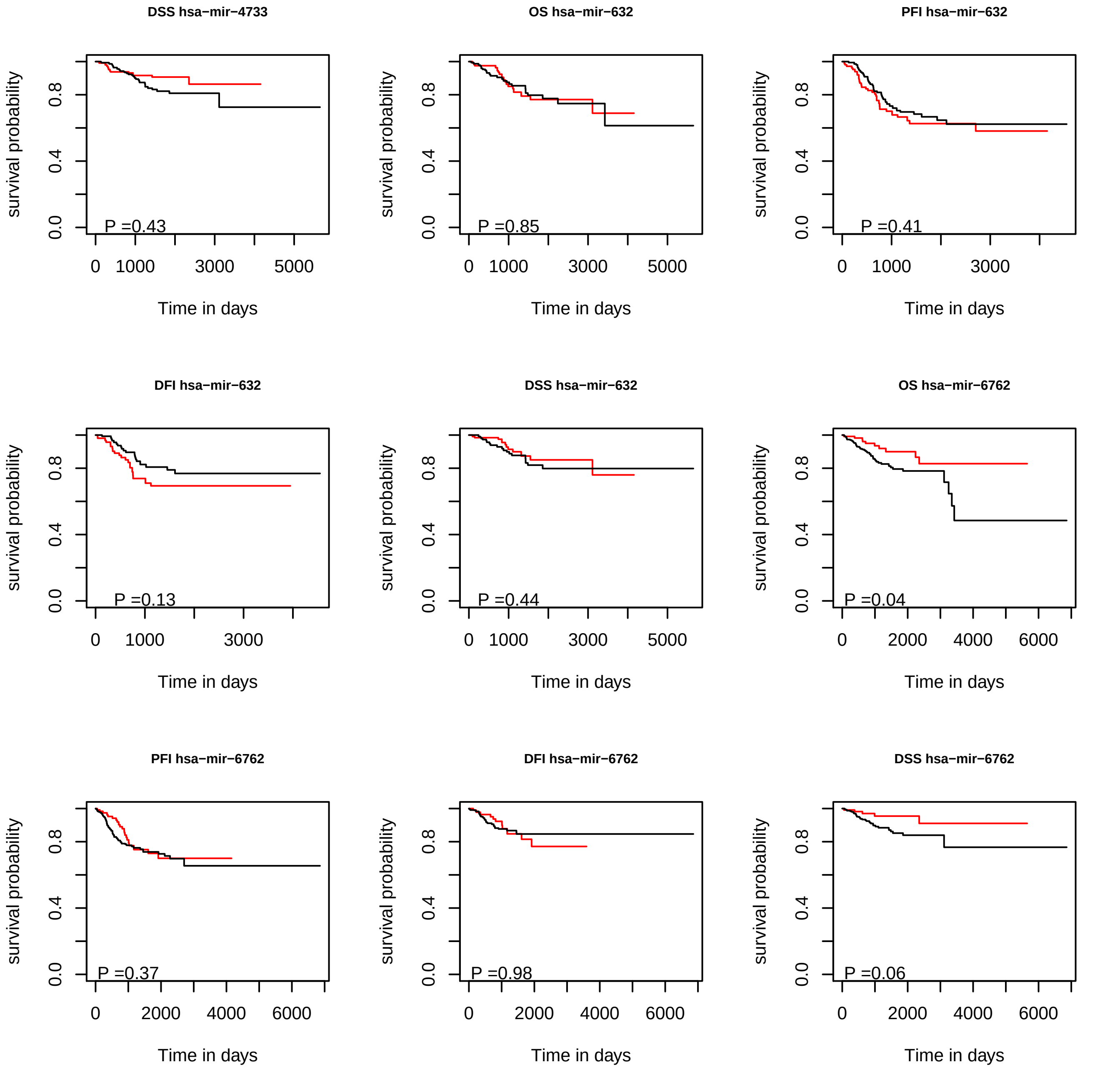
DSS hsa−mir−4733
survival probability
0.0
0.4
0.8
P =0.43
0
1000
3000
5000
Time in days
OS hsa−mir−632
survival probability
0.0
0.4
0.8
P =0.85
0
1000
3000
5000
Time in days
PFI hsa−mir−632
survival probability
0.0
0.4
0.8
P =0.41
0
1000
3000
Time in days
DFI hsa−mir−632
survival probability
0.0
0.4
0.8
P =0.13
0
1000
3000
Time in days
DSS hsa−mir−632
survival probability
0.0
0.4
0.8
P =0.44
0
1000
3000
5000
Time in days
OS hsa−mir−6762
survival probability
0.0
0.4
0.8
P =0.04
0
2000
4000
6000
Time in days
PFI hsa−mir−6762
survival probability
0.0
0.4
0.8
P =0.37
0
2000
4000
6000
Time in days
DFI hsa−mir−6762
survival probability
0.0
0.4
0.8
P =0.98
0
2000
4000
6000
Time in days
DSS hsa−mir−6762
survival probability
0.0
0.4
0.8
P =0.06
0
2000
4000
6000
Time in days

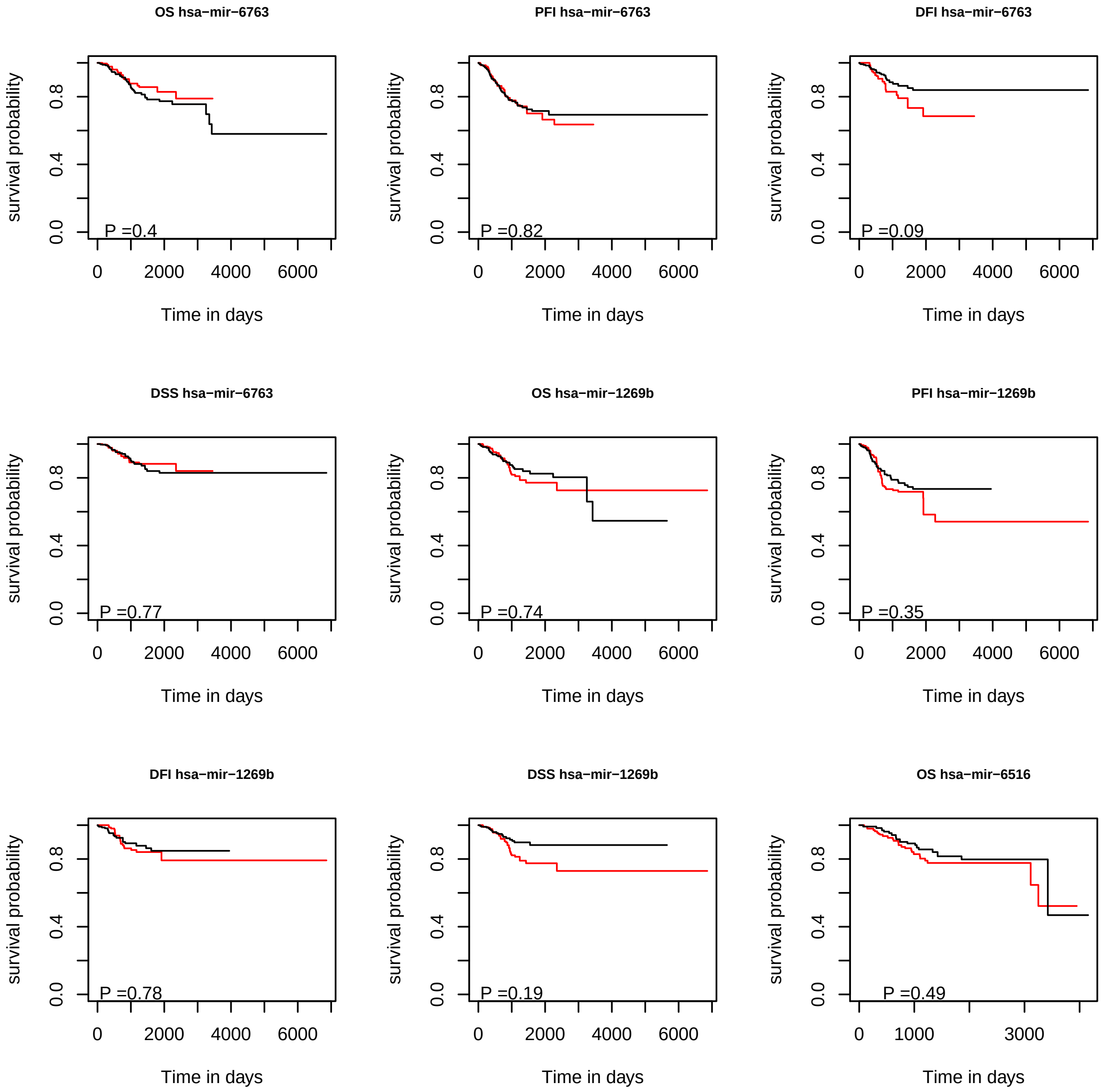
OS hsa−mir−6763
P =0.4
PFI hsa−mir−6763
P =0.82
DFI hsa−mir−6763
P =0.09
DSS hsa−mir−6763
P =0.77
OS hsa−mir−1269b
P =0.74
PFI hsa−mir−1269b
P =0.35
DFI hsa−mir−1269b
P =0.78
DSS hsa−mir−1269b
P =0.19
OS hsa−mir−6516
P =0.49
survival probability
0.0
0.4
0.8
Time in days
0
1000
2000
3000
4000
6000

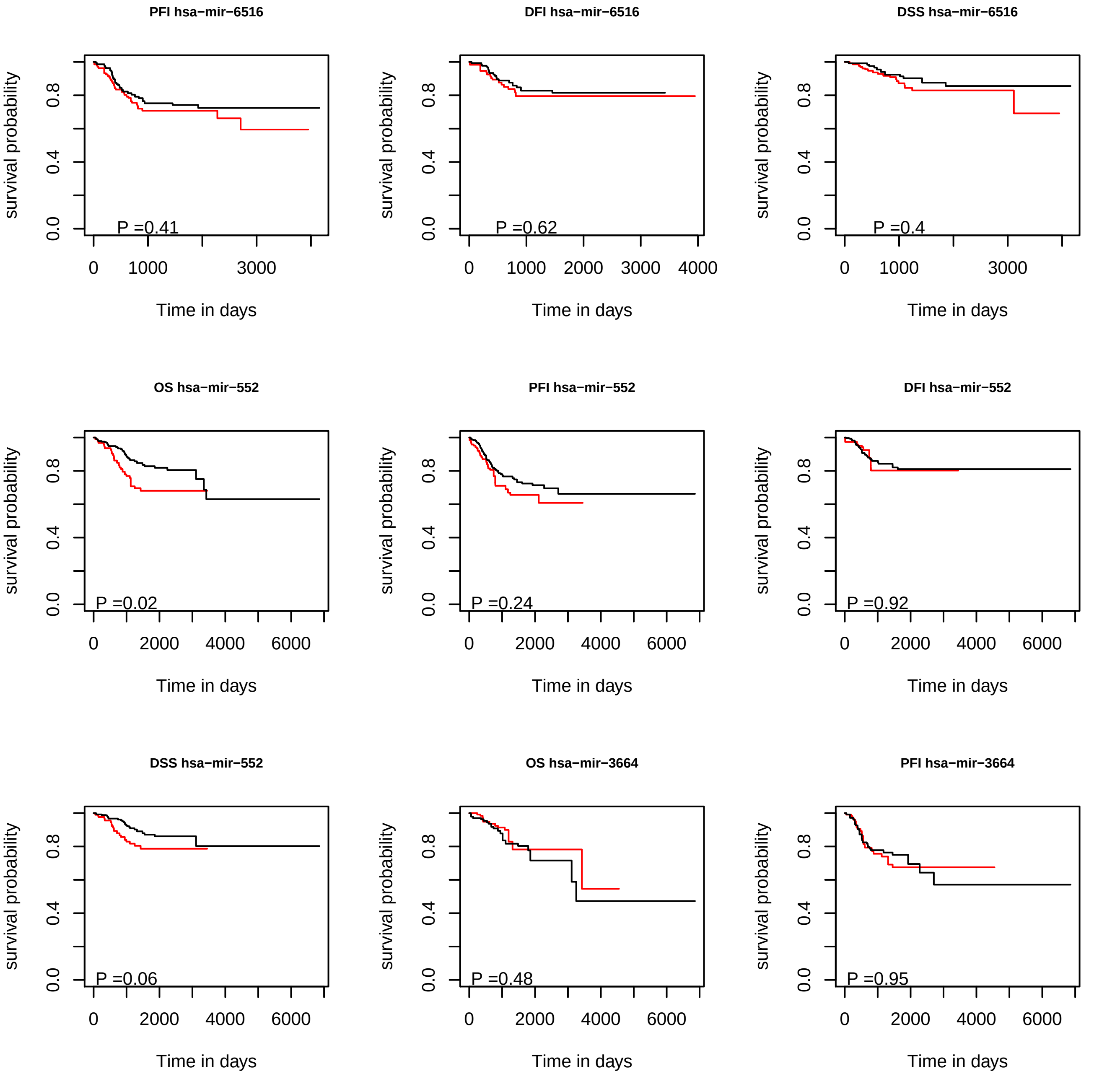
PFI hsa−mir−6516
P =0.41
DFI hsa−mir−6516
P =0.62
DSS hsa−mir−6516
P =0.4
OS hsa−mir−552
P =0.02
PFI hsa−mir−552
P =0.24
DFI hsa−mir−552
P =0.92
DSS hsa−mir−552
P =0.06
OS hsa−mir−3664
P =0.48
PFI hsa−mir−3664
P =0.95
survival probability
Time in days

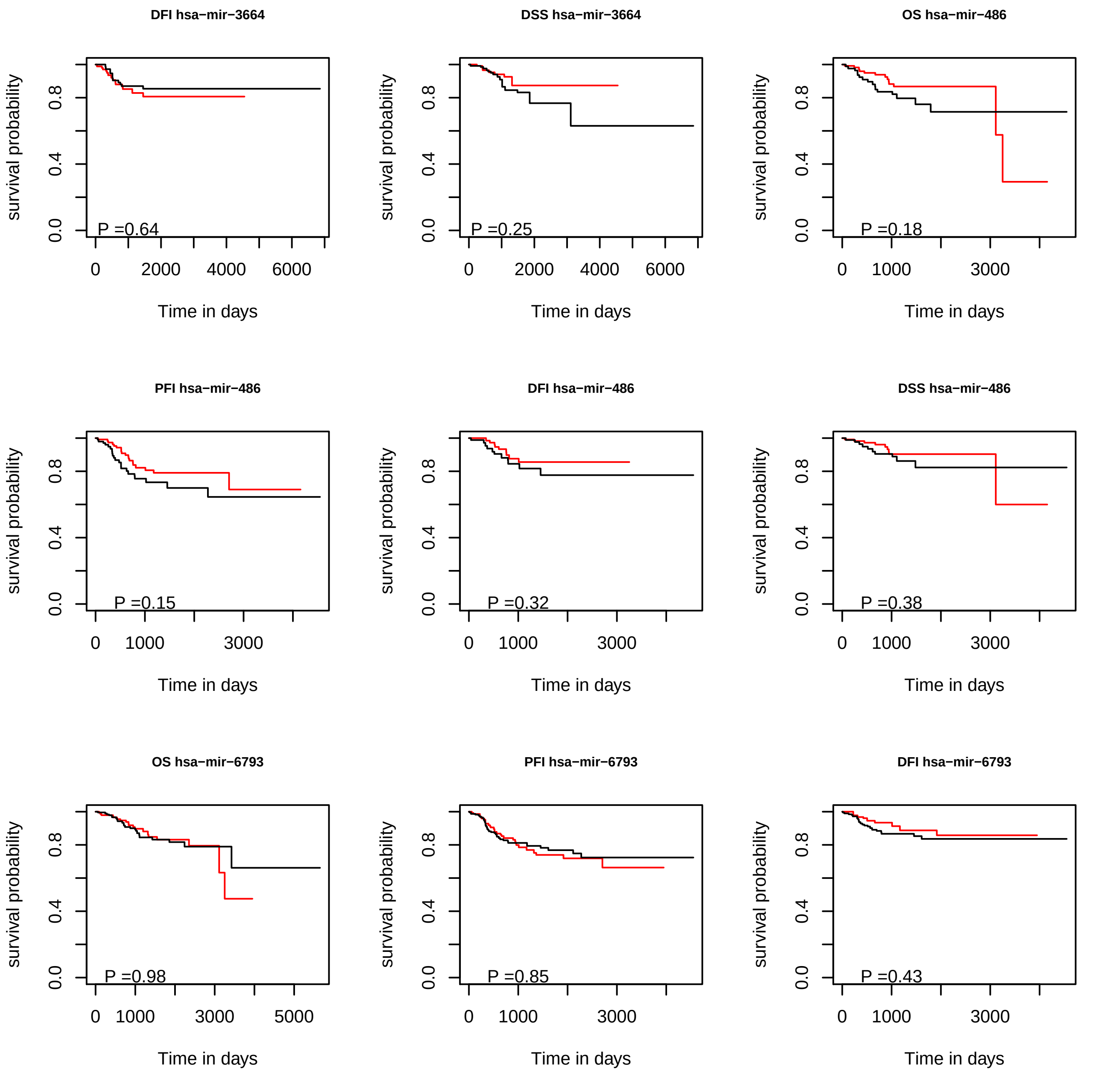
DFI hsa−mir−3664
P =0.64
DSS hsa−mir−3664
P =0.25
OS hsa−mir−486
P =0.18
PFI hsa−mir−486
P =0.15
DFI hsa−mir−486
P =0.32
DSS hsa−mir−486
P =0.38
OS hsa−mir−6793
P =0.98
PFI hsa−mir−6793
P =0.85
DFI hsa−mir−6793
P =0.43
survival probability
Time in days

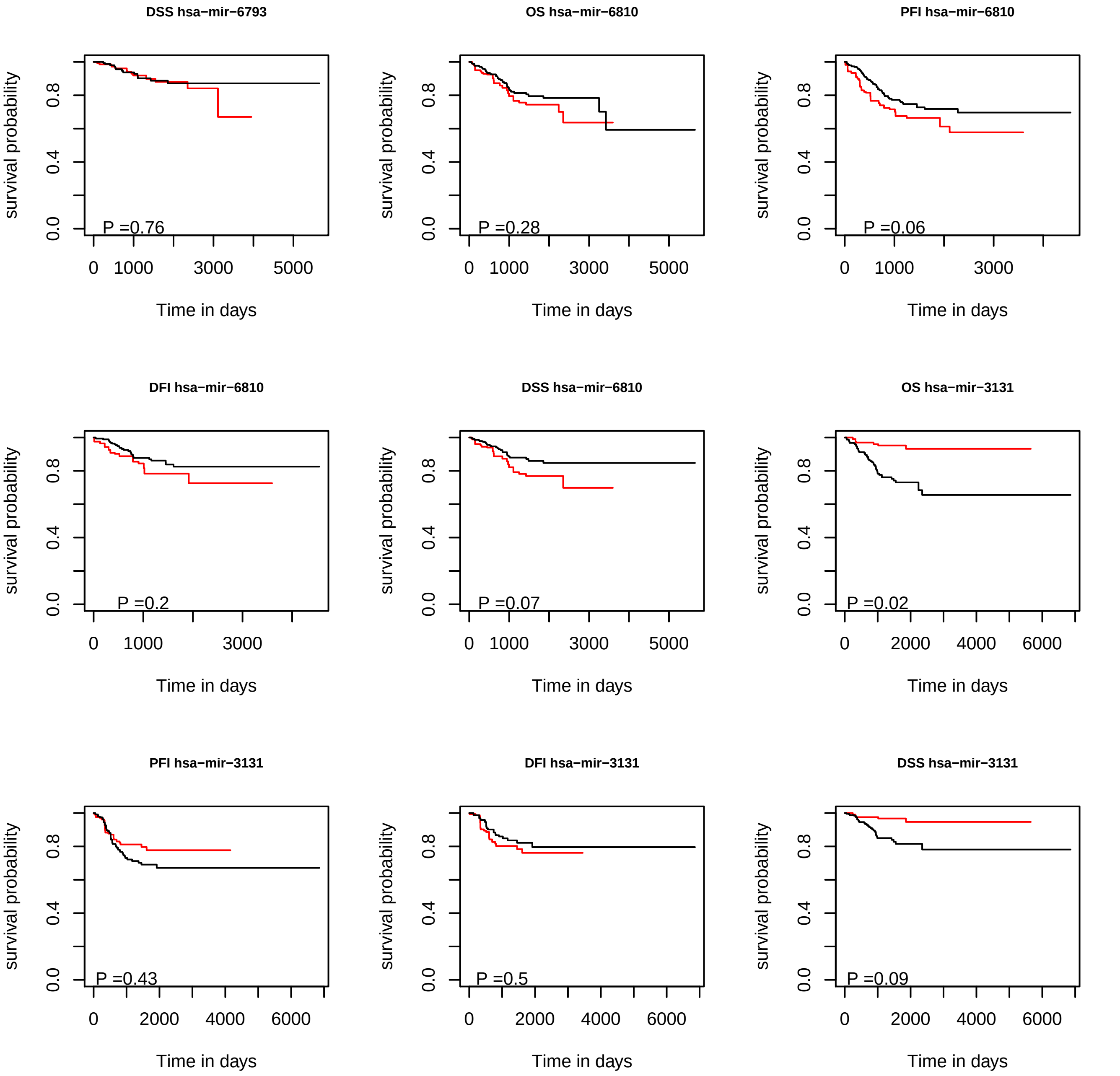
DSS hsa−mir−6793
P =0.76
OS hsa−mir−6810
P =0.28
PFI hsa−mir−6810
P =0.06
DFI hsa−mir−6810
P =0.2
DSS hsa−mir−6810
P =0.07
OS hsa−mir−3131
P =0.02
PFI hsa−mir−3131
P =0.43
DFI hsa−mir−3131
P =0.5
DSS hsa−mir−3131
P =0.09
survival probability
Time in days

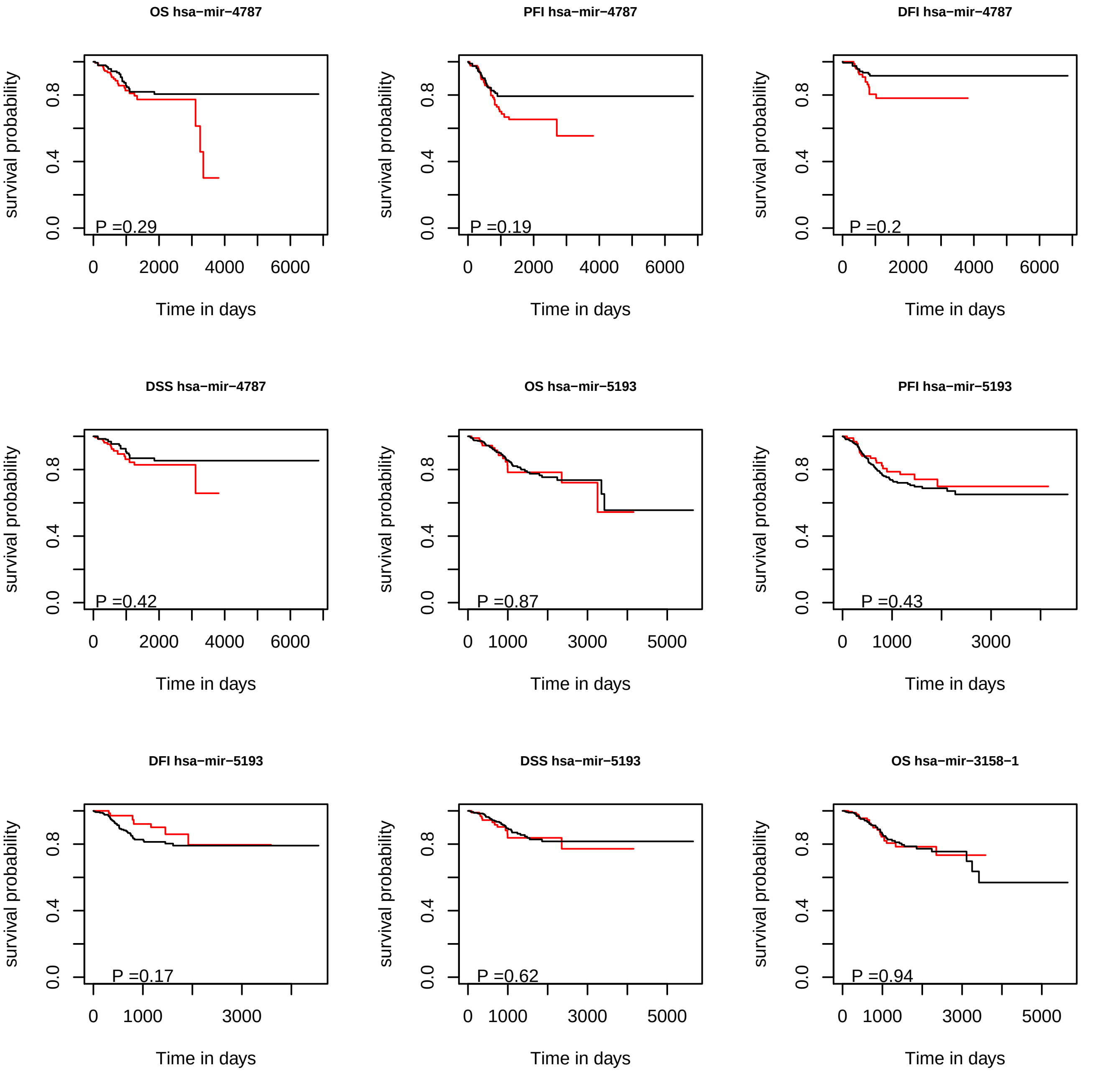
OS hsa−mir−4787
survival probability
0.0
0.4
0.8
P =0.29
0
2000
4000
6000
Time in days
PFI hsa−mir−4787
survival probability
0.0
0.4
0.8
P =0.19
0
2000
4000
6000
Time in days
DFI hsa−mir−4787
survival probability
0.0
0.4
0.8
P =0.2
0
2000
4000
6000
Time in days
DSS hsa−mir−4787
survival probability
0.0
0.4
0.8
P =0.42
0
2000
4000
6000
Time in days
OS hsa−mir−5193
survival probability
0.0
0.4
0.8
P =0.87
0
1000
3000
5000
Time in days
PFI hsa−mir−5193
survival probability
0.0
0.4
0.8
P =0.43
0
1000
3000
Time in days
DFI hsa−mir−5193
survival probability
0.0
0.4
0.8
P =0.17
0
1000
3000
Time in days
DSS hsa−mir−5193
survival probability
0.0
0.4
0.8
P =0.62
0
1000
3000
5000
Time in days
OS hsa−mir−3158−1
survival probability
0.0
0.4
0.8
P =0.94
0
1000
3000
5000
Time in days

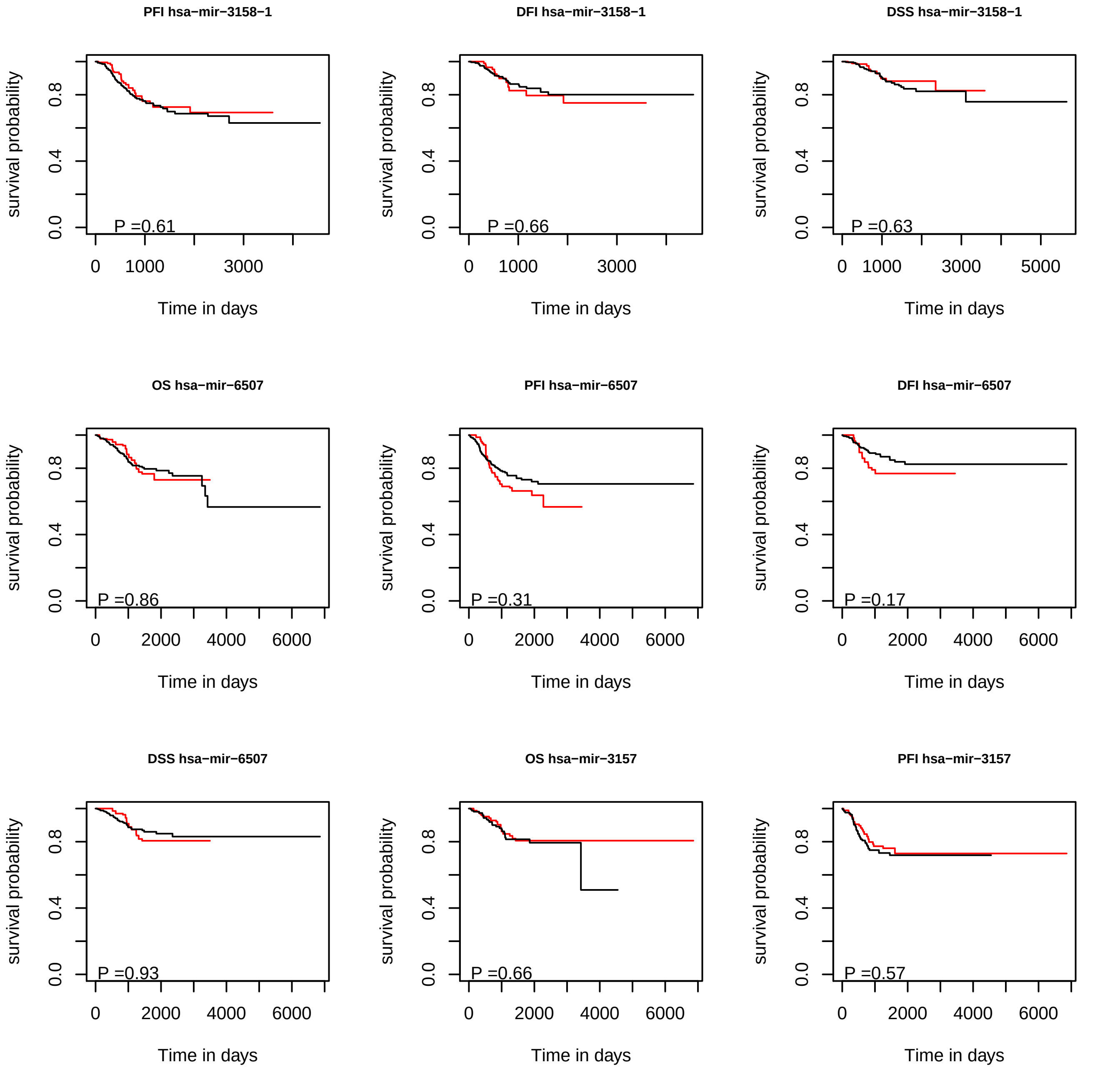
PFI hsa−mir−3158−1
P =0.61
DFI hsa−mir−3158−1
P =0.66
DSS hsa−mir−3158−1
P =0.63
OS hsa−mir−6507
P =0.86
PFI hsa−mir−6507
P =0.31
DFI hsa−mir−6507
P =0.17
DSS hsa−mir−6507
P =0.93
OS hsa−mir−3157
P =0.66
PFI hsa−mir−3157
P =0.57
survival probability
Time in days
0.0
0.4
0.8
0
1000
2000
3000
4000
5000
6000

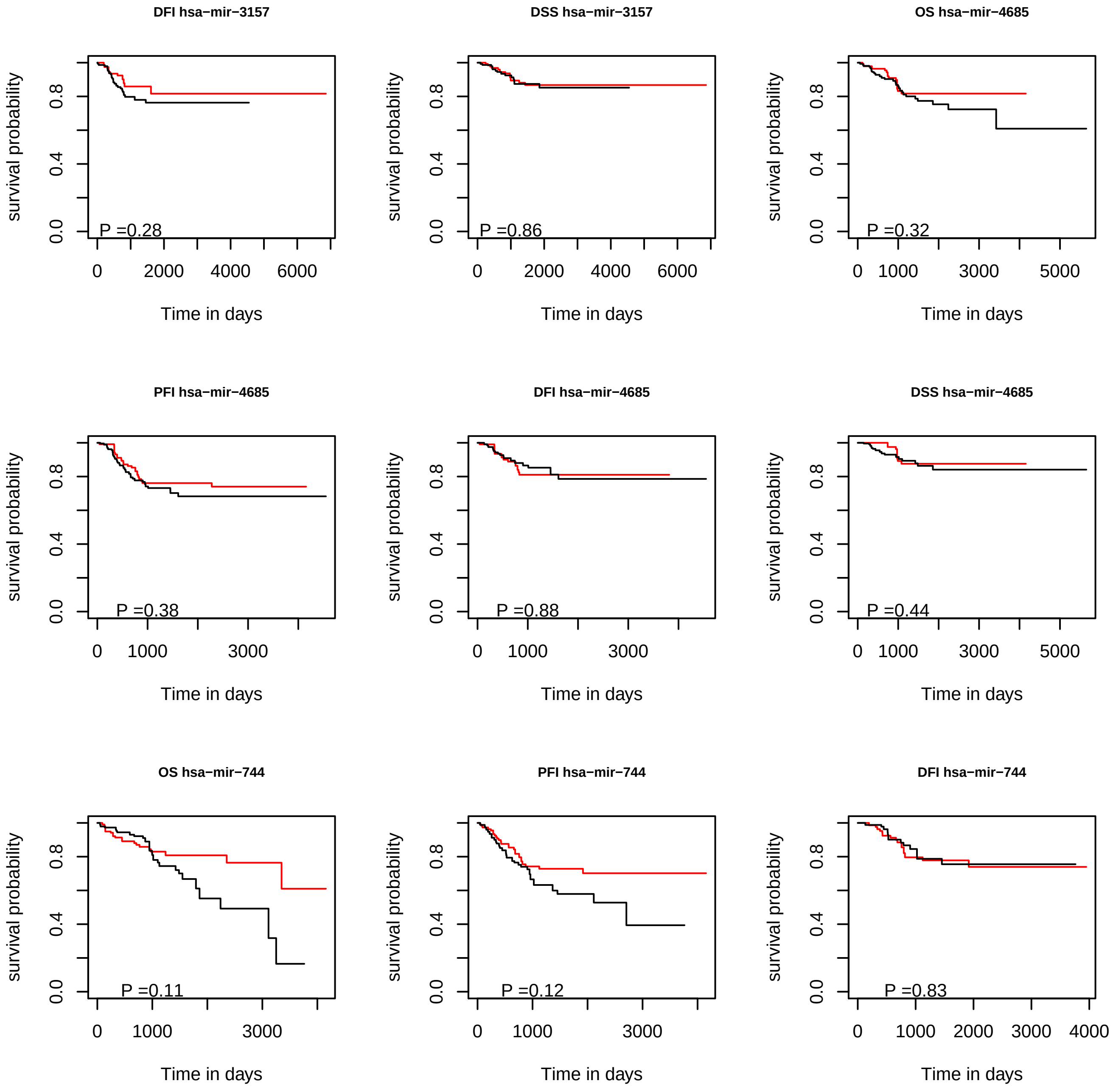

DFI hsa−mir−3157
P =0.28
DSS hsa−mir−3157
P =0.86
OS hsa−mir−4685
P =0.32
PFI hsa−mir−4685
P =0.38
DFI hsa−mir−4685
P =0.88
DSS hsa−mir−4685
P =0.44
OS hsa−mir−744
P =0.11
PFI hsa−mir−744
P =0.12
DFI hsa−mir−744
P =0.83
survival probability
0.0
0.4
0.8
Time in days
0
1000
2000
3000
4000
5000
6000

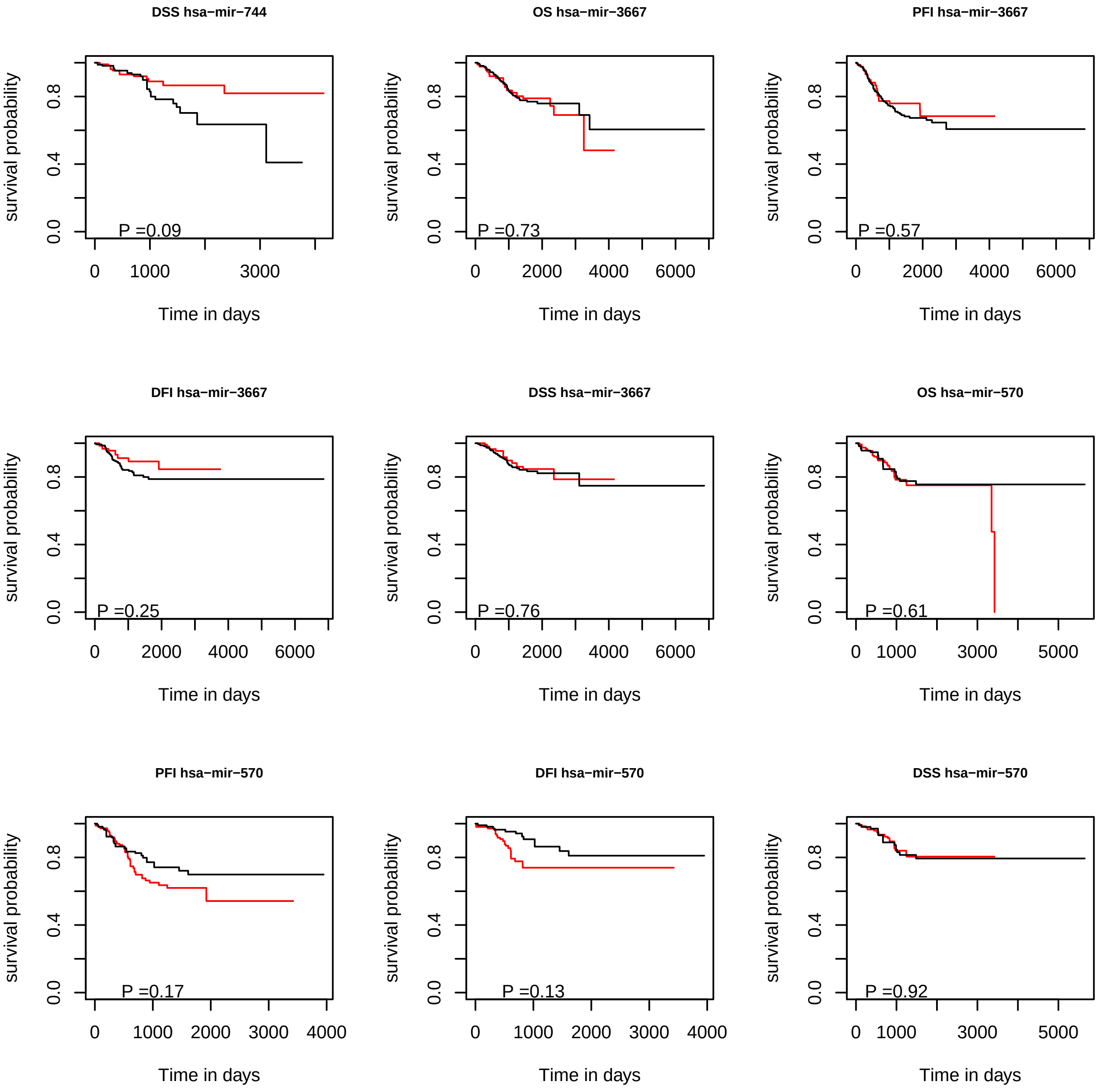
DSS hsa−mir−744
P =0.09
OS hsa−mir−3667
P =0.73
PFI hsa−mir−3667
P =0.57
DFI hsa−mir−3667
P =0.25
DSS hsa−mir−3667
P =0.76
OS hsa−mir−570
P =0.61
PFI hsa−mir−570
P =0.17
DFI hsa−mir−570
P =0.13
DSS hsa−mir−570
P =0.92
survival probability
Time in days

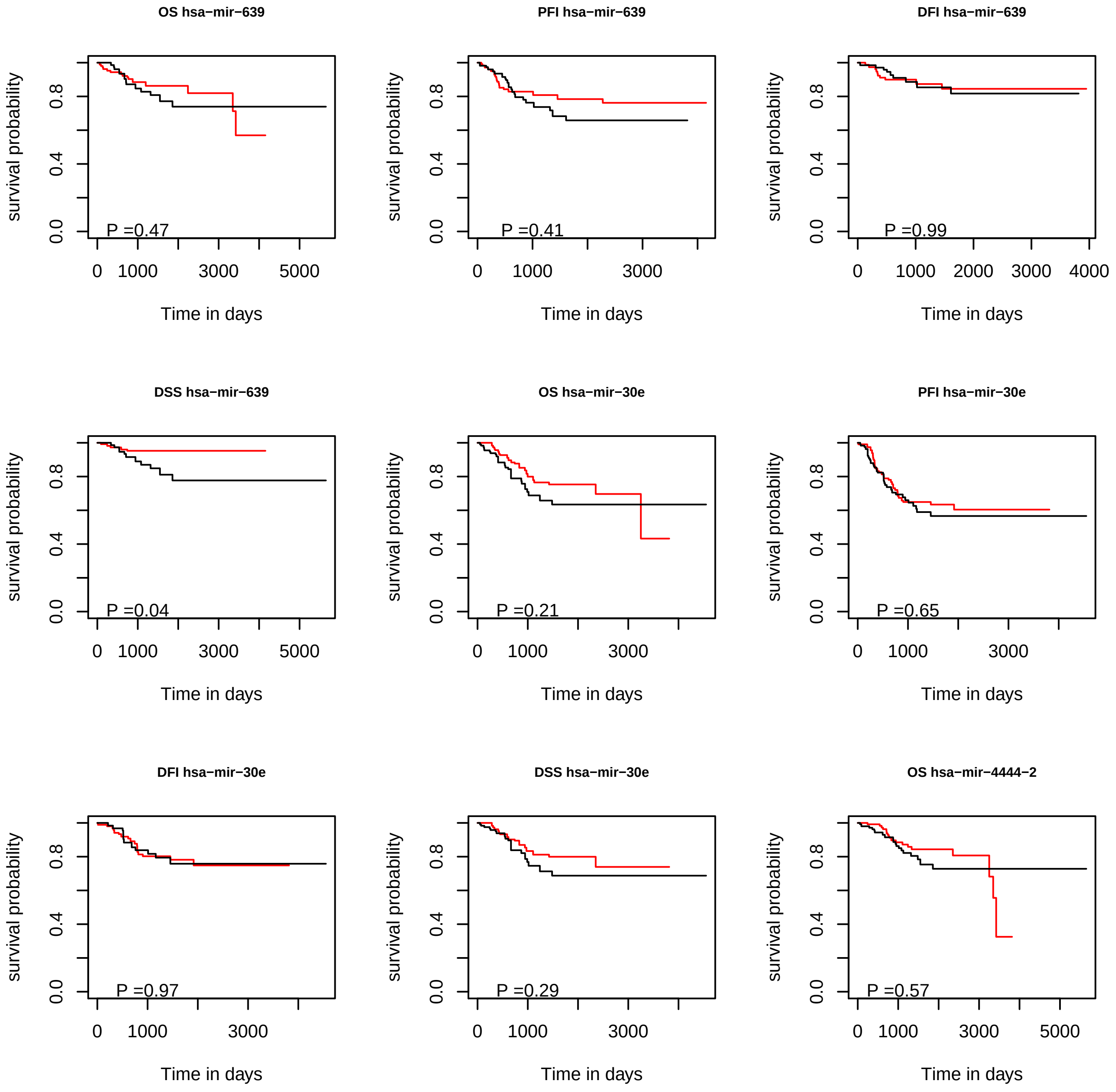

OS hsa−mir−639
P =0.47
PFI hsa−mir−639
P =0.41
DFI hsa−mir−639
P =0.99
DSS hsa−mir−639
P =0.04
OS hsa−mir−30e
P =0.21
PFI hsa−mir−30e
P =0.65
DFI hsa−mir−30e
P =0.97
DSS hsa−mir−30e
P =0.29
OS hsa−mir−4444−2
P =0.57
survival probability
Time in days

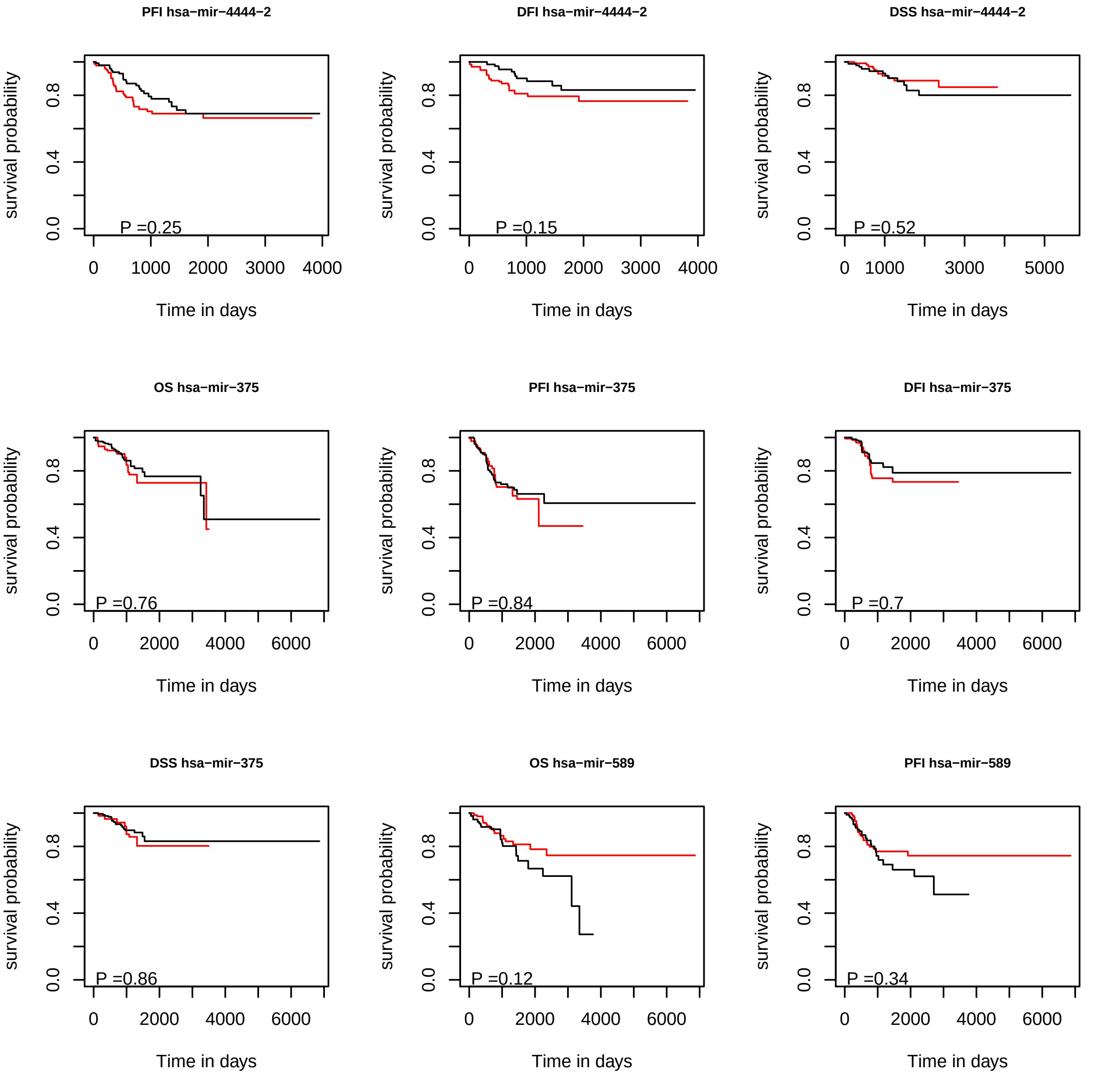
PFI hsa−mir−4444−2
P =0.25
DFI hsa−mir−4444−2
P =0.15
DSS hsa−mir−4444−2
P =0.52
OS hsa−mir−375
P =0.76
PFI hsa−mir−375
P =0.84
DFI hsa−mir−375
P =0.7
DSS hsa−mir−375
P =0.86
OS hsa−mir−589
P =0.12
PFI hsa−mir−589
P =0.34
survival probability
Time in days

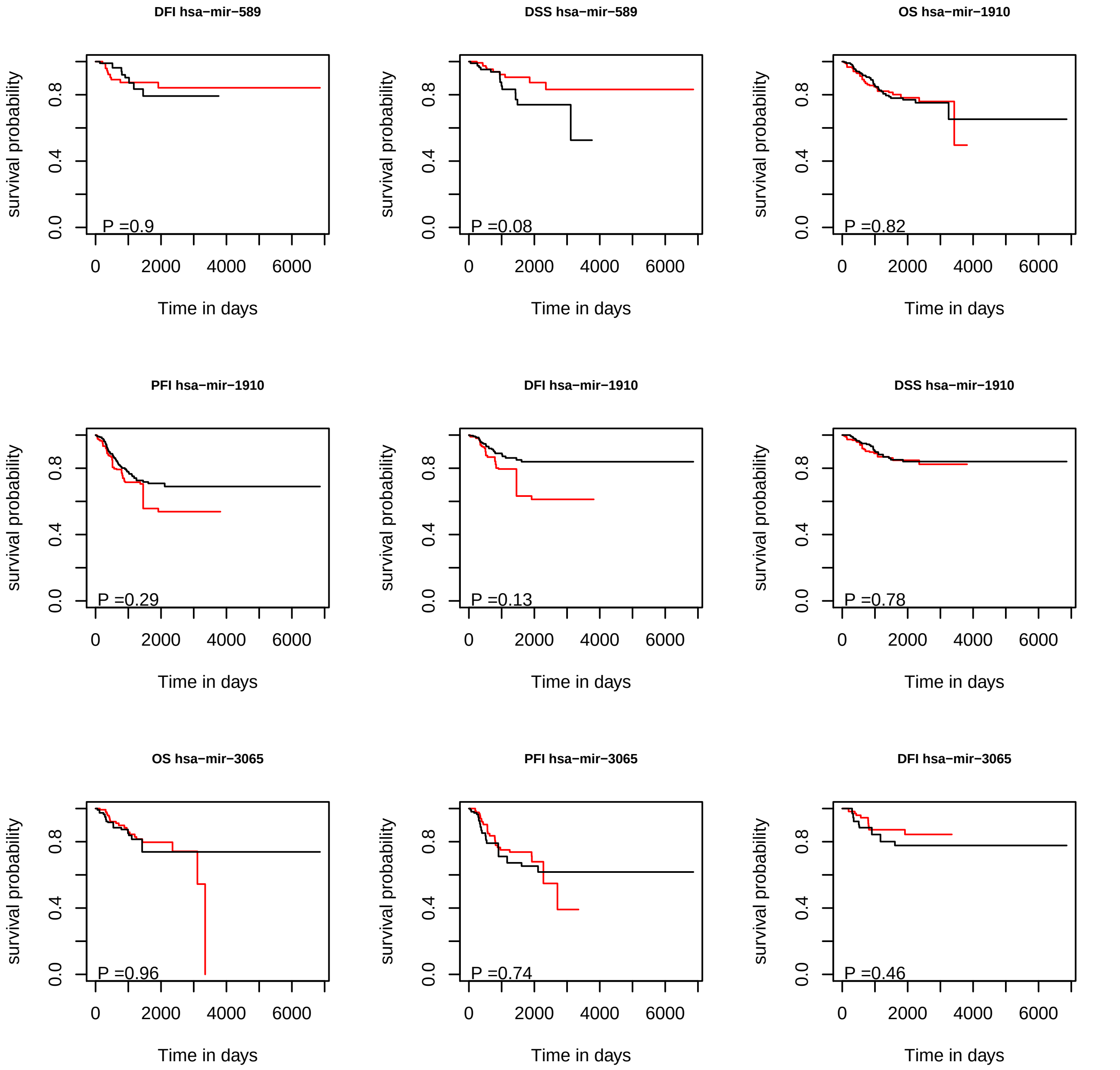

DFI hsa−mir−589
P =0.9
DSS hsa−mir−589
P =0.08
OS hsa−mir−1910
P =0.82
PFI hsa−mir−1910
P =0.29
DFI hsa−mir−1910
P =0.13
DSS hsa−mir−1910
P =0.78
OS hsa−mir−3065
P =0.96
PFI hsa−mir−3065
P =0.74
DFI hsa−mir−3065
P =0.46
survival probability
0.0
0.4
0.8
Time in days
0
2000
4000
6000

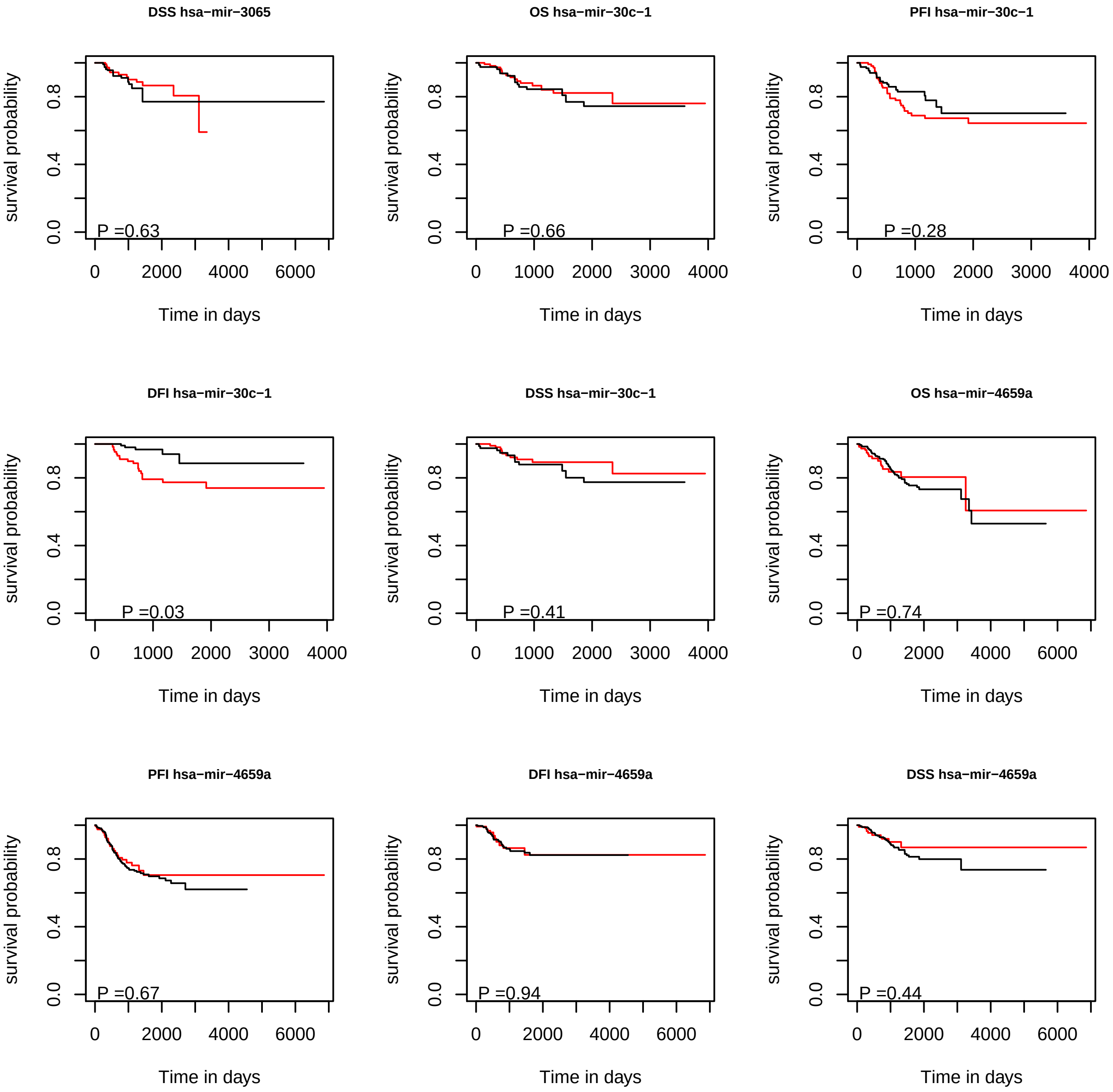
DSS hsa−mir−3065
P =0.63
OS hsa−mir−30c−1
P =0.66
PFI hsa−mir−30c−1
P =0.28
DFI hsa−mir−30c−1
P =0.03
DSS hsa−mir−30c−1
P =0.41
OS hsa−mir−4659a
P =0.74
PFI hsa−mir−4659a
P =0.67
DFI hsa−mir−4659a
P =0.94
DSS hsa−mir−4659a
P =0.44
survival probability
Time in days

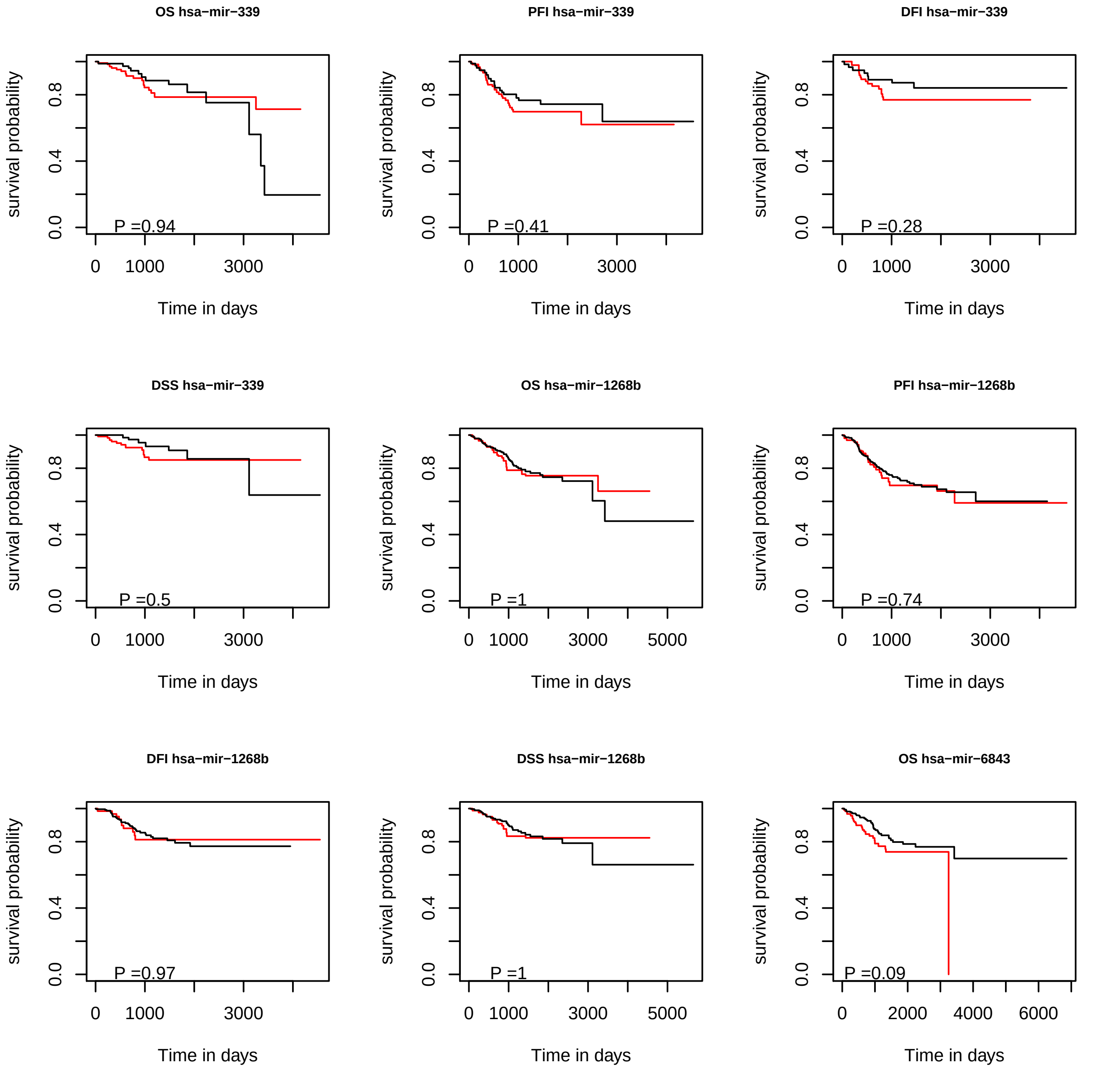
OS hsa−mir−339
P =0.94
PFI hsa−mir−339
P =0.41
DFI hsa−mir−339
P =0.28
DSS hsa−mir−339
P =0.5
OS hsa−mir−1268b
P =1
PFI hsa−mir−1268b
P =0.74
DFI hsa−mir−1268b
P =0.97
DSS hsa−mir−1268b
P =1
OS hsa−mir−6843
P =0.09
survival probability
Time in days
0.0
0.4
0.8
0
1000
2000
3000
4000
5000
6000

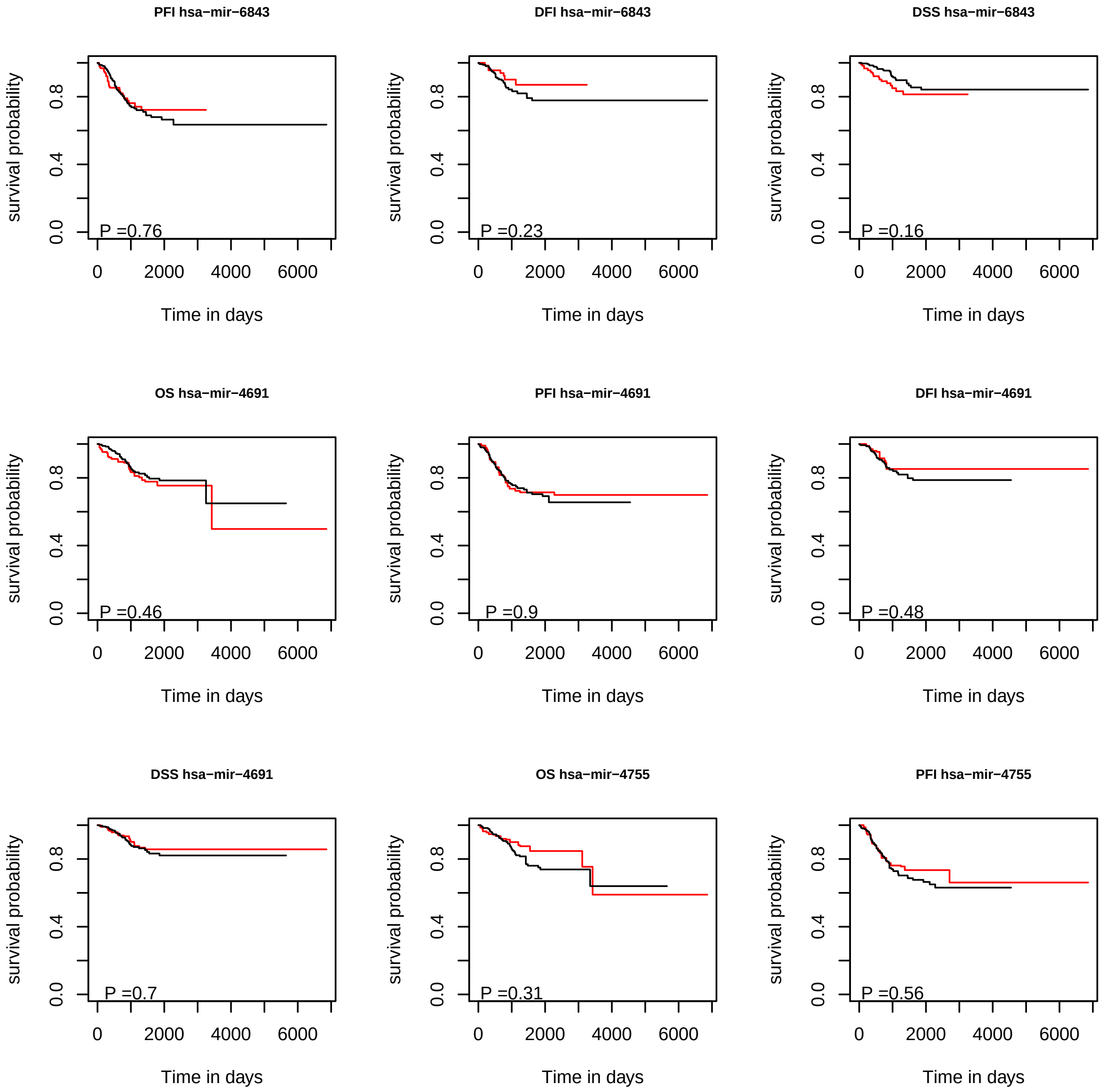
PFI hsa−mir−6843
P =0.76
DFI hsa−mir−6843
P =0.23
DSS hsa−mir−6843
P =0.16
OS hsa−mir−4691
P =0.46
PFI hsa−mir−4691
P =0.9
DFI hsa−mir−4691
P =0.48
DSS hsa−mir−4691
P =0.7
OS hsa−mir−4755
P =0.31
PFI hsa−mir−4755
P =0.56
survival probability
0.0
0.4
0.8
Time in days
0
2000
4000
6000

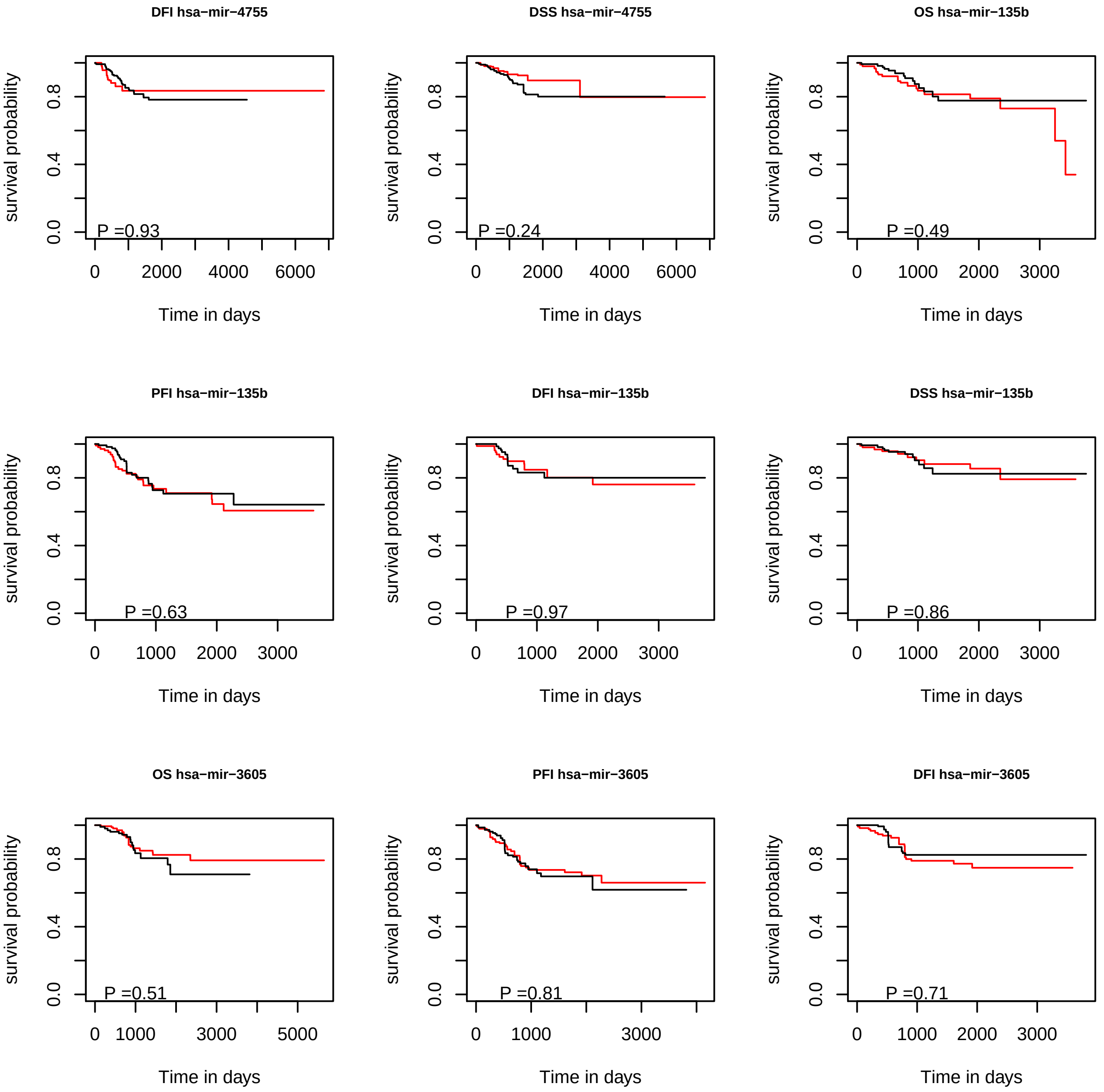
DFI hsa−mir−4755
P =0.93
DSS hsa−mir−4755
P =0.24
OS hsa−mir−135b
P =0.49
PFI hsa−mir−135b
P =0.63
DFI hsa−mir−135b
P =0.97
DSS hsa−mir−135b
P =0.86
OS hsa−mir−3605
P =0.51
PFI hsa−mir−3605
P =0.81
DFI hsa−mir−3605
P =0.71
survival probability
Time in days
0.0
0.4
0.8
0
1000
2000
3000
4000
5000
6000

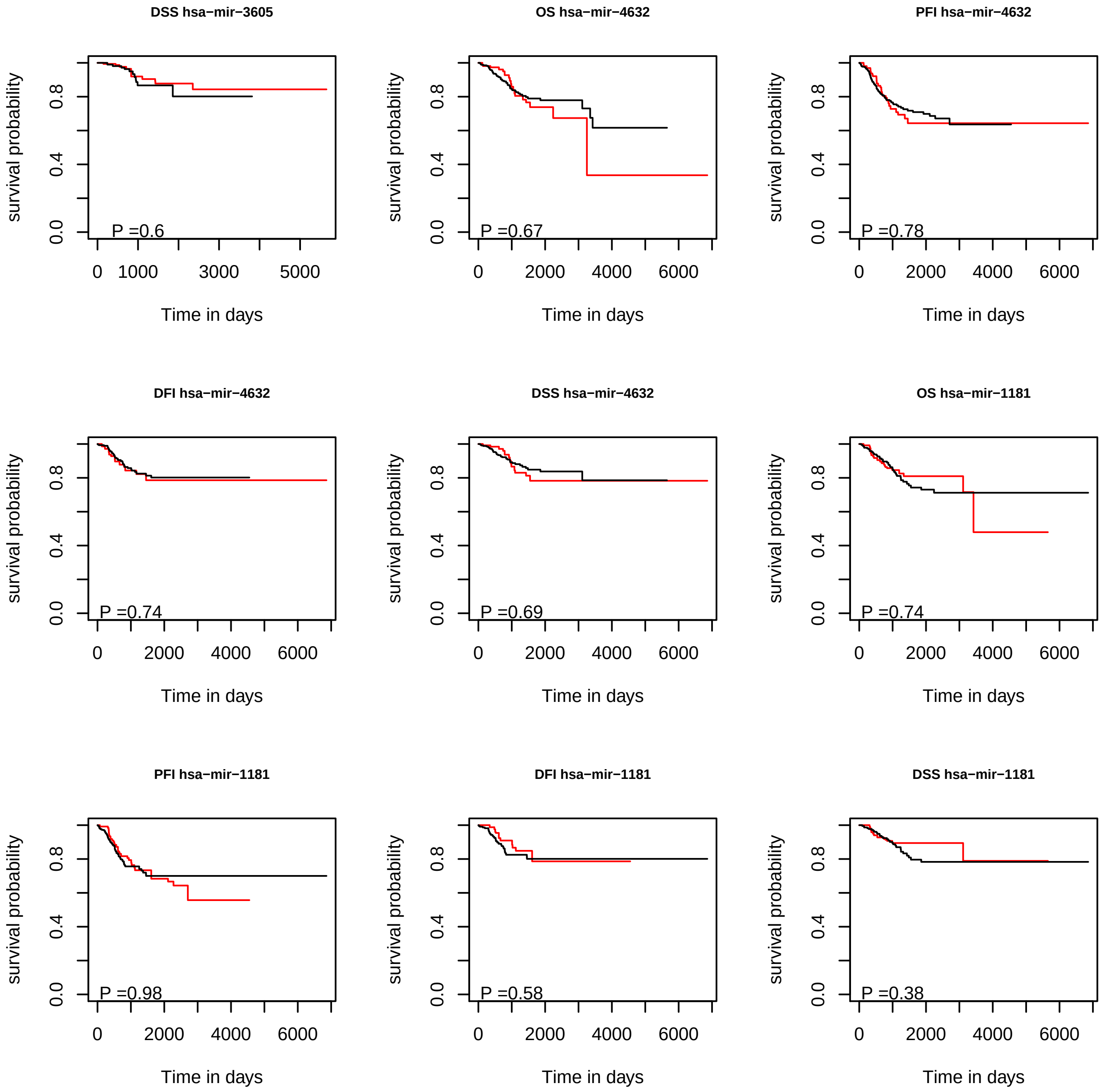
DSS hsa−mir−3605
P =0.6
OS hsa−mir−4632
P =0.67
PFI hsa−mir−4632
P =0.78
DFI hsa−mir−4632
P =0.74
DSS hsa−mir−4632
P =0.69
OS hsa−mir−1181
P =0.74
PFI hsa−mir−1181
P =0.98
DFI hsa−mir−1181
P =0.58
DSS hsa−mir−1181
P =0.38
survival probability
Time in days

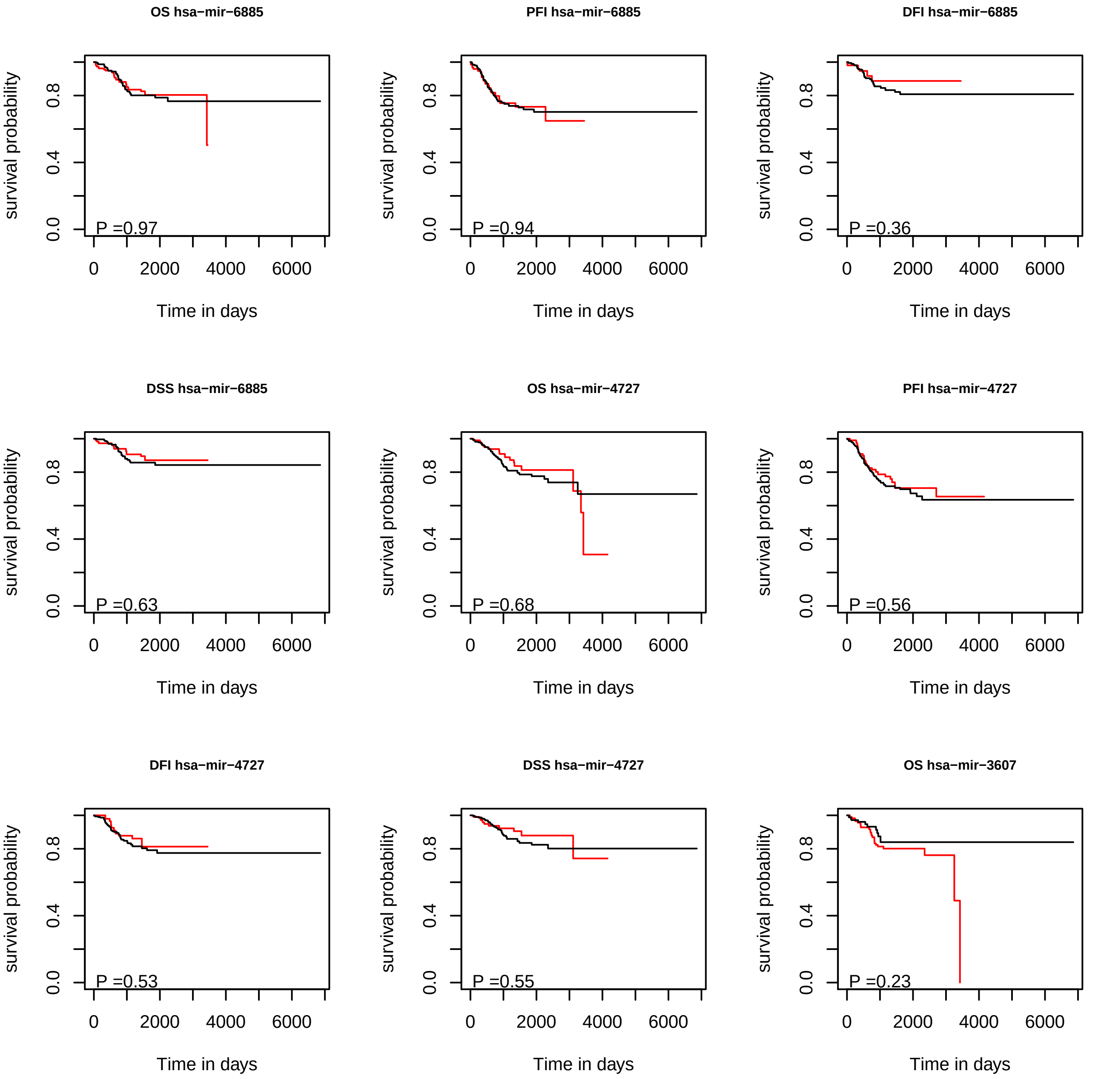
OS hsa−mir−6885
P =0.97
PFI hsa−mir−6885
P =0.94
DFI hsa−mir−6885
P =0.36
DSS hsa−mir−6885
P =0.63
OS hsa−mir−4727
P =0.68
PFI hsa−mir−4727
P =0.56
DFI hsa−mir−4727
P =0.53
DSS hsa−mir−4727
P =0.55
OS hsa−mir−3607
P =0.23
survival probability
Time in days
0.0
0.4
0.8
0
2000
4000
6000

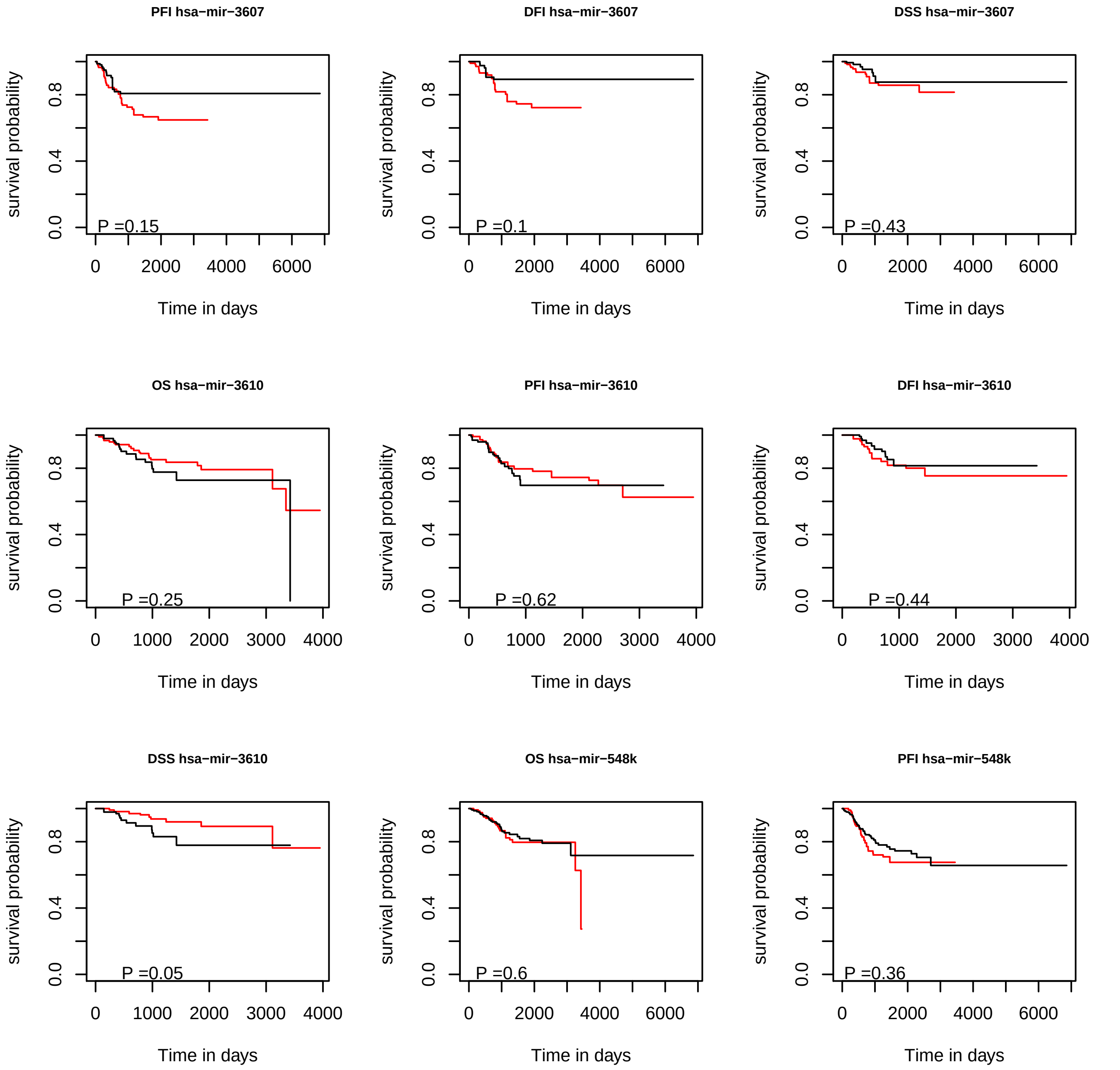
PFI hsa−mir−3607
P =0.15
DFI hsa−mir−3607
P =0.1
DSS hsa−mir−3607
P =0.43
OS hsa−mir−3610
P =0.25
PFI hsa−mir−3610
P =0.62
DFI hsa−mir−3610
P =0.44
DSS hsa−mir−3610
P =0.05
OS hsa−mir−548k
P =0.6
PFI hsa−mir−548k
P =0.36
survival probability
Time in days

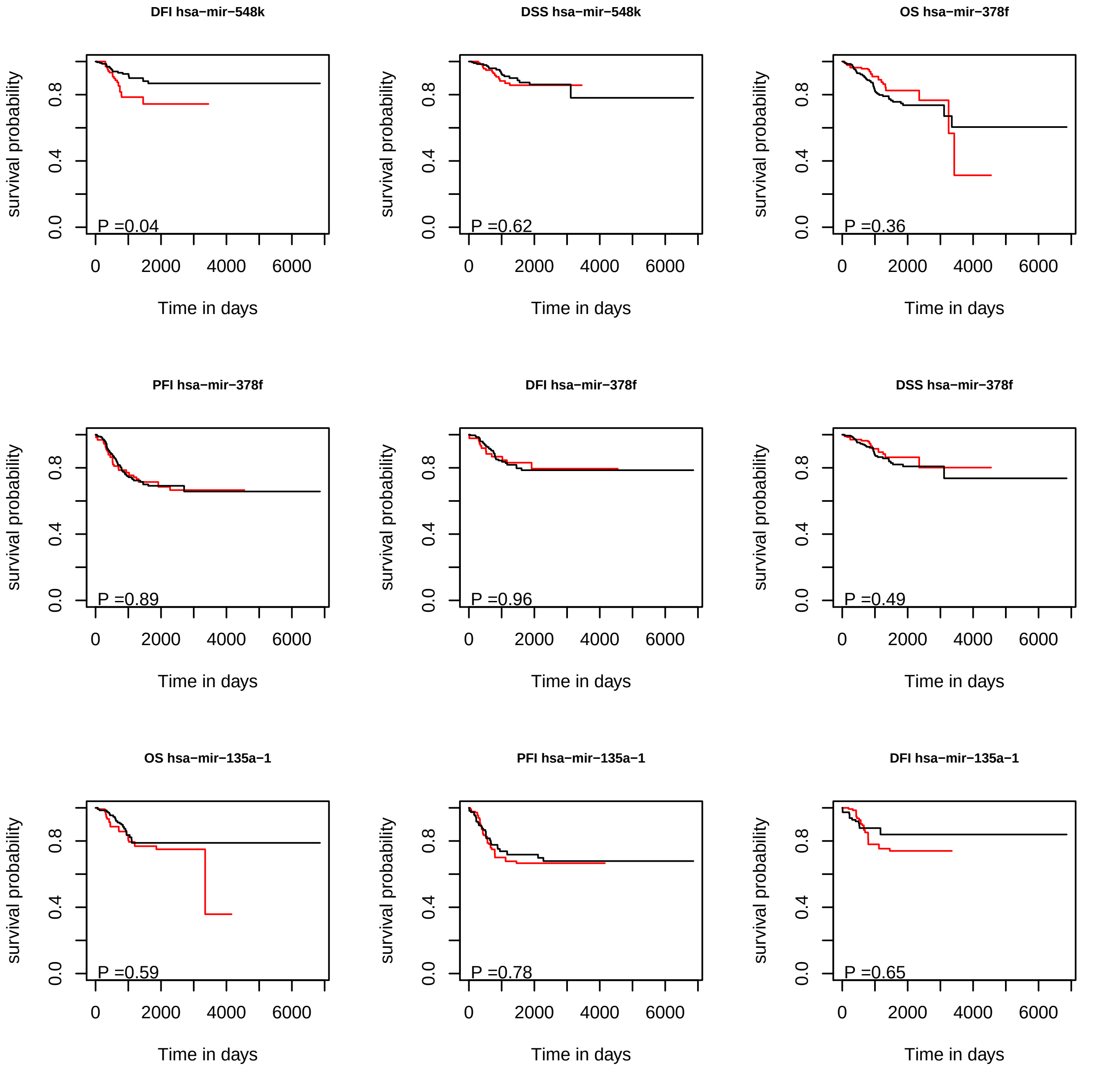
DFI hsa−mir−548k
P =0.04
DSS hsa−mir−548k
P =0.62
OS hsa−mir−378f
P =0.36
PFI hsa−mir−378f
P =0.89
DFI hsa−mir−378f
P =0.96
DSS hsa−mir−378f
P =0.49
OS hsa−mir−135a−1
P =0.59
PFI hsa−mir−135a−1
P =0.78
DFI hsa−mir−135a−1
P =0.65
survival probability
Time in days
0.0
0.4
0.8
0
2000
4000
6000

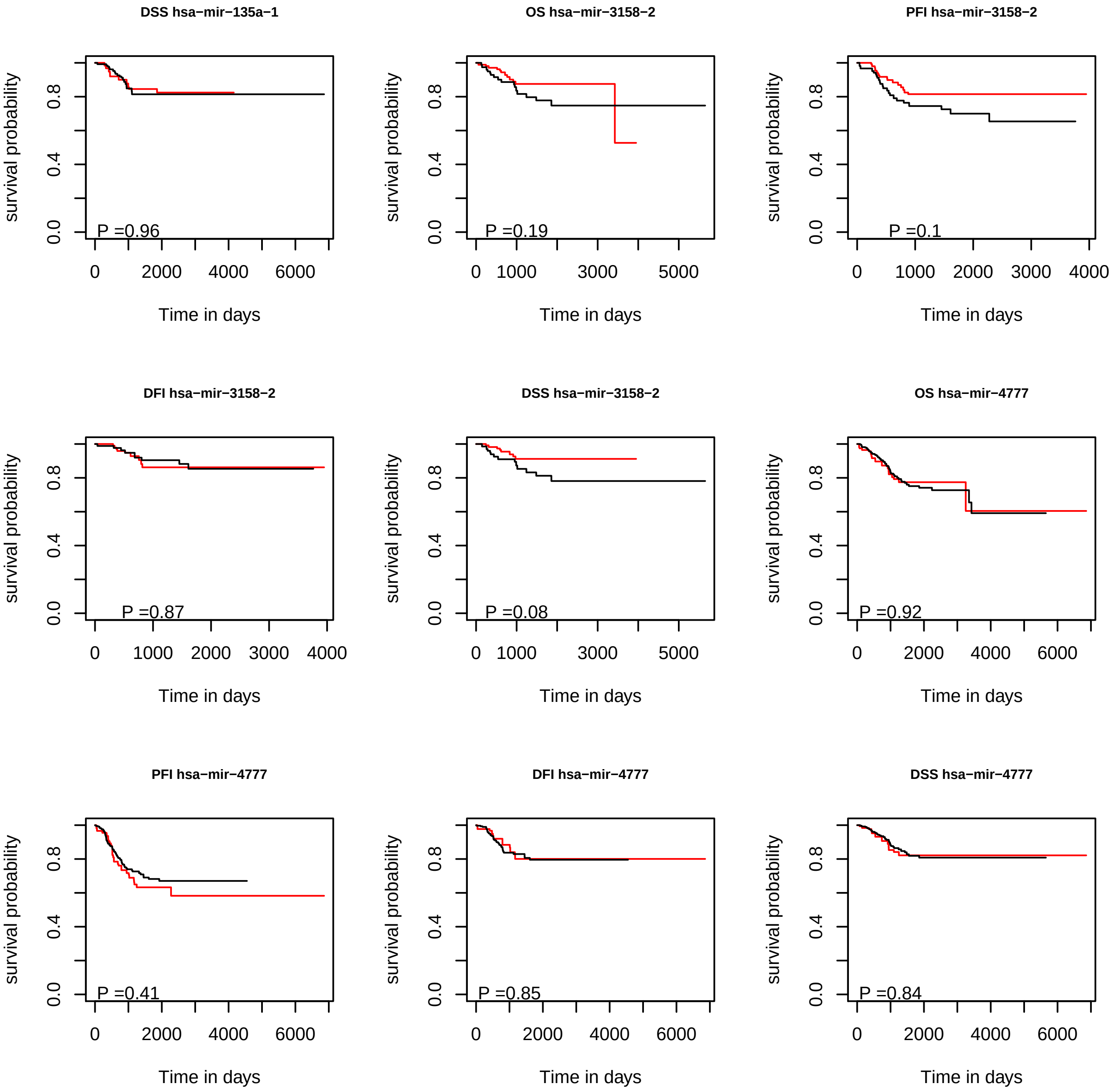
DSS hsa−mir−135a−1
P =0.96
OS hsa−mir−3158−2
P =0.19
PFI hsa−mir−3158−2
P =0.1
DFI hsa−mir−3158−2
P =0.87
DSS hsa−mir−3158−2
P =0.08
OS hsa−mir−4777
P =0.92
PFI hsa−mir−4777
P =0.41
DFI hsa−mir−4777
P =0.85
DSS hsa−mir−4777
P =0.84
survival probability
Time in days
0.0
0.4
0.8
0
1000
2000
3000
4000
5000
6000

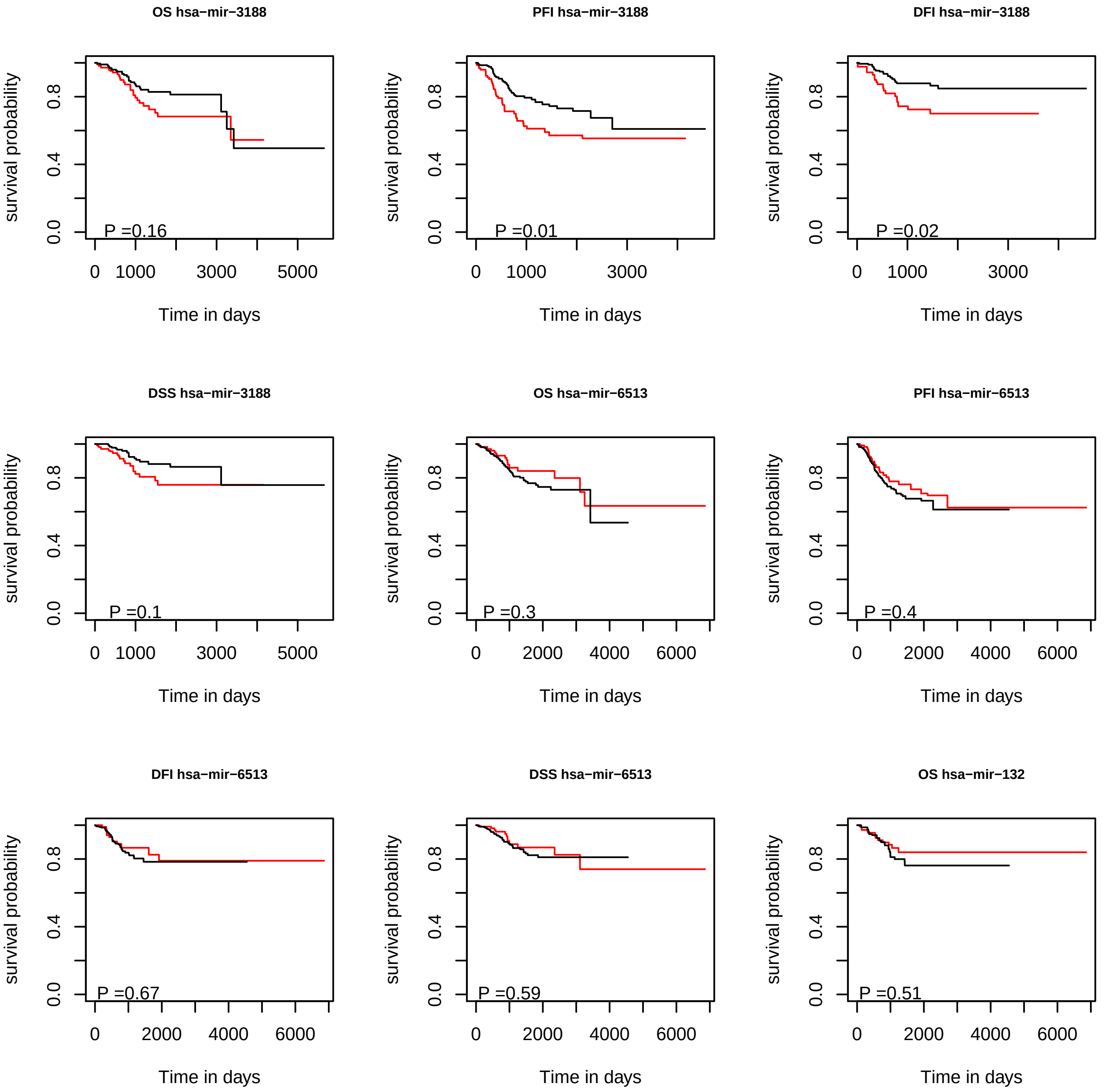
OS hsa−mir−3188
P =0.16
PFI hsa−mir−3188
P =0.01
DFI hsa−mir−3188
P =0.02
DSS hsa−mir−3188
P =0.1
OS hsa−mir−6513
P =0.3
PFI hsa−mir−6513
P =0.4
DFI hsa−mir−6513
P =0.67
DSS hsa−mir−6513
P =0.59
OS hsa−mir−132
P =0.51
survival probability
Time in days
0.0
0.4
0.8
0
1000
2000
3000
4000
5000
6000

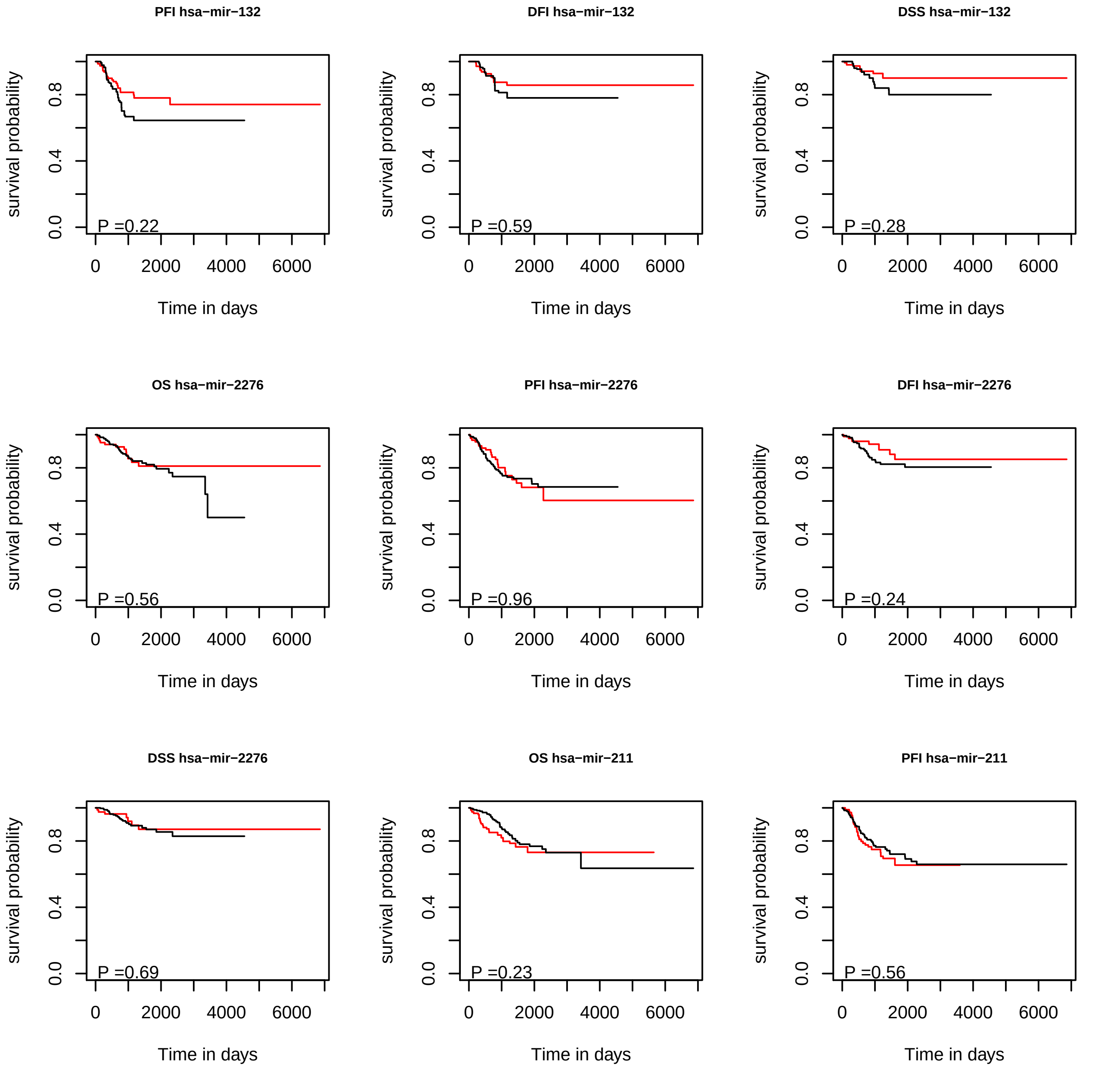
PFI hsa−mir−132
P =0.22
DFI hsa−mir−132
P =0.59
DSS hsa−mir−132
P =0.28
OS hsa−mir−2276
P =0.56
PFI hsa−mir−2276
P =0.96
DFI hsa−mir−2276
P =0.24
DSS hsa−mir−2276
P =0.69
OS hsa−mir−211
P =0.23
PFI hsa−mir−211
P =0.56
survival probability
Time in days
0.0
0.4
0.8
0
2000
4000
6000

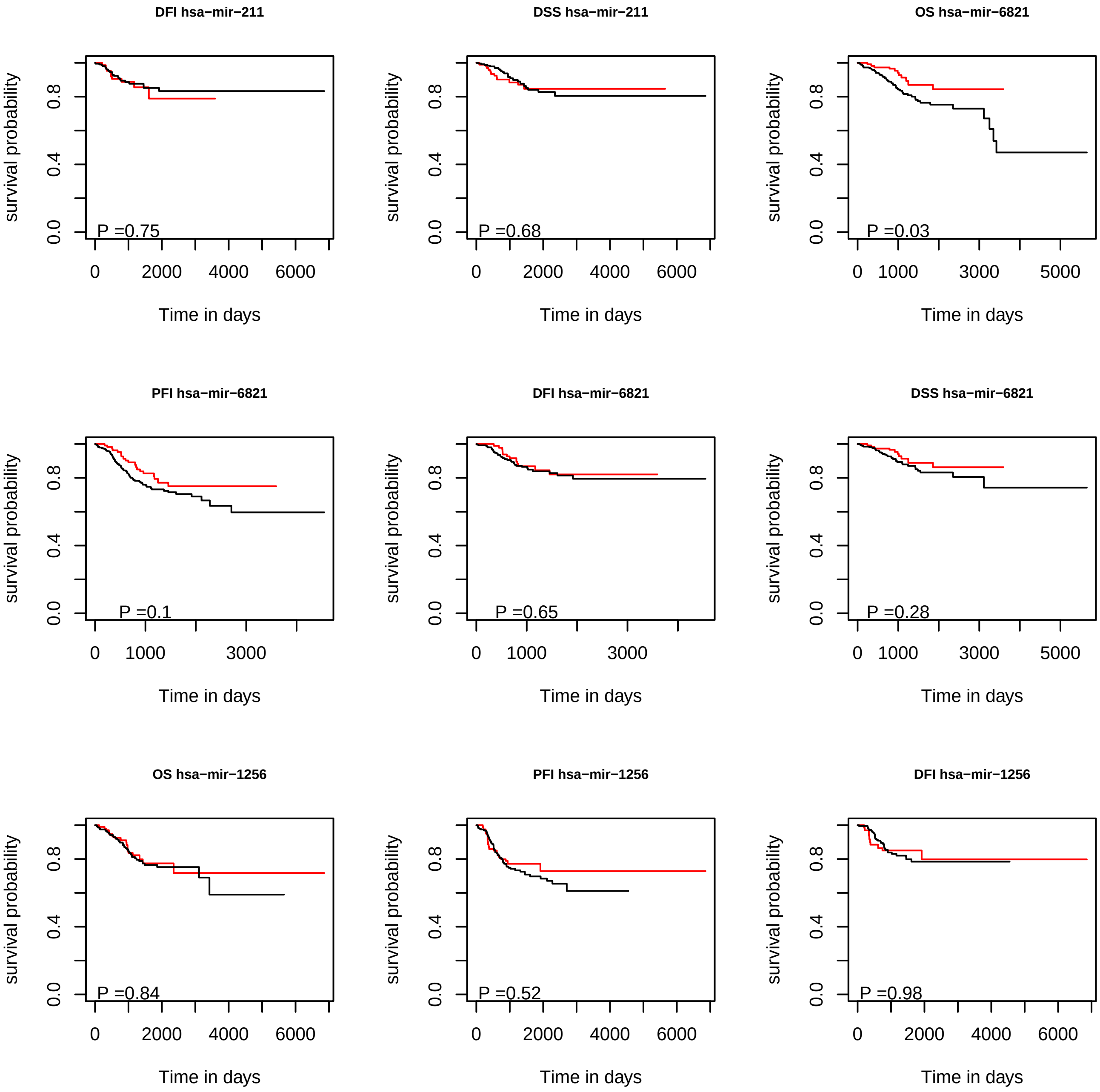
DFI hsa−mir−211
survival probability
0.0 0.4 0.8
P =0.75
0 2000 4000 6000
Time in days
DSS hsa−mir−211
survival probability
0.0 0.4 0.8
P =0.68
0 2000 4000 6000
Time in days
OS hsa−mir−6821
survival probability
0.0 0.4 0.8
P =0.03
0 1000 3000 5000
Time in days
PFI hsa−mir−6821
survival probability
0.0 0.4 0.8
P =0.1
0 1000 3000
Time in days
DFI hsa−mir−6821
survival probability
0.0 0.4 0.8
P =0.65
0 1000 3000
Time in days
DSS hsa−mir−6821
survival probability
0.0 0.4 0.8
P =0.28
0 1000 3000 5000
Time in days
OS hsa−mir−1256
survival probability
0.0 0.4 0.8
P =0.84
0 2000 4000 6000
Time in days
PFI hsa−mir−1256
survival probability
0.0 0.4 0.8
P =0.52
0 2000 4000 6000
Time in days
DFI hsa−mir−1256
survival probability
0.0 0.4 0.8
P =0.98
0 2000 4000 6000
Time in days

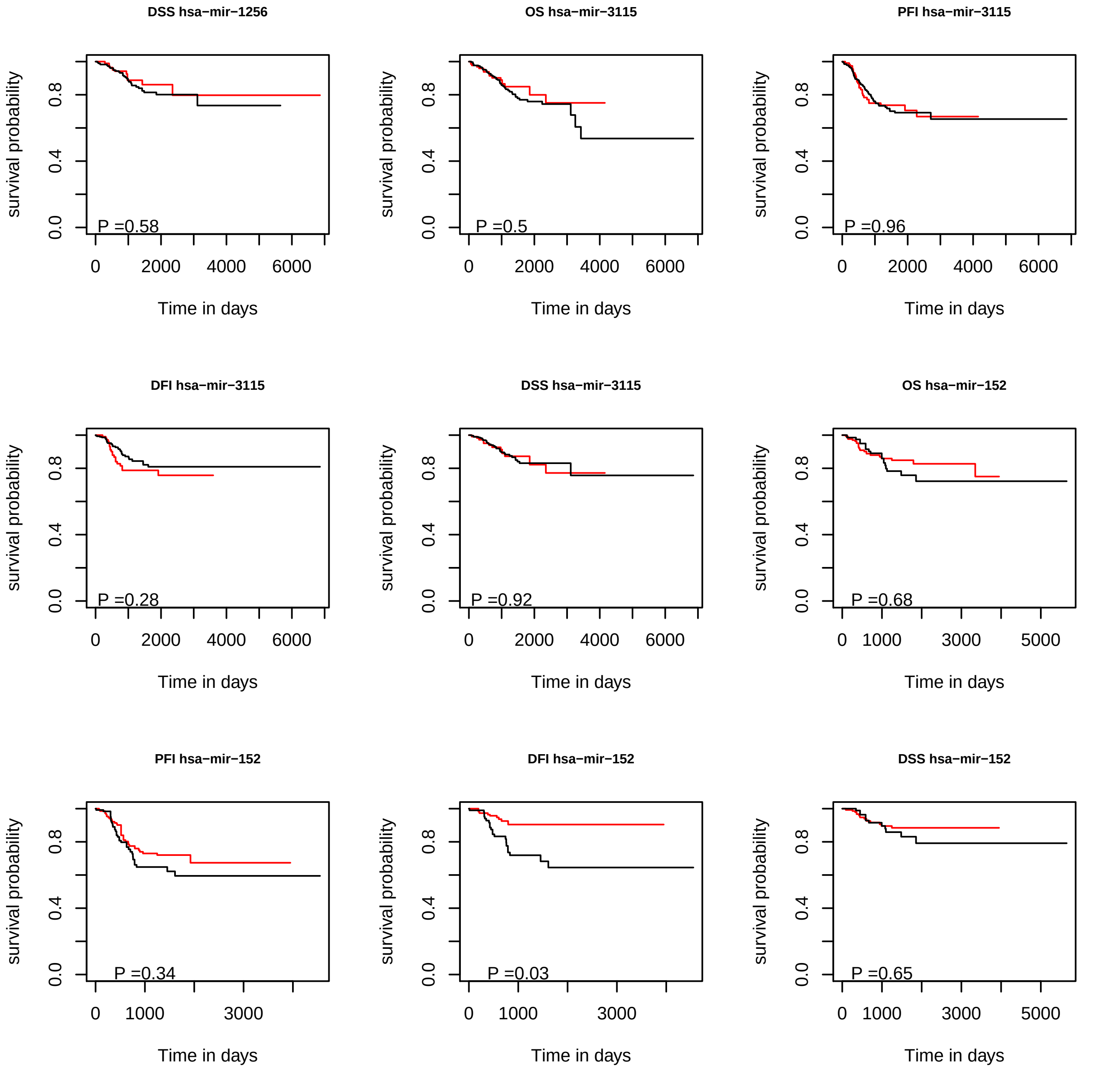
DSS hsa−mir−1256
OS hsa−mir−3115
PFI hsa−mir−3115
DFI hsa−mir−3115
DSS hsa−mir−3115
OS hsa−mir−152
PFI hsa−mir−152
DFI hsa−mir−152
DSS hsa−mir−152
survival probability
Time in days
0.0
0.4
0.8
0
1000
2000
3000
4000
5000
6000
P =0.58
P =0.5
P =0.96
P =0.28
P =0.92
P =0.68
P =0.34
P =0.03
P =0.65

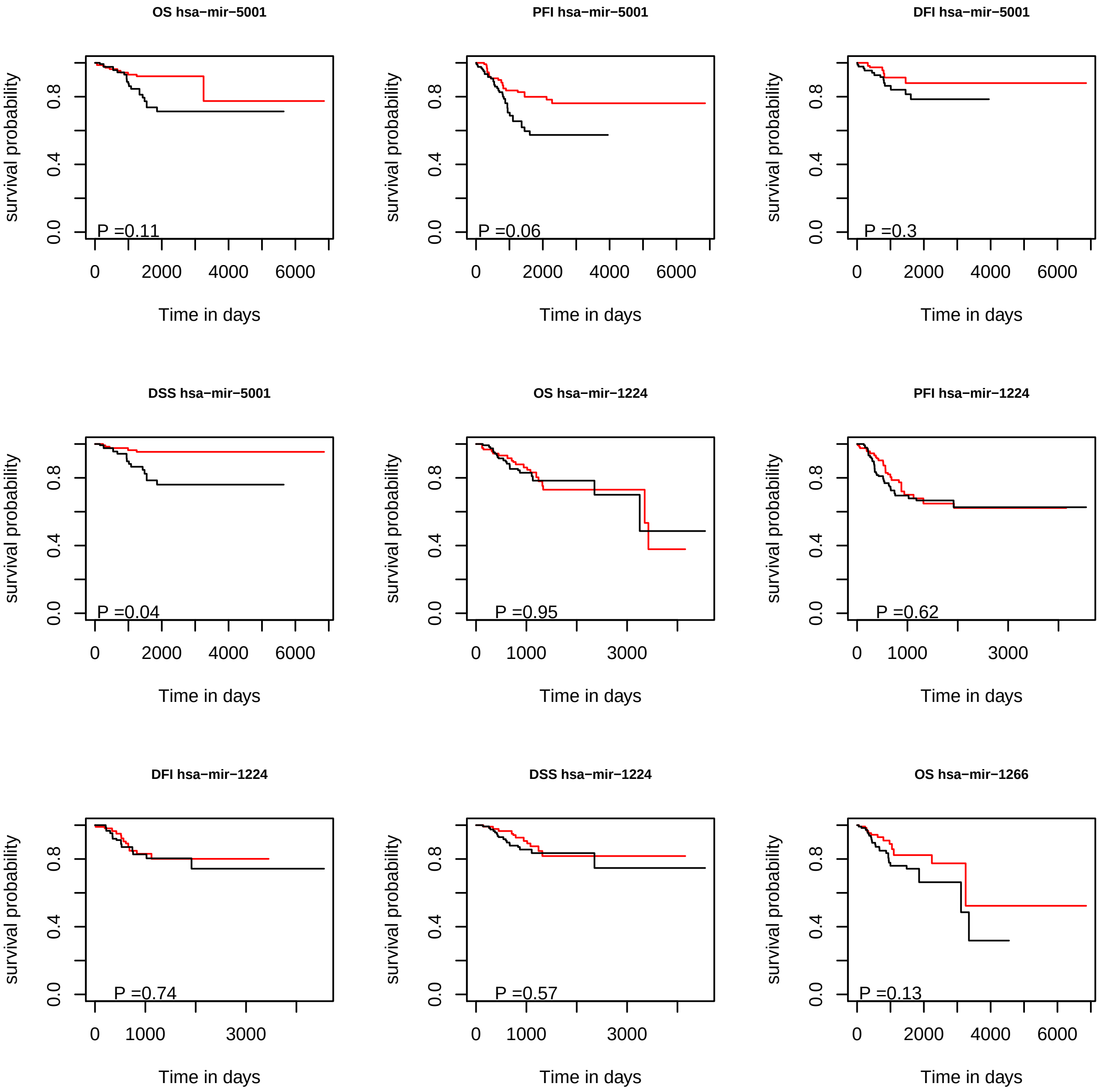
OS hsa−mir−5001
P =0.11
PFI hsa−mir−5001
P =0.06
DFI hsa−mir−5001
P =0.3
DSS hsa−mir−5001
P =0.04
OS hsa−mir−1224
P =0.95
PFI hsa−mir−1224
P =0.62
DFI hsa−mir−1224
P =0.74
DSS hsa−mir−1224
P =0.57
OS hsa−mir−1266
P =0.13
survival probability
Time in days
0.0
0.4
0.8
0
1000
2000
3000
4000
6000

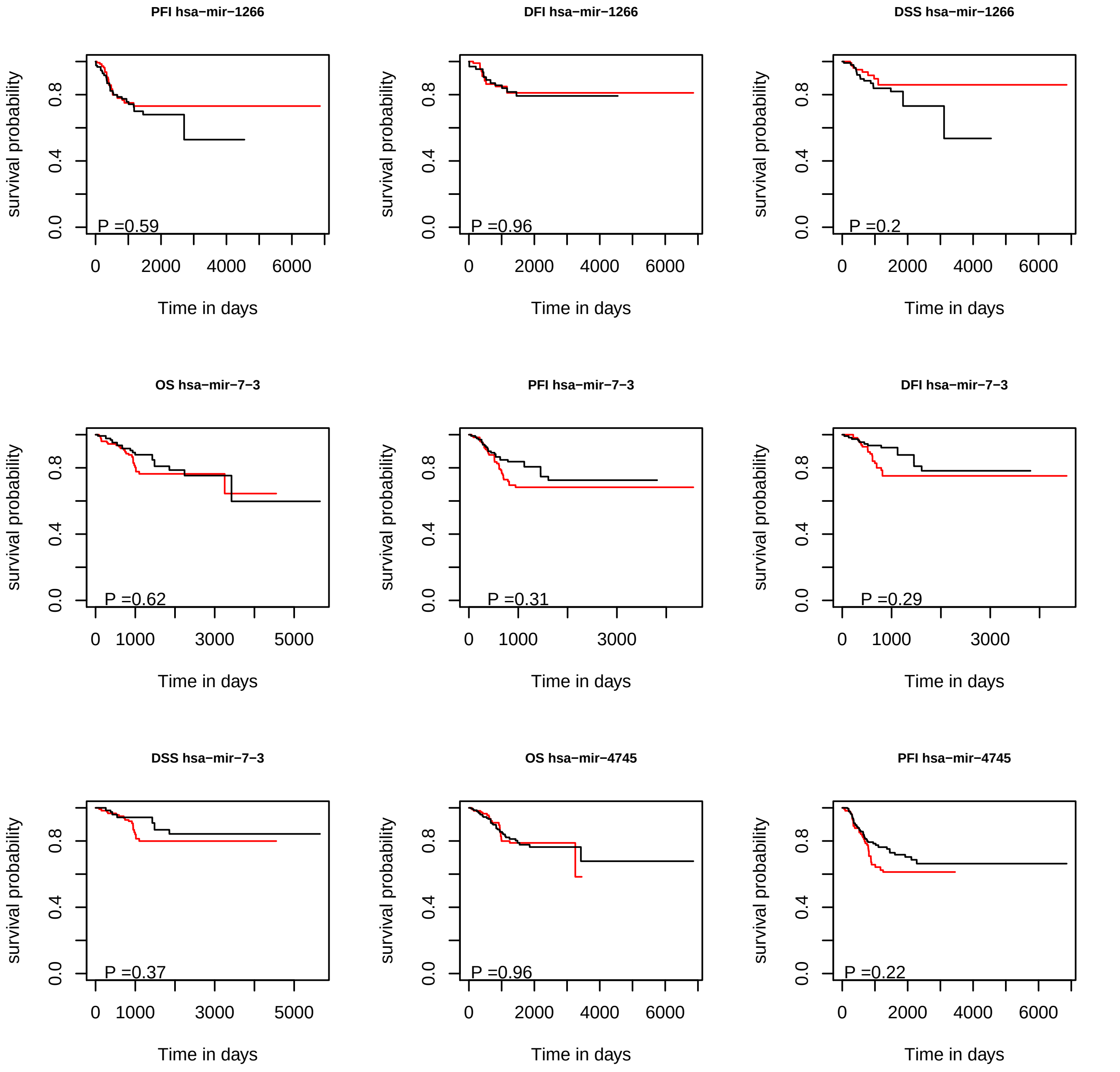
PFI hsa−mir−1266
survival probability
0.0
0.4
0.8
P =0.59
0
2000
4000
6000
Time in days
DFI hsa−mir−1266
survival probability
0.0
0.4
0.8
P =0.96
0
2000
4000
6000
Time in days
DSS hsa−mir−1266
survival probability
0.0
0.4
0.8
P =0.2
0
2000
4000
6000
Time in days
OS hsa−mir−7−3
survival probability
0.0
0.4
0.8
P =0.62
0
1000
3000
5000
Time in days
PFI hsa−mir−7−3
survival probability
0.0
0.4
0.8
P =0.31
0
1000
3000
Time in days
DFI hsa−mir−7−3
survival probability
0.0
0.4
0.8
P =0.29
0
1000
3000
Time in days
DSS hsa−mir−7−3
survival probability
0.0
0.4
0.8
P =0.37
0
1000
3000
5000
Time in days
OS hsa−mir−4745
survival probability
0.0
0.4
0.8
P =0.96
0
2000
4000
6000
Time in days
PFI hsa−mir−4745
survival probability
0.0
0.4
0.8
P =0.22
0
2000
4000
6000
Time in days

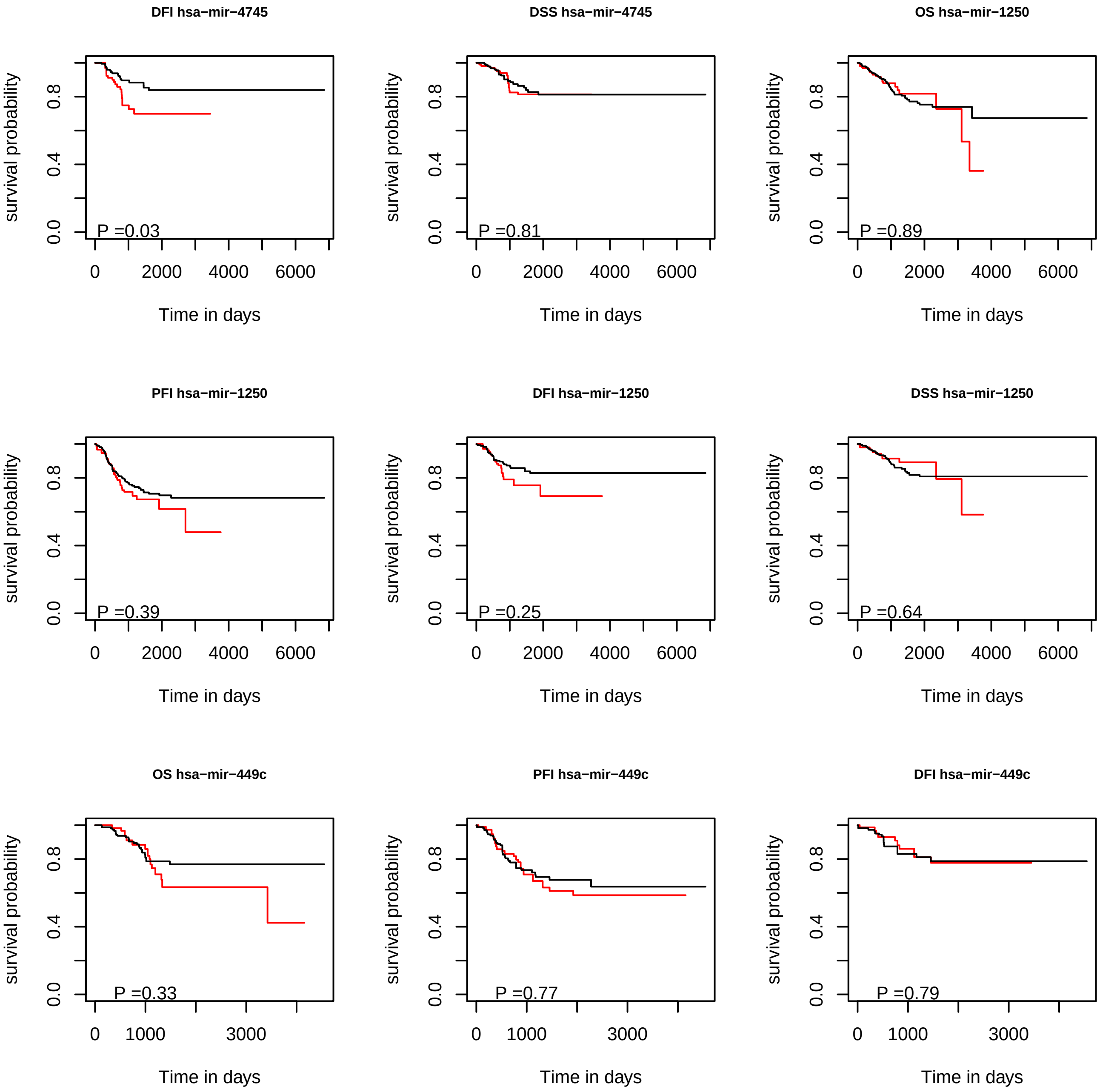
DFI hsa−mir−4745
P =0.03
DSS hsa−mir−4745
P =0.81
OS hsa−mir−1250
P =0.89
PFI hsa−mir−1250
P =0.39
DFI hsa−mir−1250
P =0.25
DSS hsa−mir−1250
P =0.64
OS hsa−mir−449c
P =0.33
PFI hsa−mir−449c
P =0.77
DFI hsa−mir−449c
P =0.79
survival probability
0.0
0.4
0.8
Time in days
0
1000
2000
3000
4000
6000

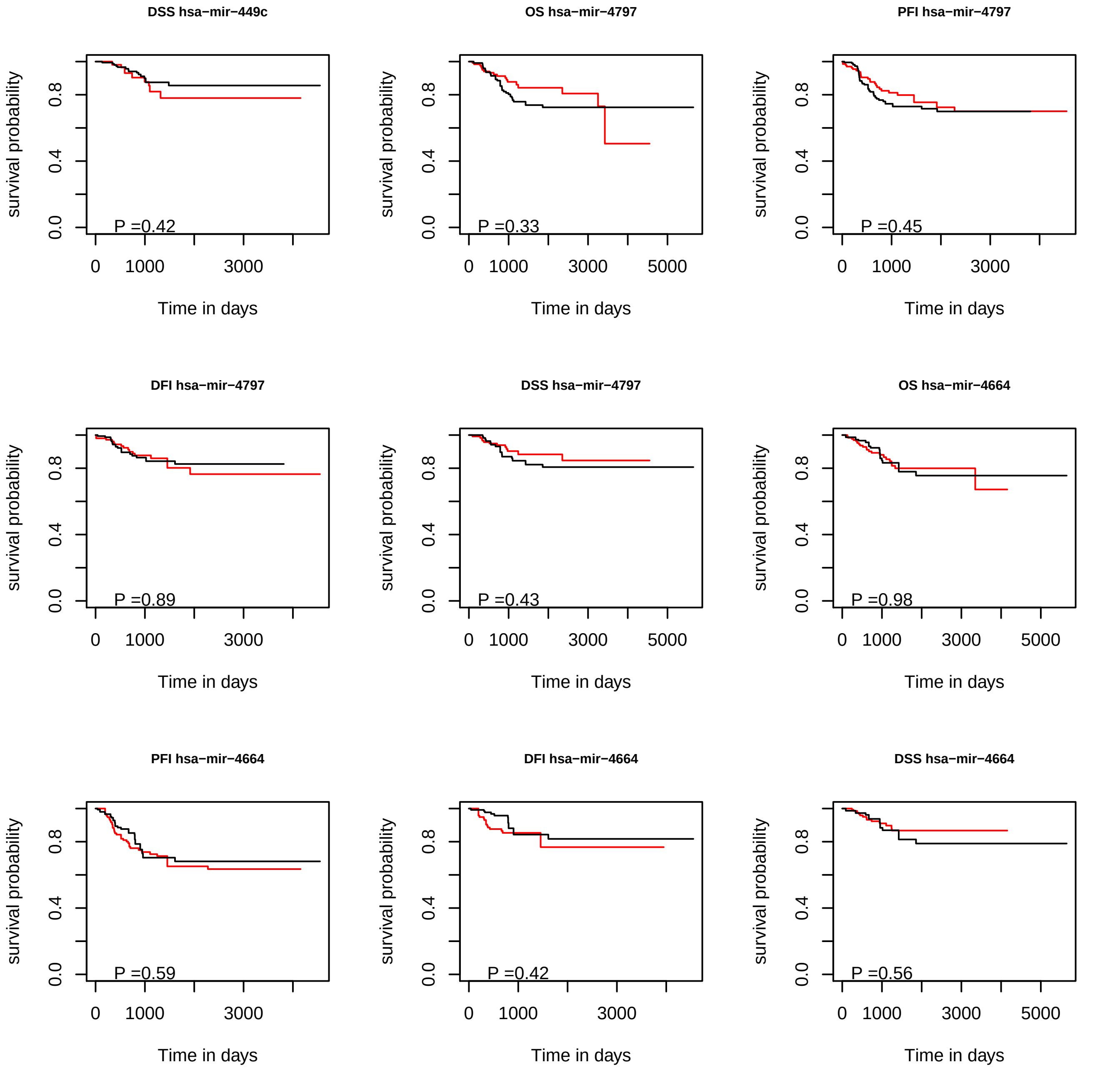
DSS hsa−mir−449c
P =0.42
OS hsa−mir−4797
P =0.33
PFI hsa−mir−4797
P =0.45
DFI hsa−mir−4797
P =0.89
DSS hsa−mir−4797
P =0.43
OS hsa−mir−4664
P =0.98
PFI hsa−mir−4664
P =0.59
DFI hsa−mir−4664
P =0.42
DSS hsa−mir−4664
P =0.56
survival probability
Time in days
0.0
0.4
0.8
0
1000
3000
5000

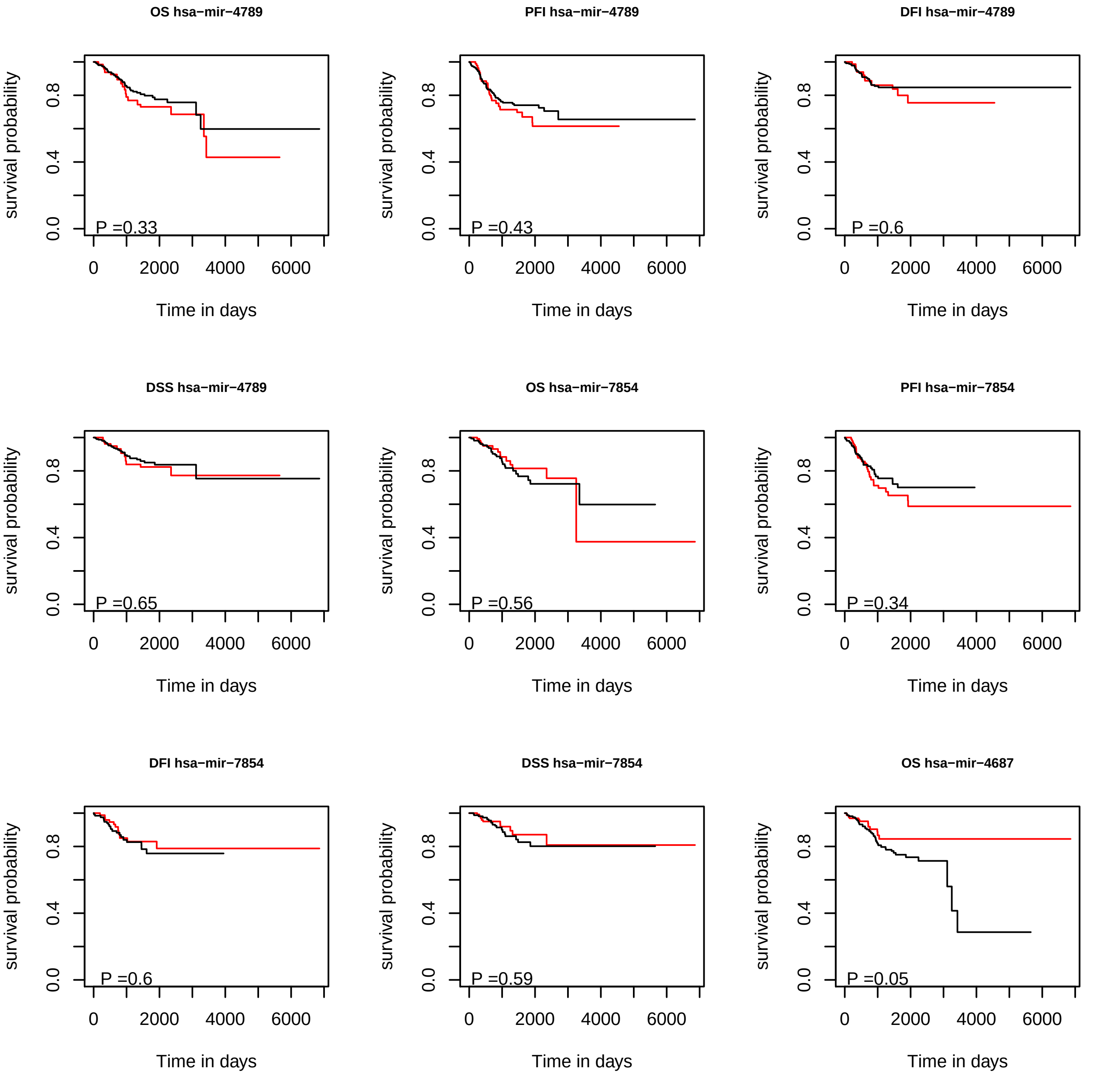
OS hsa−mir−4789
P =0.33
PFI hsa−mir−4789
P =0.43
DFI hsa−mir−4789
P =0.6
DSS hsa−mir−4789
P =0.65
OS hsa−mir−7854
P =0.56
PFI hsa−mir−7854
P =0.34
DFI hsa−mir−7854
P =0.6
DSS hsa−mir−7854
P =0.59
OS hsa−mir−4687
P =0.05
survival probability
0.0
0.4
0.8
Time in days
0
2000
4000
6000

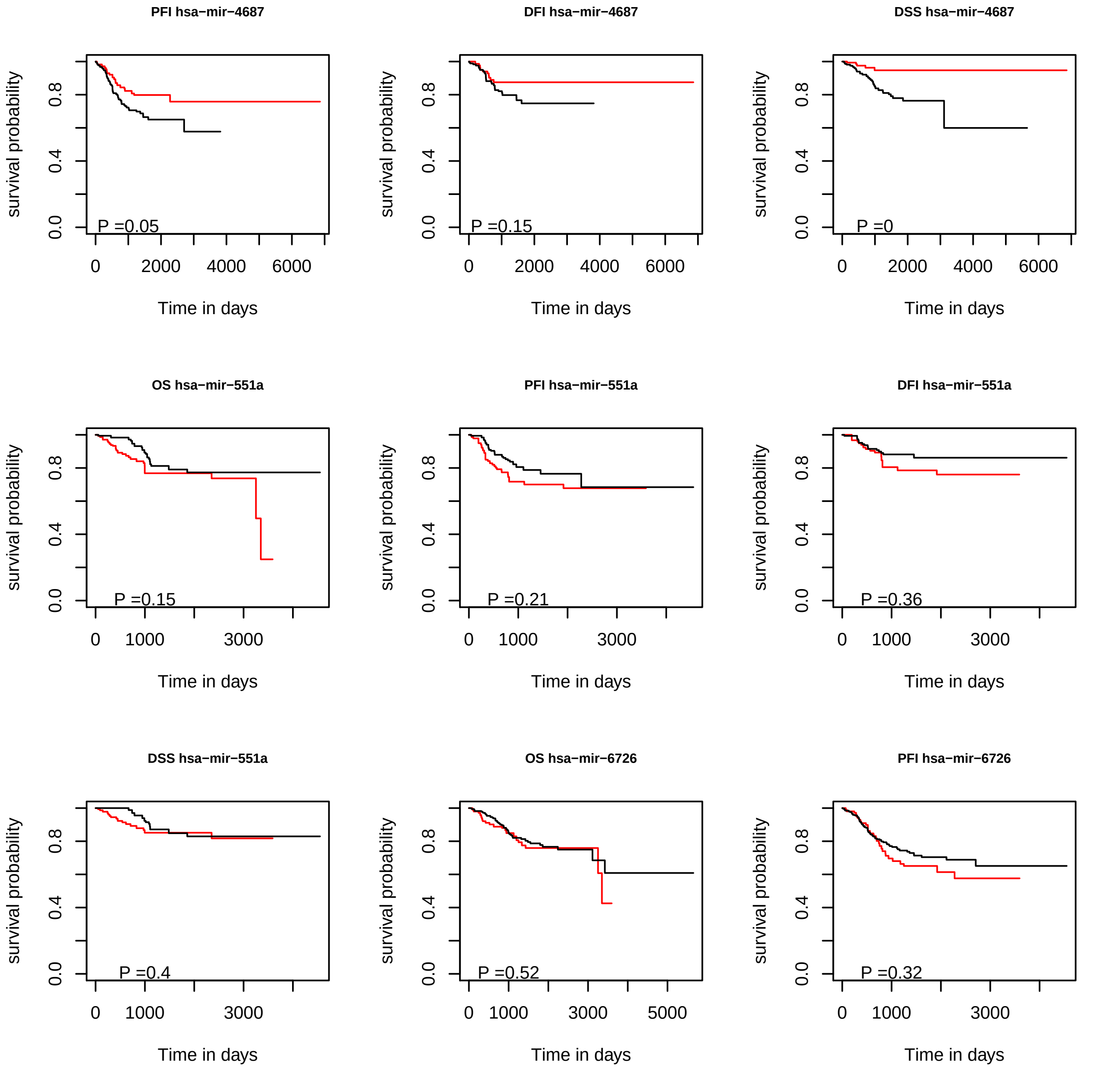
PFI hsa−mir−4687
P =0.05
DFI hsa−mir−4687
P =0.15
DSS hsa−mir−4687
P =0
OS hsa−mir−551a
P =0.15
PFI hsa−mir−551a
P =0.21
DFI hsa−mir−551a
P =0.36
DSS hsa−mir−551a
P =0.4
OS hsa−mir−6726
P =0.52
PFI hsa−mir−6726
P =0.32
survival probability
Time in days
0.0
0.4
0.8
0
1000
2000
3000
4000
5000
6000

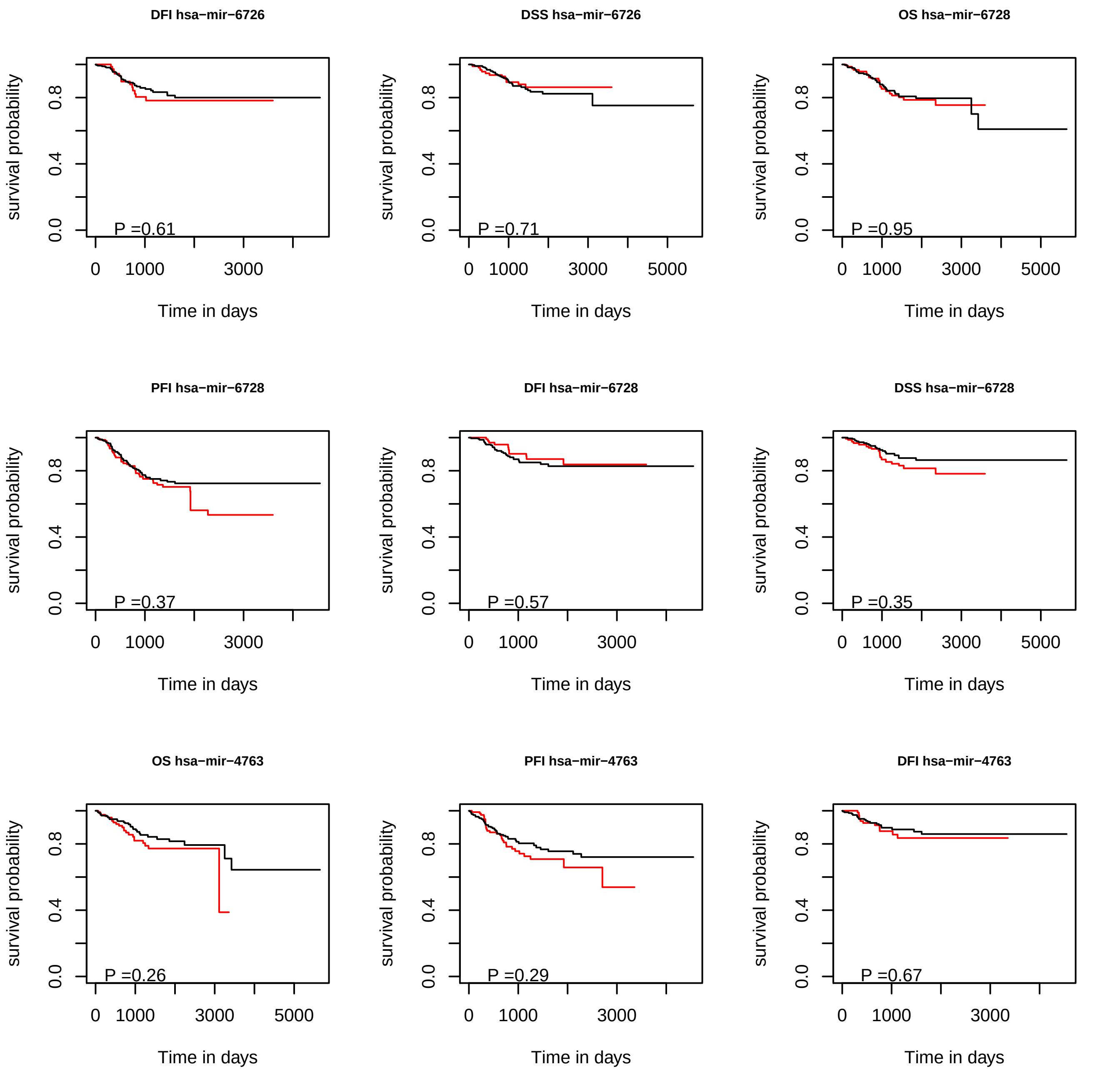
DFI hsa−mir−6726
P =0.61
DSS hsa−mir−6726
P =0.71
OS hsa−mir−6728
P =0.95
PFI hsa−mir−6728
P =0.37
DFI hsa−mir−6728
P =0.57
DSS hsa−mir−6728
P =0.35
OS hsa−mir−4763
P =0.26
PFI hsa−mir−4763
P =0.29
DFI hsa−mir−4763
P =0.67
survival probability
Time in days

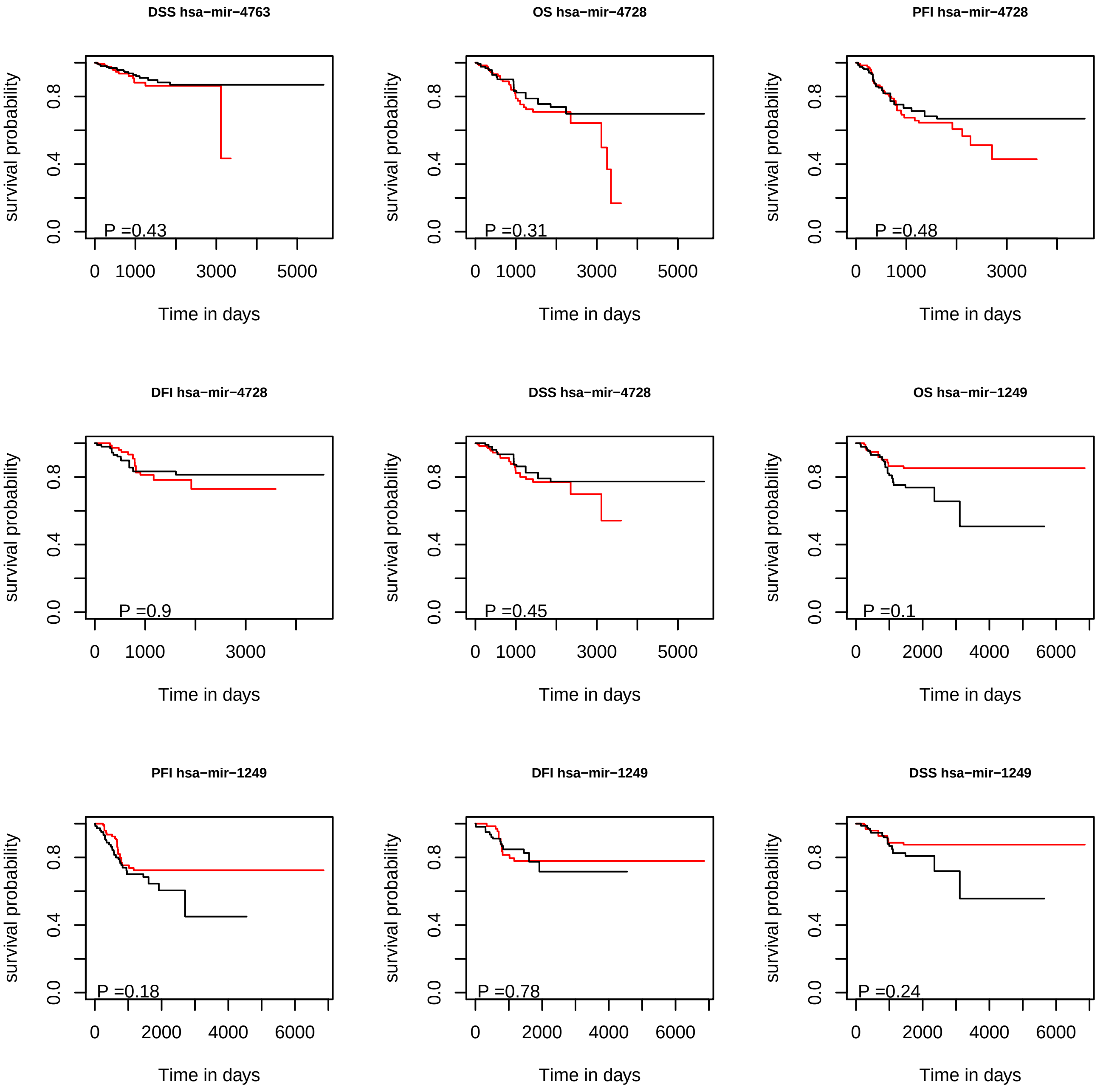
DSS hsa−mir−4763
P =0.43
OS hsa−mir−4728
P =0.31
PFI hsa−mir−4728
P =0.48
DFI hsa−mir−4728
P =0.9
DSS hsa−mir−4728
P =0.45
OS hsa−mir−1249
P =0.1
PFI hsa−mir−1249
P =0.18
DFI hsa−mir−1249
P =0.78
DSS hsa−mir−1249
P =0.24
survival probability
Time in days

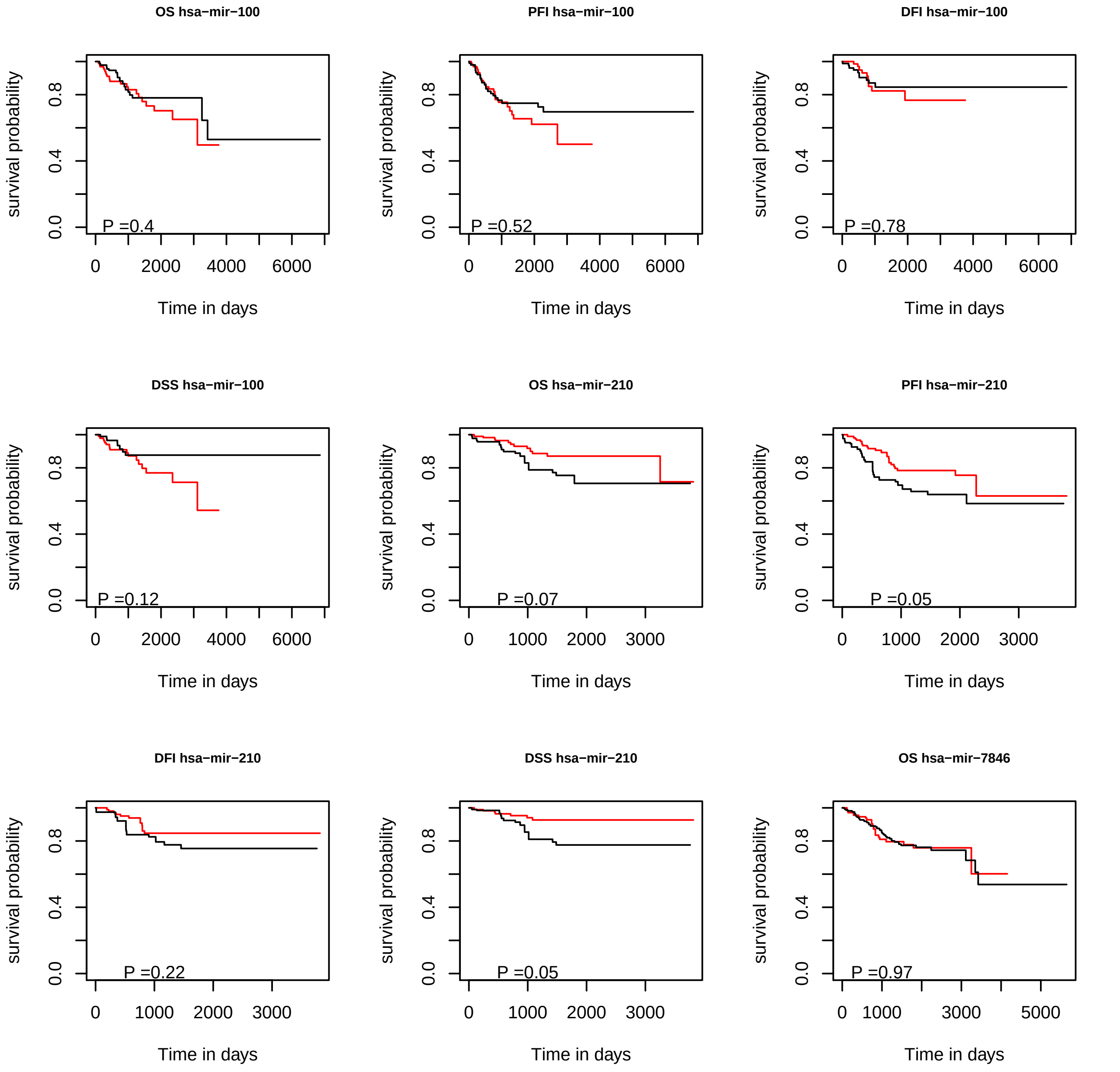
OS hsa−mir−100
P =0.4
PFI hsa−mir−100
P =0.52
DFI hsa−mir−100
P =0.78
DSS hsa−mir−100
P =0.12
OS hsa−mir−210
P =0.07
PFI hsa−mir−210
P =0.05
DFI hsa−mir−210
P =0.22
DSS hsa−mir−210
P =0.05
OS hsa−mir−7846
P =0.97
survival probability
Time in days
0.0
0.4
0.8
0
1000
2000
3000
4000
5000
6000

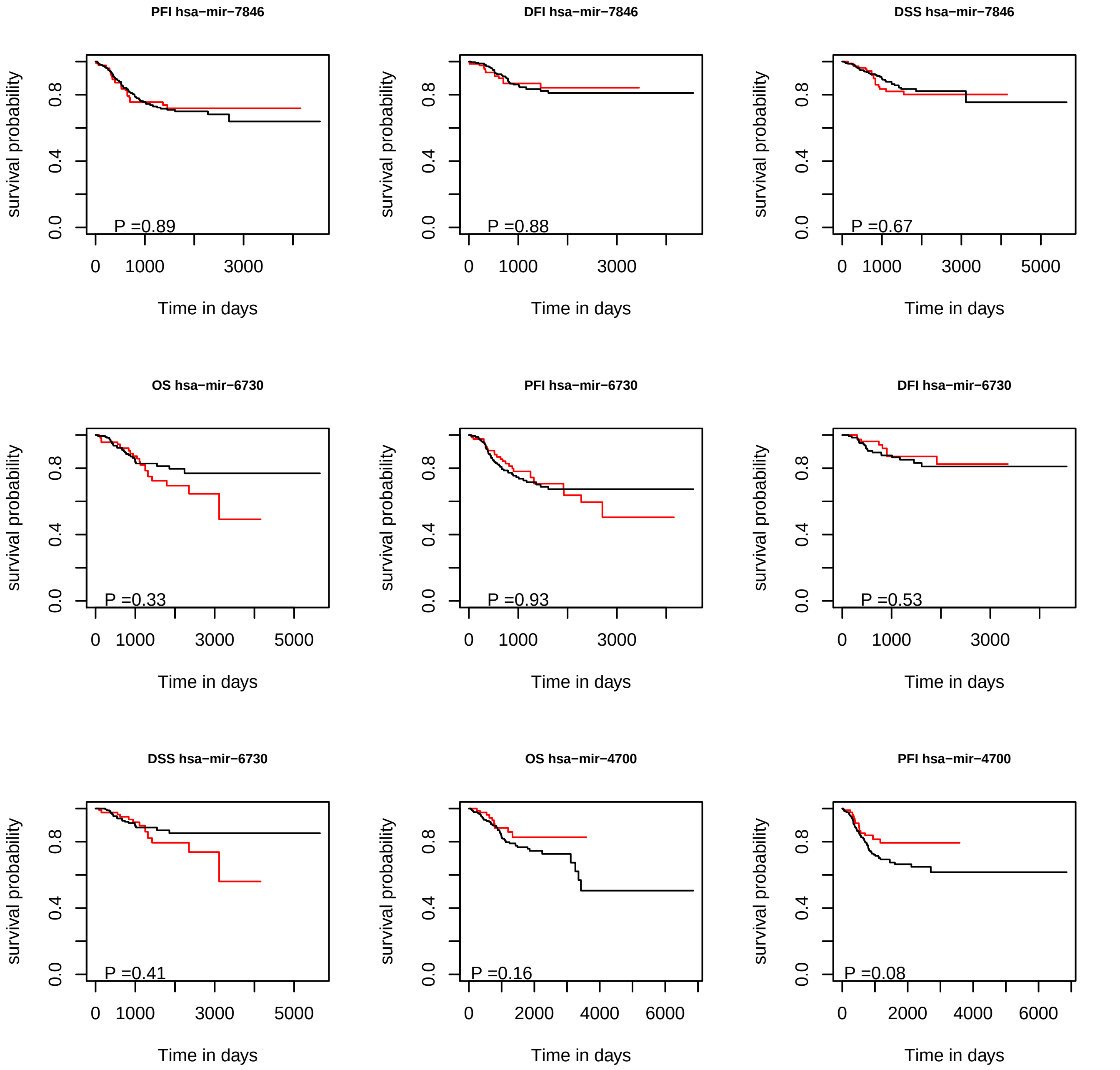

PFI hsa−mir−7846
P =0.89
DFI hsa−mir−7846
P =0.88
DSS hsa−mir−7846
P =0.67
OS hsa−mir−6730
P =0.33
PFI hsa−mir−6730
P =0.93
DFI hsa−mir−6730
P =0.53
DSS hsa−mir−6730
P =0.41
OS hsa−mir−4700
P =0.16
PFI hsa−mir−4700
P =0.08
survival probability
0.0
0.4
0.8
Time in days
0
1000
2000
3000
4000
5000
6000

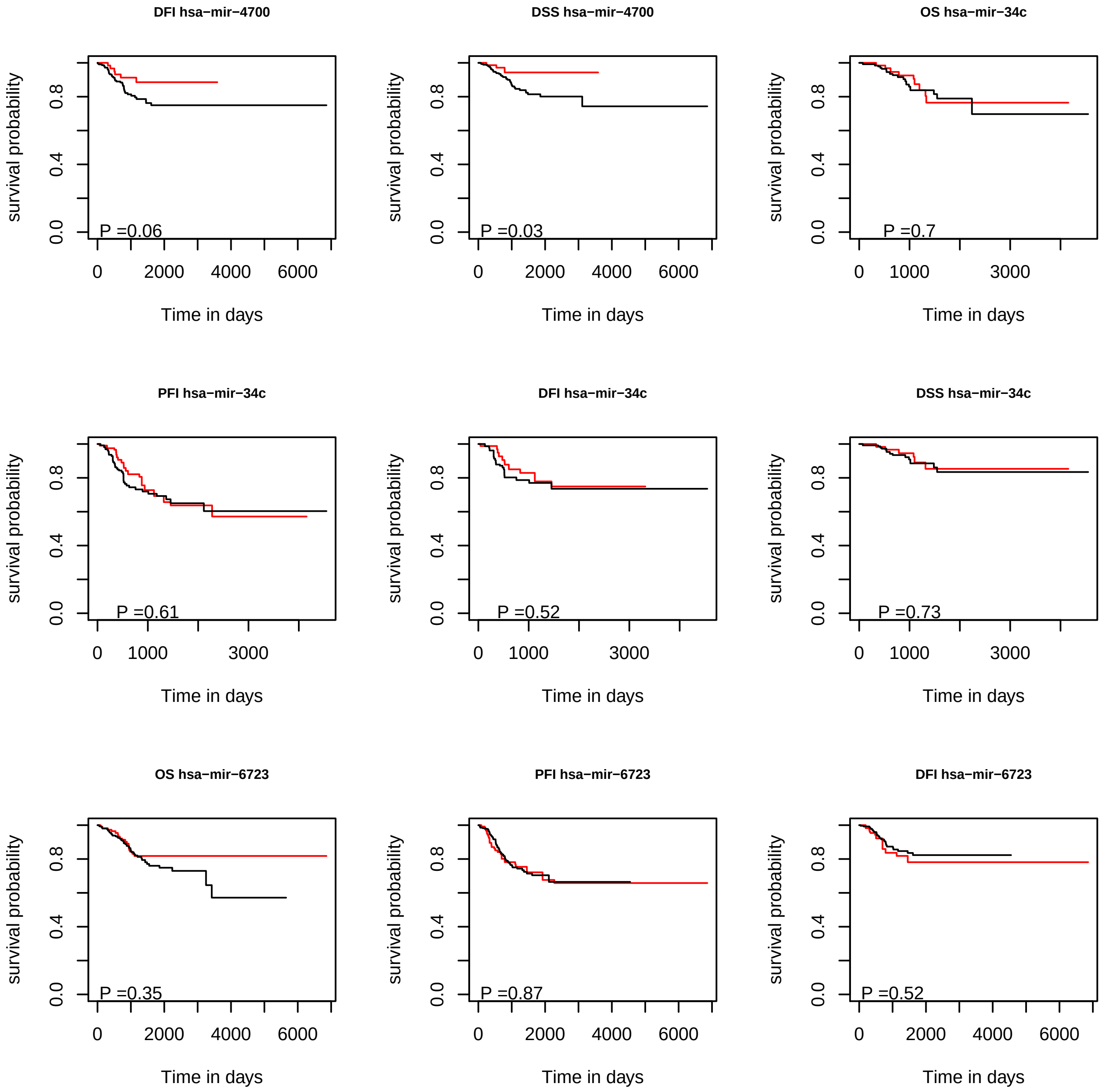
DFI hsa−mir−4700
survival probability
0.0
0.4
0.8
P =0.06
0
2000
4000
6000
Time in days
DSS hsa−mir−4700
survival probability
0.0
0.4
0.8
P =0.03
0
2000
4000
6000
Time in days
OS hsa−mir−34c
survival probability
0.0
0.4
0.8
P =0.7
0
1000
3000
Time in days
PFI hsa−mir−34c
survival probability
0.0
0.4
0.8
P =0.61
0
1000
3000
Time in days
DFI hsa−mir−34c
survival probability
0.0
0.4
0.8
P =0.52
0
1000
3000
Time in days
DSS hsa−mir−34c
survival probability
0.0
0.4
0.8
P =0.73
0
1000
3000
Time in days
OS hsa−mir−6723
survival probability
0.0
0.4
0.8
P =0.35
0
2000
4000
6000
Time in days
PFI hsa−mir−6723
survival probability
0.0
0.4
0.8
P =0.87
0
2000
4000
6000
Time in days
DFI hsa−mir−6723
survival probability
0.0
0.4
0.8
P =0.52
0
2000
4000
6000
Time in days

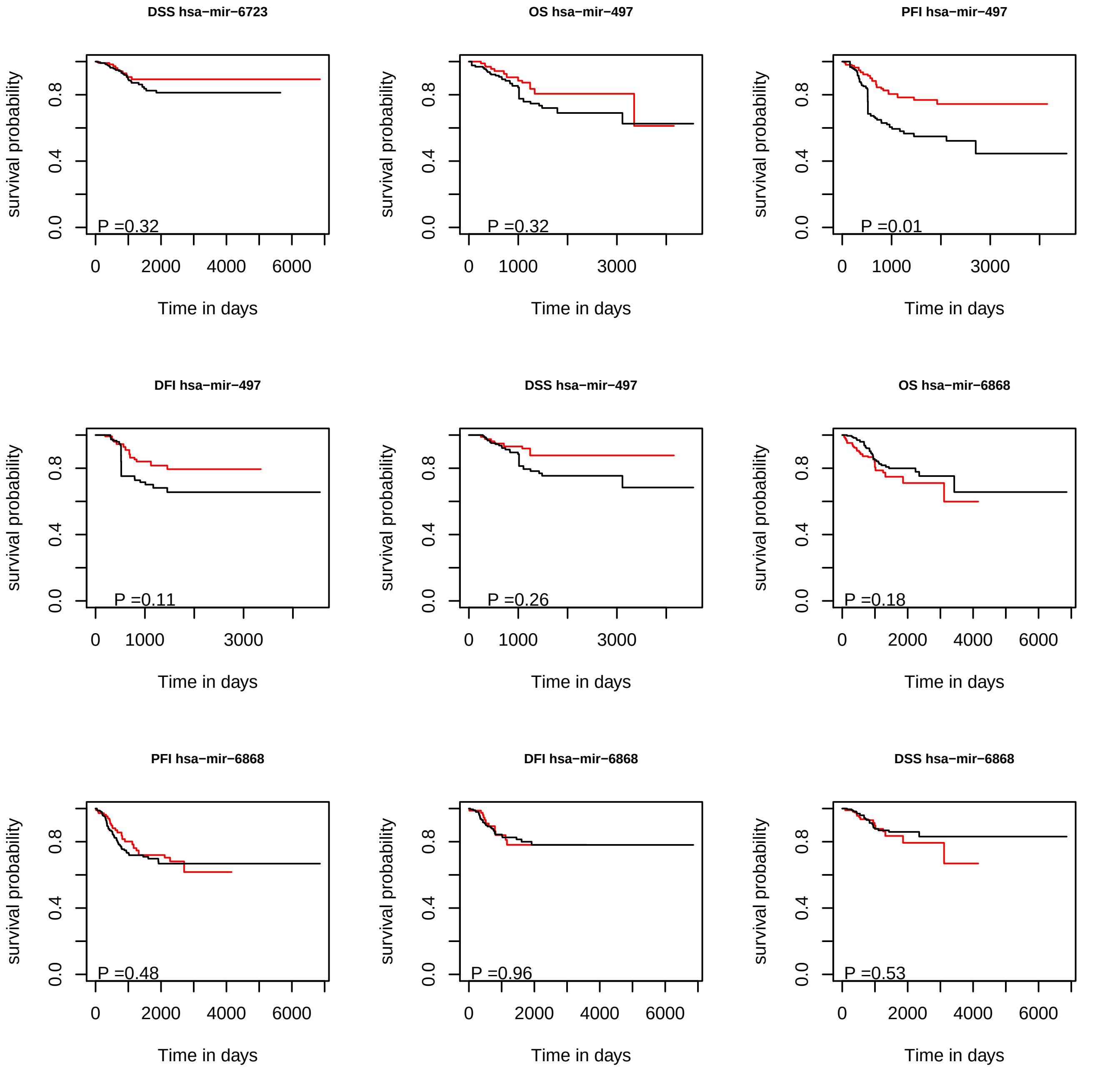
DSS hsa−mir−6723
P =0.32
OS hsa−mir−497
P =0.32
PFI hsa−mir−497
P =0.01
DFI hsa−mir−497
P =0.11
DSS hsa−mir−497
P =0.26
OS hsa−mir−6868
P =0.18
PFI hsa−mir−6868
P =0.48
DFI hsa−mir−6868
P =0.96
DSS hsa−mir−6868
P =0.53
survival probability
Time in days
0.0
0.4
0.8
0
1000
2000
3000
4000
6000

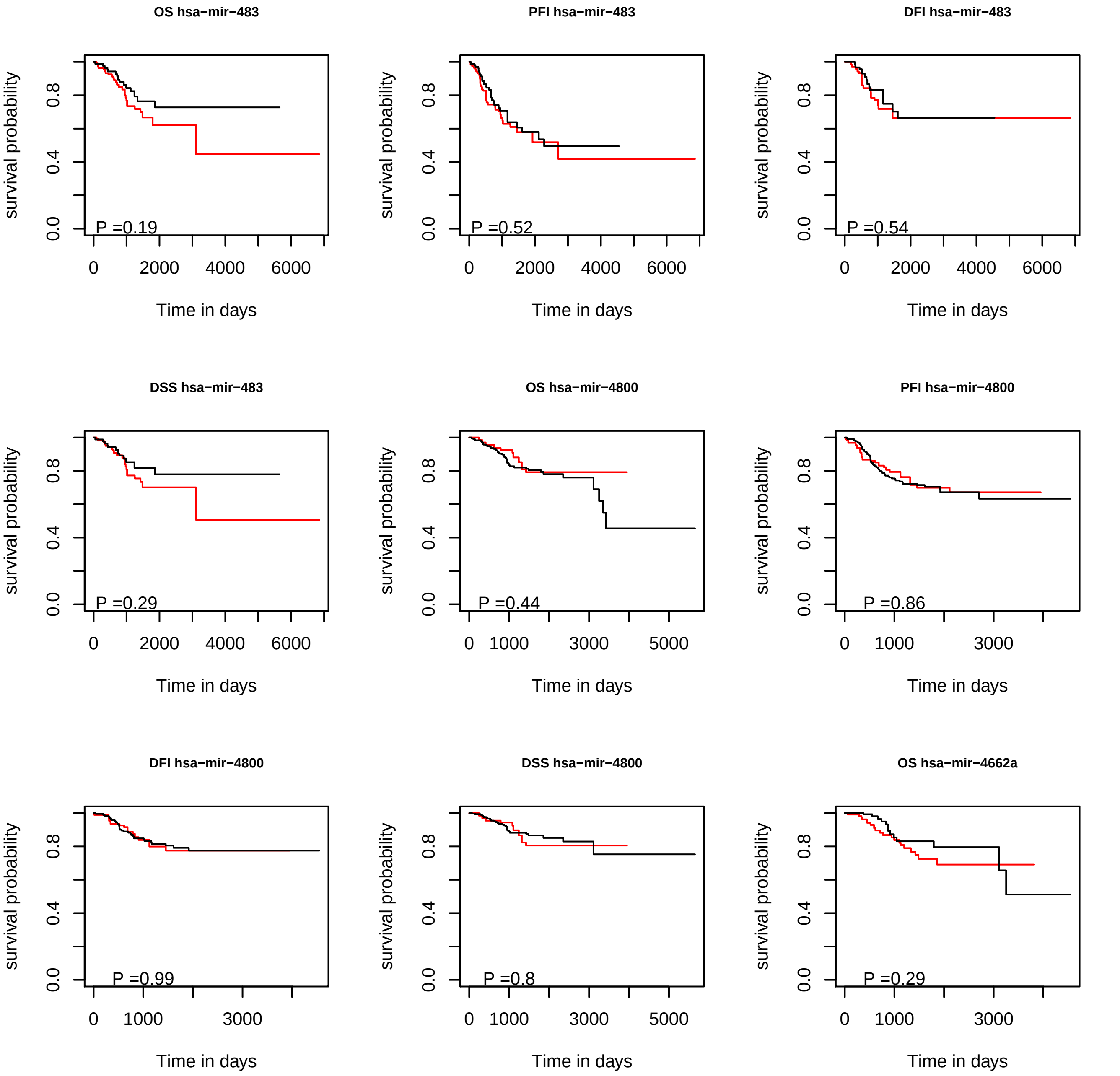
OS hsa−mir−483
PFI hsa−mir−483
DFI hsa−mir−483
DSS hsa−mir−483
OS hsa−mir−4800
PFI hsa−mir−4800
DFI hsa−mir−4800
DSS hsa−mir−4800
OS hsa−mir−4662a
survival probability
Time in days
0.0
0.4
0.8
0
1000
2000
3000
4000
5000
6000
P =0.19
P =0.52
P =0.54
P =0.29
P =0.44
P =0.86
P =0.99
P =0.8
P =0.29

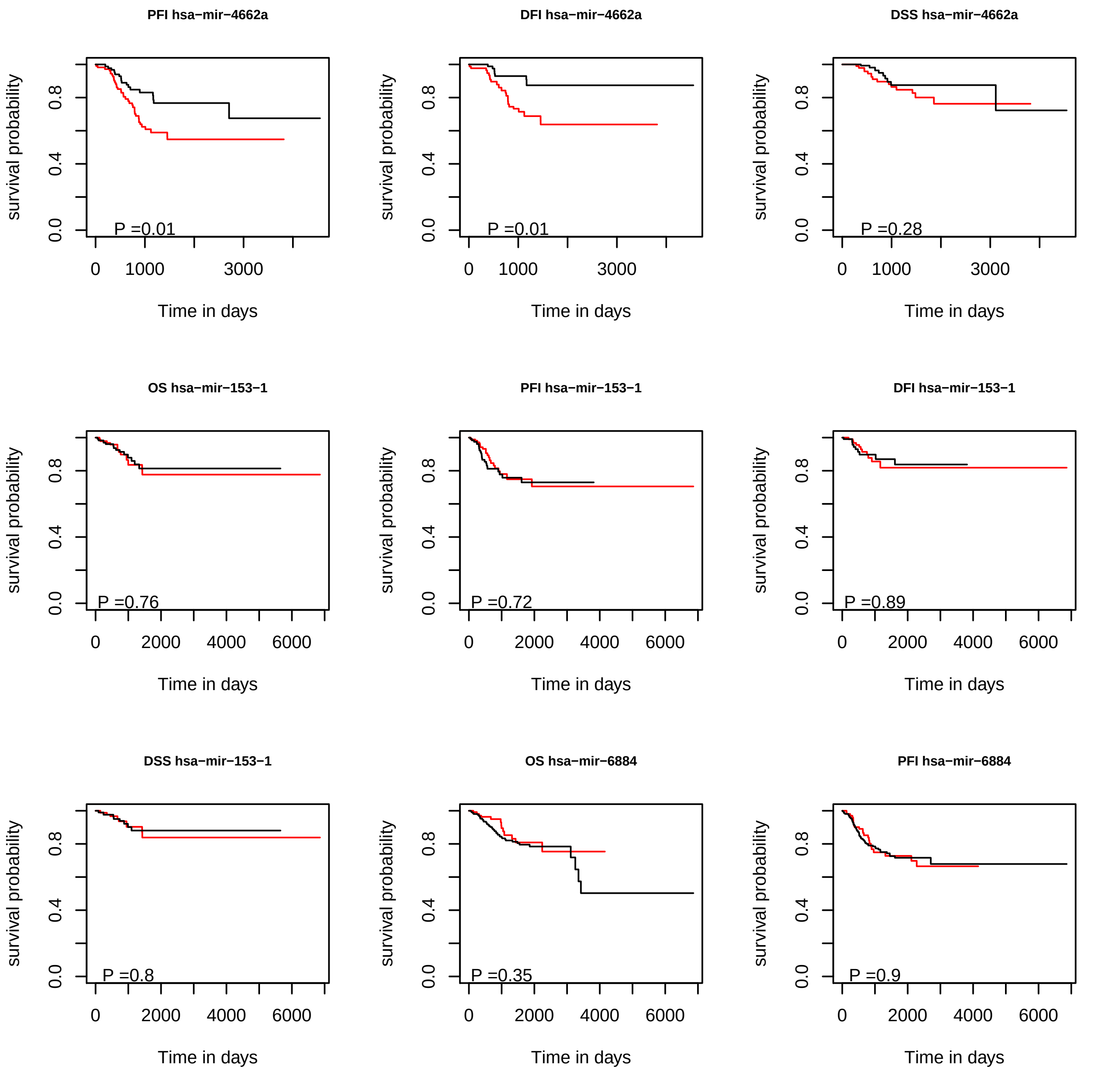
PFI hsa−mir−4662a
P =0.01
DFI hsa−mir−4662a
P =0.01
DSS hsa−mir−4662a
P =0.28
OS hsa−mir−153−1
P =0.76
PFI hsa−mir−153−1
P =0.72
DFI hsa−mir−153−1
P =0.89
DSS hsa−mir−153−1
P =0.8
OS hsa−mir−6884
P =0.35
PFI hsa−mir−6884
P =0.9
survival probability
Time in days
0.0
0.4
0.8
0
1000
3000
2000
4000
6000

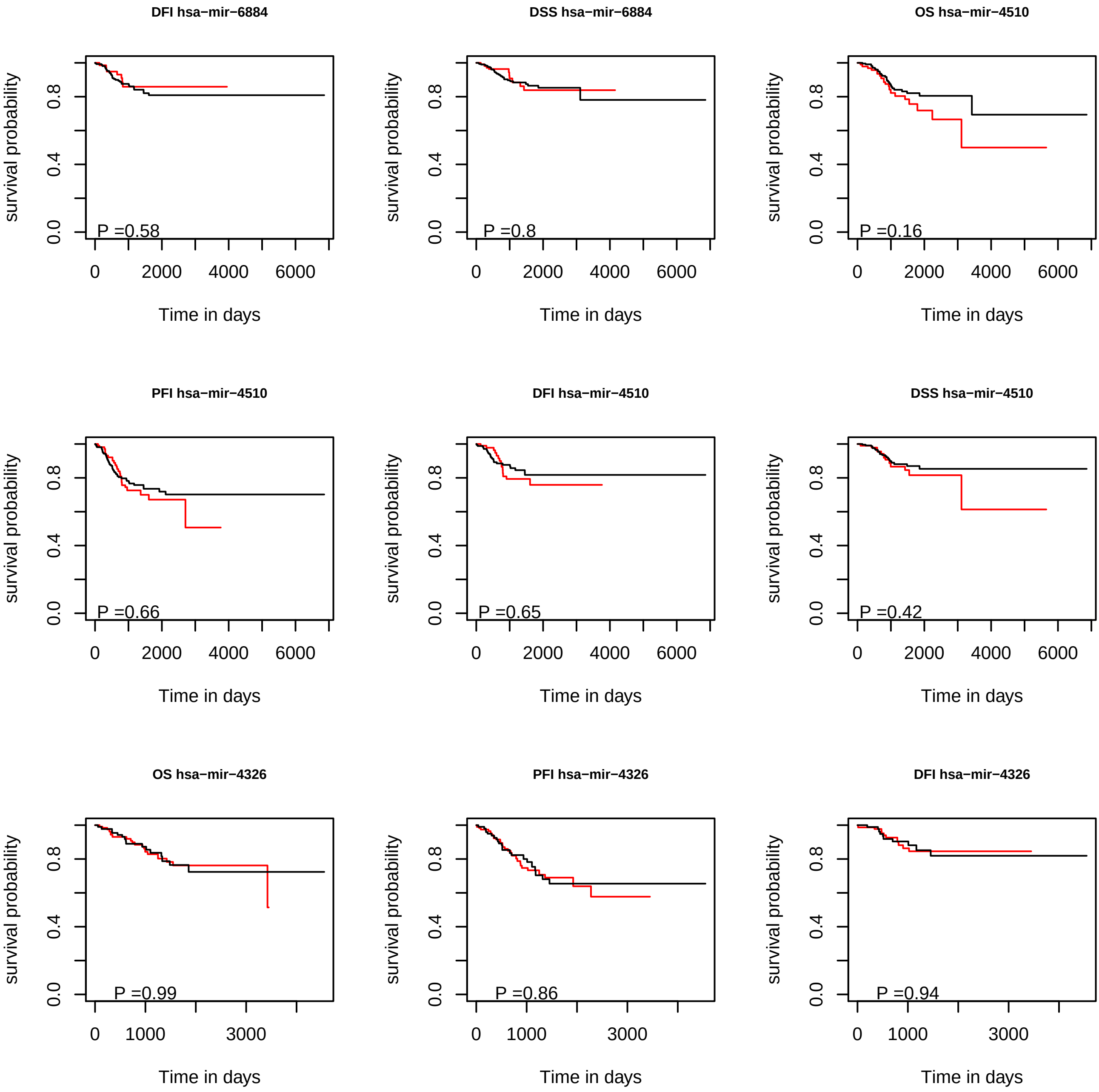
DFI hsa−mir−6884
DSS hsa−mir−6884
OS hsa−mir−4510
PFI hsa−mir−4510
DFI hsa−mir−4510
DSS hsa−mir−4510
OS hsa−mir−4326
PFI hsa−mir−4326
DFI hsa−mir−4326
survival probability
Time in days
P =0.58
P =0.8
P =0.16
P =0.66
P =0.65
P =0.42
P =0.99
P =0.86
P =0.94
0.0
0.4
0.8
0
1000
2000
3000
4000
6000

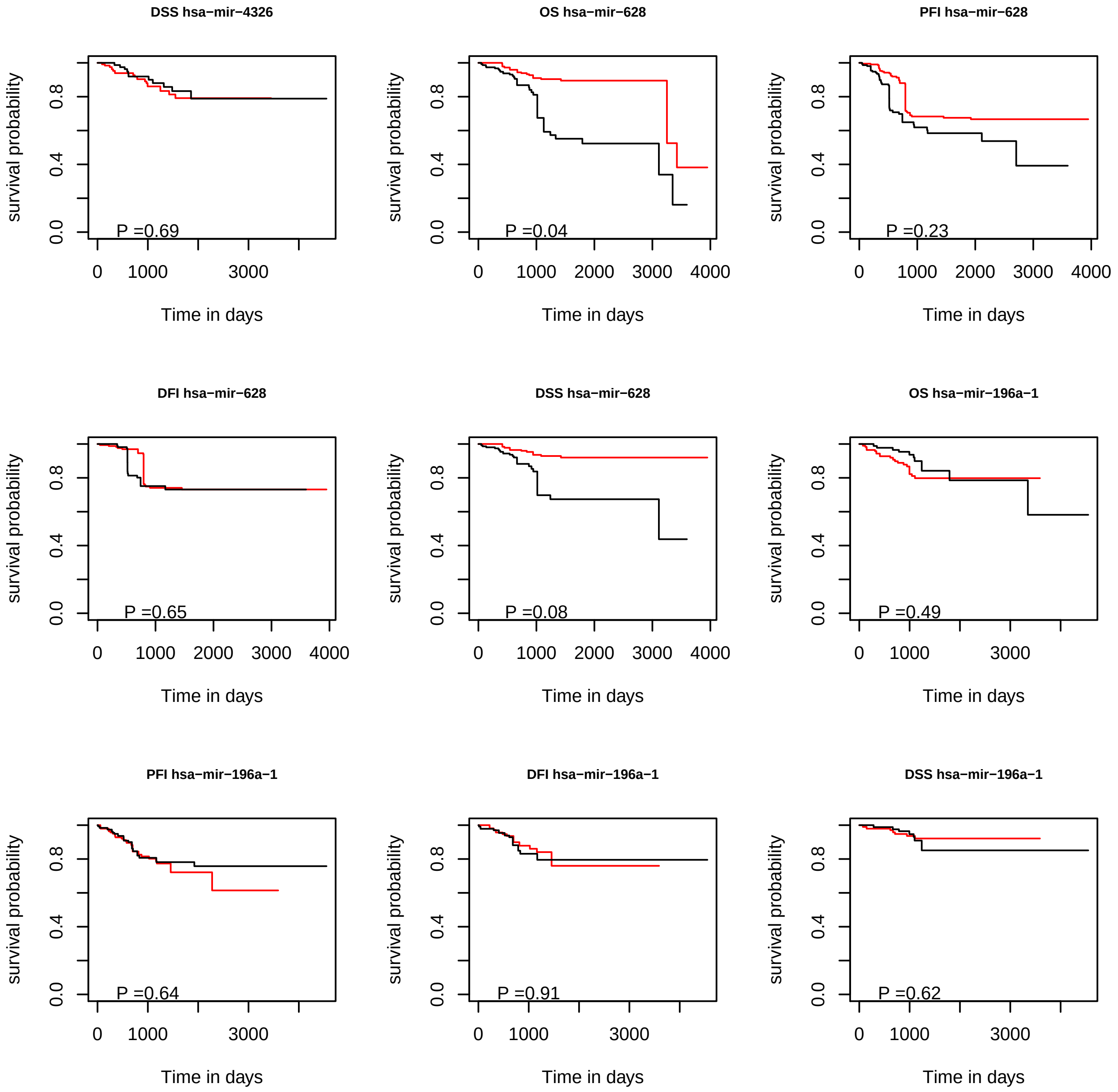
DSS hsa−mir−4326
P =0.69
OS hsa−mir−628
P =0.04
PFI hsa−mir−628
P =0.23
DFI hsa−mir−628
P =0.65
DSS hsa−mir−628
P =0.08
OS hsa−mir−196a−1
P =0.49
PFI hsa−mir−196a−1
P =0.64
DFI hsa−mir−196a−1
P =0.91
DSS hsa−mir−196a−1
P =0.62
survival probability
Time in days

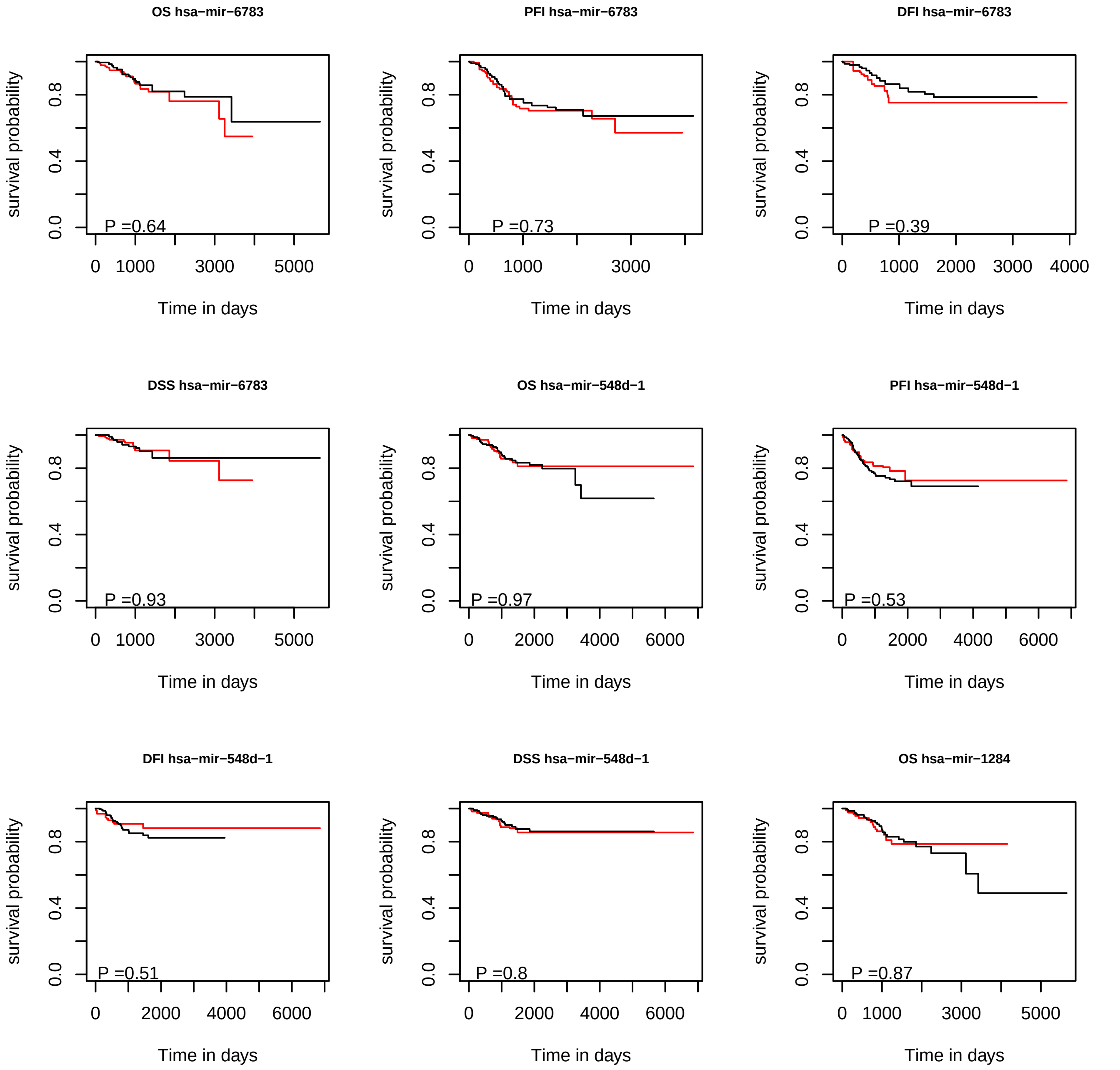
OS hsa−mir−6783
P =0.64
PFI hsa−mir−6783
P =0.73
DFI hsa−mir−6783
P =0.39
DSS hsa−mir−6783
P =0.93
OS hsa−mir−548d−1
P =0.97
PFI hsa−mir−548d−1
P =0.53
DFI hsa−mir−548d−1
P =0.51
DSS hsa−mir−548d−1
P =0.8
OS hsa−mir−1284
P =0.87
survival probability
Time in days
0.0
0.4
0.8
0
1000
2000
3000
4000
5000
6000

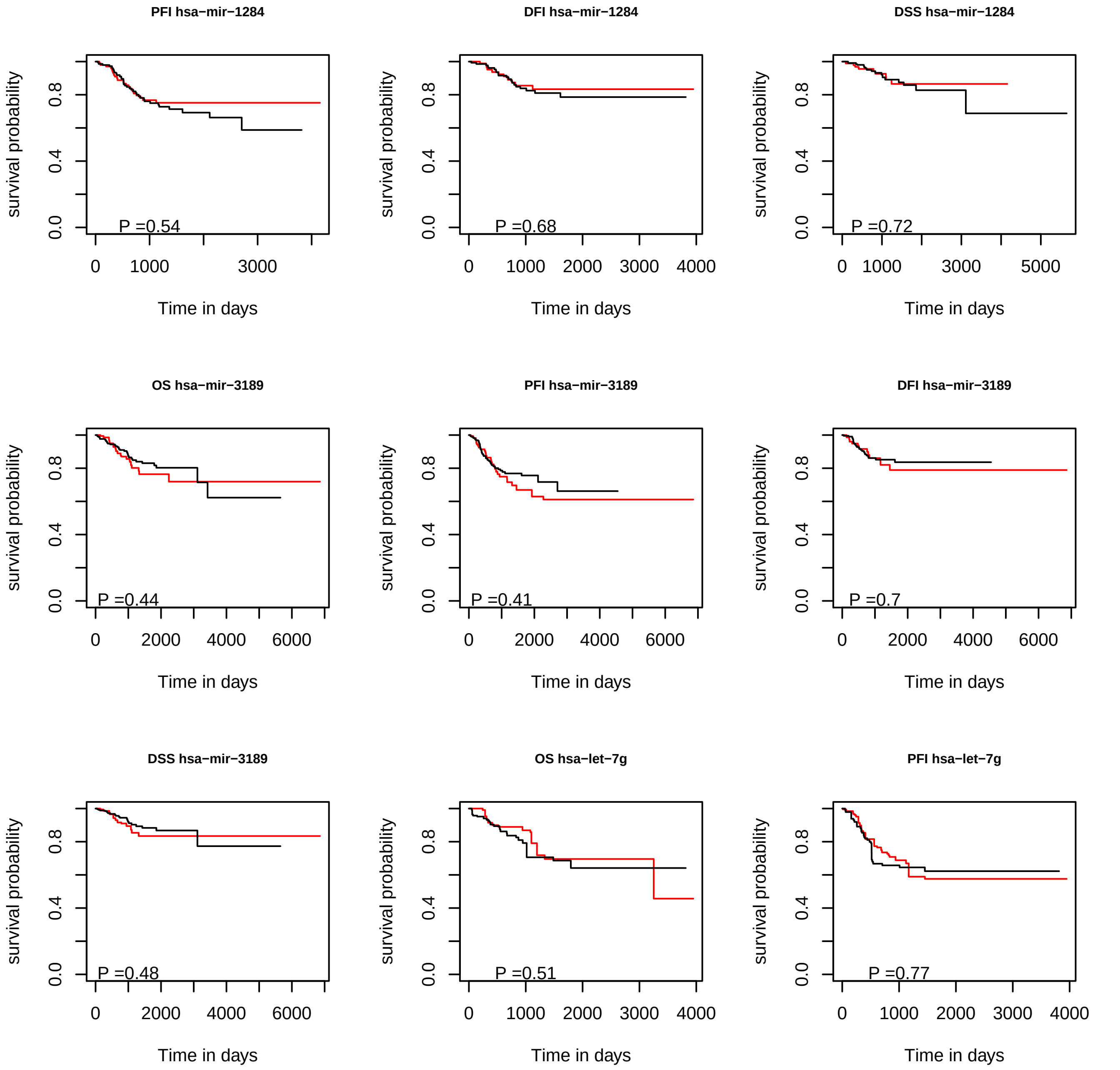
PFI hsa−mir−1284
survival probability
0.0
0.4
0.8
P =0.54
0
1000
3000
Time in days
DFI hsa−mir−1284
survival probability
0.0
0.4
0.8
P =0.68
0
1000
2000
3000
4000
Time in days
DSS hsa−mir−1284
survival probability
0.0
0.4
0.8
P =0.72
0
1000
3000
5000
Time in days
OS hsa−mir−3189
survival probability
0.0
0.4
0.8
P =0.44
0
2000
4000
6000
Time in days
PFI hsa−mir−3189
survival probability
0.0
0.4
0.8
P =0.41
0
2000
4000
6000
Time in days
DFI hsa−mir−3189
survival probability
0.0
0.4
0.8
P =0.7
0
2000
4000
6000
Time in days
DSS hsa−mir−3189
survival probability
0.0
0.4
0.8
P =0.48
0
2000
4000
6000
Time in days
OS hsa−let−7g
survival probability
0.0
0.4
0.8
P =0.51
0
1000
2000
3000
4000
Time in days
PFI hsa−let−7g
survival probability
0.0
0.4
0.8
P =0.77
0
1000
2000
3000
4000
Time in days

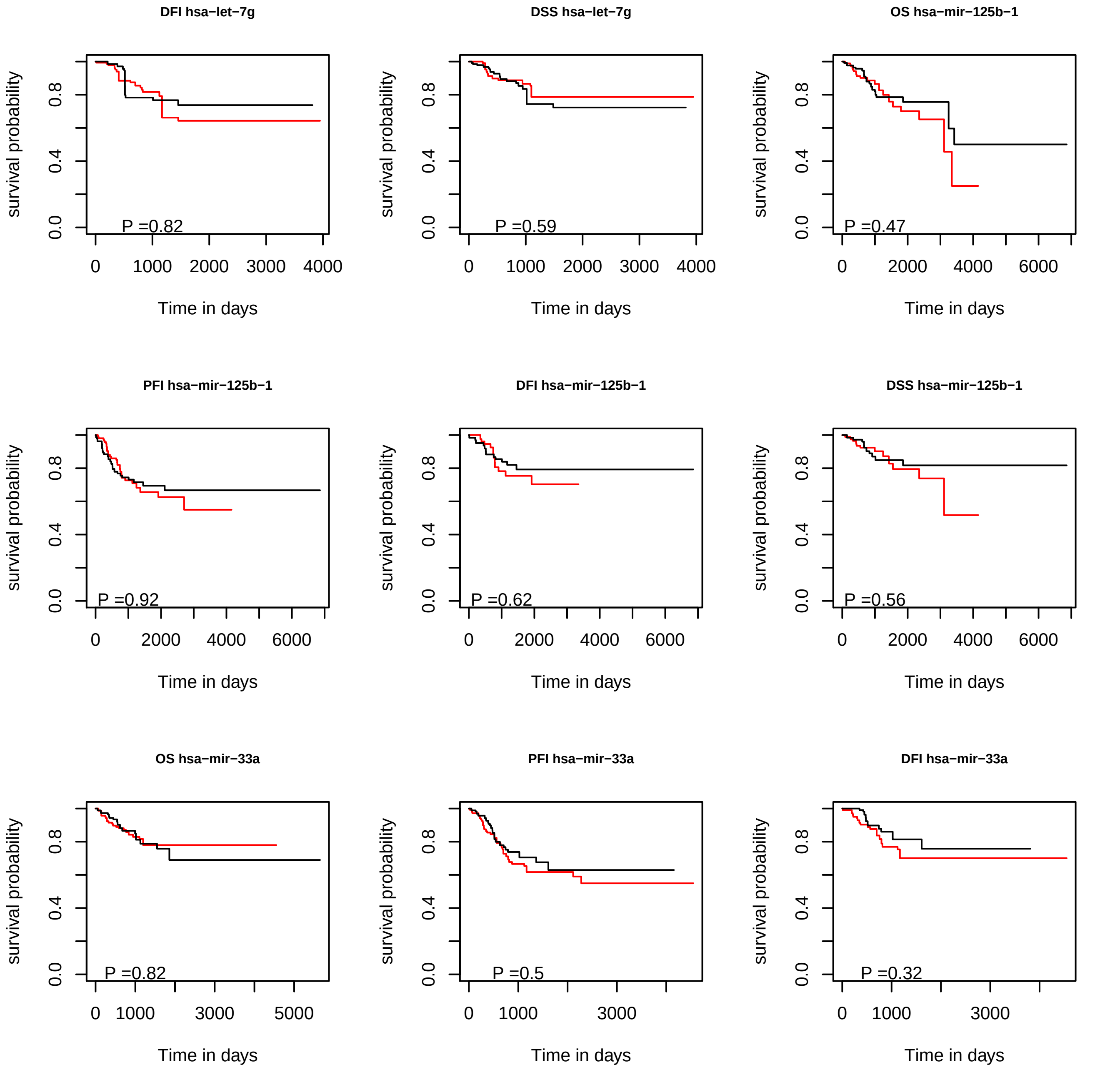
DFI hsa−let−7g
P =0.82
DSS hsa−let−7g
P =0.59
OS hsa−mir−125b−1
P =0.47
PFI hsa−mir−125b−1
P =0.92
DFI hsa−mir−125b−1
P =0.62
DSS hsa−mir−125b−1
P =0.56
OS hsa−mir−33a
P =0.82
PFI hsa−mir−33a
P =0.5
DFI hsa−mir−33a
P =0.32
survival probability
Time in days

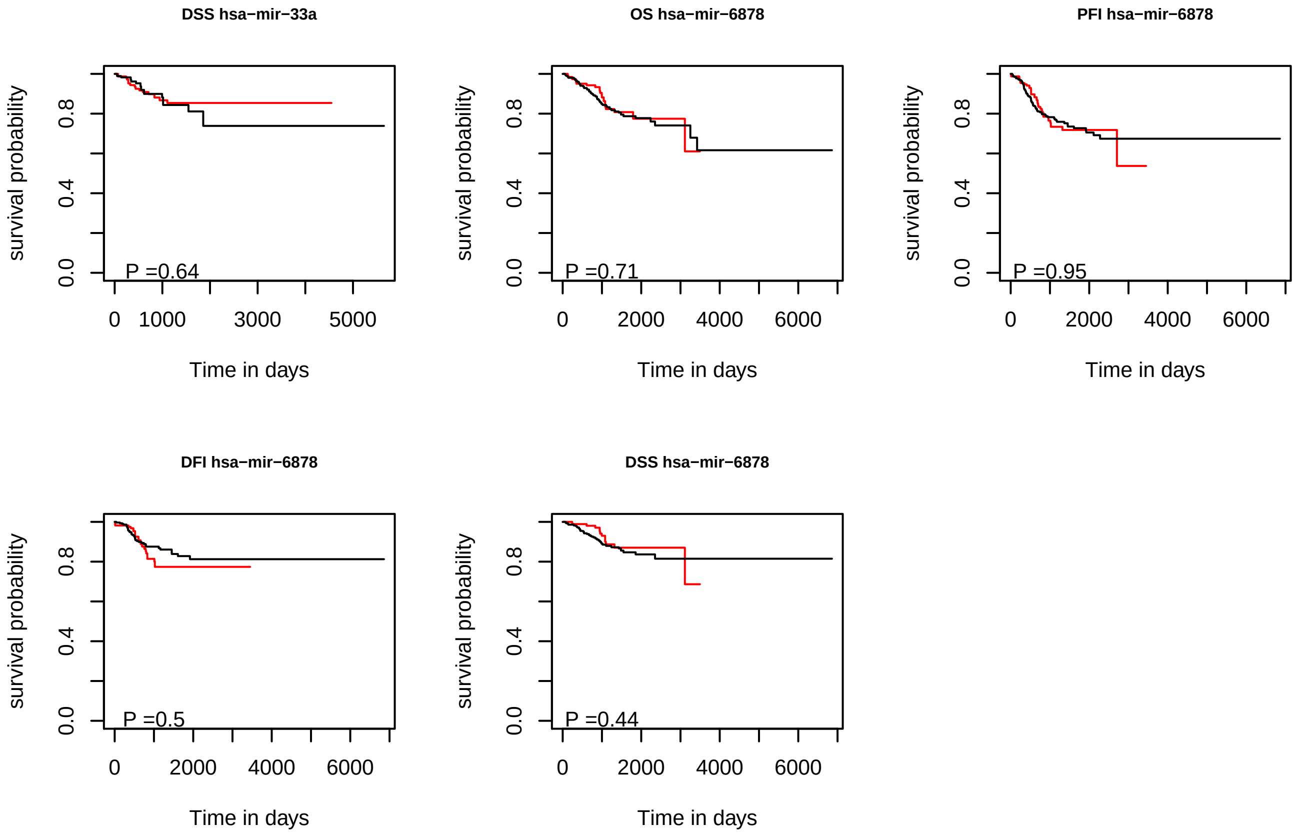
DSS hsa−mir−33a
survival probability
0.0
0.4
0.8
P =0.64
0
1000
3000
5000
Time in days
OS hsa−mir−6878
survival probability
0.0
0.4
0.8
P =0.71
0
2000
4000
6000
Time in days
PFI hsa−mir−6878
survival probability
0.0
0.4
0.8
P =0.95
0
2000
4000
6000
Time in days
DFI hsa−mir−6878
survival probability
0.0
0.4
0.8
P =0.5
0
2000
4000
6000
Time in days
DSS hsa−mir−6878
survival probability
0.0
0.4
0.8
P =0.44
0
2000
4000
6000
Time in days
