# Supplementary material for: Computing microRNA-gene interaction networks in pan-cancer using miRDriver
Source: Sci Rep. 2022 Mar 8;12:3717. doi: 10.1038/s41598-022-07628-z (PMC8904490; doi:10.1038/s41598-022-07628-z)
Supplement: Supplementary file 28 — Supplementary Information 28. [file 41598_2022_7628_MOESM28_ESM.pdf]

Computing microRNA-gene interaction networks in pan-cancer using miRDriver

Banabithi Bose, Matthew Moravec, and Serdar Bozdag

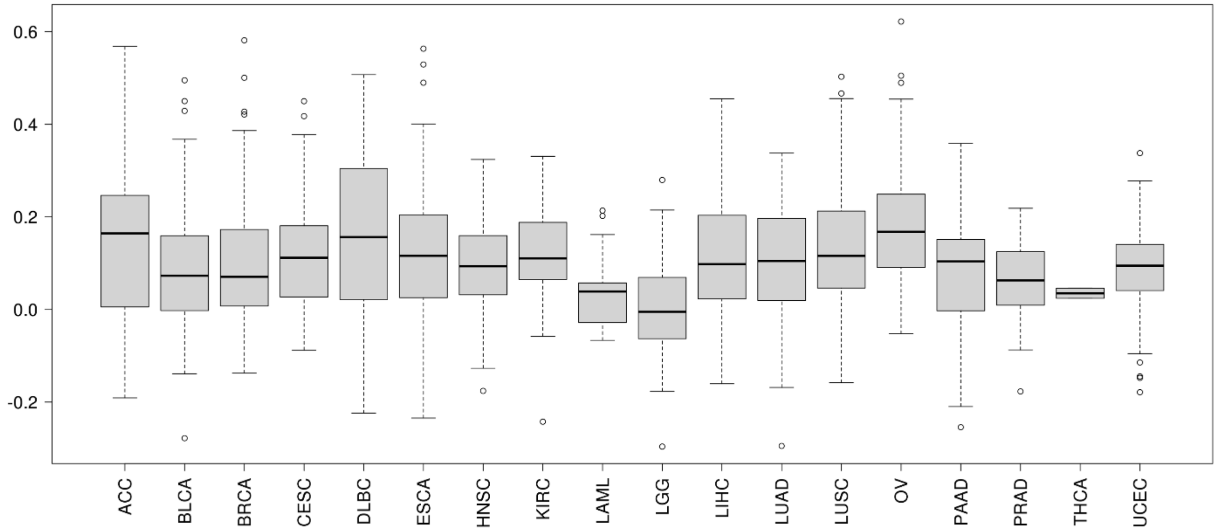

Supplemental Figure S19: Boxplots showing the Spearman correlation values between copy number and expression across all the samples of the computed miRNAs of miRDriver in eighteen different cancer types. There is a positive median distribution of correlations across all cancer types.
