# Supplementary material for: Computing microRNA-gene interaction networks in pan-cancer using miRDriver
Source: Sci Rep. 2022 Mar 8;12:3717. doi: 10.1038/s41598-022-07628-z (PMC8904490; doi:10.1038/s41598-022-07628-z)
Supplement: Supplementary file 29 — Supplementary Information 29. [file 41598_2022_7628_MOESM29_ESM.pdf]

Computing microRNA-gene interaction networks in pan-cancer using miRDriver

Banabithi Bose, Matthew Moravec, and Serdar Bozdag

Supplemental Figure S20

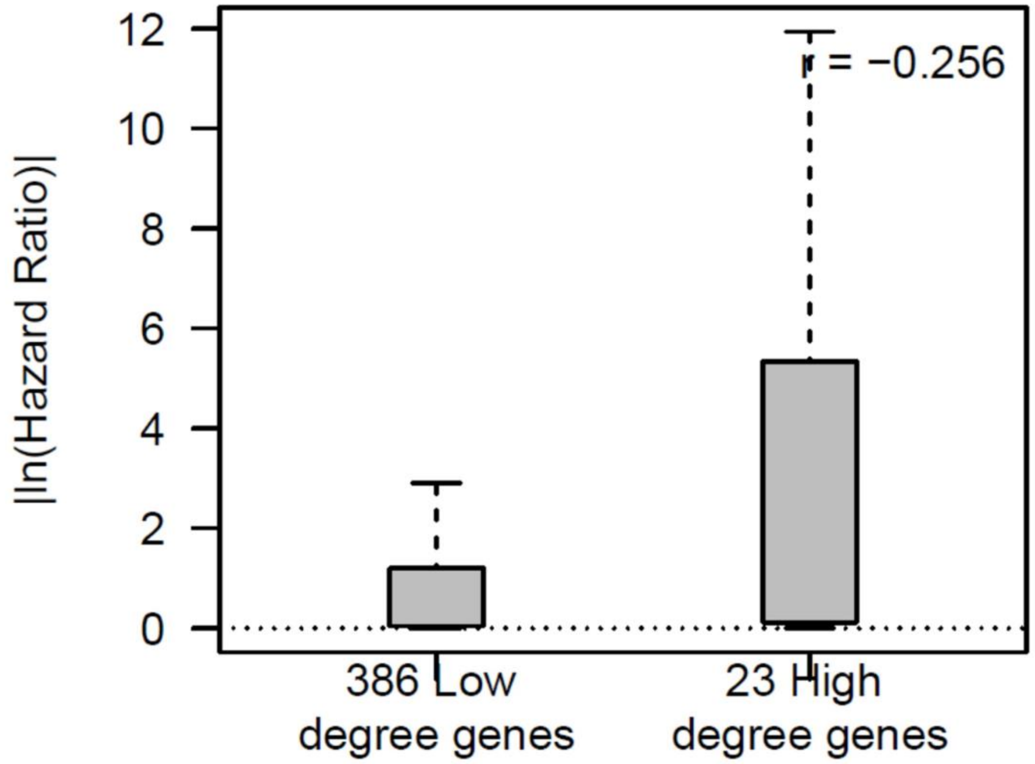

Boxplots of absolute values of natural logarithm of hazard ratios in high-degree and low-degree genes with r value of Mann–Whitney test.

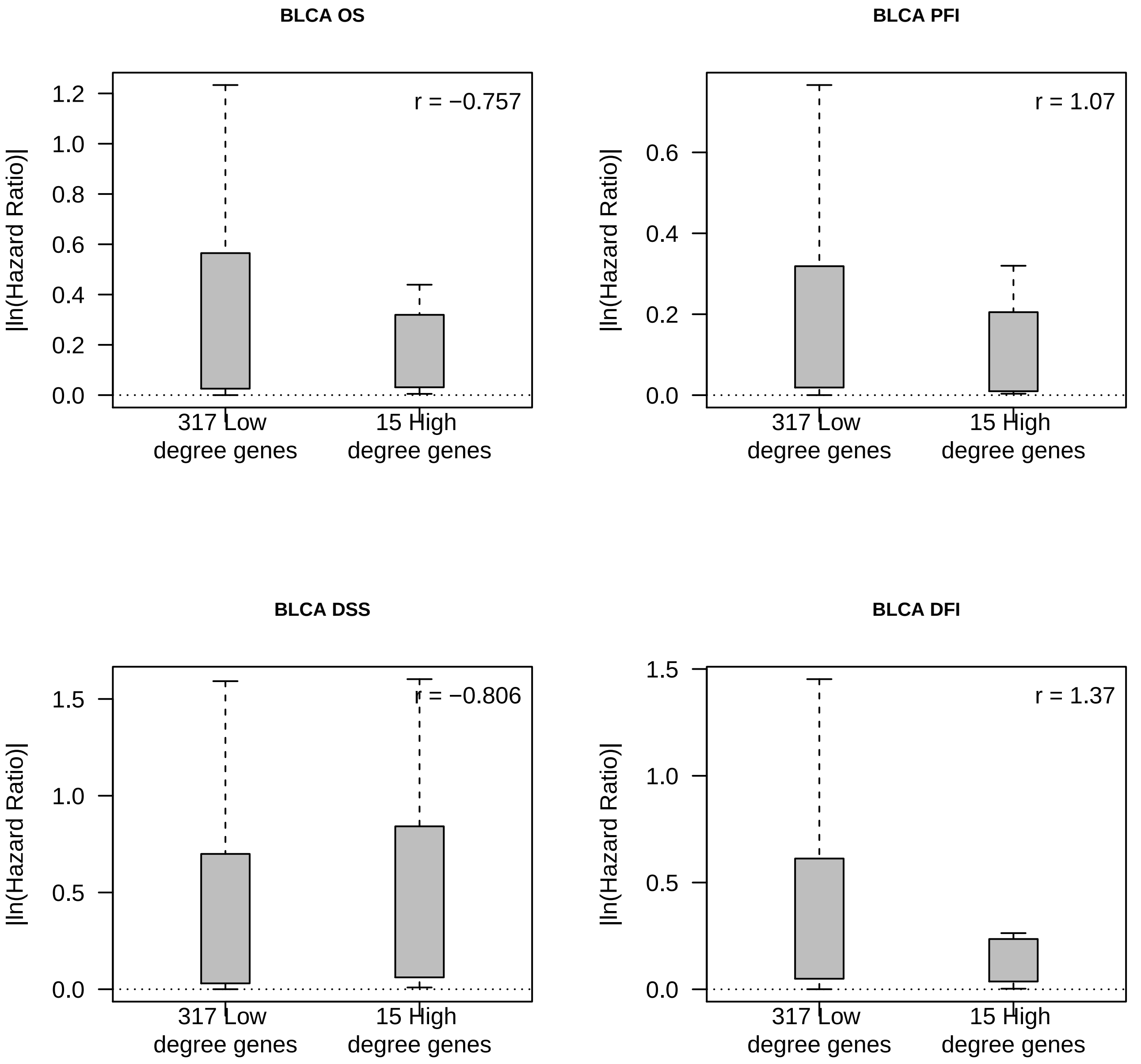
BLCA OS
r = −0.757
|ln(Hazard Ratio)|
0.0
0.2
0.4
0.6
0.8
1.0
1.2
317 Low
degree genes
15 High
degree genes
BLCA PFI
r = 1.07
|ln(Hazard Ratio)|
0.0
0.2
0.4
0.6
317 Low
degree genes
15 High
degree genes
BLCA DSS
r = −0.806
|ln(Hazard Ratio)|
0.0
0.5
1.0
1.5
317 Low
degree genes
15 High
degree genes
BLCA DFI
r = 1.37
|ln(Hazard Ratio)|
0.0
0.5
1.0
1.5
317 Low
degree genes
15 High
degree genes

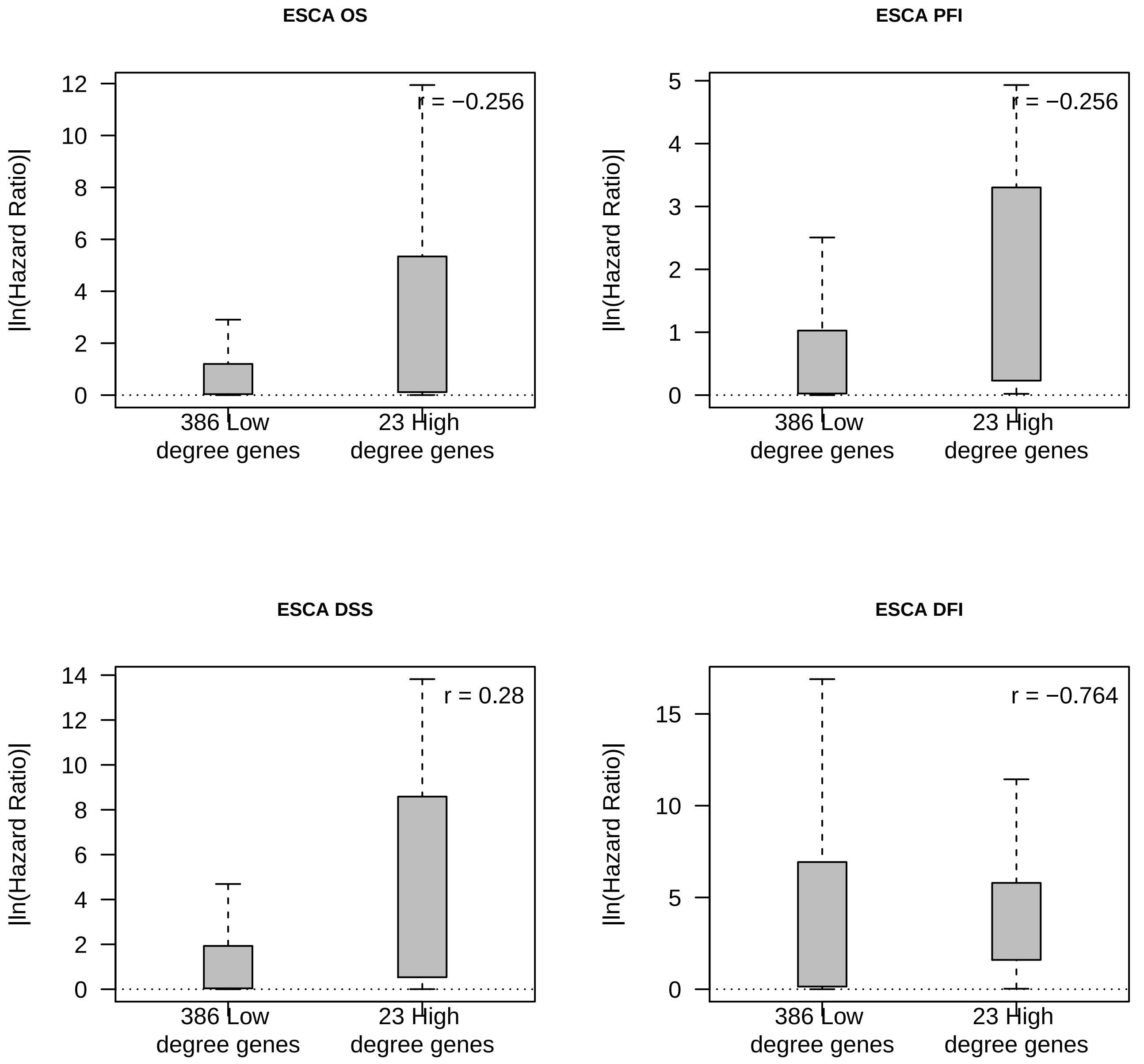
ESCA OS
r = −0.256
|ln(Hazard Ratio)|
0
2
4
6
8
10
12
386 Low
degree genes
23 High
degree genes
ESCA PFI
r = −0.256
|ln(Hazard Ratio)|
0
1
2
3
4
5
386 Low
degree genes
23 High
degree genes
ESCA DSS
r = 0.28
|ln(Hazard Ratio)|
0
2
4
6
8
10
12
14
386 Low
degree genes
23 High
degree genes
ESCA DFI
r = −0.764
|ln(Hazard Ratio)|
0
5
10
15
386 Low
degree genes
23 High
degree genes

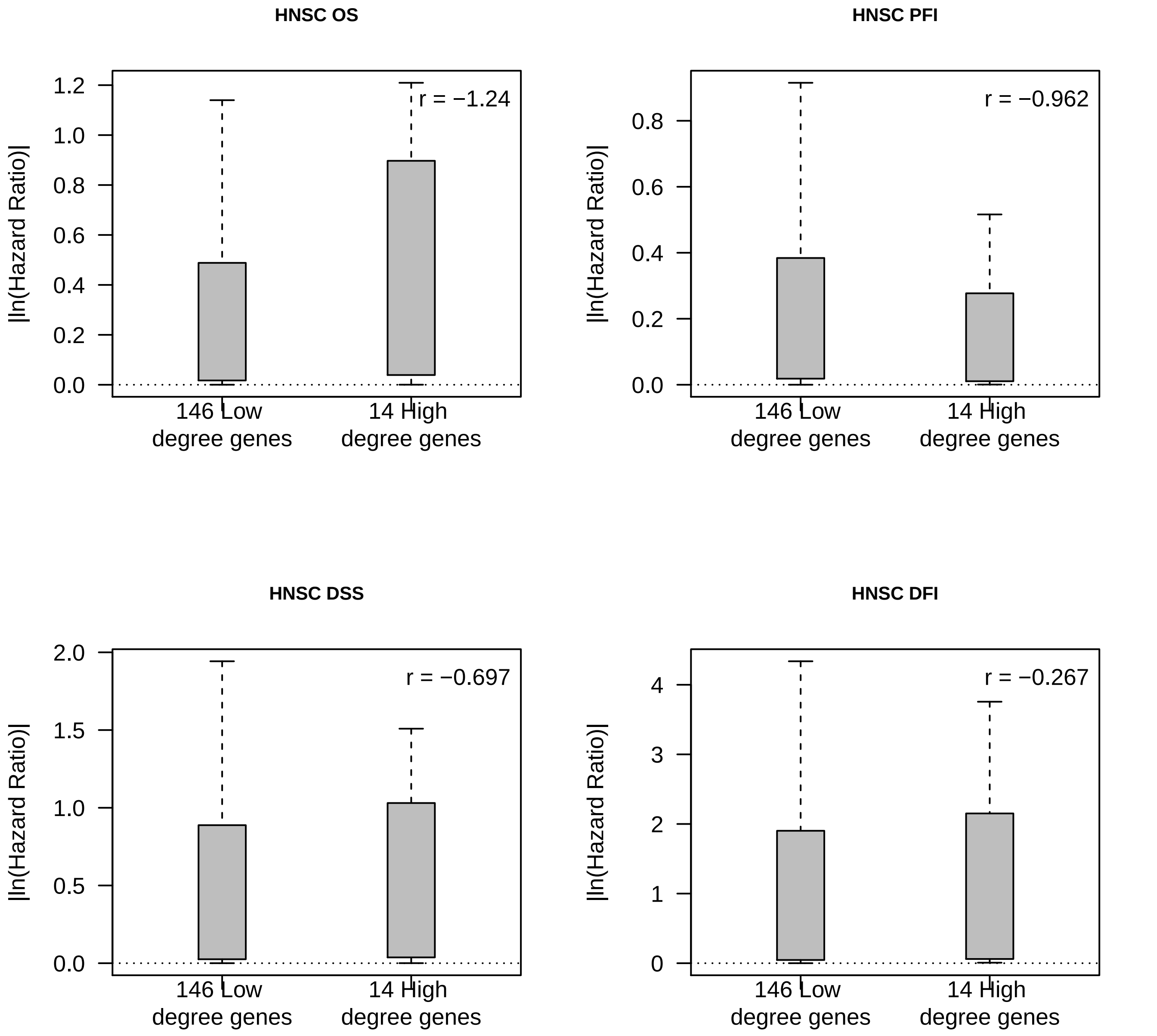
HNSC OS
r = −1.24
|ln(Hazard Ratio)|
0.0
0.2
0.4
0.6
0.8
1.0
1.2
146 Low degree genes
14 High degree genes
HNSC PFI
r = −0.962
|ln(Hazard Ratio)|
0.0
0.2
0.4
0.6
0.8
146 Low degree genes
14 High degree genes
HNSC DSS
r = −0.697
|ln(Hazard Ratio)|
0.0
0.5
1.0
1.5
2.0
146 Low degree genes
14 High degree genes
HNSC DFI
r = −0.267
|ln(Hazard Ratio)|
0
1
2
3
4
146 Low degree genes
14 High degree genes

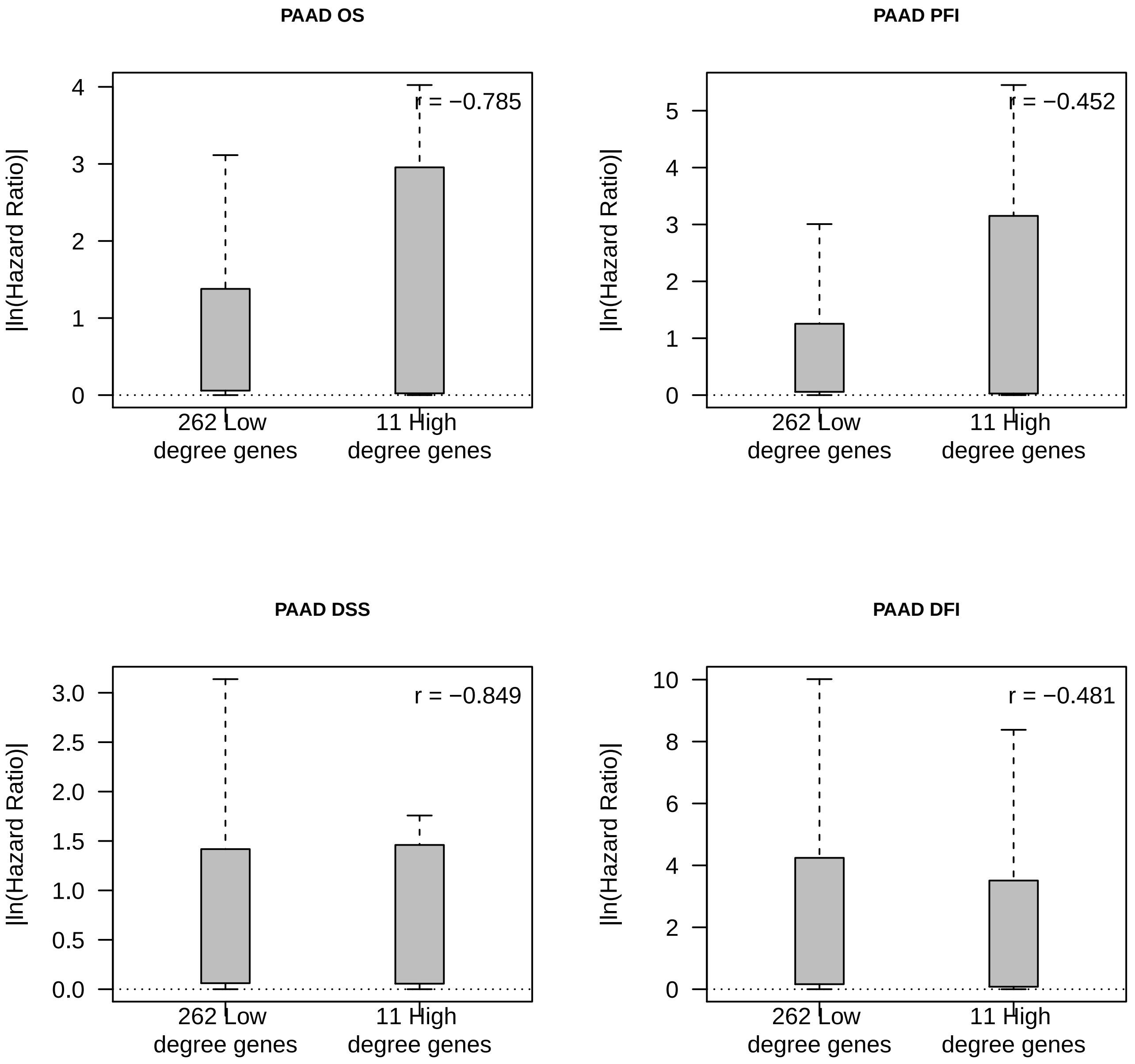
PAAD OS
r = −0.785
|ln(Hazard Ratio)|
0
1
2
3
4
262 Low degree genes
11 High degree genes
PAAD PFI
r = −0.452
|ln(Hazard Ratio)|
0
1
2
3
4
5
262 Low degree genes
11 High degree genes
PAAD DSS
r = −0.849
|ln(Hazard Ratio)|
0.0
0.5
1.0
1.5
2.0
2.5
3.0
262 Low degree genes
11 High degree genes
PAAD DFI
r = −0.481
|ln(Hazard Ratio)|
0
2
4
6
8
10
262 Low degree genes
11 High degree genes
