# Supplementary material for: Computing microRNA-gene interaction networks in pan-cancer using miRDriver
Source: Sci Rep. 2022 Mar 8;12:3717. doi: 10.1038/s41598-022-07628-z (PMC8904490; doi:10.1038/s41598-022-07628-z)
Supplement: Supplementary file 30 — Supplementary Information 30. [file 41598_2022_7628_MOESM30_ESM.pdf]

Computing microRNA-gene interaction networks in pan-cancer using miRDriver

Banabithi Bose, Matthew Moravec, and Serdar Bozdag

Supplemental Figure S21

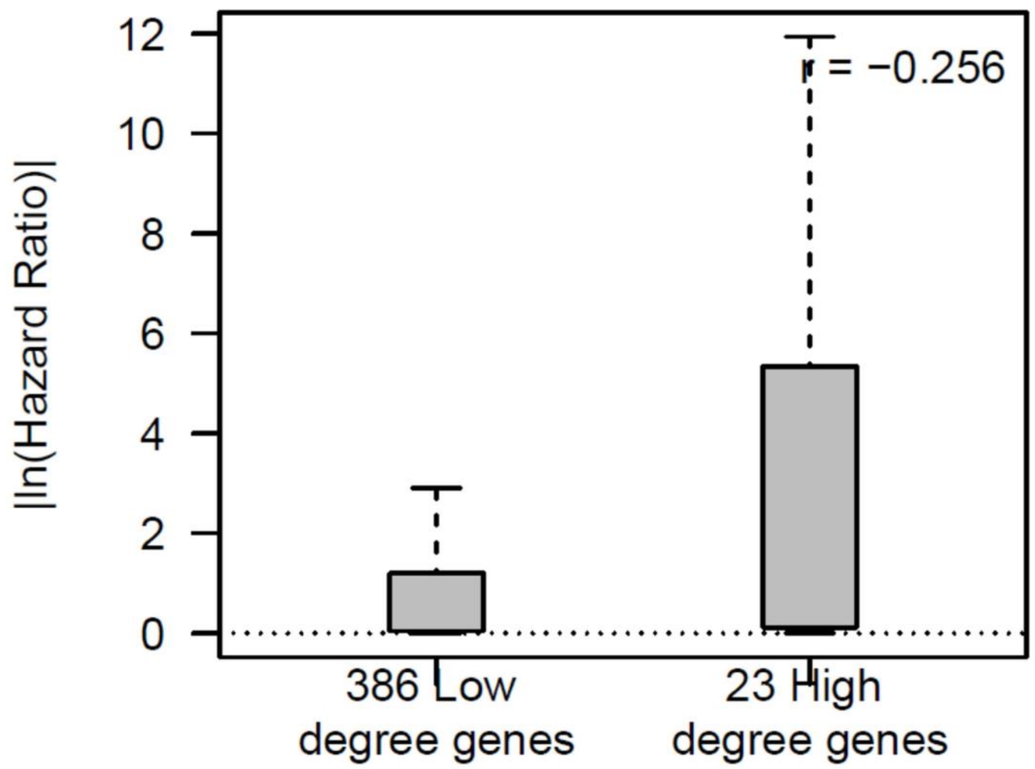

Boxplots of absolute values of natural logarithm of hazard ratios in high-degree and low-degree genes with r value of Mann–Whitney test.

Supplemental Figure S21

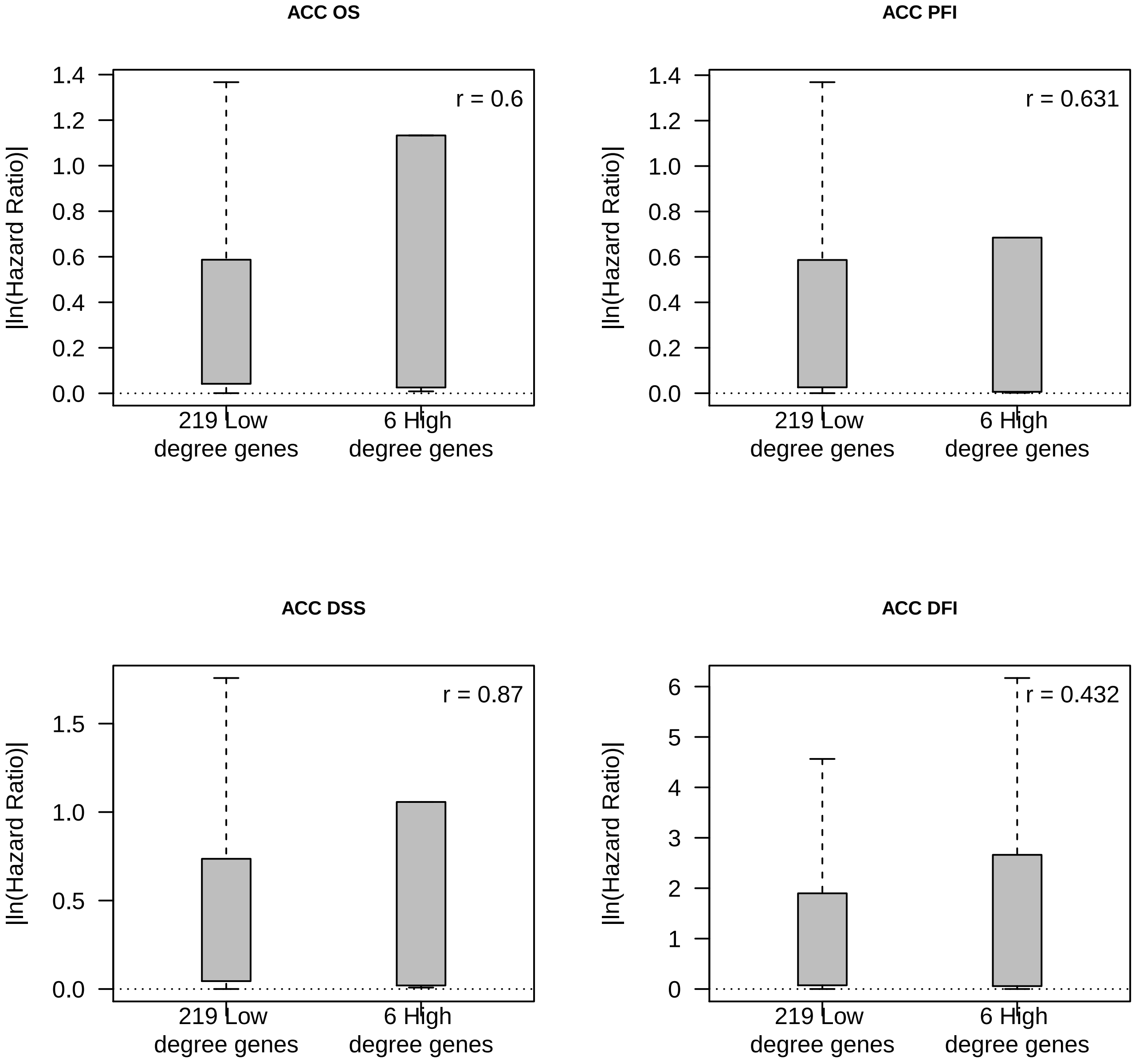

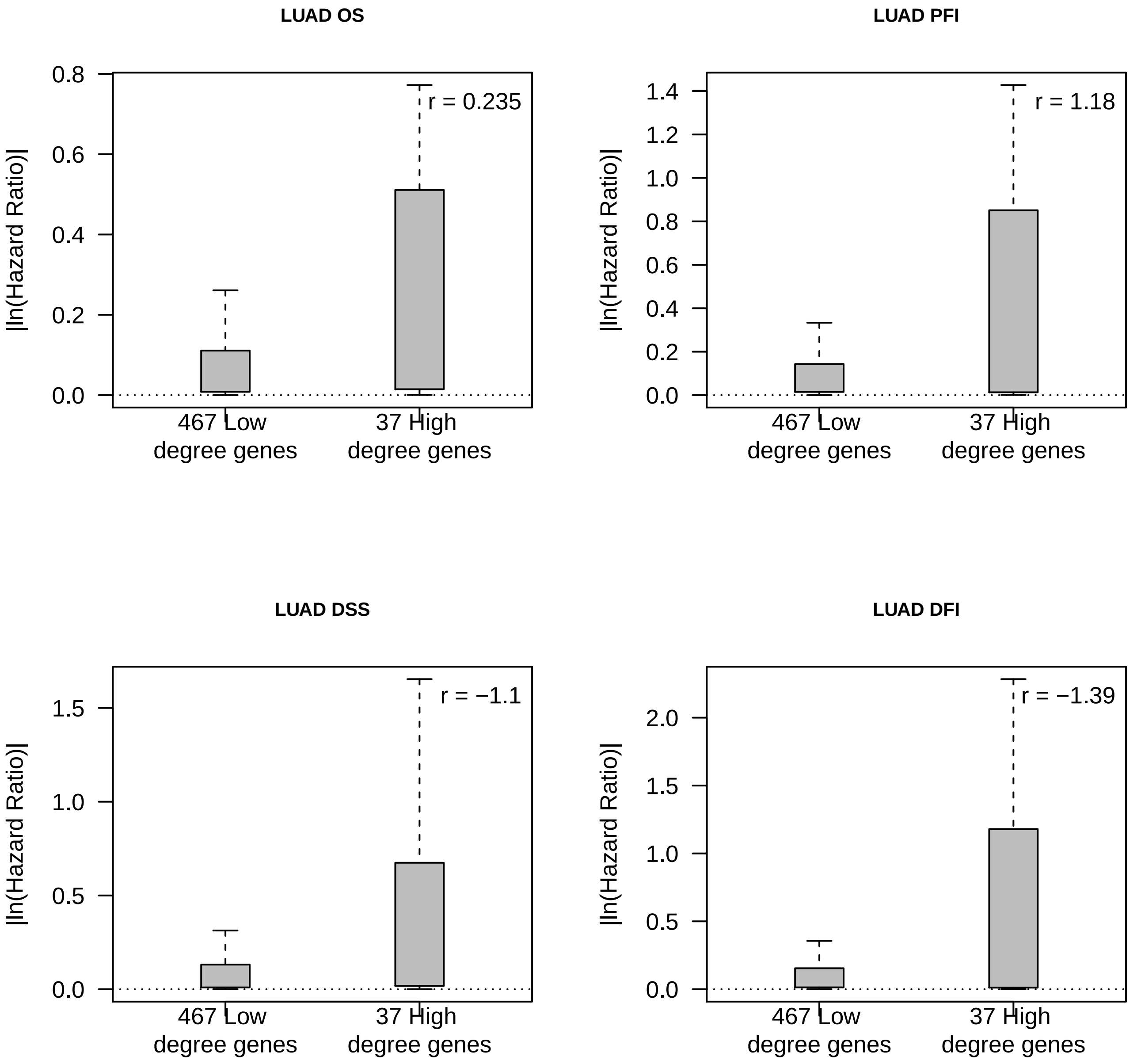
LUAD OS
r = 0.235
0.0
0.2
0.4
0.6
0.8
|ln(Hazard Ratio)|
467 Low
degree genes
37 High
degree genes
LUAD PFI
r = 1.18
0.0
0.2
0.4
0.6
0.8
1.0
1.2
1.4
|ln(Hazard Ratio)|
467 Low
degree genes
37 High
degree genes
LUAD DSS
r = −1.1
0.0
0.5
1.0
1.5
|ln(Hazard Ratio)|
467 Low
degree genes
37 High
degree genes
LUAD DFI
r = −1.39
0.0
0.5
1.0
1.5
2.0
|ln(Hazard Ratio)|
467 Low
degree genes
37 High
degree genes

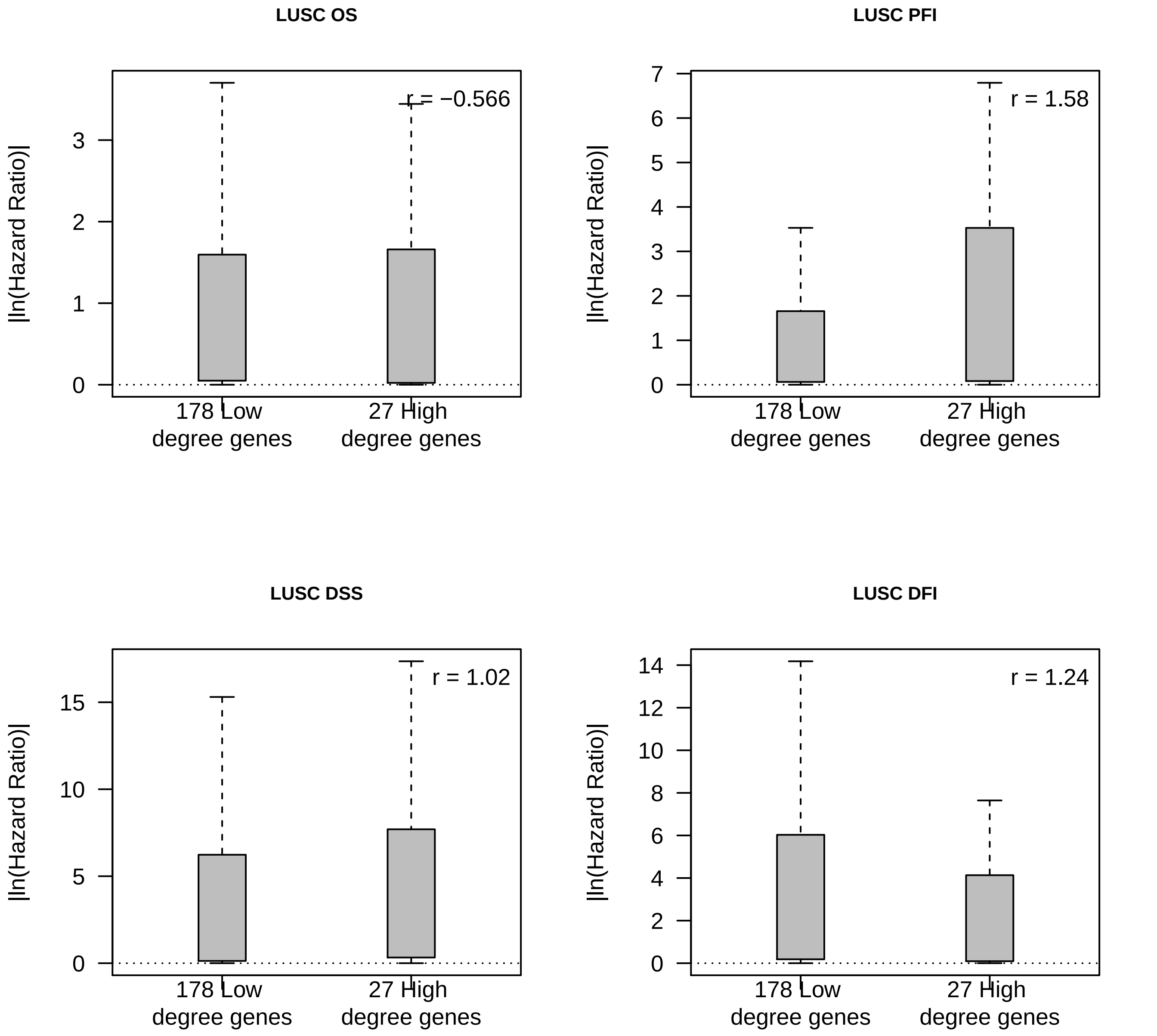

LUSC OS
r = −0.566
|ln(Hazard Ratio)|
0
1
2
3
178 Low
degree genes
27 High
degree genes
LUSC PFI
r = 1.58
|ln(Hazard Ratio)|
0
1
2
3
4
5
6
7
178 Low
degree genes
27 High
degree genes
LUSC DSS
r = 1.02
|ln(Hazard Ratio)|
0
5
10
15
178 Low
degree genes
27 High
degree genes
LUSC DFI
r = 1.24
|ln(Hazard Ratio)|
0
2
4
6
8
10
12
14
178 Low
degree genes
27 High
degree genes
