# Supplementary material for: Computing microRNA-gene interaction networks in pan-cancer using miRDriver
Source: Sci Rep. 2022 Mar 8;12:3717. doi: 10.1038/s41598-022-07628-z (PMC8904490; doi:10.1038/s41598-022-07628-z)
Supplement: Supplementary file 31 — Supplementary Information 31. [file 41598_2022_7628_MOESM31_ESM.pdf]

Computing microRNA-gene interaction networks in pan-cancer using miRDriver

Banabithi Bose, Matthew Moravec, and Serdar Bozdag

Supplemental Figure S22

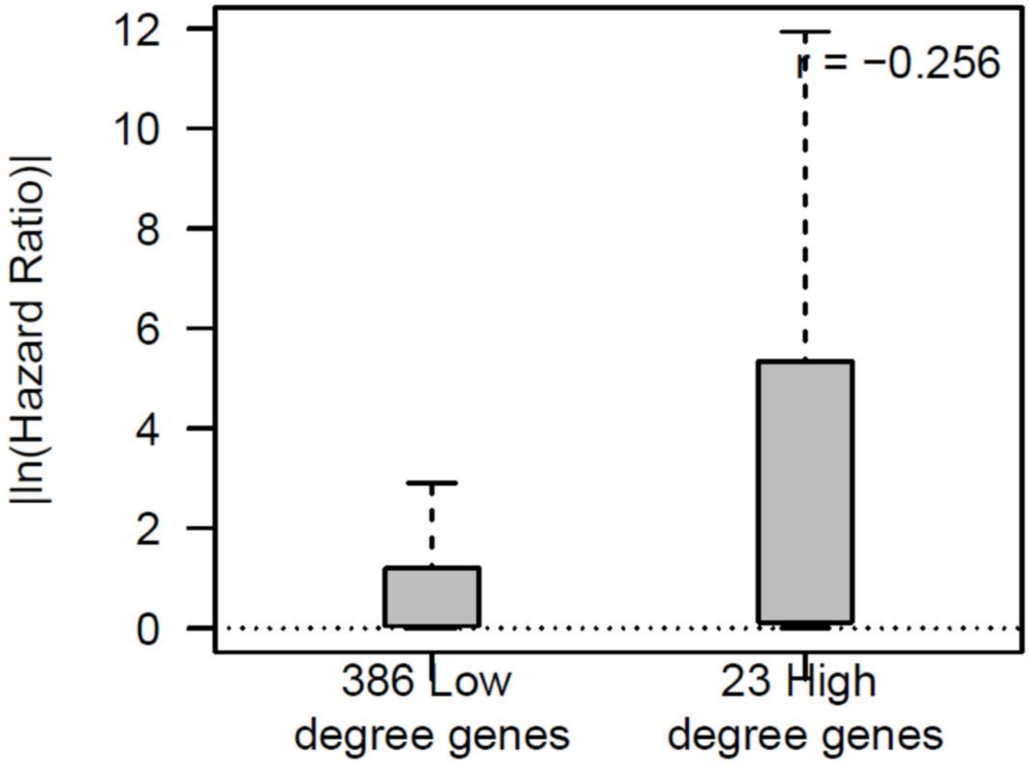

Boxplots of absolute values of natural logarithm of hazard ratios in high-degree and low-degree genes with r value of Mann–Whitney test.

Supplemental Figure S22

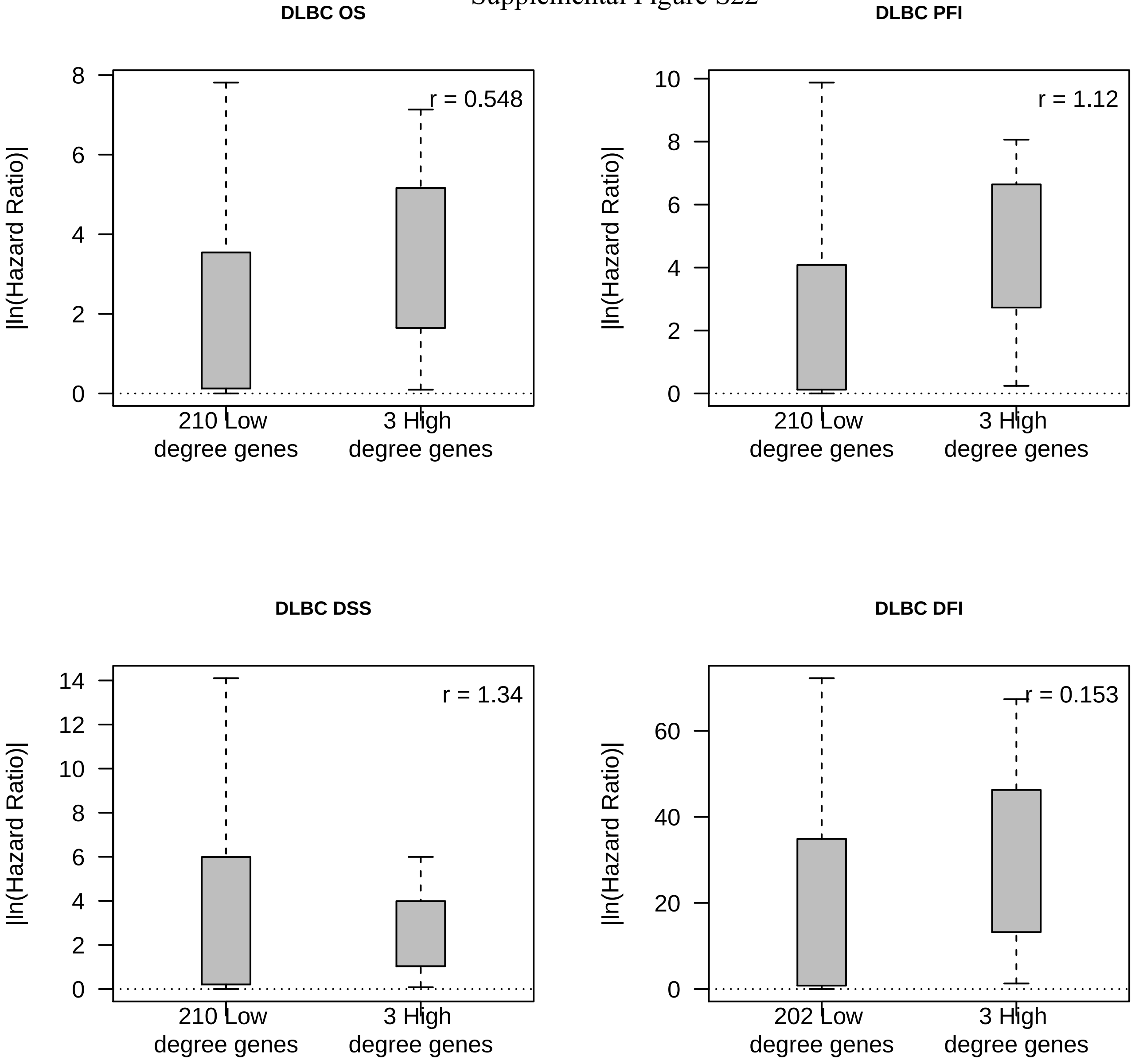

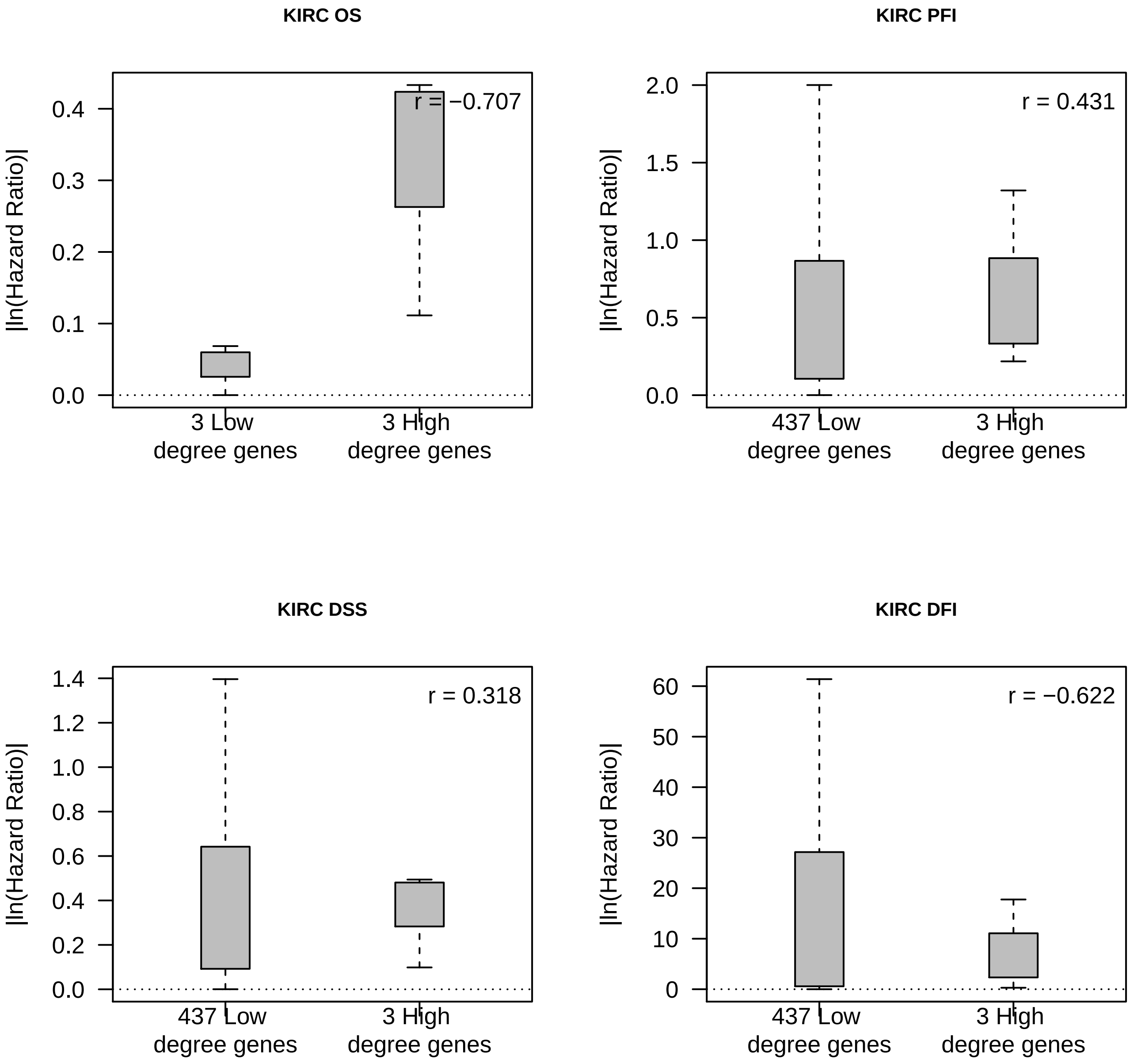
KIRC OS
|ln(Hazard Ratio)|
0.0
0.1
0.2
0.3
0.4
r = −0.707
3 Low
degree genes
3 High
degree genes
KIRC PFI
|ln(Hazard Ratio)|
0.0
0.5
1.0
1.5
2.0
r = 0.431
437 Low
degree genes
3 High
degree genes
KIRC DSS
|ln(Hazard Ratio)|
0.0
0.2
0.4
0.6
0.8
1.0
1.2
1.4
r = 0.318
437 Low
degree genes
3 High
degree genes
KIRC DFI
|ln(Hazard Ratio)|
0
10
20
30
40
50
60
r = −0.622
437 Low
degree genes
3 High
degree genes

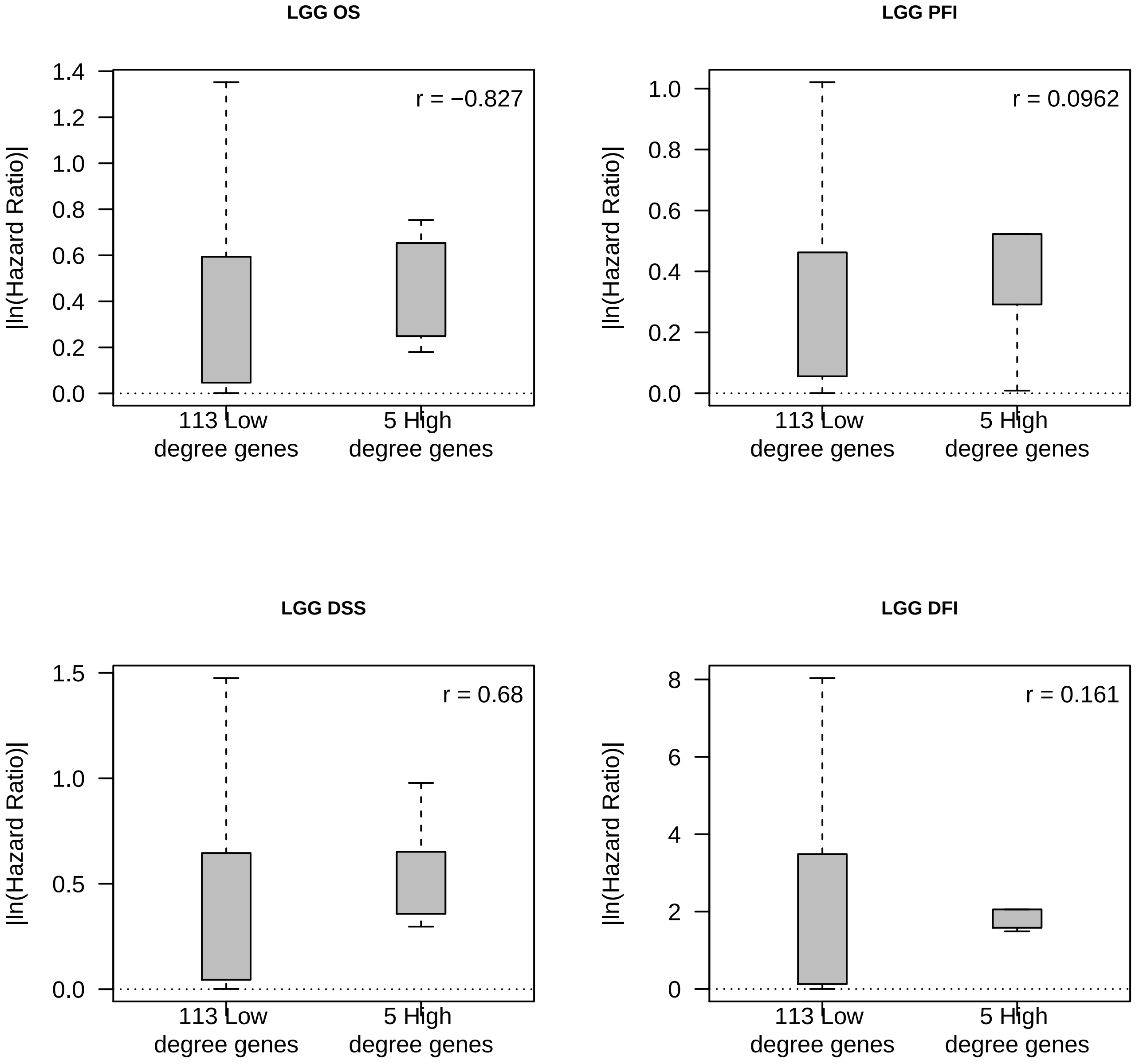

LGG OS
r = −0.827
|ln(Hazard Ratio)|
113 Low degree genes
5 High degree genes
LGG PFI
r = 0.0962
|ln(Hazard Ratio)|
113 Low degree genes
5 High degree genes
LGG DSS
r = 0.68
|ln(Hazard Ratio)|
113 Low degree genes
5 High degree genes
LGG DFI
r = 0.161
|ln(Hazard Ratio)|
113 Low degree genes
5 High degree genes
