# Supplementary material for: Computing microRNA-gene interaction networks in pan-cancer using miRDriver
Source: Sci Rep. 2022 Mar 8;12:3717. doi: 10.1038/s41598-022-07628-z (PMC8904490; doi:10.1038/s41598-022-07628-z)
Supplement: Supplementary file 32 — Supplementary Information 32. [file 41598_2022_7628_MOESM32_ESM.pdf]

Computing microRNA-gene interaction networks in pan-cancer using miRDriver

Banabithi Bose, Matthew Moravec, and Serdar Bozdag

Supplemental Figure S23

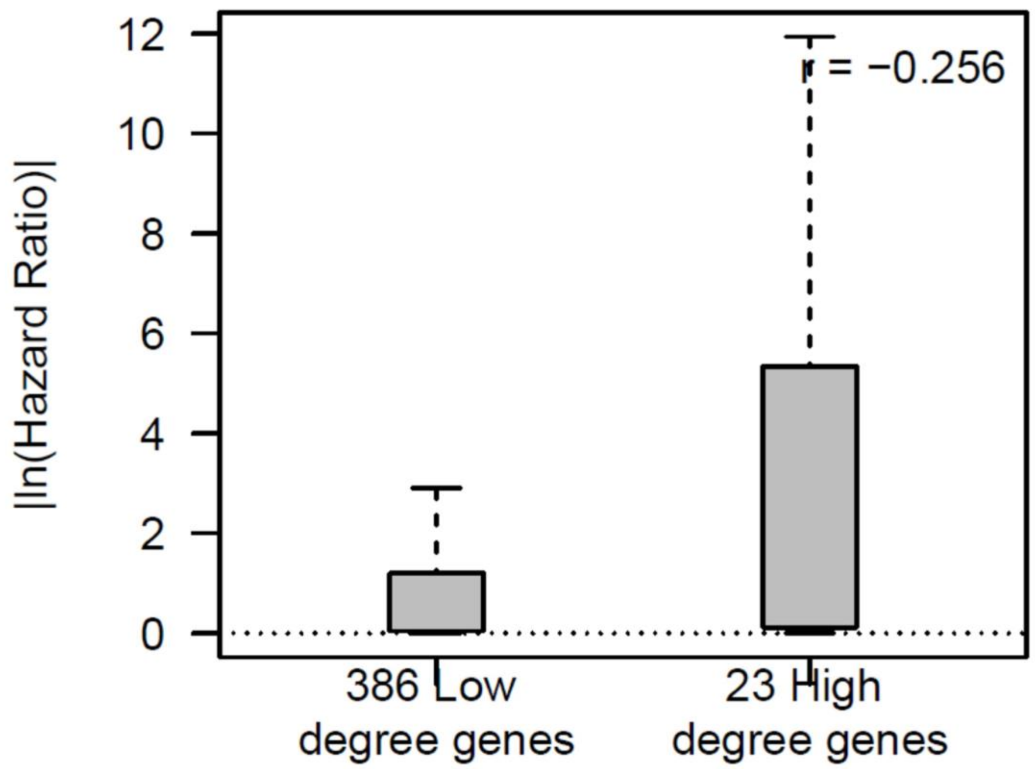

Boxplots of absolute values of natural logarithm of hazard ratios in high-degree and low-degree genes with r value of Mann–Whitney test.

Supplemental Figure S23

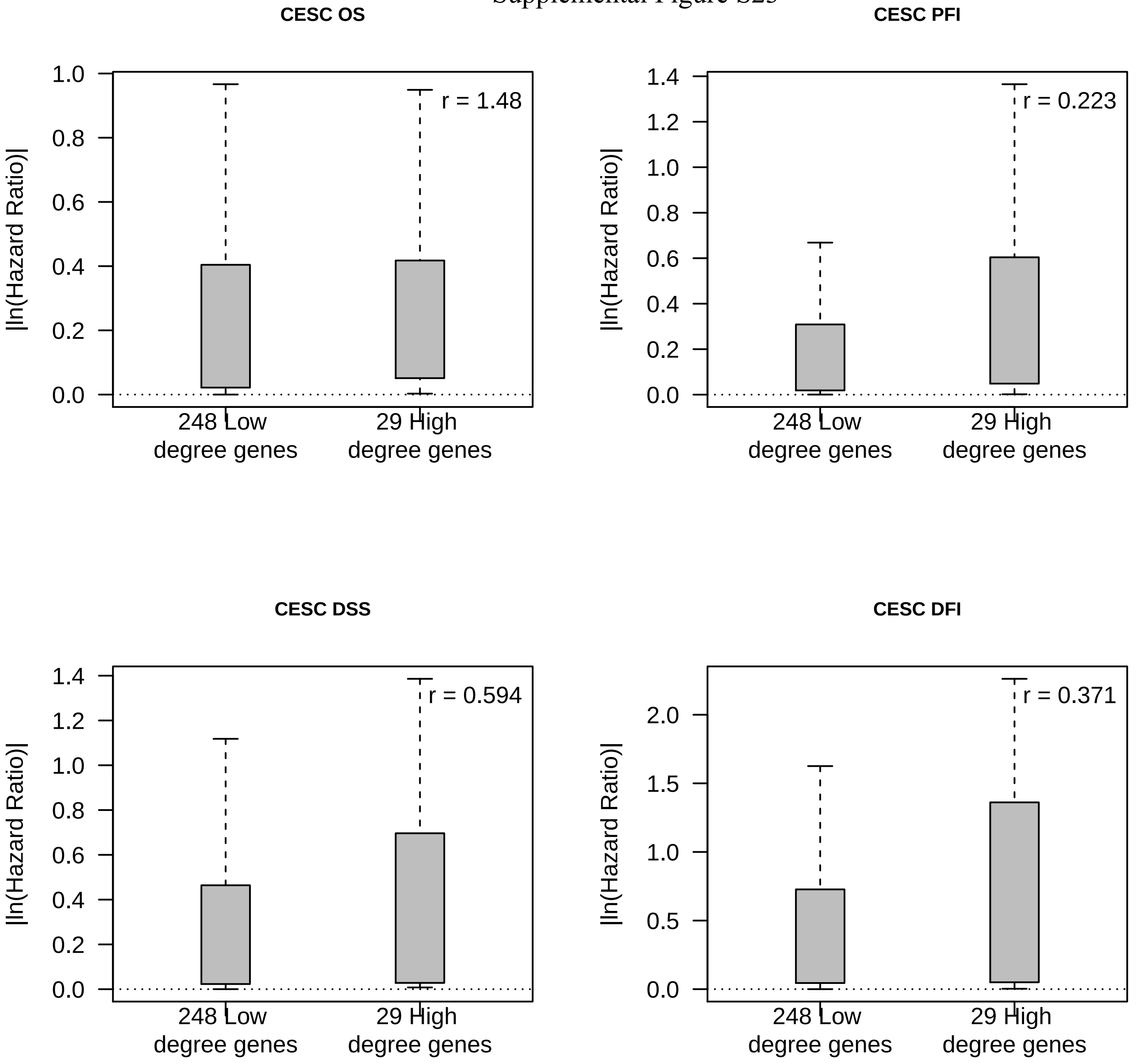

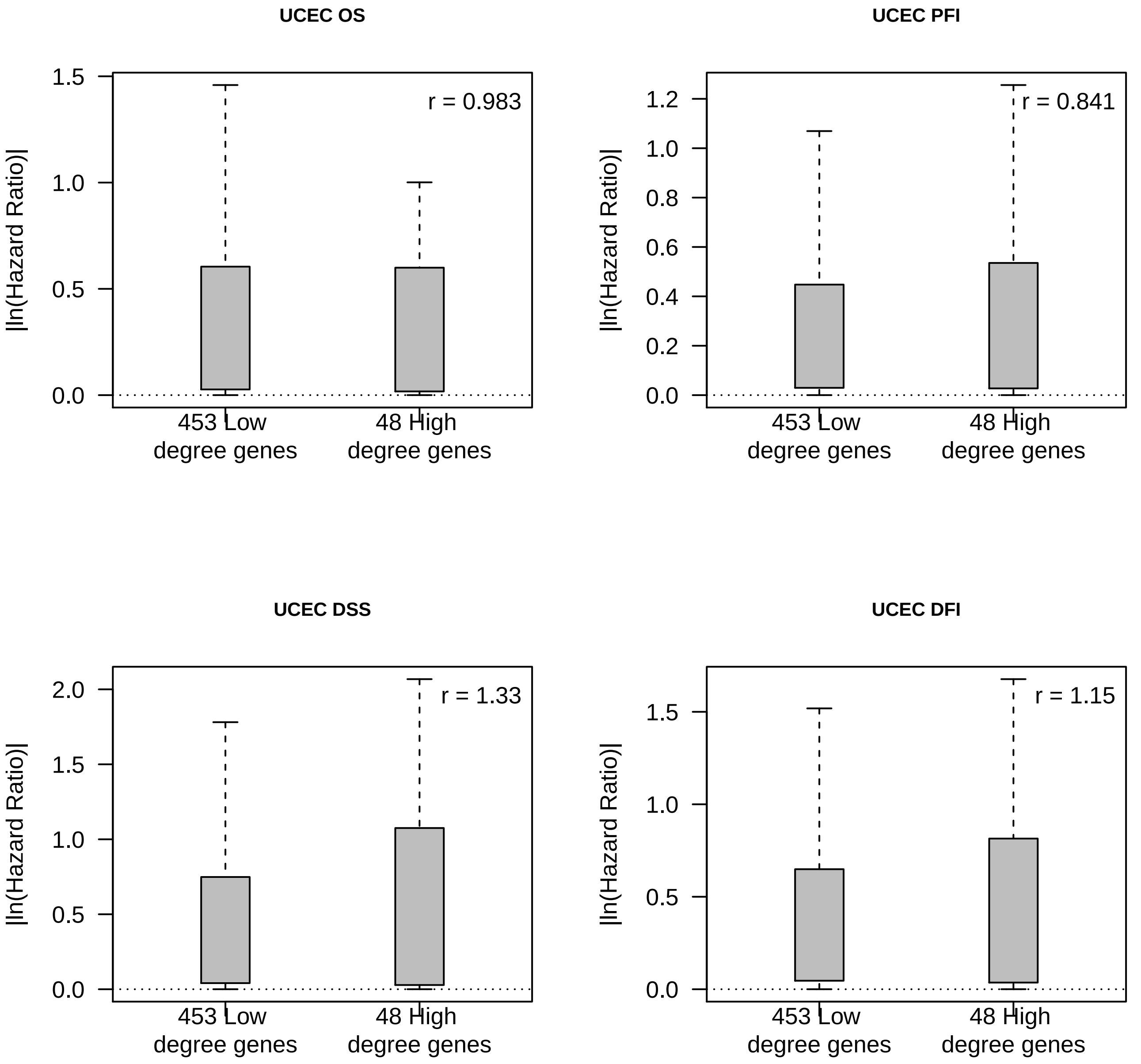
UCEC OS
r = 0.983
|ln(Hazard Ratio)|
0.0
0.5
1.0
1.5
453 Low degree genes
48 High degree genes
UCEC PFI
r = 0.841
|ln(Hazard Ratio)|
0.0
0.2
0.4
0.6
0.8
1.0
1.2
453 Low degree genes
48 High degree genes
UCEC DSS
r = 1.33
|ln(Hazard Ratio)|
0.0
0.5
1.0
1.5
2.0
453 Low degree genes
48 High degree genes
UCEC DFI
r = 1.15
|ln(Hazard Ratio)|
0.0
0.5
1.0
1.5
453 Low degree genes
48 High degree genes
