# Supplementary material for: Computing microRNA-gene interaction networks in pan-cancer using miRDriver
Source: Sci Rep. 2022 Mar 8;12:3717. doi: 10.1038/s41598-022-07628-z (PMC8904490; doi:10.1038/s41598-022-07628-z)
Supplement: Supplementary file 33 — Supplementary Information 33. [file 41598_2022_7628_MOESM33_ESM.pdf]

Computing microRNA-gene interaction networks in pan-cancer using miRDriver

Banabithi Bose, Matthew Moravec, and Serdar Bozdag

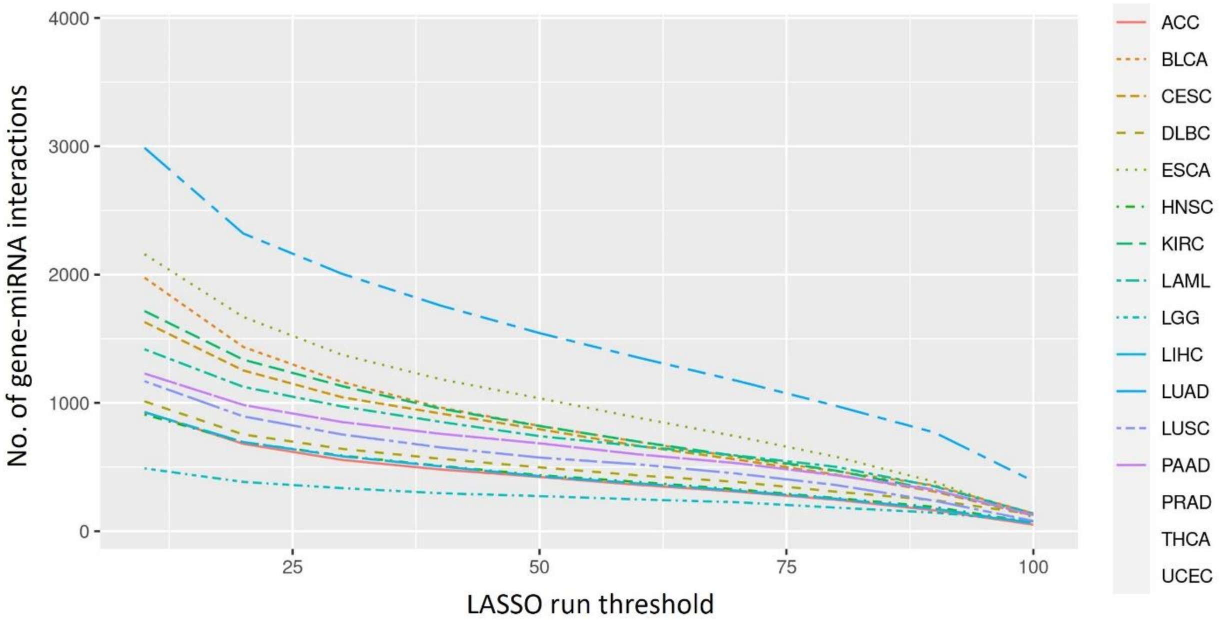

Supplemental Figure S24: Plots of the number computed miRNA-gene interactions with different LASSO runs for computing the miRNA regulators with non-zero coefficients.
