# Supplementary material for: Computing microRNA-gene interaction networks in pan-cancer using miRDriver
Source: Sci Rep. 2022 Mar 8;12:3717. doi: 10.1038/s41598-022-07628-z (PMC8904490; doi:10.1038/s41598-022-07628-z)
Supplement: Supplementary file 34 — Supplementary Information 34. [file 41598_2022_7628_MOESM34_ESM.zip › R package miRDriver Vignette 2022.pdf]

miRDriver

Banabithi Bose, Matt Moravec, Serdar Bozdag

9/9/2020

Contents

1 Introduction
2 Example Datasets
- 2.1 mirna_bedfile
- 2.2 RNA-Seq count data
- 2.3 Lesions file
- 2.4 hg38 genes coordinate bedfile
- 2.5 CNVData
- 2.6 mirnaData
- 2.7 RSeqData
- 2.8 TFData
- 2.9 Dorothea_data
- 2.10 methylationData

3 Using the functions
- 3.1 Step 1 Finding Gistic Regions
  - 3.1.1 getRegionWiseGistic
- 3.2 Step 2 Finding Differentially Expressed Trans Genes
  - 3.2.1 getDifferentiallyExpressedGenes
  - 3.2.2 makingTransAndCisGenes
- 3.3 Step 3 Gathering Cis Genes and Cis miRNA For Each Trans Gene
  - 3.3.1 gatherCisGenes
- 3.4 Step 4 Selecting Potential miRNA’s Regulating DE Trans Genes
  - 3.4.1 getTransGenePredictorFile
  - 3.4.2 lassoParallelForTransGene

4 Summary
5 Acknowledgements

1 Introduction

miRDriver is a computational tool that infers copy number aberration-based miRNA-gene interactions in cancer utilizing multi-omics datasets. miRDriver has four computational steps. In the first step, it finds frequently aberrated copy number regions of cancer samples utilizing the GISTIC2.0 tool

(https://cloud.genepattern.org/ (https://cloud.genepattern.org/)). In the second step, for each GISTIC region miRDriver computes differentially expressed (DE) genes between frequently aberrated and non-aberrated sample groups. In the third step, miRDriver retrieves DE genes and miRNAs that reside in the aberrated regions (i.e., cis genes and cis miRNAs) and retrieves DE genes that are outside of aberrated regions (i.e., trans genes). In the last step, miRDriver employs a LASSO-based regression model to compute potential miRNA regulators of the trans genes.

```
library(miRDriver)
```

2 Example Datasets

The example datasets are provided to run all four steps of miRDriver and can be accessed using data("miRDriverData") after the package installation. This includes genomic data (gene expression, copy number variation, transcription factor) and epigenomic data (methylation). The gene expression data can be RNASeq or Microarray with proper normalization, although the code has not been tested on Microarray data. The first step of miRDriver requires segmented copy-number data. The Lasso step of miRDriver requires gene-centric copy number data. The methylation data needs to be gene-centric beta values. For further details on how to preprocess the datasets, one should refer to the method paper [Bose, B. & Bozdag, S. miRDriver: A Tool to Infer Copy Number Derived miRNA-Gene Networks in Cancer. in Proceedings of the 10th ACM International Conference on Bioinformatics, Computational Biology and Health Informatics 366–375 (Association for Computing Machinery, 2019). doi:10.1145/3307339.3342172 (doi:10.1145/3307339.3342172).]. The example datasets included in this package are dummy datasets that are used to run the code in the vignette, but not to produce meaningful results. Although the example datasets in the vignette contain ENSEMBL IDs for the genes, this tool can be run with any gene id format, such as HGNC. We downloaded and pre-processed the original multi-omics datasets from TCGA (The Cancer Genome Atlas) database for eighteen different cancer types. Our pre-processed TCGA datasets can be accessed from Figshare via https://figshare.com/s/7400ad8445b2e78e4636 (https://figshare.com/s/7400ad8445b2e78e4636).

2.1 mirna_bedfile

This is an R dataframe object where the data represents the genomic position of the miRNAs. The rows in this dataset are the chromosomal regions that start with "chr" followed by chromosome numbers as integers 1-22. The columns are "chromosome number", "start position", "end position", and "miRNA ID".

```
data("miRDriverData")
mirna_bedfile[1:5, ]
```

```
##                         chr   start     end          mirna
## chr1:17358-17403     chr1   17358   17403 hsa-mir-6859-1
## chr1:567741-567783   chr1  567741  567783   hsa-mir-6723
## chr1:1102494-1102535 chr1 1102494 1102535   hsa-mir-200b
## chr1:1102536-1102565 chr1 1102536 1102565   hsa-mir-200b
## chr1:1103241-1103320 chr1 1103241 1103320   hsa-mir-200a
```

2.2 RNA-Seq count data

This is an R dataframe object that contains RNA-Seq count data for each sample and each gene in the study. The rows are the genes. The columns are the sample IDs for the patients.

```
Dummy_RnaSeq_Count_Data[1:5,1:3]
```

```
##                 TCGA-61-2097-01A TCGA-09-1670-01A TCGA-61-1911-01A
## ENSG00000001626               15              735             154[illegible]
## ENSG00000002726             5106              503              70[illegible]
## ENSG00000003989              904             7587               3[illegible]
## ENSG00000004848              625              146               2[illegible]
## ENSG00000005020             2267             3241              65[illegible]
```

2.3 Lesions file

This is a text file produced by the GISTIC2.0 tool. Here, the first 9 columns are "Unique.Name, Descriptor, Wide.Peak.Limits, Peak.Limits, Region.Limits, q.values". After the first 9 columns there are sample id's with their aberration status "0", "1", "2". "0" represents no copy number aberration, "1" represents a low level copy number aberration, and "2" represents a high level copy number aberration.

```
x <-
    read.table(
        system.file("extdata", "Dummy.gistic.lesions.txt", package =
        sep = "\t",
        header = TRUE
    )
x[1:5, c(1, 10:13)]
```

```
##             Unique.Name TCGA.13.1483.01A TCGA.13.1497.01A TCGA.13.
## 1 Amplification Peak  1                0                1
## 2 Amplification Peak  2                1                0
## 3 Amplification Peak  3                0                1
## 4 Amplification Peak  4                0                1
## 5 Amplification Peak  5                1                1
##   TCGA.13.1817.01A
## 1                0
## 2                2
## 3                2
## 4                0
## 5                0
```

2.4 hg38 genes coordinate bedfile

This is an R dataframe object that contains the coordinates for all the genes in the human genome. There are four columns, such as the chromosome, the start position, the end position and the ENSEMBLE ID. The rows are labeled with the gene IDs.

```
Genes_coord[1:5,]
```

```
##      chr start   end            gene
## 4378   1 11869 14409 ENSG00000223972
## 4482   1 14404 29570 ENSG00000227232
## 5272   1 17369 17436 ENSG00000278267
## 3968   1 29554 31109 ENSG00000243485
## 560    1 30366 30503 ENSG00000284332
```

2.5 CNVData

This is an R dataframe object with gene-centric copy number values. The first column, labeled as “sample”, contains the sample IDs. The rest of the columns are the copy number values of the genes.

```
dummy_CNVData10[1:5,1:3]
```

```
##               sample ENSG00000001626 ENSG00000002726
## 1: TCGA-09-1670-01A          0.3184          0.3252
## 2: TCGA-13-0891-01A          0.2833          0.8425
## 3: TCGA-13-1489-01A          0.2876          0.8009
## 4: TCGA-13-1497-01A          0.2384          0.9225
## 5: TCGA-13-1498-01A          0.1138          0.1126
```

2.6 mirnaData

This is an R dataframe object that contains the miRNA expression for the samples. The first column labeled as “sample”, contains the sample IDs. The rest of the columns are the miRNAs with expression values.

```
dummy_mirnaData10[1:5,1:3]
```

```
##              sample hsa-let-7a-3 hsa-let-7b
## 1: TCGA-09-1670-01A    34346.536  130664.29
## 2: TCGA-13-0891-01A     6745.882   58918.88
## 3: TCGA-13-1489-01A    18635.593  151117.36
## 4: TCGA-13-1497-01A    23146.002  159291.97
## 5: TCGA-13-1498-01A    16284.750  101423.96
```

2.7 RSeqData

This is an R dataframe object that contains the gene expression data for the samples. The first column is labeled as “Sample” and contains the Sample IDs. The rest of the columns are the genes with expression values.

```
dummy_RSeqData10_200[1:5,1:3]
```

```
##             sample ENSG00000001626 ENSG00000002726
## 1 TCGA-61-2097-01A     0.001869699       4.3178793
## 2 TCGA-09-1670-01A     0.040091684       0.1861419
## 3 TCGA-61-1911-01A     0.289759305       0.8952920
## 4 TCGA-13-1489-01A     0.074605243       5.0108765
## 5 TCGA-24-1844-01A     0.001536027       0.1058129
```

2.8 TFData

This is an R dataframe object which contains gene, transcription factor (TF) with a confidence score column for their interactions. The first column, labeled as “V1” contains the genes, the second column, labeled as “TF”, contains the transcription factors, and the third column is labeled as “Confidence”. The “Confidence” column can work with any numeric value and is supposed to filter the interactions based on a maximum threshold that we set as “5” as default. If the user uses our gene-TF interaction file which was curated from DoRothEA gene set resource (https://saezlab.github.io/dorothea/ (https://saezlab.github.io/dorothea/)) for cancer-related TF-target interactions the “Confidence” score as “5” will select the highest confidence level for the interactions. If the user provides their TF-target file then they require to provide this “Confidence” column; in the case where the confidence scores are unknown, the user can set all values in the column as “5” to run the tool.

```
dummy_TFData[1:5,]
```

```
##                 V1              TF Confidence
## 1: ENSG00000001626 ENSG00000100811          5
## 2: ENSG00000001626 ENSG00000105698          4
## 3: ENSG00000001626 ENSG00000109320          4
## 4: ENSG00000001626 ENSG00000112658          3
## 5: ENSG00000001626 ENSG00000115415          1
```

2.9 Dorothea_data

This is an R dataframe object named "Dorothea_data" containing cancer-related TF-target interactions from the DoRothEA gene set resource (https://saezlab.github.io/dorothea/ (https://saezlab.github.io/dorothea/)). DoRothEA TF-targets were curated and collected from different types of evidence such as (1) literature curated resources, (2) ChIP-seq peaks (includes ENCODE ChIP-seq profiles), (3) TF binding site motifs, and (4) interactions inferred directly from gene expression. In Dorothea_data, the first column, labeled as "V1" contains the genes, the second column, labeled as "TF", contains the TFs, and the third column labeled as "Confidence" contains a confidence level column for the interactions. This confidence level ranges from "5" (highest confidence) to "1" (lowest confidence). Interactions that are supported by all four lines of evidence from DoRothEA, manually curated by experts in specific reviews, or supported both in at least two curated resources are considered to be highly reliable and were assigned a "5" level. Level "4"-"2" are reserved for curated and/or ChIP-seq interactions with different levels of additional evidence. Finally, the "1" level is used for interactions that are uniquely supported by computational predictions. Users can apply any confidence level threshold to filter interactions. Users can also provide their TF-target file in the same format as described above. The default value has been set to "5" as we believe that using high confident interactions would be crucial to make LASSO step more stable.

```
Dorothea_data[1:5,]
```

```
##                V1              TF Confidence
## 1 ENSG00000121410 ENSG00000124827          1
## 2 ENSG00000121410 ENSG00000198046          1
## 3 ENSG00000121410 ENSG00000180535          1
## 4 ENSG00000121410 ENSG00000111249          1
## 5 ENSG00000121410 ENSG00000173041          1
```

2.10 methylationData

This is an R dataframe object that contains the gene-centric DNA methylation beta values. The first column labeled as "sample" contains the sample IDs. The rest of the columns are the genes with methylation values.

```
dummy_methylationData10[1:5,1:3]
```

```
##              sample ENSG00000001626 ENSG00000002726
## 1: TCGA-09-1670-01A      0.82434290      0.31135093
## 2: TCGA-13-0891-01A      0.88838544      0.03779997
## 3: TCGA-13-1489-01A      0.11535679      0.08279489
## 4: TCGA-13-1497-01A      0.90558531      0.82988443
## 5: TCGA-13-1498-01A      0.54302476      0.12792955
```

3 Using the functions

The following sections describe all the functions of miRDriver. The functions are dependent on each other in the sense that the user needs to finish the first step of miRDriver to start the second step and so on.

3.1 Step 1 Finding Gistic Regions

Step one of the miRDriver pipeline finds frequently aberrated copy number regions among cancer patients. This step consists of one function, *getRegionWiseGistic()*.

3.1.1 getRegionWiseGistic

This function uses the lesions file to find the aberrations for each peak. The lesions file, as stated in the example data sets section, can be created with the GISTIC2.0 module on genepattern.com

(https://cloud.genepattern.org/gp/pages/index.jsf?lsid=urn:lsid:broad.mit.edu:cancer.software.genepattern.module.analysis:00125:6.15.28 (https://cloud.genepattern.org/gp/pages/index.jsf?lsid=urn:lsid:broad.mit.edu:cancer.software.genepattern.module.analysis:00125:6.15.28)).

If the user utilizes the GISTIC2.0 to make the lesions file, then the lesions file will be labeled all_lesions.conf_90.txt. In the example code, this file is used in the function as the argument *gisticfile*. The *gistic_bedfile* argument should be either TRUE or FALSE, representing if the user would like the function to make a gistic_bedfile. The *mirdirectory* argument needs to be the file path that the user wants the results to be in. The *mirdirectory* needs to be the same for all functions in the same analysis.The argument *Aber* can be set as “Amp”,“Del” or “All” for getting genes from amplified, deleted or from both types of GISTIC regions; the default is *Aber = “All”*.

Arguments:

gisticfile - a character string for the file path of the GISTIC output file

gistic_bedfile - a logical value for whether or not one wants to create a gistic_bedfile (TRUE/FALSE)

mirdirectory - a character string for the file path of the directory one wants the results in

aber - a character string that can be set as “Amp”,“Del” or “All” for getting genes from amplified, deleted or from both types of GISTIC regions; the default is *Aber = “All”*

```
mirdirectory <- tempdir()
getRegionWiseGistic(
    gisticfile = system.file("extdata", "Dummy.gistic.lesions.txt", p
    gistic_bedfile = TRUE,
    mirdirectory = mirdirectory
)
```

```
## [1] "gistic_bedfile.rda is created in mirdirectory under 'mirDrive
```

Output: The output from this function can be found in a subdirectory of the mirdirectory entitled mirDriverFold/miRDriver_Step1. Inside mirDriverFold/miRDriver_Step1 there is a subdirectory entitled GisticResults and a subdirectory entitled Regionwise_Gistic_Files.

```
load(
    file.path(
        mirdirectory,
        "mirDriverFold",
        "miRDriver_Step1",
        "GisticResults",
        "gistic_bedfile.rda"
    )
)
region[1:5, ]
```

```
##    chr     start       end                  peak
## 1 chr1  32199266  47698949 Amplification Peak  1
## 2 chr1 119981333 163635638 Amplification Peak  2
## 3 chr1 222931614 248956422 Amplification Peak  3
## 4 chr2  28408552  29013830 Amplification Peak  4
## 5 chr2 170235978 180989290 Amplification Peak  5
```

```
x <-
    data.frame(read.table(
        file.path(
            mirdirectory,
            "mirDriverFold",
            "miRDriver_Step1",
            "Regionwise_Gistic_Files",
            "Amplification Peak  5.txt"
        ),
        sep = "\t",
        header = TRUE
    ))

x[1:5, ]
```

```
## [1] "TCGA.13.1483.01A ,1, aberrated"  " TCGA.13.1497.01A ,1, aberr
## [3] " TCGA.13.0760.01A ,1, aberrated" " TCGA.29.1702.01A ,1, aberr
## [5] " TCGA.25.2042.01A ,1, aberrated"
```

3.2 Step 2 Finding Differentially Expressed Trans Genes

Step two of the miRDriver pipeline is to find the differentially expressed (DE) genes. This step uses two functions, *getDifferentiallyExpressedGenes()* and *makingTransAndCisGenes()*.

3.2.1 getDifferentiallyExpressedGenes

This function finds the differentially expressed genes from the peak wise GISTIC region files generated by the getRegionWiseGistic function and RNASeq count data. This function uses parallel computation by setting the argument value of *ncore*; the default is *ncore = 1*. The argument *RNAcount* needs to be set to the file path for the RNASeq count data. The argument *mirdirectory* needs to be the same as in all previous functions. The argument *DEgene* can be set as “Up”,“Down” or “All” for getting up-regulated, down-regulated or both up and down genes, respectively from the GISTIC regions; the default is *DEgene = “All”*

Arguments:

ncore - an integer value for the number of cores user wants to use (default = 2)

RNAcount - R dataframe object containing RNASeq count data. The rows are the genes. The columns are the sample IDs for the patients

mirdirectory - a character string for the file path of the directory one wants the results in, must be the same as in all previous functions.

DEgene - a character string for specifying up regulated, down regulated or both type of genes by setting “Up”,“Down” or “All”, respectively (default = “All”)

```
getDifferentiallyExpressedGenes(ncore = 1,
                                RNACount = Dummy_RnaSeq_Count_Data,
                                mirdirectory = mirdirectory)
```

```
## [1] "Differential analysis has been done, output files in folder m
```

Output: The output for this function can be found in the mirdirectory in mirDriverFold/miRDriver_Step2/UpDownGeneLibrary. These files are the intermediary files for step two.

```
x <-
    read.table(
        file.path(
            mirdirectory,
            "mirDriverFold",
            "miRDriver_Step2",
            "UpDownGeneLibrary",
            "UPgene_Amplification Peak  5.txt"
        ),
        sep = "",
        header = TRUE
    )
x[1:3, ]
```

```
##         Row.Names    logFC   logCPM        F     PValue
## 1 ENSG00000018625 4.738197 6.624823 5.110309 0.04036715
## 2 ENSG00000019186 5.031303 6.079345 7.318355 0.01716726
## 3 ENSG00000036828 5.711382 4.063535 5.152652 0.03966219
```

3.2.2 makingTransAndCisGenes

This function finds the trans and cis genes for each GISTIC region peak. The trans and cis genes will be stored in R dataframe objects located in the file path ~/mirdirectory/miRDriver_Step2/Trans_Cis_Files/peak/. In the file path the mirdirectory is the mirdirectory specified in the *mirdirectory* argument and should be the same as the mirdirectory used in the previous functions. The *peak* in the file path represents the subdirectories for each peak which will follow the GISTIC region name convention, such as “Amplification Peak 1”. The *gistic_bedfile* argument needs to be set to an R dataframe object that follows the convention of having four columns, “chromosome”, “start position”, “end position”, and “peak”. The chromosome column will have the chromosome number in the form “chr1”. The start and end positions will be integers representing the start and end positions on the chromosome. The peak column will have a string representing the peak following the convention of “Amplification Peak 1”. The required gistic_bedfile can be made utilizing the *getRegionWiseGistic()* function.

Arguments:

Genes_coord_bedfile - A R dataframe object that fits the format of a bedfile for gene coordinates

gistic_bedfile - A R dataframe object that fits the format of a bedfile for the GISTIC results with amplification/deletion peaks with patients

mirdirectory - a character string for the file path of the directory one wants the results in, must be the same as all previous functions

```
load(
    file.path(
        mirdirectory,
        "mirDriverFold",
        "miRDriver_Step1",
        "GisticResults",
        "gistic_bedfile.rda"
    )
)
makingCisAndTransGenes(
    Genes_coord_bedfile = Genes_coord,
    gistic_bedfile = region,
    mirdirectory = mirdirectory
)
```

```
## [1] "Trans genes and cis genes files produced in folder mirDriverF
```

Output: The output for this function can be found in subdirectory of the mirdirectory entitled mirDriverFold/miRDriver_Step2/Trans_Cis_Files with trans_genes.Rda files under each peak/region directory.

```
load(
    file.path(
        mirdirectory,
        "mirDriverFold",
        "miRDriver_Step2",
        "Trans_Cis_Files",
        "Amplification Peak  1",
        "trans_genes.Rda"
    )
)
trans_genes[1:3, ]
```

```
## [1] "ENSG00000003989" "ENSG00000046774" "ENSG00000075891"
```

```
message("These are the trans genes")
```

```
## These are the trans genes
```

3.3 Step 3 Gathering Cis Genes and Cis miRNA For Each Trans Gene

Step three of the miRDriver pipeline is the gathering of the cis genes and cis miRNA for each trans gene. This step uses one function, *gatherCisGenes()*.

3.3.1 gatherCisGenes

This function collects all the DE cis genes and all miRNAs for each trans gene. This function uses the output from the *makingCisAndTransGene()* function as well as a *mirna_bedfile* and a *gistic_bedfile*. The gistic_bedfile is described in the *makingCisAndTransGene()* function description and can be made using the *getRegionWiseGistic()* function. The *mirna_bedfile* is described in the example datasets section and needs to be provided by the user. The output from this function is in the folders gene_all_DEcis and gene_all_mirna. In these folders, there is a folder for each trans gene where all the miRNA's or DE cis genes are listed. As with all the functions the mirdirectory needs to be consistent with the other functions.

Arguments:

ncore - An integer value that represents the number of cores that the user wants to use (default = 2)

mirna_bedfile - A dataframe in the format of a bedfile for all of the miRNA

gistic_bedfile - A dataframe in the format of a bedfile for the GISTIC results

mirdirectory - a character string for the file path of the directory one wants the results , must be the same as in all previous functions

```
load(
    file.path(
        mirdirectory,
        "mirDriverFold",
        "miRDriver_Step1",
        "GisticResults",
        "gistic_bedfile.rda"
    )
)
gatherCisGenes(
    ncore = 1,
    mirna_bedfile = mirna_bedfile,
    gistic_bedfile = region,
    mirdirectory = mirdirectory
)
```

```
## [1] "mirDriverFold/miRDriver_Step3/gene_all_mirna and gene_all_DE
```

Output: The output for this function can be found in the mirdirectory in mirDriverFold/miRDriver_Step3. The miRNA files for each gene are in the folder gene_all_mirna and the DE cis genes for each gene are in the folder gene_all_DEcis. In this case there are no DECis genes for our output due to the dummy datasets, but the dataframes will follow the pattern of the miRNA dataframe.

```
load(
    file.path(
        mirdirectory,
        "mirDriverFold",
        "miRDriver_Step3",
        "gene_all_DEcis",
        "ENSG00000036828.Rda"
    )
)
TC
```

```
##    ENSG00000036828
## 1:
```

```
message("For this trans gene we do not have any cis gene")
```

```
## For this trans gene we do not have any cis gene
```

```
load(
    file.path(
        mirdirectory,
        "mirDriverFold",
        "miRDriver_Step3",
        "gene_all_mirna",
        "ENSG00000124205.Rda"
    )
)
GM[1:3, ]
```

```
##    ENSG00000124205
## 1:    hsa-mir-4254
## 2:    hsa-mir-5585
## 3:    hsa-mir-3605
```

3.4 Step 4 Selecting Potential miRNA's Regulating DE Trans Genes

Step four of the miRDriver pipeline is to select potential miRNAs regulating DE trans genes. This step uses two functions, *getTransGenePredictorFile()* and *lassoParallelForTransGene()*.

3.4.1 getTransGenePredictorFile

This function creates a TransGenePredictorFile for each trans gene based on the miRNA and DE cis files from the output of the *gatherCisGenes()* function and the user-provided dataframes *methylationData*, *RSeqData*, *CNVData*, *TFData*, *mirnaData*. The five dataframes needed for this function are described in the example datasets section. The TransGenePredictorFiles will contain information about the possible predictors for each trans gene based on the data provided. The TransGenePredictorFiles will be saved in the file path ~/mirdirectory/mirDriverFold/miRDriver_Step4/TransGenePredFile/. As always mirdirectory in the file path represents the mirdirectory chosen for the analysis and should be the same as the mirdirectory used in all previous functions. For *TFData*, the "Confidence" column can work with integers or numeric values and is supposed to filter the interactions based on a threshold that we set as "minConfidence = 5" as default. Here, users can provide their TF-target interaction data or they can use the Dorothea cancer-related dataset that we have provided with this tool. For the details, please check the descriptions of the datasets.

Arguments:

ncore - An integer value for the number of cores the user wants to use (default=2)

methylationData - a dataframe for the methylation data

RSeqData - a dataframe for the RNASeq data

CNVData - a dataframe for the copy number variation data

TFData - a dataframe for the transcription factor data

mirnaData - a dataframe for the miRNA data

mirdirectory - a character string for the file path of the directory one wants the results in, should be the same as in previous functions

minConfidence - an integer or numeric value for the *TFData* for filtering the "Confidence" column which is supposed to filter the interactions based on a threshold that we set as "minConfidence = 5" as default

```
getTransGenePredictorFile(
    ncore = 1,
    methylationData = dummy_methylationData10,
    RSeqData = dummy_RSeqData10_200,
    CNVData = dummy_CNVData10,
    TFData = dummy_TFData,
    mirnaData = dummy_mirnaData10,
    mirdirectory = mirdirectory
)
```

```
## [1] "All predictors are gathered for trans genes in mirDriverFold/
```

Output: The output for this function can be found in the mirdirectory in mirDriverFold/miRDriver_Step4/TransGenePredFile.

```
load(
    file.path(
        mirdirectory,
        "mirDriverFold",
        "miRDriver_Step4",
        "TransGenePredFile",
        "ENSG00000108231.Rda"
    )
)
GenePred[1:3, ]
```

```
##             sample ENSG00000108231 hsa.mir.3605 hsa.mir.6732 hsa.m
## 1 TCGA-09-1670-01A      0.02984883     20.43286      0.55224     (
## 2 TCGA-13-0891-01A      0.23257792     10.64719      0.00000     (
## 3 TCGA-13-1489-01A      0.02971115     11.58758      0.00000     1
##   hsa.mir.30e hsa.mir.30c.1 hsa.mir.6734 hsa.mir.6735 hsa.mir.6736
## 1    5671.776      1.656719     0.552240     1.380599     0.27612(
## 2    5256.163      6.210860     4.436329     0.000000     0.88726(
## 3    3391.129      2.015231     0.755711     0.251904     0.00000(
##   hsa.mir.5698 hsa.mir.190b hsa.mir.92b hsa.mir.6738 hsa.mir.9.1 h
## 1     2.485078     0.000000   2564.8764     0.828359   12.149269
## 2     0.887266     0.000000    591.8063     0.000000    5.323595
## 3    12.091384     0.755711   3579.8053     0.251904  226.209636
##   hsa.mir.5187 hsa.mir.4654       beta     cnv
## 1     2.761198     0.552240 0.24405089  0.3557
## 2     4.436329     0.000000 0.03760165 -0.1073
## 3     6.549499     0.503808 0.35141537 -0.5056
```

3.4.2 lassoParallelForTransGene

This function runs a LASSO regression to select the miRNA regulators responsible for the trans genes' expression variation. This function considered the gene-centric copy number, gene-centric DNA methylation, TFs, miRNA expression as independent variables and the trans gene's expression as the response variable coming from TransGenePredictorFiles created by the *getTransPredictorFile function()*. For each trans gene, out of all its candidate predictors (i.e., independent variables), LASSO selects a set of non-zero coefficient predictors. To find accurate results, each LASSO regression is set to run multiple times (the user specifies with the argument *numCounter*), with a certain percentage of times the LASSO coefficient to be non-zero (the user specifies this with the argument *nonZeroPercent*) for the predictors to be selected. The user also needs to provide the number of cross-validations (argument *Nfolds*) for each LASSO run. Users can leverage the parallel computation option in their high-performance computing environment by setting the argument *ncore* for all the available cores. Users are recommended to choose miRNA predictors with improved confidence levels by increasing the hyperparameters (*numCounter* and *nonZeroPercent*) that control the number of LASSO runs and the minimum number of times LASSO must pick a miRNA predictor in the *lassoParallelForTransGene()* function. The output from this function is found in the file path ~/mirdirectory/mirDriverFold/miRDriver_RESULTS/. The mirdirectory in the file path represents the directory chosen by the user and specified in the argument, mirdirectory. The argument *mirdirectory* needs to be set to the same directory as the mirdirectory in the previous steps. Furthermore, considering gene expression is controlled at multiple levels, including transcriptional regulation and post-transcriptional regulation, users can run the LASSO step in two phases. An experienced R user could run *lassoParallelForTransGene()* function twice. In the first run, only the transcriptional predictors could be utilized to explain the expression variation and can be accessed from the *LassoMinCoeff" directory in the ~/mirdirectory/mirDriverFold/miRDriver_RESULTS/. In the second run, post-transcriptional predictors and the residual of the first LASSO run can be utilized as the independent and dependent variables, respectively. Alternatively, if the user has the transcriptional and post-transcriptional expression change data, both LASSO runs can be performed in any order.

Arguments:

ncore - An integer value for the number of cores the user wants to use (default=2)

Nfolds - an integer for the number of times the LASSO regression is run

numCounter - an integer for the number of folds one wants for their LASSO regression

nonZeroPercent - an integer for the percentage of appearance as a non-zero LASSO coefficient for a miRNA to be selected

mirdirectory - a character string for the file path of the directory one wants the results in

```
lassoParallelForTransGene(
    ncore = 1,
    numCounter = 6,
    Nfolds = 3,
    nonZeroPercent = 20,
    mirdirectory =  mirdirectory
)
```

```
##                genes        miRNA      av_coeff
##   1: ENSG00000002726 hsa.mir.190b  2.1798795007
##   2: ENSG00000002726 hsa.mir.4654 -0.2872436611
##   3: ENSG00000002726 hsa.mir.5187  0.2992766991
##   4: ENSG00000002726 hsa.mir.6737  1.3285735784
##   5: ENSG00000002726 hsa.mir.6738  0.8597862944
##  ---
## 324: ENSG00000119777 hsa.mir.6737 -2.1146720716
## 325: ENSG00000119777 hsa.mir.6738 -2.9358522727
## 326: ENSG00000119777  hsa.mir.765 -3.4540627671
## 327: ENSG00000119777  hsa.mir.9.1  0.0013722095
## 328: ENSG00000119777  hsa.mir.92b -0.0006194935
```

Output: The output for this function can be found in the mirdirectory in mirDriverFold/miRDriver_RESULTS. There is a field for each gene and its predictors in the LassoMinCoeff folder and a dataframe with the miRNA that are most significant.

```
load(
    file.path(
        mirdirectory,
        "mirDriverFold",
        "miRDriver_RESULTS",
        "LassoMinCoeff",
        "ENSG00000088387.Rda"
    )
)
#Showing gene-miRNA interaction network with non-zero coefficients
min_Coeff[min_Coeff$coefficients_min!=0, ][1:5,]
```

```
##         coefficients_min       genes_min predictors_min
## 1:  -0.00409136150120802 ENSG00000088387   hsa.mir.5698
## 2:    0.0255992410090255 ENSG00000088387   hsa.mir.190b
## 3:   -0.0369748725003765 ENSG00000088387   hsa.mir.4654
## 4: -2.44881388159001e-05 ENSG00000088387           beta
## 5:                  <NA>            <NA>           <NA>
```

```
load(
    file.path(
        mirdirectory,
        "mirDriverFold",
        "miRDriver_RESULTS",
        "Final_gene_mirna_network.Rda"
    )
)
Final_gene_mirna_network[1:5, ]
```

```
##              genes        miRNA   av_coeff
## 1: ENSG00000002726 hsa.mir.190b  2.1798795
## 2: ENSG00000002726 hsa.mir.4654 -0.2872437
## 3: ENSG00000002726 hsa.mir.5187  0.2992767
## 4: ENSG00000002726 hsa.mir.6737  1.3285736
## 5: ENSG00000002726 hsa.mir.6738  0.8597863
```

4 Summary

This package is designed to Infer Copy Number Derived miRNA-Gene Networks in Cancer. The four step process breaks up the different parts of the process into chunks for easy use and produces accurate results.

5 Acknowledgements

This work was supported by the National Institute of General Medical Sciences of the National Institutes of Health under Award Number R35GM133657.
